# Supplementary material for: General method for carbon–heteroatom cross-coupling reactions via semiheterogeneous red-light metallaphotocatalysis
Source: Nat Commun. 2025 Jul 31;16:7045. doi: 10.1038/s41467-025-61812-z (PMC12314023; doi:10.1038/s41467-025-61812-z)
Supplement: Supplementary file 1 — Supplementary Information [file 41467_2025_61812_MOESM1_ESM.pdf]

Supplementary Information

General Method for Carbon–Heteroatom Cross-Coupling Reactions via Semiheterogeneous Red-Light Metallaphotocatalysis

Geyang Song,[1#] Wei Zhang,[1#] Jiameng Song,[1] Qi Li,[1] Yuyu Feng,[1] Hongyu Liang,[1] Tengfei Kang,[1] Jianyang Dong,[1] Gang Li,[1] Juan Fan,[1] Xue-Peng Zhang,[1] Quan Gu,*[1] Chao Wang[1] and Dong Xue*,[1]

[1]Key Laboratory of Applied Surface and Colloid Chemistry of Ministry of Education, School of Chemistry and Chemical Engineering, Shaanxi Normal University, Xi'an, 710119, China;

[#] These authors contributed equally.

Correspondence to: guquan@snnu.edu.cn; xuedong_welcome@snnu.edu.cn

Contents

1. General Information S1
2. Optimization of reaction conditions S3
3. Synthesis of different catalysts S10
4. General procedure for amination S13
5. Multiple recovery steps for the reuse of CN-OA-m S19
6. Effect of temperature on reaction under red light excitation S20
7. Experiments on electronic and hole scavengers S21
8. Time curves S22
9. The catalytic reaction of alternating the light irradiation S23
10. Mechanistic investigations S24
11. The EPR experiment spectroscopic S33
12. Characterization data of CN-OA-m before and after recovery S35
13. Redox potentials of different organic bases S41
14. Analytical data of products S44
15. Copies of ^1H NMR, ^{13}C NMR and ^{19}F NMR spectra of products S112
16. References S400

1. General Information

Unless otherwise specified, the chemicals were obtained commercially and used without further purification. Analytical thin-layer chromatography (TLC) was conducted with TLC plates (Silica gel 60 F254, Qingdao Haiyang) and visualization on TLC was achieved by UV light developer to assist. Flash column chromatography was performed on silica gel 200-300 mesh. ^{1}H NMR spectra were recorded on a Bruker Advance 400 MHz or 600 NMR spectrometer and reported in units of parts per million (ppm) relative to tetramethylsilane (δ 0 ppm), $CDCl_3$ (δ 7.26 ppm), d_6-DMSO (δ 2.55 ppm). Multiplicities are given as: br (broad singlet), s (singlet), d (doublet), t (triplet), q (quartet), or m (multiplet). ^{13}C NMR spectra were recorded on a Bruker Advance 400 (100 MHz) or 600 (150 MHz) NMR spectrometer and reported in ppm relative to tetramethylsilane (δ 0 ppm), $CDCl_3$ (δ 77.16 ppm), d_6-DMSO (δ 39.52 ppm). HRMS (APCI or ESI) were performed on a Fourier transform ion cyclotron resonance mass spectrometer (Maxis Ultimate 300hplc). Continuous-wave electron paramagnetic resonance (EPR) spectra was recorded at on a Bruker E-500 spectrometer. Scanning Electron Microscopy (SEM) images were obtained on a field emission scanning electron microscope (HITACHI SU8220 microscope) at an acceleration voltage and the applied current of 5 kV and 10 μA. For the SEM test, the powder samples were glued on an aluminium SEM specimen holder with the conductive resin, and then the specimen holder was directly put into SEM for testing without gold spraying. Transmission Electron Microscopy (TEM) images and element mapping were obtained by a Tecnai G2 F20 transmission electron microscopy (FEI, USA) at an accelerating voltage of 200 kV. A small amount of the sample was dispersed in 1 mL ethanol and the mixture was then sonicated for 30 min at room temperature in a bath sonicator. The obtained suspension was dropped on 230-mesh carbon-coated copper grids and air-dried for TEM measurements. X-Ray Photoelectron Spectroscopy (XPS) spectra were determined on a VG ESCALAB 250 XPS system with a monochromatized Al Kα X-ray source (15 kV, 200 W). Fourier Transform Infrared (FTIR) spectra were derived from Perkin Elmer Fourier transform infrared spectrometer GX. UV-Vis absorption spectra were collected on a PerkinEImer Lambda 365 UV-VIS Spectrophotometer. Fluorescent spectra were recorded on Edinburgh FLS1000. N_2 adsorption/desorption isotherms were recorded at 77 K on a Micromeritics ASAP2460 instrument. For photoelectrochemical experiments, the as-prepared sample was coated on fluorinedoped tin oxide (FTO) glass as the working electrode. Typically, the FTO glass with a size of 1.0×3.0 cm was washed in turn with acetone, ethanol, and DI water under continuous sonication, and then dried in N2 flowing. By dispersing a certain amount of the sample in water, the sample slurry was obtained and used for spreading onto the cleaned FTOglass substrate (photoactive area of 0.25 cm2) and dried at room temperature. The photoelectrochemical tests were performed on a CHI 600E electrochemical work station (Chenhua Instrument, Shanghai, China) at room temperature in a standard three-electrode system including a working electrode, a platinum wire counter electrode, and an Ag/AgCl reference electrode. The working electrode was immersed in a sodium sulfate electrolyte solution (0.5 M) and irradiated by simulated solar light irradiation. The chopped anodic photocurrent (i-t curve) of samples was tested at an applied bias of 0.6 V vs Ag/AgCl. Electrochemical Impedance Spectroscopy (EIS) measurement was conducted in a frequency range of 0.01 Hz to 10 kHz for an amplitude of 5 mV. All reagents were obtained commercially and used without further purification. MeCN, DMF and dioxane were refluxed over CaH_2. Toluene and THF were refluxed over Na/benzophenone and distilled under an argon atmosphere. Solvents used for column chromatography were of technical grade and used after distillation. *m*DBU (1,4,5,6-tetrahydro-1,2-

dimethylpyrimidine). The red LED lamp used in the experiment was assembled by ourselves (Figure S1 and S2) and the light-emitting angle of the lamp bead is 45 degrees. The LED lamp is assembled by connecting five components. The kit consists of four 3W red LED lamp beads, thermally conductive separation aluminum substrate, thermally conductive aluminum module, LED lamp bead switch and LED driver (XC-8W600-OS, http://www.xchly.com/en/produc tshow-388-389-683.html). The red LED beads were purchased from Zhuhai Tianhui Electronics Co., Ltd. (TH-UV395T3WL-3535-60, http://www.tianhui-led.com/product/454-cn.html). The optical power was up to 470-520 mw at 0.5 cm axis distance detected by a Thorlabs' optical power and energy meter (PM 100D). The screening of light sources was performed on our self-made photocatalytic parallel reactor. We did not use band pass filters, and the specific wavelengths (620-630 nm, 660-670 nm) refer only to the max of irradiation. Furthermore, for all light sources, it only refers to the maximum value of the illumination.

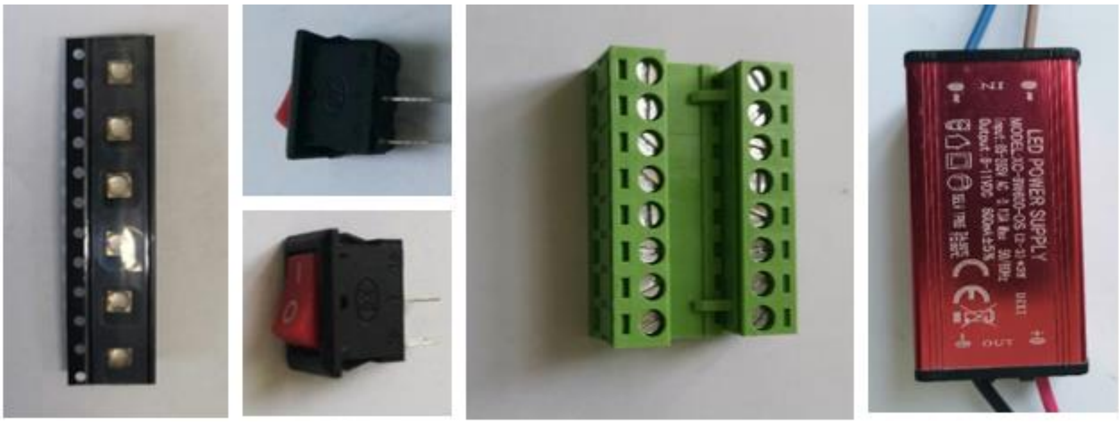

Figure S1. Light sources used in the reactions (1x10 W red LED lamp beads, LED driver, the thermalradiation of LEDs increased the temperature of reaction mixture as an average level at 85 ^{o}C approximately).

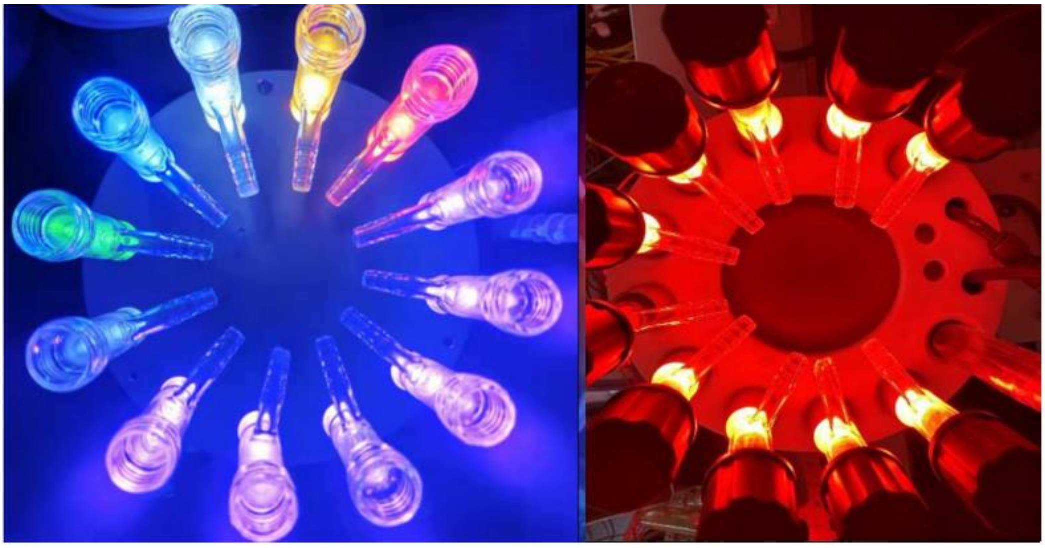

Figure S2. Our self-made photocatalytic parallel reactor. (4 x 3 W LED lamp beads, LED driver)

2. Optimization of reaction conditions

2.1 Optimization of red light C-N coupling reaction conditions

Table S1. The effect of different types of C_3N_4

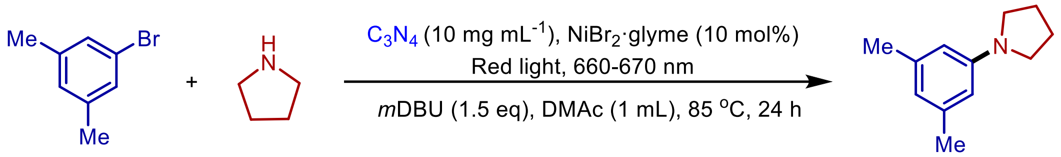

Entry	C_3N_4 (5 mg/mL)	Yield[a]
1	CN-OA-m	95
2	mpg-C_3N_4	51
3	MC-C_3N_4	83
4	PR-C_3N_4	76
5	C_3N_4	71
6	p-C_3N_4	43
7	g-C_3N_4	46

Reaction conditions: 5-Bromo-m-xylene (0.2 mmol), pyrrolidine (0.4 mmol), $NiBr_2$•glyme (10 mol%), C_3N_4 (10 mg), *m*DBU (1.5 eq), DMAc (1 mL), red LEDs (660-670 nm), Ar, 24 h.[a] Yields determined by ^{1}H NMR analysis using 1,3-benzodioxole as internal standard.

Table S2. The effect of the amount of CN-OA-m

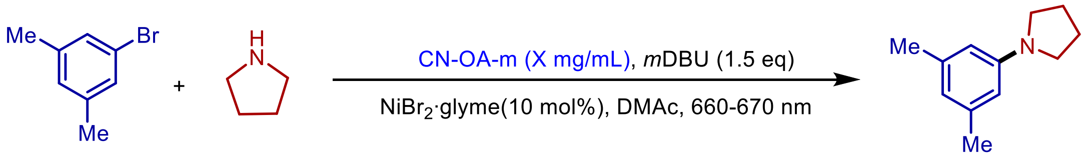

Entry	CN-OA-m (x mg/mL)	Yield[a]
1	3.33	45
2	5	86
3	10	95

Reaction conditions: 5-Bromo-m-xylene (0.2 mmol), pyrrolidine (0.4 mmol), $NiBr_2$•glyme (10 mol%), CN-OA-m (X mg), *m*DBU (1.5 eq), DMAc (1 mL), red LEDs (660-670 nm), Ar, 24 h.[a] Yields determined by ^{1}H NMR analysis using 1,3-benzodioxole as internal standard.

Table S3. The effect of solvent

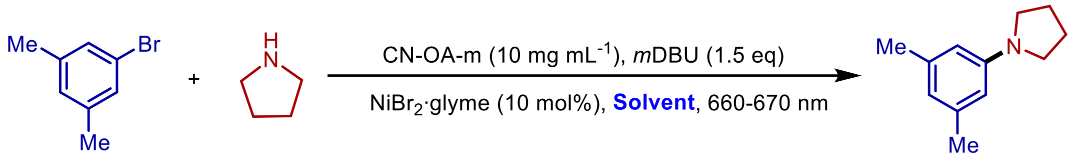

Entry	Solvent	Yield (%)[a]
1	PhMe	trace
2	DMSO	42
3	DMAc	95
4	DMF	87
5	CH_3CN	trace
6	THF	trace
7	2-Me-THF	trace
8	1,4-dioxane	trace

Reaction conditions: 5-Bromo-m-xylene (0.2 mmol), pyrrolidine (0.4 mmol), $NiBr_2$•glyme (10 mol%), CN-OA-m (10 mg), *m*DBU (1.5 eq), solvent (1 mL), red LEDs (660-670 nm), Ar, 24 h.[a] Yields determined by ^{1}H NMR analysis using 1,3-benzodioxole as internal standard.

Table S4. The effect of different Ni source

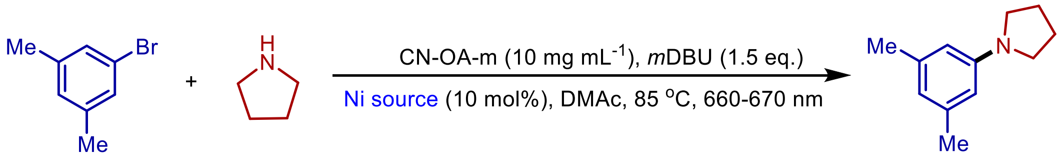

Entry	Ni source	Yield (%)[a]
1	$NiBr_2$	83%
2	$NiBr_2$•glyme	97%
3	$NiBr_2 \cdot 3H_2O$	78%
4	$NiCl_2$	40%
5	$NiCl_2$•glyme	40%
6	$Ni(OAc)_2$	20%
7	NiI_2	41%

Reaction conditions: 5-Bromo-m-xylene (0.2 mmol), pyrrolidine (0.4 mmol), Ni source (10 mol%), CN-OA-m (10 mg), *m*DBU (1.5 eq), DMAc (1 mL), red light (660-670 nm), Ar, 24 h.[a] Yields determined by ^{1}H NMR analysis using 1,3-benzodioxole as internal standard.

Table S5. The effect of amount of $NiBr_2$·glyme

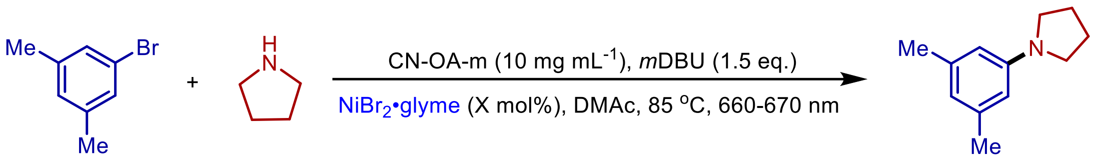

Entry	$NiBr_2$•glyme (X mol%)	Yield (%)[a]
1	1	trace
2	3	22%
3	5	45%
4	8	80%
5	10	98%

Reaction conditions: 5-Bromo-m-xylene (0.2 mmol), pyrrolidine (0.4 mmol), $NiBr_2$·glyme (X mol%), CN-OA-m (10 mg), *m*DBU (1.5 eq), DMAc (1 mL), red light (660-670 nm), Ar, 24 h.[a] Yields determined by ^{1}H NMR analysis using 1,3-benzodioxole as internal standard.

Table S6 The effect of light sources

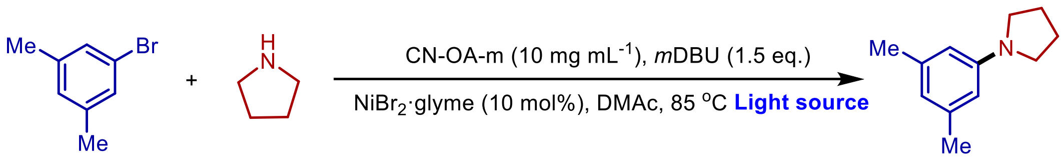

Entry	Light source	Yield (%)[a]
1	UV (360-365 nm)	n.r.
2	purple LEDs (390-395 nm)	n.r.
3	420-425 nm	24
4	blue LEDs (460-465 nm)	31
5	green LEDs (530-535 nm)	56
6	white LEDs	62
7	585-595 nm	62
8	620-630 nm	66
9	660-670 nm	96
10	Infrared (760 nm)	n.r.

Reaction conditions: 5-Bromo-m-xylene (0.2 mmol), pyrrolidine (0.4 mmol), $NiBr_2$·glyme (10 mol%), CN-OA-m (10 mg), *m*DBU (1.5 eq), DMAc (1 mL), light source, Ar, 24 h.[a] Yields determined by ^{1}H NMR analysis using 1,3-benzodioxole as internal standard.

Table S7. The effect of different types of bases

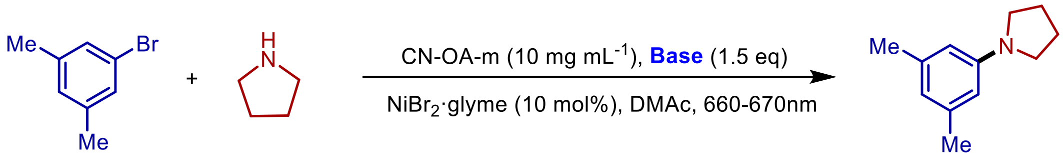

Entry	Base	Yield (%)[a]
1	DBU	96
2	BTMG	57
3	TMG	79
4	MTBD	82
5	DABCO	70
6	Quinuclidine	33
7	*m*DBU	98
8	Et_3N	trace
9	DBN	80
10	TBD	78
11	Cy_2NMe	N.R.
12	DIPEA	86

Reaction conditions: 5-Bromo-m-xylene (0.2 mmol), pyrrolidine (0.4 mmol), $NiBr_2$•glyme (10 mol%), CN-OA-m (10 mg), Base (1.5 eq), DMAc (1 mL), red LEDs (660-670 nm), Ar, 24 h.[a] Yields determined by ^1H NMR analysis using 1,3-benzodioxole as internal standard.

Table S8. The effect of the amount of *m*DBU

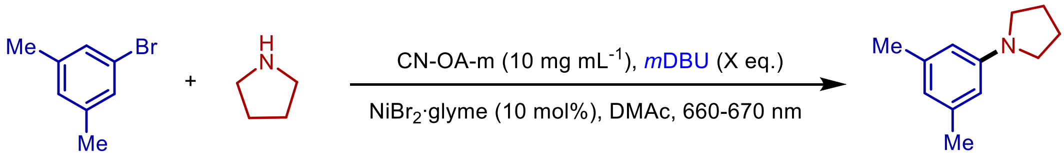

Entry	*m*DBU (X eq.)	Yield (%)[a]
1	0.5	46
2	1.0	74
3	1.5	98
4	2.0	89

Reaction conditions: 5-Bromo-m-xylene (0.2 mmol), pyrrolidine (0.4 mmol), $NiBr_2$•glyme (10 mol%), CN-OA-m (10 mg), *m*DBU (x eq), DMAc (1 mL), red LEDs (660-670 nm), Ar, 24 h.[a] Yields determined by ^1H NMR analysis using 1,3-benzodioxole as internal standard.

Table S9. The effect of concentration

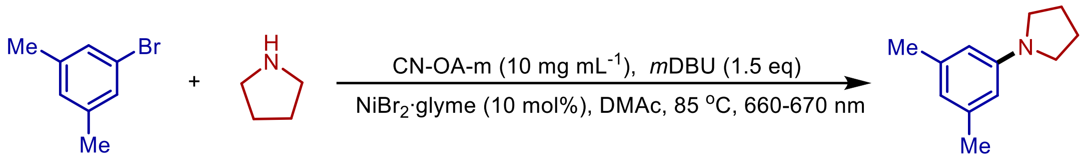

Entry	V (mL)	Yield (%)[a]
1	1.0	97
2	2.0	81
3	3.0	76
4	4.0	61

Reaction conditions: 5-Bromo-m-xylene (0.2 mmol), pyrrolidine (0.4 mmol), $NiBr_2$•glyme (10 mol%), CN-OA-m (10 mg), *m*DBU (1.5 eq), DMAc (V mL), red light (660-670 nm), Ar, 24 h.[a] Yields determined by ^{1}H NMR analysis using 1,3-benzodioxole as internal standard.

Table S10. Control experiments of C-N coupling

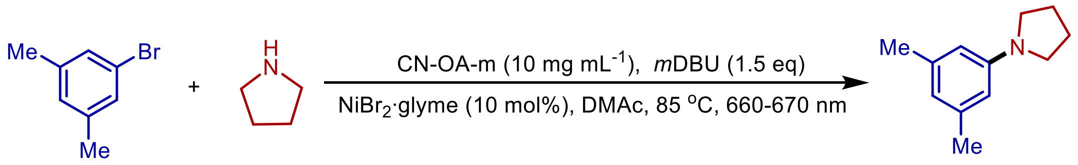

Entry	Variation from standard conditions	Yield (%)[a]
1	Standard condition	96
2	Standard condition,no CN-OA-m	N.R.
3	Standard condition, no DMTHPM	N.R.
4	Standard condition, R.T.	N.R.
5	Standard condition, no light	N.R.
6	Standard condition, air	N.R.

Reaction conditions: 5-Bromo-m-xylene (0.2 mmol), pyrrolidine (0.4 mmol), $NiBr_2$•glyme (10 mol%), CN-OA-m (10 mg), *m*DBU (1.5 eq), DMAc (1 mL), red light (660-670 nm), Ar, 24 h.[a] Yields determined by ^{1}H NMR analysis using 1,3-benzodioxole as internal standard.

2.2 Optimization of red light C-O coupling reaction conditions

Table S11. The effect of ligands

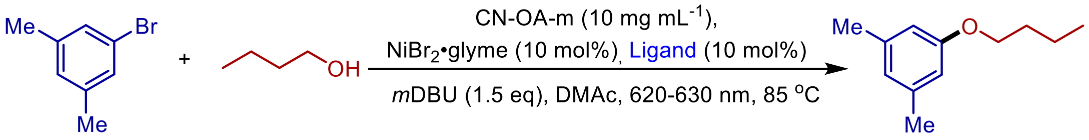

Ligand	Yield[a]	Ligand	Yield[a]
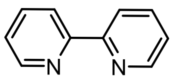	55%	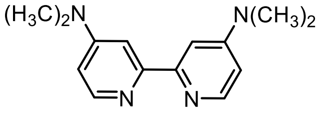	40%
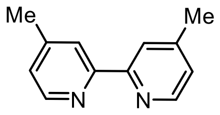	71%	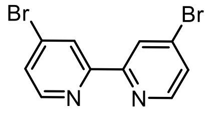	32%
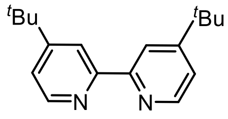	76%	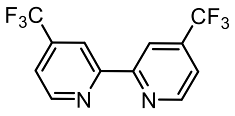	trace
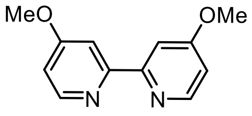	57%	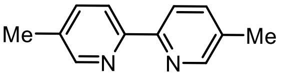	31%
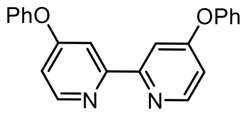	23%	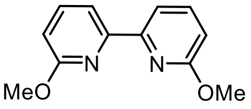	35%

Reaction conditions: 1-Bromo-3,5-dimethylbenzene (0.2 mmol), 1-butanol (0.55 mmol), $NiBr_2$·glyme (10 mol%), ligand (10 mol%), CN-OA-m (10 mg), *m*DBU (1.5 eq), DMAc (1 mL), red light (620-630 nm), Ar, 24 h.[a] Yields determined by ^{1}H NMR analysis using 1,3-benzodioxole as internal standard.

Table S12 The effect of material ratio

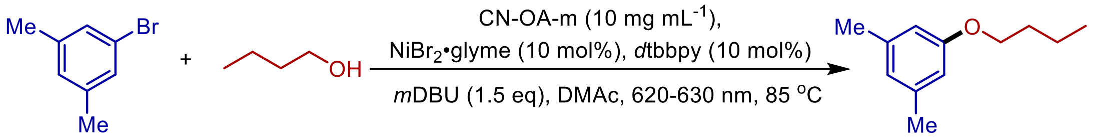

Entry	1-Butanol	Yield[a]
1	1.0	32%
2	1.5	42%
3	1.75	55%
4	2.0	61%
6	2.25	66%
7	2.5	67%
8	2.75	78%
9	3.0	91%

Reaction conditions: 1-Bromo-3,5-dimethylbenzene (0.2 mmol), 1-butanol (X mmol), $NiBr_2$·glyme (10 mol%), *d*tbbpy (10 mol%), CN-OA-m (10 mg), *m*DBU (1.5 eq), DMAc (1 mL), red light (620-630 nm), Ar, 24 h.[a] Yields determined by ^{1}H NMR analysis using 1,3-benzodioxole as internal standard.

Table S13 The effect of light sources

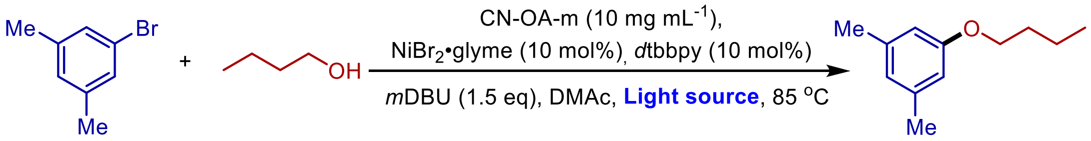

Entry	Light source	Yield (%)[a]
1	Infrared (760 nm)	n.r.
2	660-670 nm	49
3	620-630 nm	91
4	585-595 nm	10

Reaction conditions: 1-Bromo-3,5-dimethylbenzene (0.2 mmol), 1-butanol (0.6 mmol), $NiBr_2$·glyme (10 mol%), *d*tbbpy (10 mol%), CN-OA-m (10 mg), *m*DBU (1.5 eq), DMAc (1 mL), light source, Ar, 24 h.[a] Yields determined by ^{1}H NMR analysis using 1,3-benzodioxole as internal standard.

Table S14. Control experiments of C-O coupling

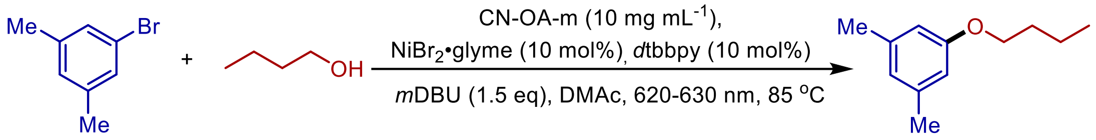

Entry	Variation from standard conditions	Yield (%)[a]
1	Standard condition	91
2	Standard condition,no CN-OA-m	N.R.
3	Standard condition, no *m*DBU	N.R.
4	Standard condition, R.T.	N.R.
5	Standard condition, no light	N.R.
6	Standard condition, air	N.R.

Reaction conditions: 1-Bromo-3,5-dimethylbenzene (0.2 mmol), 1-butanol (0.6 mmol), $NiBr_2$•glyme (10 mol %), *d*tbbpy (10 mol %), CN-OA-m (10 mg), *m*DBU (1.5 eq), DMAc (1 mL). red light (620-630 nm), Ar, 24 h.[a] Yields determined by ^{1}H NMR analysis using 1,3-benzodioxole as internal standard.

3. Synthesis of different catalysts[1]

3.1 Synthesis of different types of C_3N_4

3.1.1 Synthesis of g-C_3N_4

10 g of melamine was put into a crucible and heated to 550 °C at a heating rate of 5°C·min^{-1} under an air atmosphere for 4 hours to obtained the traditional g-C_3N_4

3.1.2 Synthesis of p-C_3N_4

10 g of melamine was put into a crucible and heated to 550 °C at a heating rate of 4°C·min^{-1} under an air atmosphere for 4 hours to obtained the traditional p-C_3N_4

3.1.3 Synthesis of C_3N_4

10 g of melamine was put into a crucible and heated to 500 °C at a heating rate of 10°C·min^{-1} under an air atmosphere for 4 hours to obtained the traditional g-C_3N_4 (g-CN). Next, 600 mg of the g-CN sample was ground with KCl (3.3 g) and LiCl (2.7 g) for 10 min, and the mixture was heated to 550 °C at a heating rate of 5 °C·min^{-1} in a muffle furnace under air atmosphere for 4 hours. The obtained product (denoted as CN)

was washed with boiling water three times, collected by centrifugation, and dried in an oven at 60°C overnight.

3.1.4 Synthesis of PR-C_3N_4

Melamine (2.0 g), KCl (4.0 g), and TAP (1.0 g) were mixed and ground fine by adding 2 mL ethanol for 10 min. Ethanol was then evaporated 80 °C to get the well-mixed powder. The mixture was transferred into a crucible with cover and annealed at 600 °C for 3 h with the heating rate of 3 °C · min^{-1} in the furnace under air atmosphere. After cooling to the room temperature, the crude product was fully immersed into the boiling deionized water to remove residual KCl. Finally, the red product was collected by centrifugation and vacuum-dried at 60 °C overnight. Pristine g-C_3N_4 was synthesized at the similar condition as PRCN by only using melamine as precursor.

3.1.5 Synthesis of MC-C_3N_4

Carbohydrazide was firstly heated at a ramping rate of 5 °C min-1 to 450 °C for 2 h under air atmosphere. The obtained red-brown powder was denoted as C-CN (Carbohydrazide derived Carbon Nitride). 1 g of C-CN was then ground with 15 g of eutectic salt (KCl-NaCl-LiCl with the mass ratio of 34:34:32 wt%) in a ceramic mortar and the mixture was heated to 550 °C for 2 h at a ramp rate of 5 °C min^{-1} under air atmosphere. The obtained powder was washed several times with deionized water to completely remove the free salts (0.1 M $AgNO_3$ solution was used to detect the presence of Cl^-), and the sample was dried overnight at 60°C. The final product was designated as MC-CN.

3.1.6 Synthesis of mpg-C_3N_4

A mixture of cyanamide (3.00 g) and colloidal silica aqueous solution (Ludox HS-40, 40 wt.%, 7.50 g) was stirred in a glass vial at room temperature for about 15 minutes until cyanamide was dissolved completely. Water was slowly evaporated upon stirring the mixture overnight at 60 ^oC. Magnetic stirring bar was removed and the white solid was transferred into a porcelain crucible and calcinated at 550 ^oC for ca. 4h under flow of nitrogen in a muffle oven. The oven was allowed to cool to room temperature, the content from the crucible was transferred into a polypropylene bottle, The resulting brown-yellow powder was treated with a 4M NH_4HF_2 for 24 h to remove the silica

template. The powders were then centrifuged and washed three times with distilled water and twice with ethanol, and dried overnight in a vacuum oven (60 °C).

3.1.7 Synthesis of CN-OA-m

Typically, 10 g of urea and 0.5 g of oxamide were mixed in 10 mL of DI water to generate a homogeneous mixture. After completely evaporation at 373 K, the resulted solids were then placed into a crucible with a cover and pre-heated in the oven at 773 K for 1 hour with a heating rate of 10 K min^{-1}. After the temperature naturally cooled dawn to room temperature, the as-prepared solids were then fully mixed with 3.3 g of KCl and 2.7 g of LiCl. The mixture was then transferred into a crucible with a cover and then heated in nitrogen flow (Ar rate: 10 L / min) oven at 823 K for two hours with a fast heating rate of 10 K min^{-1}. After the temperature naturally cooled dawn to room temperature, the resulted solids were then washed with DI water for several times to remove the residual metals. The final products were collected after the solids were dried in vacuum for overnight. It was denoted as CN-OA-m for simplify. To examine the effect of pre-heating temperature, the mixtures were also pre-heated at 673, 723, and 823 K.

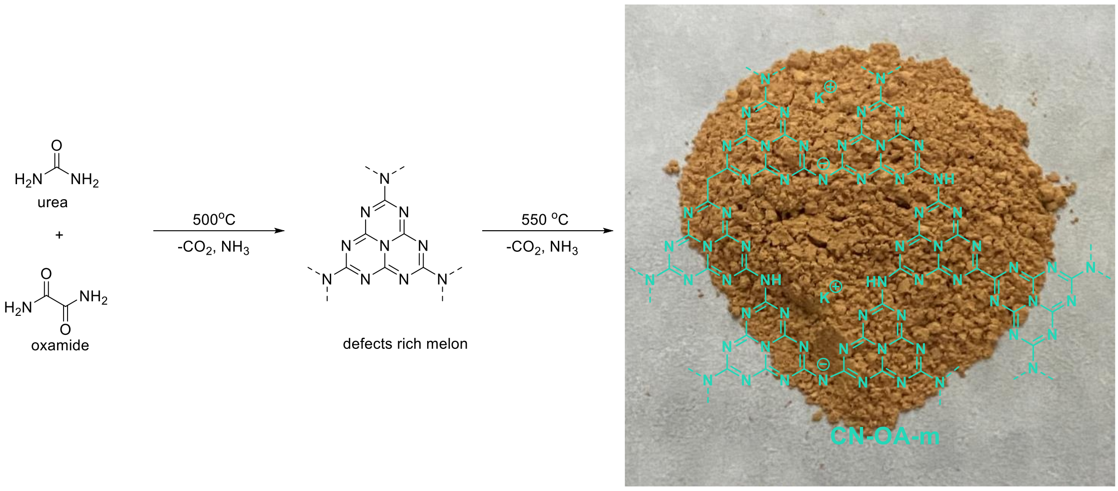

Figure S3. Synthesis of CN-OA-m.

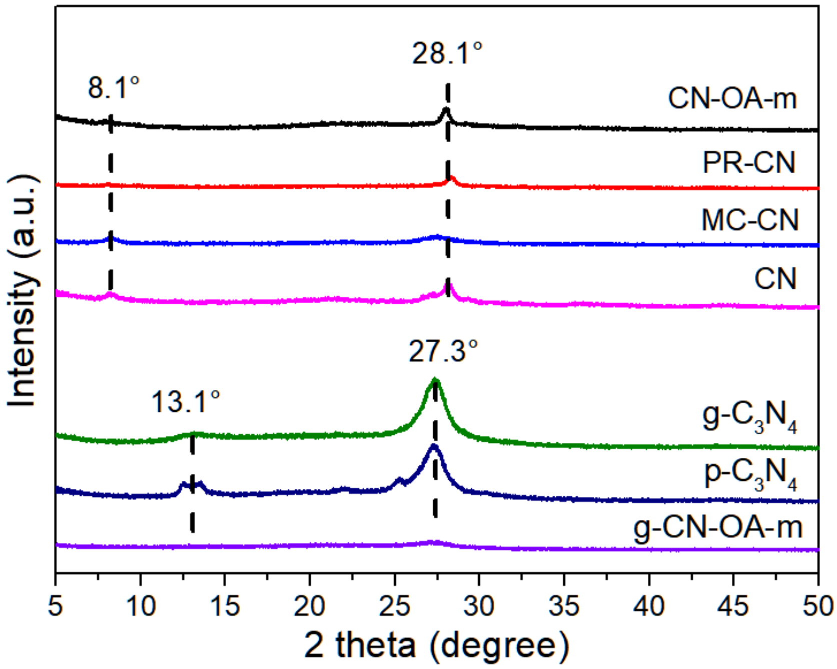

Figure S4. XRD of different types of C_3N_4

3.2 Synthesis of Ni(*d*-tbbpy)Br$_2$[2]

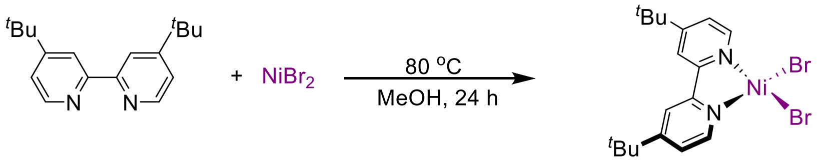

To a dry MeOH (10 mL) suspension of NiBr$_2$ (0.5 mmol), *d*-tbbpy (0.5 mmol) in an oven-dried 25 mL screw-cap vials. The resulting mixture was stirred at 80 ºC for 24 h under argon. After cooling to room temperature and removal of all the solvent, the precipitate was collected on a frit, rinsed with pentane and residual solvent was removed under vacuum to give the title compound as a green powder (430 mg, 89% yield). The compound was used without further purification. HRMS (ESI): Calcd for $C_{18}H_{24}Br_2N_2Ni$ $[M\text{-}Br]^+$: 405.0471, found: 405.0480.

4. General procedure for amination

4.1 Standard procedure for the C-N coupling of aryl bromides

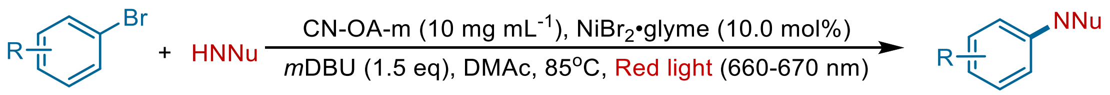

To an oven-dried 10 mL of storage tube were added $NiBr_2$•glyme (10.0 mol%), 1 mL of DMAc and a magnetic stir bar under argon atmosphere. The mixture was evacuated and backfilled with argon for 3 times. Then the aryl bromide (0.2 mmol), amine (0.4 mmol), CN-OA-m (10mg/mL) and *m*DBU (1,4,5,6-tetrahydro-1,2-dimethylpyrimidine) (1.5 eq., 0.3 mmol) were added. The tube was sealed with a Teflon screw valve. The reaction mixture was then irradiated with 10 W red LEDs (0.5 cm away from the tube, 660-670 nm) at 85 °C. After the reaction was completed, the mixture was diluted with ethyl acetate after cooling to room temperature. The organic phases were washed with saturated ammonium chloride (3 × 10 mL), dried over anhydrous sodium sulfate, and concentrated under reduced pressure. The residue was purified by flash column chromatography using petroleum ether and ethyl acetate as eluent to afford aryl amines.

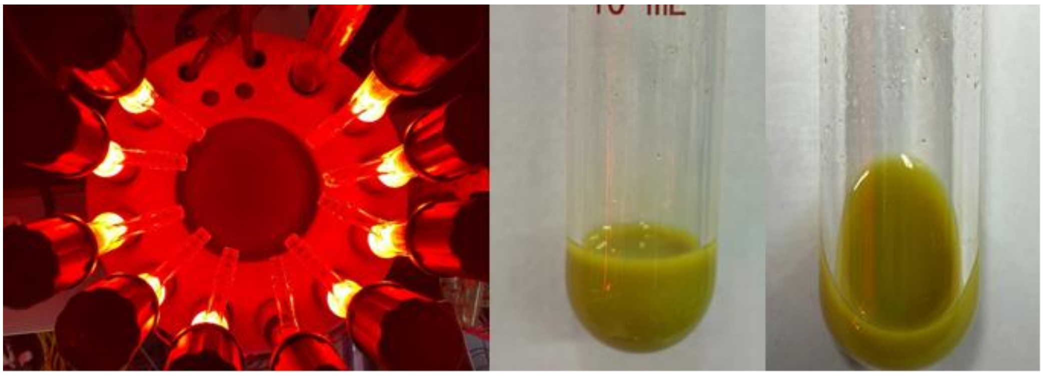

Figure S5. Pictures of the reaction setup

4.2 Standard procedure for the C-O coupling of aryl bromides

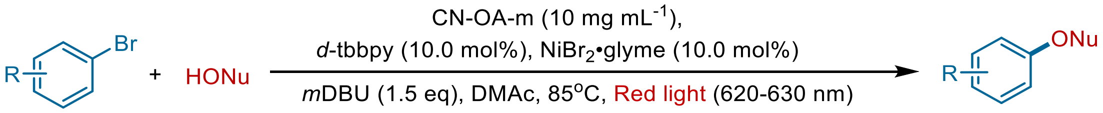

To an oven-dried 10 mL of storage tube were added *d*-tbbpy (4,4'-Di-tert-butyl-2,2'-dipyridyl) (10.0 mol%), $NiBr_2$•glyme (10.0 mol%), 1 mL of DMF and a magnetic stir bar under argon atmosphere. The mixture was evacuated and backfilled with argon for 3 times. Then aryl bromide (0.2 mmol), *O*-nucleophilic reagent (0.6 mmol), CN-OA-m (10mg/mL) and *m*DBU (1,4,5,6-tetrahydro-1,2-dimethylpyrimidine) (1.5 eq., 0.3 mmol) were added. The tube was sealed with a Teflon screw valve. The reaction mixture was then irradiated with 10 W red LEDs (0.5 cm away from the tube, 620-630

nm) at 85 °C. After the reaction was completed, the mixture was diluted with ethyl acetate after cooling to room temperature. The organic phases were washed with saturated ammonium chloride (3 × 10 mL), dried over anhydrous sodium sulfate, and concentrated under reduced pressure. The residue was purified by flash column chromatography using petroleum ether and ethyl acetate as eluent to afford aryl ethers.

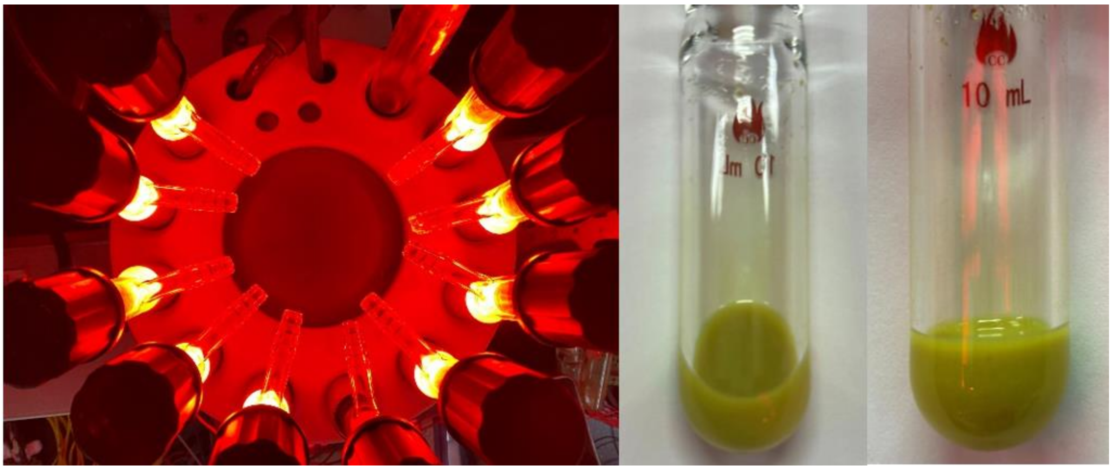

Figure S6. Pictures of the reaction setup

4.3 Standard procedure for the C-S/Se coupling of aryl bromides

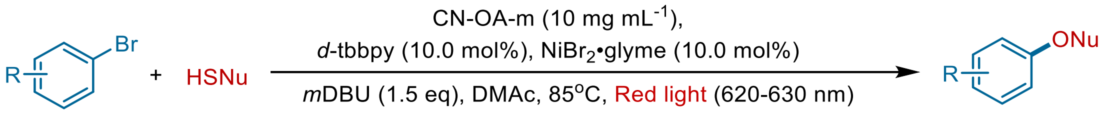

To an oven-dried 10 mL of storage tube were added *d*-tbbpy (4,4'-Di-tert-butyl-2,2'-dipyridyl) (10.0 mol%), $NiBr_2$•glyme (10.0 mol%), 1 mL of DMAc and a magnetic stir bar under argon atmosphere. The mixture was evacuated and backfilled with argon for 3 times. Then aryl bromide (0.2 mmol), *S*-nucleophilic reagent (0.4 mmol), CN-OA-m (10mg/mL) and *m*DBU (1,4,5,6-tetrahydro-1,2-dimethylpyrimidine) (1.5 eq., 0.3 mmol) were added. The tube was sealed with a Teflon screw valve. The reaction mixture was then irradiated with 10 W red LEDs (0.5 cm away from the tube, 620-630 nm) at 85 °C. After the reaction was completed, the mixture was diluted with ethyl acetate after cooling to room temperature. The organic phases were washed with saturated ammonium chloride (3 × 10 mL), dried over anhydrous sodium sulfate, and concentrated under reduced pressure. The residue was purified by flash column chromatography using petroleum ether and ethyl acetate as eluent to afford aryl sulfides.

4.4 Standard procedure for the C-N coupling under solar light

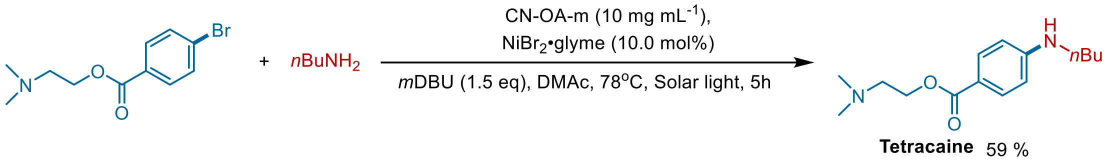

To an oven-dried 10 mL of storage tube were added $NiBr_2$•glyme (10.0 mol%), 1 mL of DMAc, and a magnetic stir bar under argon atmosphere. The mixture was evacuated and backfilled with argon for 3 times. Then the 2-(dimethylamino)ethyl 4-bromobenzoate (0.2 mmol), *n*-butylamine (0.4 mmol), CN-OA-m (10 mg/mL) and *m*DBU (1,4,5,6-tetrahydro-1,2-dimethylpyrimidine) (1.5 eq., 0.3 mmol) were added. The tube was sealed with a Teflon screw valve. The reaction mixture was then irradiated with sun light for 5h in solar light. On cooling to room temperature, 1,3-benzodioxole (0.2 mmol) was added to the reaction solution. The resulting mixture was diluted with 5 mL $CHCl_3$, the solvent was removed under reduced pressure and the residue was analyzed by ^{1}H NMR with $CDCl_3$ as solvent to give the yield of the reaction.

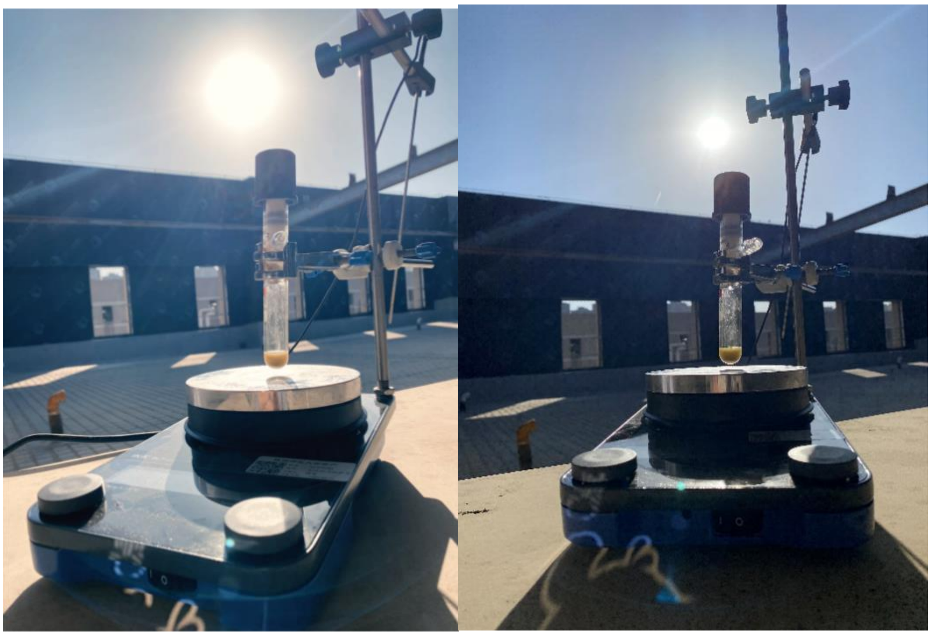

Figure S7. Pictures of the reaction setup in solar light.

4.5 Standard procedure for the C-N coupling under simulate solar light

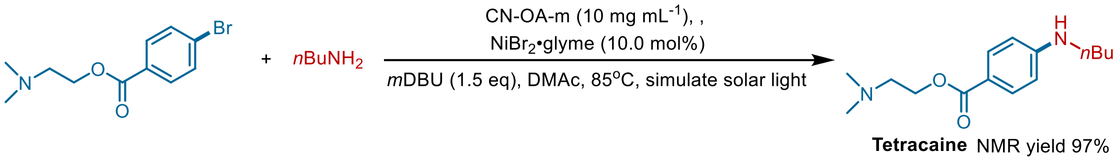

To an oven-dried 10 mL of storage tube were added $NiBr_2$•glyme (10.0 mol%), 1 mL of DMAc and a magnetic stir bar under argon atmosphere. The mixture was evacuated and backfilled with argon for 3 times. Then the 2-(dimethylamino)ethyl 4-bromobenzoate (0.2 mmol), *n*-butylamine (0.4 mmol), CN-OA-m (10mg/mL) and *m*DBU (1,4,5,6-tetrahydro-1,2-dimethylpyrimidine) (1.5 eq., 0.3 mmol) were added. The tube was sealed with a Teflon screw valve. The reaction mixture was then irradiated with simulate solar light for 24 h. On cooling to room temperature, 1,3-benzodioxole (0.2 mmol) was added to the reaction solution. The resulting mixture was diluted with 5 mL $CHCl_3$, the solvent was removed under reduced pressure and the residue was analyzed by ^{1}H NMR with $CDCl_3$ as solvent to give the yield of the reaction.

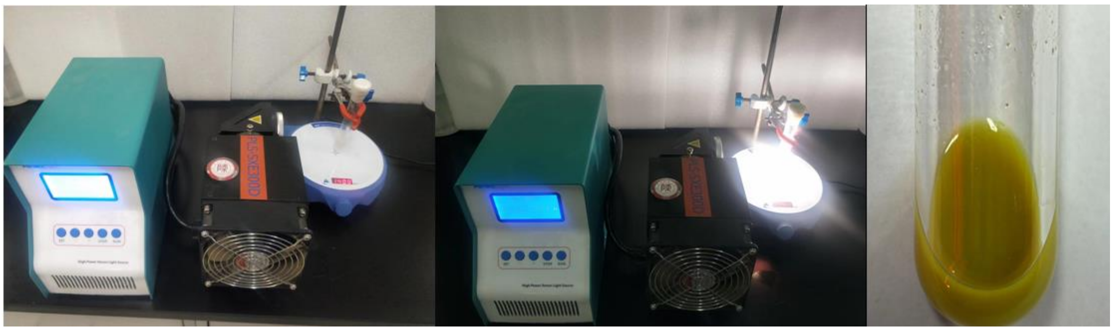

Figure S8. Pictures of the reaction setup in simulate solar light.

4.6 Procedure for the synthesis of aryl amines at gram-scale under red light

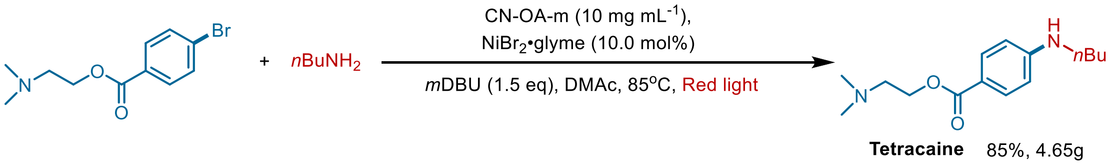

To an oven-dried 250 mL of storage tube were added $NiBr_2$•glyme (10.0 mol%), and 100 mL of DMAc with a magnetic stir bar under argon atmosphere. The mixture was evacuated and backfilled with Ar for 3 times. Then 2-(dimethylamino)ethyl 4-bromobenzoate (20 mmol), *n*-butylamine (40 mmol), CN-OA-m (10 mg/mL) and *m*DBU (1,4,5,6-tetrahydro-1,2-dimethylpyrimidine) (1.5 eq., 60 mmol) were added.

The tube was sealed with a Teflon screw valve. The tube was sealed with a Teflon screw valve. The reaction mixture was then irradiated with 4 x 30 W red LEDs (0.5 cm away from the tube, 660-670 nm) at 85 °C for 48 h. After the reaction was completed, the mixture was diluted with ethyl acetate after cooling to room temperature. The organic phases were washed with saturated ammonium chloride (3 × 100 mL), dried over anhydrous sodium sulfate, and concentrated under reduced pressure. The residue was purified by flash column chromatography using petroleum ether and ethyl acetate as eluent to afford Tetracaine.

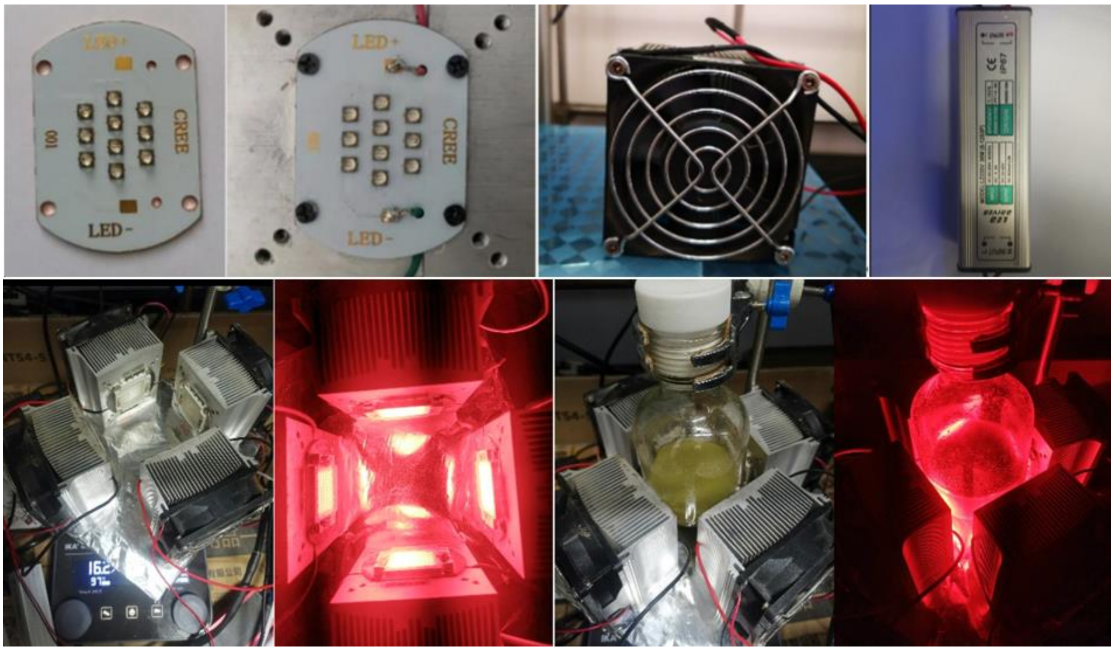

Figure S9. Pictures of the reaction setup in red light.

4.7 Procedure for the synthesis of aryl amines at gram-scale under simulate solar light

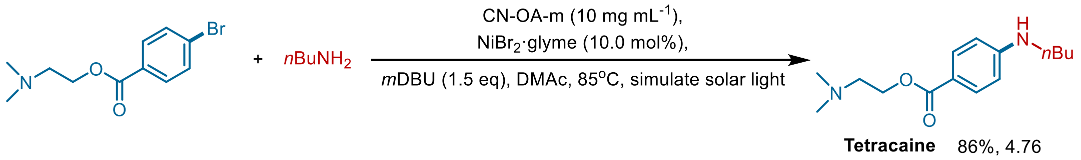

To an oven-dried 150 mL of storage tube were added $NiBr_2$•glyme (10.0 mol%), and 100 mL of DMAc with a magnetic stir bar under argon atmosphere. The mixture was evacuated and backfilled with Ar for 3 times. Then 2-(dimethylamino)ethyl 4-bromobenzoate (20 mmol), *n*-butylamine (40 mmol), CN-OA-m (10 mg/mL) and

*m*DBU (1,4,5,6-tetrahydro-1,2-dimethylpyrimidine) (1.5 eq., 60 mmol) were added. The tube was sealed with a Teflon screw valve. The tube was sealed with a Teflon screw valve. The reaction mixture was then irradiated with under simulate solar light at 85 °C for 48 h. After the reaction was completed, the mixture was diluted with ethyl acetate after cooling to room temperature. The organic phases were washed with saturated ammonium chloride (3 × 100 mL), dried over anhydrous sodium sulfate, and concentrated under reduced pressure. The residue was purified by flash column chromatography using petroleum ether and ethyl acetate as eluent to afford Tetracaine

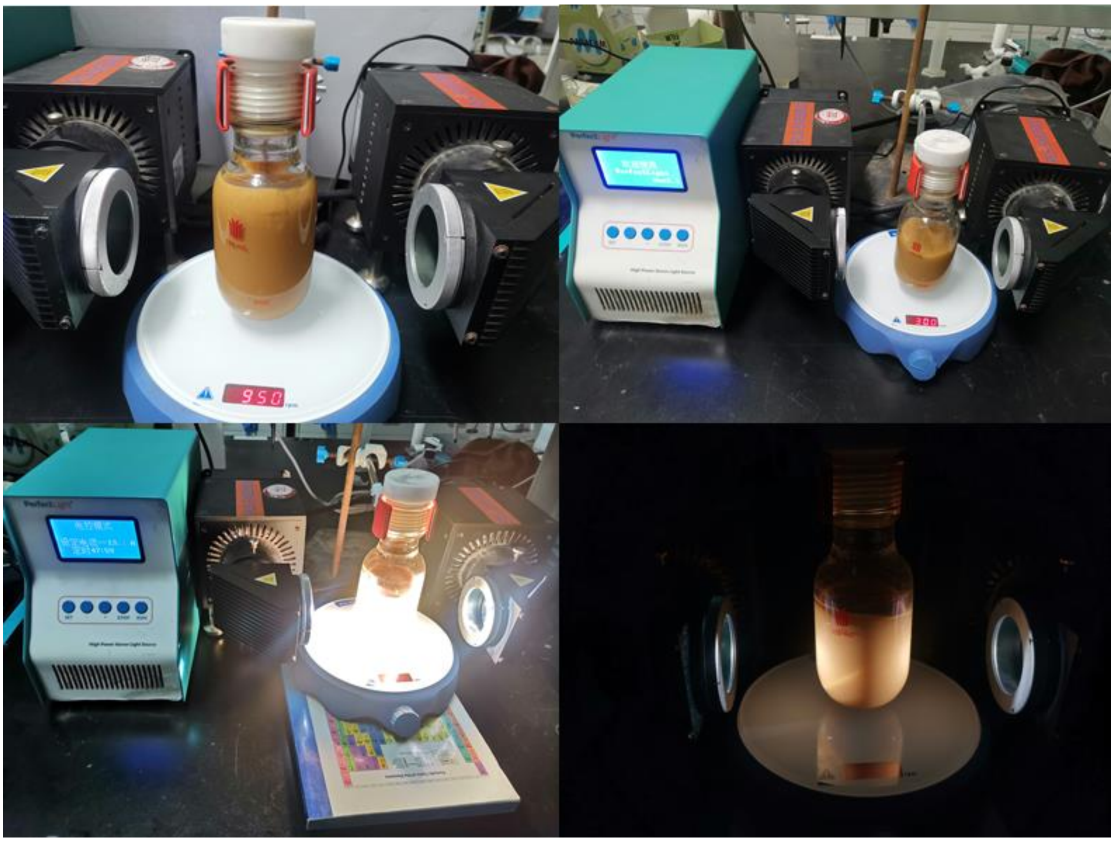

Figure S10. Pictures of gram-scale reaction setup in simulate solar light.

5. Multiple recovery steps for the reuse of CN-OA-m

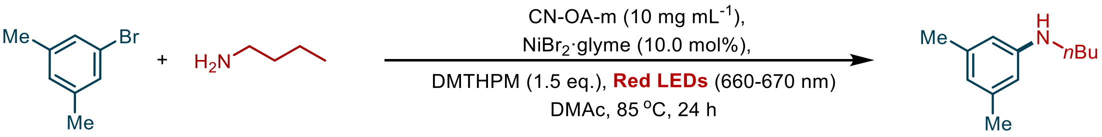

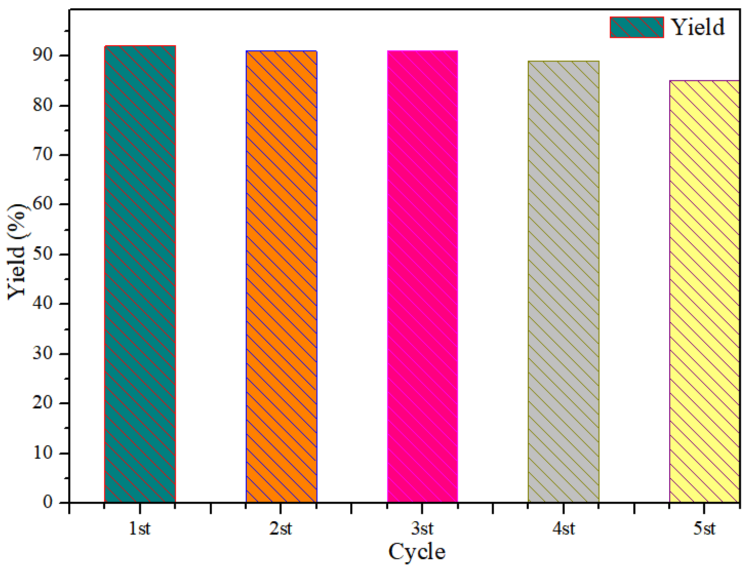

Figure S11. Recycling experiment performed under standard condition for 24 hours.

To an oven-dried 10 mL of storage tube were added $NiBr_2$•glyme (10.0 mol%), 1 mL of DMAc and a magnetic stir bar under argon atmosphere. The mixture was evacuated and backfilled with argon for 3 times. Then 5-bromo-m-xylene (0.2 mmol), butylamine (0.4 mmol), CN-OA-m (10 mg/mL) and *m*DBU (1,4,5,6-tetrahydro-1,2-dimethylpyrimidine) (1.5 eq, 0.3 mmol) were added. The tube was sealed with a Teflon screw valve. The reaction mixture was then irradiated with 10 W red LEDs (0.5 cm away from the tube, 660-670 nm) at 85 °C. After the reaction was completed, the mixture was diluted with ethyl acetate after cooling to room temperature. Add deionized water, centrifuge and recovered by filtration to obtain CN-OA-m, and it will be reacted with the raw material again for the next reaction, and it will be recycled multiple times. At the same time, 1,3-benzodioxole (0.2 mmol) was added to the reaction solution. The resulting mixtures were diluted with 5 mL $CHCl_3$, the organic phases were washed with saturated ammonium chloride (3 × 10 mL), dried over anhydrous sodium sulfate, and concentrated under reduced pressure and the residue was analyzed by ^1H NMR with $CDCl_3$ as solvent to give the yield of the reaction.

6. Effect of temperature on reaction under red light excitation

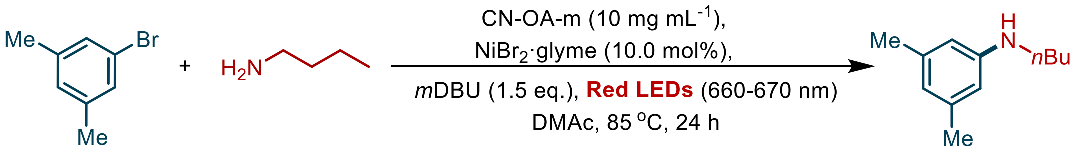

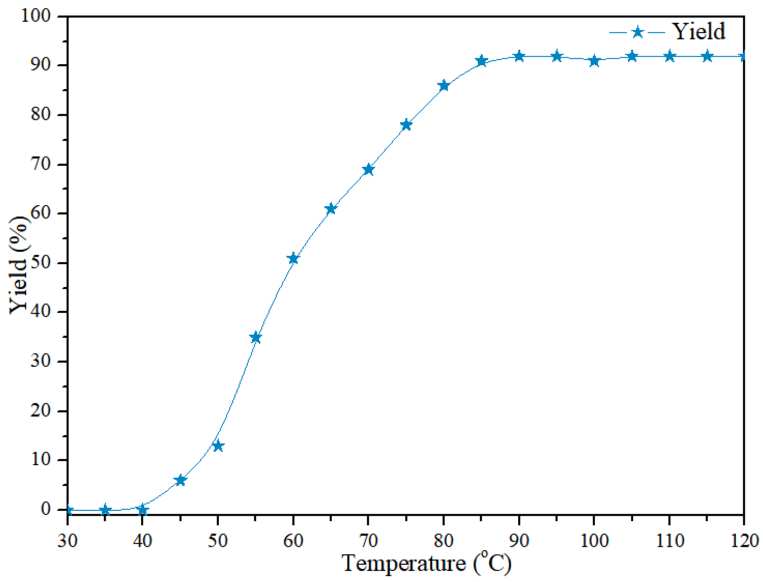

Figure S12. Effect of temperature on reaction.

To an oven-dried 10 mL of storage tube were added $NiBr_2$•glyme (10.0 mol%), 1 mL of DMAc and a magnetic stir bar under argon atmosphere. The mixture was evacuated and backfilled with argon for 3 times. Then the 5-bromo-m-xylene (0.2 mmol), butylamine (0.4 mmol), CN-OA-m (10 mg/mL) and *m*DBU (1,4,5,6-tetrahydro-1,2-dimethylpyrimidine) (1.5 eq, 0.3 mmol) were added. The tube was sealed with a Teflon screw valve. The reaction mixture was then irradiated with 10 W red LEDs (0.5 cm away from the tube, 660-670 nm) at 85 °C. After the reaction was completed, after cooling to room temperature, 1,3-benzodioxole (0.2 mmol) was added to the reaction solution. The resulting mixtures were diluted with 5 mL $CHCl_3$, the organic phases were washed with saturated ammonium chloride (3 × 10 mL), dried over anhydrous sodium sulfate, and concentrated under reduced pressure and the residue was analyzed by ^{1}H NMR with $CDCl_3$ as solvent to give the yield of the reaction and the average of three parallel reactions was taken.

7. Experiments on electronic and hole scavengers

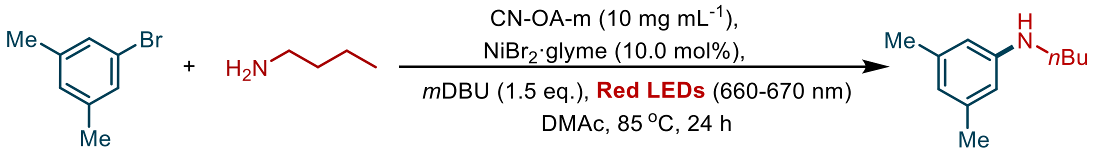

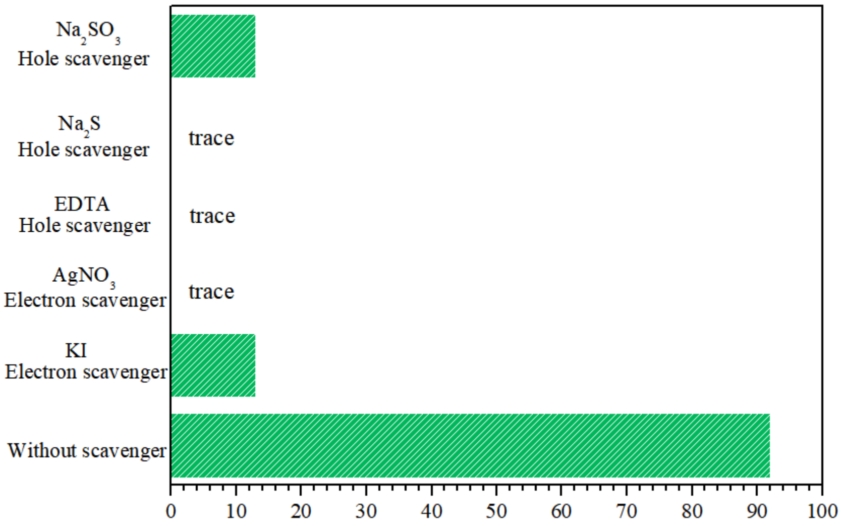

Figure S13. Experiments on electronic and hole scavengers.

To an oven-dried 10 mL of storage tube were added $NiBr_2$·glyme (10.0 mol%), 1 mL of DMAc and a magnetic stir bar under argon atmosphere. The mixture was evacuated and backfilled with argon for 3 times. Then the 5-bromo-m-xylene (0.2 mmol), butylamine (0.4 mmol), CN-OA-m (10 mg/mL) $AgNO_3$, KI or Na_2S/Na_2SO_3, EDTA (0.2 mmol) and *m*DBU (1,4,5,6-tetrahydro-1,2-dimethylpyrimidine) (1.5 eq., 0.3 mmol) were added. The tube was sealed with a Teflon screw valve. The reaction mixture was then irradiated with 10 W red LEDs (0.5 cm away from the tube, 660-670 nm) at 85 °C. After the reaction was completed, after cooling to room temperature, 1,3-benzodioxole (0.2 mmol) was added to the reaction solution. The resulting mixtures were diluted with 5 mL $CHCl_3$, the organic phases were washed with saturated ammonium chloride (3 × 10 mL), dried over anhydrous sodium sulfate, and concentrated under reduced pressure and the residue was analyzed by ^{1}H NMR with $CDCl_3$ as solvent to give the yield of the reaction.

8. Time curves

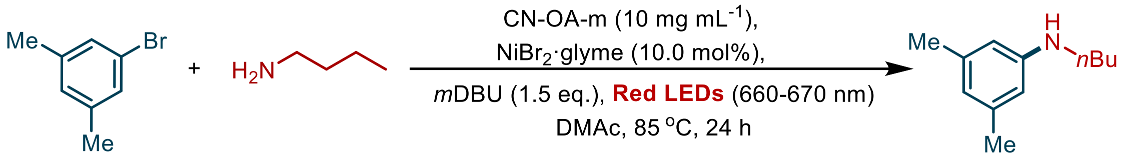

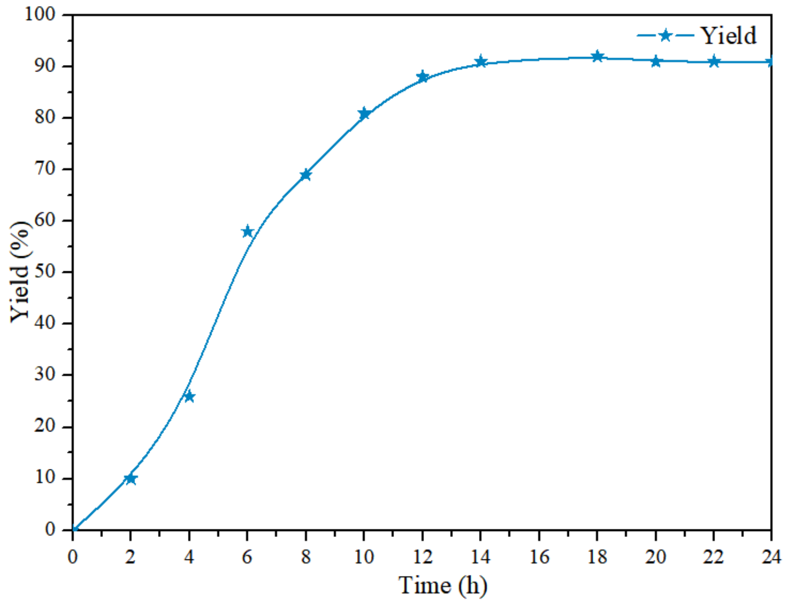

Figure S14. Effect of time on reaction.

To an oven-dried 10 mL of storage tube were added $NiBr_2$•glyme (10.0 mol%), 1 mL of DMAc and a magnetic stir bar under argon atmosphere. The mixture was evacuated and backfilled with argon for 3 times. Then the 5-bromo-m-xylene (0.2 mmol), butylamine (0.4 mmol), CN-OA-m (10 mg/mL) and *m*DBU (1,4,5,6-tetrahydro-1,2-dimethylpyrimidine) (1.5 eq, 0.3 mmol) were added. The tube was sealed with a Teflon screw valve. The reaction mixture was then irradiated with 10 W red LEDs (0.5 cm away from the tube, 660-670 nm). After the reaction was completed, after cooling to room temperature, 1,3-benzodioxole (0.2 mmol) was added to the reaction solution. The resulting mixtures were diluted with 5 mL $CHCl_3$, the organic phases were washed with saturated ammonium chloride (3 × 10 mL), dried over anhydrous sodium sulfate, and concentrated under reduced pressure and the residue was analyzed by ^{1}H NMR with $CDCl_3$ as solvent to give the yield of the reaction. and tests were performed every 2 hours and the average of three parallel reactions was taken.

9. The catalytic reaction of alternating the light irradiation

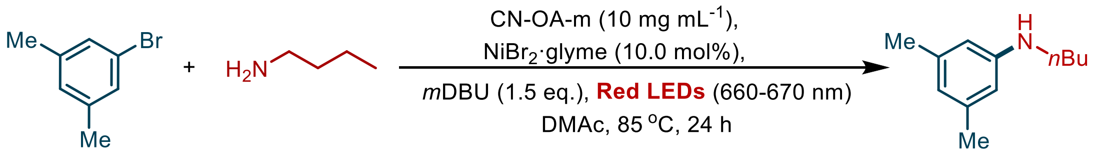

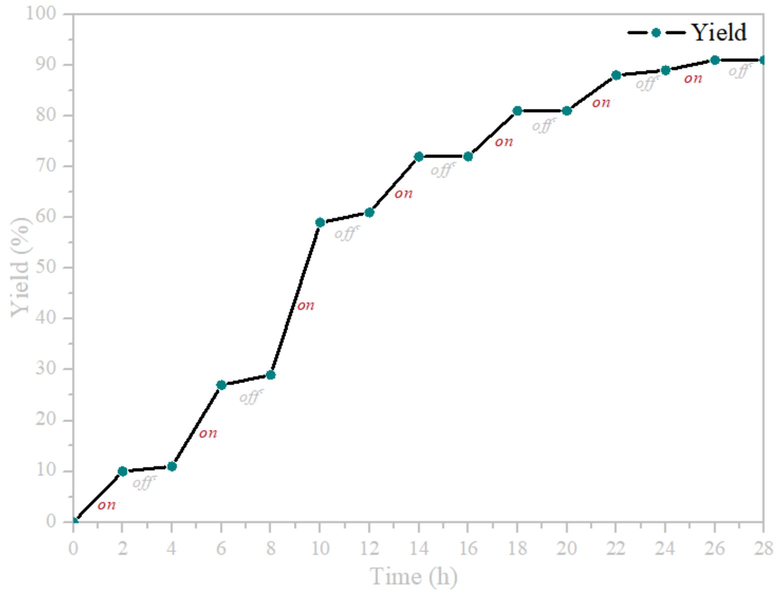

Figure S15. On/off experiments with 660-670 nm light.

To an oven-dried 10 mL of storage tube were added $NiBr_2$•glyme (10.0 mol%), 1 mL of DMAc and a magnetic stir bar under argon atmosphere. The mixture was evacuated and backfilled with argon for 3 times. Then the 5-bromo-m-xylene (0.2 mmol), butylamine (0.4 mmol), CN-OA-m (10 mg/mL) (0.2 mmol) and *m*DBU (1,4,5,6-tetrahydro-1,2-dimethylpyrimidine) (1.5 eq., 0.3 mmol) were added. The tube was sealed with a Teflon screw valve. The reaction mixture was then irradiated with 10 W red LEDs (0.5 cm away from the tube, 660-670 nm) at 85 °C. After a certain time, one was cooled to room temperature, and the other was wrapped with foil paper to continue stirring for 2 hours at 85 °C. Cooling to room temperature, 1,3-benzodioxole (0.2 mmol) was added to the reaction solution. The resulting mixtures were diluted with 5 mL $CHCl_3$, the organic phases were washed with saturated ammonium chloride (3 × 10 mL), dried over anhydrous sodium sulfate, and concentrated under reduced pressure and the residue was analyzed by ^{1}H NMR with $CDCl_3$ as solvent to give the yield of the reaction. (Each reaction was repeated three times and the average yields were used in selected 2h respectively.).

10. Mechanistic investigations

10.1 UV-Vis absorption spectra of different C_3N_4

UV-Vis absorption spectra were collected on a PerkinElmer Lambda 365 UV-VIS Spectrophotometer at room temperature.

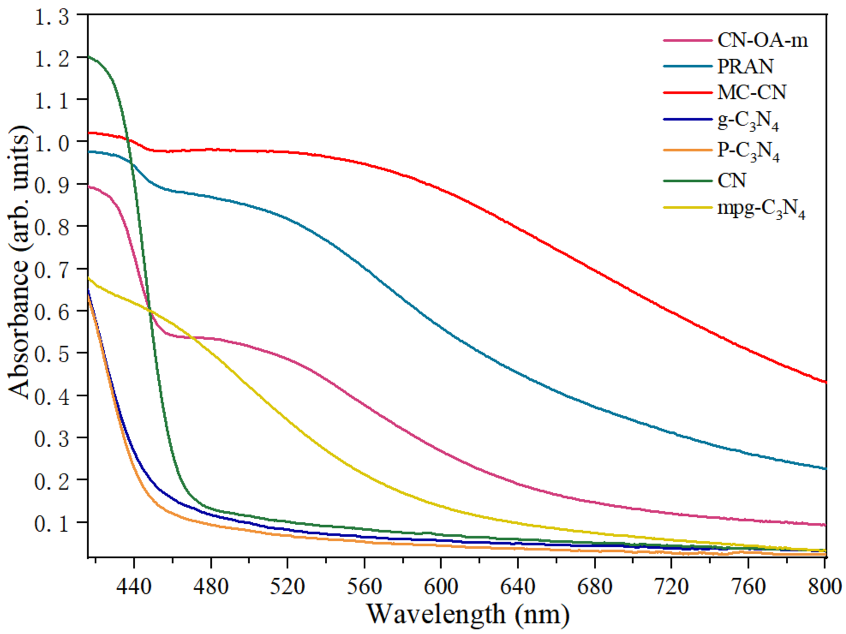

Figure S16. Absorption spectrum of different C_3N_4.

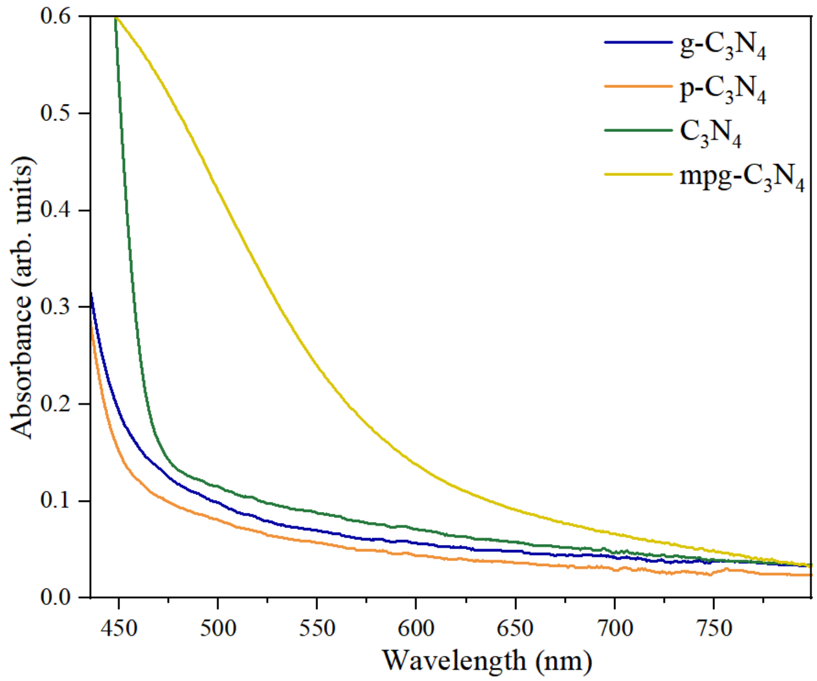

Figure S17. Absorption spectrum of division of p-C_3N_4, g-C_3N_4, mpg-C_3N_4, and C_3N_4.

10.2 XPS spectra of different C_3N_4

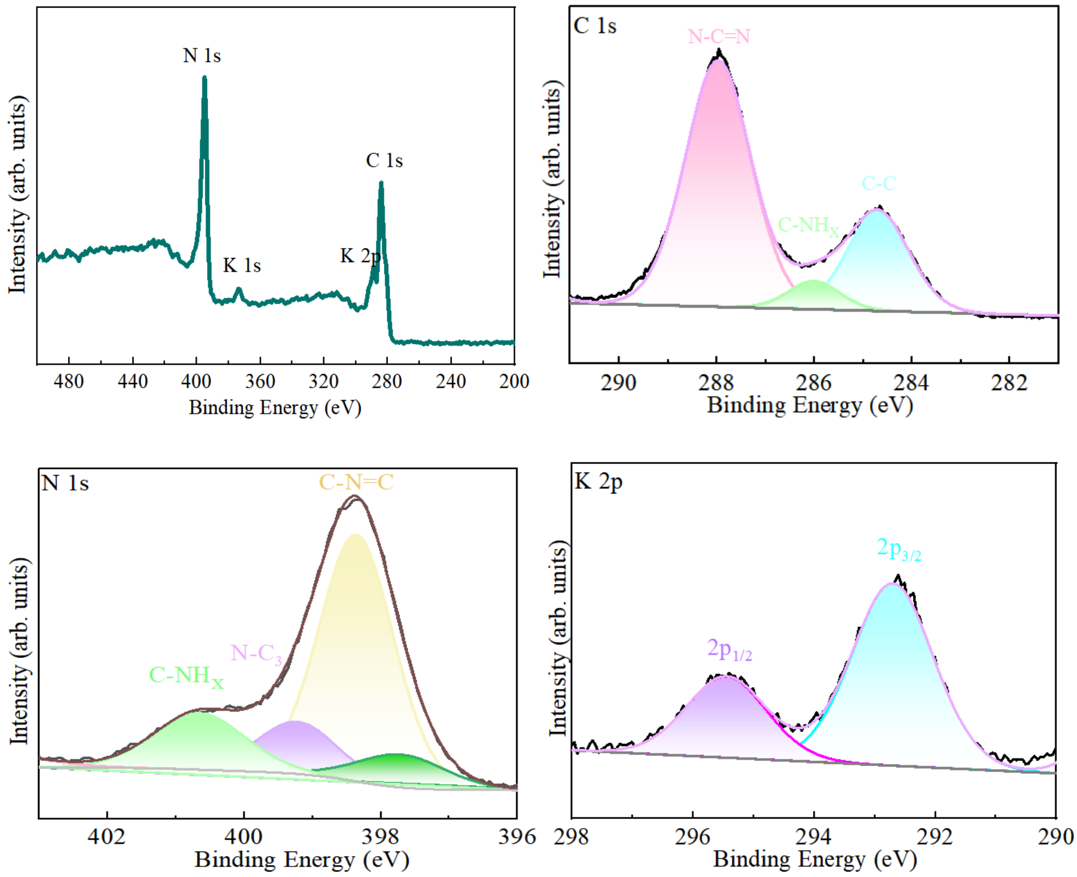

Figure S18. XPS spectra of CN-OA-m: survey spectrum, C 1s, N 1s, and (e) K 2p high-resolution spectra.

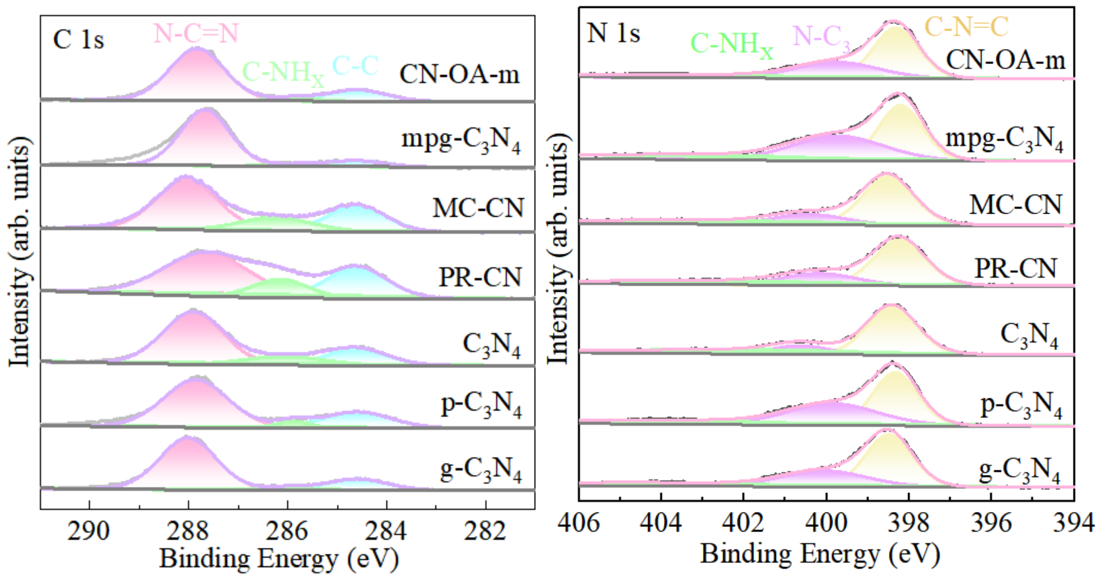

Figure S19. XPS spectra of different C_3N_4, C 1s, N 1s high-resolution spectra.

10.3 N_2 adsorption/desorption isotherm and Pore size distribution of different C_3N_4

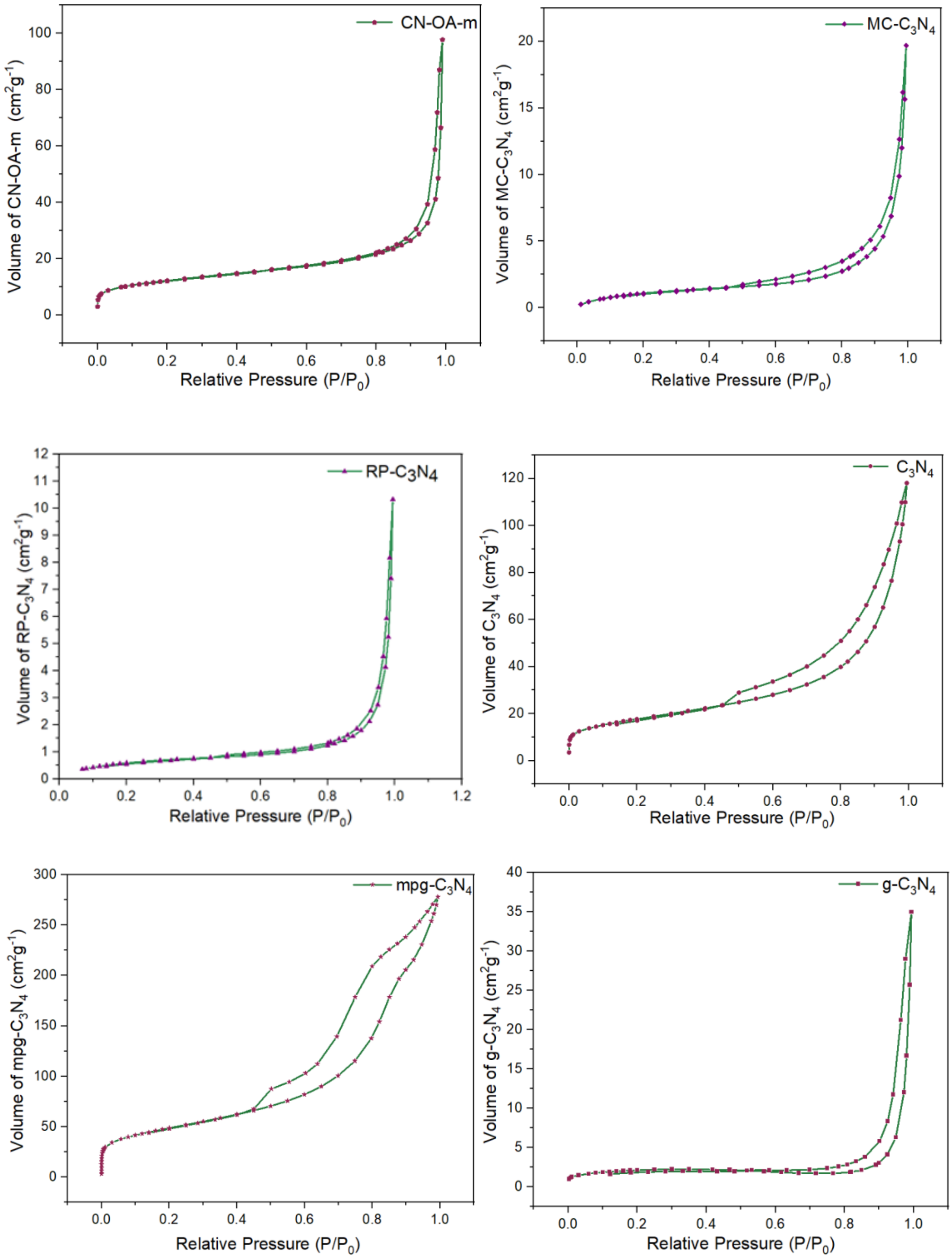

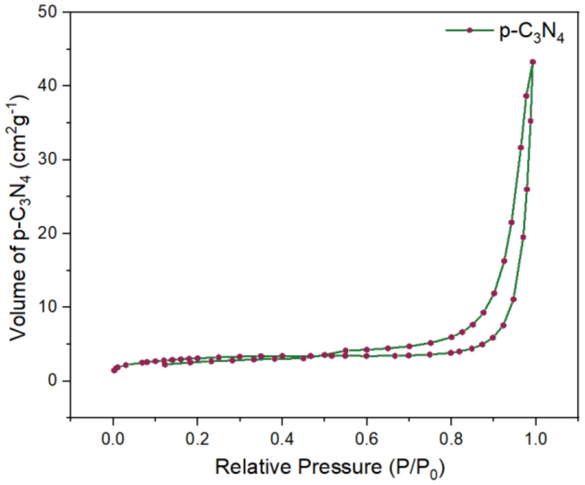

Figure S20. N_2 adsorption/desorption isotherm of different C_3N_4。

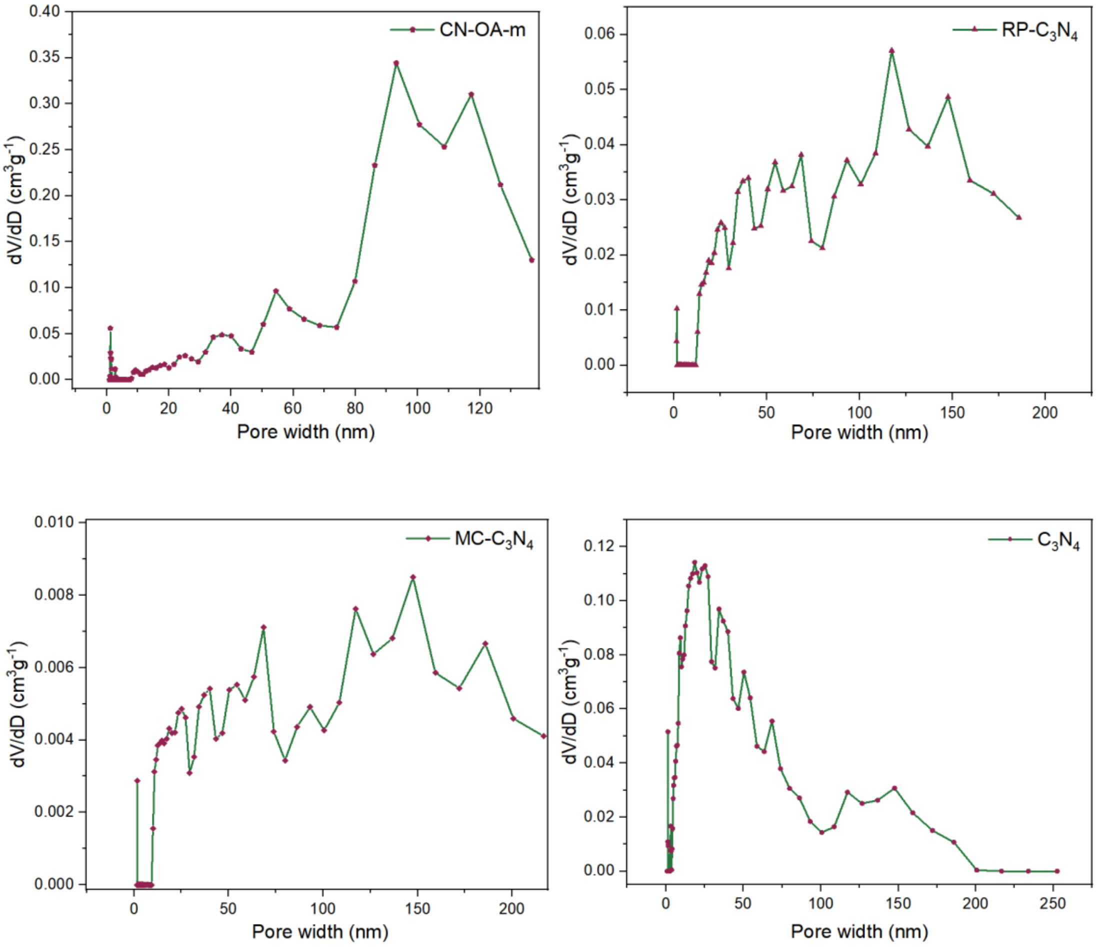

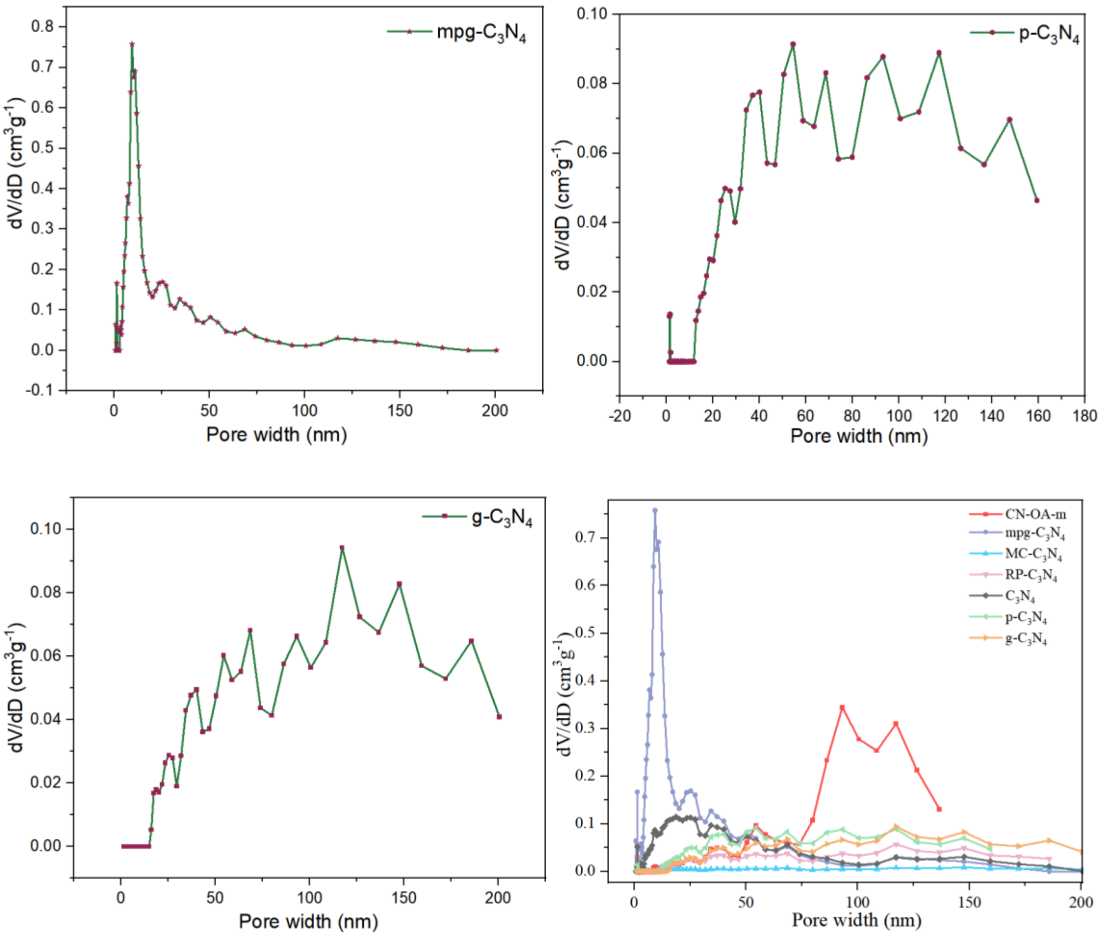

Figure S21. Pore size distribution from the N_2 adsorption branch of different C_3N_4 at 77 K.

Table S15. BET specific surface area, pore diameter and total pore volume.

Sample	Specific surface area (m^2g^{-1})	Pore diameter (nm)	Total pore volume (ccg^{-1})
CN-OA-m	43.80	13.80	0.003037
C_3N_4	63.70	11.46909	0.002461
p-C_3N_4	11.1311	24.05477	0.001051
g-C_3N_4	7.6994	28.11851	0.000792
mpg-C_3N_4	174.9604	9.83135	0.005687
RP-C_3N_4	2.45	17.17	0.000985
MC-C_3N_4	2.14	13.44	0.000152

10.4 Photoluminescence (PL) spectra of different C_3N_4

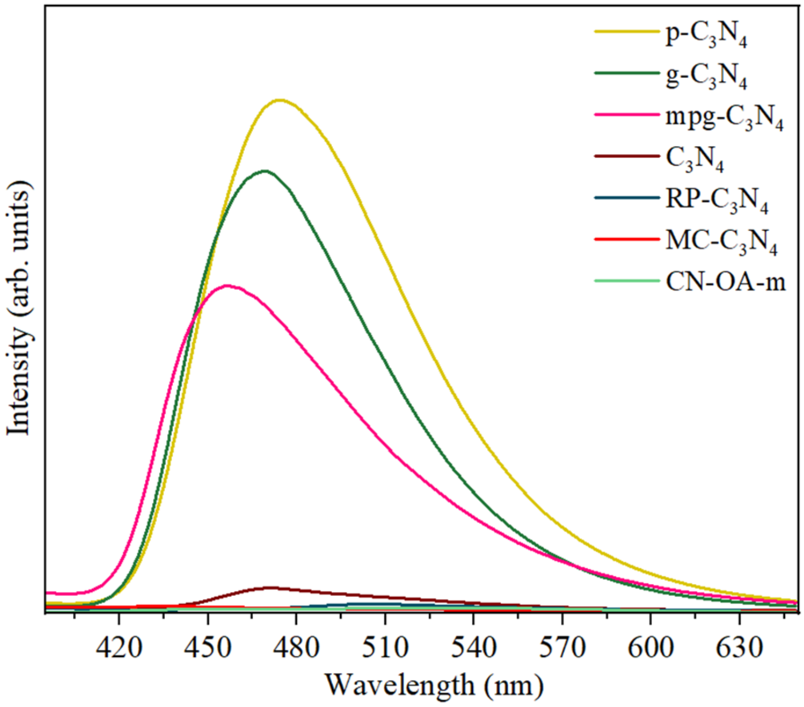

Figure S22. The photoluminescence (PL) spectra of C_3N_4.

10.5 Time-resolved fluorescence spectr

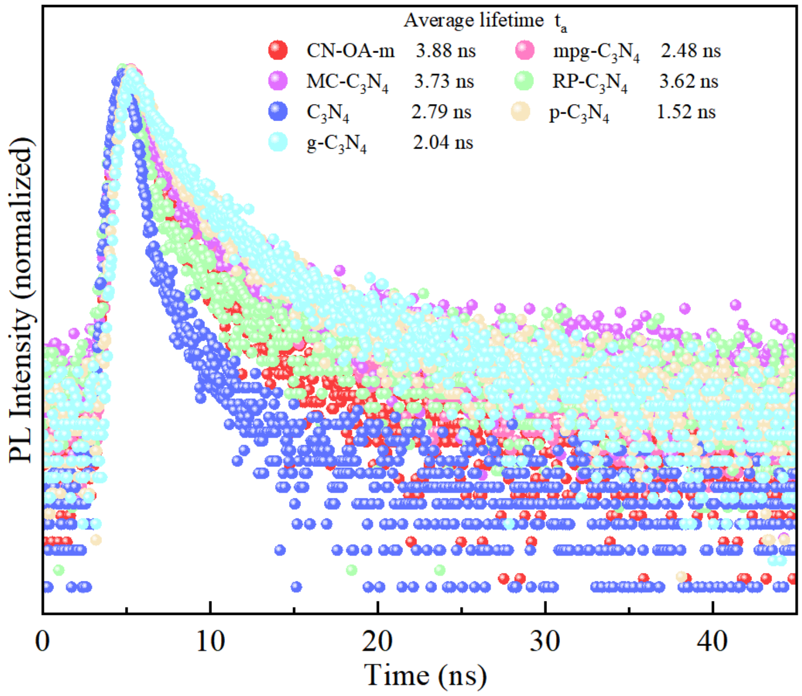

Figure S23. Time-resolved photoluminescence (TRPL) spectra of different C_3N_4.

Table S16. Paramaters of fluorescence decay curves for CN-OA-m, mpg-C_3N_4, MC-C_3N_4, RP-C_3N_4, C_3N_4, p-C_3N_4, g-C_3N_4 samples.

Sample	τ_1 (ns) (A_1%)	τ_2 (ns) (A_2%)	τ_{av} (ns)	Ex (nm)	Em (nm)
C_3N_4-OA-m	2.81(97.86)	13.87 (2.14)	3.88	340	533
mpg-C_3N_4	1.86 (98.26)	9.43 (1.74)	2.48	340	457
MC-C_3N_4	2.42(93.81)	9.03 (6.19)	3.73	340	440
PR-C_3N_4	2.33(93.87)	8.82 (6.13)	3.62	340	455
C_3N_4	2.15(98.40)	10.70(1.60)	2.79	340	471
p-C_3N_4	1.19(97.93)	5.11 (2.07)	1.52	340	474
g-C_3N_4	1.43(95.63)	5.49 (4.37)	2.04	340	469

$$\tau_{av} = \frac{A_1{\tau_1}^2 + A_2{\tau_2}^2}{A_1\tau_1 + A_1\tau_1}$$

10.6 Photocurrent responses and the electrochemical impedance spectra

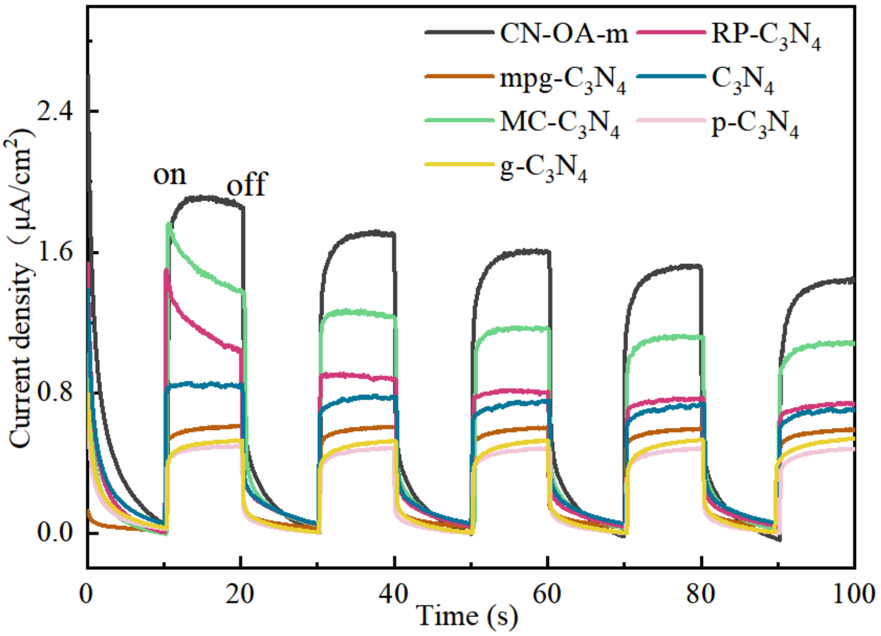

Figure S24. Transient photocurrent response of different C_3N_4.

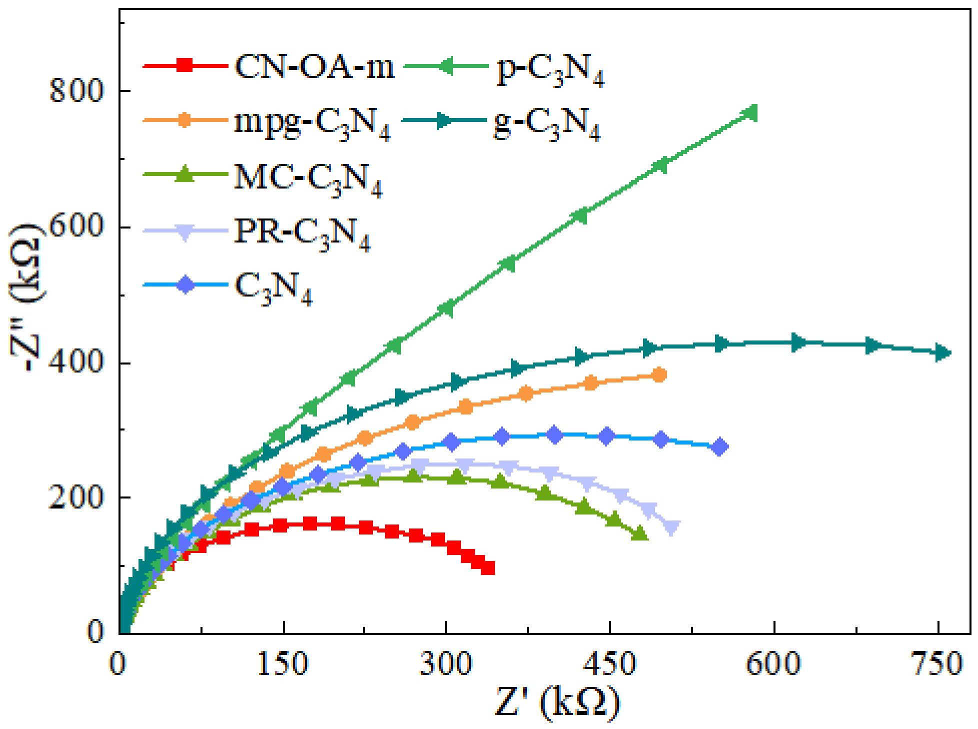

Figure S25 The electrochemical impedance spectra (EIS) of different C_3N_4 electrodes.

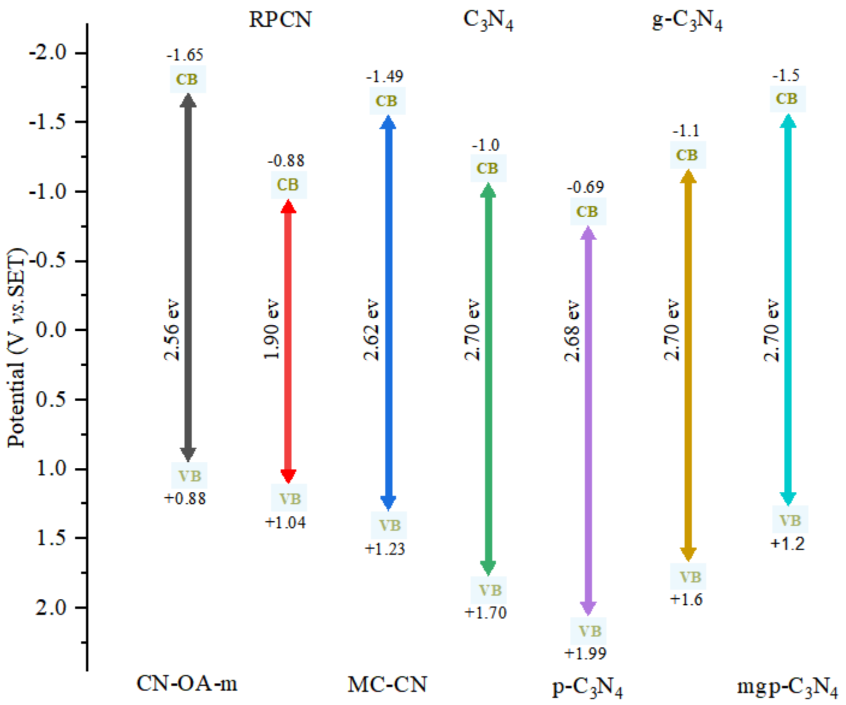

Figure S26. Electrochemical potential diagrams.

10.7 Amidation of aryl halide catalyzed via Ni(I) complex under red light

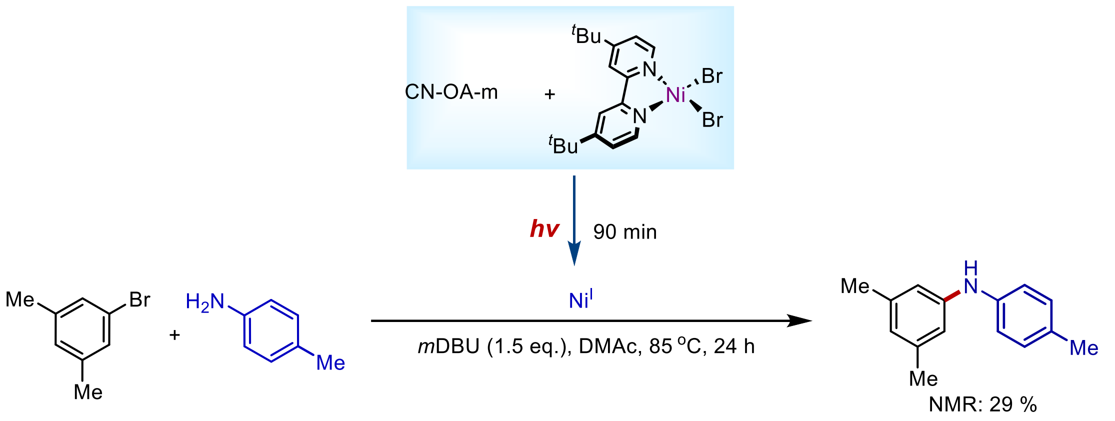

In a nitrogen-filled glove box, a magnetic stir bar, CN-OA-m (10 mg), Ni(*d*-tbbpy)Br_2 (10.0mol%), 1 mL of DMAc and a magnetic stir were placed into an oven-dried 10 mL storage tube with a high vacuum valve. The tube was sealed with a Teflon screw valve and removed from the glovebox. The reaction solution was then irradiated with red light for 90 min h at 85 °C. Then it was quickly frozen in liquid nitrogen and 5-bromo-m-xylene (0.2 mmol), *p*-Toluidine (0.4 mmol), and *m*DBU (1,4,5,6-tetrahydro-1,2-dimethylpyrimidine) were added in succession under nitrogen atmosphere. The tube was wrapped with foil paper and then stirred for 24 h at 85 °C. After cooling to room temperature, 1, 3-benzodioxole (0.2 mmol) was added to the reaction solution, the mixture was diluted with ethyl acetate. The organic phases were washed with saturated ammonium chloride (3 × 10 mL), dried over anhydrous sodium sulfate, and concentrated under reduced pressure. The residue was analyzed by 1H NMR with CDCl3 as solvent to give the yield of the reaction.

11. The EPR experiment spectroscopic

11.1 Photoinduced dtbbpy-NiBr_2 with CN-OA-m

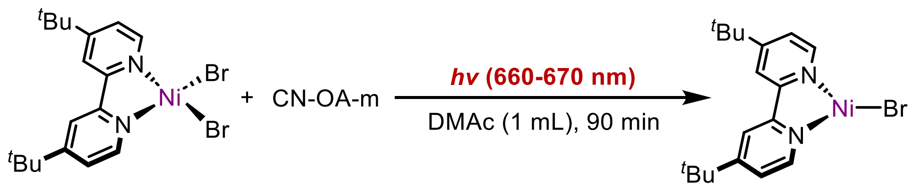

In a nitrogen-filled glove box, a magnetic stir bar, *d*-tbbpy-NiBr_2 (10 mg) with CN-

OA-m (4 mg), and 1 mL of DMAc were placed into an oven-dried EPR tube. The EPR tube was removed from the glovebox and irradiated with for 10 W red LEDs for 90 min and quickly frozen in liquid nitrogen. The EPR spectrum was collected on a frozen solution at 100 K.

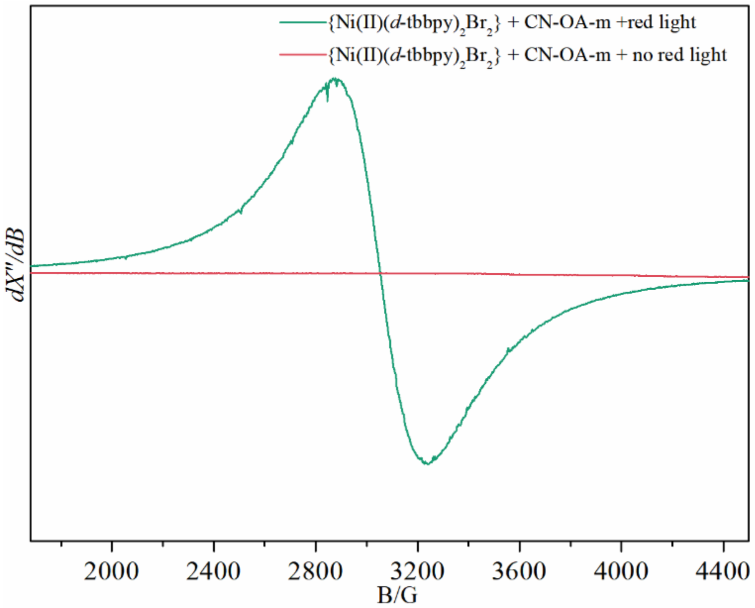

Figure S27. (a) EPR spectrum of Ni(I) species. Temperature = 100 K. Spectroscopic parameters: g = 2.204. Microwave frequency = 9.380 GHz, power = 10 Mw.

11.2 Photoinduced CN-OA-m generates hole-electron pairs

In a nitrogen-filled glove box, a magnetic stir bar, CN-OA-m (4 mg), and 1 mL of DMAc were placed into an oven-dried EPR tube. The EPR tube was removed from the glovebox and irradiated with for 10 W red LEDs for 10 min. EPR spectra was acquired at room temperature.

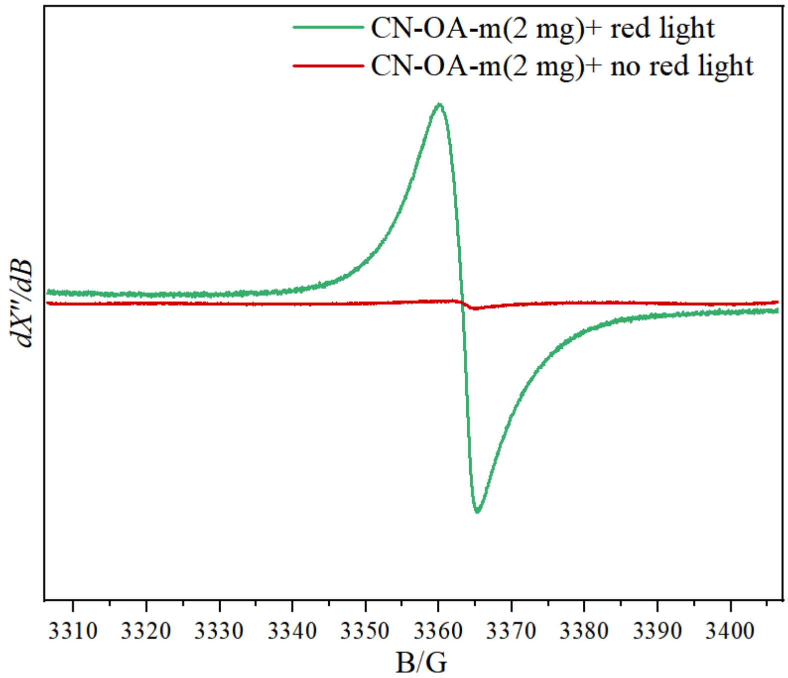

Figure S28. EPR spectrum (g = 2.003).

12. Characterization data of CN-OA-m before and after recovery

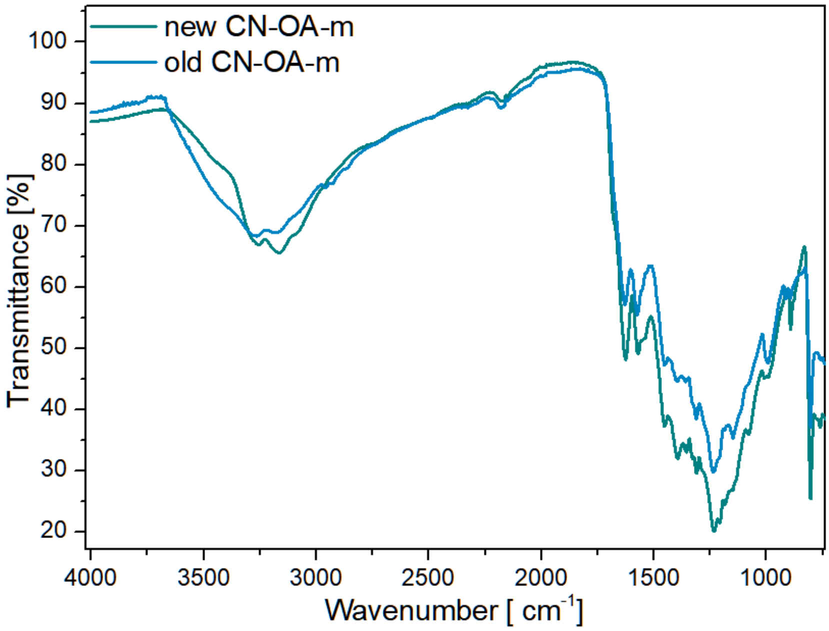

Figure S29. FTIR spectra of CN-OA-m new and recycle.

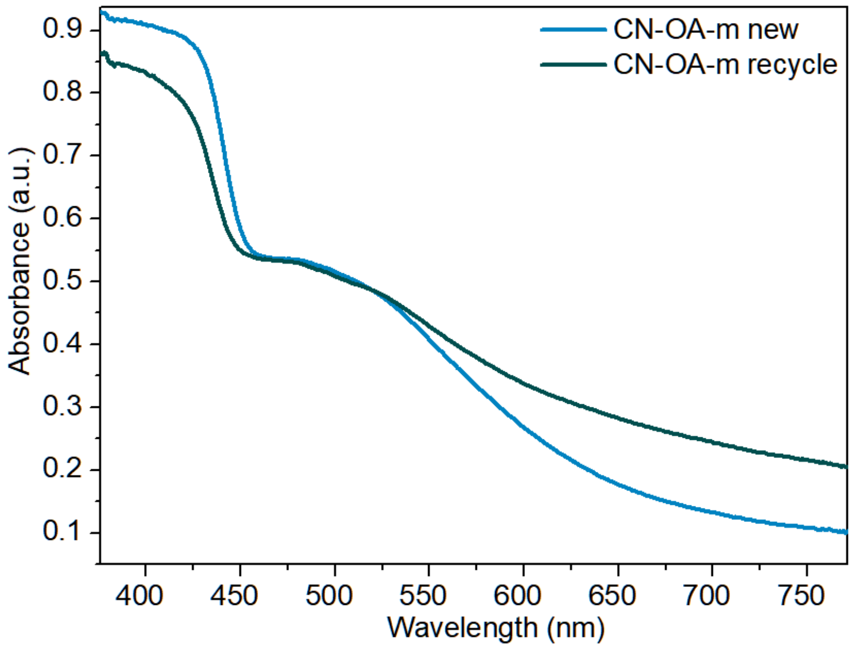

Figure S30. UV-vis spectra of CN-OA-m new and recycle.

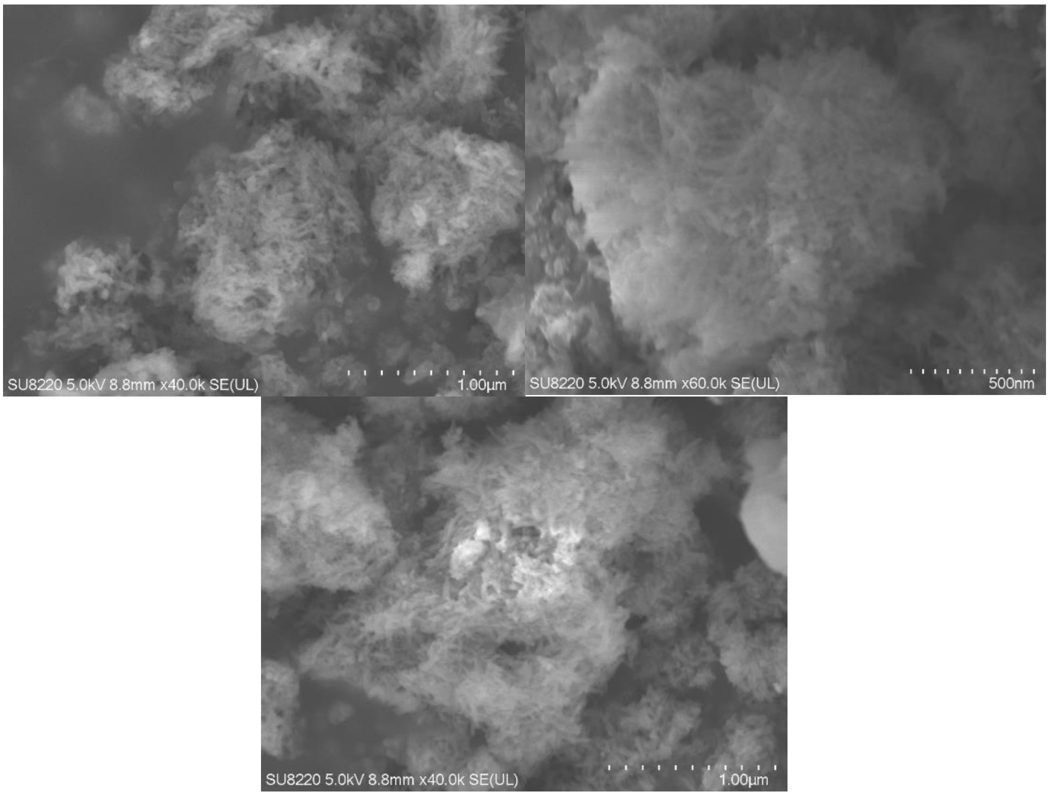

Figure S31. SEM images of new CN-OA-m.

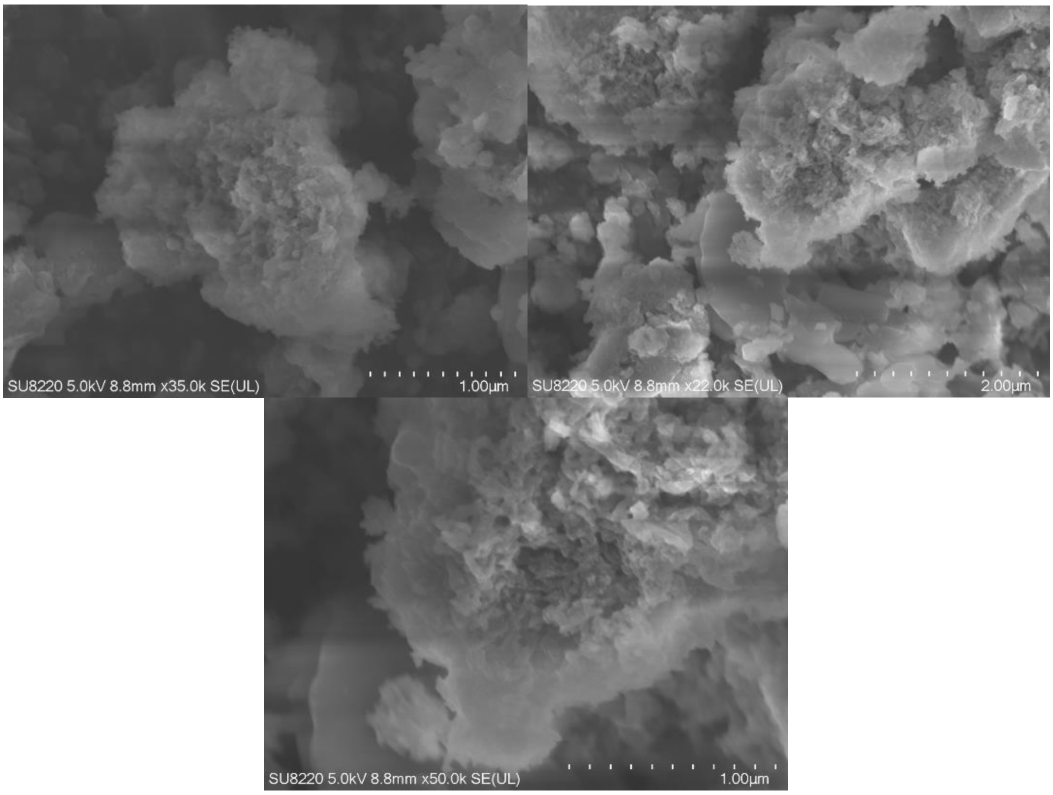

Figure S32. SEM images of recovered CN-OA-m.

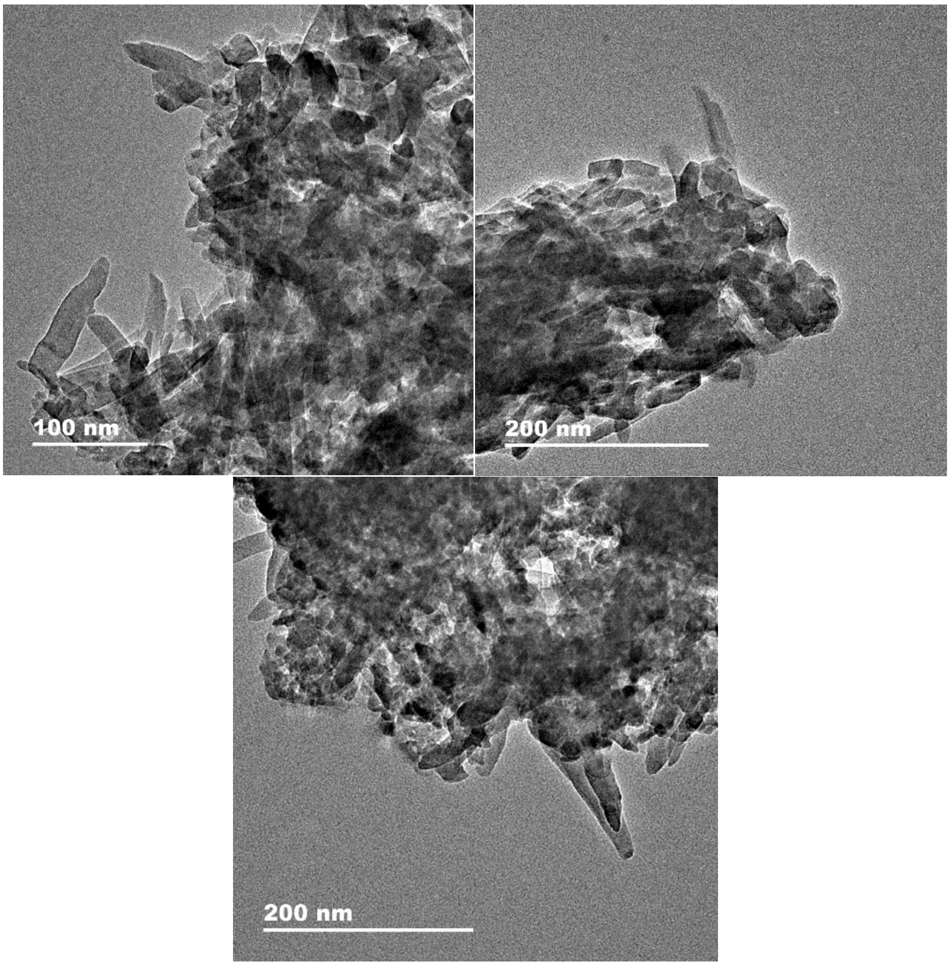

Figure S33. TEM images of new CN-OA-m.

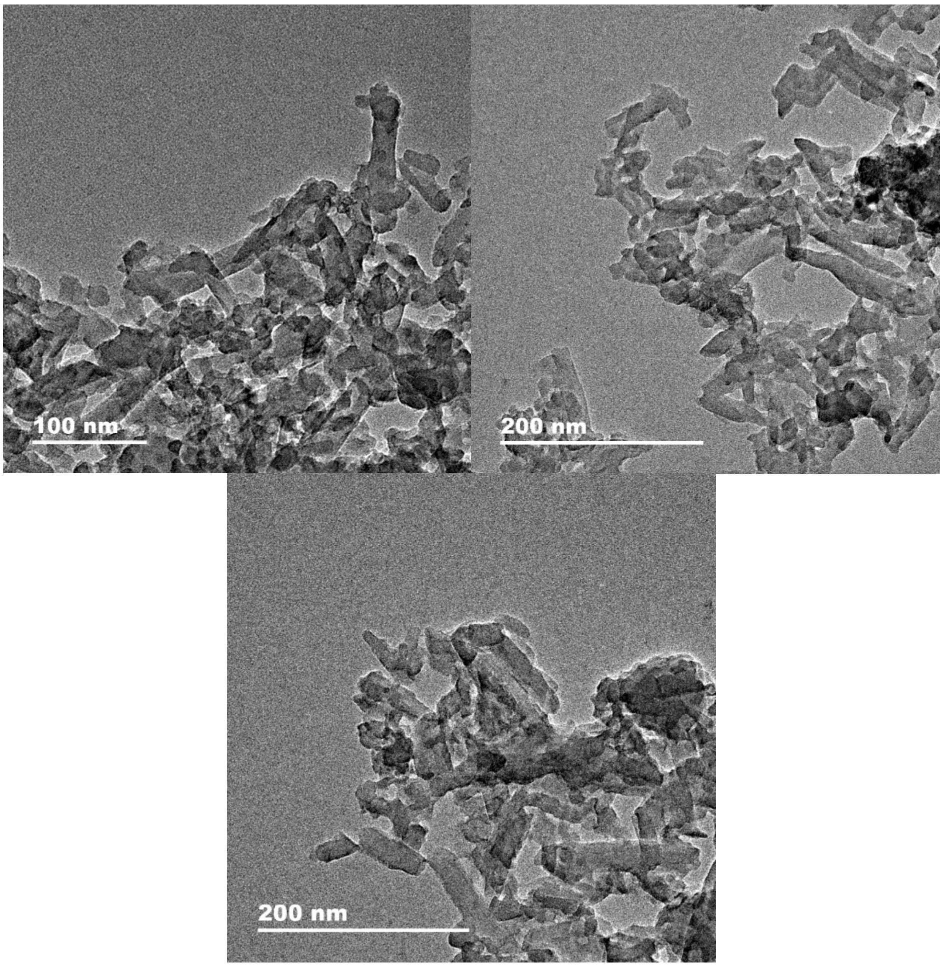

Figure S34. TEM images of recovered CN-OA-m.

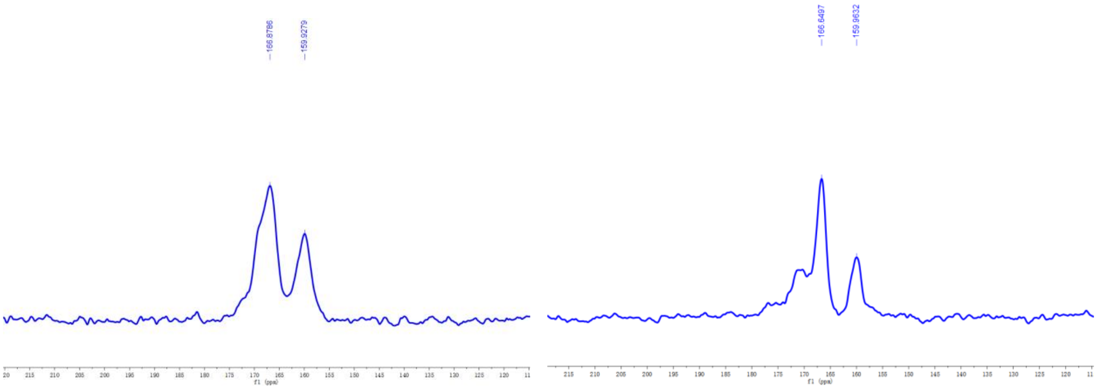

Figure S35. Solid-state ^{13}C NMR spectra new (left) and recovered (right).

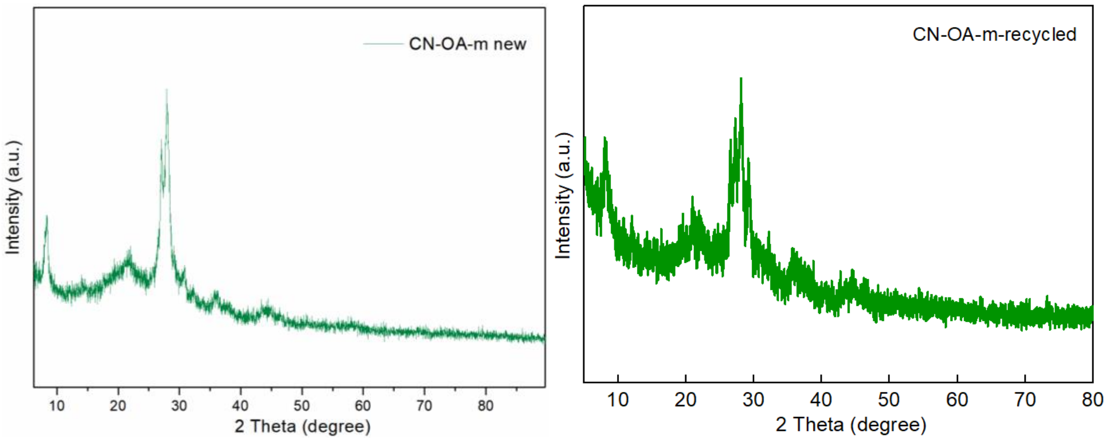

Figure S36. XRD patterns new (left) and recovered (right).

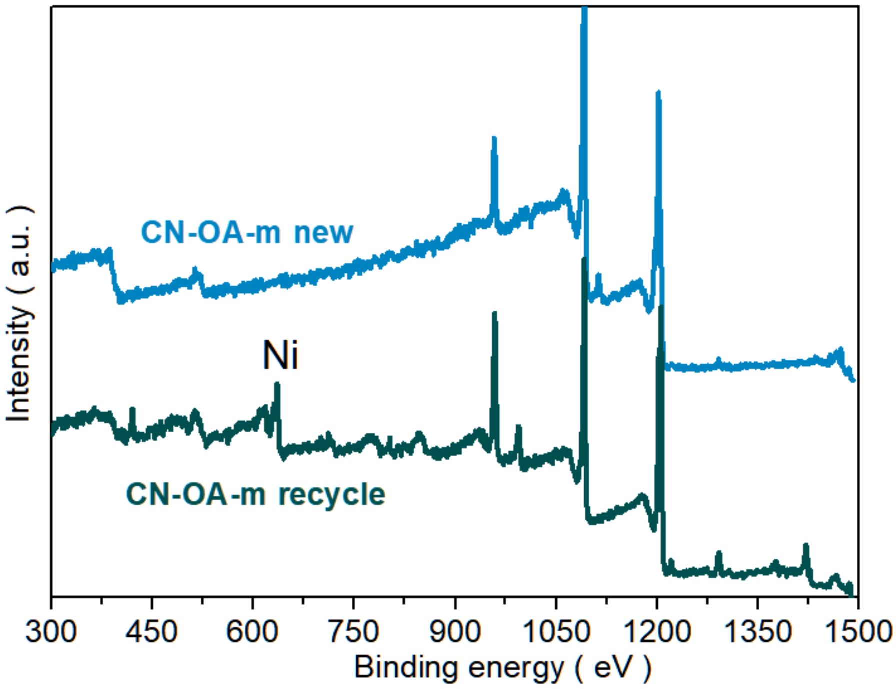

Figure S37. XPS spectra of CN-OA-m new and recycle.

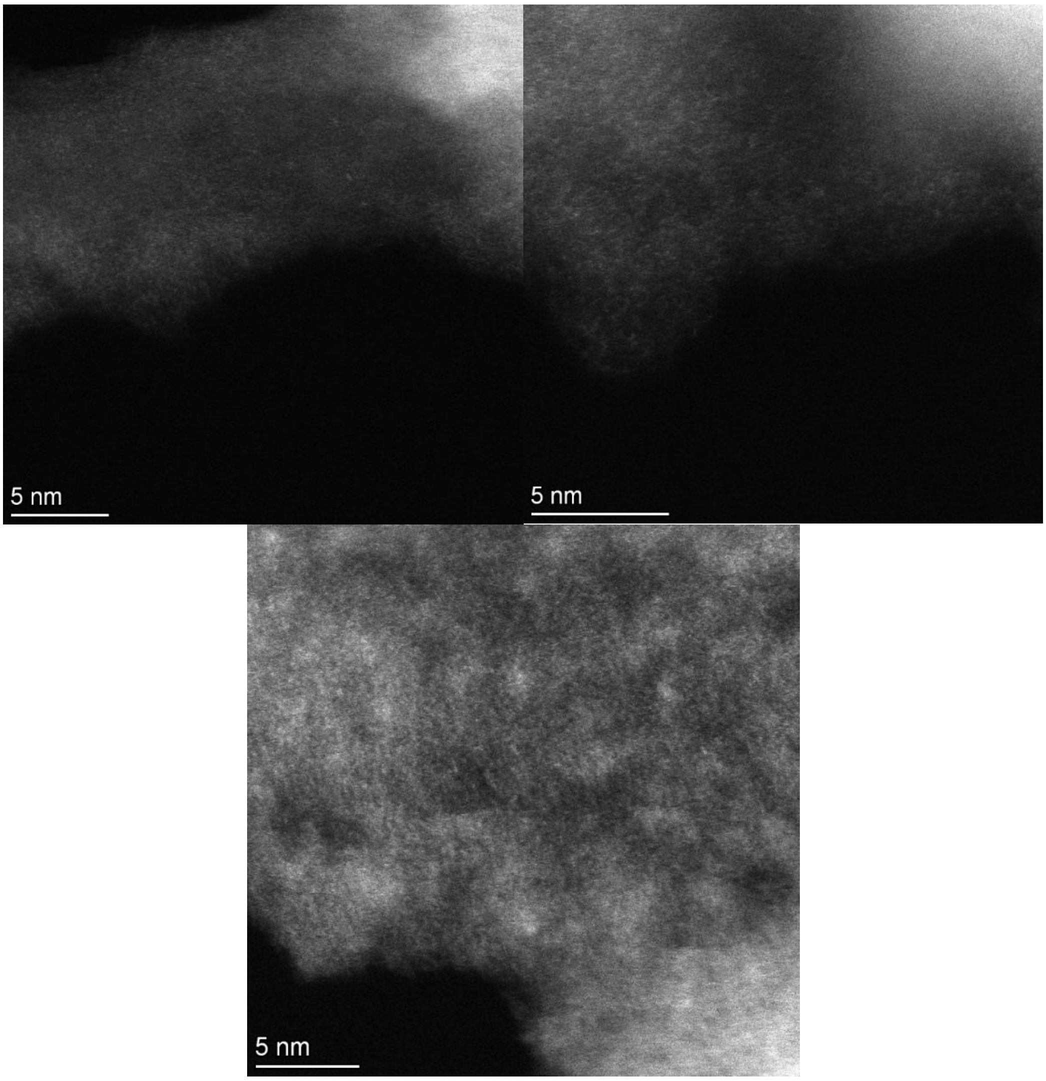

Figure S38. HAADF-STEM images of recovered CN-OA-m.

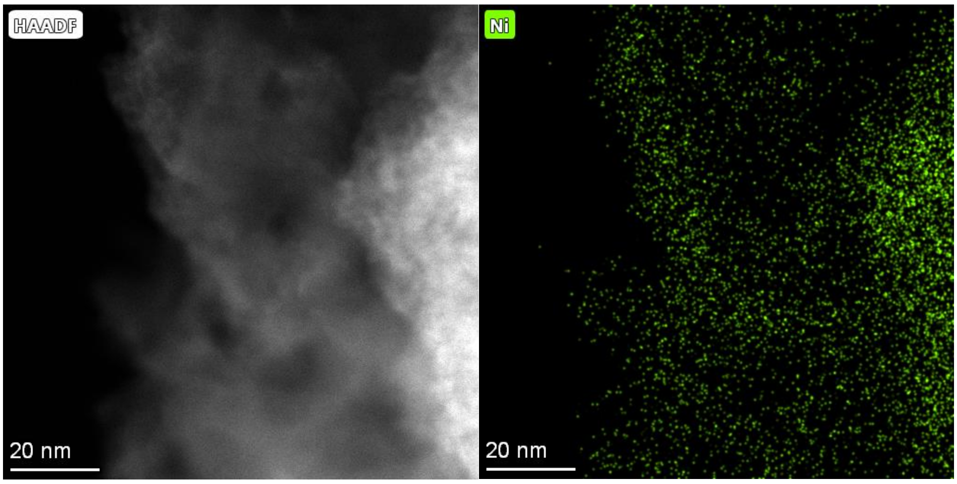

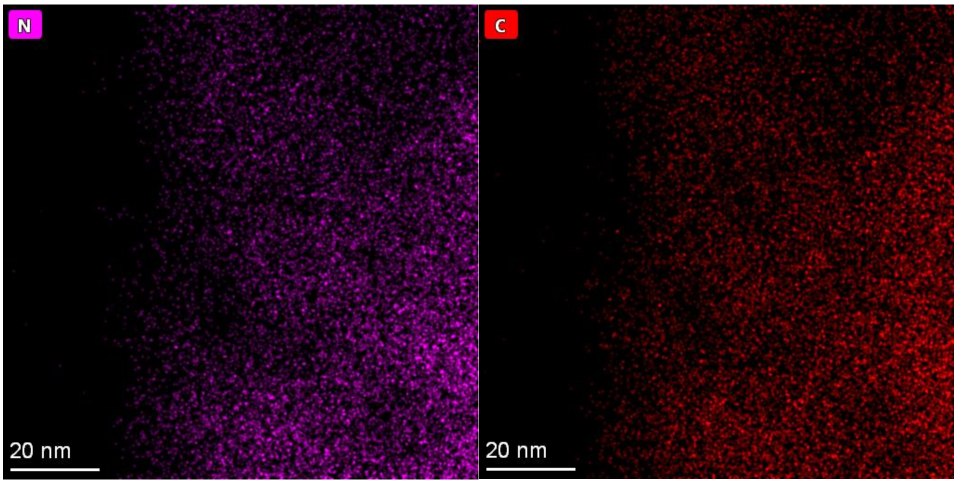

Figure S39. EDS mappings of recovered CN-OA-m show the homogeneous distribution of N, C and Ni elements in the polymers.

13. Redox potentials of different organic bases

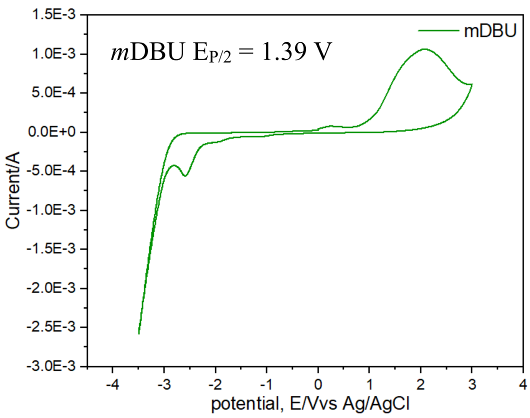

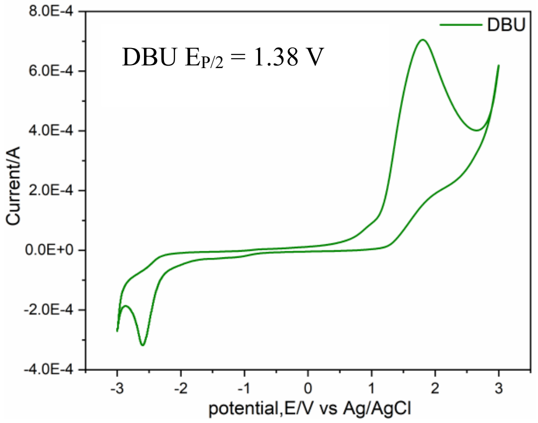

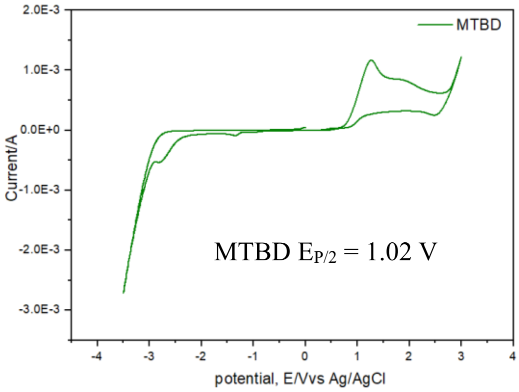

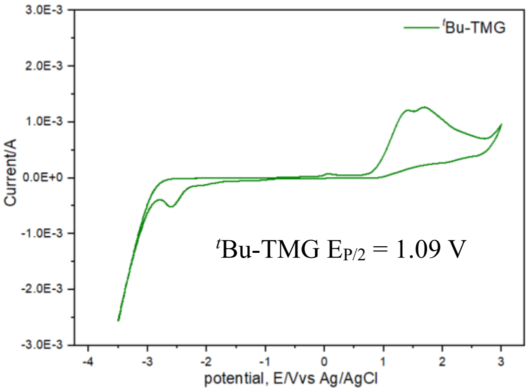

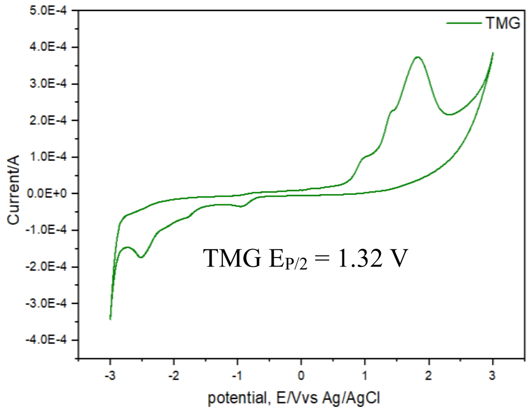
TMG
TMG EP/2 = 1.32 V
Current/A
potential, E/Vvs Ag/AgCl

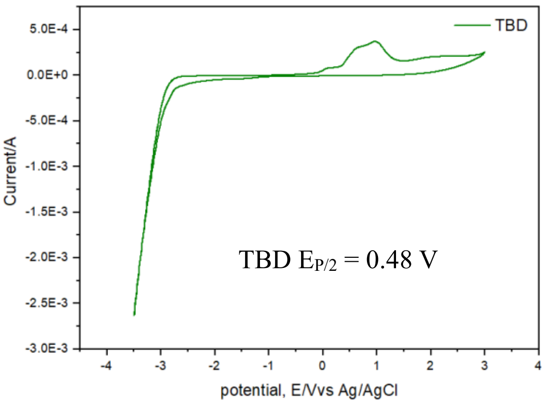
TBD
TBD EP/2 = 0.48 V
Current/A
potential, E/Vvs Ag/AgCl

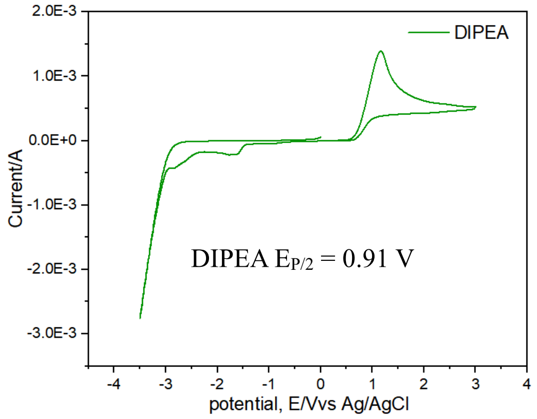
DIPEA
DIPEA EP/2 = 0.91 V
Current/A
potential, E/Vvs Ag/AgCl

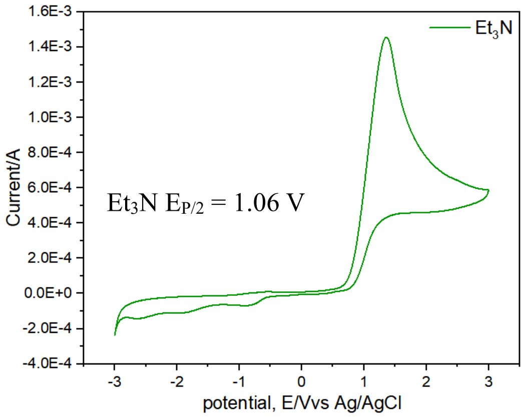
Et3N
Et3N EP/2 = 1.06 V
Current/A
potential, E/Vvs Ag/AgCl

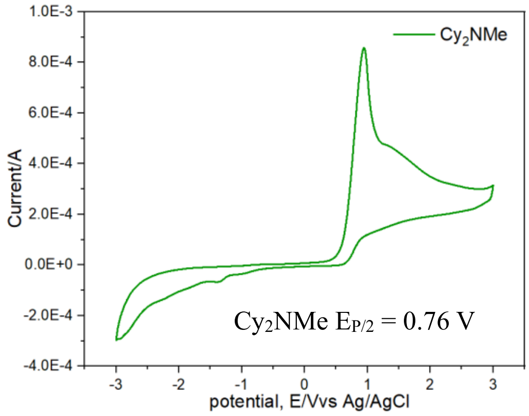
Cy2NMe
Cy2NMe EP/2 = 0.76 V
Current/A
potential, E/Vvs Ag/AgCl

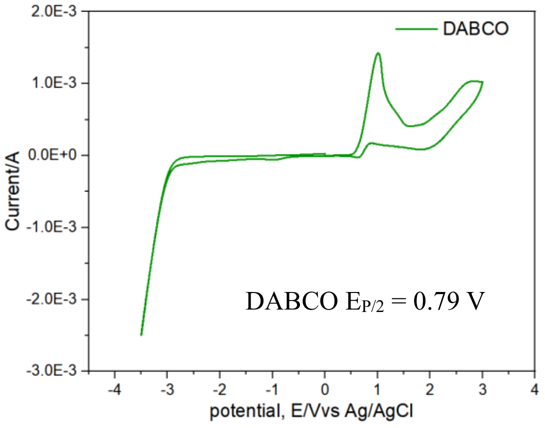
DABCO
DABCO EP/2 = 0.79 V
Current/A
potential, E/Vvs Ag/AgCl

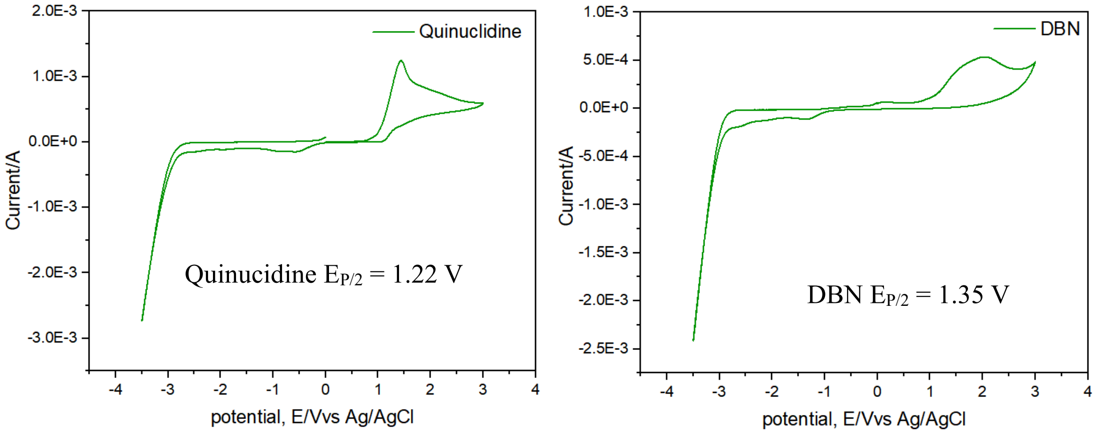

Figure S40. Redox potentials of different organic bases.

14. Analytical data of products

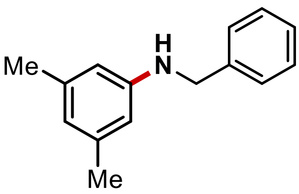

***N*-Benzyl-3,5-dimethylaniline (4):** Yellow oil; ^{1}H NMR (400 MHz, $CDCl_3$) δ 7.31 – 7.22 (m, 4H), 7.22 – 7.13 (m, 1H), 6.31 (s, 1H), 6.21 (s, 2H), 4.22 (s, 2H), 2.15 (s, 6H); ^{13}C NMR (100 MHz, $CDCl_3$) δ 148.4, 139.8, 139.1, 128.7, 127.7, 127.3, 119.8, 110.9, 48.5, 21.6; HRMS (ESI) m/z calc. for $C_{15}H_{18}N$ [M+H]$^+$: 212.1434, found: 212.1437.

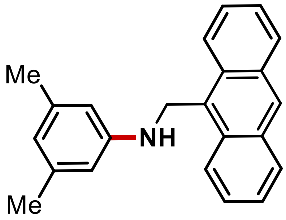

***N*-(Anthracen-9-ylmethyl)-3,5-dimethylaniline (5):** Yellow oil; ^{1}H NMR (400 MHz, $CDCl_3$) δ 8.44 (s, 1H), 8.26 – 8.24 (m, 2H), 8.07 – 7.97 (m, 2H), 7.53 – 7.41 (m, 4H), 6.47 (s, 1H), 6.43 (s, 2H), 5.10 (s, 2H), 3.71 (br, 1H), 2.30 (s, 6H); ^{13}C NMR (100 MHz, $CDCl_3$) δ 148.7, 139.2, 131.7, 130.6, 129.7, 129.2, 128.0, 126.5, 125.3, 124.4, 119.8, 110.7, 41.0, 21.7; HRMS (ESI) m/z calc. for $C_{23}H_{22}N$ [M+H]$^+$: 312.1747, found: 312.1752.

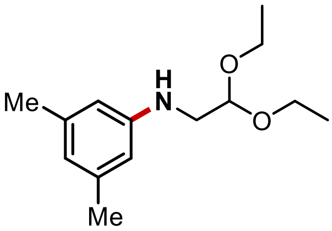

***N*-(2,2-Diethoxyethyl)-3,5-dimethylaniline (6):** Yellow oil; ^{1}H NMR (400 MHz, $CDCl_3$) δ 6.31 (s, 1H), 6.21 (s, 2H), 4.59 (t, *J* = 5.6 Hz, 1H), 3.68 – 3.61 (m, 2H), 3.59 – 3.42 (m, 2H), 3.16 (d, *J* = 5.6 Hz, 2H), 2.16 (s, 6H), 1.16 (t, *J* = 7.1 Hz, 6H); ^{13}C NMR (100 MHz, $CDCl_3$) δ 148.2, 139.0, 119.8, 111.3, 101.1, 62.4, 46.6, 21.6, 15.5; HRMS (ESI) m/z calc. for $C_{14}H_{24}NO_2$ [M+H]$^+$: 238.1802, found: 238.1805.

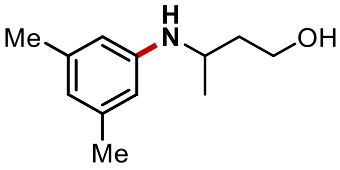

3-((3,5-Dimethylphenyl)amino)butan-1-ol (7): Yellow oil; ^{1}H NMR (400 MHz, $CDCl_3$) δ 6.41 (s, 1H), 6.32 (s, 2H), 3.93 – 3.81 (m, 2H), 3.71 – 3.66 (m, 1H), 2.24 (s, 6H), 1.85 – 1.73 (m, 2H), 1.20 (d, *J* = 6.3 Hz, 3H); ^{13}C NMR (100 MHz, $CDCl_3$) δ 147.2, 139.2, 120.5, 112.5, 61.5, 48.7, 39.4, 21.6, 21.4; HRMS (ESI) m/z calc. for $C_{12}H_{20}NO$ $[M+H]^+$: 194.1539, found: 194.1542.

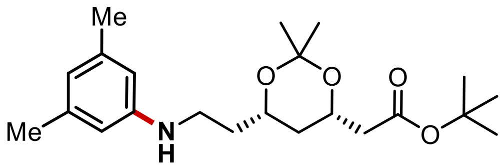

Tert-butyl 2-((4S,6S)-6-(2-((3,5-dimethylphenyl)amino)ethyl)-2,2-dimethyl-1,3-dioxan-4-yl)acetate (8): Yellow oil; ^{1}H NMR (400 MHz, $CDCl_3$) δ 6.37 (s, 1H), 6.26 (s, 2H), 4.33 – 4.20 (m, 1H), 4.02 (s, 1H), 3.31 – 3.12 (m, 2H), 2.46 – 2.40 (m, 1H), 2.33 – 2.32 (m, 1H), 2.23 (s, 6H), 1.78 – 1.73 (m, 6.3 Hz, 2H), 1.56 (d, *J* = 12.7 Hz, 2H), 1.44 (s, 9H), 1.40 (s, 3H), 1.27 (d, *J* = 10.8 Hz, 3H); ^{13}C NMR (100 MHz, $CDCl_3$) δ 170.4, 148.4, 139.0, 119.7, 111.2, 98.9, 80.8, 68.1, 66.4, 42.8, 41.2, 36.5, 35,7, 30.3, 28.3, 21.6, 19.9; HRMS (ESI) m/z calc. for $C_{22}H_{36}NO_4$ $[M+H]^+$: 378.2639, found: 378.2645.

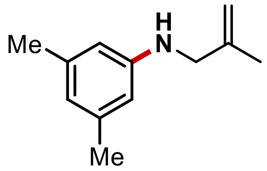

3,5-Dimethyl-N-(2-methylallyl)aniline (9): Yellow oil; ^{1}H NMR (400 MHz, $CDCl_3$) δ 6.37 (s, 1H), 6.26 (s, 2H), 4.96 (s, 1H), 4.87 (s, 1H), 3.66 (s, 2H), 2.23 (s, 6H), 1.79 (s, 3H); ^{13}C NMR (100 MHz, $CDCl_3$) δ 148.5, 143.1, 139.0, 119.6, 111.0, 110.9, 50.2, 21.6, 20.7; HRMS (ESI) m/z calc. for $C_{12}H_{18}N$ $[M+H]^+$: 176.1434, found: 176.1438.

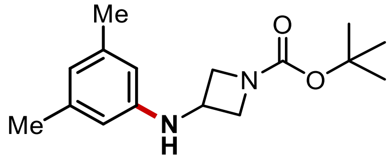

Tert-butyl 3-((3,5-dimethylphenyl)amino)azetidine-1-carboxylate (10): Yellow oil; ^{1}H NMR (400 MHz, $CDCl_3$) δ 6.44 (s, 1H), 6.15 (s, 2H), 4.32 – 4.23 (m, 2H), 4.21 – 4.17 (m, 1H), 3.73 – 7.69 (m, 2H), 2.24 (s, 6H), 1.44 (s, 9H); ^{13}C NMR (100 MHz, $CDCl_3$) δ 156.4, 146.3, 139.3, 120.8, 111.3, 79.8, 43.4, 28.5, 21.6; HRMS (ESI) m/z calc. for $C_{16}H_{25}N_2O_2$ $[M+H]^+$: 277.1911, found: 277.1915.

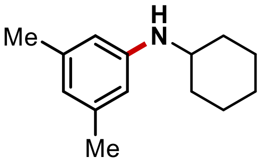

N-Cyclohexyl-3,5-dimethylaniline (11): Yellow oil; ^{1}H NMR (400 MHz, $CDCl_3$) δ 6.33 (s, 1H), 6.24 (s, 2H), 3.40 (br, 1H), 3.28 – 3.21 (m, 1H), 2.24 (s, 6H), 2.11 – 1.99 (m, 2H), 1.82 – 1.73 (m, 2H), 1.72 – 1.57 (m, 1H), 1.45 – 1.34 (m, 2H), 1.27 – 1.21 (m, 1H), 1.19 – 1.10 (m, 2H); ^{13}C NMR (100 MHz, $CDCl_3$) δ 147.6, 139.0, 119.0, 111.2, 51.7, 33.7, 26.1, 25.2, 21.6; HRMS (ESI) m/z calc. for $C_{14}H_{22}N$ $[M+H]^+$: 204.1747, found: 204.1750.

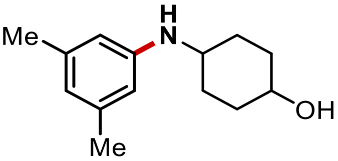

4-((3,5-Dimethylphenyl)amino)cyclohexan-1-ol (12): Yellow oil; ^{1}H NMR (400 MHz, $CDCl_3$) δ 6.36 (s, 1H), 6.23 (s, 2H), 3.75 – 3.54 (m, 1H), 3.28 – 3.20 (m, 1H), 2.24 (s, 6H), 2.15 – 2.12 (m, 2H), 2.02 – 2.00 (m, 2H), 1.54 – 1.34 (m, 2H), 1.33 – 1.11 (m, 2H); ^{13}C NMR (100 MHz, $CDCl_3$) δ 147.4, 139.1, 119.4, 111.3, 70.4, 51.3, 34.2, 31.3, 21.6; HRMS (ESI) m/z calc. for $C_{15}H_{24}NO_2$ $[M+H]^+$: 220.1696 found: 220.1693.

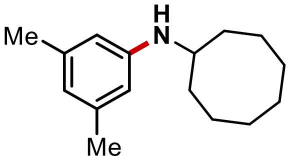

Ethyl 3-(4-(butylamino)phenyl)propanoate(13): Yellow oil; ^{1}H NMR (400 MHz, $CDCl_3$) δ 6.35 (s, 1H), 6.21 (s, 2H), 3.49 – 3.44 (m, 1H), 2.25 (s, 6H), 2.07 – 1.95 (m, 2H), 1.79 – 1.37 (m, 11H), 1.34 – 1.24 (m, 1H); ^{13}C NMR (100 MHz, $CDCl_3$) δ 147.6, 139.0, 119.0, 111.3, 53.7, 35.1, 28.6, 24.5, 21.7; HRMS (ESI) m/z calc. for $C_{16}H_{26}N$ $[M+H]^+$: 232.2060, found: 232.2063.

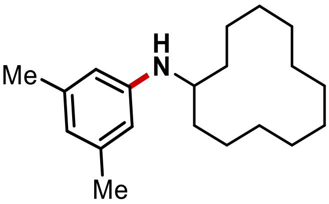

N-(3,5-Dimethylphenyl)cyclododecanamine (14): Yellow oil; ^{1}H NMR (400 MHz, $CDCl_3$) δ 6.34 (s, 1H), 6.25 (s, 2H), 3.49 – 3.46 (m, 1H), 2.23 (s, 6H), 1.43 – 1.36(m, 22H); ^{13}C NMR (100 MHz, $CDCl_3$) δ 147.8, 138.8, 118.7, 111.0, 49.4, 29.6, 24.9, 24.4, 24.2, 23.3, 22.6, 21.5, 21.2; HRMS (ESI) m/z calc. for $C_{20}H_{34}N$ $[M+H]^+$: 288.2686, found: 288.2690.

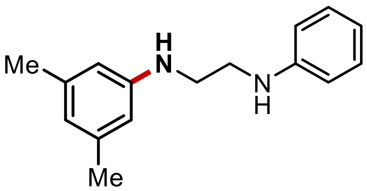

N1-(3,5-Dimethylphenyl)-N2-phenylethane-1,2-diamine (15): Yellow oil; ^{1}H NMR (400 MHz, $CDCl_3$) δ 7.22 – 7.19 (m, 2H), 6.75 (t, *J* = 7.3 Hz, 1H), 6.66 (d, *J* = 7.7 Hz, 2H), 6.43 (s, 1H), 6.32 (s, 2H), 3.39 (s, 4H), 2.26 (s, 6H); ^{13}C NMR (100 MHz, $CDCl_3$) δ 148.2, 148.1, 139.2, 129.5, 120.1, 118.0, 113.2, 111.3, 43.6, 43.5, 21.6; HRMS (ESI) m/z calc. for $C_{16}H_{21}N_2$ $[M+H]^+$: 241.1699, found: 241.1697.

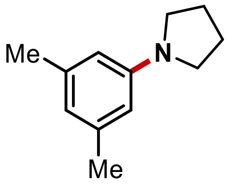

1-(3,5-Dimethylphenyl)pyrrolidine (3): Yellow oil; ^{1}H NMR (400 MHz, $CDCl_3$) δ 6.35 (s, 1H), 6.24 (s, 2H), 3.29 – 3.26 (m, 4H), 2.29 (s, 6H), 1.99 – 1.97 (m, 4H); ^{13}C NMR (100 MHz, $CDCl_3$) δ 148.3, 138.8, 117.7, 109.8, 47.8, 25.6, 21.8; HRMS (ESI) m/z calc. for $C_{12}H_{18}N$ $[M+H]^+$: 176.1434, found: 176.1437.

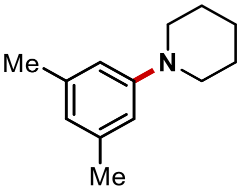

1-(3,5-Dimethylphenyl)piperidine (16): Yellow oil; ^{1}H NMR (400 MHz, $CDCl_3$) δ 6.58 (s, 2H), 6.50 (s, 1H), 3.19 – 3.06 (m, 4H), 2.28 (s, 6H), 1.77 – 1.68 (m, 4H), 1.58 – 1.57(m, 2H); ^{13}C NMR (100 MHz, $CDCl_3$) δ 152.6, 138.6, 121.4, 114.7, 51.1, 26.1, 24.6, 21.8; HRMS (ESI) m/z calc. for $C_{13}H_{20}N$ $[M+H]^+$: 190.1590, found: 190.1594.

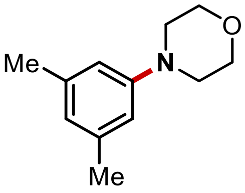

4-(3,5-Dimethylphenyl)morpholine (17): Yellow oil; ^{1}H NMR (400 MHz, $CDCl_3$) δ 6.55 (s, 3H), 3.94 – 3.75 (m, 4H), 3.18 – 3.07 (m, 4H), 2.29 (s, 6H); ^{13}C NMR (100 MHz, $CDCl_3$) δ 151.6, 138.9, 122.2, 113.9, 67.2, 49.7, 21.8; HRMS (ESI) m/z calc. for $C_{12}H_{18}NO$ $[M+H]^+$: 192.1383, found: 192.1885.

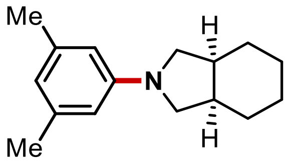

(3aR,7aS)-2-(3,5-Dimethylphenyl)octahydro-1H-isoindole (18): Yellow oil; ^{1}H NMR (400 MHz, $CDCl_3$) δ 6.31 (s, 1H), 6.17 (s, 2H), 3.29 – 3.27 (m, 2H), 3.23 – 3.09 (m, 2H), 2.30 (s, 2H), 2.27 (s, 6H), 1.62 – 1.60 (m, 2H), 1.55 – 1.45 (m, 4H), 1.42 – 1.38 (m, 2H); ^{13}C NMR (100 MHz, $CDCl_3$) δ 160.0, 138.7, 117.0, 109.0, 51.8, 37.3, 26.4, 23.1, 21.7; HRMS (ESI) m/z calc. for $C_{16}H_{24}N$ $[M+H]^+$: 230.1903, found: 230.1906.

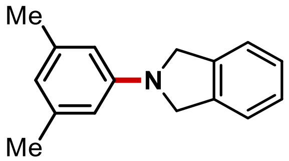

2-(3,5-Dimethylphenyl)isoindoline (19): Yellow oil; ^{1}H NMR (400 MHz, $CDCl_3$) δ 7.38 – 7.27 (m, 4H), 6.44 (s, 1H), 6.35 (s, 2H), 4.65 (s, 4H), 2.33 (s, 6H); ^{13}C NMR (100 MHz, $CDCl_3$) δ 147.4, 139.2, 138.2, 127.3, 122.7, 118.7, 109.9, 54.2, 21.9; HRMS (ESI) m/z calc. for $C_{16}H_{18}N$ $[M+H]^+$: 224.1434, found: 224.1436.

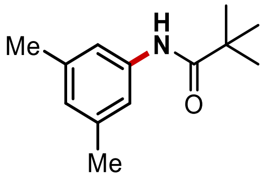

N-(3,5-Dimethylphenyl)pivalamide (20): White solid; ^{1}H NMR (400 MHz, $CDCl_3$) δ 7.17 (s, 2H), 6.75 (s, 1H), 2.29 (s, 6H), 1.30 (s, 9H); ^{13}C NMR (100 MHz, $CDCl_3$) δ 176.7, 138.8, 138.1, 126.0, 117.8, 39.7, 27.8, 21.5; HRMS (ESI) m/z calc. for $C_{13}H_{20}NO$ $[M+H]^+$: 206.1539, found: 206.1542.

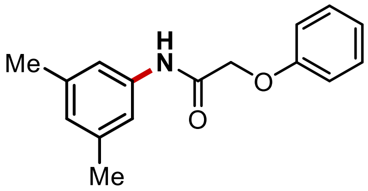

N-(3,5-Dimethylphenyl)-2-phenoxyacetamide (21): White solid; ^{1}H NMR (400 MHz, $CDCl_3$) δ 8.17 (br, 1H), 7.37 – 7.33 (m, 2H), 7.22 (s, 2H), 7.08 – 7.04 (m, 1H), 6.99 (d, J = 7.9 Hz, 2H), 6.80 (s, 1H), 4.60 (s, 2H), 2.32 (s, 6H); ^{13}C NMR (100 MHz, $CDCl_3$) δ 166.3, 157.2, 139.0, 136.8, 130.1, 126.8, 122.6, 118.0, 115.0, 67.8, 21.5; HRMS (ESI) m/z calc. for $C_{16}H_{18}NO_2$ $[M+H]^+$: 256.1332, found: 256.1335.

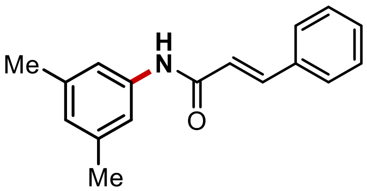

N-(3,5-Dimethylphenyl)cinnamamide (22): White solid; ^{1}H NMR (400 MHz, $CDCl_3$) δ 7.67 (d, J = 15.5 Hz, 1H), 7.49 – 7.42 (m, 2H), 7.31 – 7.30 (m, 3H), 7.26 (s, 1H), 7.19 (s, 2H), 6.71 (s, 1H), 6.47 (d, J = 15.5 Hz, 1H), 2.24 (s, 6H); ^{13}C NMR (100 MHz, $CDCl_3$) δ 166.5, 142.4, 138.9, 138.0, 134.9, 130.1, 129.0, 128.1, 126.4, 117.9, 21.5; HRMS (ESI) m/z calc. for $C_{17}H_{18}NO$ [M+H]$^+$: 252.1383, found: 252.1386.

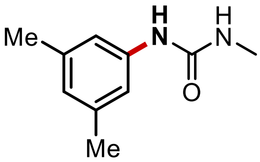

1-(3,5-Dimethylphenyl)-3-methylurea (23): White solid; ^{1}H NMR (400 MHz, $CDCl_3$) δ 6.39 (s, 1H), 6.27 (s, 2H), 2.82 (s, 3H), 2.25 (s, 6H); ^{13}C NMR (100 MHz, $CDCl_3$) δ 163.6, 149.4, 139.0, 119.7, 110.7, 31.1, 21.6; HRMS (ESI) m/z calc. for $C_{10}H_{15}N_2O$ [M+H]$^+$: 179.1179, found: 179.1181.

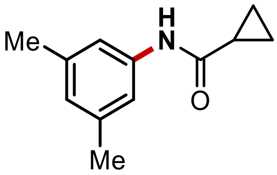

N-(3,5-Dimethylphenyl)cyclopropanecarboxamide (24): White solid; ^{1}H NMR (400 MHz, $CDCl_3$) δ 7.30 (br, 1H) 7.15 (s, 2H), 6.74 (s, 1H), 2.28 (s, 6H), 1.49 – 1.41 (m, 1H), 1.13 – 0.99 (m, 2H), 0.84 – 0.81 (m, 2H); ^{13}C NMR (100 MHz, $CDCl_3$) δ 158.8, 141.0, 138.7, 125.8, 117.4, 21.4, 15.8, 7.9; HRMS (ESI) m/z calc. for $C_{12}H_{16}NO$ [M+H]$^+$: 190.1226, found: 190.1229.

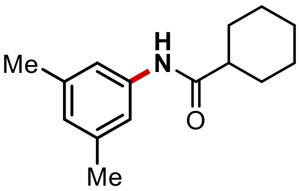

N-(3,5-Dimethylphenyl)cyclohexanecarboxamide (25): White solid; ^{1}H NMR (400 MHz, $CDCl_3$) δ 7.16 (s, 2H), 7.06 (br, 1H), 6.74 (s, 1H), 2.28 (s, 6H), 2.23 – 2.17 (m, 1H), 1.96 – 1.93 (m, 2H), 1.85 – 1.82 (m, 2H), 1.71 – 1.69 (m, 1H), 1.54 – 1.48 (m, 2H), 1.35 – 1.26 (m, 3H); ^{13}C NMR (100 MHz, $CDCl_3$) δ 174.4, 138.8, 138.1, 126.0, 117.6, 60.5, 46.7, 29.8, 25.8, 21.5; HRMS (ESI) m/z calc. for $C_{15}H_{22}NO$ [M+H]$^+$: 232.1696, found: 232.1692.

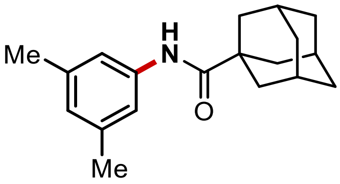

(3r,5r,7r)-N-(3,5-Dimethylphenyl)adamantane-1-carboxamide (26): White solid; ^{1}H NMR (400 MHz, $CDCl_3$) δ 7.18 (s, 3H), 6.74 (br, 1H), 2.29 (s, 6H), 2.10 (s, 3H), 1.96 – 1.95 (m, 6H), 1.81 – 1.71 (m, 6H); ^{13}C NMR (100 MHz, $CDCl_3$) δ 176.2, 138.8, 138.0, 126.0, 117.8, 41.6, 39.5, 36.6, 28.3, 21.5; HRMS (ESI) m/z calc. for $C_{19}H_{26}NO$ $[M+H]^+$: 284.2009, found: 284.2012.

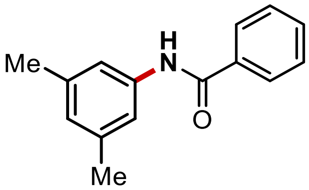

N-(3,5-Dimethylphenyl)benzamide (27): White solid; ^{1}H NMR (400 MHz, $CDCl_3$) δ 7.79 (d, J = 7.2 Hz, 2H), 7.65 (br, 1H), 7.50 – 7.46 (m, 1H), 7.43 – 7.40 (m, 2H), 7.21 (s, 2H), 6.73 (br, 1H), 2.26 (s, 6H); ^{13}C NMR (100 MHz, $CDCl_3$) δ 165.8, 139.0, 137.9, 135.3, 131.9, 128.9, 127.1, 126.5, 118.1, 21.5; HRMS (ESI) m/z calc. for $C_{15}H_{16}NO$ $[M+H]^+$: 226.1226, found: 226.1230.

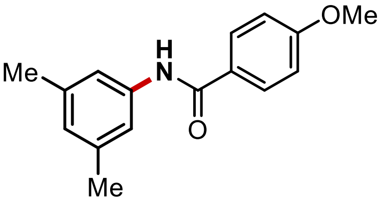

N-(3,5-Dimethylphenyl)-4-methoxybenzamide (28): White solid; ^{1}H NMR (400 MHz, $CDCl_3$) δ 7.77 – 7.74 (m, 2H), 7.57 (br, 1H), 7.20 – 7.19 (m, 3H), 6.95 – 6.85 (m, 2H), 6.72 (s, 1H), 3.80 (s, 3H), 2.25 (s, 6H); ^{13}C NMR (100 MHz, $CDCl_3$) δ 165.2, 162.6, 138.9, 138.1 129.0, 127.5, 126.3, 118.0, 114.1, 55.6, 21.5; HRMS (ESI) m/z calc. for $C_{16}H_{18}NO_2$ $[M+H]^+$: 256.1332, found: 256.1335.

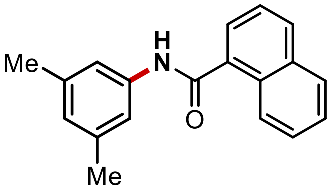

N-(3,5-Dimethylphenyl)-1-naphthamide (29): White solid; ^{1}H NMR (400 MHz, $CDCl_3$) δ 8.30 (br, 1H), 7.92 – 7.77 (m, 5H), 7.54 – 7.48 (m, 2H), 7.27 (s, 2H), 6.75 (s, 1H), 2.28 (s, 6H); ^{13}C NMR (100 MHz, $CDCl_3$) δ 165.8, 139.0, 138.0, 135.0, 132.8, 132.5, 129.1, 128.9, 128.0, 128.0,

127.5, 127.1, 126.5, 123.7, 118.1, 21.6; HRMS (ESI) m/z calc. for $C_{19}H_{18}NO$ [M+H]$^+$: 276.1383, found: 276.1386.

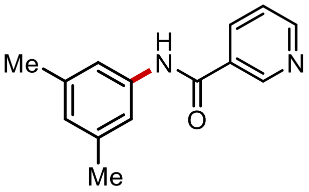

***N*-(3,5-Dimethylphenyl)nicotinamide (30):** White solid; ^{1}H NMR (400 MHz, $CDCl_3$) δ 9.11 (s, 1H), 8.77 (br, 1H), 8.22 (d, *J* = 7.8 Hz, 1H), 7.89 (s, 1H), 7.52 – 7.40 (m, 1H), 7.28 (s, 2H), 6.83 (s, 1H), 2.33 (s, 6H); ^{13}C NMR (100 MHz, $CDCl_3$) δ 153.2, 148.0, 145.5, 139.1, 137.4, 135.7, 131.2, 127.0, 123.9, 118.3, 21.5; HRMS (ESI) m/z calc. for $C_{14}H_{15}N_2O$ [M+H]$^+$: 227.1179, found: 227.1183.

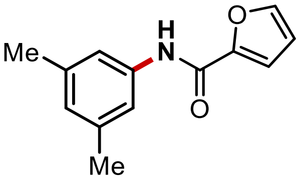

***N*-(3,5-Dimethylphenyl)furan-2-carboxamide (31):** White solid; ^{1}H NMR (400 MHz, $CDCl_3$) δ 7.97 (br, 1H), 7.53 – 7.46 (m, 1H), 7.29 (s, 2H), 7.22 (d, *J* = 3.5 Hz, 1H), 6.79 (s, 1H), 6.56 – 6.55 (dd, *J* = 3.4, 1.7 Hz, 1H), 2.32 (s, 6H); ^{13}C NMR (100 MHz, $CDCl_3$) δ 156.1, 148.2, 144.2, 139.0, 137.4, 126.4, 117.8, 115.2, 112.7, 21.5; HRMS (ESI) m/z calc. for $C_{13}H_{14}NO_2$ [M+H]$^+$: 216.1019, found: 216.1021.

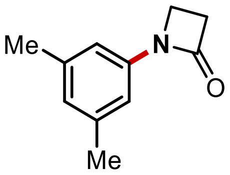

1-(3,5-Dimethylphenyl)azetidin-2-one (32): White solid; ^{1}H NMR (400 MHz, $CDCl_3$) δ 6.99 (s, 2H), 6.73 (s, 1H), 3.59 (t, *J* = 4.4 Hz, 2H), 3.08 (t, *J* = 4.4 Hz, 2H), 2.30 (s, 6H); ^{13}C NMR (100 MHz, $CDCl_3$) δ 164.6, 149.0, 139.1, 125.8, 114.0, 38.2, 36.0, 21.5; HRMS (ESI) m/z calc. for $C_{11}H_{14}NO$ [M+H]$^+$: 176.1070, found: 176.1072.

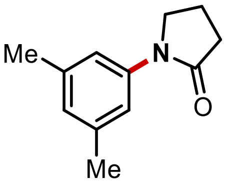

1-(3,5-Dimethylphenyl)pyrrolidin-2-one (33): White solid; ^{1}H NMR (400 MHz, $CDCl_3$) δ 7.21 (s, 2H), 6.80 (s, 1H), 3.83 (t, *J* = 7.0 Hz, 2H), 2.59 (t, *J* = 8.1 Hz, 2H), 2.32 (s, 6H), 2.17 – 2.10 (m, 2H); ^{13}C NMR (100 MHz, $CDCl_3$) δ 174.2, 139.2, 138.4, 126.4, 118.1, 49.1, 32.8, 21.5,

18.1; HRMS (ESI) m/z calc. for $C_{11}H_{14}NO$ [M+H]+: 190.1226, found: 190.1230.

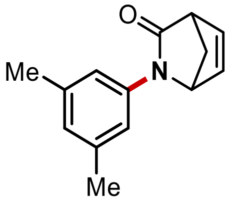

2-(3,5-Dimethylphenyl)-2-azabicyclo[2.2.1]hept-5-en-3-one (34): White solid; ^{1}H NMR (400 MHz, $CDCl_3$) δ 7.03-7.01 (m, 1H), 6.99 (s, 2H), 6.75 (s, 1H), 6.73 – 6.70 (m, 1H), 4.75 – 4.74 (m, 1H), 3.48 (s, 1H), 2.51 – 2.43 (m, 1H), 2.30 (s, 6H), 2.28 – 2.23 (m, 1H); ^{13}C NMR (100 MHz, $CDCl_3$) δ 177.5, 139.6, 139.3, 138.8, 138.6, 126.0, 116.8, 65.1, 57.6, 55.0, 21.6; HRMS (ESI) m/z calc. for $C_{14}H_{16}NO$ $[M+H]^+$: 214.1226, found: 214.1230.

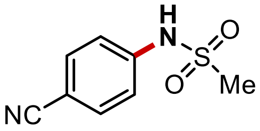

N-(4-Cyanophenyl)methanesulfonamide (35): White solid; ^{1}H NMR (400 MHz, d_6-DMSO) δ 7.70 (d, J = 8.7 Hz, 2H), 7.38 (d, J = 8.7 Hz, 2H), 3.09 (s, 3H); ^{13}C NMR (100 MHz, d_6-DMSO) δ 146.9, 137.4, 122.2, 115.7, 110.0, 42.7; HRMS (ESI) m/z calc. for $C_8H_9N_2O_2S$ $[M+H]^+$: 197.0379, found: 197.0383.

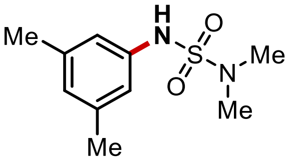

Ethyl 3-(4-(butylamino)phenyl)propanoate(36): White solid; ^{1}H NMR (400 MHz, $CDCl_3$) δ 6.41 (s, 3H), 2.93 (s, 6H), 2.29 (s, 6H); ^{13}C NMR (100 MHz, $CDCl_3$) δ 143.4, 138.7, 122.8, 115.9, 41.0, 21.9; HRMS (ESI) m/z calc. for $C_{10}H_{17}N_2O_2S$ $[M+H]^+$: 229.1005, found: 229.1010.

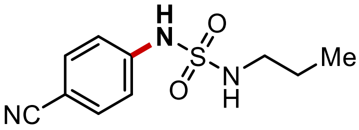

N-(4-Cyanophenyl)-**N-propyl*****sulfonamide (37):*** White solid; ^{1}H NMR (400 MHz, $CDCl_3$) δ 7.41 (d, J = 8.6 Hz, 2H), 6.54 (d, J = 8.6 Hz, 2H), 4.20 (br, 1H), 3.12 (t, J = 7.1 Hz, 2H), 1.70 – 1.61 (m, 2H), 1.02 – 0.98 (m, 3H); ^{13}C NMR (100 MHz, $CDCl_3$) δ 151.6, 133.8, 120.7, 112.2, 98.6, 45.2, 22.5, 11.6; HRMS (ESI) m/z calc. for $C_{10}H_{14}N_3O_2S$ $[M+H]^+$: 240.0801, found: 240.0805.

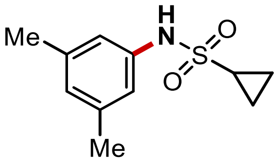

N-(3,5-Dimethylphenyl)cyclopropanesulfonamide (38): White solid; ^{1}H NMR (400 MHz, $CDCl_3$) δ 6.89 (s, 2H), 6.81 (s, 1H), 6.77 (br, 1H), 2.58 – 2.45 (m, 1H), 2.30 (s, 6H), 1.22 – 1.13 (m, 2H), 0.99 – 0.90 (m, 2H); ^{13}C NMR (100 MHz, $CDCl_3$) δ 139.2, 136.6, 127.0, 119.2, 29.7, 21.3, 5.6; HRMS (ESI) m/z calc. for $C_{11}H_{16}NO_2S$ $[M+H]^+$: 226.0896, found: 226.0893.

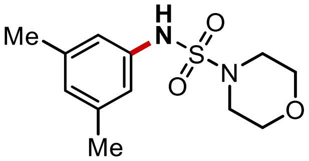

N-(3,5-Dimethylphenyl)morpholine-4-sulfonamide (39): White solid; ^{1}H NMR (400 MHz, $CDCl_3$) δ 6.56 (s, 3H), 3.93 – 3.74 (m, 4H), 3.22 – 3.05 (m, 4H), 2.29 (s, 6H); ^{13}C NMR (100 MHz, $CDCl_3$) δ 155.6, 138.8, 122.1, 113.8, 67.0, 49.7, 21.6; HRMS (ESI) m/z calc. for $C_{12}H_{19}N_2O_3S$ $[M+H]^+$: 271.1111, found: 271.1114.

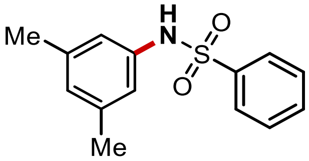

N-(3,5-Dimethylphenyl)-4-methylbenzenesulfonamide (40): White solid; ^{1}H NMR (400 MHz, $CDCl_3$) δ 7.82 (d, J = 7.9 Hz, 2H), 7.52 (t, J = 7.3 Hz, 1H), 7.43 (t, J = 7.5 Hz, 2H), 7.08 (s, 1H), 6.71 (s, 2H), 2.20 (s, 6H); ^{13}C NMR (100 MHz, $CDCl_3$) δ 139.2, 139.0. 136.2, 133.0, 128.9, 127.2, 127.1, 119.1, 21.2; HRMS (ESI) m/z calc. for $C_{14}H_{16}NO_2S$ $[M+H]^+$: 262.0896, found: 262.0990.

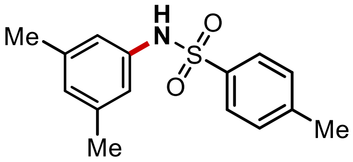

N-(3,5-Dimethylphenyl)-4-methylbenzenesulfonamide (41): White solid; ^{1}H NMR (400 MHz, $CDCl_3$) δ 7.66 (d, J = 8.3 Hz, 2H), 7.23 (d, J = 8.1 Hz, 2H), 6.73 (s, 1H), 6.68 (s, 2H), 6.52 (br, 1H), 2.38 (s, 3H), 2.21 (s, 6H); ^{13}C NMR (100 MHz, $CDCl_3$) δ 143.7, 139.1, 136.3, 129.6, 127.3, 127.0, 119.1, 105.4, 21.5, 21.2; HRMS (ESI) m/z calc. for $C_{15}H_{18}NO_2S$ $[M+H]^+$: 276.1053, found: 276.1057.

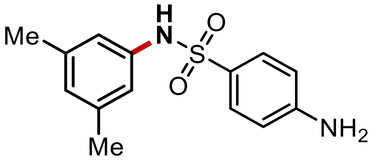

4-Amino-N-(3,5-dimethylphenyl)benzenesulfonamide (42): White solid; ^{1}H NMR (400 MHz, $CDCl_3$) δ 7.57 (d, *J* = 8.3 Hz, 2H), 6.78 (br, 1H), 6.75 (s, 1H), 6.68 (s, 2H), 6.56 (d, *J* = 8.1 Hz, 2H), 4.03 (br, 2H), 2.20 (s, 6H); ^{13}C NMR (100 MHz, $CDCl_3$) δ 148.5, 139.1, 136.9, 129.5, 128.6, 126.8, 118.9, 114.9, 21.4; HRMS (ESI) m/z calc. for $C_{14}H_{17}N_2O_2S$ $[M+H]^+$: 277.1005, found: 277.1008.

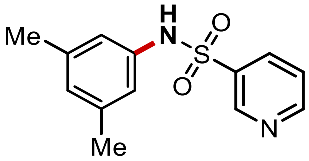

N-(3,5-Dimethylphenyl)pyridine-3-sulfonamide (43): White solid; ^{1}H NMR (400 MHz, $CDCl_3$) δ 8.99 (br, 1H), 8.75 (s, 1H), 8.04 (d, *J* = 8.0 Hz, 1H), 7.37 (s, 2H), 6.77 (s, 1H), 6.72 (s, 2H), 2.21 (s, 6H); ^{13}C NMR (100 MHz, $CDCl_3$) δ 153.2, 148.0, 139.4, 136.0, 135.5, 135.0, 127.8, 123.7, 119.6, 21.2; HRMS (ESI) m/z calc. for $C_{13}H_{15}N_2O_2S$ $[M+H]^+$: 263.0849, found: 263.0853.

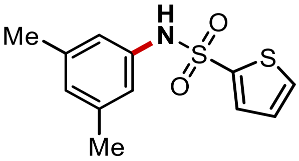

N-(3,5-Dimethylphenyl)thiophene-2-sulfonamide (44): White solid; ^{1}H NMR (400 MHz, $CDCl_3$) δ 7.53 – 7.52 (m, 1H), 7.50 – 7.48 (m, 1H), 7.02 – 7.00 (m, 1H), 6.79 (s, 1H), 6.73 (s, 2H), 6.46 (br, 1H), 2.25 (s, 6H); ^{13}C NMR (100 MHz, $CDCl_3$) δ 148.8, 139.2, 135.9, 132.8, 132.3, 127.6, 127.2, 119.5, 21.2; HRMS (ESI) m/z calc. for $C_{12}H_{14}N_2O_2S_2$ $[M+H]^+$: 268.0460, found: 268.0464.

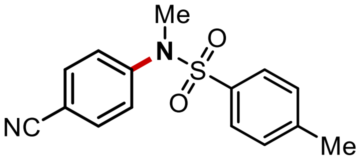

N-(4-Cyanophenyl)-N,4-dimethylbenzenesulfonamide (45): White solid; Yellow oil; ^{1}H NMR (400 MHz, $CDCl_3$) δ 7.60 – 7.59 (m, 2H), 7.42 – 7.40 (m, 2H), 7.28 – 7.26 (m, 4H), 3.18 (s, 3H), 2.42 (s, 3H); ^{13}C NMR (100 MHz, $CDCl_3$) δ 144.5, 133.2, 132.9, 129.8, 127.8, 126.2, 118.4, 116.5, 110.4, 37.6, 21.7; HRMS (ESI) m/z calc. for $C_{15}H_{15}N_2O_2S$ $[M+H]^+$: 287.0849, found: 287.0852.

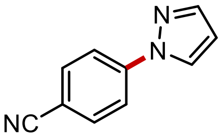

4-(1H-Pyrazol-1-yl)benzonitrile (46): Yellow oil; ^{1}H NMR (400 MHz, $CDCl_3$) δ 7.99 (s, 1H), 7.84 (d, *J* = 8.0 Hz, 2H), 7.80 – 7.67 (m, 3H), 6.53 (s, 1H); ^{13}C NMR (100 MHz, $CDCl_3$) δ

143.1, 142.6, 133.8, 126.9, 119.1, 118.5, 109.7, 109.2; HRMS (ESI) m/z calc. for $C_{10}H_8N_3$ $[M+H]^+$: 170.0713, found: 170.0716.

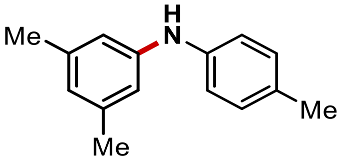

3,5-Dimethyl-N-(p-tolyl) aniline (47): **Yellow oil;** ^{1}H NMR (400 MHz, $CDCl_3$) δ 7.09 (d, *J* = 8.2 Hz, 2H), 7.00 (d, *J* = 8.3 Hz, 2H), 6.66 (s, 2H), 6.55 (s, 1H), 5.60 (br, 1H), 2.31 (s, 3H), 2.26 (s, 6H); ^{13}C NMR (100 MHz, $CDCl_3$) δ 144.0, 140.6, 139.2, 130.9, 130.0, 122.4, 119.2, 114.9, 21.6, 20.8; HRMS (ESI) m/z calc. for $C_{15}H_{18}N$ $[M+H]^+$: 212.1434, found: 212.1438.

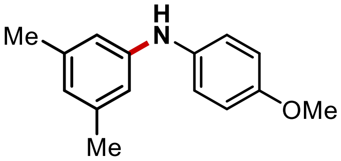

N-(4-Methoxyphenyl)-3,5-dimethylaniline (48): Yellow oil; ^{1}H NMR (400 MHz, $CDCl_3$) δ 7.06 (s, 2H), 6.86 (d, *J* = 8.8 Hz, 2H), 6.56 (s, 2H), 6.51 (s, 1H), 5.55 (br, 1H), 3.81 (s, 3H), 2.25 (s, 6H); ^{13}C NMR (100 MHz, $CDCl_3$) δ 155.4, 139.2, 136.0, 122.4, 121.8, 114.8, 113.7, 100.1, 55.7, 21.6; HRMS (ESI) m/z calc. for $C_{15}H_{18}NO$ $[M+H]^+$: 228.1383, found: 228.1386.

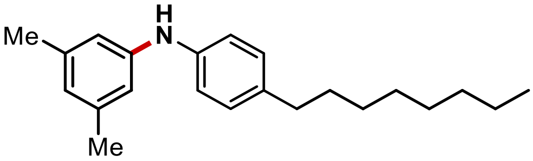

4-((3,5-Dimethylphenyl)amino)benzonitrile (49): Yellow oil; ^{1}H NMR (400 MHz, $CDCl_3$) δ 7.02 (d, *J* = 7.5 Hz, 2H), 6.95 (d, *J* = 7.7 Hz, 2H), 6.61 (s, 2H), 6.49 (s, 1H), 5.53(br, 1H), 2.48 (t, *J* = 7.5 Hz, 2H), 2.19 (s, 6H), 1.52 (s, 3H), 1.25 – 1.19 (m, 9H), 0.80 (d, *J* = 6.6 Hz, 3H); ^{13}C NMR (100 MHz, CDCl3) δ 143.8, 140.7, 139.0, 136.0, 129.1, 122.2, 118.7, 114.8, 35.3, 31.9, 31.7, 29.5, 29.4, 29.3, 22.7, 21.4, 14.1; HRMS (ESI) m/z calc. for $C_{22}H_{32}N$ $[M+H]^+$: 310.2529, found: 310.2533.

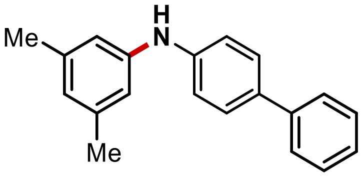

N-(3,5-Dimethylphenyl)-[1,1'-biphenyl]-4-amine (50): Yellow oil; ^{1}H NMR (400 MHz, $CDCl_3$) δ 7.57 (d, *J* = 7.2 Hz, 2H), 7.51 (d, *J* = 8.6 Hz, 2H), 7.42 (t, *J* = 7.7 Hz, 2H), 7.30 (d, *J* = 7.4 Hz, 1H), 7.13 (d, *J* = 8.6 Hz, 2H), 6.76 (s, 2H), 6.62 (s, 1H), 2.29 (s, 6H); ^{13}C NMR (100 MHz, $CDCl_3$) δ 142.8, 140.9, 139.1, 133.5, 128.7, 127.9, 126.5, 123.2, 117.8, 115.9, 21.4; HRMS (ESI) m/z calc. for $C_{20}H_{20}N$ $[M+H]^+$: 274.1590, found: 274.1594.

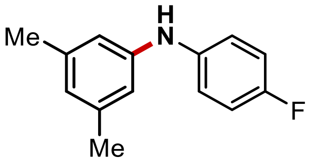

***N*-(4-Fluorophenyl)-3,5-dimethylaniline (51):** Yellow oil; ^{1}H NMR (400 MHz, $CDCl_3$) δ 7.08 – 7.01 (m, 2H), 7.01 – 6.94 (m, 2H), 6.62 (s, 2H), 6.57 (s, 1H), 2.26 (s, 6H); ^{13}C NMR (100 MHz, $CDCl_3$) δ 159.4, 157.0 (d, *J* = 250 Hz), 144.0, 139.3, 122.8, 120.8 (d, *J* = 7.8 Hz), 116.13 (d, *J* = 116 Hz), 114.9, 21.5; ^{19}F NMR (376 MHz, $CDCl_3$) δ -126.59 (s, F); HRMS (ESI) m/z calc. for $C_{14}H_{15}FN$ $[M+H]^+$: 216.1183, found: 216.1187.

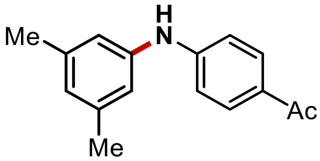

1-(4-((3,5-Dimethylphenyl)amino)phenyl)ethan-1-one (52): Yellow oil; ^{1}H NMR (400 MHz, $CDCl_3$) δ 7.86 (d, *J* = 8.8 Hz, 2H), 6.97 (d, *J* = 8.8 Hz, 2H), 6.81 (s, 2H), 6.74 (s, 1H), 2.53 (s, 3H), 2.31 (s, 6H); ^{13}C NMR (100 MHz, $CDCl_3$) δ 196.4, 148.6, 140.5, 139.3, 130.6, 128.8, 125.2, 118.5, 114.5, 26.1, 21.4; HRMS (ESI) m/z calc. for $C_{16}H_{18}NO$ $[M+H]^+$: 240.1383, found: 240.1385.

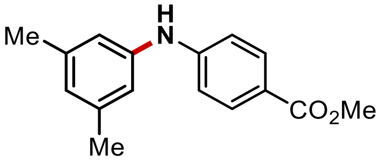

N-(4-Methoxyphenyl)-3,5-dimethylaniline (53): Yellow oil; ^{1}H NMR (400 MHz, $CDCl_3$) δ 7.91 (d, *J* = 8.8 Hz, 2H), 6.96 (d, *J* = 8.8 Hz, 2H), 6.80 (s, 2H), 6.72 (s, 1H), 3.87 (s, 3H), 2.30 (s, 6H); ^{13}C NMR (100 MHz, $CDCl_3$) δ 167.0, 148.3, 140.7, 139.3, 131.5, 125.0, 120.9, 118.3, 114.6, 51.7, 21.4; HRMS (ESI) m/z calc. for $C_{16}H_{18}NO_2$ $[M+H]^+$: 256.1332, found: 256.1336.

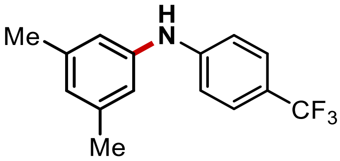

3,5-Dimethyl-N-(4-(trifluoromethyl)phenyl)aniline (54): Yellow oil; ^{1}H NMR (400 MHz, $CDCl_3$) δ 7.47 (d, *J* = 8.6 Hz, 2H), 7.03 (d, *J* = 8.5 Hz, 2H), 6.78 (s, 2H), 6.72 (s, 1H), 2.31 (s, 6H); ^{13}C NMR (100 MHz, $CDCl_3$) δ 147.1, 141.2, 139.4, 126.79 (q, *J* = 3.8 Hz), 124.9, 124.8 (q, *J* = 270.7 Hz), 121.5 (q, *J* = 32.5 Hz), 118.0, 115.5, 21.5; ^{19}F NMR (376 MHz, $CDCl_3$) δ -61.20 (s, CF_3); HRMS (ESI) m/z calc. for $C_{15}H_{15}NF_3$ $[M+H]^+$: 266.1151, found: 266.1154.

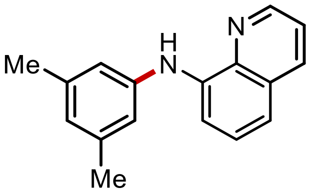

N-(3,5-Dimethylphenyl)quinolin-8-amine (55): Yellow oil; ^{1}H NMR (400 MHz, $CDCl_3$) δ 8.78 (d, J = 4.1 Hz, 1H), 8.21 (s, 1H), 8.12 (d, J = 8.3 Hz, 1H), 7.49 (d, J = 7.7 Hz, 1H), 7.45 – 7.37 (m, 2H), 7.21 – 7.19 (m, 1H), 7.04 (s, 2H), 6.70 (br, 1H), 2.34 (s, 6H); ^{13}C NMR (100 MHz, $CDCl_3$) δ 147.1, 141.7, 140.4, 139.0, 136.3, 129.9, 128.9, 127.4, 124.0, 121.5, 117.9, 116.2, 108.0, 21.5; HRMS (ESI) m/z calc. for $C_{17}H_{17}N_2$ $[M+H]^+$: 249.1386, found: 249.1390.

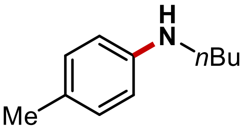

N-Butyl-4-methylaniline (56): Yellow oil; ^{1}H NMR (400 MHz, $CDCl_3$) δ 6.98 (d, J = 8.4 Hz, 2H), 6.53 (d, J = 8.4 Hz, 2H), 3.12 – 3.04 (t, J = 7.1, 2H), 2.23 (s, 3H), 1.62 – 1.54 (m, 2H), 1.46 – 1.40 (m, 2H), 0.95 (t, J = 7.3 Hz, 3H); ^{13}C NMR (100MHz, $CDCl_3$) δ 146.3, 129.7, 126.3, 112.9, 44.1, 31.8, 20.4, 20.3, 14.0; HRMS (ESI) m/z calc. for $C_{11}H_{18}N$ $[M+H]^+$: 164,1434, found: 164.1438.

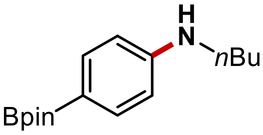

N-Butyl-4-(4,4,5,5-tetramethyl-1,3,2-dioxaborolan-2-yl)aniline (57): Yellow oil; ^{1}H NMR (400 MHz, $CDCl_3$) δ 7.62 (d, J = 8.3 Hz, 2H), 6.56 (d, J = 8.4 Hz, 2H), 3.84 (br, 1H), 3.14 (t, J = 7.1 Hz, 2H), 1.63 – 1.56 (m, 2H), 1.46 – 1.37 (m, 2H), 1.32 (s, 12H), 0.95 (t, J = 7.3 Hz, 3H); ^{13}C NMR (100 MHz, $CDCl_3$) δ 151.1, 136.5, 111.9, 83.3, 43.3, 31.7, 24.9, 20.4, 14.0; HRMS (ESI) m/z calc. for $C_{16}H_{27}BNO_2$. $[M+H]^+$: 276.2129, found: 276.2131.

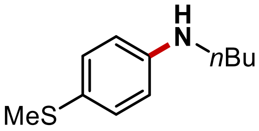

N-Bbutyl-4-(methylthio)aniline (58): Yellow oil; ^{1}H NMR (400 MHz, $CDCl_3$) δ 7.23 (d, J = 8.6 Hz, 2H), 6.55 (d, J = 8.6 Hz, 2H), 3.10 (t, J = 7.1 Hz, 2H), 2.41 (s, 3H), 1.68 – 1.53 (m, 2H), 1.51 – 1.36 (m, 2H), 0.96 (t, J = 7.3 Hz, 3H); ^{13}C NMR (100 MHz, $CDCl_3$) δ 147.6, 131.8, 123.9, 113.4, 43.8, 31.7, 20.4, 19.5, 14.0; HRMS (ESI) m/z calc. for $C_{11}H_{18}NS$ $[M+H]^+$: 196.1154, found: 196.1158.

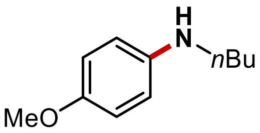

N-Butyl-4-methoxyaniline (59): Yellow oil; ^{1}H NMR (400 MHz, $CDCl_3$) δ 6.78 (d, J = 8.9 Hz, 2H), 6.58 (d, J = 8.9 Hz, 2H), 3.75 (s, 3H), 3.11 – 3.04 (m, 2H), 1.63 – 1.55 (m, 2H), 1.47-1.40 (m, 2H), 0.96 (t, J = 7.3 Hz, 3H); ^{13}C NMR (100 MHz, $CDCl_3$) δ 152.1, 143.0, 115.0, 114.2, 56.0, 44.9, 31.9, 20.5, 14.1; HRMS (ESI) m/z calc. for $C_{11}H_{18}NO$ $[M+H]^+$: 180.1383, found: 180.1386.

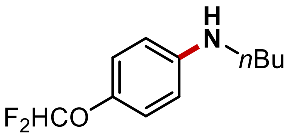

N-Butyl-4-(difluoromethoxy)aniline (60): Yellow oil; ^{1}H NMR (400 MHz, $CDCl_3$) δ 6.96 (d, J = 8.9 Hz, 2H), 6.55 (d, J = 8.9 Hz, 2H), 6.27 (t, J = 75.0 Hz, 1H), 3.09 (t, J = 7.1 Hz, 2H), 2.66 (br, 1H), 1.64 – 1.56 (m, 2H), 1.44 – 1.38 (m, 2H), 0.96 (t, J = 7.3 Hz, 3H); ^{13}C NMR (100 MHz, $CDCl_3$) δ 146.4, 142.3, 121.4, 116.6 (t, J = 258.7 Hz), 113.2, 44.1, 31.6, 20.3, 13.9; ^{19}F NMR (376 MHz, $CDCl_3$) δ -81.35 (d, J = 74.5 Hz, OCF_2); HRMS (ESI) m/z calc. for $C_{11}H_{16}F_2NO$ $[M+H]^+$: 216.1194, found: 216.1198.

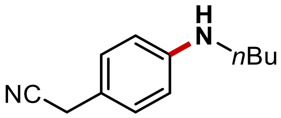

2-(4-(Butylamino)phenyl)acetonitrile (61): Yellow oil; ^{1}H NMR (400 MHz, $CDCl_3$) δ 7.09 (d, J = 8.1 Hz, 2H), 6.58 (d, J = 8.2 Hz, 2H), 3.61 (s, 2H), 3.10 (t, J = 7.1 Hz, 2H), 1.65 – 1.56 (m, 2H), 1.45 – 1.40 (m, 2H), 0.96 (t, J = 7.3 Hz, 3H); ^{13}C NMR (100 MHz, $CDCl_3$) δ 148.4 , 129.0, 118.7, 117.8, 113.1, 43.7 , 31.7, 22.9, 20.4 , 14.0; HRMS (ESI) m/z calc. for $C_{12}H_{17}N_2$ $[M+H]^+$: 189.1386, found: 189.1390.

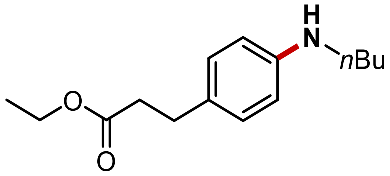

Ethyl 3-(4-(butylamino)phenyl)propanoate(62): Yellow oil; ^{1}H NMR (400 MHz, $CDCl_3$) δ 7.00 (d, J = 8.4 Hz, 2H), 6.54 (d, J = 8.4 Hz, 2H), 4.12 (q, J = 7.1 Hz, 2H), 3.09 (t, J = 7.1 Hz, 2H), 2.87 – 2.78 (m, 2H), 2.65 – 2.53 (m, 2H), 1.59 (m, 2H), 1.46 – 1.36 (m, 2H), 1.24 (t, J = 7.1 Hz, 3H), 0.95 (t, J = 7.3 Hz, 3H); ^{13}C NMR (100 MHz, $CDCl_3$) δ 173.4, 147.1 , 129.2, 129.2, 113.0, 60.4 , 44.1, 36.6, 31.9, 30.3, 20.4 , 14.4, 14.1; HRMS (ESI) m/z calc. for $C_{15}H_{24}NO_2$. $[M+H]^+$: 250.1802, found: 250.1805.

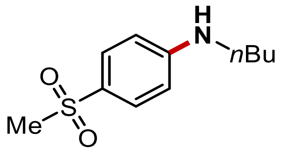

***N*-Butyl-4-(methylsulfonyl)aniline (63):** Yellow oil; ^{1}H NMR (400 MHz, $CDCl_3$) δ 7.65 (d, *J* = 8.8 Hz, 2H), 6.59 (d, *J* = 8.8 Hz, 2H), 4.37 (br, 1H), 3.15 (s, 2H), 2.98 (s, 3H), 1.70 – 1.51 (m, 2H), 1.44 – 1.38(m, 2H), 0.94 (t, *J* = 7.3 Hz, 3H); ^{13}C NMR (100 MHz, $CDCl_3$) δ 152.5, 129.3, 126.7, 111.6, 45.1, 43.0, 31.2, 20.2, 13.8; HRMS (ESI) m/z calc. for $C_{11}H_{18}NO_2S$ $[M+H]^+$: 228.1053, found: 228.1056.

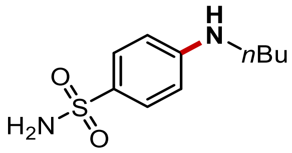

4-(Butylamino)benzenesulfonamide (64): White solid; ^{1}H NMR (400 MHz, $CDCl_3$) δ 7.70 (d, *J* = 8.4 Hz, 2H), 6.58 (d, *J* = 8.5 Hz, 2H), 4.67 (br, 2H), 3.16 (t, *J* = 7.1 Hz, 2H), 1.66 – 1.59 (m, 2H), 1.48 – 1.40 (m, 2H), 0.97 (t, *J* = 7.3 Hz, 3H); ^{13}C NMR (100 MHz, $CDCl_3$) δ 152.0, 128.7, 128.4, 111.7, 43.2, 31.4, 20.3, 14.0; HRMS (ESI) m/z calc. for $C_6H_8N_2O_2S$ $[M+H]^+$: 228.1053, found: 228.1056.

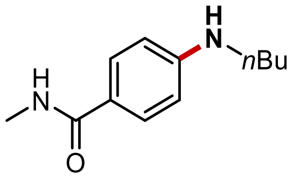

Ethyl 3-(4-(butylamino)phenyl)propanoate(65): Yellow oil; ^{1}H NMR (400 MHz, d_6-DMSO) δ 7.93 (br, 1H), 7.59 (d, *J* = 8.5 Hz, 2H), 6.53 (d, *J* = 8.5 Hz, 2H), 6.05 (br, 1H), 3.35 (s, 3H), 3.05 – 3.00 (m, 2H), 1.63 – 1.45 (m, 2H), 1.48 – 1.31 (m, 2H), 0.91 (t, *J* = 7.3 Hz, 3H); ^{13}C NMR (100 MHz, d_6-DMSO) δ 167.2, 151.8, 128.9, 121.5, 111.1, 42.6, 31.2, 26.5, 20.3, 14.2; HRMS (ESI) m/z calc. for $C_{15}H_{24}NO_2$ $[M+H]^+$: 250.1800, found: 250.1805.

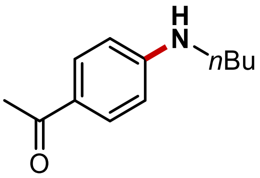

1-(4-(Butylamino)phenyl)ethan-1-one (66): Yellow oil; ^{1}H NMR (400 MHz, $CDCl_3$) δ 7.81 (d, *J* = 8.3 Hz, 2H), 6.54 (d, *J* = 8.3 Hz, 2H), 4.24 (br, 1H), 3.17 (t, *J* = 7.1 Hz, 2H), 2.48 (s, 3H), 1.71 – 1.54 (m, 2H), 1.48 – 1.31 (m, 2H), 0.96 (t, *J* = 7.3 Hz, 3H); ^{13}C NMR (100 MHz, $CDCl_3$) δ 196.3, 152.4, 130.8, 126.4, 111.2, 43.0, 31.4, 25.9, 20.2, 13.8; HRMS (ESI) m/z calc. for $C_{12}H_{18}NO$ $[M+H]^+$: 192.1383, found: 192.1386.

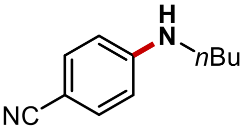

4-(Butylamino)benzonitrile (67): Yellow oil; ^{1}H NMR (400 MHz, $CDCl_3$) δ 7.39 (d, *J* = 8.3 Hz, 2H), 6.53 (d, *J* = 8.4 Hz, 2H), 4.24 (br, 1H), 3.13 (s, 2H), 1.77 – 1.55 (m, 2H), 1.47 – 1.39 (m, 2H), 0.96 (t, *J* = 7.3 Hz, 3H); ^{13}C NMR (100 MHz, $CDCl_3$) δ 151.6, 133.7, 120.6, 112.0, 98.2, 42.9, 31.2, 20.2, 13.8; HRMS (ESI) m/z calc. for $C_{11}H_{15}N_2$ $[M+H]^+$: 175.1230, found: 175.1235.

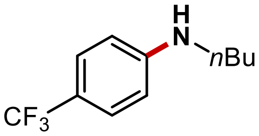

N-Butyl-4-(trifluoromethyl)aniline (68): Yellow oil; ^{1}H NMR (400 MHz, $CDCl_3$) δ 7.38 (d, *J* = 8.8 Hz, 2H), 6.59 (d, *J* = 8.6 Hz, 2H), 3.95 (br, 1H), 3.14 (t, *J* = 7.1 Hz, 2H), 1.65 – 1.58 (m, 2H), 1.49 – 1.39 (m, 2H), 0.97 (t, *J* = 7.3 Hz, 3H); ^{13}C NMR (100 MHz, $CDCl_3$) δ150.9, 126.5 (q, *J* = 3.8 Hz), 125.1 (q, *J* = 270.2 Hz), 118.4 (q, *J* = 32.6 Hz), 111.6, 43.2, 31.4, 20.2, 13.8; ^{19}F NMR (376 MHz, $CDCl_3$) δ -60.91 (s, CF_3); HRMS (ESI) m/z calc. for $C_{11}H_{15}F_3N$ $[M+H]^+$: 218.1151, found:218.1152.

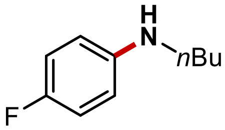

N-Butyl-4-fluoroaniline (69): Yellow oil; ^{1}H NMR (400 MHz, $CDCl_3$) δ 6.88 (t, *J* = 8.6 Hz, 2H), 6.55 – 6.52 (m, 2H), 3.07 (t, *J* = 7.1 Hz, 2H), 1.69 – 1.53 (m, 2H), 1.53 – 1.33 (m, 2H), 0.96 (t, *J* = 7.3 Hz, 3H); ^{13}C NMR (100 MHz, $CDCl_3$) δ 155.7 (d, *J* = 234.4 Hz), 144.9, 115.6 (d, *J* = 22.3 Hz), 113.5 (d, *J* = 7.4 Hz), 44.4, 31.7, 20.2, 13.9; ^{19}F NMR (376 MHz, $CDCl_3$) δ –128.59 (s, F); HRMS (ESI) m/z calc. for $C_{10}H_{15}FN$ $[M+H]^+$: 168.1183, found: 168.1186.

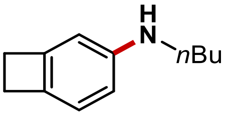

N-Butylbicyclo[4.2.0]octa-1(6),2,4-trien-3-amine (70): Yellow oil; ^{1}H NMR (400 MHz, $CDCl_3$) δ 6.85 (d, *J* = 7.9 Hz, 1H), 6.48 – 6.46 (m, 1H), 6.41 (s, 1H), 3.12 – 3.07 (m, 6H), 1.65 – 1.57 (m, 2H), 1.49 – 1.39 (m, 2H), 0.97 (t, *J* = 7.3 Hz, 3H); ^{13}C NMR (100 MHz, $CDCl_3$) δ 148.4, 146.7, 134.1, 123.2, 112.2, 107.6, 44.4, 31.8, 29.2, 28.8, 20.5, 14.0; HRMS (ESI) m/z calc. for $C_{12}H_{18}N$ $[M+H]^+$: 176.1434, found: 176.1438;

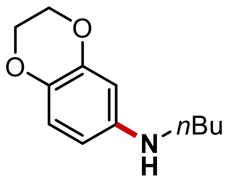

N-Butyl-2,3-dihydrobenzo[b][1,4]dioxin-6-amine (71): Yellow oil; ^{1}H NMR (400 MHz, $CDCl_3$) δ 6.69 (d, *J* = 8.4 Hz, 1H), 6.17 – 6.13 (m, 2H), 4.24 – 4.21 (m, 2H), 4.20 – 4.15 (m, 2H), 3.03 (t, *J* = 7.1 Hz, 2H), 161 – 1.54 (m, 2H), 1.46 – 1.36 (m, 2H), 0.94 (t, *J* = 7.3 Hz, 3H); ^{13}C NMR (100 MHz, $CDCl_3$) δ 144.2, 143.7, 135.6, 117.7, 107.0, 101.6, 64.9, 64.3, 44.7, 31.8, 20.4, 14.0; HRMS (ESI) m/z calc. for $C_{12}H_{18}NO_2$ $[M+H]^+$:208.1332, found: 208.1335.

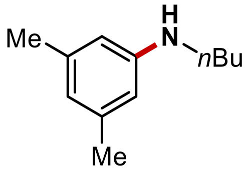

N-Butyl-3,5-dimethylaniline (72): Yellow oil; ^{1}H NMR (400 MHz, $CDCl_3$) δ 6.36 (s, 1H), 6.26 (s, 2H), 3.10 (t, *J* = 7.1 Hz, 2H), 2.24 (s, 6H), 1.61 – 1.58 (m, 2H), 1.46 – 1.34 (m, 2H), 0.96 (t, *J* = 7.3 Hz, 3H); ^{13}C NMR (100 MHz, $CDCl_3$) δ 148.6, 138.9, 119.2, 110.7, 43.8, 31.8, 21.5, 20.3, 13.9; HRMS (ESI) m/z calc. for $C_{12}H_{20}N$ $[M+H]^+$: 178.1590, found: 178.1592.

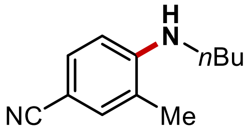

4-(Butylamino)-3-methylbenzonitrile (73): Yellow oil; ^{1}H NMR (400 MHz, $CDCl_3$) δ 7.38 (d, *J* = 8.4 Hz, 1H), 7.26 (d, *J* = 1.9 Hz, 1H), 6.54 (d, *J* = 8.5 Hz, 1H), 3.97 (s, 1H), 3.21 – 3.16 (m, 2H), 2.10 (s, 3H), 1.69 – 1.59 (m, 2H), 1.47 – 1.40 (m, 2H), 0.97 (t, *J* = 7.3 Hz, 3H); ^{13}C NMR (100 MHz, $CDCl_3$) δ 149.7, 133.3, 130.9, 121.6, 120.6, 108.9, 97.9, 43.1, 31.3, 20.2, 17.1, 13.8; HRMS (ESI) m/z calc. for $C_{12}H_{17}N_2$ $[M+H]^+$: 189.1386, found: 189.1390.

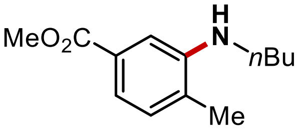

Methyl 4-(butylamino)-3-methylbenzoate (74): Yellow oil; ^{1}H NMR (400 MHz, $CDCl_3$) δ 7.83 (d, *J* = 2.0 Hz, 1H), 7.73 (s, 1H), 6.56 (d, *J* = 8.5 Hz, 1H), 3.85 (s, 3H), 3.21 (t, *J* = 7.1 Hz, 2H), 2.14 (s, 3H), 1.67 – 1.65 (m, 2H), 1.47 – 1.45 (m, 2H), 0.98 (t, *J* = 7.3 Hz, 3H); ^{13}C NMR (100 MHz, $CDCl_3$) δ 167.6, 150.2, 131.5, 129.8, 120.5, 117.5, 108.2, 51.5, 43.2, 31.5, 20.3, 17.2, 13.9; HRMS (ESI) m/z calc. for $C_{13}H_{20}NO_2$ $[M+H]^+$: 222.1489, found: 222.1491.

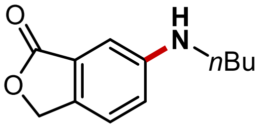

6-(Butylamino)isobenzofuran-1(3H)-one (75): Yellow oil; ^{1}H NMR (400 MHz, CDCl$_3$) δ 7.63 (s, 1H), 7.51 (d, *J* = 8.1 Hz, 1H), 7.20 (d, *J* = 8.1 Hz, 1H), 6.80 (br, 1H), 4.50 (s, 2H), 3.43 – 3.38 (m, 2H), 1.73 – 1.50 (m, 2H), 1.49 – 1.29 (m, 2H), 0.95 (t, *J* = 7.3 Hz, 3H); ^{13}C NMR (100 MHz, CDCl$_3$) δ 168.4, 138.6, 137.8, 133.8, 132.2, 130.8, 121.8, 63.8, 40.1, 31.5, 20.1, 13.7; HRMS (ESI) m/z calc. for $C_{12}H_{16}NO_2$ [M+H]$^+$: 206.1176, found: 206.1180.

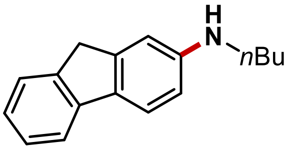

N-Butyl-9H-fluoren-2-amine (76): Yellow oil; ^{1}H NMR (400 MHz, CDCl$_3$) δ 7.62 (d, *J* = 7.6 Hz, 1H), 7.58 (d, *J* = 8.2 Hz, 1H), 7.46 (d, *J* = 7.4 Hz, 1H), 7.31 (t, *J* = 7.5 Hz, 1H), 7.18 (t, *J* = 7.4 Hz, 1H), 6.81 (s, 1H), 6.65 – 6.62 (m, 1H), 3.82 (s, 2H), 3.19 (t, *J* = 7.1 Hz, 2H), 1.69 – 1.62 (m, 2H), 1.55 – 1.40 (m, 2H), 0.99 (t, *J* = 7.3 Hz, 3H); ^{13}C NMR (100 MHz, CDCl$_3$) δ 148.2, 145.3, 142.6, 142.3, 131.8, 126.7, 124.8, 124.7, 120.8, 118.5, 112.1, 109.2, 44.2, 37.1, 31.9, 20.5, 14.1; HRMS (ESI) m/z calc. for $C_{17}H_{20}N$ [M+H]$^+$: 238.1590, found: 238.1594.

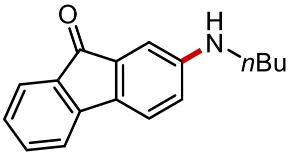

2-(Butylamino)-9H-fluoren-9-one (77): red oil; ^{1}H NMR (400 MHz, CDCl$_3$) δ 7.54 (d, *J* = 7.3 Hz, 1H), 7.35 (d, *J* = 7.4 Hz, 1H), 7.25 – 7.29 (m, 3H), 7.10 (t, *J* = 7.4 Hz, 1H), 6.89 (d, *J* = 2.2 Hz, 1H), 6.63 – 6.58 (m, 1H), 3.88 (br, 1H), 3.15 (t, *J* = 7.1 Hz, 2H), 1.66 – 1.55 (m, 2H), 1.47 – 1.40 (m, 3H), 0.96 (t, *J* = 7.3 Hz, 3H); ^{13}C NMR (100 MHz, CDCl$_3$) δ 194.8, 149.6, 146.0, 135.9, 134.1 132.9, 126.84, 124.1, 121.3, 120.8, 118.8, 117.2, 108.4, 43.7, 35.0, 20.3, 13.9; HRMS (ESI) m/z calc. for $C_{17}H_{18}NO$ [M+H]$^+$: 252.1383, found: 252.1385.

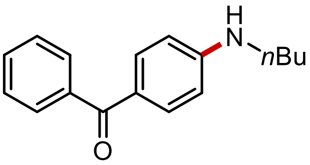

(4-(Butylamino)phenyl)(phenyl)methanone (78): yellow oil; ^{1}H NMR (400 MHz, CDCl$_3$) δ 7.81 – 7.67 (m, 4H), 7.53 – 7.47 (m, 1H), 7.47 – 7.38 (m, 2H), 6.55 (d, *J* = 8.8 Hz, 2H), 4.50 (br, 1H), 3.15 (t, *J* = 7.1 Hz, 2H), 1.67 – 1.46 (m, 2H), 1.45 – 1.37 (m, 2H), 0.93 (t, *J* = 7.3 Hz, 3H); ^{13}C NMR (150 MHz, CDCl$_3$) δ 195.2, 152.5, 139.3, 133.1, 131.2, 129.4, 128.1, 125.6, 111.2, 43.0, 31.4, 20.23, 13.9; HRMS (ESI) m/z calc. for $C_{17}H_{20}NO$ [M+H]$^+$: 254.1539, found: 254.1537.

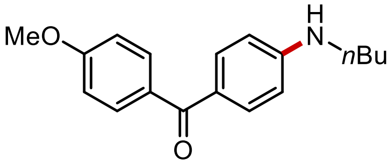

(4-(Butylamino)phenyl)(4-methoxyphenyl)methanone (79): yellow oil; ^{1}H NMR (400 MHz, $CDCl_3$) δ 7.74 (d, *J* = 8.7 Hz, 2H), 7.70 (d, *J* = 8.7 Hz, 2H), 6.94 (d, *J* = 8.7 Hz, 2H), 6.57 (d, *J* = 8.7 Hz, 2H), 4.43 (br, 1H), 3.86 (s, 3H), 3.17 (t, *J* = 7.1 Hz, 2H), 1.69 – 1.57 (m, 2H), 1.48 – 1.39 (m, 2H), 0.95 (t, *J* = 7.3 Hz, 3H); ^{13}C NMR (100 MHz, $CDCl_3$) δ 194.1, 162.3, 152.1, 132.7, 131.8, 131.6, 126.1, 113.3, 111.1, 55.4, 43.0, 31.4, 20.2, 13.8; HRMS (ESI) m/z calc. for $C_{18}H_{22}NO_2$ $[M+H]^+$: 284.1645, found: 284.1648.

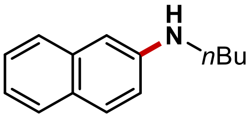

N-Butylnaphthalen-2-amine (80): Yellow oil; ^{1}H NMR (400 MHz, $CDCl_3$) δ 7.65 (d, *J* = 8.1 Hz, 1H), 7.60 (d, *J* = 8.6 Hz, 2H), 7.34 (t, *J* = 7.5 Hz, 1H), 7.17 (t, *J* = 7.4 Hz, 1H), 6.88 – 6.81 (m, 1H), 6.78 (s, 1H), 3.19 (t, *J* = 7.1 Hz, 2H), 1.71 – 1.59 (m, 2H), 1.54 – 1.36 (m, 2H), 0.97 (t, *J* = 7.3 Hz, 3H); ^{13}C NMR (100 MHz, $CDCl_3$) δ 146.2, 135.4, 128.9, 127.7, 127.5, 126.3, 125.9, 121.8, 118.0, 104.3, 43.8, 31.6, 20.4, 14.0; HRMS (ESI) m/z calc. for $C_{14}H_{18}N$ $[M+H]^+$: 200.1434, found: 200.1439.

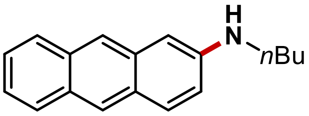

N-Butylanthracen-2-amine (81): Yellow oil; ^{1}H NMR (400 MHz, $CDCl_3$) δ 8.22 (s, 1H), 8.11 (s, 1H), 7.88 (t, *J* = 9.0 Hz, 2H), 7.78 (d, *J* = 9.0 Hz, 1H), 7.41 – 7.34 (m, 1H), 7.33 – 7.29 (m, 1H), 6.91 – 6.88 (m, 1H), 6.85 (s, 1H), 3.27 (t, *J* = 7.1 Hz, 2H), 1.75 – 1.68 (m, 2H), 1.53 – 1.47 (m, 2H), 1.01 (t, *J* = 7.3 Hz, 3H); ^{13}C NMR (100 MHz, $CDCl_3$) δ 145.2, 133.8, 132.5, 129.4, 129.3, 128.2, 127.5, 127.4, 126.1, 125.2, 123.5, 122.4, 120.3, 101.3, 43.7, 31.5, 20.4, 14.0; HRMS (ESI) m/z calc. for $C_{18}H_{20}N$ $[M+H]^+$: 250.1590, found: 250.1595.

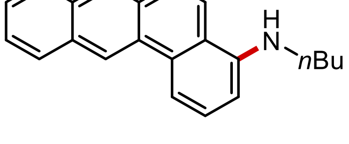

N-Butyltetraphen-4-amine (82): yellow oil; ^{1}H NMR (400 MHz, $CDCl_3$) δ 9.15 (s, 1H), 8.34 (s, 1H), 8.24 (d, *J* = 8.2 Hz, 1H), 8.13 – 8.07 (m, 1H), 8.06 – 7.99 (m, 1H), 7.77 (d, *J* = 9.5 Hz, 1H), 7.67 (d, *J* = 9.3 Hz, 1H), 7.59 – 7.48 (m, 3H), 6.86 (d, *J* = 7.8 Hz, 1H), 4.32 (br, 1H), 3.36 – 3.24 (m, 2H), 1.79 (m, 2H), 1.55 (m, 3H), 1.02 (q, *J* = 7.6 Hz, 3H); ^{13}C NMR (100 MHz, $CDCl_3$) δ 144.7, 132.0, 131.9, 131.4, 130.4, 129.4, 128.6, 127.7, 126.5, 125.8, 125.7, 125.3, 122.2, 119.4, 119.0, 112.2, 107.8, 44.2, 31.7, 20.5, 14.0; HRMS (ESI) m/z calc. for $C_{18}H_{13}N$ $[M+H]^+$: 243.1043, found: 243.1047.

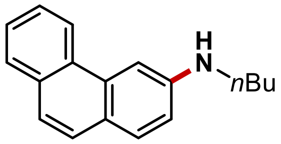

N-Butylphenanthren-3-amine (83): Yellow oil;^{1}H NMR (400 MHz, $CDCl_3$) δ 8.62 (d, *J* = 8.0 Hz, 1H), 7.91 – 7.84 (m, 1H), 7.74 – 7.70 (m, 2H), 7.68 – 7.56 (m, 3H), 7.53 – 7.51 (m, 1H), 6.98 – 6.96 (m, 1H), 3.33 (t, *J* = 7.1 Hz, 2H), 1.77 – 1.69 (m, 2H), 1.58 – 1.49(m, 2H), 1.10 – 1.01 (m, 3H); ^{13}C NMR (100 MHz, $CDCl_3$) δ 147.4, 132.9, 132.1, 129.7, 129.6, 128.6, 127.0, 126.4, 125.7, 124.8, 122.8, 122.7, 116.1, 102.3, 43.9, 31.8, 20.5, 14.1; HRMS (ESI) m/z calc. for $C_{18}H_{20}N$ $[M+H]^+$: 250.1590, found: 250.1593.

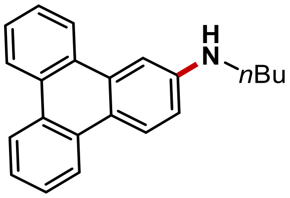

N-Pentyltriphenylen-2-amine (84): Yellow oil; ^{1}H NMR (400 MHz, $CDCl_3$) δ 8.68 – 8.54 (m, 3H), 8.50 (d, *J* = 8.3 Hz, 1H), 8.44 (d, *J* = 8.9 Hz, 1H), 7.71 (d, *J* = 2.3 Hz, 1H), 7.69 – 7.58 (m, 3H), 7.56 – 7.52 (m, 1H), 6.99 – 6.97 (m, 1H), 3.33 (t, *J* = 7.1 Hz, 2H), 1.76 – 1.69 (m, 2H), 1.60 – 1.44 (m, 2H), 1.03 (t, *J* = 7.3 Hz, 3H); ^{13}C NMR (100 MHz, $CDCl_3$) δ 147.8, 131.4, 130.7, 130.4, 129.8, 128.2, 127.2, 127.1, 126.9, 125.4, 124.7, 123.5, 123.4, 123.3, 122.3, 121.2, 115.4, 104.0, 43.9, 31.8, 20.5, 14.1; HRMS (ESI) m/z calc. for $C_{23}H_{24}N$ $[M+H]^+$: 314.1903, found: 314.1907.

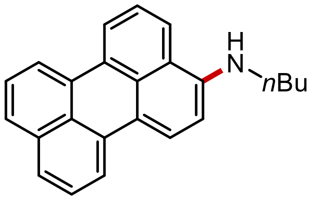

N-Butylperylen-3-amine (85): yellow oil; ^{1}H NMR (400 MHz, $CDCl_3$) δ 8.12 (dd, *J* = 7.4, 4.5 Hz, 3H), 8.02 (d, *J* = 8.3 Hz, 2H), 7.63 (d, *J* = 8.1 Hz, 2H), 7.54 (d, *J* = 8.3 Hz, 1H), 7.48 – 7.38 (m, 5H), 6.58 (br, 1H), 3.25 (br, 2H), 1.78 – 1.66 (m, 2H), 1.57 – 1.42 (m, 2H), 0.99 (t, *J* = 7.3 Hz, 3H); ^{13}C NMR (100 MHz, $CDCl_3$) δ 134.8, 131.3, 128.8, 127.8, 126.5, 125.3, 123.8, 120.6, 120.2, 119.6, 43.8, 31.6, 20.5, 14.0; HRMS (ESI) m/z calc. for $C_{24}H_{22}N$ $[M+H]^+$: 324.1747, found: 324.1750.

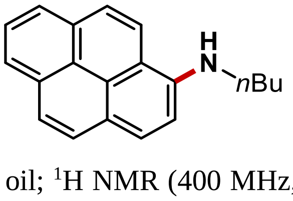

N-Butylpyren-1-amine (86): Yellow oil; ^{1}H NMR (400 MHz, $CDCl_3$) δ 8.08 – 7.98 (m, 3H), 7.99 – 7.94 (m, 2H), 7.93 – 7.88 (m, 2H), 7.77 – 7.75 (m, 1H), 7.33 (d, *J* = 8.4 Hz, 1H), 3.44 (t, *J* = 7.1 Hz, 2H), 1.91 – 1.75 (m, 2H), 1.65 – 1.51 (m, 2H), 1.05 (t, *J* = 7.4 Hz, 3H); ^{13}C NMR (100 MHz, $CDCl_3$) δ 142.7, 132.5, 131.7, 127.8, 126.4, 126.0, 125.9, 125.8, 125.7, 123.7, 123.1,

119.3, 116.3, 108.9, 44.2, 31.8, 20.5, 14.0; HRMS (ESI) m/z calc. for $C_{20}H_{20}N$ $[M+H]^+$: 274.1590, found: 274.1594.

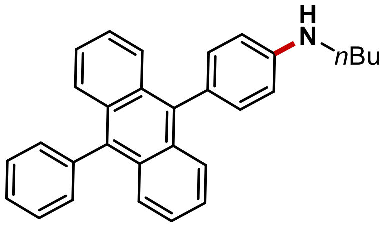

***N*-Butyl-4-(10-phenylanthracen-9-yl)aniline (87):** Yellow oil; ^{1}H NMR (400 MHz, $CDCl_3$) δ 7.89 – 7.82 (m, 2H), 7.72 – 7.65 (m, 2H), 7.62 – 7.58 (m, 2H), 7.57 – 7.51 (m, 1H), 7.51 – 7.45 (m, 2H), 7.35 – 7.30 (m, 4H), 7.29 – 7.27 (m, 2H), 6.85 – 6.83 (m, 2H), 3.27 (t, *J* = 7.1 Hz, 2H), 1.77 – 1.70 (m, 2H), 1.58 – 1.49 (m, 2H), 1.04 (t, *J* = 7.3 Hz, 3H); ^{13}C NMR (100 MHz, $CDCl_3$) δ 148.0, 139.5, 138.0, 136.6, 132.4, 131.5, 130.5, 130.1, 128.5, 127.6, 127.5, 127.4, 127.0, 125.0, 124.8, 112.7, 44.0, 32.0, 20.6, 14.1; HRMS (ESI) m/z calc. for $C_{30}H_{28}N$ $[M+H]^+$: 402.2216, found: 402.2220.

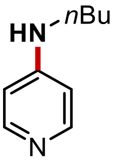

***N*-Butylpyridin-4-amine (88):** White solid; ^{1}H NMR (400 MHz, $CDCl_3$) δ 8.10 (d, *J* = 4.9 Hz, 2H), 6.52 (d, *J* = 5.6 Hz, 2H), 5.25 (br, 1H), 3.20 – 3.15 (m, 2H), 1.72 – 1.67 (m, 2H), 1.51 – 1.43 (m, 2H), 0.96 (t, *J* = 7.3 Hz, 3H); ^{13}C NMR (100 MHz, $CDCl_3$) δ 154.6, 147.4, 107.4, 42.4, 31.0, 20.1, 13.8; HRMS (ESI) m/z calc. for $C_9H_{15}N_2$ $[M+H]^+$: 151.1230, found: 151.1234.

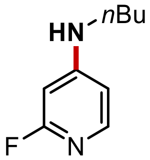

***N*-Butyl-2-fluoropyridin-4-amine (89):** Yellow oil; ^{1}H NMR (400 MHz, $CDCl_3$) δ 7.86 (d, *J* = 5.4 Hz, 1H), 6.69 – 6.68 (m, 1H), 6.53 (d, *J* = 1.5 Hz, 1H), 4.70 (br, 1H), 3.23 – 3.18 (m, 2H), 1.67 – 1.53 (m, 2H), 1.46 – 1.36 (m, 2H), 0.94 (t, *J* = 7.3 Hz, 3H); ^{13}C NMR (100 MHz, $CDCl_3$) δ 159.8, 149.1, 133.7, 116.0, 109.0, 42.1, 31.6, 20.3, 13.9; ^{19}F NMR (376 MHz, $CDCl_3$) δ -71.37 (s, F); HRMS (ESI) m/z calc. for $C_9H_{14}FN_2$ $[M+H]^+$: 169.1136,found:169.1140.

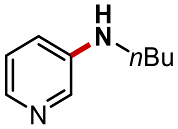

***N*-Butylpyridin-3-amine (90):** Yellow oil; ^{1}H NMR (400 MHz, $CDCl_3$) δ 8.43 – 8.15 (m, 2H), 7.40 – 7.38 (m, 1H), 7.18 (d, *J* = 8.1 Hz, 1H), 4.05 (br, 1H), 3.44 (t, *J* = 7.1 Hz, 2H), 1.97 –

1.90 (m, 2H), 1.85 – 1.68 (m, 2H), 1.28 (t, *J* = 7.3 Hz, 3H); ^{13}C NMR (100 MHz, CDCl$_3$) δ 144.6, 138.4, 136.0, 123.8, 118.4, 43.4, 31.6, 20.3, 13.9; HRMS (ESI) m/z calc. for $C_9H_{15}N_2$ [M+H]$^+$: 151.1230, found: 151.1231.

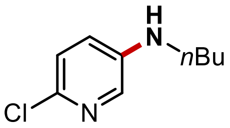

***N*-Butyl-6-chloropyridin-3-amine (91):** White solid; ^{1}H NMR (400 MHz, CDCl$_3$) δ 7.75 (d, *J* = 2.5 Hz, 1H), 7.08 (d, *J* = 8.6 Hz, 1H), 6.86 – 6.83 (m, 1H), 3.11 – 3.08 (m, 2H), 1.69 – 1.56 (m, 2H), 1.47 – 1.38 (m, 2H), 0.96 (t, *J* = 7.3 Hz, 3H); ^{13}C NMR (100 MHz, CDCl$_3$) δ 143.5, 134.4, 124.0, 122.0, 100.0, 43.5, 31.4, 20.2, 13.8; HRMS (ESI) m/z calc. for $C_9H_{14}ClN_2$ [M+H]$^+$: 185.0840, found: 185.0845.

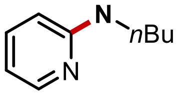

***N*-Butylpyridin-2-amine (92):** White solid; ^{1}H NMR (400 MHz, CDCl$_3$) δ 8.06 (d, *J* = 4.4 Hz, 1H), 7.46 – 7.37 (m, 1H), 6.56 – 6.54 (m, 1H), 6.37 (d, *J* = 8.4 Hz, 1H), 4.56 (br, 1H), 3.27 – 3.22 (m, 2H), 1.64 – 1.57 (m 2H), 1.48 – 1.38 (m, 2H), 0.95 (t, *J* = 7.3 Hz, 3H); ^{13}C NMR (100 MHz, CDCl$_3$) δ 158.9, 148.0, 137.5, 112.6, 106.3, 42.0, 31.7, 20.2, 13.9; HRMS (ESI) m/z calc. for $C_9H_{15}N_2$ [M+H]$^+$: 151.1230, found: 151.1234.

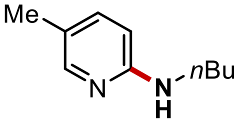

***N*-Butyl-5-methylpyridin-2-amine (93):** Yellow oil; ^{1}H NMR (400 MHz, CDCl$_3$) δ 7.92 (d, *J* = 5.1 Hz, 1H), 6.40 (d, *J* = 5.1 Hz, 1H), 6.19 (s, 1H), 4.46 (br, 1H), 3.25 – 3.20 (m, 2H), 2.23 (s, 3H), 1.64 – 1.56 (m, 2H), 1.47 – 1.38 (m, 2H), 0.95 (t, *J* = 7.3 Hz, 3H); ^{13}C NMR (100 MHz, CDCl$_3$) δ 159.2, 148.5, 147.7, 114.2, 106.5, 42.1, 31.7, 21.2, 20.2, 13.9; HRMS (ESI) m/z calc. for $C_{10}H_{17}N_2$ [M+H]$^+$: 165.1386, found: 165.1381.

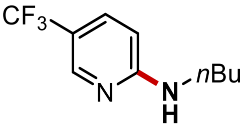

***N*-Butyl-5-(trifluoromethyl)pyridin-2-amine (94):** Yellow oil; ^{1}H NMR (400 MHz, CDCl$_3$) δ 8.31 (s, 1H), 7.57 (dd, *J* = 8.8, 2.3 Hz, 1H), 6.38 (d, *J* = 8.8 Hz, 1H), 4.96 (br, 1H), 3.30 (q, *J* = 6.9 Hz, 2H), 1.71 – 1.54 (m, 2H), 1.47-1.38 (m, 2H), 0.96 (t, *J* = 7.3 Hz, 3H); ^{13}C NMR (100 MHz, CDCl$_3$) δ 160.6, 146.2 (q, *J* = 4.4 Hz), 134.6 (q, *J* = 3.1 Hz), 124.8 (d, *J* = 270.1 Hz),

115.3 (q, J = 32.5 Hz), 105.7, 42.0, 31.6, 20.3, 13.9; ^{19}F NMR (376 MHz, $CDCl_3$) δ -64.83 (s, CF_3); HRMS (ESI) m/z calc. for $C_{10}H_{14}F_3N_2$ $[M+H]^+$: 219.1104, found: 219.1108.

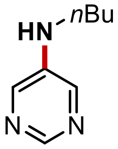

***N*-Butylpyrimidin-5-amine (95):** Yellow oil; ^{1}H NMR (400 MHz, $CDCl_3$) δ 8.56 (s, 1H), 8.09 (s, 2H), 3.74 (br, 1H), 3.14 (t, J = 6.8 Hz, 2H), 1.69 – 1.58 (m, 2H), 1.48 – 1.39 (m, 2H), 0.96 (t, J = 7.3 Hz, 3H); ^{13}C NMR (100 MHz, $CDCl_3$) δ 148.4, 142.1, 140.8, 43.0, 31.4, 20.2, 13.9; HRMS (ESI) m/z calc. for $C_8H_{14}N_3$ $[M+H]^+$: 152.1182, found: 152.1185.

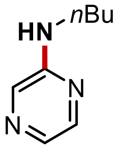

***N*-Bbutylpyrazin-2-amine (96):** Yellow oil; ^{1}H NMR (400 MHz, $CDCl_3$) δ 7.96 (s, 1H), 7.86 (s, 1H), 7.77 (s, 1H), 4.61 (br, 1H), 3.33 (q, J = 6.6 Hz, 2H), 1.70 – 1.52 (m, 2H), 1.52 – 1.35 (m, 2H), 0.95 (t, J = 7.3 Hz, 3H); ^{13}C NMR (100 MHz, $CDCl_3$) δ 154.9, 142.1, 132.7, 131.9, 41.4, 31.7, 20.3, 13.9; HRMS (ESI) m/z calc. for $C_8H_{14}N_3$ $[M+H]^+$: 152.1182, found: 152.1186.

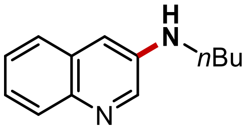

***N*-Butylquinolin-3-amine (97):** Yellow oil; ^{1}H NMR (400 MHz, $CDCl_3$) δ 8.42 (d, J = 2.8 Hz, 1H), 8.00 – 7.90 (m, 1H), 7.61 – 7.60 (m, 1H), 7.45 – 7.35 (m, 2H), 7.00 (d, J = 2.7 Hz, 1H), 3.95 (br, 1H), 3.20 (t, J = 7.1 Hz, 2H), 1.72 – 1.65 (m, 2H), 1.53 – 1.44 (m, 2H), 0.99 (t, J = 7.3 Hz, 3H); ^{13}C NMR (100 MHz, $CDCl_3$) δ 143.5, 142.1, 142.0, 129.8, 129.1, 127.0, 126.0, 124.9, 110.0, 43.5, 31.4, 20.4, 14.0; HRMS (ESI) m/z calc. for $C_{13}H_{17}N_2$ $[M+H]^+$: 201.1386, found: 201.1390.

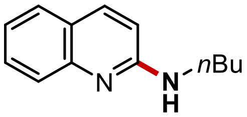

***N*-Butylquinolin-2-amine (98):** Yellow oil; ^{1}H NMR (400 MHz, $CDCl_3$) δ 7.81 (d, J = 8.9 Hz, 1H), 7.67 (d, J = 8.4 Hz, 1H), 7.57 (d, J = 7.9 Hz, 1H), 7.54 – 7.49 (m, 1H), 7.24 – 7.14 (m, 1H), 6.63 (d, J = 8.9 Hz, 1H), 4.73 (br, 1H), 3.48 (q, J = 7.0 Hz, 2H), 1.69 – 1.62 (m, 2H), 1.51 – 1.42 (m, 2H), 0.98 (t, J = 7.3 Hz, 3H); ^{13}C NMR (100 MHz, $CDCl_3$) δ 157.3, 148.3, 137.5, 129.7, 127.6, 126.2, 123.5, 122.0, 111.1, 41.7, 32.0, 20.4, 14.0; HRMS (ESI) m/z calc. for $C_{13}H_{17}N_2$ $[M+H]^+$: 201.1386, found: 201.1390.

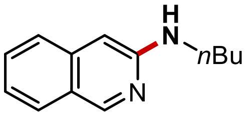

N-Butylisoquinolin-3-amine (99): Yellow oil; ^{1}H NMR (400 MHz, $CDCl_3$) δ 8.82 (s, 1H), 7.73 (d, *J* = 8.2 Hz, 1H), 7.54 (d, *J* = 8.3 Hz, 1H), 7.50 – 7.43 (m, 1H), 7.22 – 7.14 (m, 1H), 6.48 (s, 1H), 4.64 (br, 1H), 3.26 (q, *J* = 6.9 Hz, 2H), 1.70 – 1.65 (m, 2H), 1.51 – 1.46 (m, 2H), 0.99 (t, *J* = 7.4 Hz, 3H); ^{13}C NMR (100 MHz, $CDCl_3$) δ 155.8, 151.9, 139.3, 130.5, 128.0, 124.9, 123.6, 122.4, 95.5, 43.0, 31.5, 20.5, 14.0; HRMS (ESI) m/z calc. for $C_{13}H_{17}N_2$ $[M+H]^+$: 201.1386, found: 201.1390.

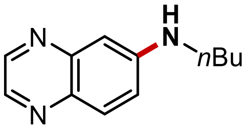

N-Butylquinoxalin-6-amine (100): Yellow oil;^{1}H NMR (400 MHz, $CDCl_3$) δ 8.61 (d, *J* = 1.7 Hz, 1H), 8.47 (d, *J* = 1.8 Hz, 1H), 7.80 (d, *J* = 9.1 Hz, 1H), 7.10 – 7.07 (m, 1H), 6.93 – 6.92 (m, 1H), 4.27 (br, 1H), 3.25 (t, *J* = 7.1 Hz, 2H), 1.77 – 1.59 (m, 2H), 1.58 – 1.37 (m, 2H), 0.97 (t, *J* = 7.4 Hz, 3H); ^{13}C NMR (100 MHz, $CDCl_3$) δ 149.5, 145.7, 145.0, 140.2, 138.1, 130.1, 122.4, 103.4, 43.5, 31.2, 20.4, 14.0; HRMS (ESI) m/z calc. for $C_{12}H_{16}N_3$ $[M+H]^+$: 202.1339, found:202.1340.

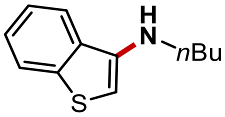

N-Butylbenzo[b]thiophen-3-amine (101): Yellow oil; ^{1}H NMR (400 MHz, $CDCl_3$) δ 7.79 – 7.77 (m, 1H), 7.61 – 7.52 (m, 1H), 7.40 – 7.30 (m, 2H), 6.04 (s, 1H), 3.80 (br, 1H), 3.24 (t, *J* = 7.1 Hz, 2H), 1.77 – 1.70 (m, 2H), 1.55 – 1.46 (m, 2H), 1.00 (t, *J* = 7.4 Hz, 3H); ^{13}C NMR (100 MHz, $CDCl_3$) δ 141.8, 139.4, 133.0, 124.8, 123.5, 123.4, 119.4, 94.7, 45.7, 31.8, 20.6, 14.1; HRMS (ESI) m/z calc. for $C_{12}H_{16}NS$ $[M+H]^+$: 206.0998, found: 206.0994.

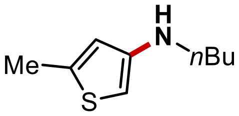

N-Butyl-5-methylthiophen-3-amine (102): Yellow oil; ^{1}H NMR (400 MHz, $CDCl_3$) δ 6.31 (s, 1H), 5.68 (s, 1H), 3.03 (t, *J* = 7.1 Hz, 2H), 2.39 (s, 3H), 1.67 – 1.54 (m, 2H), 1.44 – 1.40 (m, 2H), 0.97 – 0.93 (m, 3H); ^{13}C NMR (100 MHz, $CDCl_3$) δ 148.4, 139.4, 118.8, 92.7, 45.9, 31.9, 20.5, 15.8, 14.1; HRMS (ESI) m/z calc. for $C_9H_{16}NS$ $[M+H]^+$: 170.0998, found: 170.0995.

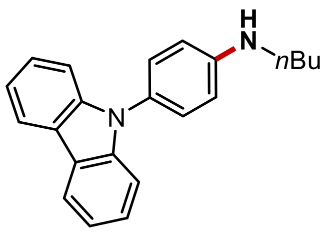

N-Butyl-4-(9H-carbazol-9-yl)aniline (103): Yellow oil; ^{1}H NMR (400 MHz, $CDCl_3$) δ 8.12 (d, J = 7.7 Hz, 2H), 7.41 – 7.19 (m, 8H), 6.77 – 6.69 (m, 2H), 4.28 – 3.39 (br, 1H), 3.16 (t, J = 7.1 Hz, 2H), 1.66 – 1.61 (m, 2H), 1.49 – 1.43 (m, 2H), 0.99 (t, J = 7.3 Hz, 3H); ^{13}C NMR (100 MHz, $CDCl_3$) δ 148.1, 141.8, 128.6, 126.8, 125.8, 123.1, 120.3, 119.4, 113.4, 110.0, 43.9, 31.8, 20.5, 14.1; HRMS (ESI) m/z calc. for $C_{22}H_{23}N_2$. $[M+H]^+$: 315.1856, found: 315.1860.

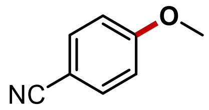

1-Methoxy-3,5-dimethylbenzene (104): Yellow oil; ^{1}H NMR (400 MHz, $CDCl_3$) δ 7.57 (d, J = 8.9 Hz, 2H), 6.94 (d, J = 8.9 Hz, 2H), 3.85 (s, 3H); ^{13}C NMR (100 MHz, $CDCl_3$) δ 162.9, 134.1, 119.3, 114.8, 104.0, 55.6; HRMS (ESI) m/z calc. for C_8H_8NO $[M+H]^+$: 134.0600, found: 134.0605.

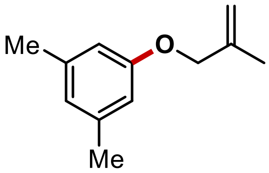

1,3-Dimethyl-5-((2-methylallyl)oxy)benzene (105): Yellow solid; ^{1}H NMR (400 MHz, $CDCl_3$) δ 6.61 (s, 1H), 6.57 (s, 2H), 5.10 (s, 1H), 4.98 (s, 1H), 4.41 (s, 2H), 2.29 (s, 6H), 1.84 (s, 3H); ^{13}C NMR (100 MHz, $CDCl_3$) δ 159.1, 141.3, 139.3, 122.7, 112.7, 112.6, 71.8, 21.6, 19.6; HRMS (ESI) m/z calc. for $C_{12}H_{17}O$ $[M+H]^+$: 177.1274, found: 177.1278.

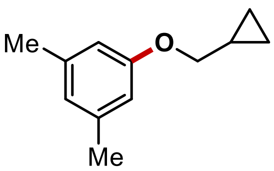

1-(Cyclopropylmethoxy)-3,5-dimethylbenzene (106): Yellow solid; H NMR (400 MHz, $CDCl_3$) δ 6.61 (s, 1H), 6.57 (s, 2H), 3.79 (d, J = 6.9 Hz, 2H), 2.30 (s, 6H), 1.34 – 1.19 (m, 1H), 0.69 – 0.62 (m, 2H), 0.41 – 0.31 (m, 2H); ^{13}C NMR (100 MHz, $CDCl_3$) δ 159.2, 139.3, 122.5, 112.5, 72.7, 21.6, 10.5, 3.3; HRMS (ESI) m/z calc. for $C_{12}H_{17}O$ $[M+H]^+$: 177.1274, found: 177.1270.

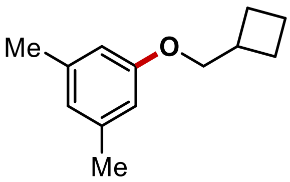

1-(Cyclobutylmethoxy)-3,5-dimethylbenzene (107): Yellow solid; ^{1}H NMR (400 MHz, $CDCl_3$) δ 6.60 (s, 1H), 6.56 (s, 2H), 3.91 (d, *J* = 6.7 Hz, 2H), 2.81 – 2.71 (m, 1H), 2.30 (s, 6H), 2.19 – 2.10 (m, 2H), 2.00 – 1.84 (m, 4H); ^{13}C NMR (100 MHz, $CDCl_3$) δ 159.5, 139.2, 122.4, 112.5, 72.1, 34.9, 25.0, 21.6, 18.8; HRMS (ESI) m/z calc. for $C_{13}H_{19}O$ $[M+H]^+$: 191.1430, found: 191.1435.

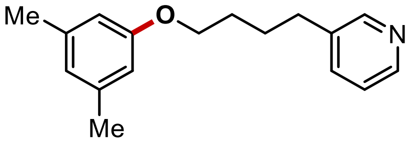

3-(4-(3,5-Dimethylphenoxy)butyl)pyridine (108): Yellow oil; ^{1}H NMR (400 MHz, $CDCl_3$) δ 8.53 – 8.38 (m, 2H), 7.52 (d, *J* = 7.5 Hz, 1H), 7.23 – 7.18 (m, 1H), 6.59 (s, 1H), 6.52 (s, 2H), 3.95 (s, 2H), 2.69 (s, 2H), 2.28 (s, 6H), 1.81 (t, *J* = 4.4 Hz, 4H); ^{13}C NMR (100 MHz, $CDCl_3$) δ 159.2, 150.1, 147.5, 139.3, 137.6, 136.0, 123.4, 122.5, 112.4, 67.4, 32.8, 28.9, 278, 21.5; HRMS (ESI) m/z calc. for $C_{17}H_{22}NO$ $[M+H]^+$: 256.1696, found: 256.1702.

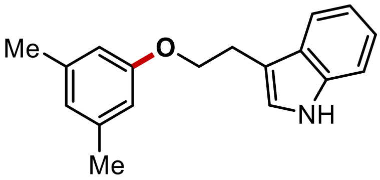

3-(2-(3,5-Dimethylphenoxy)ethyl)-1H-indole (109): Yellow solid; ^{1}H NMR (400 MHz, $CDCl_3$) δ 8.08 (br, 1H), 7.67 (d, *J* = 7.8 Hz, 1H), 7.37 (d, *J* = 8.0 Hz, 1H), 7.25 – 7.08 (m, 3H), 6.59 (d, *J* = 11.2 Hz, 3H), 4.23 (t, J = 7.2 Hz, 2H), 3.25 (t, *J* = 7.2 Hz, 2H), 2.28 (s, 6H); ^{13}C NMR (100 MHz, $CDCl_3$) δ 159.10, 139.30, 136.30, 127.70, 122.6, 122.3, 122.2, 119.5, 119.0, 112.8, 112.5, 111.3, 68.12, 25.6, 21.6; HRMS (ESI) m/z calc. for $C_{18}H_{20}NO$ $[M+H]^+$: 266.1539, found: 266.1544.

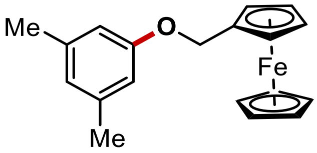

1-(Ferrocenemethoxy)-3,5-dimethylbenzene (110): White solid; ^{1}H NMR (400 MHz, $CDCl_3$) δ 6.61 (s, 1H), 6.59 (s, 2H), 4.76 (s, 2H), 4.34 – 4.29 (m, 2H), 4.19 (s, 7H), 2.30 (s, 6H); ^{13}C

NMR (100 MHz, $CDCl_3$) δ 159.2, 139.30, 122.7, 112.6, 69.2, 68.7, 68.7, 66.5, 21.6; HRMS (ESI) m/z calc. for $C_{19}H_{21}FeO$ $[M+H]^+$: 321.0936, found: 321.0944.

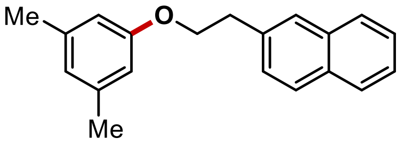

2-(2-(3,5-Dimethylphenoxy)ethyl)naphthalene (111): Yellow solid;^{1}H NMR (400 MHz, $CDCl_3$) δ 8.01 (d, J = 8.2 Hz, 1H), 7.78 (d, J = 7.9 Hz, 1H), 7.75 – 7.60 (m, 1H), 7.53 – 7.36 (m, 2H), 7.36 (t, J = 9.2 Hz, 2H), 6.50 (s, 1H), 6.46 (s, 2H), 4.19 (t, J = 7.4 Hz, 2H), 3.48 (t, J = 7.4 Hz, 2H), 2.18 (s, 6H); ^{13}C NMR (100 MHz, $CDCl_3$) δ 158.9, 139.2, 134.2, 133.9, 132.2, 128.9, 127.3, 127.0, 126.1, 125.6, 125.6, 123.6, 122.6, 112.4, 67.9, 33.0, 21.4; HRMS (ESI) m/z calc. for $C_{20}H_{21}O$ $[M+H]^+$: 277.1587, found: 277.1591.

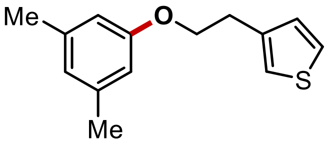

3-(2-(3,5-Dimethylphenoxy)ethyl)thiophene (112): Yellow oil; ^{1}H NMR (400 MHz, $CDCl_3$) δ 7.21 – 7.16 (m, 1H), 7.01 (d, J = 1.8 Hz, 1H), 6.96 (d, J = 4.9 Hz, 1H), 6.52 (s, 1H), 6.47 (s, 2H), 4.07 (t, J = 6.9 Hz, 2H), 3.03 (t, J = 6.9 Hz, 2H), 2.20 (s, 6H); ^{13}C NMR (100 MHz, $CDCl_3$) δ 159.0, 139.3, 138.8, 128.6, 125.5, 122.7, 121.6, 112.5, 68.0, 30.5, 21.6; HRMS (ESI) m/z calc. for $C_{14}H_{17}OS$ $[M+H]^+$: 233.0995, found: 233.0999.

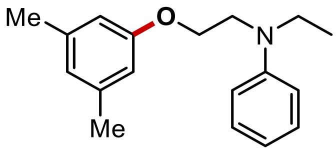

N-(2-(3,5-Dimethylphenoxy)ethyl)-N-ethylaniline (113): White solid; ^{1}H NMR (400 MHz, $CDCl_3$) δ 7.18 – 7.11 (m, 2H), 6.70 – 6.56 (m, 3H), 6.51 (s, 1H), 6.44 (s, 2H), 4.00 (t, J = 6.3 Hz, 2H), 3.62 (t, J = 6.3 Hz, 2H), 3.39 (q, J = 7.0 Hz, 2H), 2.19 (s, 6H), 1.12 (t, J = 7.0 Hz, 3H); ^{13}C NMR (100 MHz, $CDCl_3$) δ 158.9, 147.7, 139.4, 129.5, 122.8, 116.1, 112.4, 112.0, 65.3, 49.9, 45.8, 21.6, 12.5, 1.2; HRMS (ESI) m/z calc. for $C_{18}H_{24}NO$ $[M+H]^+$: 270.1852, found: 270.1859.

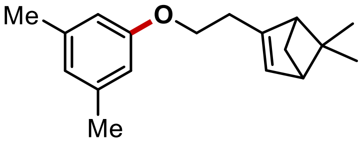

2-(2-(3,5-Dimethylphenoxy)ethyl)-5,5-dimethylbicyclo[2.1.1]hex-2-ene (114): White solid; ^{1}H NMR (400 MHz, $CDCl_3$) δ 6.59 (s, 1H), 6.53 (s, 2H), 5.36 (d, *J* = 1.2 Hz, 1H), 3.98 – 3.92 (m, 2H), 2.50 – 2.34 (m, 4H), 2.29 (d, *J* = 2.2 Hz, 6H), 2.10 (s, 2H), 1.30 (d, *J* = 2.4 Hz, 3H), 1.22 – 1.88 (m, 1H), 0.86 (d, *J* = 2.4 Hz, 3H); ^{13}C NMR (100 MHz, $CDCl_3$) δ 159.2, 144.9, 139.2, 122.5, 118.5, 112.5, 66.3, 46.1, 41.0, 38.2, 36.8, 31.8, 31.5, 26.5, 21.6; HRMS (ESI) m/z calc. for $C_{18}H_{25}O$ $[M+H]^+$: 257.1900, found: 257.1906.

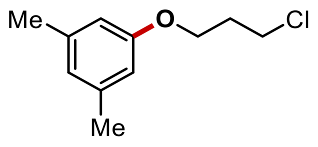

1-(3-Chloropropoxy)-3,5-dimethylbenzene (115): Yellow solid; ^{1}H NMR (400 MHz, $CDCl_3$) δ 6.62 (s, 1H), 6.55 (s, 2H), 4.09 (t, *J* = 5.8 Hz, 2H), 3.74 (t, *J* = 6.4 Hz, 2H), 2.30 (s, 6H), 2.26 – 2.18 (m, 2H); ^{13}C NMR (100 MHz, $CDCl_3$) δ 158.9, 139.4, 122.8, 112.4, 64.2, 41.8, 32.5, 21.6; HRMS (ESI) m/z calc. for $C_{11}H_{16}ClO$ $[M+H]^+$: 199.0884, found: 199.0890.

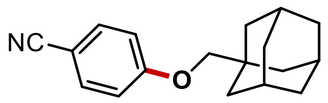

4-(((3r,5r,7r)-Adamantan-1-yl)methoxy)benzonitrile (116): Yellow solid; ^{1}H NMR (400 MHz, $CDCl_3$) δ 7.63 (d, *J* = 8.5 Hz, 2H), 6.93 (d, *J* = 8.8 Hz, 2H), 3.53 (s, 2H), 2.02 (s, 3H), 1.80 – 1.59 (m, 12H); ^{13}C NMR (100 MHz, $CDCl_3$) δ 163.1, 134.0, 118.2, 115.4, 103.6, 78.7, 39.5, 37.2, 33.9, 28.2; HRMS (ESI) m/z calc. for $C_{18}H_{22}NO$ $[M+H]^+$: 268.1696, found: 268.1699.

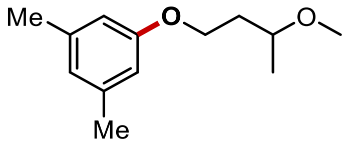

1-(3-Methoxybutoxy)-3,5-dimethylbenzene (117): Yellow solid; ^{1}H NMR (400 MHz, $CDCl_3$) δ 6.59 (s, 1H), 6.55 (s, 2H), 4.11 – 3.94 (m, 2H), 3.61 – 3.52 (m, 1H), 3.34 (s, 3H), 2.29 (s, 6H), 2.01 – 1.81 (m, 2H), 1.21 (d, *J* = 6.2 Hz, 3H); ^{13}C NMR (100 MHz, $CDCl_3$) δ 159.2, 139.3, 122.5, 112.4, 73.9, 64.5, 56.3, 36.5, 21.6, 19.3; HRMS (ESI) m/z calc. for $C_{13}H_{21}O_2$ $[M+H]^+$: 209.1536, found: 209.1541.

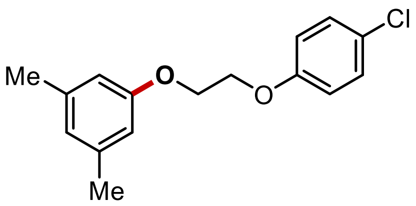

1-(2-(4-Chlorophenoxy)ethoxy)-3,5-dimethylbenzene (118): Yellow oil; ^{1}H NMR (400 MHz, $CDCl_3$) δ 7.16 (d, *J* = 9.2 Hz, 2H), 6.80 (d, *J* = 8.8 Hz, 2H), 6.55 (s, 1H), 6.50 (s, 2H), 4.20 (s, 4H), 2.21 (s, 6H); ^{13}C NMR (100 MHz, $CDCl_3$) δ 158.7, 157.5, 139.4, 129.5, 126.1, 123.1, 116.2, 112.6, 67.1, 66.4, 21.6; HRMS (ESI) m/z calc. for $C_{16}H_{18}ClO_2$ $[M+H]^+$: 277.0990, found: 277.0995.

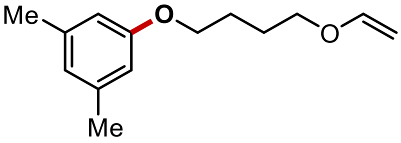

1,3-Dimethyl-5-(4-(vinyloxy)butoxy)benzene (119): Yellow oil; ^{1}H NMR (400 MHz, $CDCl_3$) δ 6.59 (s, 1H), 6.53 (s, 2H), 6.53 – 6.44 (m, 1H), 4.19 (d, *J* = 14.3 Hz, 1H), 4.00 – 3.94 (m, 3H), 3.75 (t, *J* = 5.7 Hz, 2H), 2.28 (s, 6H), 1.86 (s, 4H); ^{13}C NMR (100 MHz, $CDCl_3$) δ 159.2, 152.0, 139.3, 122.5, 112.4, 86.6, 67.7, 67.4, 26.1, 26.0, 21.6; HRMS (ESI) m/z calc. for $C_{14}H_{21}O_2$ $[M+H]^+$: 221.1536, found: 221.1538.

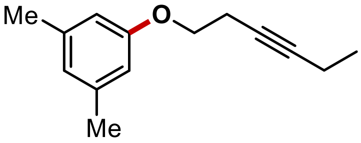

1-(Hex-3-yn-1-yloxy)-3,5-dimethylbenzene (120): Yellow oil; ^{1}H NMR (400 MHz, $CDCl_3$) δ 6.61 (s, 1H), 6.56 (s, 2H), 4.03 (t, *J* = 7.3 Hz, 2H), 2.66 – 2.59 (m, 2H), 2.29 (s, 6H), 2.23 – 2.14 (m, 2H), 1.14 (t, *J* = 7.5 Hz, 3H); ^{13}C NMR (100 MHz, $CDCl_3$) δ 158.8, 139.3, 122.8, 112.6, 83.5, 75.4, 66.7, 21.6, 20.0, 14.3, 12.6; HRMS (ESI) m/z calc. for $C_{14}H_{19}O$ $[M+H]^+$: 203.1430, found: 203.1436.

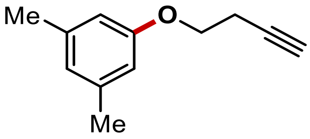

1-(But-3-yn-1-yloxy)-3,5-dimethylbenzene (121): Yellow oil; ^{1}H NMR (400 MHz, $CDCl_3$) δ 7.05 (s, 2H), 6.93 (s, 1H), 3.80 (t, *J* = 6.2 Hz, 2H), 2.68 (t, *J* = 6.3 Hz, 2H), 2.28 (s, 6H); ^{13}C NMR (100 MHz, $CDCl_3$) δ 138.0, 130.0, 129.5, 123.0, 85.6, 83.0, 61.4, 24.0, 21.2; HRMS (ESI) m/z calc. for $C_{12}H_{15}O$ $[M+H]^+$: 175.1117, found: 175.1122.

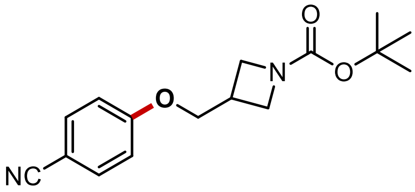

Tert-butyl 3-((4-cyanophenoxy)methyl)azetidine-1-carboxylate (122): Yellow solid; ^{1}H NMR (400 MHz, $CDCl_3$) δ 7.55 (d, *J* = 8.8 Hz, 2H), 6.92 (d, *J* = 8.9 Hz, 2H), 4.12 – 4.03 (m, 4H), 3.78 – 3.72 (m, 2H), 3.04 – 2.88 (m, 1H), 1.41 (s, 9H); ^{13}C NMR (100 MHz, $CDCl_3$) δ 161.9, 156.3, 134.1, 119.1, 115.2, 104.4, 79.6, 69.6, 28.4, 28.1; HRMS (ESI) m/z calc. for $C_{16}H_{21}N_2O_3$ $[M+H]^+$: 289.1547, found: 289.1550.

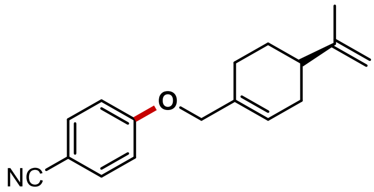

(S)-4-((4-(Prop-1-en-2-yl)cyclohex-1-en-1-yl)methoxy)benzonitrile (123): Yellow solid; ^{1}H NMR (400 MHz, $CDCl_3$) δ 7.55 (d, *J* = 8.5 Hz, 2H), 6.95 (d, *J* = 8.4 Hz, 2H), 5.84 (s, 1H), 4.72 (d, *J* = 8.4 Hz, 2H), 4.43 (s, 2H), 2.18 (d, *J* = 12.5 Hz, 4H), 1.99 (t, *J* = 15.0 Hz, 1H), 1.87 (d, *J* = 12.6 Hz, 1H), 1.73 (s, 3H), 1.60 – 1.45 (m, 1H); ^{13}C NMR (100 MHz, $CDCl_3$) δ 162.3, 149.4, 134.0, 132.6, 126.2, 119.4, 115.5, 109.0, 103.9, 72.6, 40.9, 30.5, 27.3, 26.2, 20.8; HRMS (ESI) m/z calc. for $C_{17}H_{23}NO$ $[M+H]^+$: 254.1539, found: 254.1543.

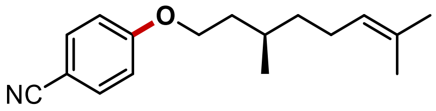

(R)-4-((3,7-Dimethyloct-6-en-1-yl)oxy)benzonitrile (124): White solid; ^{1}H NMR (400 MHz, $CDCl_3$) δ 7.56 (d, *J* = 8.8 Hz, 2H), 6.93 (d, *J* = 8.7 Hz, 2H), 5.09 (t, *J* = 7.0 Hz, 1H), 4.09 – 3.96 (m, 2H), 2.10 – 1.91 (m, 2H), 1.89 – 1.79 (m, 1H), 1.71 – 1.64 (s, 4H), 1.64 – 1.54 (s, 4H), 1.43 – 1.33 (m, 1H), 1.29 – 1.15 (m, 1H), 0.95 (d, *J* = 6.5 Hz, 3H); ^{13}C NMR (100 MHz, $CDCl_3$) δ 162.5, 134.0, 131.5, 124.6, 119.4, 115.3, 103.7, 66.8, 37.1, 35.9, 29.5, 25.8, 25.5, 19.6, 17.8; HRMS (ESI) m/z calc. for $C_{17}H_{24}NO$ $[M+H]^+$: 258.1582, found: 258.1588.

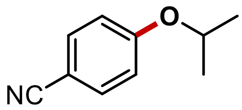

4-Isopropoxybenzonitrile (125): Yellow oil; ^{1}H NMR (400 MHz, $CDCl_3$) δ 7.54 (d, *J* = 8.6 Hz, 2H), 6.90 (d, *J* = 8.5 Hz, 2H), 4.68 – 4.50 (m, 1H), 1.34 (d, *J* = 6.1 Hz, 6H); ^{13}C NMR (100 MHz, $CDCl_3$) δ 161.5, 134.1, 119.4, 116.2, 103.4, 70.5, 21.9; HRMS (ESI) m/z calc. for $C_{10}H_{12}NO$ $[M+H]^+$: 162.0913, found: 162.0919.

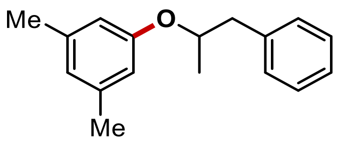

1,3-Dimethyl-5-((1-phenylpropan-2-yl)oxy)benzene (126): ^{1}H NMR (400 MHz, $CDCl_3$) δ 7.30 – 7.08 (m, 5H), 6.50 (s, 1H), 6.44 (s, 2H), 4.00 – 3.95 (m, 1H), 3.84 (t, J = 8.5 Hz, 1H), 3.21 – 3.06 (m, 1H), 2.19 (s, 6H), 1.33 (d, J = 7.0 Hz, 3H); ^{13}C NMR (100 MHz, $CDCl_3$) δ 159.2, 144.0, 139.3, 128.6, 127.6, 126.7, 122.6, 112.5, 73.4, 39.8, 21.6, 18.3; HRMS (ESI) m/z calc. for $C_{17}H_{21}O$ $[M+H]^+$: 241.1587, found: 241.1594.

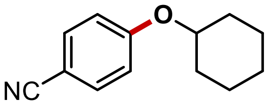

4-(Cyclohexyloxy)benzonitrile (127): Yellow solid; ^{1}H NMR (400 MHz, $CDCl_3$) δ 7.54 (d, J = 8.0 Hz, 2H), 6.91 (d, J = 8.0 Hz, 2H), 4.34 – 4.28 (m, 1H), 1.96 (d, J = 11.1 Hz, 2H), 1.79 (s, 2H), 1.58 – 1.49 (m, 3H), 1.45 – 1.28 (m, 3H); ^{13}C NMR (100 MHz, $CDCl_3$) δ 161.4, 134.0, 119.5, 116.3, 103.4, 75.8, 31.6, 25.5, 23.6; HRMS (ESI) m/z calc. for $C_{13}H_{16}NO$ $[M+H]^+$: 202.1226, found: 202.1232.

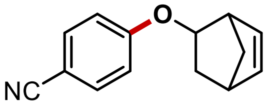

4-(Bicyclo[2.2.1]hept-5-en-2-yloxy)benzonitrile (128): Yellow solid; ^{1}H NMR (400 MHz, $CDCl_3$) δ 7.54 (d, J = 8.6 Hz, 2H), 6.91 (d, J = 8.7 Hz, 2H), 6.38 – 6.34 (m, 1H), 6.05 – 6.01 (m, 1H), 4.98 – 4.88 (m, 1H), 3.26 (s, 1H), 2.93 (s, 1H), 2.23 – 2.15 (m, 1H), 1.62 – 1.51 (m, 1H), 1.39 (d, J = 9.0 Hz, 1H), 1.13 – 1.00 (m, 1H); ^{13}C NMR (100 MHz, $CDCl_3$) δ 162.1, 138.6, 134.0, 131.8, 116.2, 103.7, 78.9, 47.6, 46.2, 42.6, 35.2, 29.8; HRMS (ESI) m/z calc. for $C_{14}H_{14}NO$ $[M+H]^+$: 212.1070, found: 212.1077.

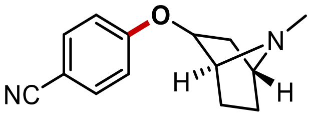

4-(((1S,4R)-7-Methyl-7-azabicyclo[2.2.1]heptan-2-yl)oxy)benzonitrile (129): Yellow solid; ^{1}H NMR (400 MHz, $CDCl_3$) δ 7.51 (d, J = 8.5 Hz, 2H), 6.88 (d, J = 8.5 Hz, 2H), 4.65 – 4.47 (m, 1H), 3.28 (s, 2H), 2.36 (s, 3H), 2.15 – 2.05 (m, 2H), 1.88 (t, J = 11.3 Hz, 2H), 1.65 (q, J = 6.1 Hz, 2H); ^{13}C NMR (100 MHz, $CDCl_3$) δ 161.1, 133.9, 119.4, 116.2, 103.6, 70.3, 60.2, 38.3, 35.4, 26.8; HRMS (ESI) m/z calc. for $C_{14}H_{17}N_2O$ $[M+H]^+$: 229.1335, found: 229.1338.

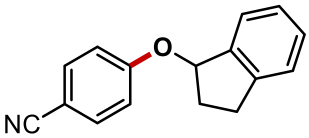

4-((2,3-Dihydro-1H-inden-1-yl)oxy)benzonitrile (130): Yellow solid; ^{1}H NMR (400 MHz, $CDCl_3$) δ 7.54 (d, *J* = 8.9 Hz, 2H), 7.34 (d, *J* = 7.5 Hz, 1H), 7.26 (d, *J* = 6.0 Hz, 2H), 7.23 – 7.16 (m, 1H), 6.98 (d, *J* = 8.8 Hz, 2H), 5.77 – 5.72 (m, 1H), 3.16 – 3.04 (m, 1H), 2.95 – 2.84 (m, 1H), 2.57 – 2.46 (m, 1H), 2.21 – 2.06 (m, 1H); ^{13}C NMR (100 MHz, $CDCl_3$) δ 161.9, 144.2, 140.8, 134.2, 129.5, 127.0, 125.4, 125.2, 119.4, 116.4, 104.1, 82.0, 32.1, 30.3; HRMS (ESI) m/z calc. for $C_{16}H_{14}NO$ $[M+H]^+$: 236.1070, found: 236.1077.

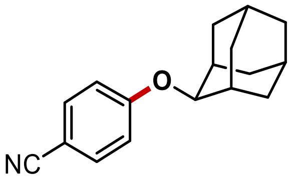

4-(((1R,3S,5r,7r)-Adamantan-2-yl)oxy)benzonitrile (131): Yellow solid; H NMR (400 MHz, $CDCl_3$) δ 7.55 (d, *J* = 8.9 Hz, 2H), 6.94 (d, *J* = 8.9 Hz, 2H), 4.48 (s, 1H), 2.12 (d, *J* = 15.6 Hz, 4H), 1.90 (t, *J* = 15.2 Hz, 4H), 1.78 (d, *J* = 11.5 Hz, 4H), 1.59 – 1.48 (m, 2H); ^{13}C NMR (100 MHz, $CDCl_3$) δ 161.4, 134.1, 119.5, 116.5, 103.4, 80.2, 37.4, 36.4, 31.5, 27.3, 27.2; HRMS (ESI) m/z calc. for $C_{17}H_{20}NO$ $[M+H]^+$: 254.1539, found: 254.1544.

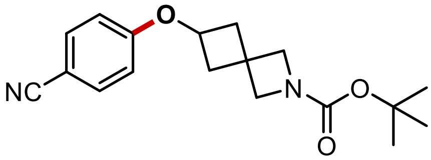

Tert-butyl 6-(4-cyanophenoxy)-2-azaspiro[3.3]heptane-2-carboxylate (132): Yellow solid; ^{1}H NMR (400 MHz, $CDCl_3$) δ 7.54 (d, *J* = 8.8 Hz, 2H), 6.80 (d, *J* = 8.8 Hz, 2H), 4.63 – 4.54 (m, 1H), 3.96 (s, 2H), 3.92 (s, 2H), 2.76 – 2.68 (m, 2H), 2.38 – 2.29 (m, 2H), 1.41 (s, 9H); ^{13}C NMR (100 MHz, $CDCl_3$) δ 160.6, 156.1, 134.1, 119.2, 115.7, 104.2, 79.7, 67.4, 41.0, 31.0, 28.4; HRMS (ESI) m/z calc. for $C_{16}H_{23}N_2O_3$ $[M+H]^+$: 315.1703, found: 315.1706.

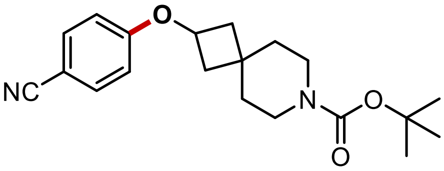

Tert-butyl 2-(4-cyanophenoxy)-7-azaspiro[3.5]nonane-7-carboxylate (133): Yellow solid; ^{1}H NMR (400 MHz, $CDCl_3$) δ 7.51 (d, *J* = 8.7 Hz, 2H), 6.79 (d, *J* = 8.7 Hz, 2H), 4.72 – 4.63 (m, 1H), 3.41 – 3.24 (m, 4H), 2.44 – 2.35 (m, 2H), 1.97 – 1.87 (m, 2H), 1.62 – 1.50 (m, 4H), 1.41 (s, 9H); ^{13}C NMR (100 MHz, $CDCl_3$) δ 160.80, 154.8, 134.0, 119.3, 115.7, 103.9, 79.5, 68.4, 41.0, 39.6, 32.0, 28.5; HRMS (ESI) m/z calc. for $C_{20}H_{27}N_2O_3$ $[M+H]^+$: 343.2016, found: 343.2019.

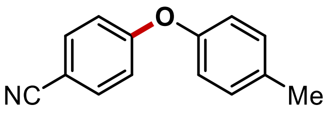

4-(P-tolyloxy)benzonitrile (134): White solid; ^{1}H NMR (400 MHz, $CDCl_3$) δ 7.66 – 7.55 (m, 3H), 7.20 (d, *J* = 8.1 Hz, 2H), 6.96 (t, *J* = 8.7 Hz, 3H), 2.37 (s, 3H); ^{13}C NMR (100 MHz, $CDCl_3$) δ 162.2, 152.5, 134.2, 133.5, 132.8, 130.8, 120.5, 117.7, 105.6, 20.9; HRMS (ESI) m/z calc. for $C_{14}H_{12}NO$ $[M+H]^+$: 210.0913, found: 210.0920.

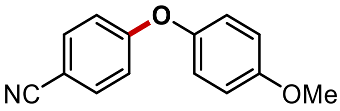

4-(P-tolyloxy)benzonitrile (135): White solid; ^{1}H NMR (400 MHz, $CDCl_3$) δ 7.57 (d, *J* = 8.4 Hz, 2H), 7.07 – 6.88 (m, 6H), 3.83 (s, 3H); ^{13}C NMR (100 MHz, $CDCl_3$) δ 162.7, 157.2, 148.1, 134.2, 122.0, 119.1, 117.3, 115.4, 105.5, 55.8; HRMS (ESI) m/z calc. for $C_{14}H_{12}NO_2$ $[M+H]^+$: 226.0863, found: 226.0869.

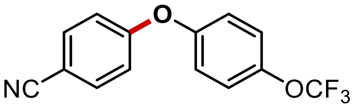

4-(4-(Trifluoromethoxy)phenoxy)benzonitrile (136): White solid; ^{1}H NMR (400 MHz, CDCl3) δ 7.56 (d, *J* = 8.9 Hz, 2H), 7.19 (d, *J* = 1.9 Hz, 2H), 7.01 (d, *J* = 9.0 Hz, 2H), 6.95 (d, *J* = 8.9 Hz, 2H); ^{13}C NMR (100 MHz, $CDCl_3$) δ 161.1, 153.3, 134.3, 123.0, 121.4, 120.0 (q, *J* =277.2 Hz, 2H), 118.6, 118.2, 106.6, 100.0; ^{19}F NMR (376 MHz, $CDCl_3$) δ -59.12 (s, CF_3); HRMS (ESI) m/z calc. for $C_{14}H_9F_3NO_2$ $[M+H]^+$: 280.0580, found: 280.0585.

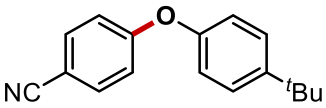

4-(4-(Tert-butyl)phenoxy)benzonitrile (137): White solid; ^{1}H NMR (400 MHz, $CDCl_3$) δ 7.58 (d, *J* = 8.8 Hz, 2H), 7.41 (d, *J* = 8.7 Hz, 2H), 7.04 – 6.92 (m, 4H), 1.34 (s, 9H); ^{13}C NMR (100 MHz, $CDCl_3$) δ 162.1, 148.3, 134.2, 132.3, 129.3, 127.2, 120.1, 117.8, 105.7, 34.6, 31.6; HRMS (ESI) m/z calc. for $C_{14}H_{12}NO_2$ $[M+H]^+$: 226.0863, found: 226.0869.

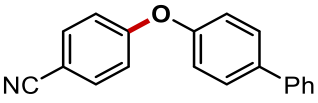

4-([1,1'-Biphenyl]-4-yloxy)benzonitrile (138): White solid; ^{1}H NMR (400 MHz, $CDCl_3$) δ 7.64 – 7.56 (m, 6H), 7.46 (t, *J* = 7.6 Hz, 2H), 7.37 (t, *J* = 7.3 Hz, 1H), 7.14 (d, *J* = 8.6 Hz, 2H), 7.06

(d, J = 8.8 Hz, 2H); ^{13}C NMR (100 MHz, CDCl3) δ 161.7, 154.4, 140.2, 138.4, 134.3, 129.0, 127.6, 127.1, 120.8, 119.0, 118.2, 106.1; HRMS (ESI) m/z calc. for $C_{19}H_{14}NO$ $[M+H]^+$: 272.1070, found: 272.1077.

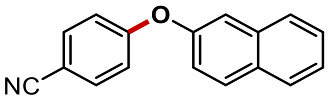

4-(Naphthalen-2-yloxy)benzonitrile (139): Yellow solid; ^{1}H NMR (400 MHz, $CDCl_3$) δ 7.84 – 7.66 (m, 2H), 7.69 (d, J = 7.7 Hz, 1H), 7.53 (d, J = 7.0 Hz, 2H), 7.48 – 7.36 (m, 3H), 7.15 (d, J = 8.9 Hz, 1H), 6.98 (d, J = 7.1 Hz, 2H); ^{13}C NMR (100 MHz, $CDCl_3$) δ 161.8, 152.6, 134.3, 133.5, 131.2, 130.6, 128.0, 127.5, 127.1, 125.8, 120.4, 118.9, 118.3, 116.9, 106.2; HRMS (ESI) m/z calc. for $C_{17}H_{12}NO$ $[M+H]^+$: 246.0913, found: 246.0917.

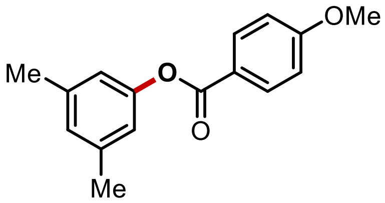

3,5-Dimethylphenyl 4-methoxybenzoate (140): Yellow solid; ^{1}H NMR (400 MHz, $CDCl_3$) δ 8.14 (d, J = 8.8 Hz, 2H), 6.98 (d, J = 8.8 Hz, 2H), 6.89 (s, 1H), 6.82 (s, 2H), 3.90 (s, 3H), 2.34 (s, 6H); ^{13}C NMR (100 MHz, $CDCl_3$) δ 165.3, 164.0, 151.1, 139.4, 132.4, 127.6, 122.3, 119.5, 114.0, 55.7, 21.4; HRMS (ESI) m/z calc. for $C_{15}H_{17}O_3$ $[M+H]^+$: 257.1172, found: 257.1179.

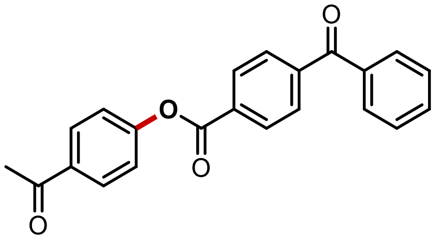

4-Acetylphenyl 4-benzoylbenzoate (141): White solid; ^{1}H NMR (400 MHz, $CDCl_3$) δ 8.32 (d, J = 8.3 Hz, 2H), 8.07 (d, J = 8.6 Hz, 2H), 7.92 (d, J = 8.3 Hz, 2H), 7.83 (d, J = 7.2 Hz, 2H), 7.64 (t, J = 7.4 Hz, 1H), 7.52 (t, J = 7.6 Hz, 2H), 7.36 (d, J = 8.6 Hz, 2H), 2.64 (s, 3H); ^{13}C NMR (100 MHz, $CDCl_3$) δ 197.0, 196.0, 164.00, 154.6, 142.4, 136.9, 135.2, 133.3, 132.2, 130.3, 130.3, 130.2, 130.1, 128.7, 122.0, 26.8; HRMS (ESI) m/z calc. for $C_{22}H_{17}O_4$ $[M+H]^+$: 345.1121, found: 345.1128.

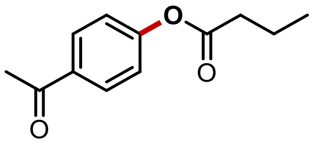

4-Acetylphenyl butyrate (142): Yellow solid; ^{1}H NMR (400 MHz, $CDCl_3$) δ 7.99 (d, *J* = 8.6 Hz, 2H), 7.19 (d, *J* = 8.6 Hz, 2H), 2.60 (s, 3H), 2.57 (t, *J* = 7.4 Hz, 2H), 1.87 – 1.74 (m, 2H), 1.05 (t, *J* = 7.4 Hz, 3H); ^{13}C NMR (100 MHz, $CDCl_3$) δ 197.0, 171.7, 154.6, 134.8, 130.1, 121.9, 36.4, 26.6, 18.5, 13.8; HRMS (ESI) m/z calc. for $C_{12}H_{15}O_3$ $[M+H]^+$: 207.1016, found: 207.1022.

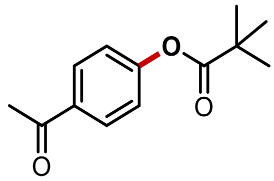

4-Acetylphenyl butyrate (143): Yellow solid; ^{1}H NMR (400 MHz, $CDCl_3$) δ 7.99 (d, *J* = 8.7 Hz, 2H), 7.16 (d, *J* = 8.7 Hz, 2H), 2.60 (s, 3H), 1.37 (s, 9H); ^{13}C NMR (100 MHz, $CDCl_3$) δ 197.1, 176.7, 155.1, 134.7, 130.1, 121.9, 39.4, 27.2, 26.8; HRMS (ESI) m/z calc. for $C_{13}H_{17}O_3$ $[M+H]^+$: 221.1272, found: 221.1278.

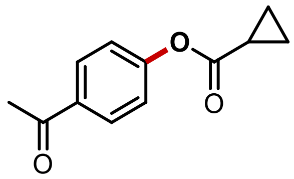

4-Acetylphenyl cyclopropanecarboxylate (144): Yellow oil; ^{1}H NMR (400 MHz, $CDCl_3$) δ 7.97 (d, *J* = 8.6 Hz, 2H), 7.19 (d, *J* = 8.6 Hz, 2H), 2.58 (s, 3H), 1.90 – 1.80 (m, 1H), 1.21 – 1.14 (m, 2H), 1.07 – 1.00 (m, 2H); ^{13}C NMR (100 MHz, $CDCl_3$) δ 197.0, 173.0, 154.7, 134.7, 123.0, 121.9, 26.7, 13.1, 9.6; HRMS (ESI) m/z calc. for $C_{12}H_{13}O_3$ $[M+H]^+$: 205.0859, found: 205.0864.

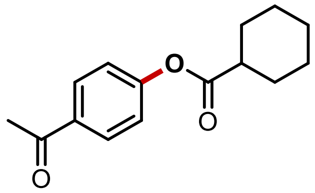

4-Acetylphenyl cyclohexanecarboxylate (145): Yellow oil; ^{1}H NMR (400 MHz, $CDCl_3$) δ 7.97 (d, *J* = 8.6 Hz, 2H), 7.16 (d, *J* = 8.6 Hz, 2H), 2.58 (s, 3H), 2.57 – 2.52 (m, 1H), 2.08 – 2.02 (m, 2H), 1.86 – 1.76 (m, 2H), 1.73 – 1.65 (m, 1H), 1.64 – 1.52 (m, 2H), 1.42 – 1.24 (m, 3H); ^{13}C NMR (100 MHz, $CDCl_3$) δ 197.0, 174.0, 154.8, 134.7, 123.0, 121.9, 43.3, 29.0, 26.7, 25.8, 25.4; HRMS (ESI) m/z calc. for $C_{15}H_{19}O_3$ $[M+H]^+$: 247.1329, found: 247.1334.

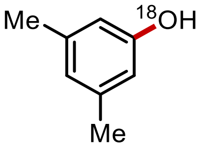

3,5-Dimethylphenol-^{18}O (146): Yellow oil; ^{1}H NMR (400 MHz, $CDCl_3$) δ 6.58 (s, 1H), 6.46 (s, 2H), 2.26 (s, 6H); ^{13}C NMR (100 MHz, $CDCl_3$) δ 155.6, 139.7, 122.7, 113.2, 21.4; HRMS (ESI) m/z calc. for $C_8H_{11}{}^{18}O$ $[M+H]^+$: 125.0847, found: 125.0855.

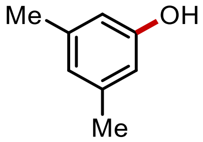

3,5-Dimethylphenol (147): White solid; ^{1}H NMR (400 MHz, $CDCl_3$) δ 6.57 (s, 1H), 6.46 (s, 2H), 4.64 (br, 1H), 2.26 (s, 6H); ^{13}C NMR (100 MHz, $CDCl_3$) δ 155.60, 139.7, 122.7, 113.2, 21.4; HRMS (ESI) m/z calc. for $C_8H_{11}O$ $[M+H]^+$: 123.0804, found: 123.0810.

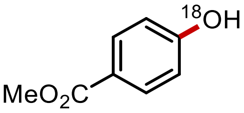

Methyl 4-(hydroxy)benzoate (148): White solid; ^{1}H NMR (400 MHz, $CDCl_3$) δ 7.95 (d, J = 8.8 Hz, 2H), 6.87 (d, J = 8.8 Hz, 2H), 3.89 (s, 3H); ^{13}C NMR (100 MHz, $CDCl_3$) δ 167.3, 160.1, 132.1, 122.7, 115.4, 52.2; HRMS (ESI) m/z calc. for $C_8H_9O_2{}^{18}O$ $[M+H]^+$: 155.0589, found: 155.0594.

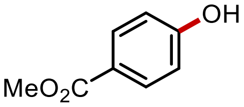

Methyl 4-hydroxybenzoate (149): White solid; ^{1}H NMR (400 MHz, CDC13) δ 7.95 (d, J = 8.5 Hz, 2H), 6.88 (d, J = 8.5 Hz, 2H), 6.61 (br, 1H), 3.89 (s, 3H); ^{13}C NMR (100 MHz, $CDCl_3$) δ 167.6, 160.5, 132.1, 122.4, 115.4, 52.2; HRMS (ESI) m/z calc. for $C_8H_9O_3$ $[M+H]^+$: 153.0546, found: 153.0550.

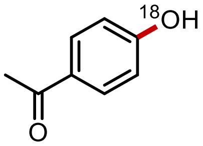

1-(4-(Hydroxy)phenyl)ethan-1-one (150): White solid; ^{1}H NMR (400 MHz, $CDCl_3$) δ 7.90 (d, J = 8.6 Hz, 2H), 6.94 (d, J = 8.7 Hz, 2H), 2.58 (s, 3H); ^{13}C NMR (100 MHz, $CDCl_3$) δ 198.6, 161.5, 131.3, 129.7, 115.7, 26.4; HRMS (ESI) m/z calc. for $C_8H_9O^{18}O$ $[M+H]^+$: 139.0640, found: 139.0644.

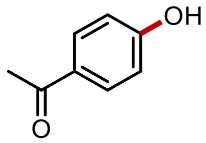

1-(4-Hydroxyphenyl)ethan-1-one (151): White solid; ^{1}H NMR (400 MHz, $CDCl_3$) δ 7.91 (d, J = 8.7 Hz, 2H), 6.94 (d, J = 8.8 Hz, 2H), 2.58 (s, 3H); ^{13}C NMR (100 MHz, $CDCl_3$) δ 198.7, 161.5, 131.4, 129.7, 115.7, 26.4; HRMS (ESI) m/z calc. for $C_8H_9O_2$ $[M+H]^+$: 137.0597, found: 137.0592.

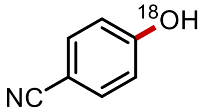

4-(Hydroxy)benzonitrile (152): White solid; ^{1}H NMR (400 MHz, $CDCl_3$) δ 7.52 (d, J = 7.4 Hz, 2H), 6.93 (s, 2H); ^{13}C NMR (100 MHz, $CDCl_3$) δ 160.6, 134.4, 119.5, 116.6, 103.0; HRMS (ESI) m/z calc. for $C_7H_6N^{18}O$ $[M+H]^+$: 122.0486, found: 122.0489.

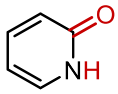

Pyridin-3-ol (153): Yellow solid; ^{1}H NMR (400 MHz, $CDCl_3$) δ 8.31 – 8.24 (m, 1H), 7.78 – 7.71 (m, 1H), 7.14 – 7.03 (m, 2H); ^{13}C NMR (100 MHz, $CDCl_3$) δ 162.1, 148.2, 139.7, 120.1, 114.1; HRMS (ESI) m/z calc. for C_5H_6NO $[M+H]^+$: 96.0444, found: 96.0450.

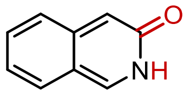

Isoquinolin-3-ol (154): White solid; ^{1}H NMR (400 MHz, $CDCl_3$) δ 9.04 (br, 1H), 7.95 (d, J = 8.3 Hz, 1H), 7.78 (d, J = 8.4 Hz, 1H), 7.67 – 7.61 (m, 1H), 7.52 – 7.46 (m, 1H), 7.41 (s, 1H); ^{13}C NMR (100 MHz, $CDCl_3$) δ 159.1, 151.8, 139.3, 130.9, 127.8, 126.9, 126.3, 126.0, 107.2; HRMS (ESI) m/z calc. for C_9H_8NO $[M+H]^+$: 146.0600, found: 146.0608.

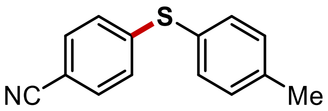

4-(P-tolylthio)benzonitrile (155): White solid; ^{1}H NMR (400 MHz, $CDCl_3$) δ 7.34 – 7.27 (m, 4H), 7.13 (d, J = 8.4 Hz, 2H), 7.01 (d, J = 8.5 Hz, 2H), 2.29 (s, 3H); ^{13}C NMR (100 MHz, $CDCl_3$) δ 146.5, 139.9, 134.9, 132.3, 130.8, 129.8, 128.5, 126.7, 108.3, 21.3; HRMS (ESI) m/z calc. for $C_{14}H_{12}NS$ $[M+H]^+$: 226.0685, found: 226.0693.

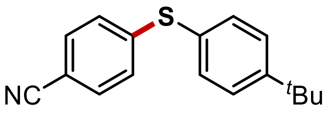

4-((4-(Tert-butyl)phenyl)thio)benzonitrile (156): Yellow solid; ^{1}H NMR (400 MHz, $CDCl_3$) δ 7.36 (d, J = 7.2 Hz, 6H), 7.05 (d, J = 8.1 Hz, 2H), 1.25 (s, 9H); ^{13}C NMR (100 MHz, $CDCl_3$) δ 153.0, 146.4, 134.6, 132.4, 127.1, 127.0, 127.0, 118.9, 108.5, 34.9, 31.3; HRMS (ESI) m/z calc. for $C_{17}H_{18}NS$ $[M+H]^+$: 268.1154, found: 268.1159.

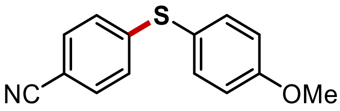

4-((4-Methoxyphenyl)thio)benzonitrile (157): Yellow solid; ^{1}H NMR (400 MHz, $CDCl_3$) δ 7.45 (t, J = 9.4 Hz, 4H), 7.07 (d, J = 8.3 Hz, 2H), 6.97 (d, J = 8.4 Hz, 2H), 3.85 (s, 3H); ^{13}C NMR (100 MHz, $CDCl_3$) δ 161.1, 147.5, 137.2, 132.3, 126.2, 120.5, 119.0, 115.7, 108.1, 55.6; HRMS (ESI) m/z calc. for $C_{14}H_{12}NOS$ $[M+H]^+$: 242.0634, found: 242.0639.

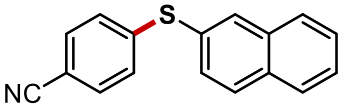

4-(Naphthalen-2-ylthio)benzonitrile (158): White solid; ^{1}H NMR (400 MHz, $CDCl_3$) δ 8.07 (s, 1H), 7.92 – 7.79 (m, 3H), 7.61 – 7.53 (m, 2H), 7.51 – 7.44 (m, 3H), 7.20 (d, J = 8.4 Hz, 2H); ^{13}C NMR (100 MHz, $CDCl_3$) δ 145.6, 134.3, 133.9, 133.3, 132.4, 130.7, 129.8, 128.1, 128.0, 127.9, 127.5, 127.4, 127.1, 118.8, 108.9; HRMS (ESI) m/z calc. for $C_{17}H_{12}NS$ $[M+H]^+$: 262.0685, found: 262.0691.

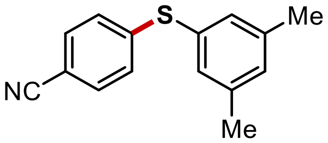

4-((3,5-Dimethylphenyl)thio)benzonitrile (159): Yellow solid; ^{1}H NMR (400 MHz, $CDCl_3$) δ 7.35 (d, J = 8.5 Hz, 2H), 7.04 (t, J = 8.8 Hz, 4H), 6.95 (s, 1H), 2.22 (s, 6H); ^{13}C NMR (100 MHz, $CDCl_3$) δ 146.3, 139.8, 132.3, 132.2, 131.3, 128.7, 127.2, 118.9, 108.5, 21.2; HRMS (ESI) m/z calc. for $C_{15}H_{14}NS$ $[M+H]^+$: 240.0841, found: 240.0847.

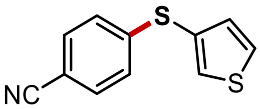

4-(Thiophen-3-ylthio)benzonitrile (160): Yellow solid; ^{1}H NMR (400 MHz, $CDCl_3$) δ 7.59 (d, J = 5.4 Hz, 1H), 7.47 (d, J = 8.4 Hz, 2H), 7.34 (d, J = 3.5 Hz, 1H), 7.17 – 7.10 (m, 3H); ^{13}C

NMR (100 MHz, $CDCl_3$) δ 146.5, 137.9, 133.0, 132.4, 128.6, 127.3, 125.8, 118.8, 108.8; HRMS (ESI) m/z calc. for $C_{11}H_8NS_2$ $[M+H]^+$: 218.0093, found: 218.0097.

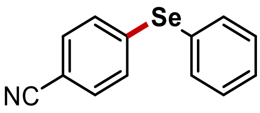

4-(Phenylselanyl)benzonitrile (161): Yellow solid; ^1H NMR (400 MHz, $CDCl_3$) δ 7.63 – 7.58 (m, 2H), 7.47 – 7.31 (m, 7H); ^{13}C NMR (100 MHz, $CDCl_3$) δ 141.0, 135.7, 132.5, 130.3, 130.0, 129.2, 127.6, 118.8, 109.7; HRMS (ESI) m/z calc. for $C_{13}H_{10}NSe$ $[M+H]^+$: 259.9973, found: 259.9978.

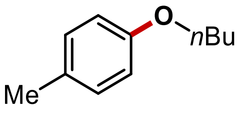

1-Butoxy-4-methylbenzene (162): Yellow oil; ^1H NMR (400 MHz, $CDCl_3$) δ 7.08 (d, J = 8.4 Hz, 2H), 6.81 (d, J = 8.5 Hz, 2H), 3.94 (t, J = 6.5 Hz, 2H), 2.29 (s, 3H), 1.81 – 1.72 (m, 2H), 1.55 – 1.43 (m, 2H), 0.98 (t, J = 7.4 Hz, 3H); ^{13}C NMR (100 MHz, $CDCl_3$) δ 157.1, 130.0, 129.8, 114.5, 67.9, 31.6, 20.6, 19.4, 14.0; HRMS (ESI) m/z calc. for $C_{11}H_{17}O$ $[M+H]^+$: 165.1274, found: 165.1277.

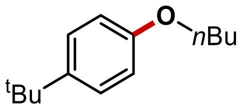

1-Butoxy-4-(tert-butyl)benzene (163): Yellow solid; ^1H NMR (400 MHz, $CDCl_3$) δ 7.31 (d, J = 8.6 Hz, 2H), 6.85 (d, J = 8.6 Hz, 2H), 3.96 (t, J = 6.5 Hz, 2H), 1.82 – 1.72 (m, 2H), 1.56 – 1.45 (m, 2H), 1.32 (s, 9H), 0.99 (t, J = 7.4 Hz, 3H); ^{13}C NMR (100 MHz, $CDCl_3$) δ 157.1, 143.3, 126.3, 114.1, 67.8, 34.2, 31.7,31.6, 19.4, 14.0; HRMS (ESI) m/z calc. for $C_{14}H_{23}O$ $[M+H]^+$: 207.1734, found: 207.1739.

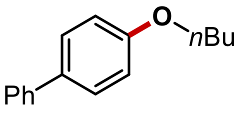

4-Butoxy-1,1'-biphenyl (164): White solid; ^1H NMR (400 MHz, $CDCl_3$) δ 7.60 – 5.50 (m, 4H), 7.42 (t, J = 7.6 Hz, 2H), 7.31 (t, J = 7.3 Hz, 1H), 6.98 (d, J = 8.7 Hz, 2H), 4.02 (t, J = 6.5 Hz, 2H), 1.86 – 1.76 (m, 2H), 1.60 – 1.47 (m, 2H), 1.01 (t, J = 7.4 Hz, 3H); ^{13}C NMR (100 MHz, $CDCl_3$) δ 158.9, 141.1, 133.7, 128.8, 128.2, 126. 9, 126.7, 114.9, 67.9, 31.5, 19.4, 14.0; HRMS (ESI) m/z calc. for $C_{16}H_{19}O$ $[M+H]^+$: 227.1430, found: 227.1435.

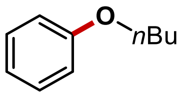

Butoxybenzene (165): White solid; ^{1}H NMR (400 MHz, $CDCl_3$) δ 7.19 (t, *J* = 7.9 Hz, 2H), 6.90-6.77 (m, 3H), 3.88 (t, *J* = 6.5 Hz, 2H), 1.78 – 1.64 (m, 2H), 1.50 – 1.38 (m, 2H), 0.90 (t, *J* = 7.4 Hz, 3H); ^{13}C NMR (100 MHz, CDCl3) δ 159.3, 129.5, 120.6, 114.7, 67.7, 31.5, 19.4, 13.4; HRMS (ESI) m/z calc. for $C_{10}H_{15}O$ $[M+H]^+$: 151.1117, found: 151.1112.

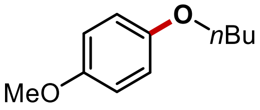

1-Butoxy-4-methoxybenzene (166): White solid; ^{1}H NMR (400 MHz, $CDCl_3$) δ 6.87 – 6.79 (m, 4H), 3.91 (t, *J* = 6.5 Hz, 2H), 3.77 (s, 3H), 1.79 – 1.70 (m, 2H), 1.54 – 1.42 (m, 2H), 0.97 (t, *J* = 7.4 Hz, 3H); ^{13}C NMR (100 MHz, $CDCl_3$) δ 153.8, 153.5, 115.6, 114.8, 68.5, 55.9, 31.6, 19.4, 14.0; HRMS (ESI) m/z calc. for $C_{11}H_{17}O_2$ $[M+H]^+$: 181.1223, found: 181.1225.

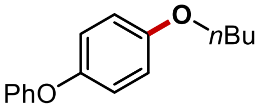

1-Butoxy-4-phenoxybenzene (167): White solid; ^{1}H NMR (400 MHz, $CDCl_3$) δ 7.33 – 7.27 (m, 2H), 7.05 (t, *J* = 7.4 Hz, 1H), 7.01 – 6.93 (m, 4H), 6.92 – 6.86 (m, 2H), 3.96 (t, *J* = 6.5 Hz, 2H), 1.80 – 1.74 (m, 2H), 1.57 – 1.46 (m, 2H), 1.00 (t, *J* = 7.4 Hz, 3H); ^{13}C NMR (100 MHz, $CDCl_3$) δ 158.7, 155.6, 150.1, 129.7, 122.5, 120.9, 117.7, 115.6, 68.3, 31.5, 19.4, 14.0; HRMS (ESI) m/z calc. for $C_{16}H_{19}O_2$ $[M+H]^+$: 243.1380, found: 243.1387.

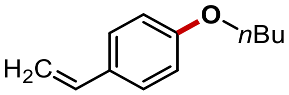

1-Butoxy-4-vinylbenzene (168): Yellow oil; ^{1}H NMR (400 MHz, $CDCl_3$) δ 7.33 (d, *J* = 8.2 Hz, 2H), 6.85 (d, *J* = 8.2 Hz, 2H), 6.70 – 6.60 (m, 1H), 5.60 (d, *J* = 17.6 Hz, 1H), 5.11 (d, *J* = 10.9 Hz, 1H), 3.97 (t, *J* = 6.5 Hz, 2H), 1.83 – 1.70 (m, 2H), 1.53 – 1.43 (m, 2H), 0.98 (t, *J* = 7.4 Hz, 3H); ^{13}C NMR (100 MHz, $CDCl_3$) δ 136.5, 127.5, 114.7, 111.5, 67.9, 31.5, 19.4, 14.0; HRMS (ESI) m/z calc. for $C_{12}H_{17}O$ $[M+H]^+$: 177.1274, found: 177.1279.

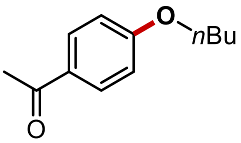

1-(4-Butoxyphenyl)ethan-1-one (169): White solid; ^{1}H NMR (400 MHz, $CDCl_3$) δ 7.92 (d, *J* = 8.9 Hz, 2H), 6.91 (d, *J* = 8.8 Hz, 2H), 4.02 (t, *J* = 6.5 Hz, 2H), 2.55 (s, 3H), 1.83 – 1.73 (m, 2H), 1.56 – 1.44 (m, 2H), 0.98 (t, *J* = 7.4 Hz, 3H); ^{13}C NMR (100 MHz, $CDCl_3$) δ 197.0, 163.3, 130.7, 130.2, 114.3, 68.1, 31.3, 26.5, 19.3, 13.9; HRMS (ESI) m/z calc. for $C_{12}H_{17}O_2$ $[M+H]^+$: 193.1223, found: 193.1226.

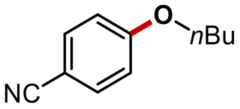

4-Butoxybenzonitrile (170): Yellow solid; ^{1}H NMR (400 MHz, $CDCl_3$) δ 7.56 (d, *J* = 8.6 Hz, 2H), 6.92 (d, *J* = 8.7 Hz, 2H), 4.00 (t, *J* = 6.5 Hz, 2H), 1.83 – 1.72 (m, 2H), 1.55 – 1.44 (m, 2H), 0.97 (t, *J* = 7.4 Hz, 3H); ^{13}C NMR (100 MHz, $CDCl_3$) δ 162.6, 134.1, 119.4, 115.3, 103.8, 68.2, 31.1, 19.3, 13.9; HRMS (ESI) m/z calc. for $C_{11}H_{14}NO$ $[M+H]^+$: 176.1070, found: 176.1075.

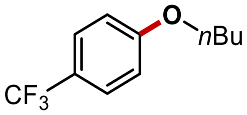

1-Butoxy-4-(trifluoromethyl)benzene (171): Yellow oil; ^{1}H NMR (400 MHz, $CDCl_3$) δ 7.53 (d, *J* = 8.6 Hz, 2H), 6.95 (d, *J* = 8.6 Hz, 2H), 4.00 (t, *J* = 6.5 Hz, 2H), 1.84 – 1.73 (m, 2H), 1.56 – 1.45 (m, 2H), 0.99 (t, *J* = 7.4 Hz, 3H); ^{13}C NMR (100 MHz, $CDCl_3$) δ 161.7, 126.8 (q, *J* = 3.8 Hz), 124.5 (q, *J* = 271.0 Hz), 122.6 (q, *J* = 32.6 Hz), 114.4, 67.9, 31.1, 19.2, 13.8; ^{19}F NMR (376 MHz, $CDCl_3$) δ -61.94 (s, CF_3); HRMS (ESI) m/z calc. for $C_{11}H_{14}F_3O$ $[M+H]^+$: 219.0991, found: 219.0998.

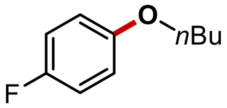

1-Butoxy-4-fluorobenzene (172): Yellow oil; ^{1}H NMR (400 MHz, $CDCl_3$) δ 6.92 – 6.81 (m, 2H), 6.79 – 6.68 (m, 2H), 3.87 – 3.79 (m, 2H), 1.72 – 1.60 (m, 2H), 1.47 – 1.32 (m, 2H), 0.92 – 0.84 (m, 3H); ^{13}C NMR (100 MHz, $CDCl_3$) δ 157.1 (d, *J* = 237.7 Hz), 155.3, 115.7 (d, *J* = 23.1 Hz), 115.4 (d, *J* = 7.9 Hz), 68.3, 31.4, 19.2, 13.8; ^{19}F NMR (376 MHz, $CDCl_3$) δ -124.49 (s, F); HRMS (ESI) m/z calc. for $C_{10}H_{14}FO$ $[M+H]^+$: 169.1023, found: 169.1027.

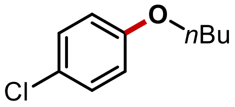

1-Butoxy-4-chlorobenzene (173): Yellow oil; ^{1}H NMR (400 MHz, $CDCl_3$) δ 7.22 (d, *J* = 9.0 Hz, 2H), 6.82 (d, *J* = 9.0 Hz, 2H), 3.93 (t, *J* = 6.5 Hz, 2H), 1.820 – 1.71 (m, 2H), 1.54 – 1.42

(m, 2H), 0.97 (t, J = 7.4 Hz, 3H); ^{13}C NMR (100 MHz, $CDCl_3$) δ 157.9, 129.4, 125.4, 115.9, 68.2, 31.4, 19.4, 14.0; HRMS (ESI) m/z calc. for $C_{10}H_{14}ClO$ $[M+H]^+$: 185.0728, found: 185.0732.

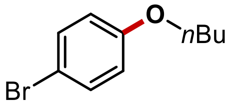

1-Bromo-4-butoxybenzene (174): Yellow solid; ^{1}H NMR (400 MHz, $CDCl_3$) δ 7.36 (d, J = 8.9 Hz, 2H), 6.77 (d, J = 8.9 Hz, 2H), 3.92 (t, J = 6.5 Hz, 2H), 1.80 – 1.71 (m, 2H), 1.53 – 1.42 (m, 2H), 0.97 (t, J = 7.4 Hz, 3H); ^{13}C NMR (100 MHz, $CDCl_3$) δ 158.4, 132.3, 116.5, 112.7, 68.1, 31.4, 19.4, 14.0; HRMS (ESI) m/z calc. for $C_{10}H_{14}BrO$ $[M+H]^+$: 229.0223, found: 229.0228.

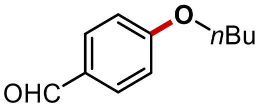

4-Butoxybenzaldehyde (175): Yellow oil; ^{1}H NMR (400 MHz, CDCl3) δ 8.05 (d, J = 8.4 Hz, 2H), 6.93 (d, J = 8.5 Hz, 2H), 4.03 (t, J = 6.3 Hz, 2H), 1.82 – 1.75 (m, 2H), 1.57 – 1.45 (m, 2H), 0.99 (t, J = 7.2 Hz, 3H); ^{13}C NMR (100 MHz, $CDCl_3$) δ 191.0, 171.7, 163.9, 132.5, 114.4, 68.1, 31.3, 19.3, 13.9; HRMS (ESI) m/z calc. for $C_{11}H_{15}O_2$ $[M+H]^+$: 179.1067, found: 179.1071.

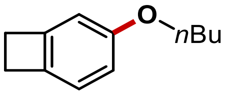

3-Butoxybicyclo[4.2.0]octa-1(6),2,4-triene (176): Yellow solid; ^{1}H NMR (400 MHz, $CDCl_3$) δ 6.93 (d, J = 8.0 Hz, 1H), 6.76 – 6.71 (m, 1H), 6.67 (s, 1H), 3.92 (t, J = 6.5 Hz, 2H), 3.10 (s, 4H), 1.80 – 1.70 (m, 2H), 1.54 – 1.43 (m, 2H), 0.97 (t, J = 7.4 Hz, 3H); ^{13}C NMR (100 MHz, $CDCl_3$) δ 159.1, 146.6, 137.3, 123.6, 114.1 109.6, 68.2, 31.6, 29.1, 28.9, 19.4, 14.0; HRMS (ESI) m/z calc. for $C_{12}H_{17}O$ $[M+H]^+$: 177.1274, found: 177.1278.

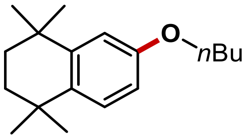

6-Butoxy-1,1,4,4-tetramethyl-1,2,3,4-tetrahydronaphthalene (177): Yellow solid; ^{1}H NMR (400 MHz, $CDCl_3$) δ 7.24 (d, J = 8.7 Hz, 1H), 6.86 (d, J = 2.7 Hz, 1H), 6.75 – 6.72 (m, 1H), 3.96 (t, J = 6.5 Hz, 2H), 1.82 – 1.73 (m, 2H), 1.71 – 1.66 (m, 4H), 1.57 – 1.46 (m, 2H), 1.29 (d, J = 8.3 Hz, 12H), 1.00 (t, J = 7.4 Hz, 3H); ^{13}C NMR (100 MHz, $CDCl_3$) δ 157.0, 146.4, 137.1, 127.5, 112.5, 111.9, 67.6, 35.4, 35.4, 34.6, 33.8, 32.2, 32.0, 31.7, 19.5, 14.0; HRMS (ESI) m/z calc. for $C_{18}H_{29}O$ $[M+H]^+$: 261.2213, found: 261.2217.

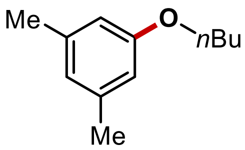

1-Butoxy-3,5-dimethylbenzene (178): White solid; ^{1}H NMR (400 MHz, $CDCl_3$) δ 6.59 (s, 1H), 6.54 (s, 2H), 3.94 (t, J = 6.5 Hz, 2H), 2.29 (s, 6H), 1.80 – 1.71 (m, 2H), 1.54 – 1.43 (m, 2H), 0.98 (t, J = 7.4 Hz, 3H); ^{13}C NMR (100 MHz, $CDCl_3$) δ 159.4, 139.3, 122.4, 112.5, 67.6, 31.6, 21.6, 19.4, 14.0; HRMS (ESI) m/z calc. for $C_{12}H_{19}O$ [M+H]$^+$: 179.1430, found: 179.1435.

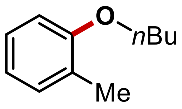

1-Butoxy-2-methylbenzene (179): White solid; ^{1}H NMR (400 MHz, $CDCl_3$) δ 7.14 (t, J = 7.3 Hz, 2H), 6.86 – 6.79 (m, 2H), 3.97 (t, J = 6.3 Hz, 2H), 2.23 (s, 3H), 1.84 – 1.73 (m, 2H), 1.54 – 1.46 (m, 2H), 0.98 (t, J = 7.4 Hz, 3H); ^{13}C NMR (100 MHz, $CDCl_3$) δ 157.4, 130.7, 127.0, 126.8, 120.2, 111.1, 67.8, 31.7, 19.5, 16.3, 14.0; HRMS (ESI) m/z calc. for $C_{11}H_{17}O$ [M+H]$^+$: 165.1274, found: 165.1277.

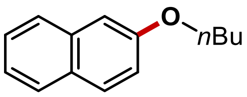

2-Butoxynaphthalene (180): White solid; ^{1}H NMR (400 MHz, $CDCl_3$) δ 7.81 – 7.71 (m, 3H), 7.47 – 7.41 (m, 1H), 7.37 – 7.31 (m, 1H), 7.20 – 7.14 (m, 2H), 4.10 (t, J = 6.5 Hz, 2H), 1.91 – 1.81 (m, 2H), 1.63 – 1.51 (m, 2H), 1.03 (t, J = 7.4 Hz, 3H); ^{13}C NMR (100 MHz, $CDCl_3$) δ 157.3, 134.8, 129.4, 129.0, 127.8, 126.8, 126.4, 123.6, 119.2, 106.7, 67.9, 31.5, 19.5, 14.0; HRMS (ESI) m/z calc. for $C_{14}H_{17}O$ [M+H]$^+$: 201.1274, found: 201.1279.

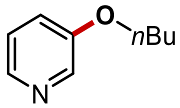

3-Butoxypyridine (181): White solid; ^{1}H NMR (400 MHz, $CDCl_3$) δ 8.04 (d, J = 2.8 Hz, 1H), 7.22 – 7.13 (m, 3H), 3.98 (t, J = 6.5 Hz, 2H), 1.83 – 1.71 (m, 2H), 1.55 – 1.42 (m, 2H), 0.97 (t, J = 7.4 Hz, 3H); ^{13}C NMR (100 MHz, $CDCl_3$) δ 154.7, 142.4, 136.8, 125.0, 124.5, 68.7, 31.2, 19.2, 13.9; HRMS (ESI) m/z calc. for $C_9H_{14}NO$ [M+H]$^+$: 152.1070, found: 152.1077.

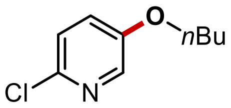

5-Butoxy-2-chloropyridine (182): White solid; ^{1}H NMR (400 MHz, $CDCl_3$) δ 8.03 (d, *J* = 2.8 Hz, 1H), 7.25 – 7.13 (m, 2H), 3.98 (t, *J* = 6.5 Hz, 2H), 1.81 – 1.72 (m, 2H), 1.54 – 1.42 (m, 2H), 0.97 (t, *J* = 7.4 Hz, 3H); ^{13}C NMR (100 MHz, $CDCl_3$) δ 154.7, 142.4, 136.8, 125.0, 124.5, 68.7, 31.2, 19.2, 13.9; HRMS (ESI) m/z calc. for $C_9H_{13}ClNO$ $[M+H]^+$: 186.0680, found: 186.0686.

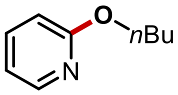

2-Butoxypyridine (183): Yellow oil; 1H NMR (400 MHz, $CDCl_3$) δ 8.15 – 8.12 (m, 1H), 7.58 – 7.49 (m, 1H), 6.89 – 6.79 (m, 1H), 6.71 (d, *J* = 8.4 Hz, 1H), 4.28 (t, *J* = 6.7 Hz, 2H), 1.80 – 1.70 (m, 2H), 1.53 – 1.42 (m, 2H), 0.97 (t, *J* = 7.4 Hz, 3H); ^{13}C NMR (100 MHz, $CDCl_3$) δ 164.3, 147.0, 138.6, 116.6, 111.2, 65.8, 31.3, 19.4, 14.0; HRMS (ESI) m/z calc. for $C_9H_{14}NO$ $[M+H]^+$: 152.1070, found: 152.1077.

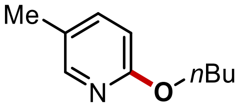

2-Butoxy-5-methylpyridine (184): Yellow oil; ^{1}H NMR (400 MHz, $CDCl_3$) δ 7.93 (d, *J* = 0.9 Hz, 1H), 7.37 – 7.32 (m, 1H), 6.62 (d, *J* = 8.4 Hz, 1H), 4.23 (t, *J* = 6.7 Hz, 2H), 2.21 (s, 3H), 1.77 – 1.68 (m, 2H), 1.54 – 1.37 (m, 2H), 0.95 (t, *J* = 7.4 Hz, 3H); ^{13}C NMR (100 MHz, $CDCl_3$) δ 162.5, 146.4, 139.6, 125.4, 110.5, 65.7, 31.4, 19.4, 17.5, 14.0; HRMS (ESI) m/z calc. for $C_{10}H_{16}NO$ $[M+H]^+$: 166.1226, found: 166.1270.

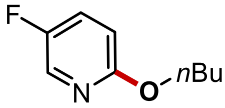

2-Butoxy-5-fluoropyridine (185): Yellow solid; ^{1}H NMR (400 MHz, $CDCl_3$) δ 7.96 (d, J = 3.0 Hz, 1H), 7.33 – 7.27 (m, 1H), 6.70 – 6.64 (m, 1H), 4.24 (t, J = 6.7 Hz, 2H), 1.77 – 1.68 (m, 2H), 1.51 – 1.40 (m, 2H), 0.96 (t, J = 7.4 Hz, 3H); ^{13}C NMR (100 MHz, $CDCl_3$) δ 160.5, δ 155.4 (d, J = 244.9 Hz), 133.2 (d, J = 25.8 Hz), 126.6 (d, J = 21.3 Hz), 111.7 (d, J = 4.6 Hz), 66.4, 31.3, 19.4, 14.0; ^{19}F NMR (376 MHz, $CDCl_3$) δ -118.28 (s, F); HRMS (ESI) m/z calc. for $C_9H_{13}FNO$ $[M+H]^+$: 170.0976, found: 170.0981.

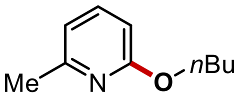

2-Butoxy-6-methylpyridine (186): Yellow solid; ^{1}H NMR (400 MHz, $CDCl_3$) δ 7.43 (t, *J* = 7.7 Hz, 1H), 6.68 (d, *J* = 7.2 Hz, 1H), 6.50 (d, *J* = 8.2 Hz, 1H), 4.25 (t, *J* = 6.6 Hz, 2H), 2.43 (s,

3H), 1.79 – 1.70 (m, 2H), 1.53 – 1.42 (m, 2H), 0.97 (t, *J* = 7.4 Hz, 3H); ^{13}C NMR (100 MHz, $CDCl_3$) δ 163.8, 156.5, 138.8, 115.6, 107.1, 65.7, 31.4, 24.3, 19.5, 14.0; HRMS (ESI) m/z calc. for $C_{10}H_{16}NO$ $[M+H]^+$: 166.1226, found: 166.1230.

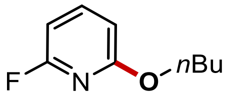

2-Butoxy-6-fluoropyridine (187): White solid; ^{1}H NMR (400 MHz, $CDCl_3$) δ 7.62 (q, *J* = 8.1 Hz, 1H), 6.59 – 6.55 (m, 1H), 6.45 – 6.40 (m, 1H), 4.29 – 4.23 (m, 2H), 1.78 – 1.69 (m, 2H), 1.54 – 1.40 (m, 2H), 0.97 (t, *J* = 7.4 Hz, 3H); ^{13}C NMR (100 MHz, $CDCl_3$) δ 163.4 (d, *J* = 10.9 Hz), 161.1, 142.4 (d, *J* = 8.0 Hz), 107.1 (d, *J* = 5.1 Hz), 99.6 (d, *J* = 35.7 Hz), 66.4, 31.0, 19.2, 13.8; ^{19}F NMR (376 MHz, $CDCl_3$) δ -68.59 (s, F); HRMS (ESI) m/z calc. for $C_9H_{13}FNO$ $[M+H]^+$: 170.0976, found: 170.0982.

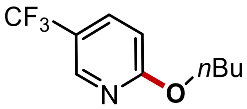

2-Butoxy-5-(trifluoromethyl)pyridine (188): Yellow solid; ^{1}H NMR (400 MHz, $CDCl_3$) δ 8.42 (s, 1H), 7.76 – 7.71 (m, 1H), 6.78 (d, *J* = 8.7 Hz, 1H), 4.35 (t, *J* = 6.7 Hz, 2H), 1.80 – 1.71 (m, 2H), 1.52 – 1.43 (m, 2H), 0.97 (t, *J* = 7.4 Hz, 3H); ^{13}C NMR (100 MHz, $CDCl_3$) δ 166.3, 145.1 (q, *J* = 4.4 Hz), 135.6 (q, *J* = 3.1 Hz), 124.3 (q, *J* = 8.1 Hz), 119.8 (q, *J* = 12.9 Hz), 111.4, 66.7, 31.1, 19.4, 13.9; ^{19}F NMR (376 MHz, $CDCl_3$) δ -63.94 (s, CF_3); HRMS (ESI) m/z calc. for $C_{10}H_{13}F_3NO$ $[M+H]^+$: 220.0944, found: 220.0950.

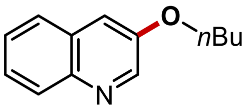

3-Butoxyquinoline (189): White solid; ^{1}H NMR (400 MHz, $CDCl_3$) δ 8.68 (s, 1H), 8.04 (d, *J* = 8.2 Hz, 1H), 7.71 (d, *J* = 7.5 Hz, 1H), 7.57 – 7.46 (m, 2H), 7.37 (d, *J* = 2.7 Hz, 1H), 4.09 (t, *J* = 6.5 Hz, 2H), 1.90 – 1.81 (m, 2H), 1.61 – 1.49 (m, 2H), 1.01 (t, *J* = 7.4 Hz, 3H); ^{13}C NMR (100 MHz, $CDCl_3$) δ 152.8, 145.0, 143.5, 129.3, 129.0, 127.2, 126.8, 126.7, 113.1, 68.2, 31.2, 19.4, 14.0; HRMS (ESI) m/z calc. for $C_{13}H_{16}NO$ $[M+H]^+$: 202.1226, found: 202.1233.

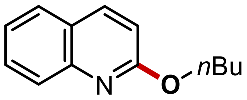

2-Butoxyquinoline (190): White solid; ^{1}H NMR (400 MHz, $CDCl_3$) δ 7.96 (d, *J* = 8.8 Hz, 1H), 7.85 (d, *J* = 8.4 Hz, 1H), 7.70 (d, *J* = 8.0 Hz, 1H), 7.65 – 7.58 (m, 1H), 7.41 – 7.33 (m, 1H), 6.90 (d, *J* = 8.8 Hz, 1H), 4.50 (t, *J* = 6.7 Hz, 2H), 1.88 – 1.79 (m, 2H), 1.60 – 1.49 (m, 2H),

1.02 (t, *J* = 7.4 Hz, 3H); ^{13}C NMR (100 MHz, CDCl$_3$) δ 162.5, 146.8, 138.7, 129.5, 127.5, 127.3, 125.2, 123.9, 113.4, 65.8, 31.3, 19.5, 14.1; HRMS (ESI) m/z calc. for $C_{13}H_{16}NO$ [M+H]$^+$: 202.1226, found: 202.1235.

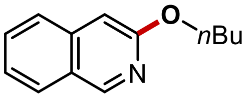

3-Butoxyisoquinoline (191): White solid; ^{1}H NMR (400 MHz, CDCl$_3$) δ 8.93 (s, 1H), 7.84 (d, *J* = 8.3 Hz, 1H), 7.65 (d, *J* = 8.4 Hz, 1H), 7.53 (t, *J* = 7.6 Hz, 1H), 7.33 (t, *J* = 7.5 Hz, 1H), 6.97 (s, 1H), 4.33 (t, *J* = 6.6 Hz, 2H), 1.89 – 1.77 (m, 2H), 1.60 – 1.47 (m, 2H), 0.99 (t, *J* = 7.4 Hz, 3H); ^{13}C NMR (100 MHz, CDCl$_3$) δ 161.4, 150.8, 139.4, 130.4, 127.7, 125.6, 125.3, 124.3, 101.3, 66.8, 31.5, 19.4, 14.0; HRMS (ESI) m/z calc. for $C_{13}H_{16}NO$ [M+H]$^+$: 202.1226, found: 202.1230.

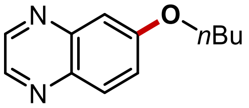

6-Butoxyquinoxaline (192): White solid; ^{1}H NMR (400 MHz, CDCl$_3$) δ 8.74 (d, *J* = 1.8 Hz, 1H), 8.67 (d, *J* = 1.9 Hz, 1H), 7.97 (d, *J* = 9.2 Hz, 1H), 7.43 – 7.38 (m, 1H), 7.35 (d, *J* = 2.7 Hz, 1H), 4.13 (t, *J* = 6.5 Hz, 2H), 1.90 – 1.81 (m, 2H), 1.60 – 1.48 (m, 2H), 1.00 (t, *J* = 7.4 Hz, 3H); ^{13}C NMR (100 MHz, CDCl$_3$) δ 160.5, 145.0, 144.9, 142.4, 139.3, 130.5, 124.0, 107.4, 68.5, 31.2, 19.4, 13.9; HRMS (ESI) m/z calc. for $C_{12}H_{15}N_2O$ [M+H]$^+$: 203.1179, found: 203.1183.

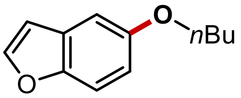

5-Butoxybenzofuran (193): Yellow solid; ^{1}H NMR (400 MHz, CDCl$_3$) δ 7.59 (d, *J* = 1.9 Hz, 1H), 7.39 (d, *J* = 8.8 Hz, 1H), 7.06 (d, *J* = 2.3 Hz, 1H), 6.93 – 6.88 (m, 1H), 6.70 (s, 1H), 4.00 (t, *J* = 6.5 Hz, 2H), 1.94 – 1.74 (m, 2H), 1.58 – 1.48 (m, 2H), 1.00 (t, *J* = 7.4 Hz, 3H); ^{13}C NMR (100 MHz, CDCl$_3$) δ 155.6, 150.1, 145.8, 128.1, 113.8, 111.9, 106.8, 104.6, 68.7, 31.6, 19.4, 14.0; HRMS (ESI) m/z calc. for $C_{12}H_{15}O_2$ [M+H]$^+$: 191.1067, found: 191.1072.

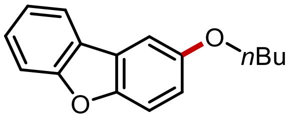

2-Butoxydibenzo[b,d]furan (194): Yellow solid; ^{1}H NMR (400 MHz, CDCl$_3$) δ 7.91 (d, *J* = 7.7 Hz, 1H), 7.55 (d, *J* = 8.2 Hz, 1H), 7.49 – 7.41 (m, 3H), 7.32 (t, *J* = 7.5 Hz, 1H), 7.07 -7.02 (m, 1H), 4.07 (t, *J* = 6.5 Hz, 2H), 1.89 – 1.78 (m, 2H), 1.62 – 1.49 (m, 2H), 1.02 (t, *J* = 7.4 Hz, 3H); ^{13}C NMR (100 MHz, CDCl3) δ 157.1, 155.6, 151.0, 127.2, 124.8, 124.7, 122.5, 120.7, 115.9,

112.2, 111.9, 104.8, 68.9, 31.6, 19.5, 14.0; HRMS (ESI) m/z calc. for $C_{16}H_{17}O_2$ $[M+H]^+$: 241.1223, found: 241.1229.

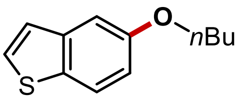

5-Butoxybenzo[b]thiophene (195): Yellow solid; ^{1}H NMR (400 MHz, $CDCl_3$) δ 7.63 (d, J = 8.8 Hz, 1H), 7.33 (d, J = 5.3 Hz, 1H), 7.21 – 7.12 (m, 2H), 6.91 (d, J = 8.7 Hz, 1H), 3.93 (t, J = 6.5 Hz, 2H), 1.77 – 1.66 (m, 2H), 1.49 – 1.38 (m, 2H), 0.91 (t, J = 7.4 Hz, 3H); ^{13}C NMR (100 MHz, $CDCl_3$) δ 157.1, 140.8, 132.1, 127.5, 123.7, 123.1, 115.3, 106.7, 68.2, 31.5, 19.4, 14.0; HRMS (ESI) m/z calc. for $C_{12}H_{15}OS$ $[M+H]^+$: 207.0838, found: 207.0840.

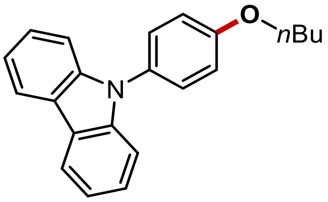

9-(4-Butoxyphenyl)-4,9-dihydro-3H-carbazole (196): Yellow solid; ^{1}H NMR (400 MHz, $CDCl_3$) δ 8.18 (d, J = 7.7 Hz, 2H), 7.49 – 7.40 (m, 4H), 7.37 (d, J = 8.1 Hz, 2H), 7.33 – 7.28 (m, 2H), 7.12 (d, J = 8.8 Hz, 2H), 4.09 (t, J = 6.5 Hz, 2H), 1.92 – 1.83 (m, 2H), 1.65 – 1.53 (m, 2H), 1.07 (t, J = 7.4 Hz, 3H); ^{13}C NMR (100 MHz, $CDCl_3$) δ 158.6, 141.5, 130.2, 128.7, 126.0, 123.2, 120.4, 119.7, 115.7, 109.9, 68.2, 31.5, 19.4, 14.0; HRMS (ESI) m/z calc. for $C_{22}H_{22}NO$ $[M+H]^+$: 316.1696, found: 316.1700.

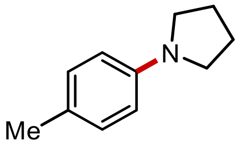

1-(p-Tolyl)pyrrolidine (197): yellow oil; ^{1}H NMR (400 MHz, $CDCl_3$) δ 7.03 (d, J = 8.2 Hz, 2H), 6.49 (d, J = 8.3 Hz, 2H), 3.24 (t, J = 6.4 Hz, 4H), 2.24 (s, 3H), 1.98 (t, J = 6.6 Hz, 4H); ^{13}C NMR (10 MHz, $CDCl_3$) δ 146.2, 129.6, 124.5, 111.8, 47.8, 25.4, 20.3; HRMS (ESI) m/z calc. for $C_{11}H_{16}N$ $[M+H]^+$: 162.1277, found: 162.1278.

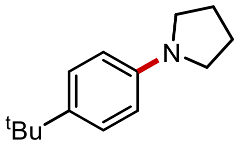

1-(4-(Tert-butyl)phenyl)pyrrolidine (198): yellow oil; ^{1}H NMR (400 MHz, $CDCl_3$) δ 7.26 (d, J = 8.6 Hz, 2H), 6.53 (d, J = 8.7 Hz, 2H), 3.27 (t, J = 6.5 Hz, 4H), 1.97 (t, J = 6.6 Hz, 4H), 1.29 (s, 9H); ^{13}C NMR (100 MHz, $CDCl_3$) δ 145.9, 138.0, 125.9, 111.3, 47.7, 33.7, 31.6, 25.5; HRMS (ESI) m/z calc. for $C_{14}H_{22}N$ $[M+H]^+$: 204.1747, found: 207.1750.

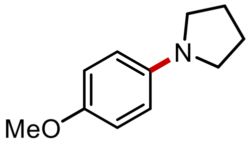

1-(4-Methoxyphenyl)pyrrolidine (199): yellow oil; ^{1}H NMR (400 MHz, $CDCl_3$) δ 6.84 (d, J = 8.5 Hz, 2H), 6.55 (d, J = 7.7 Hz, 2H), 3.75 (s, 3H), 3.23 (s, 4H), 1.99 (s, 4H); ^{13}C NMR (100 MHz, $CDCl_3$) δ 138.2, 116.4, 115.1, 112.8, 56.0, 48.4, 25.4; HRMS (ESI) m/z calc. for $C_{11}H_{16}NO$ $[M+H]^+$: 178.1226, found: 178.1228.

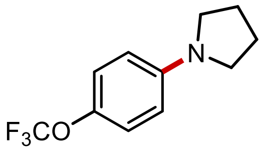

1-(4-(Trifluoromethoxy)phenyl)pyrrolidine (200): yellow oil; ^{1}H NMR (400 MHz, $CDCl_3$) δ 7.07 (d, J = 8.9 Hz, 2H), 6.48 (d, J = 9.0 Hz, 2H), 3.26 (t, J = 6.6 Hz, 4H), 2.01 (t, J = 6.6 Hz, 4H); ^{13}C NMR (100 MHz, $CDCl_3$) δ 146.7, 139.1, 122.3, 120.82 (q, J = 254.8 Hz), 111.6, 47.8, 25.5; ^{19}F NMR (376 MHz, $CDCl_3$) δ -58.52 (s, OCF_3); HRMS (ESI) m/z calc. for $C_{11}H_{13}F_3NO$ $[M+H]^+$: 232.0944, found: 232.0946.

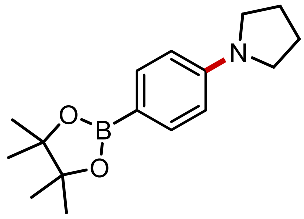

1-(4-(4,4,5,5-Tetramethyl-1,3,2-dioxaborolan-2-yl)phenyl)pyrrolidine (201): yellow oil; ^{1}H NMR (400 MHz, $CDCl_3$) δ 7.67 (d, J = 8.4 Hz, 2H), 6.53 (d, J = 8.5 Hz, 2H), 3.30 (t, J = 6.6 Hz, 4H), 1.99 (t, J = 6.6 Hz, 4H), 1.32 (s, 12H); ^{13}C NMR (100 MHz, $CDCl_3$) δ 150.0, 136.2, 110.9, 83.1, 47.4, 25.5, 24.9; HRMS (ESI) m/z calc. for $C_{16}H_{25}BNO_2$ $[M+H]^+$: 274.1973, found: 274.1975.

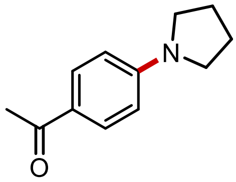

1-(4-(Pyrrolidin-1-yl)phenyl)ethan-1-one (202): yellow oil; ^{1}H NMR (400 MHz, $CDCl_3$) δ 7.86 (d, J = 8.7 Hz, 1H), 6.51 (d, J = 8.7 Hz, 1H), 3.36 (t, J = 6.5 Hz, 2H), 2.50 (s, 2H), 2.04 (t, J = 6.5 Hz, 2H); ^{13}C NMR (100 MHz, $CDCl_3$) δ 196.4, 151.0, 130.7, 124.9, 110.6, 47.5, 25.9, 25.4; HRMS (ESI) m/z calc. for $C_{12}H_{16}NO$ $[M+H]^+$: 190.1226, found: 190.1230.

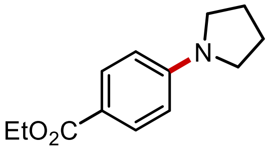

Methyl 4-(pyrrolidin-1-yl)benzoate (203): yellow oil; ^{1}H NMR (400 MHz, $CDCl_3$) δ 7.90 (d, J = 8.9 Hz, 1H), 6.49 (d, J = 8.9 Hz, 1H), 4.31 (q, J = 7.1 Hz, 1H), 3.33 (t, J = 6.6 Hz, 2H), 2.02

(t, J = 6.6 Hz, 2H), 1.36 (t, J = 7.1 Hz, 2H); ^{13}C NMR (100 MHz, $CDCl_3$) δ 167.2, 150.8, 131.3, 116.6, 110.6, 60.0, 47.5, 25.5, 14.5; HRMS (ESI) m/z calc. for $C_{13}H_{18}NO_2$ [M+H]$^+$: 220.1332, found: 220.1335.

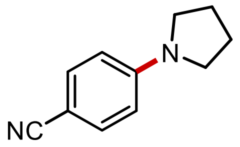

4-(Pyrrolidin-1-yl)benzonitrile (204): yellow oil; ^{1}H NMR (400 MHz, $CDCl_3$) δ 7.43 (d, J = 8.8 Hz, 2H), 6.49 (d, J = 8.8 Hz, 2H), 3.32 (t, J = 6.6 Hz, 4H), 2.04 (t, J = 6.6 Hz, 4H); ^{13}C NMR (100 MHz, $CDCl_3$) δ 150.0, 133.5, 121.1, 111.5, 96.5, 47.5, 25.4; HRMS (ESI) m/z calc. for $C_{11}H_{13}N_2$ [M+H]$^+$: 173.1073, found: 173.1076.

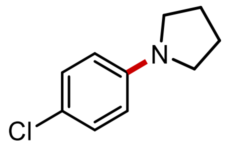

1-(4-Chlorophenyl)pyrrolidine (205): yellow oil; ^{1}H NMR (400 MHz, $CDCl_3$) δ 7.14 (d, J = 8.9 Hz, 2H), 6.45 (d, J = 8.9 Hz, 2H), 3.24 (t, J = 6.6 Hz, 4H), 2.00 (t, J = 6.6 Hz, 4H); ^{13}C NMR (100 MHz, $CDCl_3$) δ 146.5, 128.9, 120.1, 112.6, 47.7, 25.5; HRMS (ESI) m/z calc. for $C_{10}H_{13}ClN$ [M+H]$^+$: 182.0731, found: 182.0735.

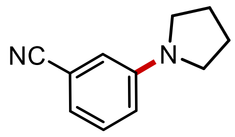

3-(Pyrrolidin-1-yl)benzonitrile (206): yellow oil; ^{1}H NMR (400 MHz, $CDCl_3$) δ 7.30 – 7.24 (m, 1H), 6.89 (d, J = 7.4 Hz, 1H), 6.77 – 6.69 (m, 2H), 3.27 (t, J = 6.6 Hz, 4H), 2.03 (t, J = 6.6 Hz, 4H); ^{13}C NMR (100 MHz, $CDCl_3$) δ 147.7, 129.7, 119.9, 118.5, 115.8, 114.3, 112.7, 47.5, 25.5; HRMS (ESI) m/z calc. for $C_{11}H_{13}N_2$ [M+H]$^+$: 173.1073, found: 173.1077.

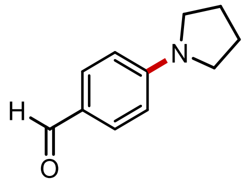

4-(Pyrrolidin-1-yl)benzaldehyde (207): yellow oil; ^{1}H NMR (400 MHz, $CDCl_3$) δ 9.72 (br, 1H), 7.73 (d, J = 8.7 Hz, 2H), 6.57 (d, J = 8.7 Hz, 2H), 3.39 (t, J = 6.6 Hz, 4H), 2.05 (t, J = 6.6 Hz, 4H); ^{13}C NMR (100 MHz, $CDCl_3$) δ 190.3, 152.0, 132.2, 124.9, 111.2, 47.7, 25.4; HRMS (ESI) m/z calc. for $C_{11}H_{14}NO$ [M+H]$^+$: 176.1070, found: 176.1073.

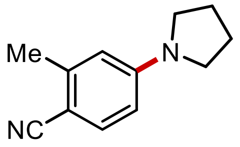

2-Methyl-4-(pyrrolidin-1-yl)benzonitrile (208): yellow oil; ^{1}H NMR (400 MHz, $CDCl_3$) δ 7.38 (d, J = 9.3 Hz, 1H), 6.36 – 6.32 (m, 2H), 3.31 (t, J = 6.6 Hz, 4H), 2.45 (d, J = 5.0 Hz, 4H), 2.02 (s, 3H); ^{13}C NMR (100 MHz, $CDCl_3$) δ 150.1, 143.0, 133.8, 120.4, 112.3, 109.3, 97.4, 47.5, 25.4, 20.9; HRMS (ESI) m/z calc. for $C_{12}H_{15}N_2$ [M+H]$^+$: 187.1230, found: 187.1234.

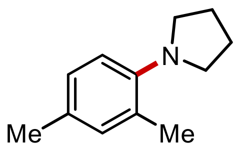

1-(2,4-Dimethylphenyl)pyrrolidine (209): yellow oil; ^{1}H NMR (400 MHz, $CDCl_3$) δ 6.95 (s, 1H), 6.92 (d, *J* = 8.2 Hz, 1H), 6.83 (d, *J* = 8.1 Hz, 1H), 3.13 (s, 4H), 2.29 (s, 3H), 2.25 (s, 3H), 1.92 (t, *J* = 6.1 Hz, 4H); ^{13}C NMR (100 MHz, $CDCl_3$) δ 147.1, 132.5, 129.5, 126.8, 116.3, 109.8, 51.4, 24.9, 20.6, 20.2; HRMS (ESI) m/z calc. for $C_{12}H_{18}N$ $[M+H]^+$: 176.1434, found: 176.1435.

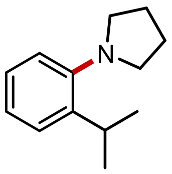

1-(2-Isopropylphenyl)pyrrolidine (210): yellow oil; ^{1}H NMR (400 MHz, $CDCl_3$) δ 7.25 (d, *J* = 5.3 Hz, 1H), 7.11 (t, *J* = 6.9 Hz, 1H), 7.07 – 6.95 (m, 2H), 3.41 (dt, *J* = 13.7, 6.8 Hz, 1H), 3.09 (s, 4H), 1.92 (s, 4H), 1.23 (d, *J* = 6.9 Hz, 6H); ^{13}C NMR (101 MHz, $CDCl_3$) δ 148.4, 142.6, 126.5, 126.0, 122.3, 118.1, 52.8, 27.4, 24.7, 24.3; HRMS (ESI) m/z calc. for $C_{13}H_{20}N$ $[M+H]^+$: 190.1590, found: 190.1593.

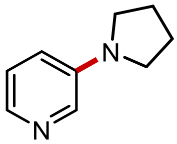

3-(Pyrrolidin-1-yl)pyridine (211): yellow oil; ^{1}H NMR (400 MHz, $CDCl_3$) δ 7.98 (d, *J* = 2.3 Hz, 1H), 7.91 (d, *J* = 4.3 Hz, 1H), 7.10 (dd, *J* = 8.4, 4.6 Hz, 1H), 6.80 (dd, *J* = 8.4, 1.9 Hz, 1H), 3.29 (t, *J* = 6.6 Hz, 4H), 2.02 (t, *J* = 6.6 Hz, 4H); ^{13}C NMR (101 MHz, $CDCl_3$) δ 143.78 (s), 136.76 (s), 134.23 (s), 123.55 (s), 117.78 (s), 47.27 (s), 25.4; HRMS (ESI) m/z calc. for $C_9H_{13}N_2$ $[M+H]^+$: 149.1073, found: 149.1076.

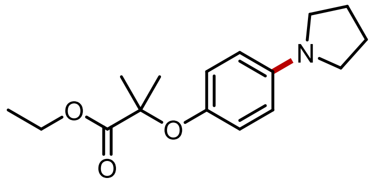

3-(Pyrrolidin-1-yl)pyridine (212): yellow oil; ^{1}H NMR (400 MHz, $CDCl_3$) δ 6.84 (d, *J* = 8.8 Hz, 2H), 6.44 (d, *J* = 8.5 Hz, 2H), 4.24 (q, *J* = 7.1 Hz, 2H), 3.23 (t, *J* = 6.1 Hz, 4H), 1.98 (t, *J* = 6.3 Hz, 4H), 1.51 (s, 6H), 1.30 (t, *J* = 7.1 Hz, 3H); ^{13}C NMR (100 MHz, $CDCl_3$) δ 174.8, 146.9, 144.9, 122.6, 113.4, 80.1, 61.5, 44.6, 32.1, 25.6, 20.6, 14.5, 14.2; HRMS (ESI) m/z calc. for $C_{16}H_{24}NO_3$ $[M+H]^+$: 278.1751, found: 278.1755.

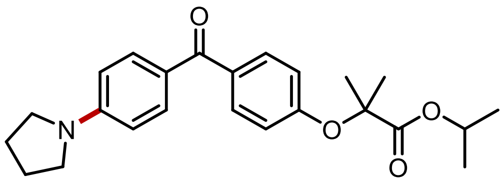

Isopropyl 2-methyl-2-(4-(4-(pyrrolidin-1-yl)benzoyl)phenoxy)propanoate (213): yellow oil; ^{1}H NMR (400 MHz, $CDCl_3$) δ 7.75 (d, *J* = 8.8 Hz, 2H), 7.68 (d, *J* = 8.8 Hz, 2H), 6.86 (d, *J* =

8.8 Hz, 2H), 6.52 (d, J = 8.9 Hz, 2H), 5.09 (dt, J = 12.6, 6.3 Hz, 1H), 3.34 (t, J = 6.5 Hz, 4H), 2.09 – 1.96 (m, 4H), 1.65 (s, 6H), 1.21 (d, J = 6.3 Hz, 6H); ^{13}C NMR (150 MHz, $CDCl_3$) δ 194.0, 173.4, 158.3, 150.7, 132.7, 131.3, 127.5, 124.5, 117.2, 110.6, 79., 69.2, 47.6, 25.4, 25.3, 21.5; HRMS (ESI) m/z calc. for $C_{24}H_{30}NO_4$ [M+H]$^+$: 396.2169, found: 396.2173.

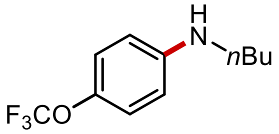

***N*-butyl-4-(trifluoromethoxy)aniline (214):** yellow oil; ^{1}H NMR (400 MHz, $CDCl_3$) δ ^{1}H NMR (400 MHz, $CDCl_3$) δ 7.02 (d, J = 8.2 Hz, 2H), 6.53 (d, J = 9.0 Hz, 2H), 3.65 (br, 1H), 3.08 (t, J = 7.1 Hz, 2H), 1.64 – 1.58 (m, 2H), 1.48 – 1.39 (m, 2H), 0.96 (t, J = 7.3 Hz, 3H); ^{13}C NMR (100 MHz, $CDCl_3$) δ 147.3, 140.2 (q, J = 1.9 Hz), 122.4, 120.8 (q, J = 255.1 Hz), 112.8, 43.8, 31.5, 20.3, 13.9; ^{19}F NMR (376 MHz, $CDCl_3$) δ -58.41 (s, OCF_3); HRMS (ESI) m/z calc. for $C_{11}H_{15}F_3NO$ [M+H]$^+$: 234.1100, found: 234.1103.

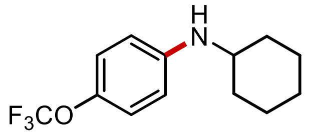

***N*-cyclohexyl-4-(trifluoromethoxy)aniline (215):** yellow oil; ^{1}H NMR (400 MHz, $CDCl_3$) δ 7.00 (d, J = 8.4 Hz, 2H), 6.52 (d, J = 9.0 Hz, 2H), 3.60 (br, 1H), 3.23 – 3.19 (m, 1H), 1.82 – 1.72 (m, 2H), 1.70 – 1.59 (m, 1H), 1.39 – 1.34 (m, 3H), 1.24 – 1.07 (m, 4H); ^{13}C NMR (100 MHz, $CDCl_3$) δ 146.2, 139.9 (q, J = 2.0 Hz), 122.4, 120.73 (q, J = 255.1 Hz), 113.2, 51.9, 33.3, 25.9, 25.0; ^{19}F NMR (376 MHz, $CDCl_3$) δ -58.28 (s, OCF_3); HRMS (ESI) m/z calc. for $C_{13}H_{17}F_3NO$ [M+H]$^+$: 260.1257, found: 260.1260.

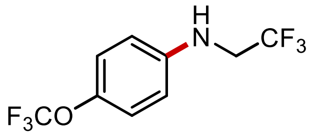

***N*-(2,2,2-Trifluoroethyl)-4-(trifluoromethoxy)aniline (216):** yellow oil; ^{1}H NMR (400 MHz, $CDCl_3$) δ 7.06 (d, J = 8.7 Hz, 2H), 6.64 (d, J = 8.9 Hz, 2H), 4.06 (br, 1H), 3.81 – 3.65 (m, 2H); ^{13}C NMR (100 MHz, $CDCl_3$) δ 145.1, 141.60 (q, J = 2.0 Hz), 124.9 (q, J = 279.9 Hz), 122.5, 120.7 (q, J = 255.6 Hz), 113.5, 46.07 (q, J = 33.8 Hz); ^{19}F NMR (376 MHz, $CDCl_3$) δ -58.60 (s, OCF_3), -72.48 (s, CF_3); HRMS (ESI) m/z calc. for $C_9H_8F_6NO$ [M+H]$^+$: 260.0505, found: 260.0507.

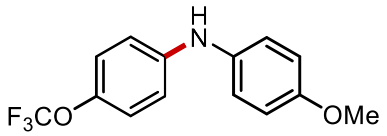

4-Methoxy-N-(4-(trifluoromethoxy)phenyl)aniline (217): yellow oil; ^{1}H NMR (400 MHz, $CDCl_3$) δ 7.12 – 7.01 (m, 4H), 6.93 – 6.78 (m, 4H), 5.55 (br, 1H), 3.80 (s, 3H); ^{13}C NMR (100 MHz, $CDCl_3$) δ 155.8, 144.3, 141.7, 135.1, 122.9, 122.4, 120.7 (q, J = 255.6 Hz), 115.8, 114.8, 55.6; ^{19}F NMR (376 MHz, $CDCl_3$) δ -58.42 (s, OCF_3); HRMS (ESI) m/z calc. for $C_{14}H_{13}F_3NO_2$ [M+H]$^+$: 284.0893, found: 284.0896.

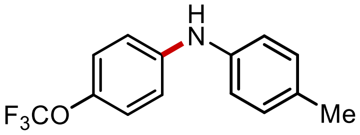

4-Methyl-N-(4-(trifluoromethoxy)phenyl)aniline (218): yellow oil; ^{1}H NMR (400 MHz, $CDCl_3$) δ 7.09 (d, *J* = 8.3 Hz, 2H), 7.07 (d, *J* = 9.1 Hz, 2H), 6.98 (d, *J* = 8.4 Hz, 2H), 6.95 (d, *J* = 9.0 Hz, 2H), 5.66 (br, 1H), 2.30 (s, 3H); ^{13}C NMR (100 MHz, $CDCl_3$) δ 143.1, 142.2 (q, *J* = 1.9 Hz), 139.8, 131.7, 130.0, 122.3, 120.67 (q, J = 255.6 Hz), 119.4, 117.1, 20.7; ^{19}F NMR (376 MHz, $CDCl_3$) δ -58.35 (s, OCF_3); HRMS (ESI) m/z calc. for $C_{14}H_{13}F_3NO$ $[M+H]^+$: 268.0944, found: 268.0946.

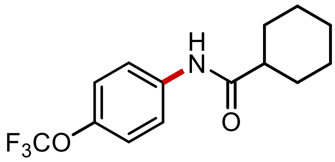

N-(4-(Trifluoromethoxy)phenyl)cyclohexanecarboxamide (219): yellow solid;^{1}H NMR (400 MHz, $CDCl_3$) δ 7.63 (br, 1H), 7.55 (d, *J* = 8.9 Hz, 2H), 7.14 (d, *J* = 8.6 Hz, 2H), 2.28 – 2.23 (m, 1H), 1.98 – 1.91 (m, 2H), 1.83 (s, 2H), 1.73 – 1.63 (m, 1H), 1.59 – 1.46 (m, 2H), 1.35 – 1.22 (m, 3H); ^{13}C NMR (100 MHz, $CDCl_3$) δ 175.2, 145.3, 142.5, 121.3, 120.5 (q, *J* = 256.8 Hz), 116.2, 46.5, 29.6, 25.6; ^{19}F NMR (376 MHz, $CDCl_3$) δ -58.16 (s, OCF_3); HRMS (ESI) m/z calc. for $C_{14}H_{17}F_3NO_2$ $[M+H]^+$: 288.1206, found: 288.1208.

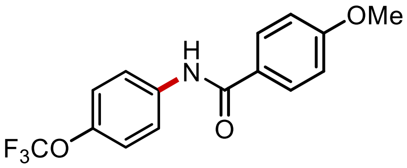

4-Methoxy-N-(4-(trifluoromethoxy)phenyl)benzamide (220): yellow solid; ^{1}H NMR (400 MHz, DMSO) δ 10.28 (br, 1H), 7.96 (d, *J* = 8.7 Hz, 2H), 7.88 (d, *J* = 9.0 Hz, 2H), 7.35 (d, *J* = 8.7 Hz, 2H), 7.07 (d, *J* = 8.7 Hz, 2H), 3.84 (s, 3H); ^{13}C NMR (100 MHz, DMSO) δ 165.6, 162.5, 144.2, 139.9, 130.1, 127.0, 122.1, 121.9, 120.6 (q, *J* = 258.0 Hz), 114.1, 55.9; ^{19}F NMR (376 MHz, $CDCl_3$) δ -57.87 (s, OCF_3); HRMS (ESI) m/z calc. for $C_{15}H_{13}F_3NO_3$ $[M+H]^+$: 312.0842, found: 312.0846.

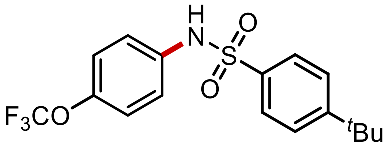

4-(Tert-butyl)-N-(4-(trifluoromethoxy)phenyl)benzenesulfonamide (221): yellow solid; ^{1}H NMR (400 MHz, $CDCl_3$) δ 7.81 – 7.72 (m, 3H, CH_2, NH), 7.46 (d, *J* = 8.6 Hz, 2H), 7.15 (d, *J* = 9.1 Hz, 2H), 7.07 (d, *J* = 8.6 Hz, 2H), 1.29 (s, 9H); ^{13}C NMR (100 MHz, $CDCl_3$) δ 157.2, 146.20 (q, *J* = 1.8 Hz), 135.7, 135.4, 127.1, 126.2, 122.4, 120.4 (q, *J* = 257.1 Hz), 122.0, 35.2, 31.0; ^{19}F NMR (376 MHz, $CDCl_3$) δ -58.30 (s, OCF_3); HRMS (ESI) m/z calc. for $C_{17}H_{19}F_3NO_3S$ $[M+H]^+$: 374.1032, found: 374.1035.

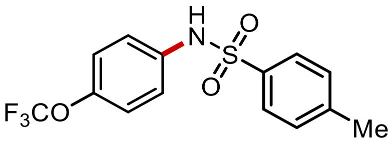

4-methyl-N-(4-(trifluoromethoxy)phenyl)benzenesulfonamide (222): yellow solid; ^{1}H NMR (400 MHz, $CDCl_3$) δ 7.96 (br, 1H), 7.72 (d, *J* = 8.2 Hz, 2H), 7.22 (d, *J* = 8.1 Hz, 2H), 7.15 (d, *J* = 9.0 Hz, 2H), 7.05 (d, *J* = 8.7 Hz, 2H), 2.35 (s, 3H); ^{13}C NMR (100 MHz, $CDCl_3$) δ 146.2 (q, *J* = 1.8 Hz), 144.3, 135.7, 135.5, 129.8, 127.3, 122.5, 121.9, 120.38 (q, *J* = 257.1 Hz), 21.5; ^{19}F NMR (376 MHz, $CDCl_3$) δ -58.29 (s, OCF_3); HRMS (ESI) m/z calc. for $C_{14}H_{13}F_3NO_3S$ $[M+H]^+$: 332.0563, found: 332.0566.

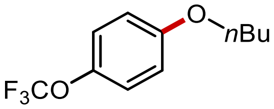

1-Butoxy-4-(trifluoromethoxy)benzene (223): yellow oil; ^{1}H NMR (400 MHz, $CDCl_3$) δ 7.13 (d, *J* = 8.8 Hz, 2H), 6.87 (d, *J* = 9.1 Hz, 2H), 3.94 (t, *J* = 6.5 Hz, 2H), 1.79 – 1.75 (m, 2H), 1.55 – 1.40 (m, 2H), 0.98 (t, *J* = 7.4 Hz, 3H); ^{13}C NMR (100 MHz, $CDCl_3$) δ 157.7, 142.5, 122.4, 120.6 (d, *J* = 255.9 Hz), 115.2, 68.1, 31.2, 19.2, 13.8; ^{19}F NMR (376 MHz, $CDCl_3$) δ -58.16 (s, OCF_3); HRMS (ESI) m/z calc. for $C_{11}H_{14}F_3O_2$ $[M+H]^+$: 235.0940, found: 235.0943.

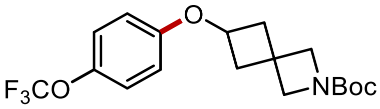

Tert-butyl 6-(4-(trifluoromethoxy)phenoxy)-2-azaspiro[3.3]heptane-2-carboxylate (224): yellow oil; ^{1}H NMR (400 MHz, $CDCl_3$) δ 7.11 (d, *J* = 8.9 Hz, 2H), 6.75 (d, *J* = 9.0 Hz, 2H), 4.53 (m, 1H), 3.98 (s, 2H), 3.94 (s, 2H), 2.77 – 2.61 (m, 2H), 2.43 – 2.24 (m, 2H), 1.44 (s, 9H); ^{13}C NMR (100 MHz, $CDCl_3$) δ 156.2, 155.7, 142.9, 122.5, 120.6 (q, *J* = 256.1 Hz), 115.6, 79.6, 67.2, 60.7, 41.1, 31.0, 28.4; ^{19}F NMR (376 MHz, $CDCl_3$) δ -58.51 (s, OCF_3); HRMS (ESI) m/z calc. for $C_{18}H_{23}F_3NO_4$ $[M+H]^+$: 374.1574, found: 374.1577.

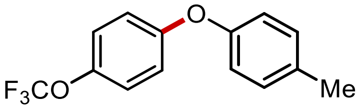

1-methyl-4-(4-(trifluoromethoxy)phenoxy)benzene (225): yellow oil; ^{1}H NMR (400 MHz, $CDCl_3$) δ 7.15 (d, *J* = 8.1 Hz, 4H), 6.96 (d, *J* = 8.9 Hz, 2H), 6.92 (d, *J* = 8.3 Hz, 2H), 2.34 (s, 3H); ^{13}C NMR (100 MHz, $CDCl_3$) δ 156.5, 154.2, 144.1, 133.6, 130.4, 122.5, 120.5 (q, *J* = 256.4 Hz), 119.3, 118.9, 22.6; ^{19}F NMR (376 MHz, $CDCl_3$) δ -58.20 (s, OCF_3); HRMS (ESI) m/z calc. for $C_{14}H_{12}F_3O_2$ $[M+H]^+$: 269.0784, found: 269.0788.

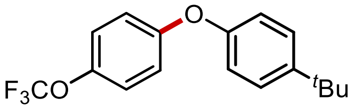

1-(Tert-butyl)-4-(4-(trifluoromethoxy)phenoxy)benzene (226): yellow oil; ^{1}H NMR (400 MHz, $CDCl_3$) δ 7.36 (d, *J* = 8.7 Hz, 2H), 7.16 (d, *J* = 8.7 Hz, 2H), 6.98 (d, *J* = 9.0 Hz, 2H), 6.94 (d, *J* = 8.7 Hz, 2H), 1.32 (s, 9H); ^{13}C NMR (100 MHz, $CDCl_3$) δ 171.1, 156.3, 154.2, 146.8, 126.7, 122.5, 120.53 (d, *J* = 256.4 Hz), 119.2, 118.7, 34.4, 31.5; ^{19}F NMR (376 MHz, $CDCl_3$) δ -58.30 (s, OCF_3); HRMS (ESI) m/z calc. for $C_{17}H_{18}F_3O_2$ $[M+H]^+$: 311.1253, found: 311.1256.

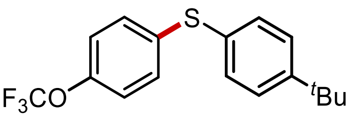

(4-(Tert-butyl)phenyl)(4-(trifluoromethoxy)phenyl)sulfane (227): yellow oil; ^{1}H NMR (400 MHz, $CDCl_3$) δ 7.36 (d, *J* = 8.6 Hz, 2H), 7.33 (d, *J* = 8.5 Hz, 2H), 7.26 (d, *J* = 8.8 Hz, 2H), 7.10 (d, *J* = 8.5 Hz, 2H), 1.31 (s, 9H); ^{13}C NMR (100 MHz, $CDCl_3$) δ 151.3, 147.8 (q, *J* = 1.7 Hz), 136.0, 132.2, 130.9, 130.6, 126.6, 121.6, 120.5 (d, *J* = 257.3 Hz), 34.7, 31.3; ^{19}F NMR (376 MHz, $CDCl_3$) δ -57.80 (s, OCF_3); HRMS (ESI) m/z calc. for $C_{17}H_{18}F_3OS$ $[M+H]^+$: 327.1025, found: 327.1028.

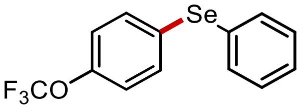

Phenyl(4-(trifluoromethoxy)phenyl)selane (228): yellow oil; ^{1}H NMR (400 MHz, $CDCl_3$) δ 7.50 – 7.44 (m, 2H), 7.46 – 7.41 (m, 2H), 7.32 – 7.23 (m, 3H), 7.09 (d, *J* = 8.2 Hz, 2H); ^{13}C NMR (100 MHz, $CDCl_3$) δ 148.6 (q, *J* = 1.8 Hz), 133.9, 133.5, 130.4, 129.9, 129.6, 127.9, 121.8, 120.5 (d, *J* = 257.4 Hz); ^{19}F NMR (376 MHz, $CDCl_3$) δ -58.48 (s, OCF_3); HRMS (ESI) m/z calc. for $C_{13}H_{10}F_3OSe$ $[M+H]^+$: 318.9843, found: 318.9846.

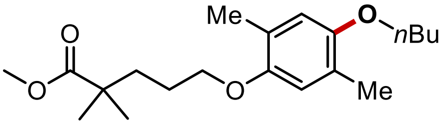

Methyl 5-(4-butoxy-2,5-dimethylphenoxy)-2,2-dimethylpentanoate (229): Yellow solid; ^{1}H NMR (400 MHz, $CDCl_3$) δ 7.06 (d, *J* = 7.4 Hz, 1H), 6.72 (d, *J* = 7.4 Hz, 1H), 4.15 (t, *J* = 6.6 Hz, 2H), 3.98 (s, 2H), 2.38 (s, 3H), 2.26 (s, 3H), 1.80 (d, *J* = 1.3 Hz, 4H), 1.73 – 1.64 (m, 2H), 1.53 – 1.41 (m, 3H), 1.30 (s, 6H), 1.02 (t, *J* = 7.4 Hz, 3H); ^{13}C NMR (100 MHz, $CDCl_3$) δ 177.7, 157.0, 136.3, 130.3, 123.5, 123.3, 120.7, 111.9, 67.9, 64.2, 42.1, 37.2, 30.7, 25.2, 25.2, 21.4, 19.2, 15.7, 13.7; HRMS (ESI) m/z calc. for $C_{20}H_{33}O_4$ $[M+H]^+$: 337.2373, found: 337.2373.

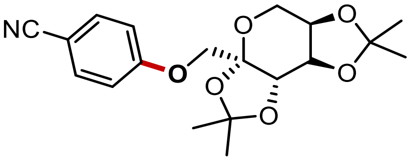

4-(((5aR,8aS,8bS)-2,2,7,7-Tetramethyltetrahydro-3aH-bis([1,3]dioxolo)[4,5-b:4',5'-d]pyran-3a-yl)methoxy)benzonitrile (230): Yellow solid; ^{1}H NMR (400 MHz, $CDCl_3$) δ 7.57 (d, *J* = 8.8 Hz, 2H), 6.97 (d, *J* = 8.8 Hz, 2H), 4.65 – 4.60 (m 1H), 4.48 (d, *J* = 2.5 Hz, 1H), 4.25 (d, *J* = 7.9 Hz, 1H), 4.18 (d, *J* = 10.3 Hz, 1H), 4.07 (d, *J* = 10.3 Hz, 1H), 3.97 – 3.91 (m, 1H), 3.77 (d, *J* = 13.0 Hz, 1H), 1.54 (s, 3H), 1.46 (s, 3H), 1.41 (s, 3H), 1.33 (s, 3H); ^{13}C NMR (100 MHz, $CDCl_3$) δ 161.8, 134.1, 119.1, 115.6, 109.2, 109.2, 104.7, 101.6, 70.9, 70.2, 70.2, 69.1, 61.4, 26.6, 26.0, 25.4, 24.1; HRMS (ESI) m/z calc. for $C_{19}H_{24}NO_6$ $[M+H]^+$: 362.1598, found: 362.1602.

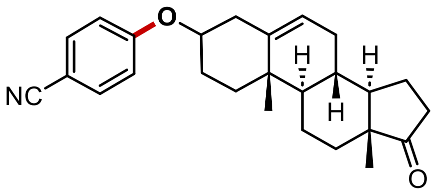

4-(((8S,9R,10R,13R,14R)-10,13-Dimethyl-17-oxo-2,3,4,5,8,9,10,11,12,13,14,15,16, 17-tetradecahydro-1H-cyclopenta[a]phenanthren-3-yl)oxy)benzonitrile (231): Yellow solid; ^{1}H NMR (400 MHz, $CDCl_3$) δ 7.55 (d, J = 8.7 Hz, 2H), 6.91 (d, J = 8.8 Hz, 2H), 5.44 (d, J = 5.1 Hz, 1H), 4.27 – 4.10 (m, 1H), 2.57 – 2.37 (m, 3H), 2.23 – 1.92 (m, 6H), 1.72 – 1.65 (m, 4H), 1.59 – 1.47 (m, 2H), 1.36 – 1.13 (m, 2H),1.15 – 1.11 (m, 1H), 1.09 (s, 3H), 1.07 – 1.01 (m, 1H), 0.89 (s, 3H); ^{13}C NMR (100 MHz, $CDCl_3$) δ 221.0, 161.3, 140.0, 134.2, 122.3, 119.4, 116.2, 103.7, 51.9, 50.4, 47.7, 38.4, 37.1, 37.1, 36.0, 31.0, 28.0, 22.0, 20.5, 19.6, 13.7; HRMS (ESI) m/z calc. for $C_{26}H_{32}NO_2$ $[M+H]^+$: 390.2438, found: 390.2445.

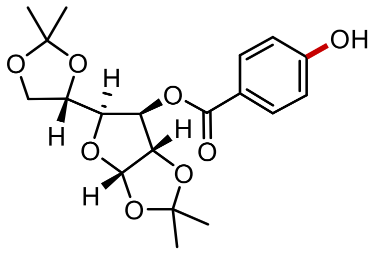

(3aS,5R,6R,6aS)-2,2,3a,5,6a-Pentamethyl-5-((S)-2,2,4-Trimethyl-1,3-dioxolan-4-yl)tetrahydrofuro [2,3-d][1,3]dioxol-6-yl 4-hydroxybenzoate (232): White solid; ^{1}H NMR (400 MHz, $CDCl_3$) δ 7.93 (d, J = 8.7 Hz, 2H), 6.84 (d, J = 8.7 Hz, 2H), 4.68 – 4.62 (m, 2H), 4.45 (d, J = 2.6 Hz, 1H), 4.36 – 4.24 (m, 2H), 3.97 – 3.92 (m, 1H), 3.83 – 3.77 (m, 1H), 1.53 (s, 3H), 1.46 (s, 3H), 1.35 (d, J = 5.7 Hz, 6H); ^{13}C NMR (100 MHz, $CDCl_3$) δ 166.2, 160.9, 132.2, 121.8, 115.5, 109.4, 109.1, 101.9, 70.9, 70.7, 70.2, 65.2, 61.4, 26.6, 26.0, 25.6, 24.1; HRMS (ESI) m/z calc. for $C_{20}H_{27}O_7$ $[M+H]^+$: 379.1751, found: 379.1759.

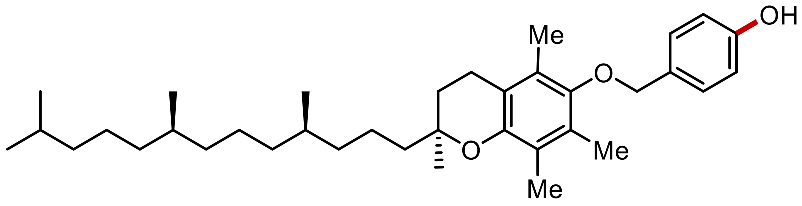

4-((((S)-2,5,7,8-Tetramethyl-2-((4R,8R,12R)-4,8,12,16-tetramethylheptadecyl) chroman-6-yl)oxy) methyl)phenol (233): White solid; ^{1}H NMR (400 MHz, $CDCl_3$) δ 7.44 (d, J = 8.3 Hz, 2H), 7.29 (d, J = 8.3 Hz, 2H), 4.57 (s, 2H), 2.52 (q, J = 6.8 Hz, 2H), 2.11 (s, 3H), 2.06 (s, 3H),

2.02 (s, 3H), 1.79 – 1.66 (m, 4H), 1.59 -1.48 (m, 6H), 1.49 -1.42 (m, 6H), 1.08 – 0.96 (m, 6H), 0.89 – 0.81 (s, 16H); ^{13}C NMR (100 MHz, $CDCl_3$) δ 148.2, 148.1, 137.3, 131.7, 129.4, 128.0, 126.0, 123.2, 121.8, 117.8, 75.0, 74.0, 40.2, 39.5, 37.6, 37.6, 37.6, 37.4, 33.0, 32.9, 31.5, 28.1, 25.0, 24.6, 24.0, 22.9, 21.2, 20.8, 19.9, 19.8, 13.0, 12.1,12.0; HRMS (ESI) m/z calc. for $C_{36}H_{57}O_3$ $[M+H]^+$: 537.4302, found: 537.4306.

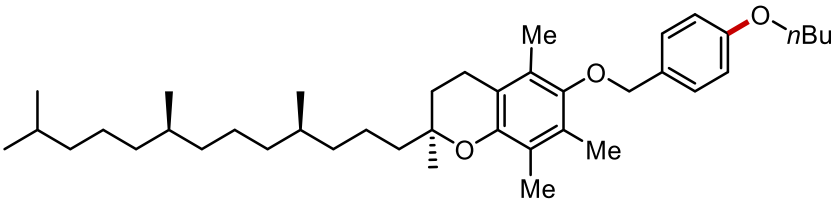

(S)-6-((4-Bbutoxybenzyl)oxy)-2,5,7,8-tetramethyl-2-((4R,8R,12R)-4,8,12,16-tetramethylheptadecyl) chromane (234): Yellow solid; ^{1}H NMR (400 MHz, $CDCl_3$) δ 7.42 (d, J = 8.5 Hz, 2H), 6.94 (d, J = 8.5 Hz, 2H), 4.64 (s, 2H), 4.01 (t, J = 6.5 Hz, 2H), 2.61 (t, J = 6.6 Hz, 2H), 2.24 (s, 3H), 2.18 (s, 3H), 2.13 (s, 3H), 1.89 – 1.76 (m, 5H), 1.65 – 1.49 (m, 7H), 1.49 – 1.29 (m, 10H), 1.21 – 1.08 (s, 8H), 1.01 (t, J = 7.4 Hz, 3H), 0.99 – 0.81 (s, 16H); ^{13}C NMR (100 MHz, $CDCl_3$) δ 159.1, 148.3, 148.0, 130.2, 129.6, 128.1, 126.1, 123.0, 117.7, 114.6, 74.9, 74.7, 67.9, 40.2, 39.5, 37.6, 37.6, 37.6, 37.4, 33.0, 32.9, 31.5, 28.1, 25.0, 24.6, 24.0, 22.9, 22.8, 21.2, 20.8, 19.9, 19.8, 19.4, 14.0, 13.1, 12.2, 12.0; HRMS (ESI) m/z calc. for $C_{40}H_{65}O_3$ $[M+H]^+$: 593.4928, found: 593.4934.

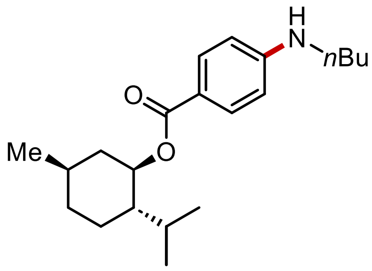

2-Isopropyl-5-methylcyclohexyl 4-(butylamino)benzoate (235): Yellow oil; ^{1}H NMR (400 MHz, $CDCl_3$) δ 7.86 (d, J = 8.7 Hz, 2H), 6.54 (d, J = 8.7 Hz, 2H), 4.92 – 4.83 (m, 1H), 4.08 (br, 1H), 3.16 (t, J = 7.1 Hz, 2H), 2.11 (d, J = 12.0 Hz, 1H), 2.02 – 1.91 (m, 1H), 1.78 – 1.67 (m, 2H), 1.65-1.58 (m, 2H), 1.57 – 1.47 (m, 2H), 1.43 (dd, J = 15.0, 7.4 Hz, 2H), 1.19 – 0.99 (m, 2H), 0.98 – 0.94 (m, 3H), 0.92 – 0.89 (m, 7H), 0.79 – 0.78 (m, 3H); ^{13}C NMR (100 MHz, $CDCl_3$) δ 166.5, 152.2, 131.7, 118.9, 111.4, 74.0, 47.6, 43.2, 41.3, 34.6, 31.6, 31.6, 26.7, 23.9, 22.2, 20.9, 20.3, 16.8, 14.0; HRMS (ESI) m/z calc. for $C_{21}H_{34}NO_2$ $[M+H]^+$: 332.2584, found: 332.2587.

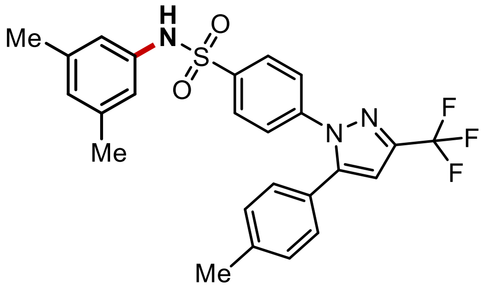

N-(3,5-Dimethylphenyl)-4-(5-(p-tolyl)-3-(trifluoromethyl)-1H-pyrazol-1-yl)benzene sulfonamide (236): **Yellow oil;** ^{1}H NMR (400 MHz, $CDCl_3$) δ 7.76 (d, *J* = 8.6 Hz, 2H), 7.39 (d, *J* = 8.6 Hz, 2H), 7.14 (d, *J* = 7.9 Hz, 2H), 7.05 (d, *J* = 8.0 Hz, 2H), 6.87 (br, 1H), 6.76 (s, 1H), 6.72 (s, 1H), 6.69 (s, 2H), 2.37 (s, 3H), 2.22 (s, 6H); ^{13}C NMR (100 MHz, $CDCl_3$) δ 145.3, 144.1 (q, *J* = 38.5 Hz), 142.6, 139.8, 139.3, 138.5, 135.8, 129.7, 128.7, 128.3, 127.6, 125.7, 125.4, 121.0 (q, *J* = 269.2 Hz), 119.4, 106.3, 21.3, 21.2; ^{19}F NMR (376 MHz, $CDCl_3$) δ -62.46 (s, CF_3); HRMS (ESI) m/z calc. for $C_{25}H_{23}F_3N_3O_2S$ $[M+H]^+$: 486.1458, found: 486.1562.

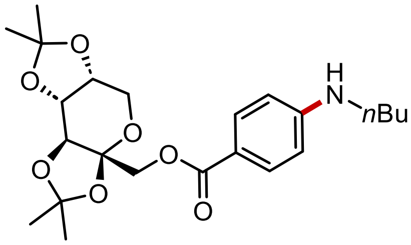

(2,2,7,7-Tetramethyltetrahydro-3aH-bis([1,3]dioxolo)[4,5-b:4',5'-d]pyran-3a-yl)methyl 4-(butylamino)benzoate (237): Yellow oil; ^{1}H NMR (400 MHz, $CDCl_3$) δ 7.89 (d, *J* = 8.8 Hz, 2H), 6.53 (d, *J* = 8.8 Hz, 2H), 4.66 – 4.59 (m, 2H), 4.49 – 4.42 (m, 1H), 4.2 – 4.20 (m, 2H), 4.11 (br, 1H), 3.99 – 3.85 (m, 1H), 3.83 – 8.71 (m, 1H), 3.16 (t, *J* = 7.1 Hz, 2H), 1.65 – 1.61 (m, 2H), 1.54 (s, 3H), 1.48 (s, 3H), 1.46 – 1.40 (m, 2H), 1.39 (s, 3H), 1.35 (s, 3H), 0.96 (t, *J* = 7.3 Hz, 3H); ^{13}C NMR (100 MHz, $CDCl_3$) δ 166.1, 152.3, 131.8, 117.6, 111.3, 109.2, 108.7, 101.9, 70.9, 70.5, 70.2, 64.5, 61.3, 43.0, 31.4, 26.6, 25.9, 25.6, 24.1, 20.2, 13.8; HRMS (ESI) m/z calc. for $C_{23}H_{34}NO_7$. $[M+H]^+$: 436.2330, found: 436.2335.

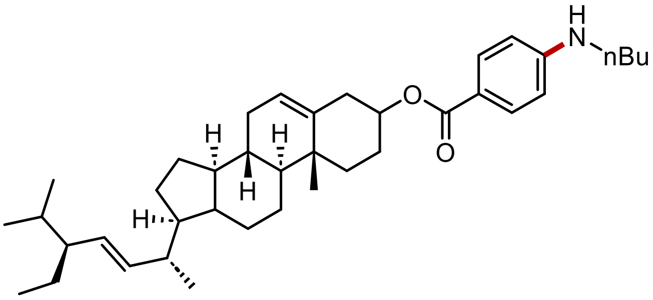

((8S,9S,10R,14R,17R)-17-((2R,5S,E)-5-Ethyl-6-methylhept-3-en-2-yl)-10-methyl-2,3,4,7,8,9, 10,11,12,13,14,15,16,17-tetradecahydro-1H-cyclopenta[a]phenanthren-3-yl 4-(butylamino) benzoate (238): Yellow solid; ^{1}H NMR (400 MHz, $CDCl_3$) δ 7.86 (d, *J* = 8.7 Hz, 2H), 6.53 (d, *J* = 8.8 Hz, 2H), 5.40 (d, *J* = 4.8 Hz, 1H), 5.16 (dd, *J* = 15.1, 8.6 Hz, 1H), 5.02 (dd, *J* = 15.2,

8.6 Hz, 1H), 4.89 – 4.80 (m, 1H), 4.06 (br, 1H), 3.16 (t, *J* = 7.1 Hz, 2H), 2.43 (d, *J* = 7.0 Hz, 2H), 2.09 – 1.93 (m, 4H), 1.93 – 1.85 (m, 1H), 1.78 – 1.66 (m, 2H), 1.66 – 1.37 (m, 13H), 1.21 – 1.13 (m, 5H), 1.12 – 1.05 (m, 3H), 1.04 – 1.02 (m, 2H), 1.02 – 0.99 (m, 2H), 0.98 – 0.96 (m, 3H), 0.89 – 0.86 (m, 3H), 0.85 – 0.3 (m, 1H), 0.82 – 0.79 (m, 5H), 0.74 – 0.70 (m, 3H); ^{13}C NMR (100 MHz, $CDCl_3$) δ 166.3, 152.0, 140.0, 138.3, 131.5, 129.3, 122.5, 118.8, 111.3, 73.6, 56.8, 56.0, 51.2, 50.1, 43.1, 42.2, 40.5, 39.7, 38.4, 37.1, 36.7, 32.01, 31.9, 31.8, 31.4, 28.9, 28.0, 25.4, 24.4, 21.2, 21.1, 21.0, 20.2, 19.4, 19.0, 13.8, 12.3, 12.1; HRMS (ESI) m/z calc. for $C_{39}H_{60}NO_2$ $[M+H]^+$: 574.4619, found: 574.4623.

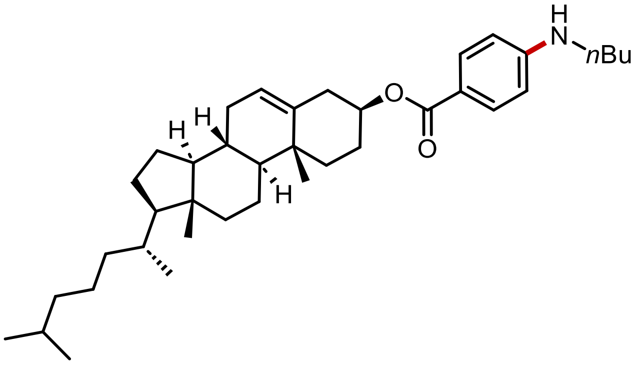

(3S,8S,9S,10R,13R,14S,17R)-10,13-Dimethyl-17-((R)-6-methylheptan-2-yl)-2,3,4,7,8, 9,10,11, 12,13,14,15,16,17-tetradecahydro-1H-cyclopenta[a]phenanthren-3-yl 4-(butylamino) benzoate (239)**:** Yellow solid; ^{1}H NMR (400 MHz, $CDCl_3$) δ 7.79 (d, *J* = 7.6 Hz, 2H), 6.47 (d, *J* = 7.8 Hz, 2H), 5.33 (s, 1H), 4.73 (s, 1H), 4.16 (br, 1H), 3.09 (t, *J* = 6.4 Hz, 2H), 2.36 (d, *J* = 6.8 Hz, 2H), 1.98 – 1.71 (m, 5H), 1.59 – 1.22 (m, 14H), 1.23 – 1.00 (m, 8H), 1.12 – 0.96 (m, 4H), 0.94 – 0.82 (m, 8H), 0.79 (d, *J* = 6.3 Hz, 6H), 0.61 (s, 3H); ^{13}C NMR (100 MHz, $CDCl_3$) δ 166.3, 151.9, 140.0, 131.5, 122.5, 118.8, 111.4, 73.7, 56.7, 56.1, 50.0, 43.2, 42.3, 39.8, 39.5, 38.4, 37.1, 36.7, 36.2, 35.8, 32.0, 31.9, 31.4, 28.3, 28.0, 24.3, 23.8, 22.9, 22.6, 21.1, 20.2, 19.4, 18.7, 13.9, 11.9; HRMS (ESI) m/z calc. for $C_{38}H_{60}NO_2$ $[M+H]^+$: 562.4619, found: 562.4615.

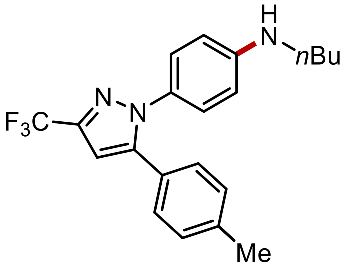

N-butyl-4-(5-(p-tolyl)-3-(trifluoromethyl)-1H-pyrazol-1-yl)aniline (240) : Yellow solid; ^{1}H NMR (400 MHz, $CDCl_3$) δ 7.10 – 6.93 (m, 6H), 6.60 (s, 1H), 6.46 (d, *J* = 8.8 Hz, 2H), 3.77 (br, 1H), 3.03 (t, *J* = 7.1 Hz, 2H), 2.26 (s, 3H), 1.59 – 1.48 (m, 2H), 1.39 – 1.28 (m, 2H), 0.88 (t, *J* = 7.3 Hz, 3H); ^{13}C NMR (100 MHz, $CDCl_3$) δ 148.3, 144.4, 142.4 (q, *J* = 38.0 Hz), 138.6, 129.4, 129.2, 128.6, 126.8, 126.6, 121.5 (q, *J* = 268.8 Hz), 112.5, 104.51 (q, *J* = 1.8 Hz), 43.7, 31.4, 21.3, 20.3, 13.9; ^{19}F NMR (376 MHz, $CDCl_3$) δ -62.12 (s, CF_3); HRMS (ESI) m/z calc. for $C_{21}H_{23}F_3N_3$ $[M+H]^+$: 374.1839, found: 374.1843.

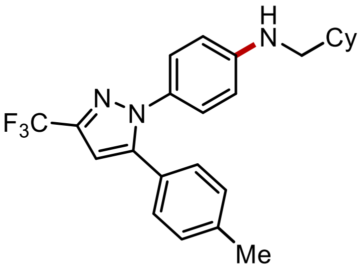

***N*-(Cyclohexylmethyl)-4-(5-(p-tolyl)-3-(trifluoromethyl)-1*H*-pyrazol-1-yl)aniline (241):** Yellow solid; ^{1}H NMR (400 MHz, $CDCl_3$) δ 7.10 – 6.91 (m, 6H), 6.59 (s, 1H), 6.42 (d, J = 8.8 Hz, 2H), 3.83 (br, 1H), 2.85 (d, J = 6.7 Hz, 2H), 2.25 (s, 3H), 1.91 – 1.56 (m, 5H), 1.69 – 1.42 (m, 1H), 1.24 – 1.08 (m, 3H), 0.96 – 0.82 (m, 2H); ^{13}C NMR (100 MHz, $CDCl_3$) δ 148.6, 144.4, 142.3 (q, J = 38.1 Hz), 138.6, 129.2, 129.1, 128.6, 126.7, 126.6, 121.52 (q, J = 268.8 Hz), 112.4, 104.48 (q, J = 1.9 Hz), 50.5, 37.5, 31.2, 26.5, 25.9, 21.2; ^{19}F NMR (376 MHz, $CDCl_3$) δ -61.9 (s, CF_3); HRMS (ESI) m/z calc. for $C_{24}H_{27}F_3N_3$ $[M+H]^+$: 414.2152, found: 414.2158.

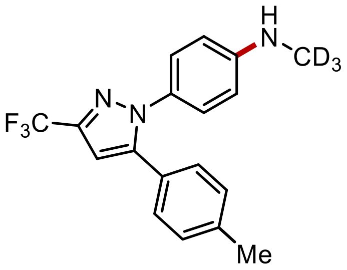

***N*-(Methyl-d3)-4-(5-(p-tolyl)-3-(trifluoromethyl)-1*H*-pyrazol-1-yl)aniline (242):** Yellow solid; ^{1}H NMR (400 MHz, $CDCl_3$) δ 7.09 – 6.97 (m, 6H), 6.60 (s, 1H), 6.44 (d, J = 8.7 Hz, 2H), 3.65 (br, 1H), 2.26 (s, 3H); ^{13}C NMR (100 MHz, $CDCl_3$) δ 149.3, 144.6, 142.4 (q, J = 37.8 Hz), 138.7, 129.4, 129.2, 128.5, 126.9, 126.7, 121.6 (q, J = 268.6 Hz), 112.2 (q, J = 6.2 Hz), 104.6, 29.8 (q, J_{C-D} = 40.9 Hz), 21.3; ^{19}F NMR (376 MHz, $CDCl_3$) δ -61.88 (s, CF_3); HRMS (ESI) m/z calc. for $C_{18}H_{14}D_3F_3N_3$ $[M+H]^+$: 335.1557, found: 335.1554.

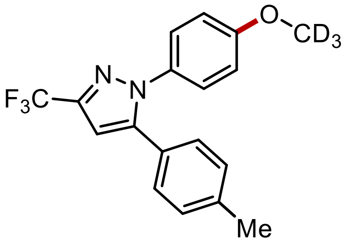

1-(4-(Methoxy-d3)phenyl)-5-(p-tolyl)-3-(trifluoromethyl)-1H-pyrazole (243): Yellow solid: ^{1}H NMR (400 MHz, $CDCl_3$) δ 7.23 (d, J = 8.9 Hz, 2H), 7.15 – 7.07 (m, 4H), 6.86 (d, J = 8.9 Hz, 2H), 6.70 (br, 1H), 2.35 (s, 3H); ^{13}C NMR (150 MHz, $CDCl_3$) δ 159.6, 144.8, 142.9 (q, J = 38.0 Hz), 139.1, 132.6, 129.5, 128.7, 127.0, 126.5, 121.5 (q, J = 268.9 Hz), 114.3, 105.0, 54.9 (q, J_{C-D} = 43.5 Hz), 21.4; ^{19}F NMR (376 MHz, $CDCl_3$) δ -61.99 **(s,** CF_3**);** HRMS (ESI) m/z calc. for $C_{18}H_{14}D_3F_3N_2O$ $[M+H]^+$: 336.1398, found: 336.1405.

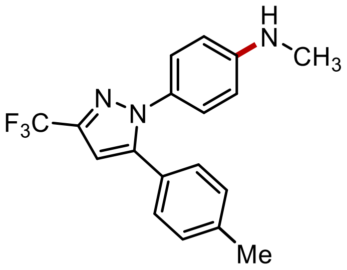

N,N-Dimethyl-4-(5-(p-tolyl)-3-(trifluoromethyl)-1H-pyrazol-1-yl)aniline(244): Yellow solid; ^{1}H NMR (400 MHz, $CDCl_3$) δ 7.07 – 7.00 (m, 6H), 6.60 (s, 1H), 6.45 (d, *J* = 8.8 Hz, 2H), 3.76 (s, 1H), 2.76 (s, 3H), 2.26 (s, 3H); ^{13}C NMR (100 MHz, $CDCl_3$) δ 149.2, 144.5, 142.4 (q, *J* = 38.1 Hz), 138.6, 129.4, 129.3, 128.6, 126.7, 126.6, 121.50 (q, *J* = 268.7 Hz), 112.1, 104.5 (q, *J* = 2.0 Hz), 30.6, 21.3; ^{19}F NMR (376 MHz, $CDCl_3$) δ -61.89 (s, CF_3); HRMS (ESI) m/z calc. for $C_{18}H_{17}F_3N_3$ $[M+H]^+$: 332.1369, found: 332.1375.

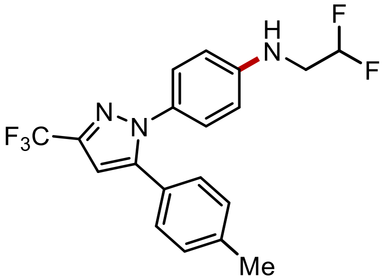

N-(2,2-Difluoroethyl)-4-(5-(p-tolyl)-3-(trifluoromethyl)-1H-pyrazol-1-yl)aniline (245): Yellow solid; ^{1}H NMR (400 MHz, $CDCl_3$) δ 7.08 – 6.97 (m, 6H), 6.60 (s, 1H), 6.49 (d, *J* = 8.7 Hz, 2H), 5.81 (tt, *J* = 55.9, 4.0 Hz, 1H), 3.99 (br, 1H), 3.43 (td, *J* = 14.5, 3.8 Hz, 2H), 2.25 (s, 3H); ^{13}C NMR (100 MHz, $CDCl_3$) δ 146.9, 144.6, 142.6 (q, *J* = 38.2 Hz), 138.9, 130.6, 129.3, 128.6, 126.9, 126.4, 121.5 (q, *J* = 268.7 Hz), 114.3 (t, *J* = 242.1 Hz), 112.9, 104.7 (q, *J* = 1.9 Hz), 46.3 (t, *J* = 26.0 Hz), 21.2; ^{19}F NMR (376 MHz, $CDCl_3$) δ -61.92 (s, CF_3), -122.50 (dt, *J* = 56.6, 14.7 Hz, CF_2); HRMS (ESI) m/z calc. for $C_{19}H_{17}F_5N_3$ $[M+H]^+$: 382.1337, found: 382.1342.

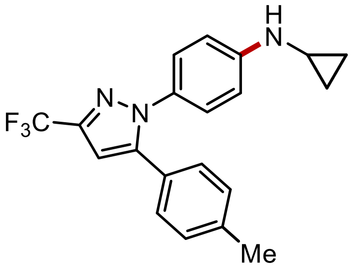

N-cyclopropyl-4-(5-(p-tolyl)-3-(trifluoromethyl)-1H-pyrazol-1-yl)aniline (246): Yellow solid; ^{1}H NMR (400 MHz, $CDCl_3$) δ 7.11 – 6.96 (m, 6H), 6.72 – 6.54 (m, 3H), 4.22 (br, 1H), 2.38 – 2.31 (m, 1H), 2.26 (s, 3H), 0.69 – 0.62 (m, 2H), 0.46 – 0.40 (m, 2H); ^{13}C NMR (100 MHz, CDCl3) δ 148.7, 144.5, 142.4 (q, *J* = 38.1 Hz), 138.7, 129.9, 129.2, 128.6, 128.5, 126.6, 121.50 (q, *J* = 268.8 Hz), 112.9, 104.54 (q, J = 2.0 Hz), 25.3, 21.3, 7.5; ^{19}F NMR (376 MHz, $CDCl_3$) δ -61.89 (s, CF_3); HRMS (ESI) m/z calc. for $C_{20}H_{19}F_3N_3$ $[M+H]^+$: 358.1526, found: 358.1531.

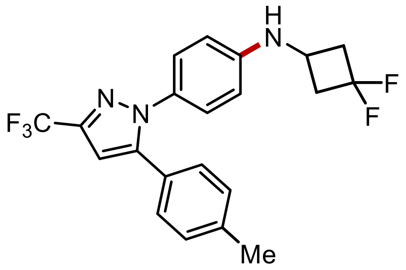

N-(3,3-Difluorocyclobutyl)-4-(5-(p-tolyl)-3-(trifluoromethyl)-1H-pyrazol-1-yl)aniline(247): Yellow solid; ^{1}H NMR (400 MHz, $CDCl_3$) δ 7.08 – 7.00 (m, 6H), 6.60 (s, 1H), 6.40 (d, *J* = 8.8 Hz, 2H), 3.99 (s, 1H), 3.76 (s, 1H), 3.03 – 2.88 (m, 2H), 2.44 – 2.28 (m, 2H), 2.26 (s, 3H); ^{13}C NMR (100 MHz, $CDCl_3$) δ 146.5, 144.5, 142.6 (q, *J* = 38.1 Hz), 138.8, 130.5, 129.3, 128.6, 126.9, 126.5, 121.4 (d, *J* = 268.8 Hz), 118.8 (dd, *J* = 282.1, 272.3 Hz), 113.0, 104.7 (q, *J* = 2.0 Hz), 43.3 (dd, *J* = 23.3, 21.8 Hz), 38.2 (dd, *J* = 15.5, 7.6 Hz), 21.2; ^{19}F NMR (376 MHz, $CDCl_3$) δ -61.93 (s, CF_3), -83.09 – -83.77 (m, F), -94.86 – -95.59 (m, F); HRMS (ESI) m/z calc. for $C_{21}H_{19}F_5N_3$ $[M+H]^+$: 408.1494, found: 408.1499.

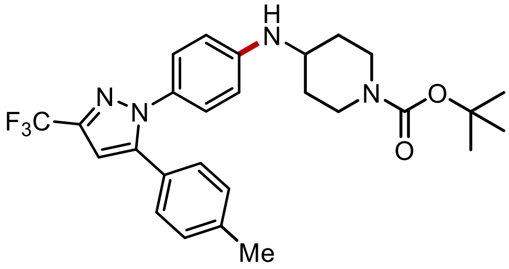

Tert-butyl 4-((4-(5-(p-tolyl)-3-(trifluoromethyl)-1H-pyrazol-1-yl)phenyl)amino)piperidine-1-carboxylate (248): Yellow solid; ^{1}H NMR (400 MHz, $CDCl_3$) δ 7.09 – 6.95 (m, 6H), 6.60 (s, 1H), 6.45 (d, *J* = 8.7 Hz, 2H), 3.97 (s, 2H), 3.42 – 3.27 (m, 1H), 2.83 (t, *J* = 12.0 Hz, 2H), 2.26 (s, 3H), 2.04 – 1.83 (m, 2H), 1.39 (s, 9H), 1.29 – 1.18 (m, 2H); ^{13}C NMR (100 MHz, $CDCl_3$) δ 154.8, 146.7, 144.5, 142.4 (q, *J* = 38.1 Hz), 138.7, 129.6, 129.3, 128.6, 126.7, 126.6, 121.5 (d, *J* = 268.8 Hz), 113.0, 104.58 (q, *J* = 1.8 Hz), 79.7, 50.2, 42.6, 32.2, 28.4, 21.3; ^{19}F NMR (376 MHz, $CDCl_3$) δ -61.90 (s, CF_3); HRMS (ESI) m/z calc. for $C_{27}H_{32}F_3N_4O_2$ $[M+H]^+$: 501.2472, found: 501.2476.

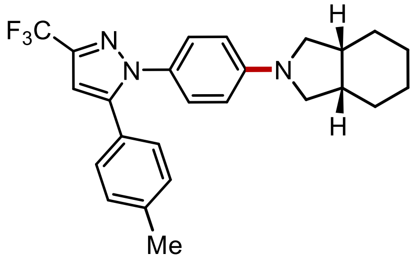

(3aR,7aS)-2-(4-(5-(p-Tolyl)-3-(trifluoromethyl)-1H-pyrazol-1-yl)phenyl)octahydro-1H-isoindole (249): Yellow solid; ^{1}H NMR (400 MHz, $CDCl_3$) δ 7.04 (dd, *J* = 13.9, 8.4 Hz, 6H), 6.59 (s, 1H), 6.33 (d, *J* = 8.8 Hz, 2H), 3.21 (dd, *J* = 8.8, 6.8 Hz, 2H), 3.07 (dd, *J* = 9.1, 5.0 Hz,

2H), 2.34 – 2.17 (m, 5H), 1.65 – 1.52 (m, 2H), 1.49 – 1.36 (m, 4H), 1.36 – 1.25 (m, 2H); ^{13}C NMR (100 MHz, $CDCl_3$) δ 148.0, 144.3, 142.2 (q, J = 38.1 Hz), 138.5, 129.2, 128.6, 127.6, 126.8, 126.5, 121.57 (q, J = 268.7 Hz), 110.8, 104.43 (q, J = 2.0 Hz), 51.9, 37.3, 26.3, 23.0, 21.3; ^{19}F NMR (376 MHz, $CDCl_3$) δ -61.82 (s, CF_3); HRMS (ESI) m/z calc. for $C_{25}H_{27}F_3N_3$ $[M+H]^+$: 426.2152, found: 426.2155.

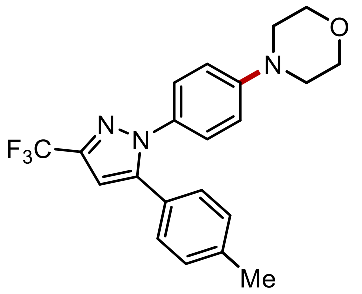

4-(4-(5-(p-Tolyl)-3-(trifluoromethyl)-1H-pyrazol-1-yl)phenyl)morpholine (250): Yellow solid; ^{1}H NMR (400 MHz, $CDCl_3$) δ 7.11 (d, J = 8.9 Hz, 2H), 7.03 (s, 4H), 6.76 (d, J = 9.0 Hz, 2H), 6.60 (s, 1H), 3.85 – 3.69 (m, 4H), 3.15 – 2.99 (m, 4H), 2.26 (s, 3H); ^{13}C NMR (100 MHz, $CDCl_3$) δ 151.0, 144.6, 142.7 (q, J = 38.1 Hz), 138.8, 131.5, 129.3, 128.6, 126.5, 126.4, 121.4 (q, J = 268.8 Hz), 115.3, 104.8 (q, J = 2.0 Hz), 66.7, 48.8, 21.3; ^{19}F NMR (376 MHz, $CDCl_3$) δ -61.94 (s, CF_3); HRMS (ESI) m/z calc. for $C_{21}H_{21}F_3N_3O$ $[M+H]^+$: 388.1631, found: 388.1635.

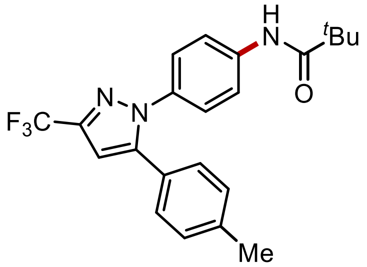

N-(4-(5-(p-Tolyl)-3-(trifluoromethyl)-1H-pyrazol-1-yl)phenyl)pivalamide(251): Yellow solid; ^{1}H NMR (400 MHz, $CDCl_3$) δ 7.54 (d, J = 8.8 Hz, 2H), 7.39 (br, 1H), 7.11 (m, 6H), 6.70 (s, 1H), 2.35 (s, 3H), 1.32 (s, 9H); ^{13}C NMR (100 MHz, $CDCl_3$) δ 176.7, 144.78, 142.7 (q, J = 38.4 Hz), 139.1, 138.1, 135.1, 129.4, 128.7, 126.1, 121.3 (q, J = 268.7 Hz), 120.1, 112.1, 105.2, 39.7, 27.6, 21.3; ^{19}F NMR (376 MHz, $CDCl_3$) δ -62.20 (s, CF_3); HRMS (ESI) m/z calc. for $C_{22}H_{23}F_3N_3O$ $[M+H]^+$: 402.1788, found: 402.1793.

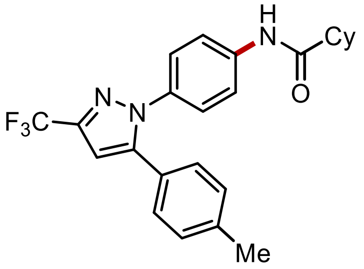

N-(4-(5-(p-Tolyl)-3-(trifluoromethyl)-1H-pyrazol-1-yl)phenyl)cyclohexanecarboxamide (252): Yellow solid; ^{1}H NMR (400 MHz, $CDCl_3$) δ 7.53 (d, J = 8.5 Hz, 1H), 7.35 (br, 1H), 7.26

– 7.21 (m, 2H), 7.11 (dd, *J* = 12.6, 9.5 Hz, 5H), 6.70 (s, 1H), 2.34 (s, 3H), 2.22 (d, *J* = 7.4 Hz, 1H), 1.98 – 1.93 (m, 2H), 1.88 – 1.82 (m, 2H), 1.77 – 1.70 (m, 1H), 1.56 – 1.51 (m, 2H), 1.35 – 1.29 (m, 3H); ^{13}C NMR (100 MHz, $CDCl_3$) δ 174.5, 144.8, 143.0 (q, *J* = 38.0 Hz), 139.1, 138.2, 135.0, 129.4, 128.7, 126.1, 121.3 (q, *J* = 269.0 Hz), 119.8; 112.1, 105.18 (q, *J* = 1.8 Hz), 41.4, 29.6, 25.6, 22.6, 21.3; ^{19}F NMR (376 MHz, $CDCl_3$) δ-62.12 (s, CF_3); HRMS (ESI) m/z calc. for $C_{24}H_{25}F_3N_3O$ $[M+H]^+$: 428.1944, found: 428.1948.

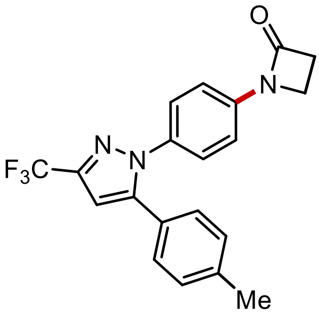

1-(4-(5-(p-Tolyl)-3-(trifluoromethyl)-1H-pyrazol-1-yl)phenyl)azetidin-2-one (253): Yellow solid; ^{1}H NMR (400 MHz, $CDCl_3$) δ 7.27 (d, *J* = 8.9 Hz, 1H), 7.20 (d, *J* = 10.8 Hz, 3H), 7.08 – 6.98 (m, 4H), 6.63 (s, 1H), 3.58 (t, *J* = 4.5 Hz, 2H), 3.08 (t, *J* = 4.5 Hz, 2H), 2.28 (s, 3H); ^{13}C NMR (150 MHz, $CDCl_3$) δ 164.5, 144.8, 143.1 (q, *J* = 38.3 Hz), 139.1, 138.3, 134.9, 129.4, 128.7, 126.4, 126.2, 121.3 (d, *J* = 268.7 Hz), 116.4, 105.2 (q, *J* = 2.1 Hz), 38.2, 36.4, 21.3; ^{19}F NMR (376 MHz, $CDCl_3$) δ -61.88 (s, CF_3); HRMS (ESI) m/z calc. for $C_{20}H_{17}F_3N_3O$ $[M+H]^+$: 372.1318, found: 372.1322.

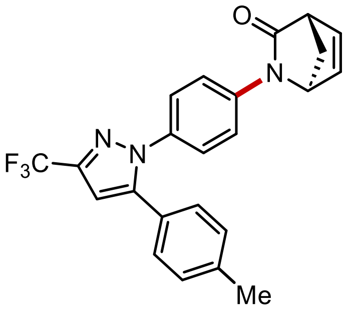

(1S,4S)-2-(4-(5-(p-Tolyl)-3-(trifluoromethyl)-1H-pyrazol-1-yl)phenyl)-2-azabicyclo[2.2.1] hept-5-en-3-one (254): Yellow oid; ^{1}H NMR (400 MHz, $CDCl_3$) δ 7.33 (d, *J* = 8.9 Hz, 2H), 7.19 (s, 3H), 7.04 (d, *J* = 3.0 Hz, 4H), 6.99 – 6.81 (m, 1H), 6.67 (t, *J* = 3.5 Hz, 1H), 6.62 (s, 1H), 4.73 (d, *J* = 1.7 Hz, 1H), 3.45 (s, 1H), 2.42 (d, *J* = 8.1 Hz, 1H), 2.28 (s, 3H), 2.23 (d, *J* = 8.0 Hz, 1H); ^{13}C NMR (100 MHz, $CDCl_3$) δ 177.3, 144.7, 143.0 (q, *J* = 38.3 Hz), 139.5, 139.1, 138.8, 134.9, 129.5, 128.7, 126.0, 121.3 (q, *J* = 268.9 Hz), 118.5, 105.25 (q, *J* = 1.8 Hz), 64.5, 57.0, 54.6, 21.3; ^{19}F NMR (376 MHz, $CDCl_3$) δ -62.07(s, CF_3); HRMS (ESI) m/z calc. for $C_{29}H_{19}F_3N_3O$ $[M+H]^+$: 410.1475, found: 410.1779.

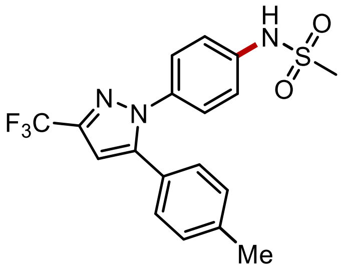

***N*-(4-(5-(p-Tolyl)-3-(trifluoromethyl)-1H-pyrazol-1-yl)phenyl)methanesulfonamide (255):** Yellow solid; ^{1}H NMR (400 MHz, $CDCl_3$) δ 7.23 (d, *J* = 8.7 Hz, 2H), 7.15 (d, *J* = 8.7 Hz, 2H), 7.11 (s, 1H), 7.05 (q, *J* = 8.2 Hz, 4H), 6.64 (s, 1H), 2.95 (s, 3H), 2.29 (s, 3H); ^{13}C NMR (100 MHz, $CDCl_3$) δ 144.9, 143.3 (q, *J* = 38.4 Hz), 139.3, 137.0, 136.2, 129.5, 128.7, 126.8, 126.0, 121.2 (d, *J* = 268.9 Hz), 120.2, 105.4 (q, *J* = 2.0 Hz), 39.6, 21.3; ^{19}F NMR (376 MHz, $CDCl_3$) δ -62.05(s, CF_3); HRMS (ESI) m/z calc. for $C_{18}H_{17}F_3N_3O_2S$ $[M+H]^+$: 396.0988, found: 396.0993.

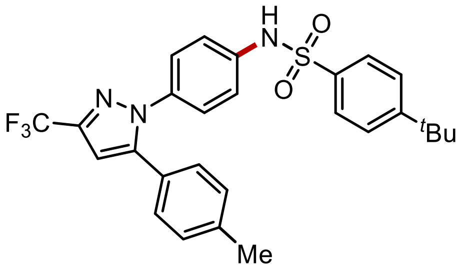

4-Methyl-N-(4-(5-(p-tolyl)-3-(trifluoromethyl)-1H-pyrazol-1-yl)phenyl)benzenesulfonamide (256): Yellow solid; ^{1}H NMR (400 MHz, $CDCl_3$) δ 7.64 (d, *J* = 8.5 Hz, 2H), 7.39 (d, *J* = 8.4 Hz, 2H), 7.19 (s, 1H), 7.10 (br, 1H), 7.07 – 6.96 (m, 7H), 6.61 (s, 1H), 2.28 (s, 3H), 1.24 (s, 9H); ^{13}C NMR (100 MHz, $CDCl_3$) δ 157.3, 144.8, 142.6 (d, *J* = 38.5 Hz), 139.3, 136.8, 136.0, 135.7, 129.4, 128.6, 127.1, 126.5, 126.2, 126.0, 121.6 (q, *J* = 277.9 Hz), 120.1, 105.3 (q, *J* = 1.9 Hz), 35.2, 31.0, 21.3; ^{19}F NMR (376 MHz, $CDCl_3$) δ 61.89 (s, CF_3); HRMS (ESI) m/z calc. for $C_{27}H_{27}F_3N_3O_2S$ $[M+H]^+$: 514.1771, found: 514.1776.

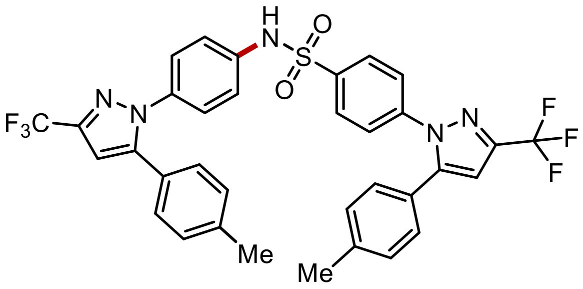

4-(5-(p-Tolyl)-3-(trifluoromethyl)-1H-pyrazol-1-yl)-N-(4-(5-(p-tolyl)-3-(trifluoromethyl)-1H-pyrazol-1-yl)phenyl)benzenesulfonamide (257): Yellow solid; ^{1}H NMR (400 MHz, $CDCl_3$) δ 8.14 (br, 1H), 7.67 (s, 1H), 7.65 (s, 1H), 7.32 (s, 1H), 7.29 (s, 1H), 7.21 –7.01 (m, 6H), 6.99 – 6.91 (m, 6H), 6.64 (s, 1H), 6.60 (s, 1H), 2.24 (s, 3H), 2.22 (s, 3H); δ ^{13}C NMR (100 MHz, $CDCl_3$) δ 145.3, 144.9, 144.2 (q, *J* = 38.6 Hz), 143.2 (q, *J* = 38.5 Hz), 142.8, 139.9, 139.3,

138.6, 136.6, 136.4, 129.8, 129.4, 128.7, 128.6, 128.3, 126.4, 126.0, 125.6, 125.3, 121.8, 121.2 (q, J = 269.0 Hz), 121.0 (q, J = 269.2 Hz), 106.4, 105.4, 21.2, 21.2; ^{19}F NMR (376 MHz, $CDCl_3$) δ -62.10 (s, CF_3), -62.33 (s, CF_3); HRMS (ESI) m/z calc. for $C_{34}H_{26}F_6N_5O_2S$ $[M+H]^+$: 682.1706, found: 682.1710.

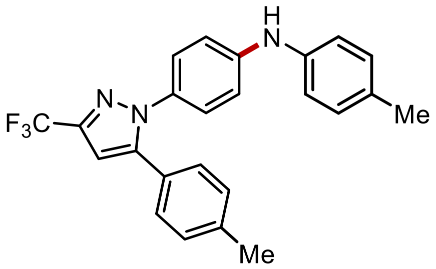

4-Methyl-N-(4-(5-(p-tolyl)-3-(trifluoromethyl)-1H-pyrazol-1-yl)phenyl)aniline(258): Yellow solid; ^{1}H NMR (400 MHz, $CDCl_3$) δ 7.08 – 7.03 (m, 7H), 7.02 (s, 1H), 6.93 (d, J = 8.2 Hz, 2H), 6.83 (d, J = 8.7 Hz, 2H), 6.61 (s, 1H), 5.62 (br, 1H), 2.27 (s, 3H), 2.23 (s, 3H); ^{13}C NMR (100 MHz, $CDCl_3$) δ 144.6, 144.5, 142.62 (q, J = 38.0 Hz), 139.1, 138.8, 132.2, 131.5, 130.0, 129.3, 128.6, 126.7, 126.5, 121.4 (q, J = 268.8 Hz); 120.0, 115.85, 104.8, 21.3, 20.7; ^{19}F NMR (376 MHz, $CDCl_3$) δ -62.03 (s, CF_3), HRMS (ESI) m/z calc. for $C_{24}H_{21}F_3N_3$ $[M+H]^+$: 408.1682, found: 408.1686.

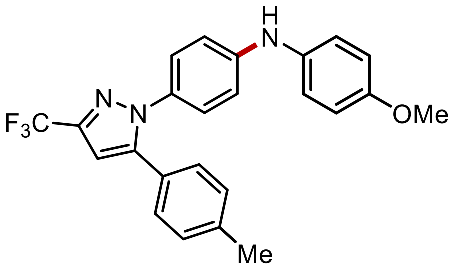

4-methoxy-N-(4-(5-(p-tolyl)-3-(trifluoromethyl)-1H-pyrazol-1-yl)phenyl)aniline (259): Yellow solid; ^{1}H NMR (400 MHz, $CDCl_3$) δ 7.10 – 6.96 (m, 8H), 6.80 (d, J = 8.9 Hz, 2H), 6.74 (d, J = 8.8 Hz, 2H), 6.61 (s, 1H), 5.53 (s, 1H), 3.73 (s, 3H), 2.28 (s, 3H); ^{13}C NMR (100 MHz, $CDCl_3$) δ 156.0, 145.6, 144.5, 142.55 (q, J = 38.1 Hz), 138.8, 134.6, 131.0, 129.3, 128.6, 126.7, 126.5, 123.2, 121.44 (q, J = 268.7 Hz), 114.8, 114.8, 104.7 (q, J = 1.8 Hz), 55.6, 21.3; ^{19}F NMR (376 MHz, $CDCl_3$) δ -61.99 (s, CF_3), HRMS (ESI) m/z calc. for $C_{24}H_{21}F_3N_3O$ $[M+H]^+$: 424.1631, found: 424.1636.

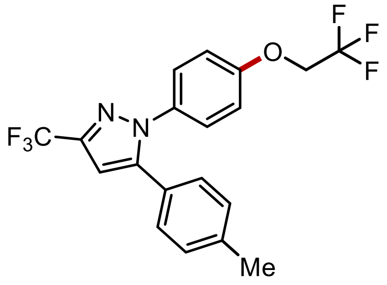

5-(p-Tolyl)-1-(4-(2,2,2-trifluoroethoxy)phenyl)-3-(trifluoromethyl)-1H-pyrazole (260): colourless oil; ^{1}H NMR (400 MHz, $CDCl_3$) δ 7.26 – 7.14 (m, 2H), 7.03 (q, *J* = 8.2 Hz, 4H), 6.83 (d, *J* = 8.9 Hz, 2H), 6.62 (s, 1H), 4.27 (q, *J* = 8.0 Hz, 2H), 2.26 (s, 3H); ^{13}C NMR (100 MHz, $CDCl_3$) δ 157.0, 144.8, 143.1 (q, *J* = 38.3 Hz), 139.2, 134.1, 129.4, 128.7, 127.1, 126.2, 121.3 (d, *J* = 268.9 Hz), 123.1 (q, *J* = 278.0 Hz), 115.3, 105.2 (q, *J* = 1.9 Hz), 65.9 (q, *J* = 35.9 Hz), 21.2; ^{19}F NMR (376 MHz, $CDCl_3$) δ -62.08 (s, CF_3), -73.79 (t, *J* = 8.6 Hz, CF_3)**;** HRMS (ESI) m/z calc. for $C_{19}H_{15}F_6N_2O$ $[M+H]^+$: 401.1083, found: 401.1086.

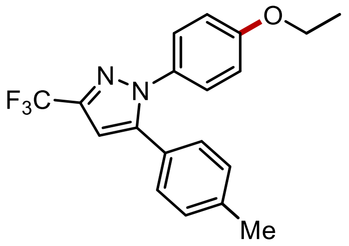

1-(4-Ethoxyphenyl)-5-(p-tolyl)-3-(trifluoromethyl)-1H-pyrazole (261): colourless oil; ^{1}H NMR (400 MHz, $CDCl_3$) δ 7.13 (d, *J* = 8.9 Hz, 2H), 7.08 – 6.98 (m, 4H), 6.77 (d, *J* = 8.9 Hz, 2H), 6.61 (s, 1H), 3.95 (q, *J* = 7.0 Hz, 2H), 2.26 (s, 3H), 1.33 (t, *J* = 7.0 Hz, 3H); ^{13}C NMR (100 MHz, $CDCl_3$) δ 158.8, 144.7, 142.7 (q, *J* = 38.1 Hz), 138.9, 132.3, 129.3, 128.6, 126.9, 126.4, 121.4 (q, *J* = 268.9 Hz), 114.7, 104.8 (q, *J* = 1.9 Hz), 63.8, 21.3, 14.7; ^{19}F NMR (376 MHz, $CDCl_3$) δ -62.12 (s, CF_3); HRMS (ESI) m/z calc. for $C_{19}H_{18}F_3N_2O$ $[M+H]^+$: 347.1366, found: 347.1371.

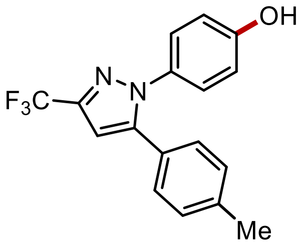

4-(5-(p-Tolyl)-3-(trifluoromethyl)-1H-pyrazol-1-yl)phenol (262)*:* colourless oil; ^{1}H NMR (400 MHz, $CDCl_3$) δ 7.30 (d, *J* = 8.9 Hz, 2H), 7.13 (d, *J* = 3.7 Hz, 5H), 6.99 (d, *J* = 8.9 Hz, 2H), 6.72 (s, 1H), 2.36 (s, 3H); ^{13}C NMR (100 MHz, $CDCl_3$) δ 155.5, 143.8, 143.2 (q, *J* = 38.8 Hz), 138.2, 134.0, 128.4, 127.6, 126.2, 125.1, 121.3 (q, *J* = 269.9 Hz), 118.3, 104.3 (q, *J* = 2.1 Hz), 20.28 (s); ^{19}F NMR (376 MHz, $CDCl_3$) δ -62.12 (s, CF_3); HRMS (ESI) m/z calc. for $C_{17}H_{14}F_3N_2O$ $[M+H]^+$: 319.1053, found: 319.1056.

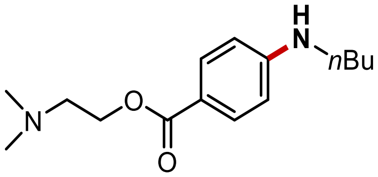

2-(Dimethylamino)ethyl 4-(butylamino)benzoate (263): White solid; ^{1}H NMR (400 MHz, $CDCl_3$) δ 7.84 (d, *J* = 8.8 Hz, 2H), 6.50 (d, *J* = 8.8 Hz, 2H), 4.35 (t, *J* = 5.9 Hz, 2H), 4.17 (br, 1H), 3.17 – 3.12 (m, 2H), 2.67 (t, *J* = 5.9 Hz, 2H), 2.31 (s, 6H), 1.59 – 1.54 (m, 2H), 1.48 –

1.41 (m, 2H), 0.93 (t, J = 7.3 Hz, 3H); ^{13}C NMR (100 MHz, $CDCl_3$) δ 166.9, 152.3, 131.7, 117.9, 111.3, 62.4, 58.0, 45.9, 43.1, 31.4, 20.3, 13.9; HRMS (ESI) m/z calc. for $C_{11}H_{16}N_2O_2$. $[M+H]^+$: 208.1206, found: 208.1210.

15. Copies of ^{1}H NMR, ^{13}C NMR and ^{19}F NMR spectra of products

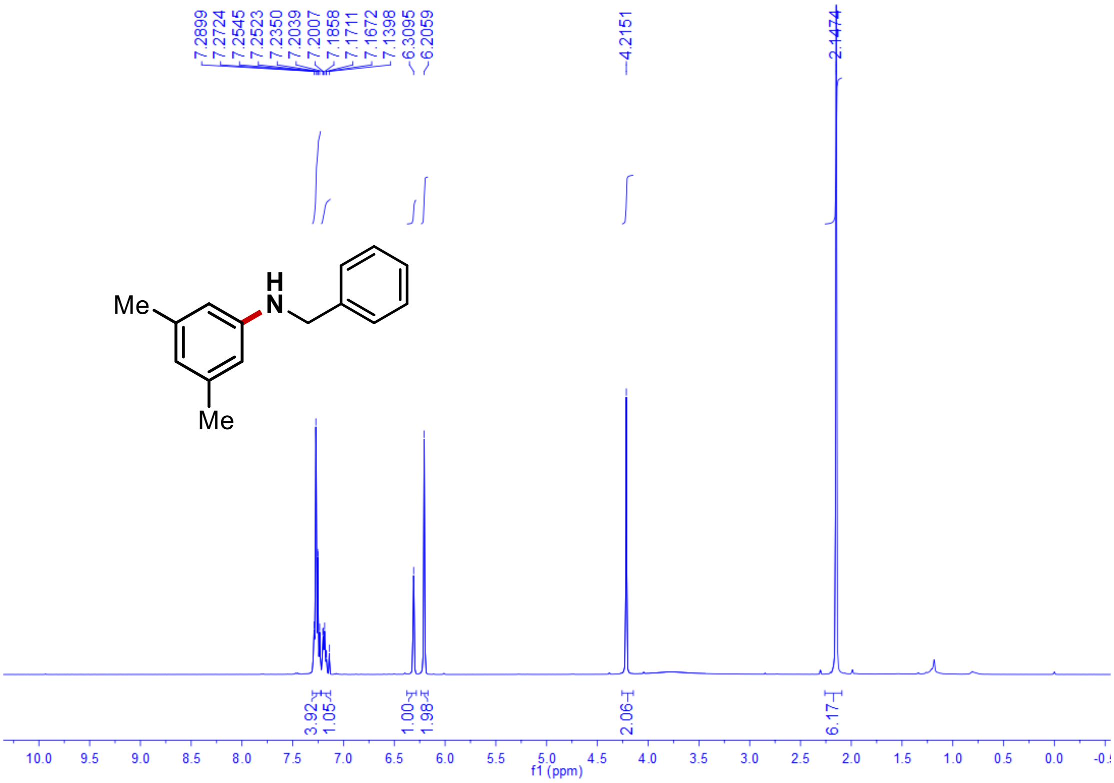

^{1}H NMR (400 MHz, $CDCl_3$) Spectrum

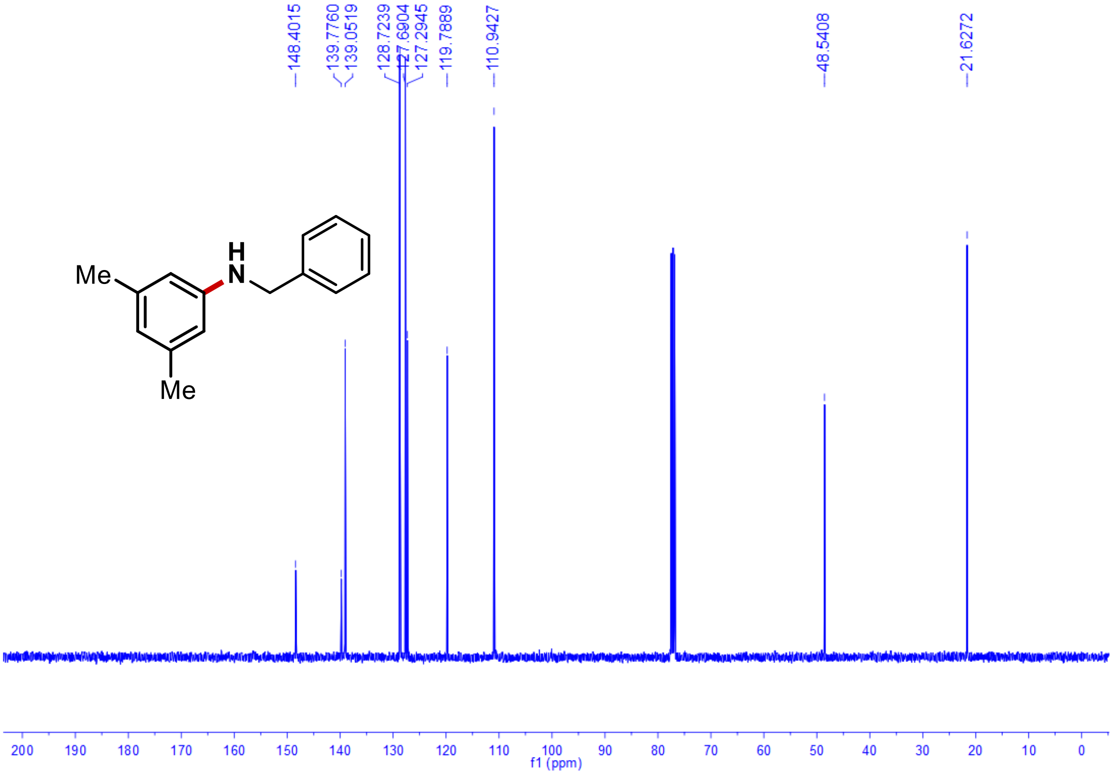

^{13}C NMR (100 MHz, $CDCl_3$) Spectrum

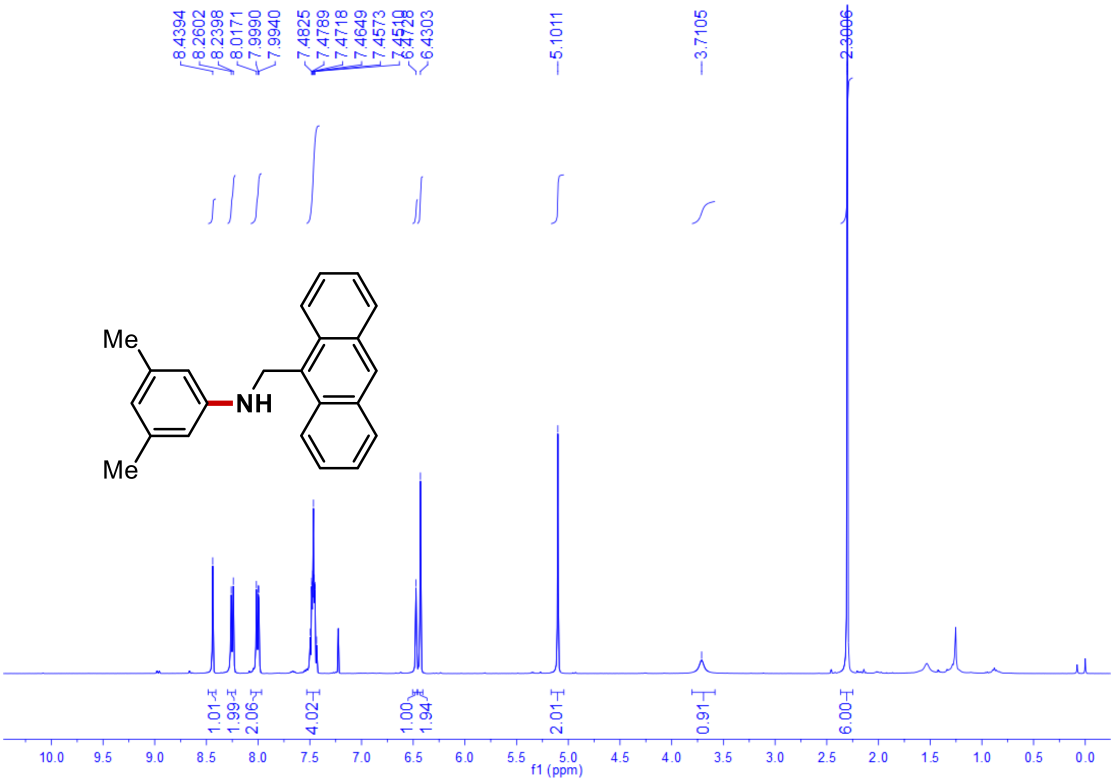

^{1}**H NMR** (400 MHz, $CDCl_3$) Spectrum

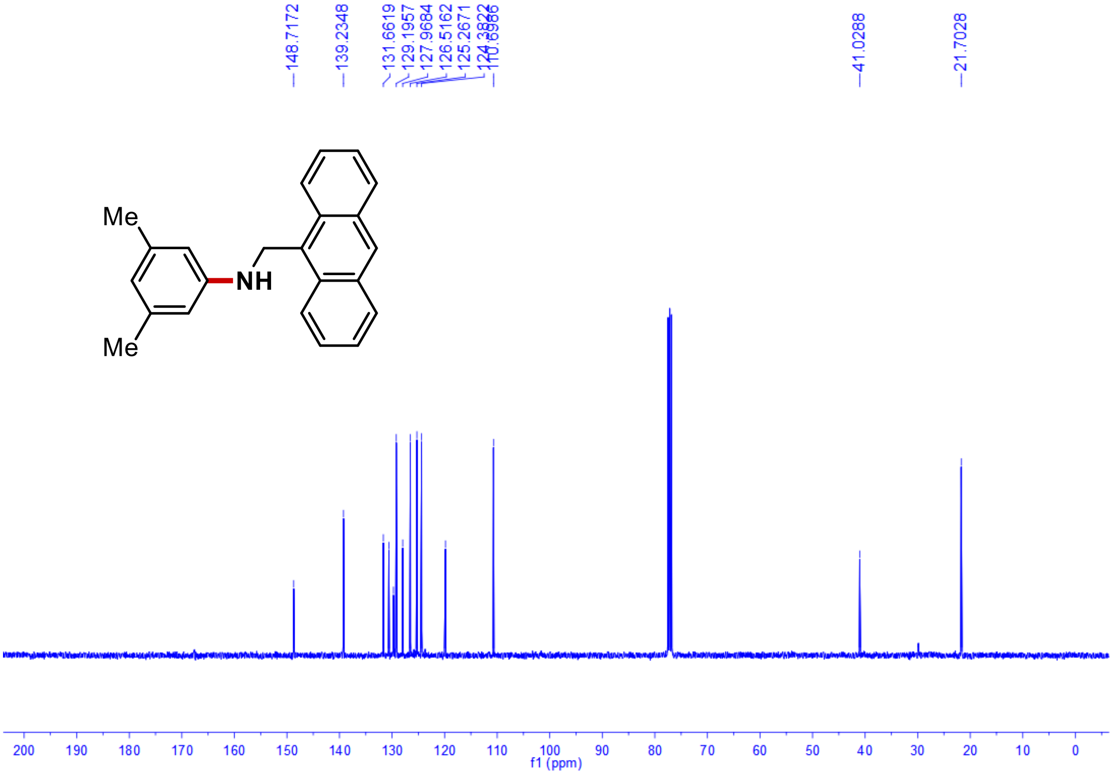

^{13}**C NMR** (100 MHz, $CDCl_3$) Spectrum

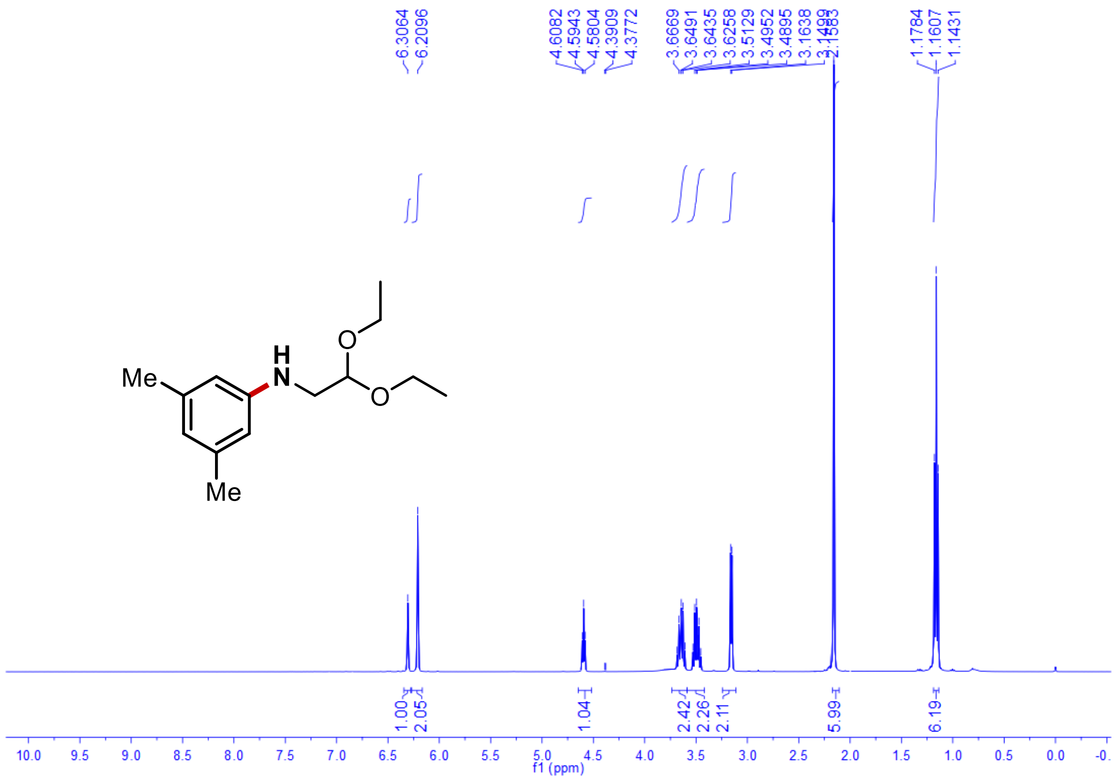

^{1}**H NMR** (400 MHz, $CDCl_3$) Spectrum

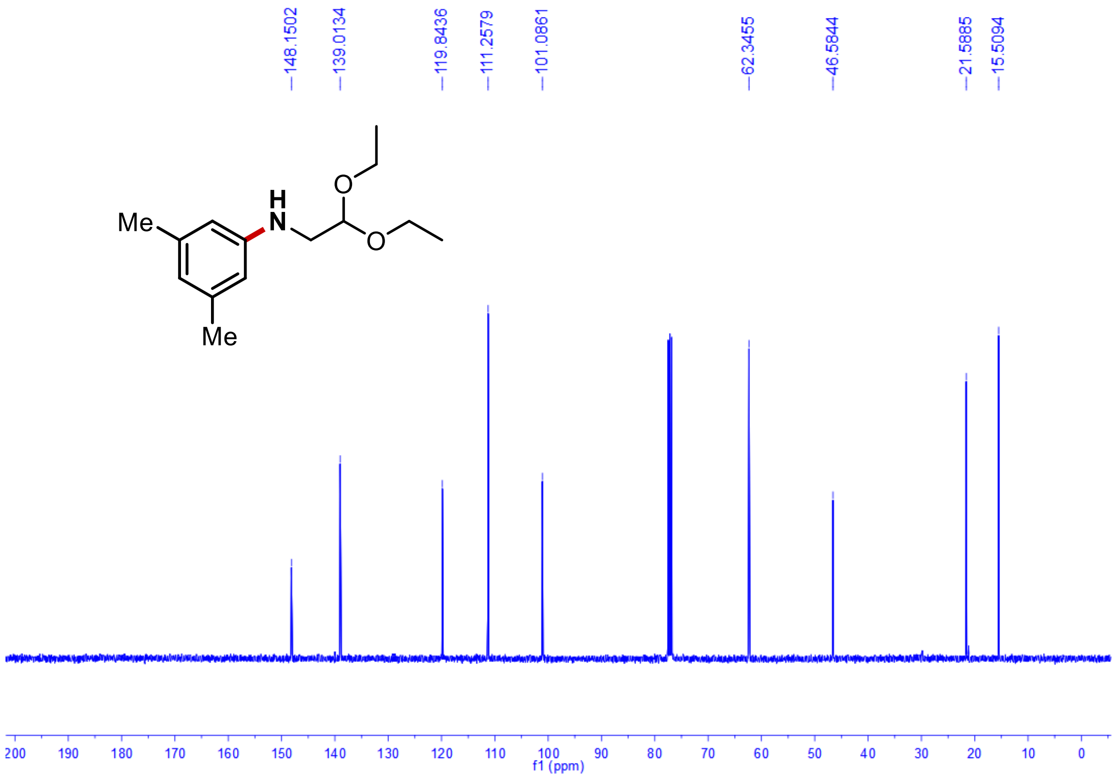

^{13}**C NMR** (100 MHz, $CDCl_3$) Spectrum

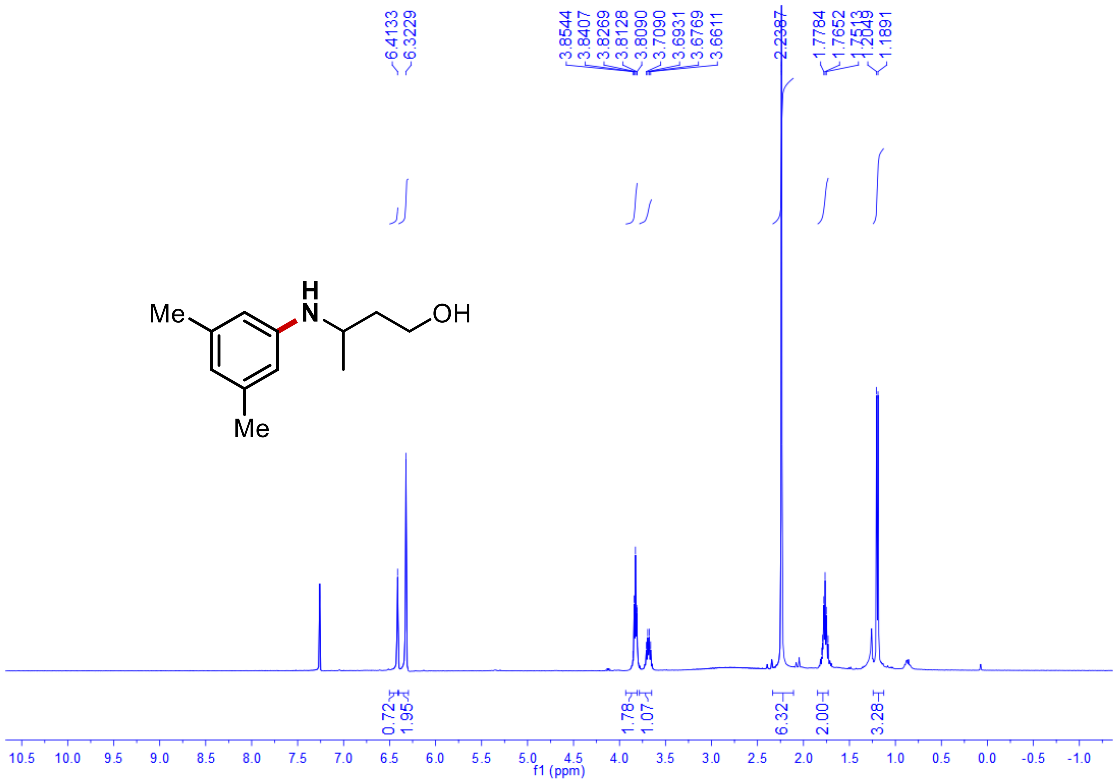

^{1}**H NMR** (400 MHz, CDCl$_3$) Spectrum

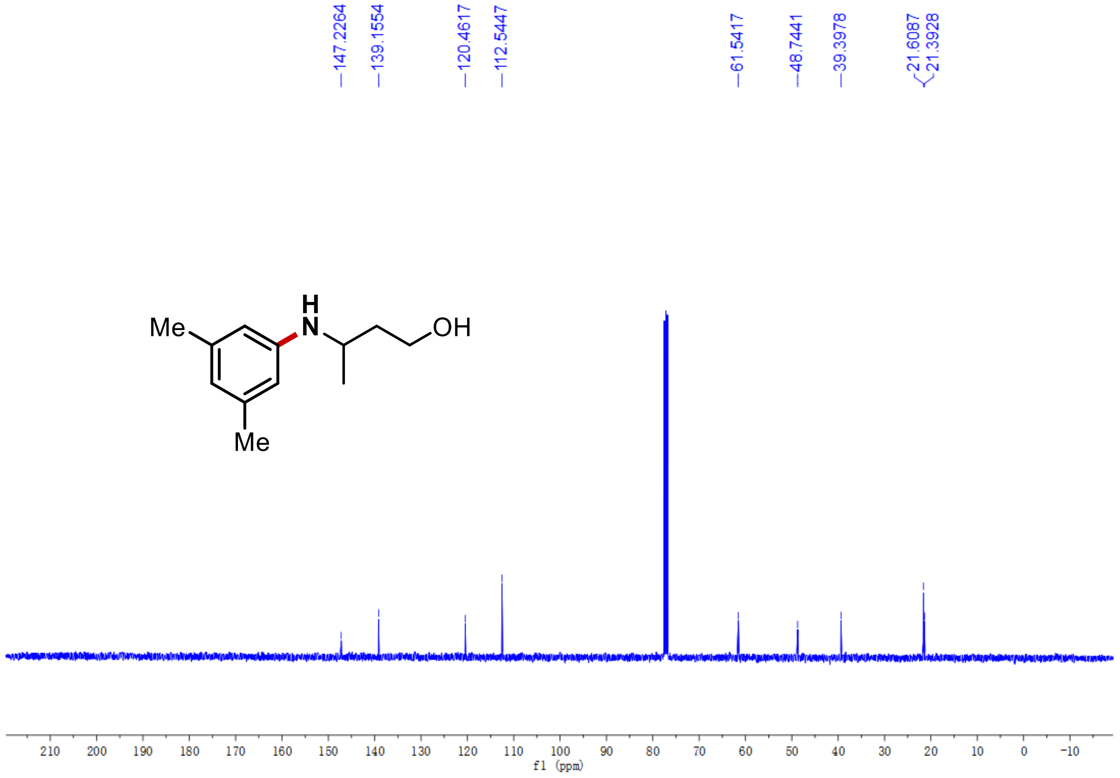

^{13}**C NMR** (100 MHz, CDCl$_3$) Spectrum

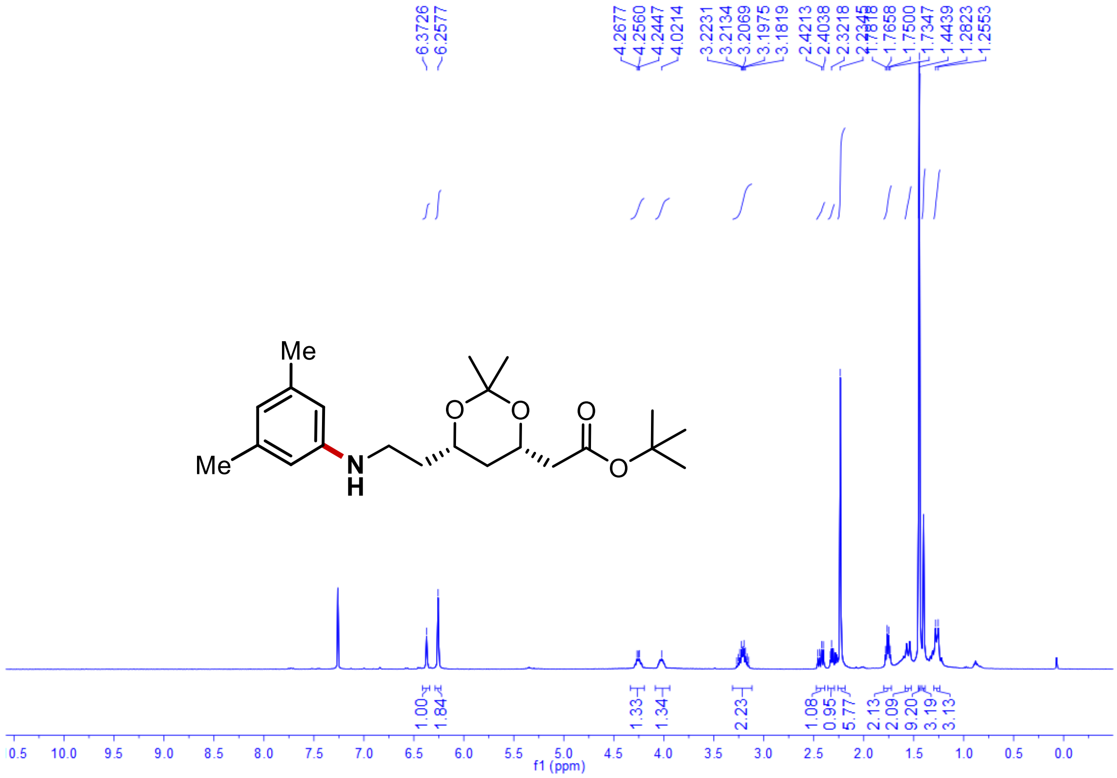

^{1}H NMR (400 MHz, $CDCl_3$) Spectrum

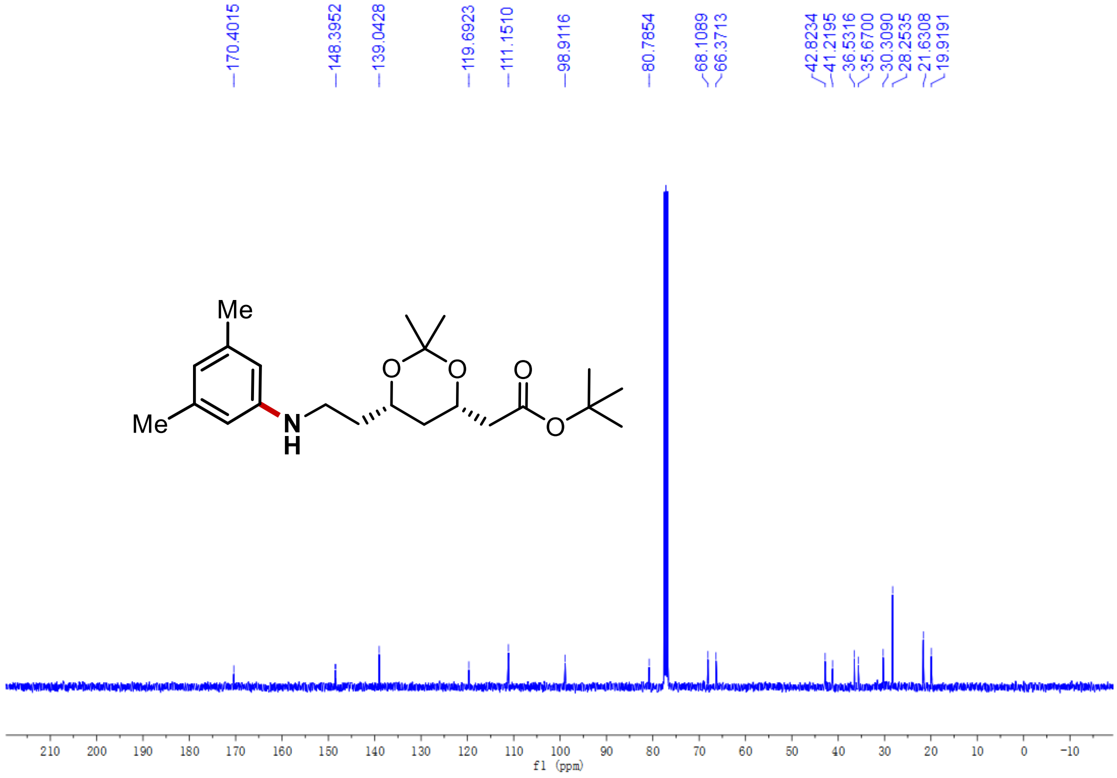

^{13}C NMR (100 MHz, $CDCl_3$) Spectrum

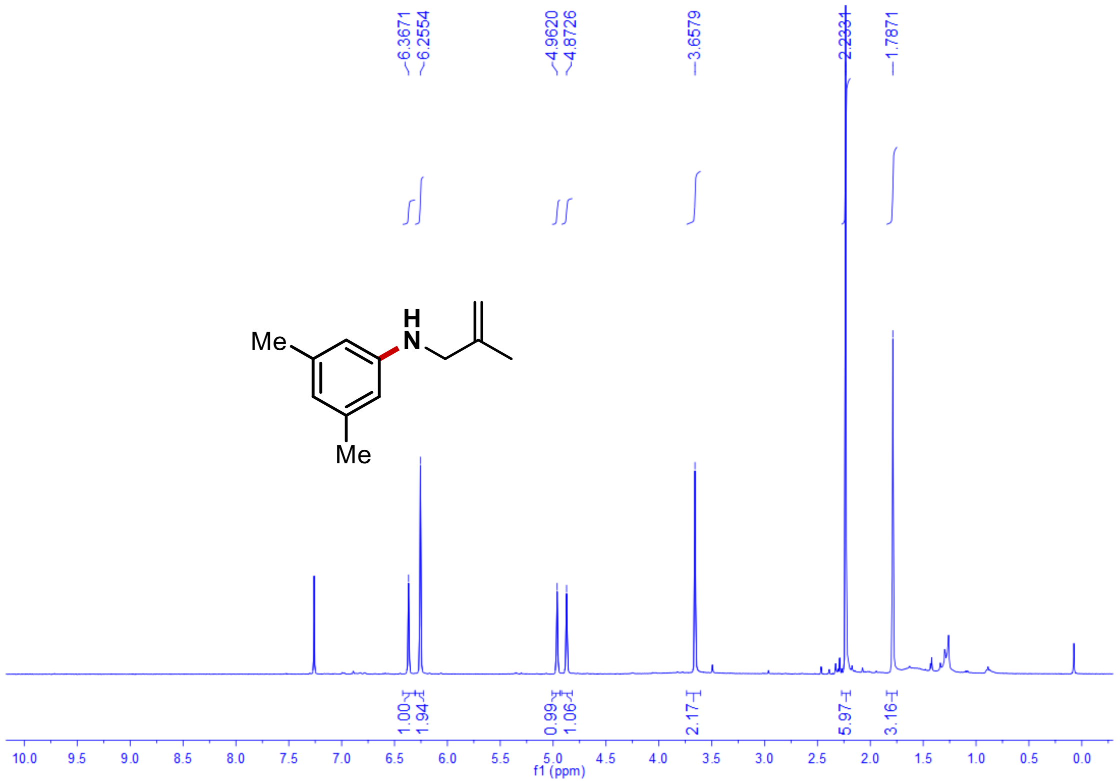

^{1}H NMR (400 MHz, $CDCl_3$) Spectrum

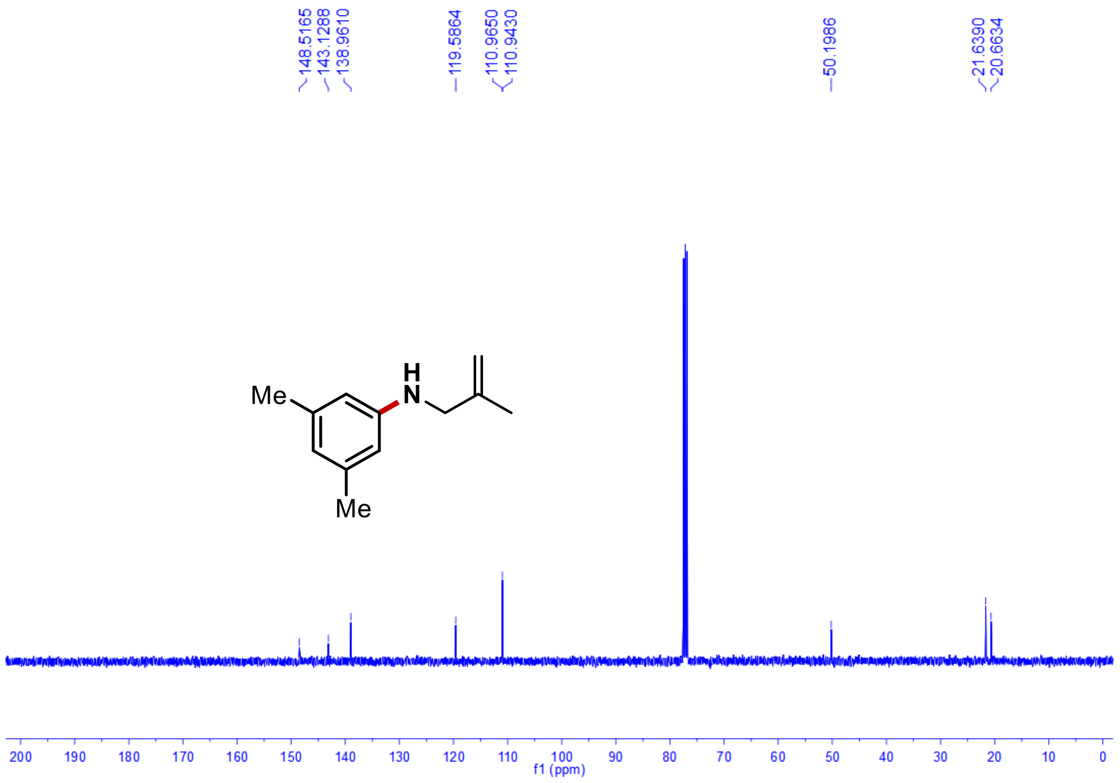

^{13}C NMR (100 MHz, $CDCl_3$) Spectrum

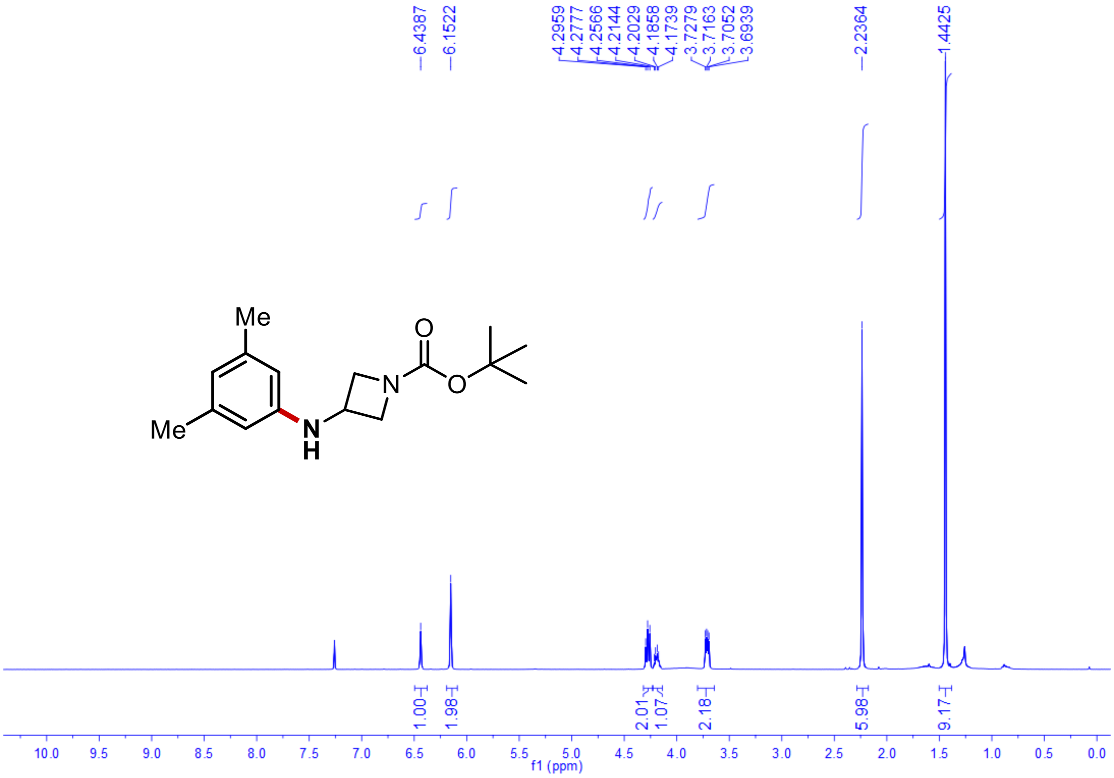

¹H NMR (400 MHz, $CDCl_3$) Spectrum

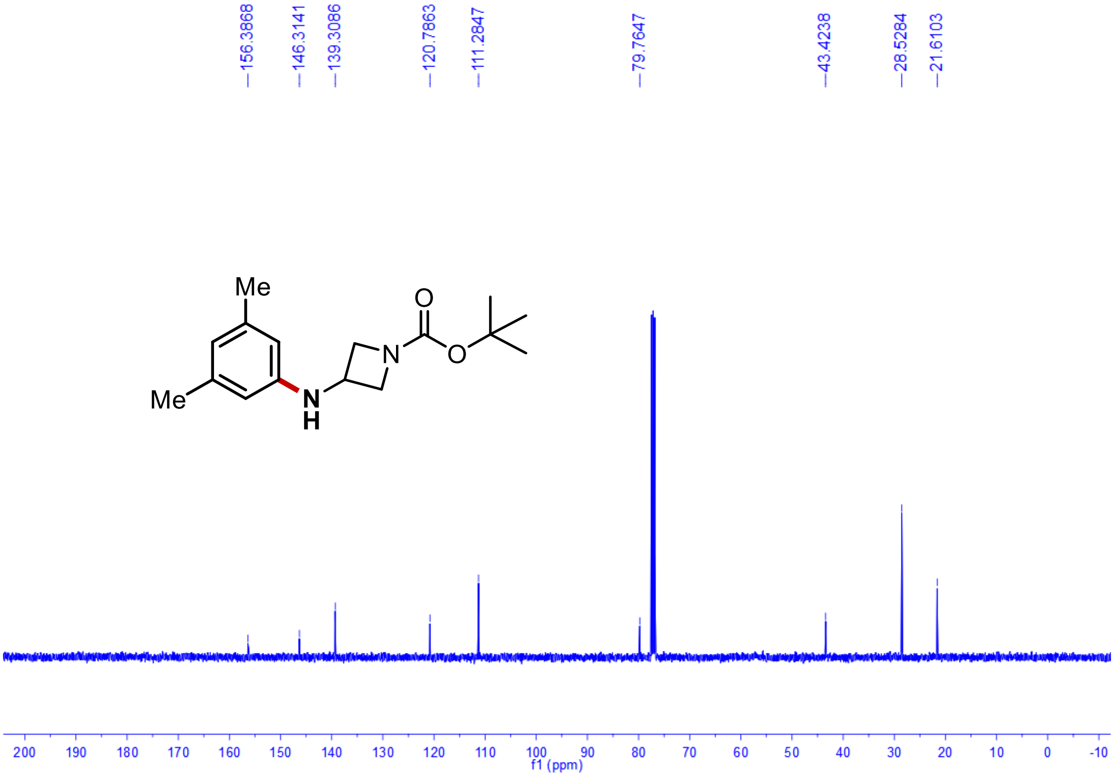

¹³C NMR (100 MHz, $CDCl_3$) Spectrum

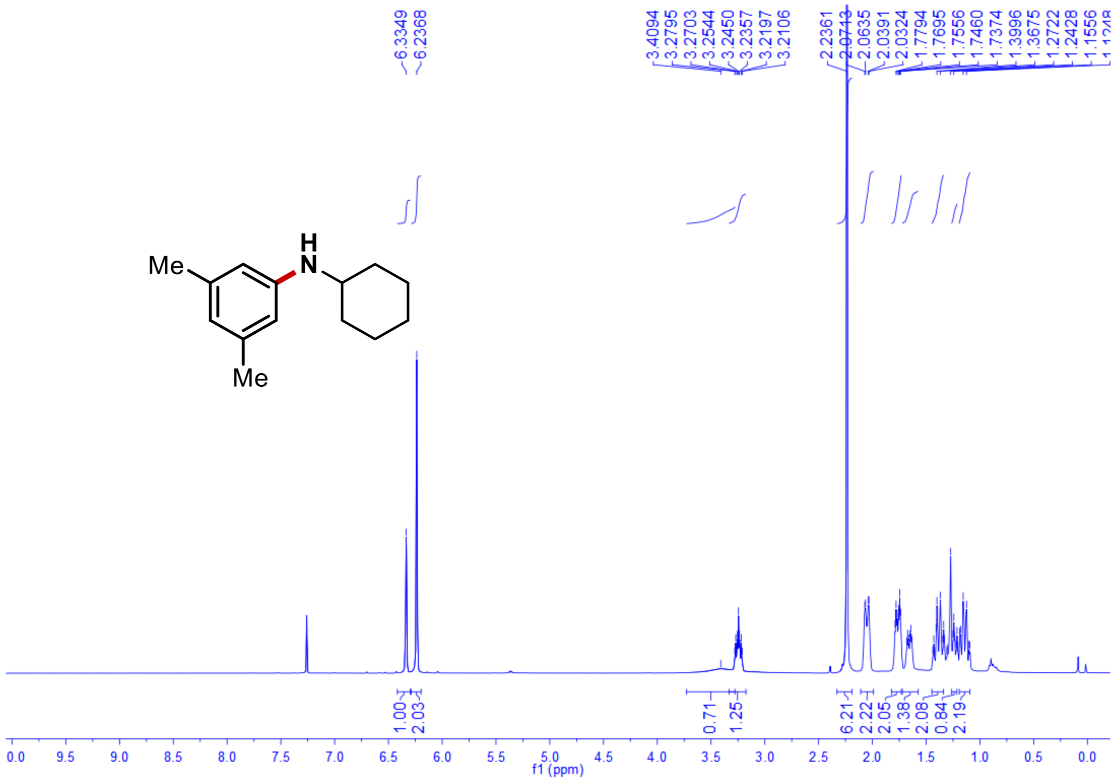

^{1}**H NMR** (400 MHz, $CDCl_3$) Spectrum

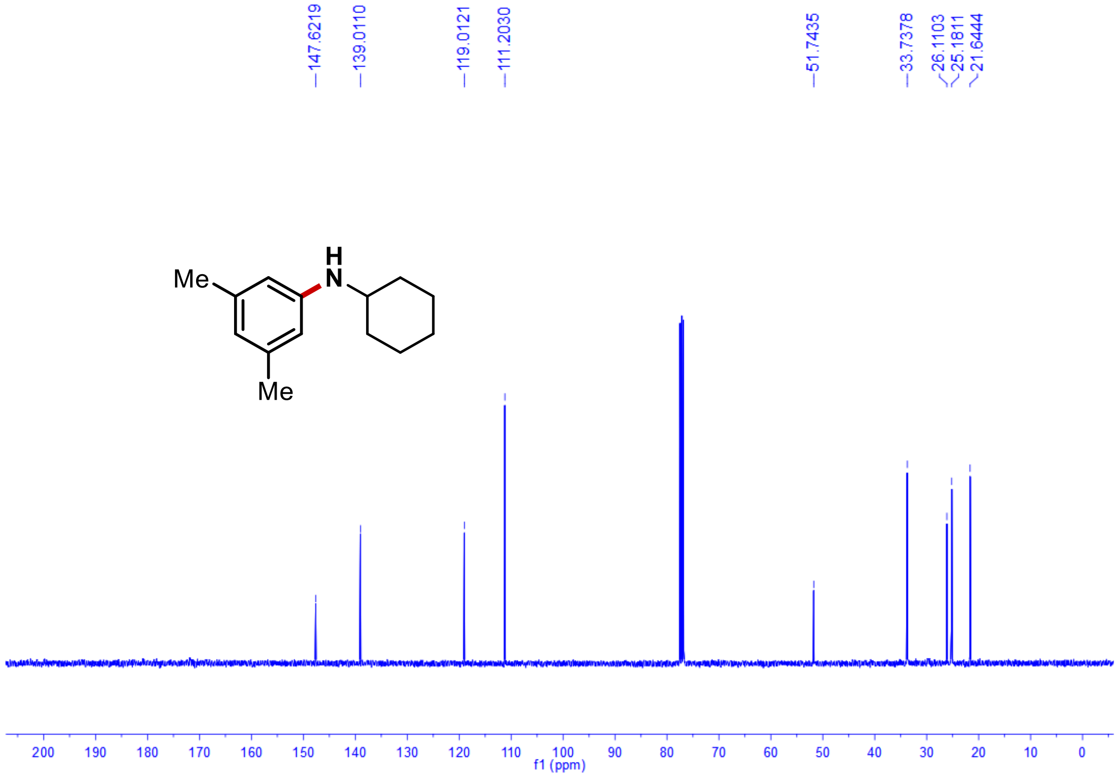

^{13}**C NMR** (100 MHz, $CDCl_3$) Spectrum

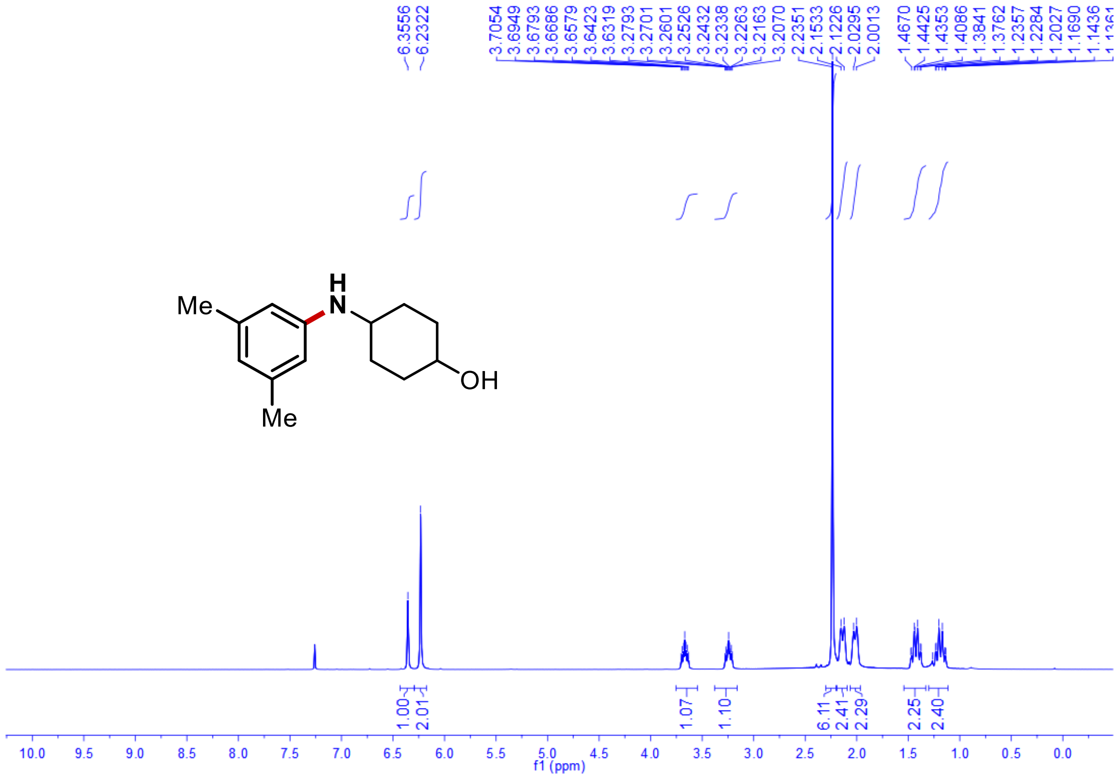

^{1}H NMR (400 MHz, $CDCl_3$) Spectrum

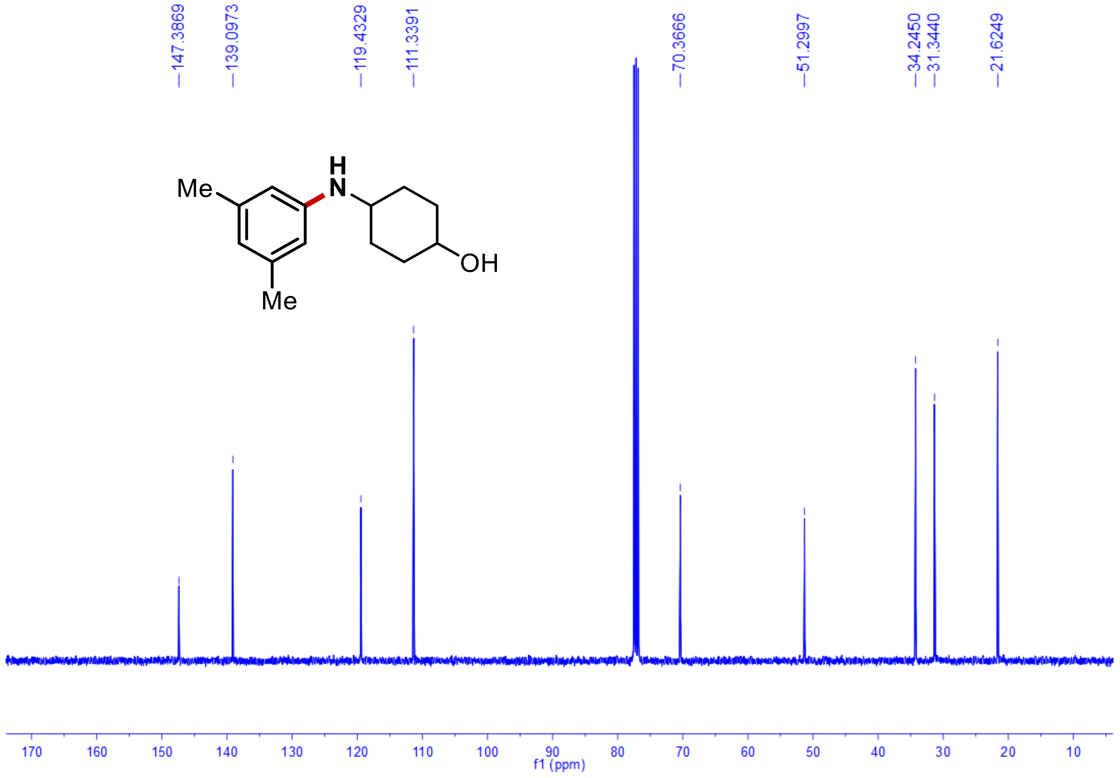

^{13}C NMR (100 MHz, $CDCl_3$) Spectrum

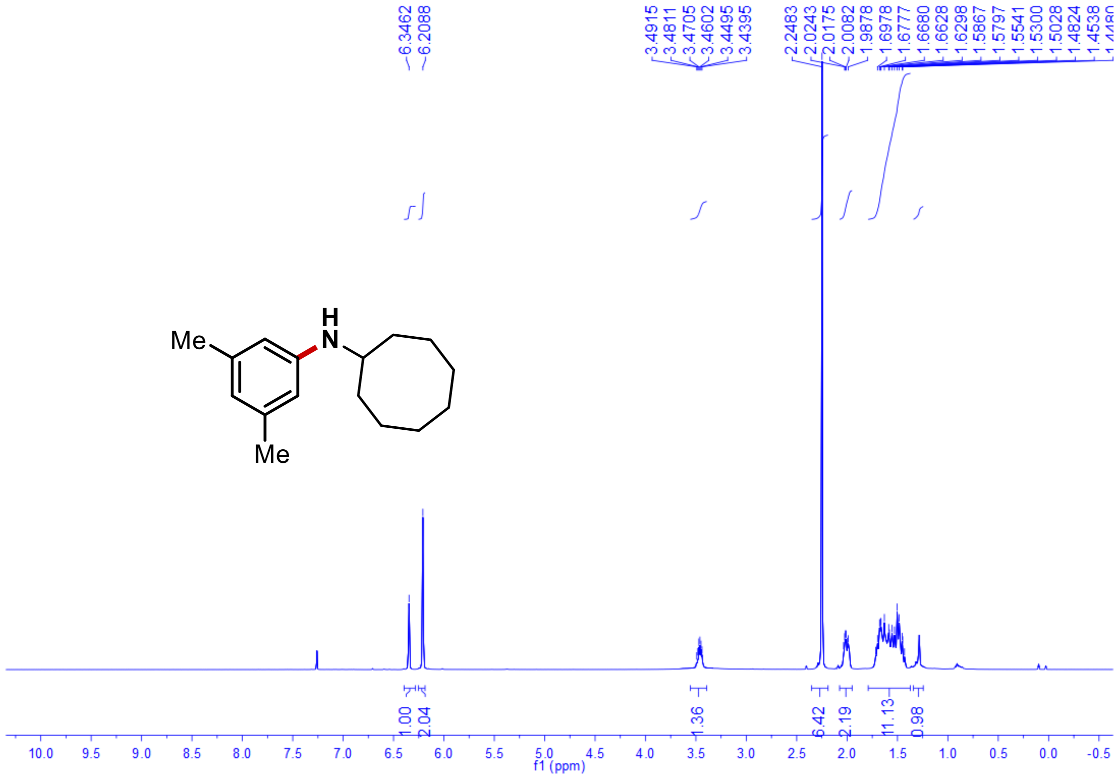

^{1}H NMR (400 MHz, $CDCl_3$) Spectrum

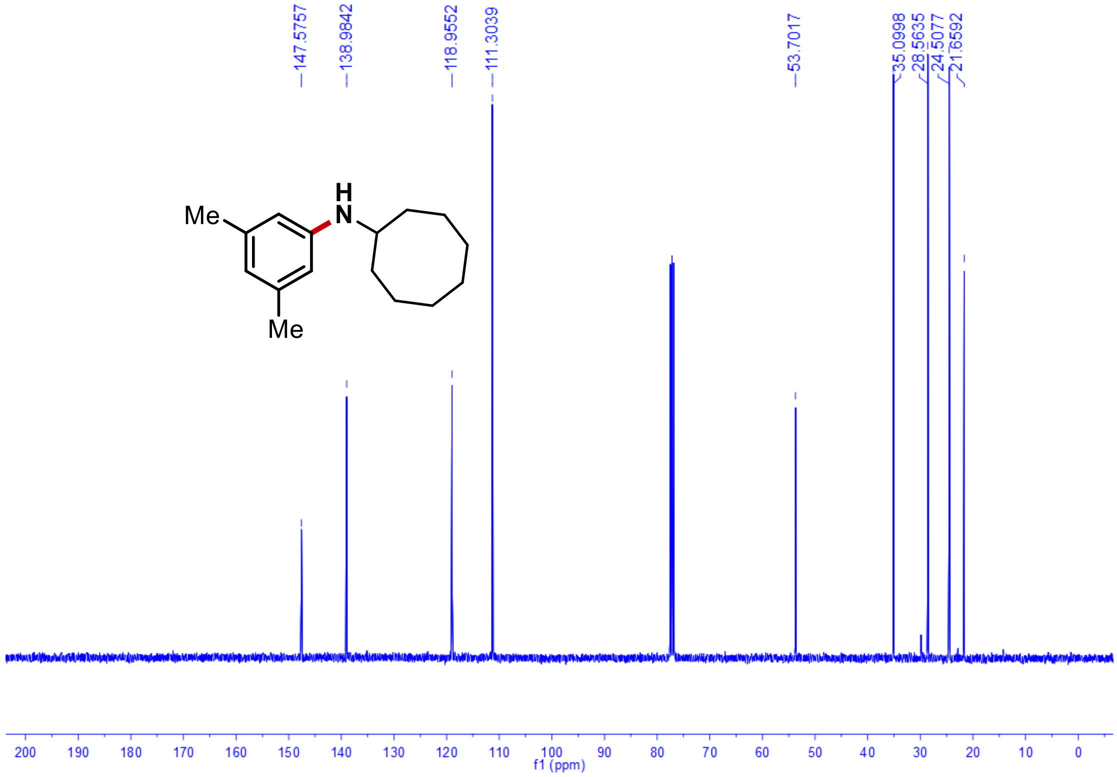

^{13}C NMR (100 MHz, $CDCl_3$) Spectrum

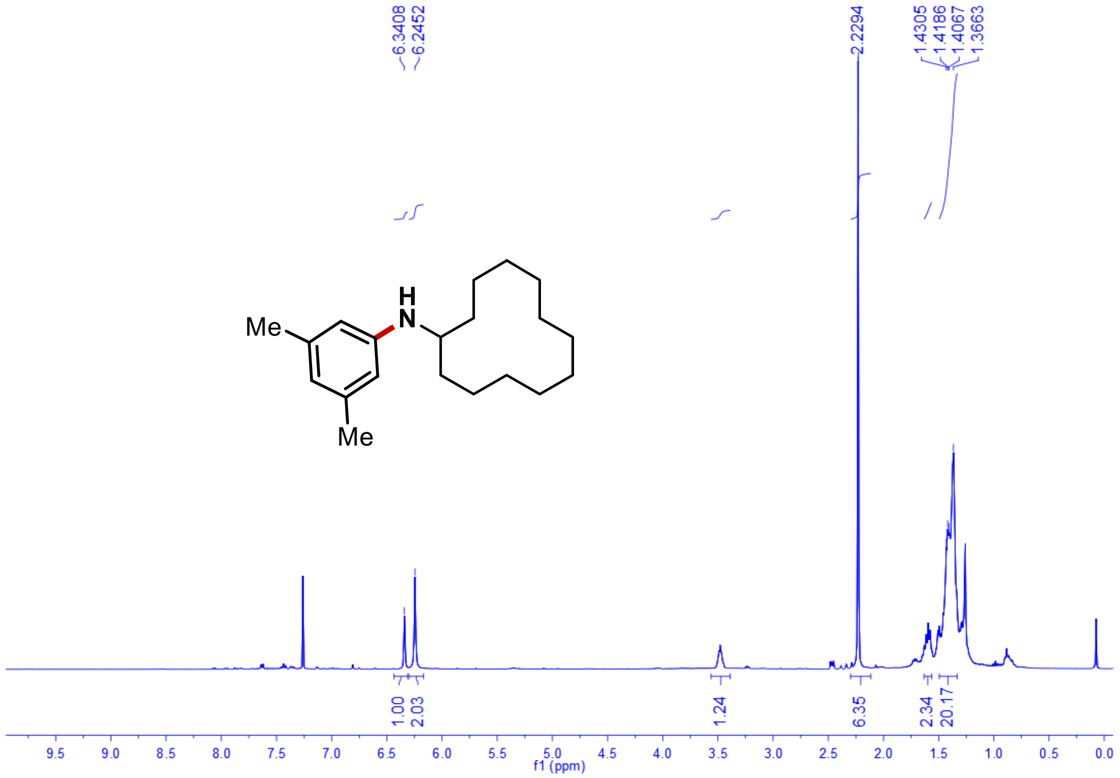

^{1}H NMR (400 MHz, $CDCl_3$) Spectrum

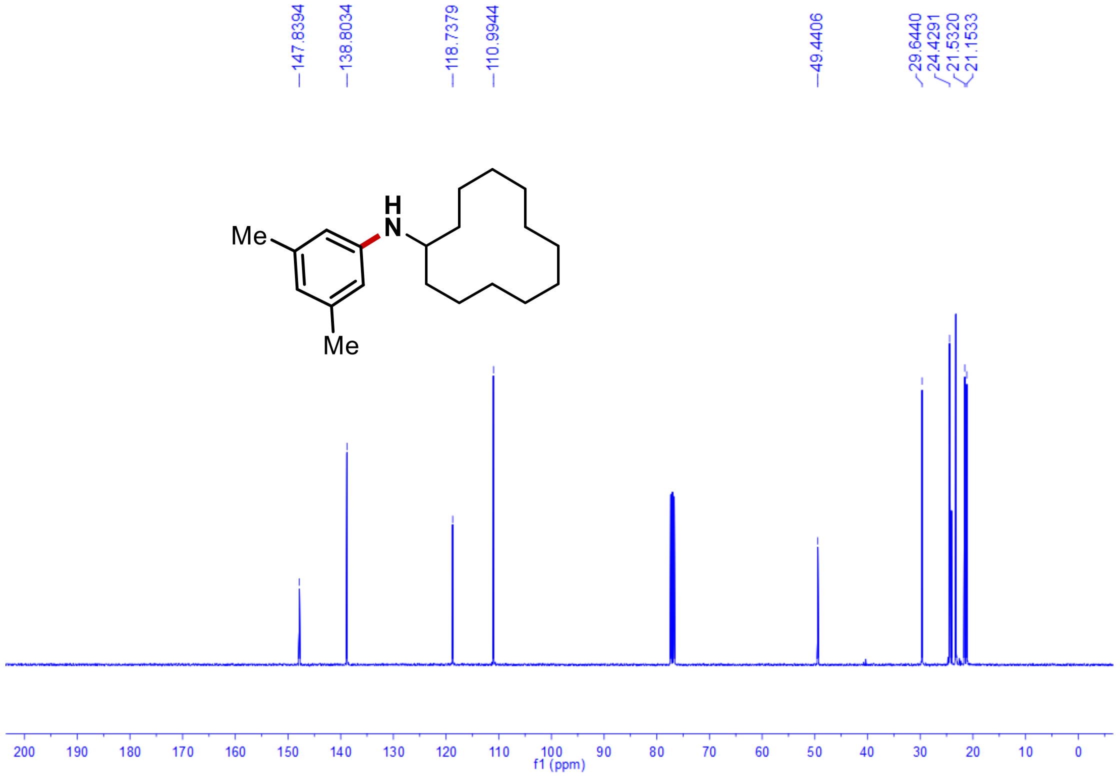

^{13}C NMR (100 MHz, $CDCl_3$) Spectrum

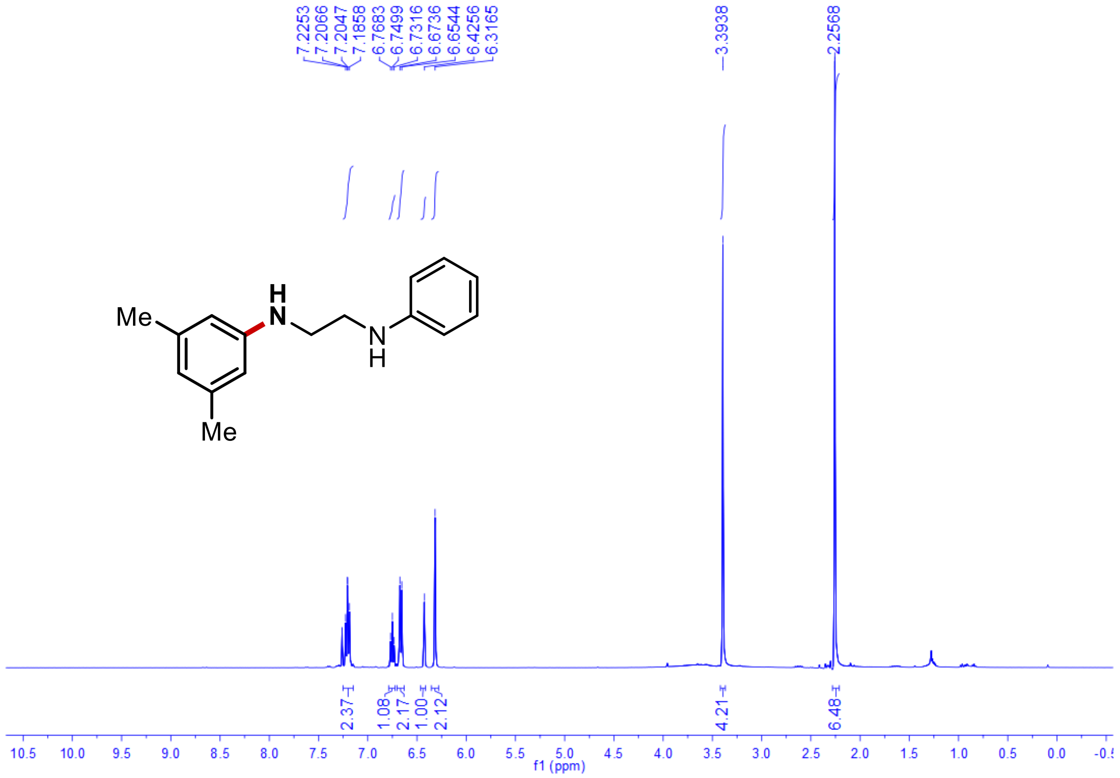

^{1}H NMR (400 MHz, $CDCl_3$) Spectrum

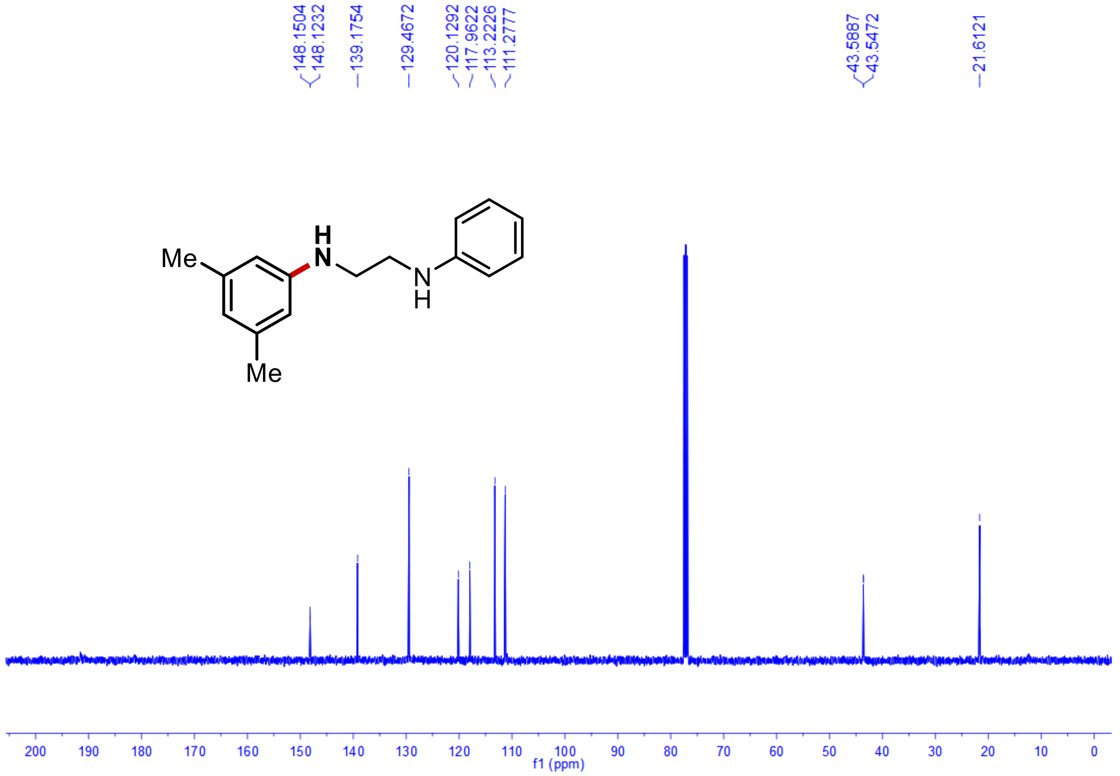

^{13}C NMR (100 MHz, $CDCl_3$) Spectrum

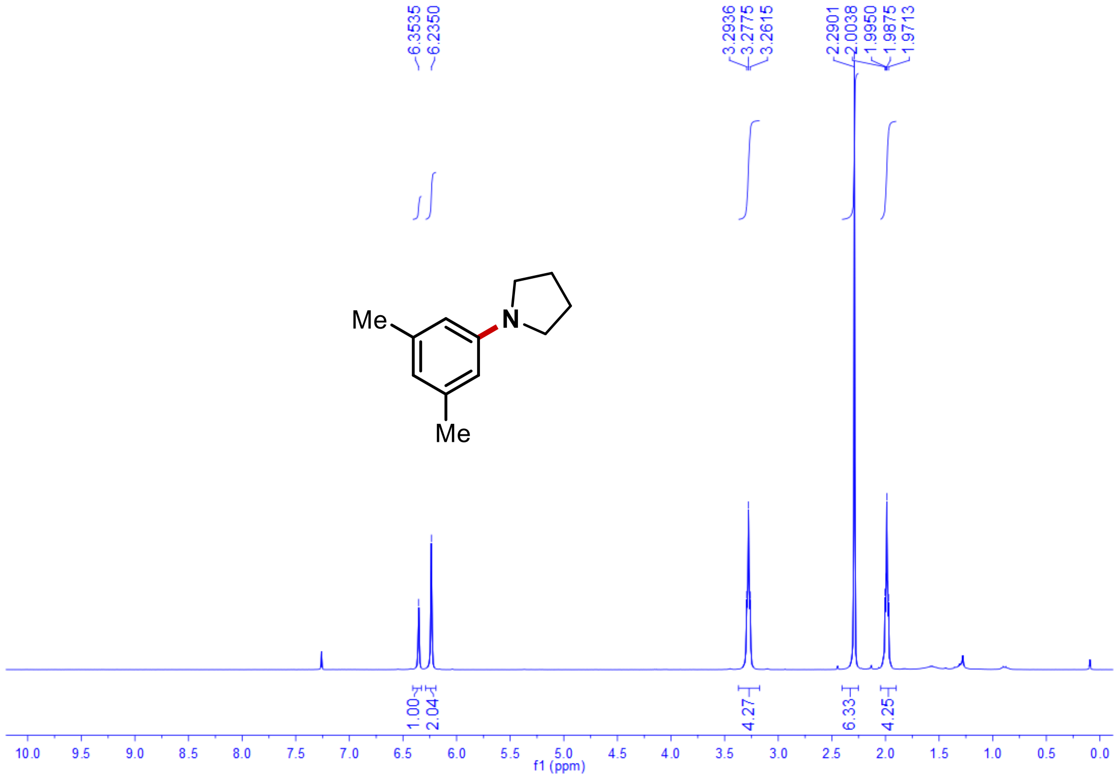

^{1}H NMR (400 MHz, $CDCl_3$) Spectrum

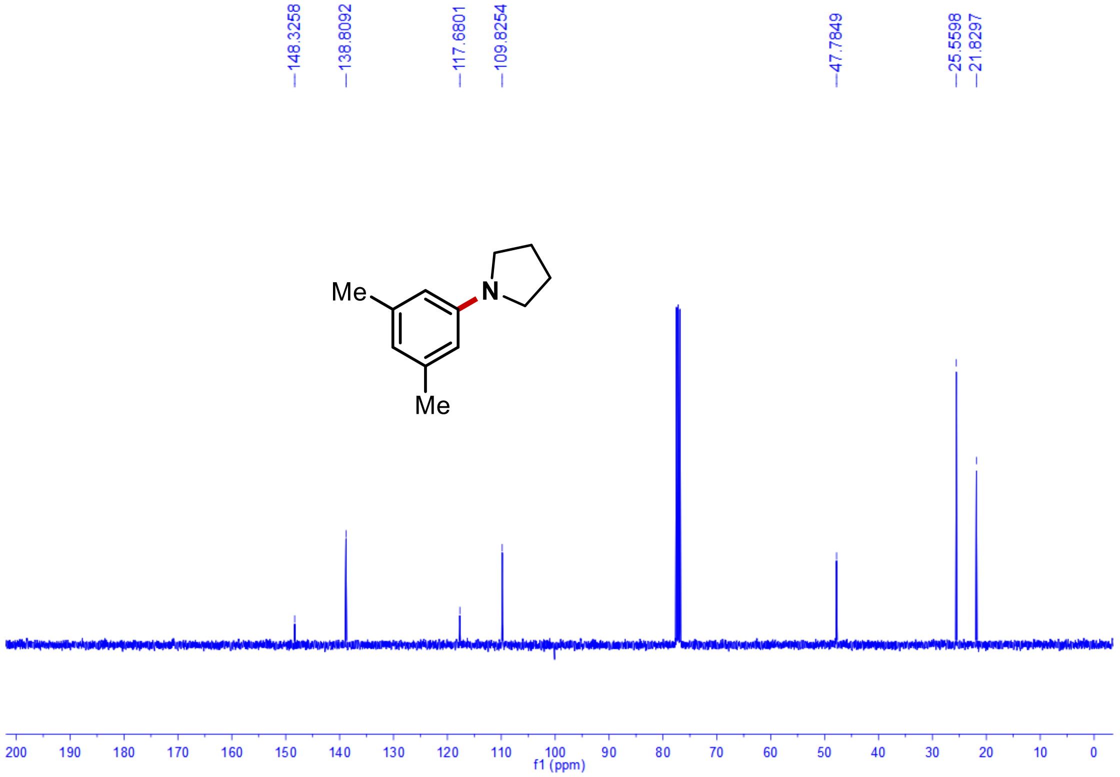

^{13}C NMR (100 MHz, $CDCl_3$) Spectrum

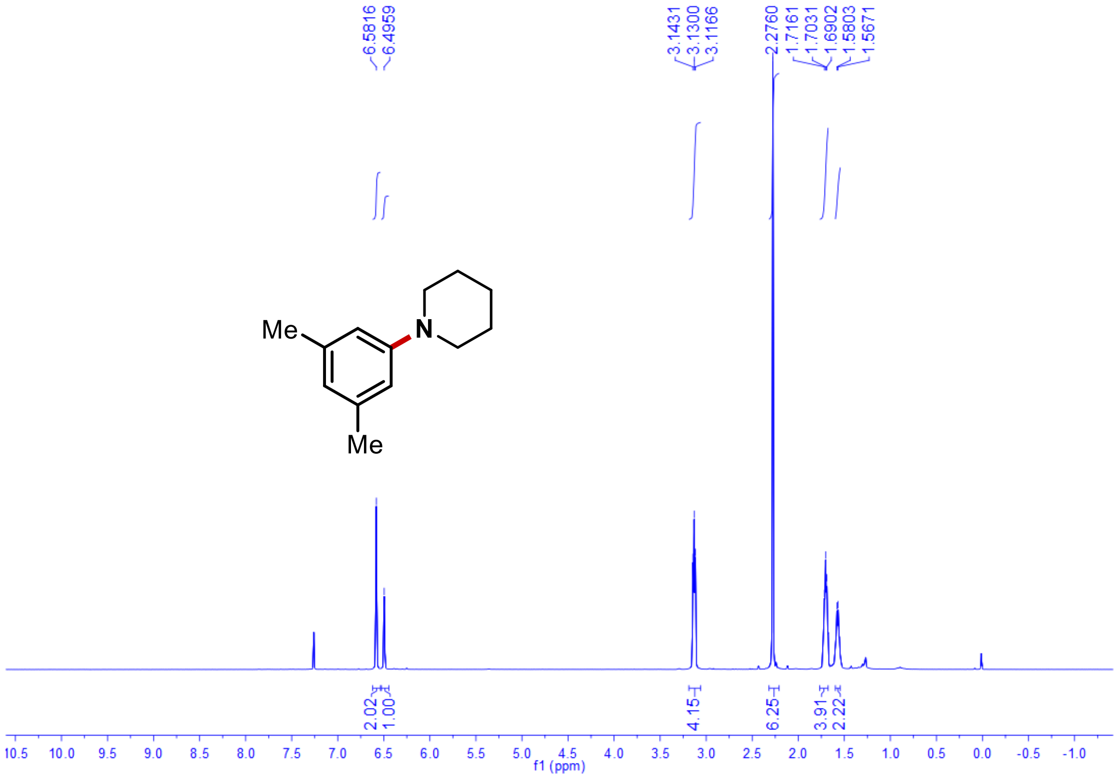

^{1}H NMR (400 MHz, $CDCl_3$) Spectrum

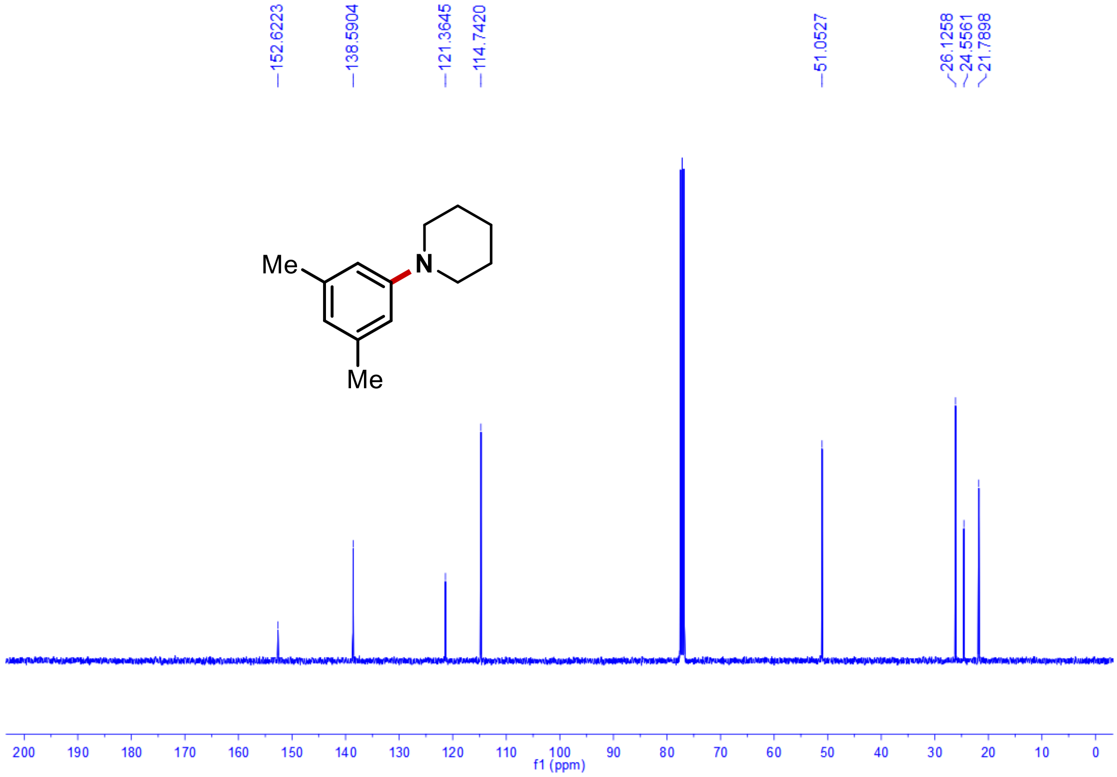

^{13}C NMR (100 MHz, $CDCl_3$) Spectrum

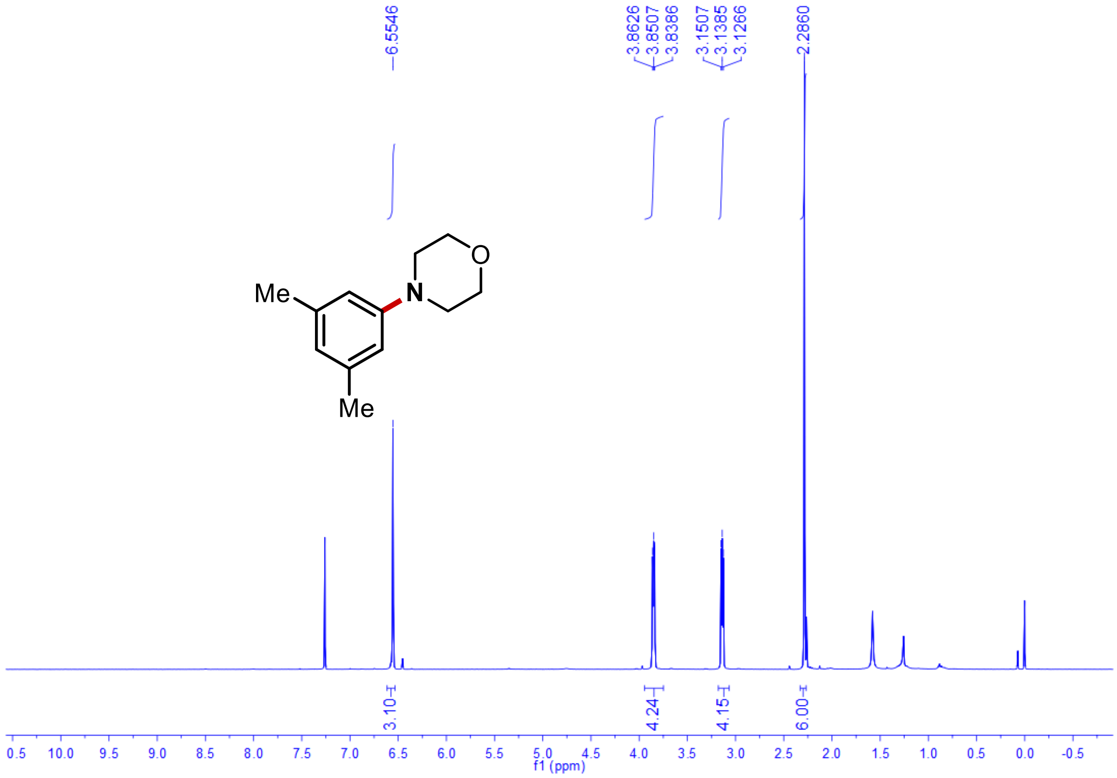

^{1}H NMR (400 MHz, $CDCl_3$) Spectrum

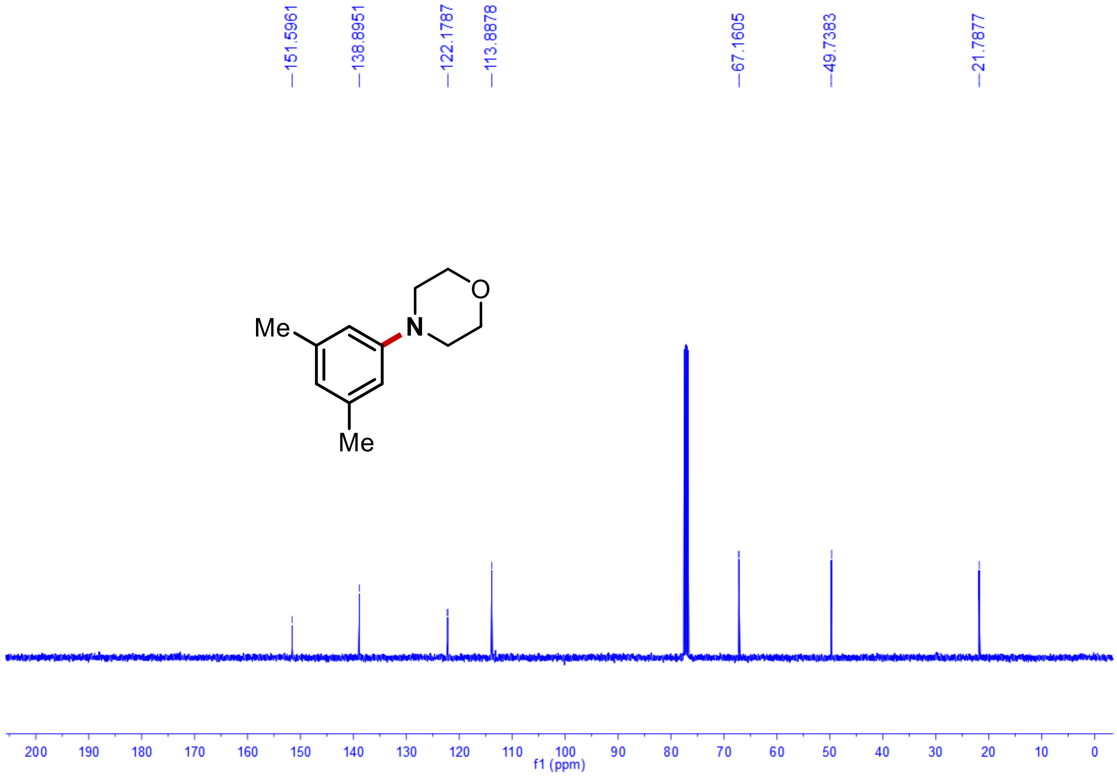

^{13}C NMR (100 MHz, $CDCl_3$) Spectrum

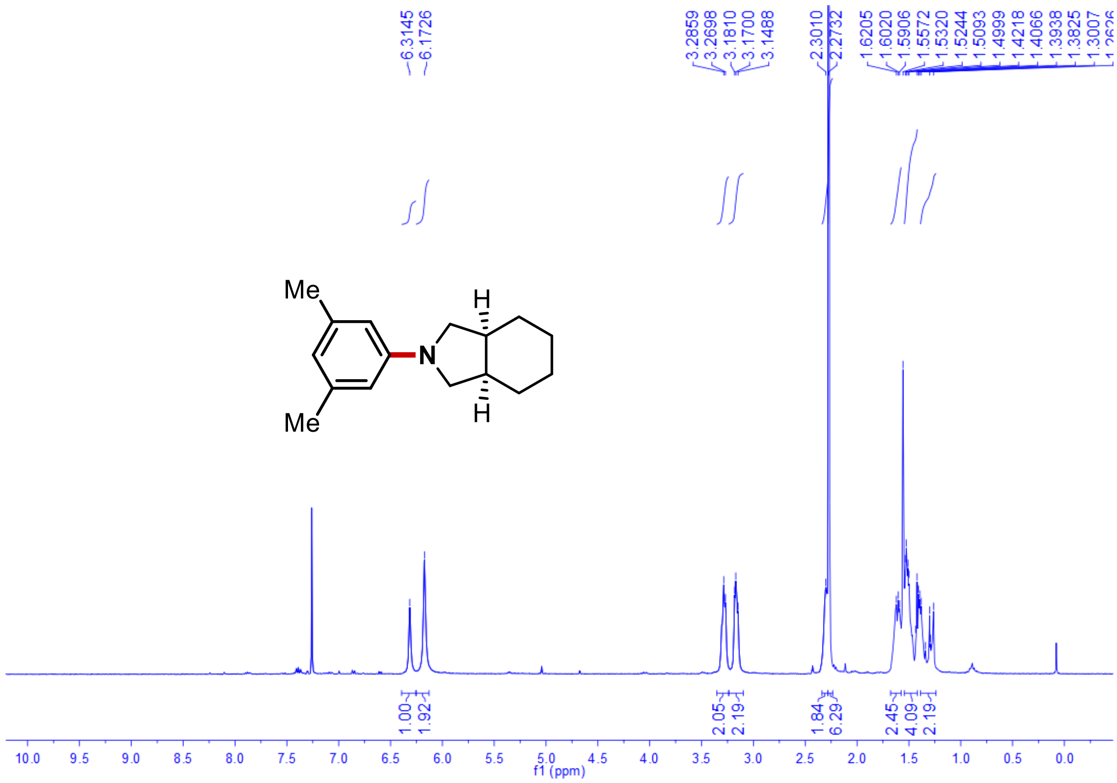

^{1}H NMR (400 MHz, $CDCl_3$) Spectrum

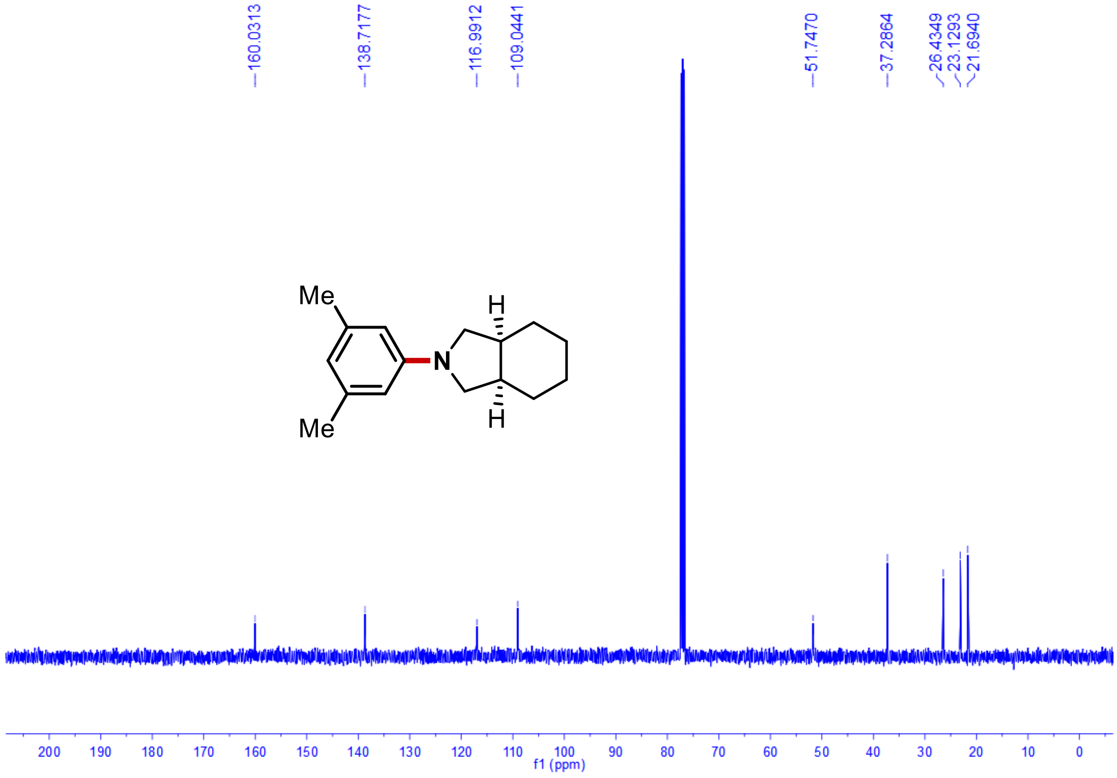

^{13}C NMR (100 MHz, $CDCl_3$) Spectrum

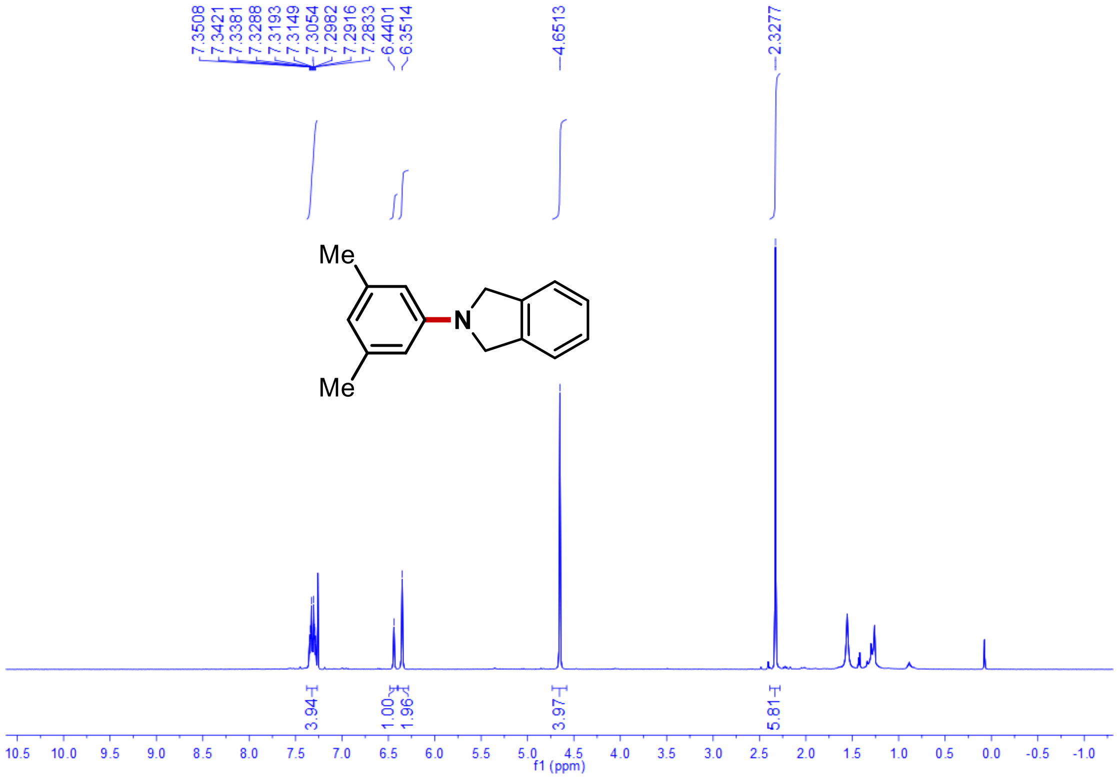

^{1}H NMR (400 MHz, $CDCl_3$) Spectrum

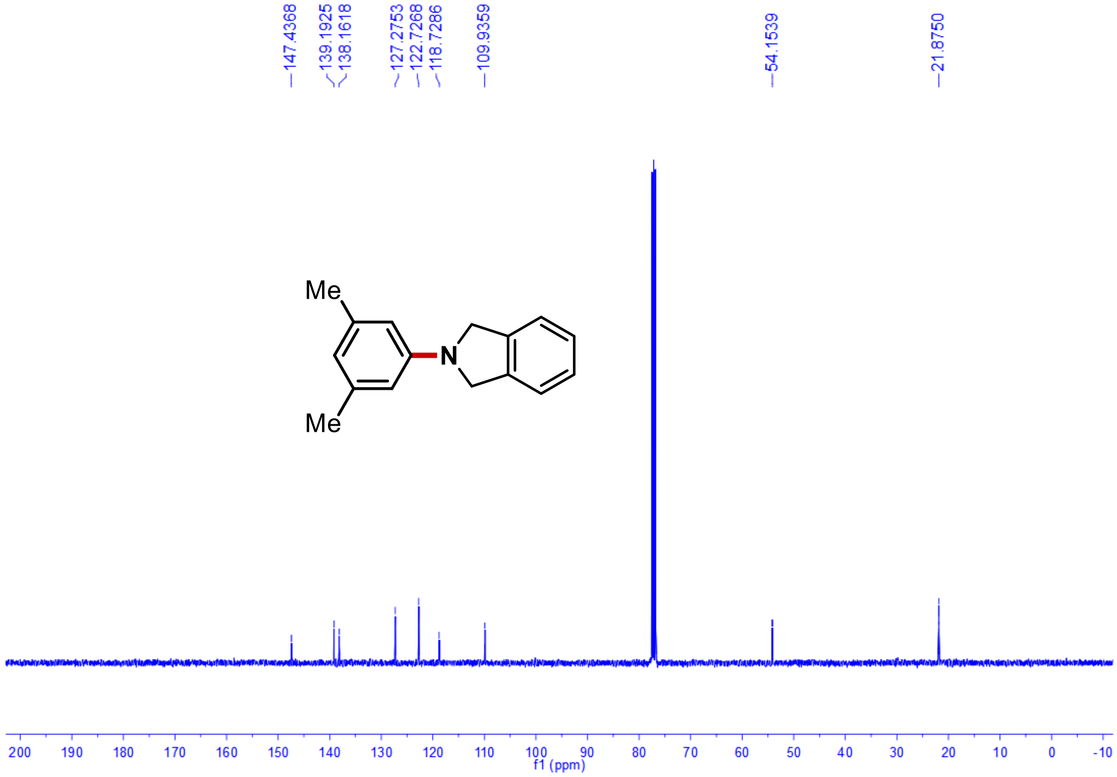

^{13}C NMR (100 MHz, $CDCl_3$) Spectrum

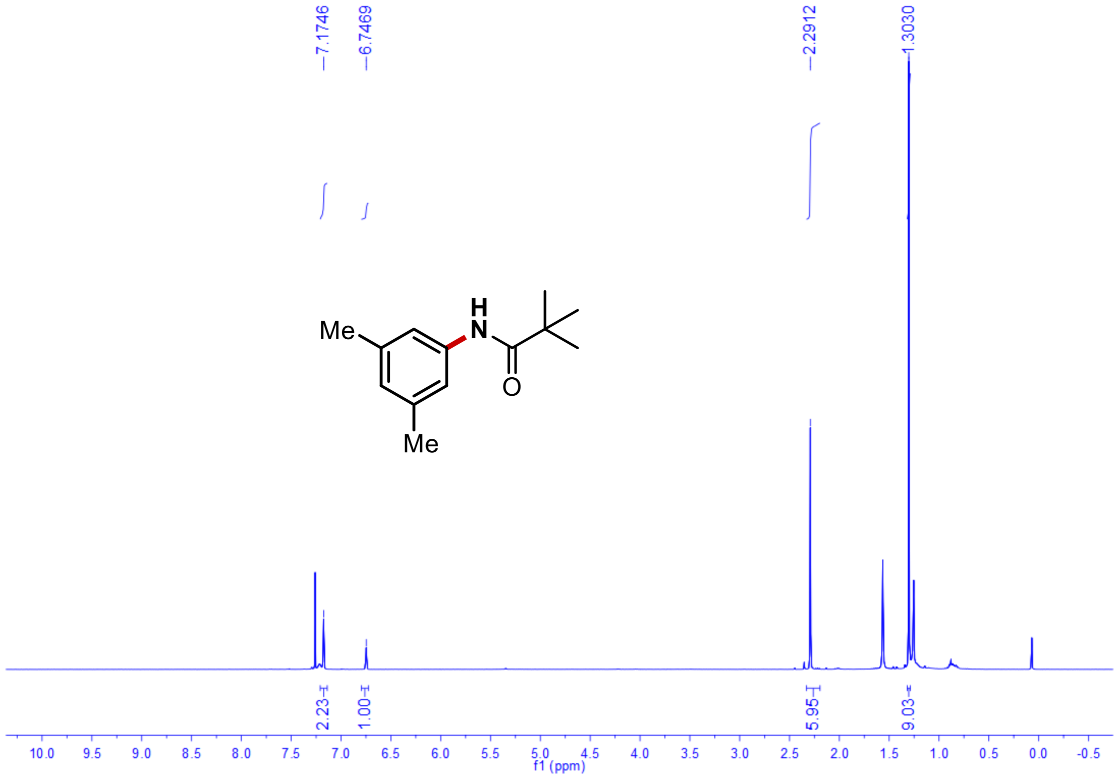

^{1}H NMR (400 MHz, $CDCl_3$) Spectrum

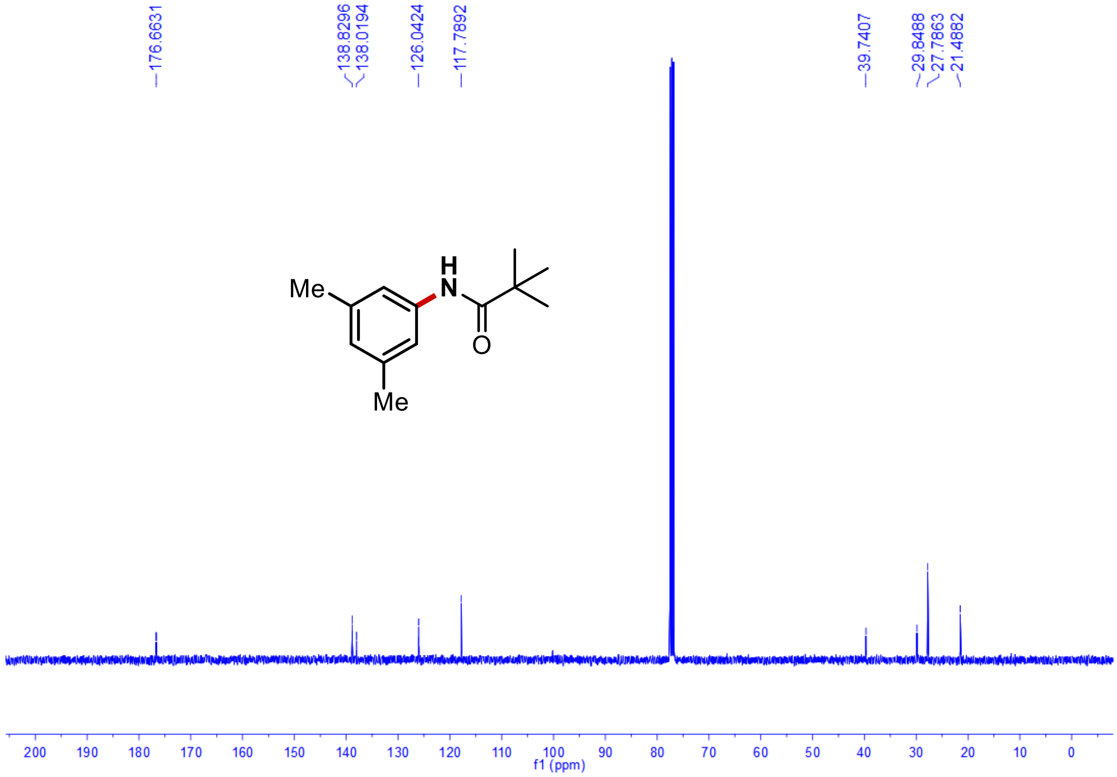

^{13}C NMR (100 MHz, $CDCl_3$) Spectrum

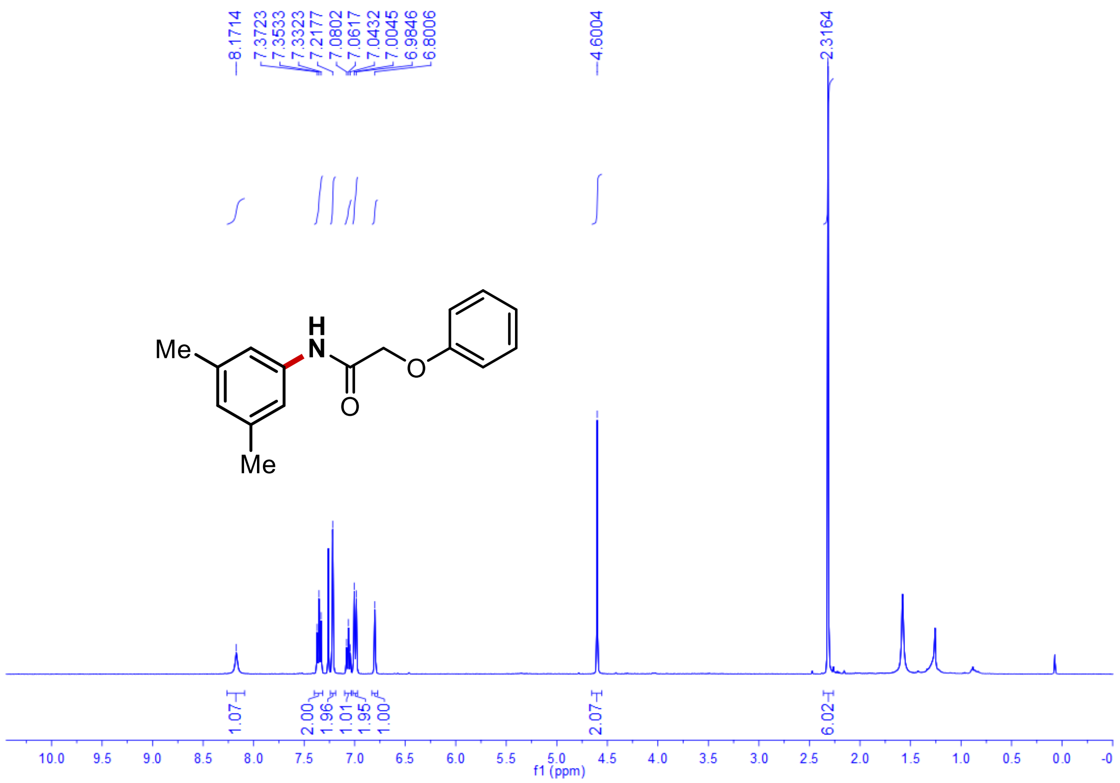

^{1}H NMR (400 MHz, $CDCl_3$) Spectrum

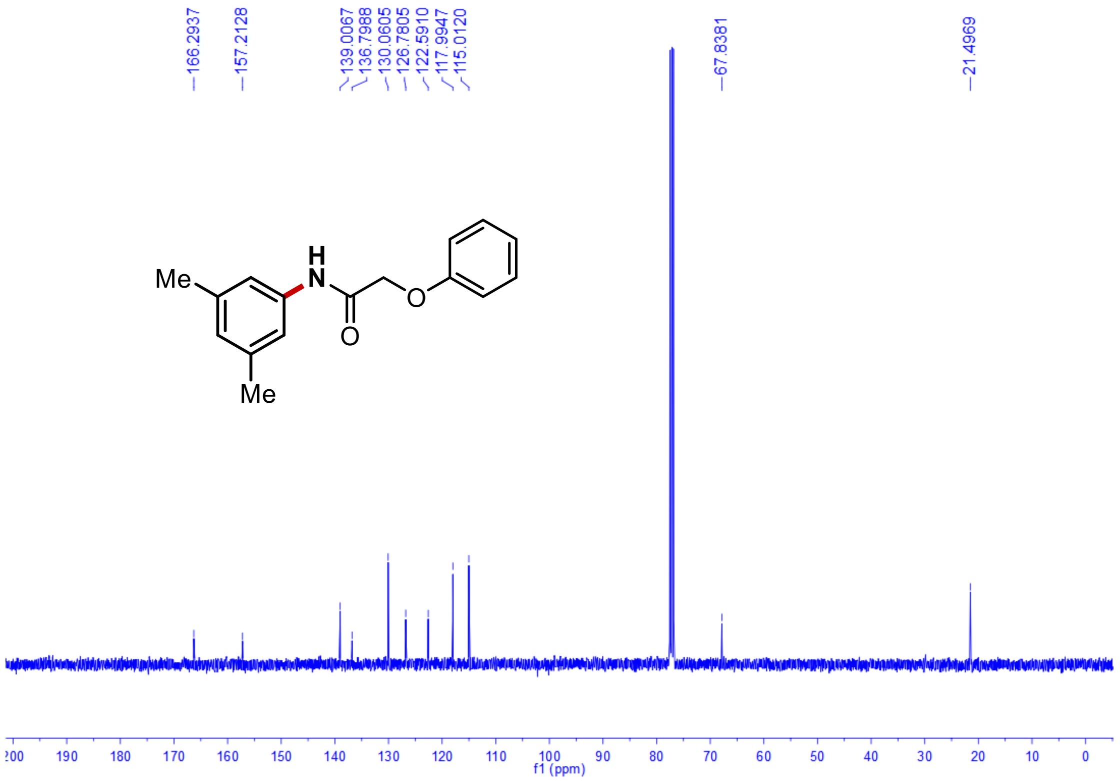

^{13}C NMR (100 MHz, $CDCl_3$) Spectrum

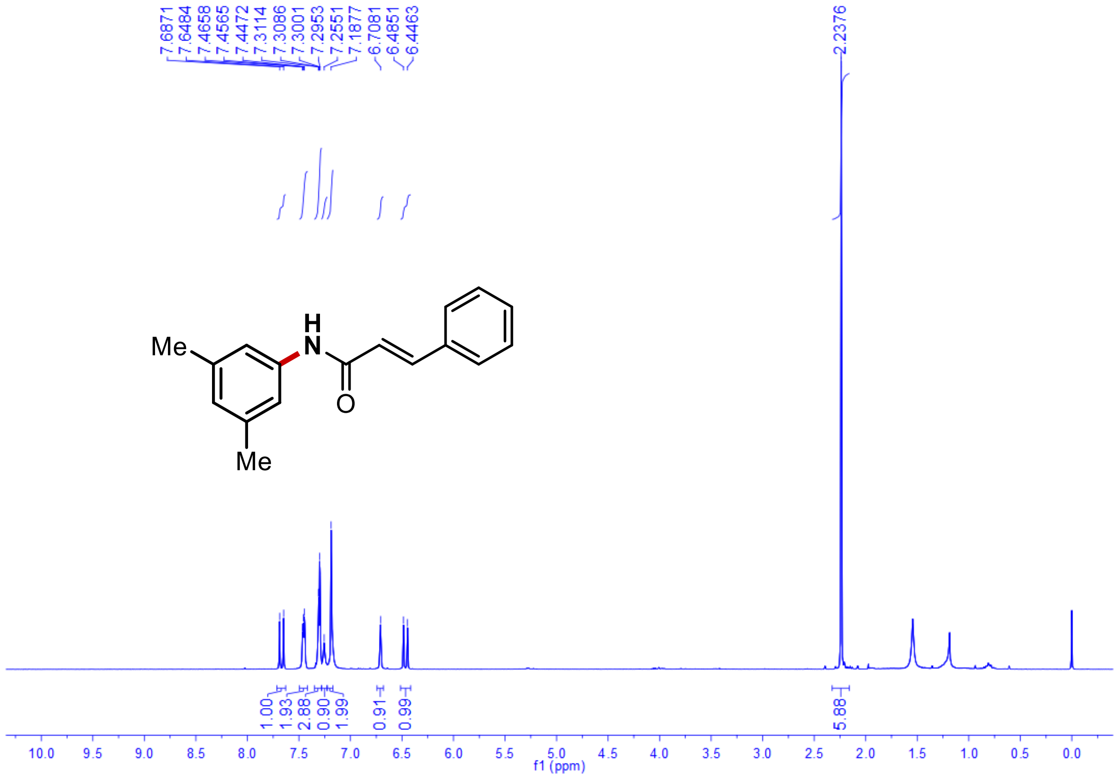

^{1}H NMR (400 MHz, $CDCl_3$) Spectrum

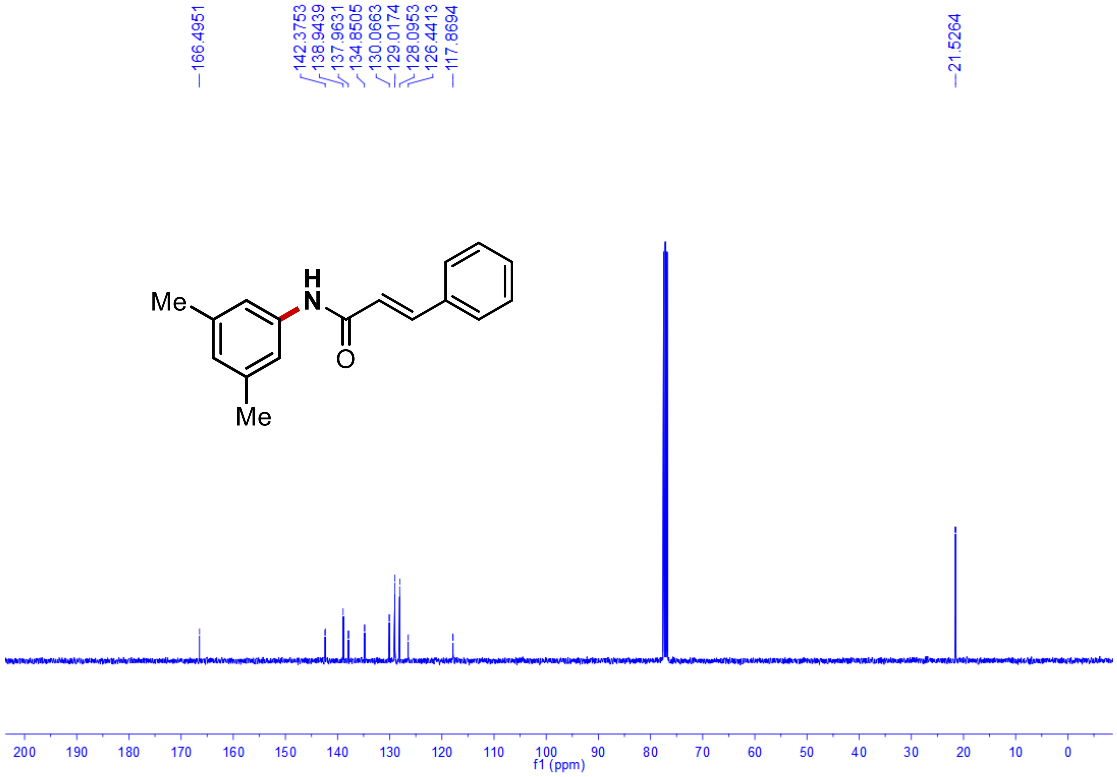

^{13}C NMR (100 MHz, $CDCl_3$) Spectrum

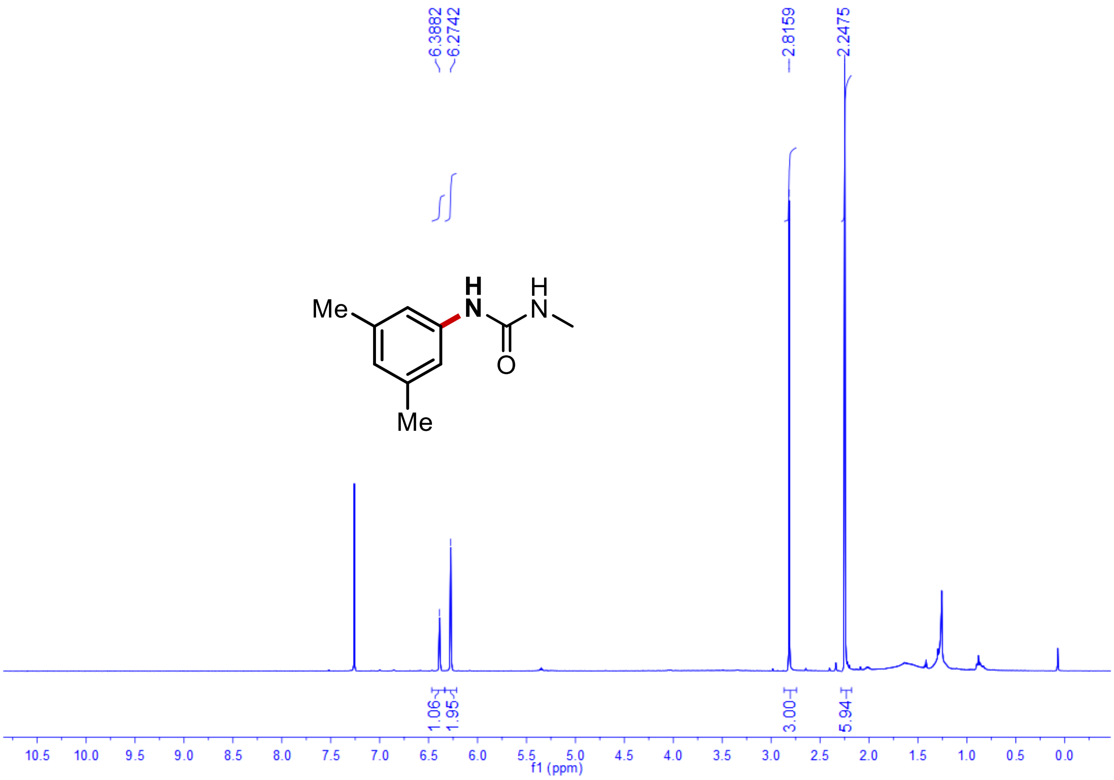

^{1}H NMR (400 MHz, $CDCl_3$) Spectrum

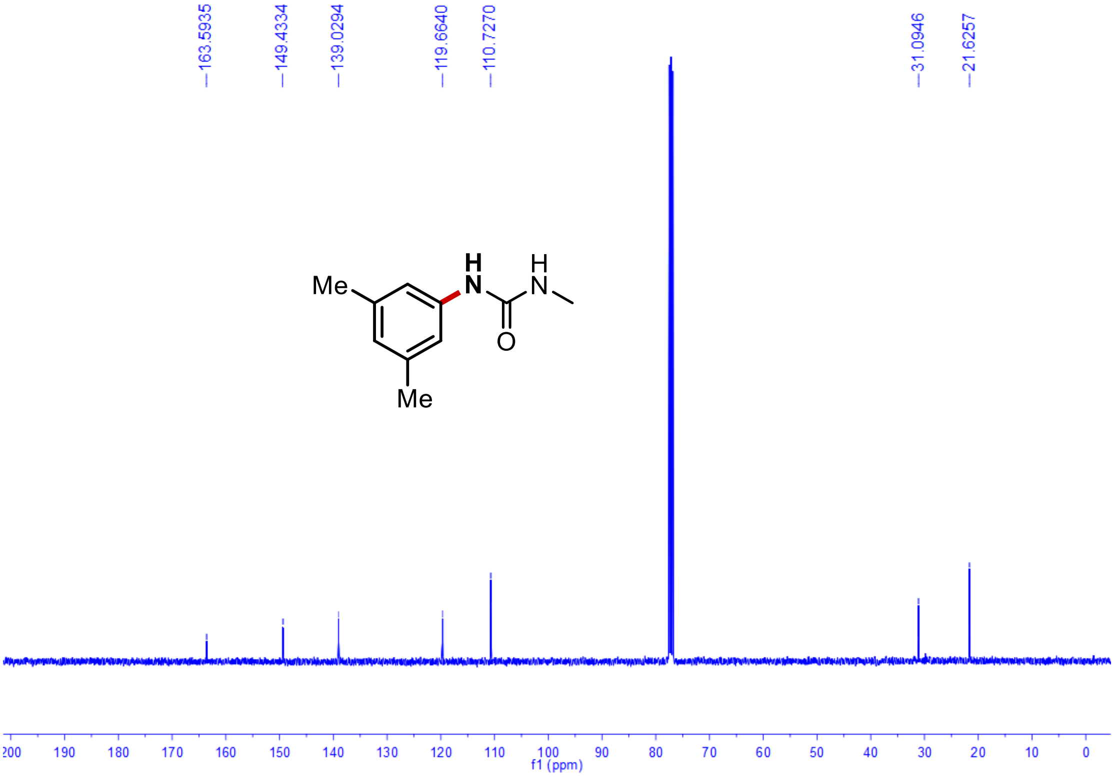

^{13}C NMR (100 MHz, $CDCl_3$) Spectrum

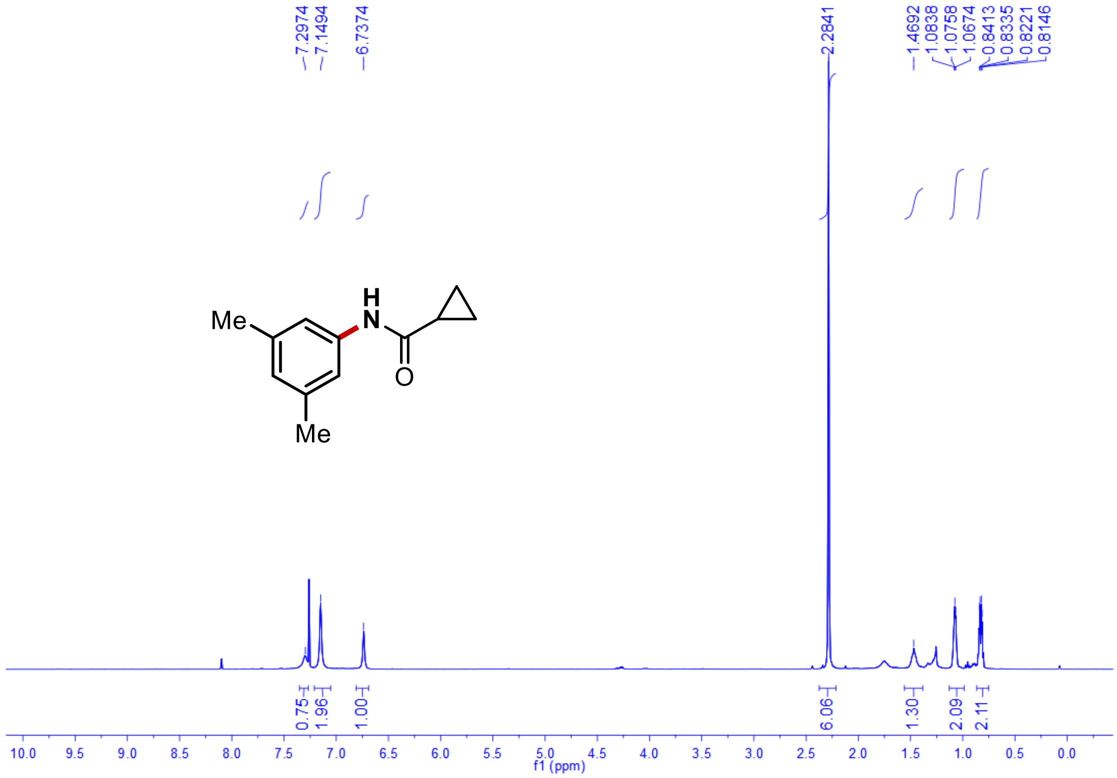

^{1}H NMR (400 MHz, $CDCl_3$) Spectrum

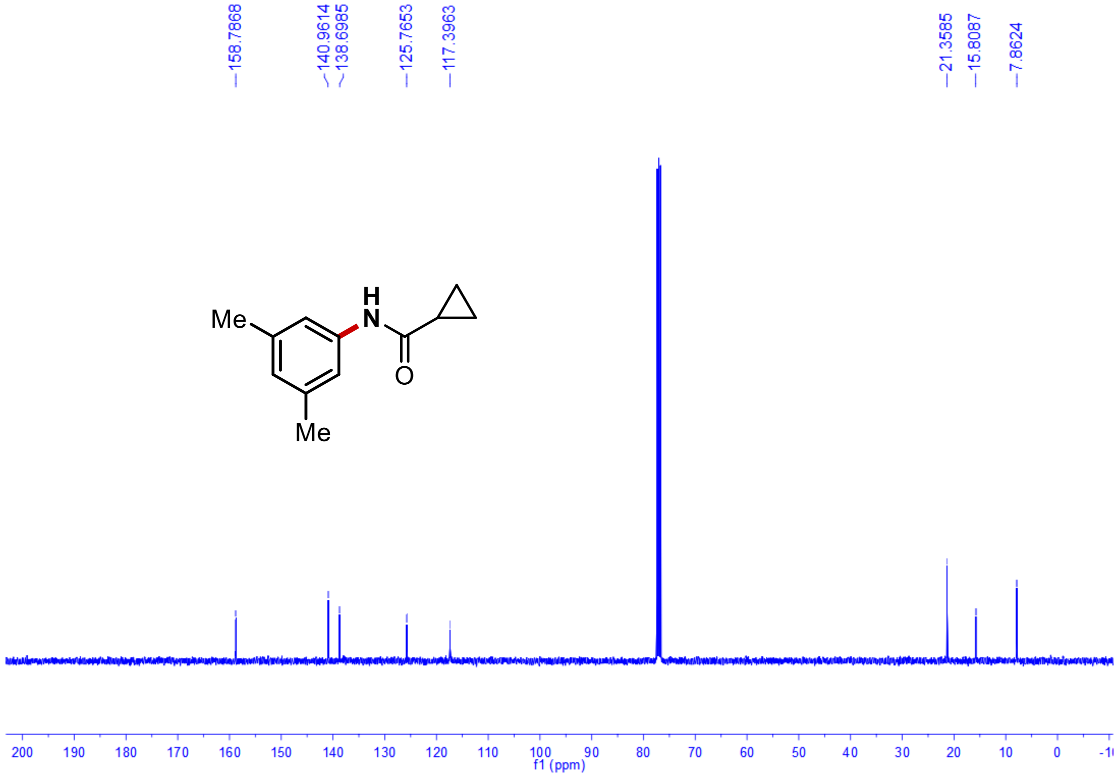

^{13}C NMR (100 MHz, $CDCl_3$) Spectrum

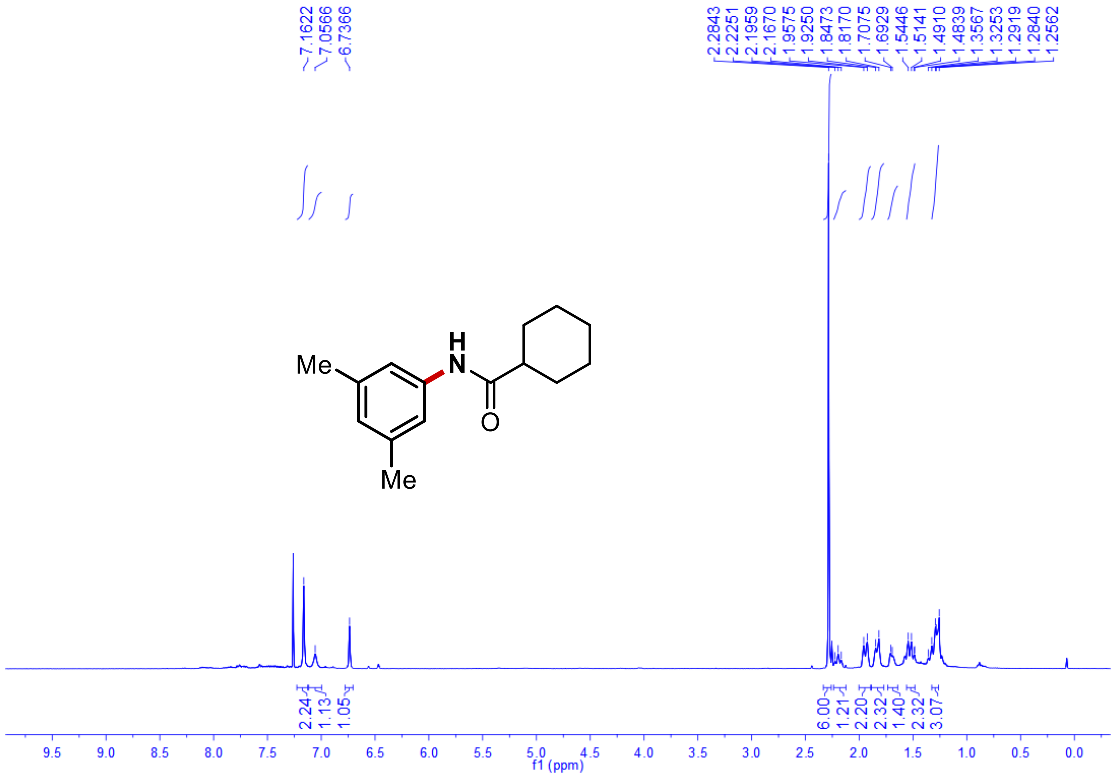

^{1}H NMR (400 MHz, $CDCl_3$) Spectrum

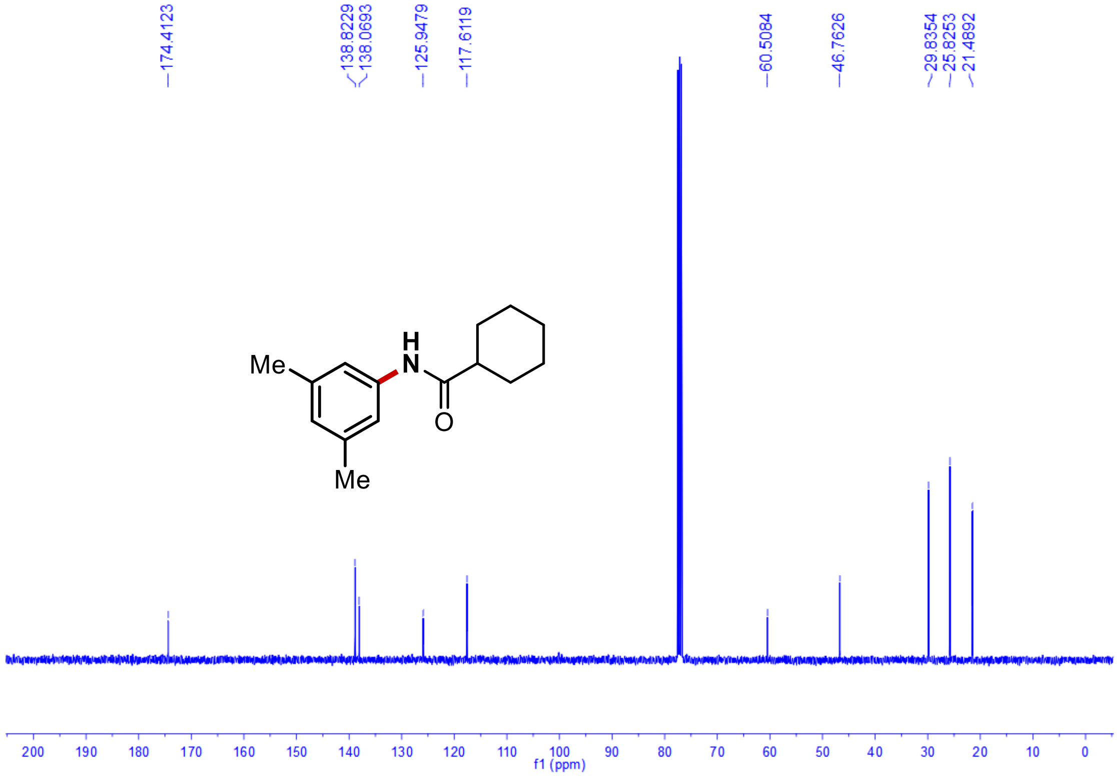

^{13}C NMR (100 MHz, $CDCl_3$) Spectrum

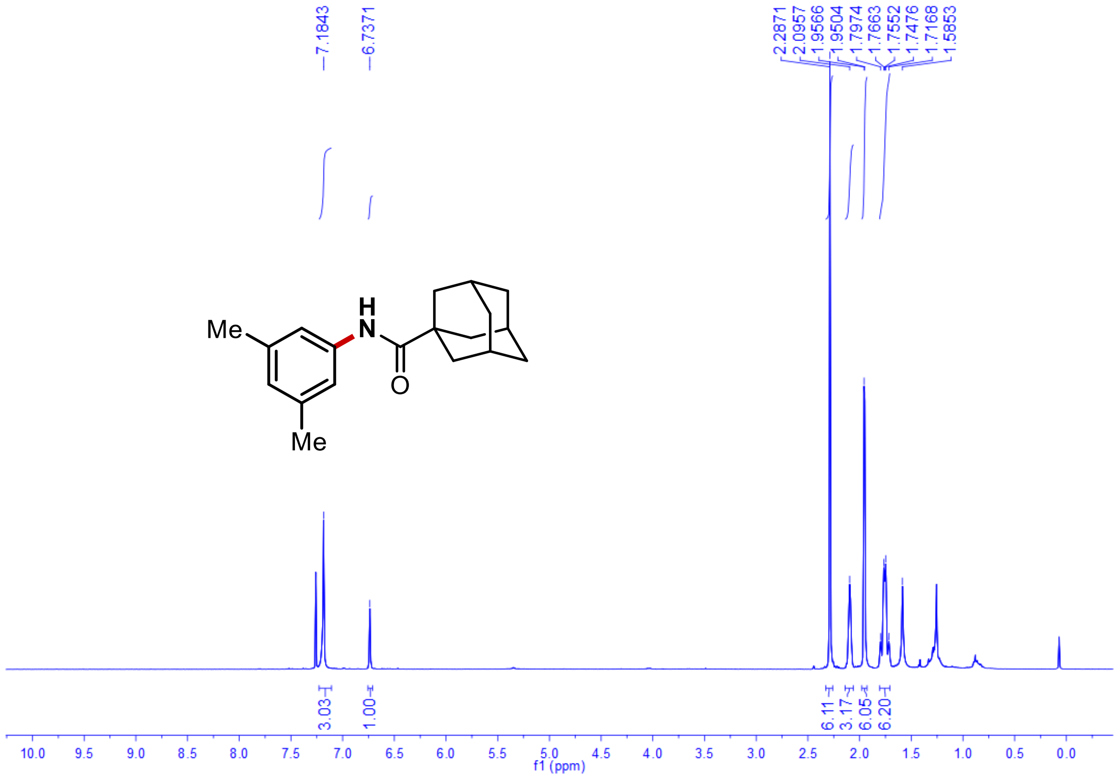

^{1}H NMR (400 MHz, $CDCl_3$) Spectrum

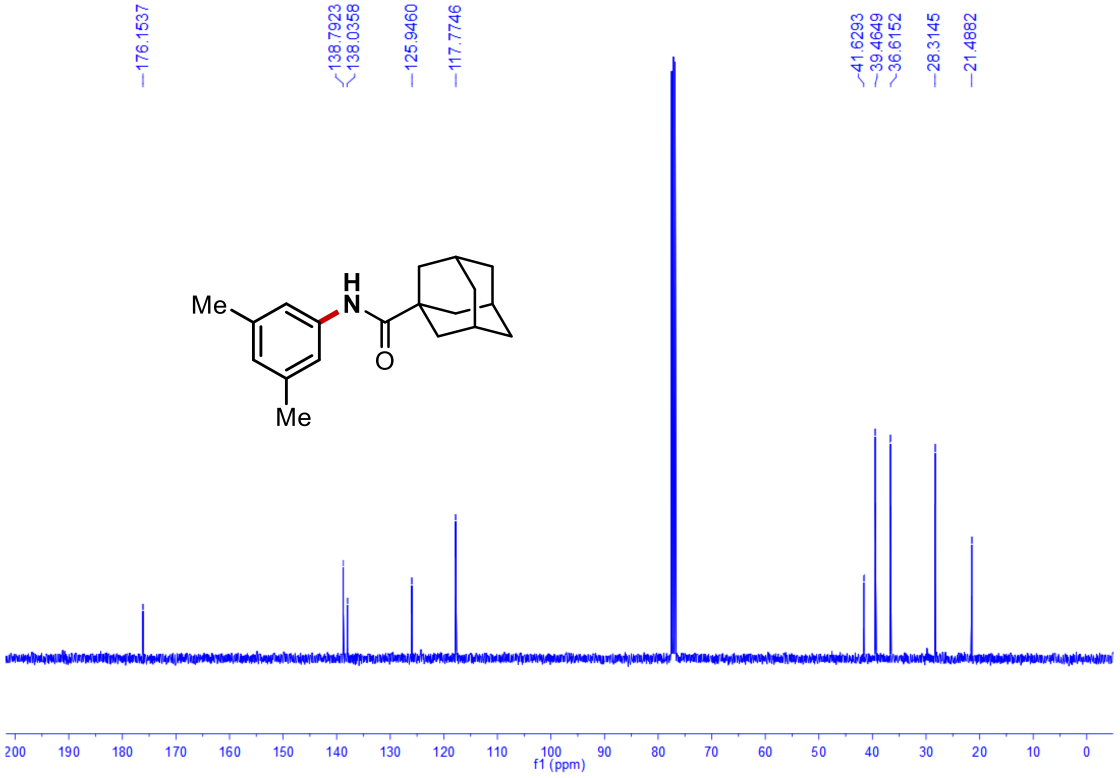

^{13}C NMR (100 MHz, $CDCl_3$) Spectrum

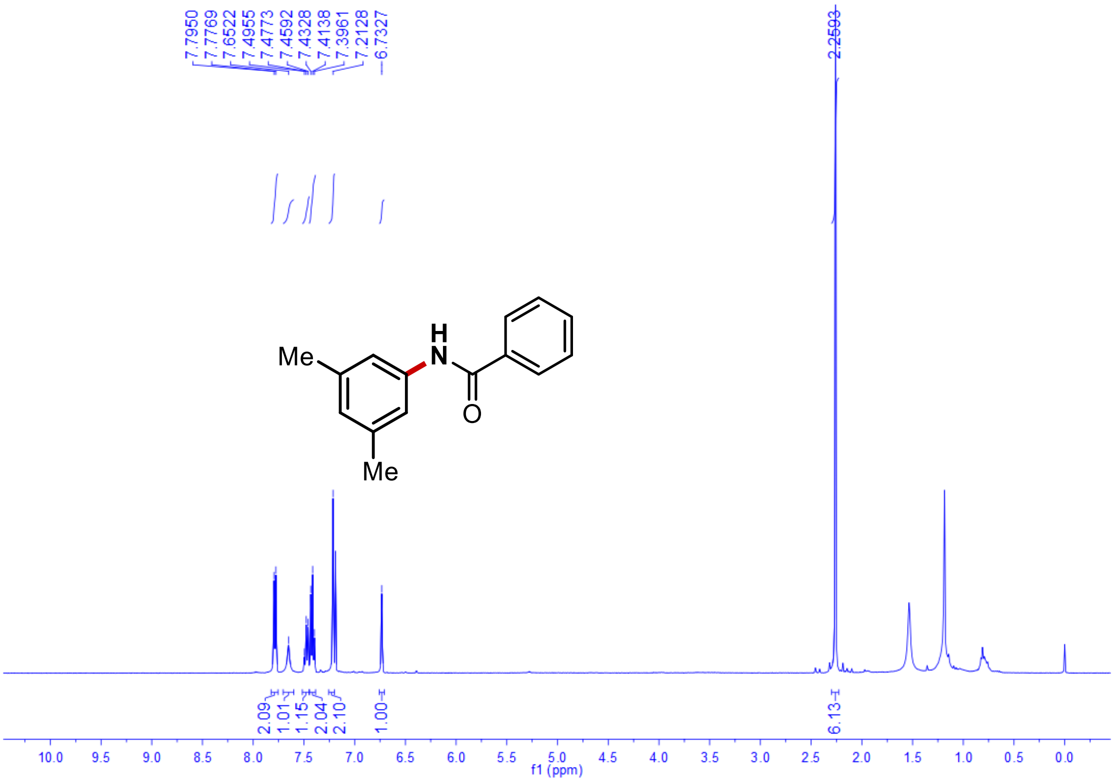

^{1}H NMR (400 MHz, $CDCl_3$) Spectrum

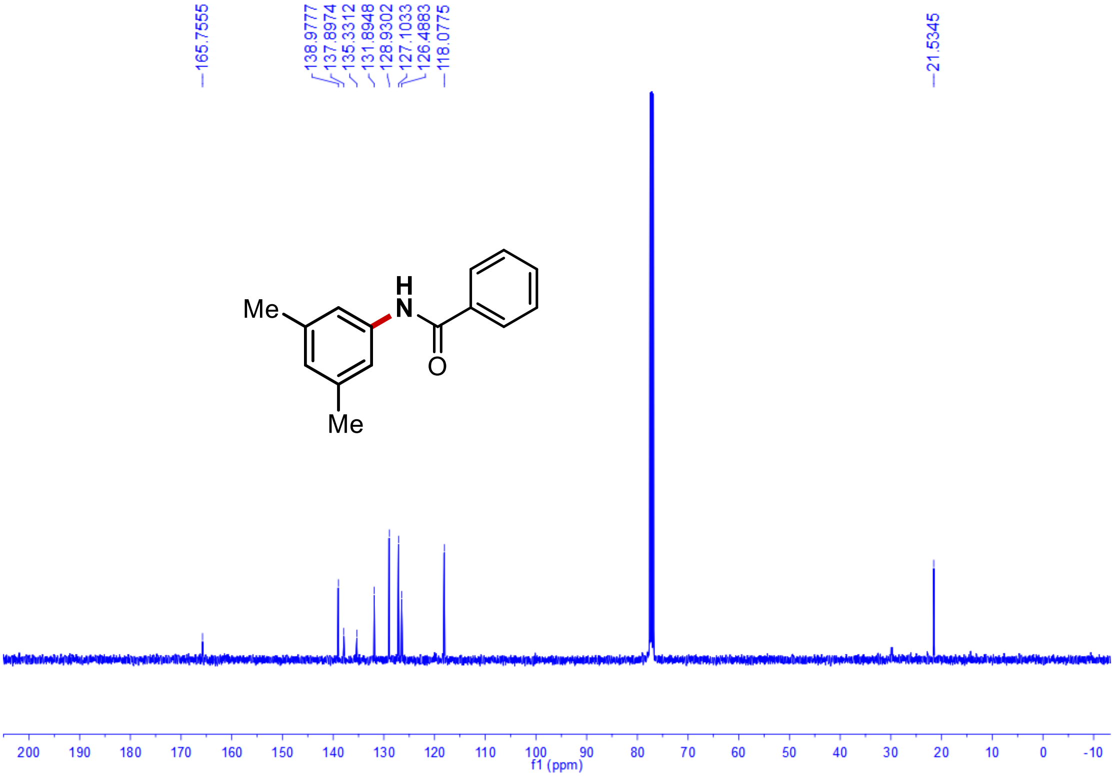

^{13}C NMR (100 MHz, $CDCl_3$) Spectrum

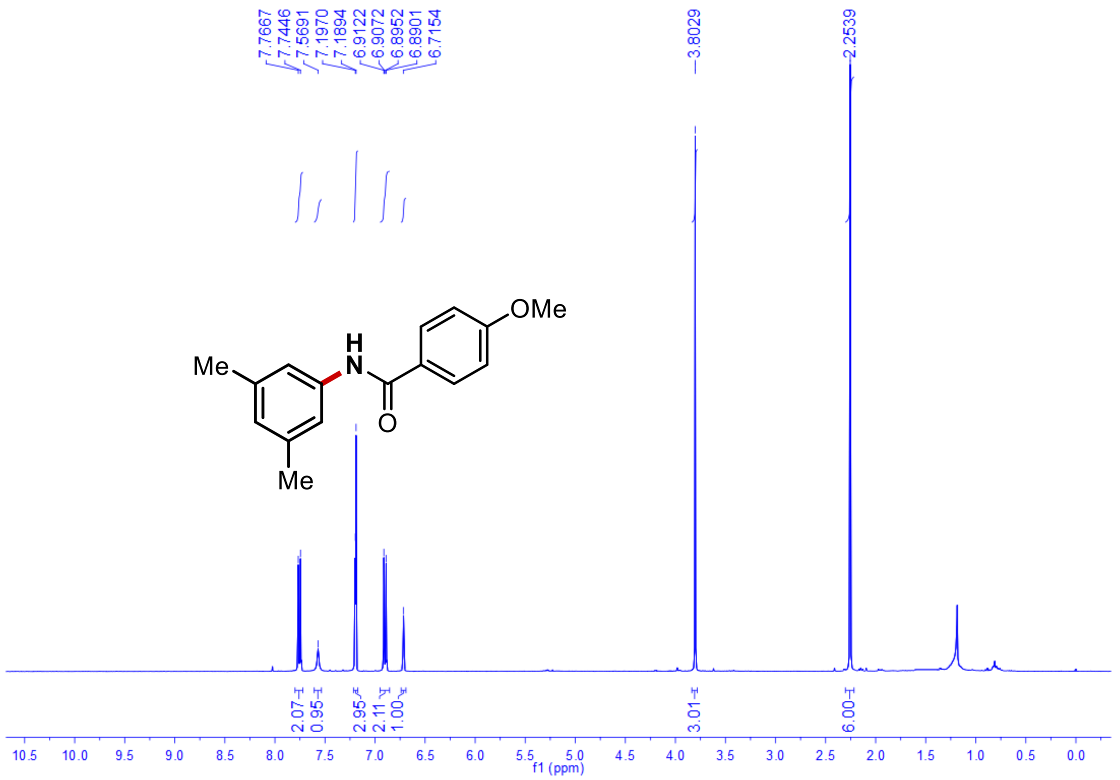

^{1}H NMR (400 MHz, $CDCl_3$) Spectrum

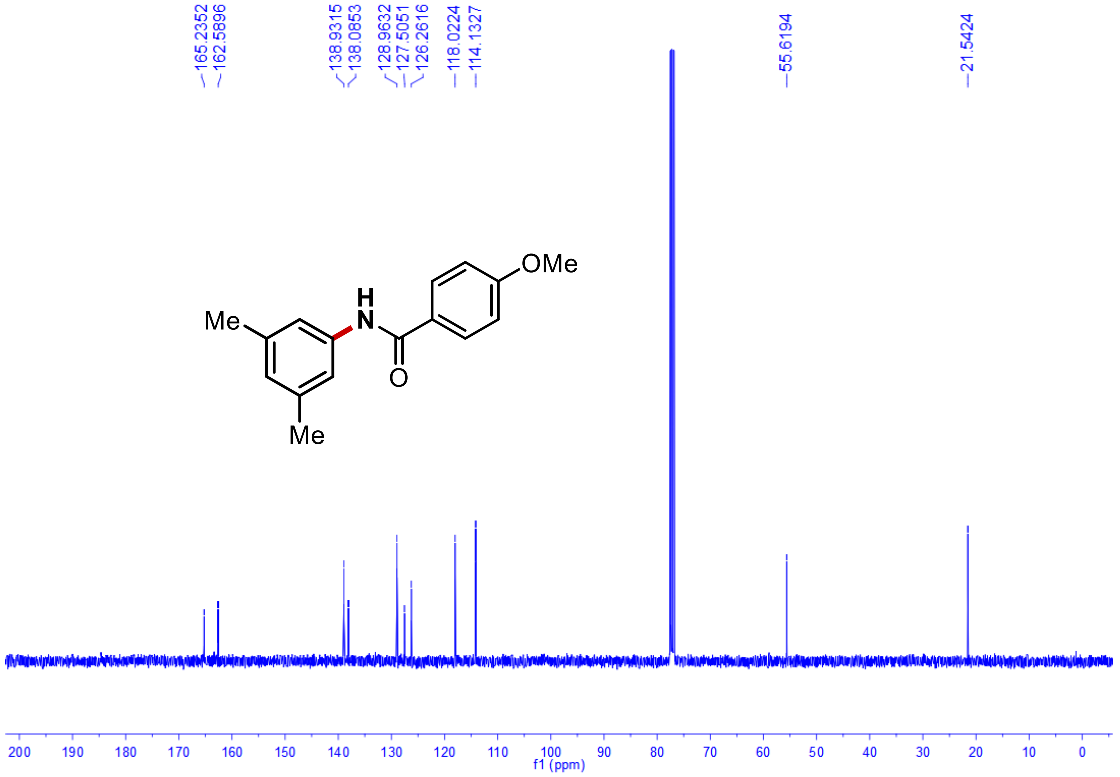

^{13}C NMR (100 MHz, $CDCl_3$) Spectrum

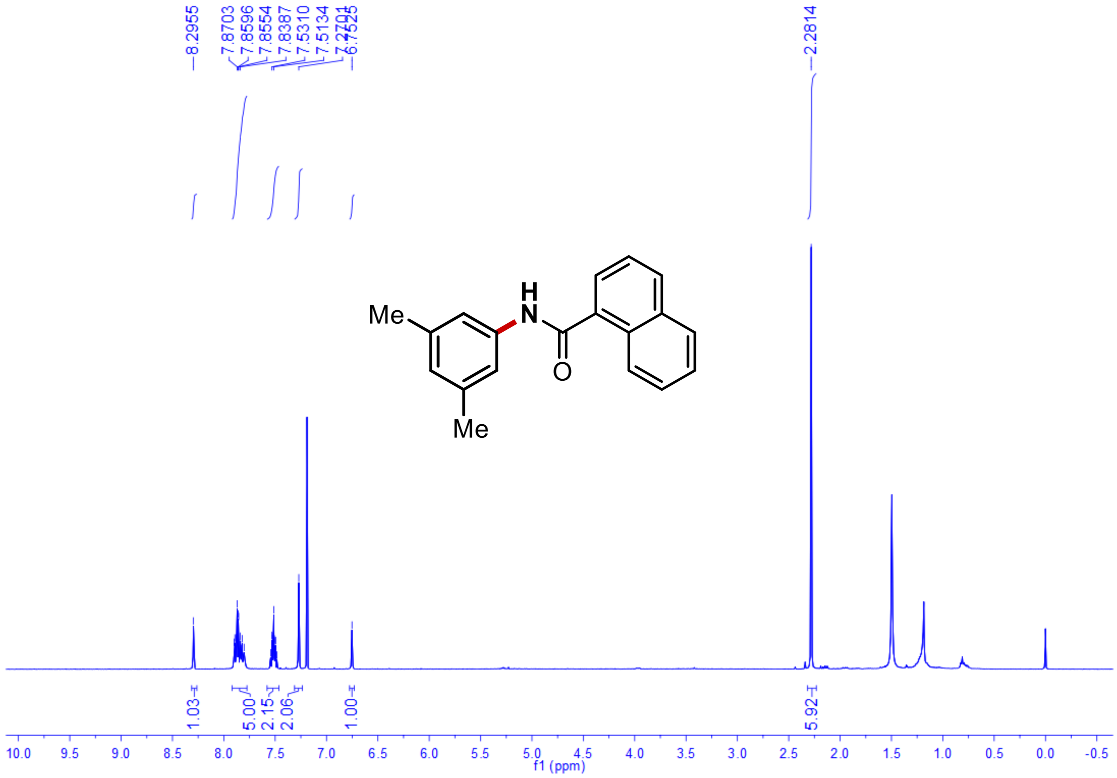

^{1}H NMR (400 MHz, $CDCl_3$) Spectrum

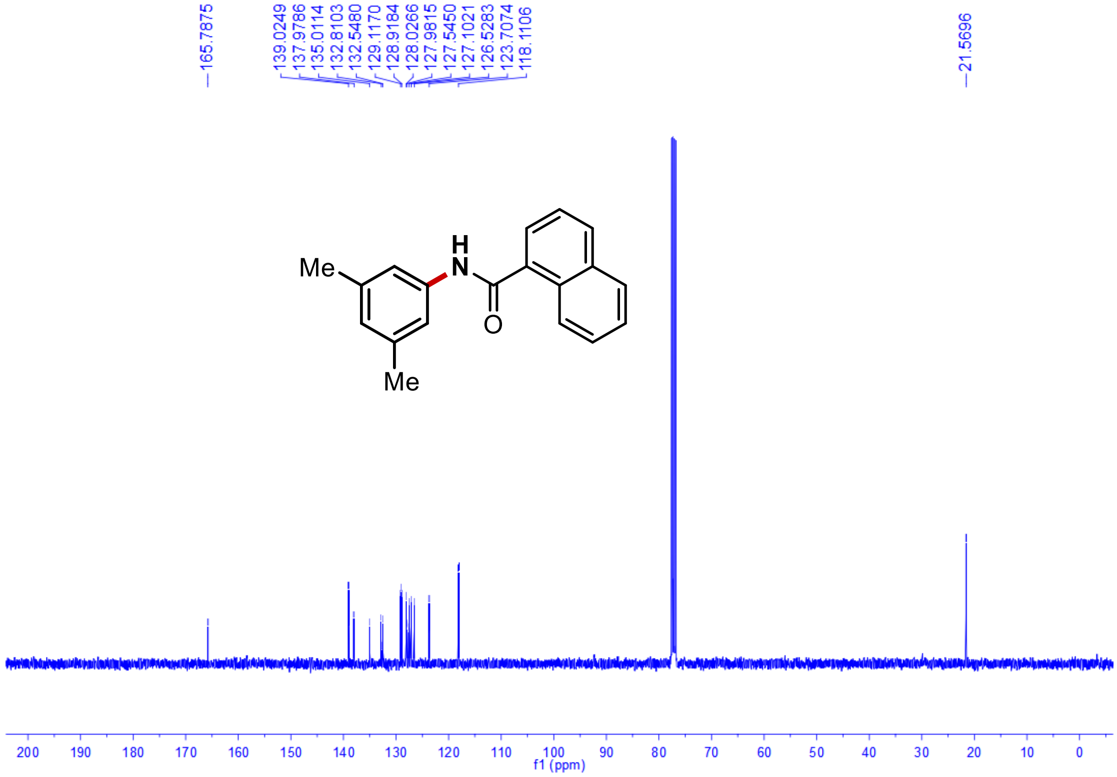

^{13}C NMR (100 MHz, $CDCl_3$) Spectrum

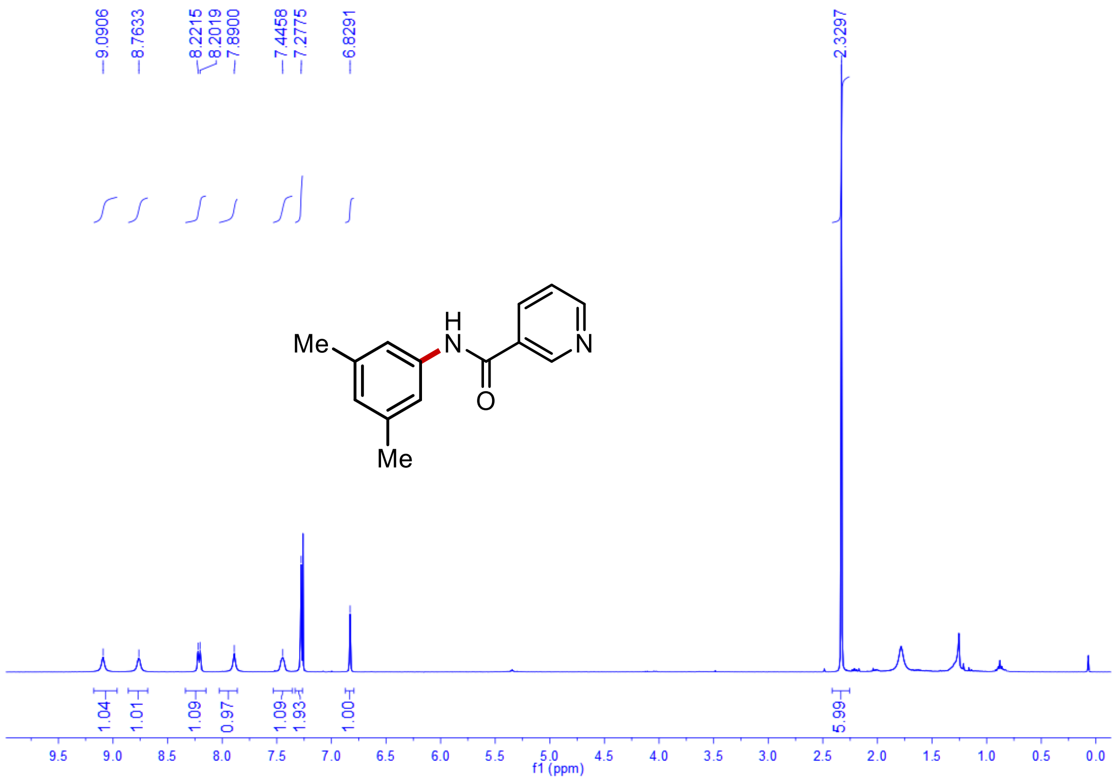

^{1}H NMR (400 MHz, $CDCl_3$) Spectrum

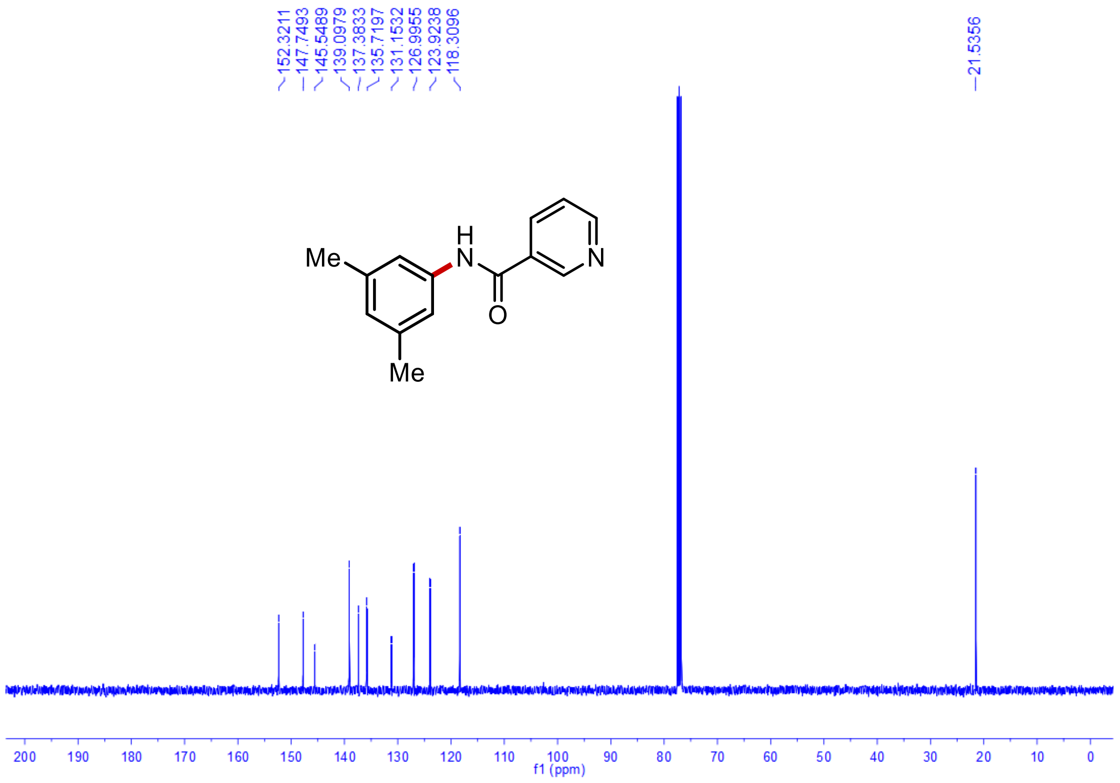

^{13}C NMR (100 MHz, $CDCl_3$) Spectrum

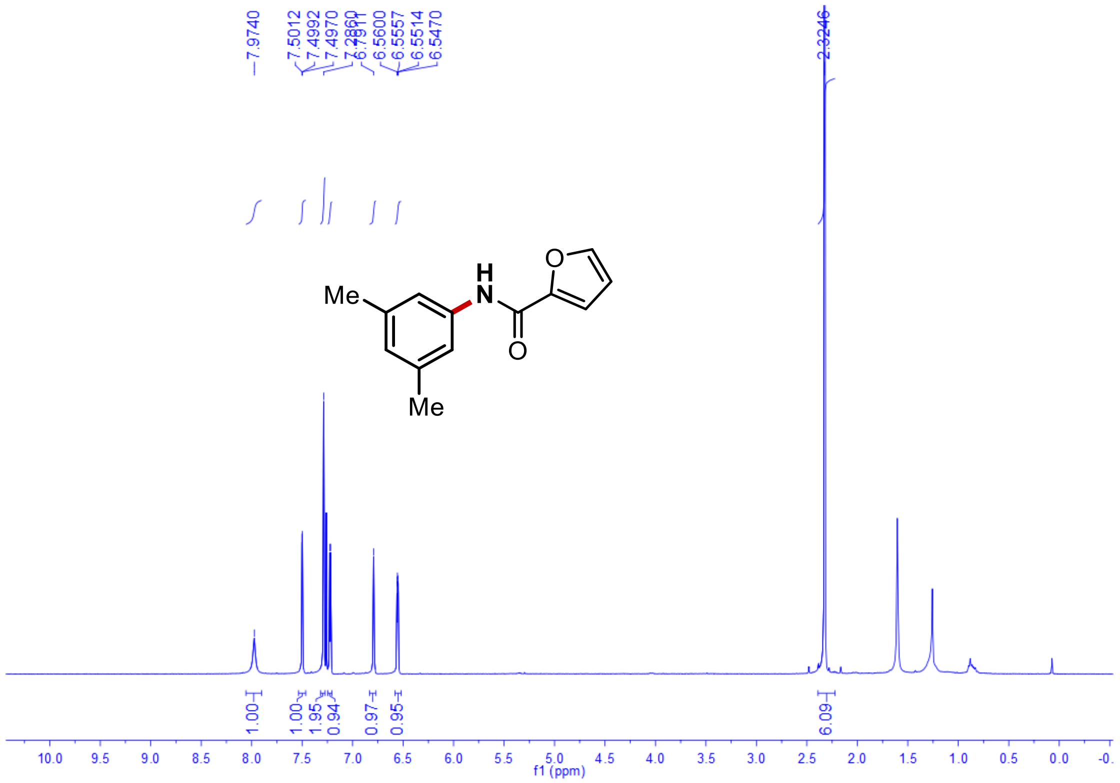

^{1}**H NMR** (400 MHz, $CDCl_3$) Spectrum

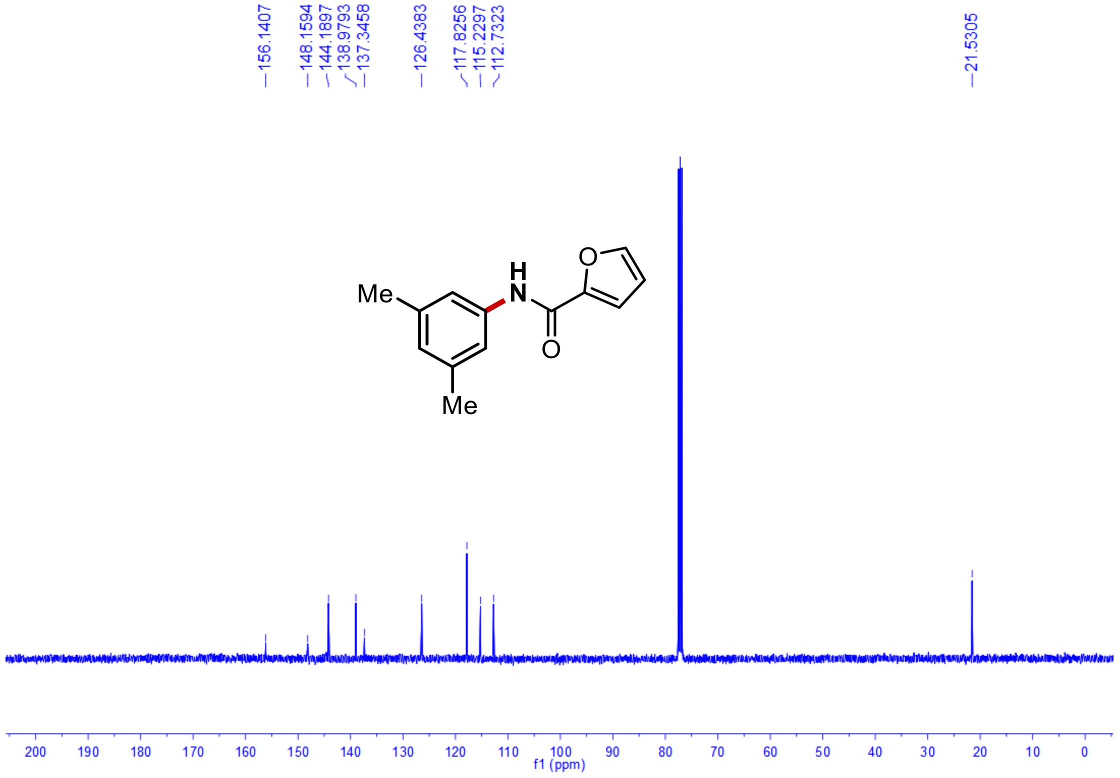

^{13}**C NMR** (100 MHz, $CDCl_3$) Spectrum

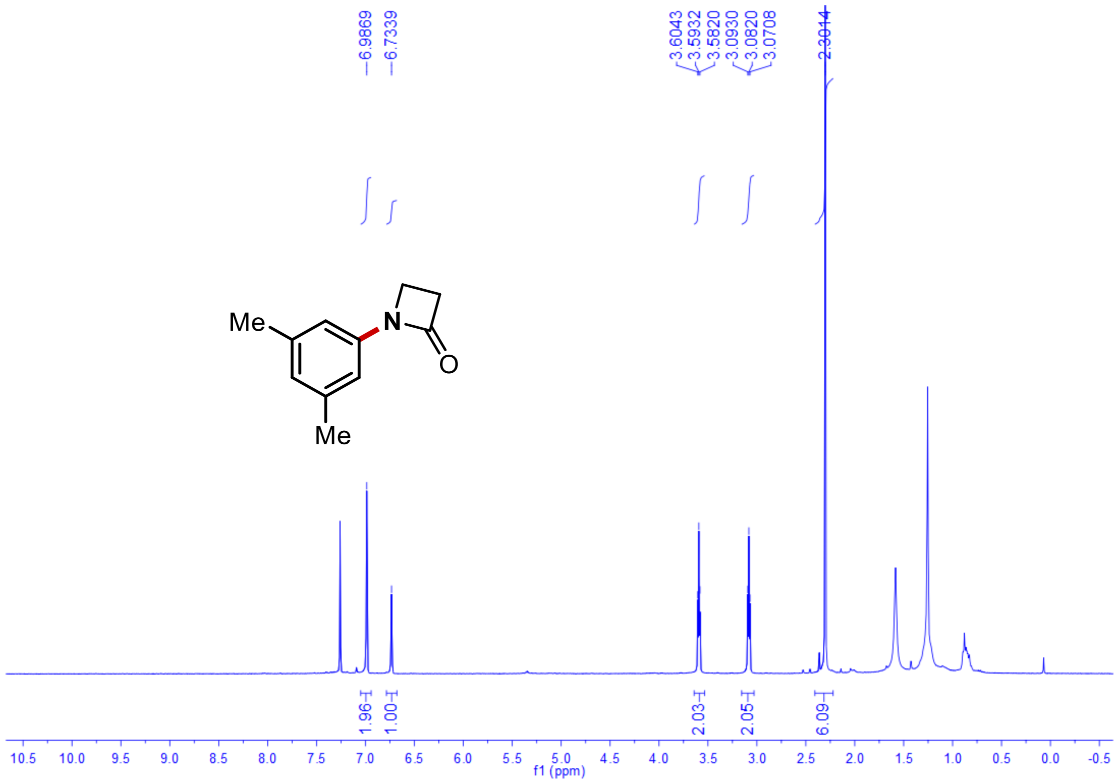

^{1}H NMR (400 MHz, $CDCl_3$) Spectrum

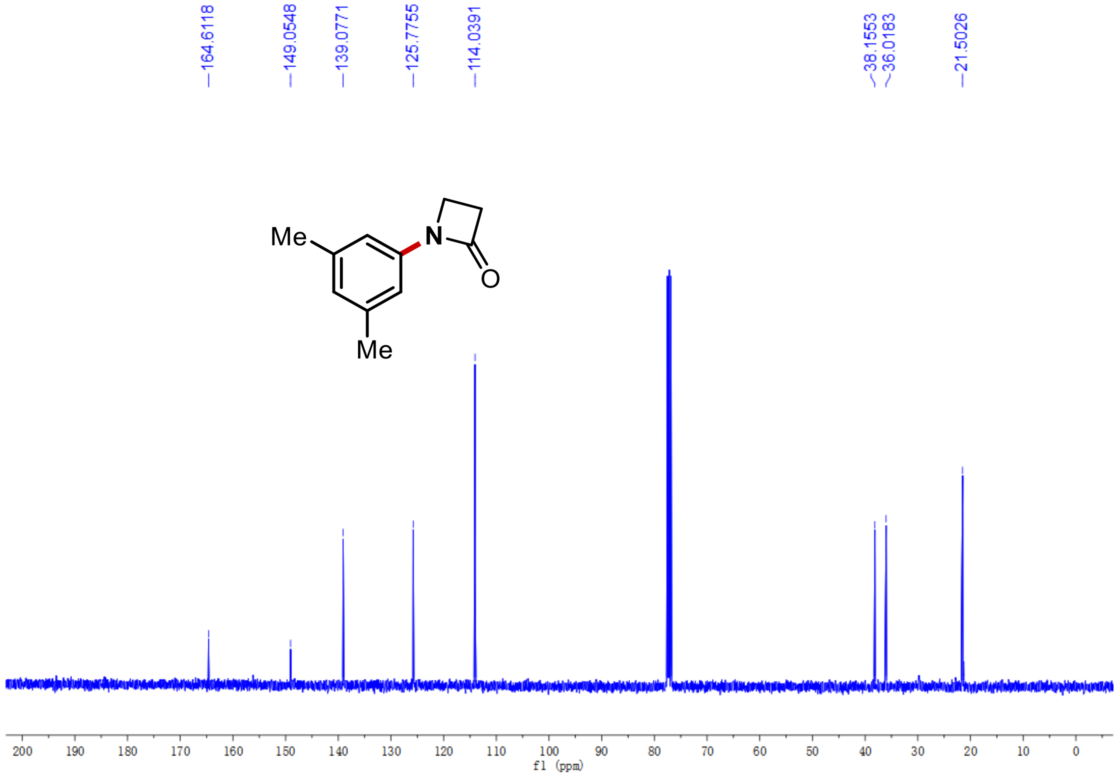

^{13}C NMR (100 MHz, $CDCl_3$) Spectrum

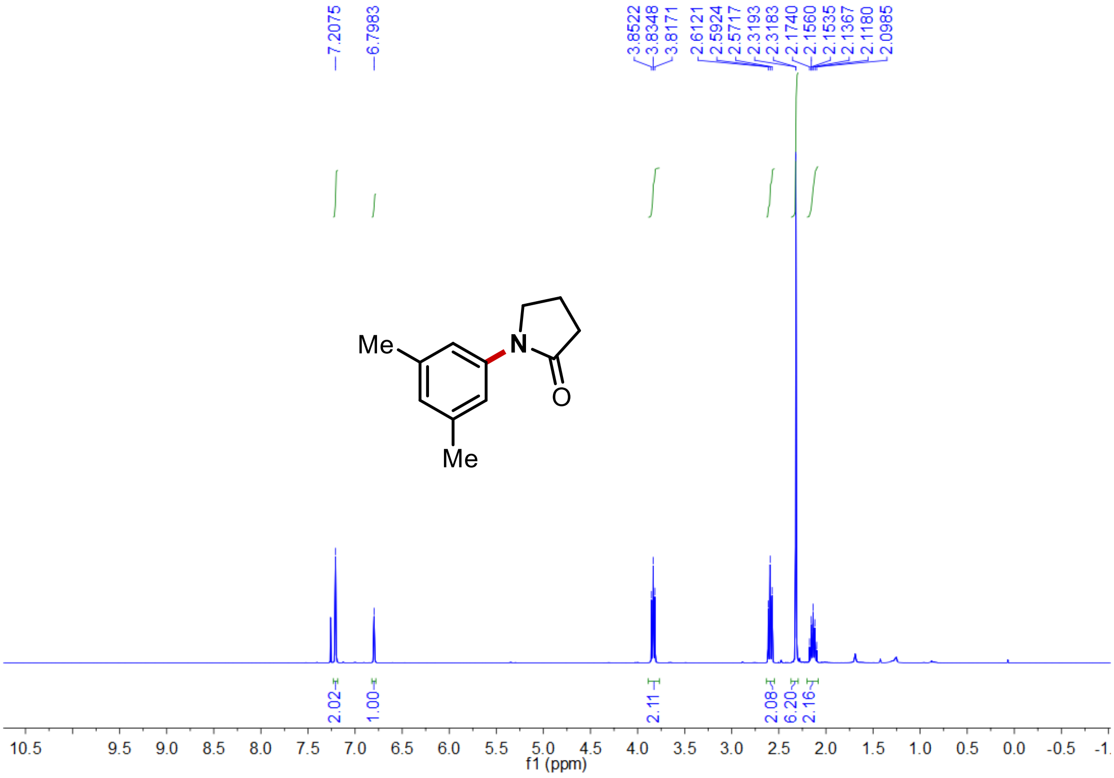

^{1}H NMR (400 MHz, $CDCl_3$) Spectrum

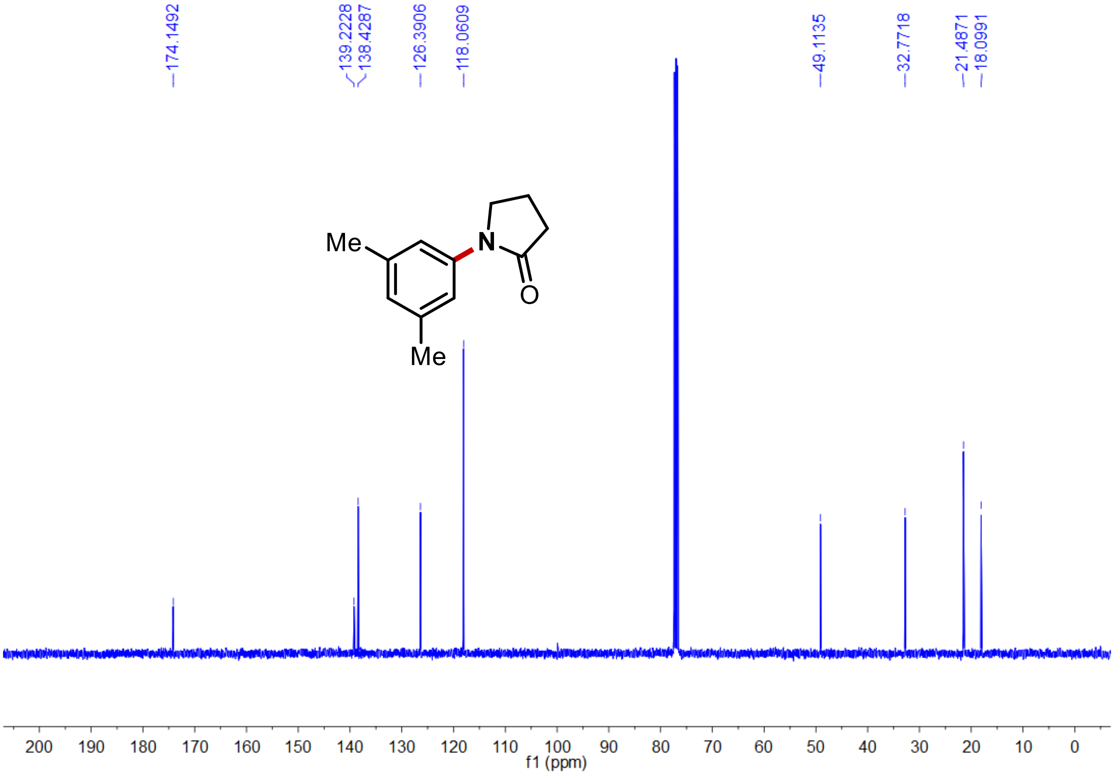

^{13}C NMR (100 MHz, $CDCl_3$) Spectrum

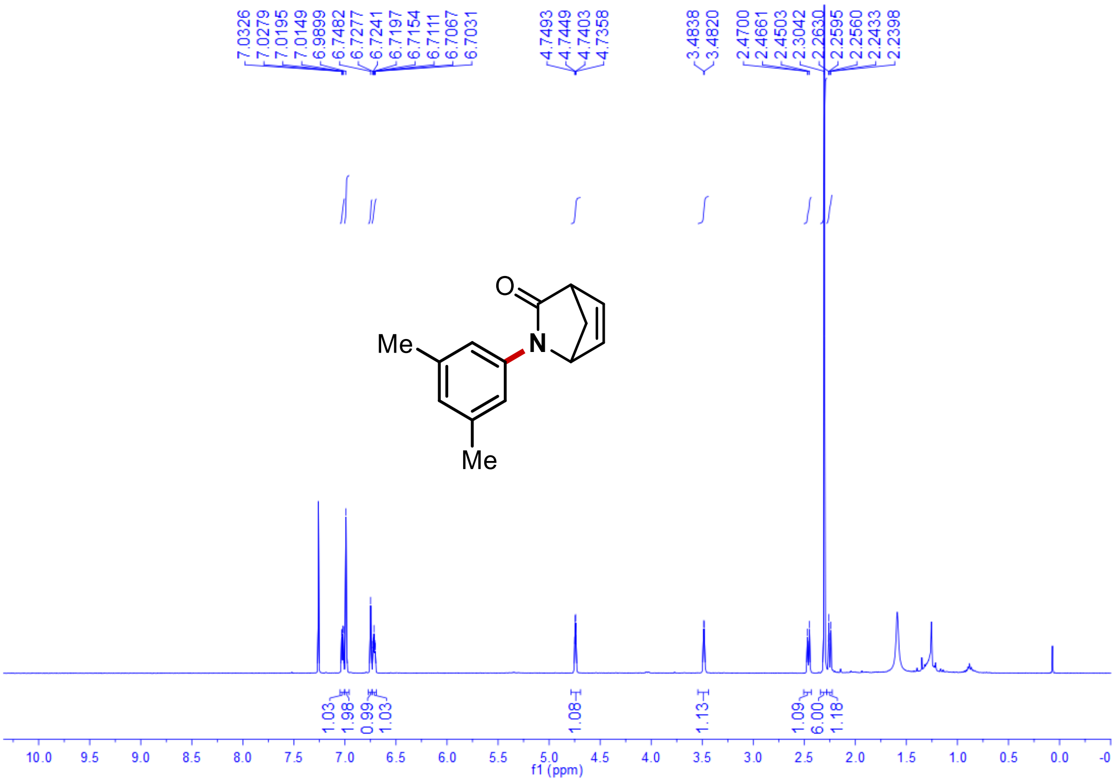

^{1}H NMR (400 MHz, $CDCl_3$) Spectrum

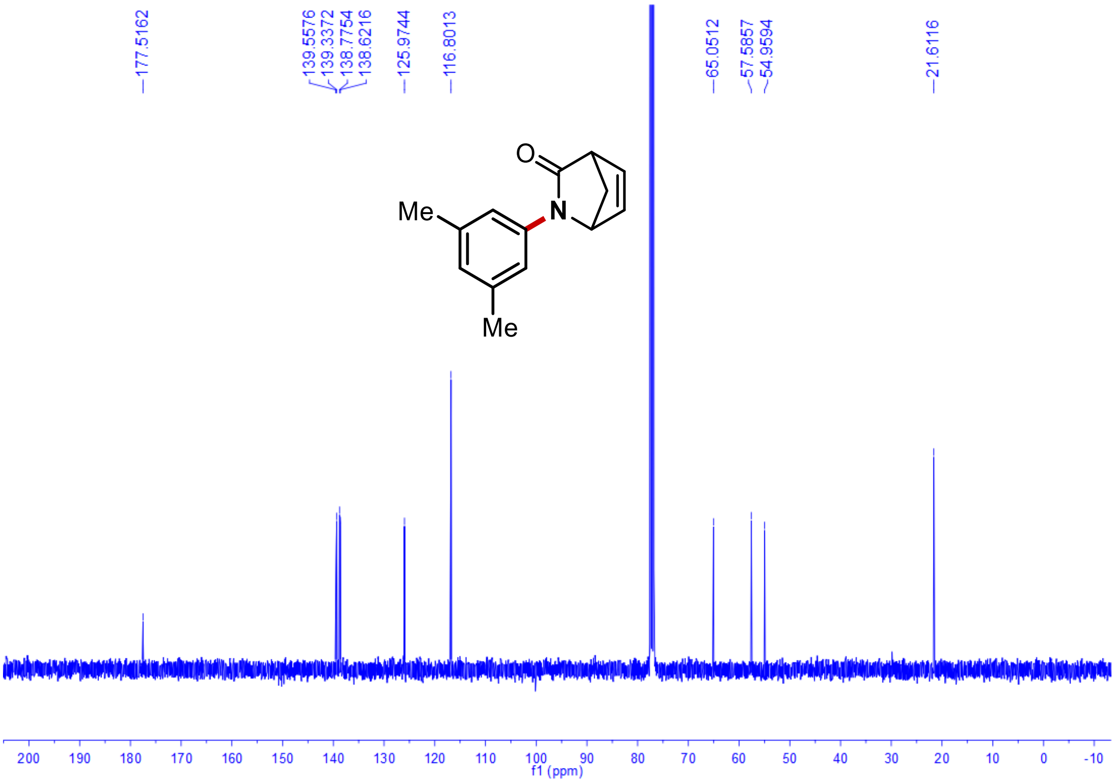

^{13}C NMR (100 MHz, $CDCl_3$) Spectrum

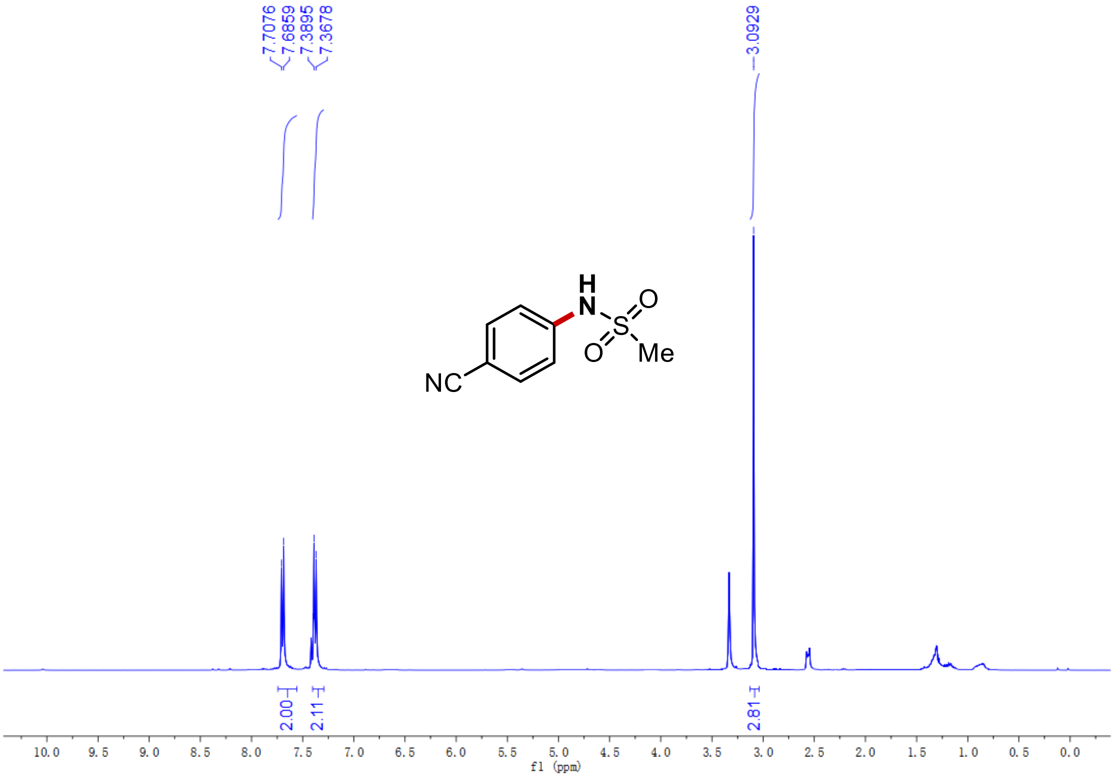

^{1}H NMR (400 MHz, $CDCl_3$) Spectrum

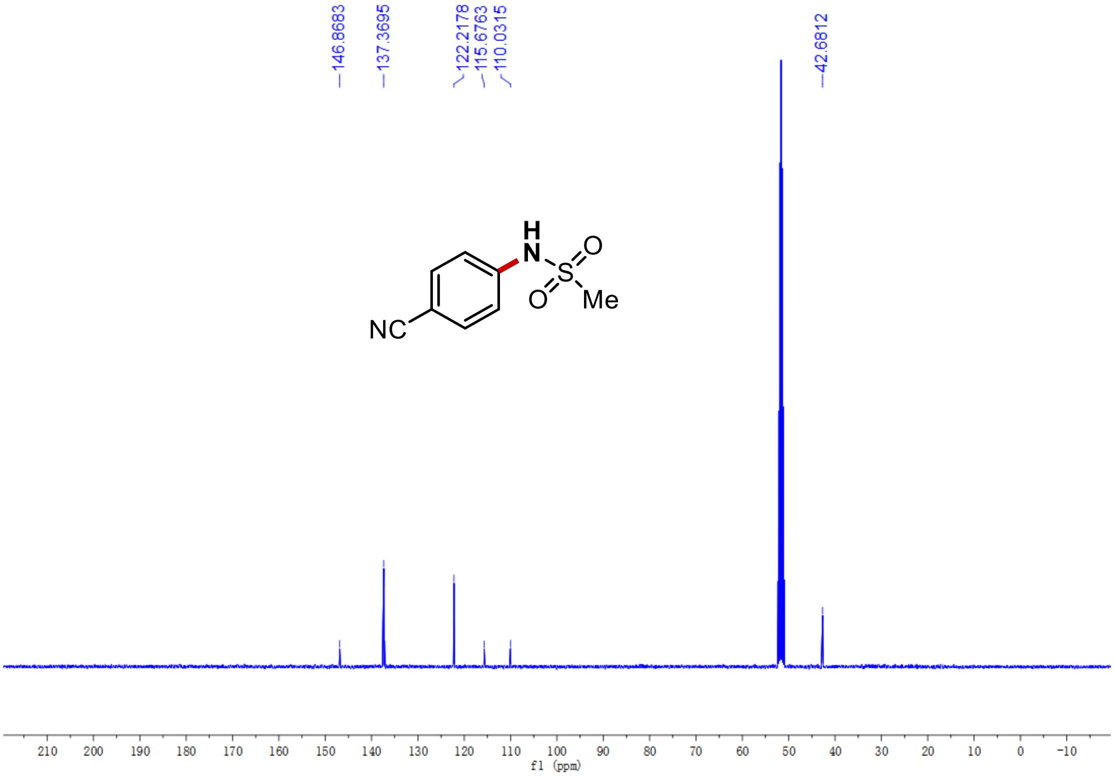

^{13}C NMR (100 MHz, $CDCl_3$) Spectrum

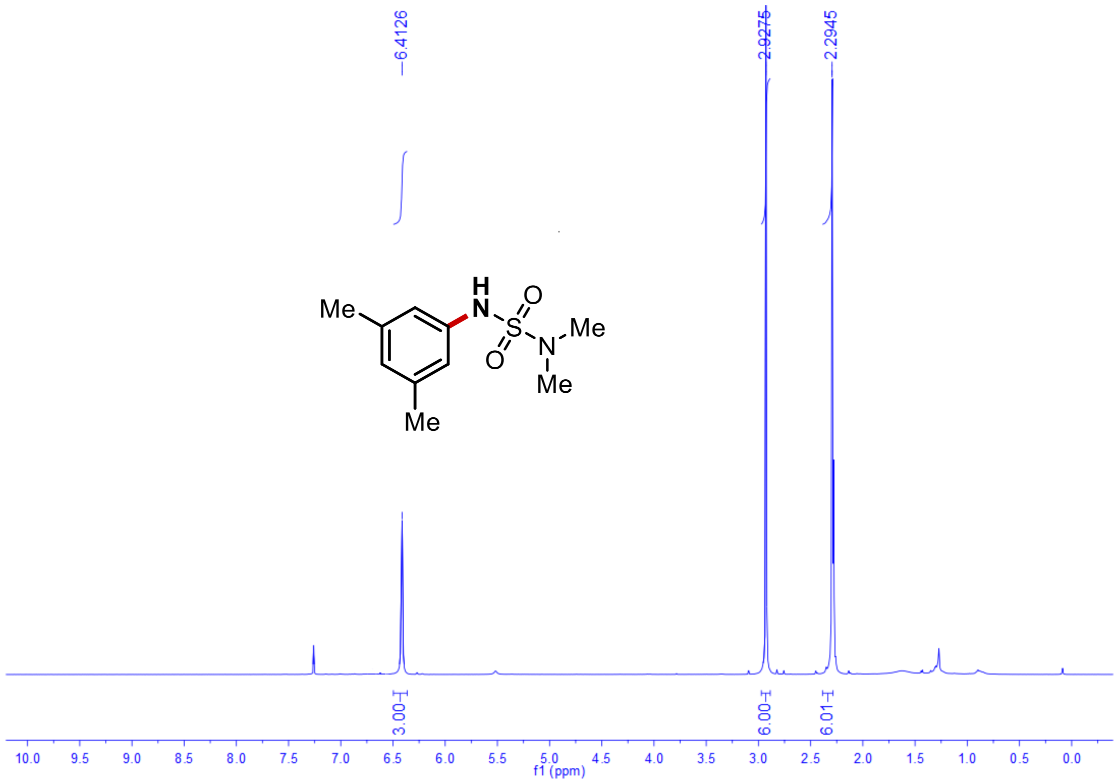

^{1}H NMR (400 MHz, $CDCl_3$) Spectrum

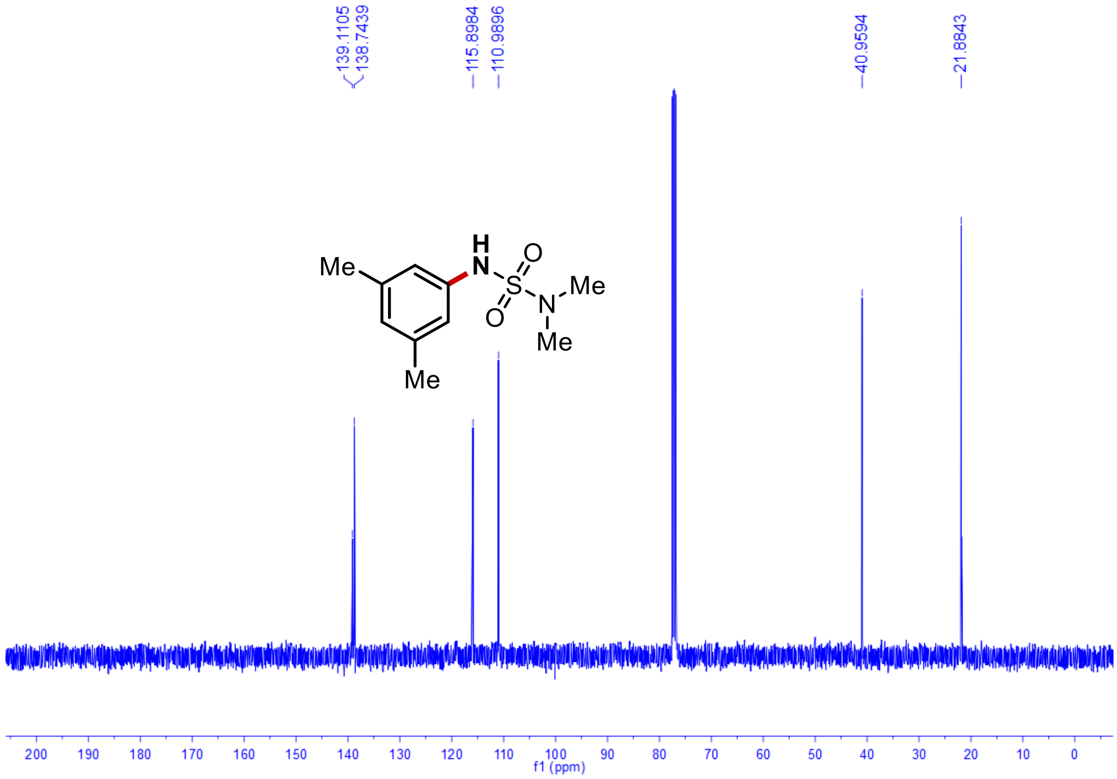

^{13}C NMR (100 MHz, $CDCl_3$) Spectrum

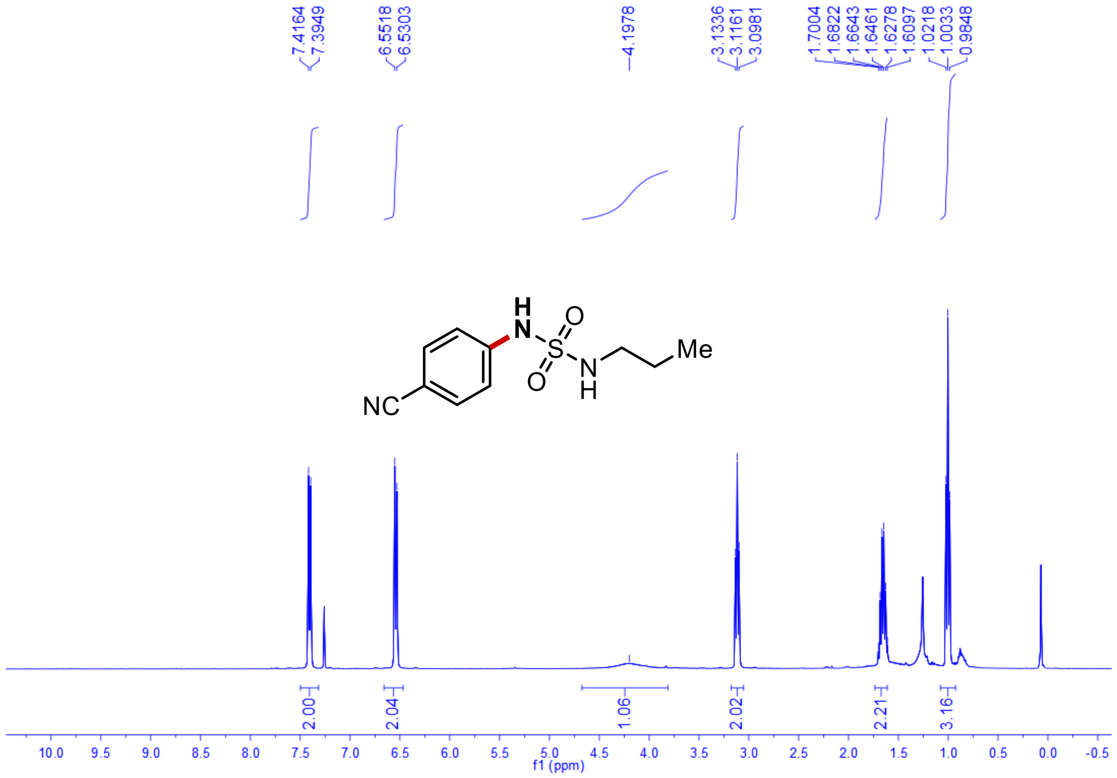

^{1}H NMR (400 MHz, $CDCl_3$) Spectrum

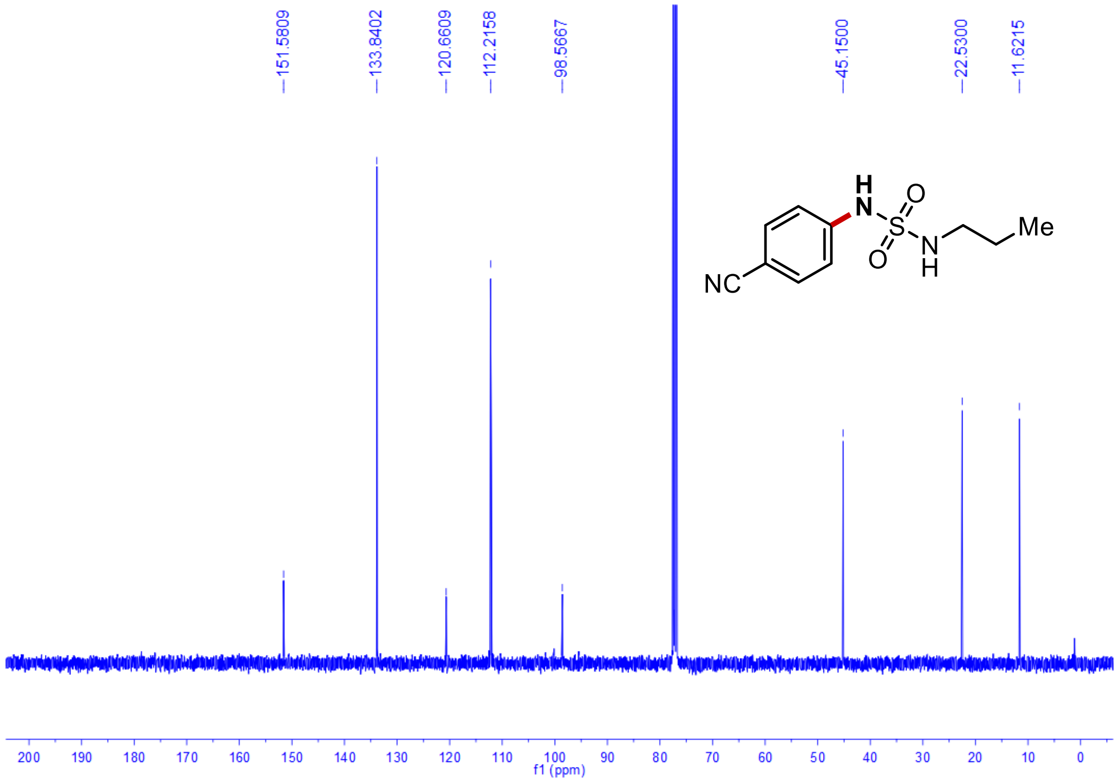

^{13}C NMR (100 MHz, $CDCl_3$) Spectrum

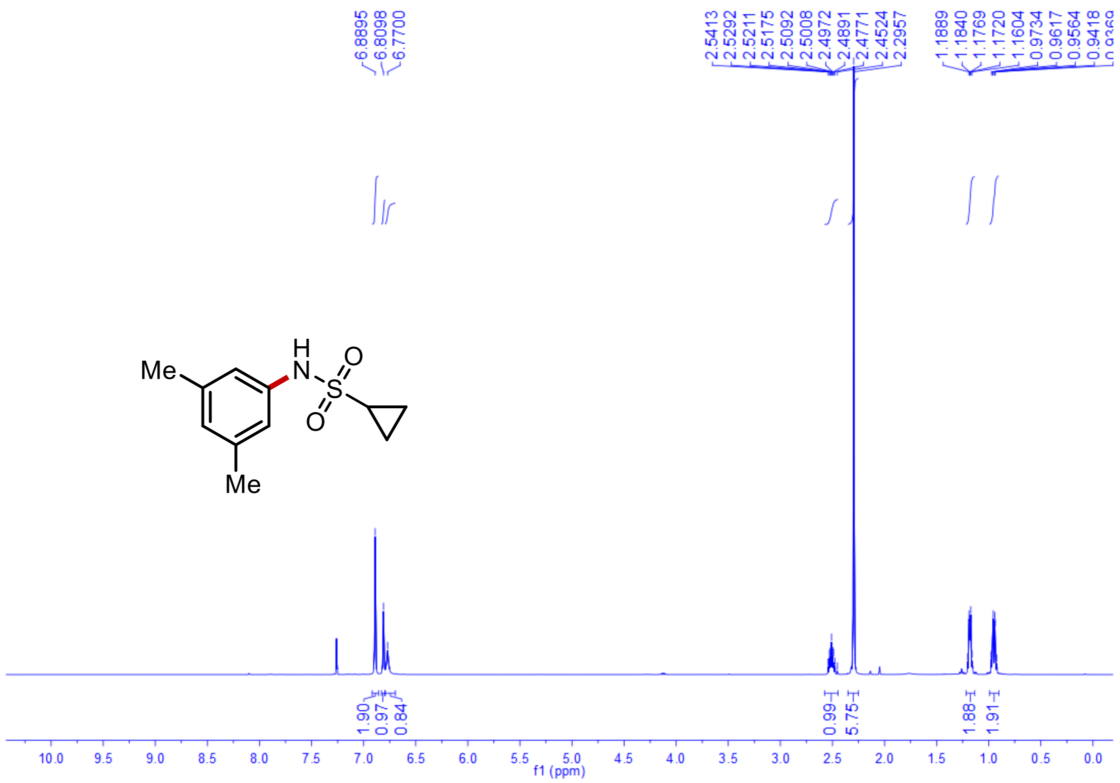

^{1}H NMR (400 MHz, $CDCl_3$) Spectrum

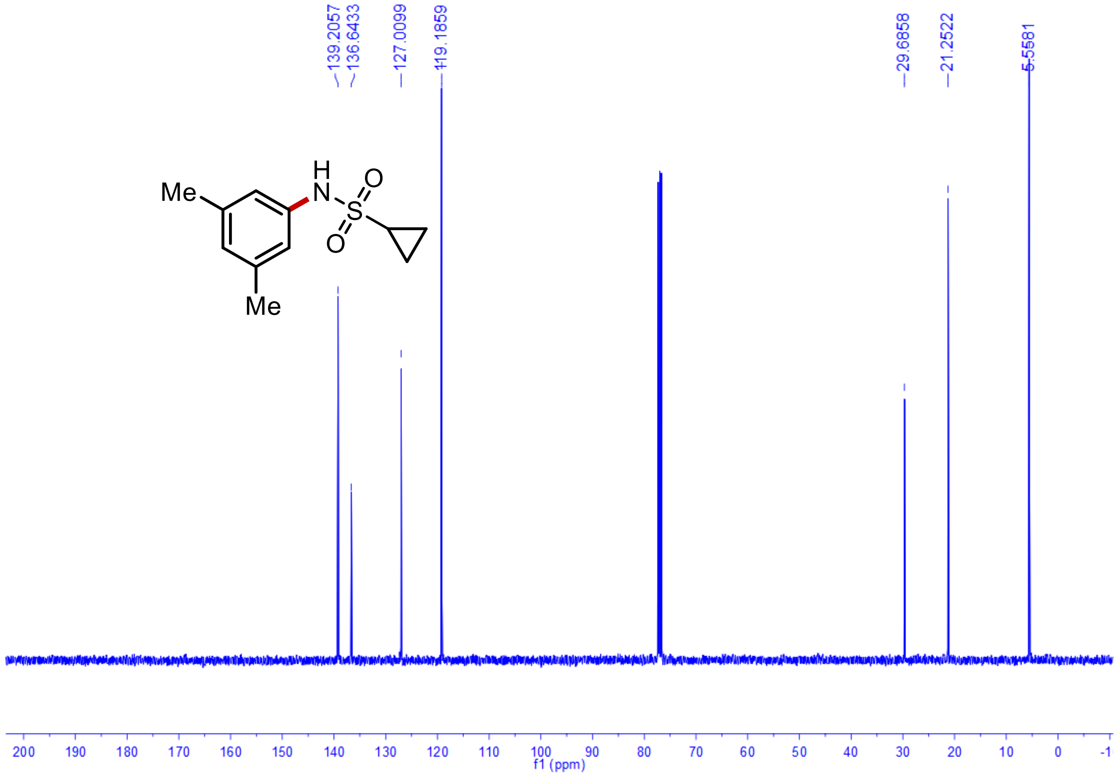

^{13}C NMR (100 MHz, $CDCl_3$) Spectrum

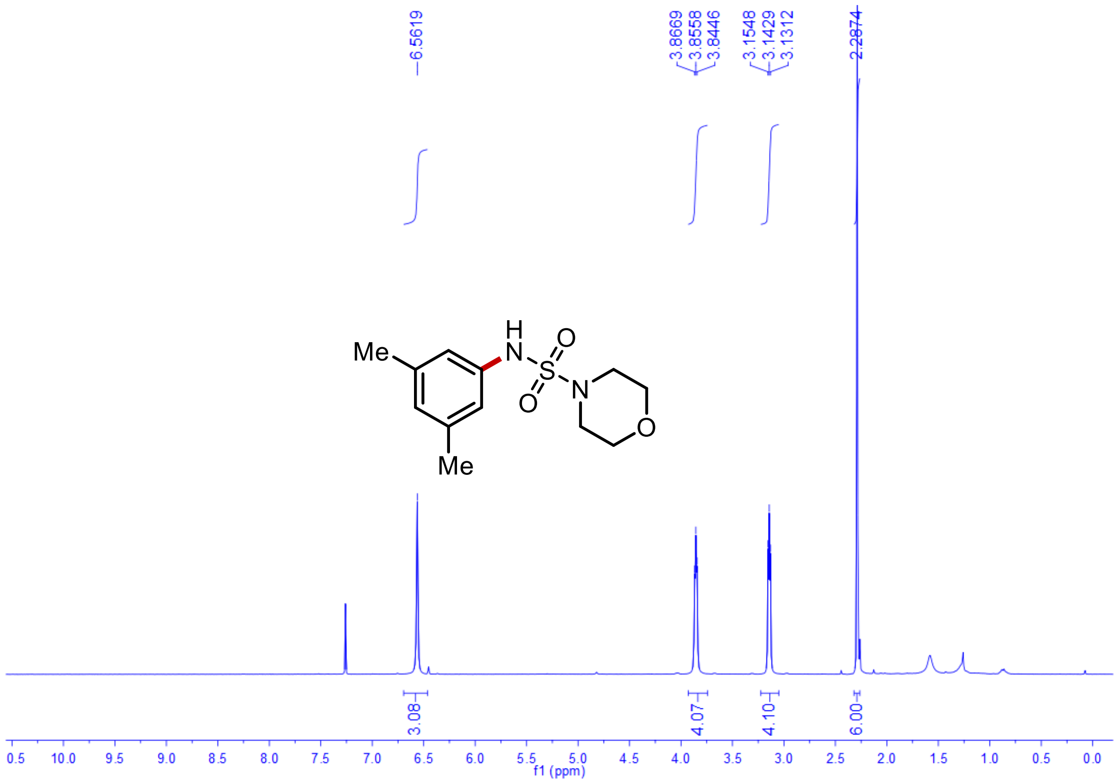

^{1}**H NMR** (400 MHz, $CDCl_3$) Spectrum

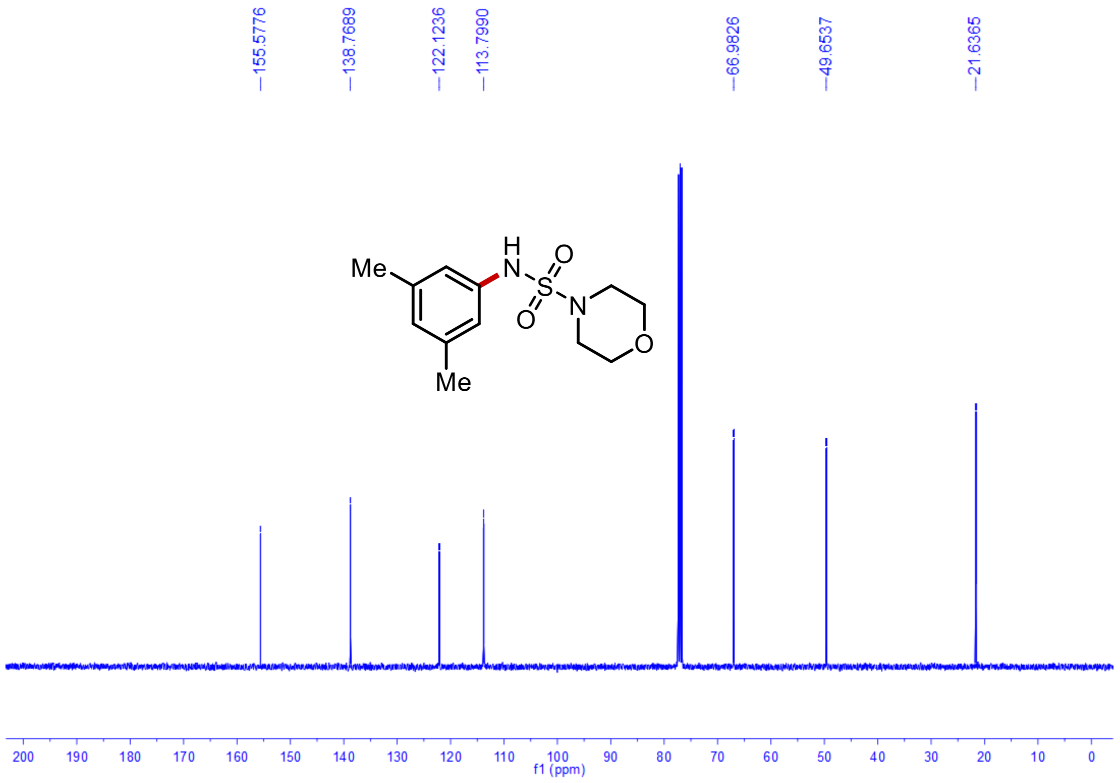

^{13}**C NMR** (100 MHz, $CDCl_3$) Spectrum

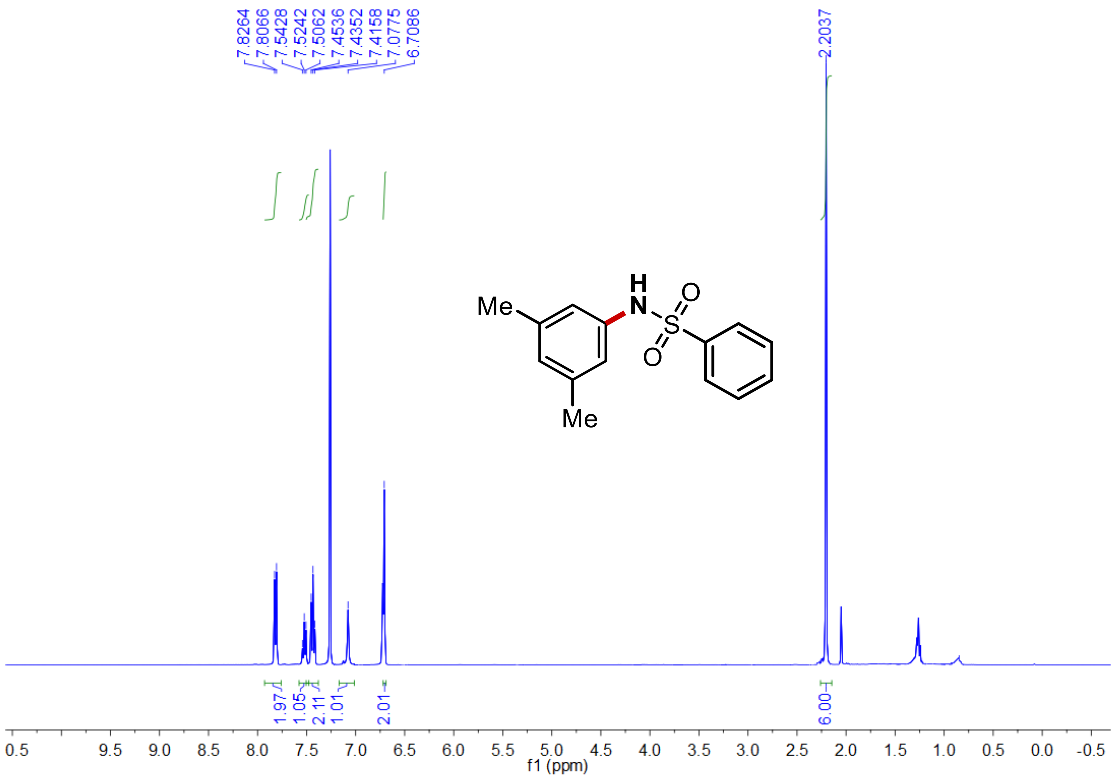

^{1}H NMR (400 MHz, $CDCl_3$) Spectrum

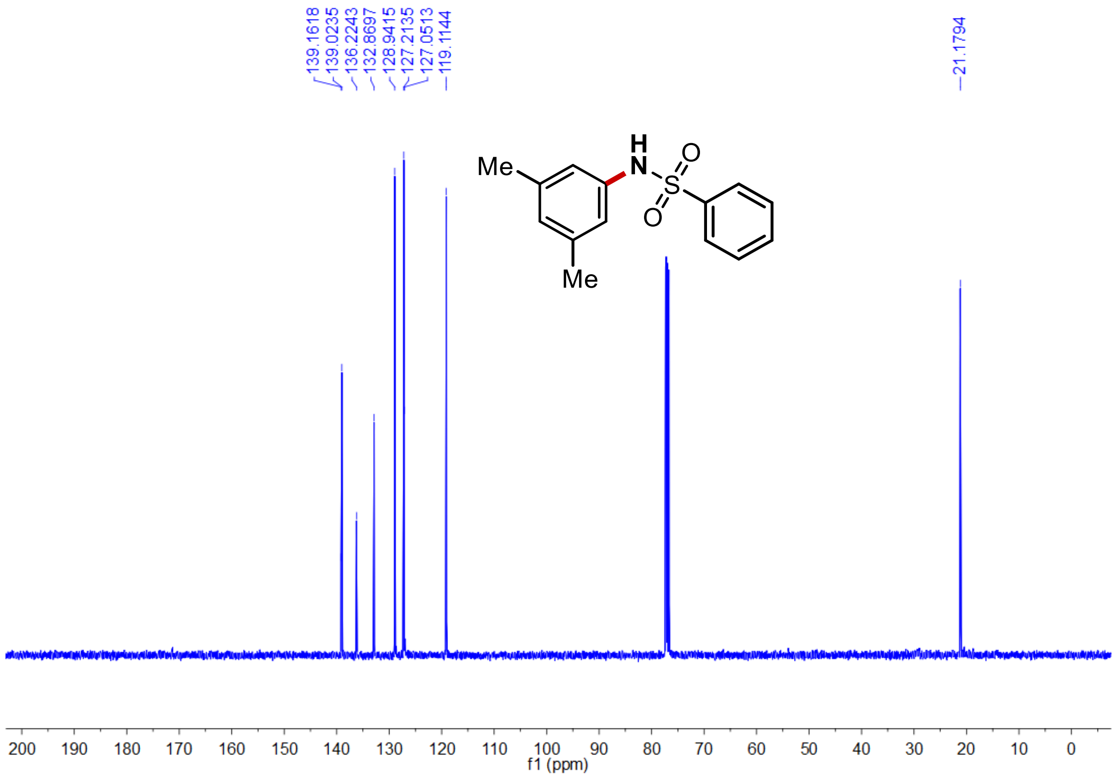

^{13}C NMR (100 MHz, $CDCl_3$) Spectrum

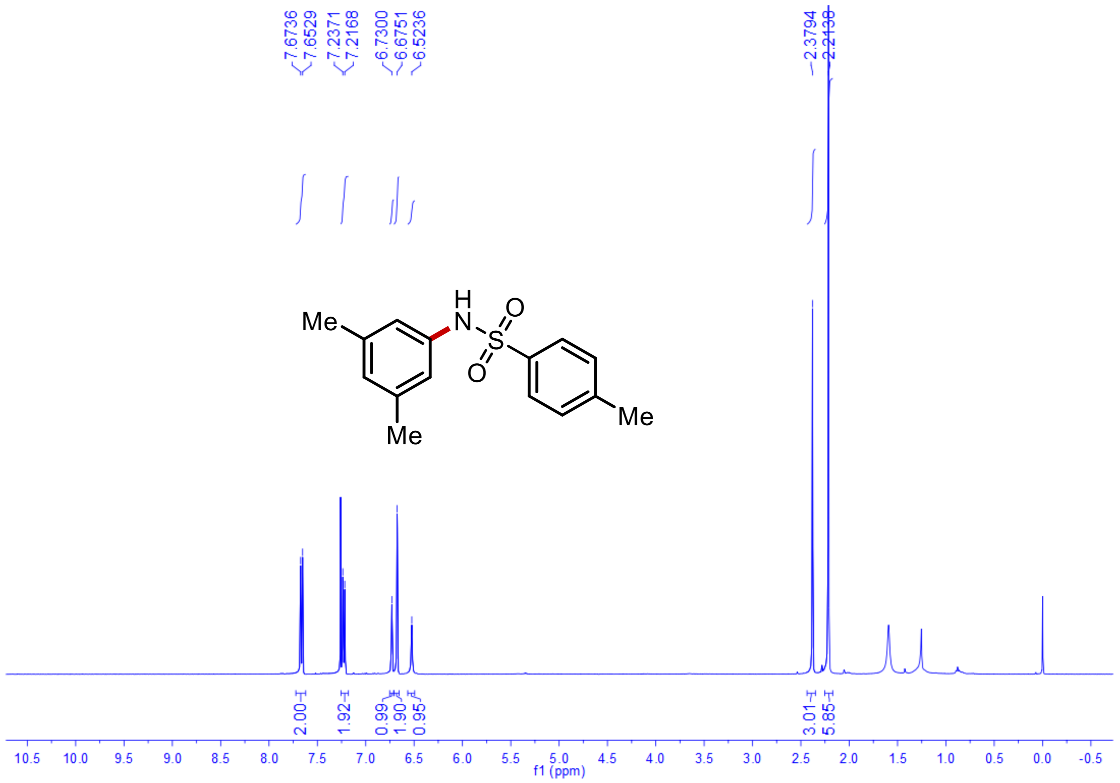

^{1}**H NMR** (400 MHz, $CDCl_3$) Spectrum

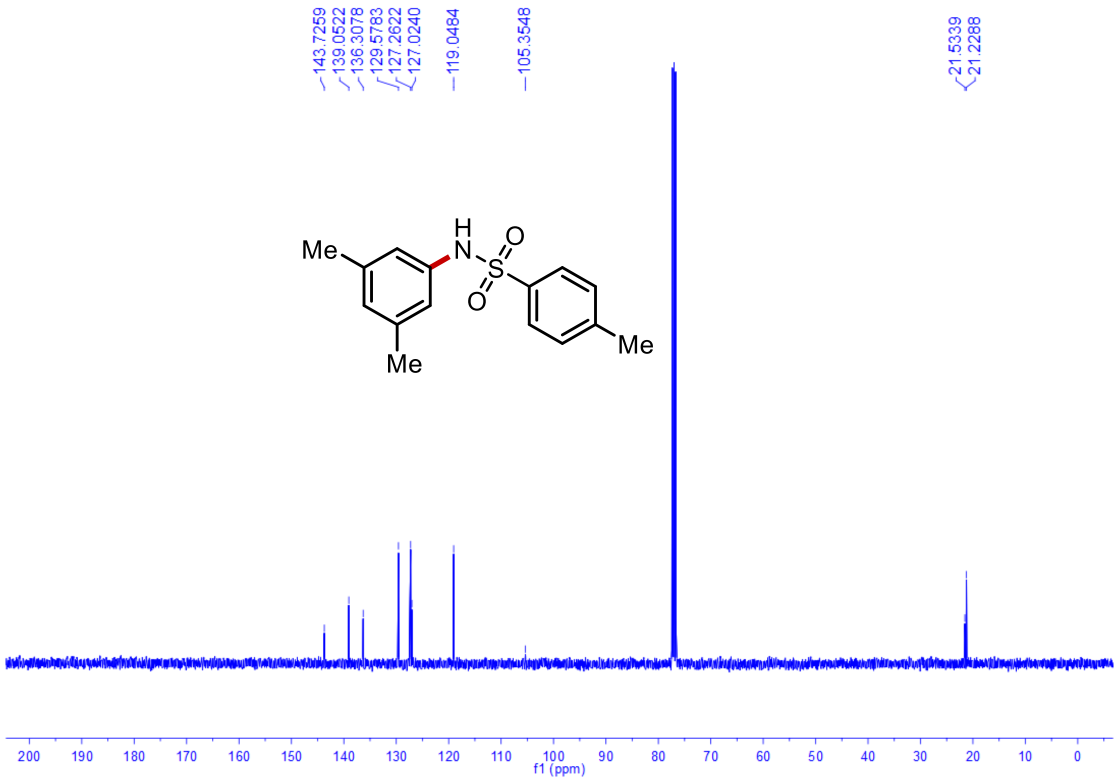

^{13}**C NMR** (100 MHz, $CDCl_3$) Spectrum

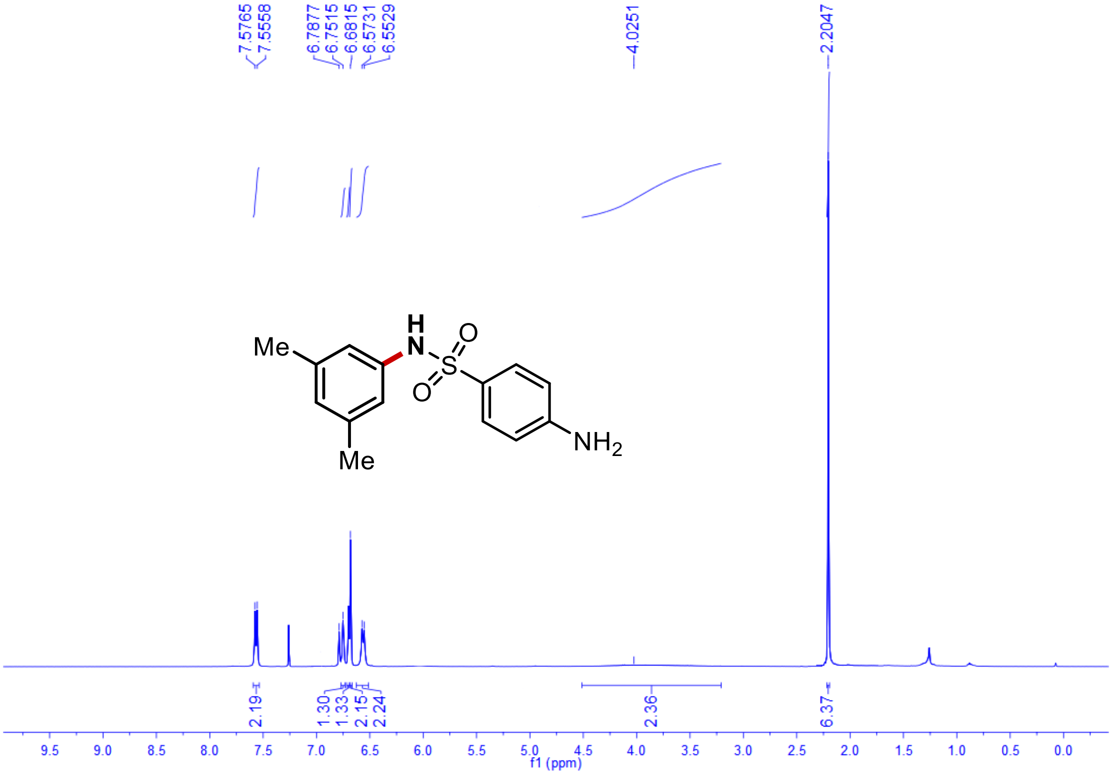

^{1}H NMR (400 MHz, $CDCl_3$) Spectrum

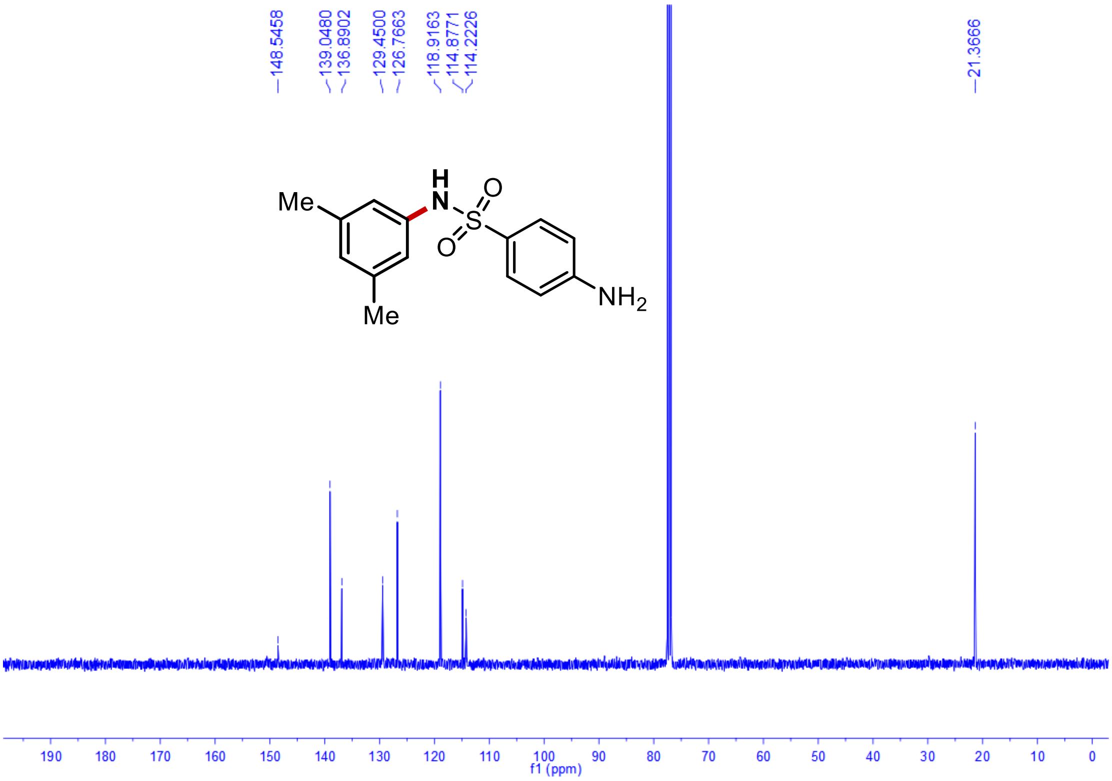

^{13}C NMR (100 MHz, $CDCl_3$) Spectrum

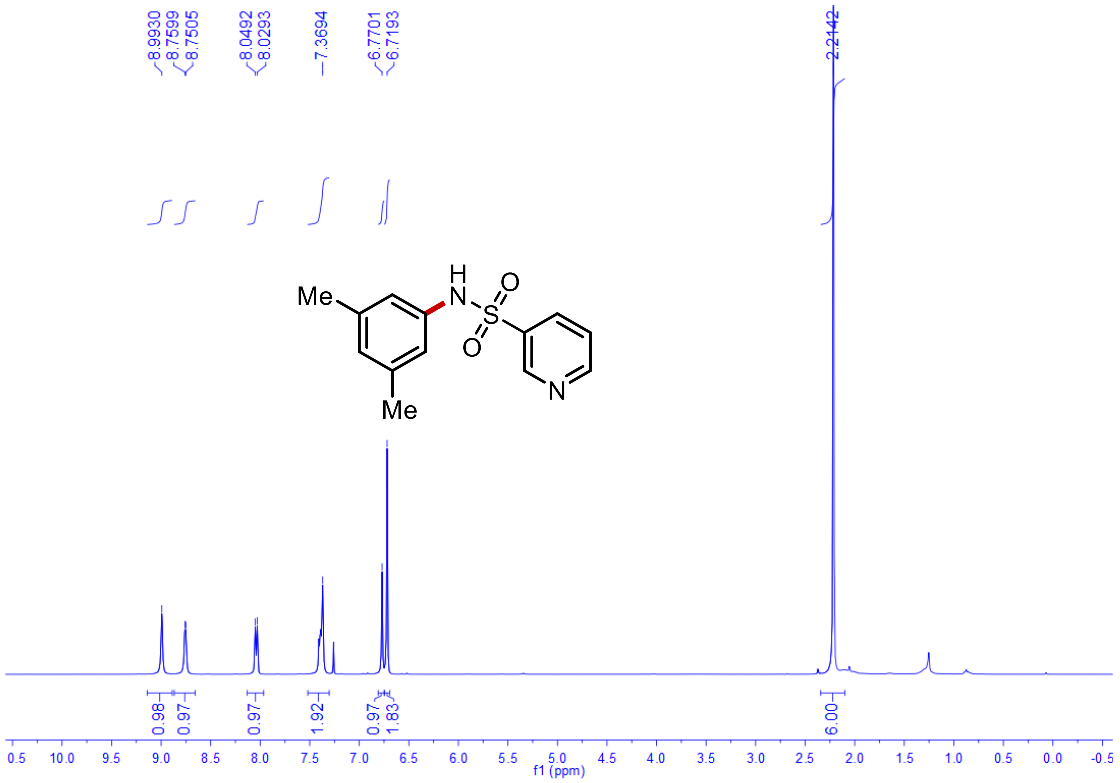

^{1}H NMR (400 MHz, $CDCl_3$) Spectrum

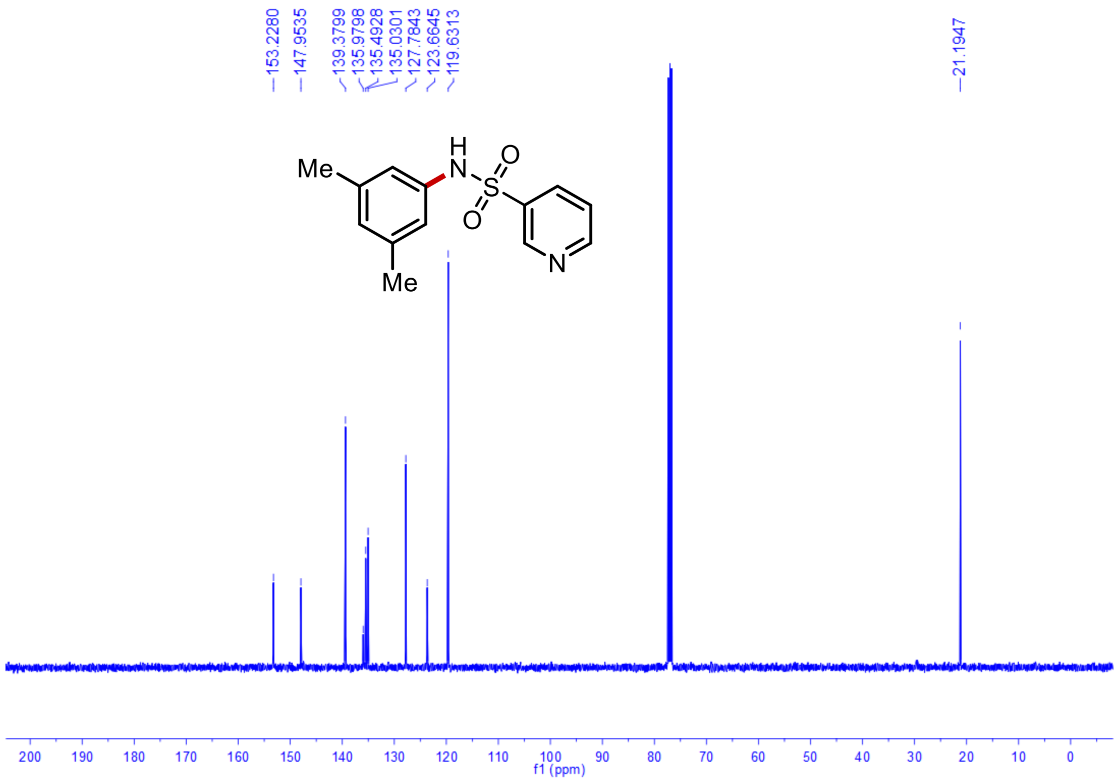

^{13}C NMR (100 MHz, $CDCl_3$) Spectrum

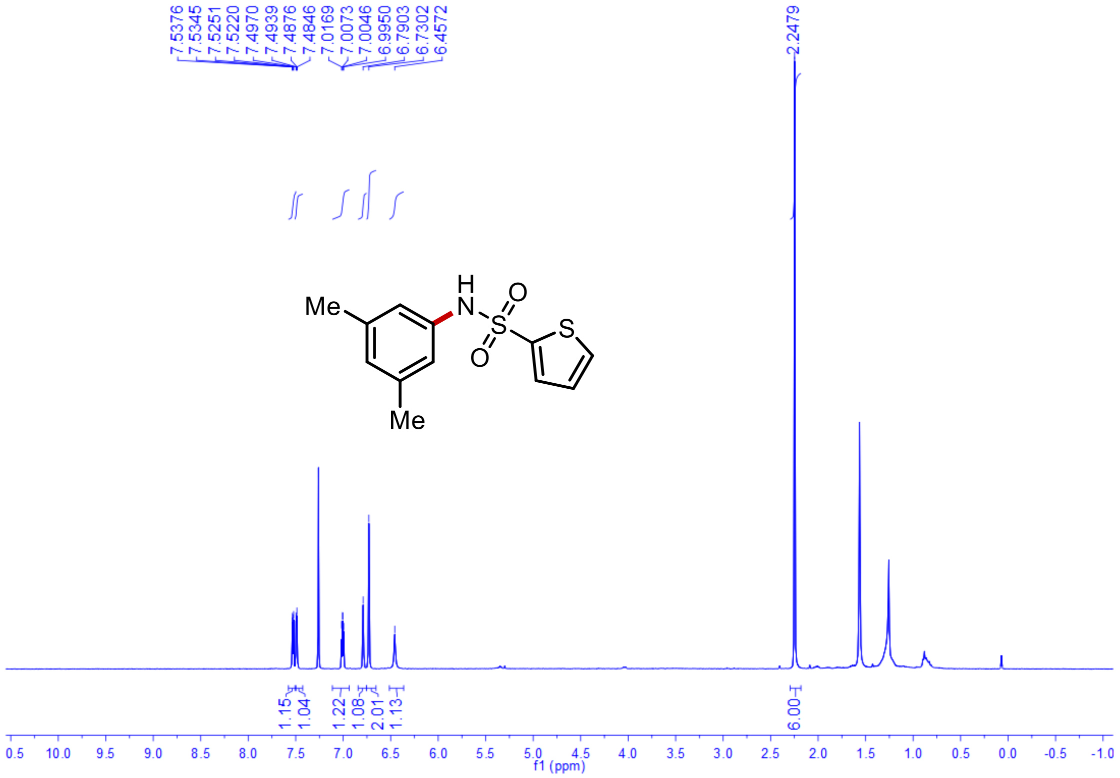

^{1}**H NMR** (400 MHz, $CDCl_3$) Spectrum

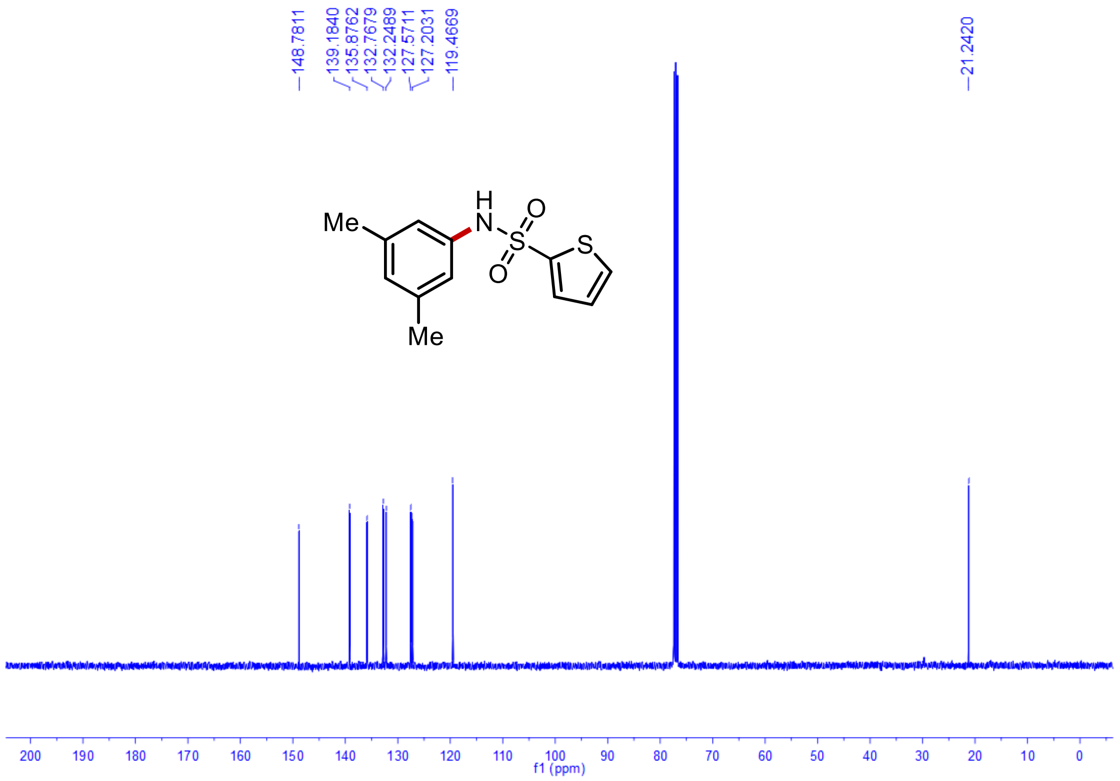

^{13}**C NMR** (100 MHz, $CDCl_3$) Spectrum

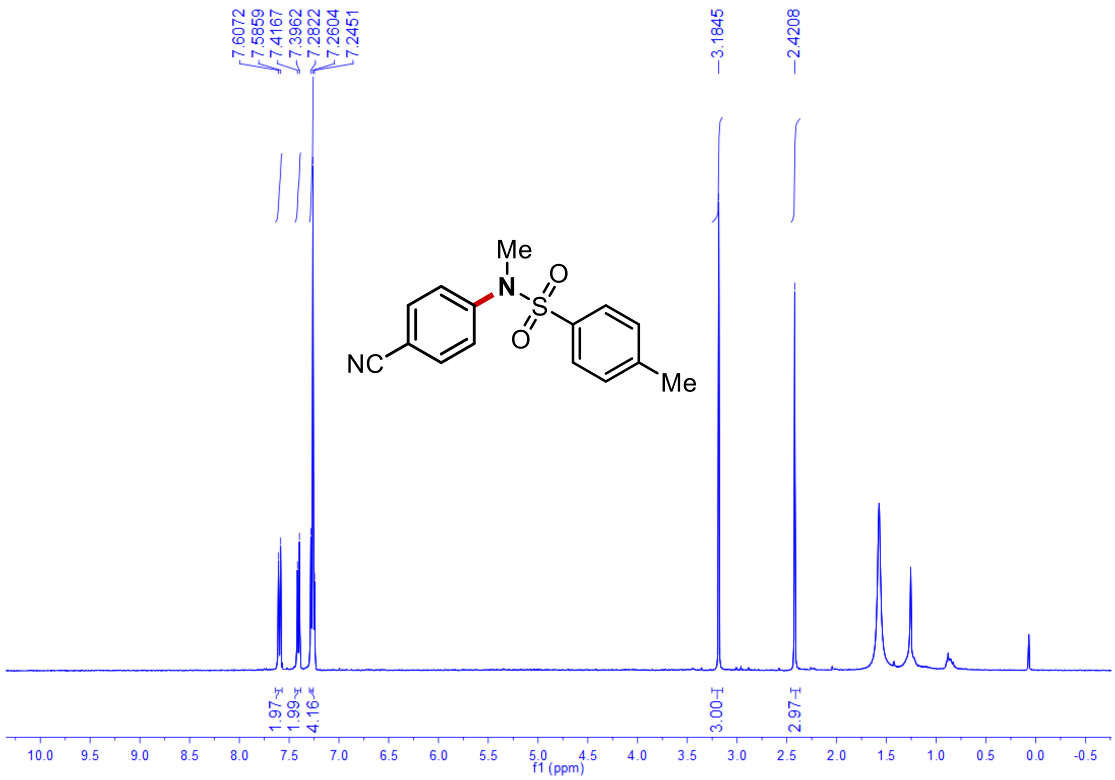

^{1}H NMR (400 MHz, $CDCl_3$) Spectrum

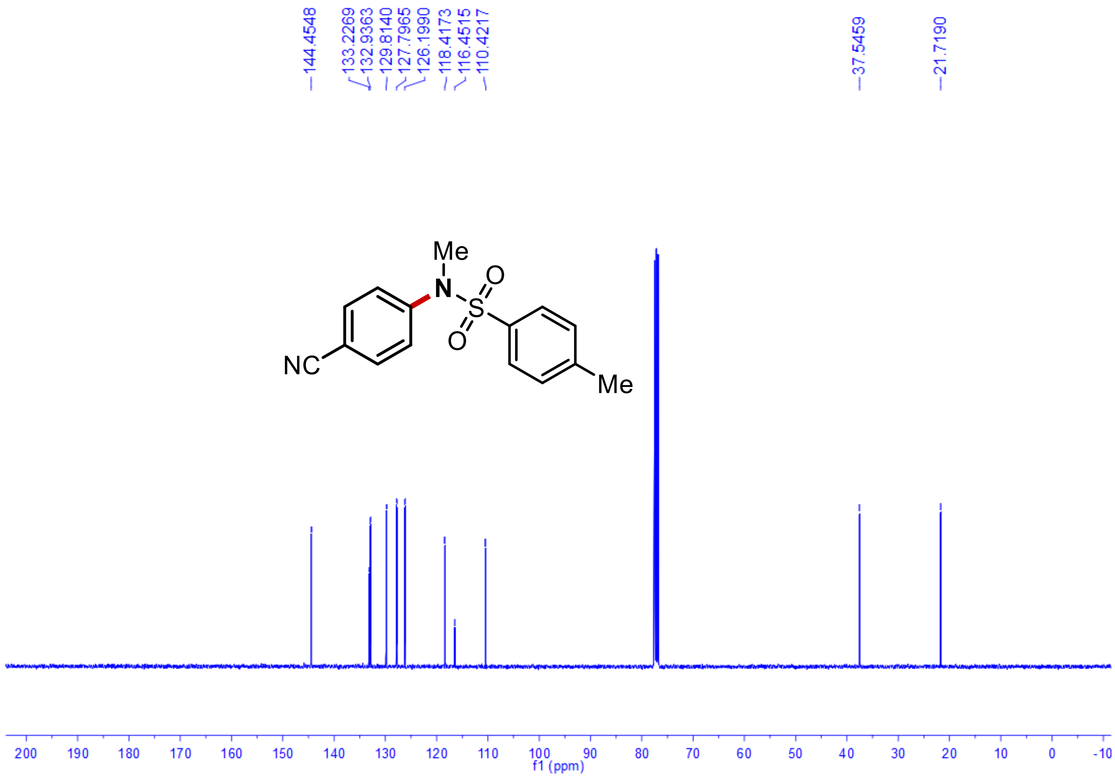

^{13}C NMR (100 MHz, $CDCl_3$) Spectrum

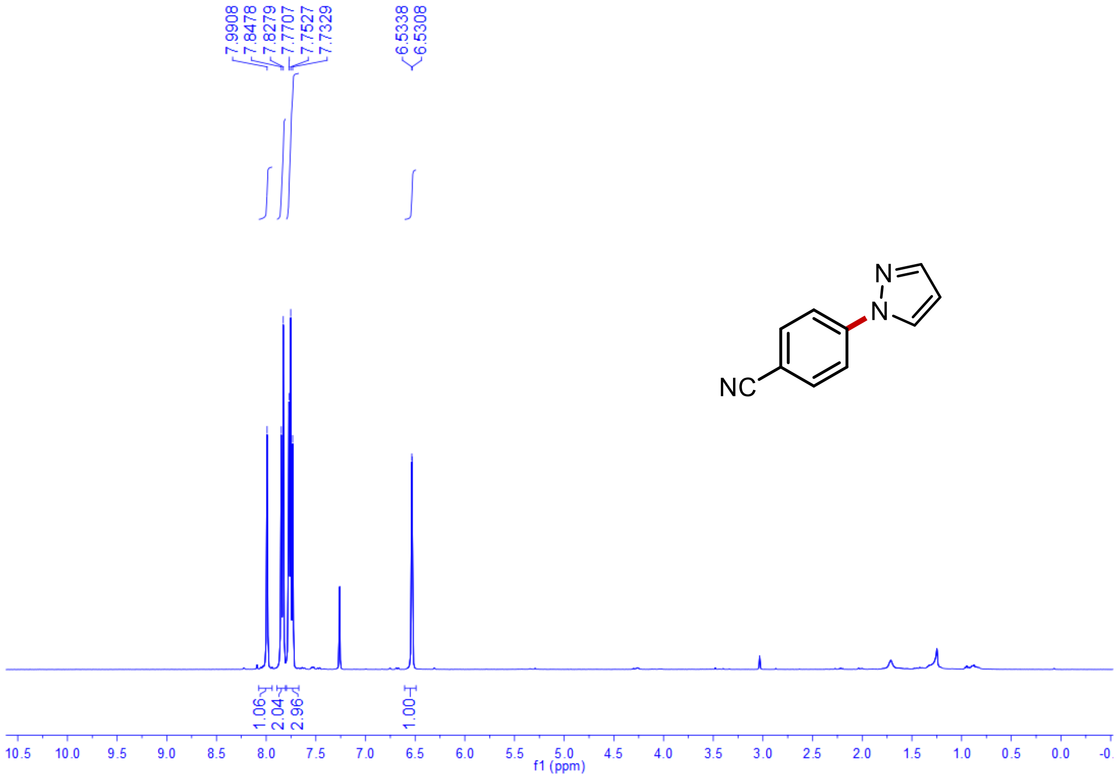

^{1}H NMR (400 MHz, $CDCl_3$) Spectrum

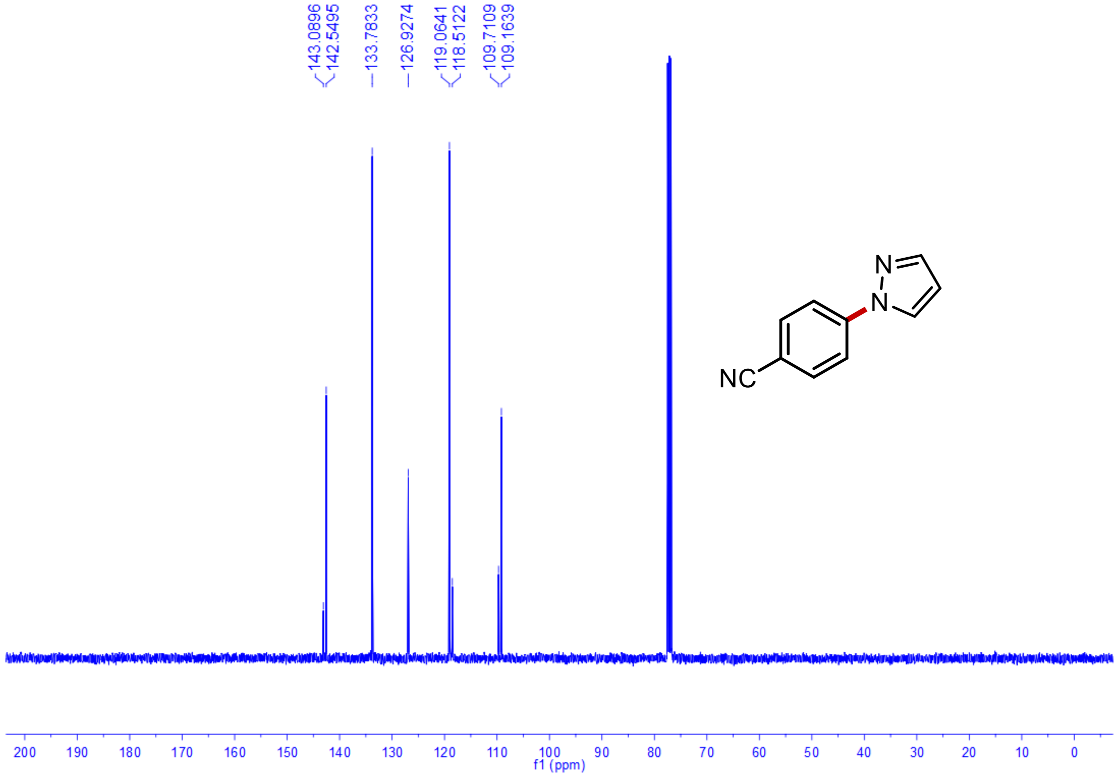

^{13}C NMR (100 MHz, $CDCl_3$) Spectrum

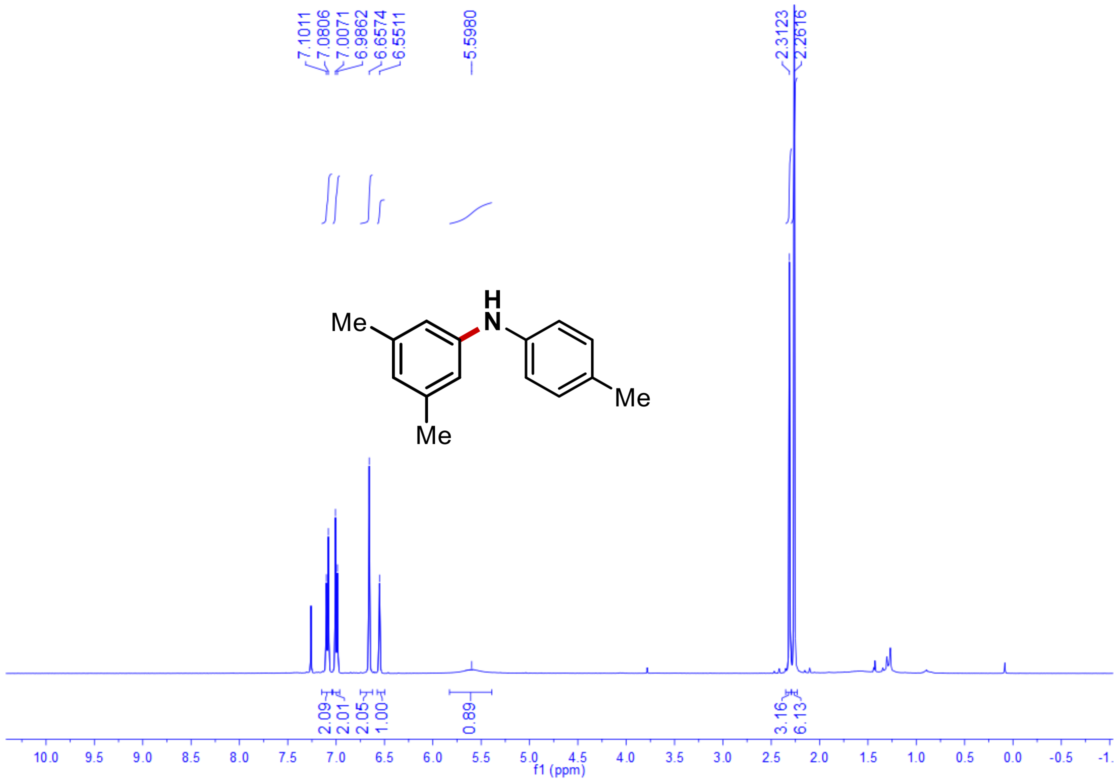

^{1}H NMR (400 MHz, $CDCl_3$) Spectrum

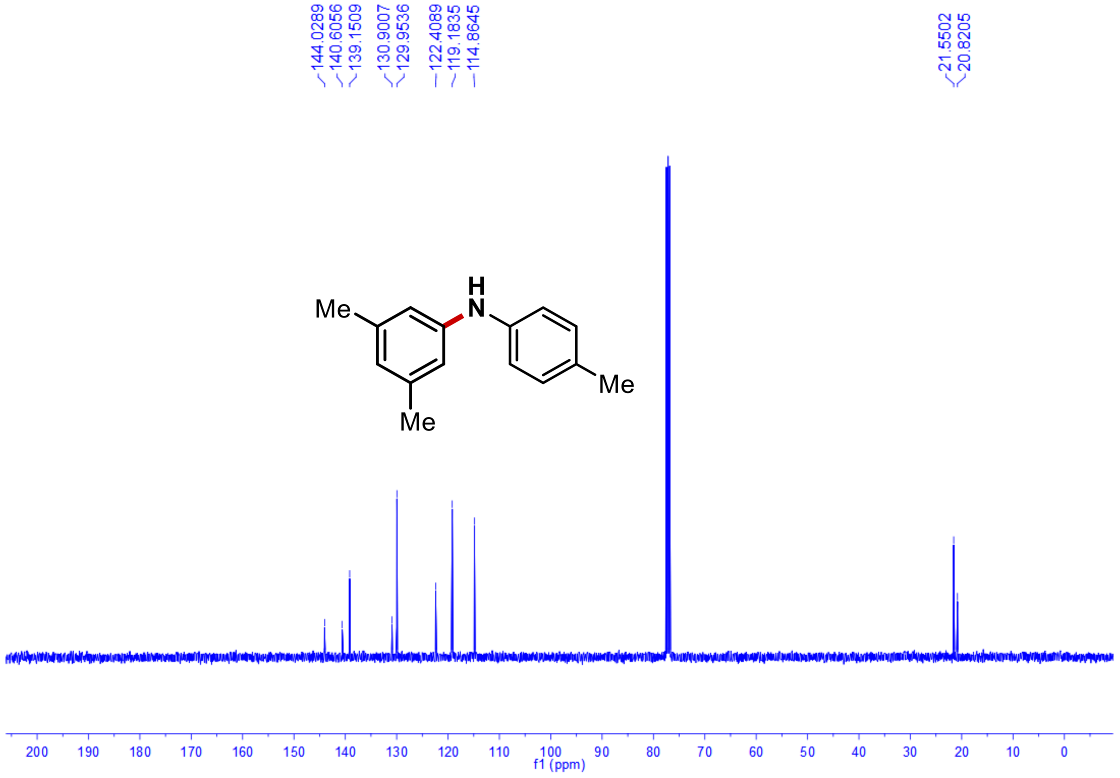

^{13}C NMR (100 MHz, $CDCl_3$) Spectrum

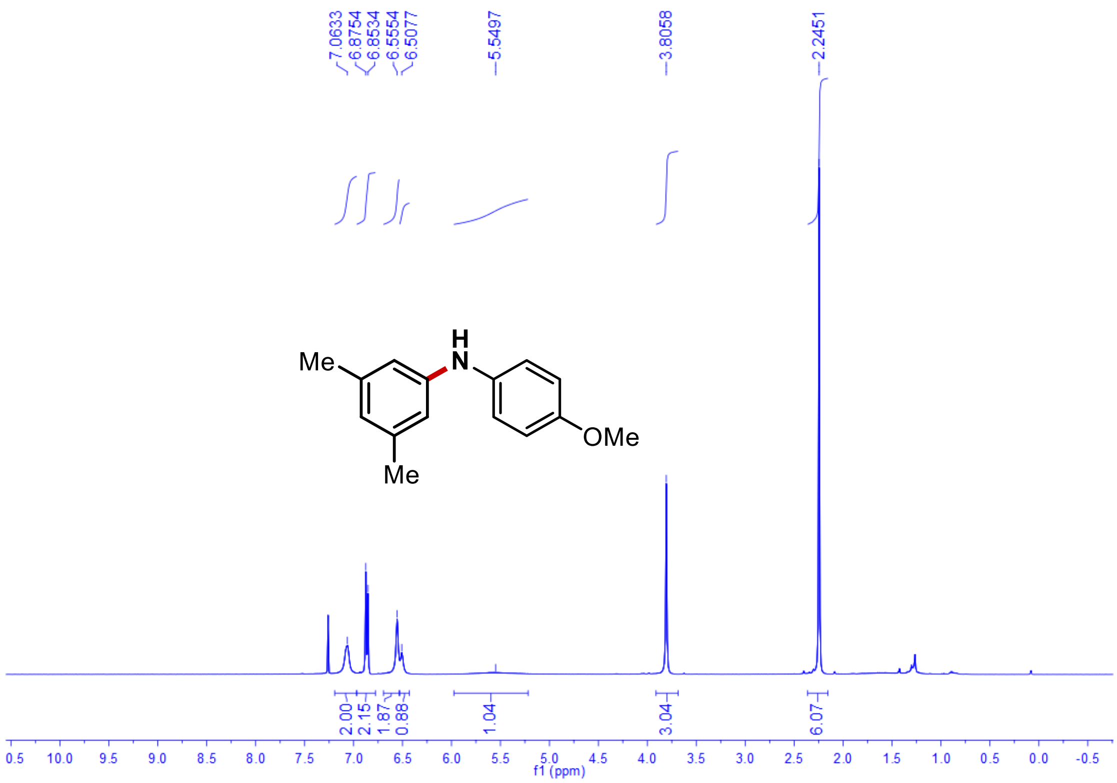

^{1}**H NMR** (400 MHz, $CDCl_3$) Spectrum

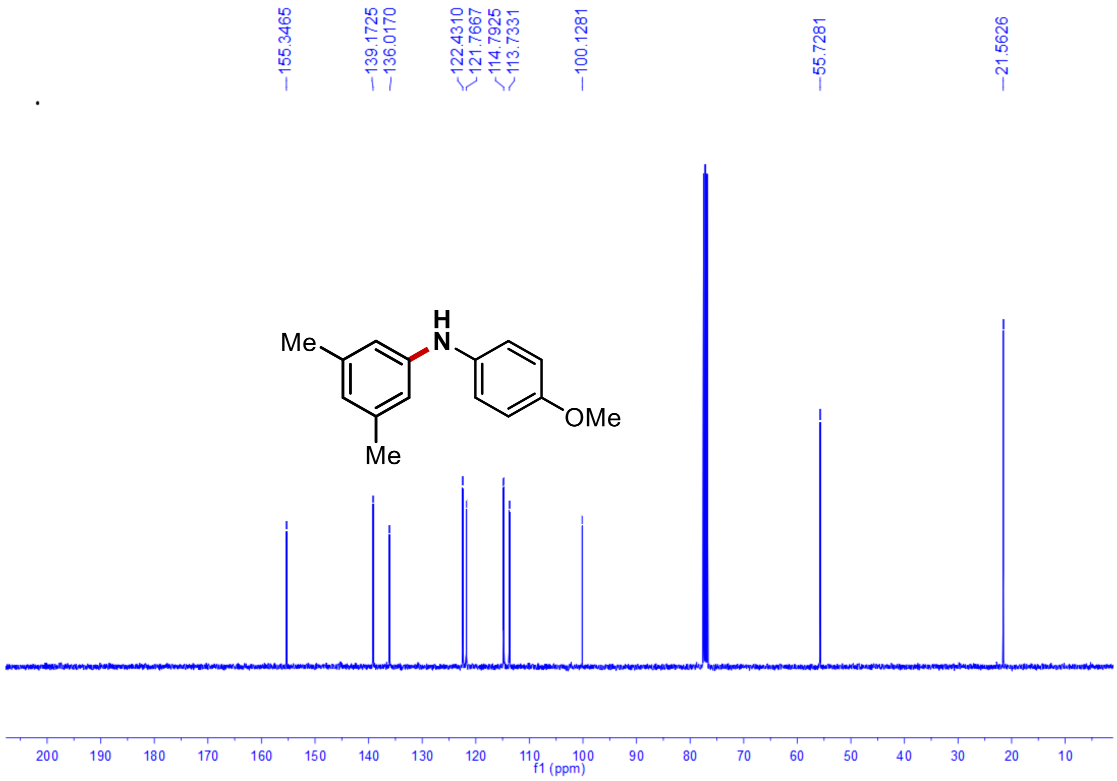

^{13}**C NMR** (100 MHz, $CDCl_3$) Spectrum

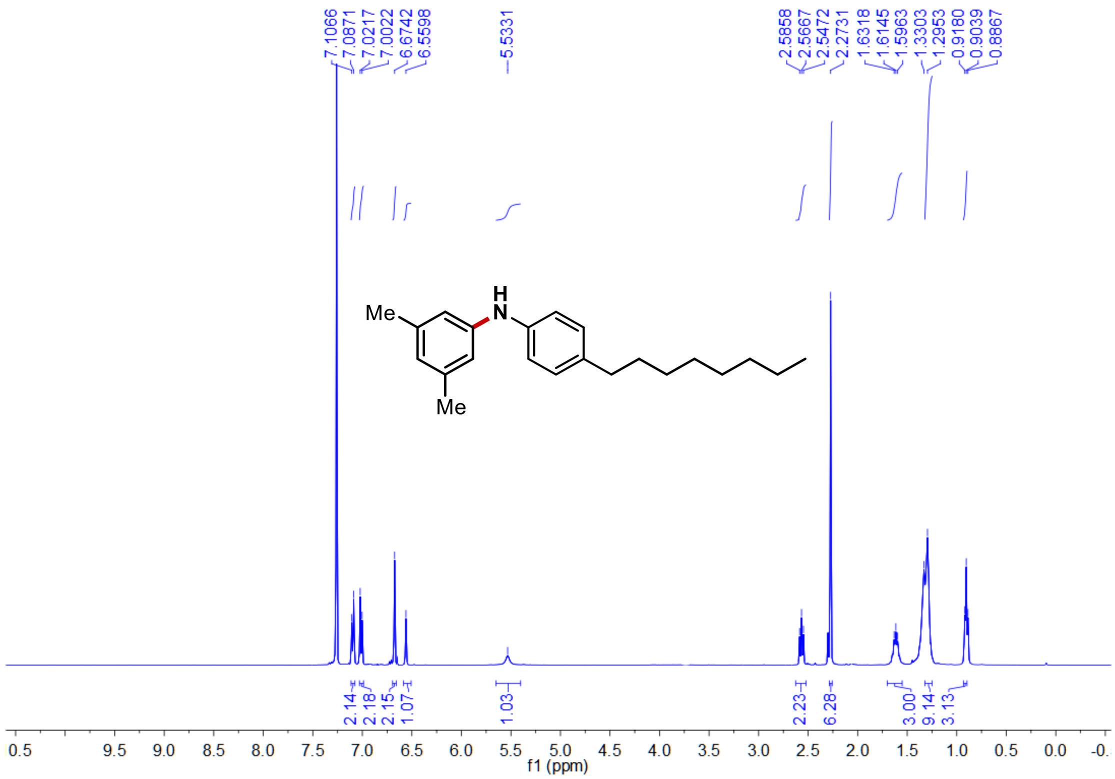

^{1}H NMR (400 MHz, $CDCl_3$) Spectrum

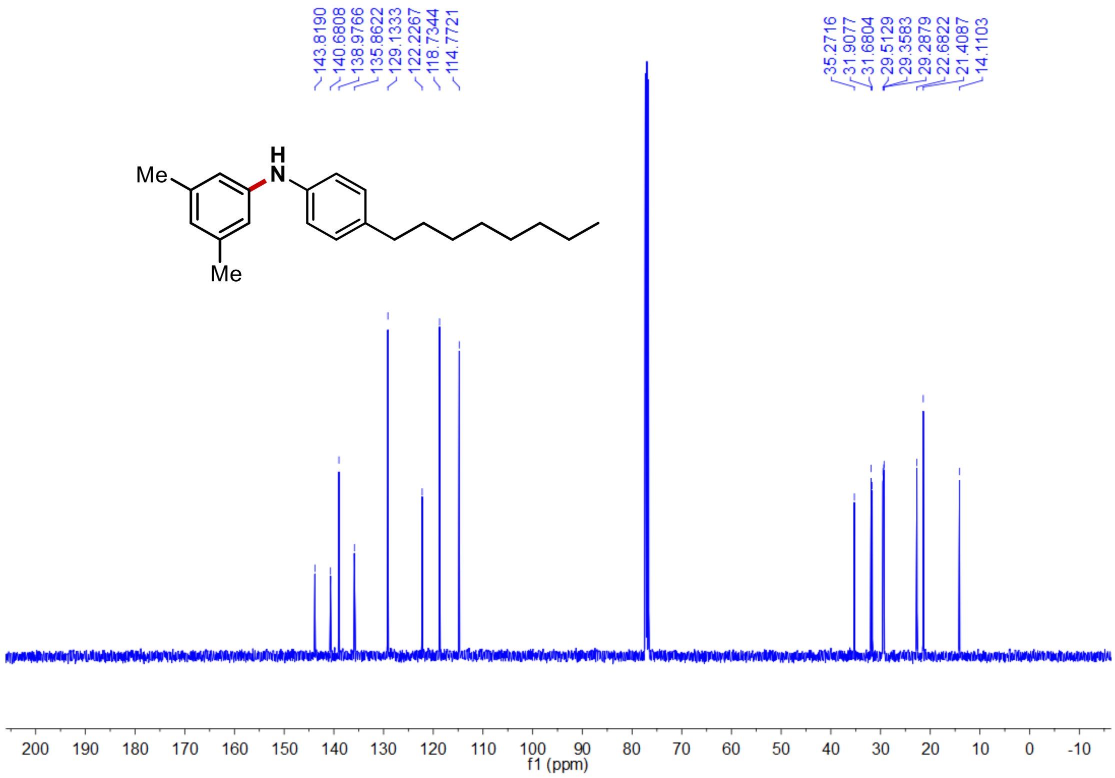

^{13}C NMR (100 MHz, $CDCl_3$) Spectrum

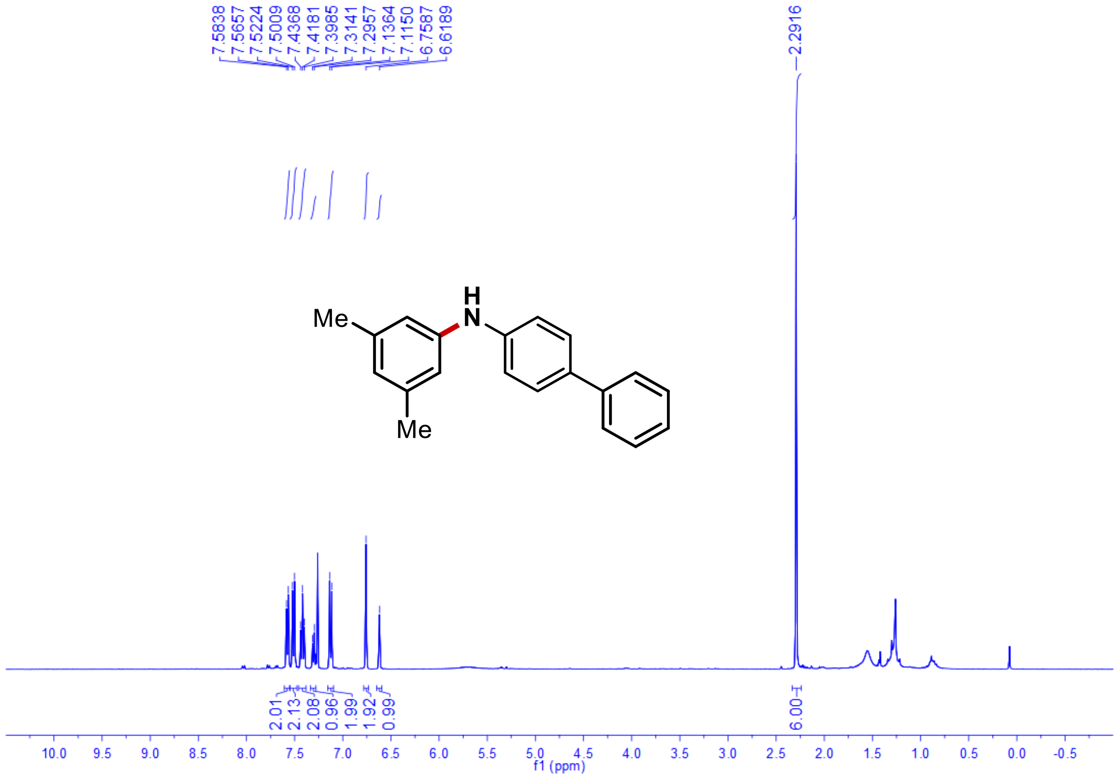

^{1}H NMR (400 MHz, $CDCl_3$) Spectrum

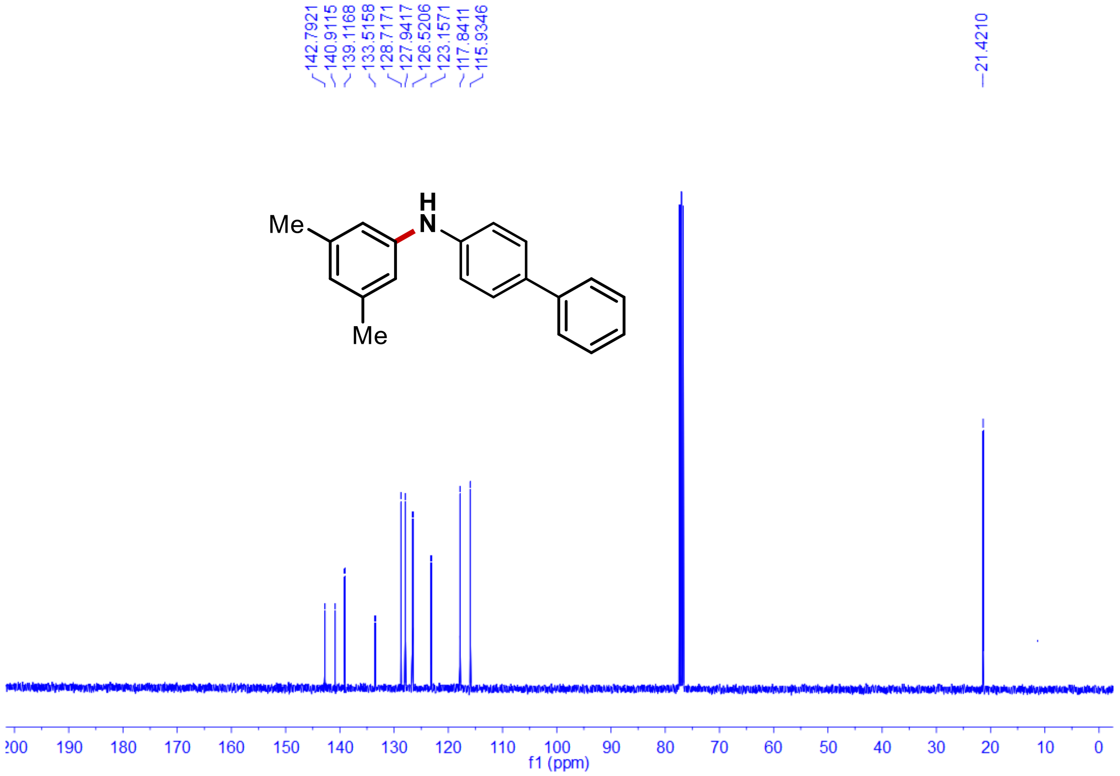

^{13}C NMR (100 MHz, $CDCl_3$) Spectrum

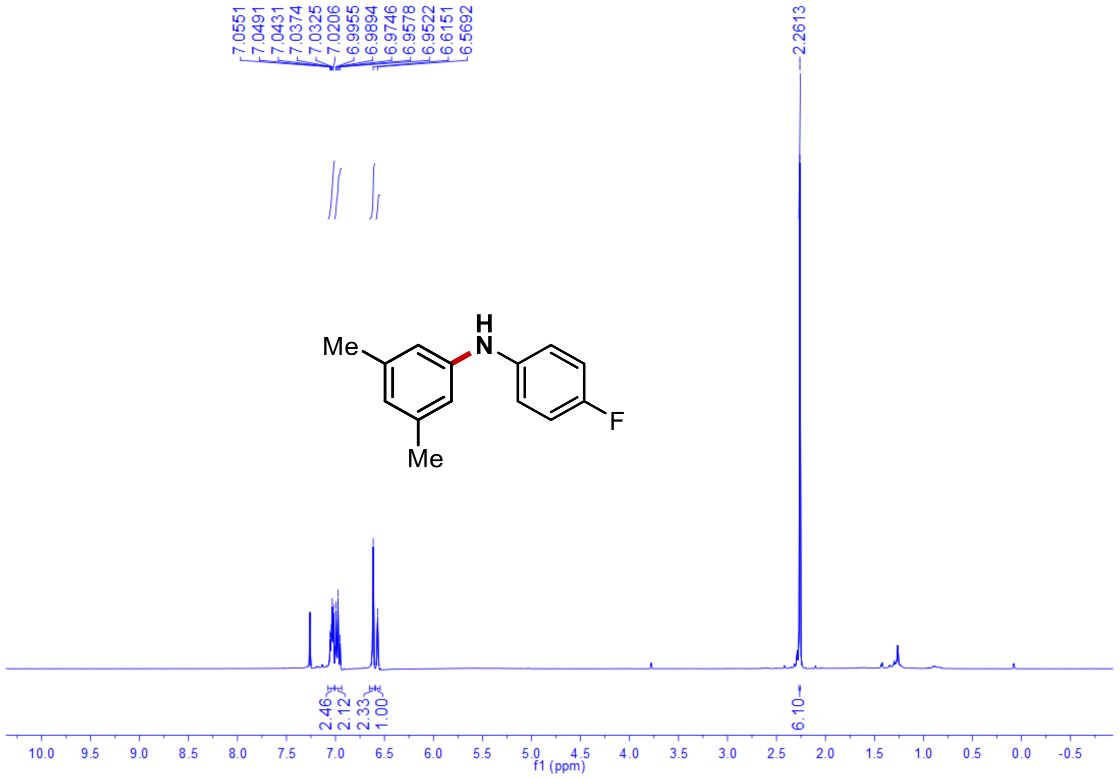

^{1}H NMR (400 MHz, $CDCl_3$) Spectrum

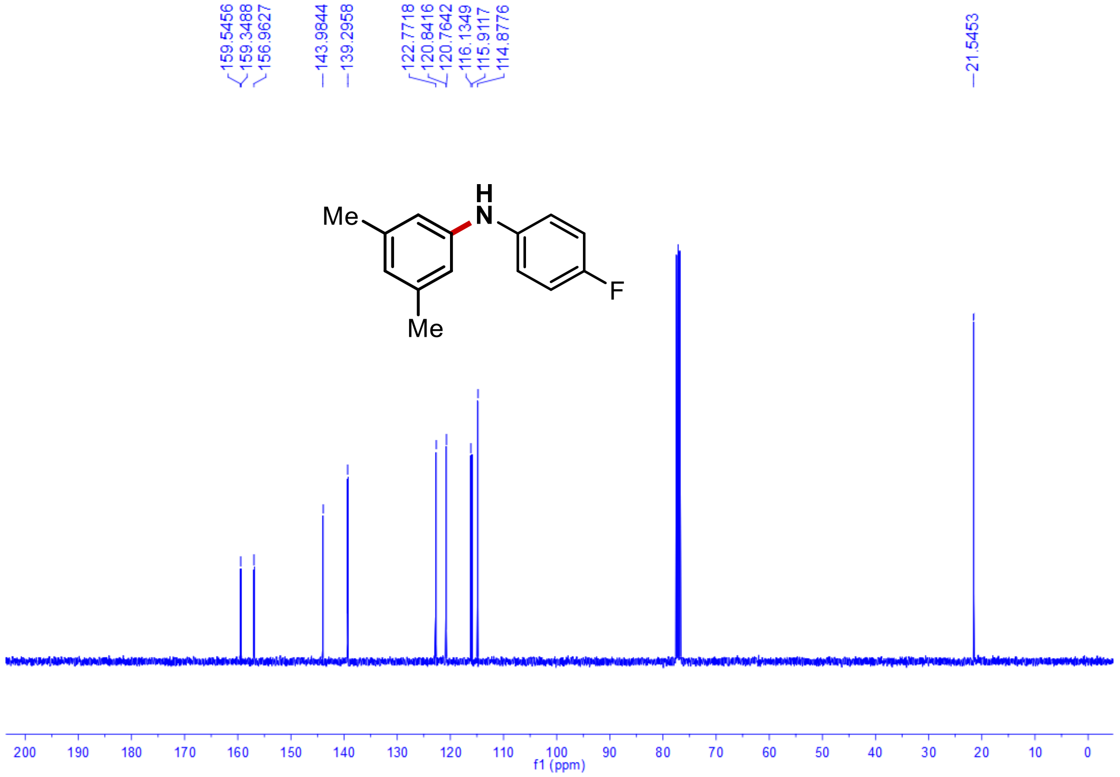

^{13}C NMR (100 MHz, $CDCl_3$) Spectrum

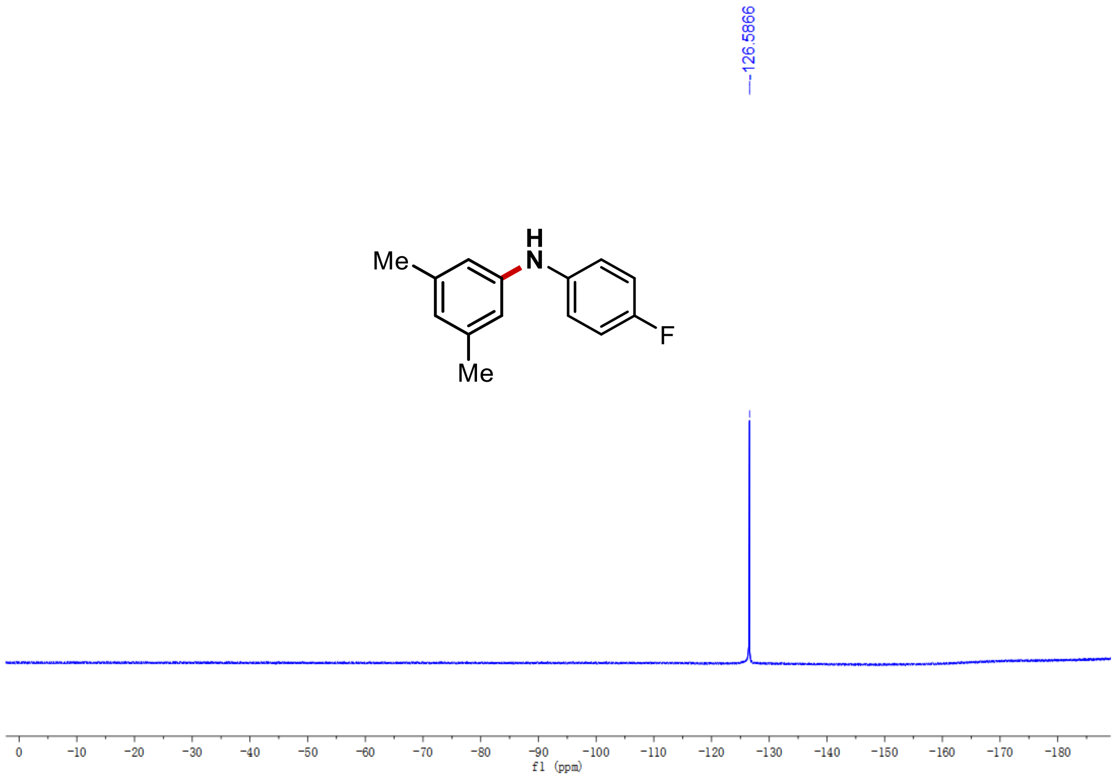

^{19}F NMR (376 MHz, $CDCl_3$) Spectrum

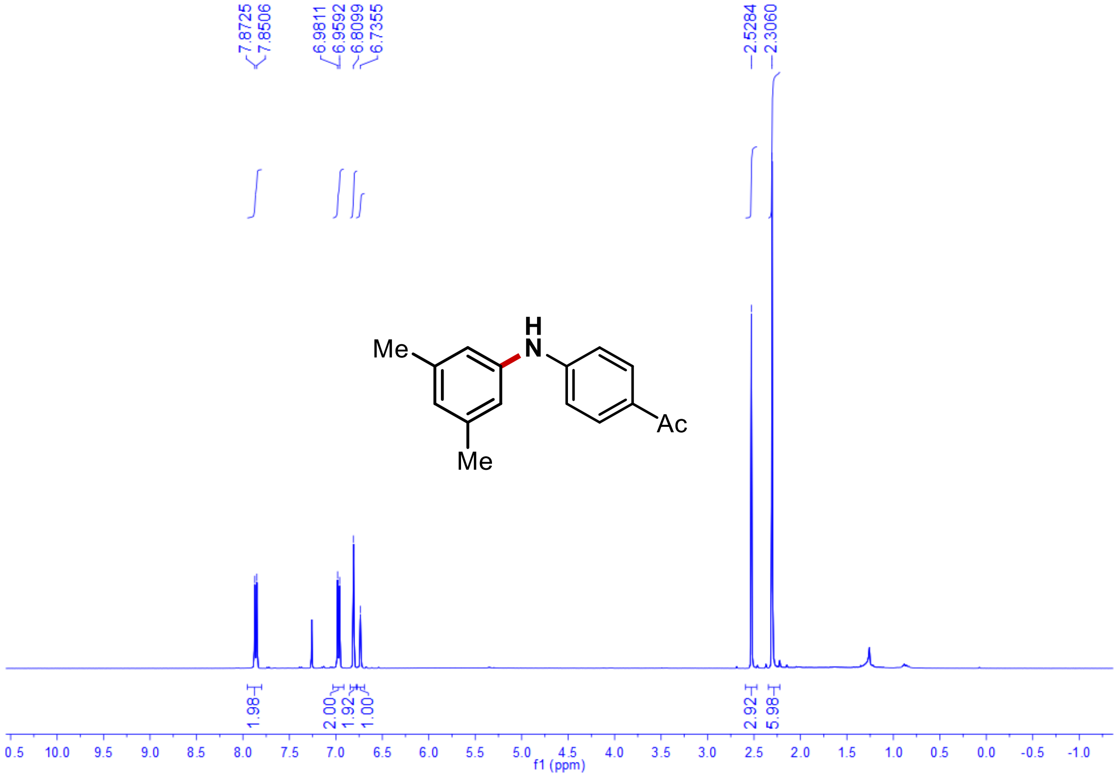

^{1}H NMR (400 MHz, $CDCl_3$) Spectrum

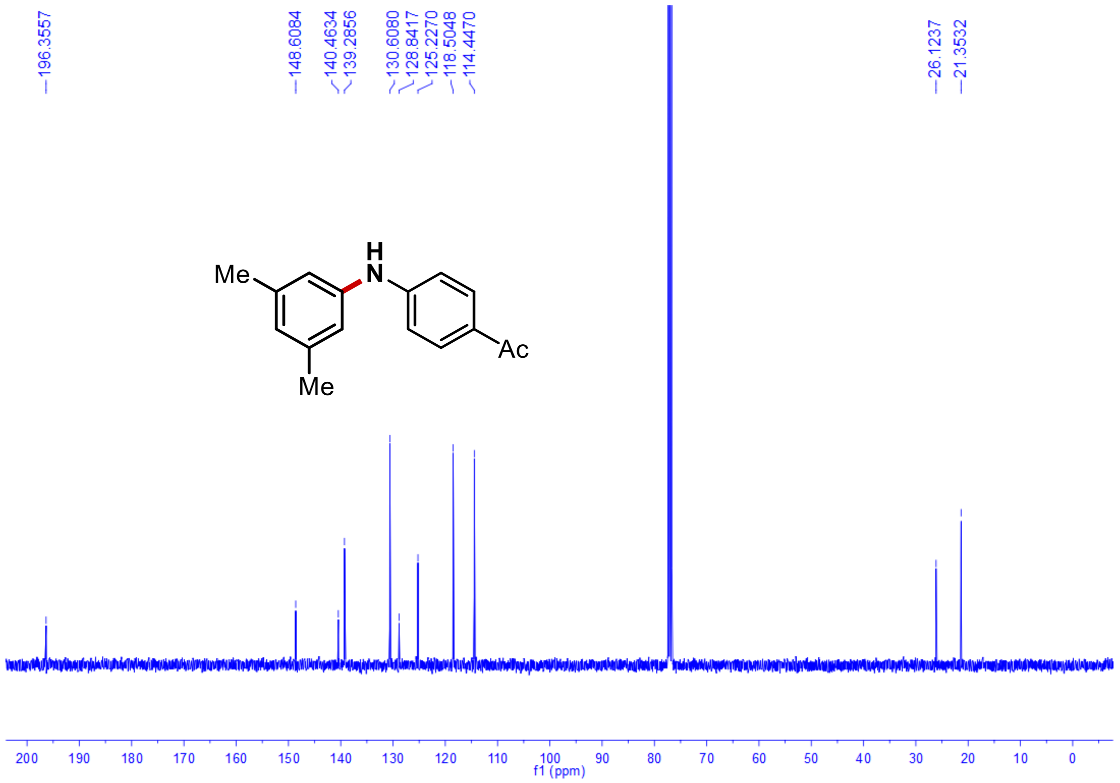

^{13}C NMR (100 MHz, $CDCl_3$) Spectrum

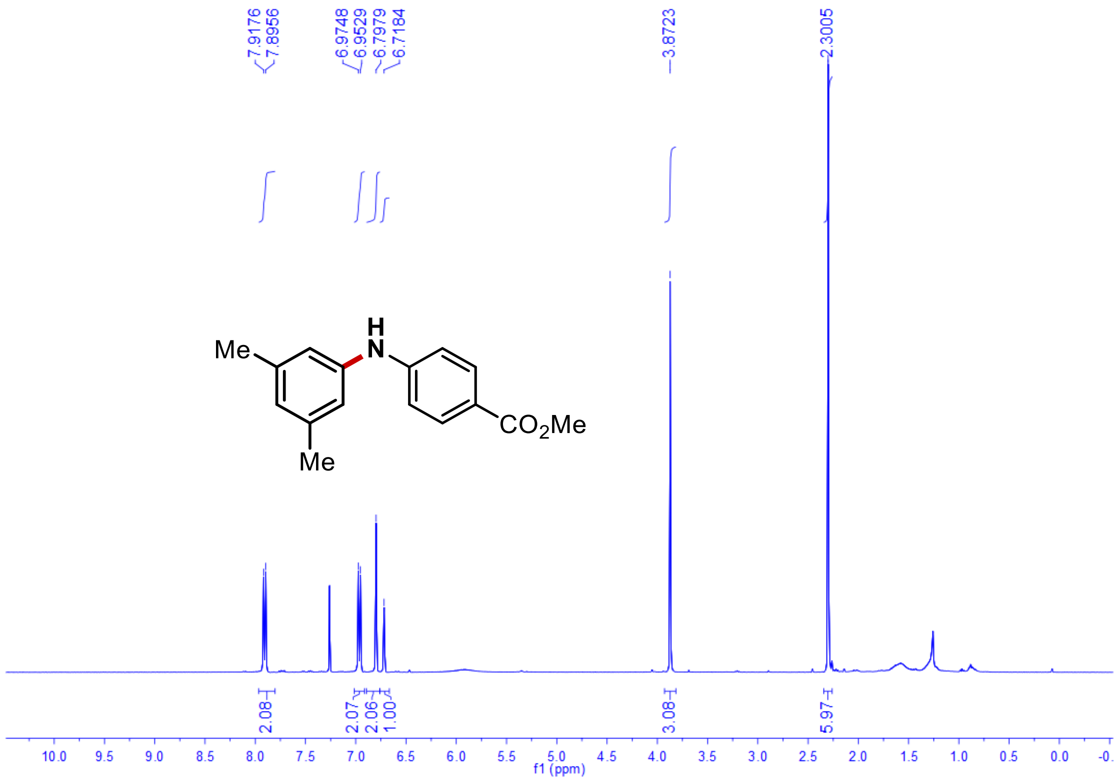

^{1}H NMR (400 MHz, $CDCl_3$) Spectrum

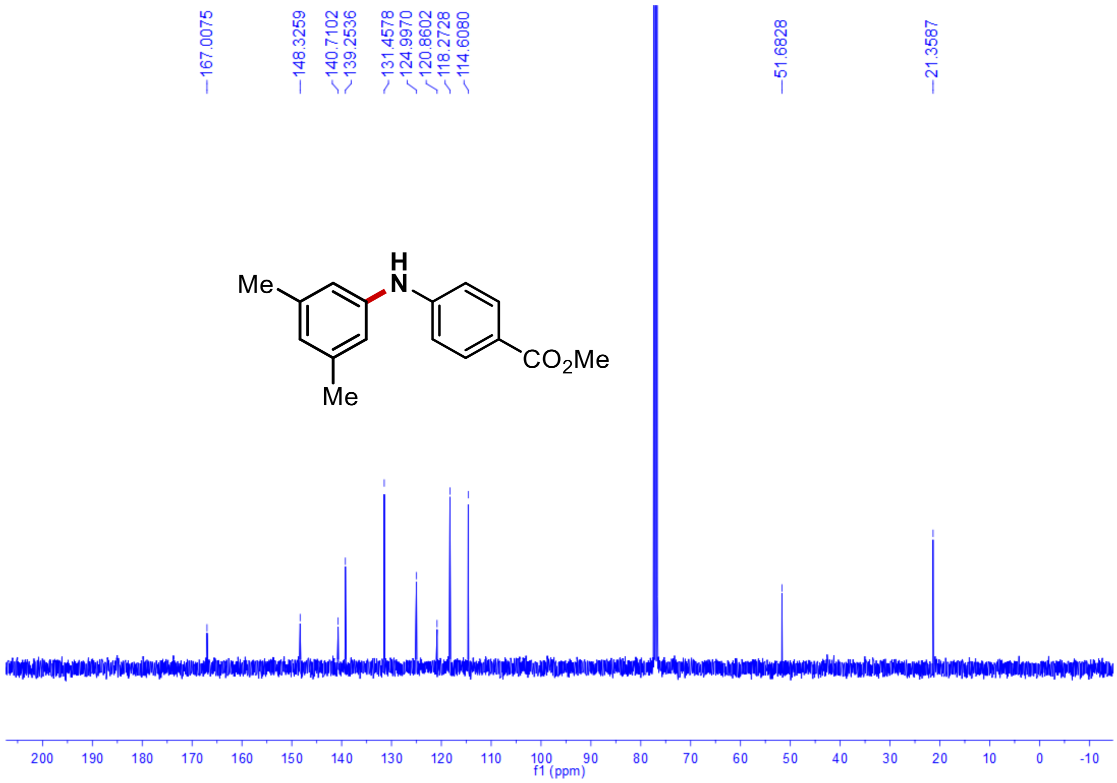

^{13}**C NMR** (100 MHz, $CDCl_3$) Spectrum

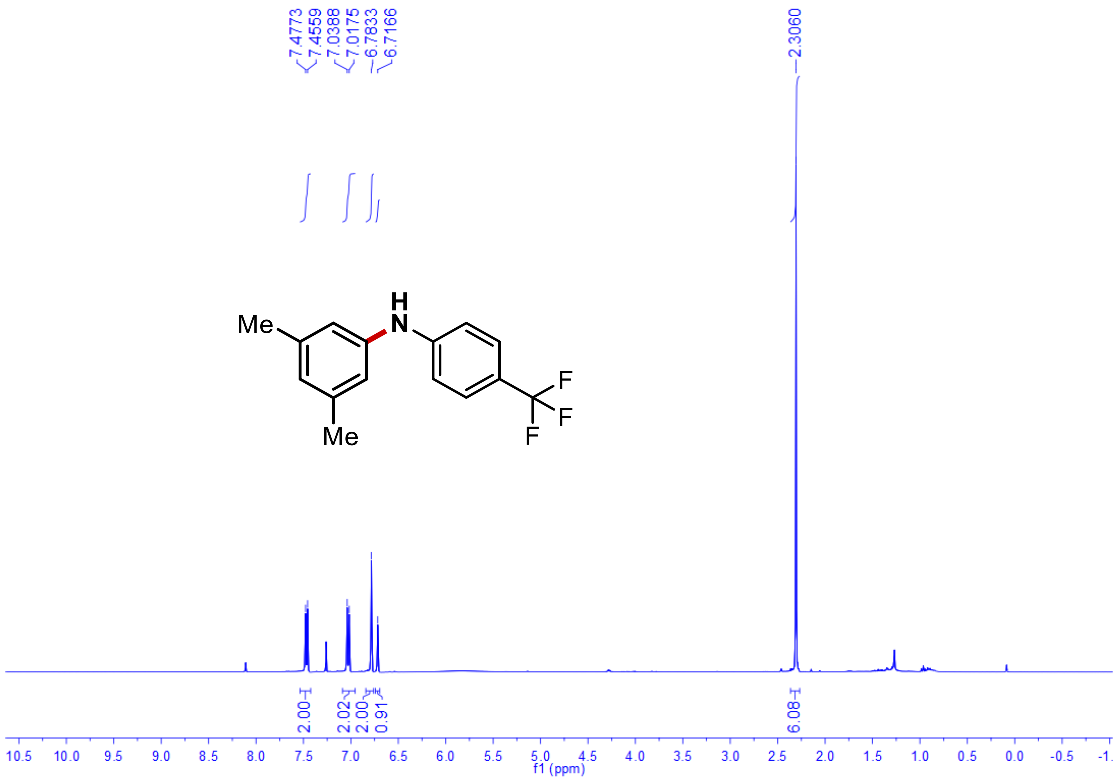

^{1}**H NMR** (400 MHz, $CDCl_3$) Spectrum

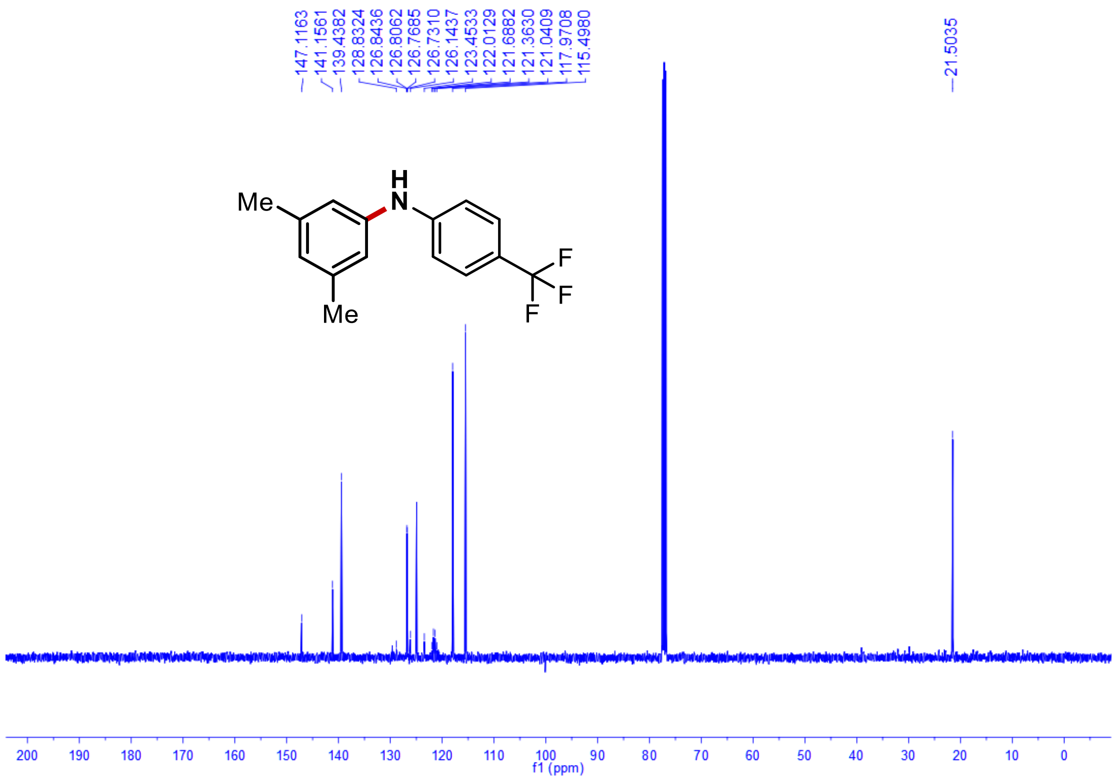

^{13}C NMR (100 MHz, $CDCl_3$) Spectrum

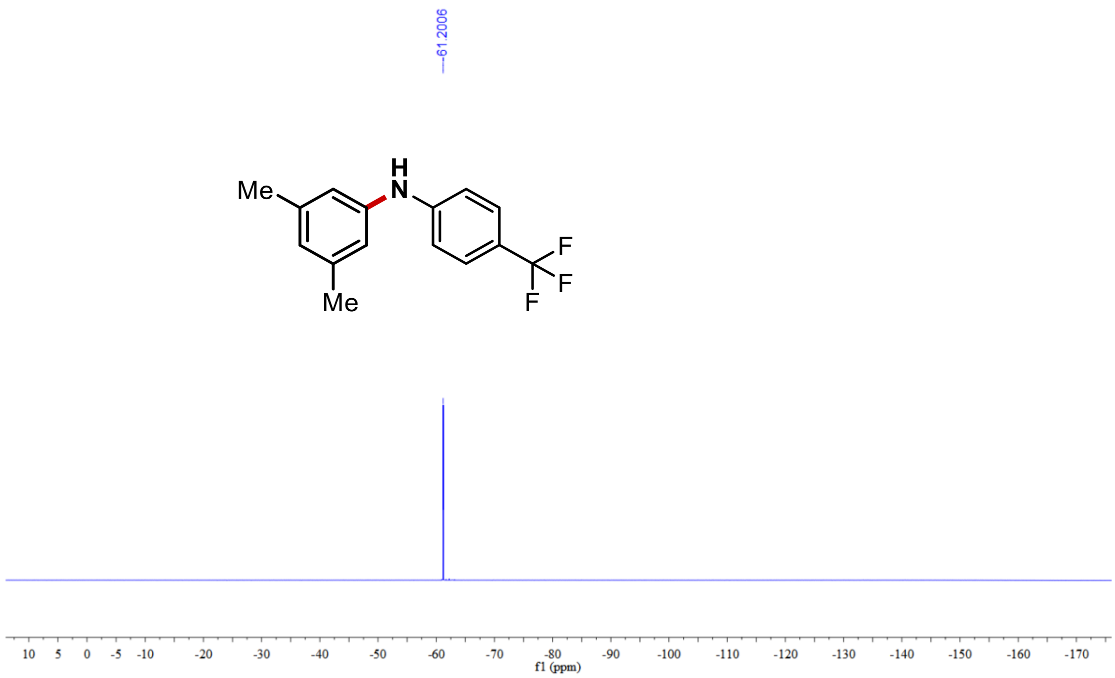

^{19}F NMR (376 MHz, $CDCl_3$) Spectrum

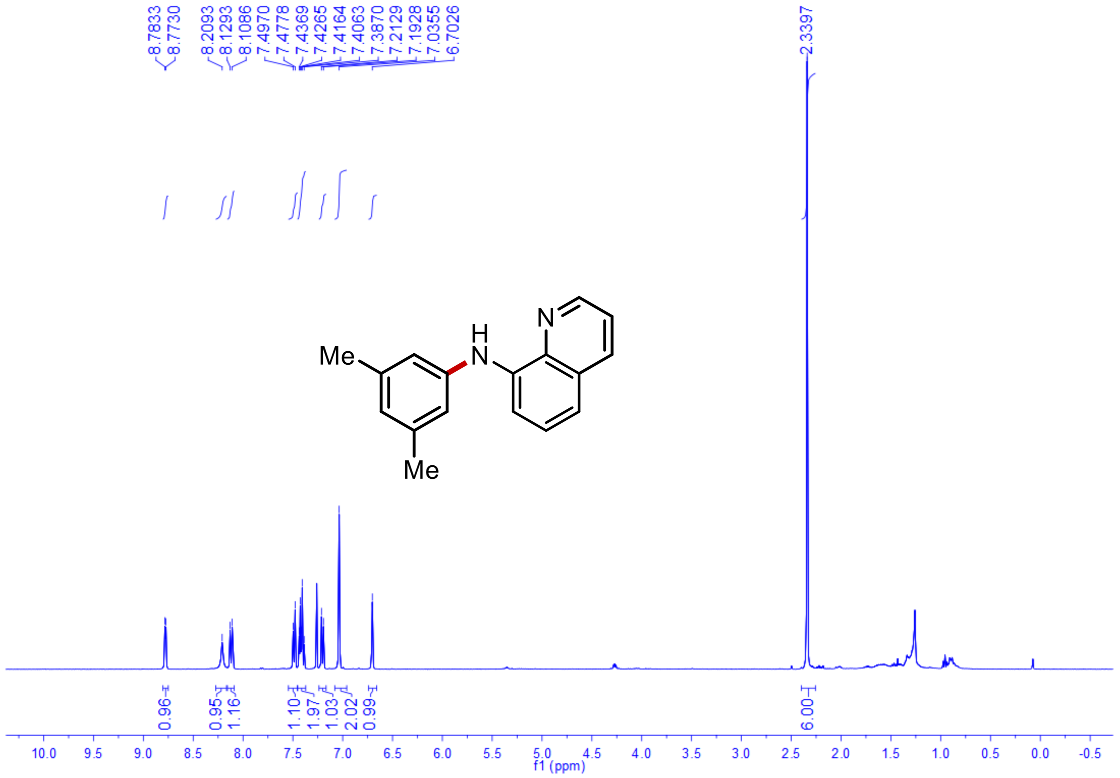

^{1}H NMR (400 MHz, $CDCl_3$) Spectrum

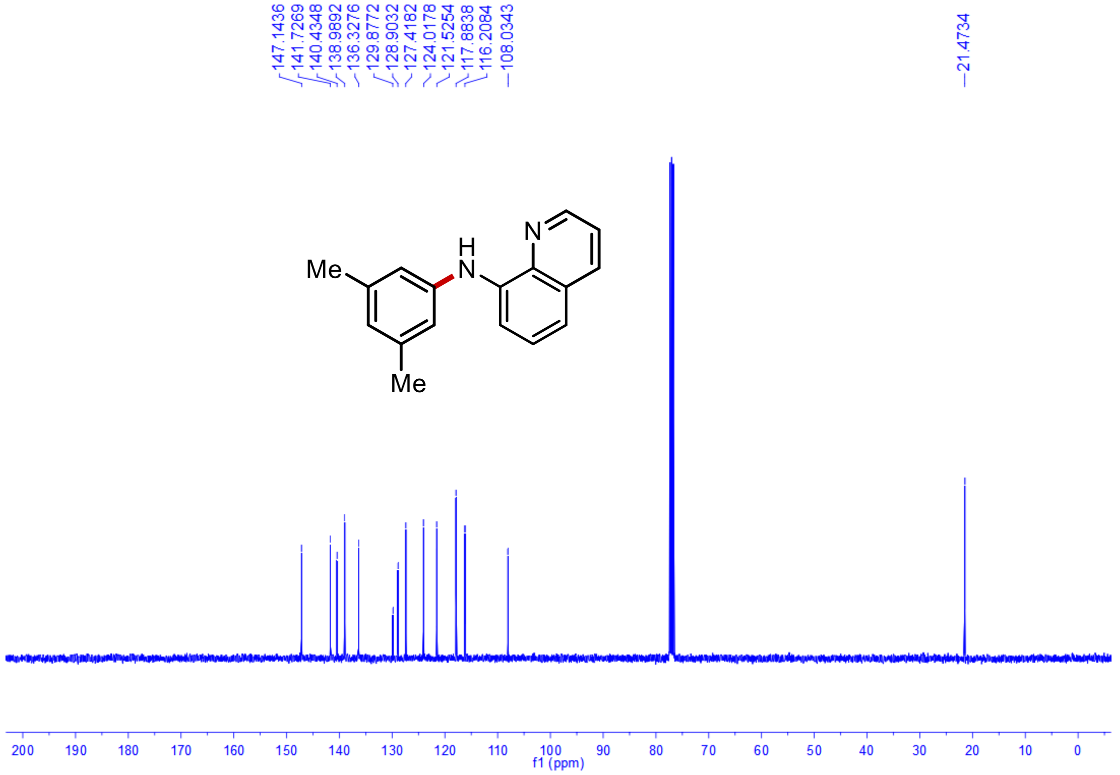

^{13}C NMR (100 MHz, $CDCl_3$) Spectrum

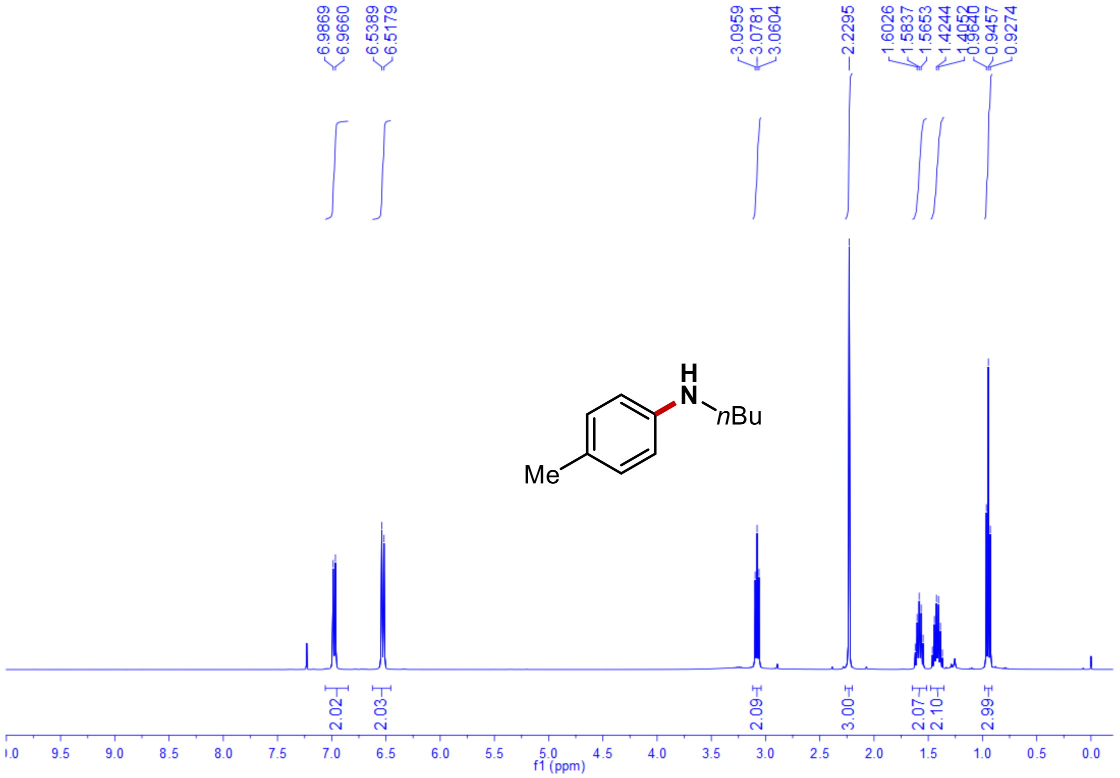

^{1}H NMR (400 MHz, $CDCl_3$) Spectrum

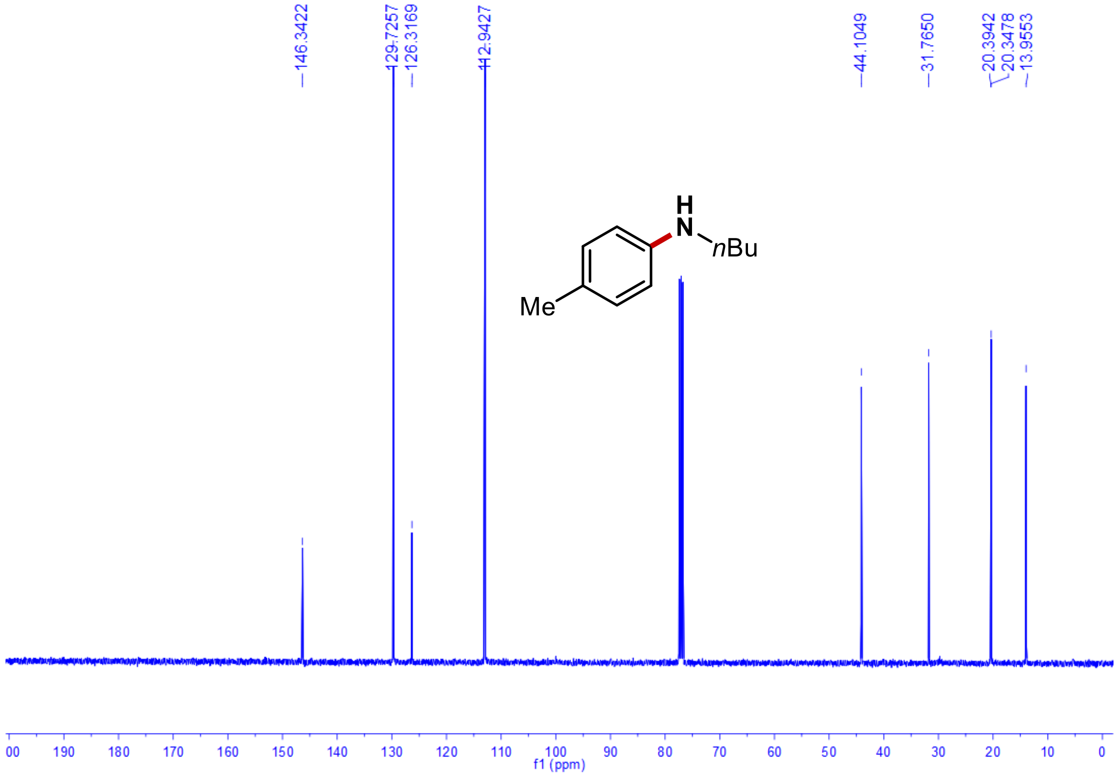

^{13}C NMR (100 MHz, $CDCl_3$) Spectrum

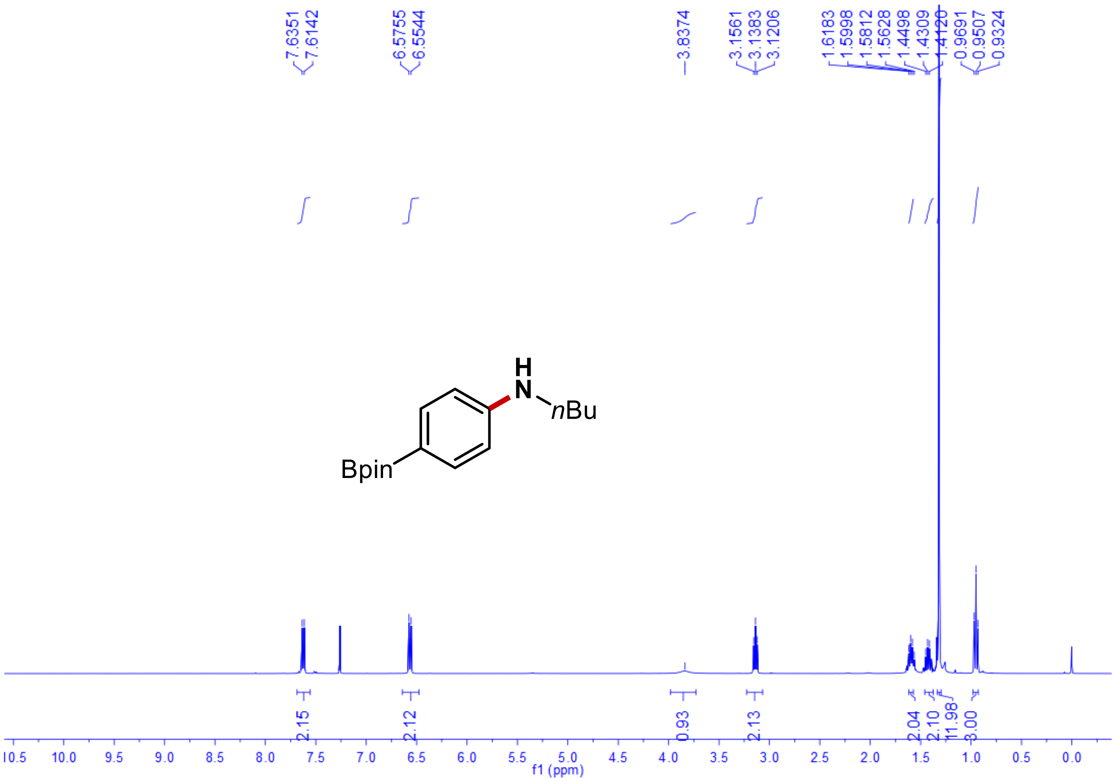

^{1}H NMR (400 MHz, $CDCl_3$) Spectrum

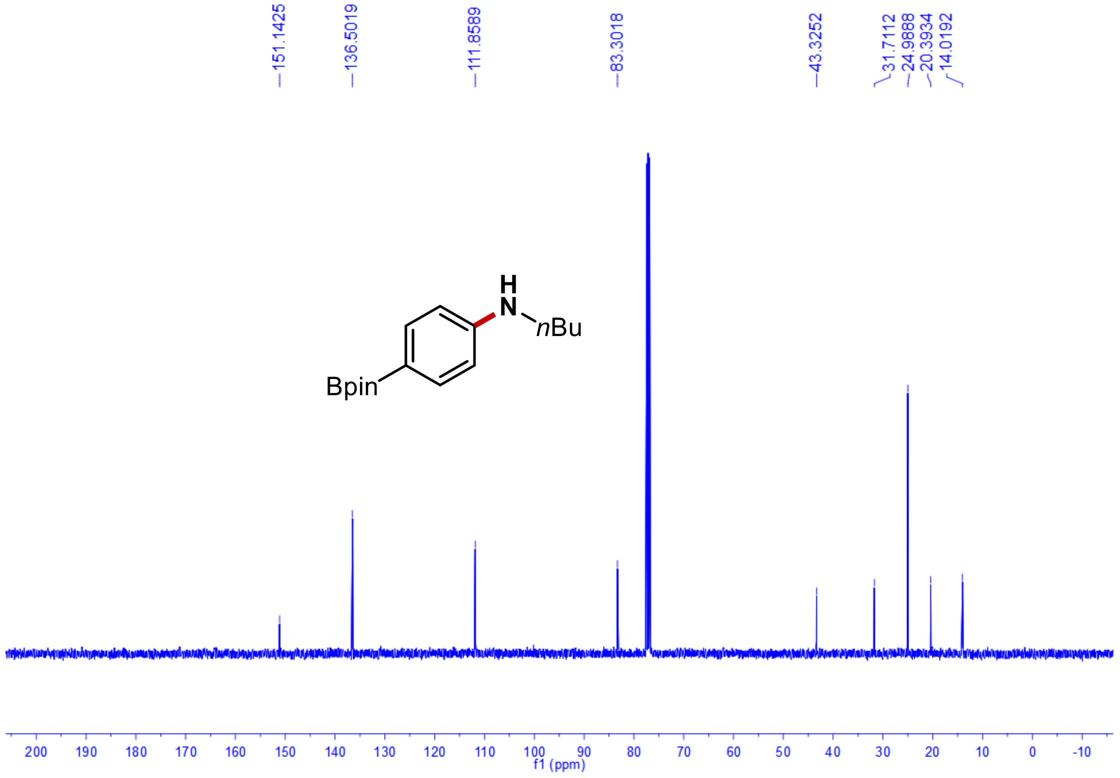

^{13}C NMR (100 MHz, $CDCl_3$) Spectrum

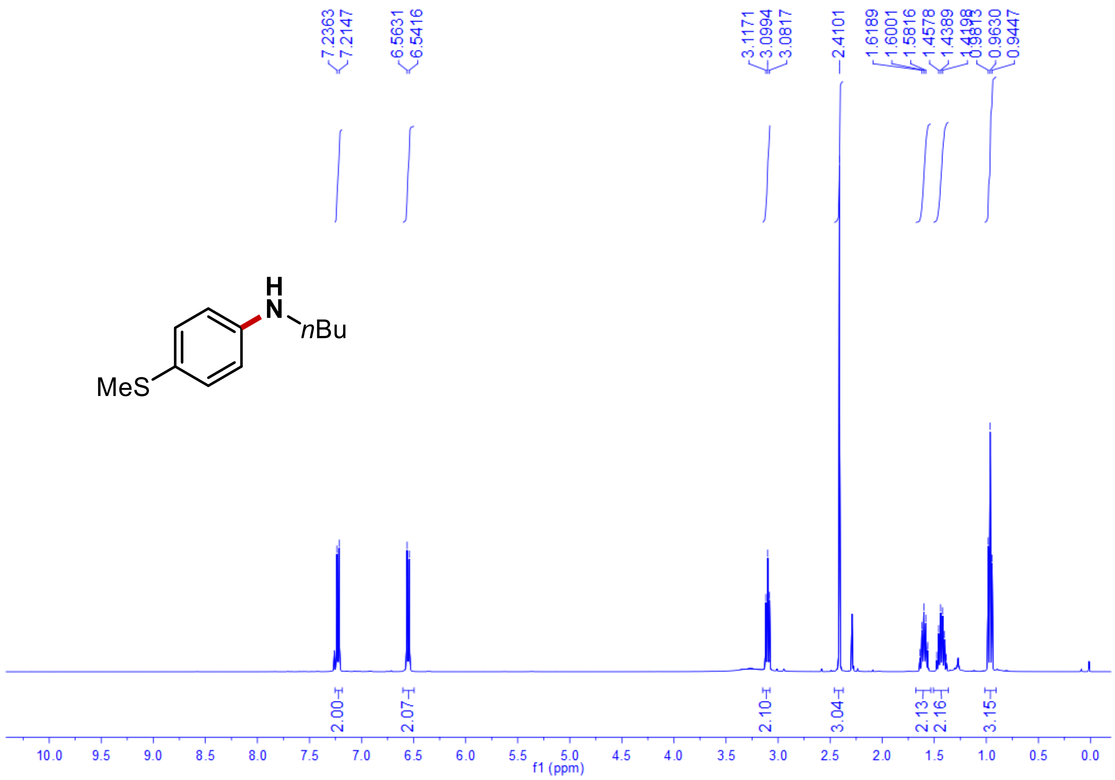

¹H NMR (400 MHz, $CDCl_3$) Spectrum

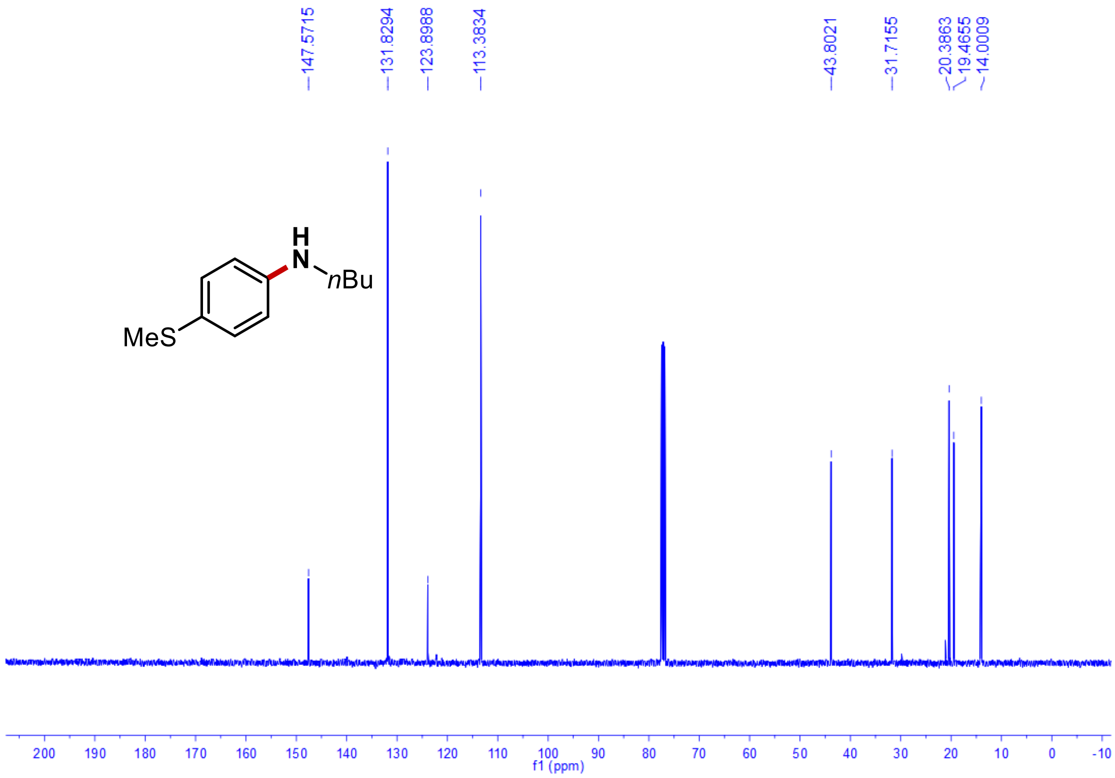

¹³C NMR (100 MHz, $CDCl_3$) Spectrum

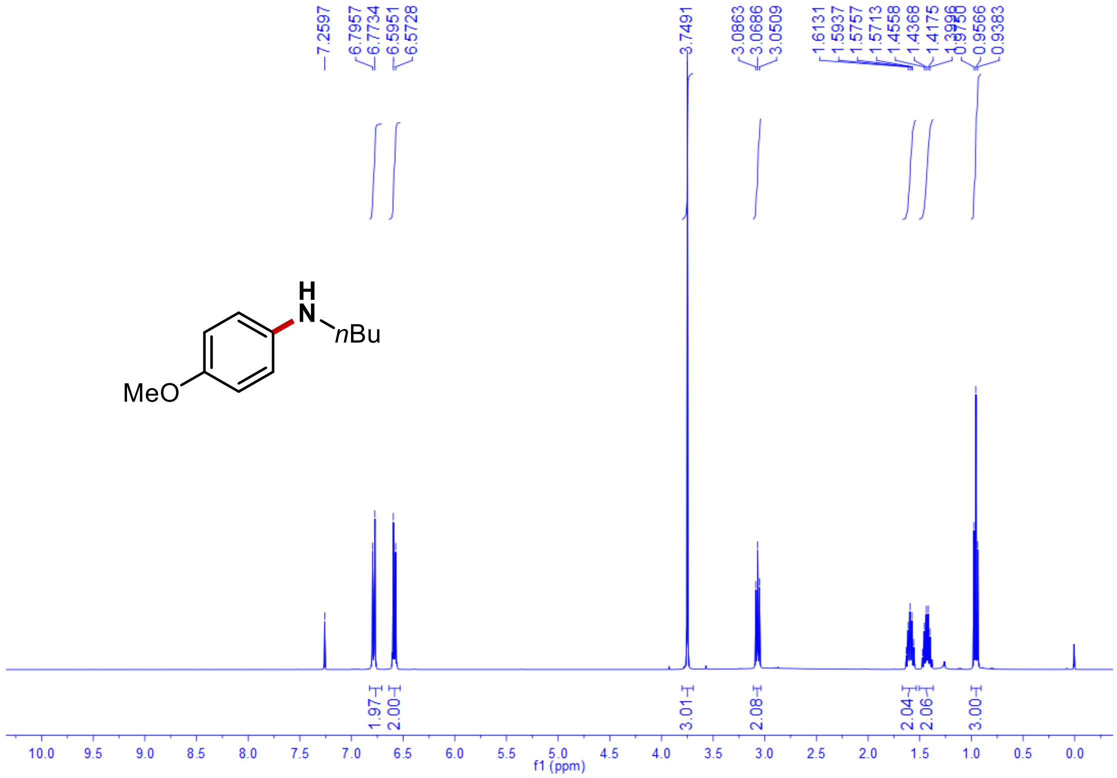

^{1}**H NMR** (400 MHz, $CDCl_3$) Spectrum

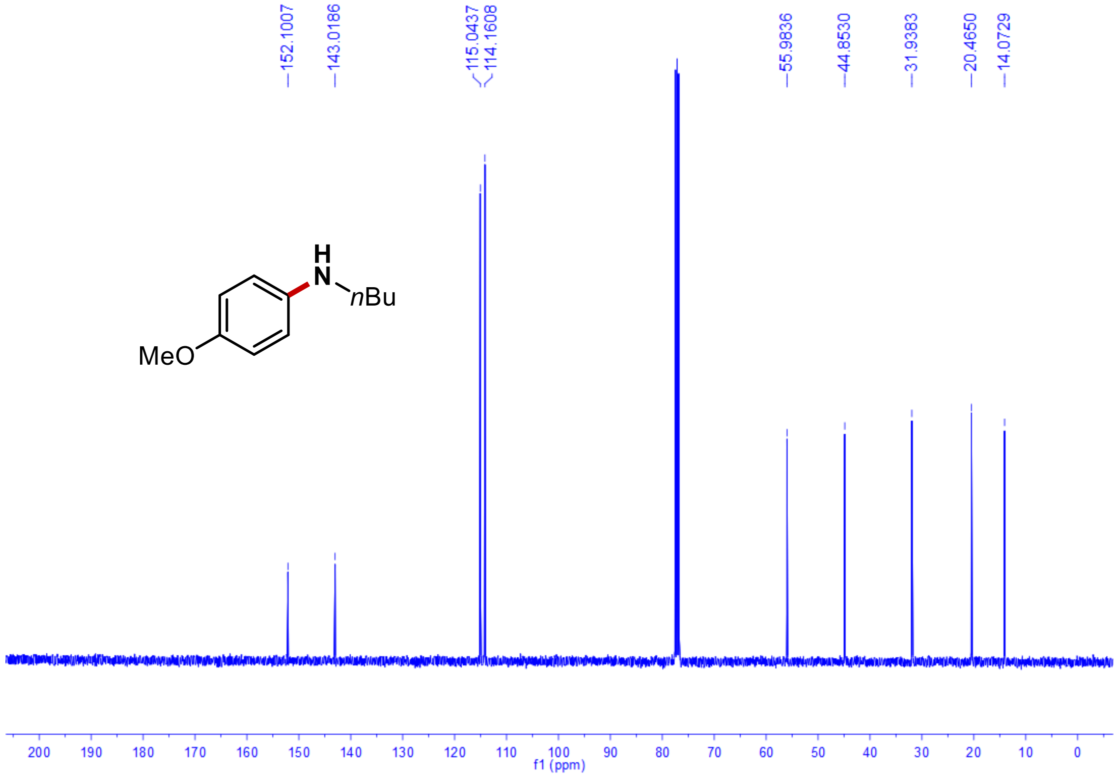

^{13}**C NMR** (100 MHz, $CDCl_3$) Spectrum

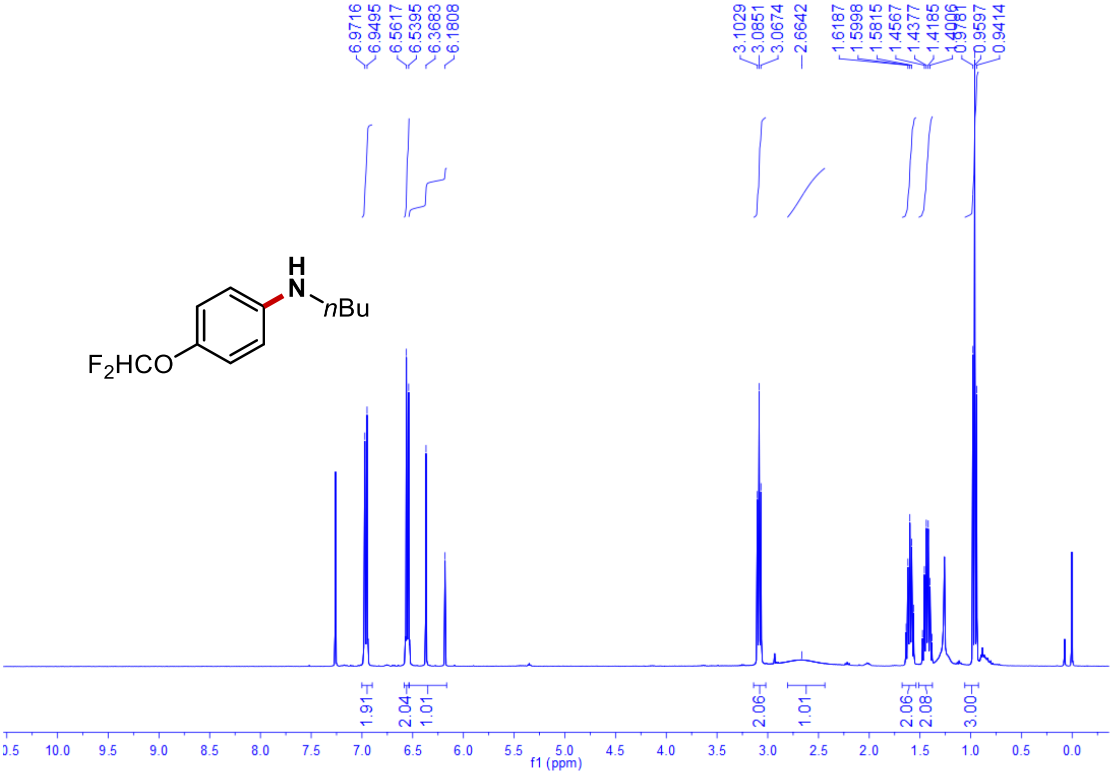

^{1}H NMR (400 MHz, $CDCl_3$) Spectrum

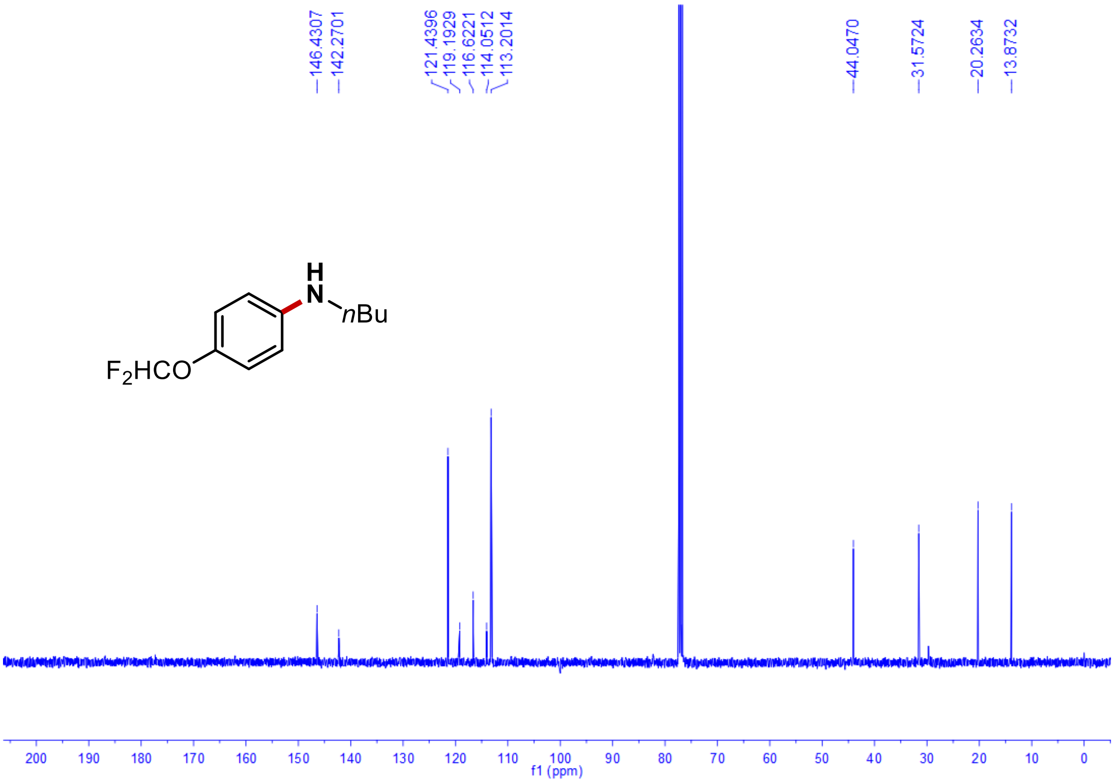

^{13}C NMR (100 MHz, $CDCl_3$) Spectrum

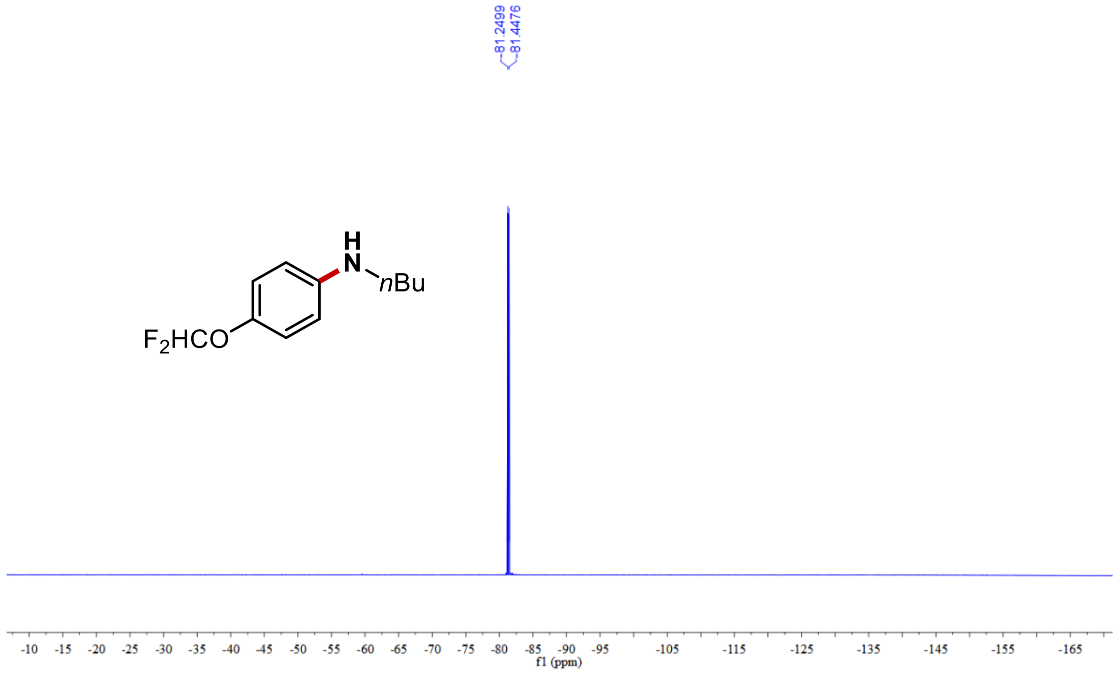

^{19}**F NMR** (376 MHz, $CDCl_3$) Spectrum

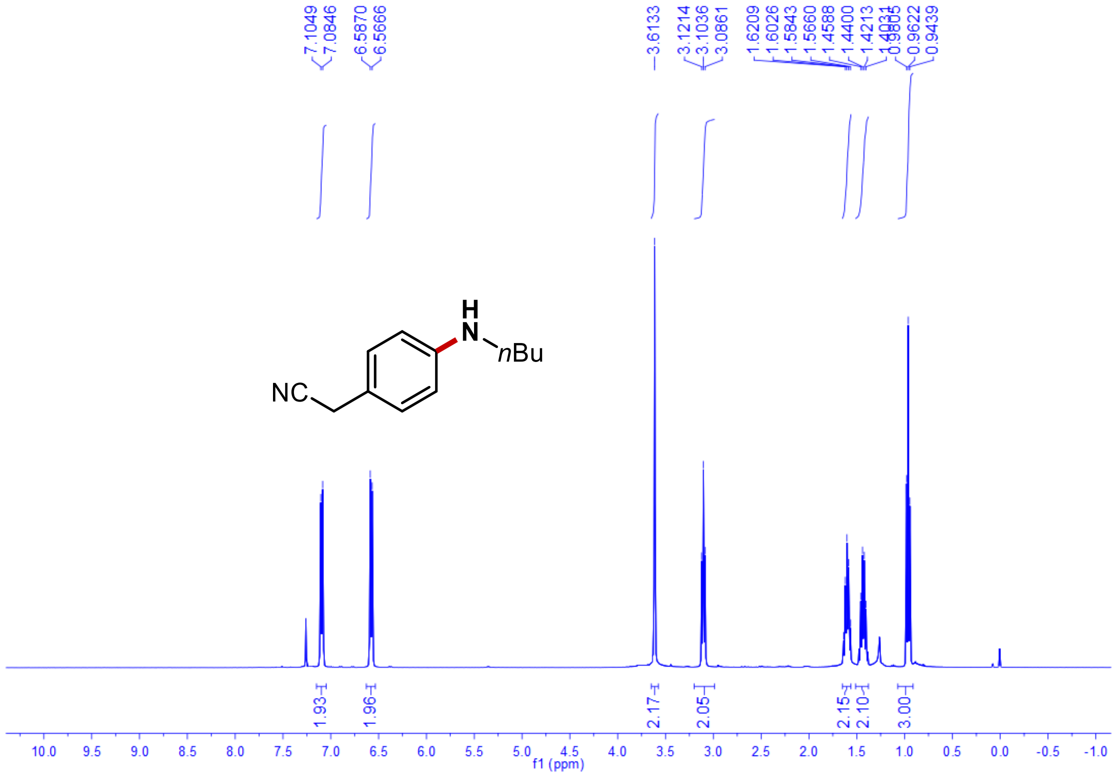

^{1}**H NMR** (400 MHz, $CDCl_3$) Spectrum

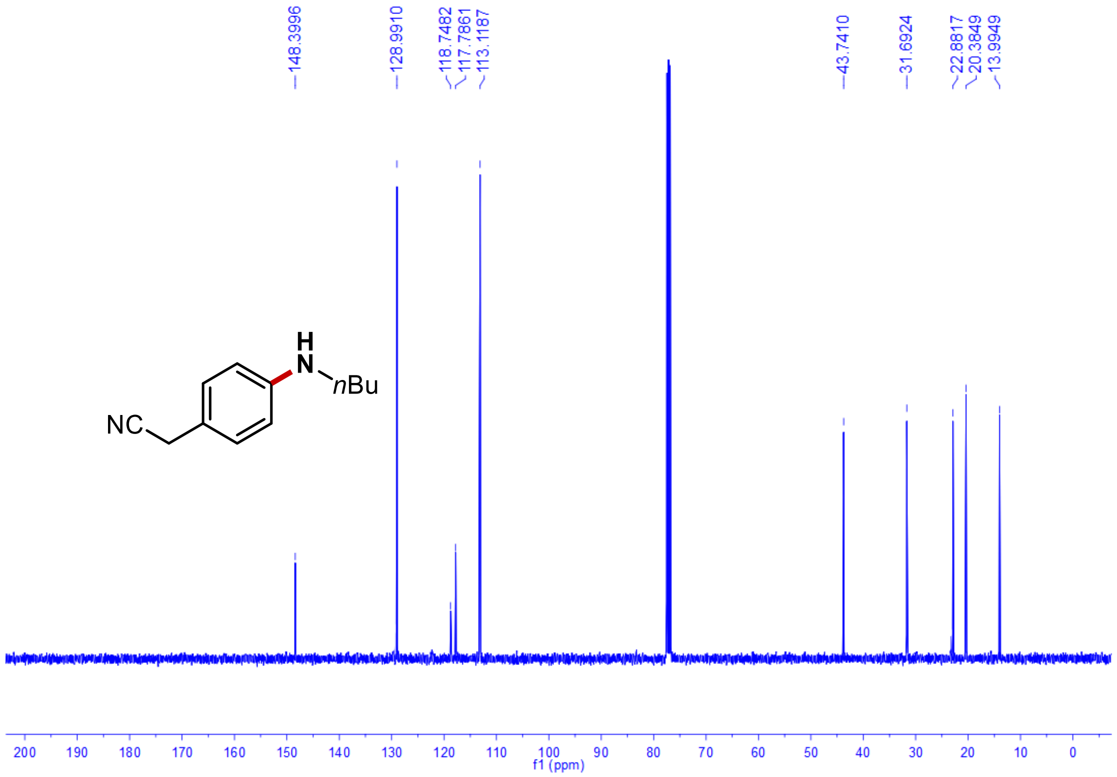

^{13}**C NMR** (100 MHz, $CDCl_3$) Spectrum

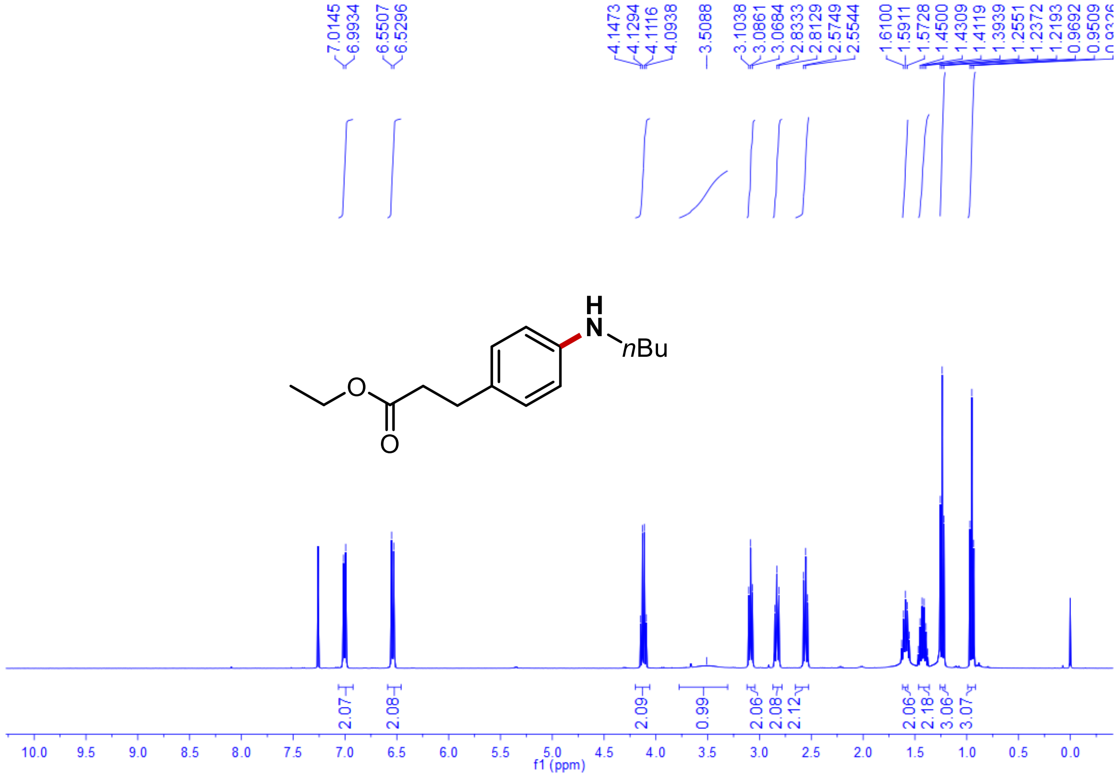

^{1}**H NMR** (400 MHz, $CDCl_3$) Spectrum

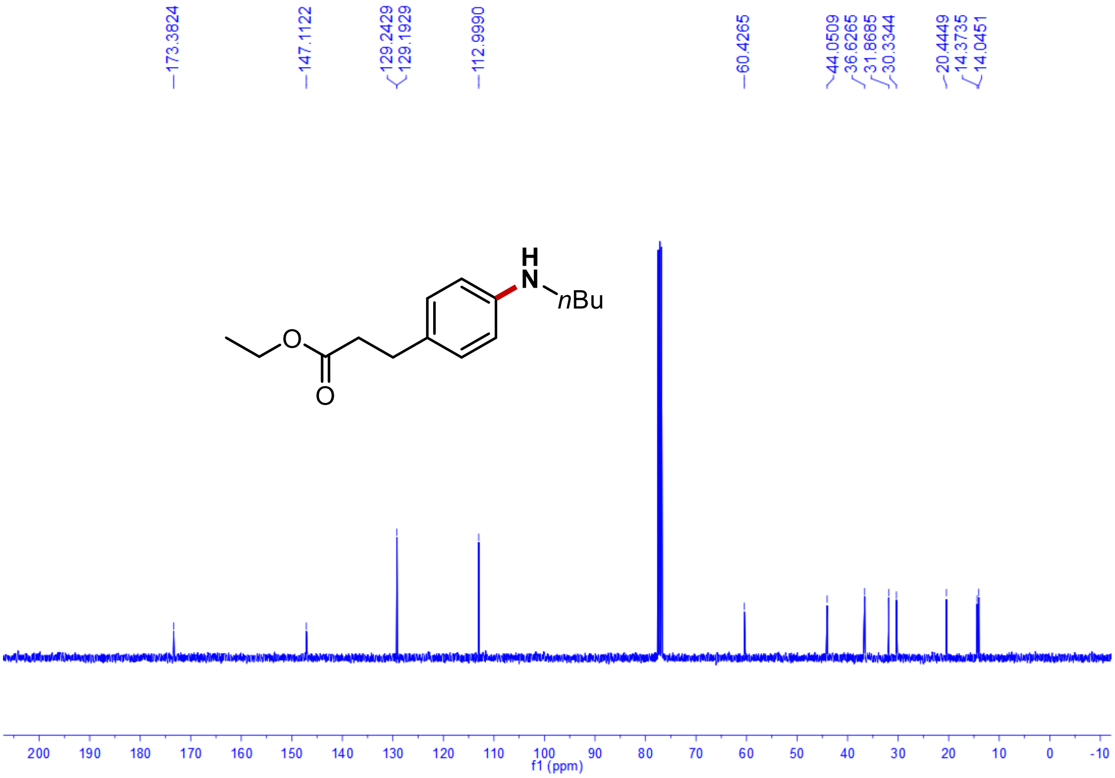

^{13}C NMR (100 MHz, $CDCl_3$) Spectrum

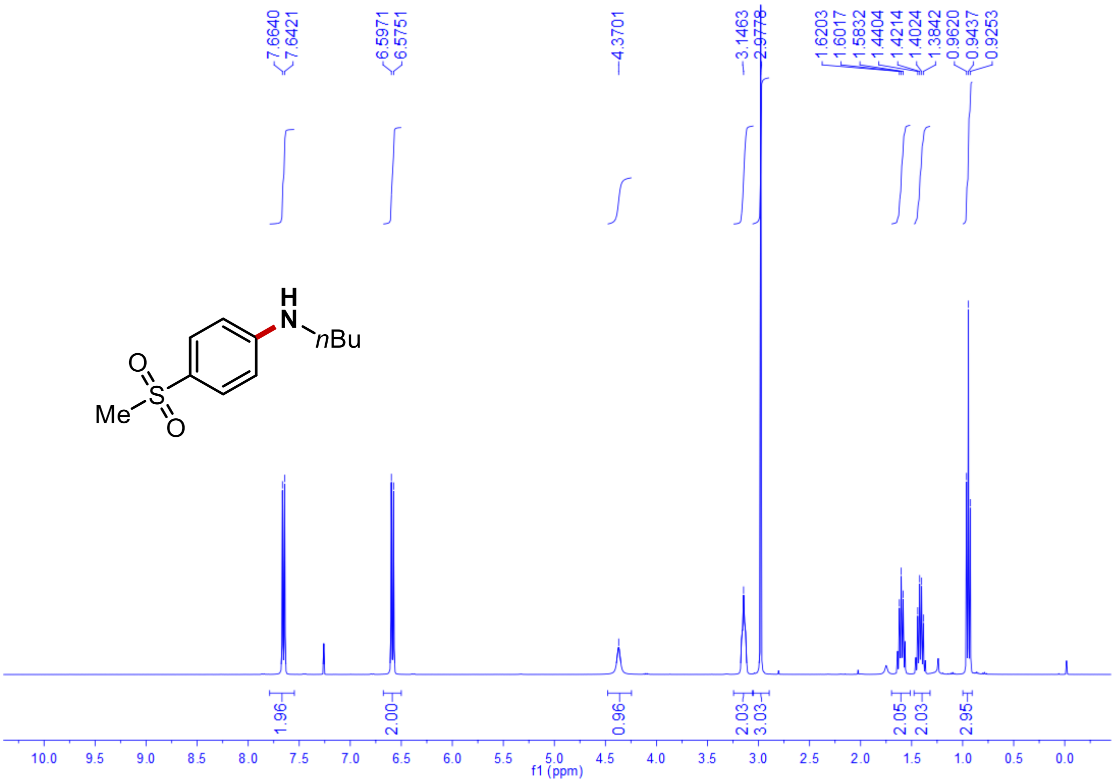

^{1}H NMR (400 MHz, $CDCl_3$) Spectrum

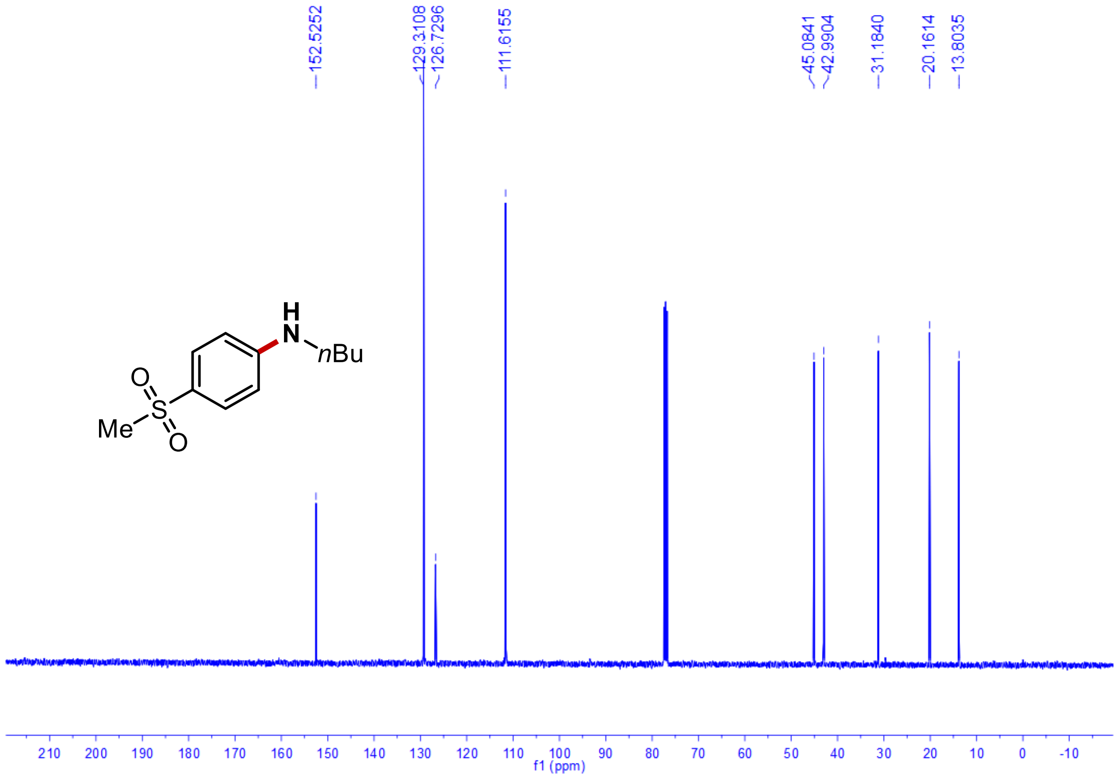

^{13}C NMR (100 MHz, $CDCl_3$) Spectrum

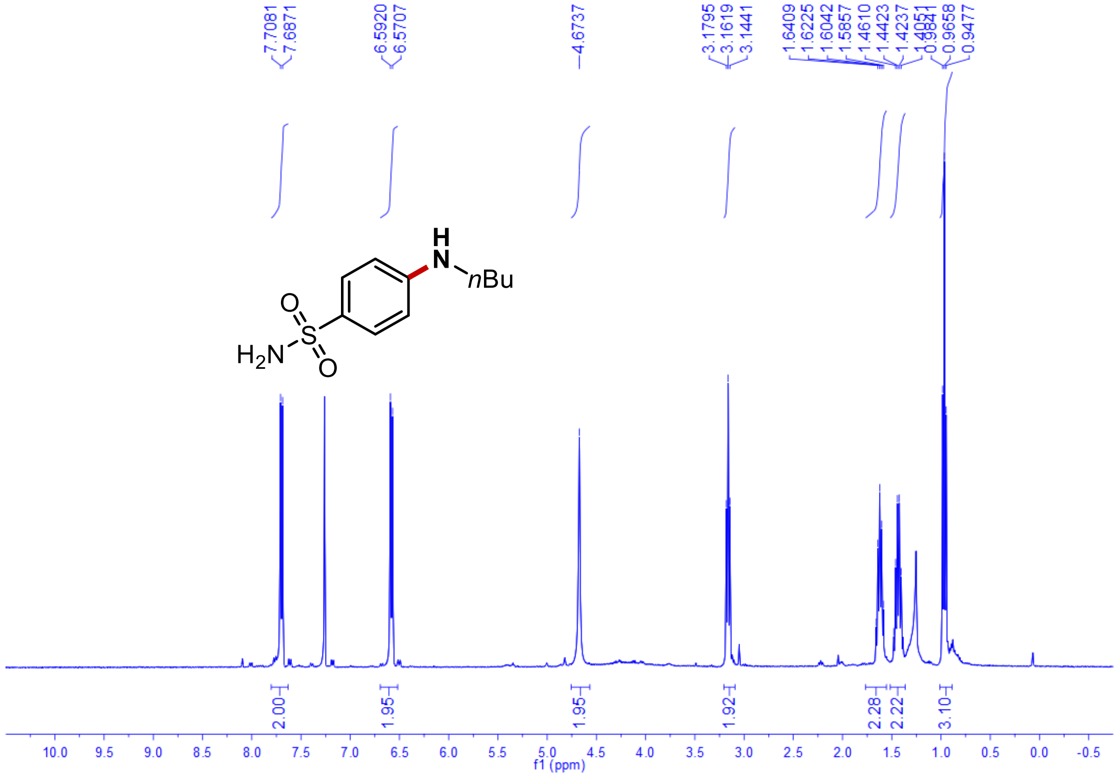

^{1}H NMR (400 MHz, $CDCl_3$) Spectrum

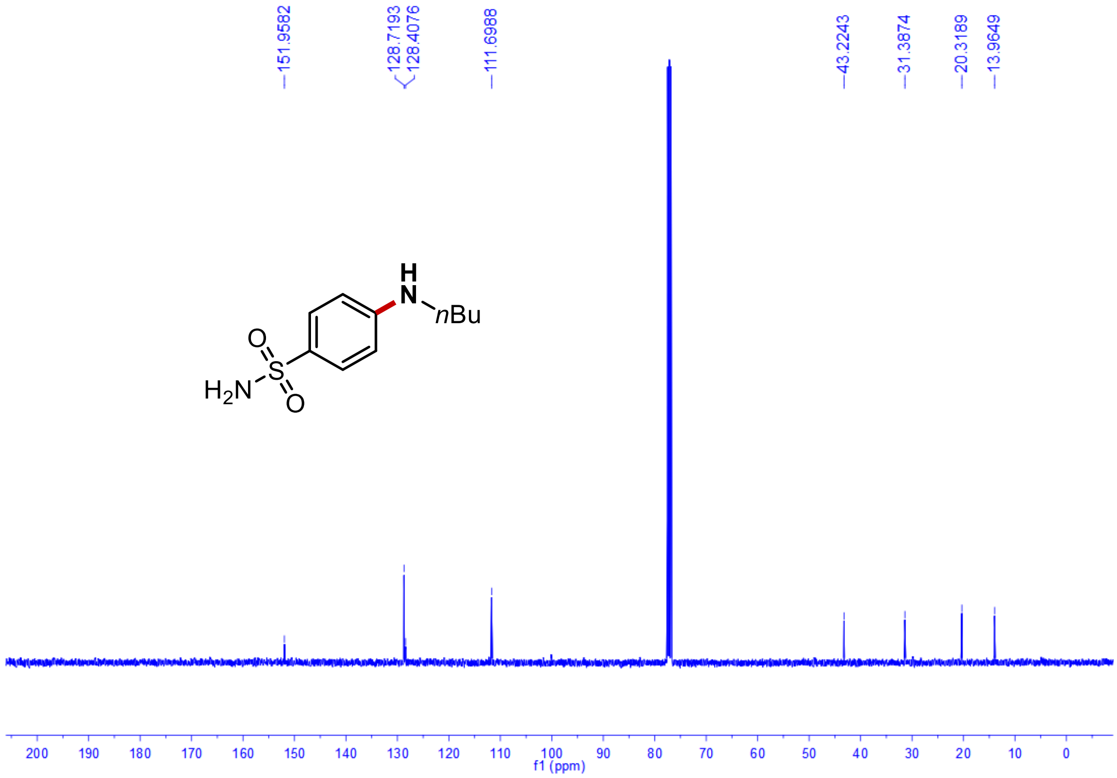

^{13}**C NMR** (100 MHz, $CDCl_3$) Spectrum

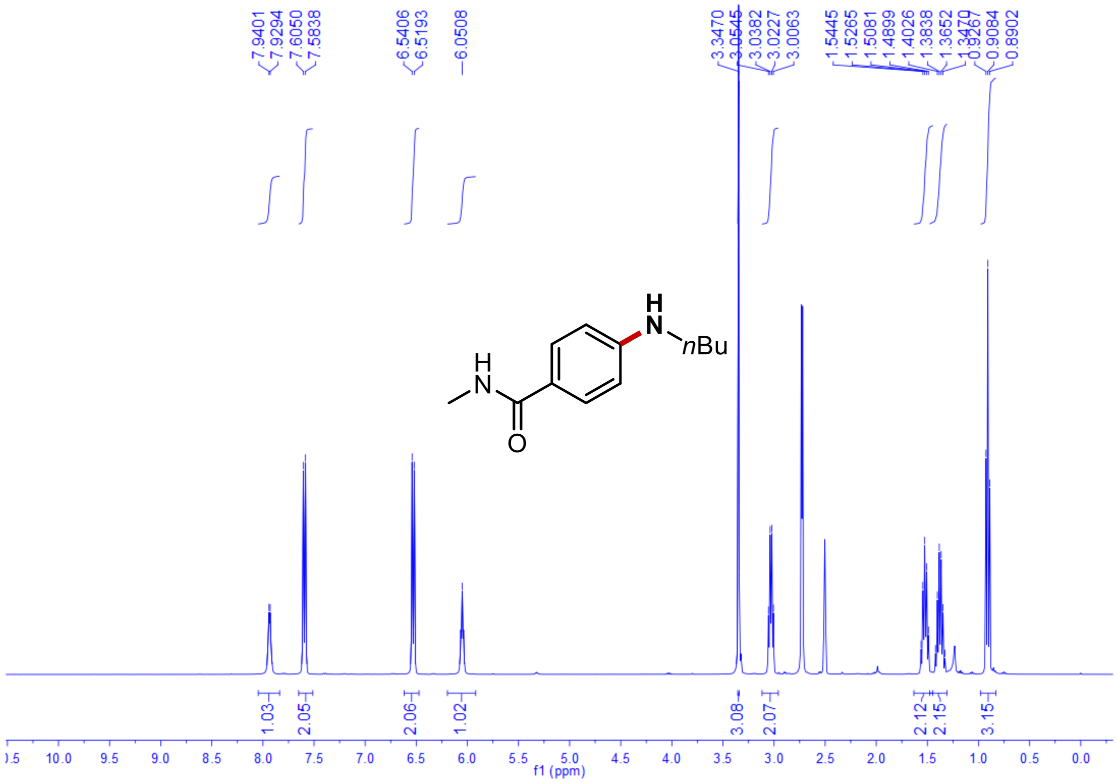

^{1}**H NMR** (400 MHz, $CDCl_3$) Spectrum

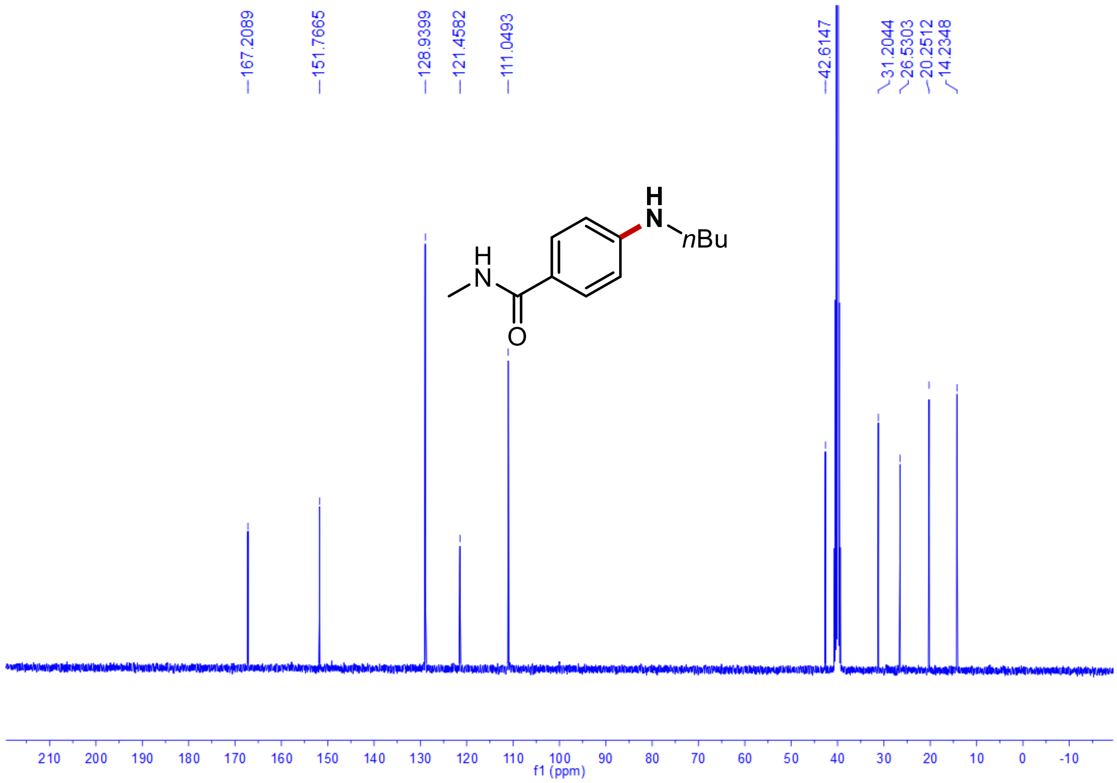

^{13}C NMR (100 MHz, $CDCl_3$) Spectrum

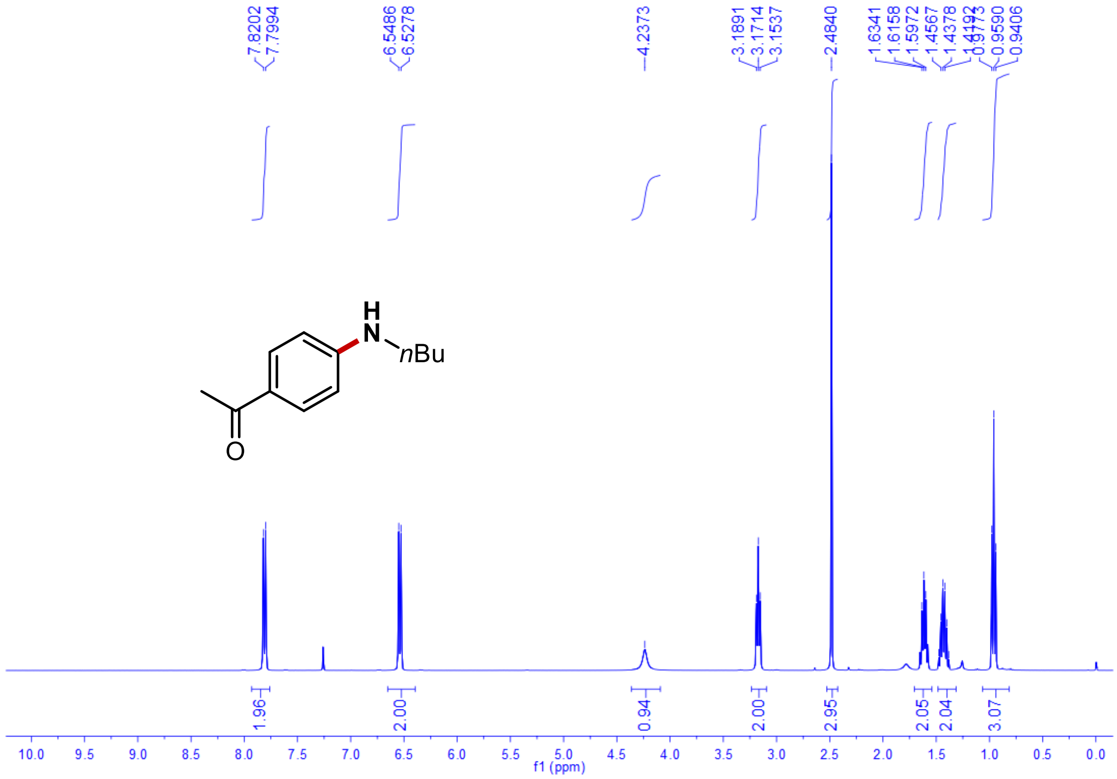

^{1}H NMR (400 MHz, $CDCl_3$) Spectrum

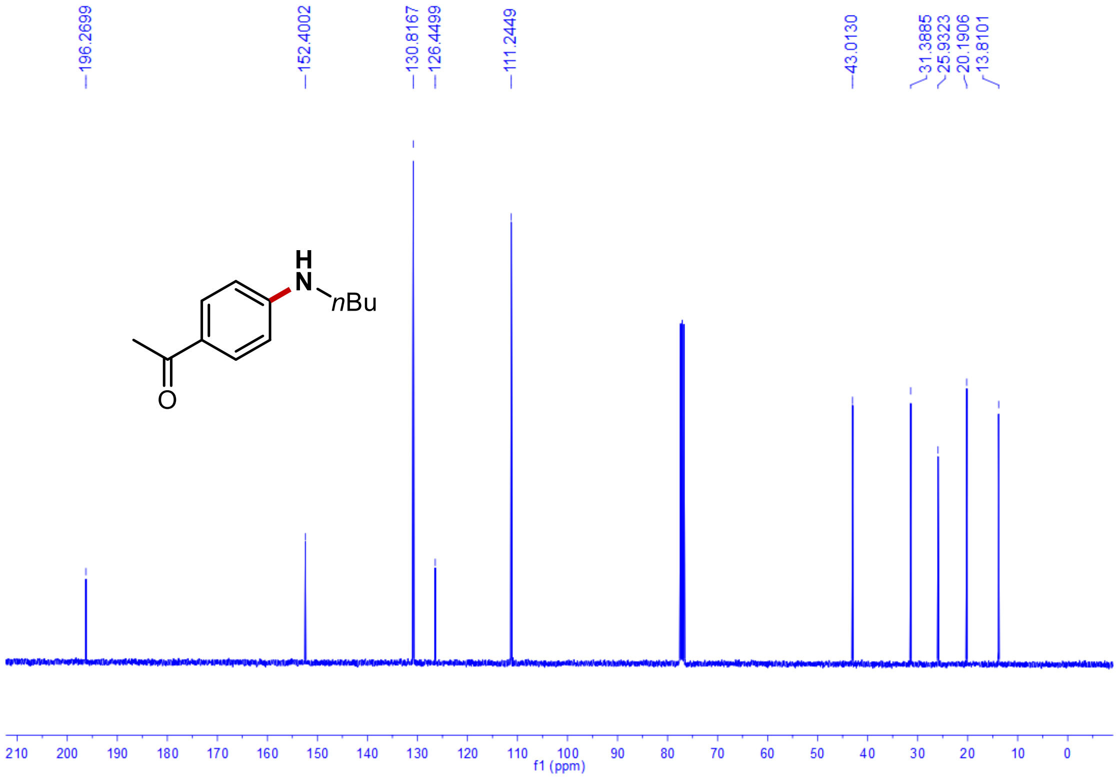

^{13}**C NMR** (100 MHz, $CDCl_3$) Spectrum

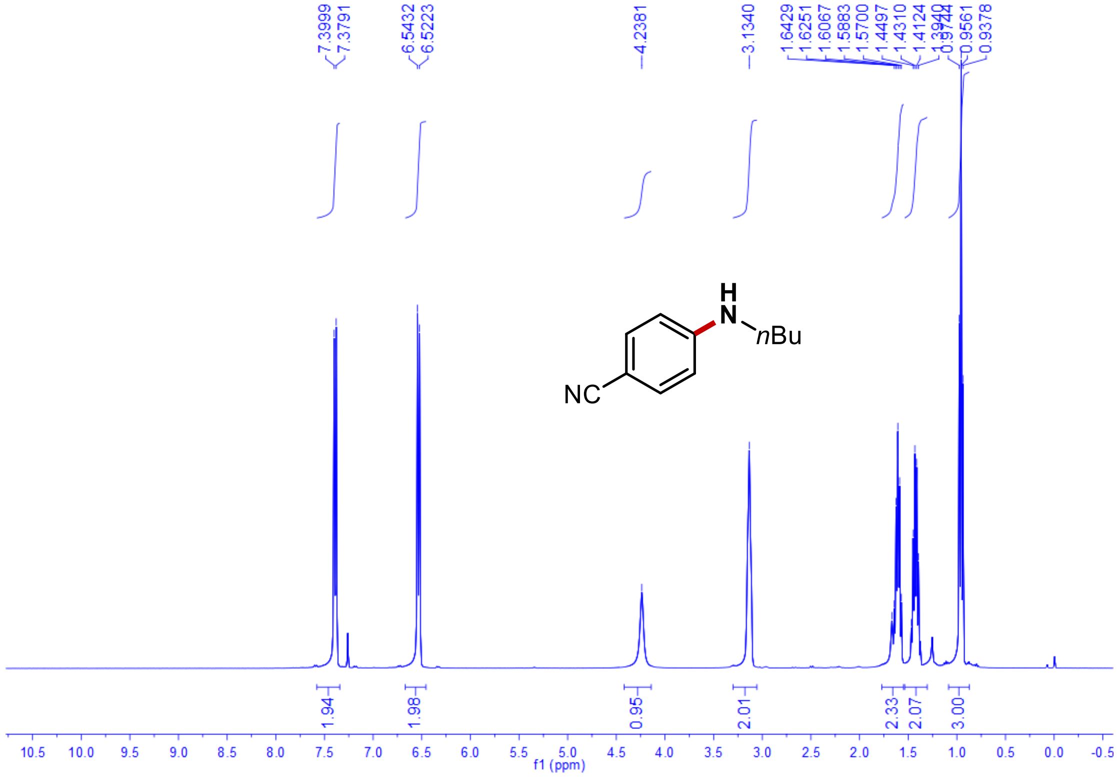

^{1}**H NMR** (400 MHz, $CDCl_3$) Spectrum

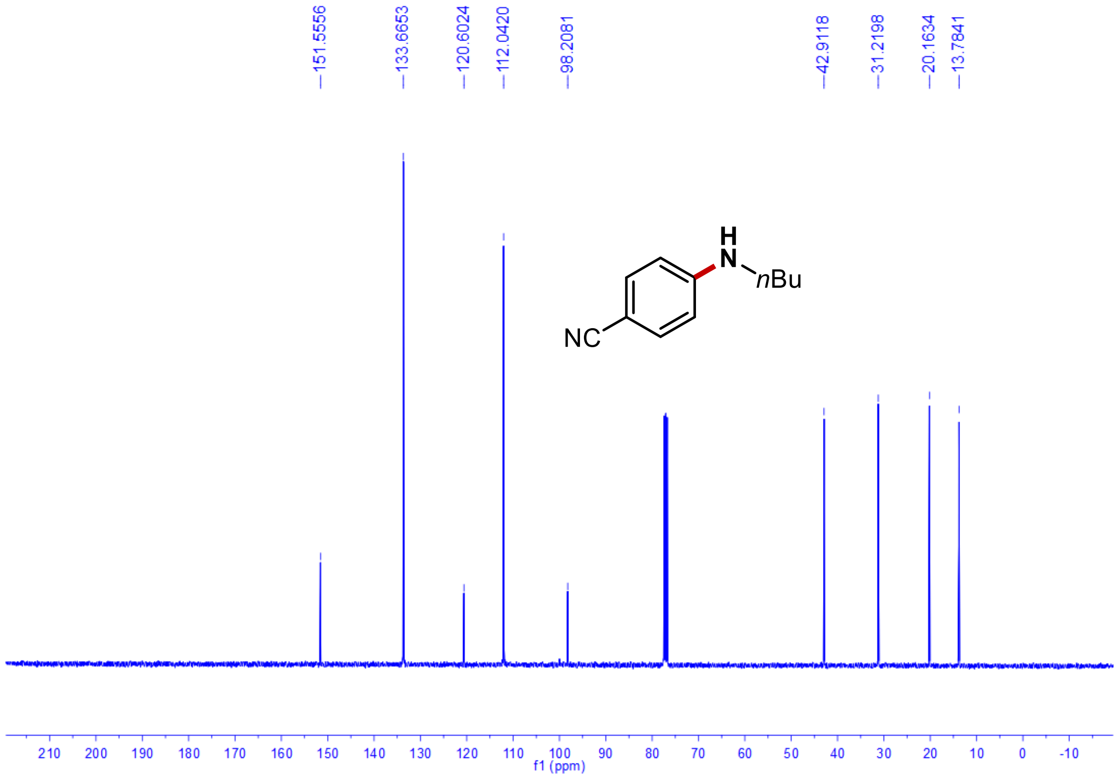

^{13}C NMR (100 MHz, $CDCl_3$) Spectrum

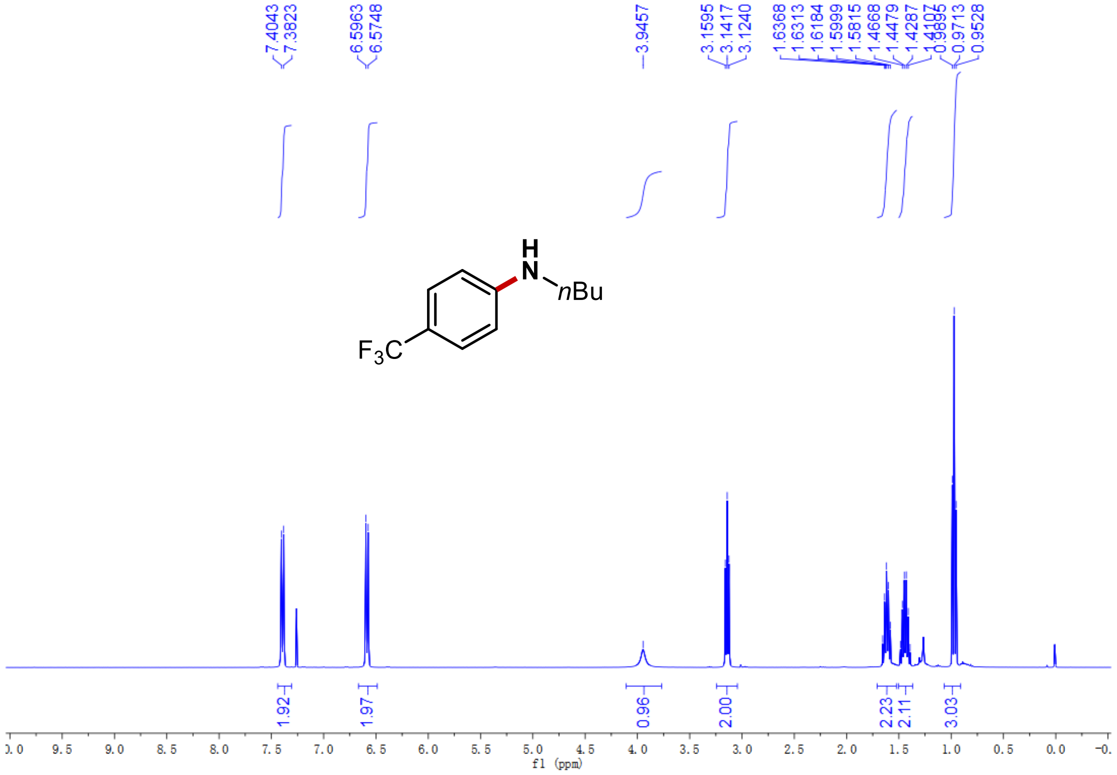

^{1}H NMR (400 MHz, $CDCl_3$) Spectrum

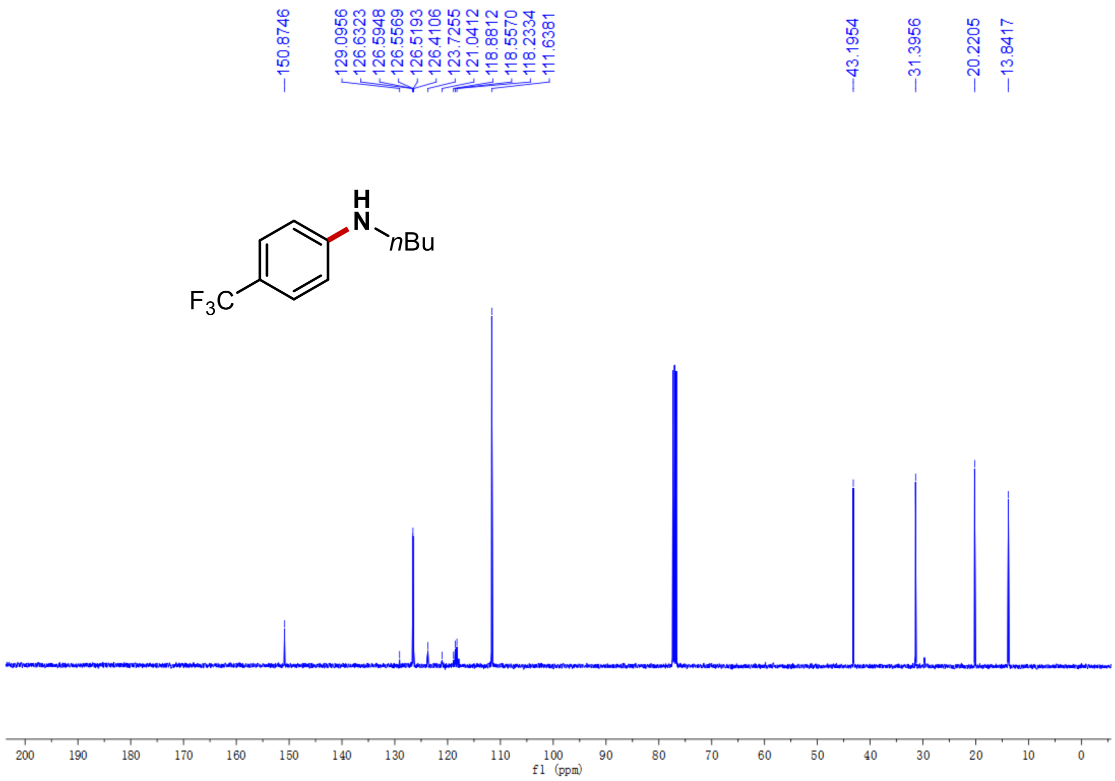

^{13}C NMR (100 MHz, $CDCl_3$) Spectrum

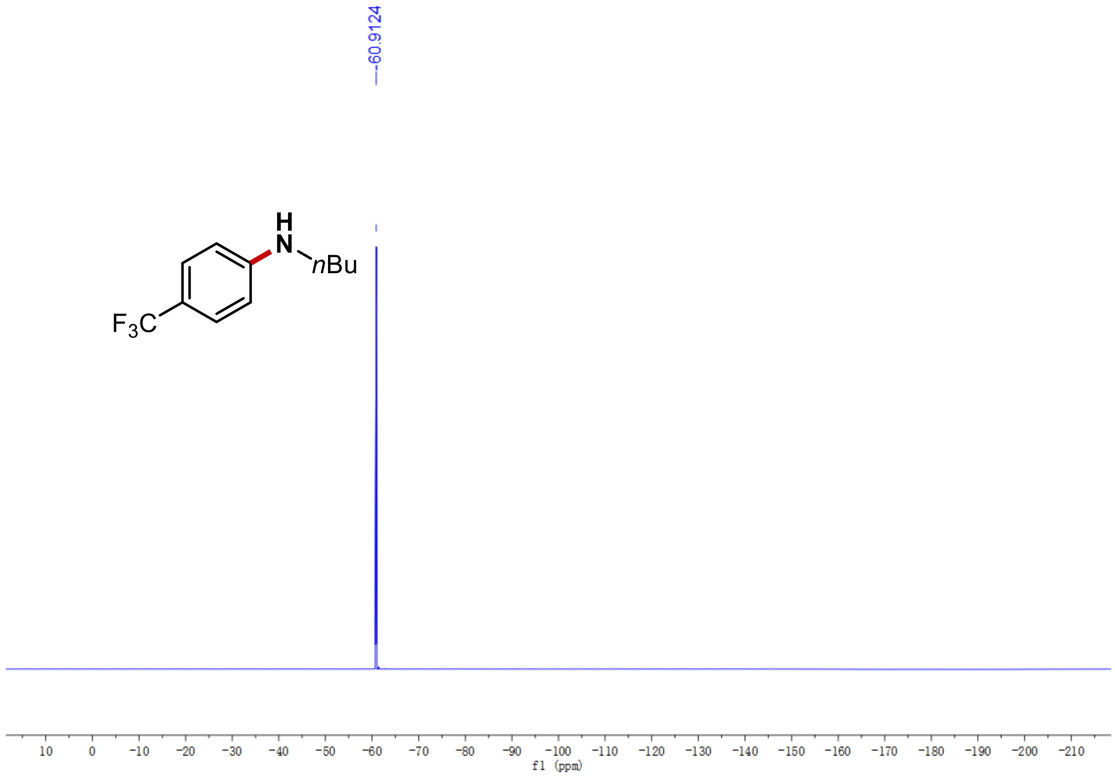

^{19}F NMR (376 MHz, $CDCl_3$) Spectrum

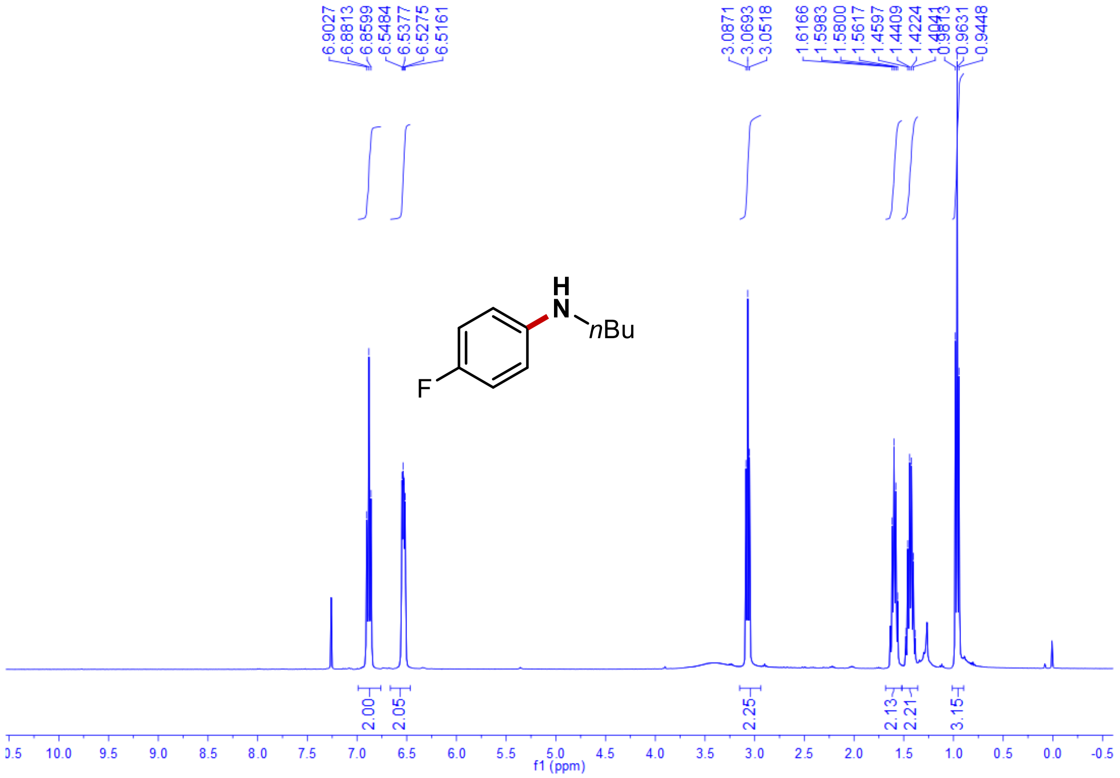

^{1}H NMR (400 MHz, $CDCl_3$) Spectrum

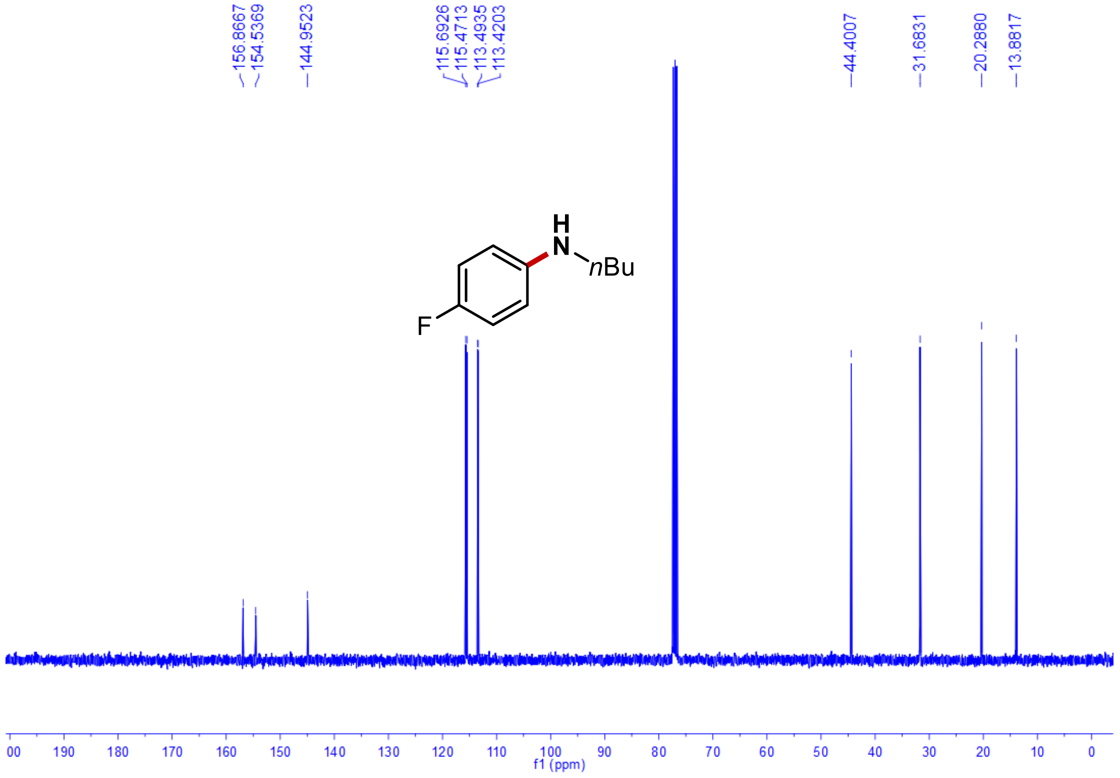

^{13}C NMR (100 MHz, $CDCl_3$) Spectrum

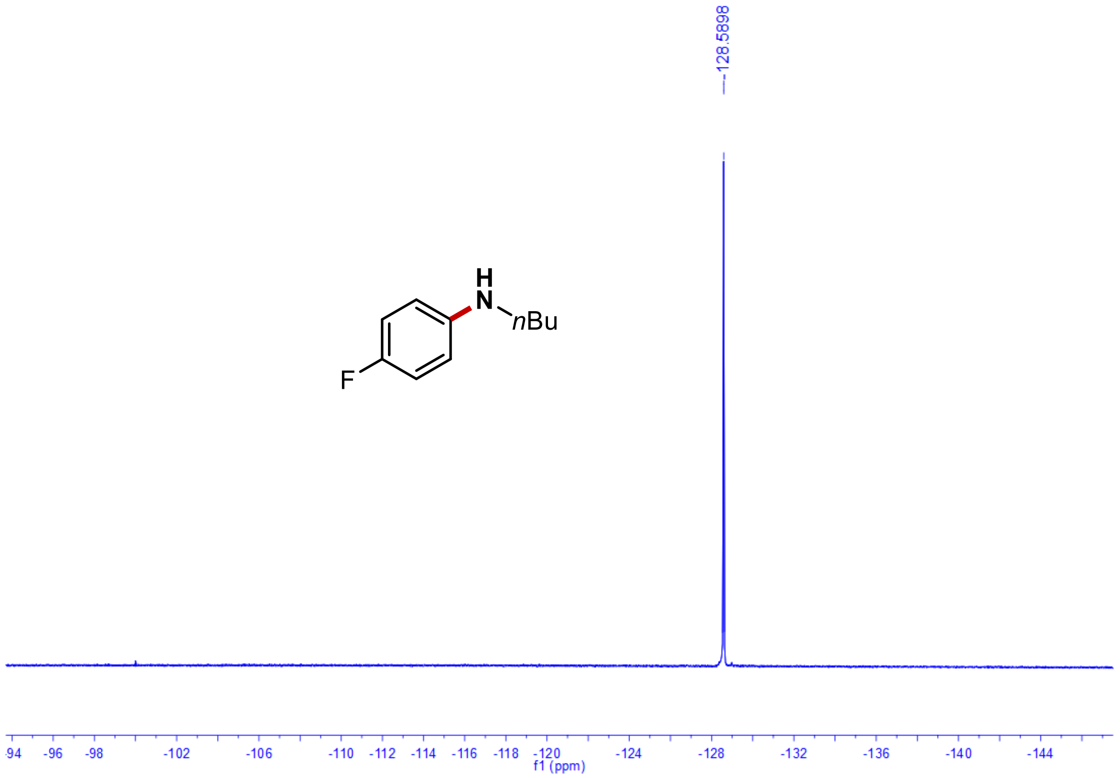

^{19}F NMR (376 MHz, $CDCl_3$) Spectrum

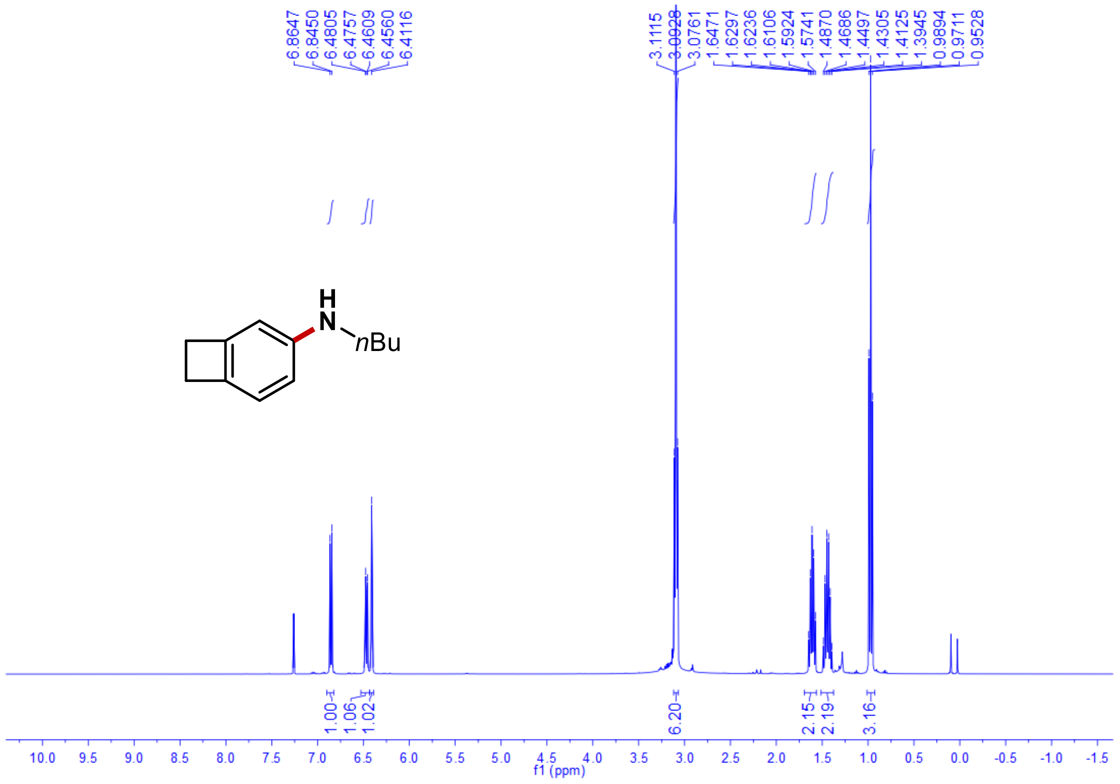

^{1}H NMR (400 MHz, $CDCl_3$) Spectrum

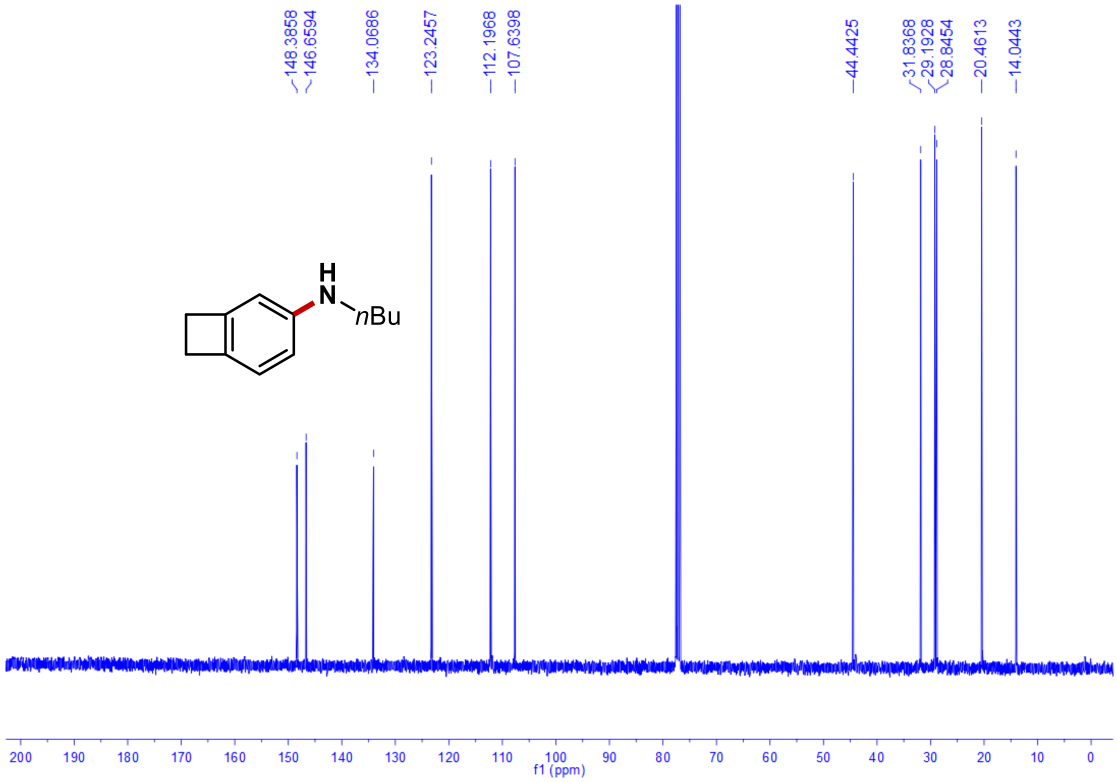

^{13}C NMR (100 MHz, $CDCl_3$) Spectrum

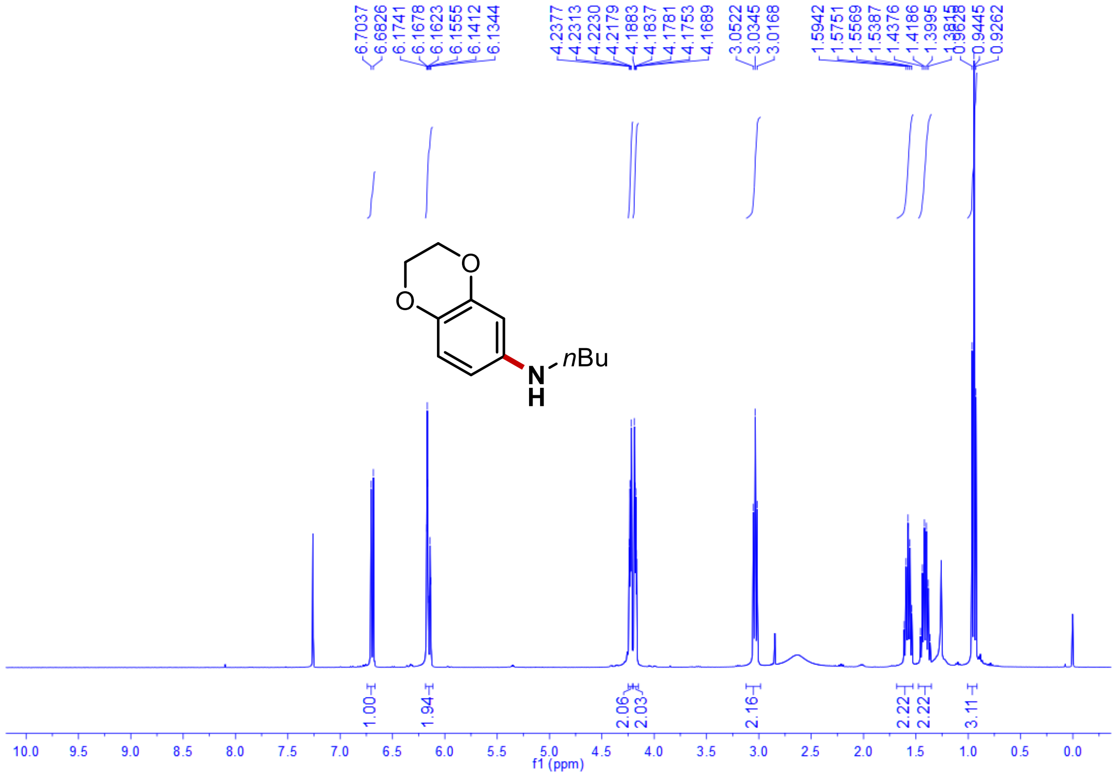

^{1}H NMR (400 MHz, $CDCl_3$) Spectrum

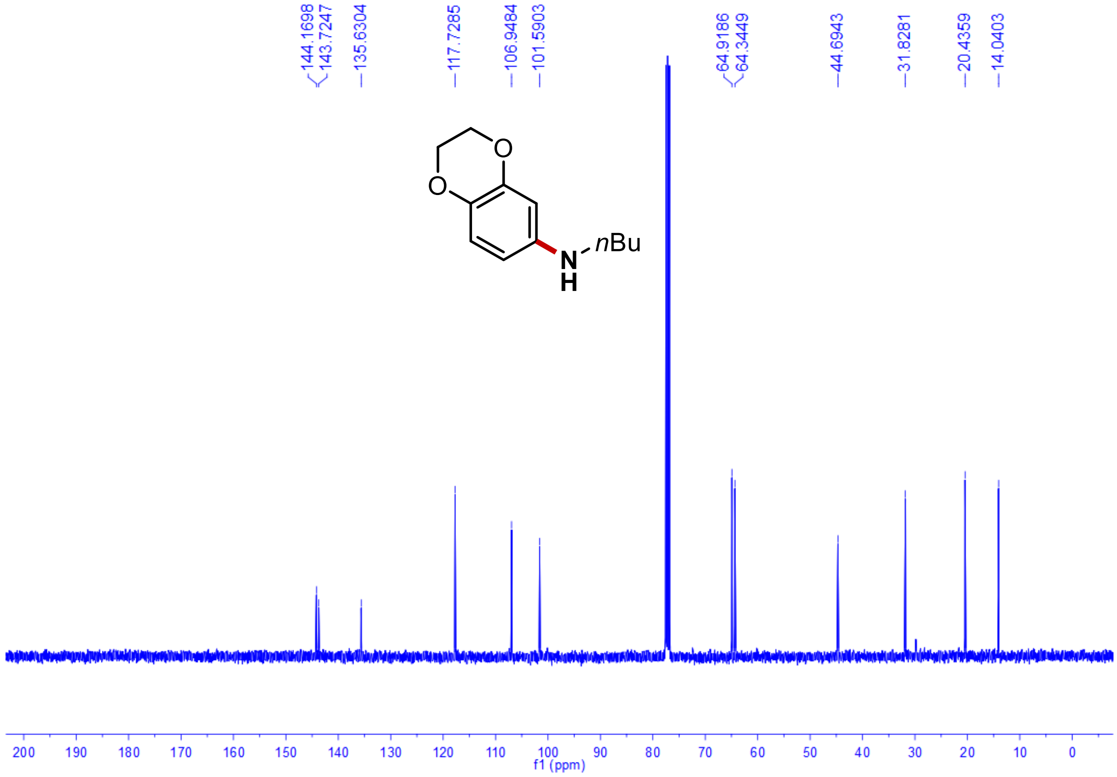

^{13}C NMR (100 MHz, $CDCl_3$) Spectrum

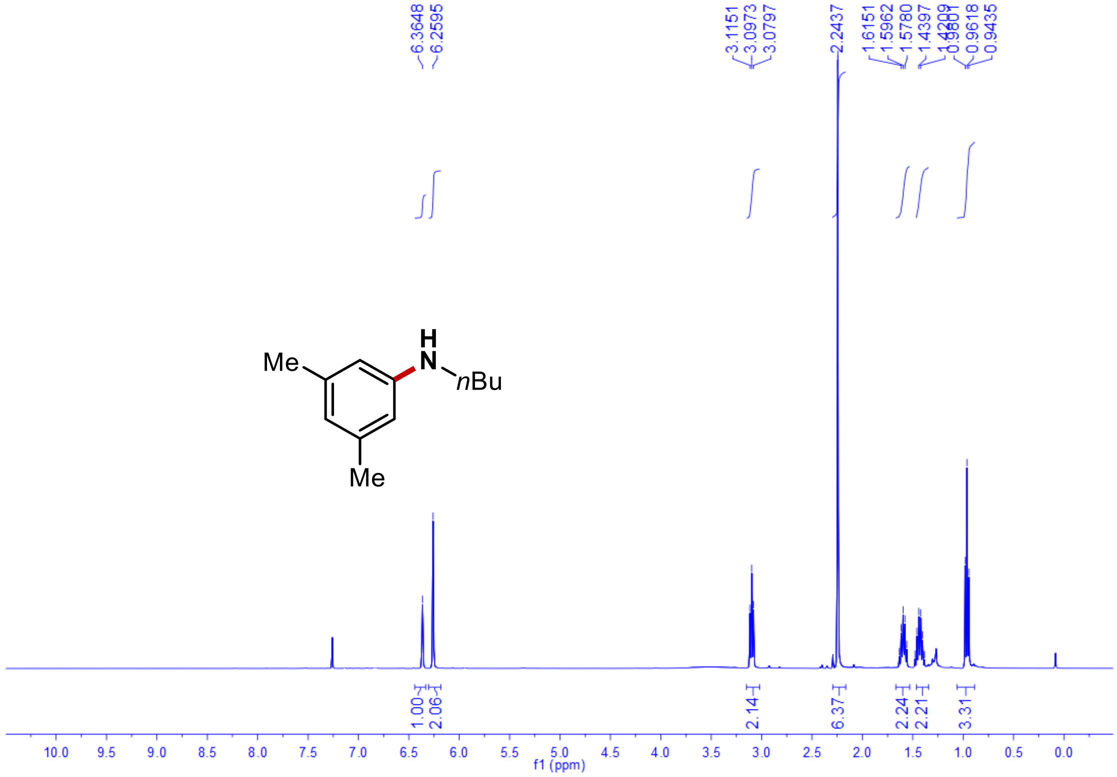

^{1}H NMR (400 MHz, $CDCl_3$) Spectrum

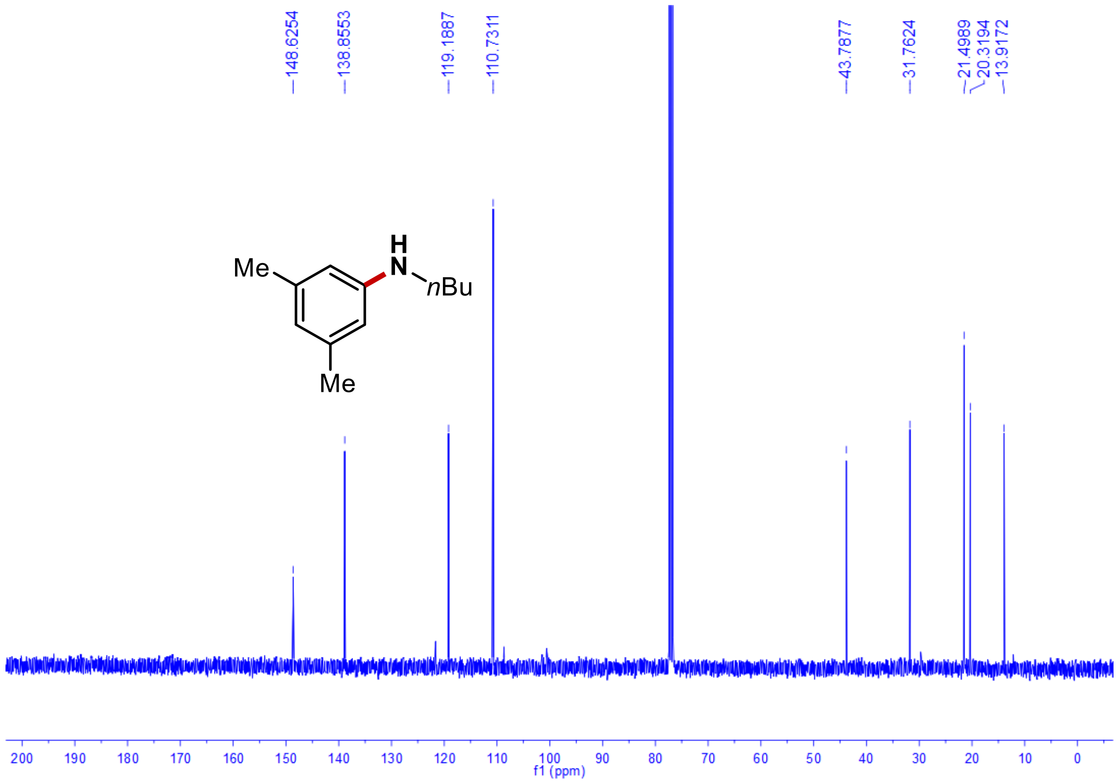

^{13}C NMR (100 MHz, $CDCl_3$) Spectrum

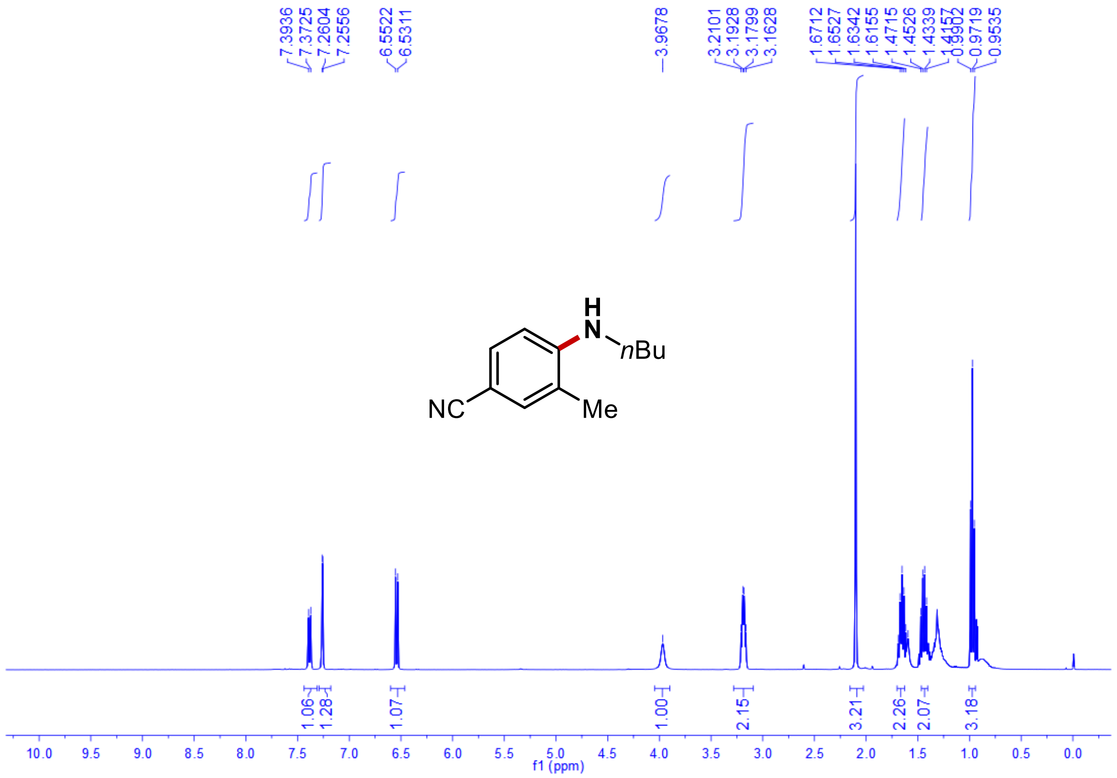

^{1}H NMR (400 MHz, $CDCl_3$) Spectrum

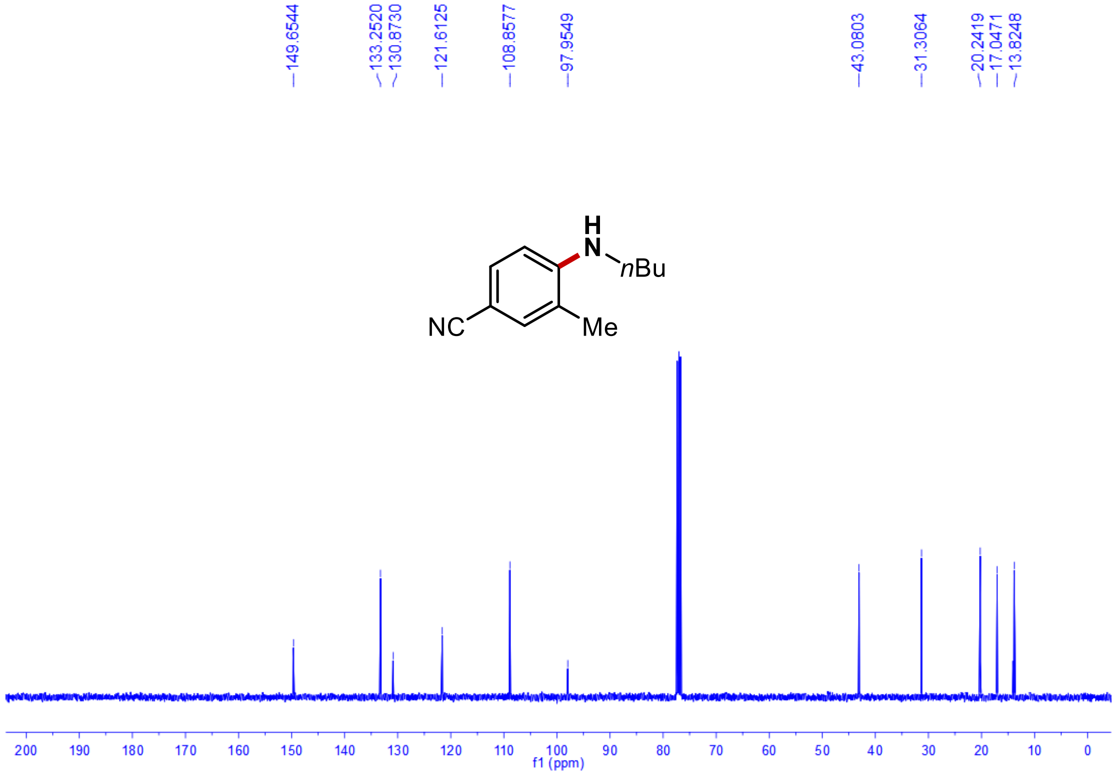

^{13}C NMR (100 MHz, $CDCl_3$) Spectrum

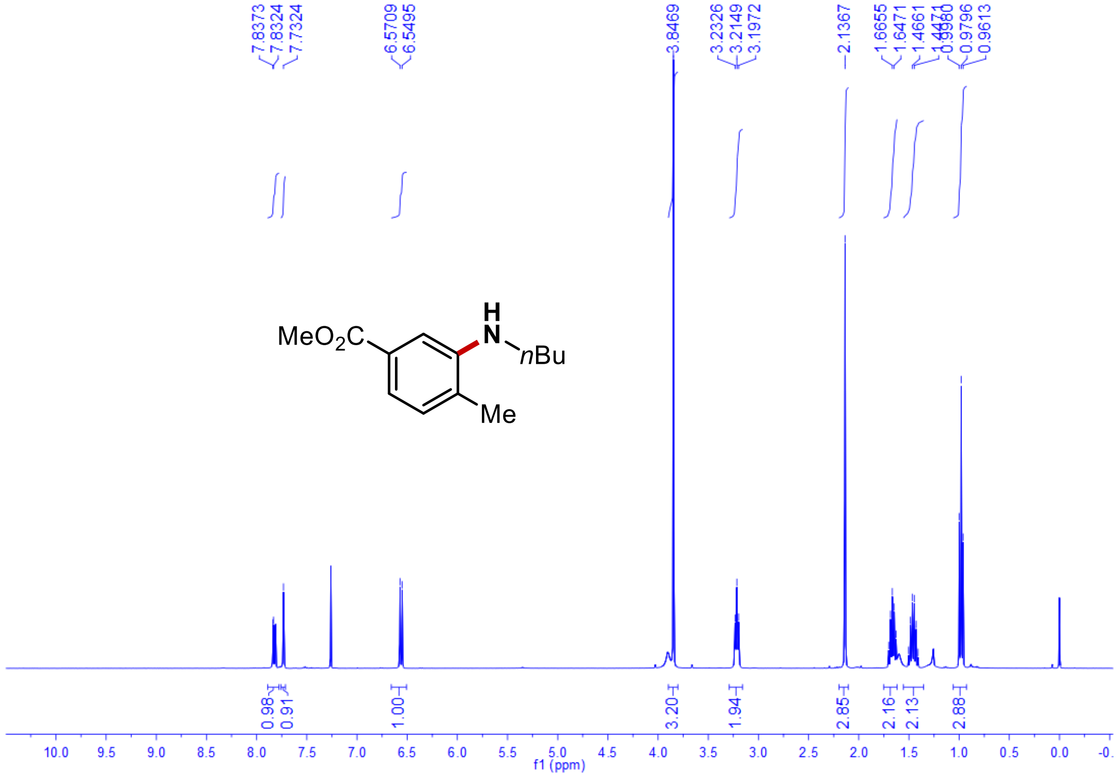

^{1}H NMR (400 MHz, $CDCl_3$) Spectrum

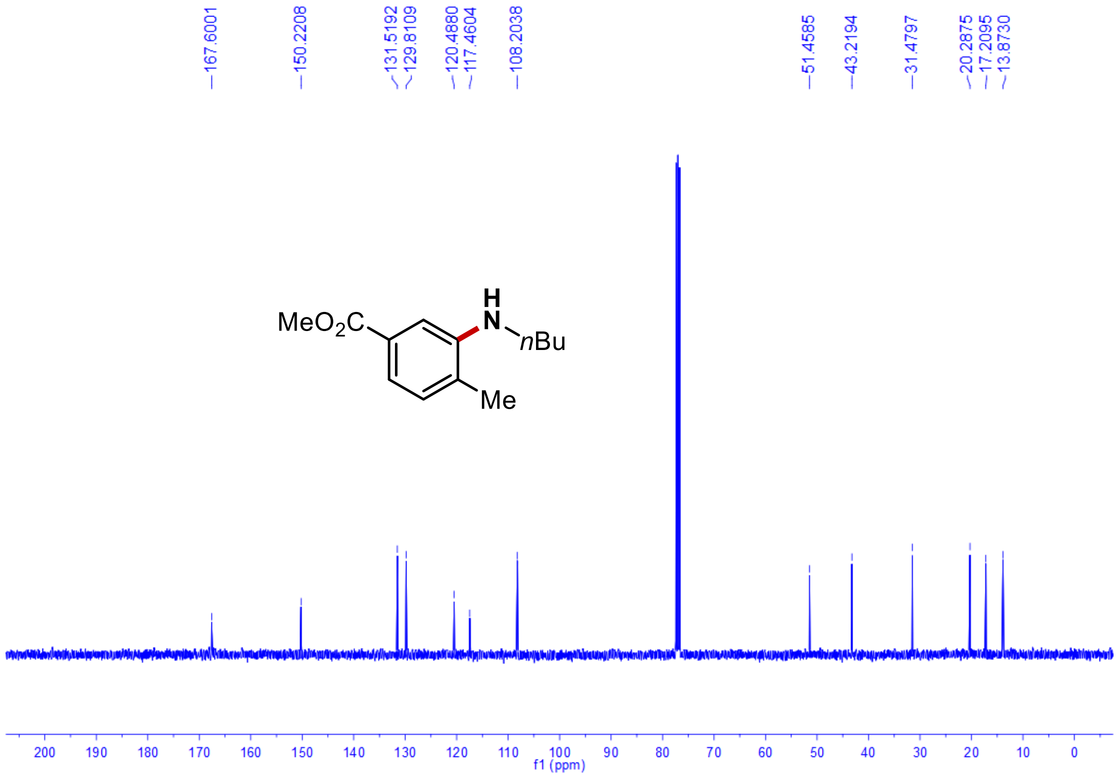

^{13}C NMR (100 MHz, $CDCl_3$) Spectrum

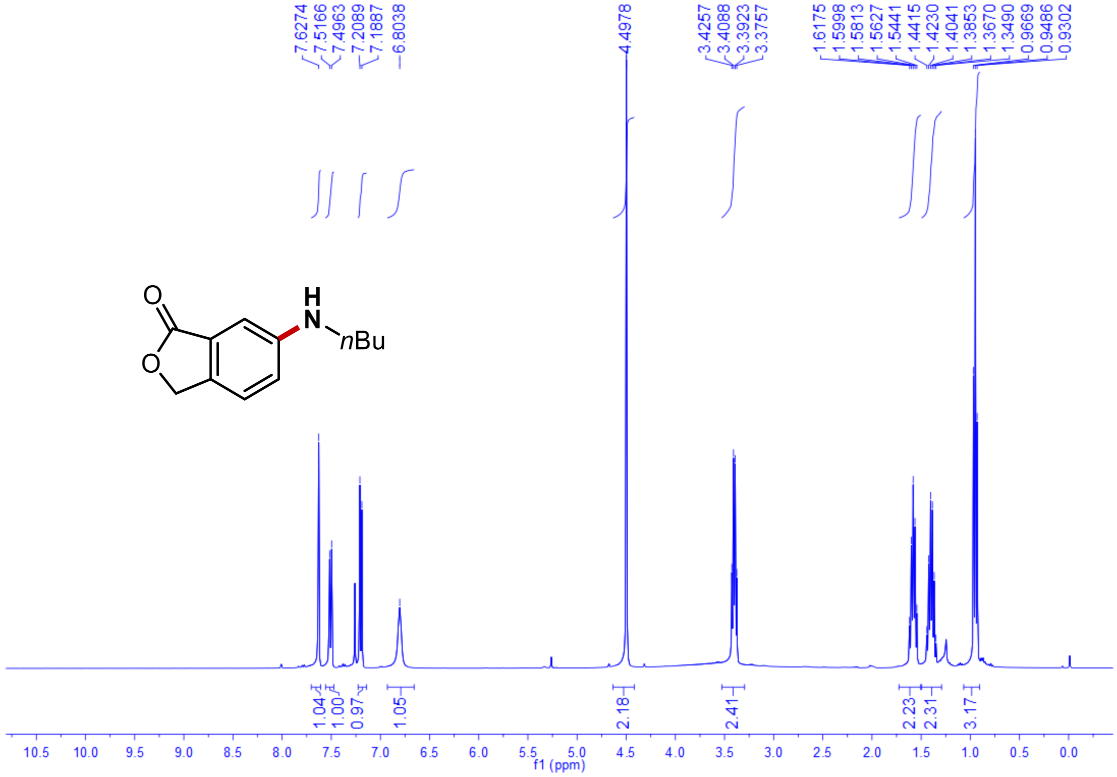

^{1}H NMR (400 MHz, $CDCl_3$) Spectrum

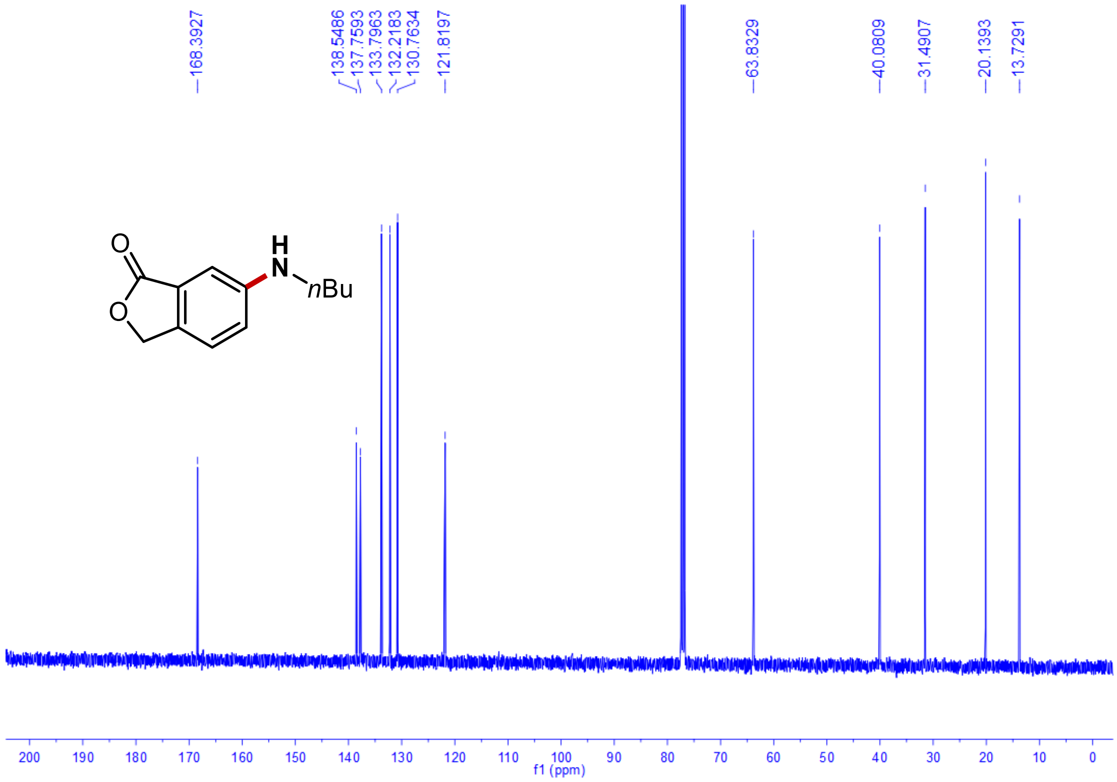

^{13}C NMR (100 MHz, $CDCl_3$) Spectrum

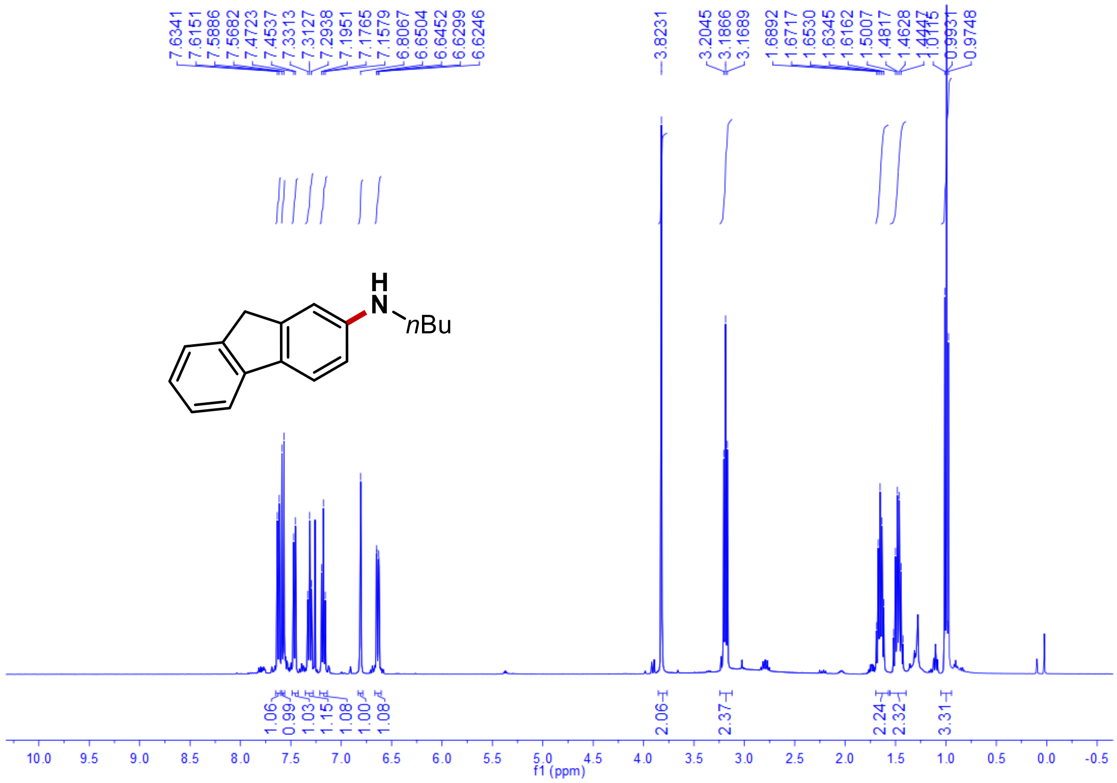

^{1}H NMR (400 MHz, $CDCl_3$) Spectrum

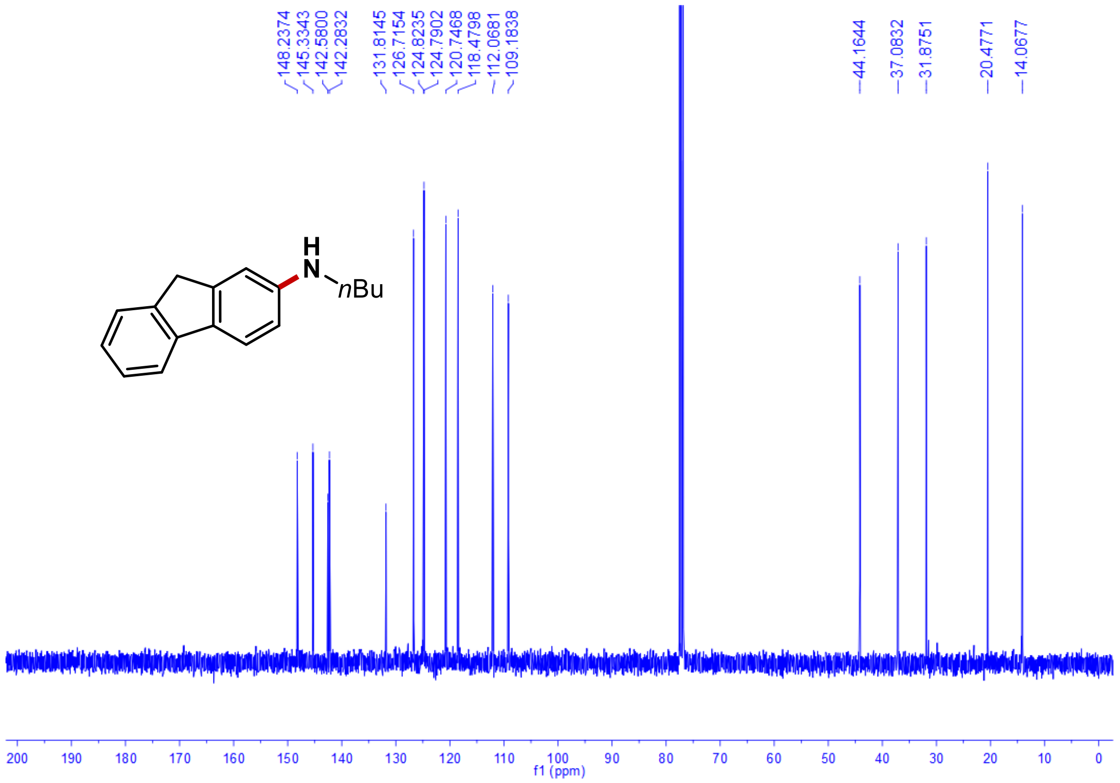

^{13}C NMR (100 MHz, $CDCl_3$) Spectrum

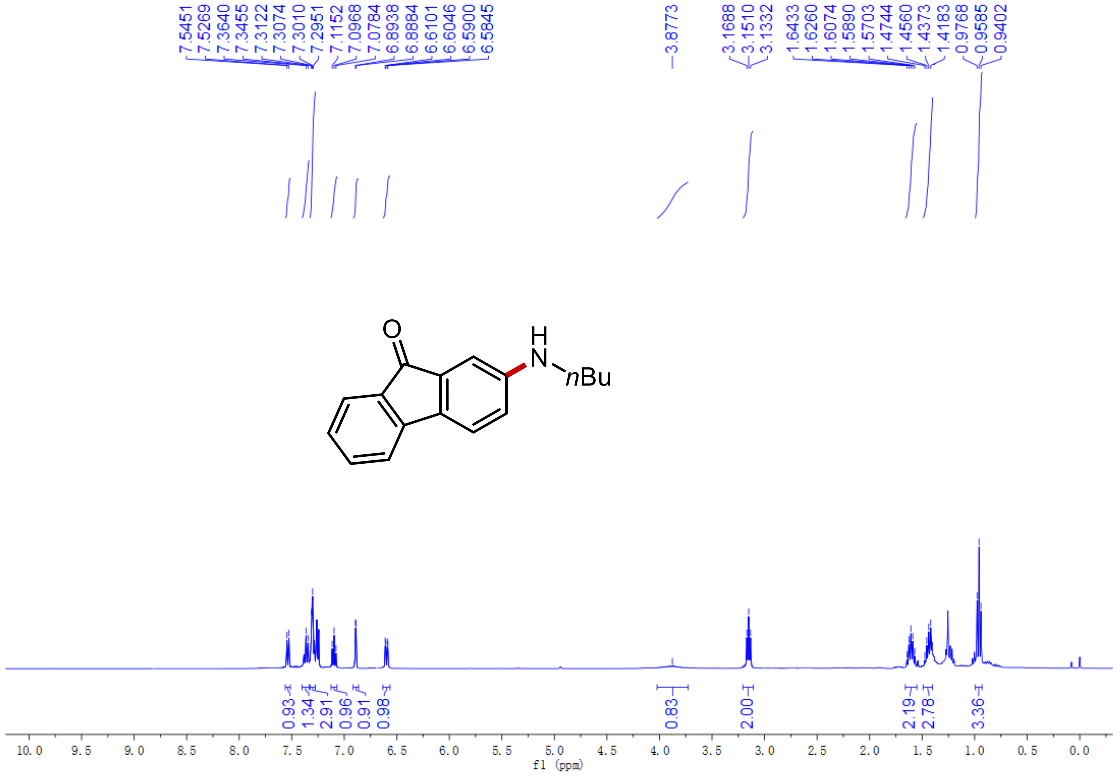

^{1}H NMR (400 MHz, $CDCl_3$) Spectrum

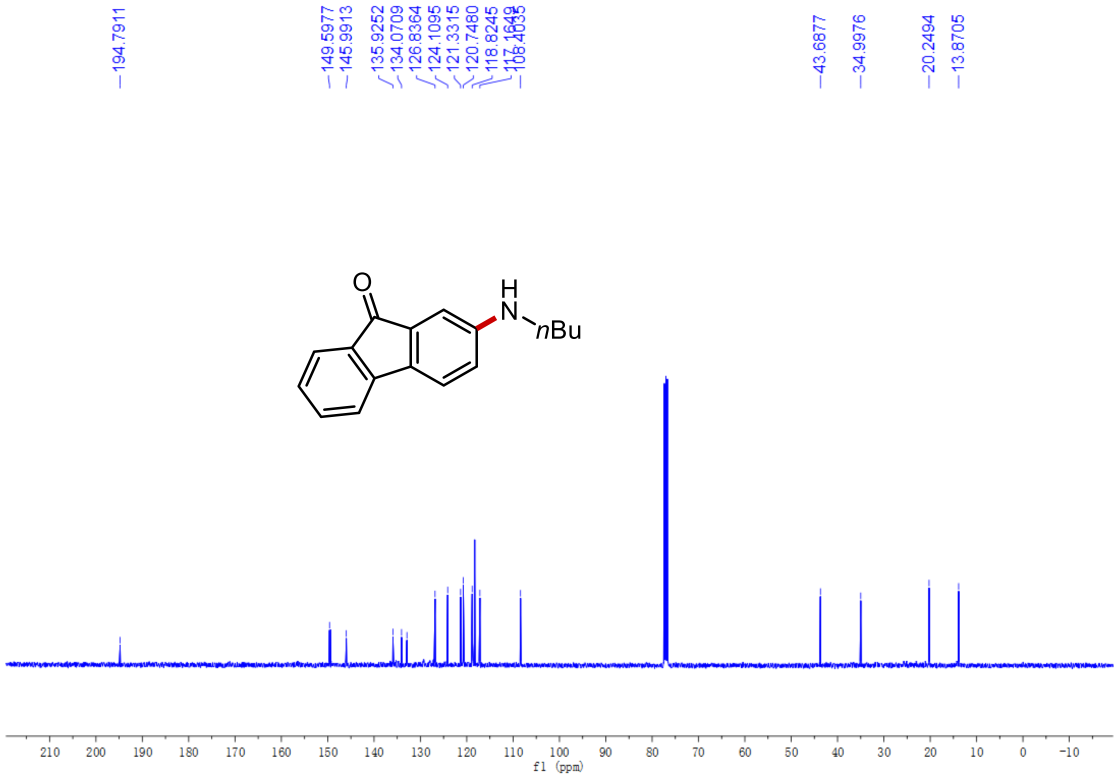

^{13}C NMR (100 MHz, $CDCl_3$) Spectrum

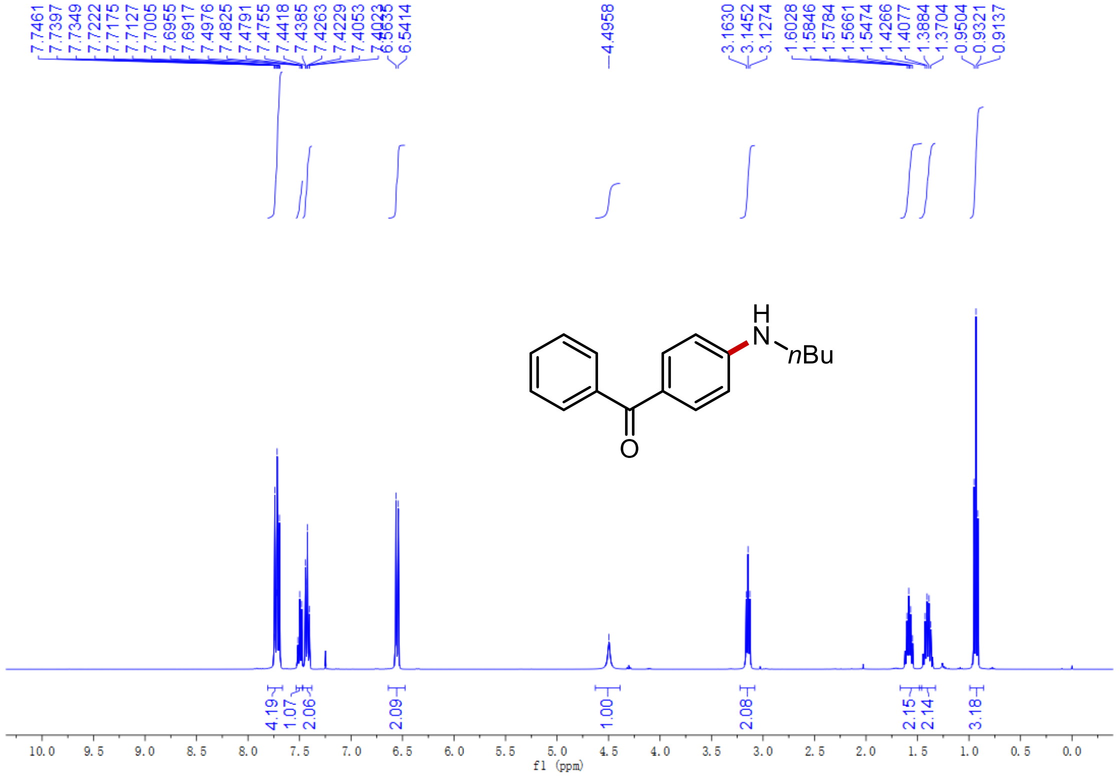

^{1}H NMR (400 MHz, $CDCl_3$) Spectrum

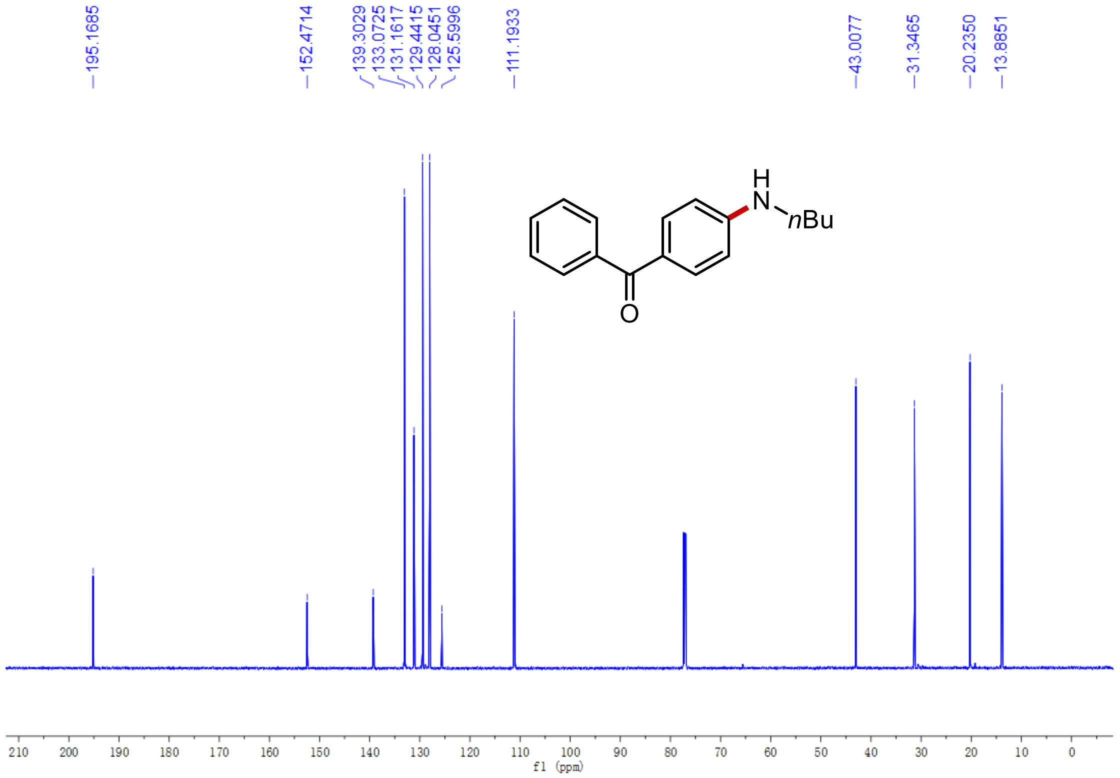

^{13}C NMR (150 MHz, $CDCl_3$) Spectrum

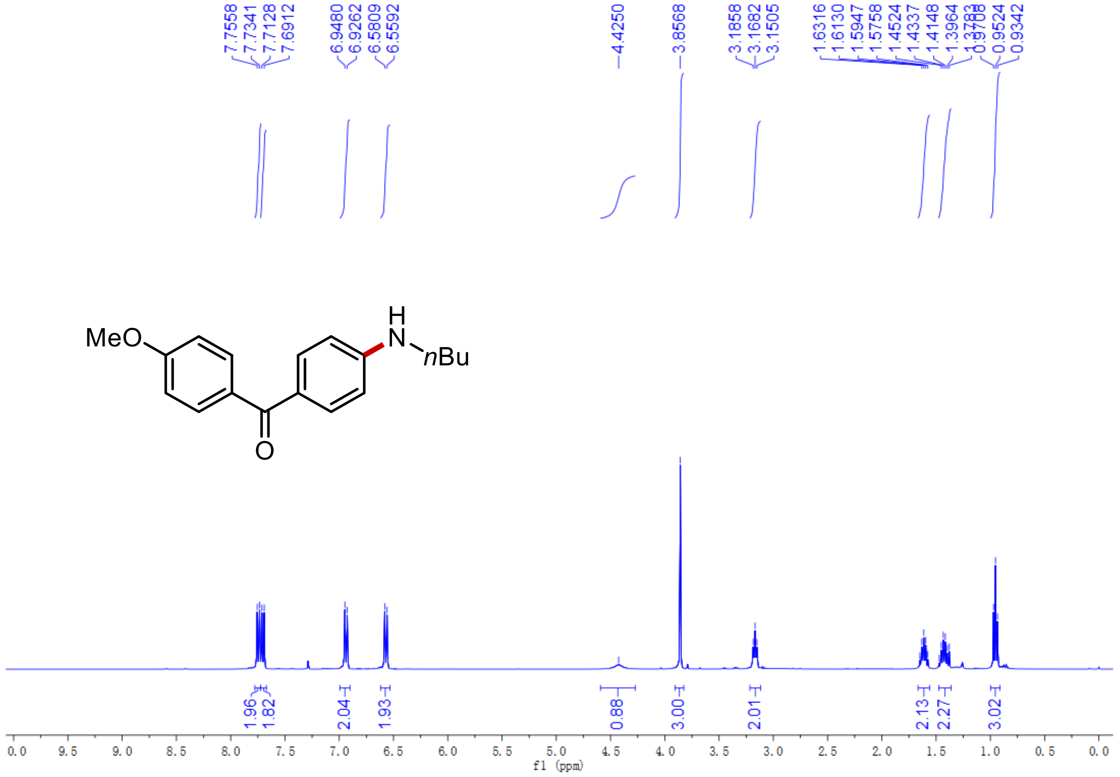

^{1}H NMR (400 MHz, $CDCl_3$) Spectrum

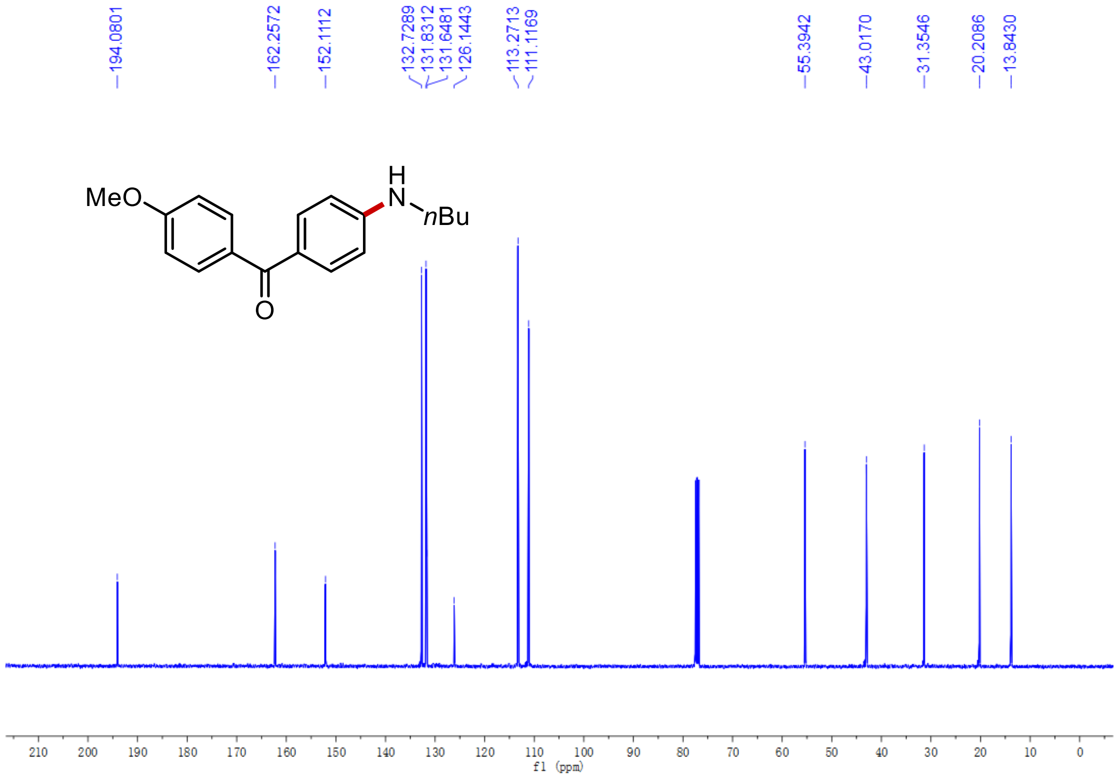

^{13}C NMR (100 MHz, $CDCl_3$) Spectrum

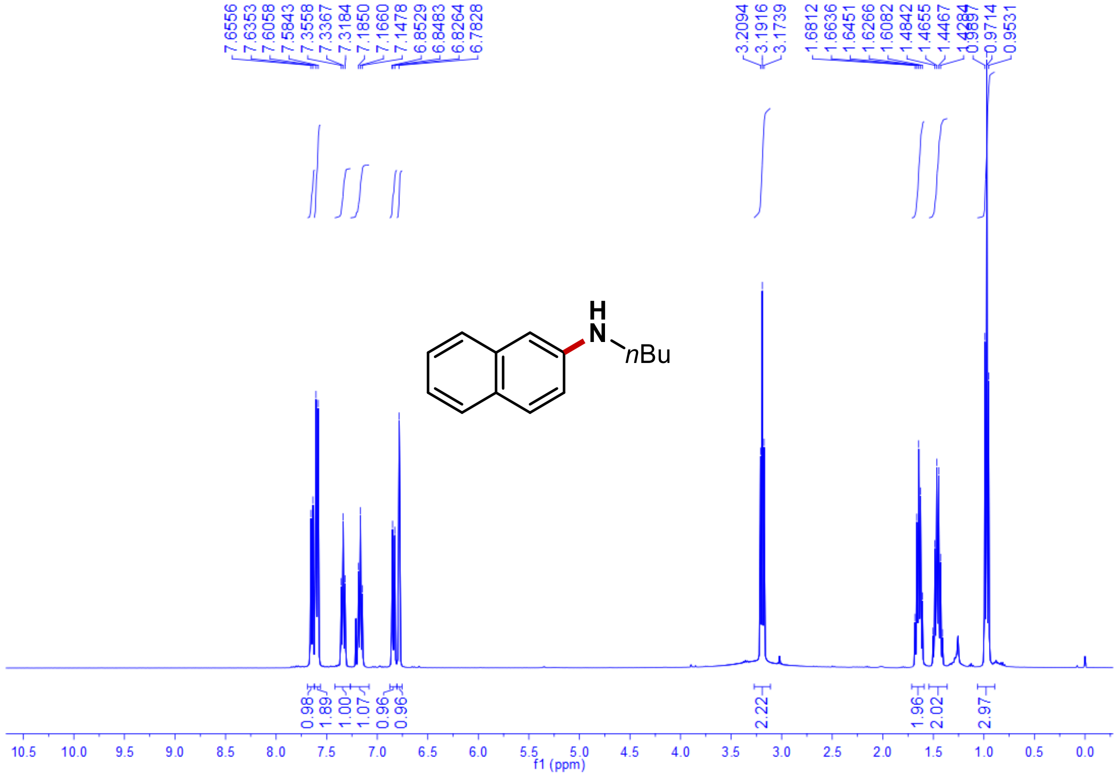

^{1}H NMR (400 MHz, $CDCl_3$) Spectrum

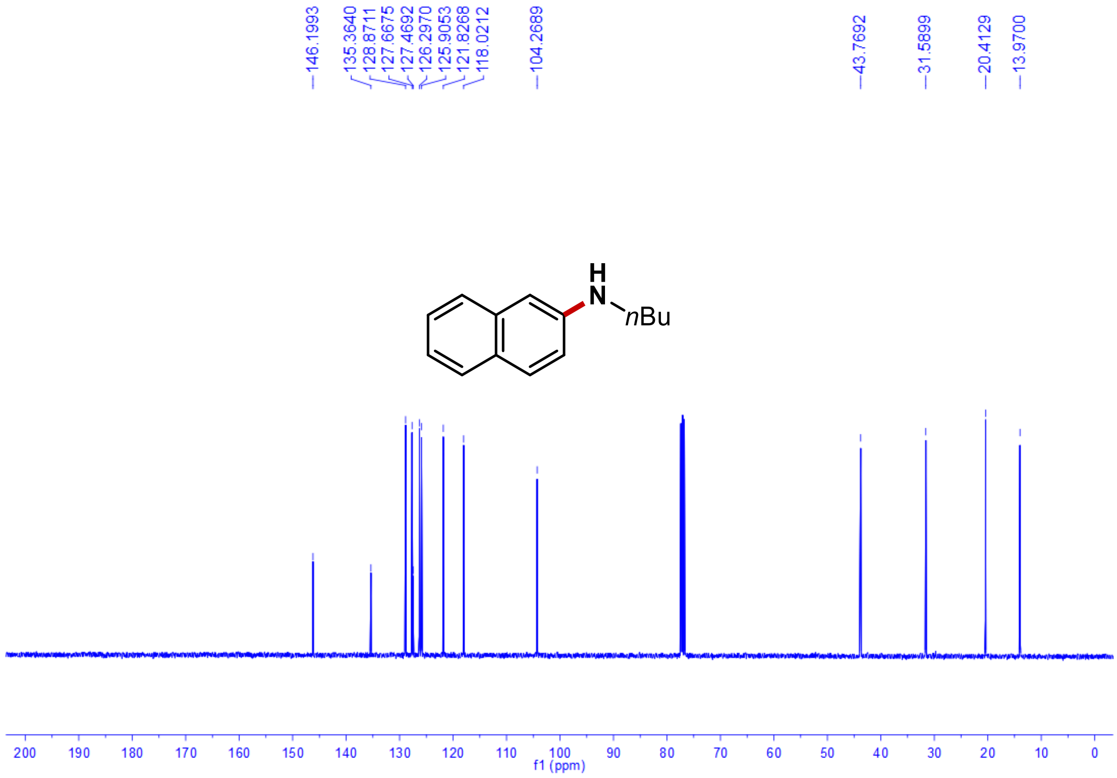

^{13}**C NMR** (100 MHz, $CDCl_3$) Spectrum

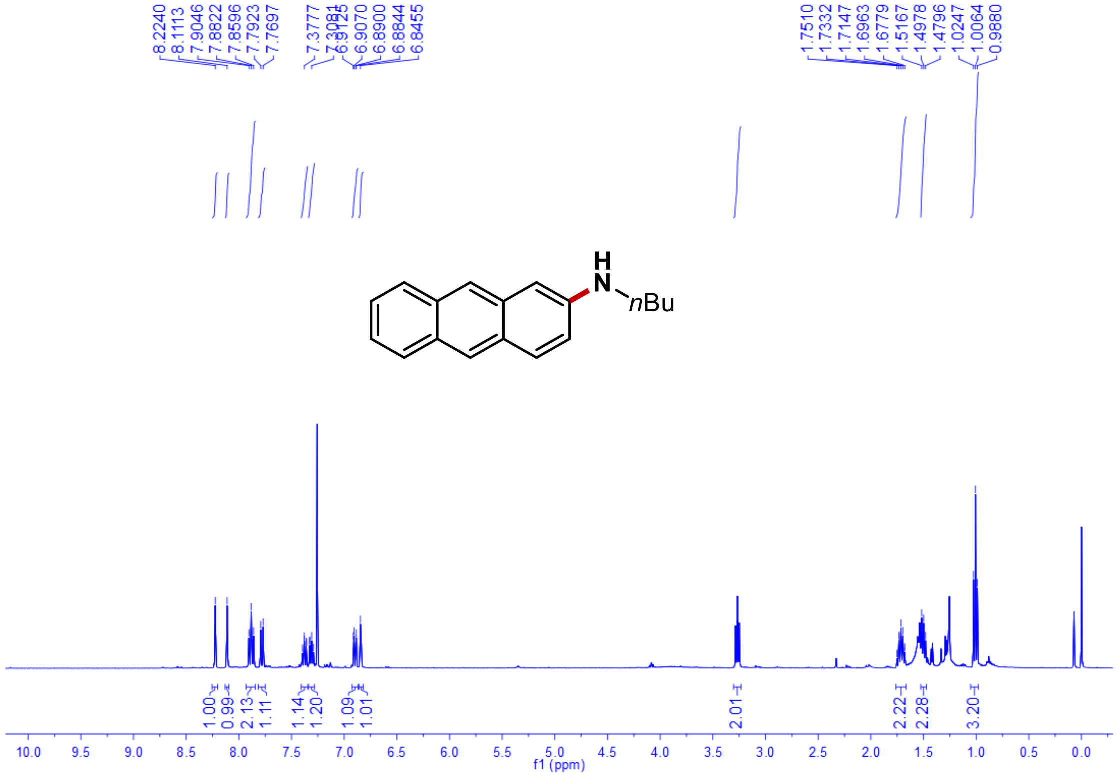

^{1}**H NMR** (400 MHz, $CDCl_3$) Spectrum

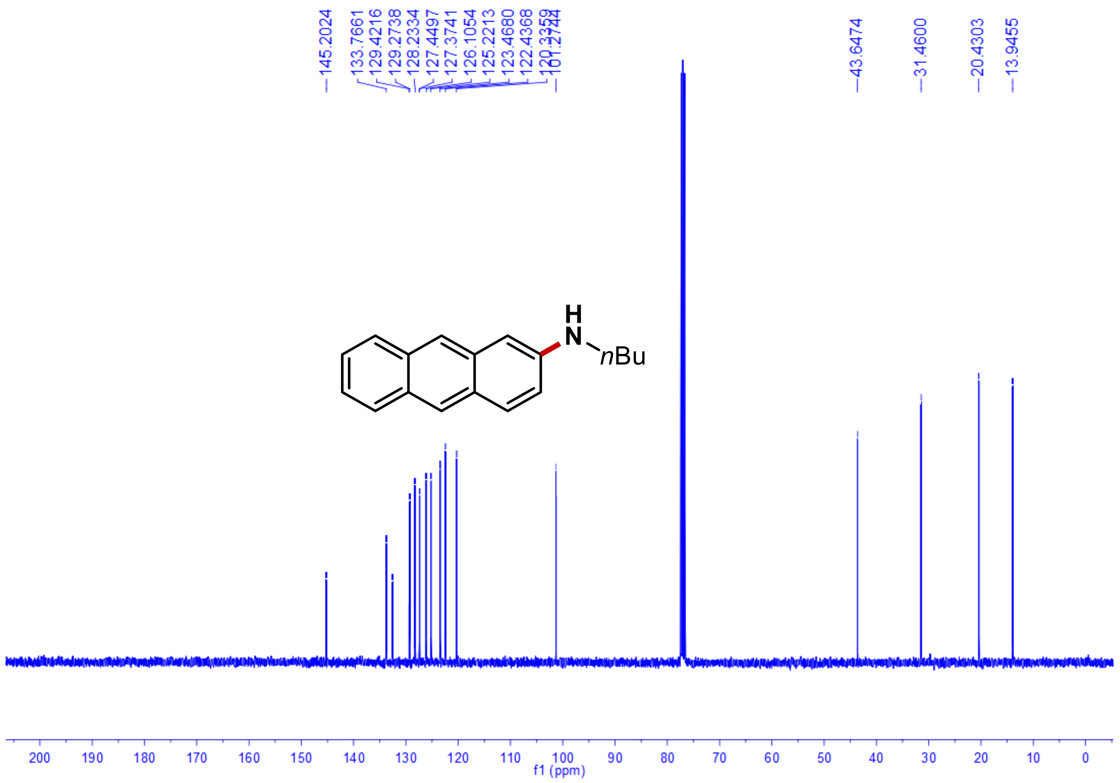

^{13}C NMR (100 MHz, $CDCl_3$) Spectrum

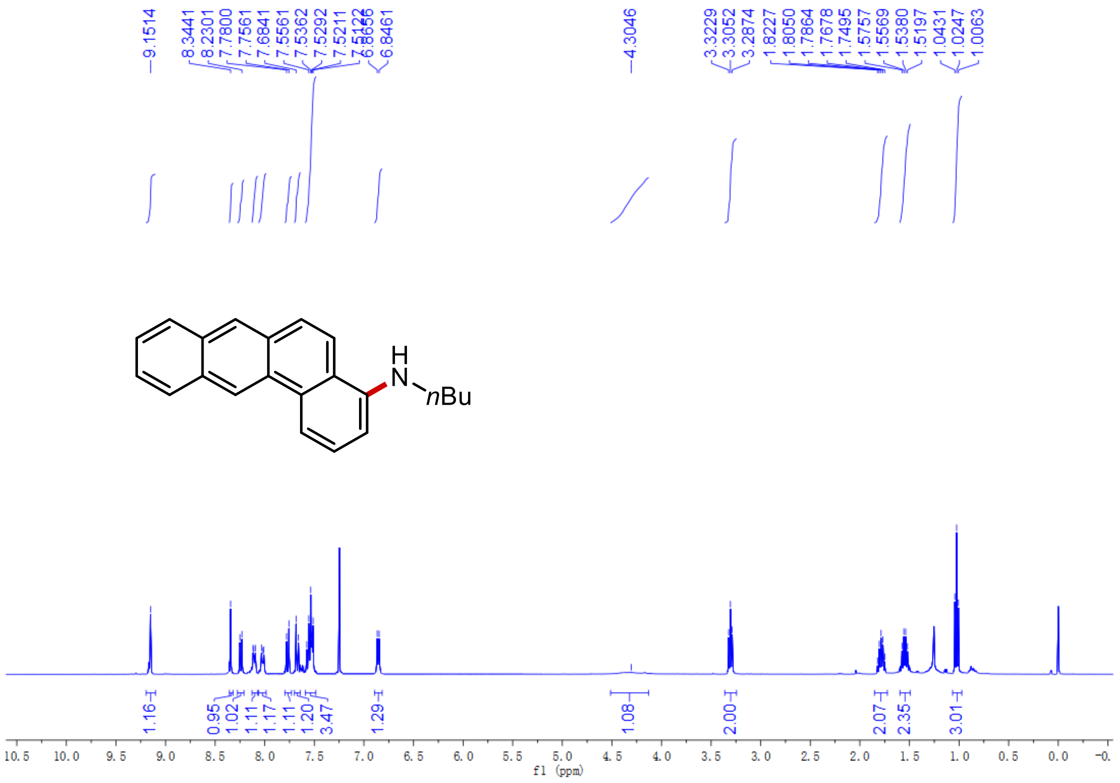

^{1}H NMR (400 MHz, $CDCl_3$) Spectrum

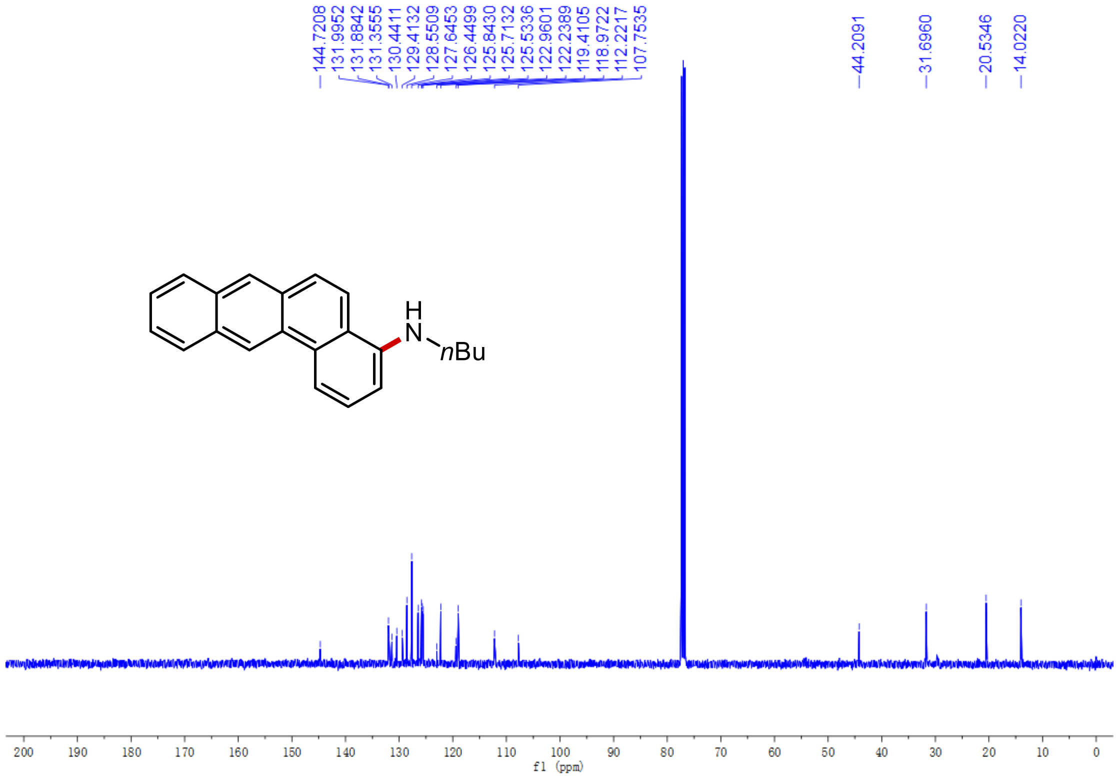

^{13}C NMR (100 MHz, $CDCl_3$) Spectrum

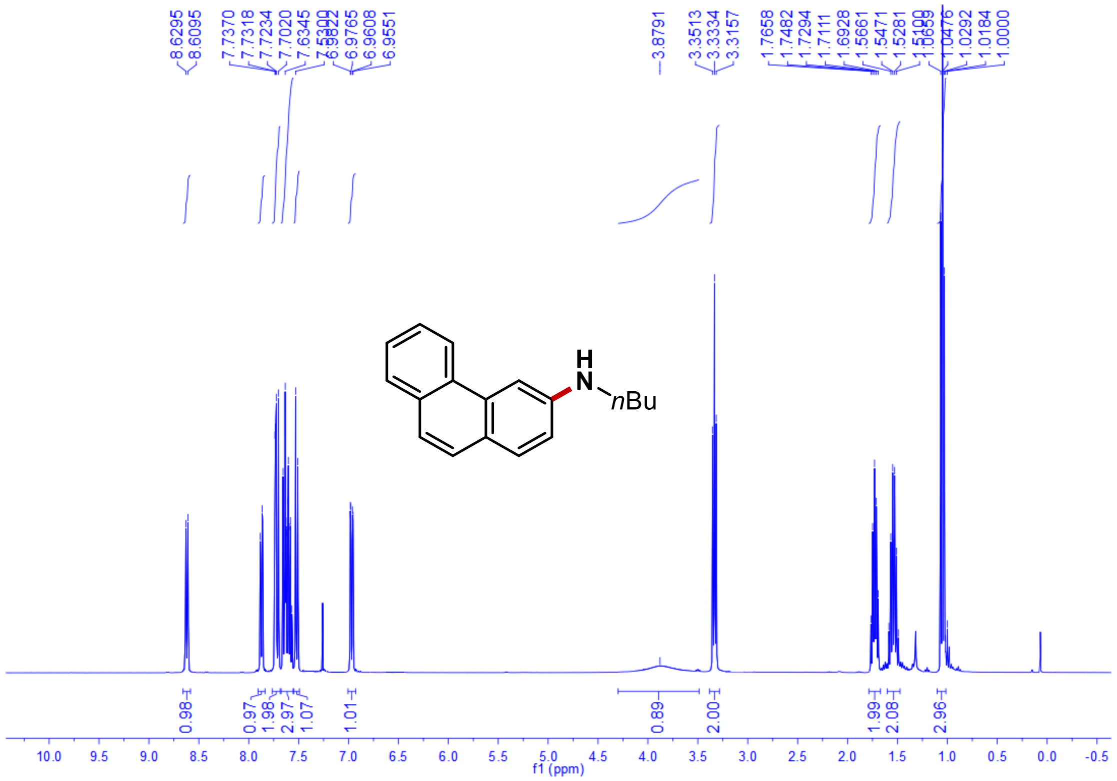

^{1}H NMR (400 MHz, $CDCl_3$) Spectrum

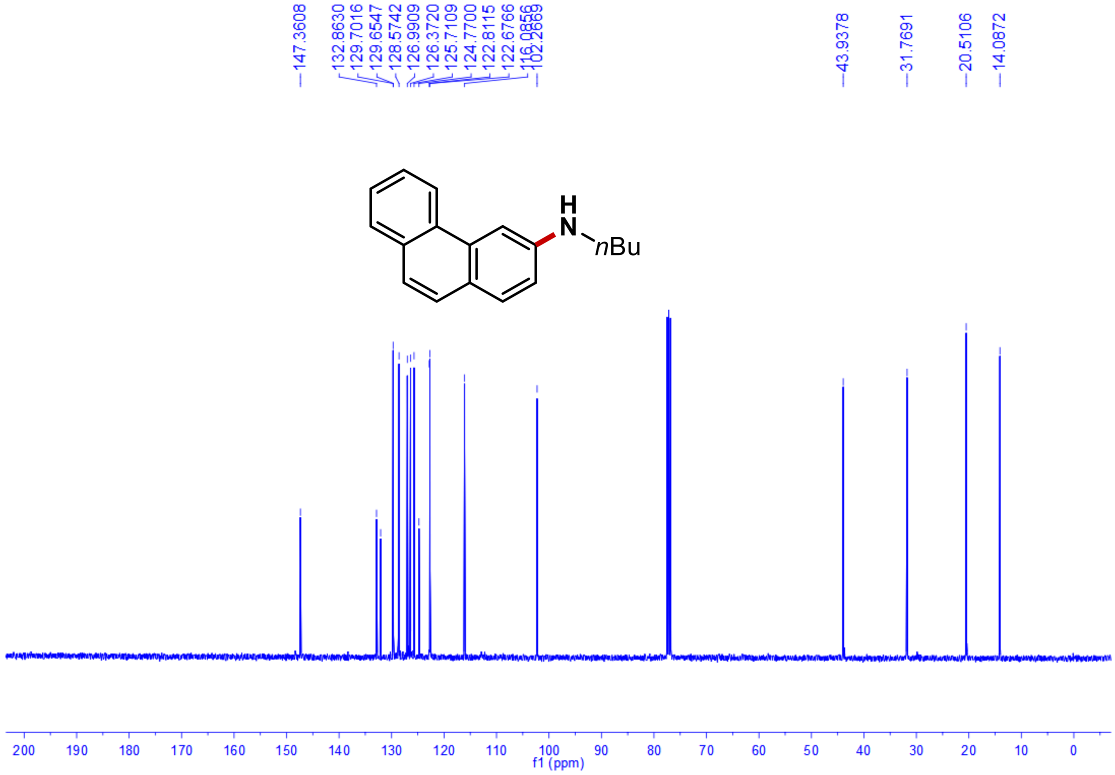

^{13}C NMR (100 MHz, $CDCl_3$) Spectrum

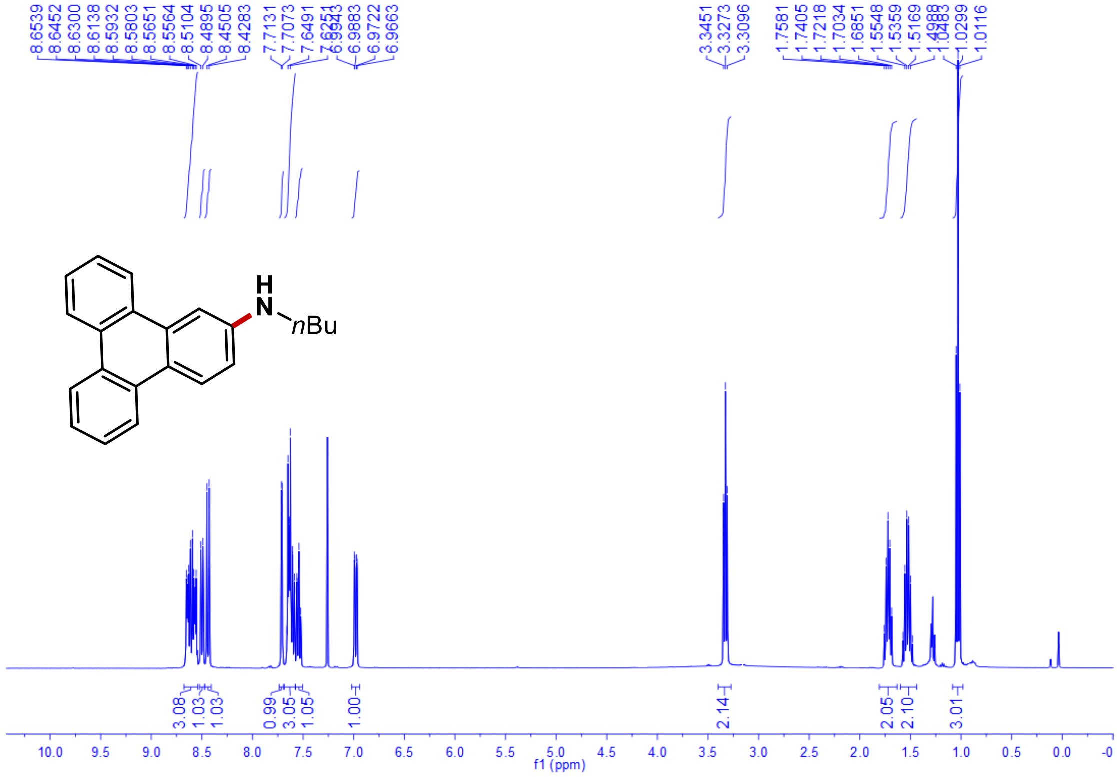

^{1}H NMR (400 MHz, $CDCl_3$) Spectrum

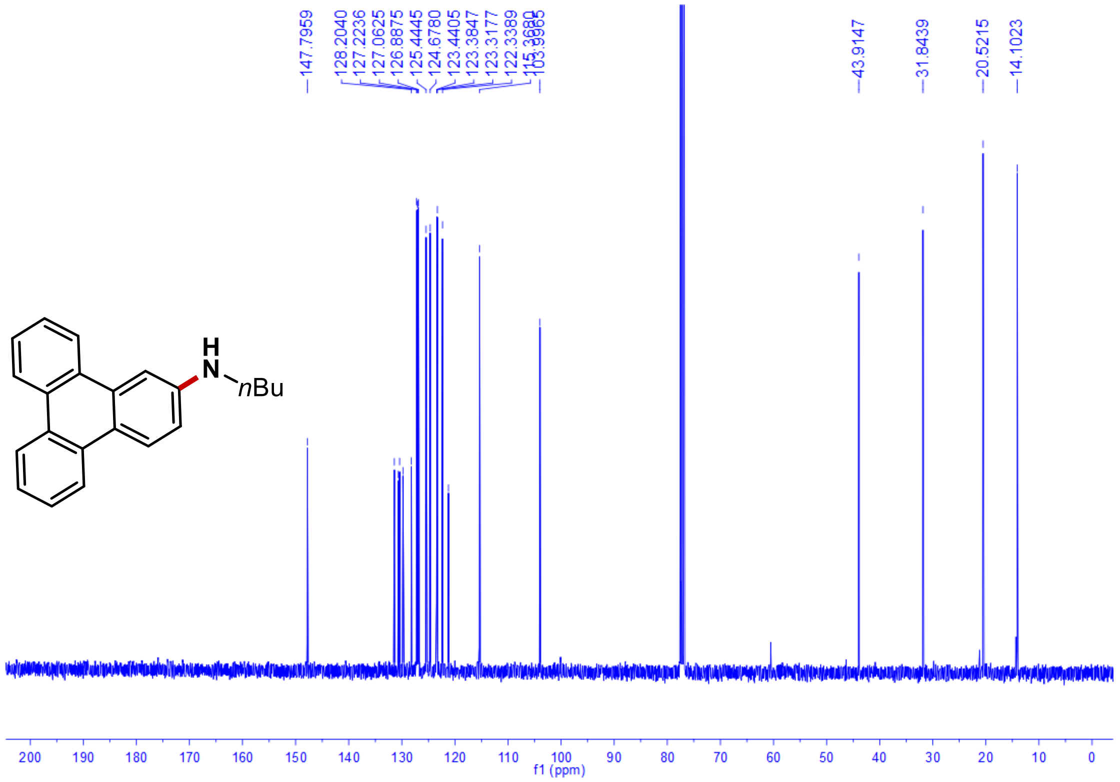

^{13}C NMR (100 MHz, $CDCl_3$) Spectrum

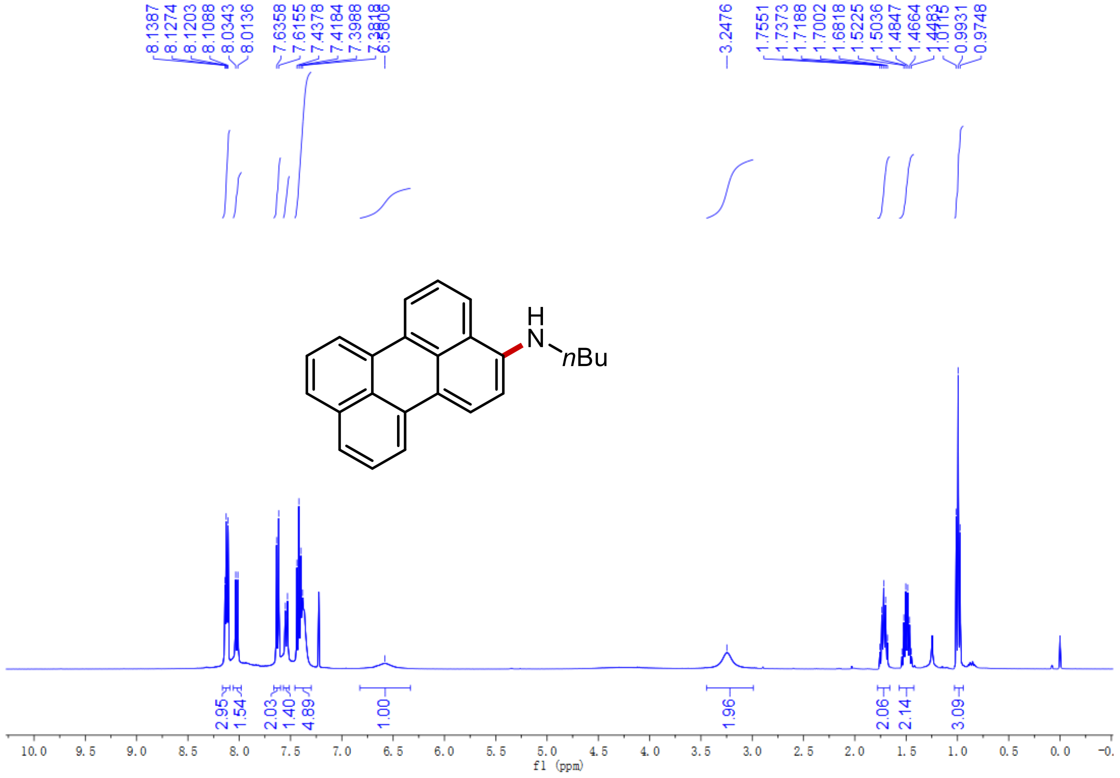

^{1}H NMR (400 MHz, $CDCl_3$) Spectrum

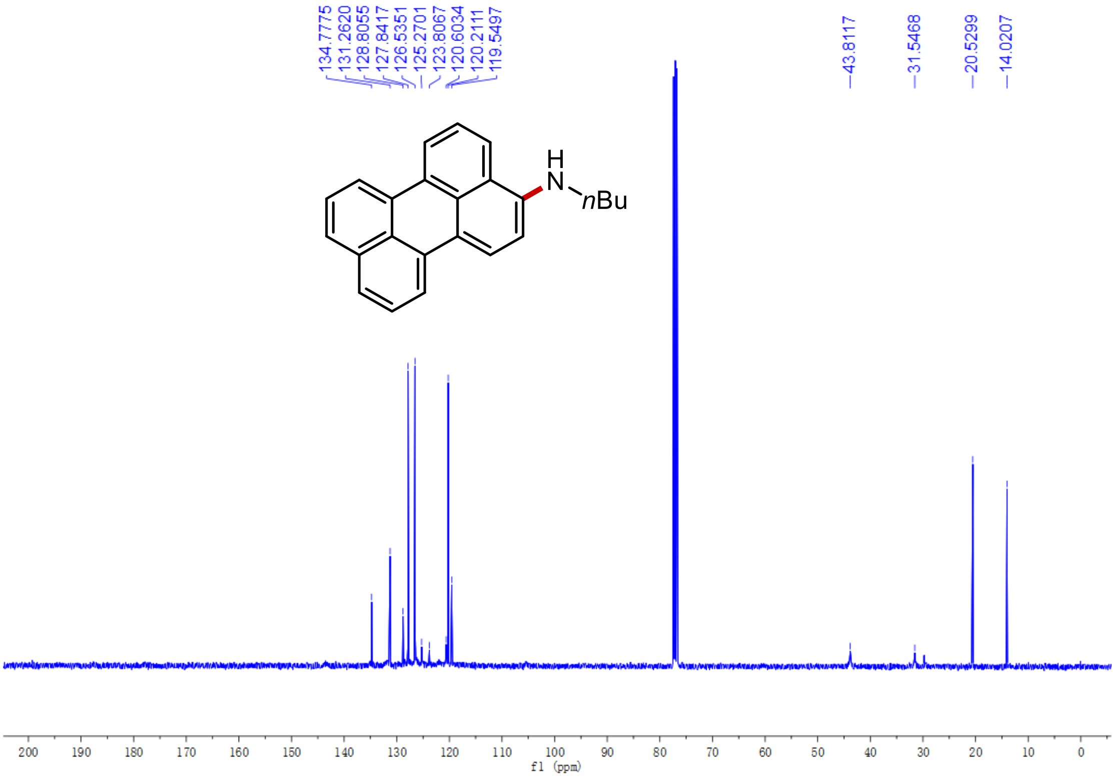

^{13}C NMR (100 MHz, $CDCl_3$) Spectrum

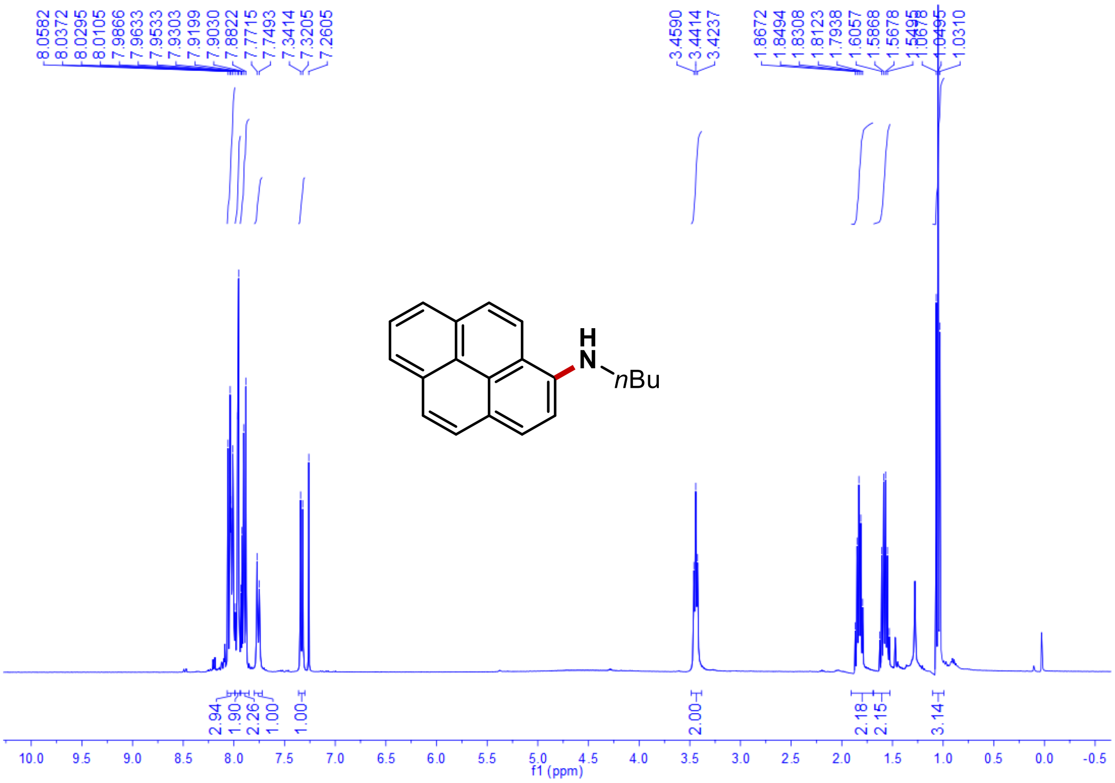

^{1}H NMR (400 MHz, $CDCl_3$) Spectrum

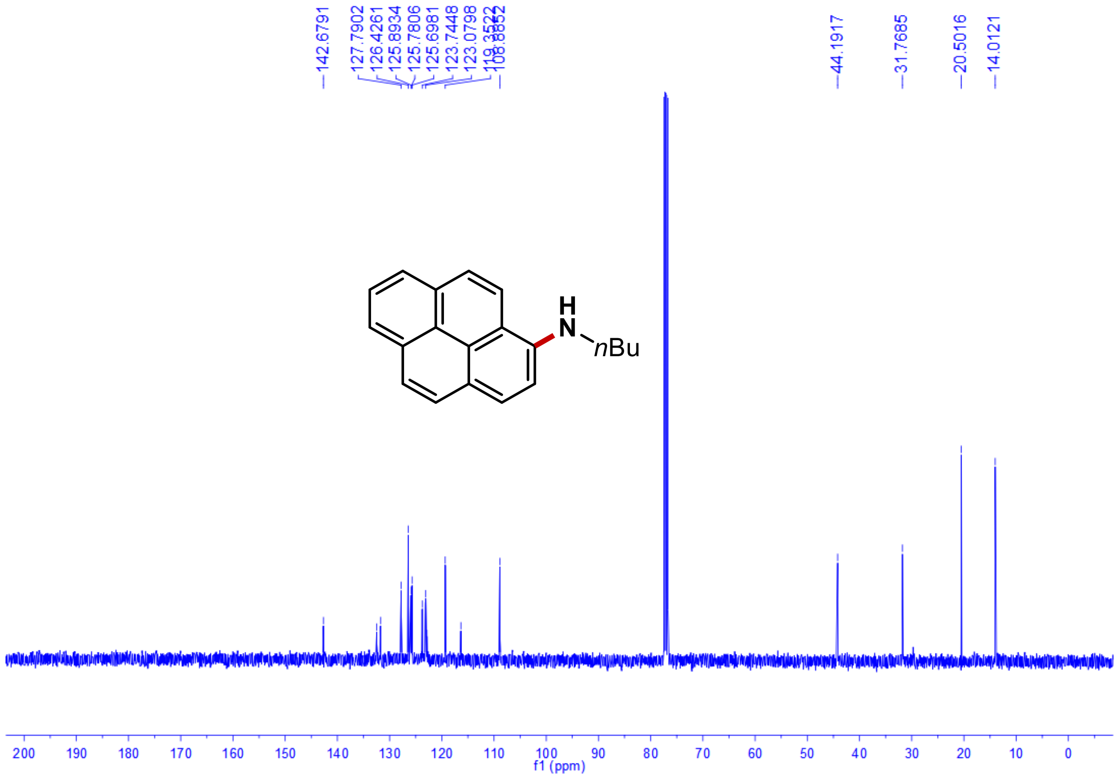

^{13}C NMR (100 MHz, $CDCl_3$) Spectrum

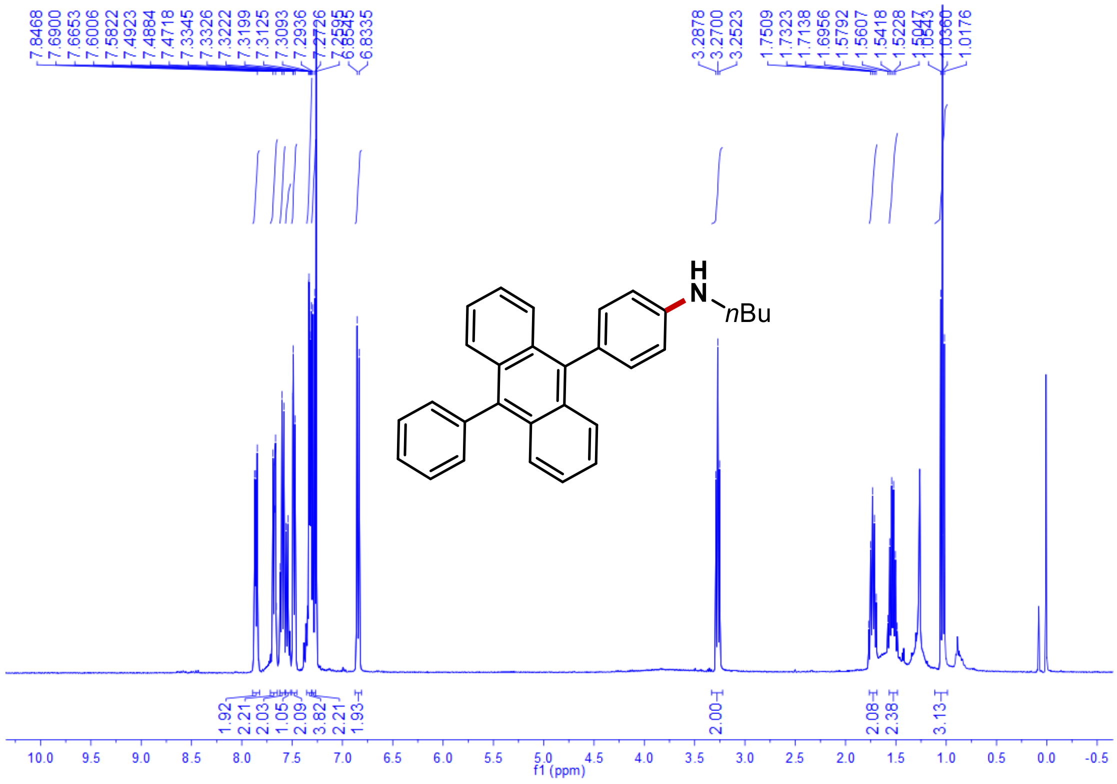

^{1}H NMR (400 MHz, $CDCl_3$) Spectrum

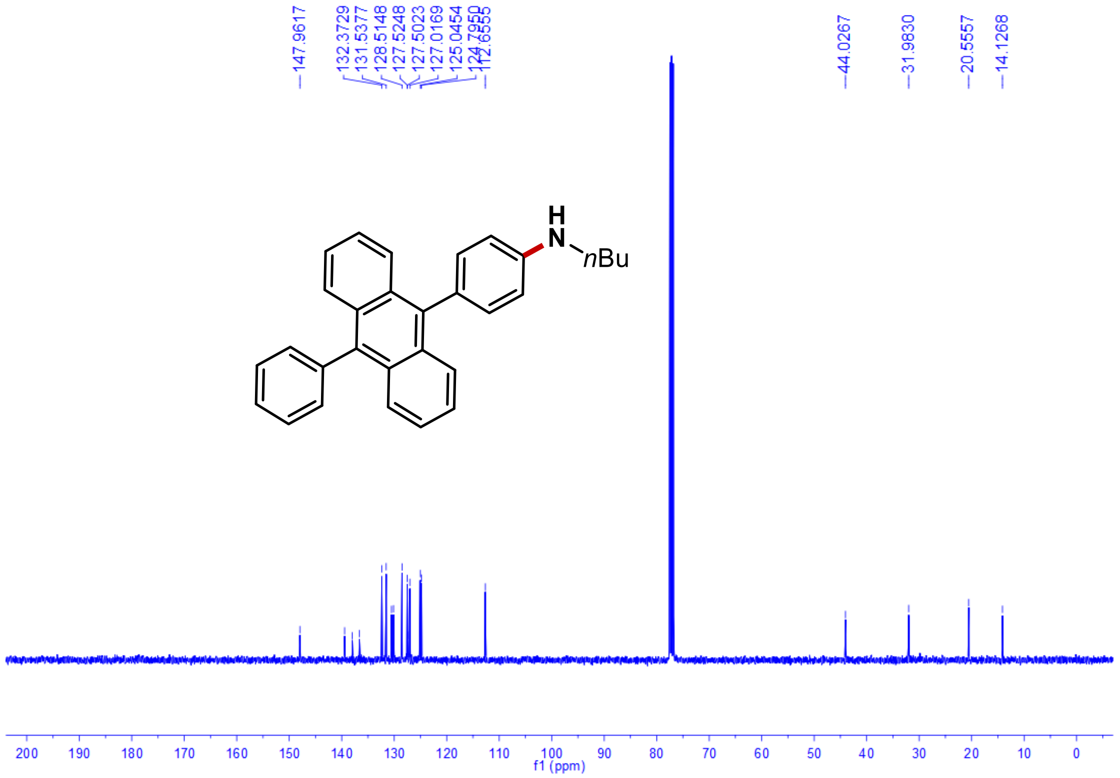

^{13}C NMR (100 MHz, $CDCl_3$) Spectrum

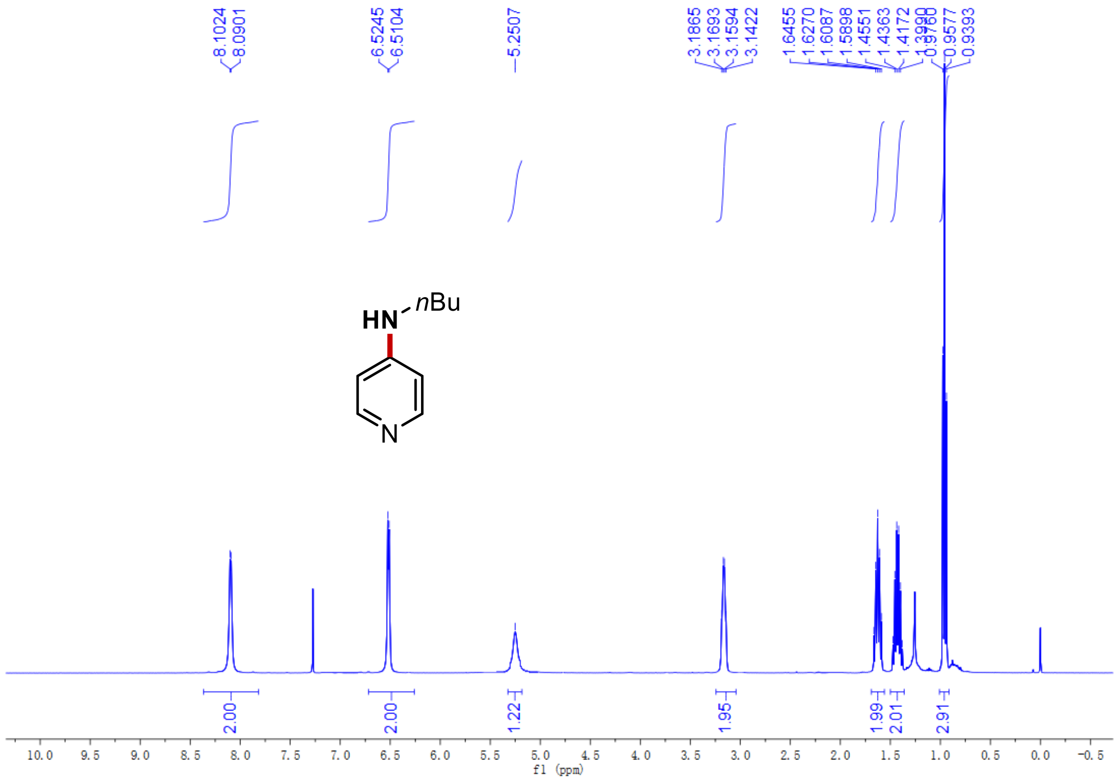

^{1}H NMR (400 MHz, $CDCl_3$) Spectrum

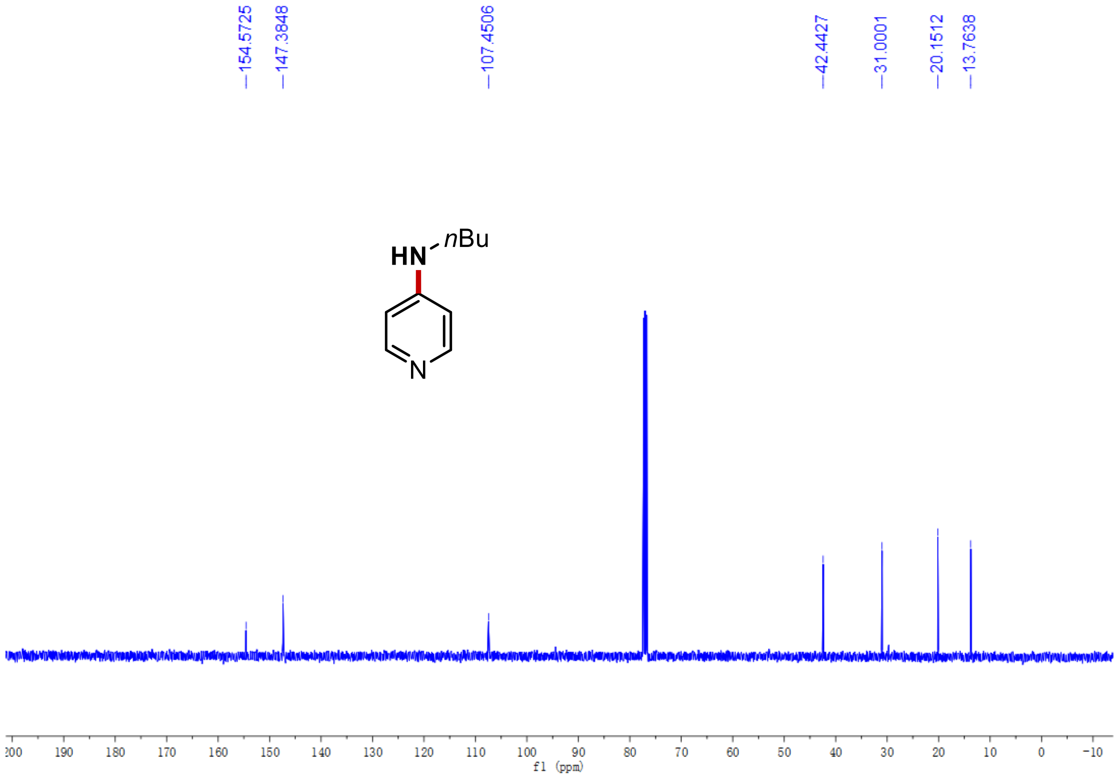

^{13}C NMR (100 MHz, $CDCl_3$) Spectrum

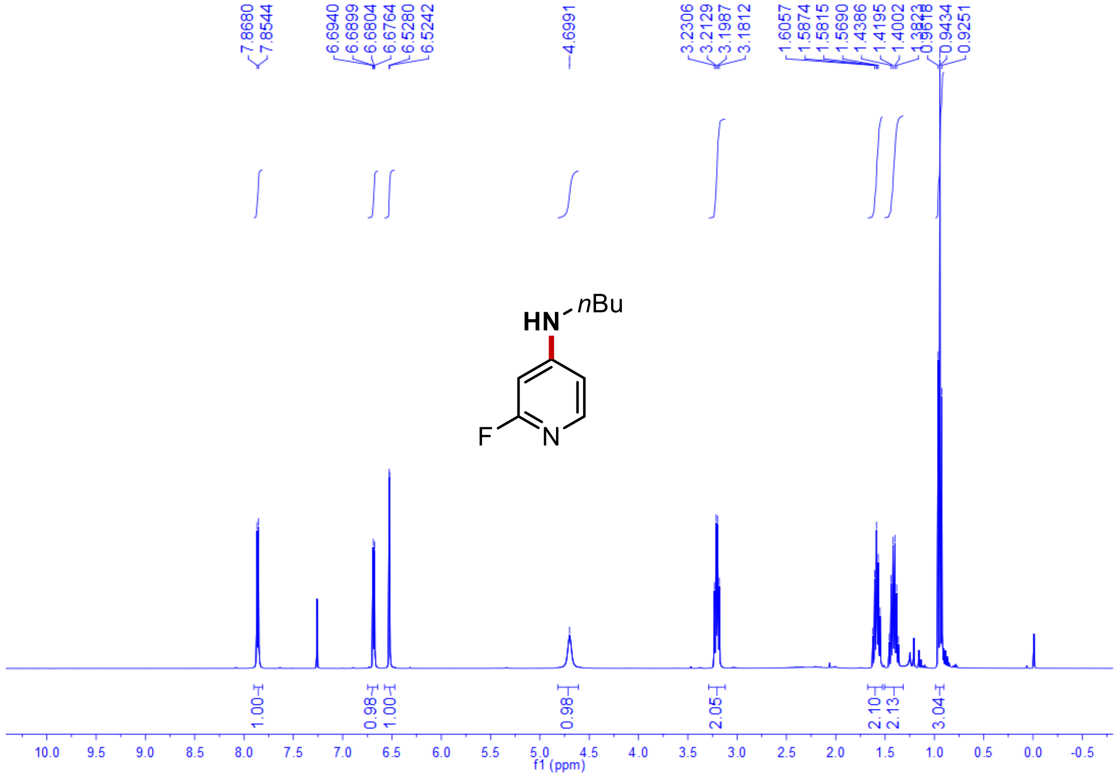

^{1}H NMR (400 MHz, $CDCl_3$) Spectrum

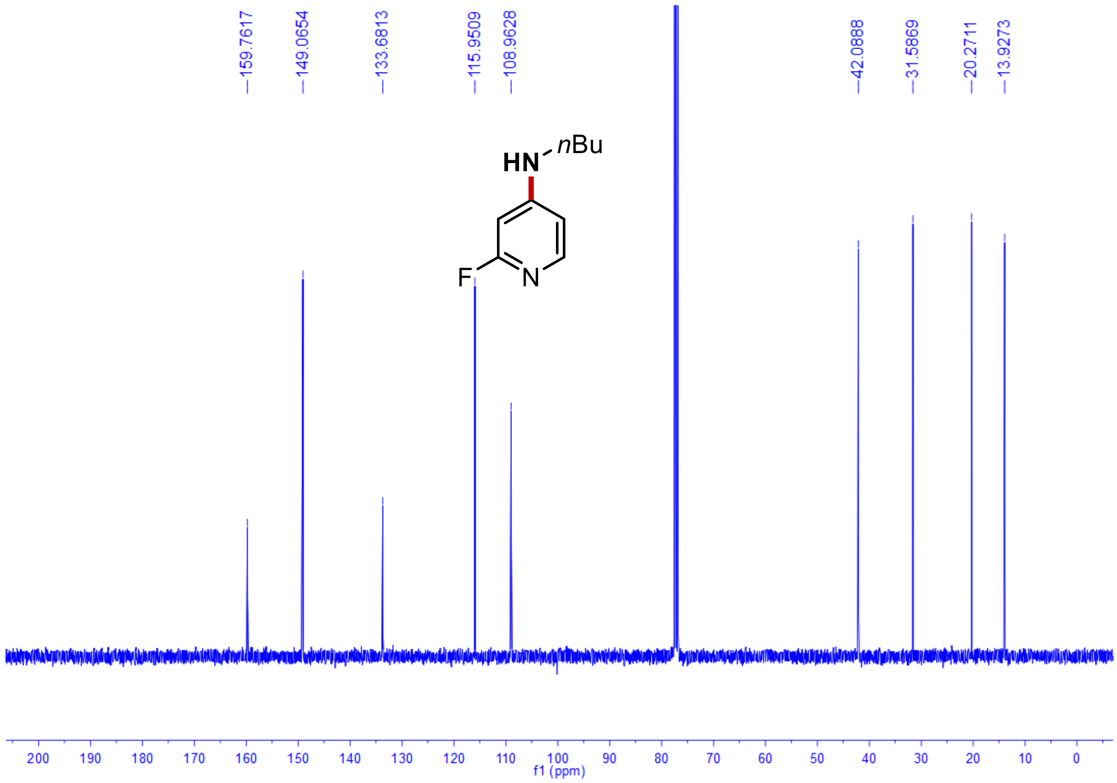

^{13}C NMR (100 MHz, $CDCl_3$) Spectrum

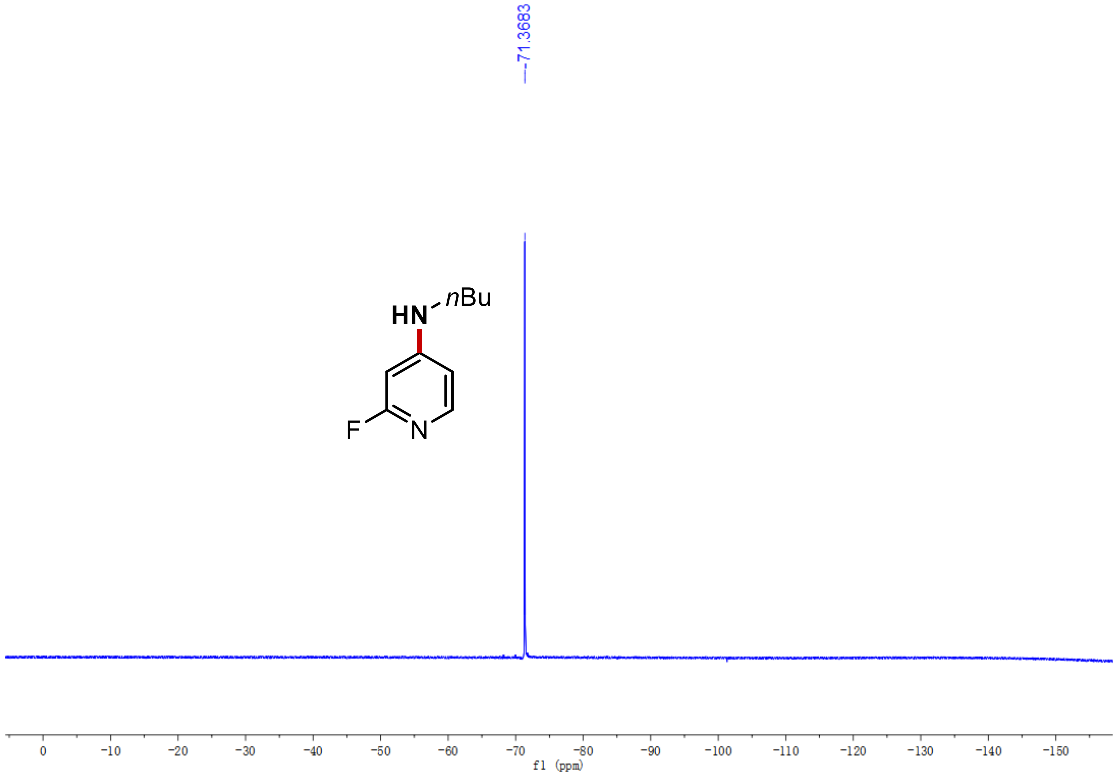

^{19}F NMR (376 MHz, $CDCl_3$) Spectrum

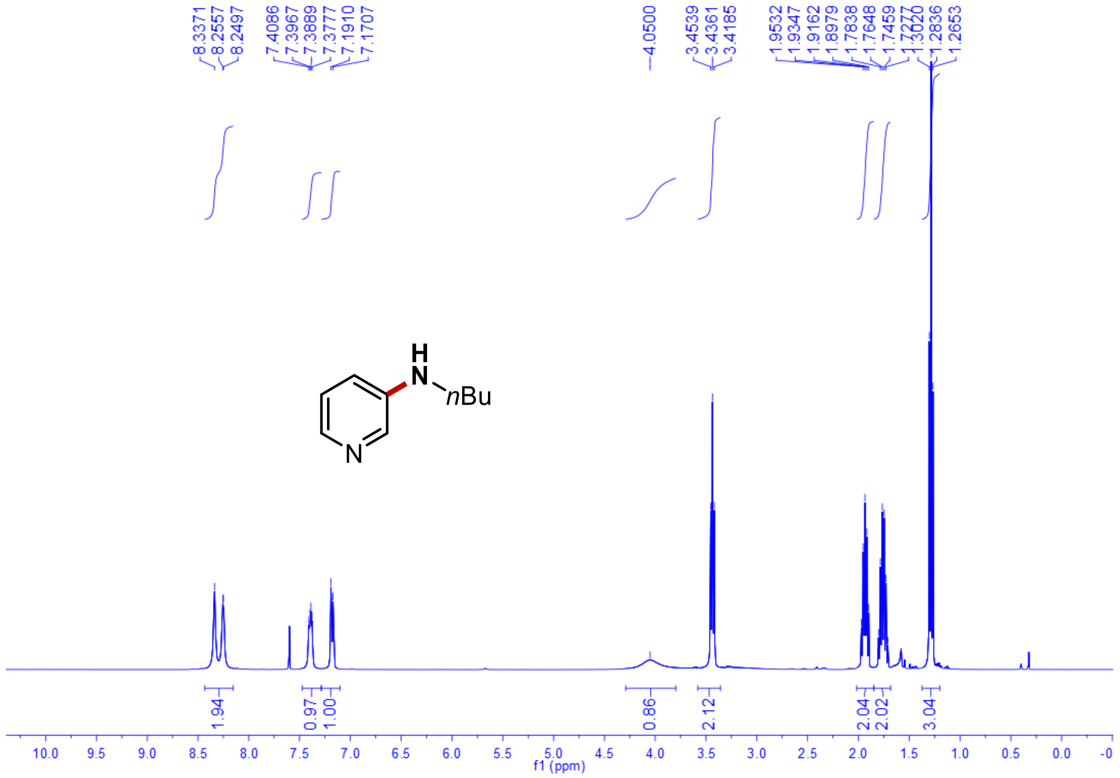

^{1}H NMR (400 MHz, $CDCl_3$) Spectrum

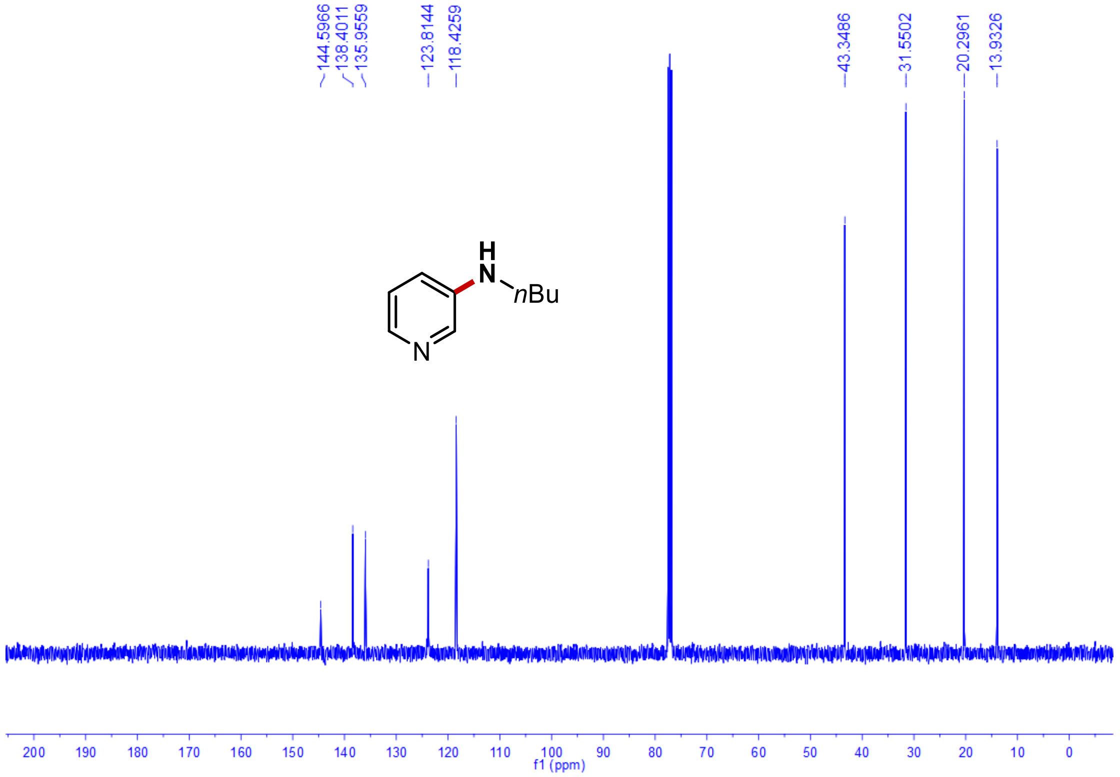

^{13}C NMR (100 MHz, $CDCl_3$) Spectrum

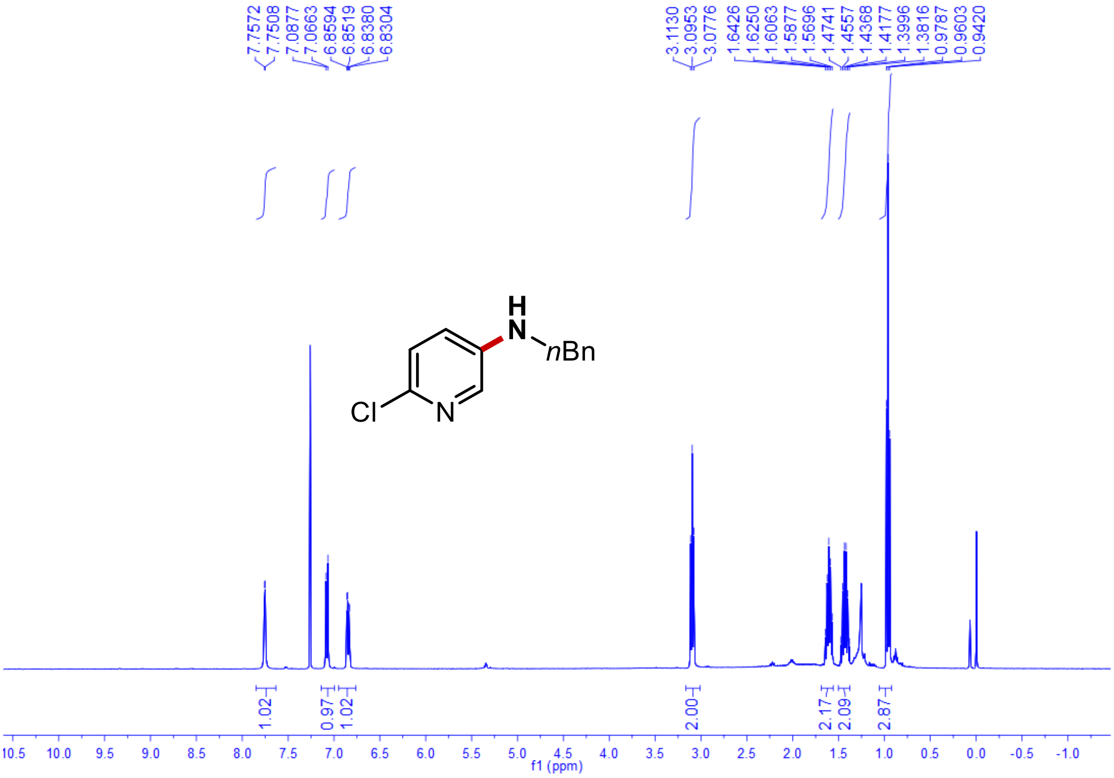

^{1}H NMR (400 MHz, $CDCl_3$) Spectrum

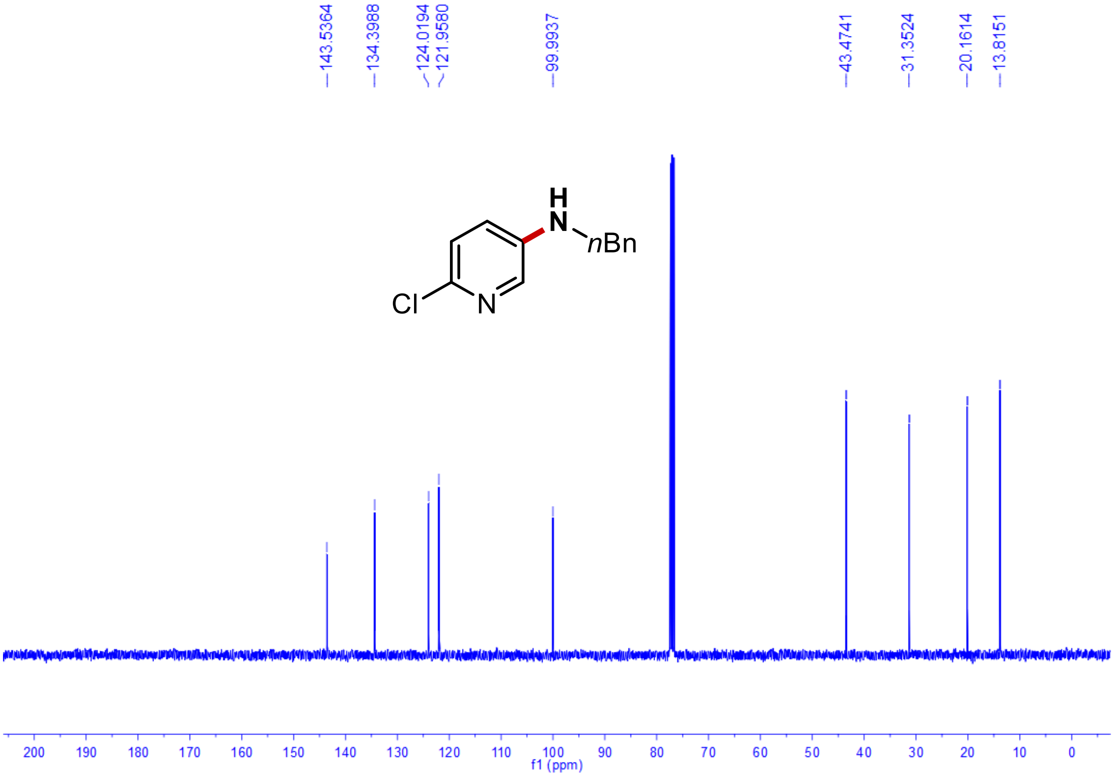

^{13}C NMR (100 MHz, $CDCl_3$) Spectrum

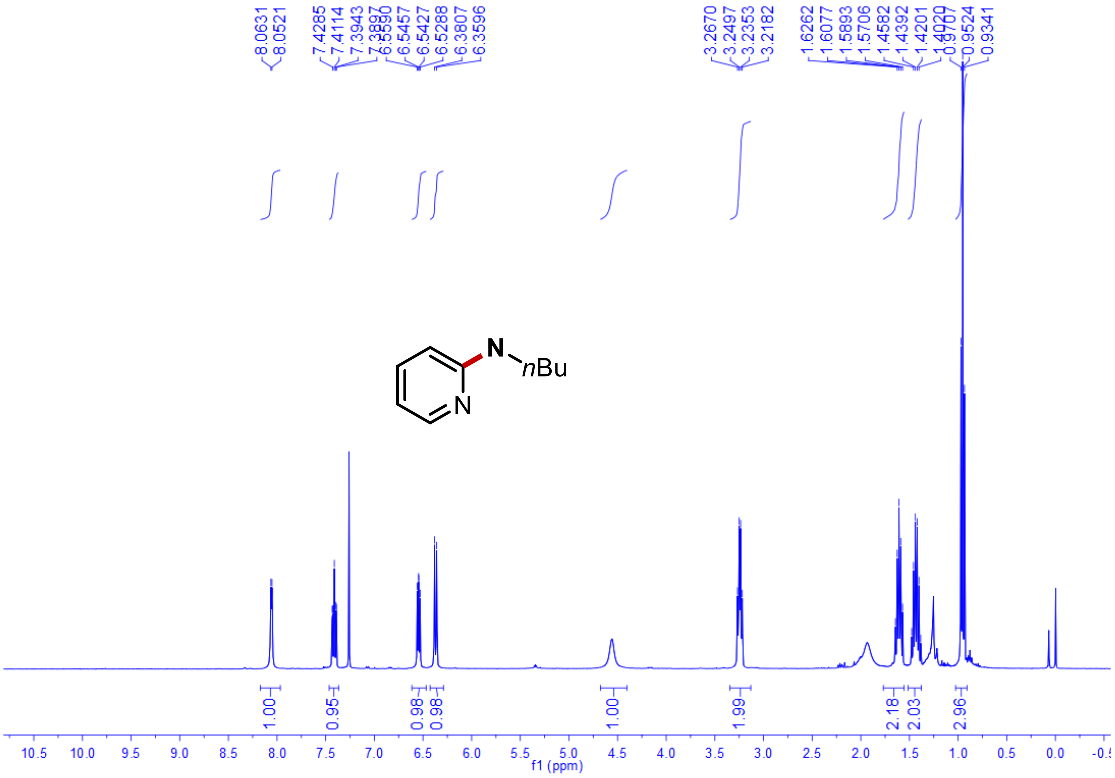

^{1}H NMR (400 MHz, $CDCl_3$) Spectrum

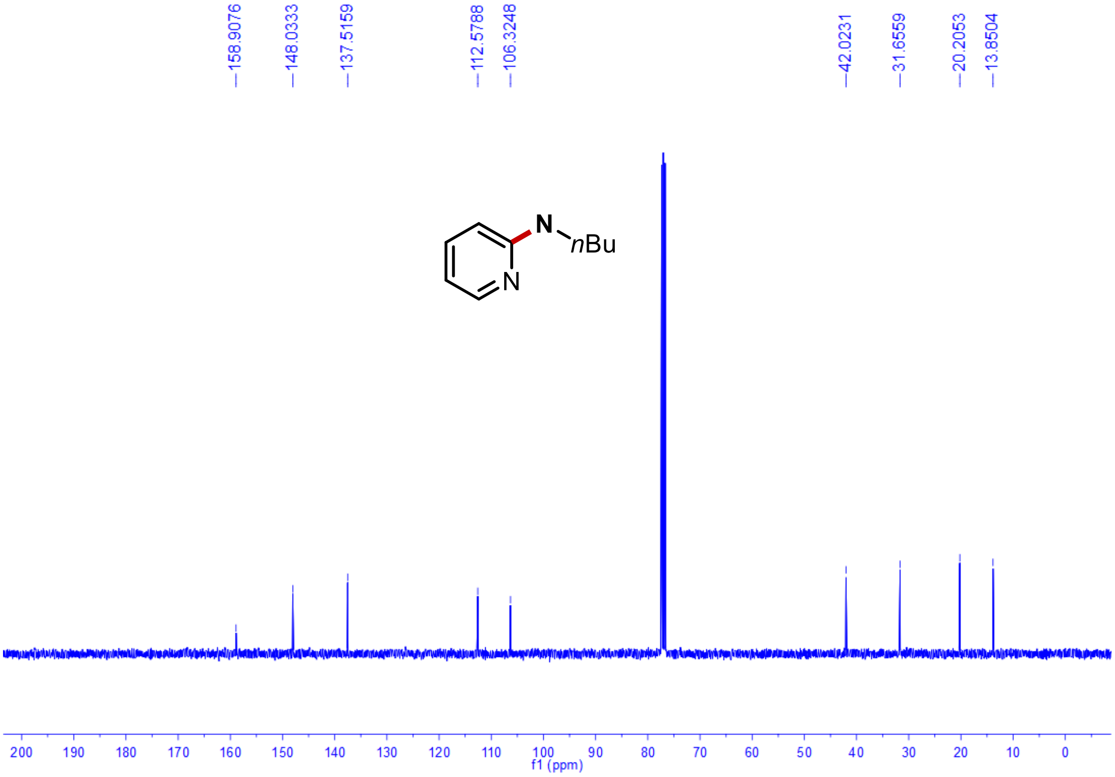

^{13}C NMR (100 MHz, $CDCl_3$) Spectrum

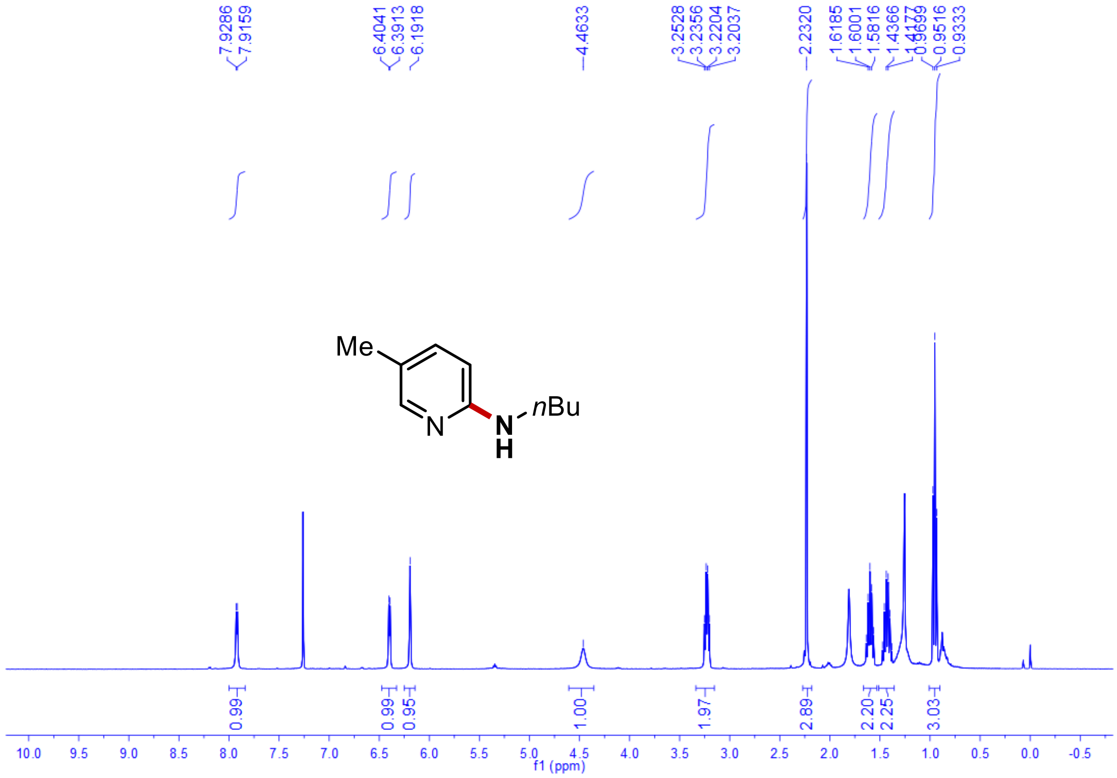

^{1}H NMR (400 MHz, $CDCl_3$) Spectrum

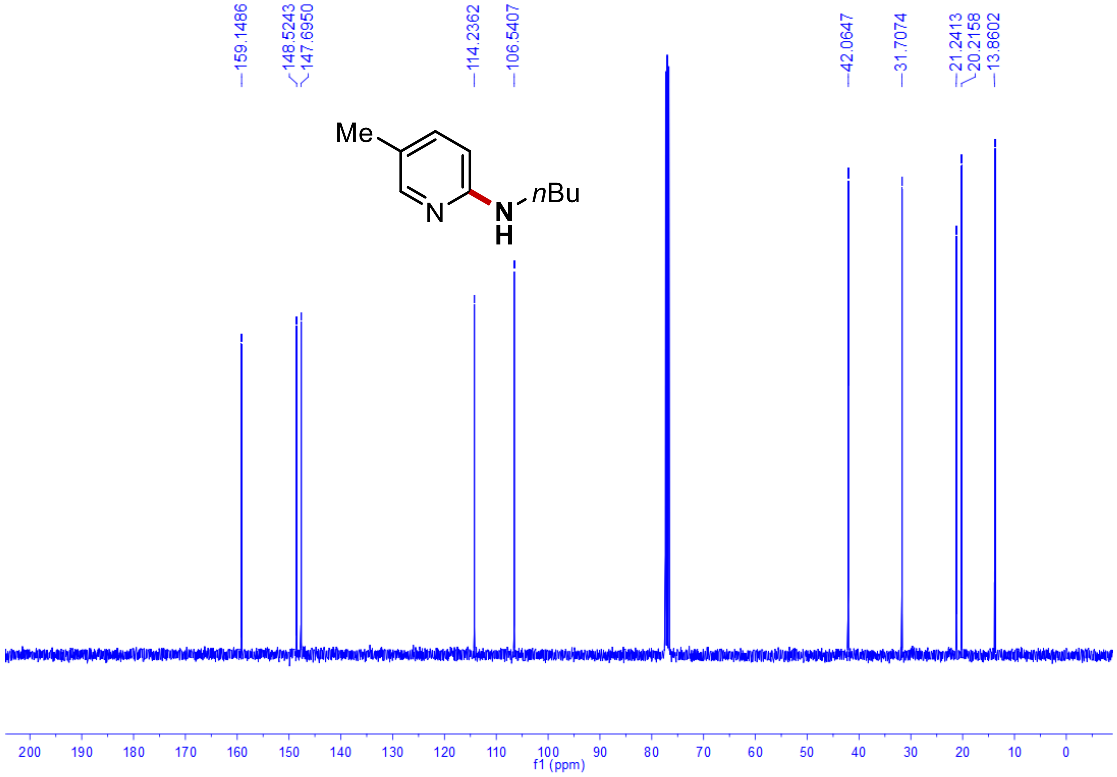

^{13}C NMR (100 MHz, $CDCl_3$) Spectrum

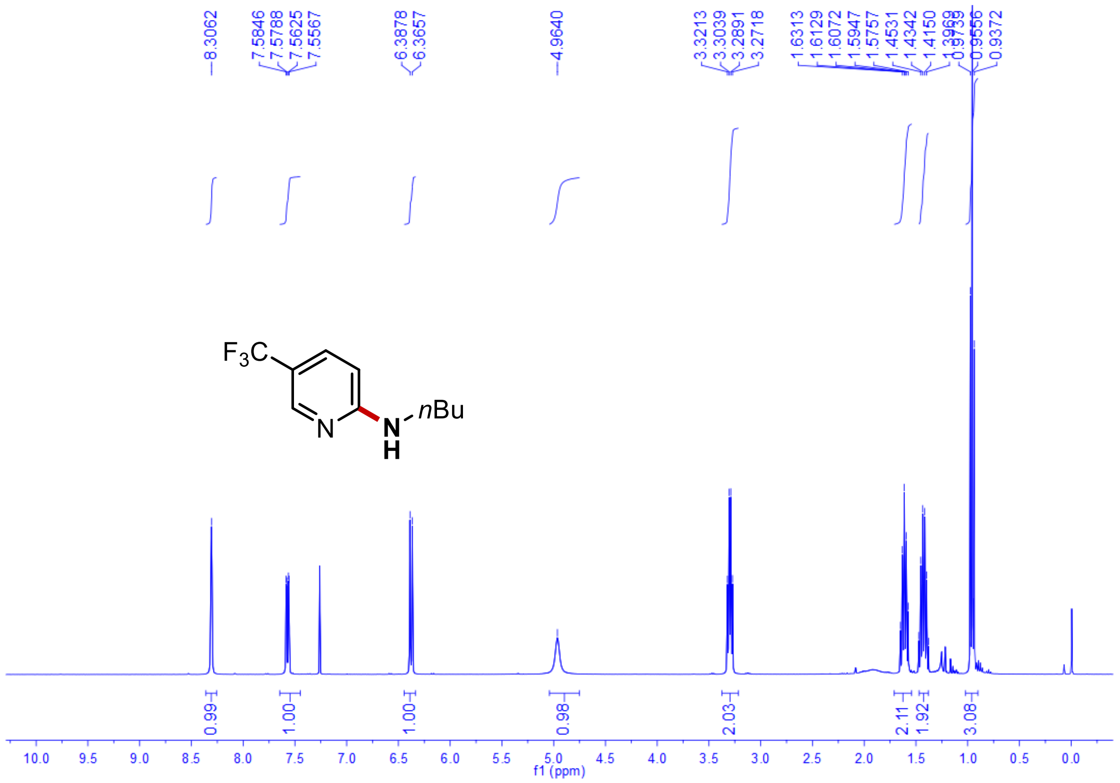

^{1}**H NMR** (400 MHz, $CDCl_3$) Spectrum

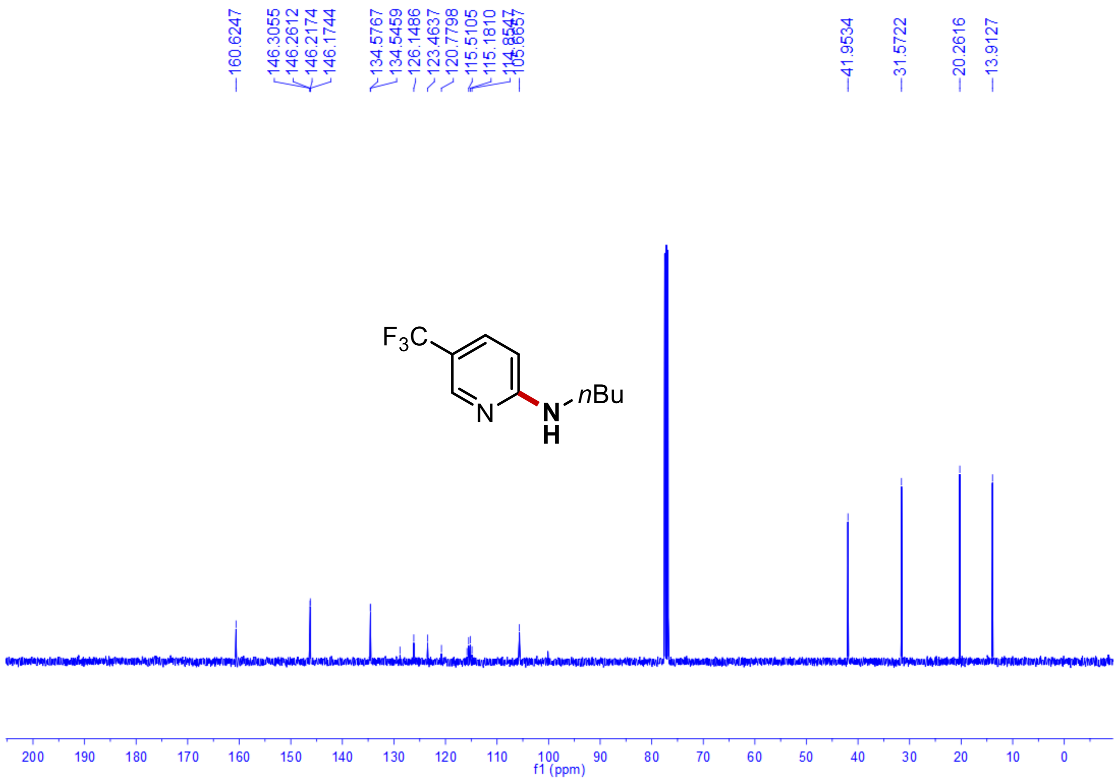

^{13}**C NMR** (100 MHz, $CDCl_3$) Spectrum

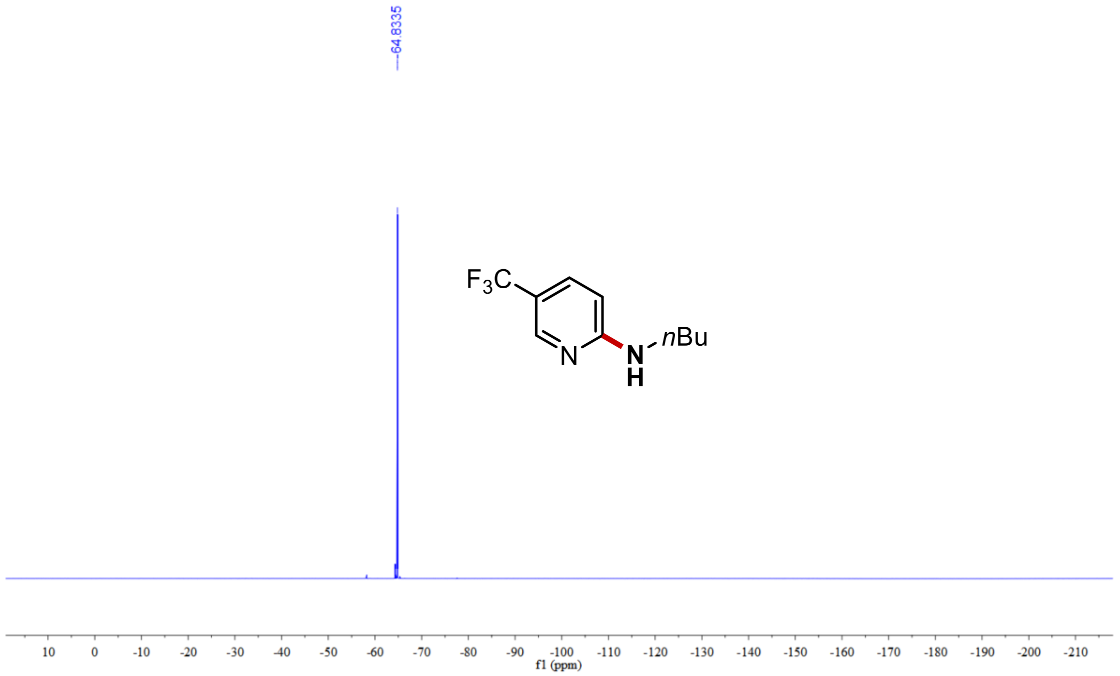

^{19}F NMR (376 MHz, $CDCl_3$) Spectrum

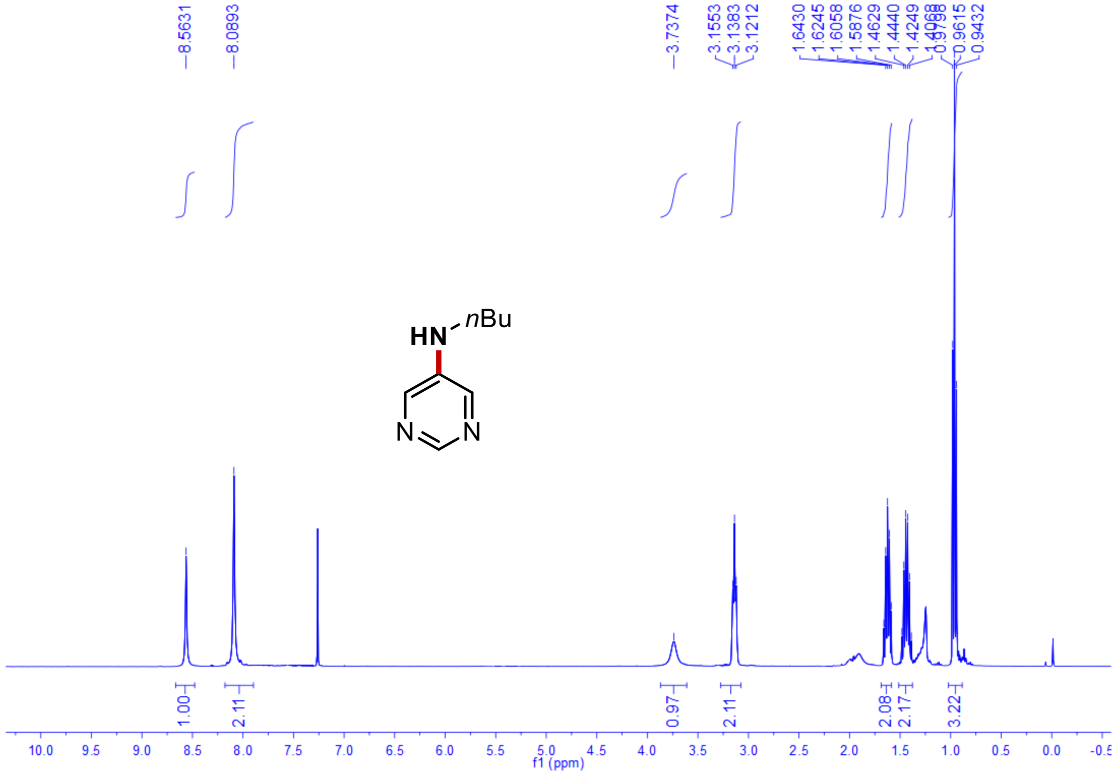

^{1}H NMR (400 MHz, $CDCl_3$) Spectrum

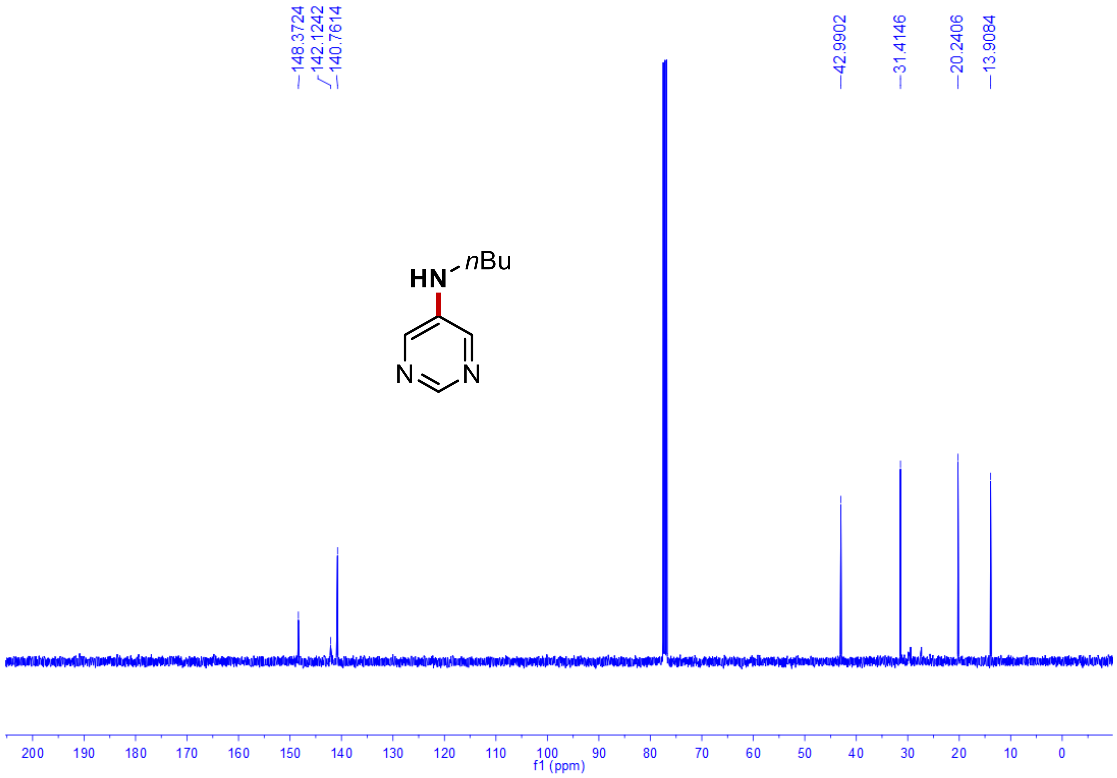

^{13}C NMR (100 MHz, $CDCl_3$) Spectrum

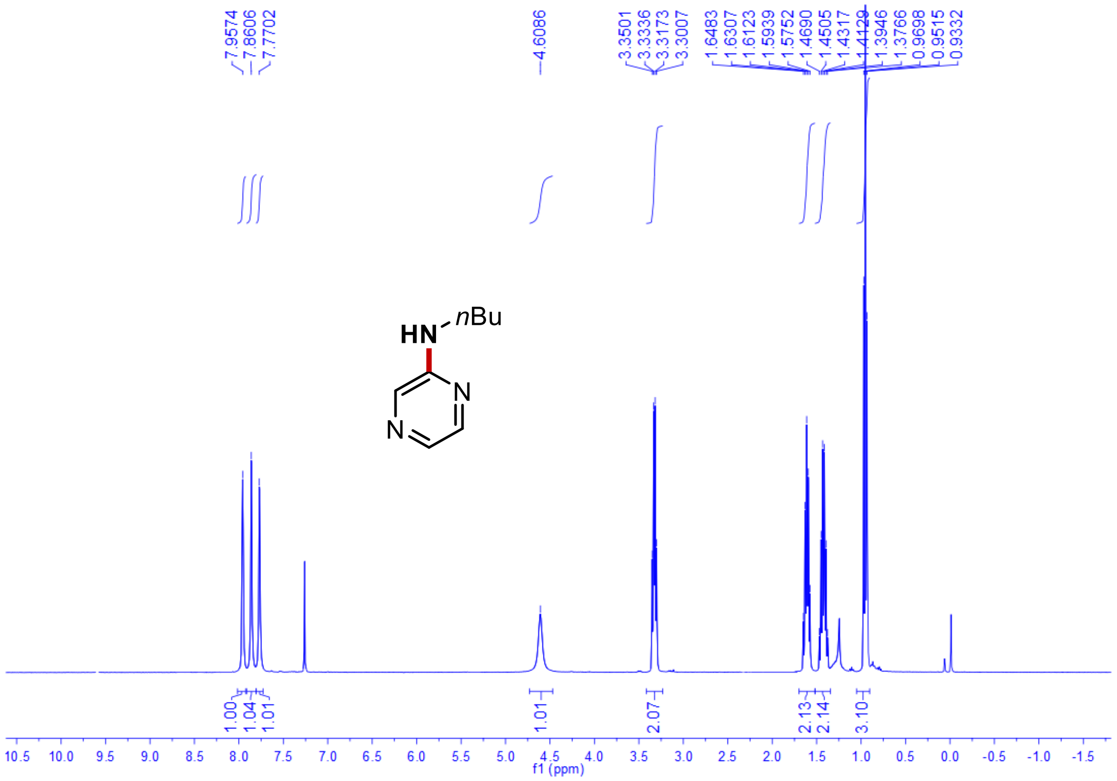

^{1}H NMR (400 MHz, $CDCl_3$) Spectrum

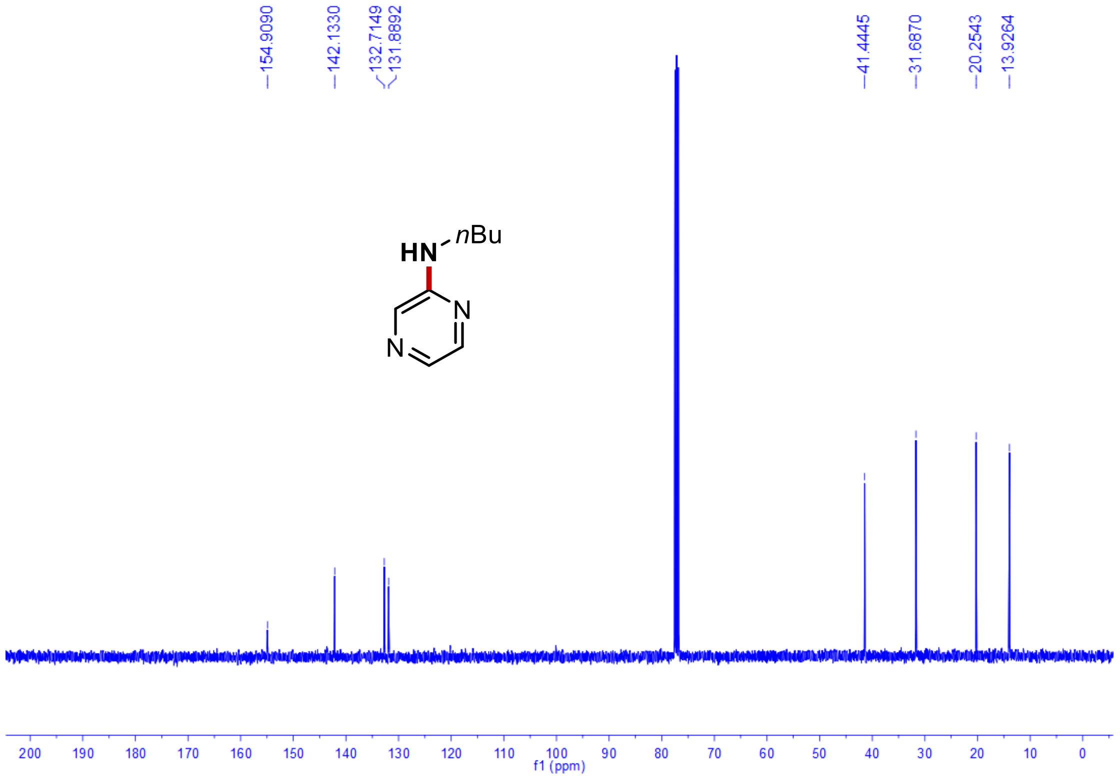

^{13}C NMR (100 MHz, $CDCl_3$) Spectrum

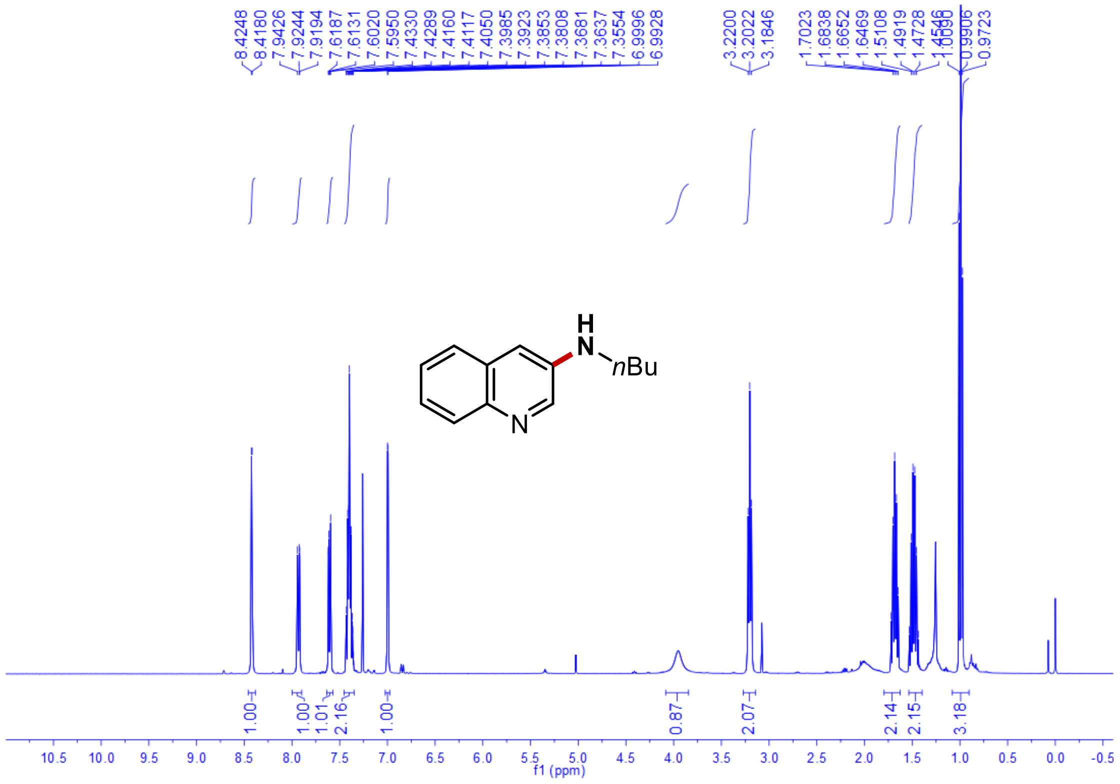

^{1}H NMR (400 MHz, $CDCl_3$) Spectrum

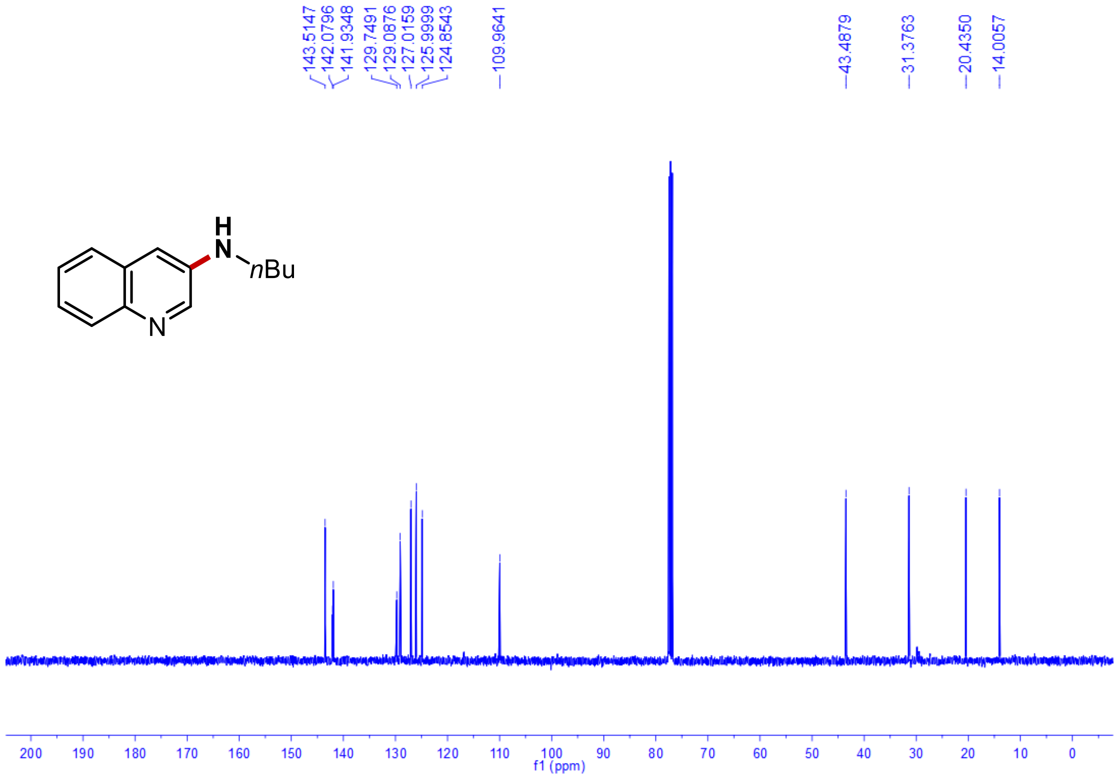

^{13}C NMR (100 MHz, $CDCl_3$) Spectrum

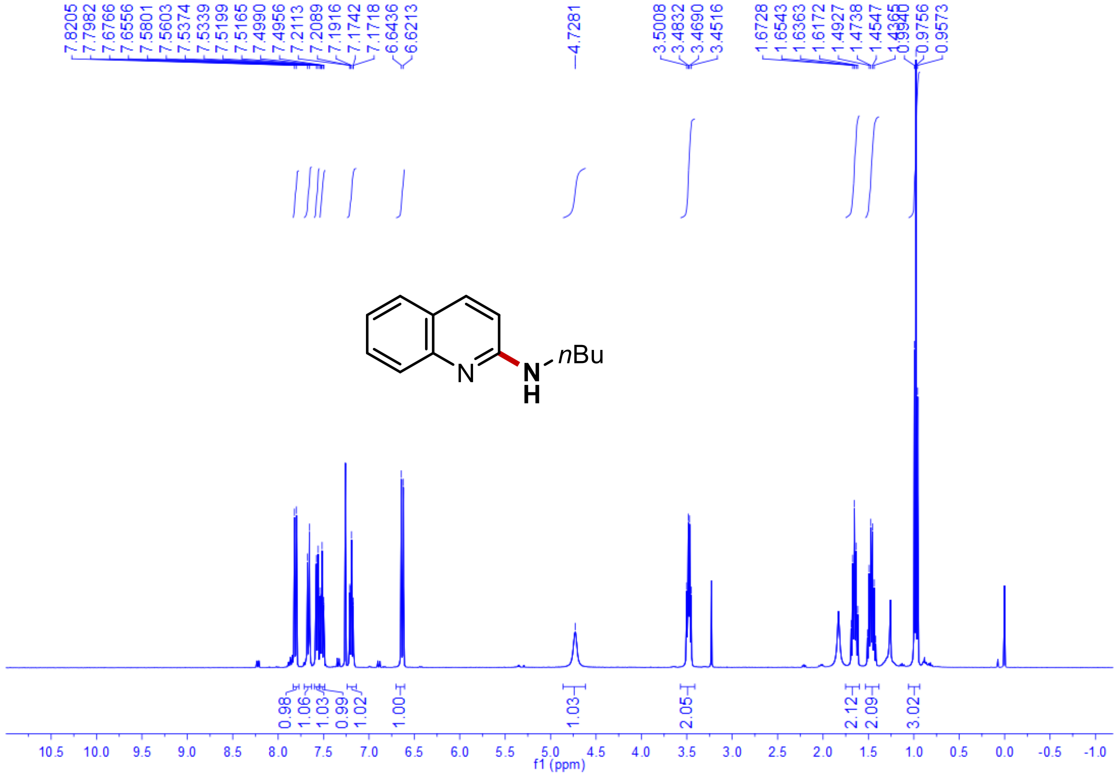

^{1}H NMR (400 MHz, $CDCl_3$) Spectrum

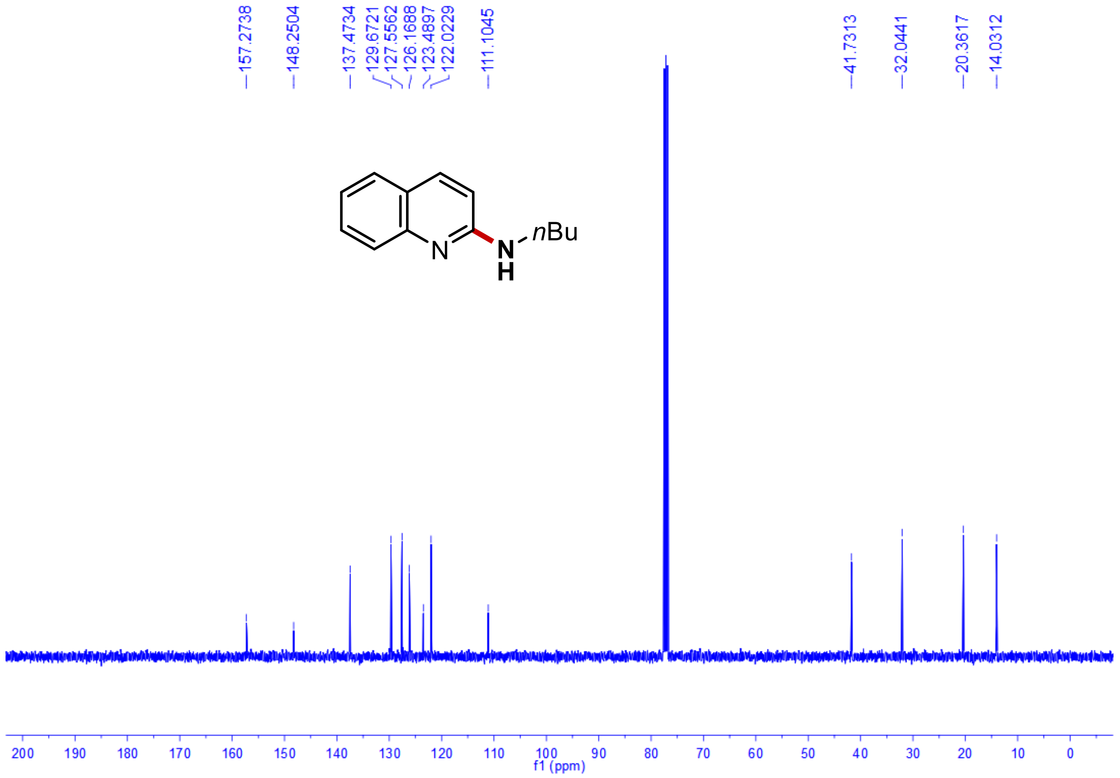

^{13}C NMR (100 MHz, $CDCl_3$) Spectrum

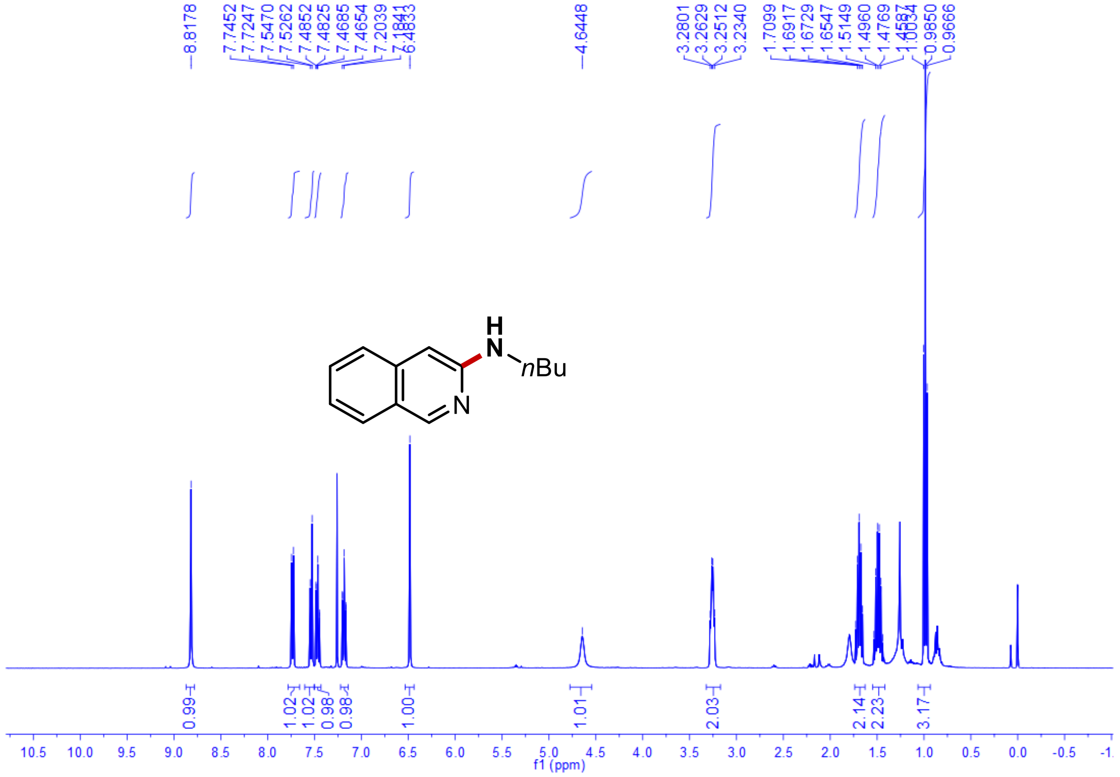

^{1}H NMR (400 MHz, $CDCl_3$) Spectrum

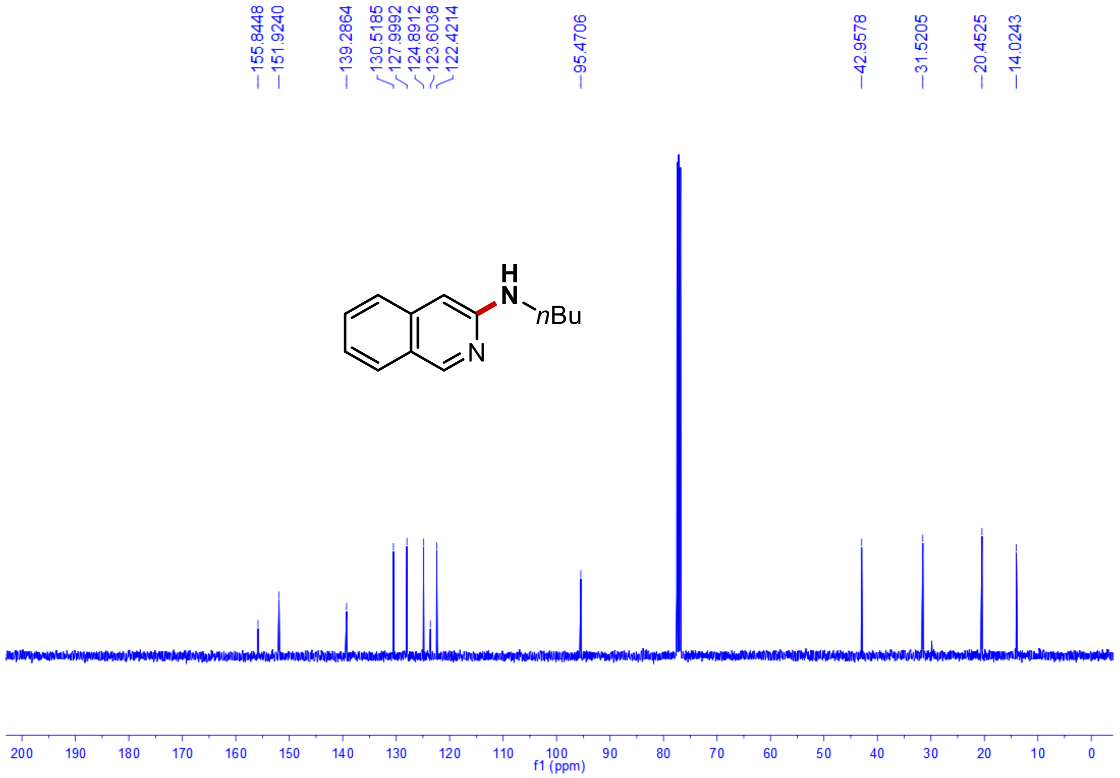

^{13}C NMR (100 MHz, $CDCl_3$) Spectrum

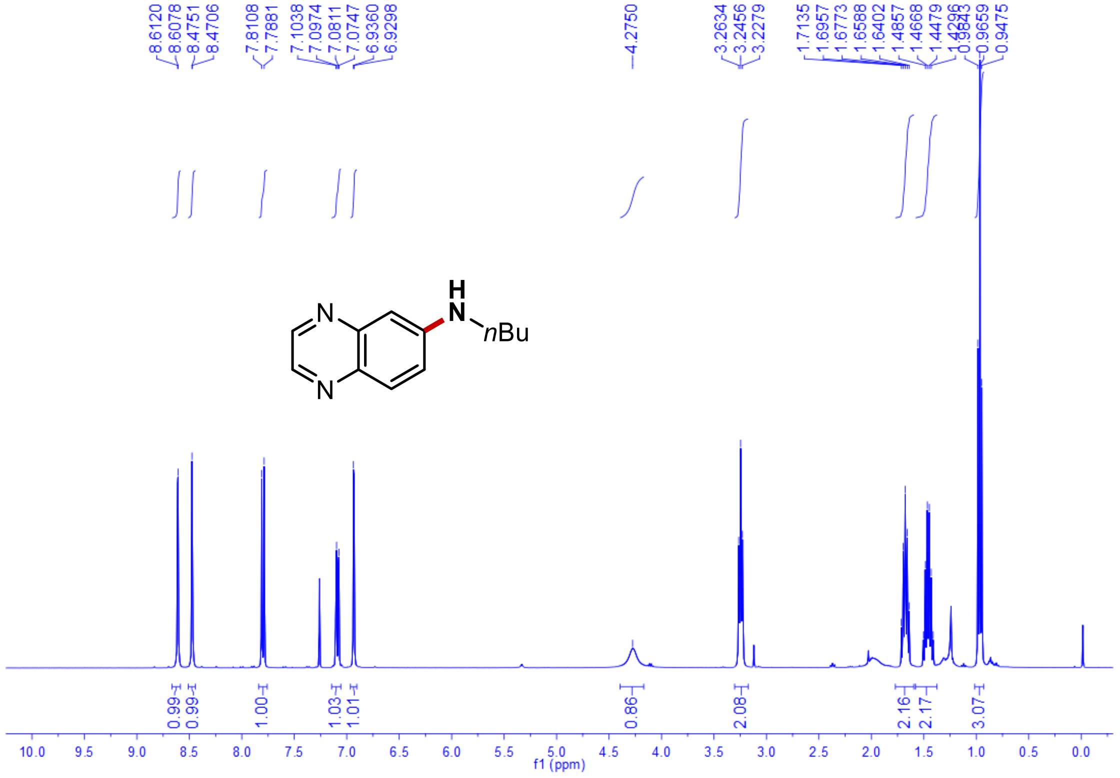

^{1}H NMR (400 MHz, $CDCl_3$) Spectrum

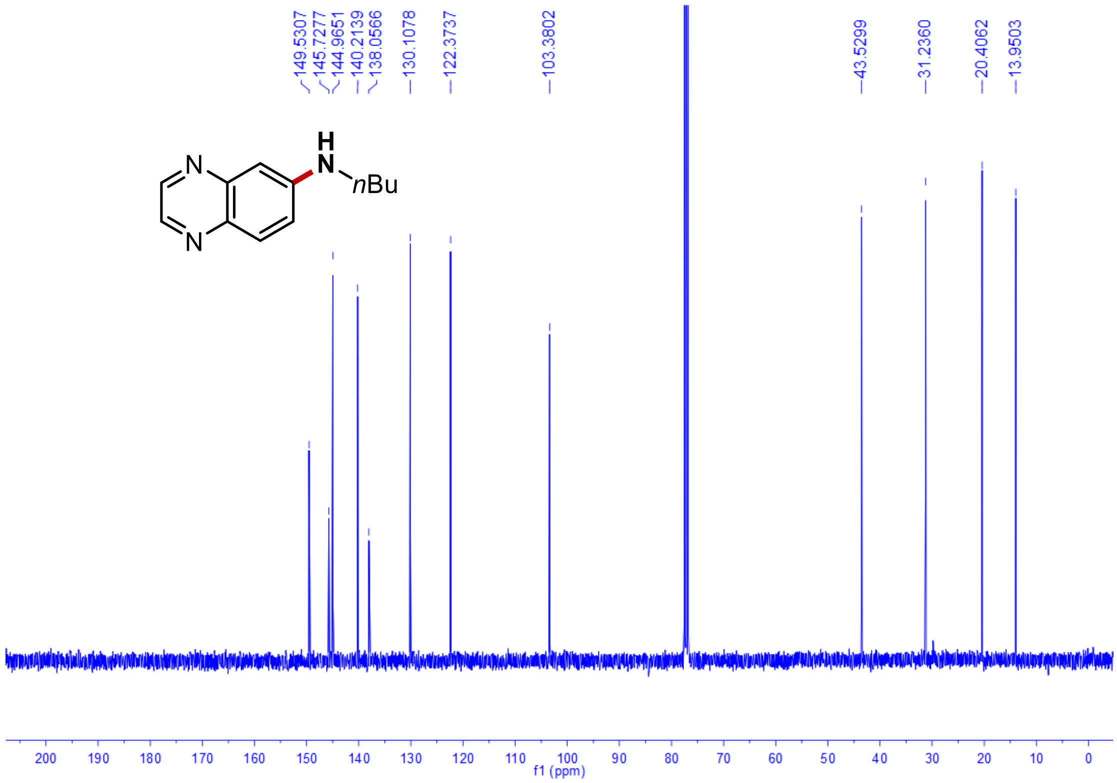

^{13}C NMR (100 MHz, $CDCl_3$) Spectrum

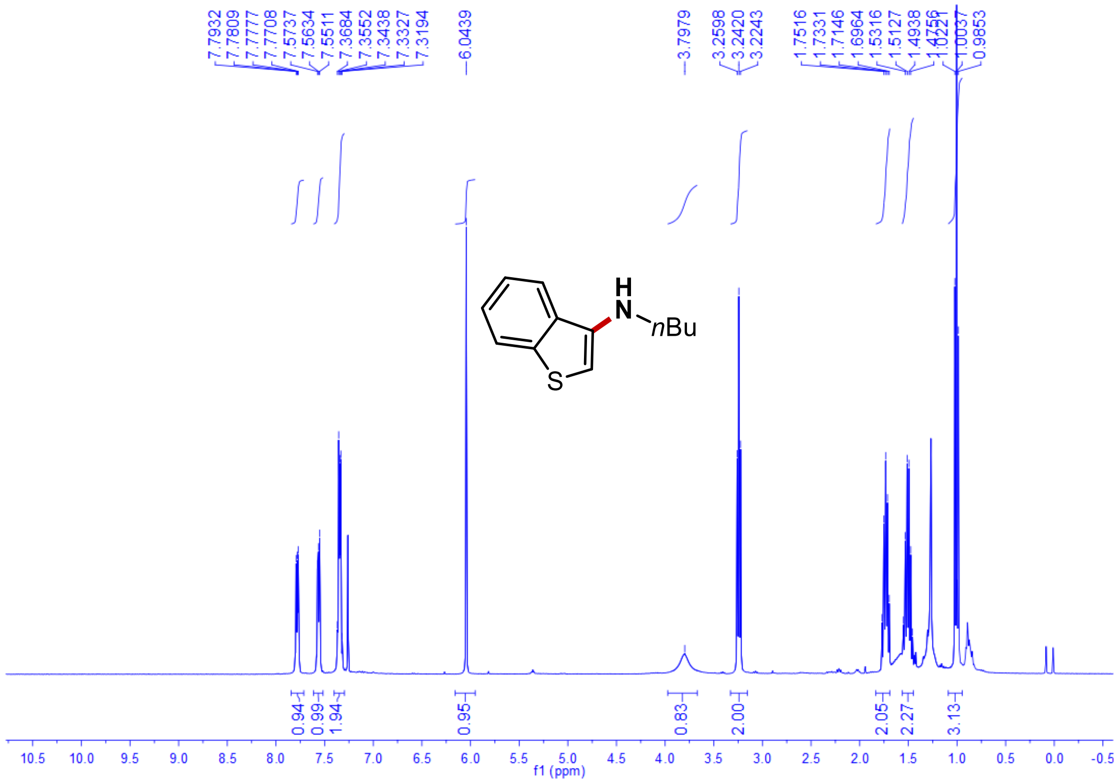

^{1}H NMR (400 MHz, $CDCl_3$) Spectrum

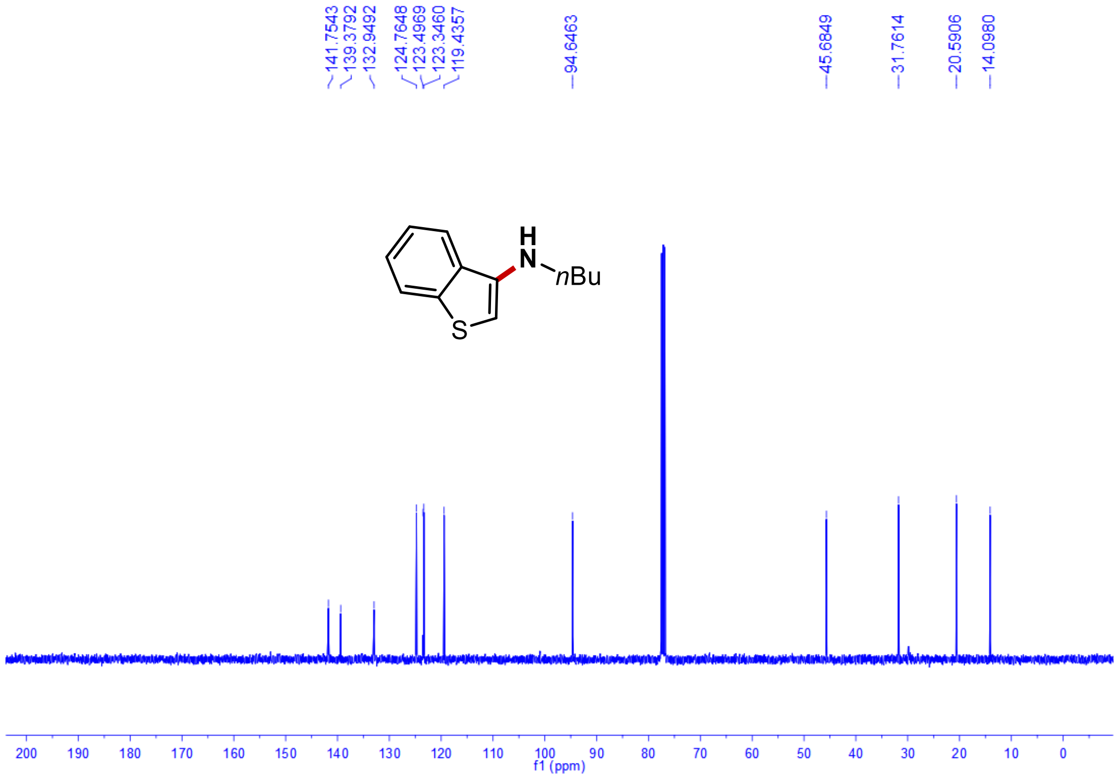

^{13}C NMR (100 MHz, $CDCl_3$) Spectrum

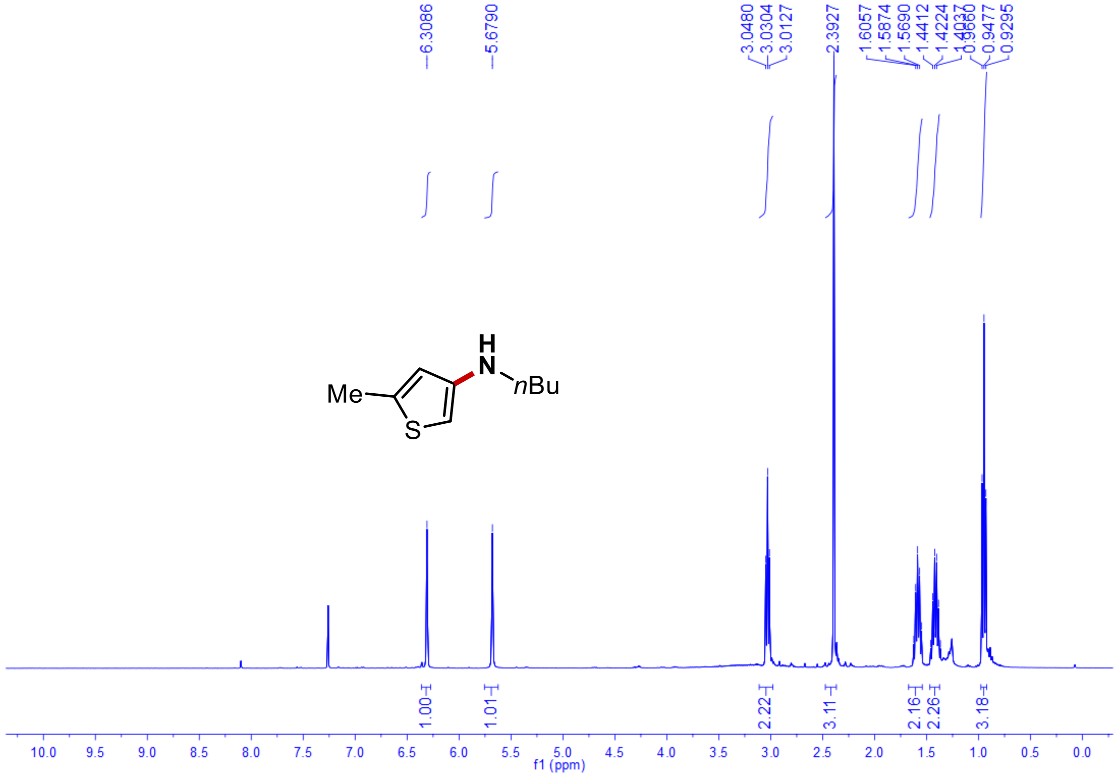

^{1}H NMR (400 MHz, $CDCl_3$) Spectrum

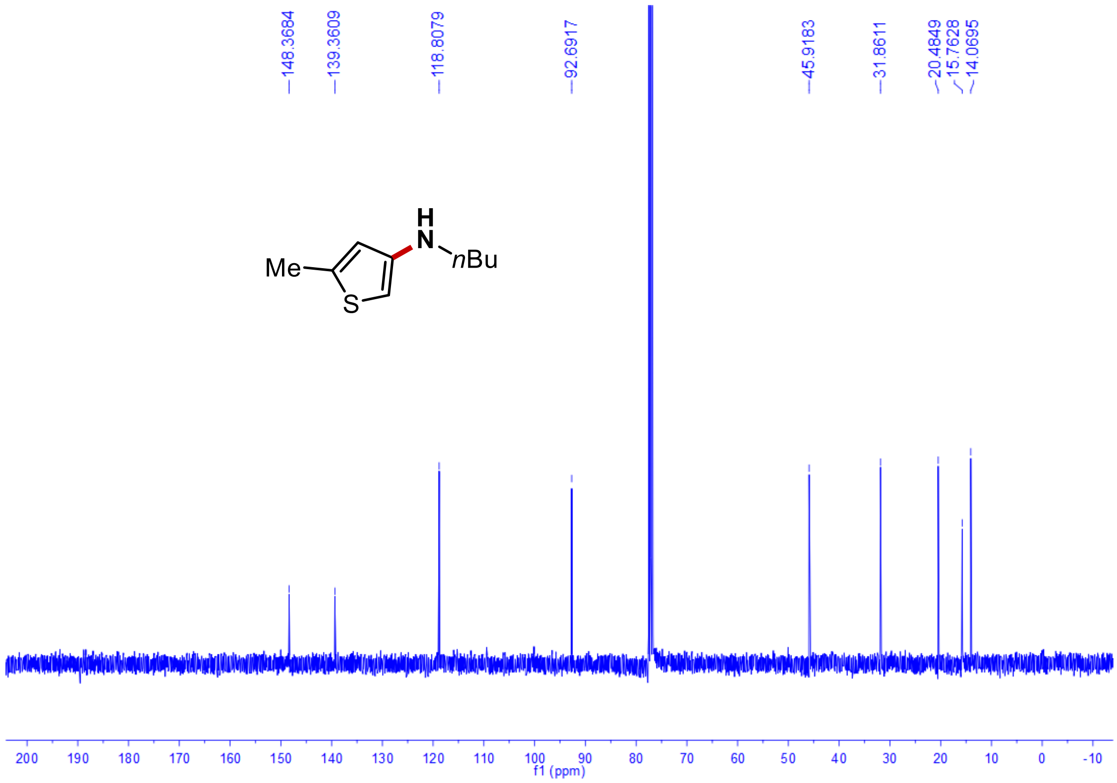

^{13}C NMR (100 MHz, $CDCl_3$) Spectrum

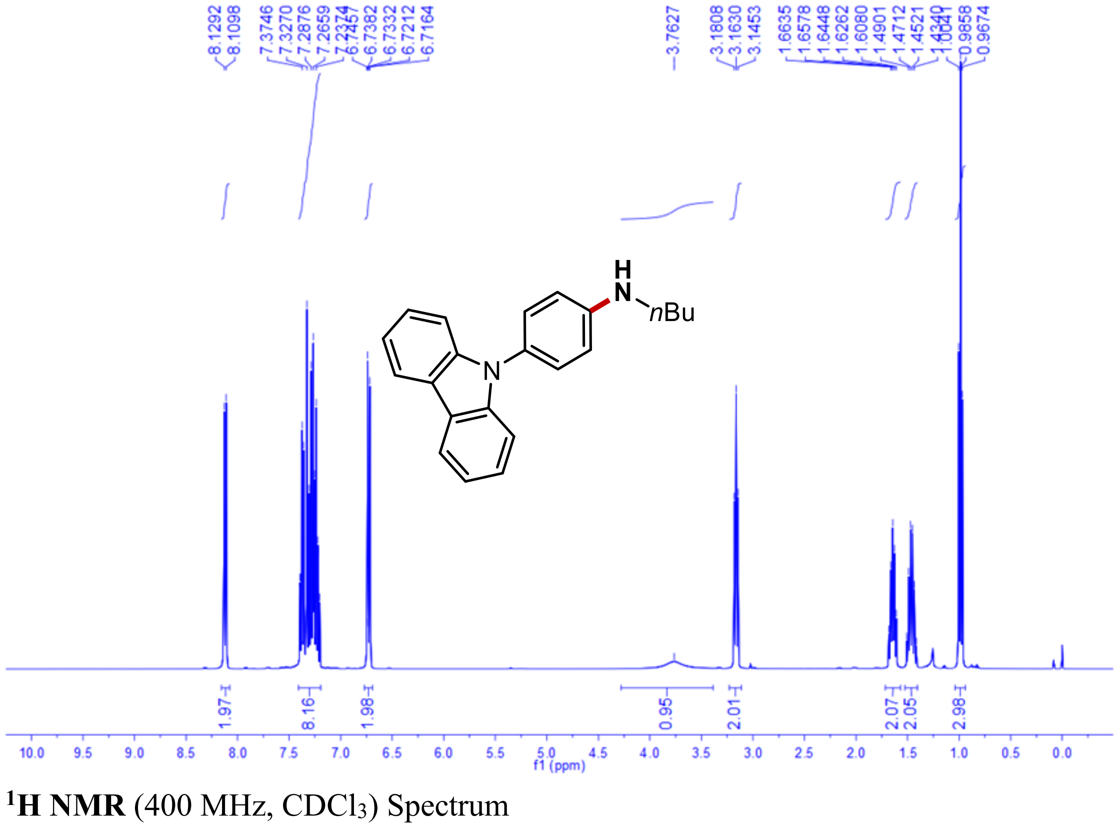

^{1}H NMR (400 MHz, $CDCl_3$) Spectrum

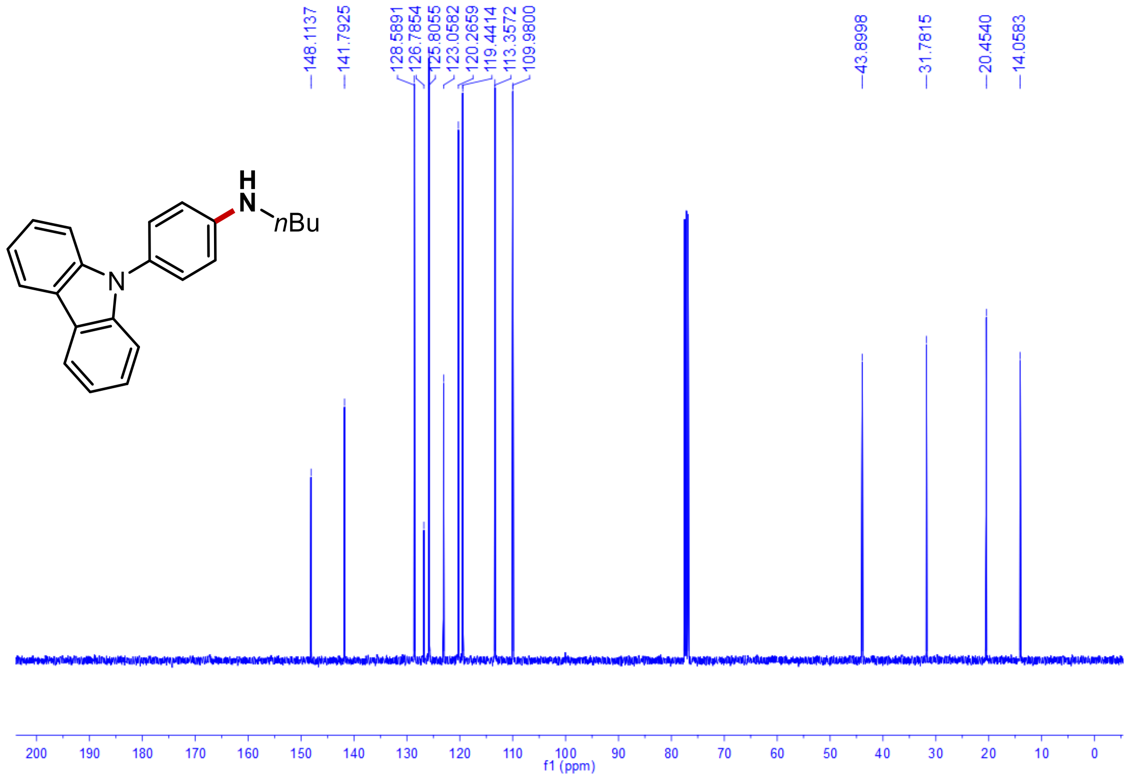

^{13}**C NMR** (100 MHz, $CDCl_3$) Spectrum

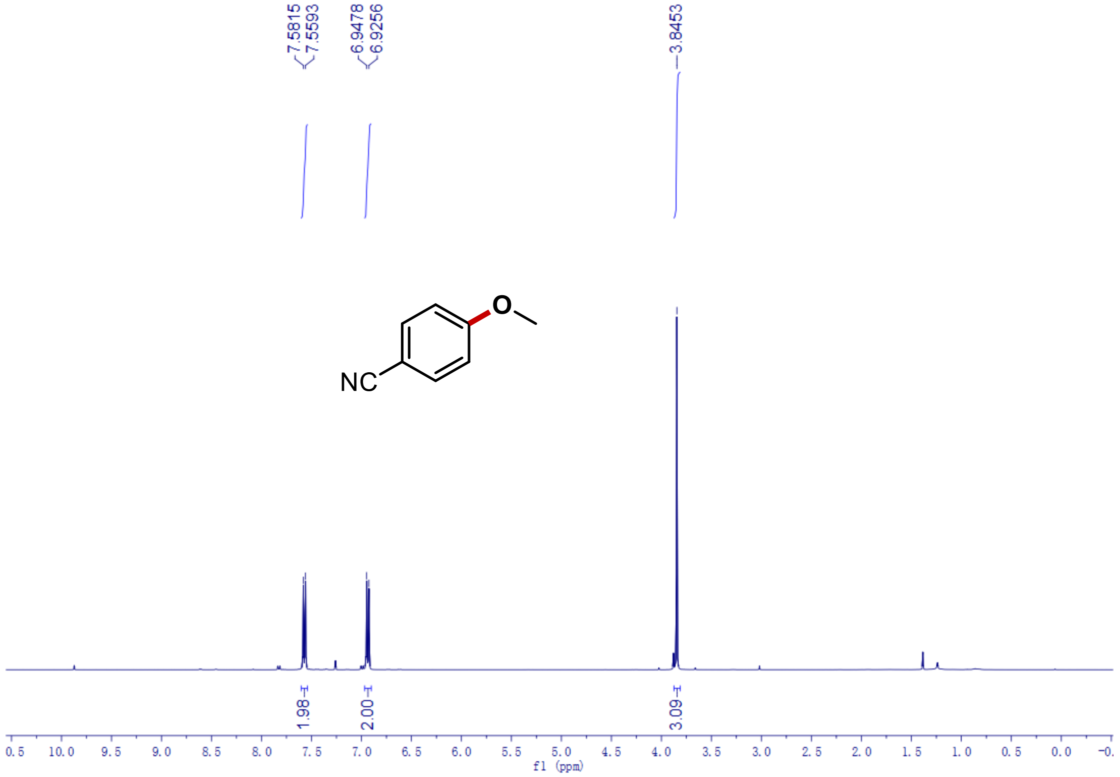

^{1}**H NMR** (400 MHz, $CDCl_3$) Spectrum

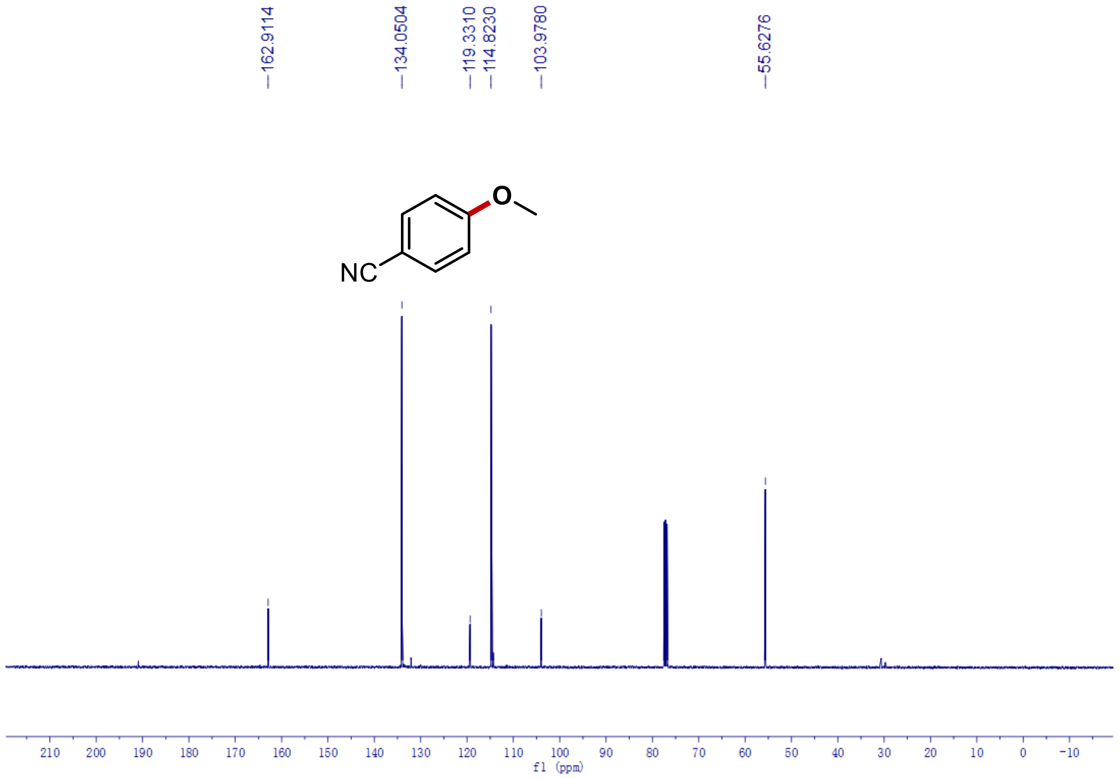

^{13}C NMR (100 MHz, $CDCl_3$) Spectrum

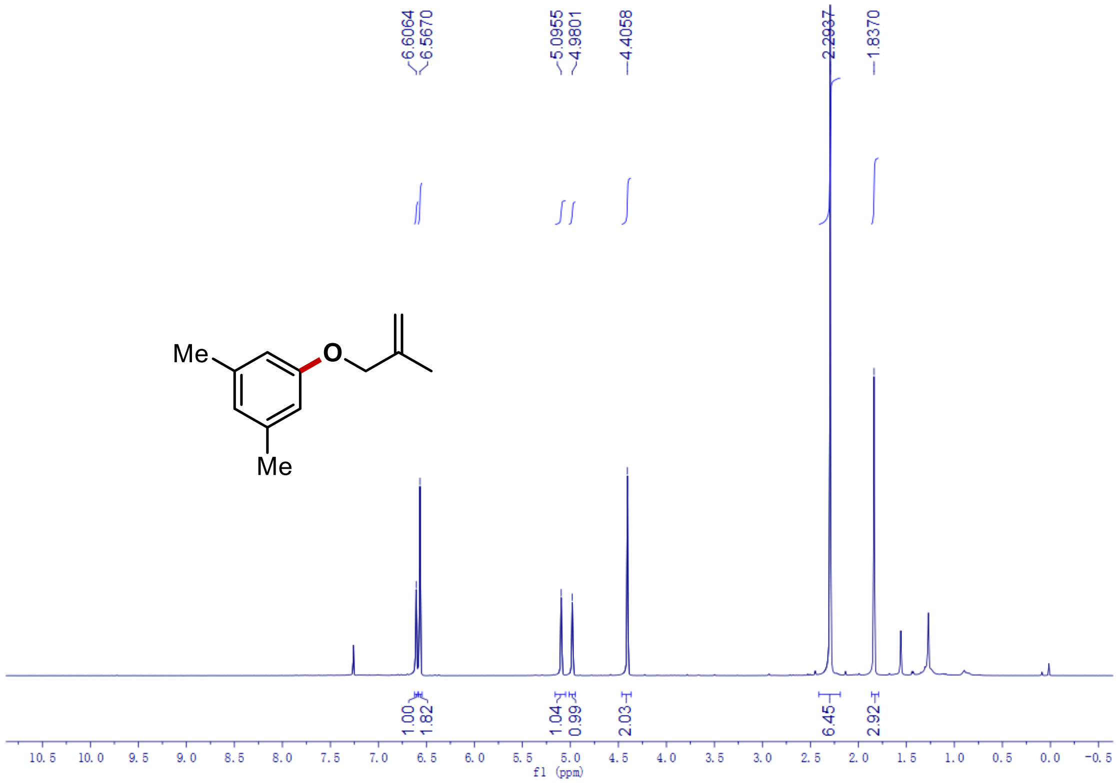

^{1}H NMR (400 MHz, $CDCl_3$) Spectrum

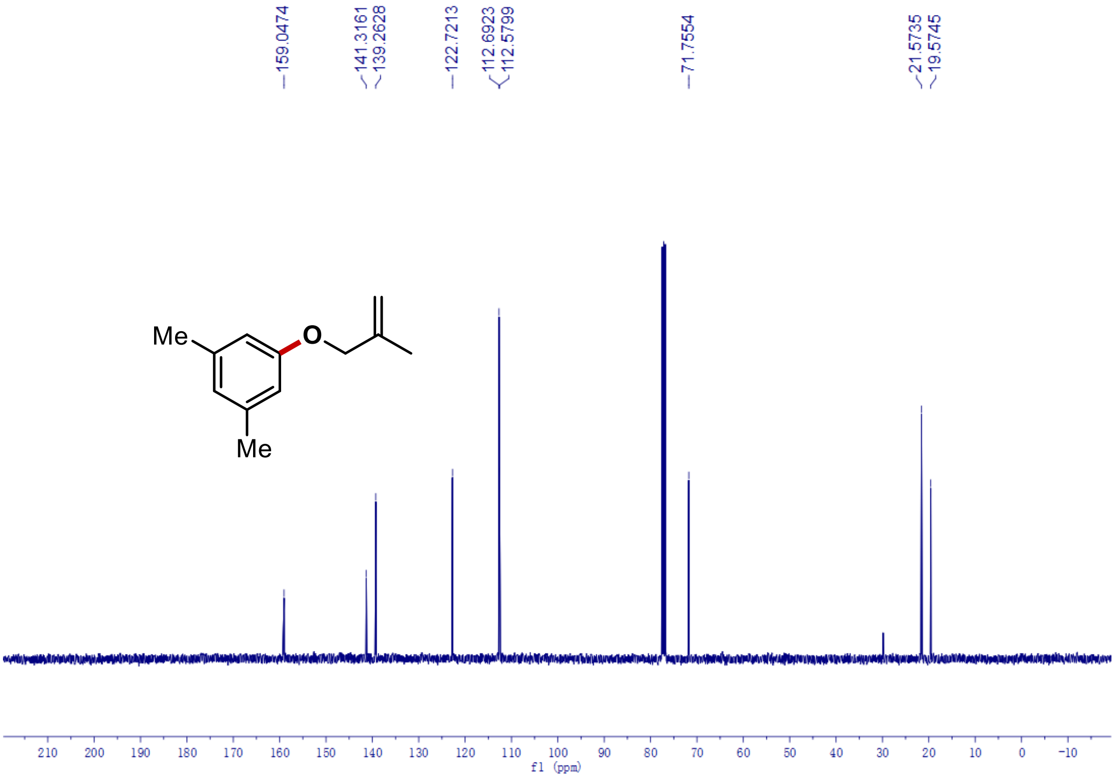

^{13}**C NMR** (100 MHz, $CDCl_3$) Spectrum

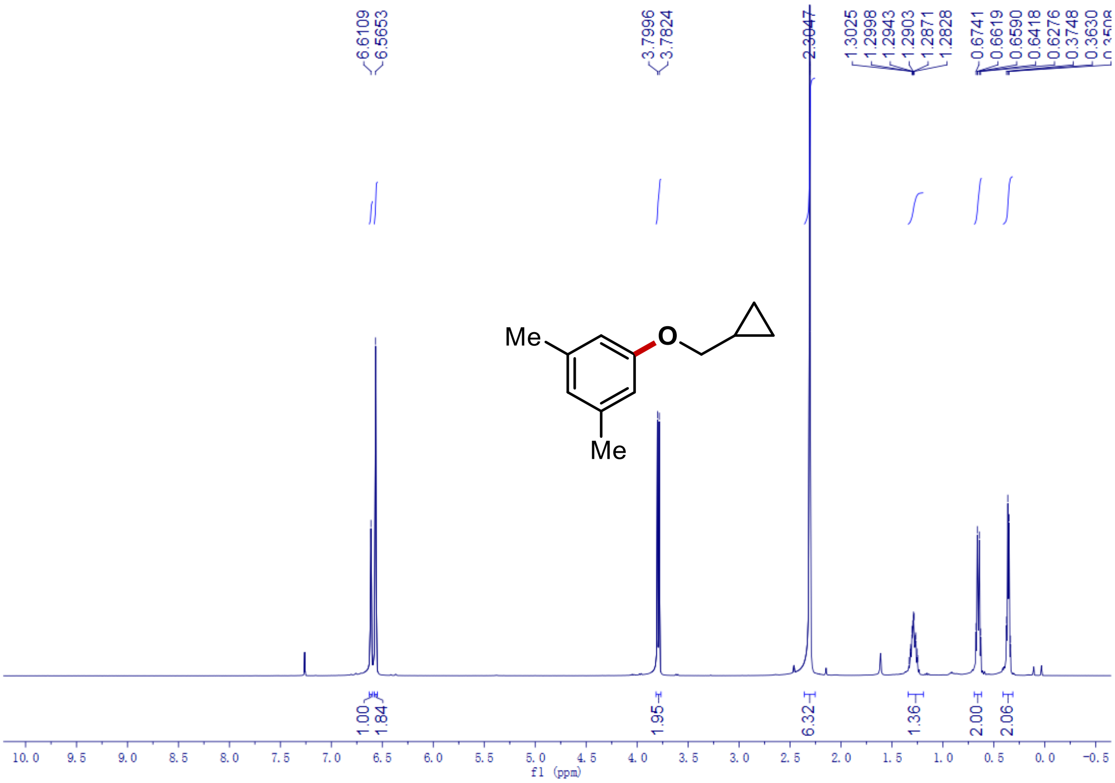

^{1}**H NMR** (400 MHz, $CDCl_3$) Spectrum

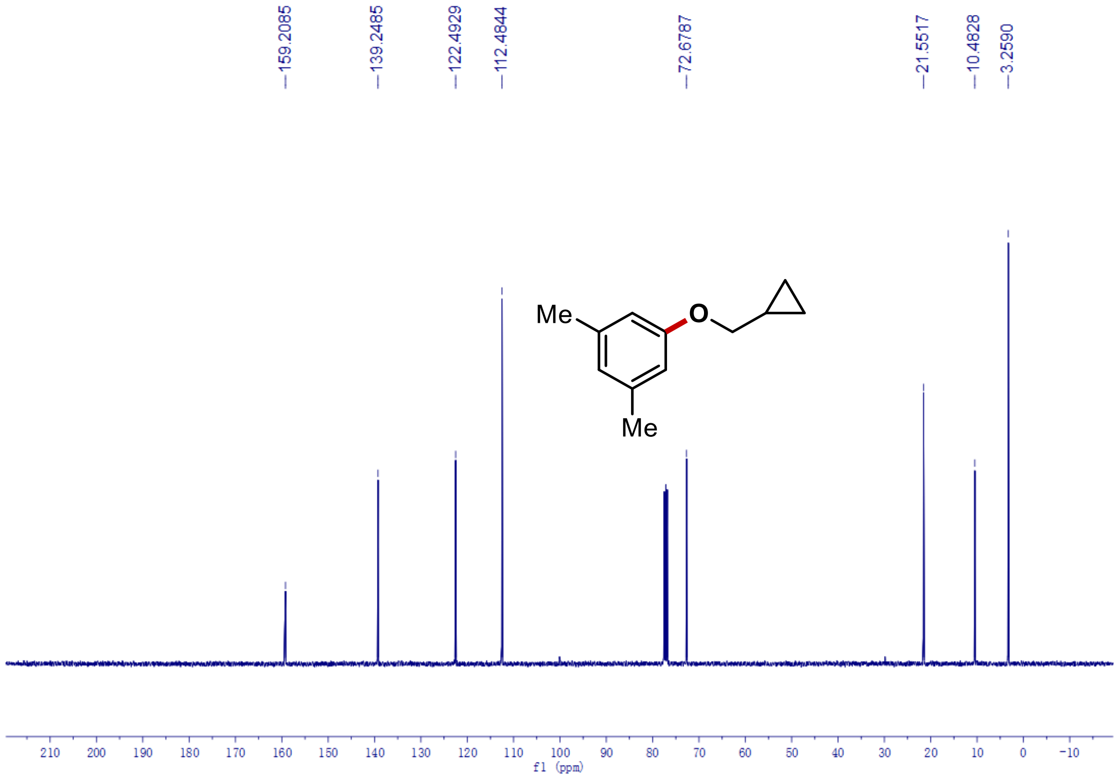

^{13}**C NMR** (100 MHz, $CDCl_3$) Spectrum

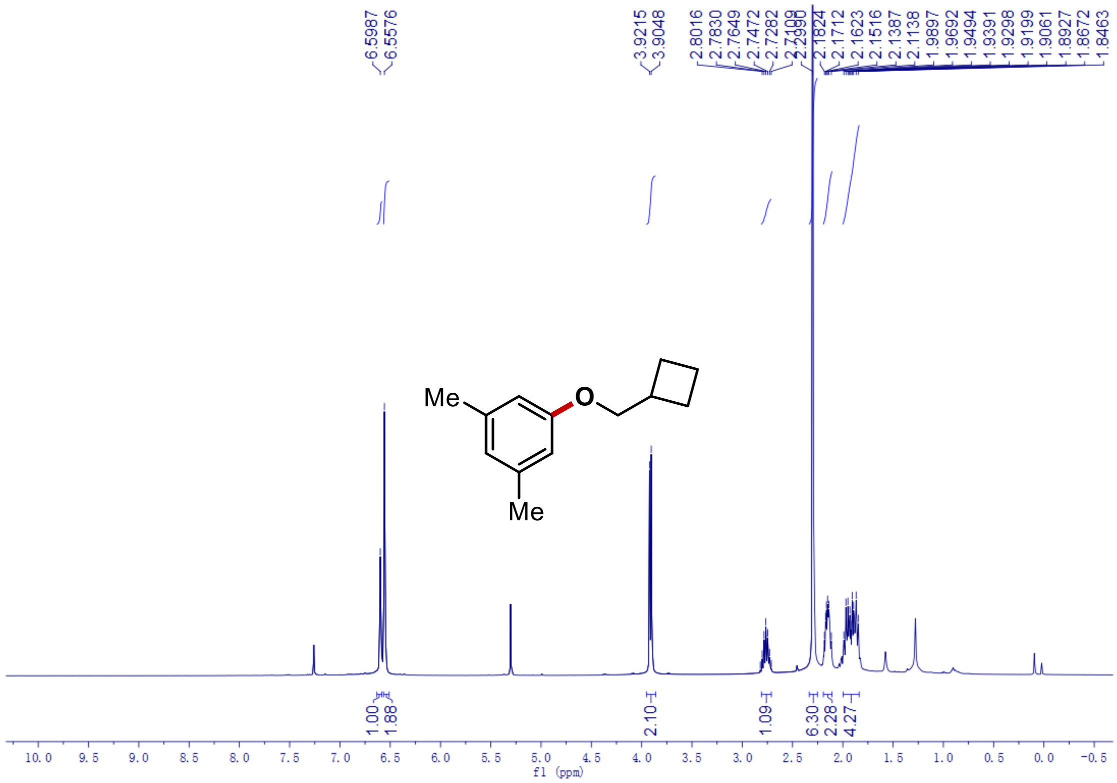

^{1}**H NMR** (400 MHz, $CDCl_3$) Spectrum

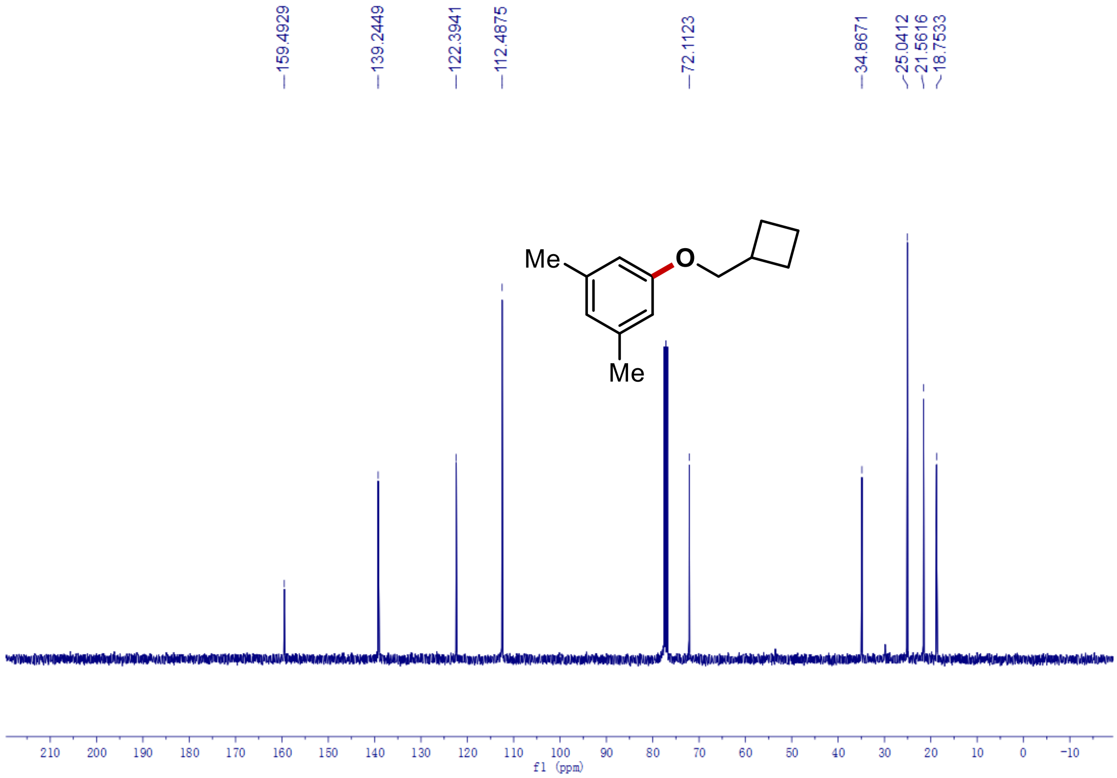

^{13}C NMR (100 MHz, $CDCl_3$) Spectrum

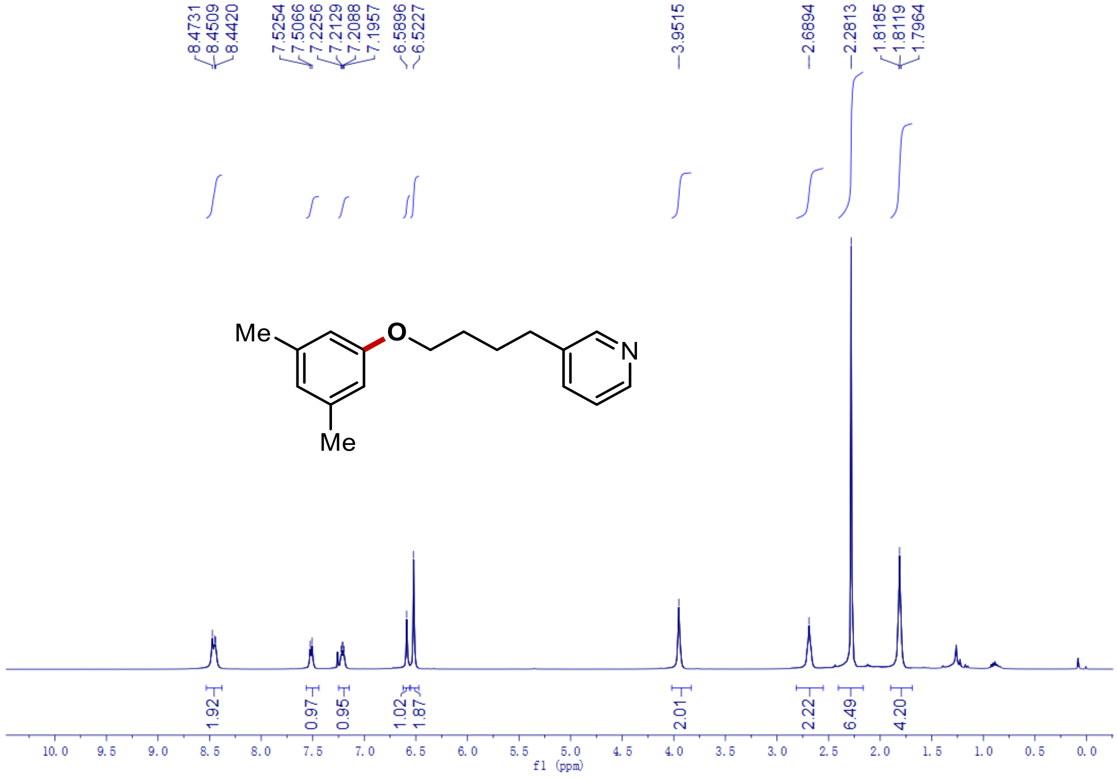

^{1}H NMR (400 MHz, $CDCl_3$) Spectrum

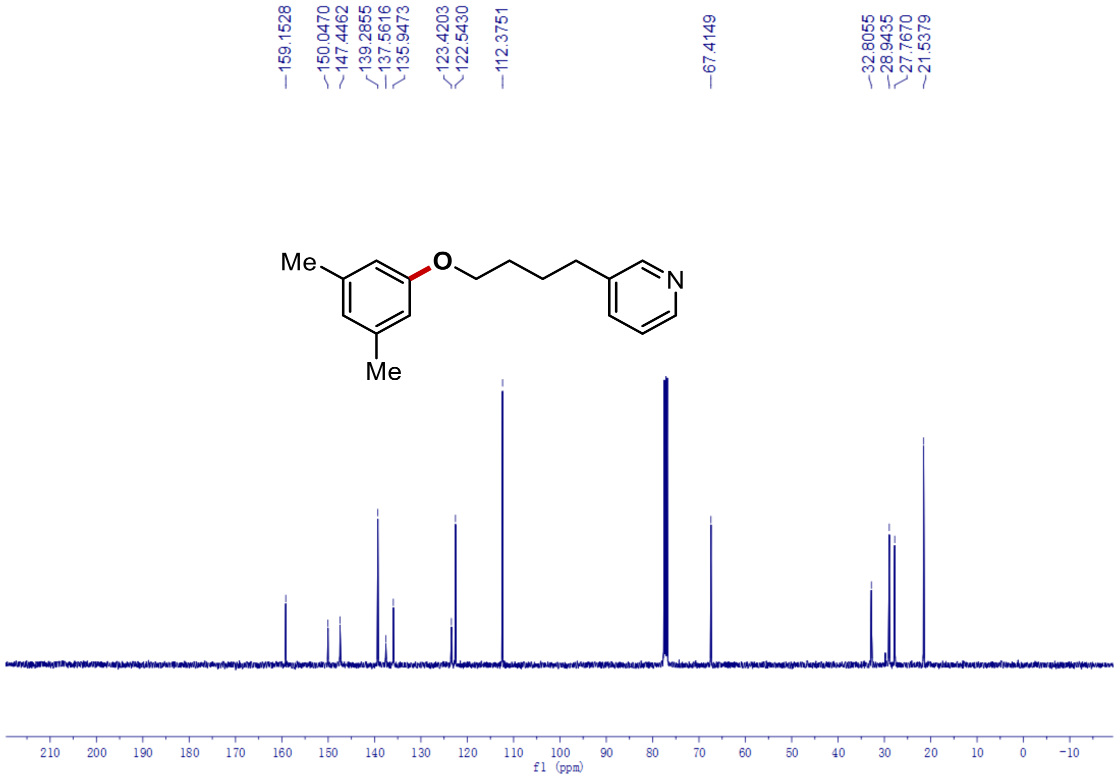

^{13}C NMR (100 MHz, $CDCl_3$) Spectrum

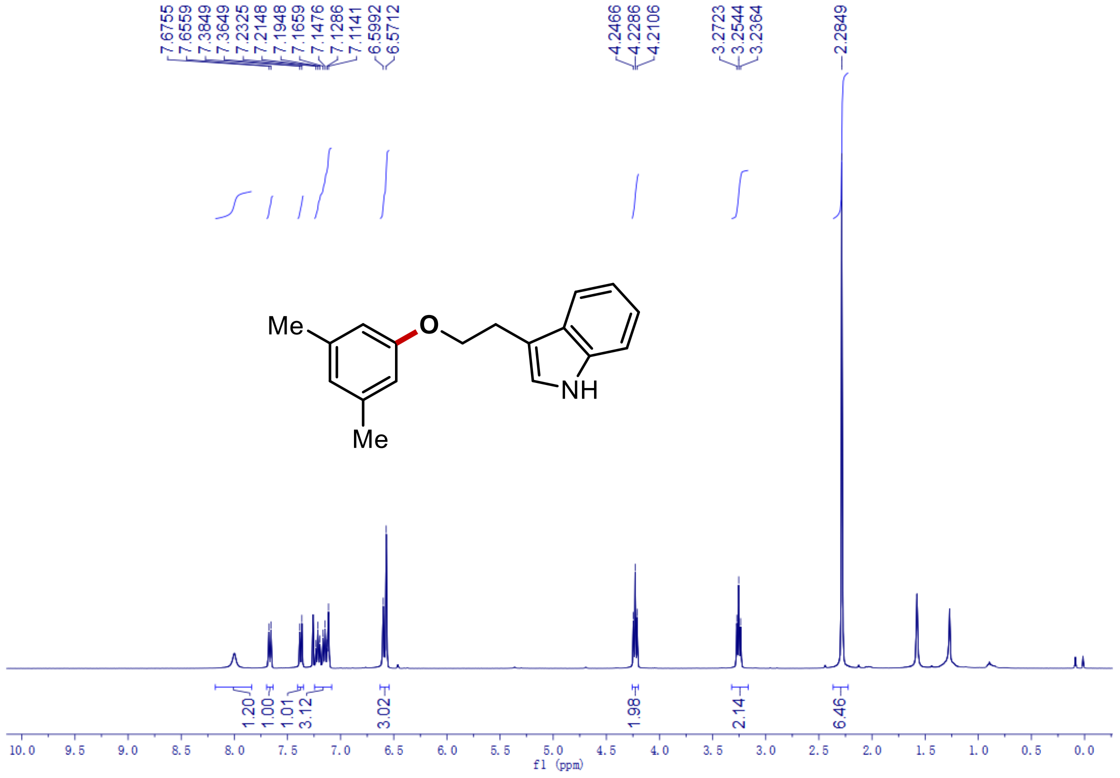

^{1}H NMR (400 MHz, $CDCl_3$) Spectrum

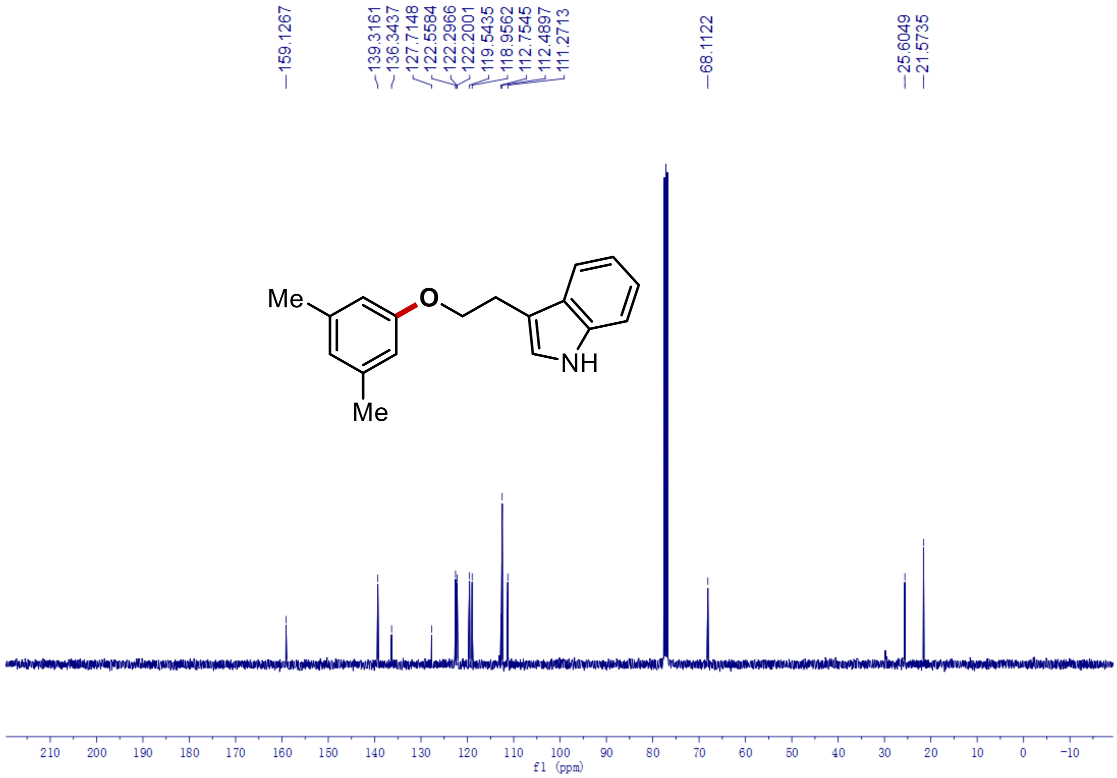

^{13}C NMR (100 MHz, $CDCl_3$) Spectrum

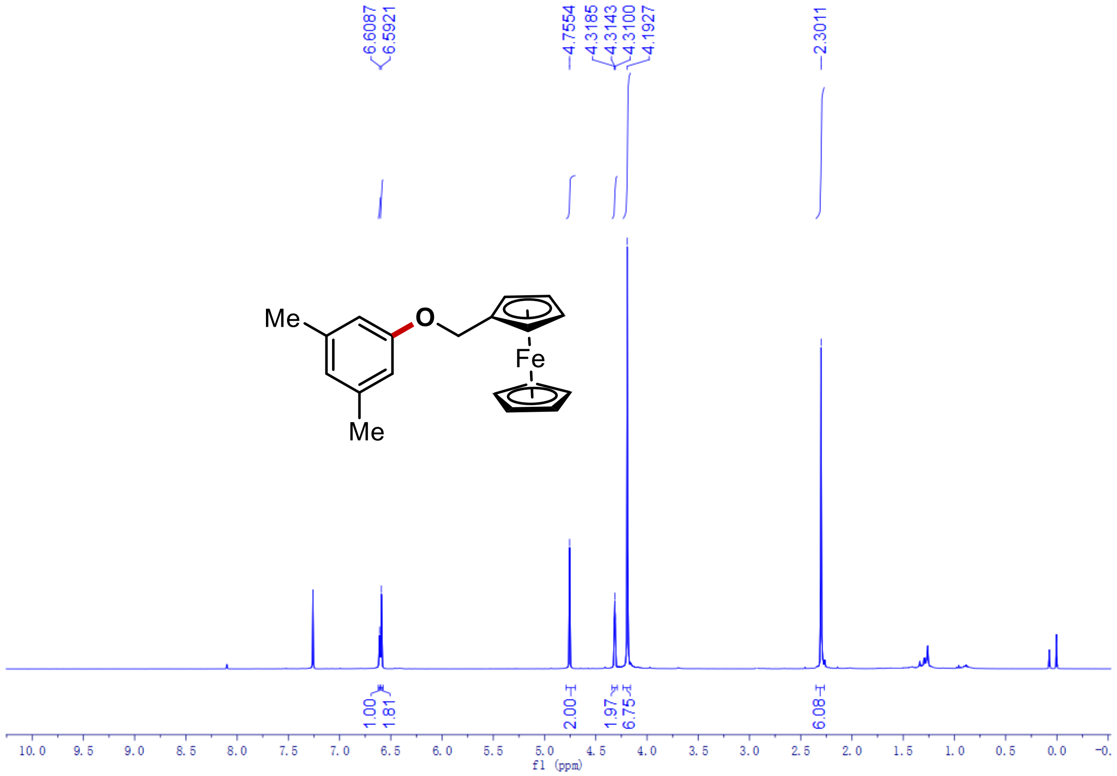

^{1}H NMR (400 MHz, $CDCl_3$) Spectrum

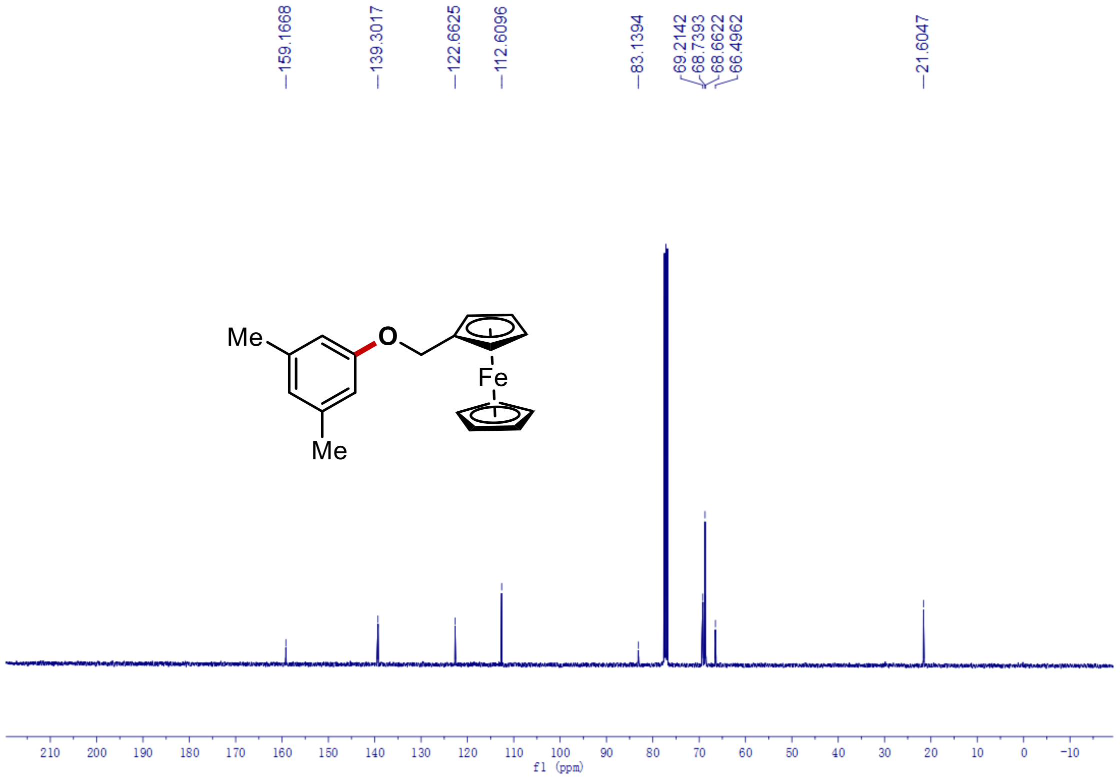

^{13}C NMR (100 MHz, $CDCl_3$) Spectrum

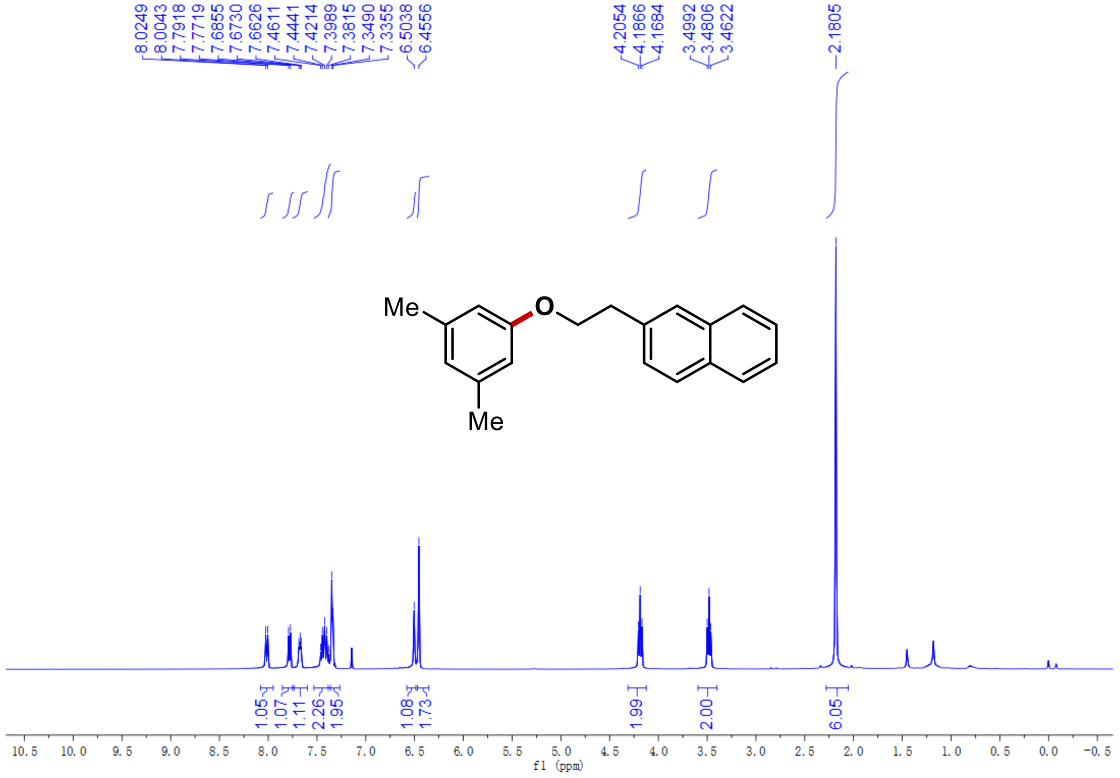

^{1}H NMR (400 MHz, $CDCl_3$) Spectrum

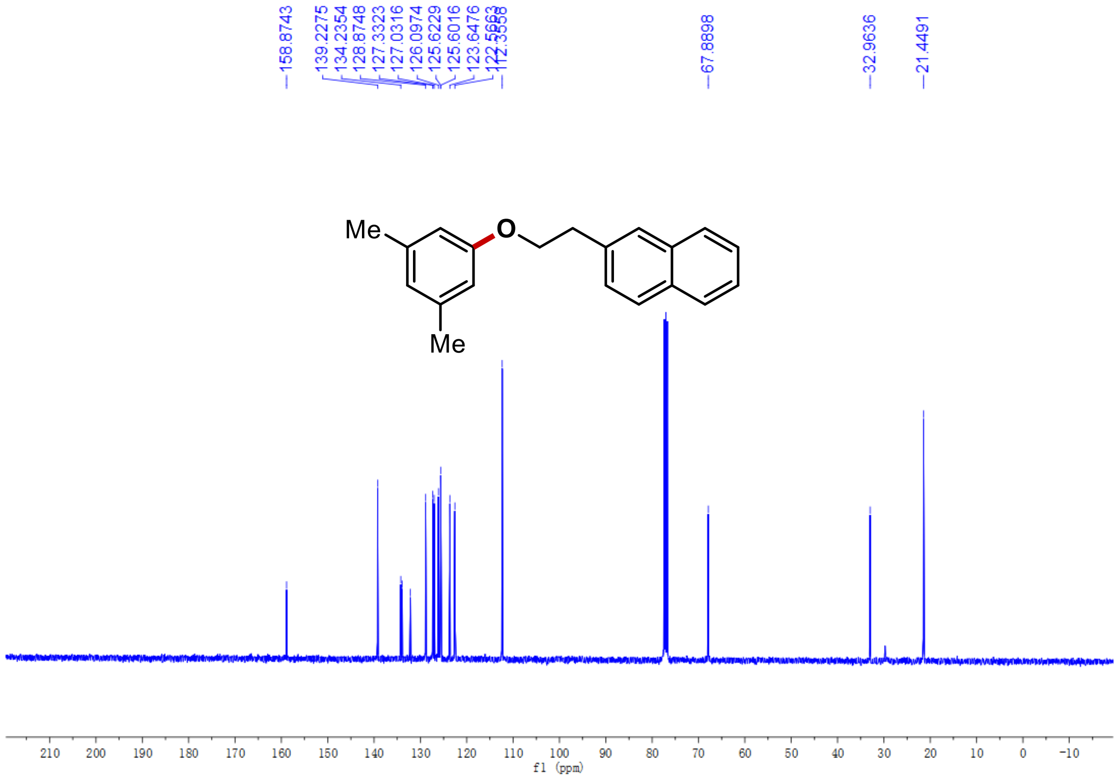

^{13}C NMR (100 MHz, $CDCl_3$) Spectrum

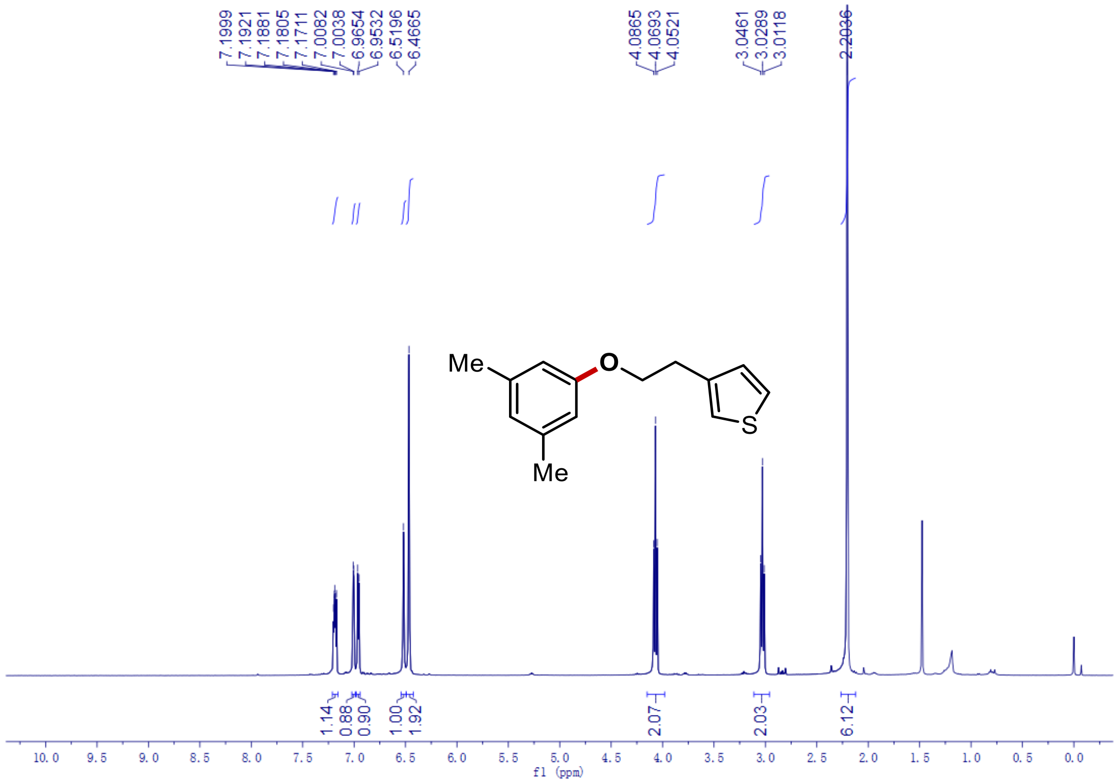

^{1}H NMR (400 MHz, $CDCl_3$) Spectrum

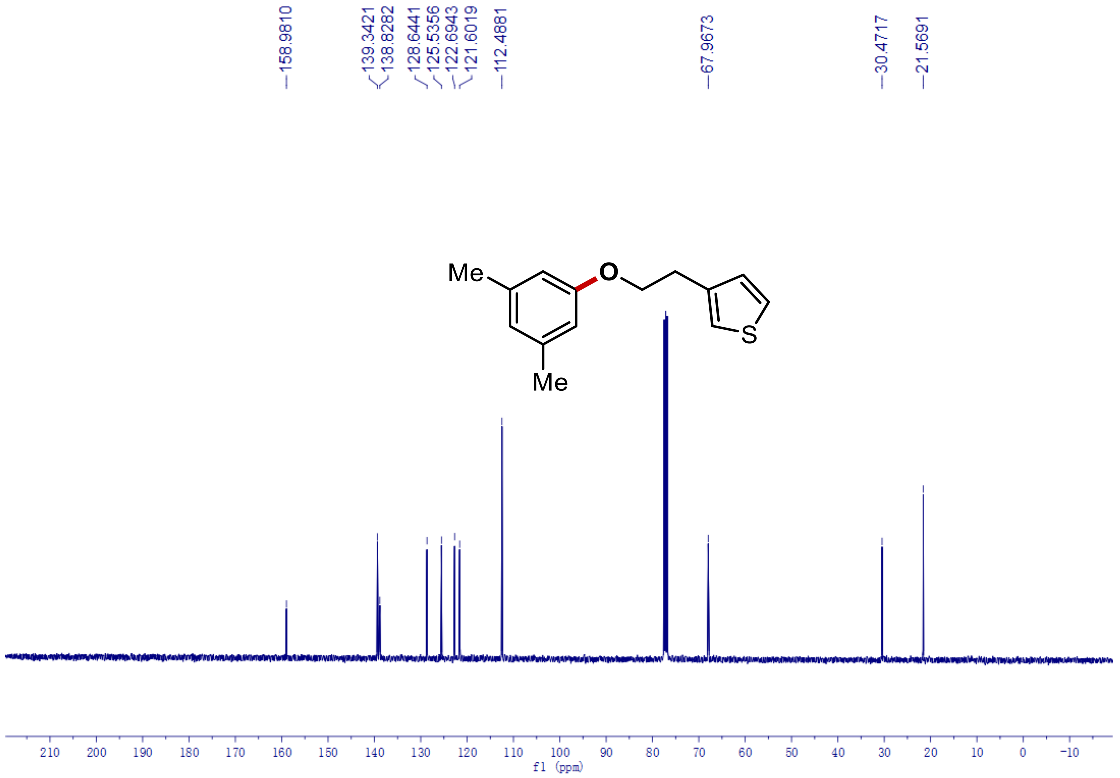

^{13}C NMR (100 MHz, $CDCl_3$) Spectrum

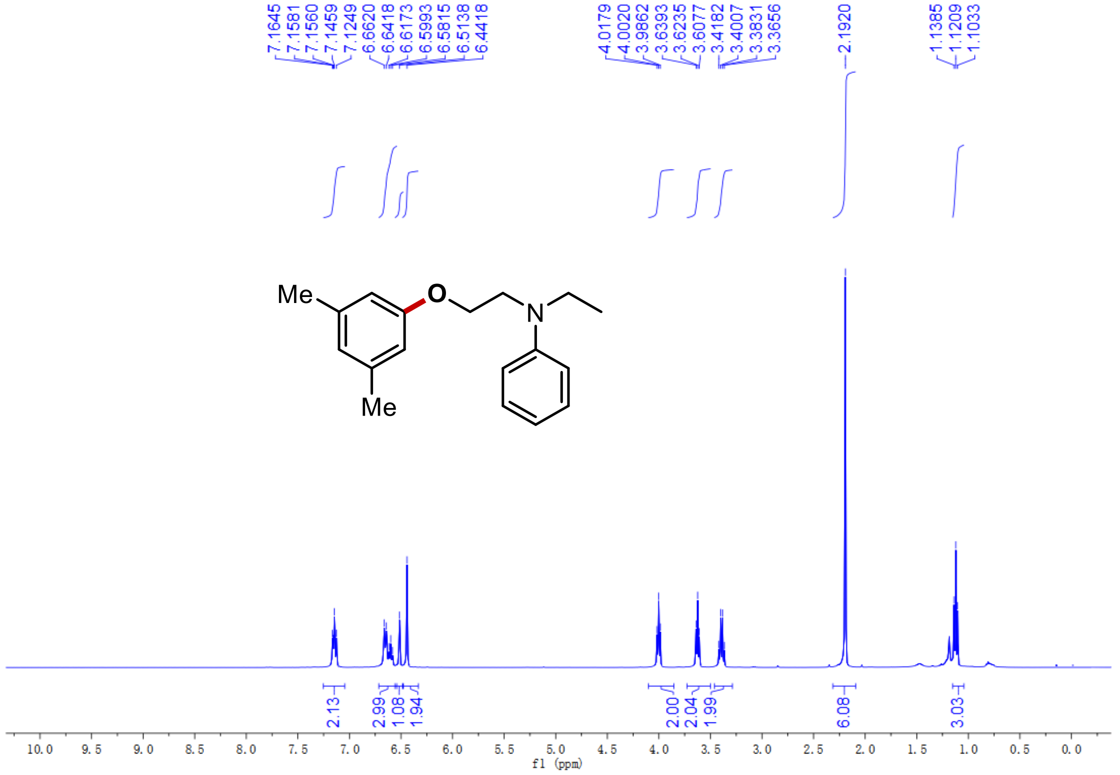

^{1}H NMR (400 MHz, $CDCl_3$) Spectrum

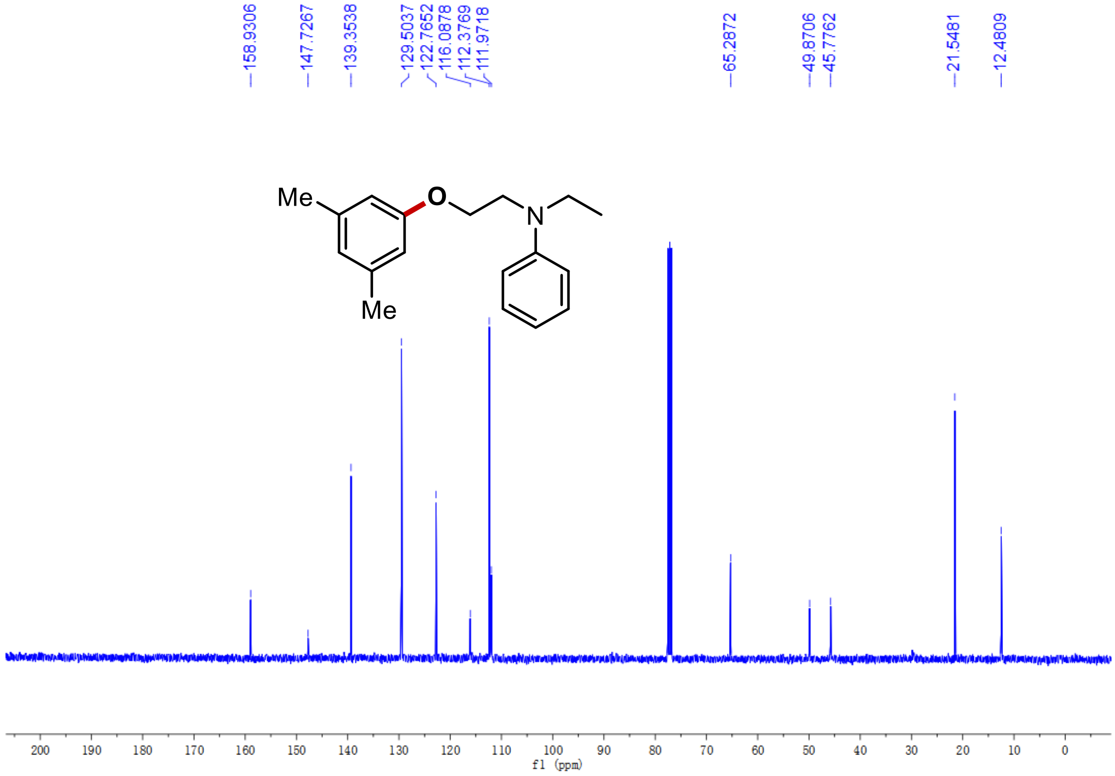

^{13}**C NMR** (100 MHz, $CDCl_3$) Spectrum

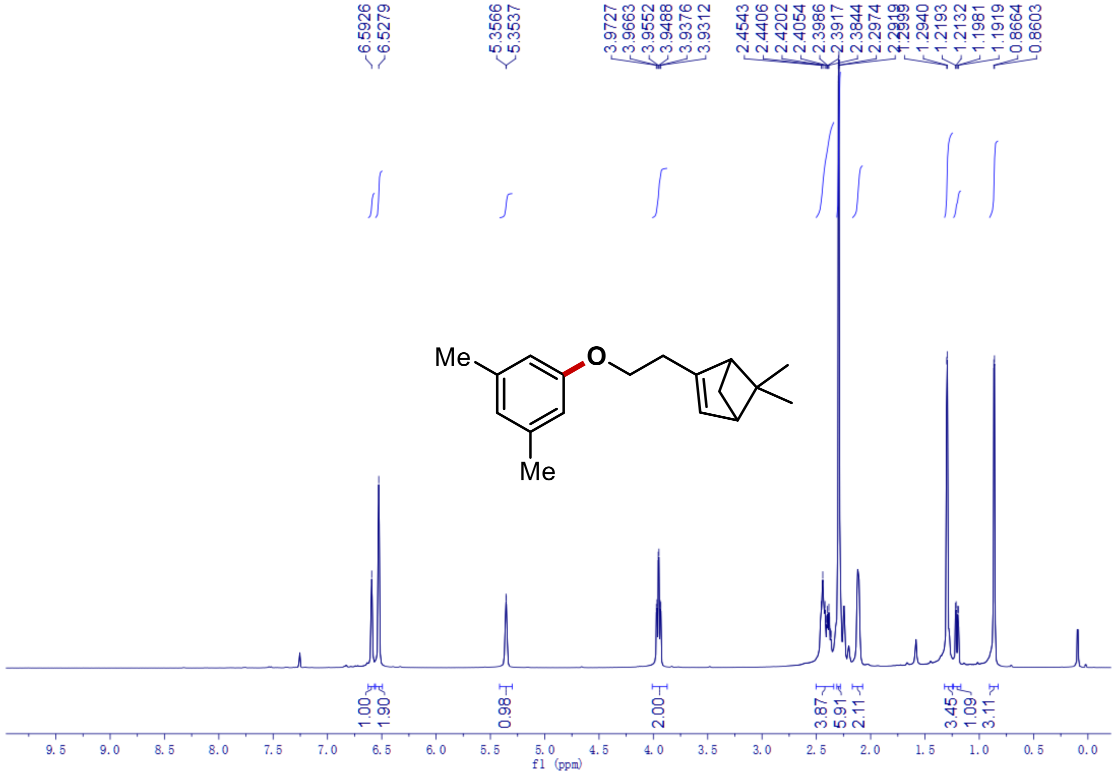

^{1}**H NMR** (400 MHz, $CDCl_3$) Spectrum

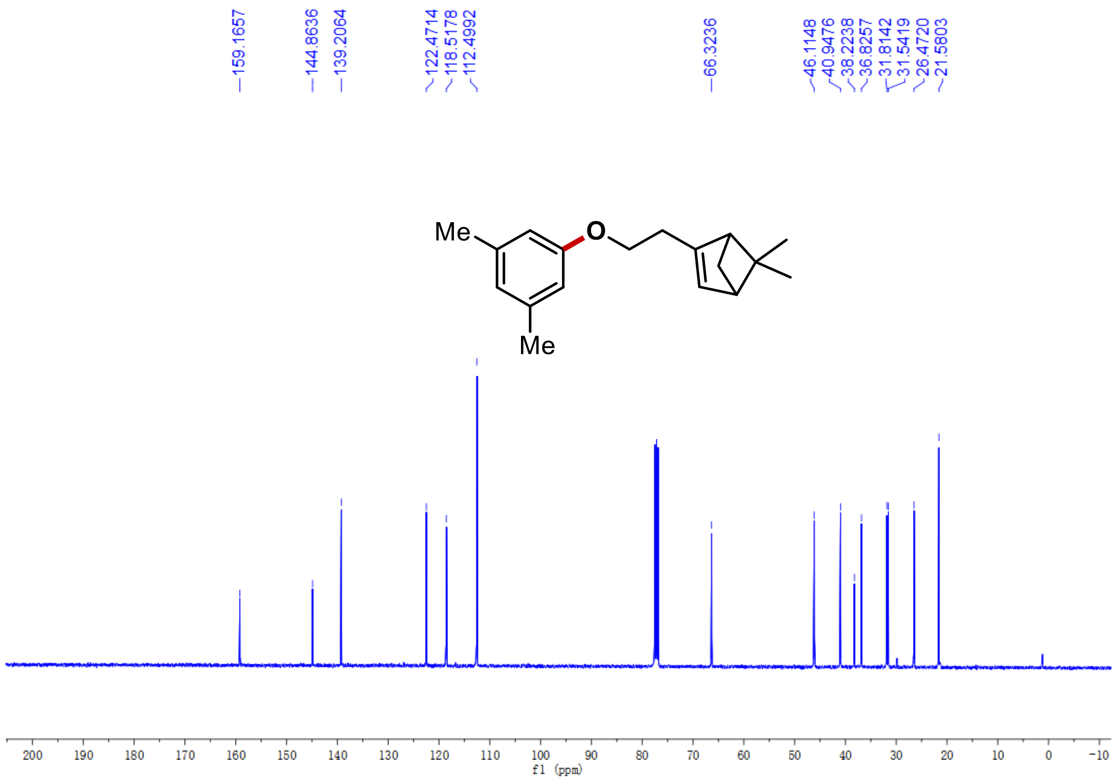

^{13}C NMR (100 MHz, $CDCl_3$) Spectrum

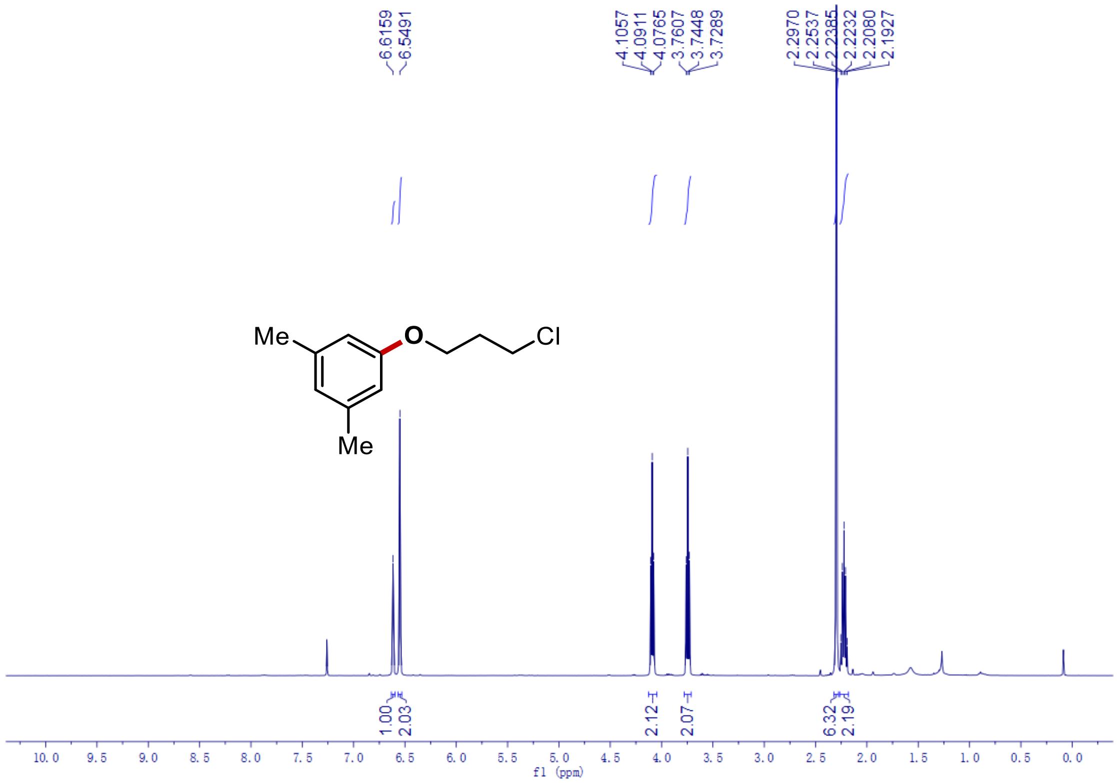

^{1}H NMR (400 MHz, $CDCl_3$) Spectrum

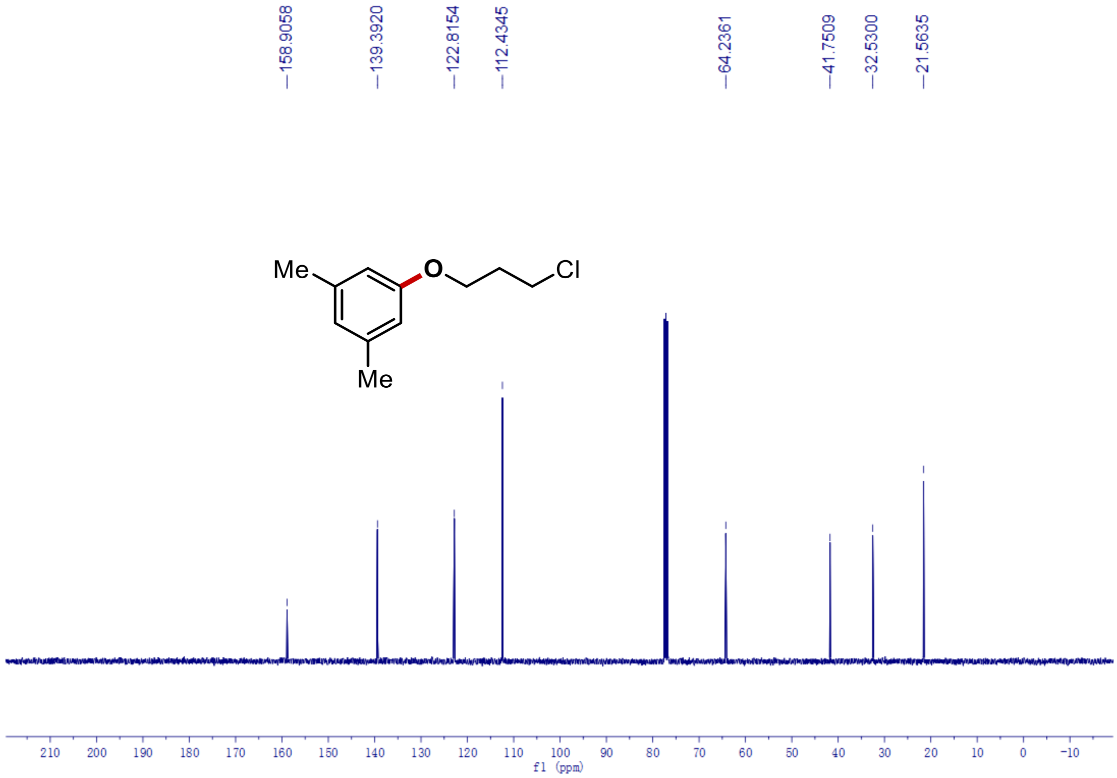

^{13}C NMR (100 MHz, $CDCl_3$) Spectrum

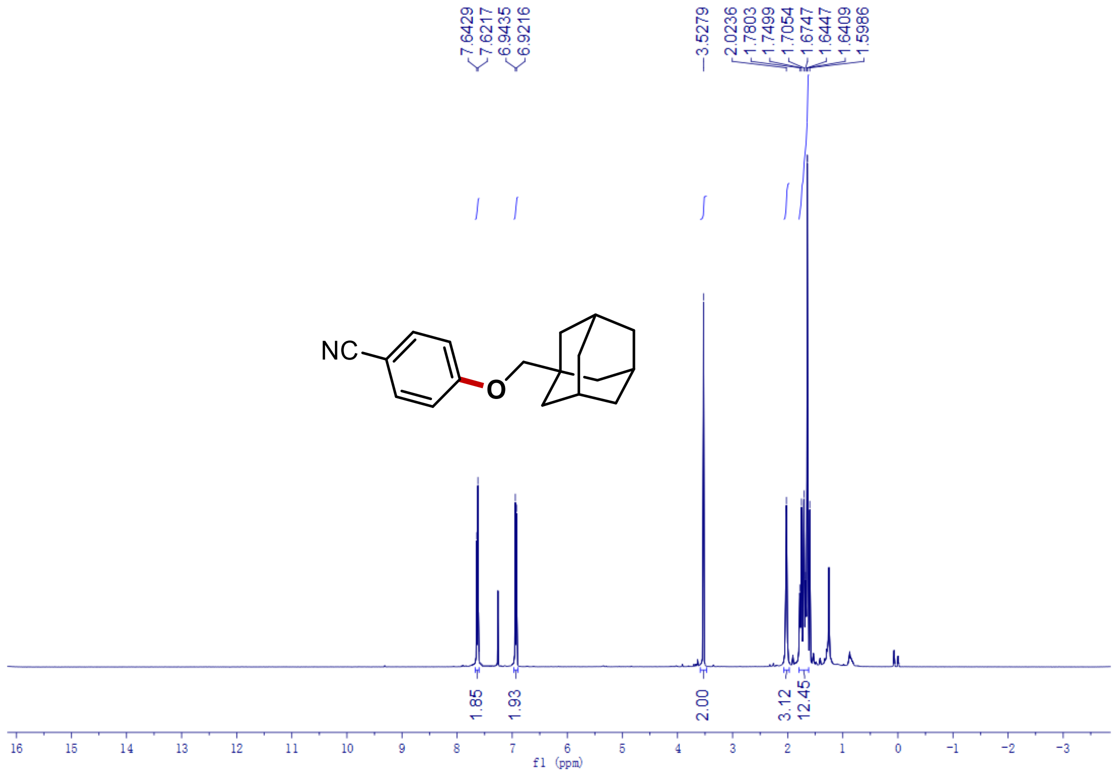

^{1}H NMR (400 MHz, $CDCl_3$) Spectrum

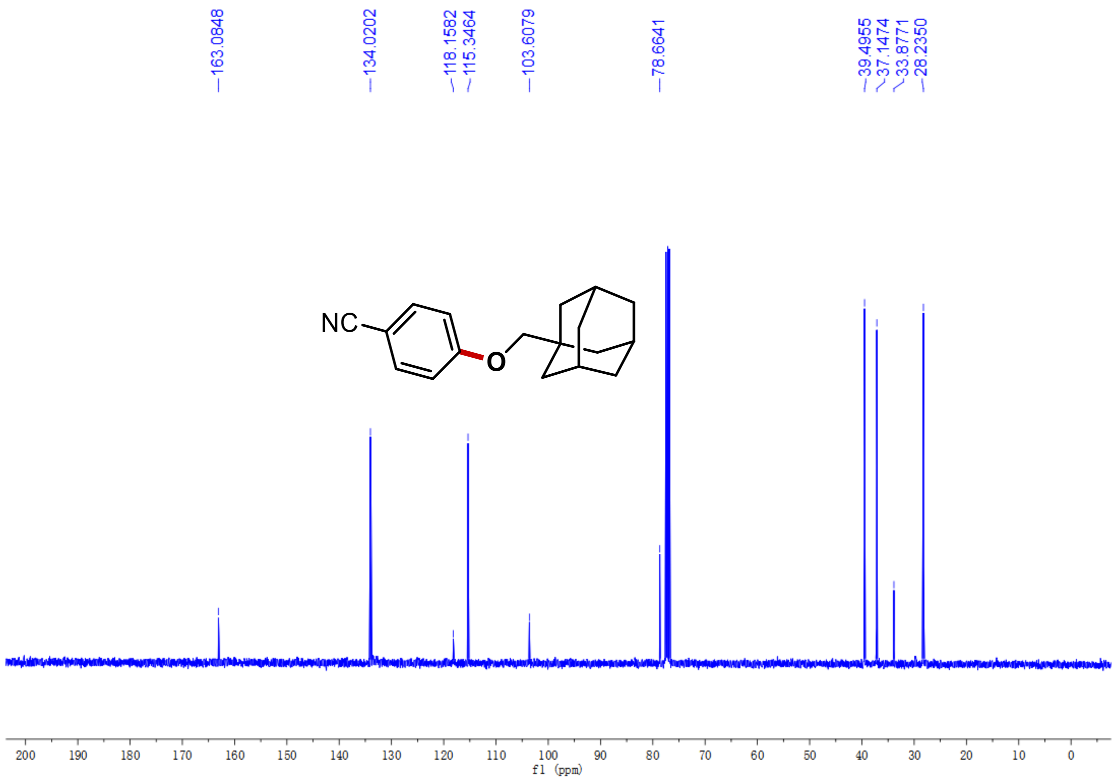

^{13}C NMR (100 MHz, $CDCl_3$) Spectrum

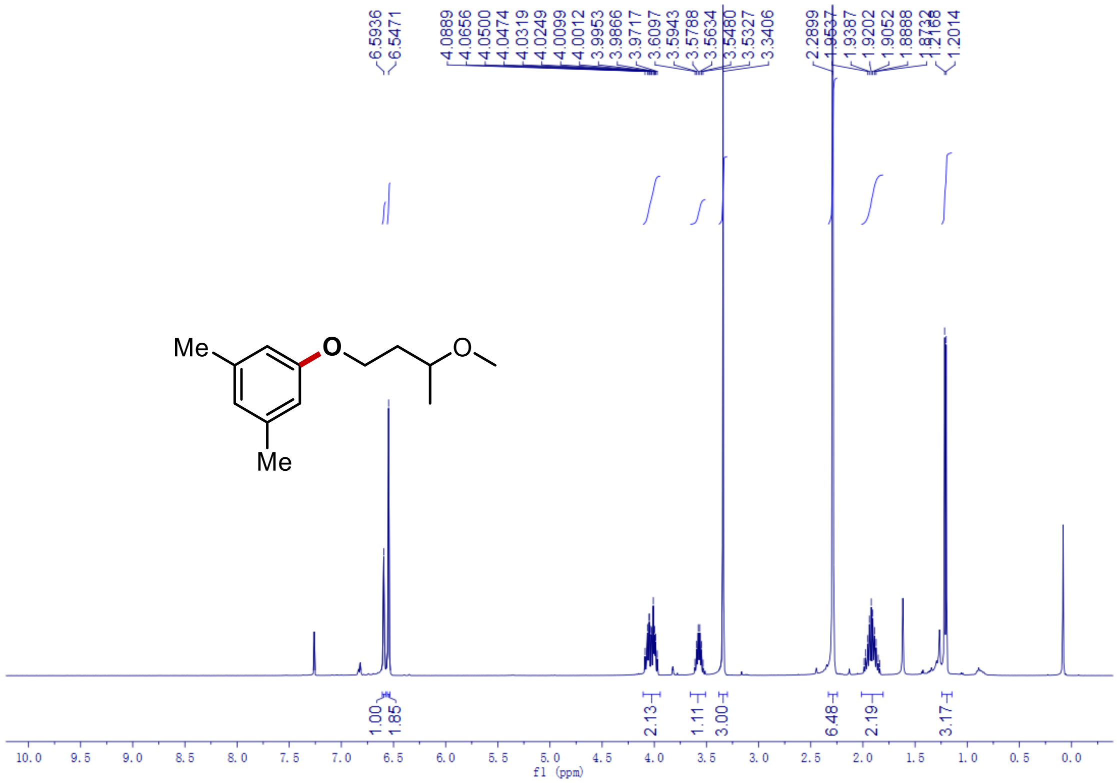

^{1}H NMR (400 MHz, $CDCl_3$) Spectrum

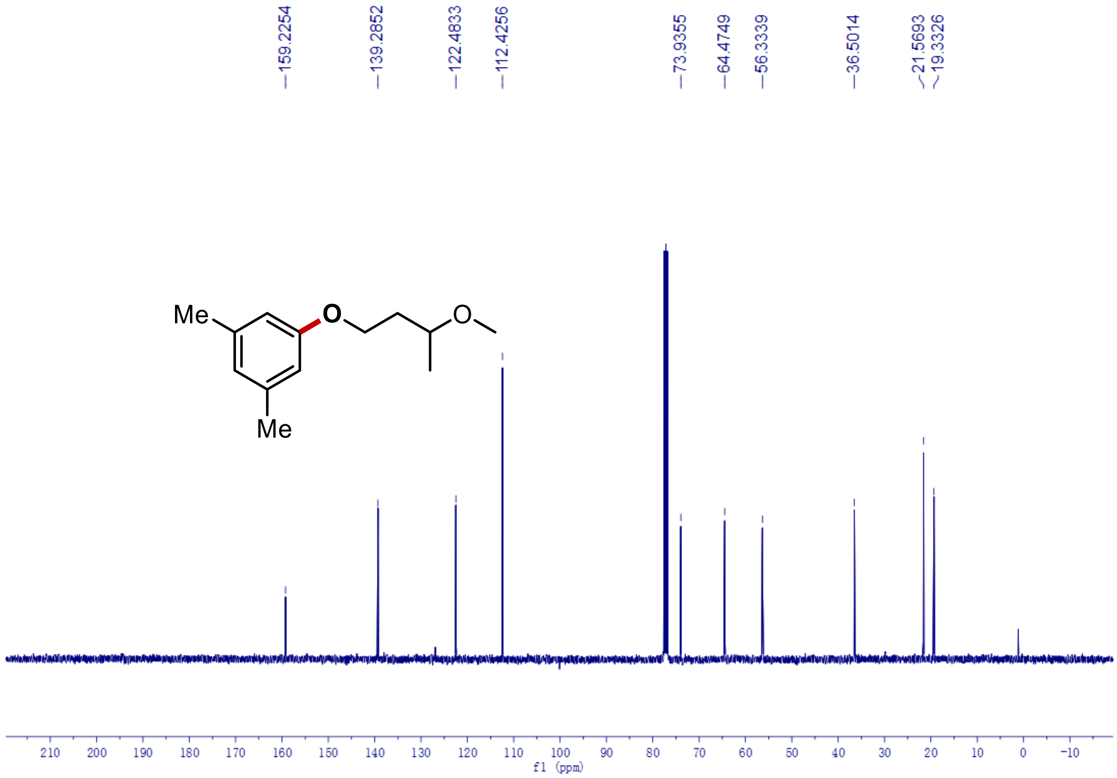

^{13}C NMR (100 MHz, $CDCl_3$) Spectrum

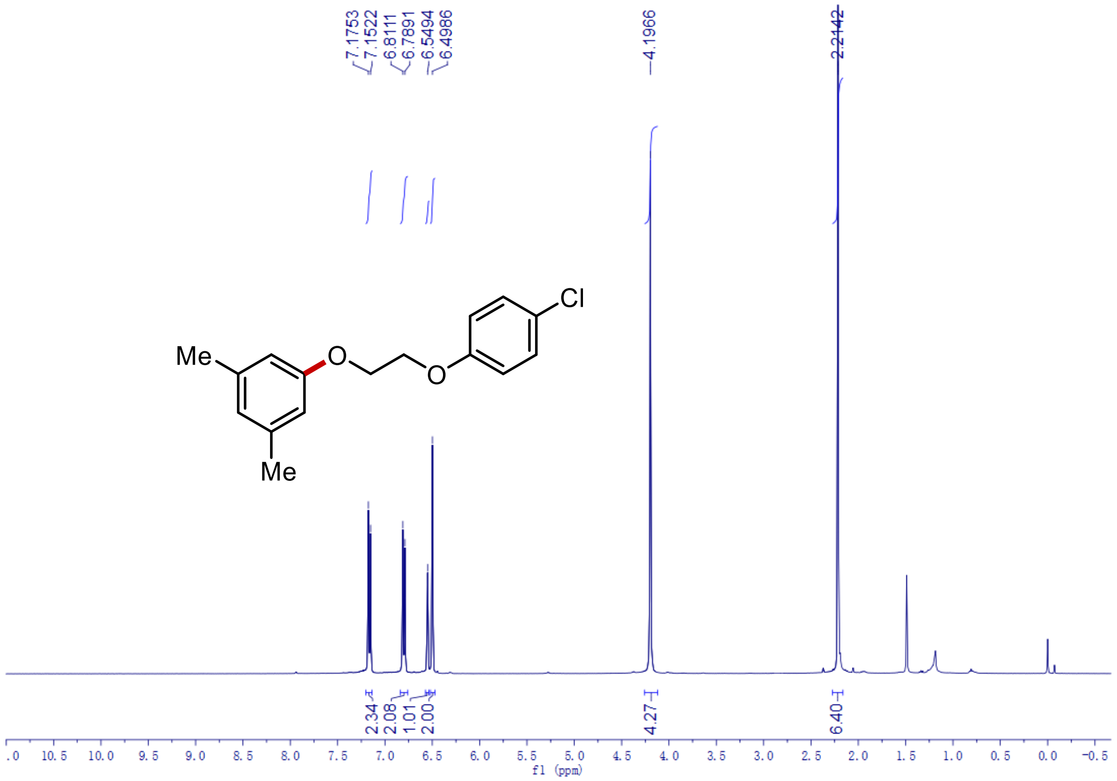

^{1}H NMR (400 MHz, $CDCl_3$) Spectrum

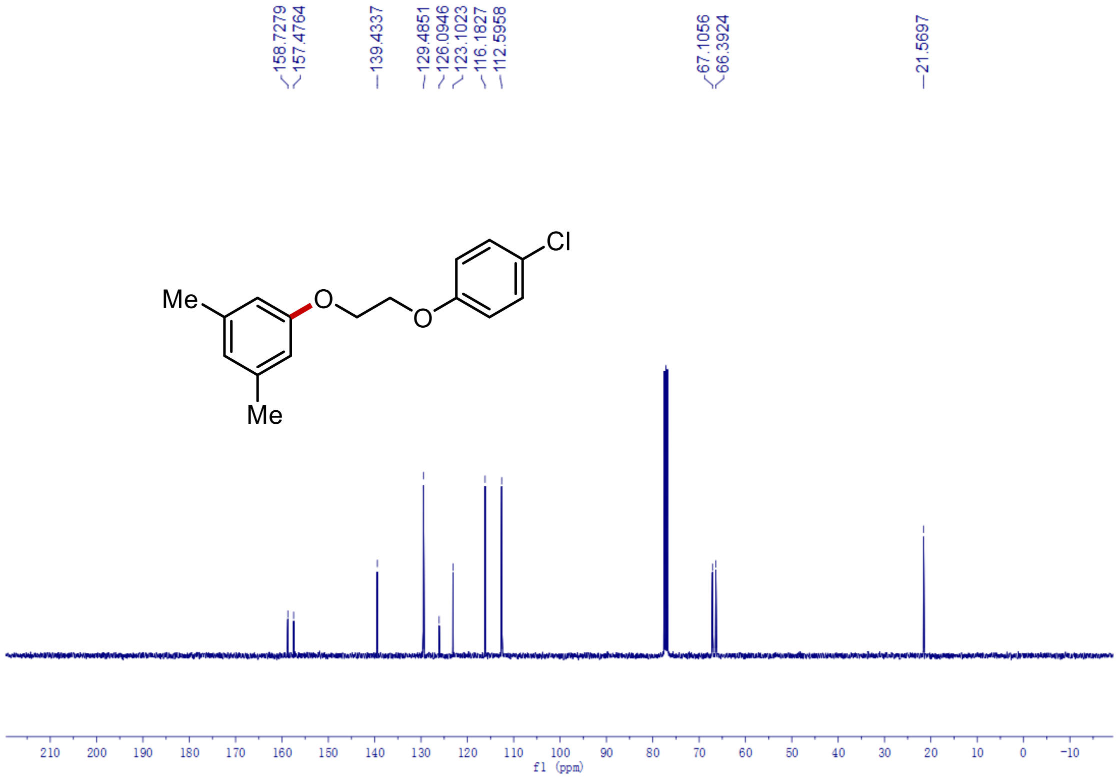

^{13}**C NMR** (100 MHz, $CDCl_3$) Spectrum

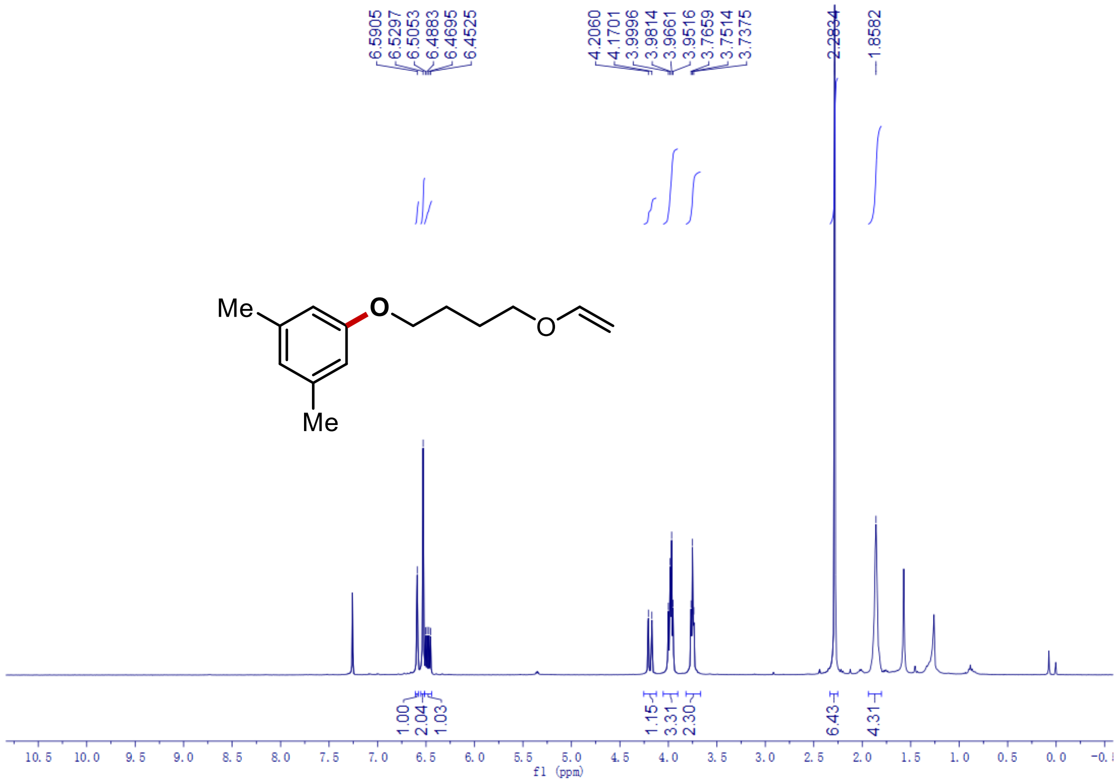

^{1}**H NMR** (400 MHz, $CDCl_3$) Spectrum

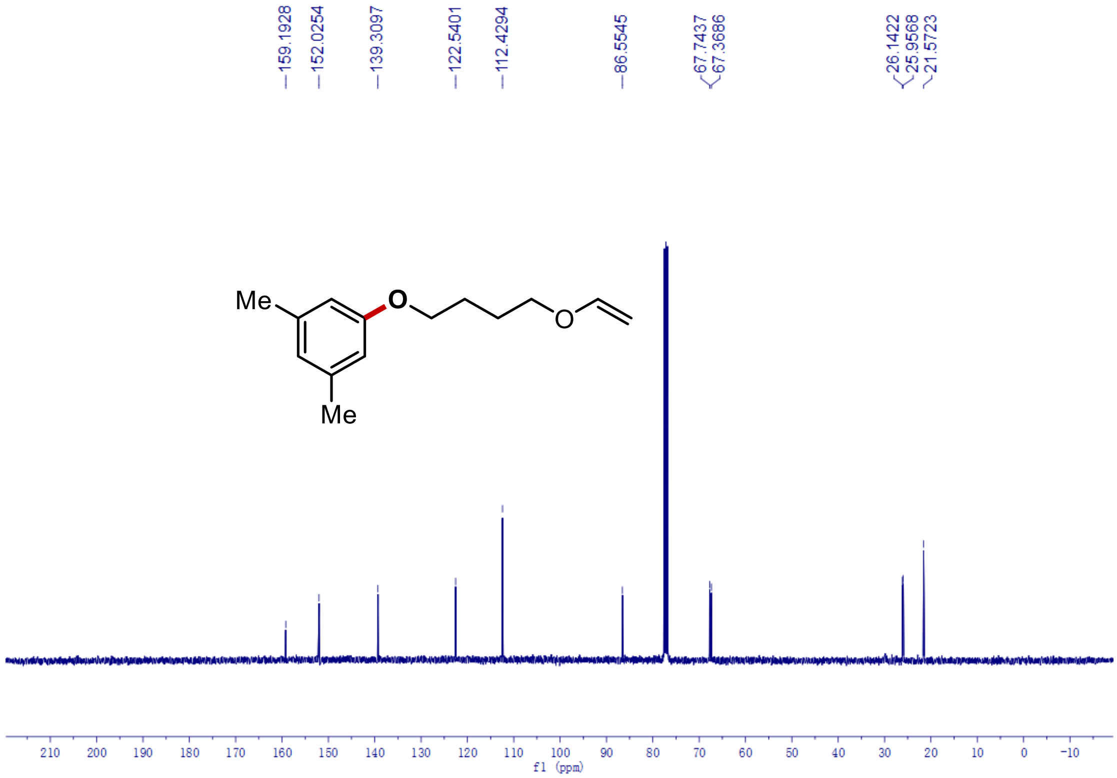

^{13}**C NMR** (100 MHz, $CDCl_3$) Spectrum

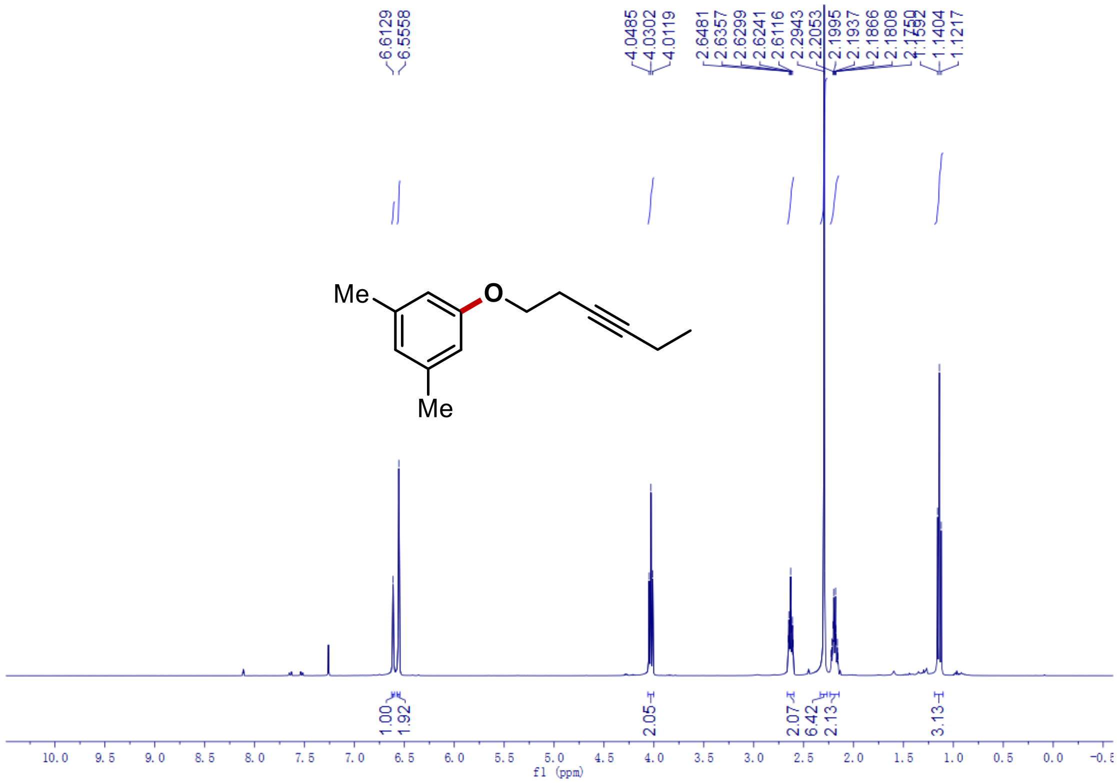

^{1}**H NMR** (400 MHz, $CDCl_3$) Spectrum

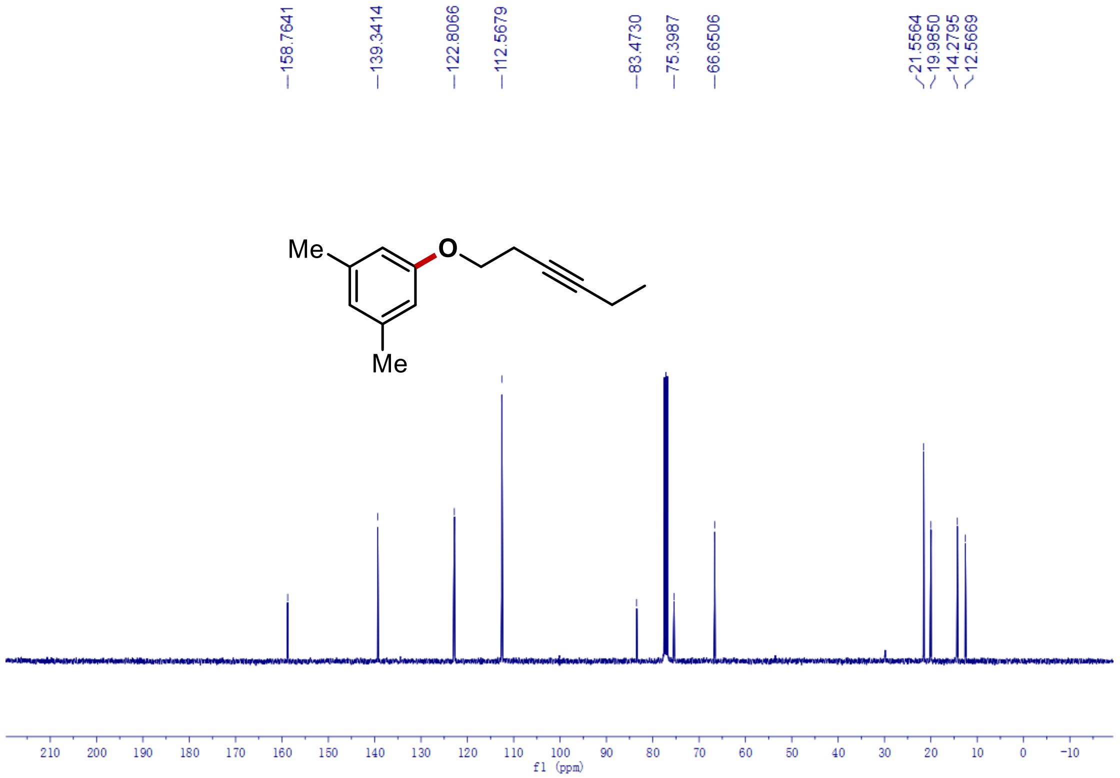

^{13}C NMR (100 MHz, $CDCl_3$) Spectrum

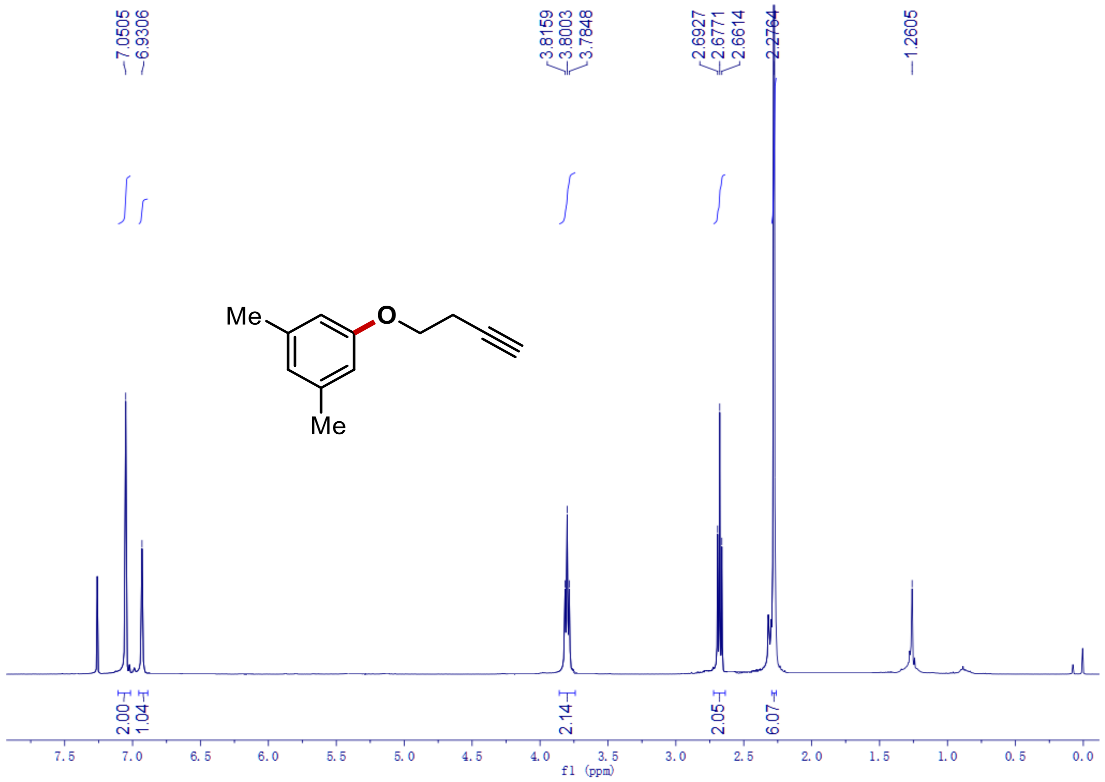

^{1}H NMR (400 MHz, $CDCl_3$) Spectrum

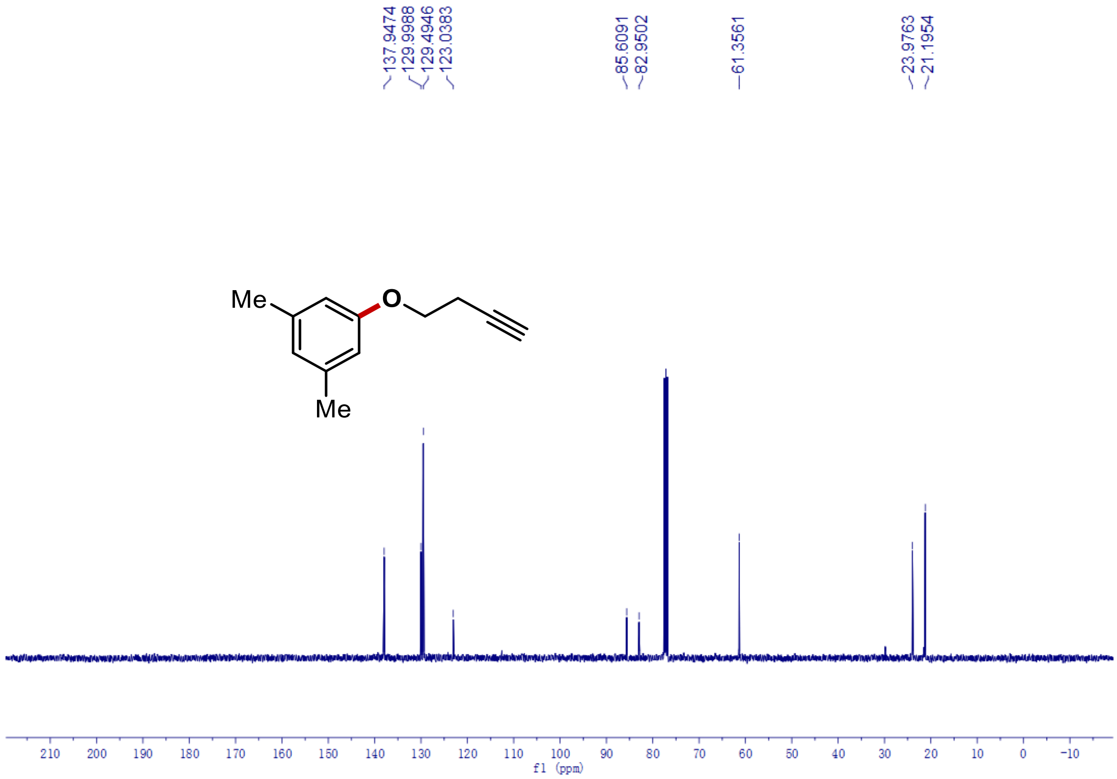

^{13}C NMR (100 MHz, $CDCl_3$) Spectrum

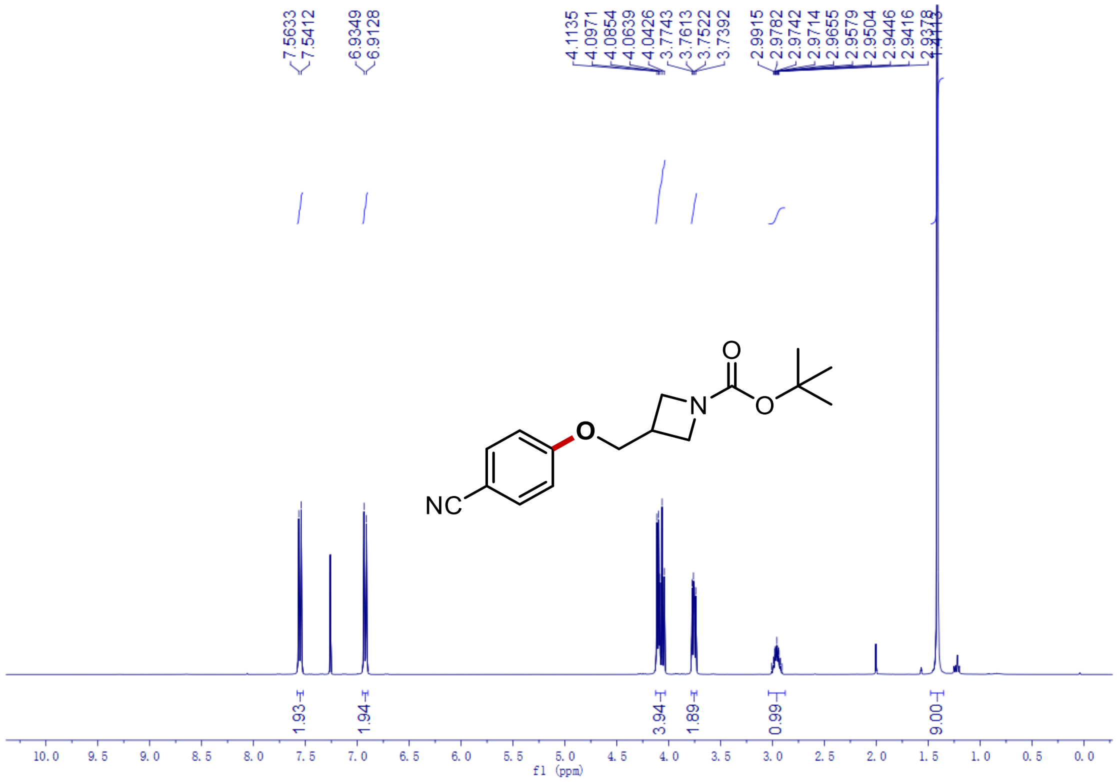

^{1}H NMR (400 MHz, $CDCl_3$) Spectrum

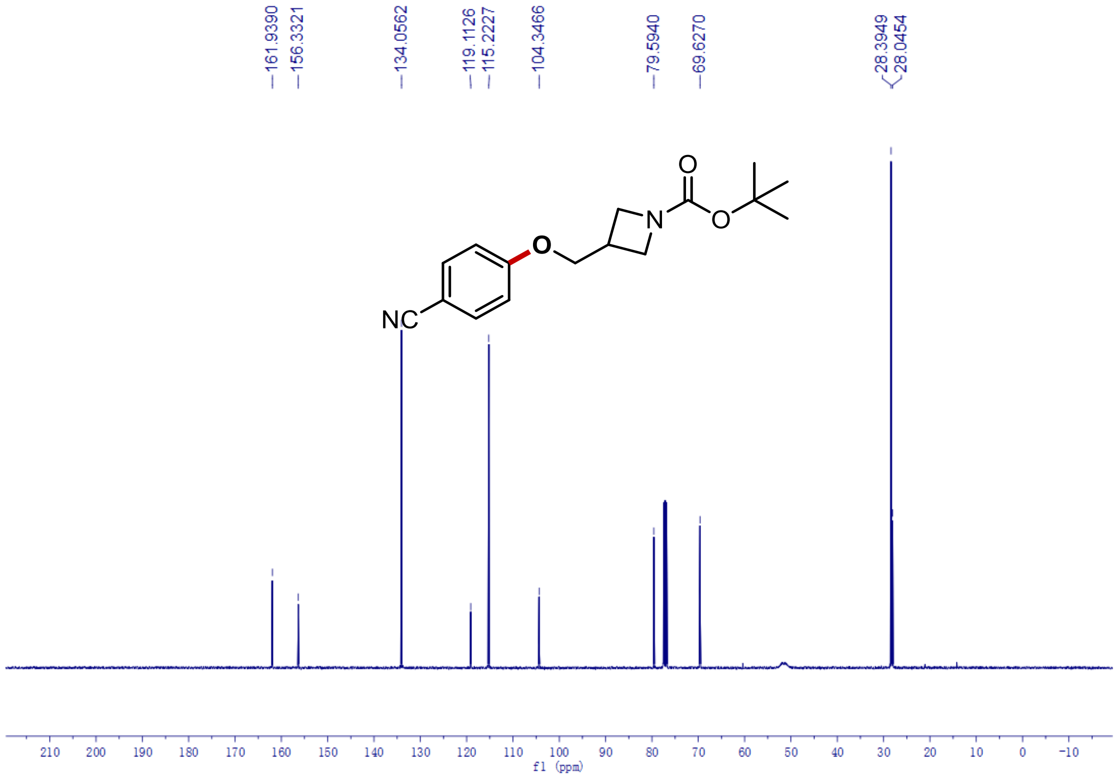

^{13}C NMR (100 MHz, $CDCl_3$) Spectrum

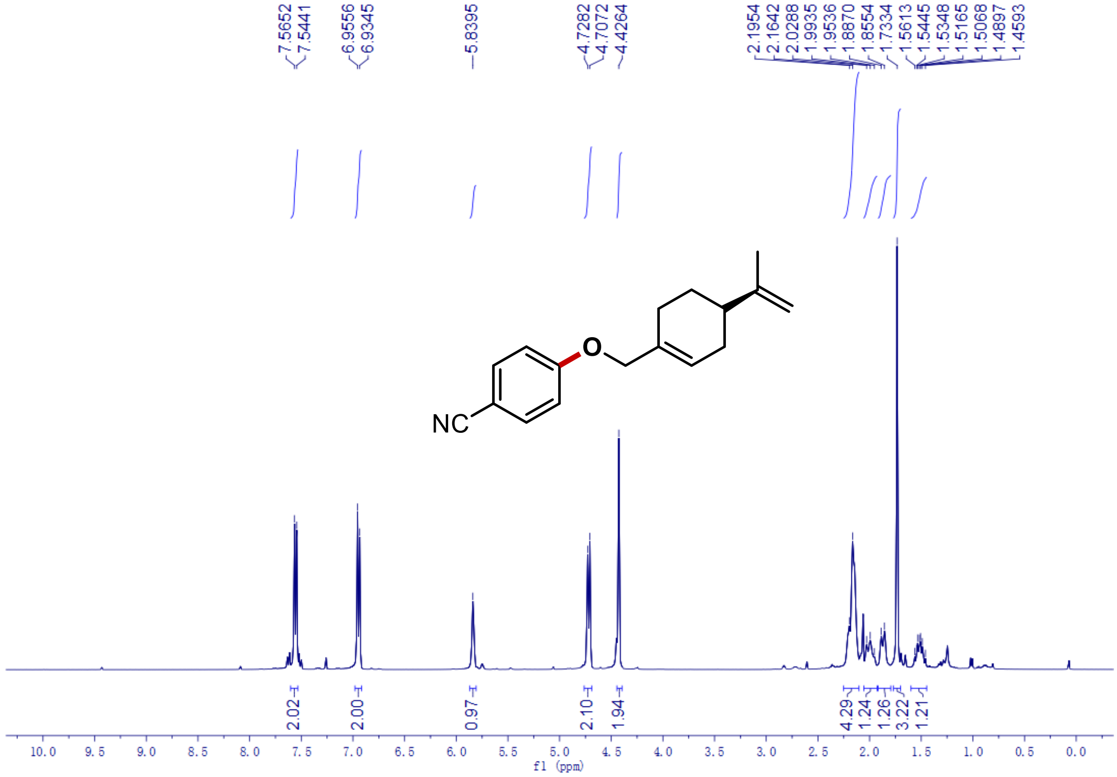

^{1}H NMR (400 MHz, $CDCl_3$) Spectrum

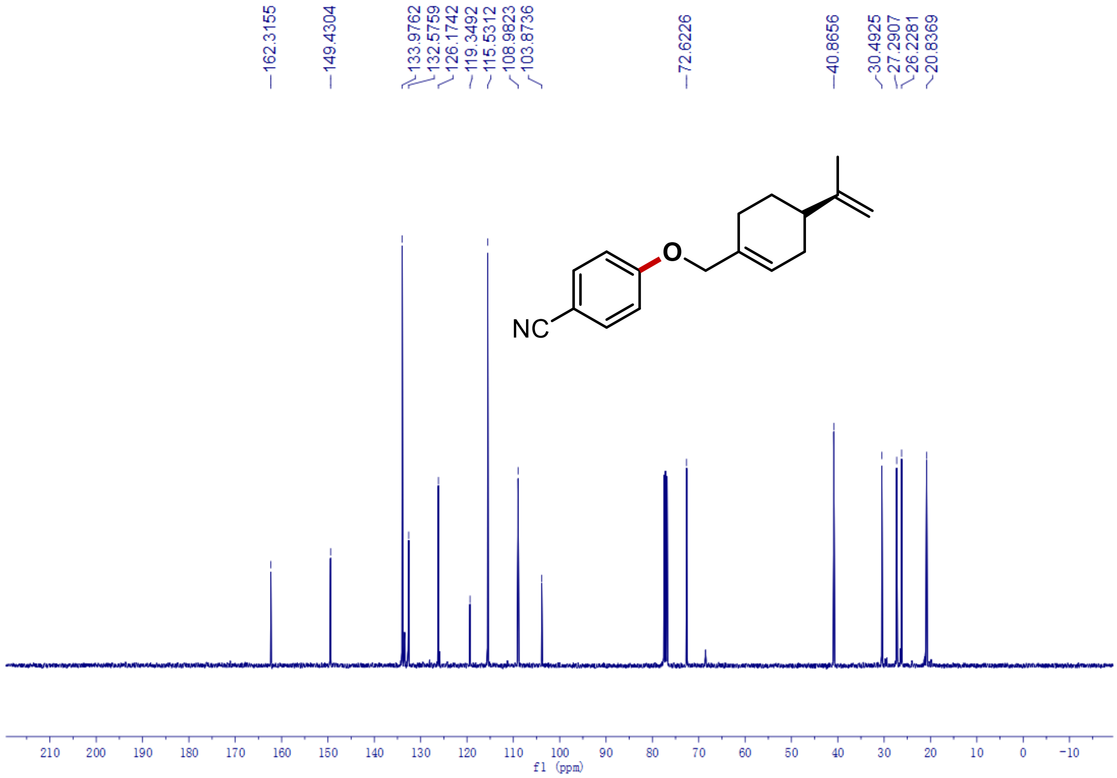

^{13}C NMR (100 MHz, $CDCl_3$) Spectrum

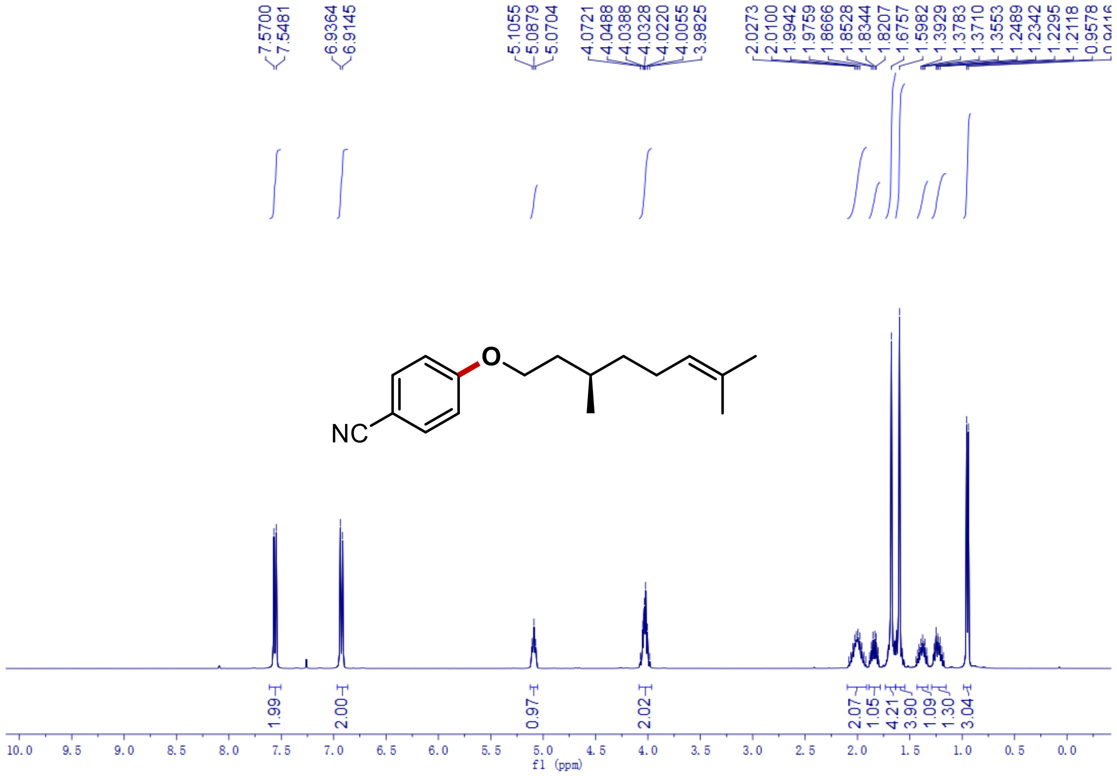

^{1}H NMR (400 MHz, $CDCl_3$) Spectrum

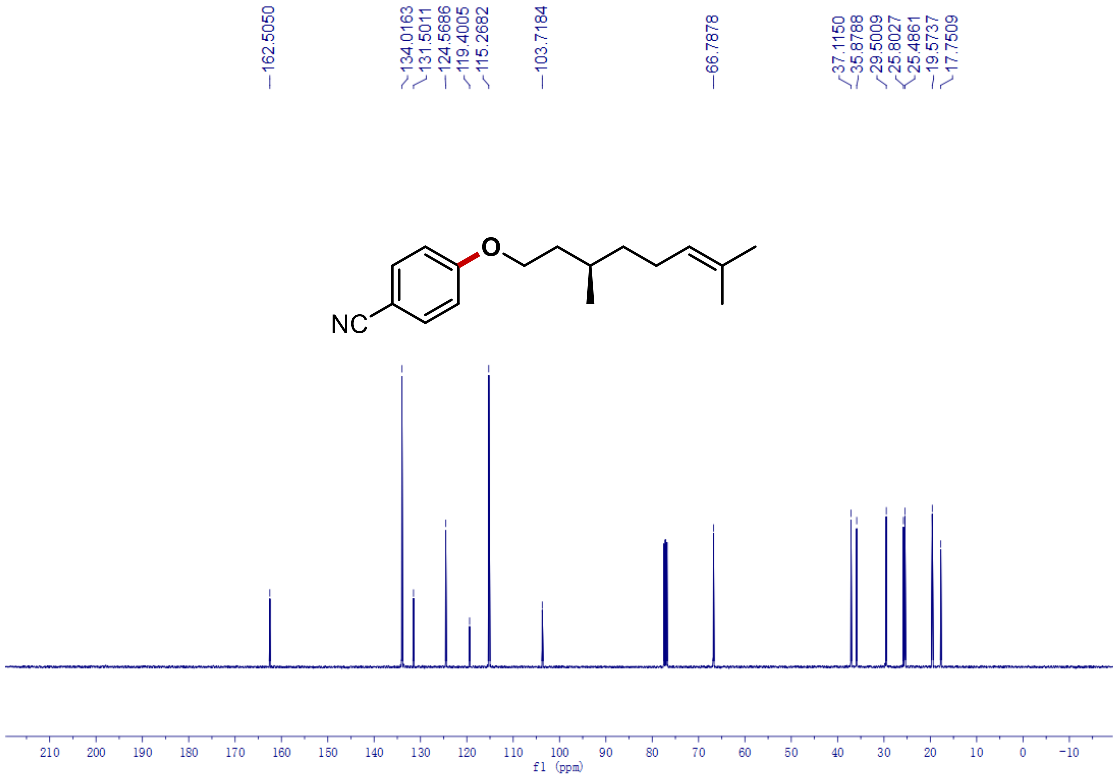

^{13}C **NMR** (100 MHz, $CDCl_3$) Spectrum

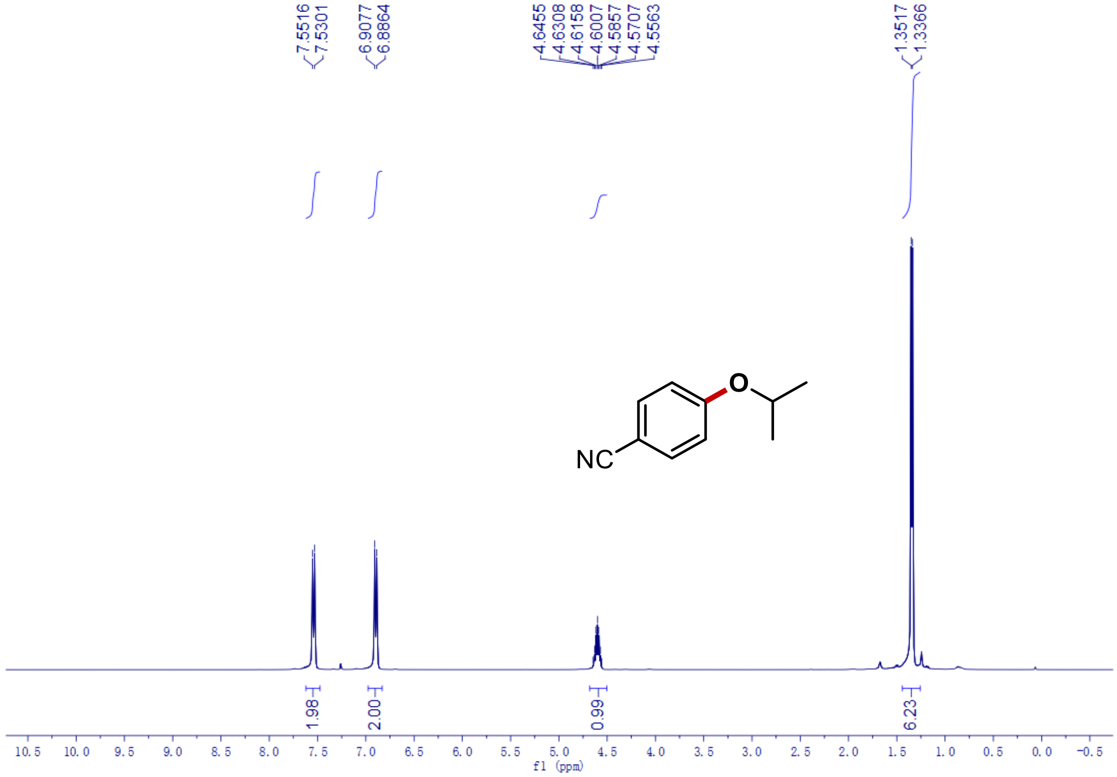

^{1}H **NMR** (400 MHz, $CDCl_3$) Spectrum

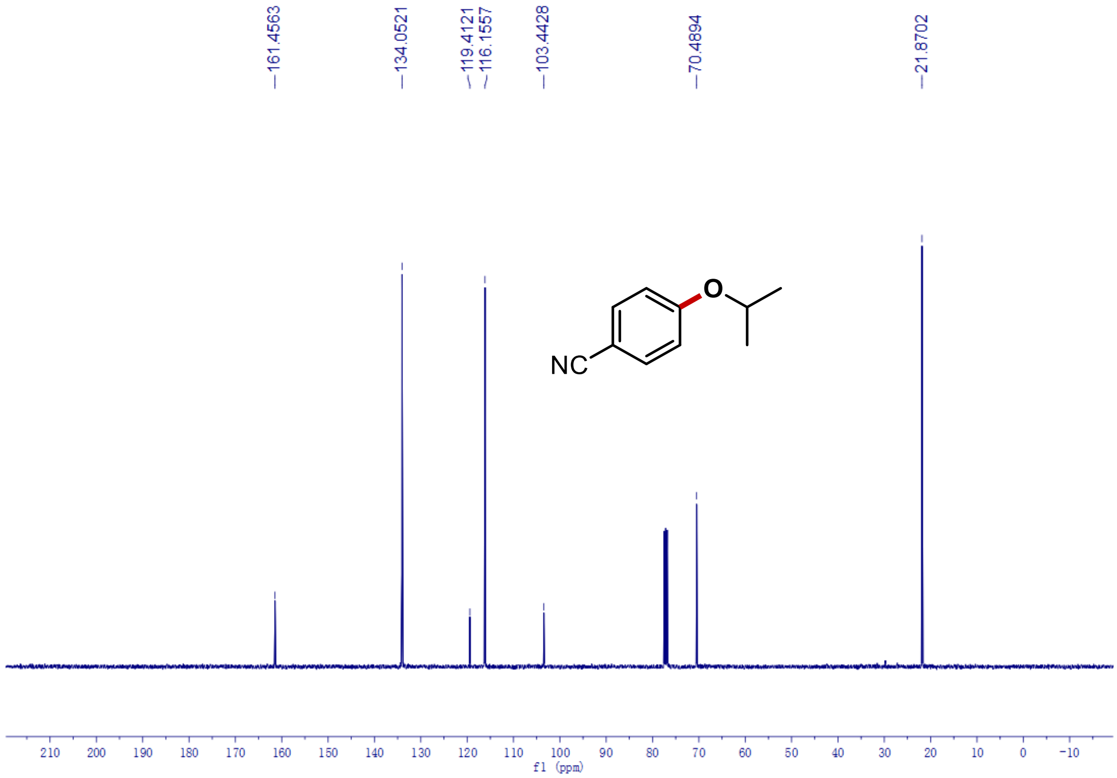

^{13}C NMR (100 MHz, $CDCl_3$) Spectrum

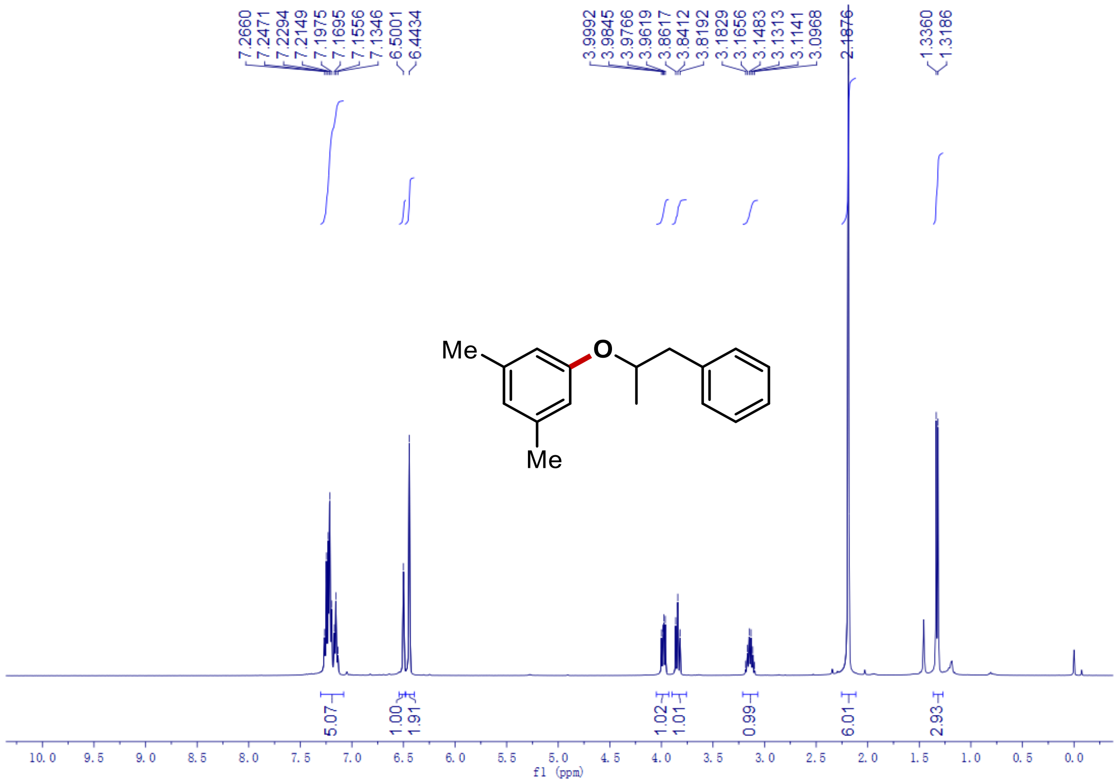

^{1}H NMR (400 MHz, $CDCl_3$) Spectrum

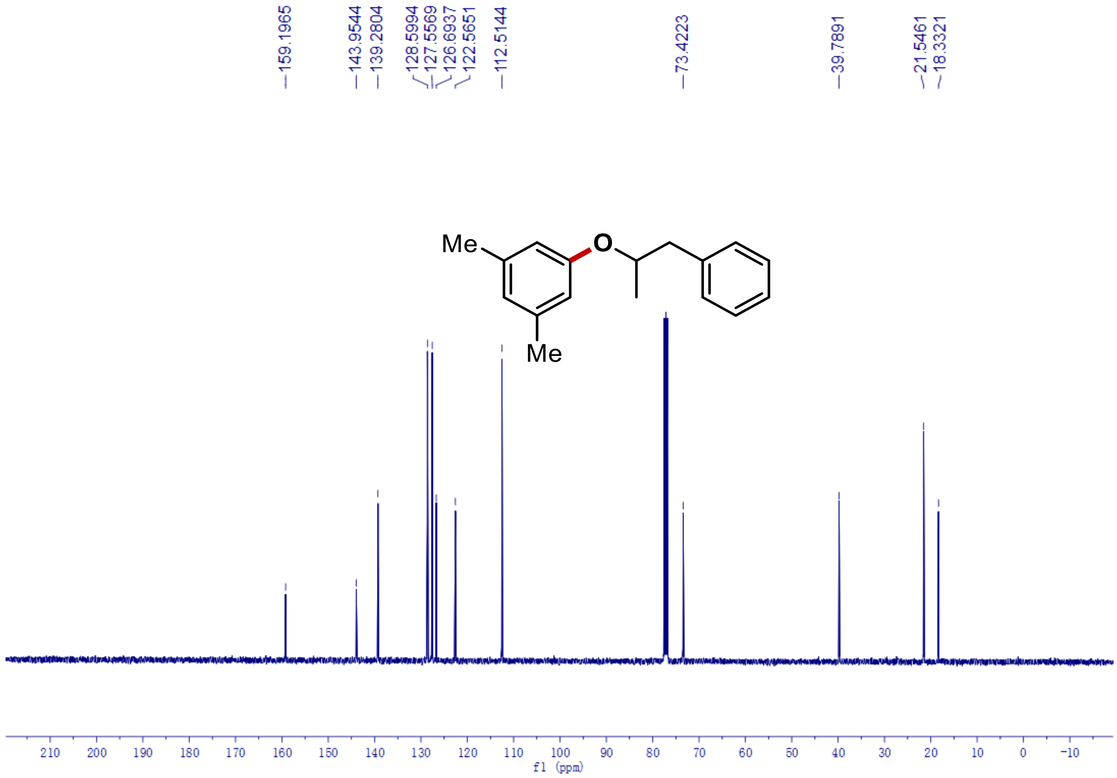

^{13}C NMR (100 MHz, $CDCl_3$) Spectrum

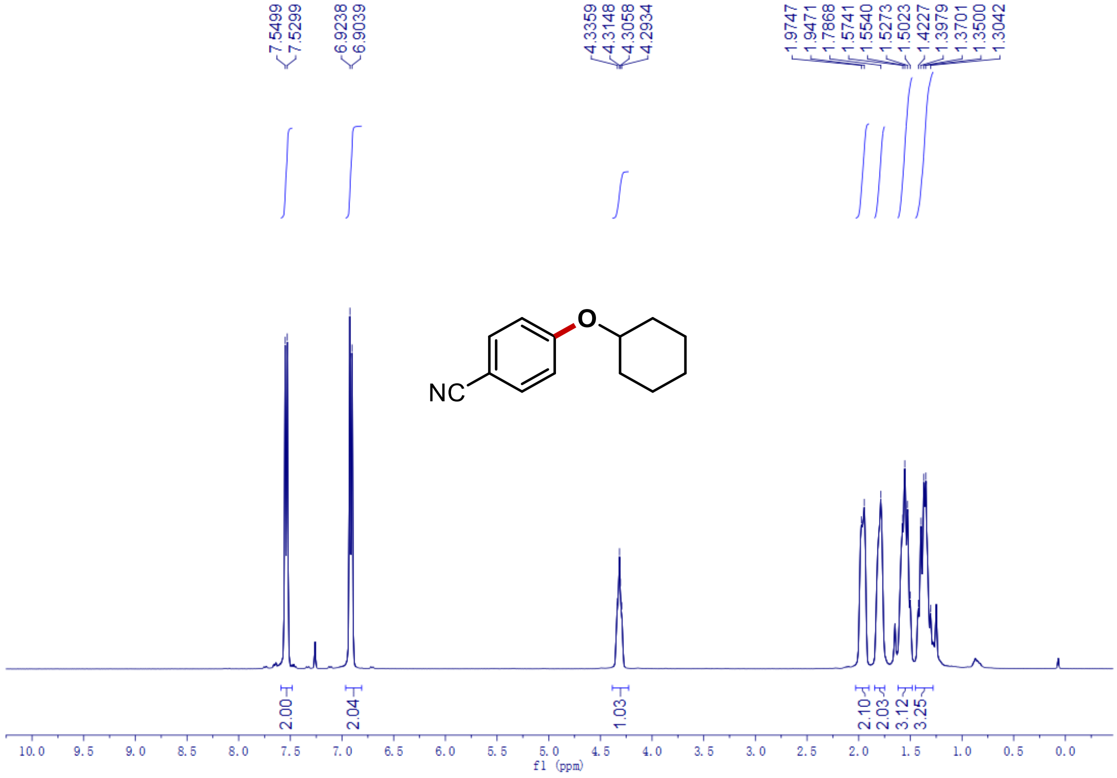

^{1}H NMR (400 MHz, $CDCl_3$) Spectrum

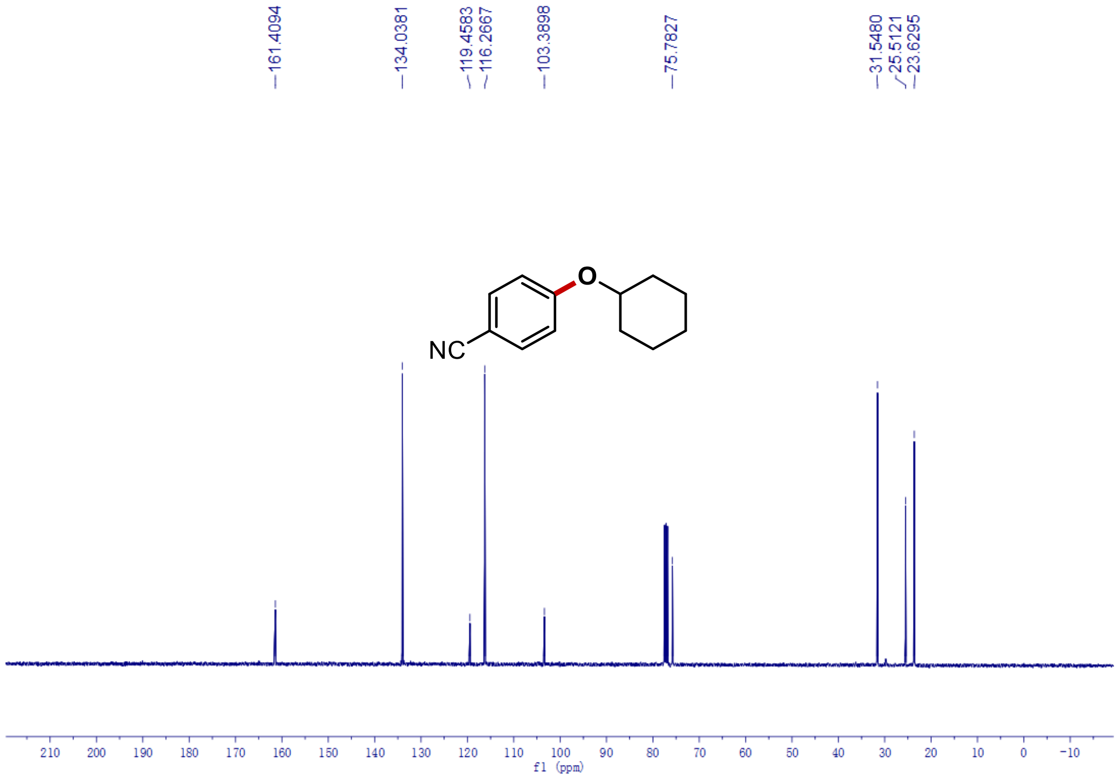

^{13}C NMR (100 MHz, $CDCl_3$) Spectrum

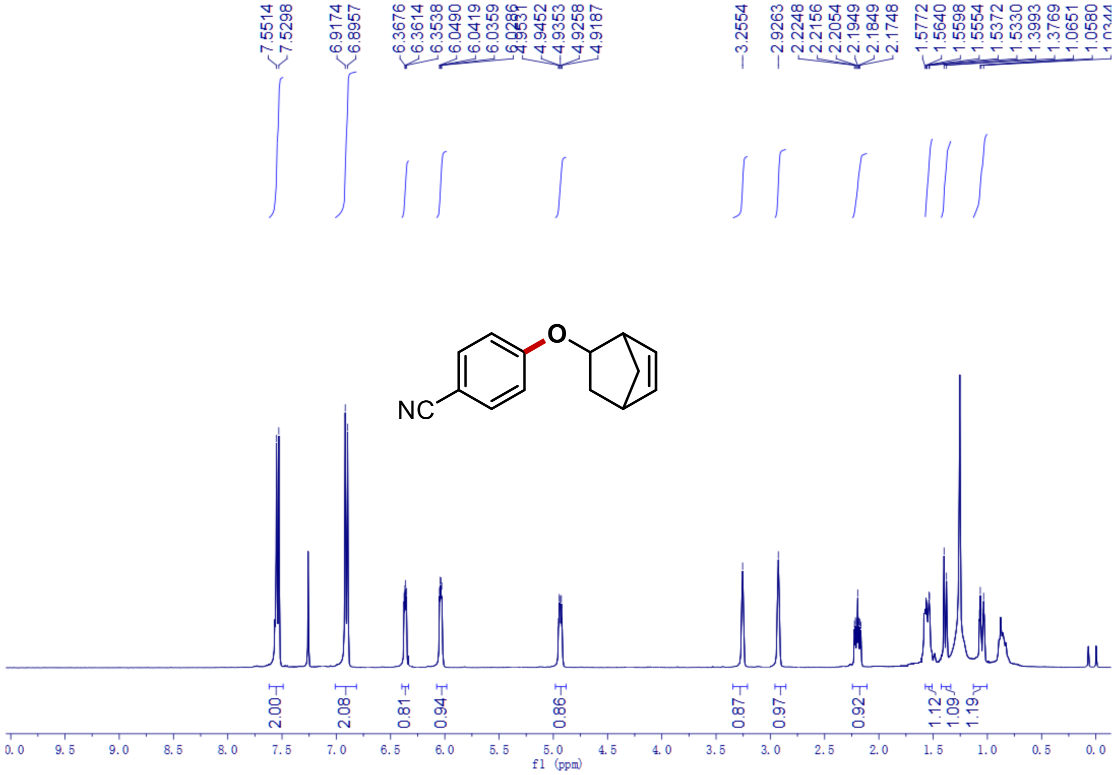

^{1}H NMR (400 MHz, $CDCl_3$) Spectrum

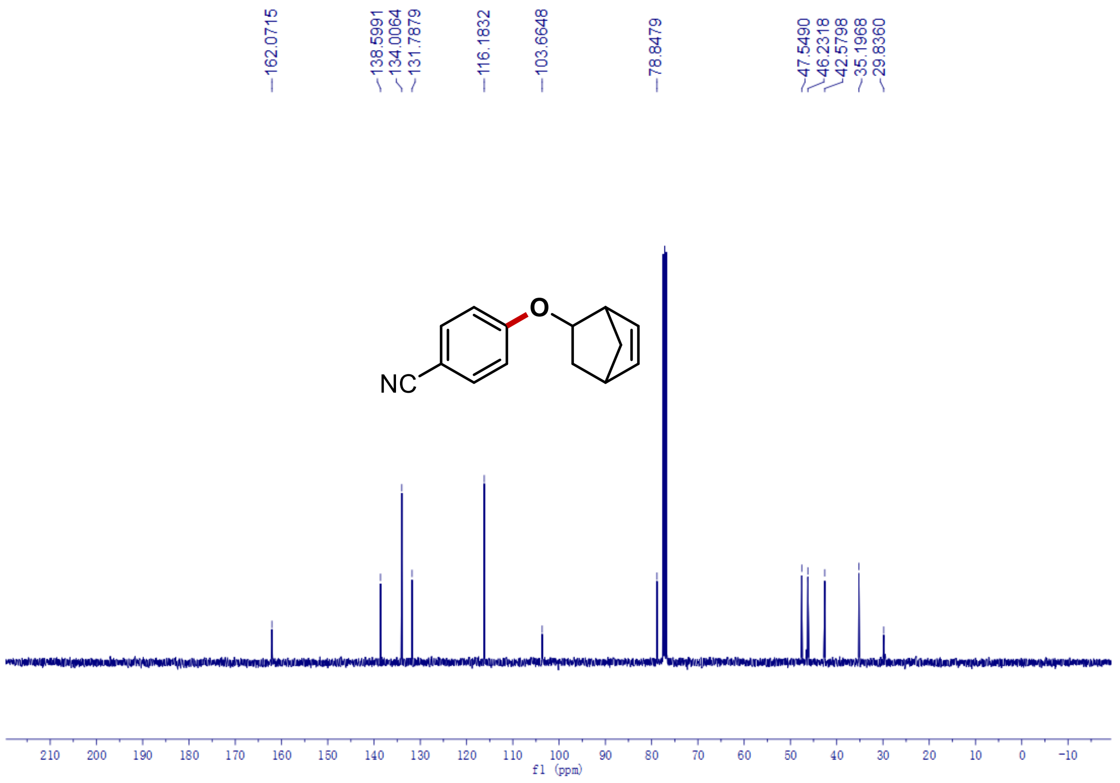

^{13}C NMR (100 MHz, $CDCl_3$) Spectrum

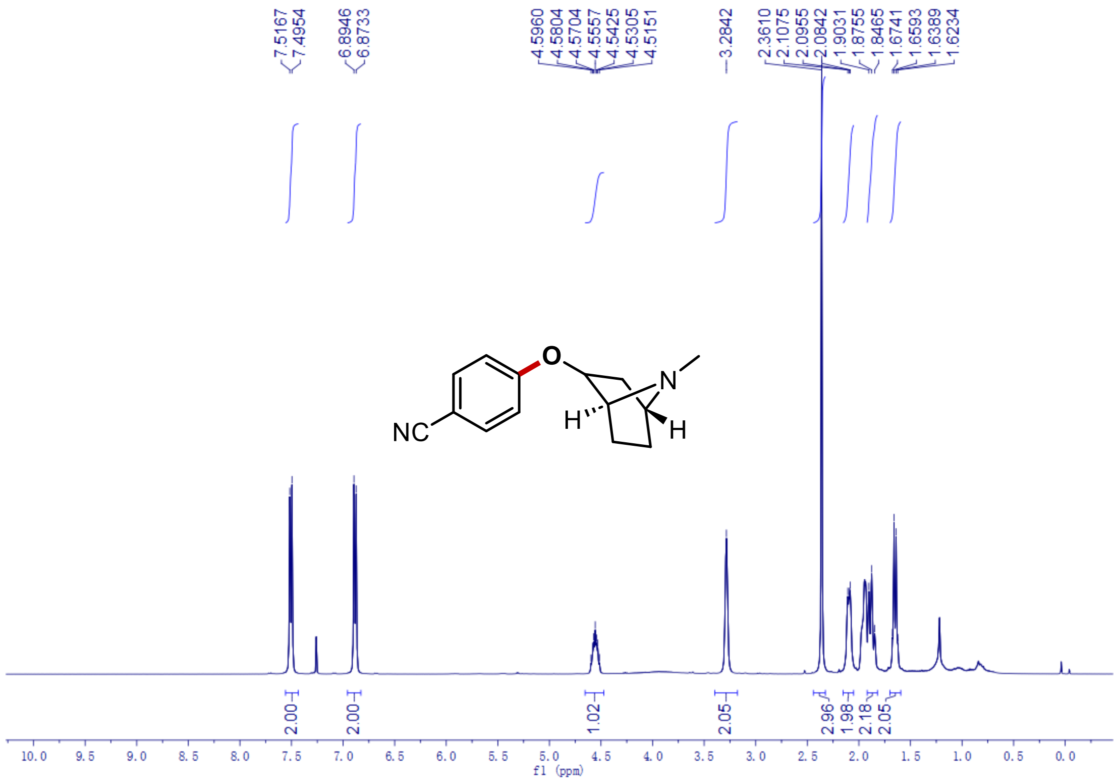

^{1}H NMR (400 MHz, $CDCl_3$) Spectrum

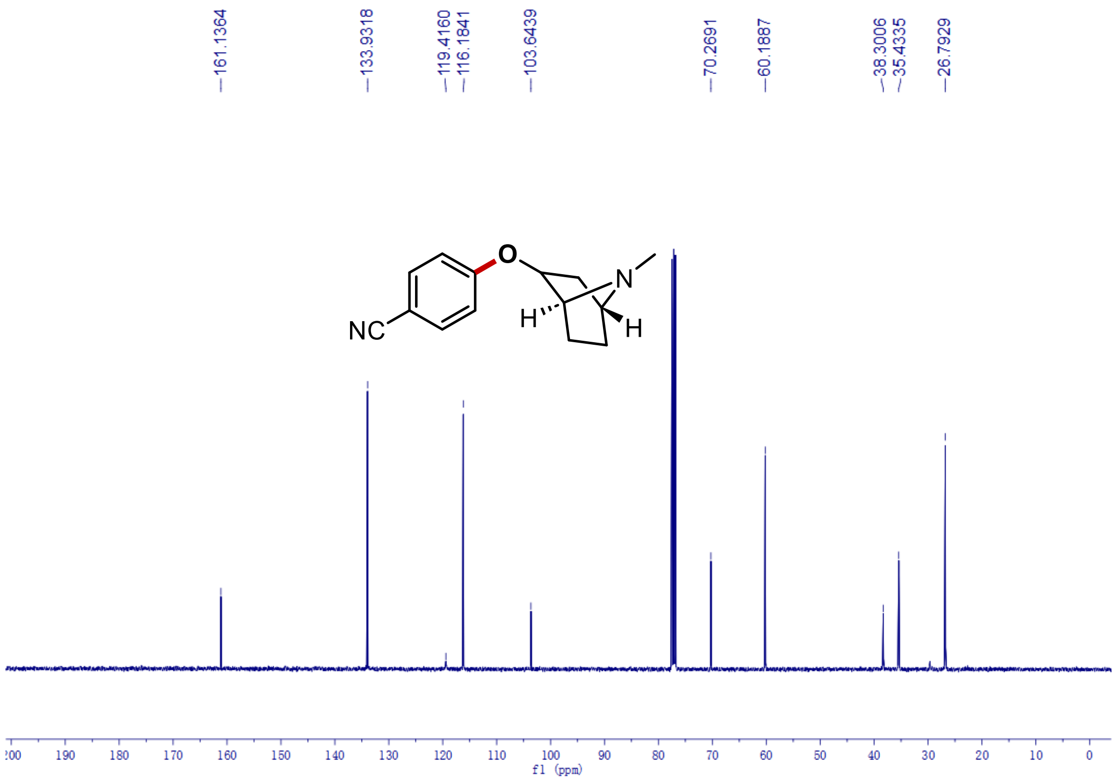

^{13}C NMR (100 MHz, $CDCl_3$) Spectrum

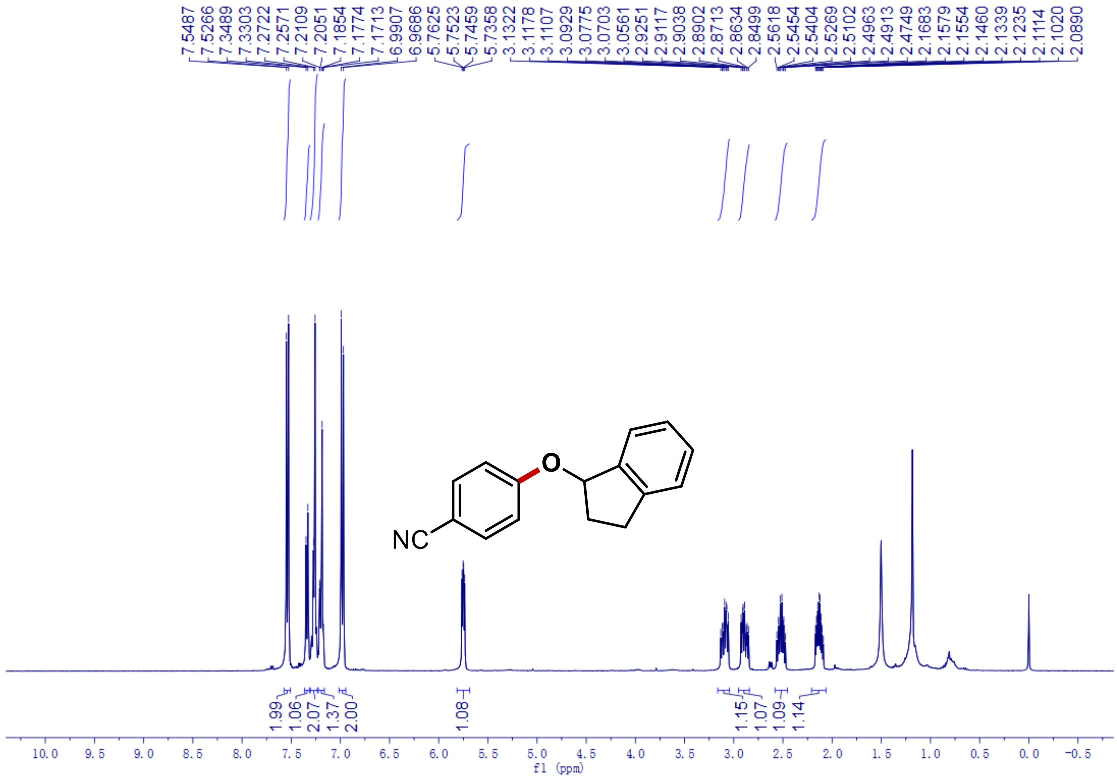

^{1}H NMR (400 MHz, $CDCl_3$) Spectrum

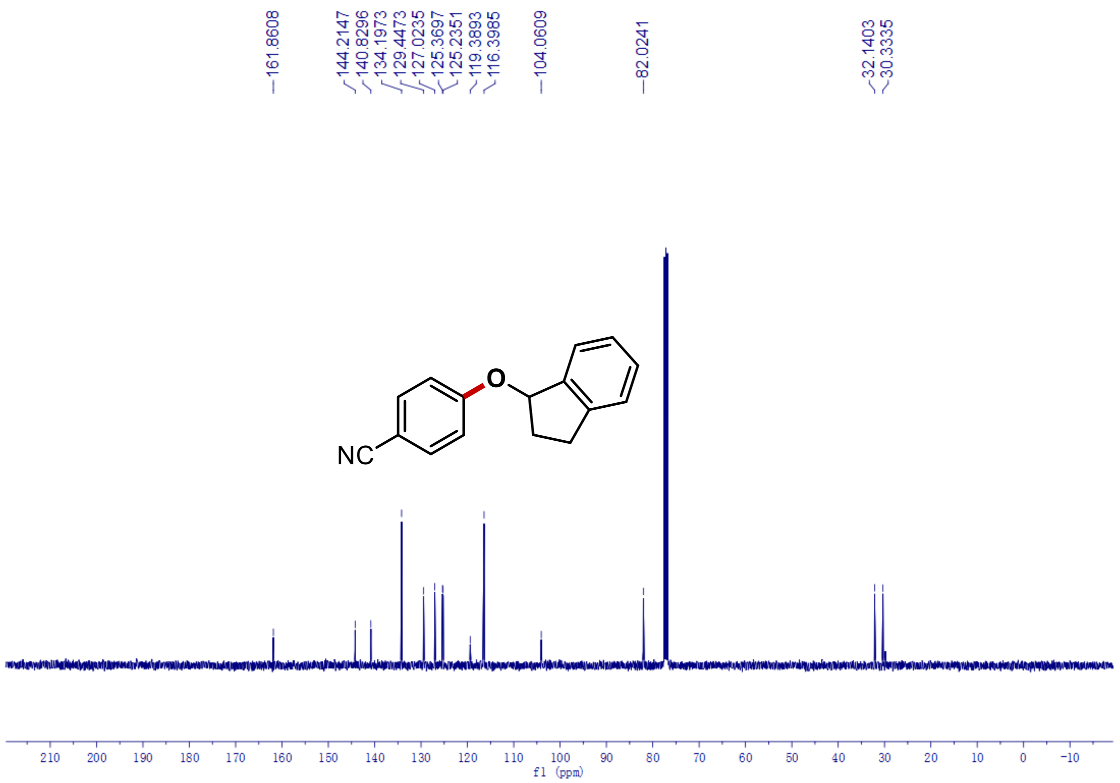

^{13}C NMR (100 MHz, $CDCl_3$) Spectrum

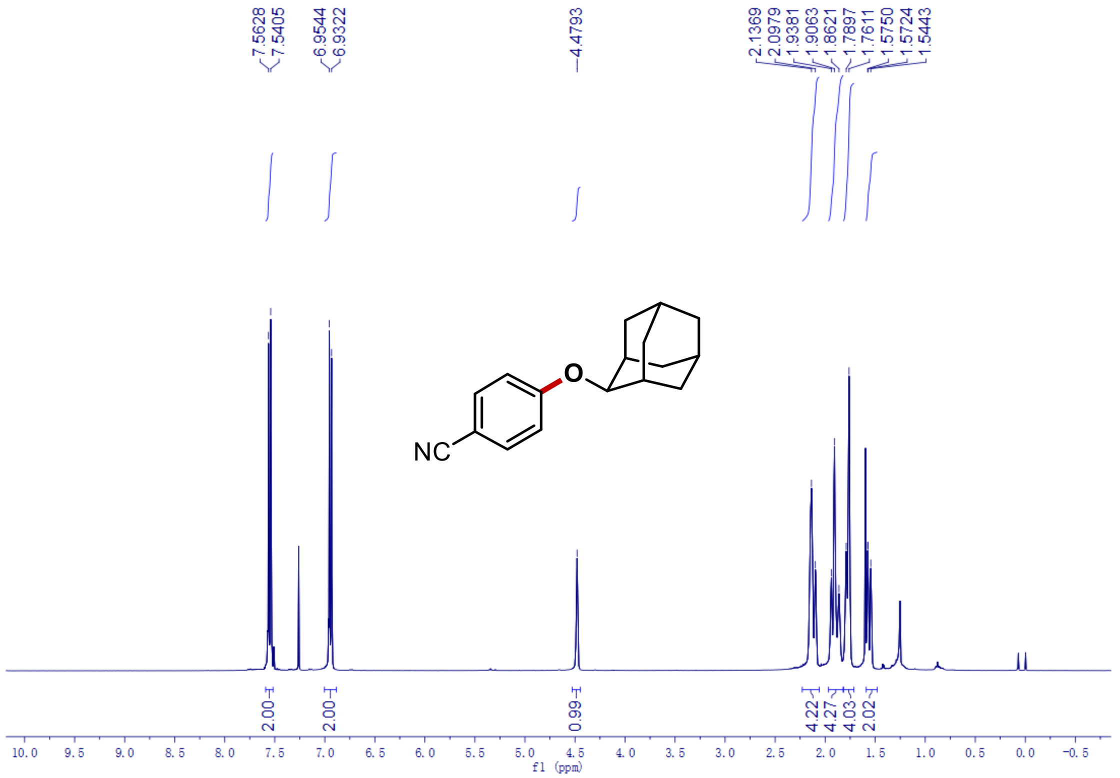

^{1}H NMR (400 MHz, $CDCl_3$) Spectrum

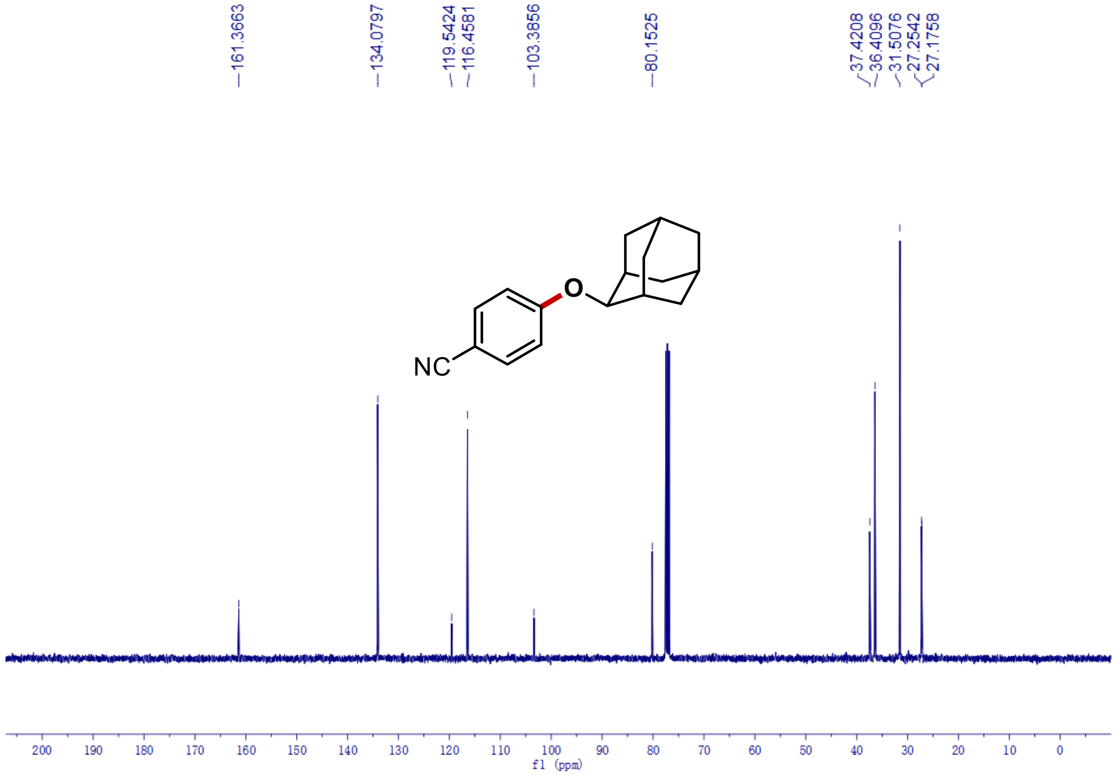

^{13}C NMR (100 MHz, $CDCl_3$) Spectrum

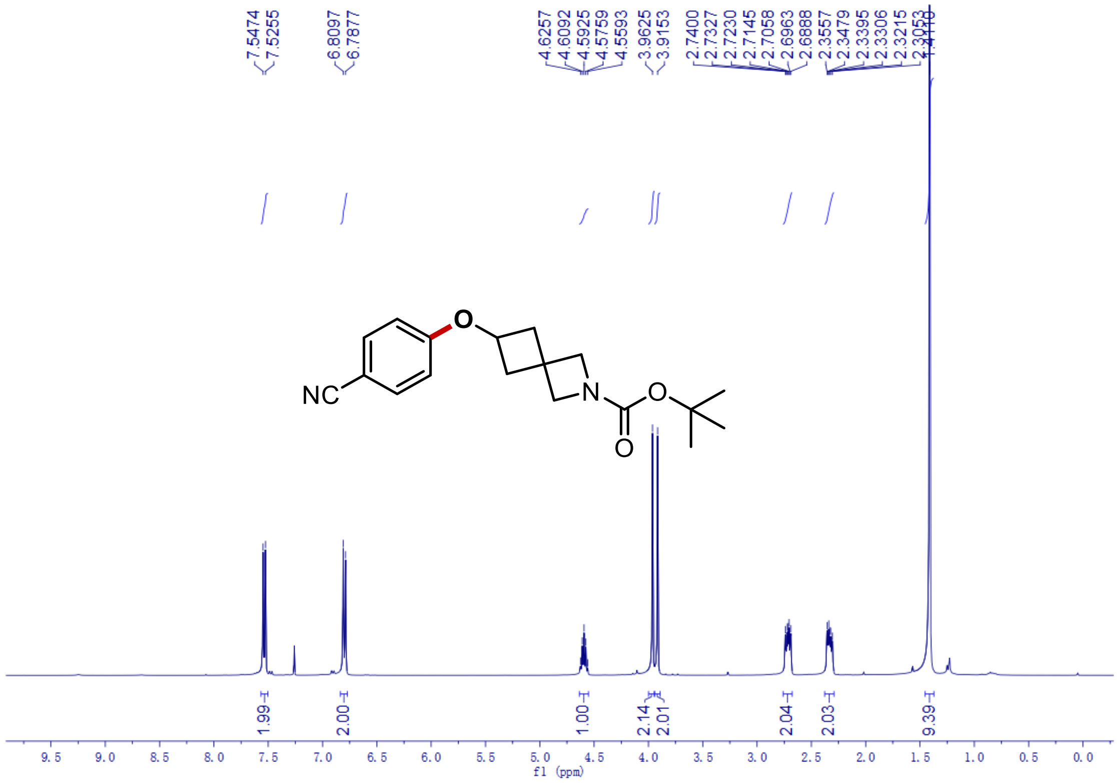

^{1}H NMR (400 MHz, $CDCl_3$) Spectrum

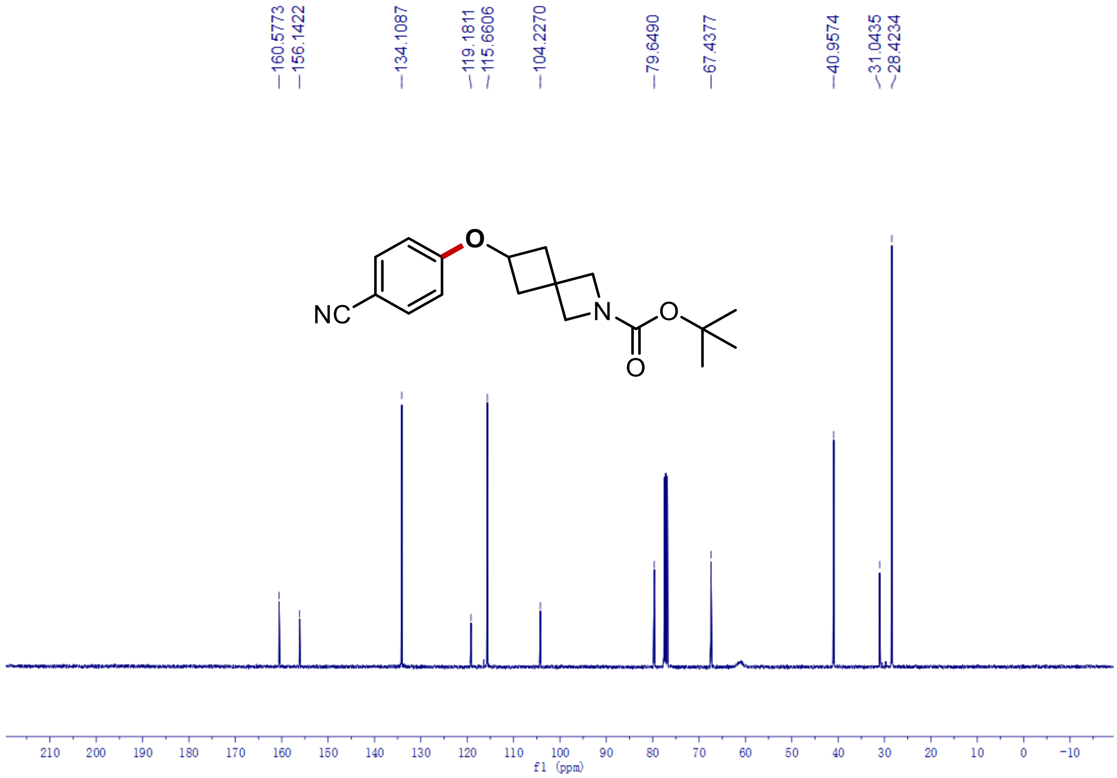

^{13}**C NMR** (100 MHz, $CDCl_3$) Spectrum

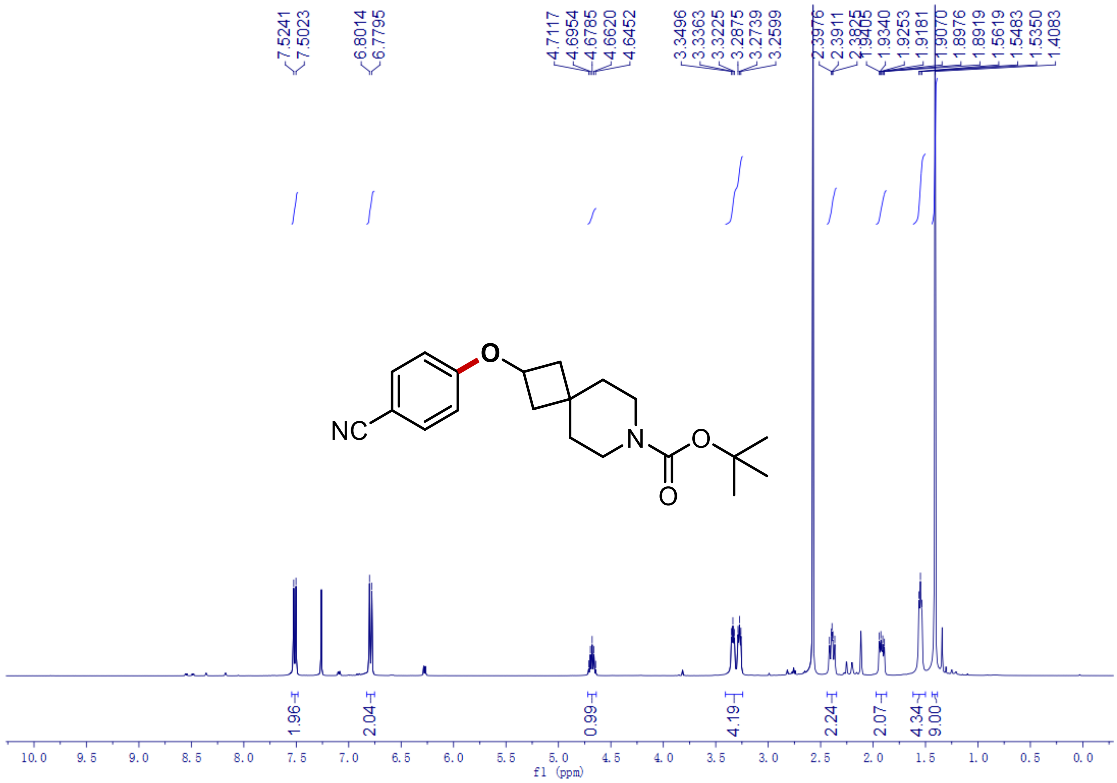

^{1}**H NMR** (400 MHz, $CDCl_3$) Spectrum

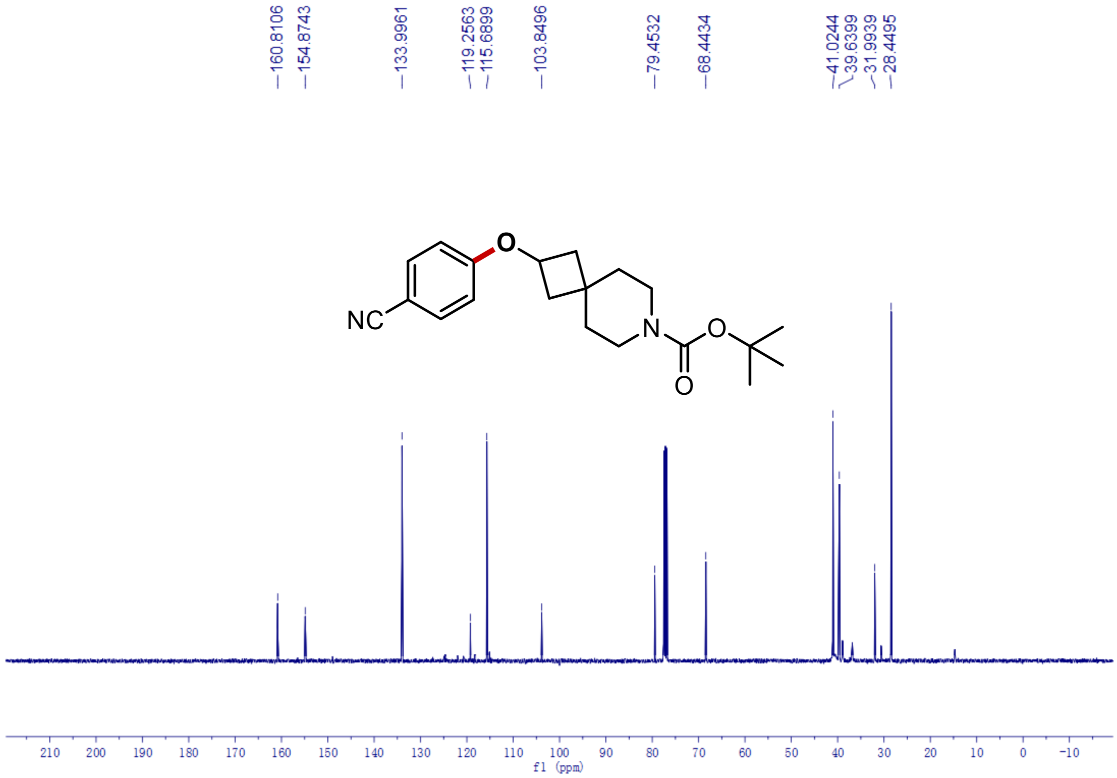

^{13}C NMR (100 MHz, $CDCl_3$) Spectrum

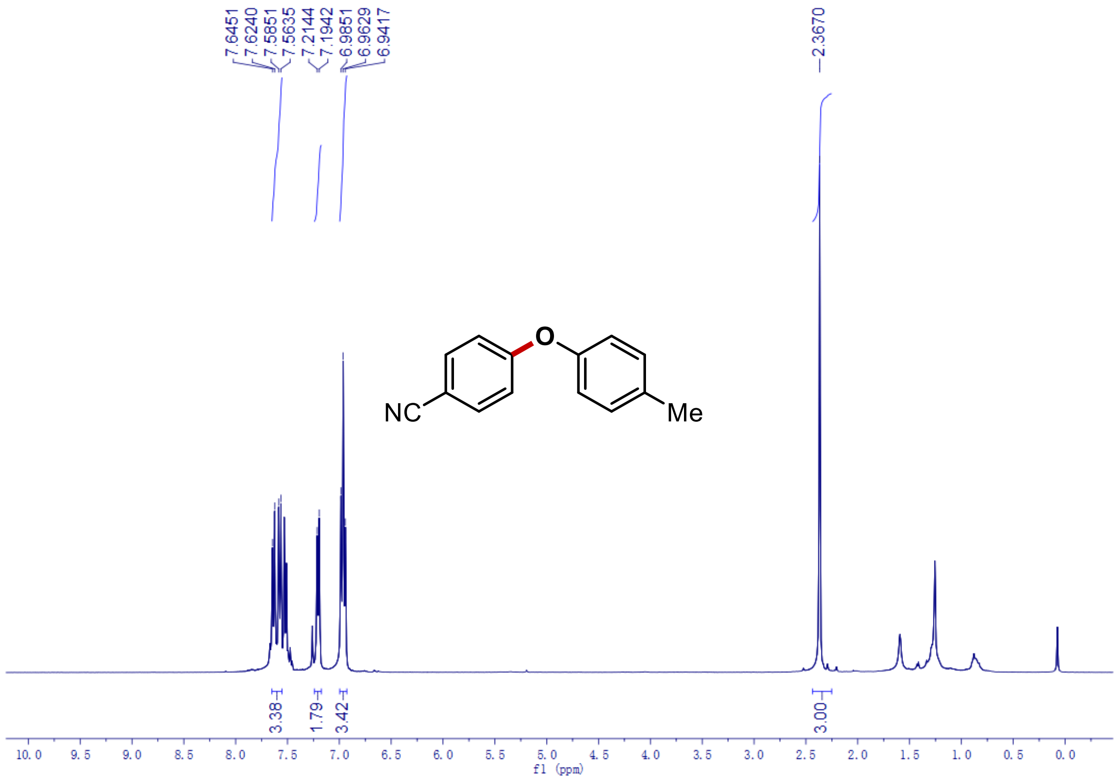

^{1}H NMR (400 MHz, $CDCl_3$) Spectrum

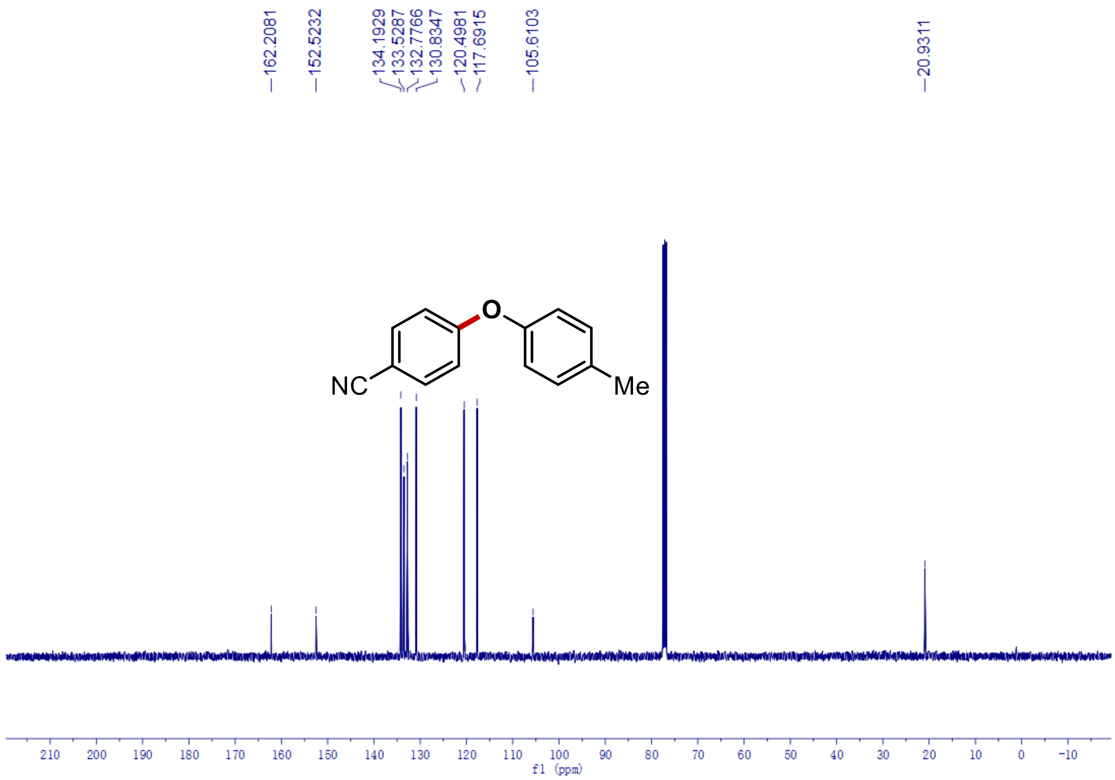

^{13}C NMR (100 MHz, $CDCl_3$) Spectrum

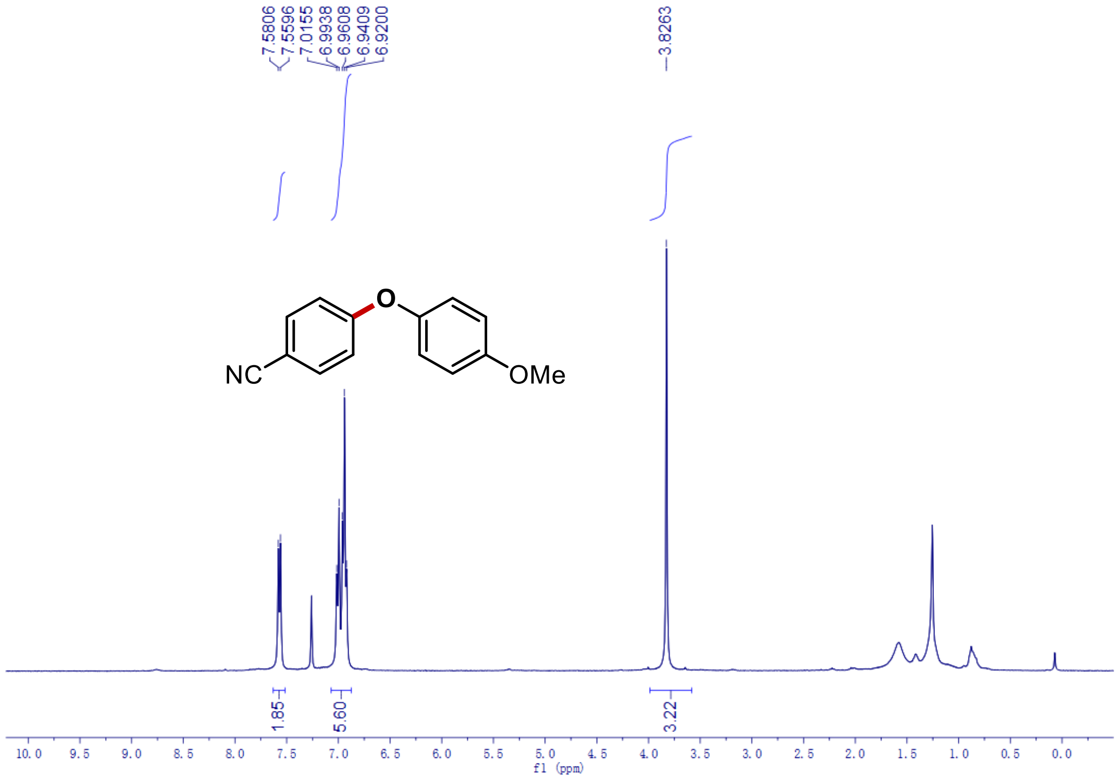

^{1}H NMR (400 MHz, $CDCl_3$) Spectrum

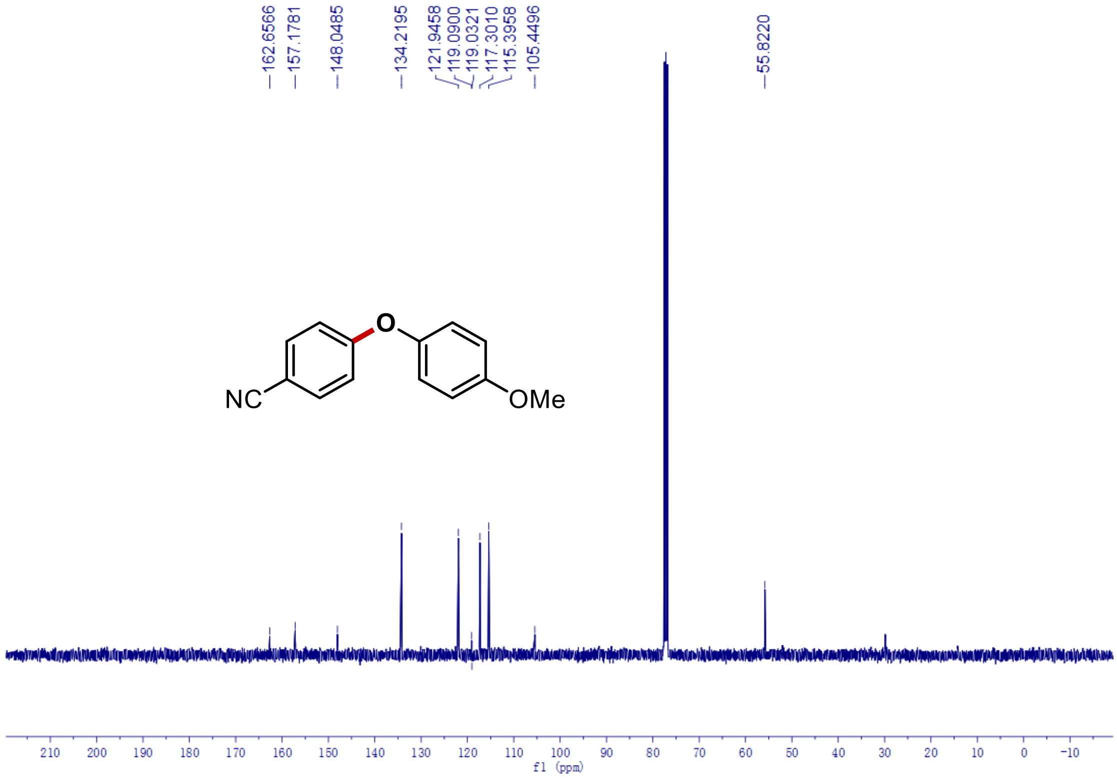

^{13}**C NMR** (100 MHz, $CDCl_3$) Spectrum

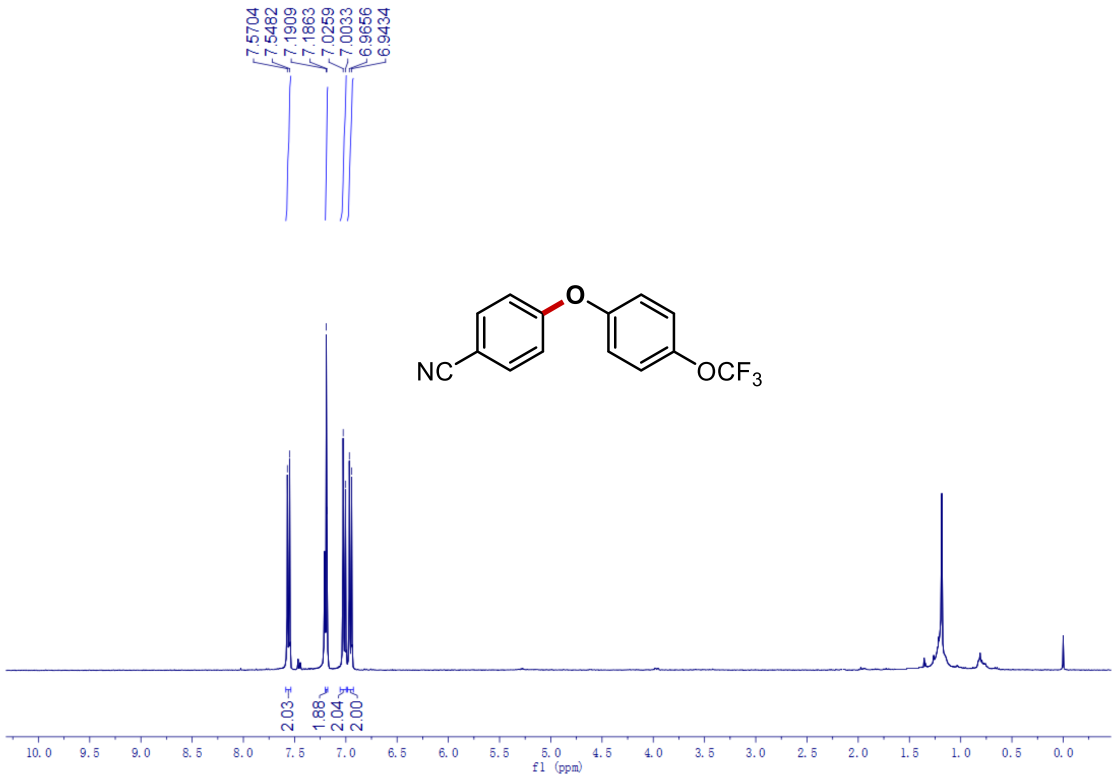

^{1}**H NMR** (400 MHz, $CDCl_3$) Spectrum

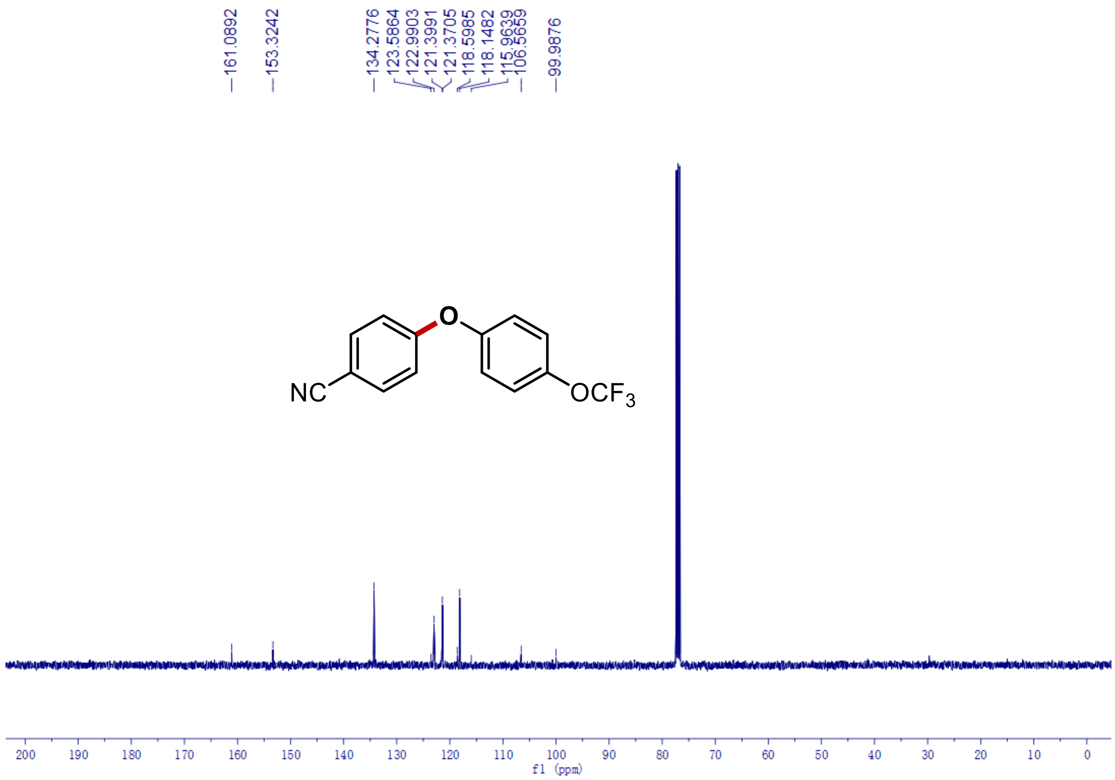

^{13}C NMR (100 MHz, $CDCl_3$) Spectrum

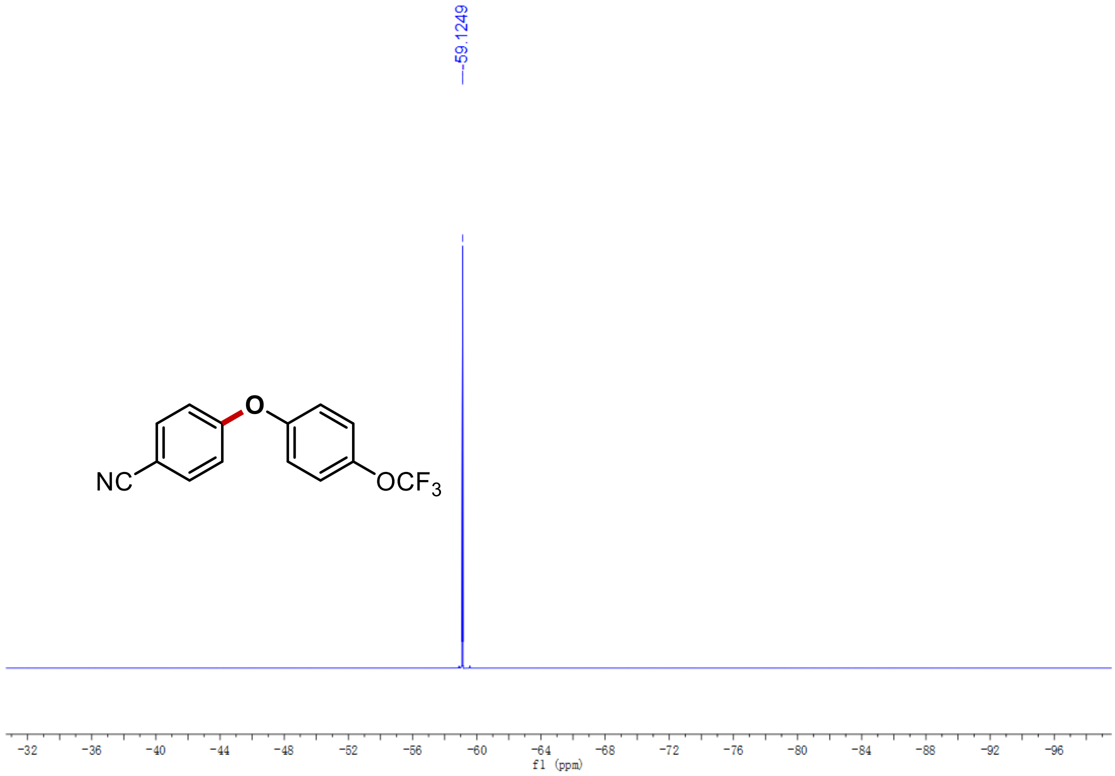

^{19}F NMR (376 MHz, $CDCl_3$) Spectrum

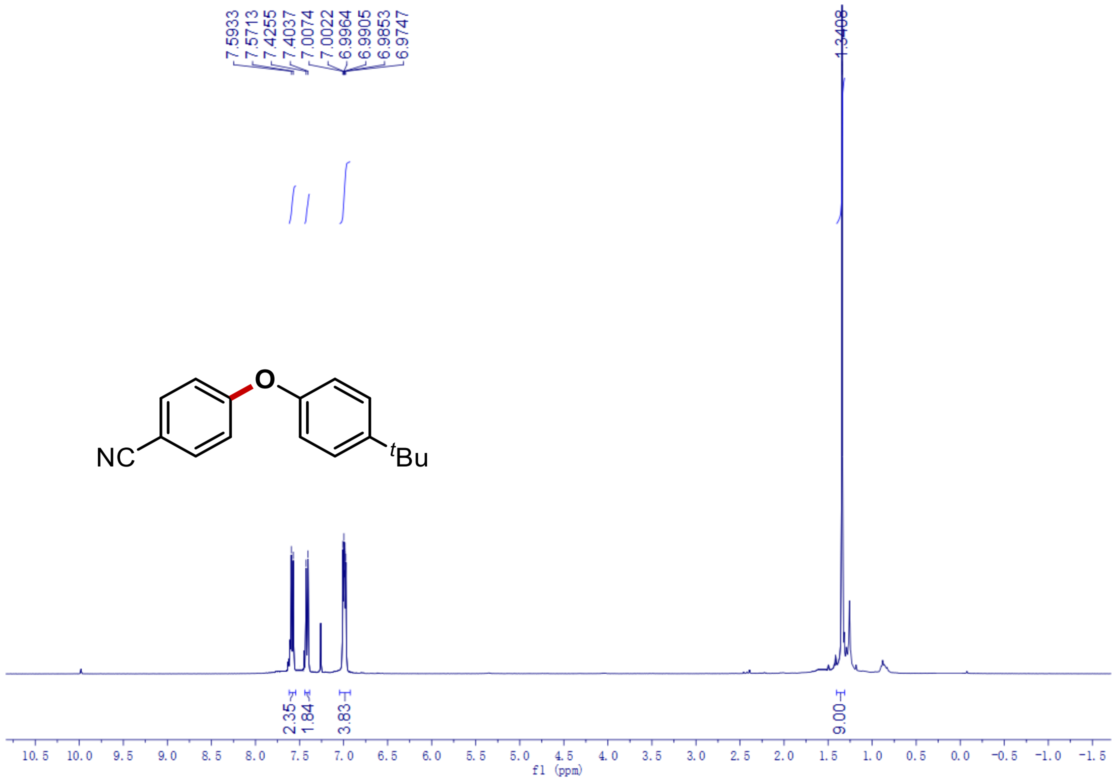

^{1}H NMR (400 MHz, $CDCl_3$) Spectrum

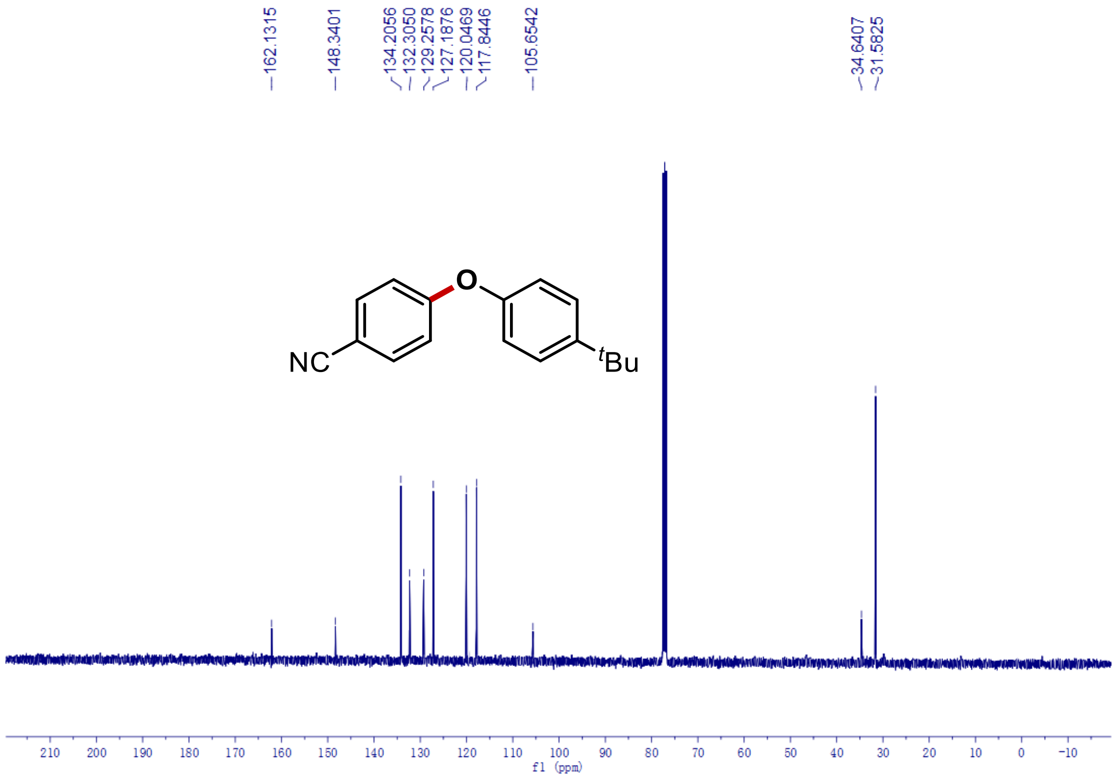

^{13}C NMR (100 MHz, $CDCl_3$) Spectrum

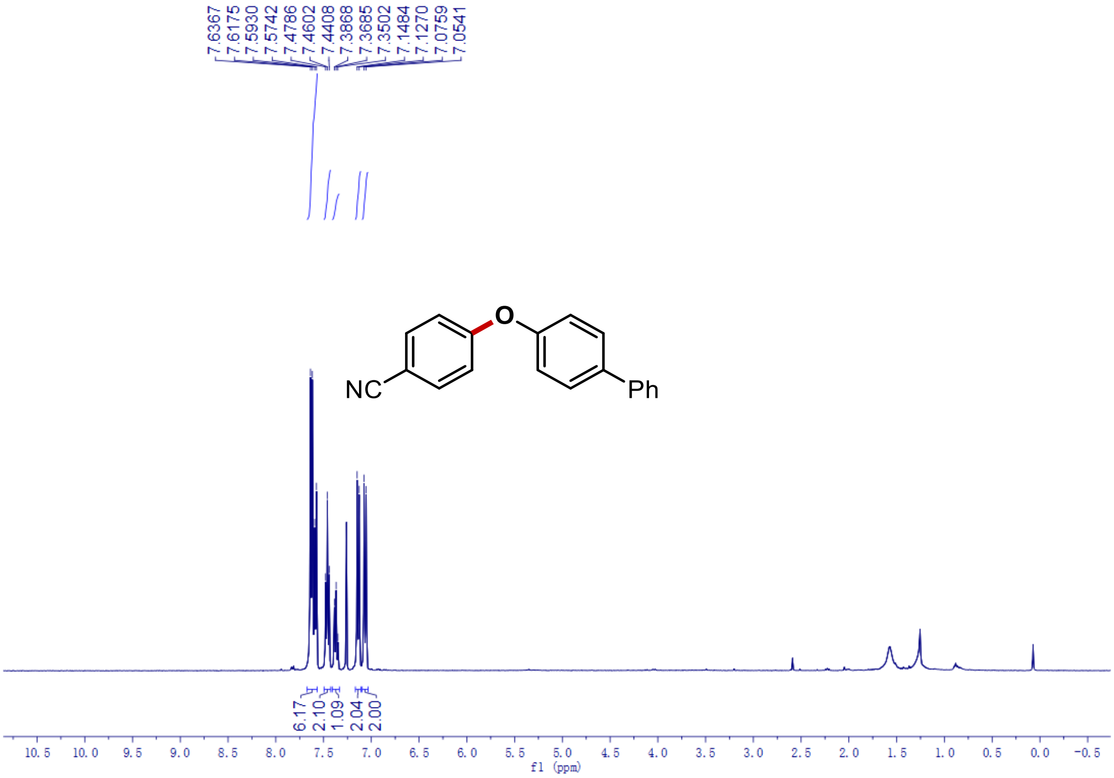

^{1}H NMR (400 MHz, $CDCl_3$) Spectrum

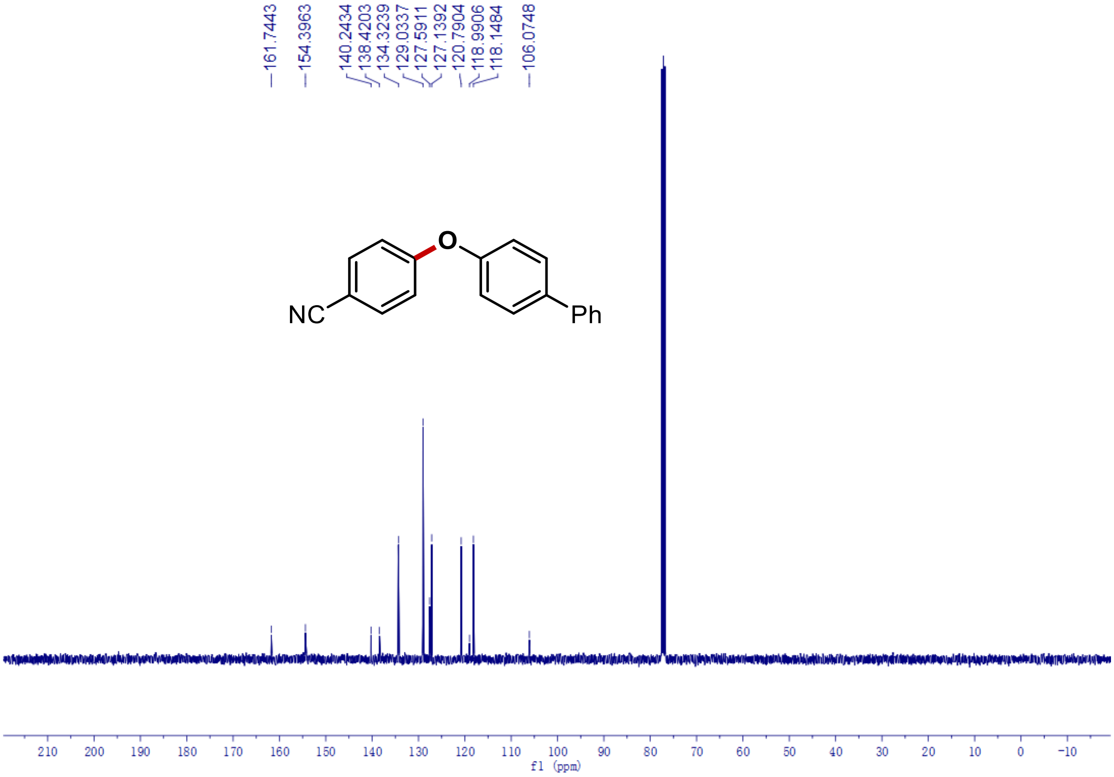

^{13}C NMR (100 MHz, $CDCl_3$) Spectrum

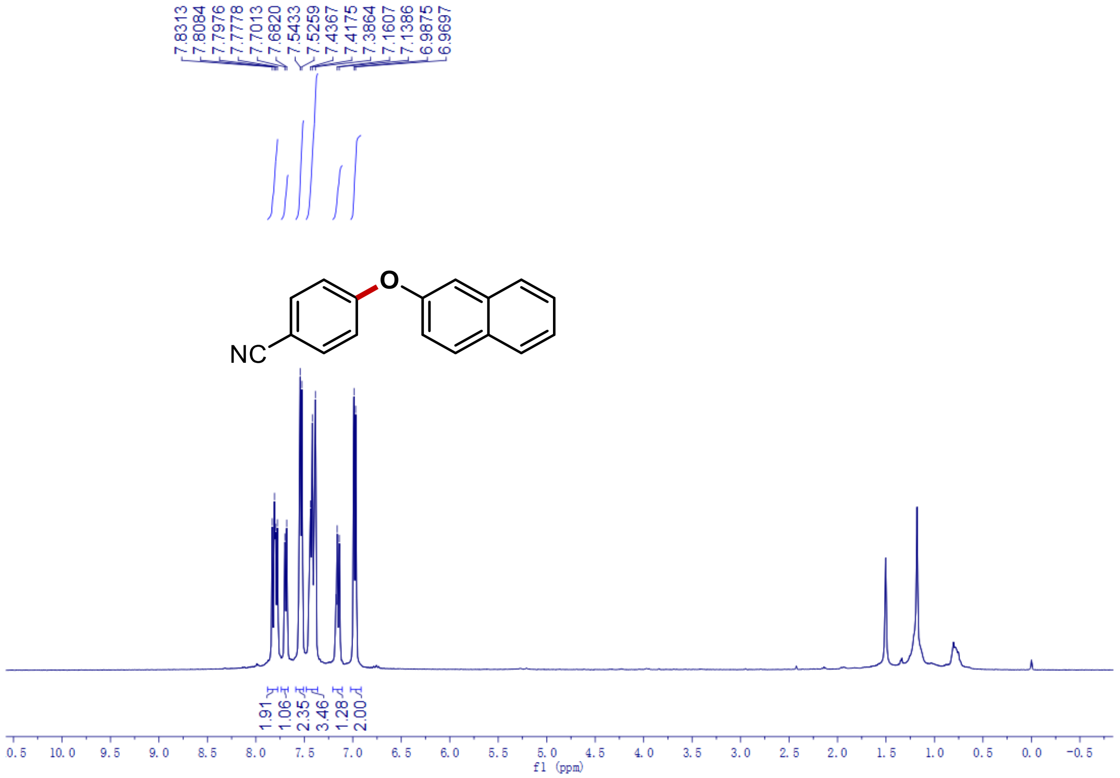

^{1}H NMR (400 MHz, $CDCl_3$) Spectrum

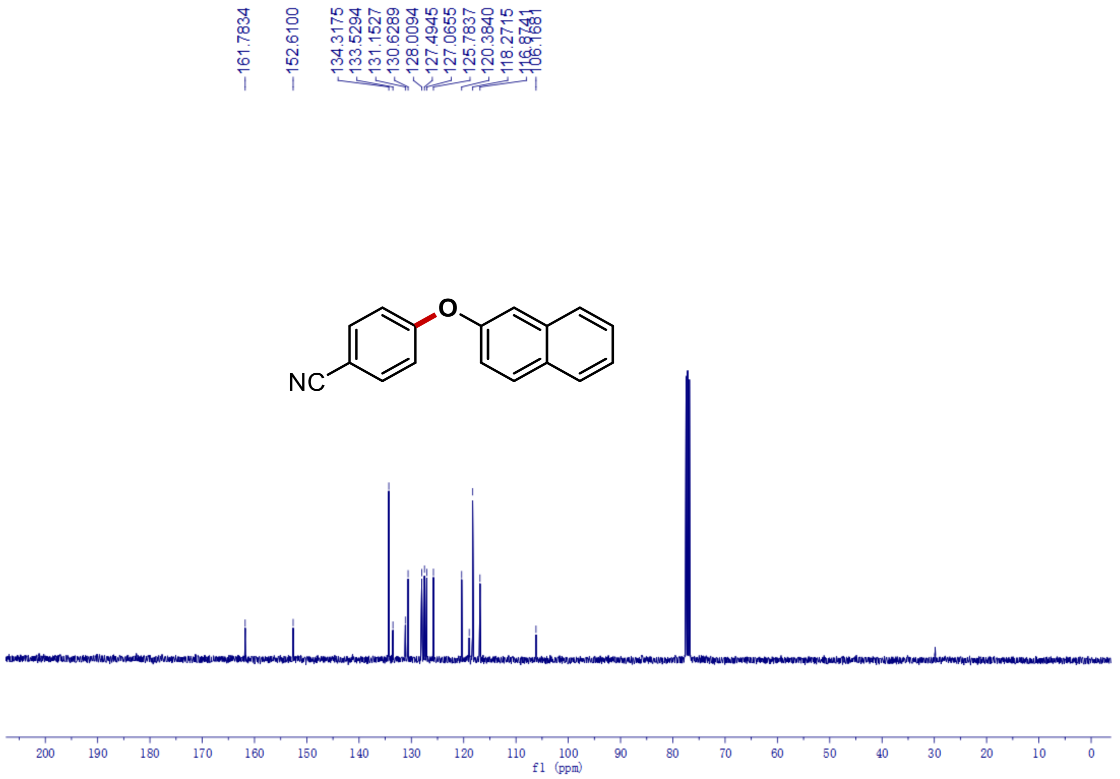

^{13}C NMR (100 MHz, $CDCl_3$) Spectrum

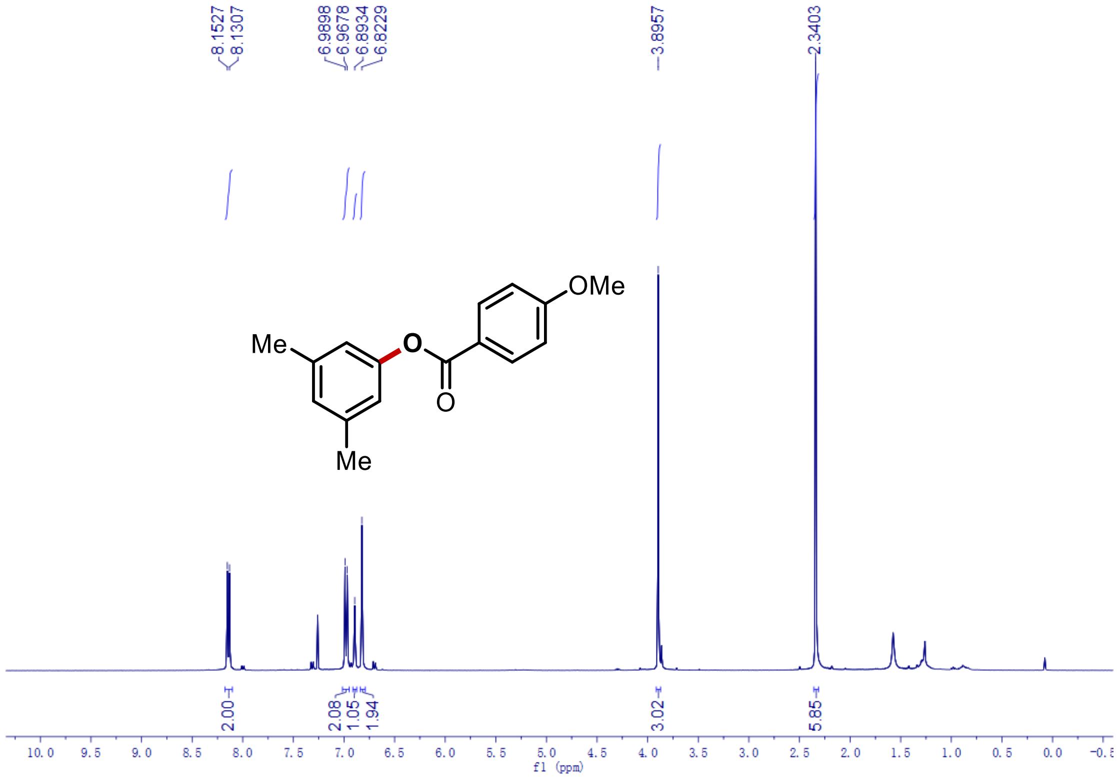

^{1}H NMR (400 MHz, $CDCl_3$) Spectrum

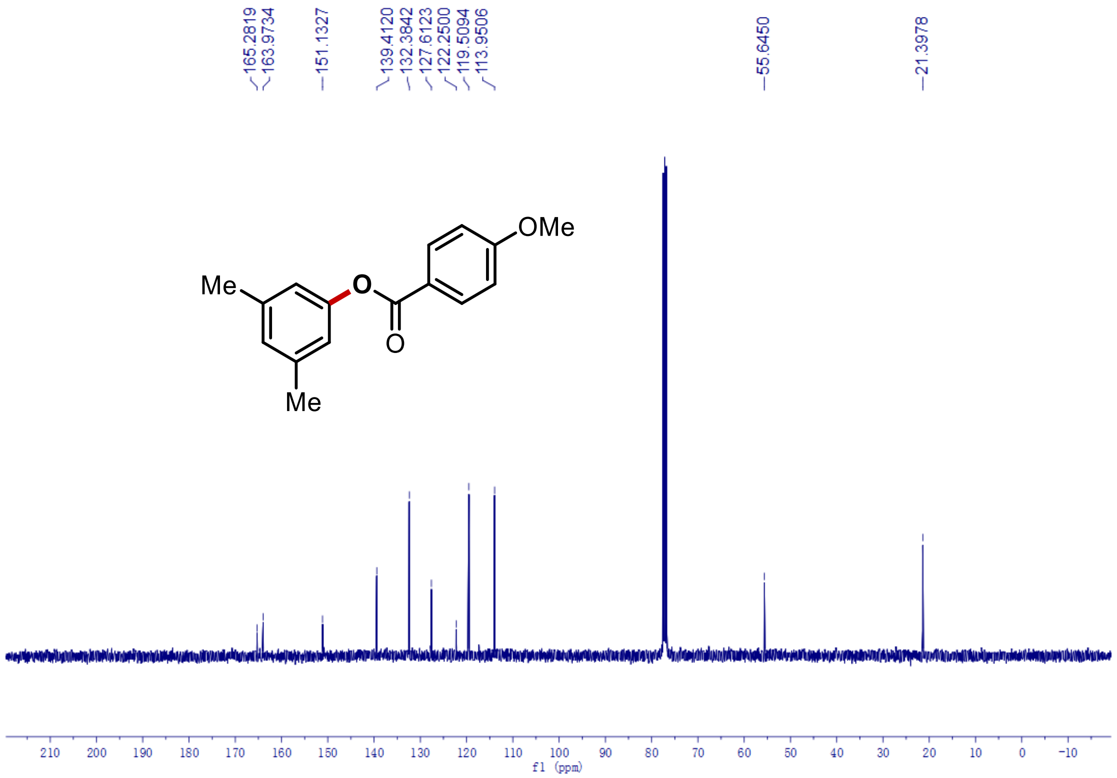

^{13}C NMR (100 MHz, $CDCl_3$) Spectrum

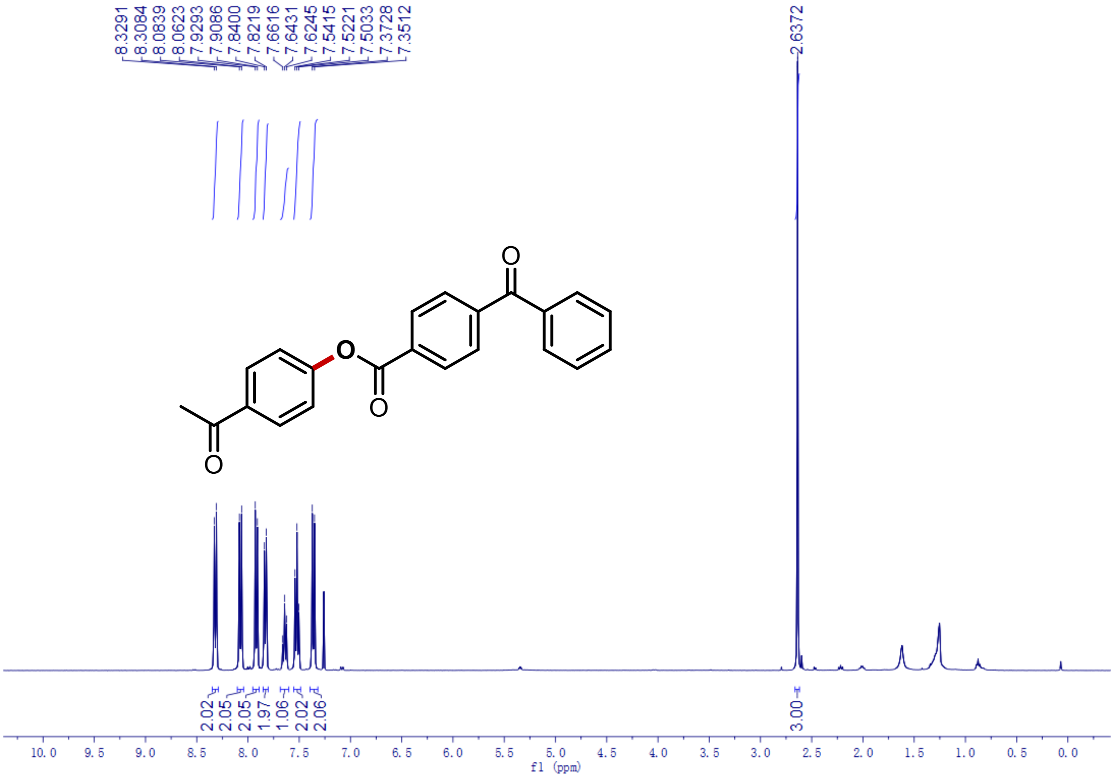

^{1}H NMR (400 MHz, $CDCl_3$) Spectrum

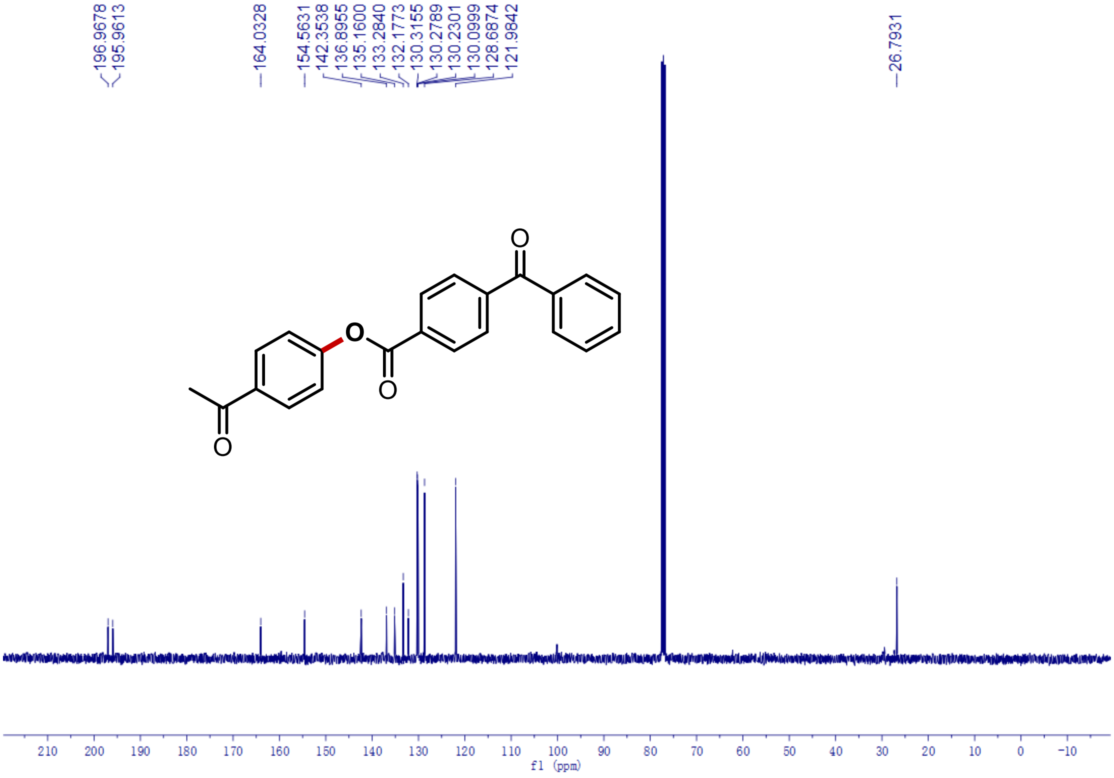

^{13}C NMR (100 MHz, $CDCl_3$) Spectrum

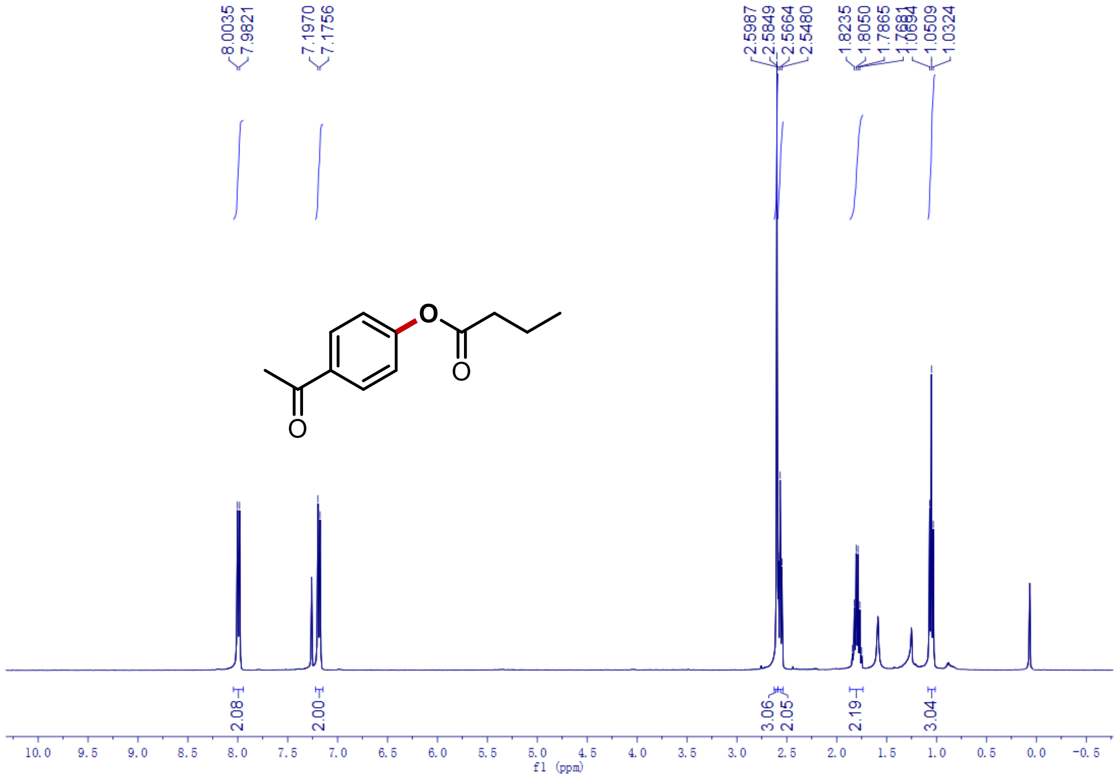

^{1}**H NMR** (400 MHz, $CDCl_3$) Spectrum

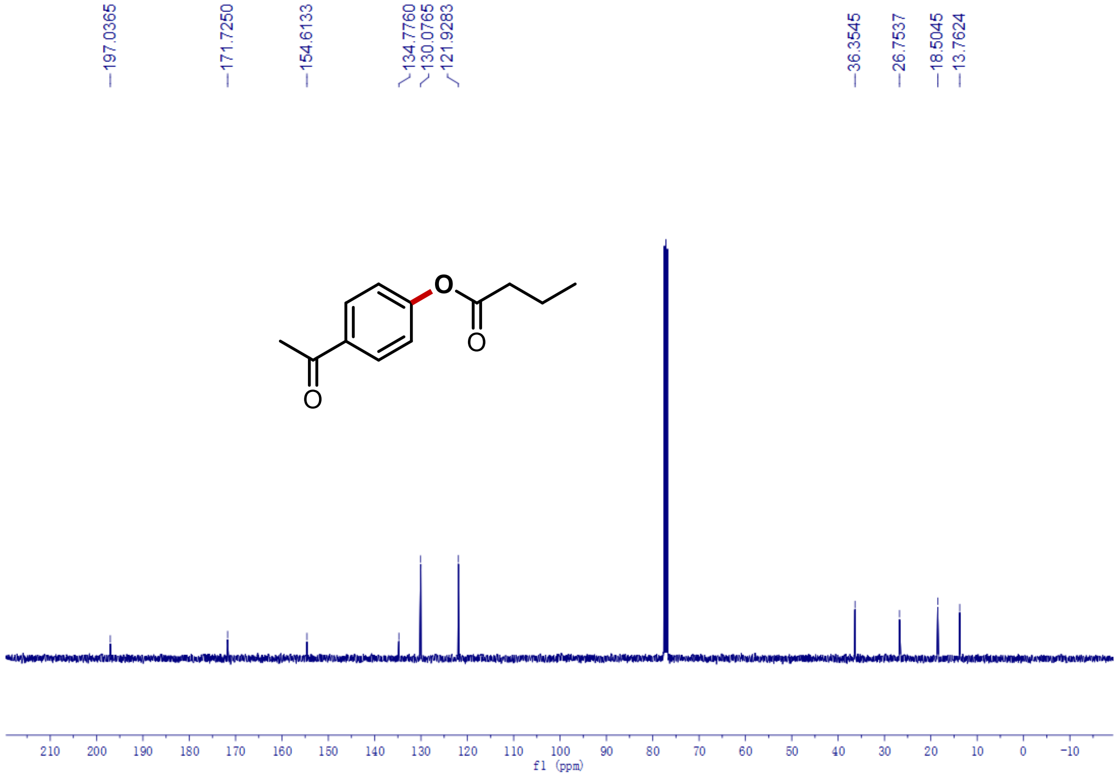

^{13}**C NMR** (100 MHz, $CDCl_3$) Spectrum

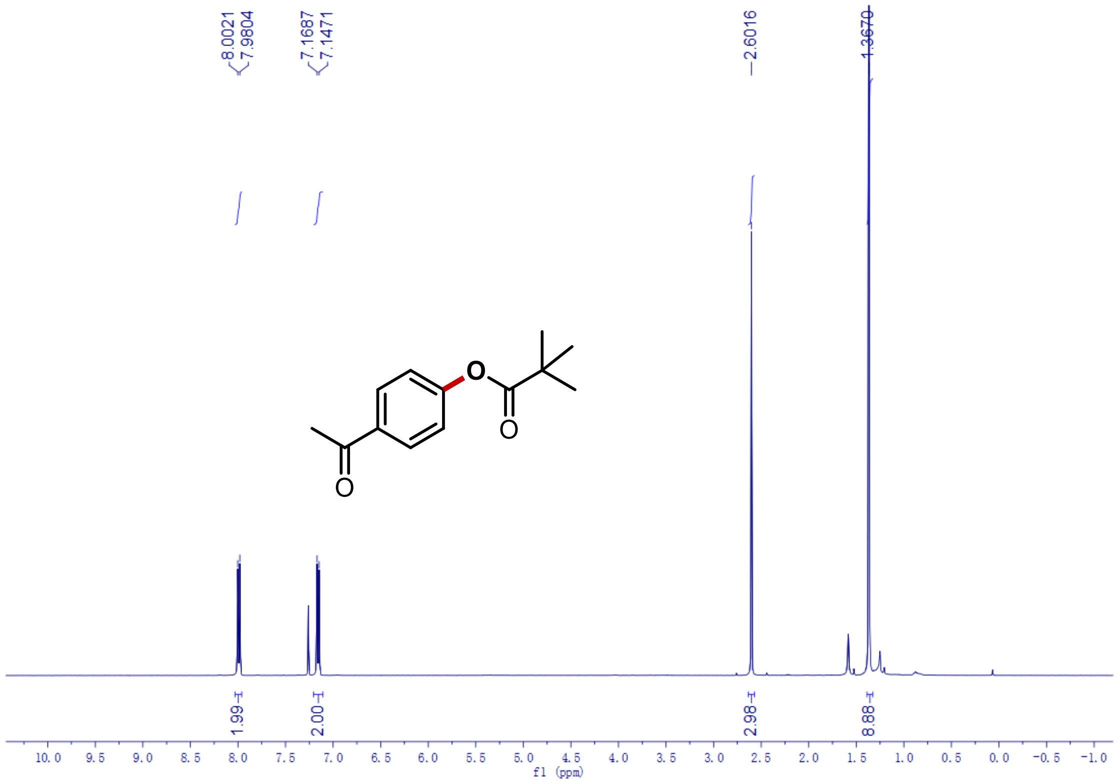

¹H NMR (400 MHz, $CDCl_3$) Spectrum

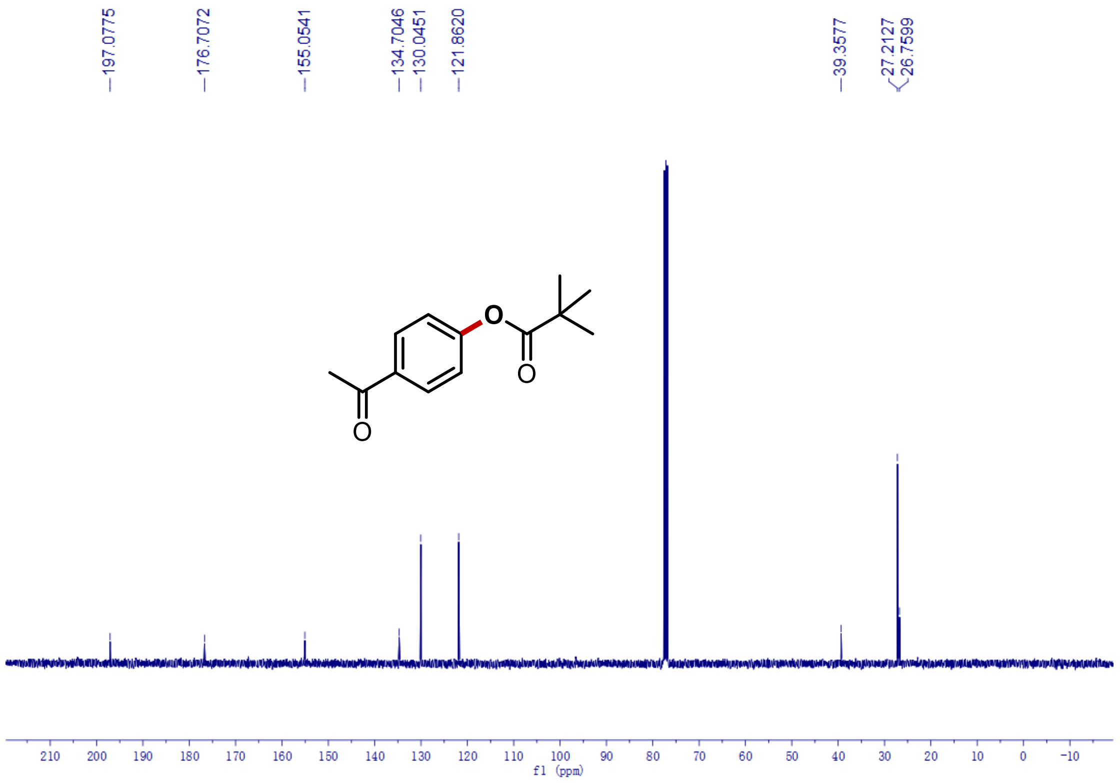

¹³C NMR (100 MHz, $CDCl_3$) Spectrum

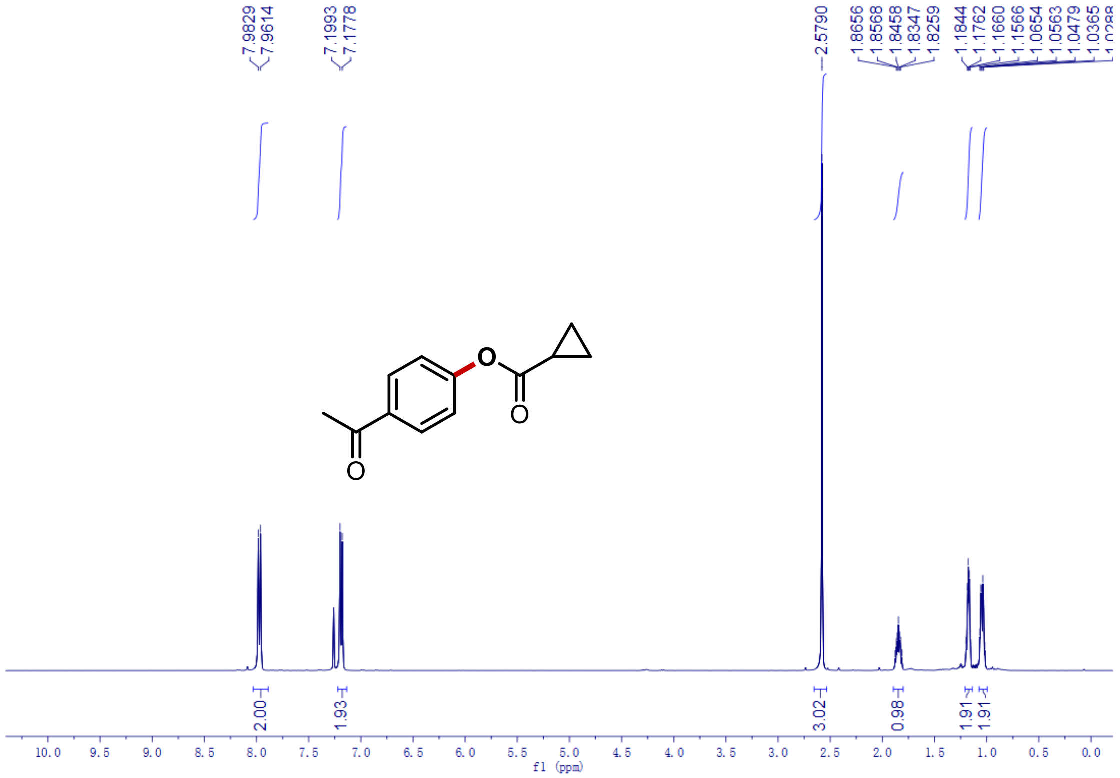

^{1}**H NMR** (400 MHz, $CDCl_3$) Spectrum

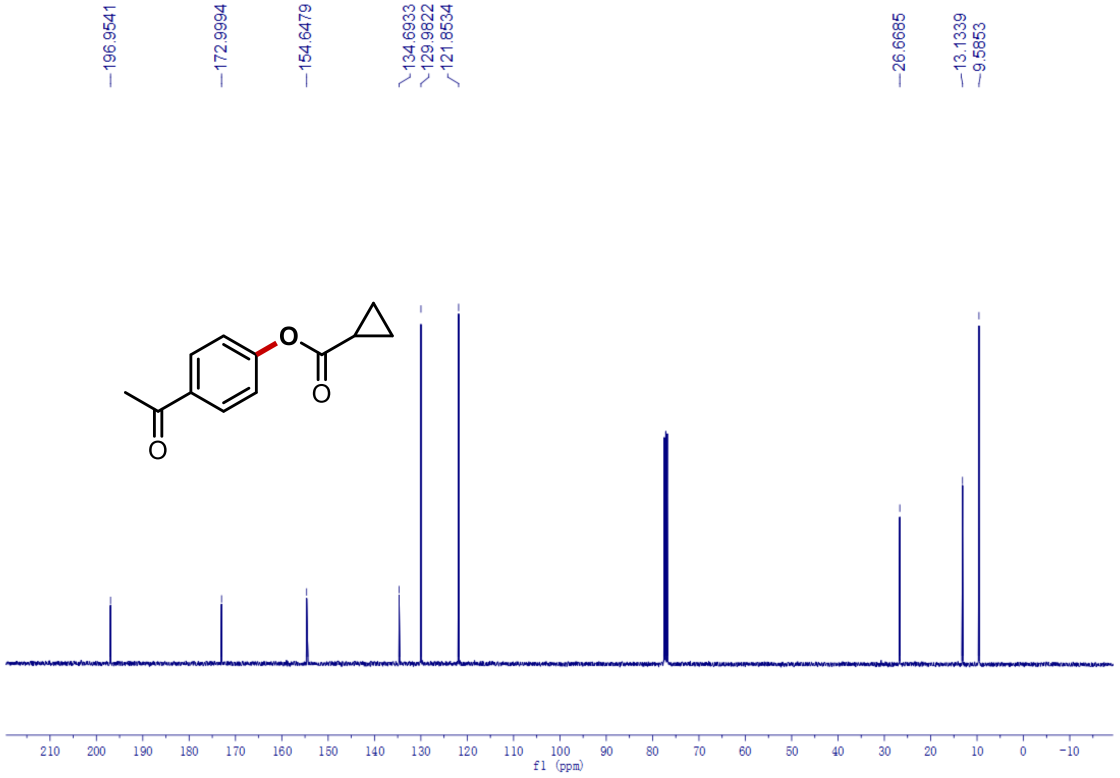

^{13}**C NMR** (100 MHz, $CDCl_3$) Spectrum

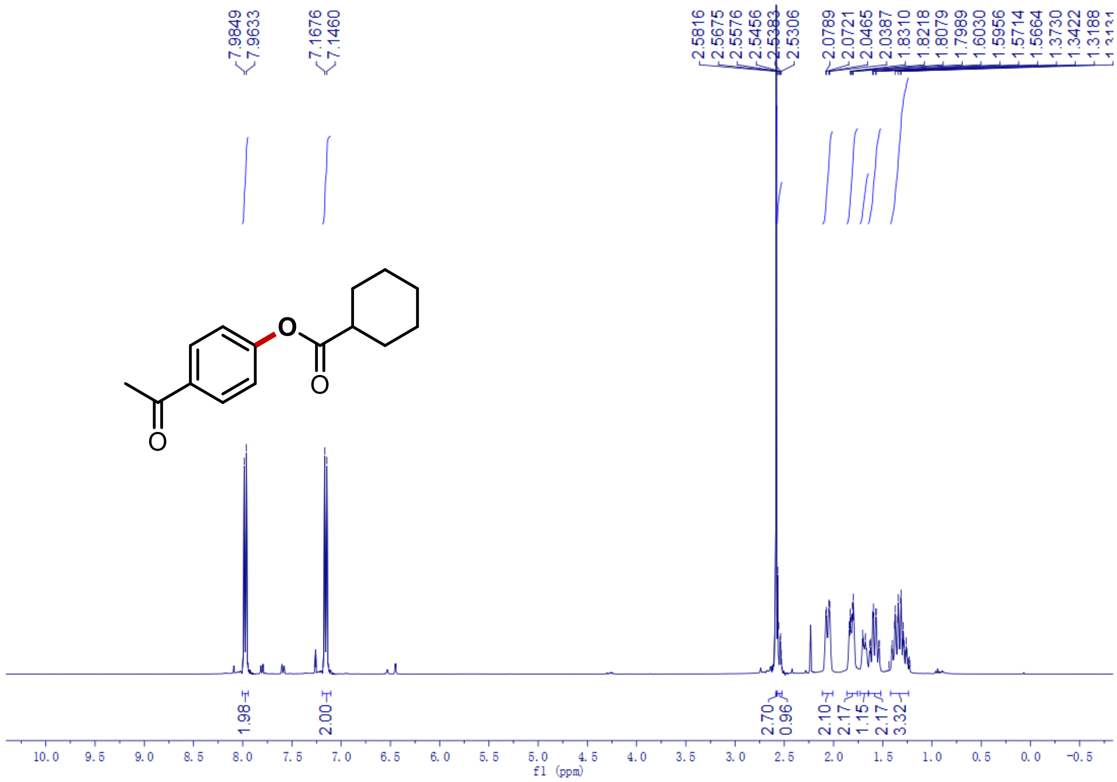

^{1}H NMR (400 MHz, $CDCl_3$) Spectrum

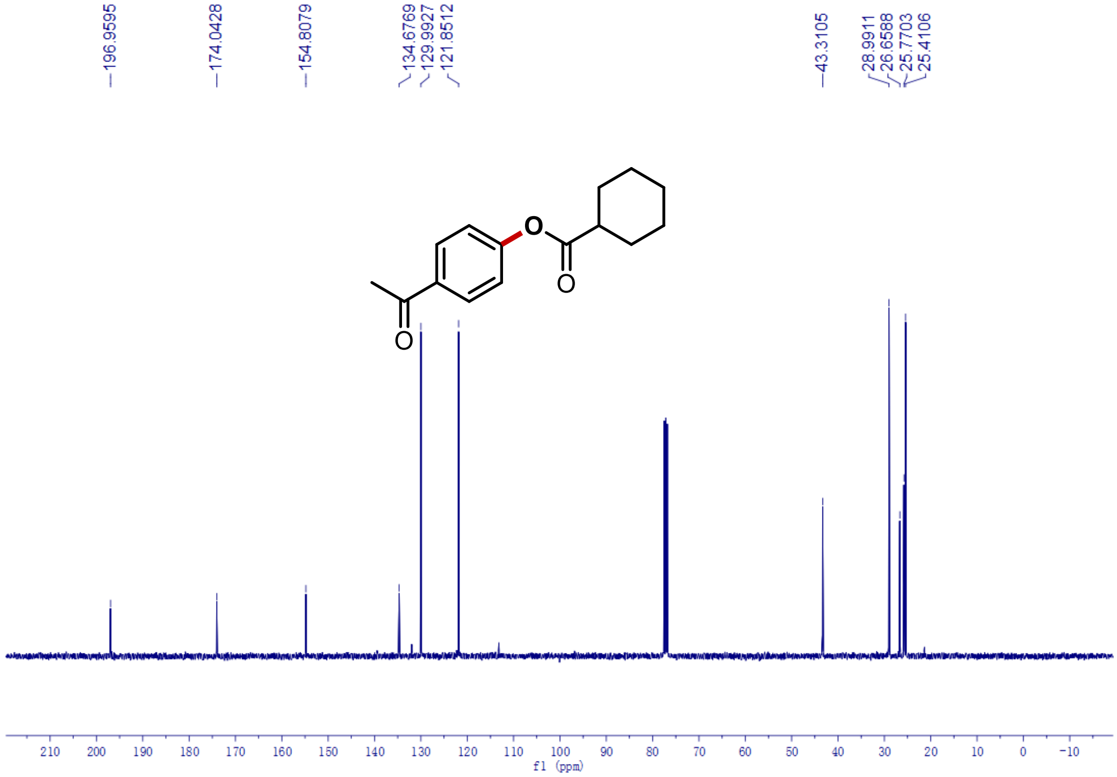

^{13}C NMR (100 MHz, $CDCl_3$) Spectrum

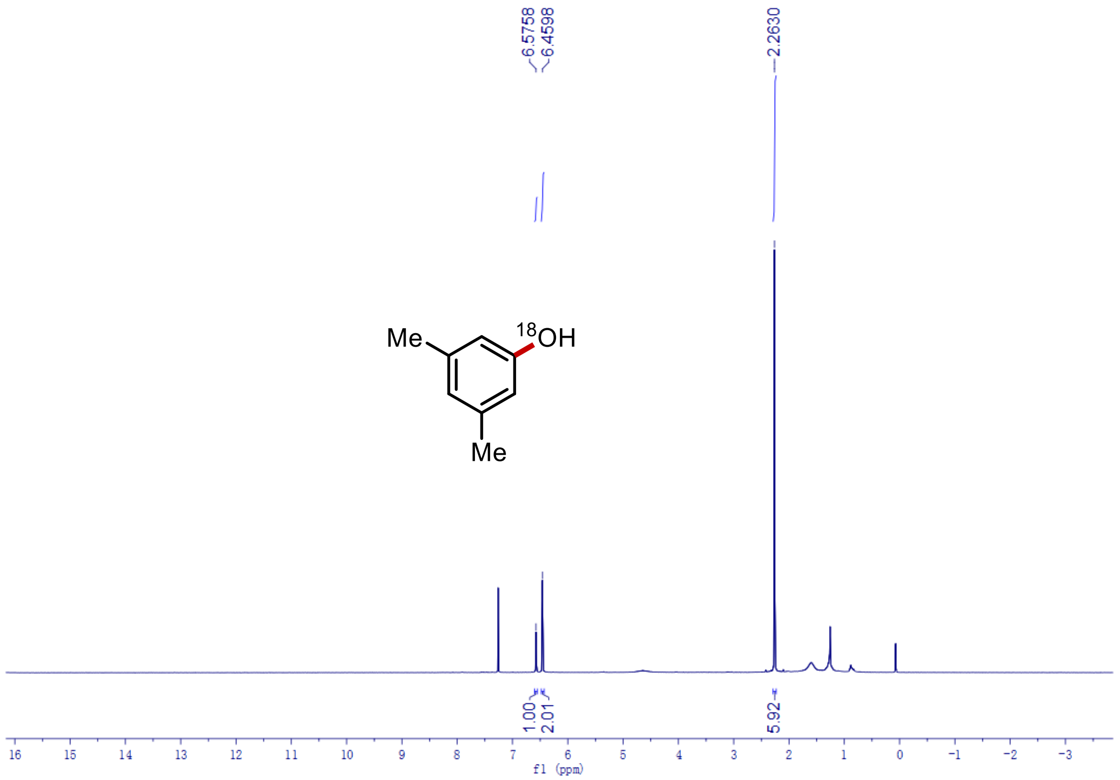

^{1}H NMR (400 MHz, $CDCl_3$) Spectrum

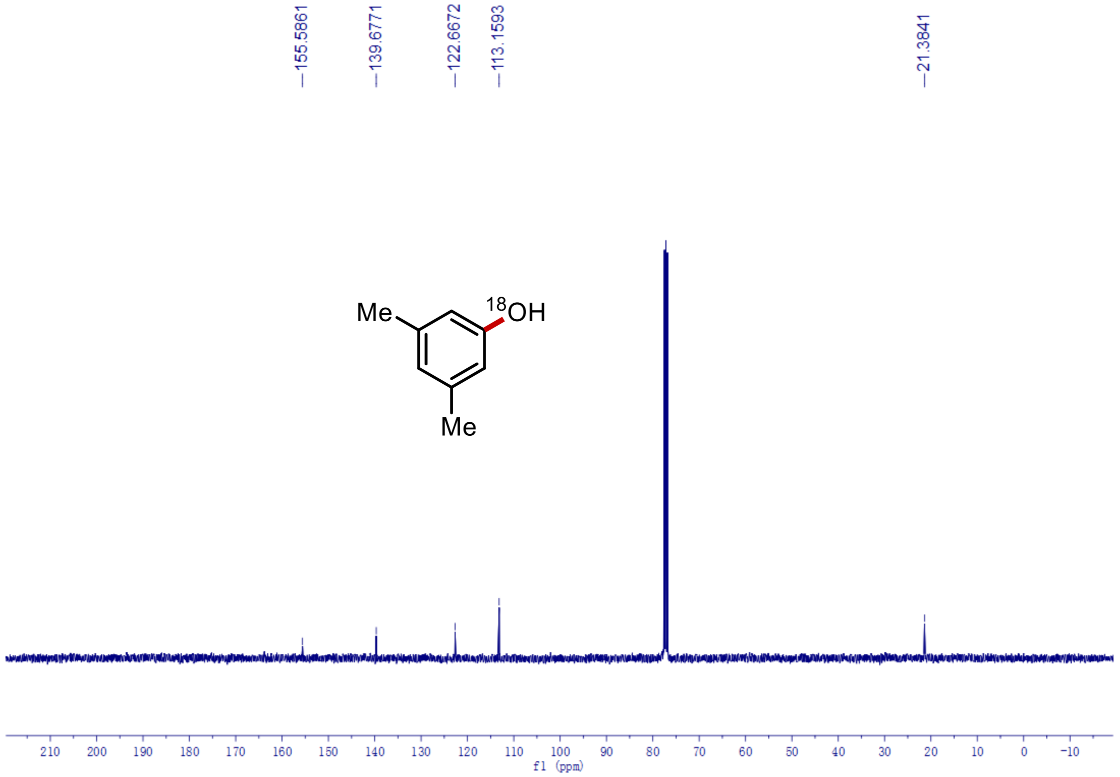

^{13}C NMR (100 MHz, $CDCl_3$) Spectrum

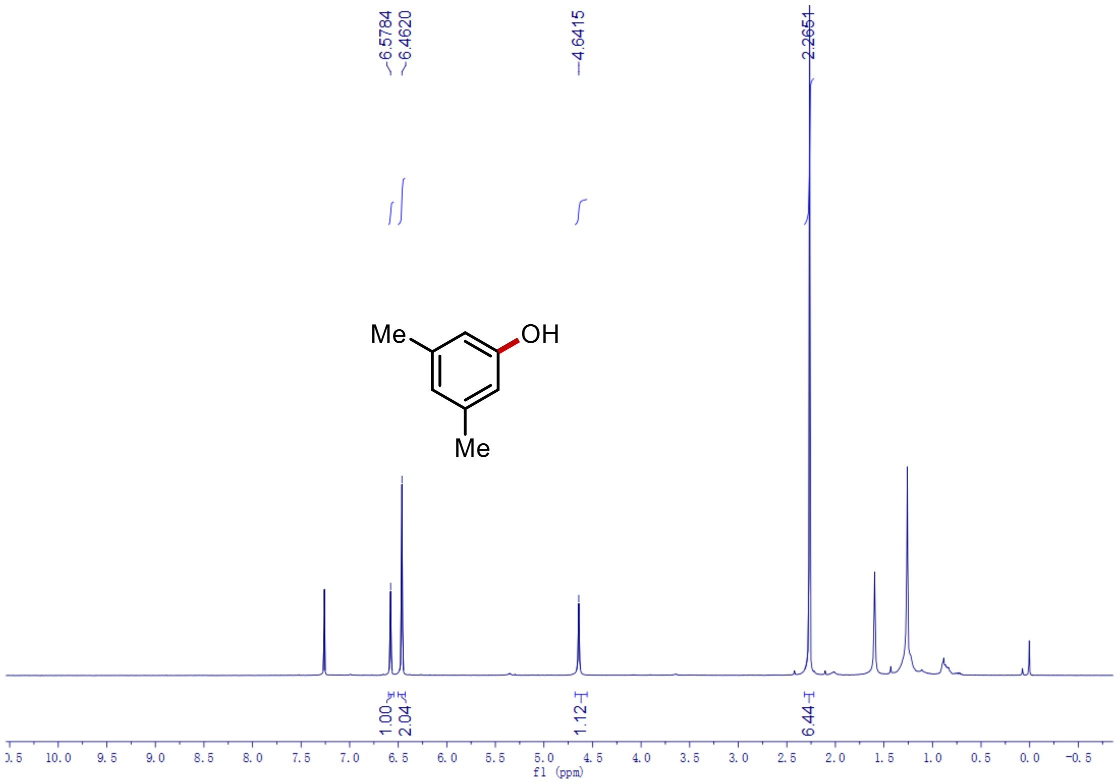

^{1}H NMR (400 MHz, $CDCl_3$) Spectrum

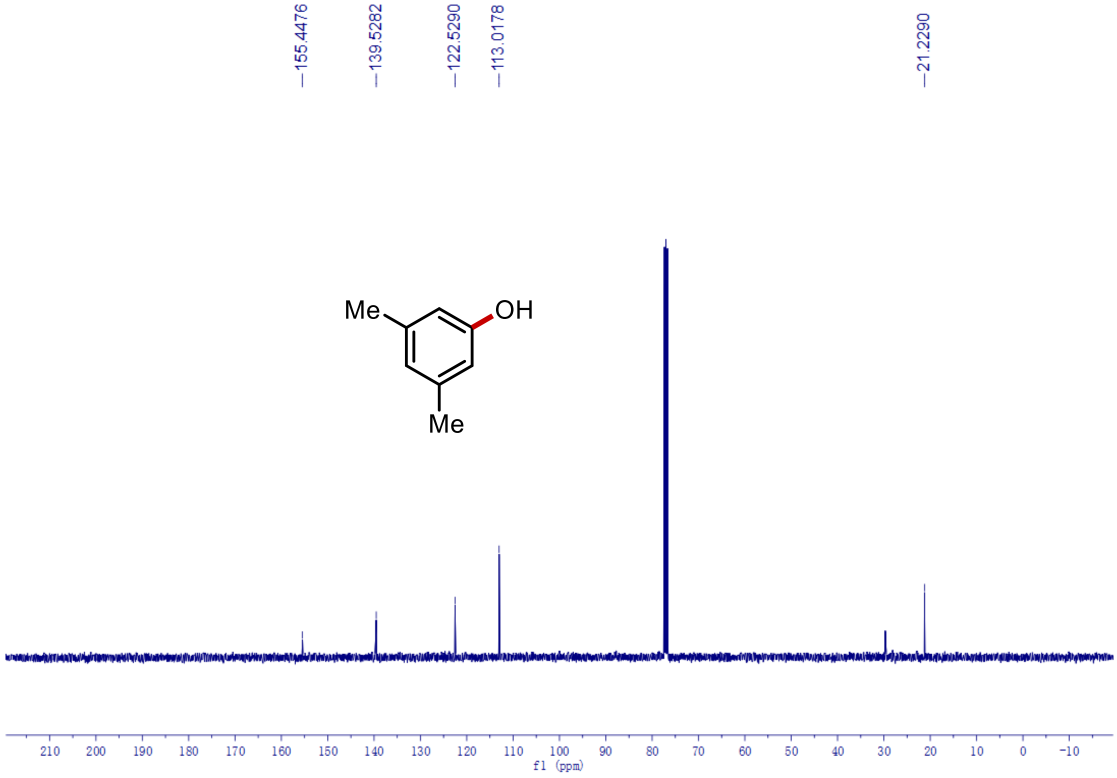

^{13}C NMR (100 MHz, $CDCl_3$) Spectrum

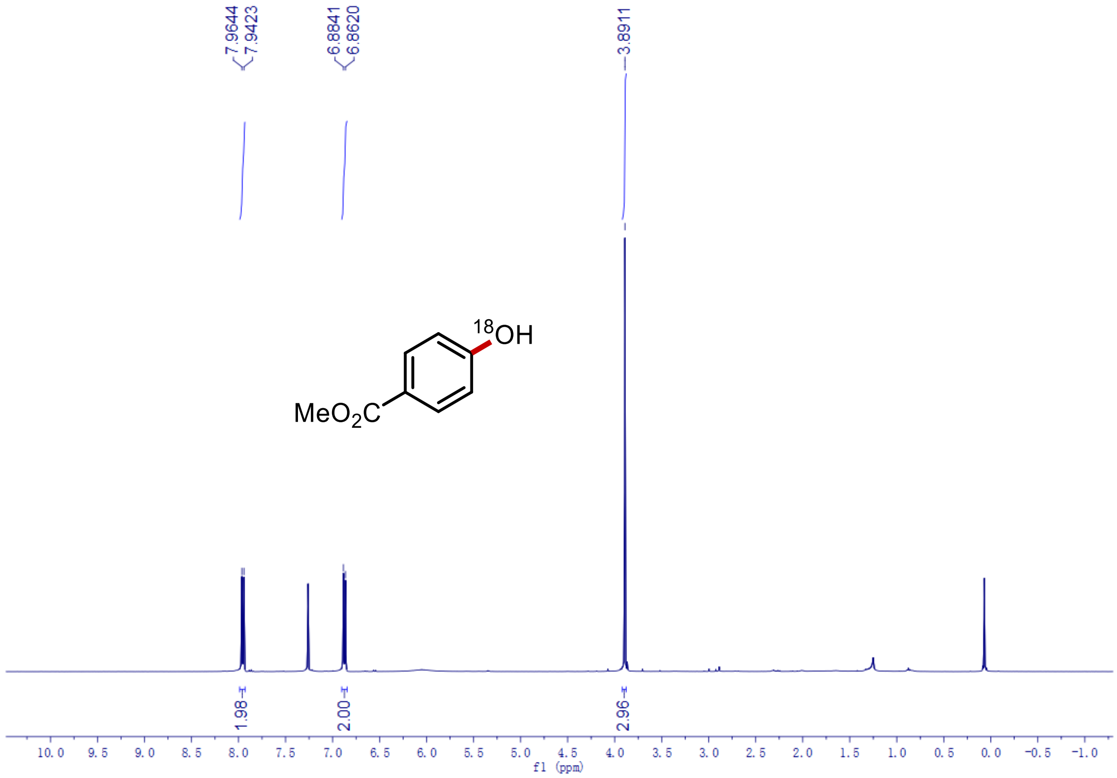

^{1}H NMR (400 MHz, $CDCl_3$) Spectrum

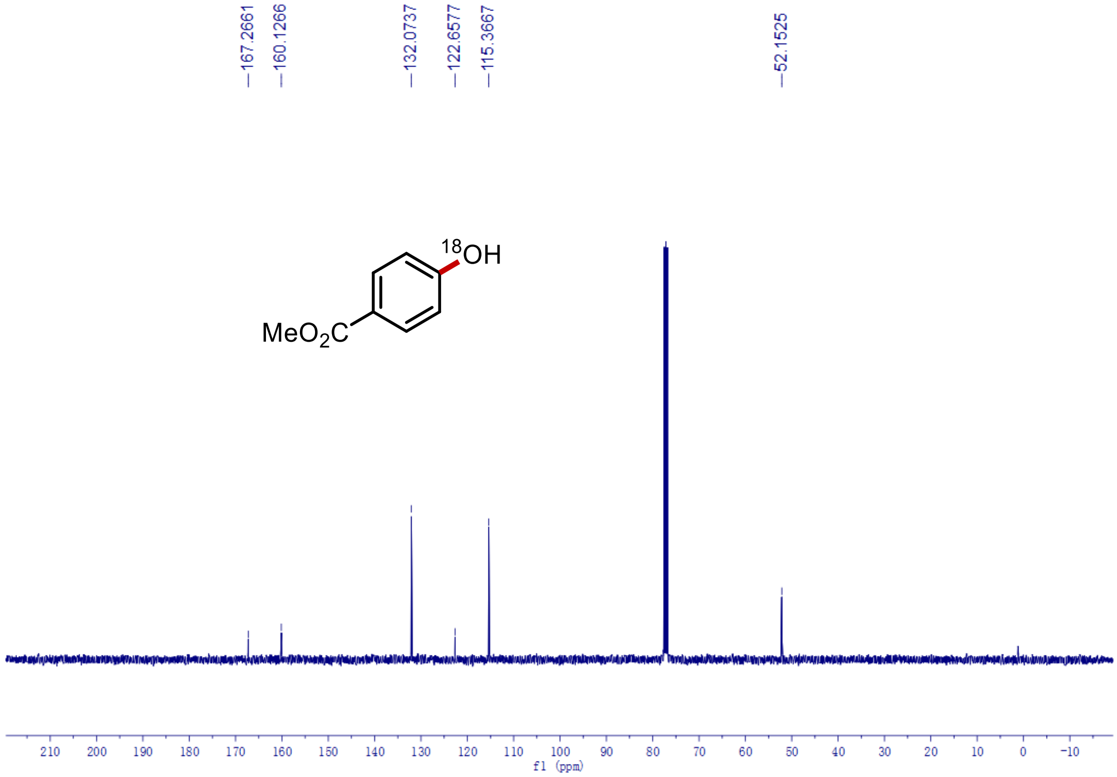

^{13}C NMR (100 MHz, $CDCl_3$) Spectrum

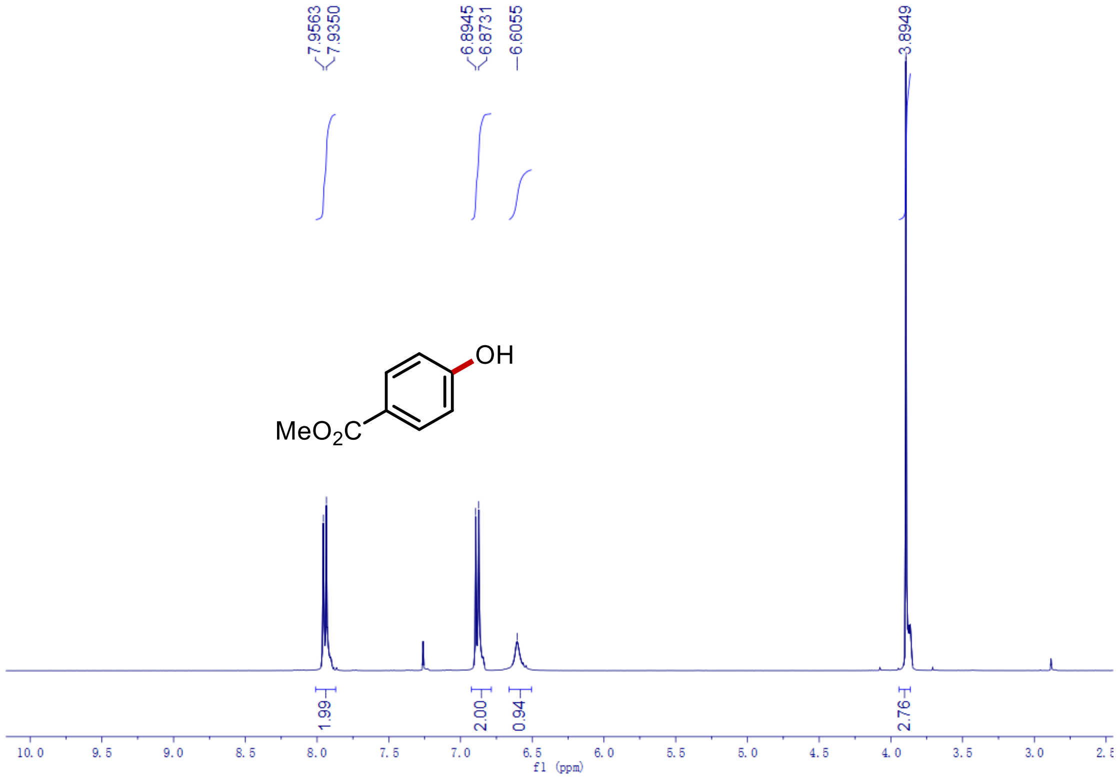

¹H NMR (400 MHz, $CDCl_3$) Spectrum

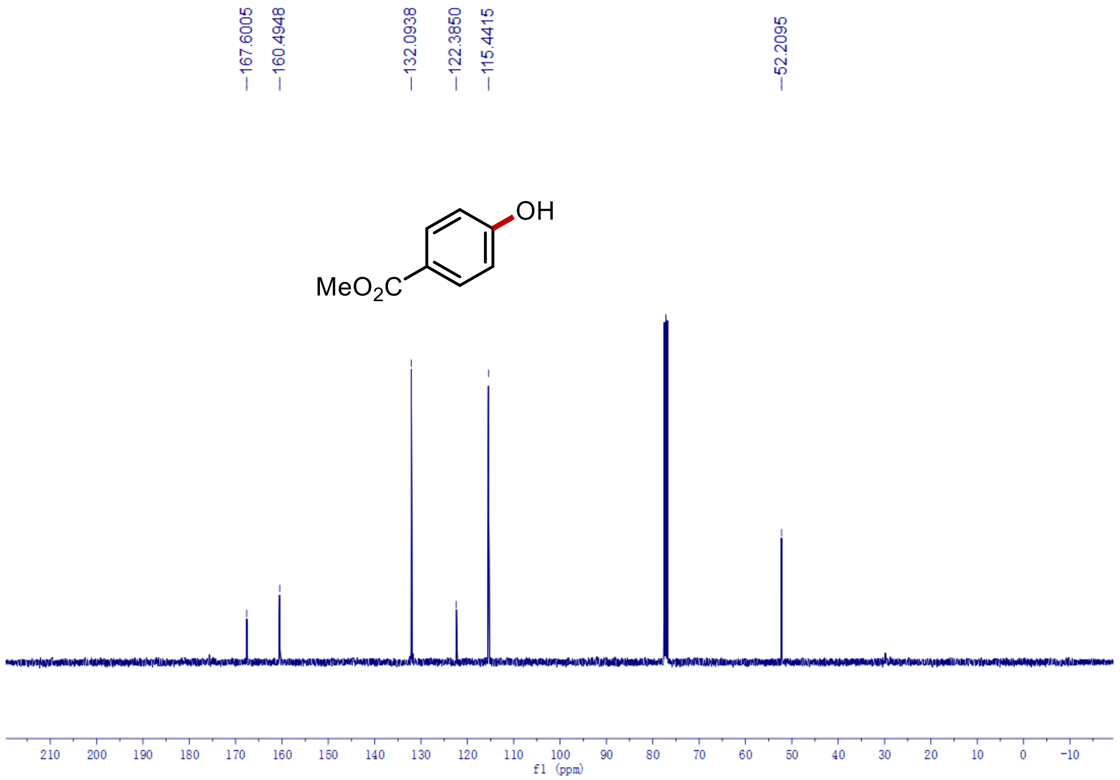

¹³C NMR (100 MHz, $CDCl_3$) Spectrum

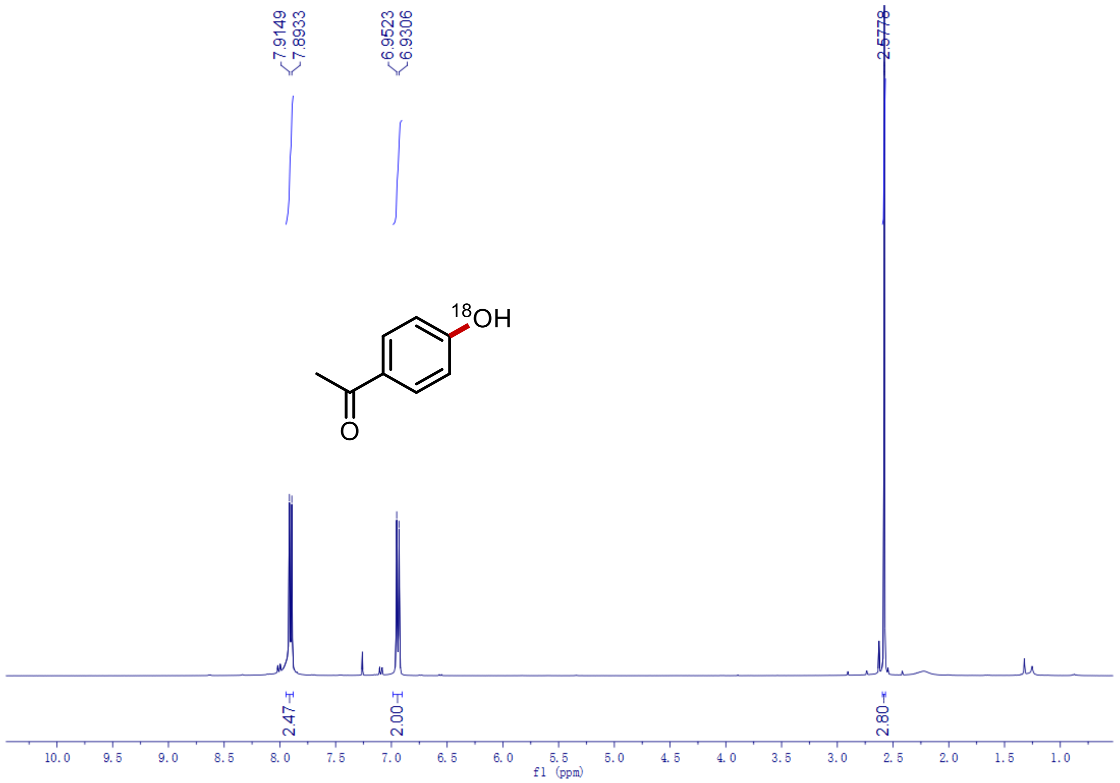

^{1}H NMR (400 MHz, $CDCl_3$) Spectrum

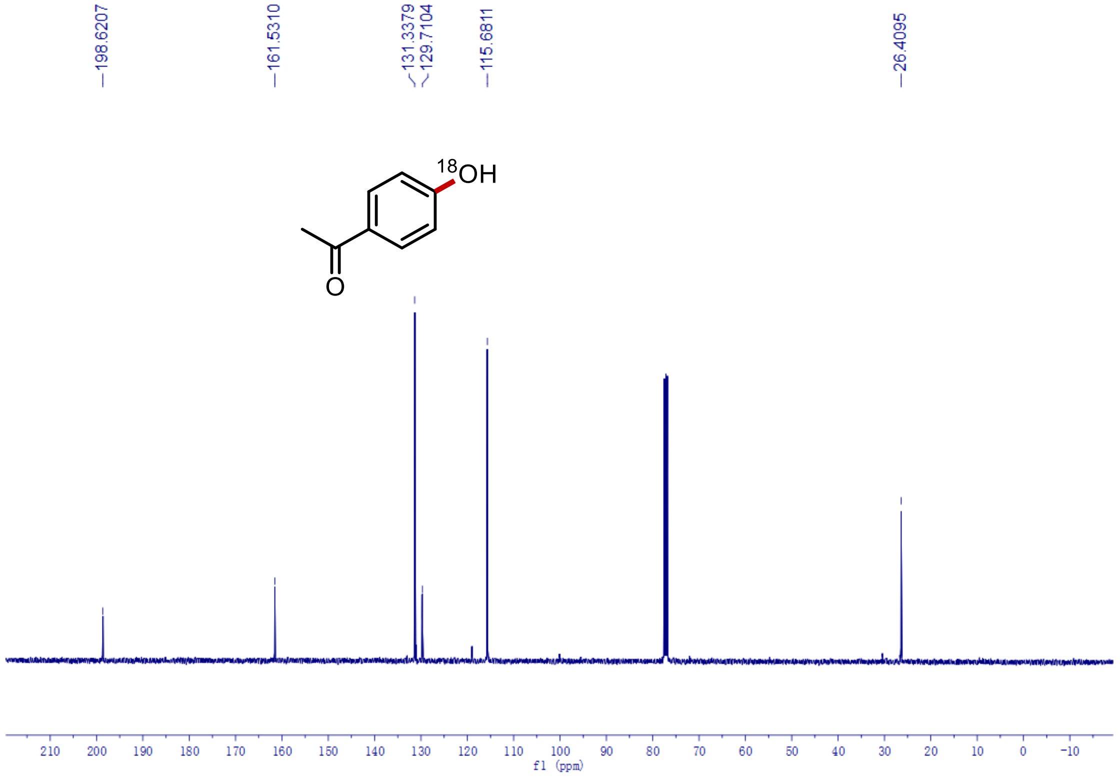

^{13}C NMR (100 MHz, $CDCl_3$) Spectrum

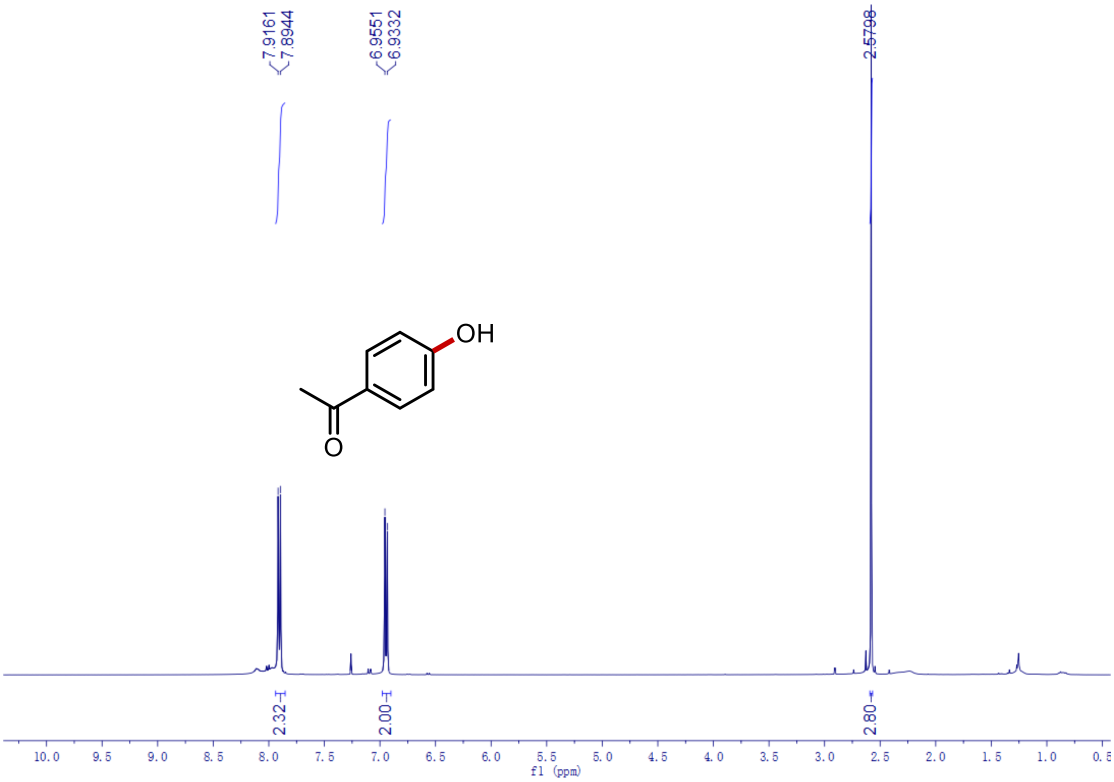

^{1}H NMR (400 MHz, $CDCl_3$) Spectrum

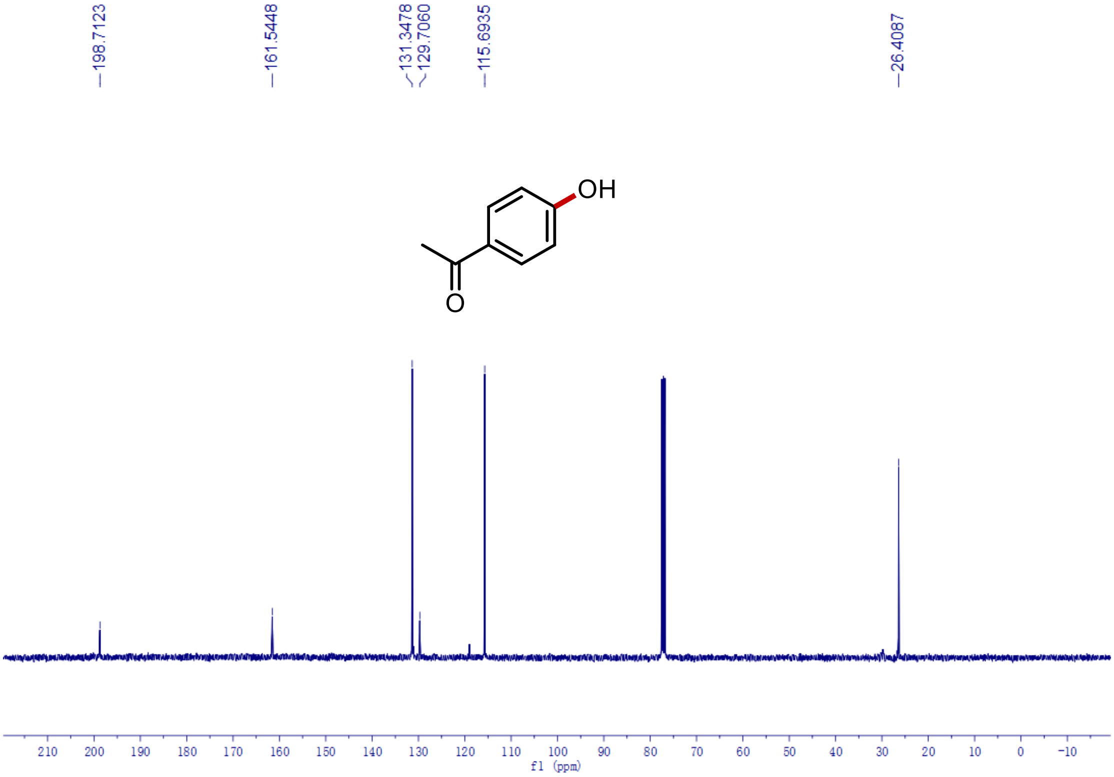

^{13}C NMR (100 MHz, $CDCl_3$) Spectrum

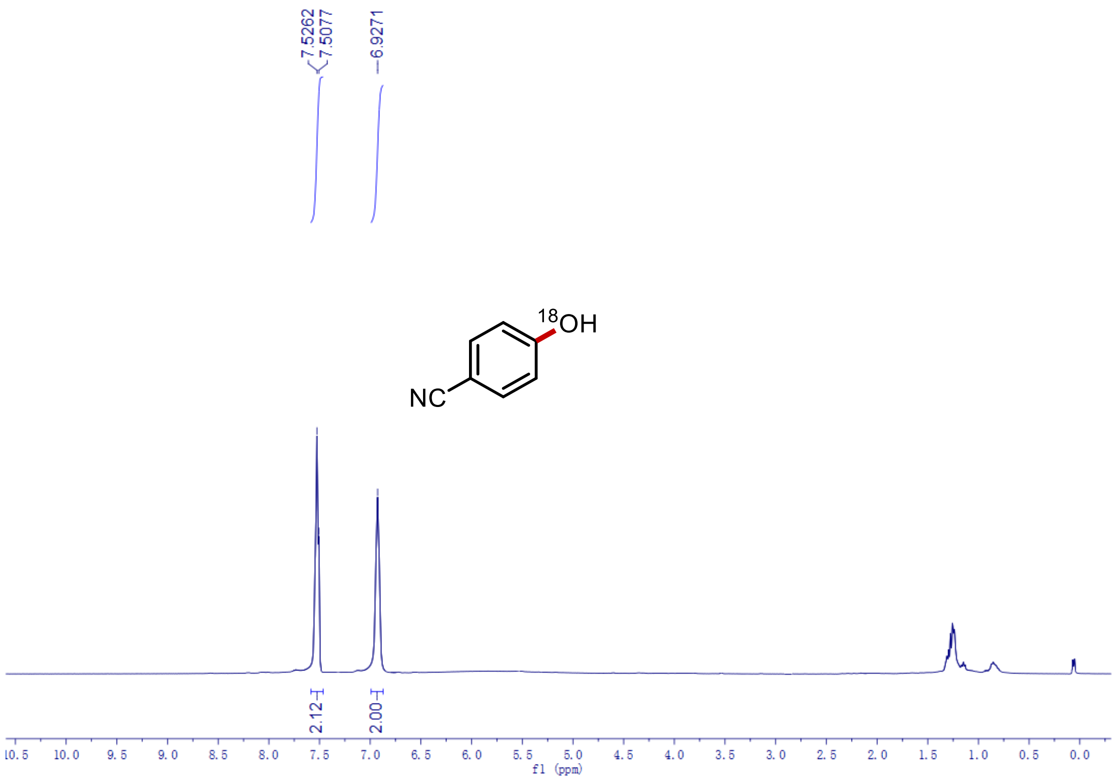

^{1}H NMR (400 MHz, $CDCl_3$) Spectrum

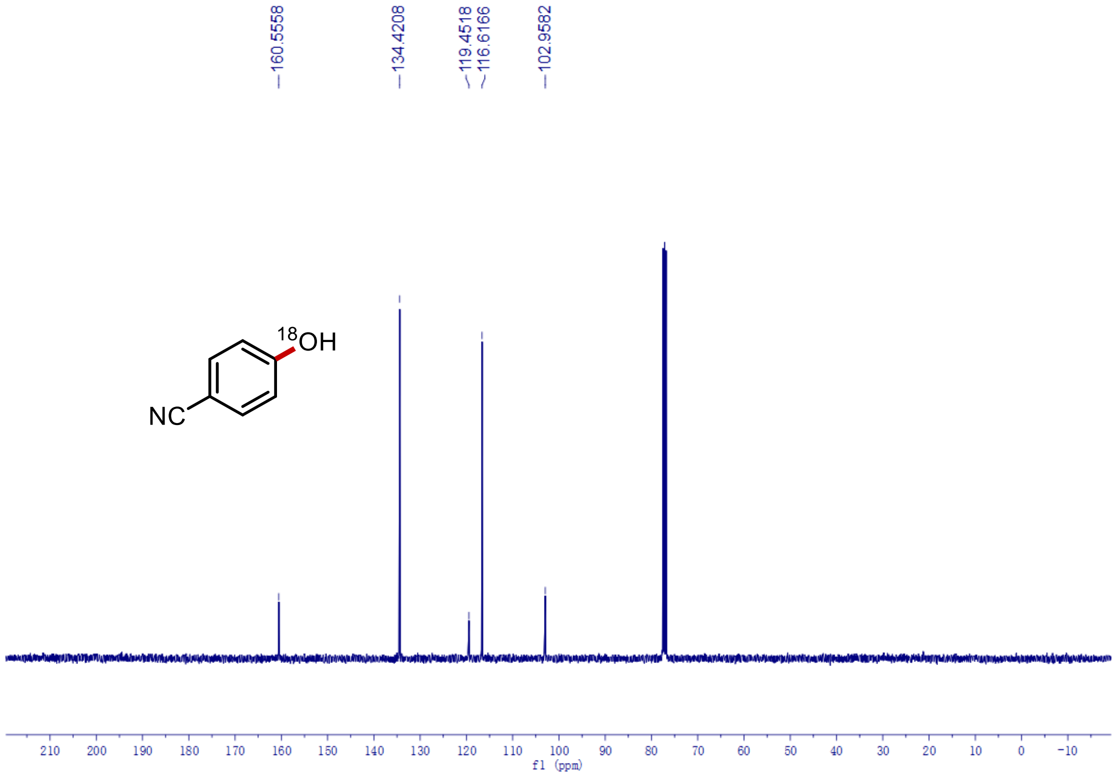

^{13}C NMR (100 MHz, $CDCl_3$) Spectrum

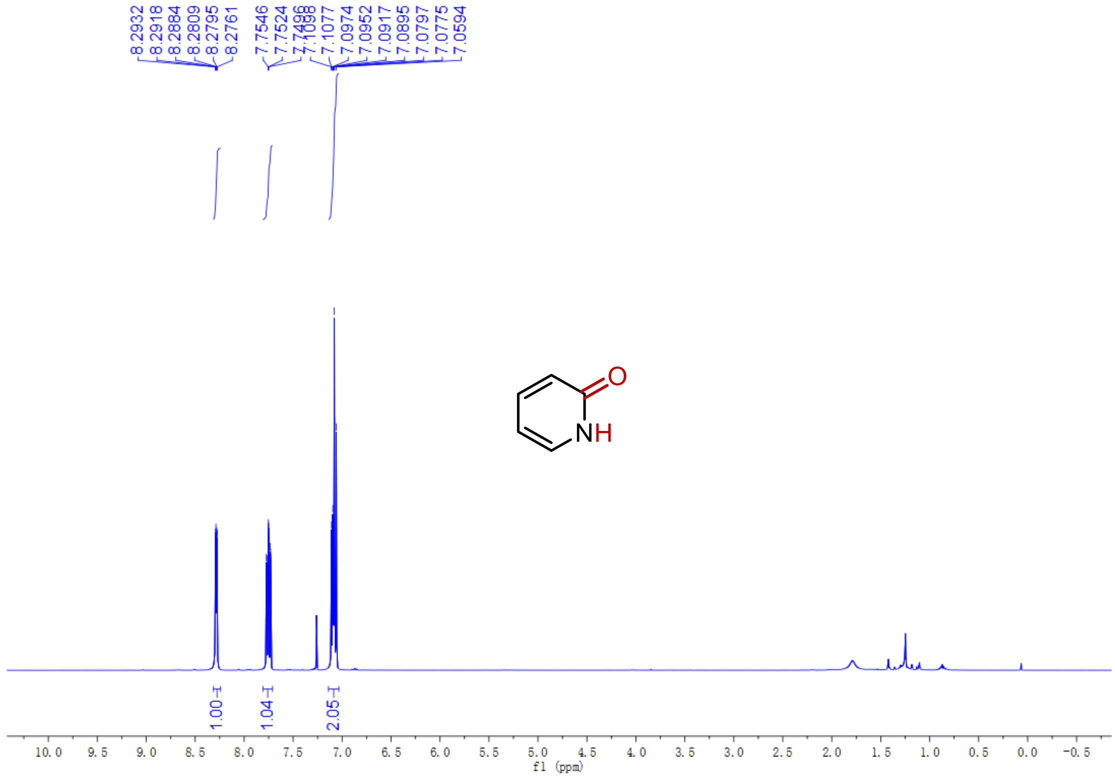

^{1}H NMR (400 MHz, $CDCl_3$) Spectrum

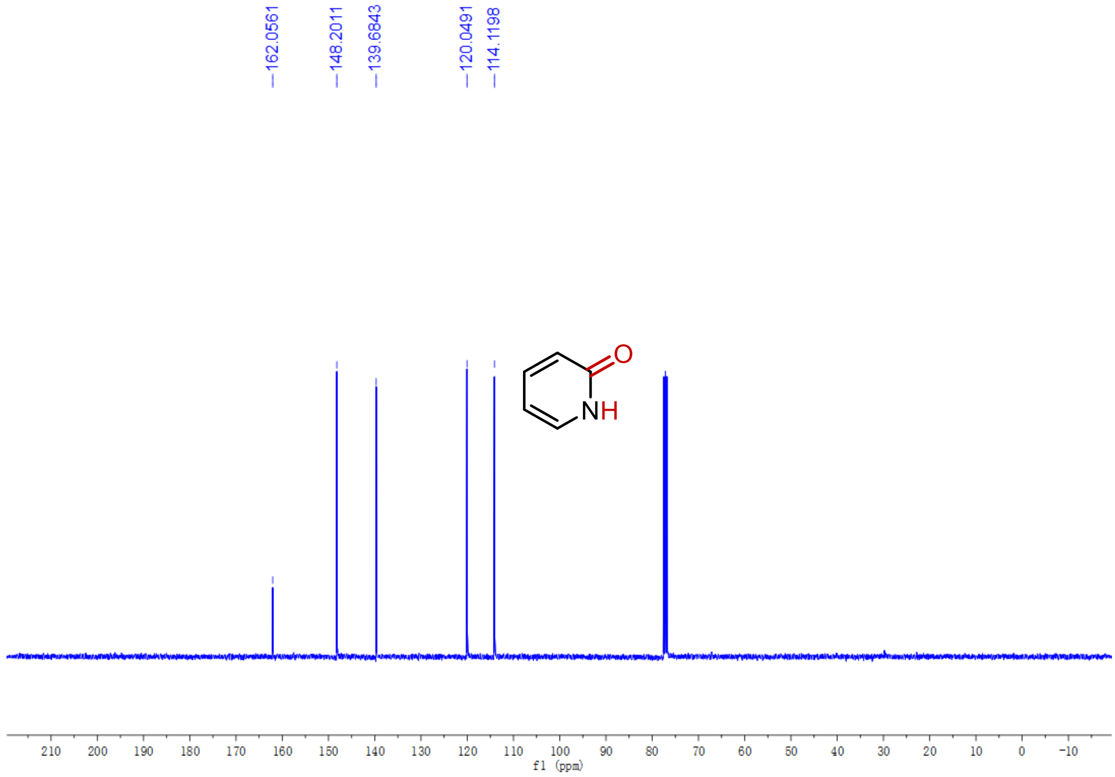

^{13}C NMR (100 MHz, $CDCl_3$) Spectrum

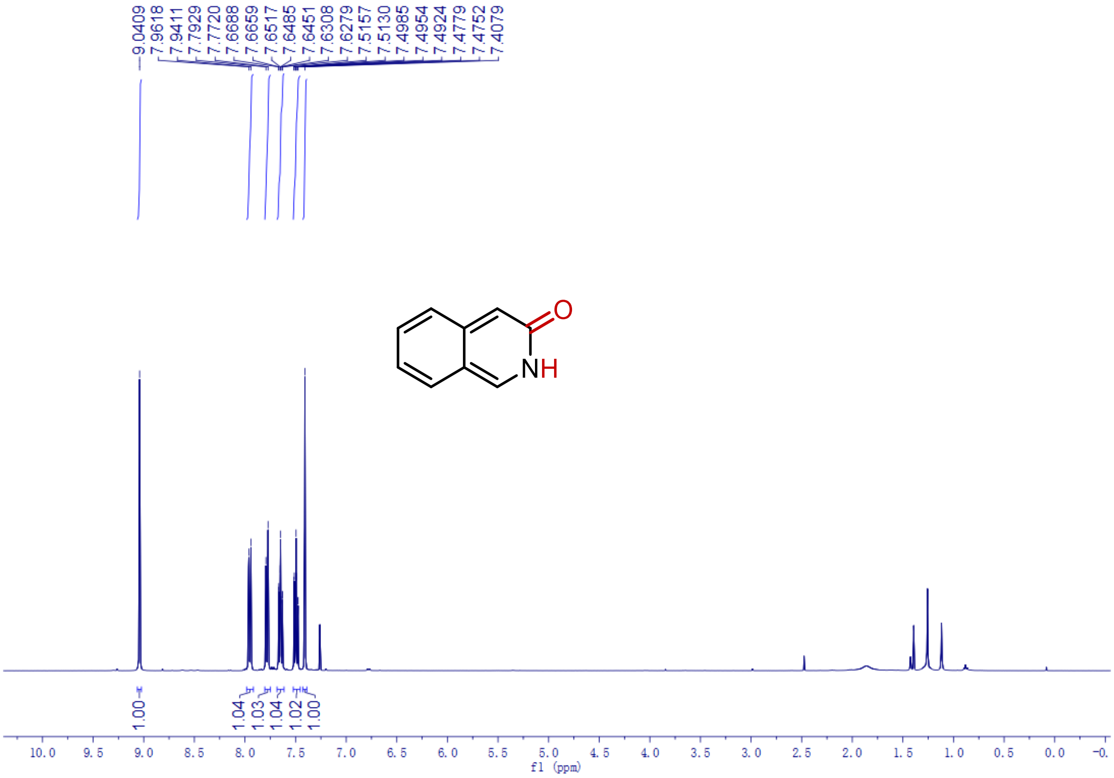

^{1}H NMR (400 MHz, $CDCl_3$) Spectrum

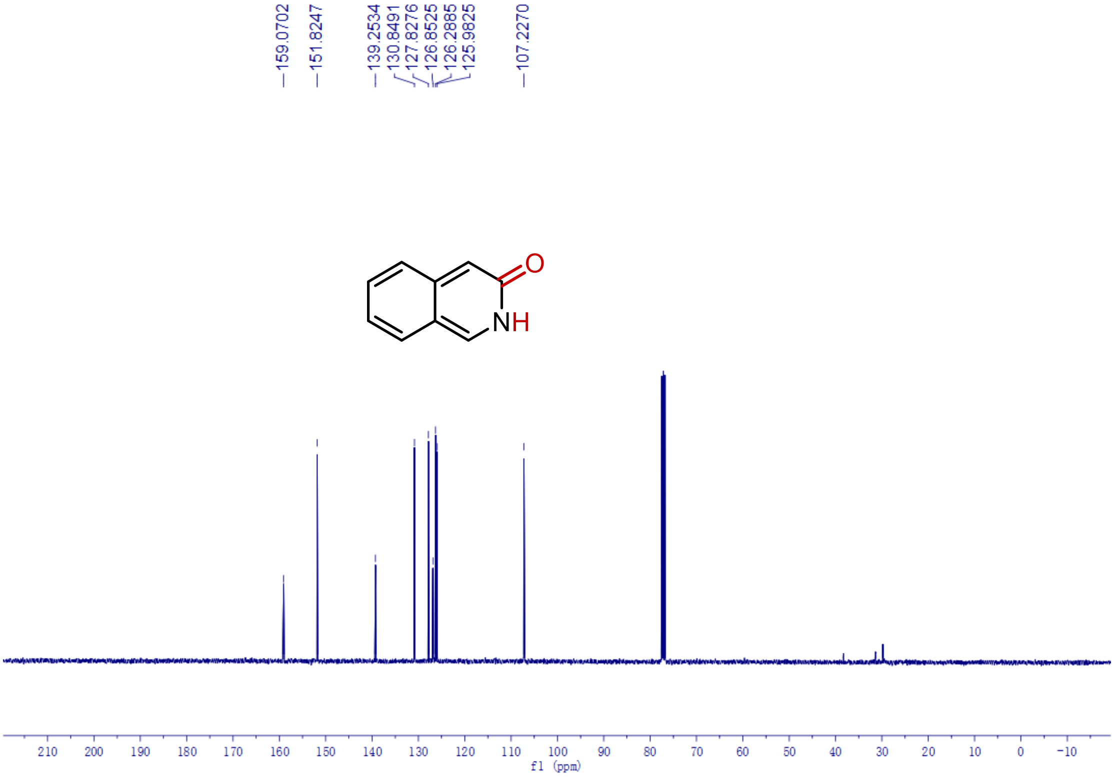

^{13}C NMR (100 MHz, $CDCl_3$) Spectrum

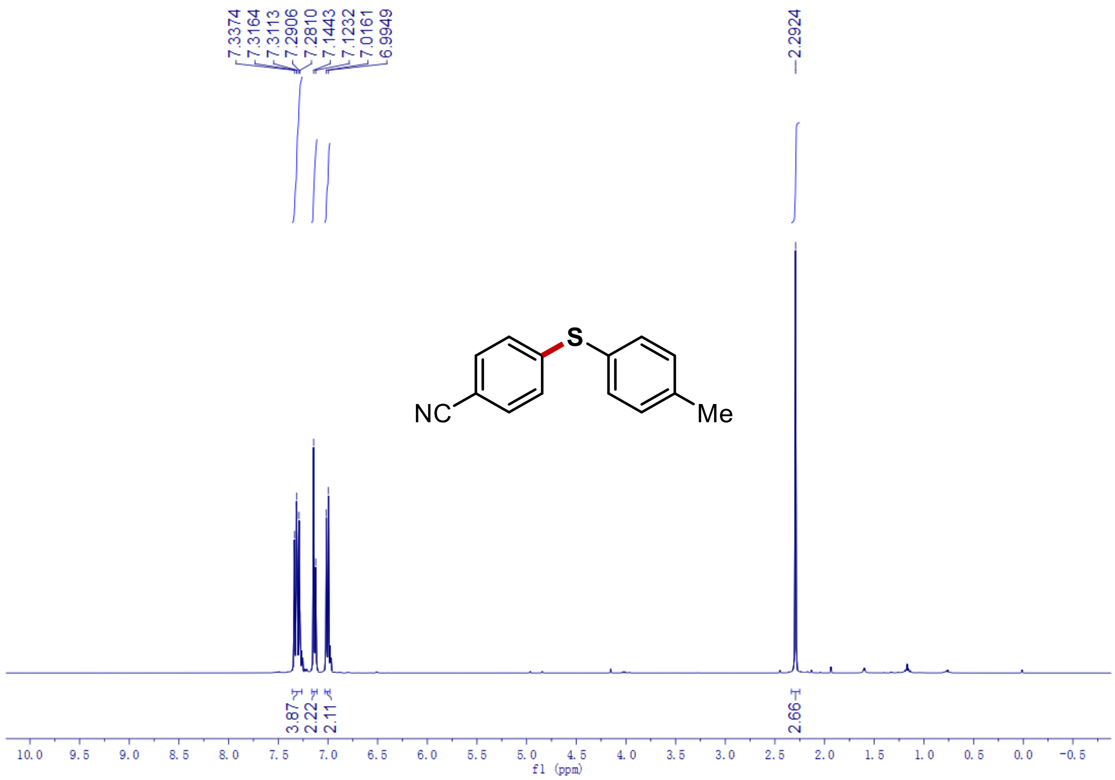

^{1}H NMR (400 MHz, $CDCl_3$) Spectrum

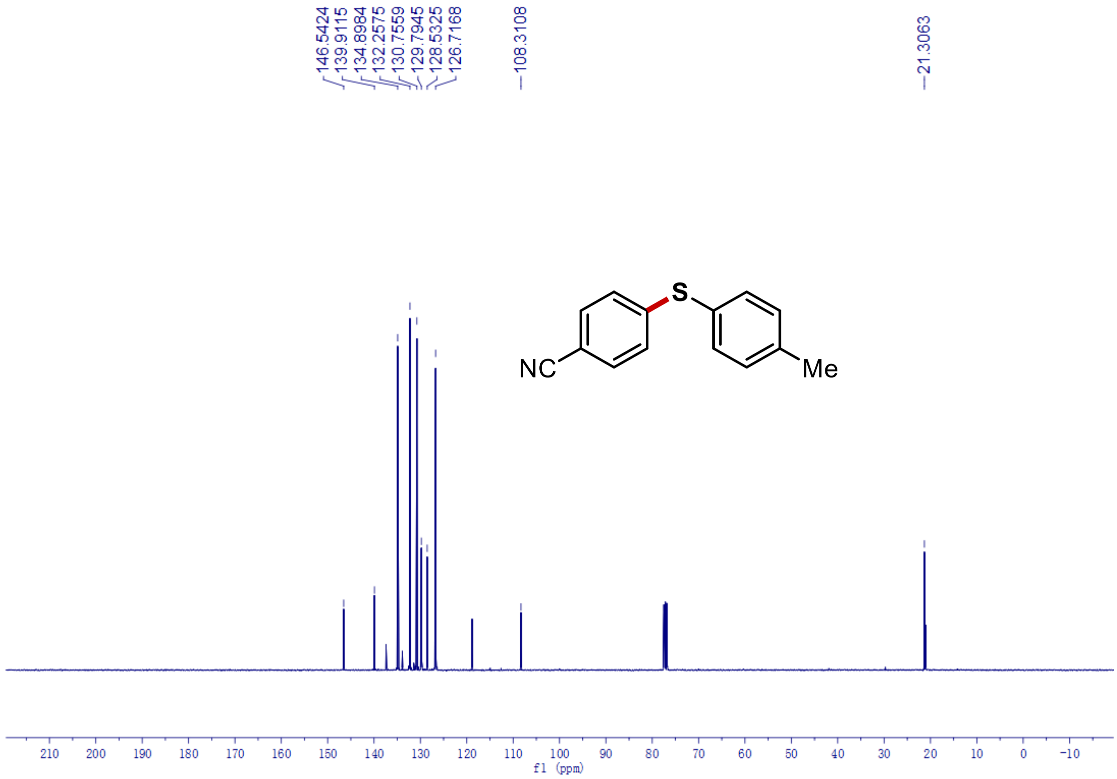

^{13}C NMR (100 MHz, $CDCl_3$) Spectrum

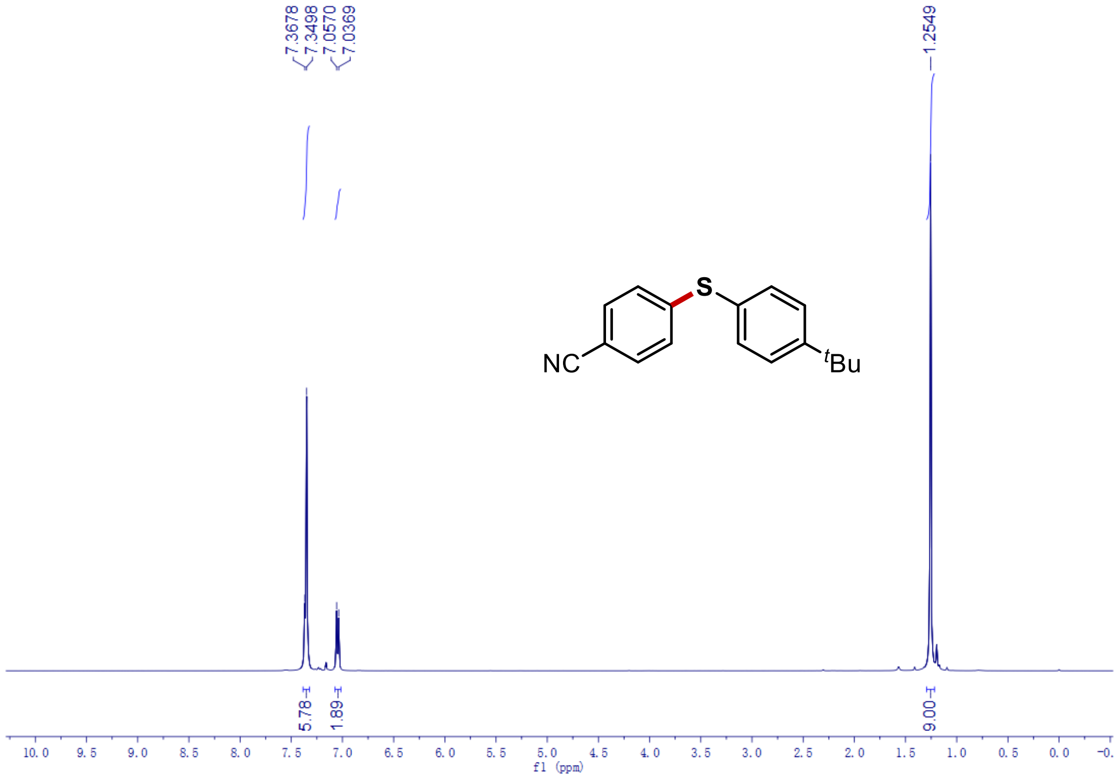

^{1}H NMR (400 MHz, $CDCl_3$) Spectrum

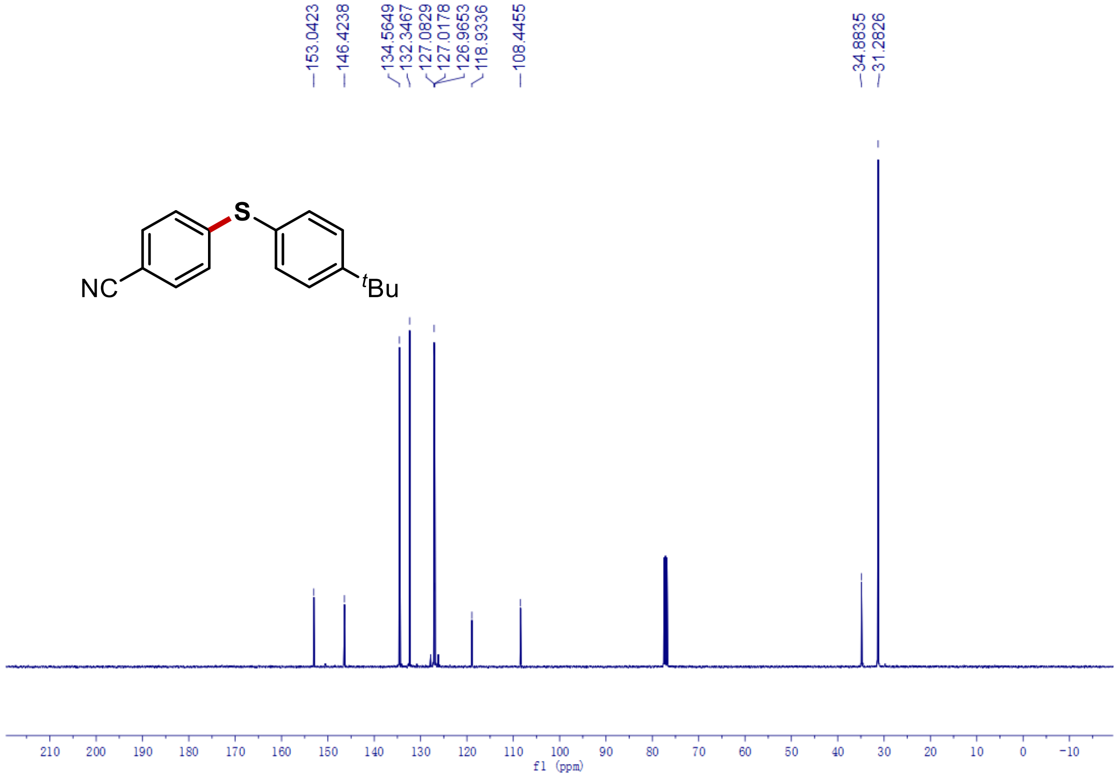

^{13}C NMR (100 MHz, $CDCl_3$) Spectrum

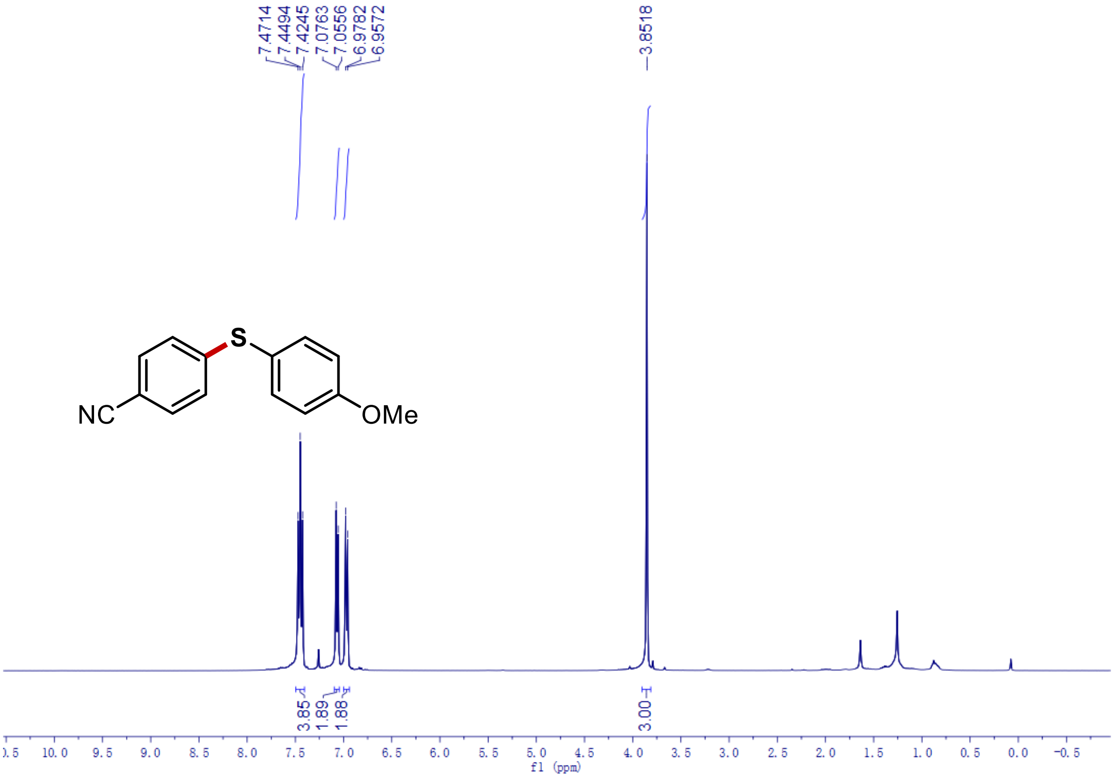

^{1}H NMR (400 MHz, $CDCl_3$) Spectrum

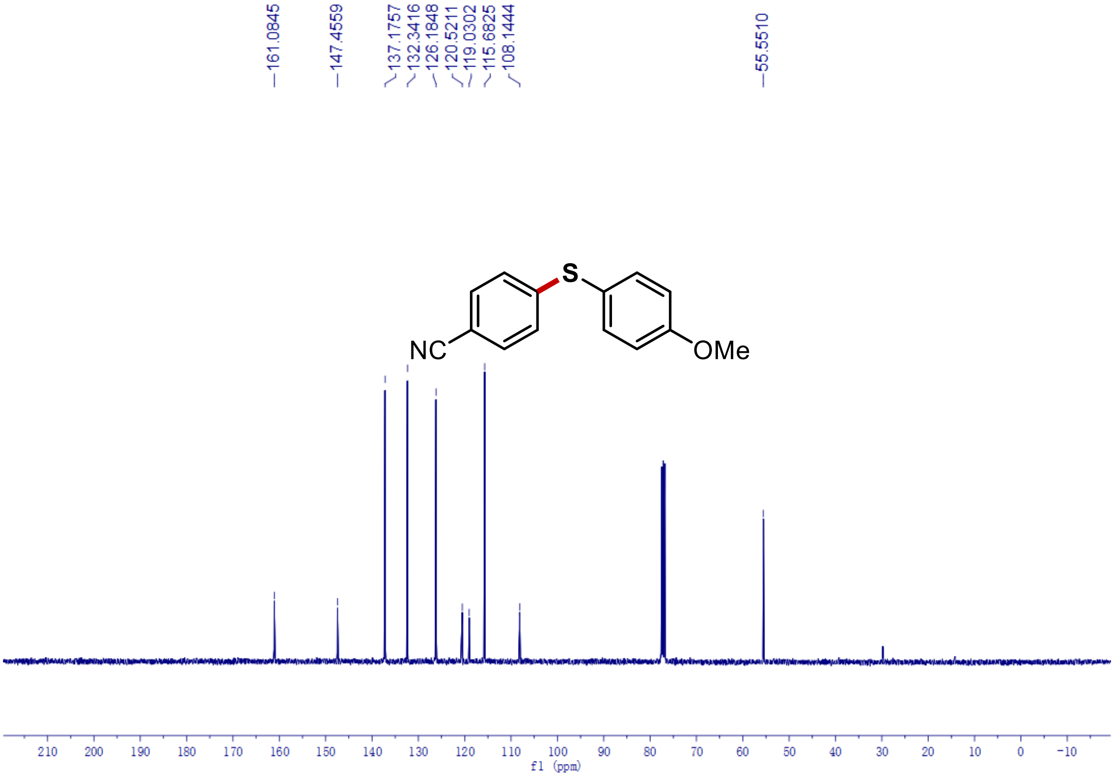

^{13}C NMR (100 MHz, $CDCl_3$) Spectrum

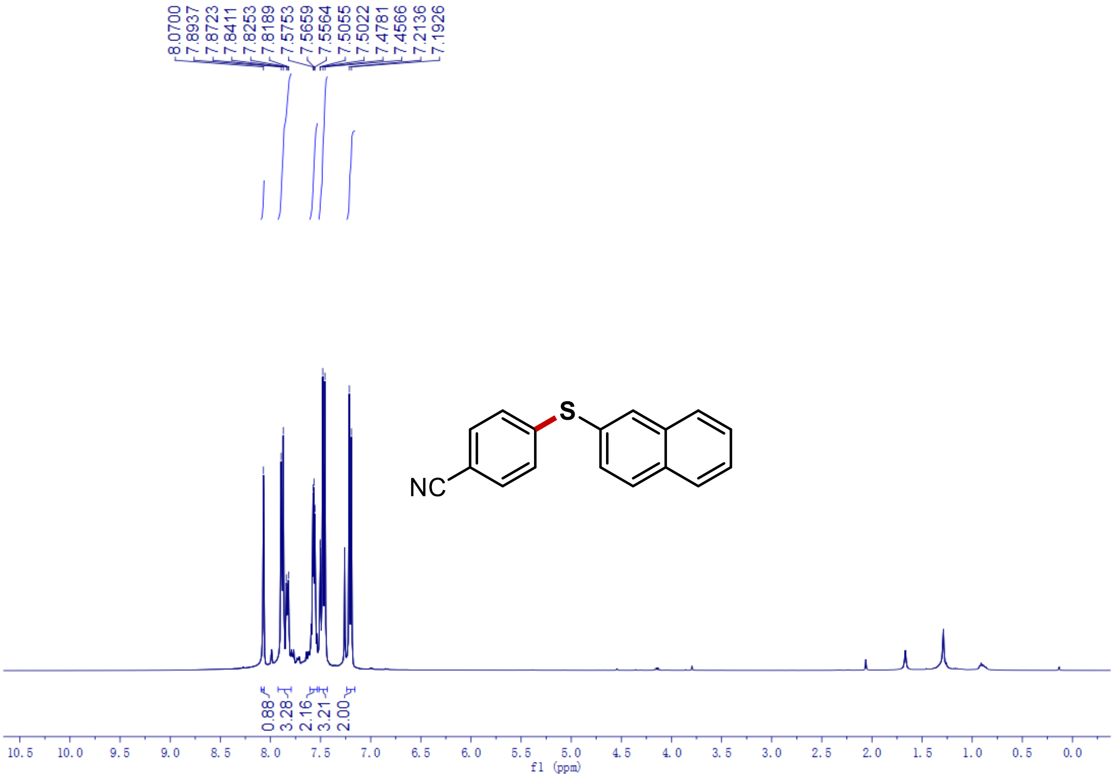

¹H NMR (400 MHz, $CDCl_3$) Spectrum

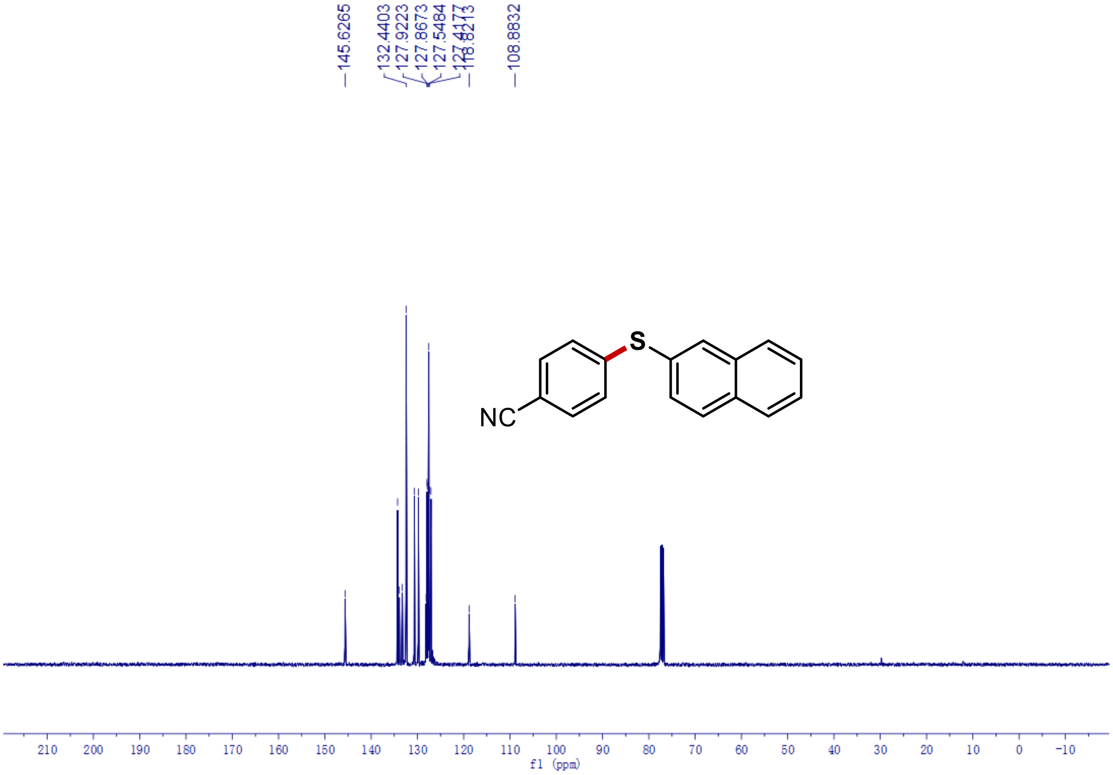

¹³C NMR (100 MHz, $CDCl_3$) Spectrum

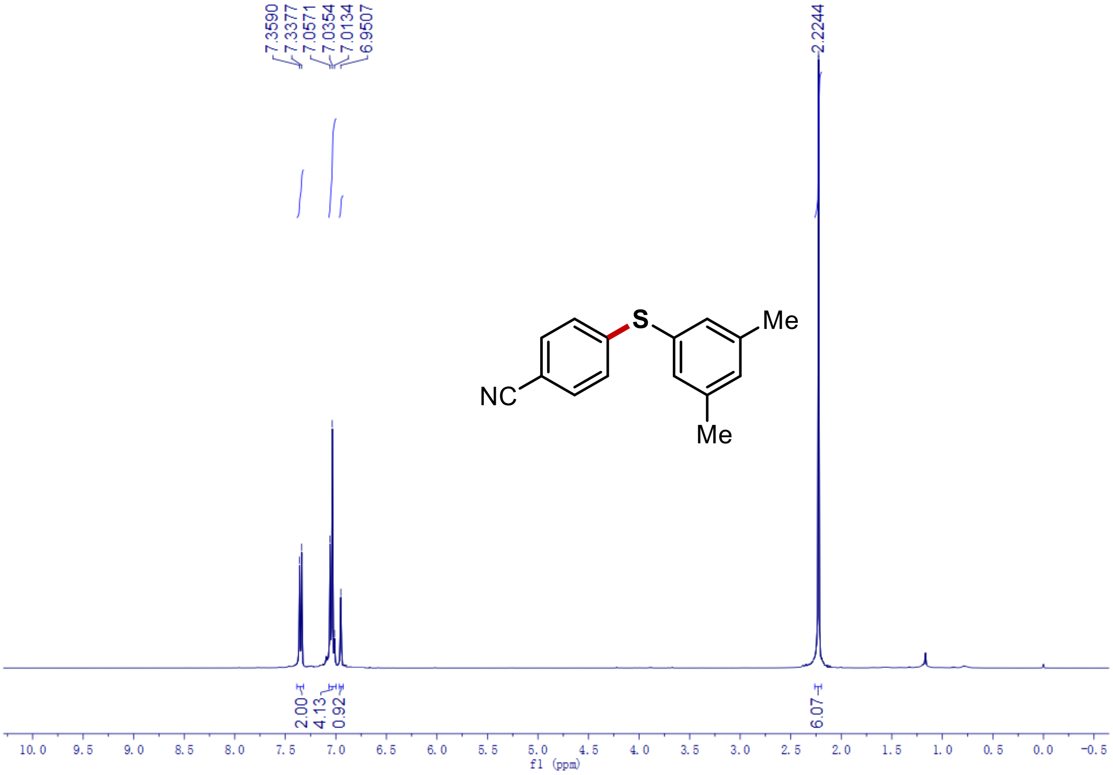

^{1}H NMR (400 MHz, $CDCl_3$) Spectrum

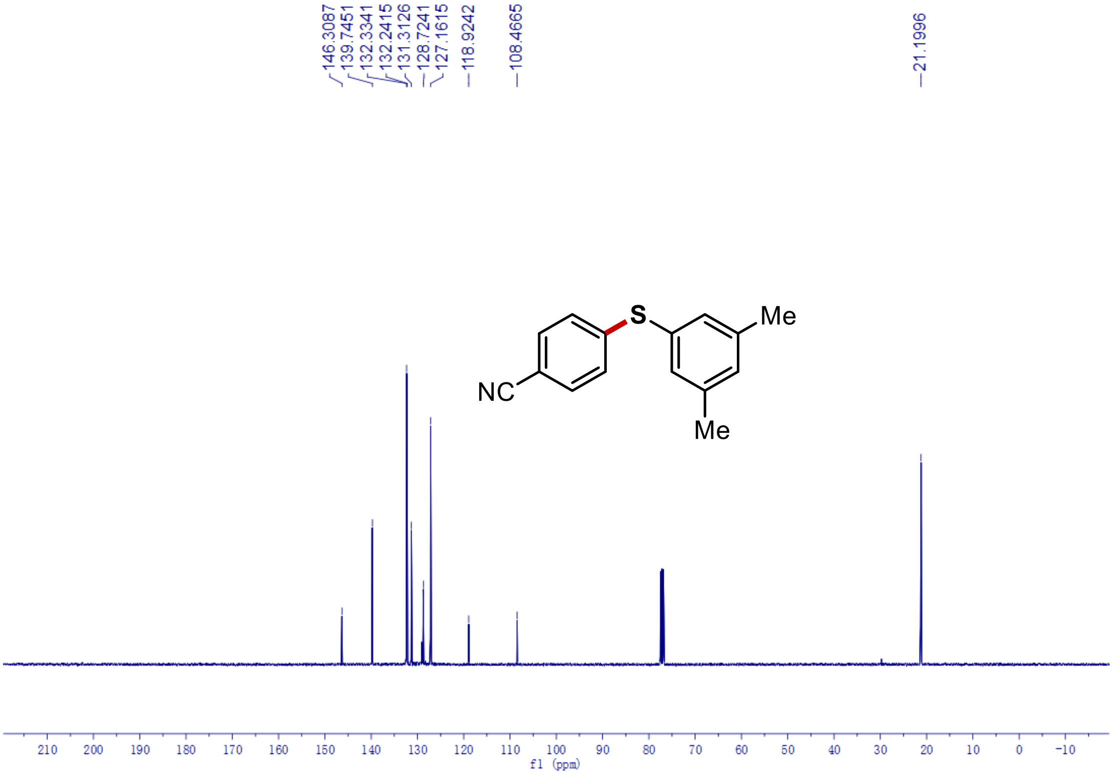

^{13}C NMR (100 MHz, $CDCl_3$) Spectrum

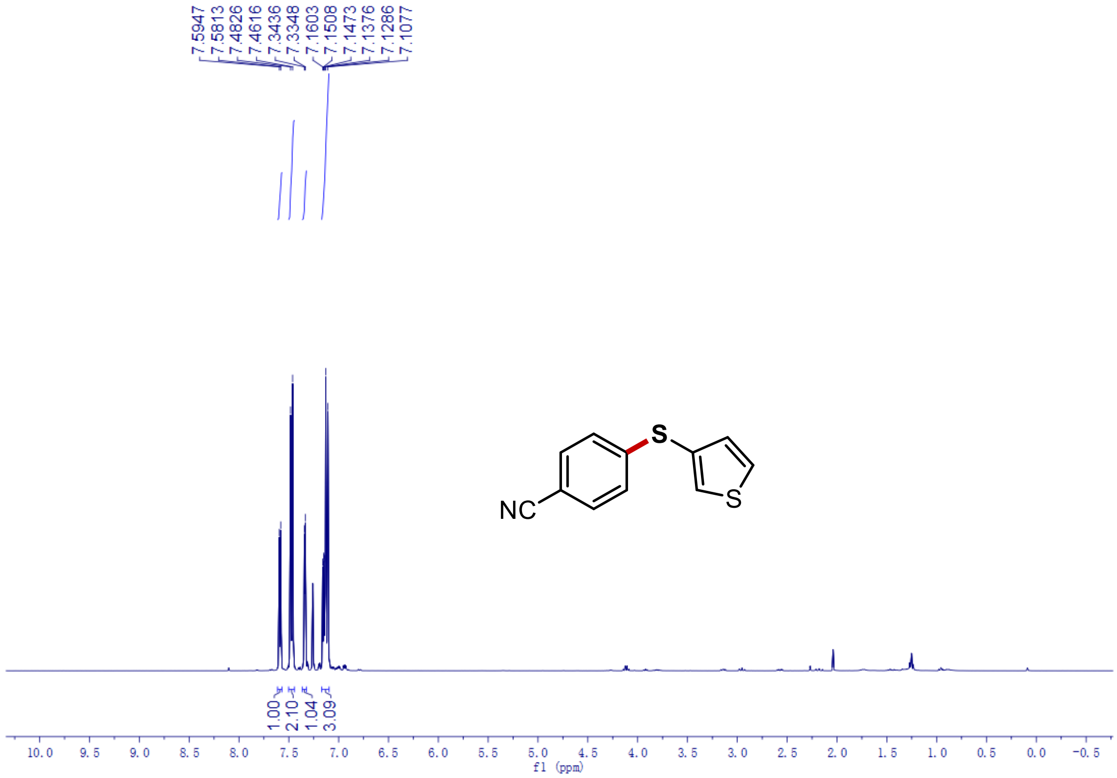

¹H NMR (400 MHz, $CDCl_3$) Spectrum

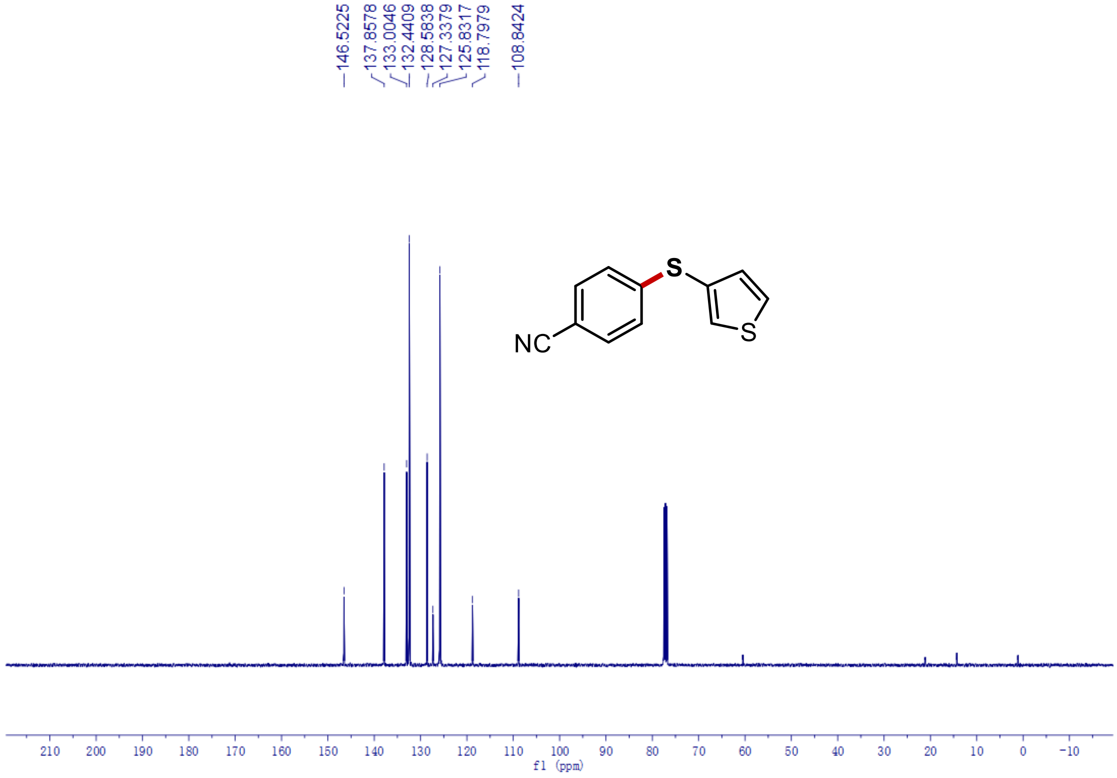

¹³C NMR (100 MHz, $CDCl_3$) Spectrum

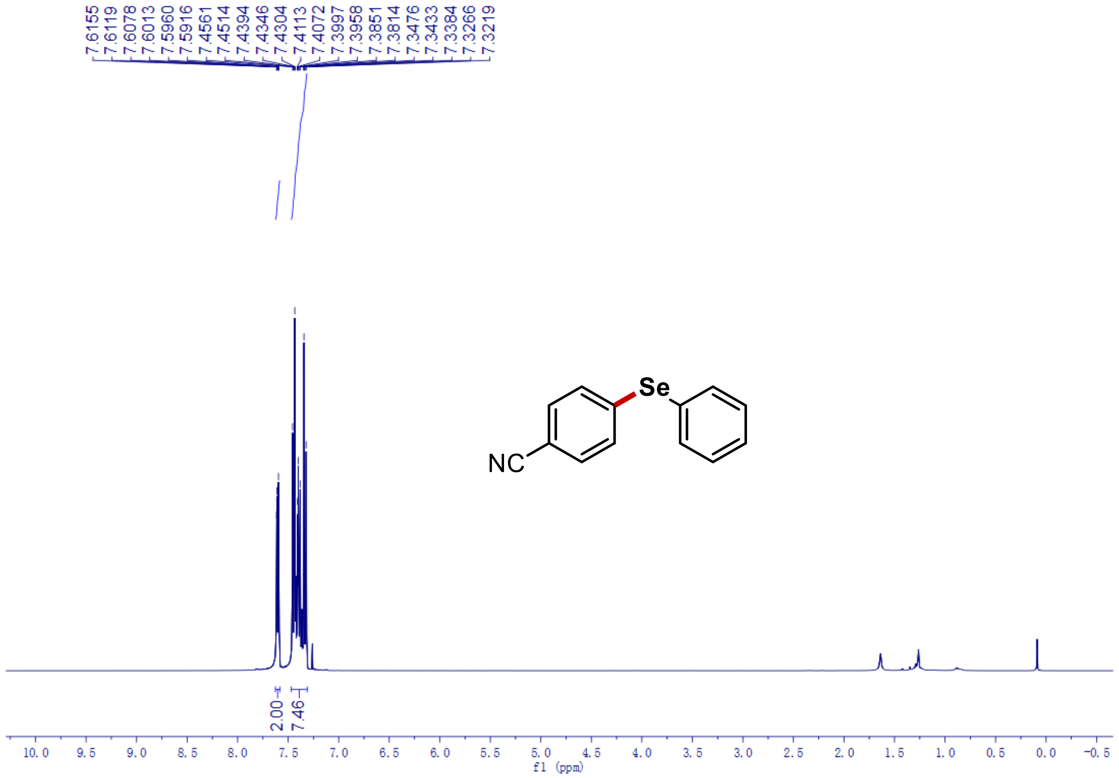

^{1}**H NMR** (400 MHz, $CDCl_3$) Spectrum

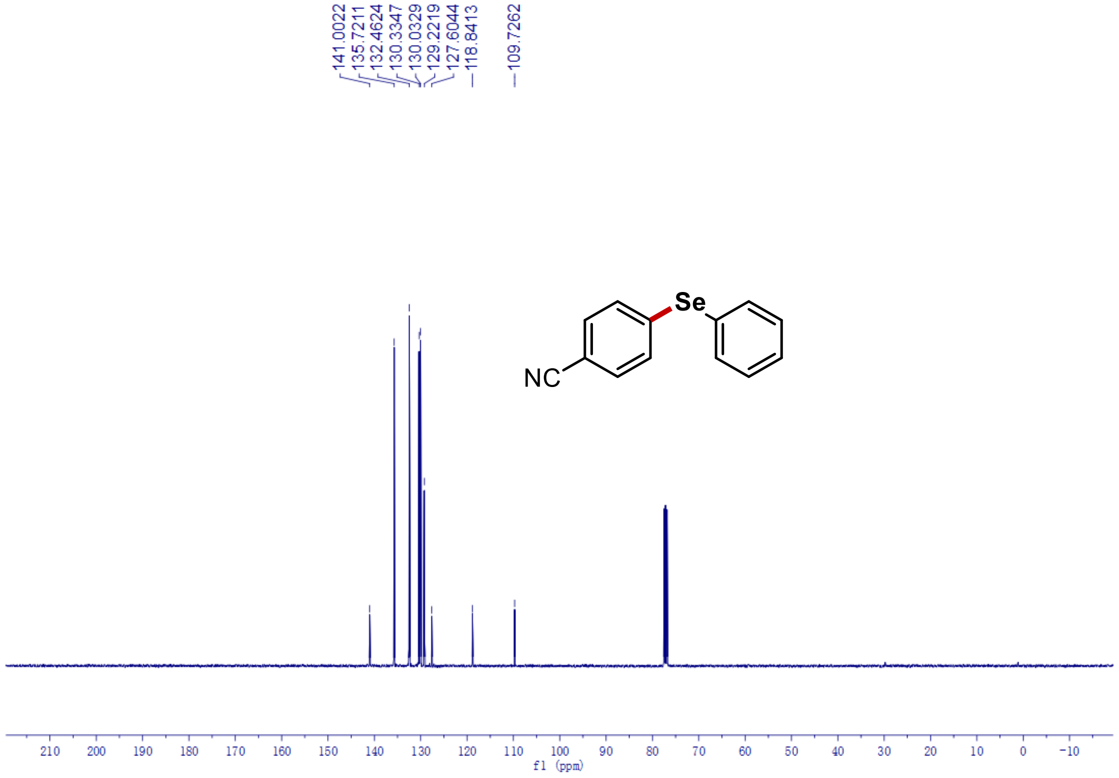

^{13}**C NMR** (100 MHz, $CDCl_3$) Spectrum

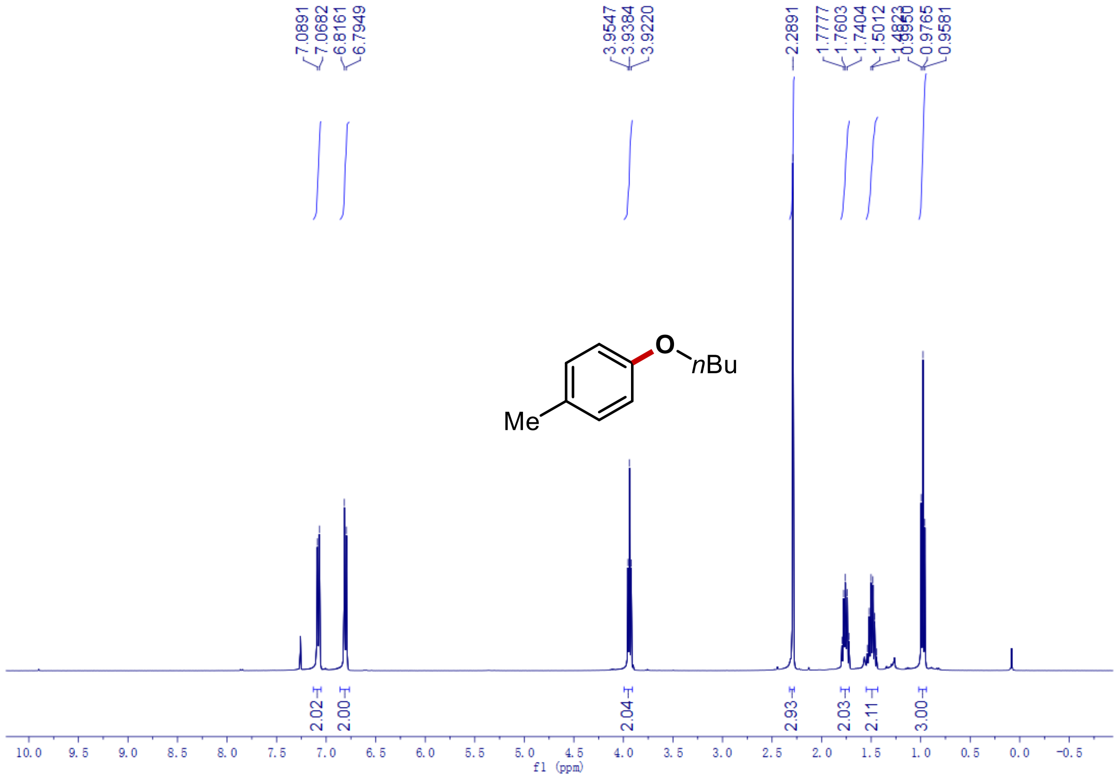

^{1}H NMR (400 MHz, $CDCl_3$) Spectrum

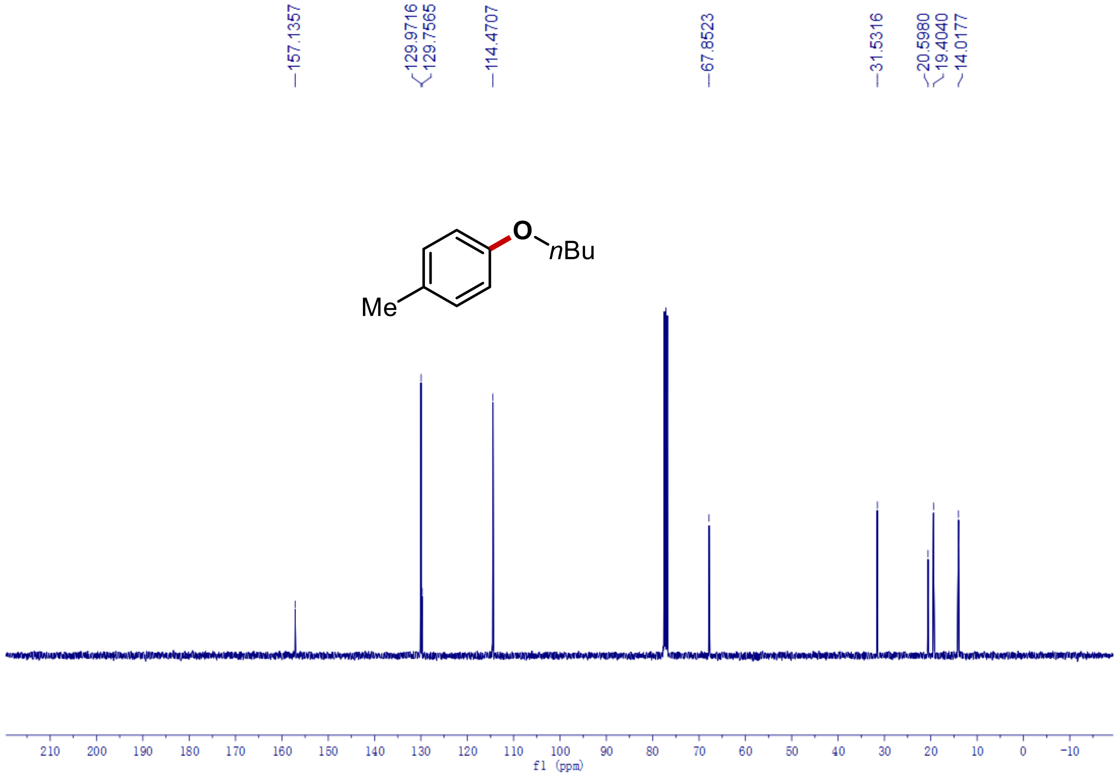

^{13}C NMR (100 MHz, $CDCl_3$) Spectrum

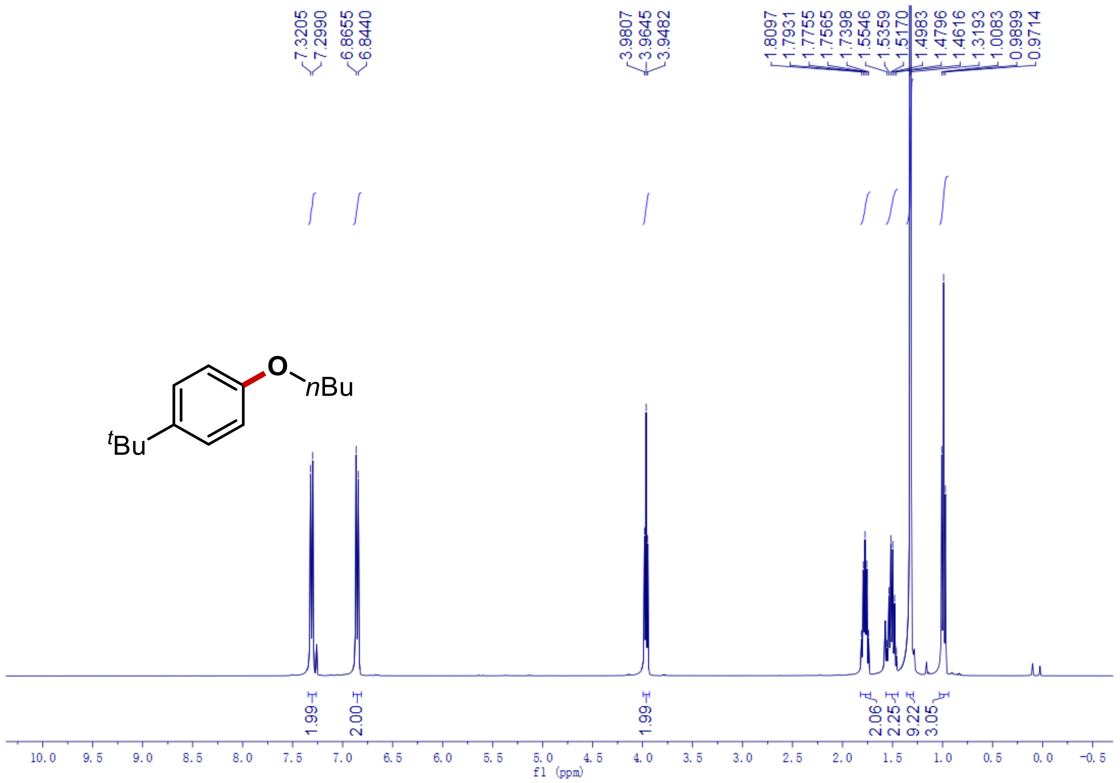

^{1}H NMR (400 MHz, $CDCl_3$) Spectrum

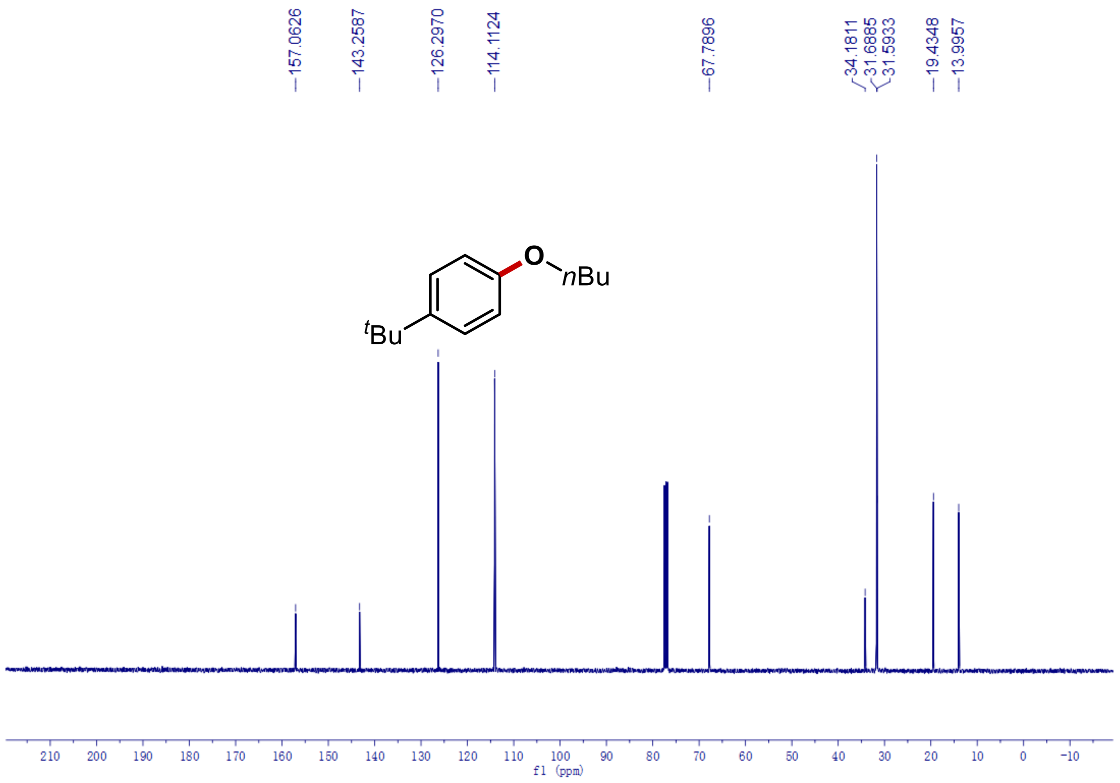

^{13}C NMR (100 MHz, $CDCl_3$) Spectrum

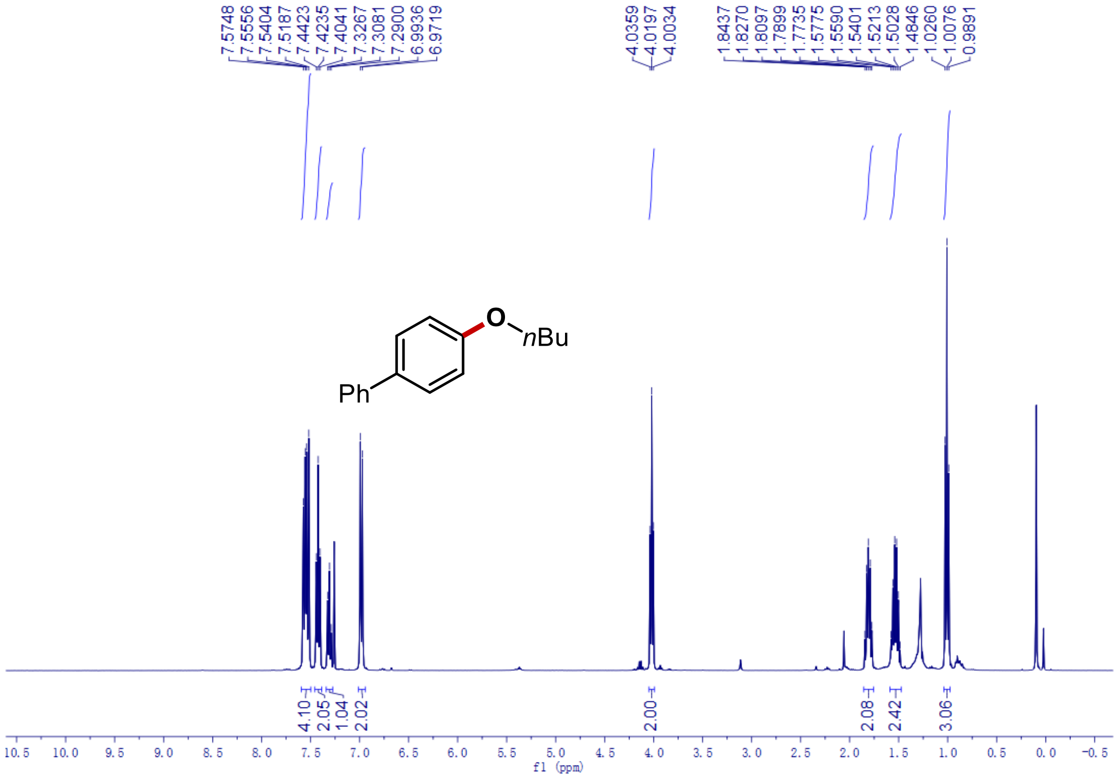

^{1}H NMR (400 MHz, $CDCl_3$) Spectrum

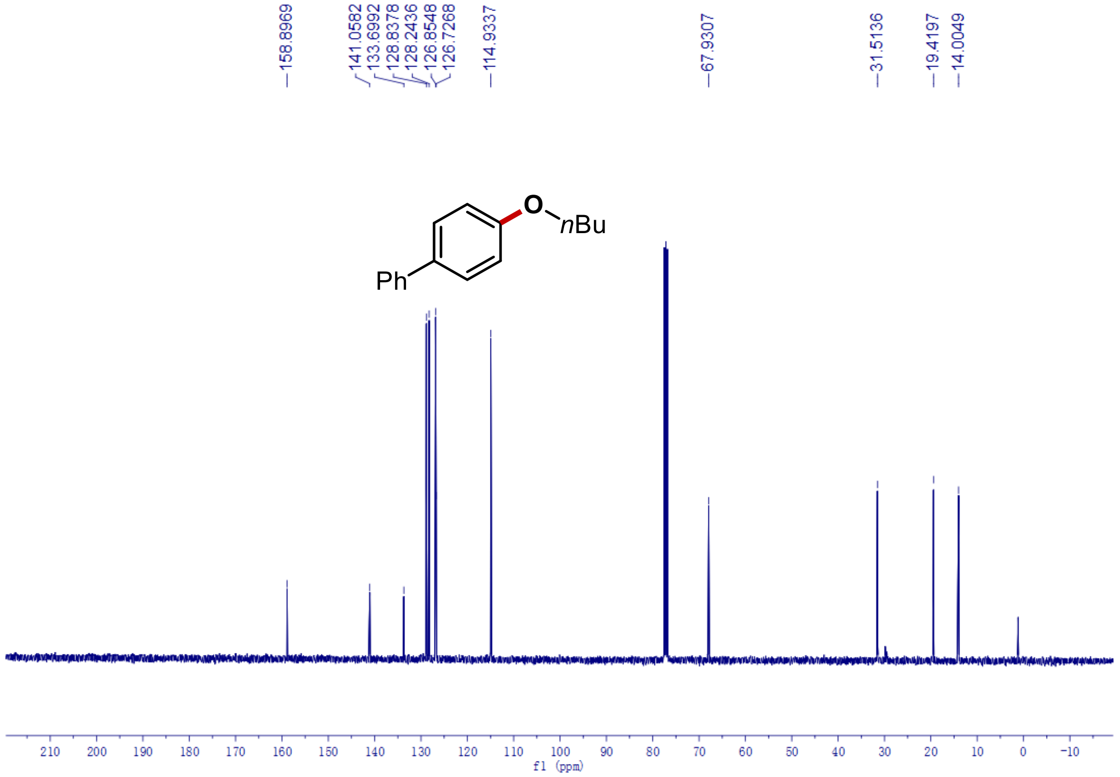

^{13}C NMR (100 MHz, $CDCl_3$) Spectrum

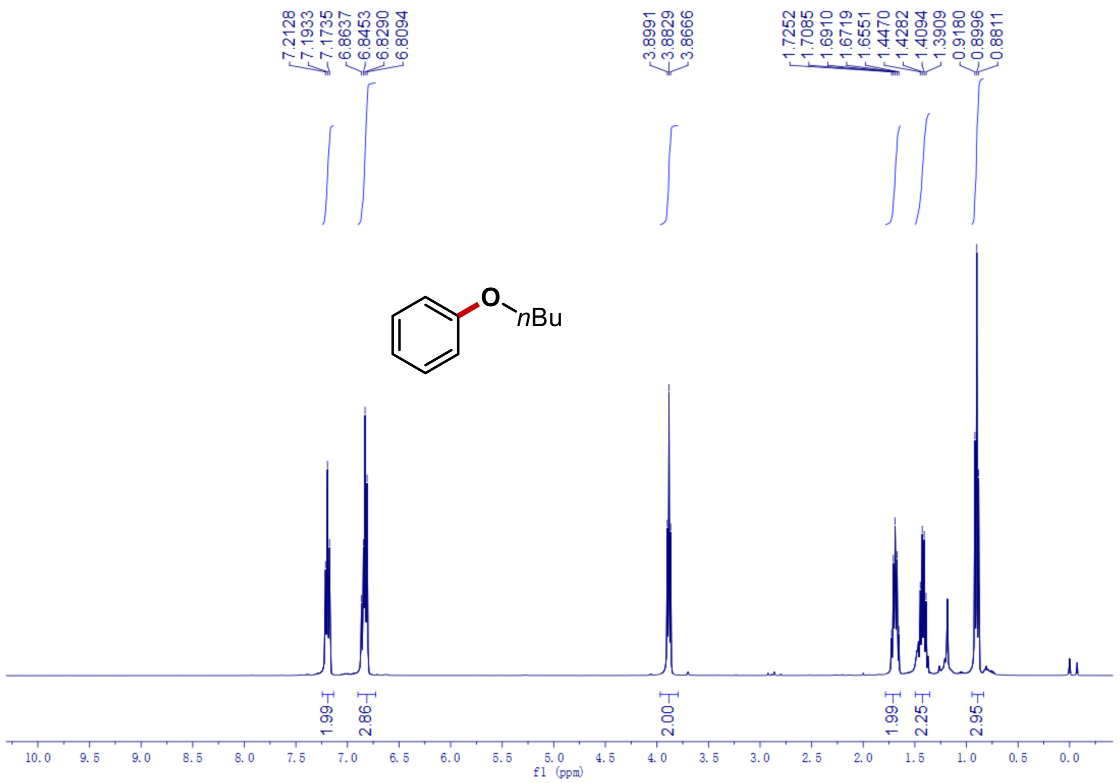

^{1}H NMR (400 MHz, $CDCl_3$) Spectrum

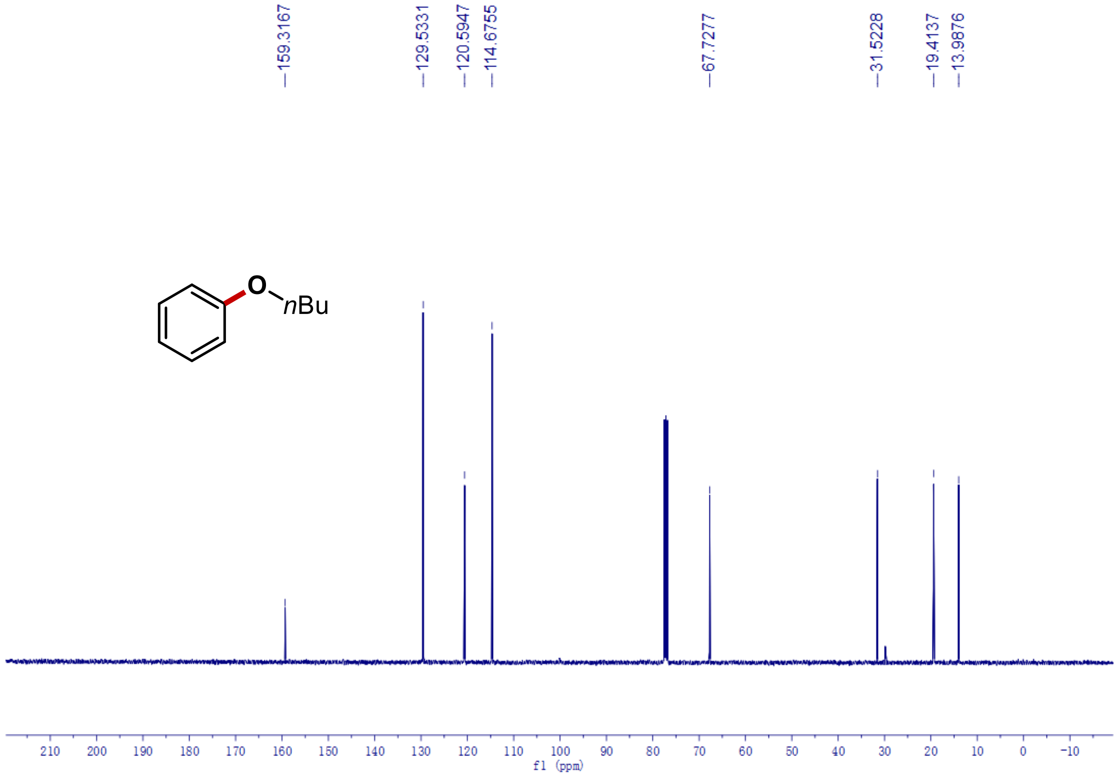

^{13}C NMR (100 MHz, $CDCl_3$) Spectrum

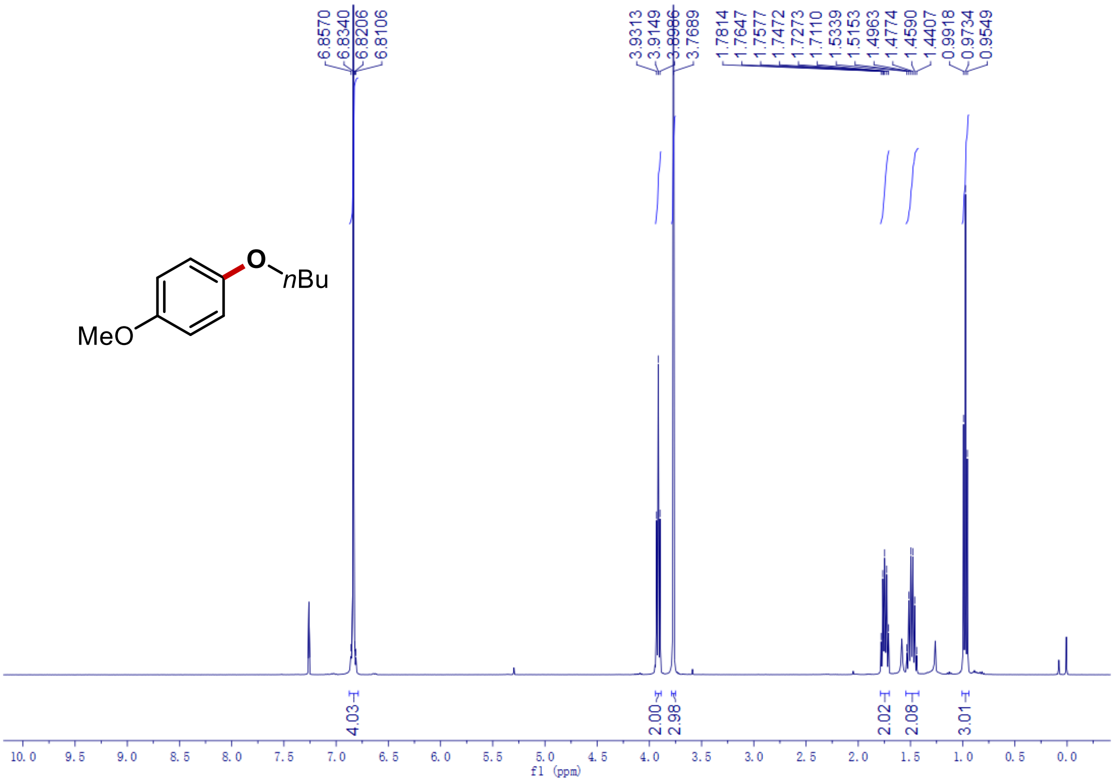

^{1}H NMR (400 MHz, $CDCl_3$) Spectrum

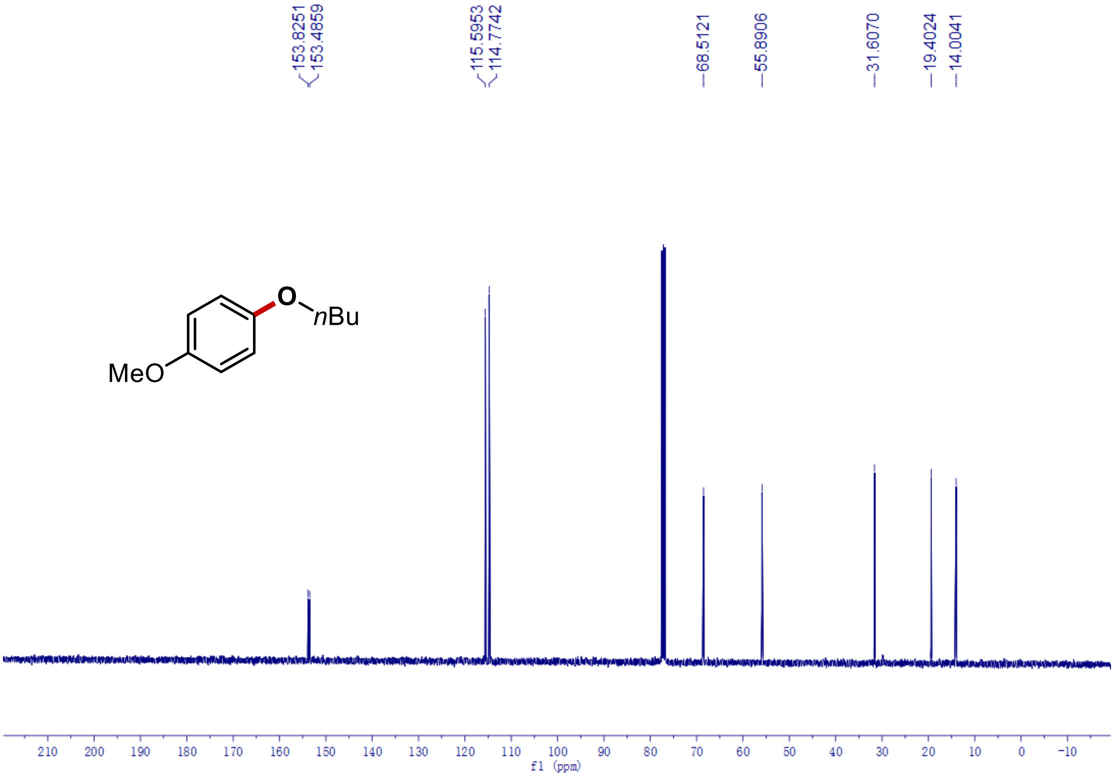

^{13}C NMR (100 MHz, $CDCl_3$) Spectrum

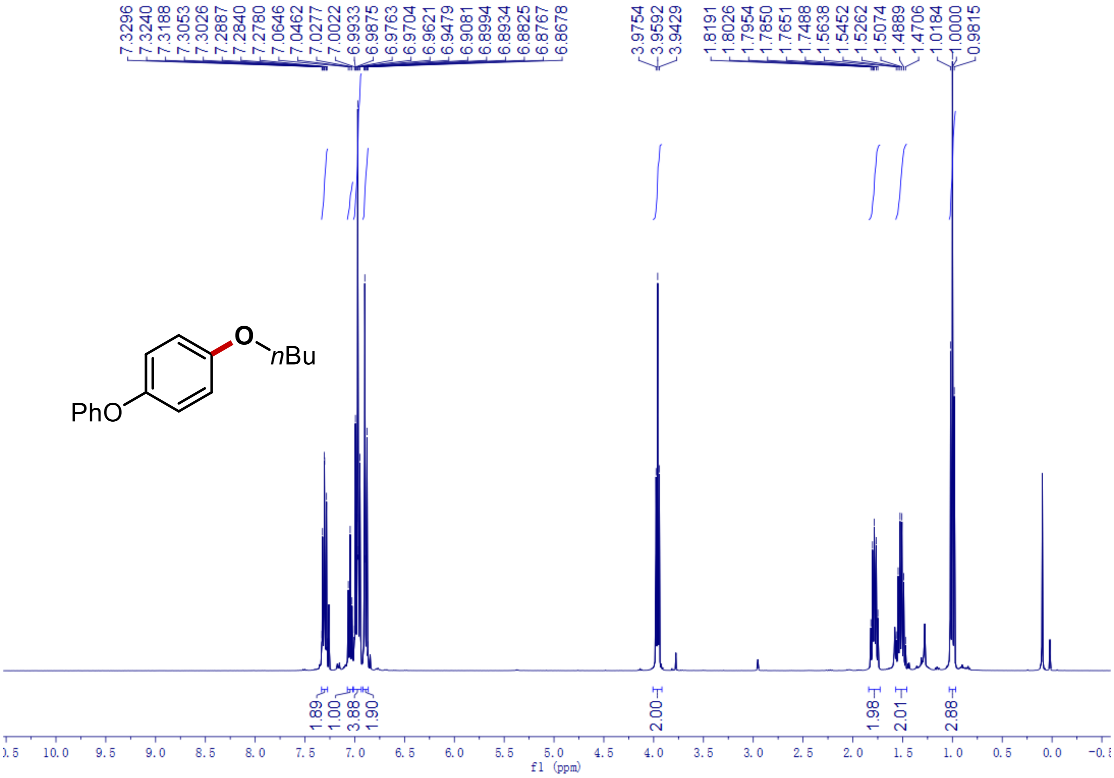

^{1}H NMR (400 MHz, $CDCl_3$) Spectrum

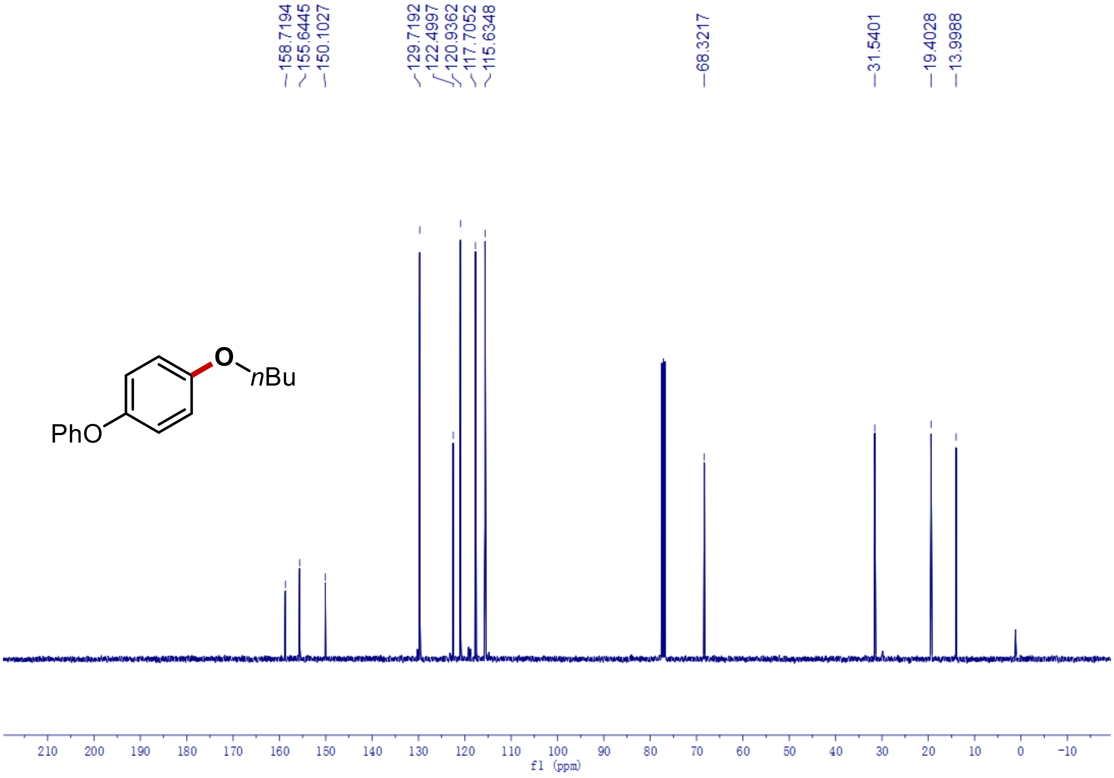

^{13}C NMR (100 MHz, $CDCl_3$) Spectrum

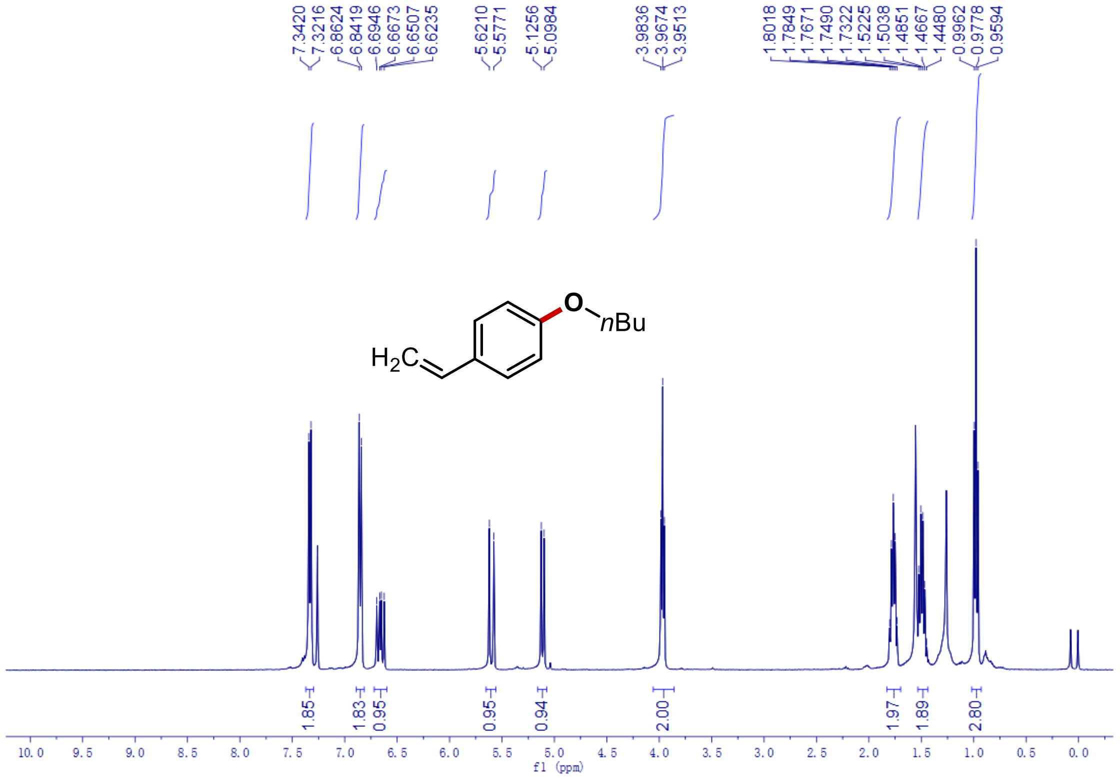

^{1}H NMR (400 MHz, $CDCl_3$) Spectrum

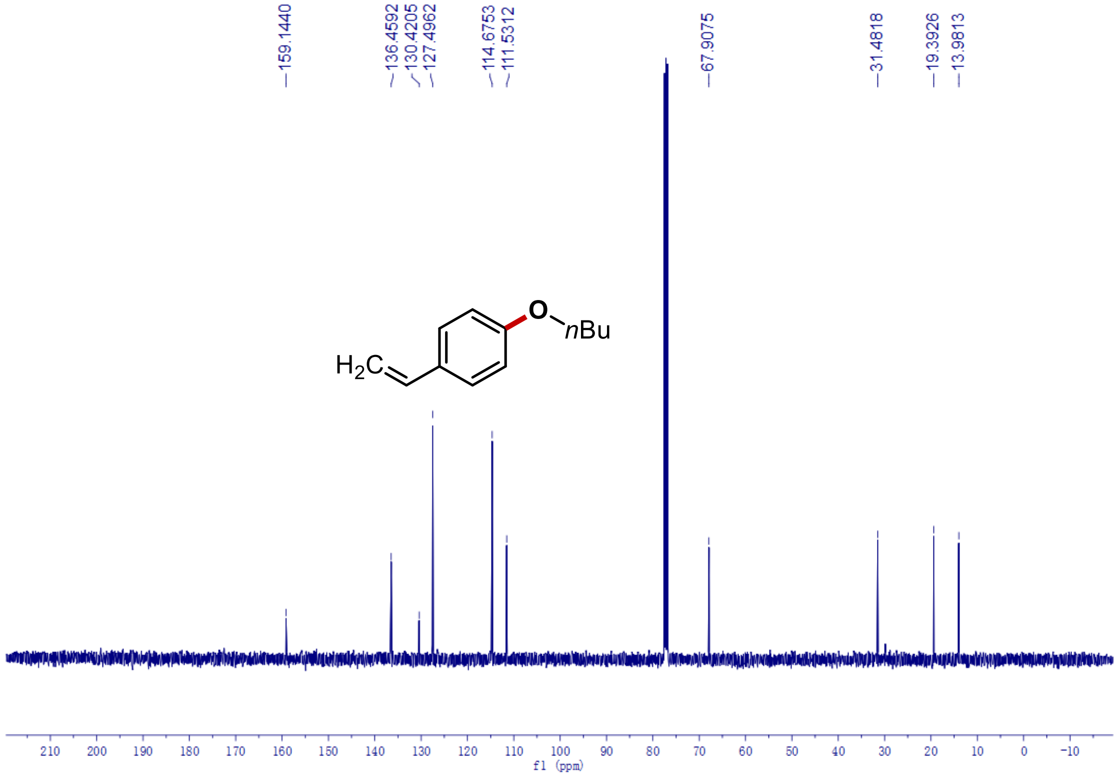

^{13}C NMR (100 MHz, $CDCl_3$) Spectrum

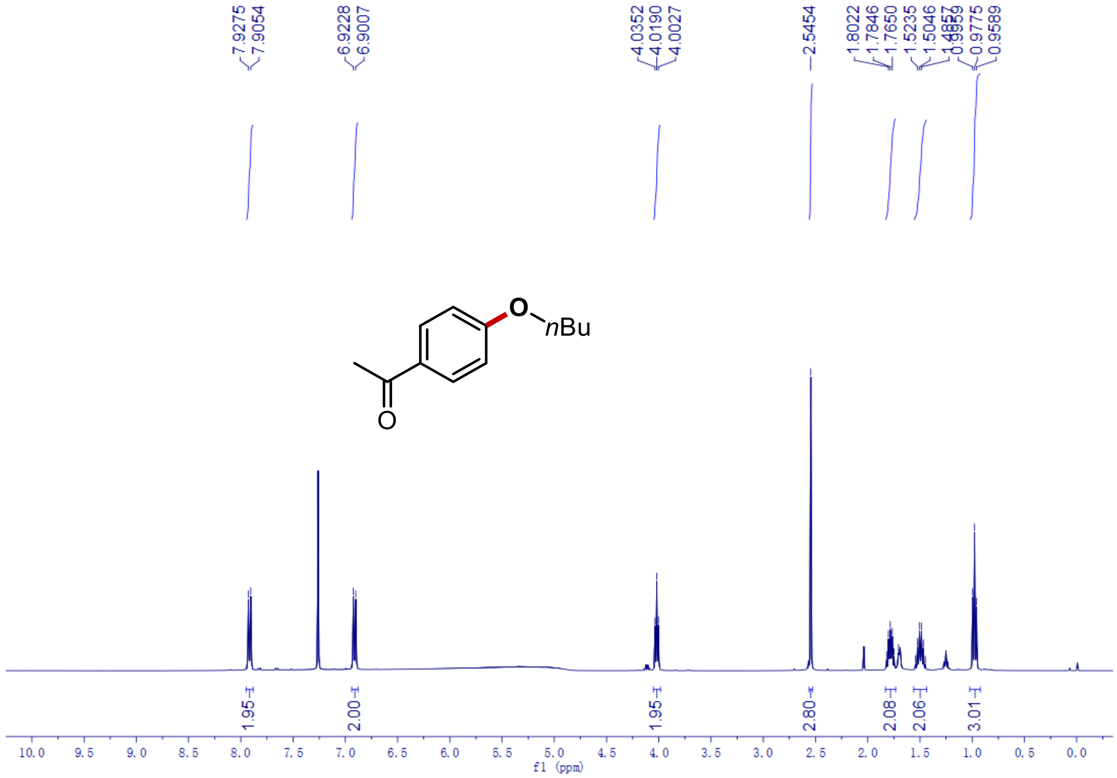

^{1}H NMR (400 MHz, $CDCl_3$) Spectrum

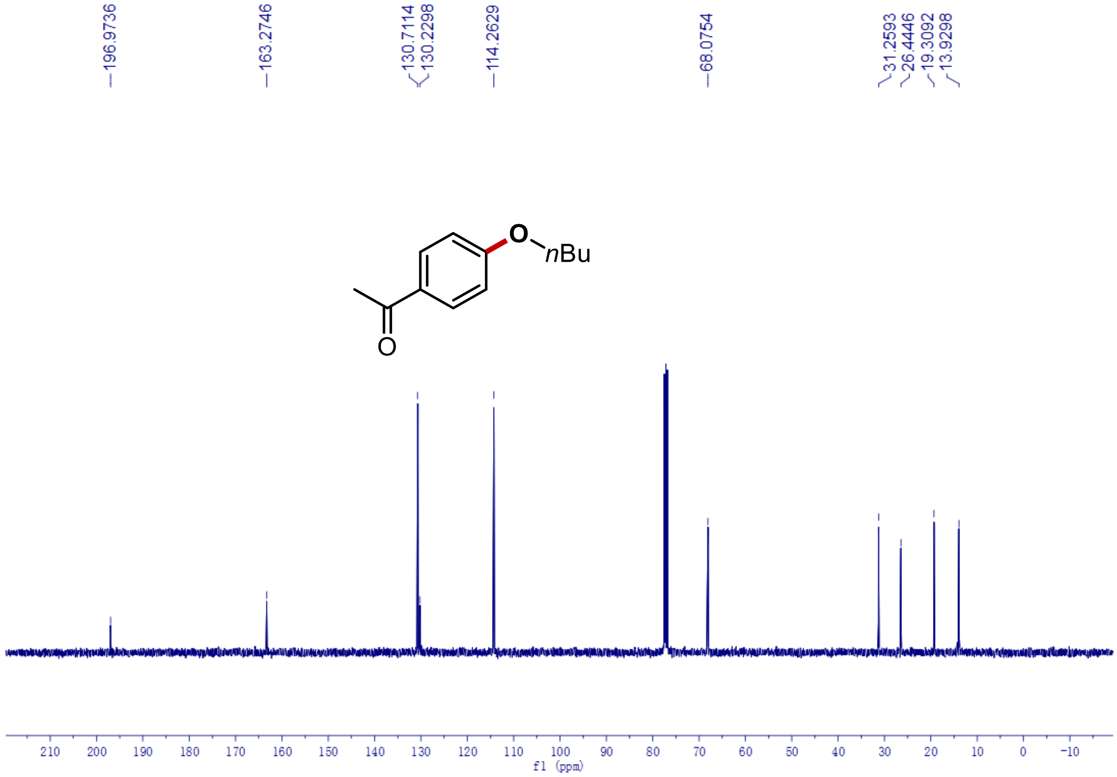

^{13}C NMR (100 MHz, $CDCl_3$) Spectrum

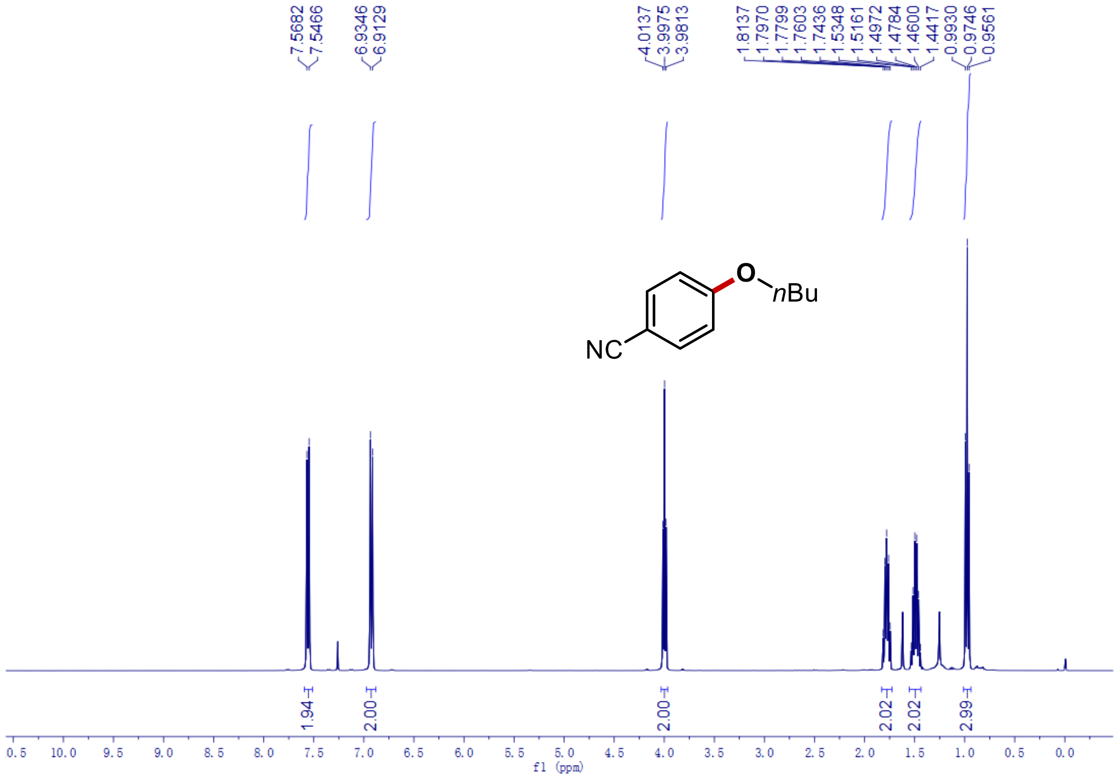

^{1}H NMR (400 MHz, $CDCl_3$) Spectrum

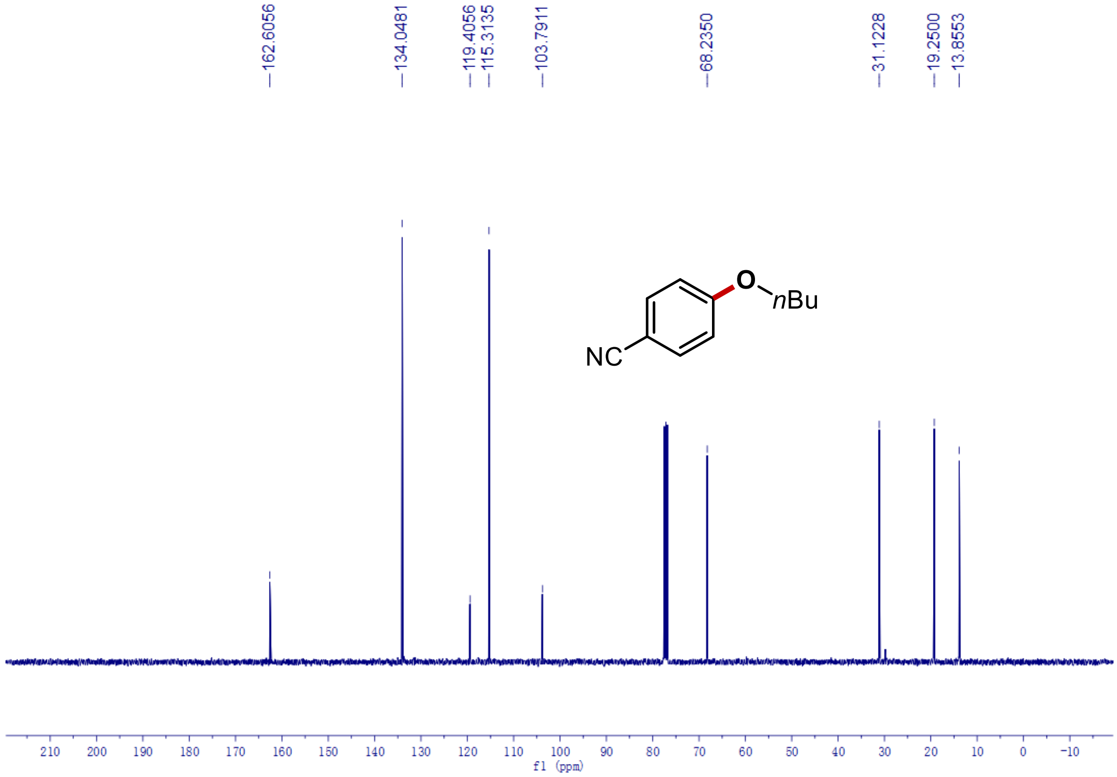

^{13}C NMR (100 MHz, $CDCl_3$) Spectrum

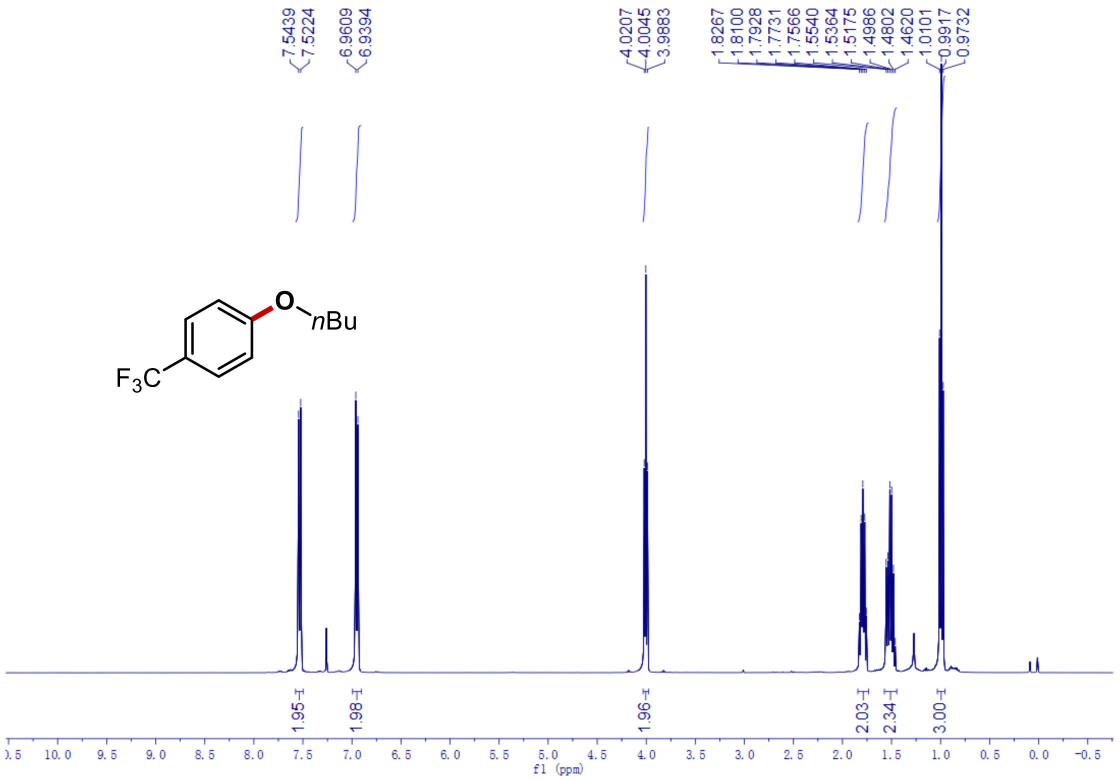

^{1}H NMR (400 MHz, $CDCl_3$) Spectrum

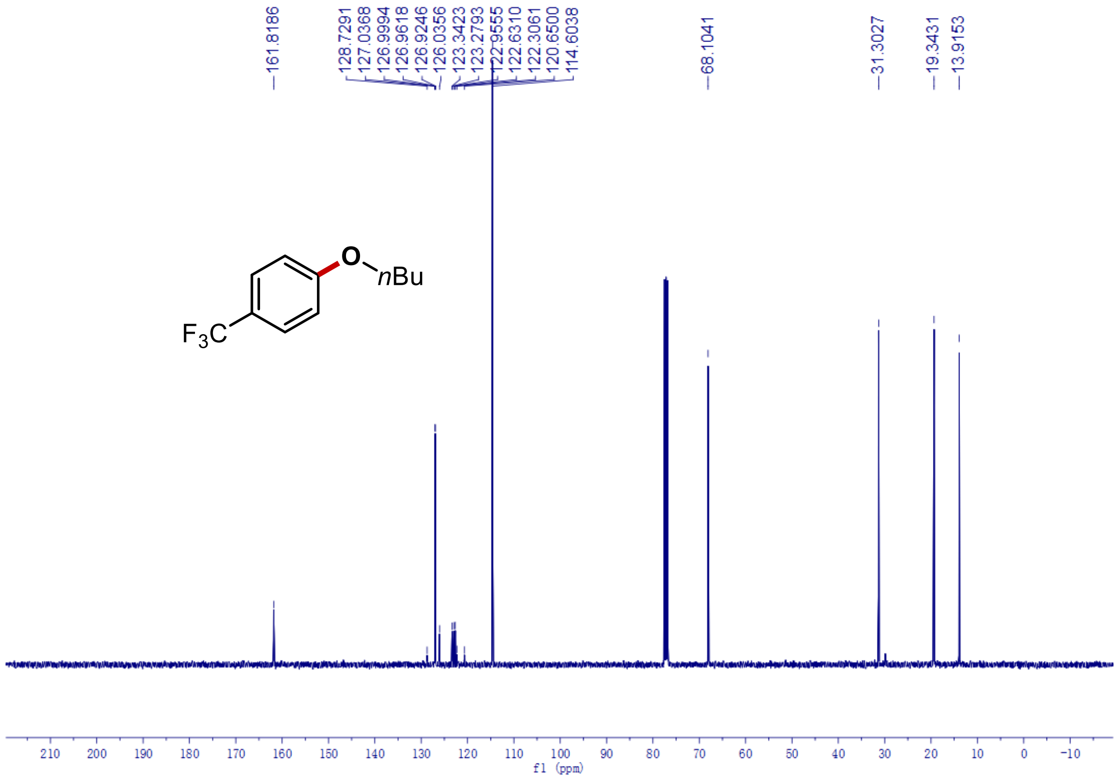

^{13}C NMR (100 MHz, $CDCl_3$) Spectrum

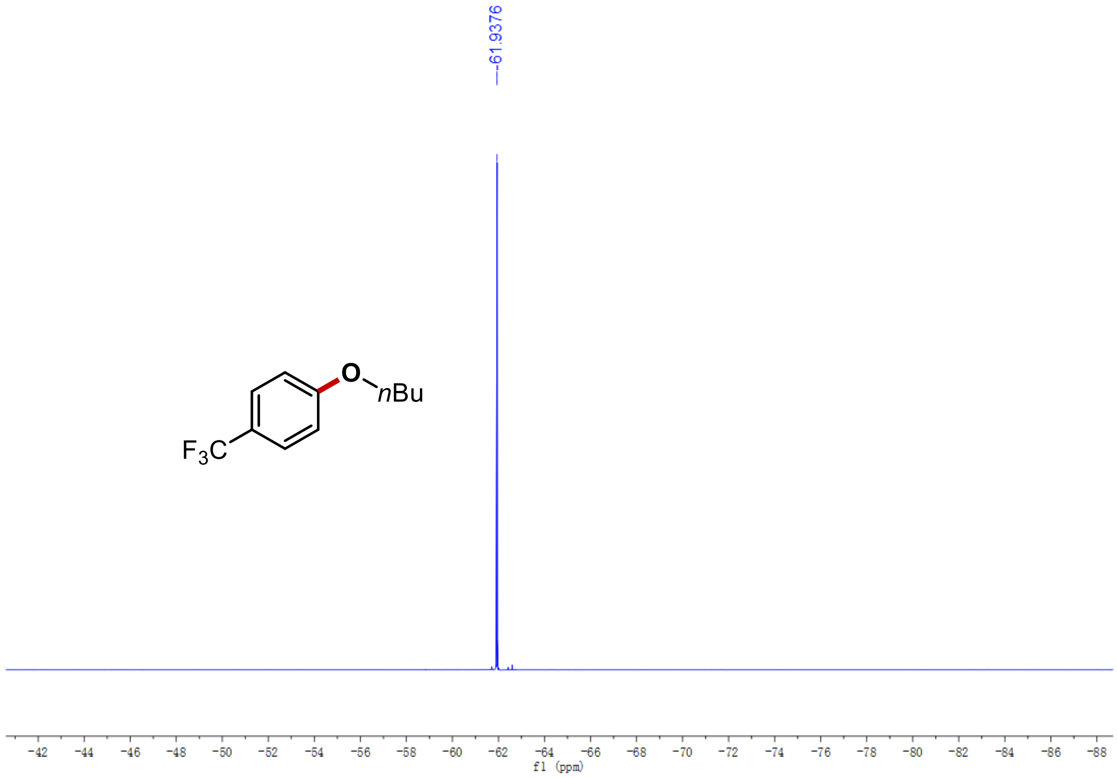

^{19}F NMR (376 MHz, $CDCl_3$) Spectrum

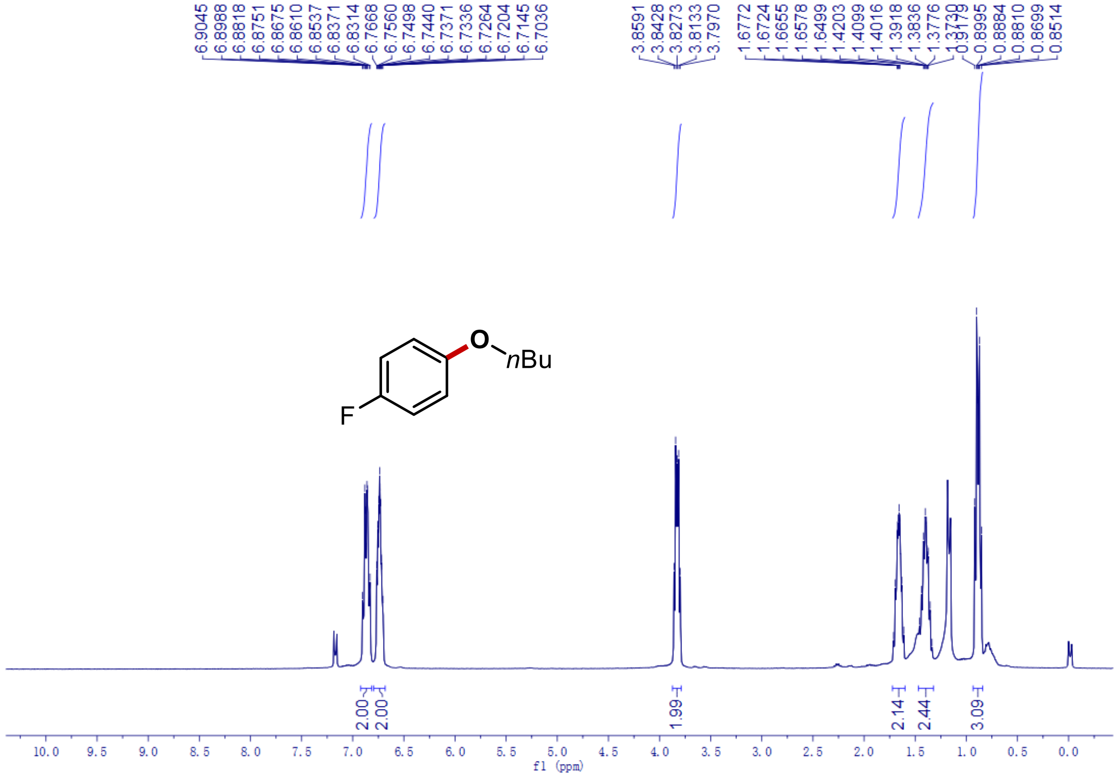

^{1}H NMR (400 MHz, $CDCl_3$) Spectrum

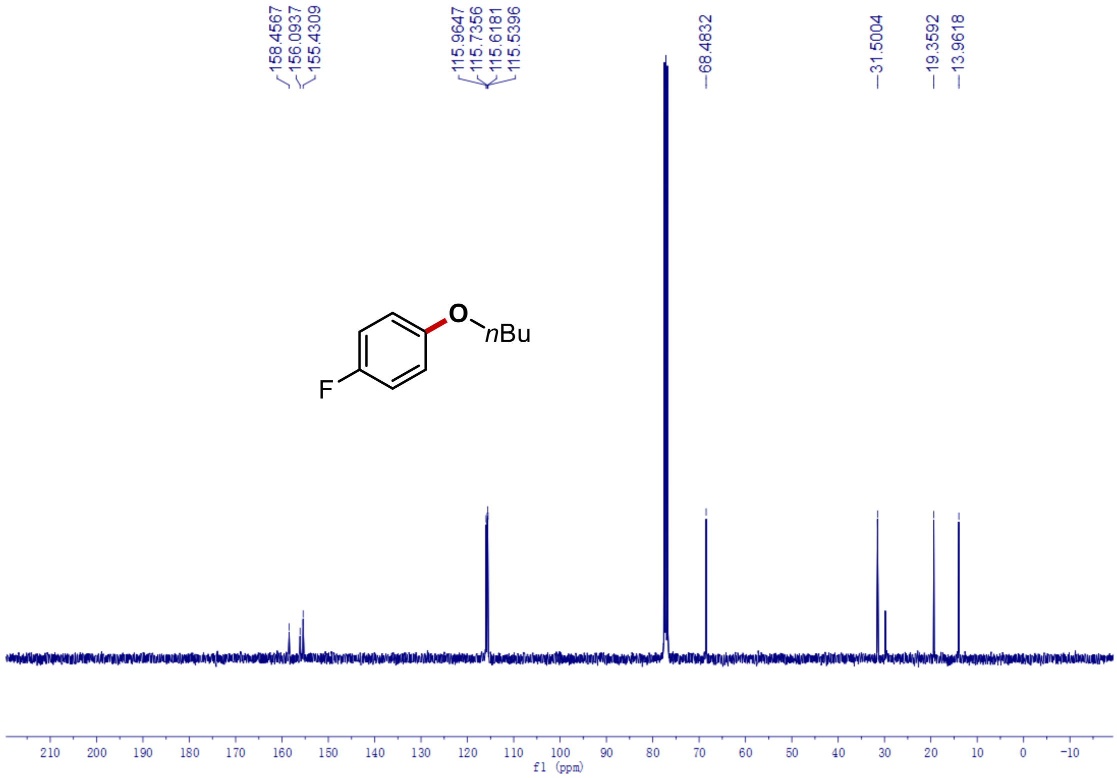

13C NMR (100 MHz, $CDCl_3$) Spectrum

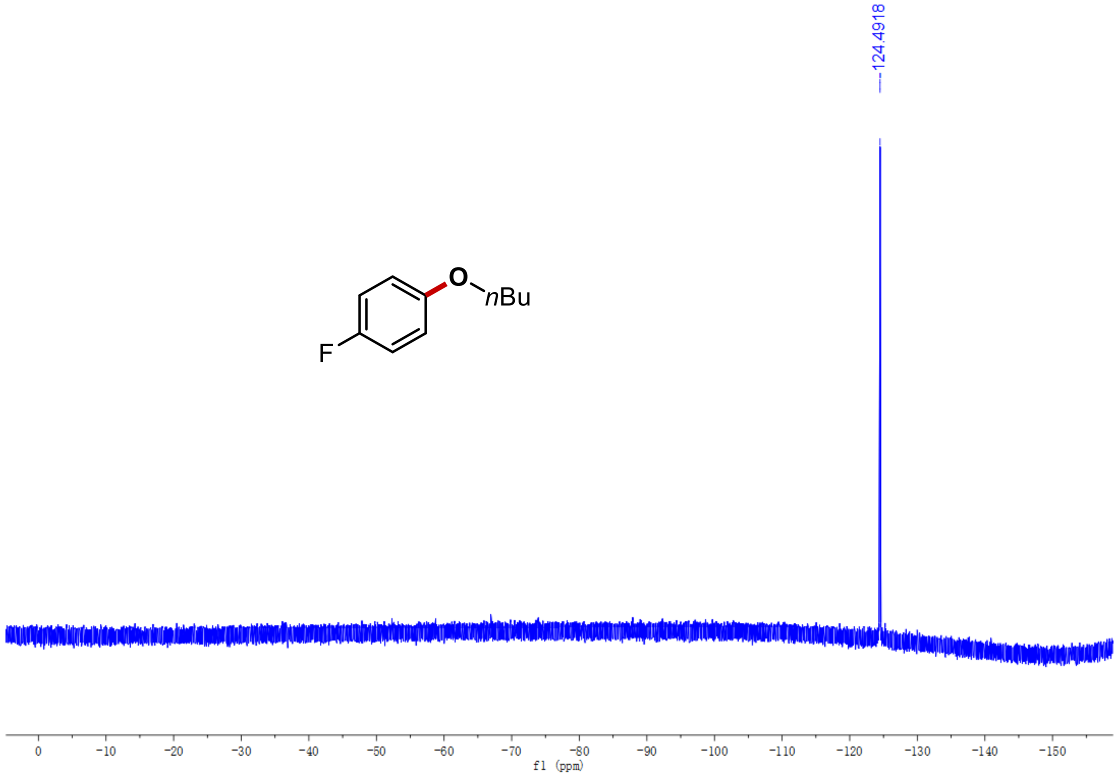

19F NMR (376 MHz, $CDCl_3$) Spectrum

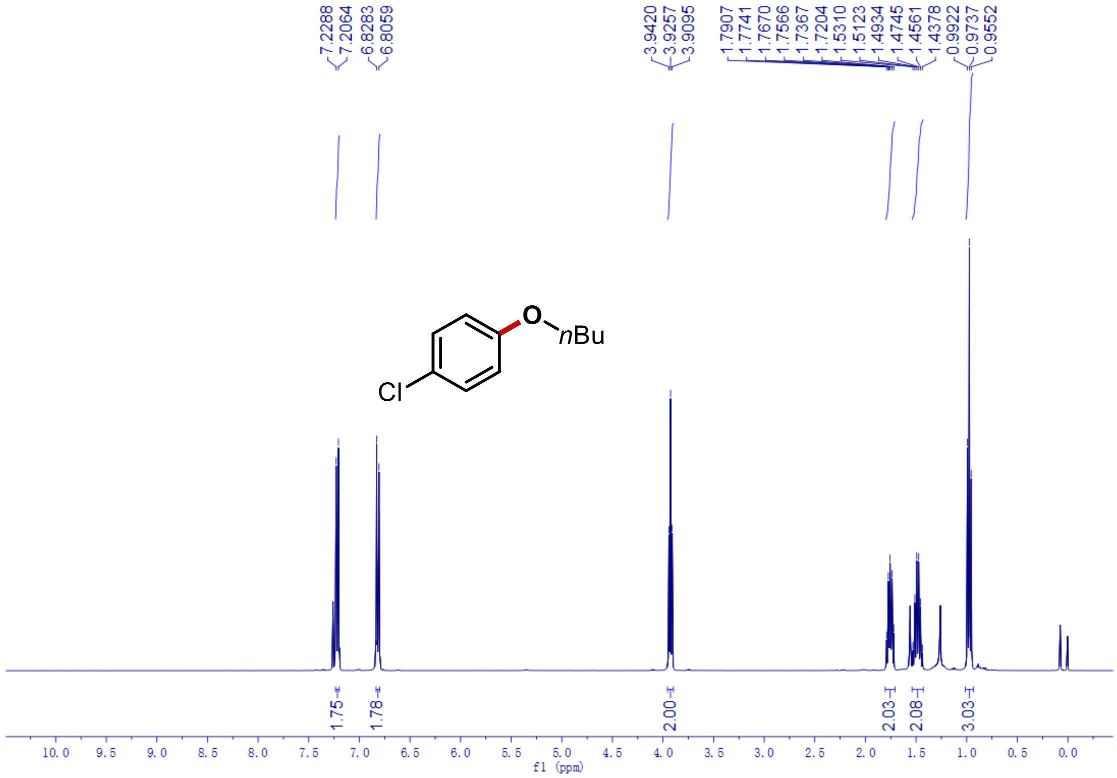

^{1}H NMR (400 MHz, $CDCl_3$) Spectrum

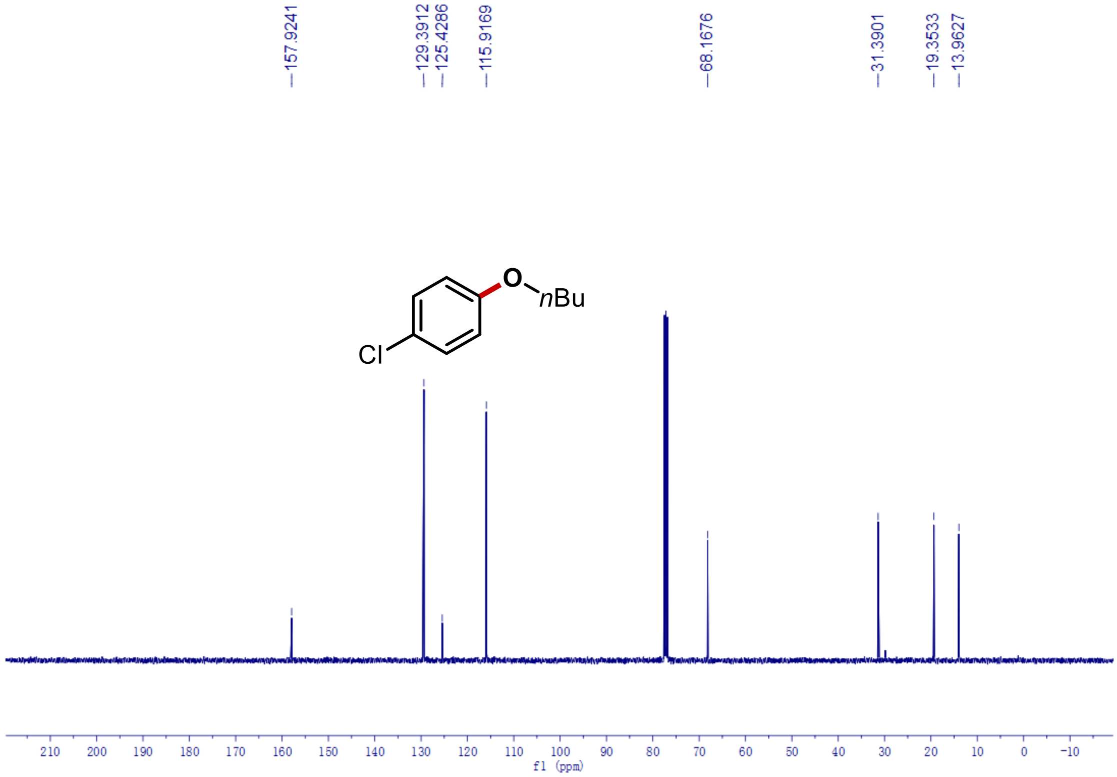

^{13}C NMR (100 MHz, $CDCl_3$) Spectrum

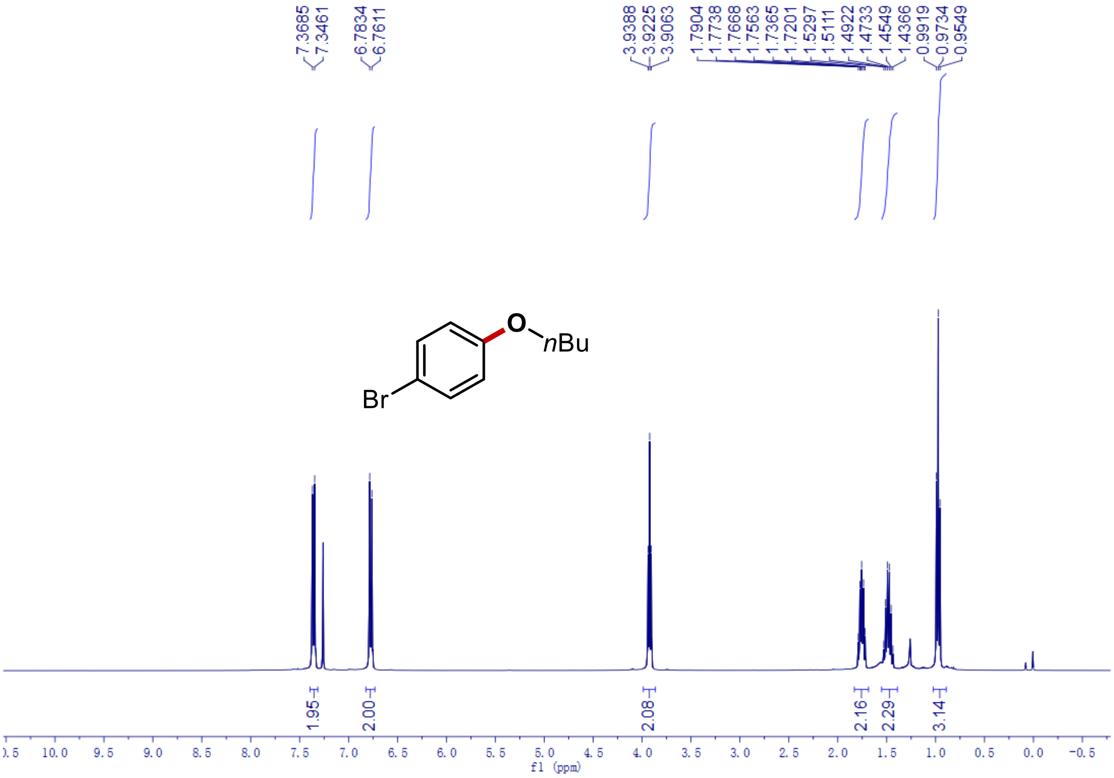

^{1}H NMR (400 MHz, $CDCl_3$) Spectrum

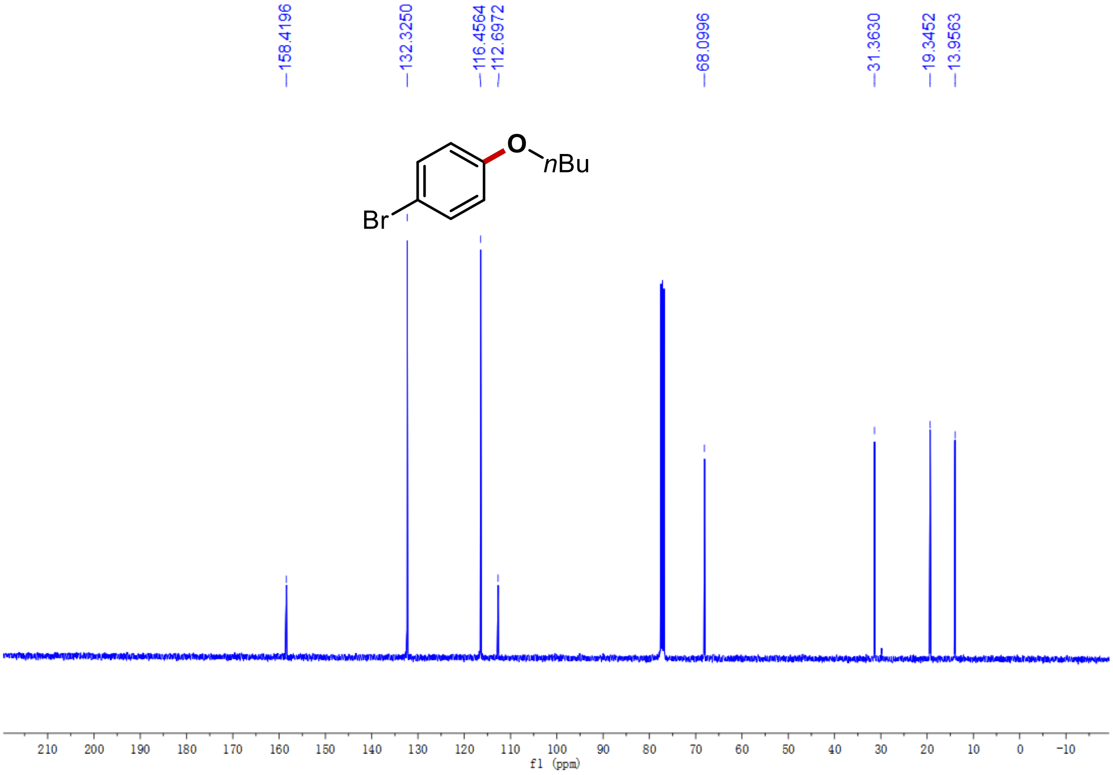

^{13}C NMR (100 MHz, $CDCl_3$) Spectrum

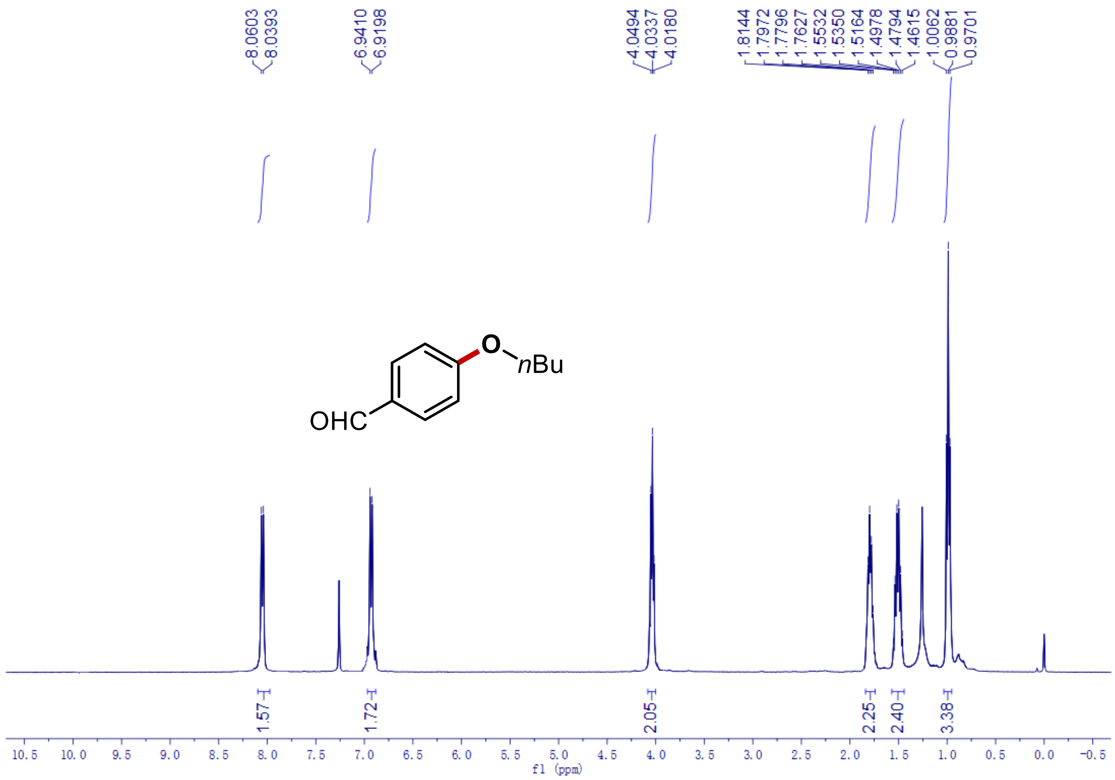

¹H NMR (400 MHz, $CDCl_3$) Spectrum

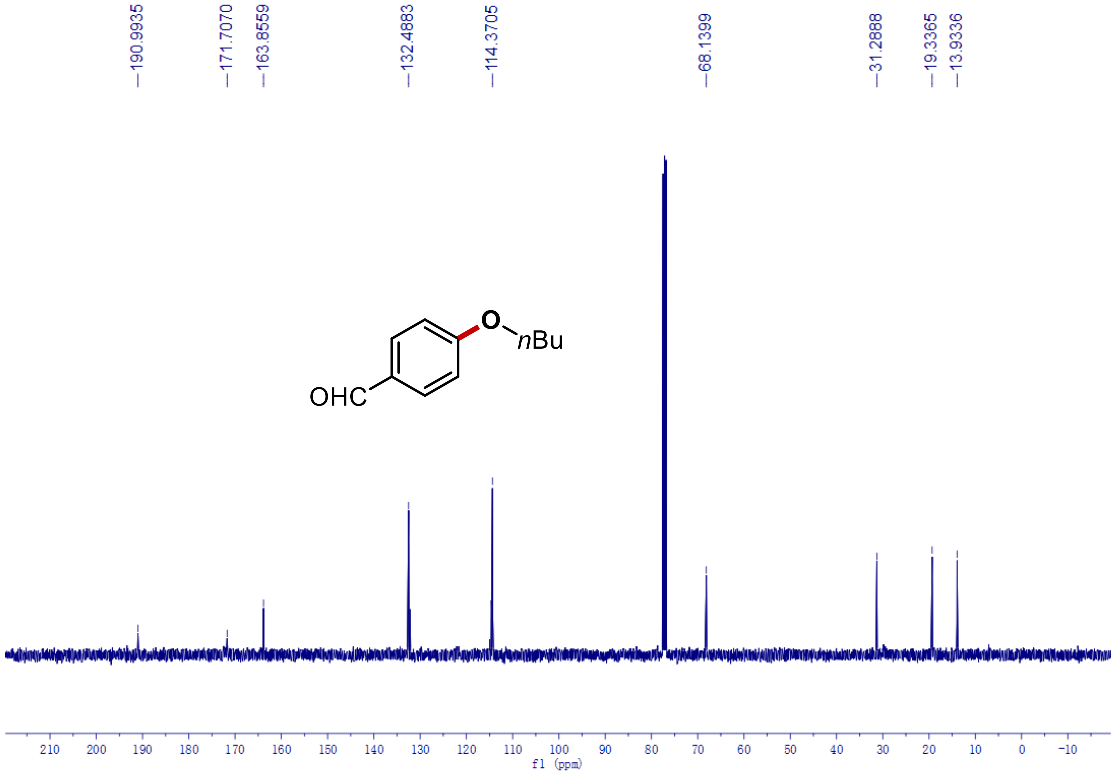

¹³C NMR (100 MHz, $CDCl_3$) Spectrum

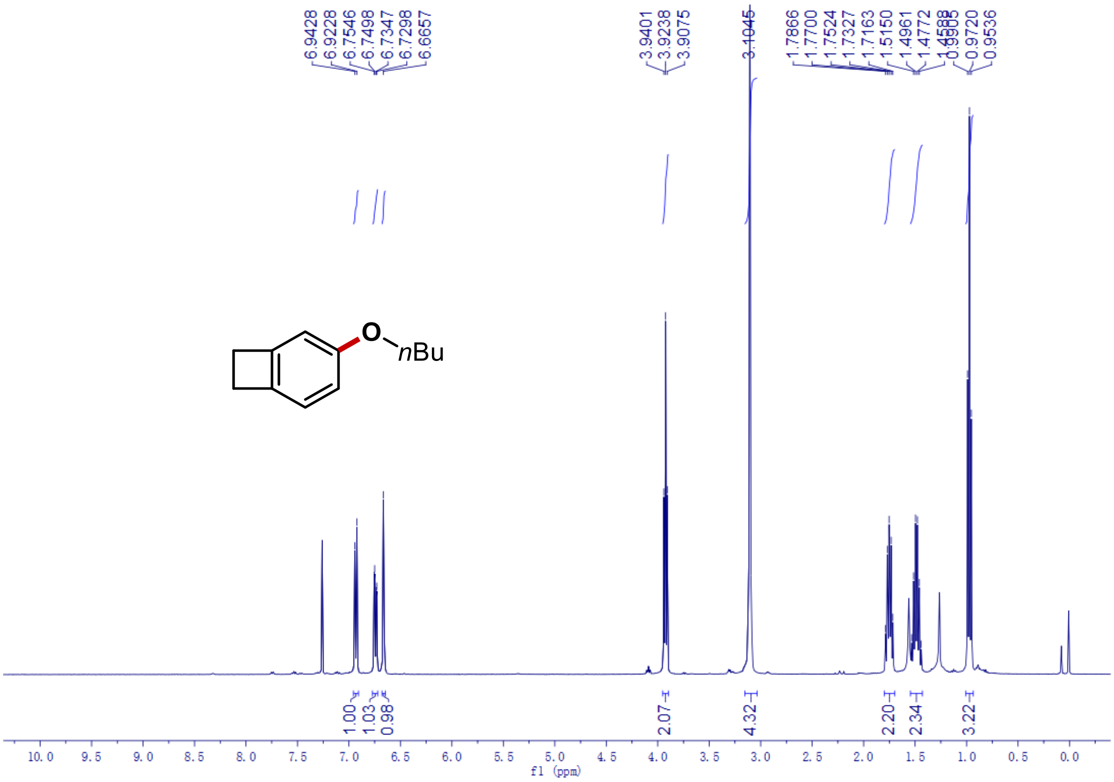

^{1}H NMR (400 MHz, $CDCl_3$) Spectrum

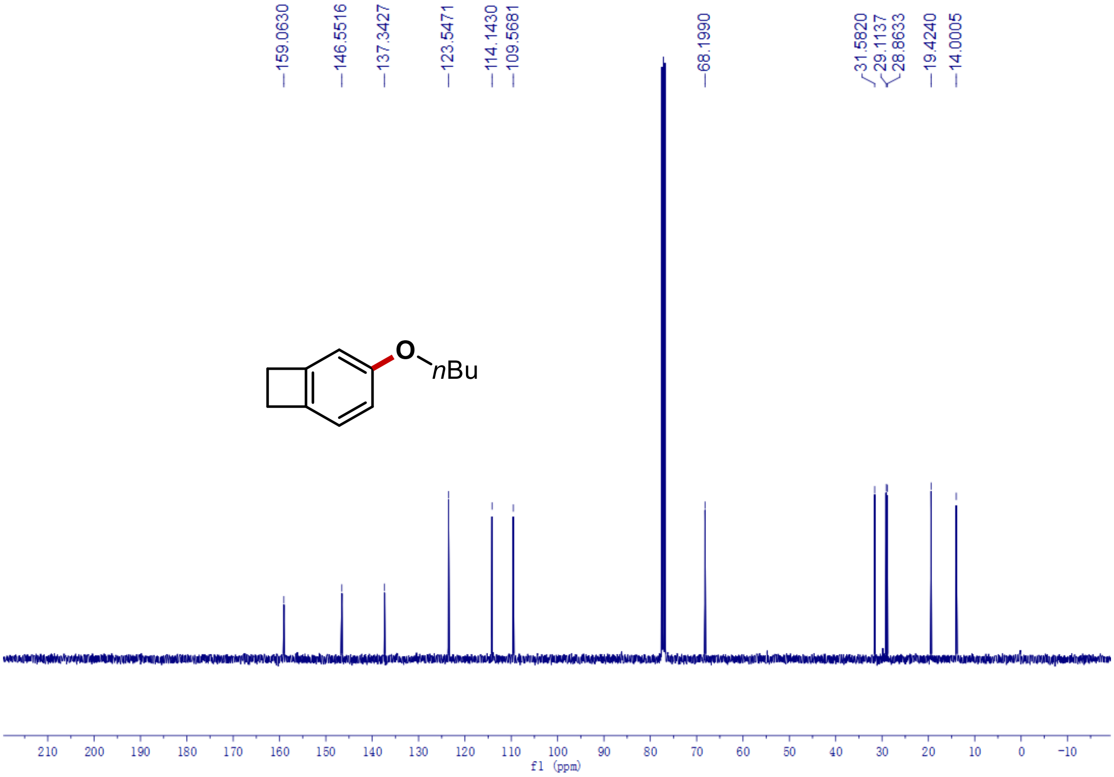

^{13}C NMR (100 MHz, $CDCl_3$) Spectrum

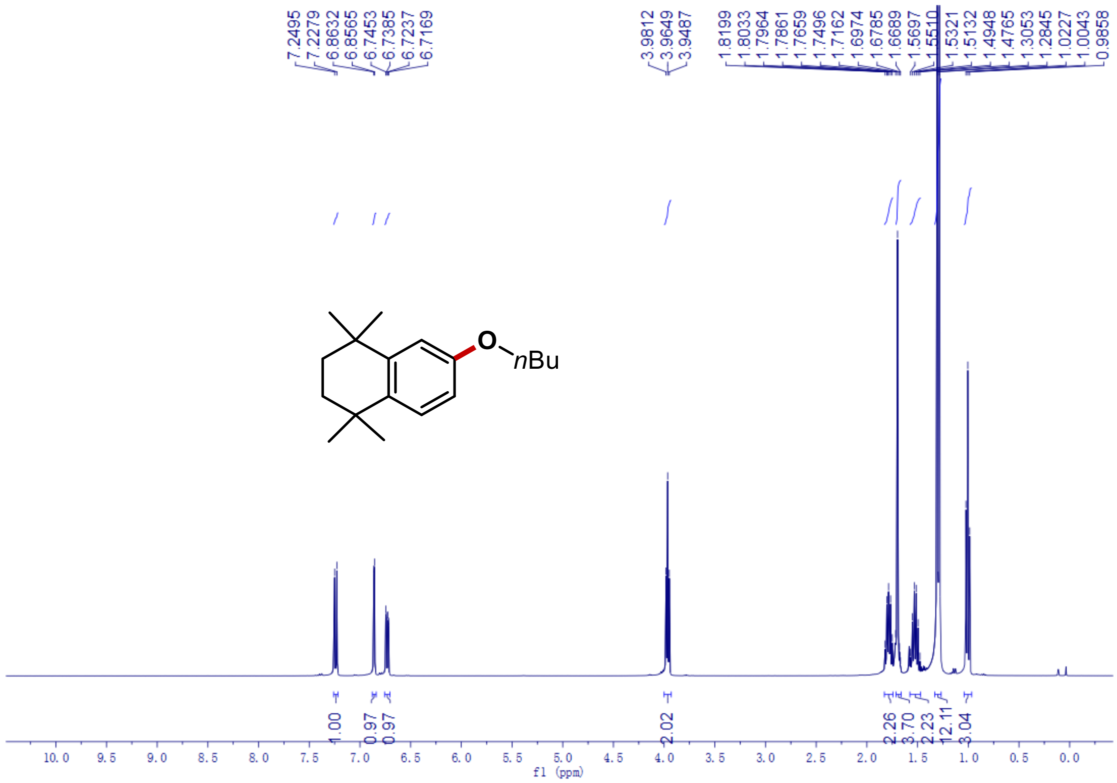

^{1}H NMR (400 MHz, $CDCl_3$) Spectrum

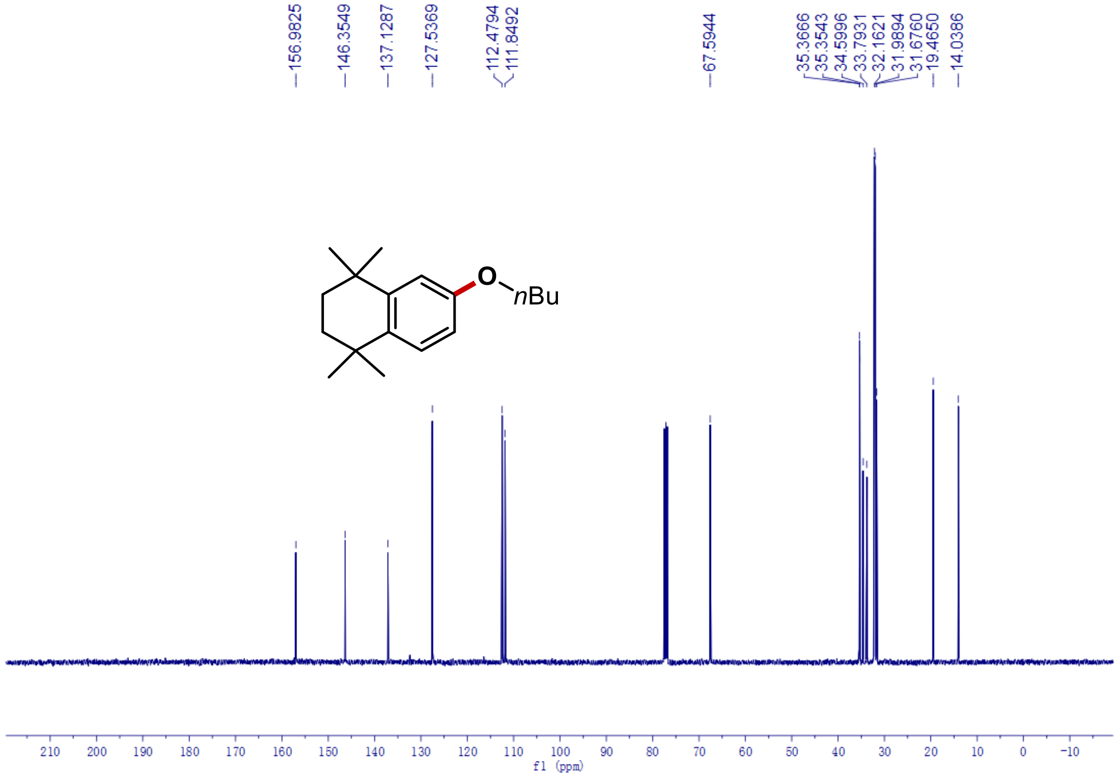

^{13}C NMR (100 MHz, $CDCl_3$) Spectrum

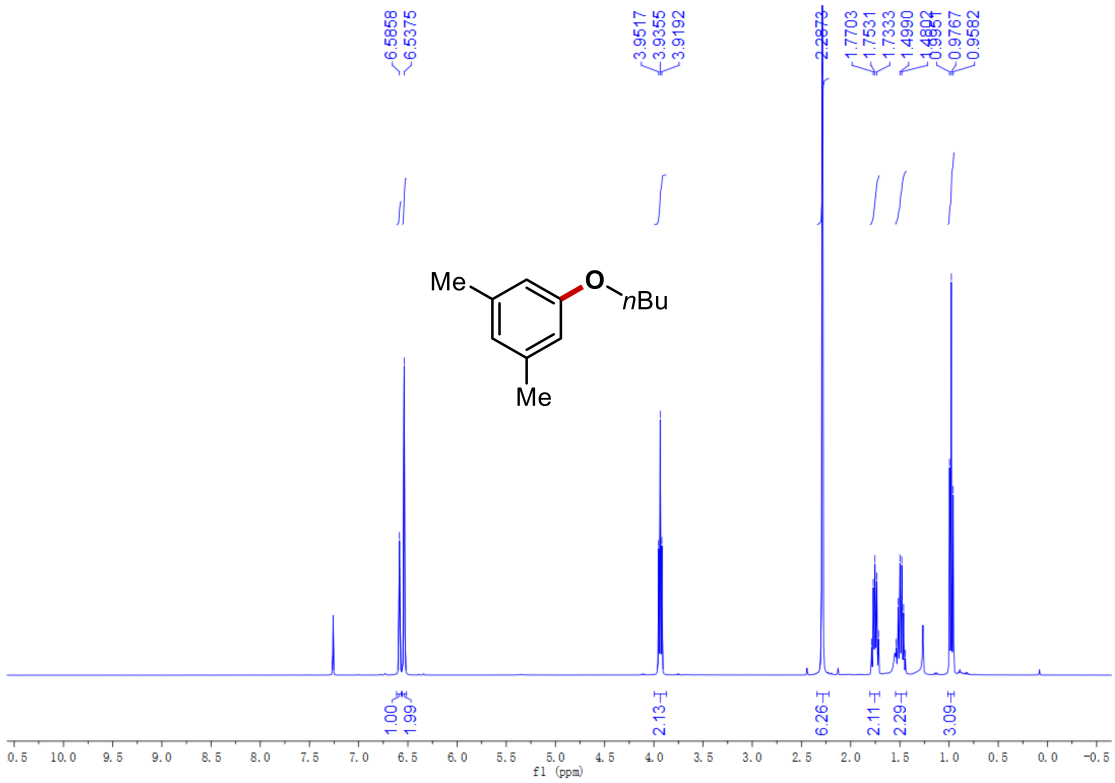

1H NMR (400 MHz, CDCl3) Spectrum

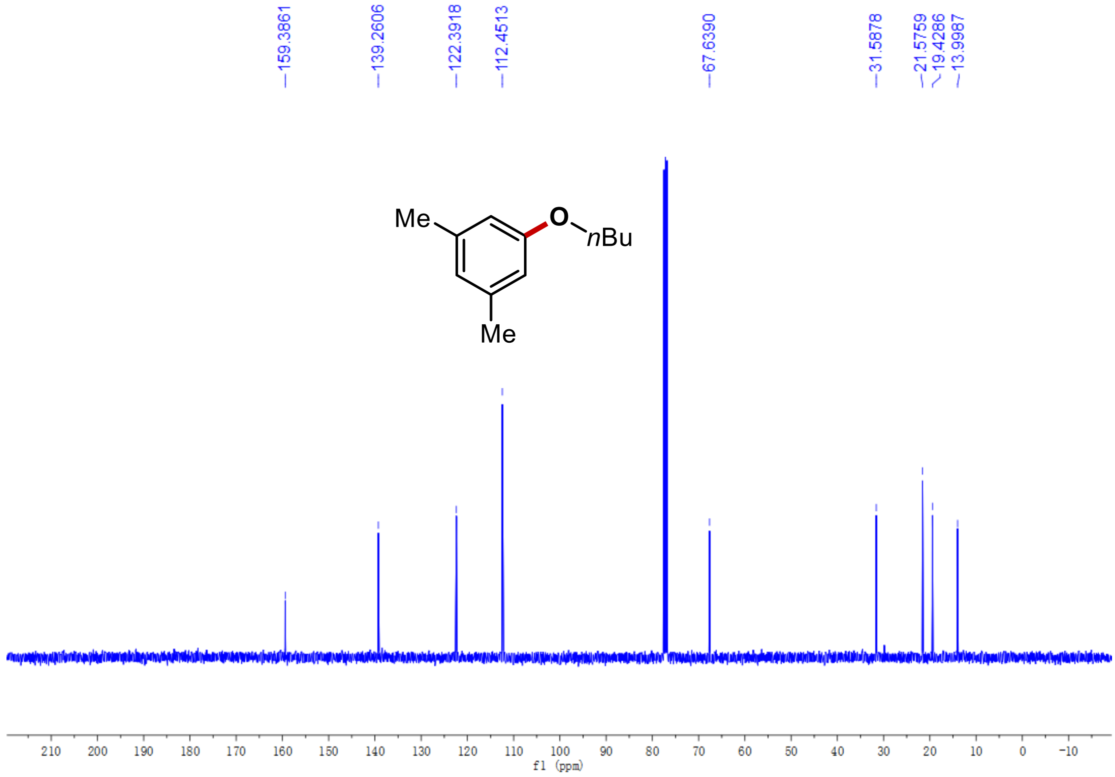

13C NMR (100 MHz, CDCl3) Spectrum

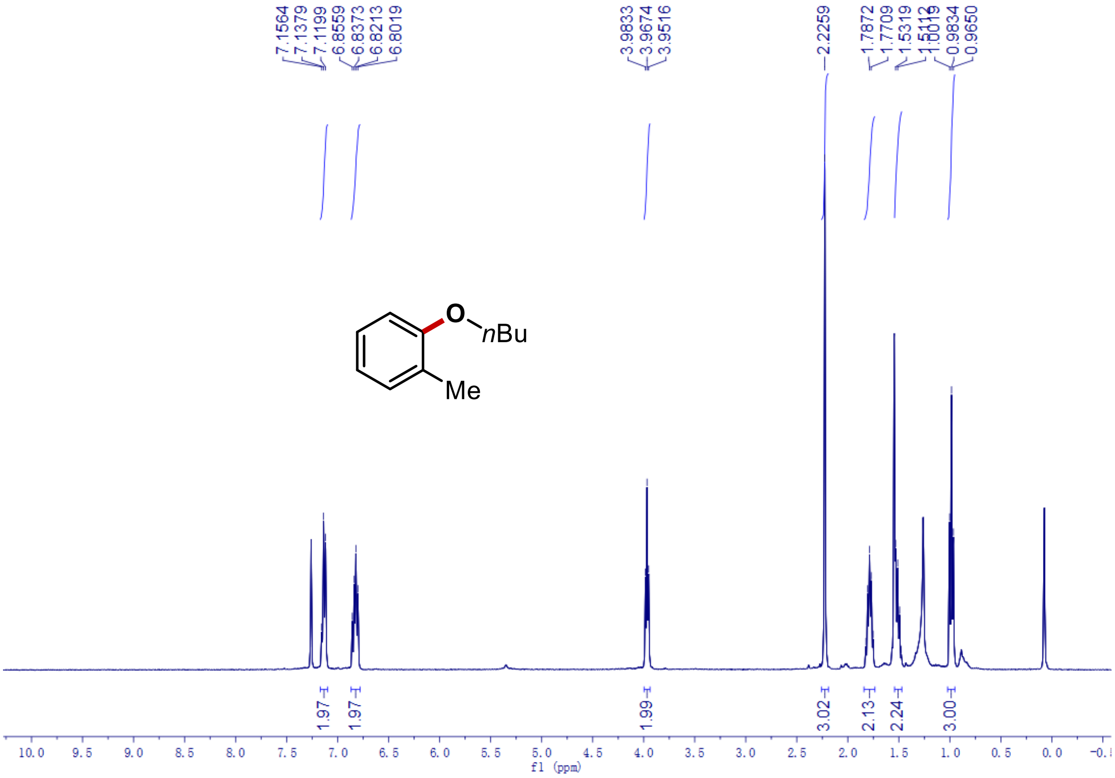

^{1}H NMR (400 MHz, $CDCl_3$) Spectrum

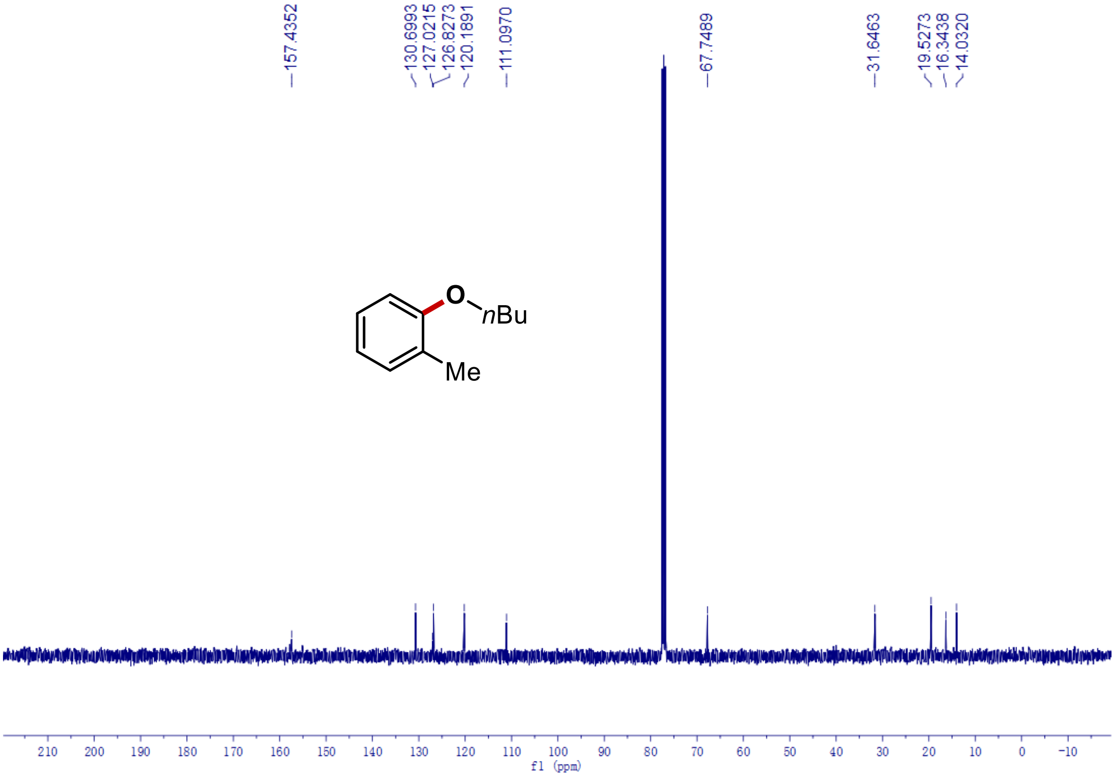

^{13}C NMR (100 MHz, $CDCl_3$) Spectrum

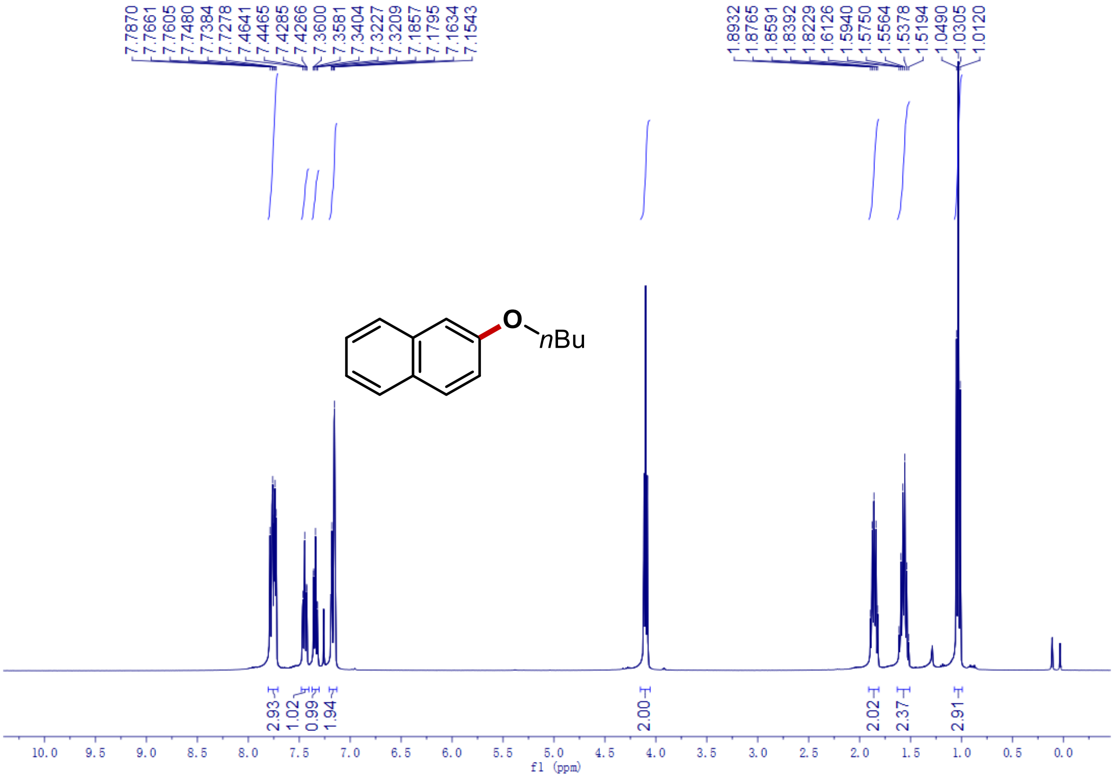

^{1}H NMR (400 MHz, $CDCl_3$) Spectrum

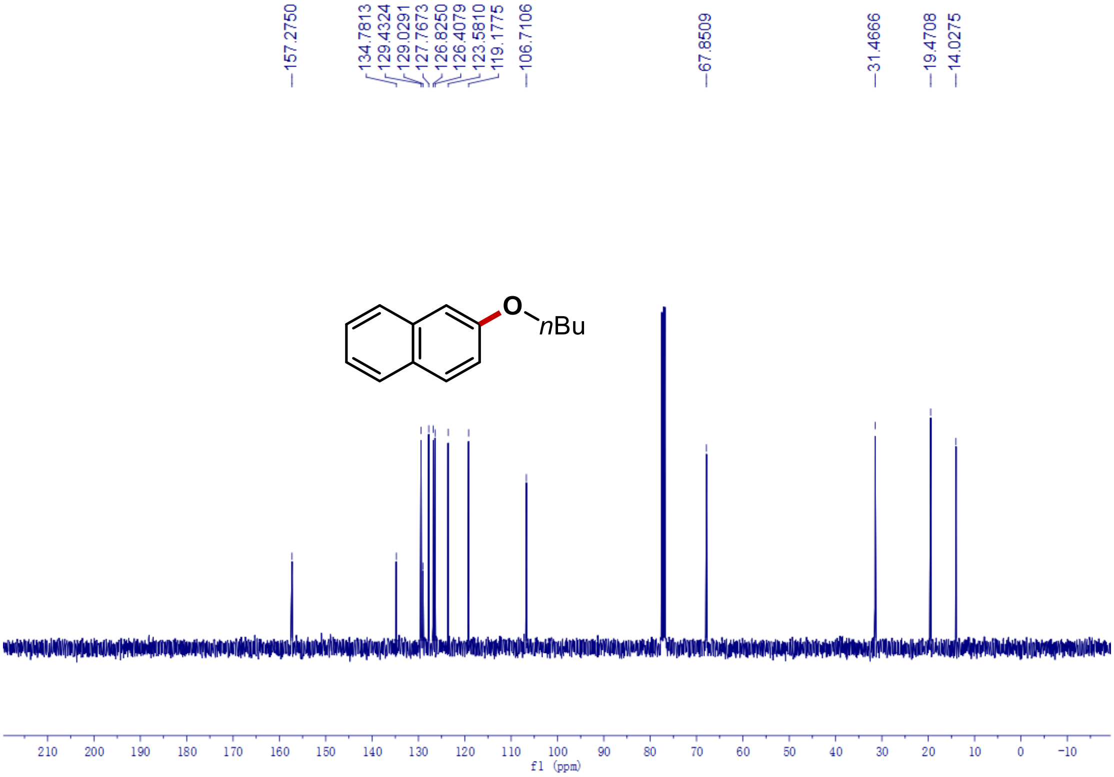

^{13}C NMR (100 MHz, $CDCl_3$) Spectrum

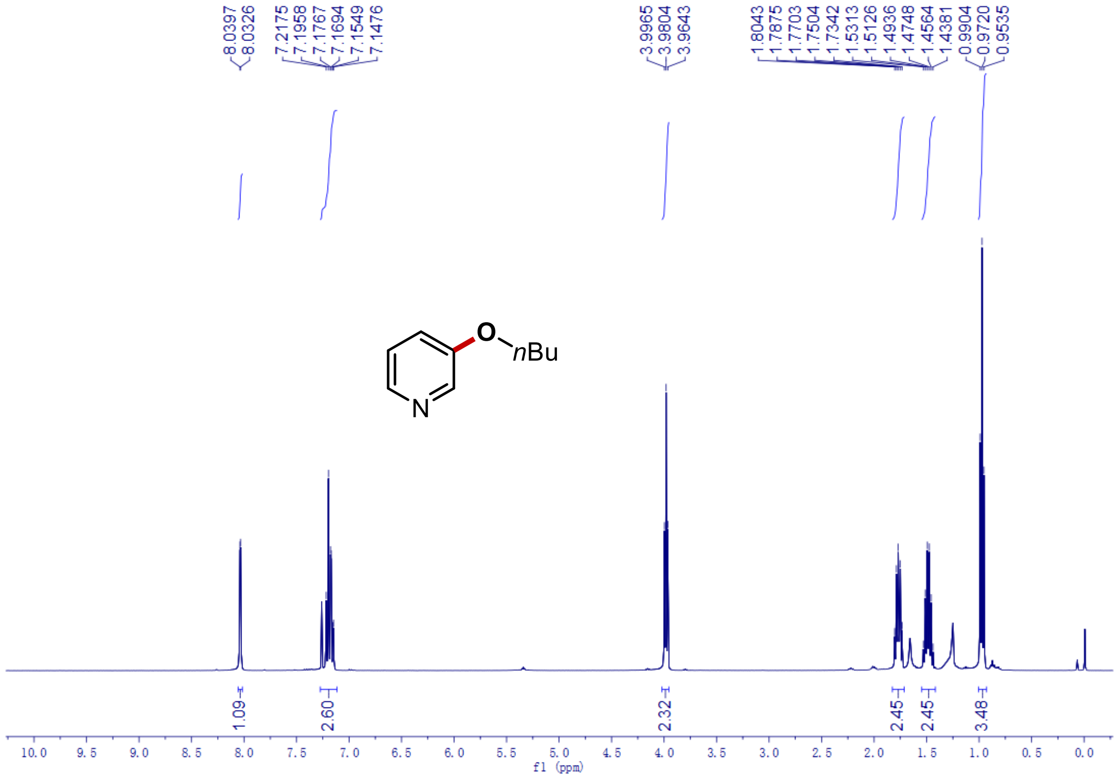

^{1}H NMR (400 MHz, $CDCl_3$) Spectrum

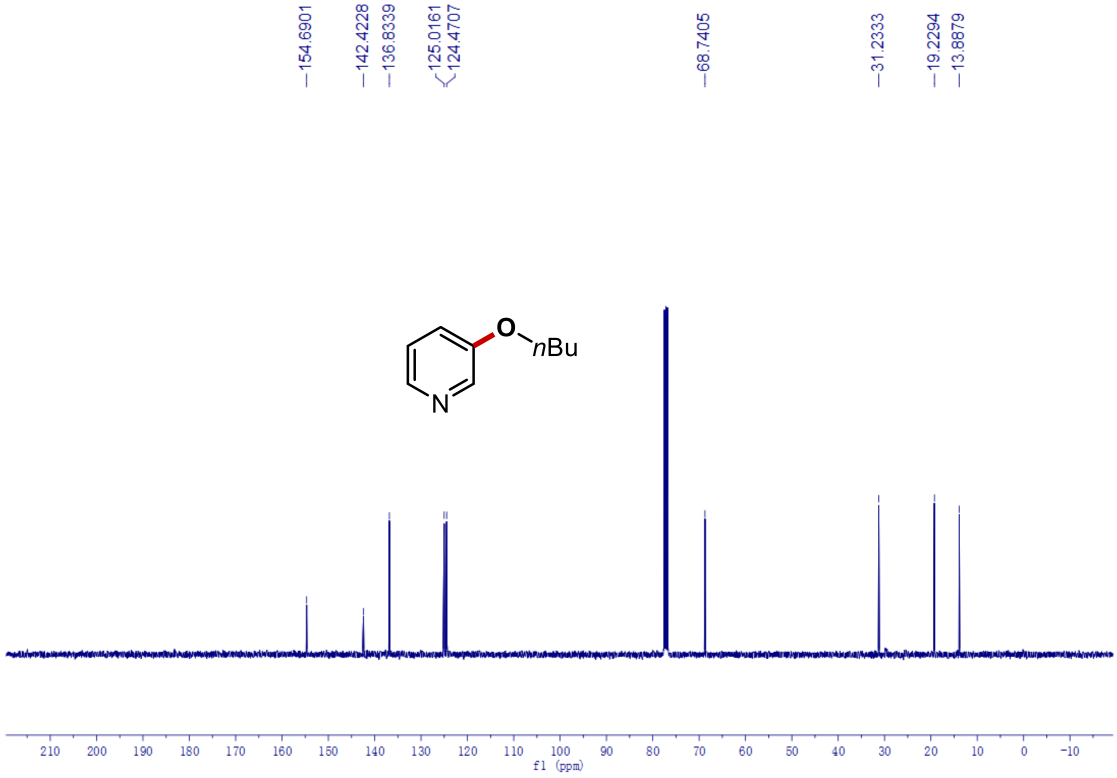

^{13}C NMR (100 MHz, $CDCl_3$) Spectrum

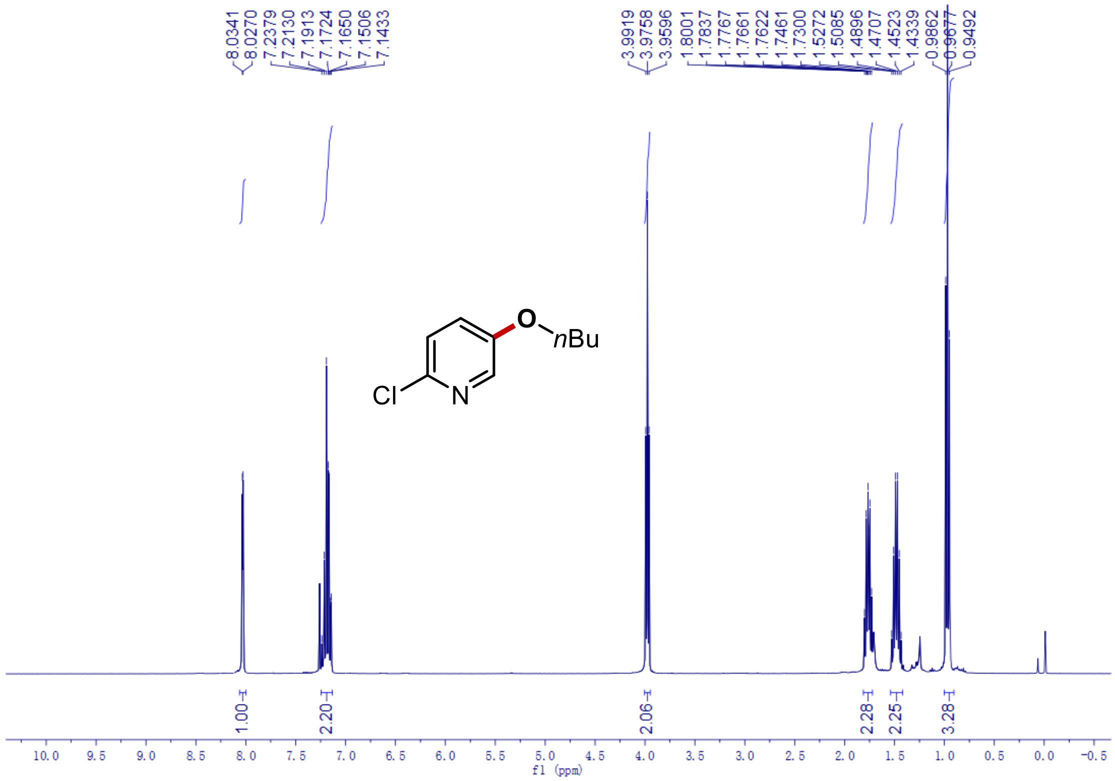

^{1}H NMR (400 MHz, $CDCl_3$) Spectrum

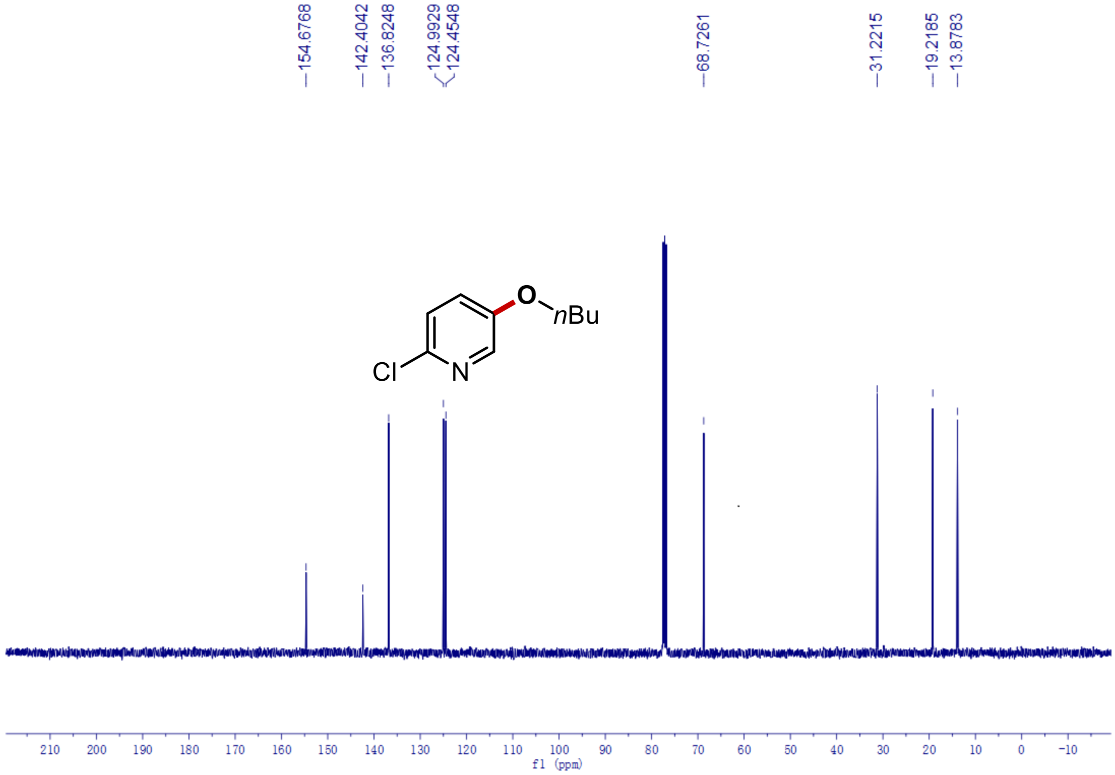

^{13}C NMR (100 MHz, $CDCl_3$) Spectrum

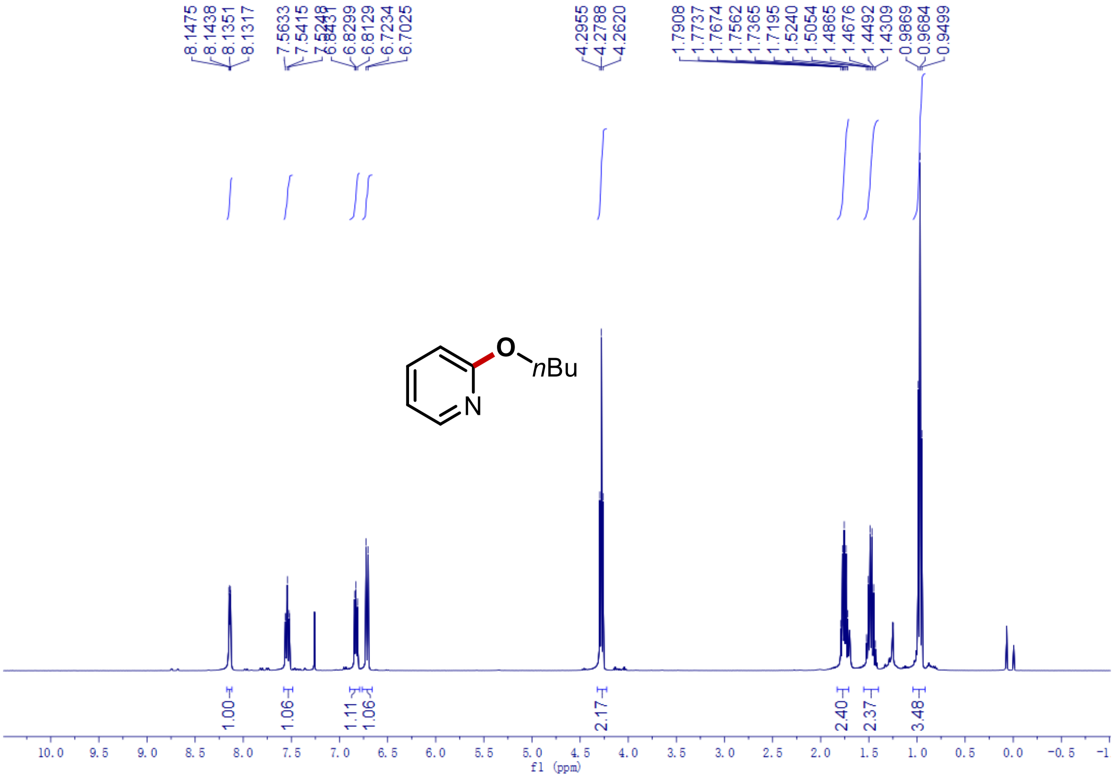

^{1}H NMR (400 MHz, $CDCl_3$) Spectrum

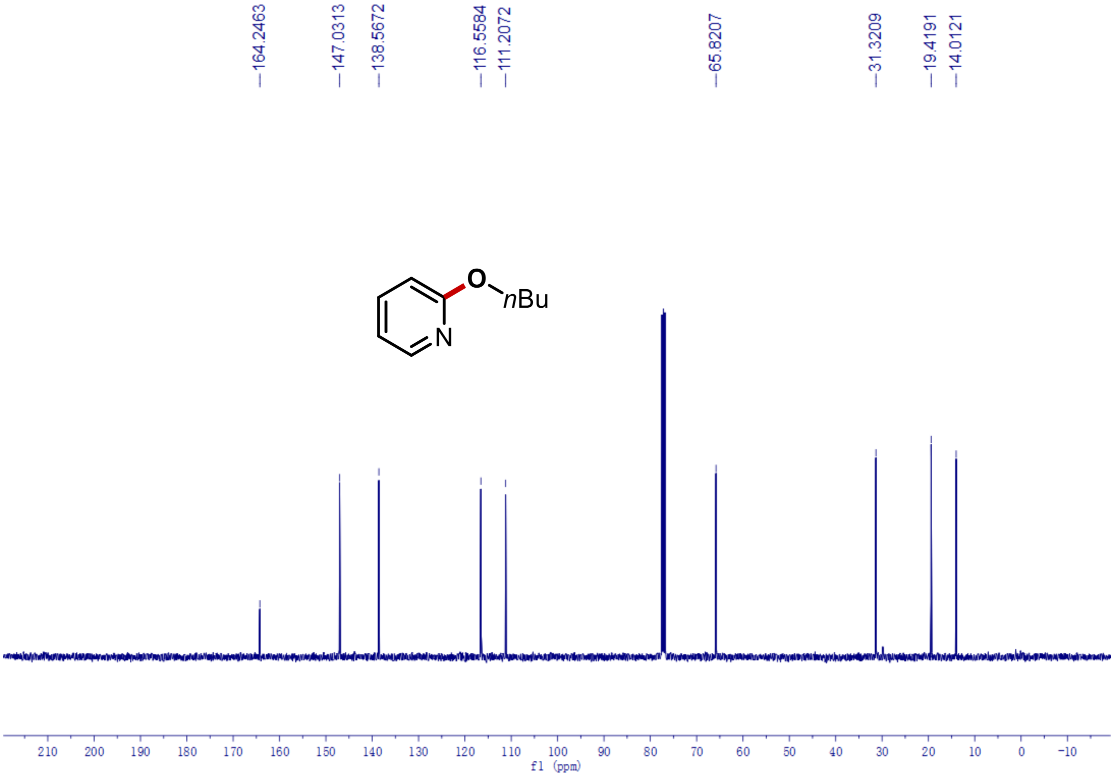

^{13}C NMR (100 MHz, $CDCl_3$) Spectrum

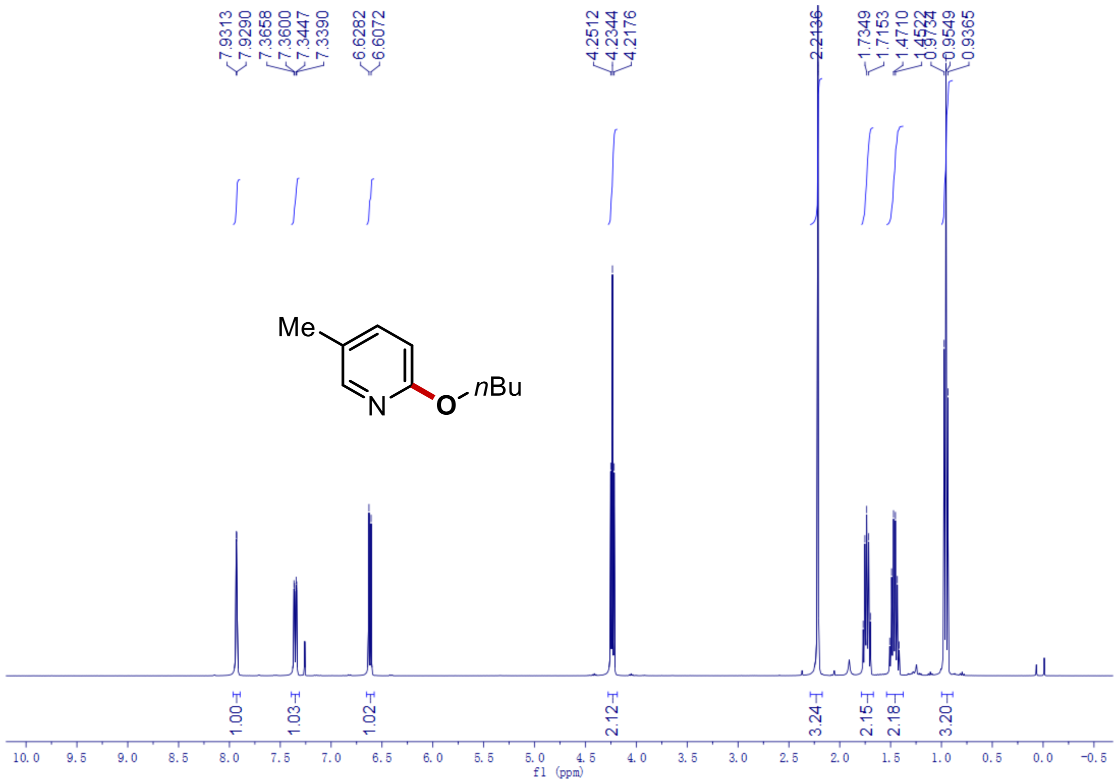

^{1}H NMR (400 MHz, $CDCl_3$) Spectrum

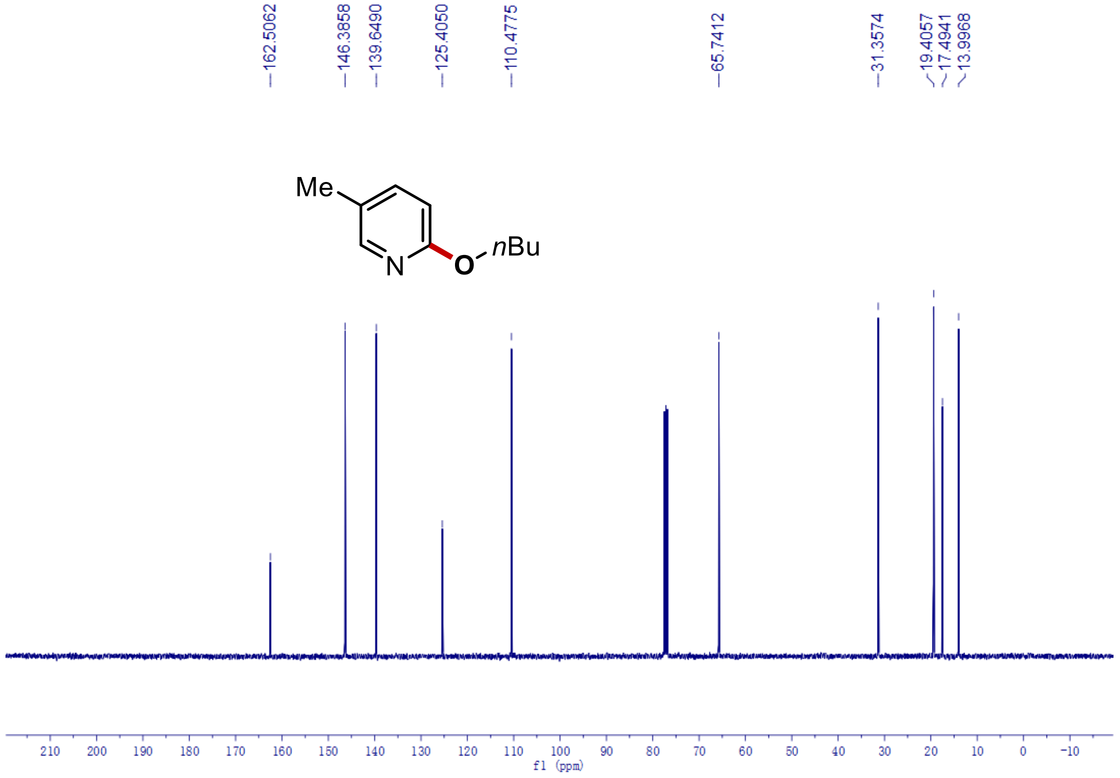

^{13}C NMR (100 MHz, $CDCl_3$) Spectrum

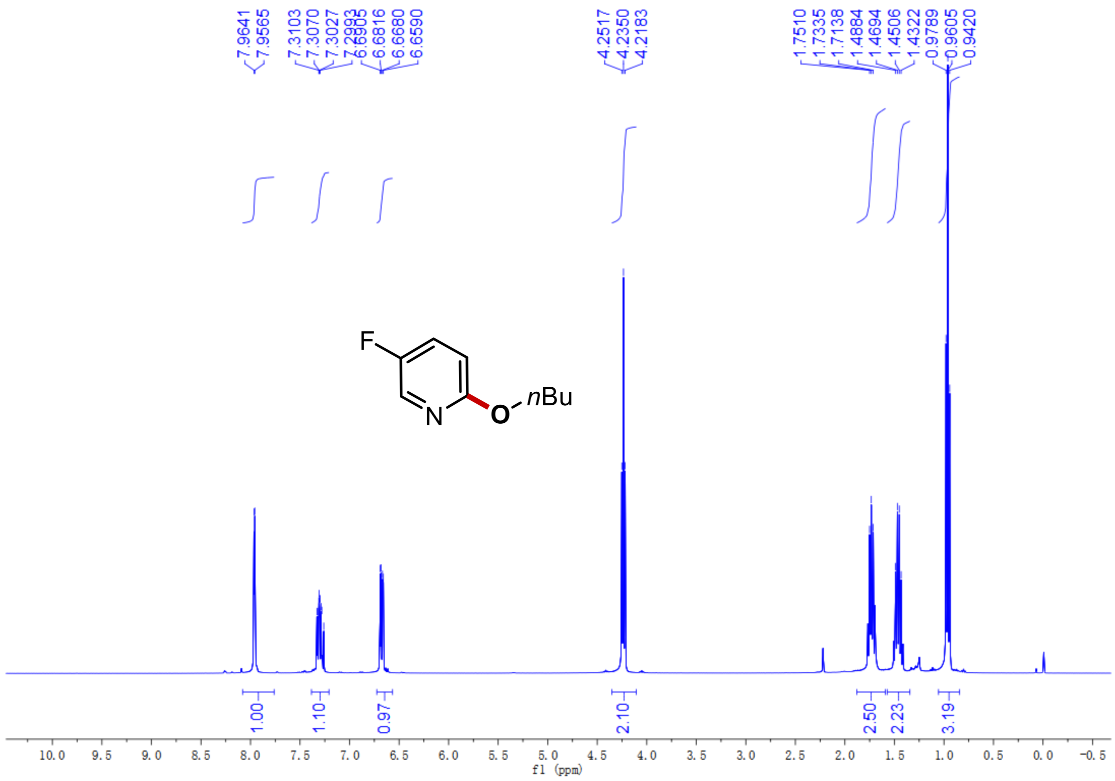

^{1}H NMR (400 MHz, $CDCl_3$) Spectrum

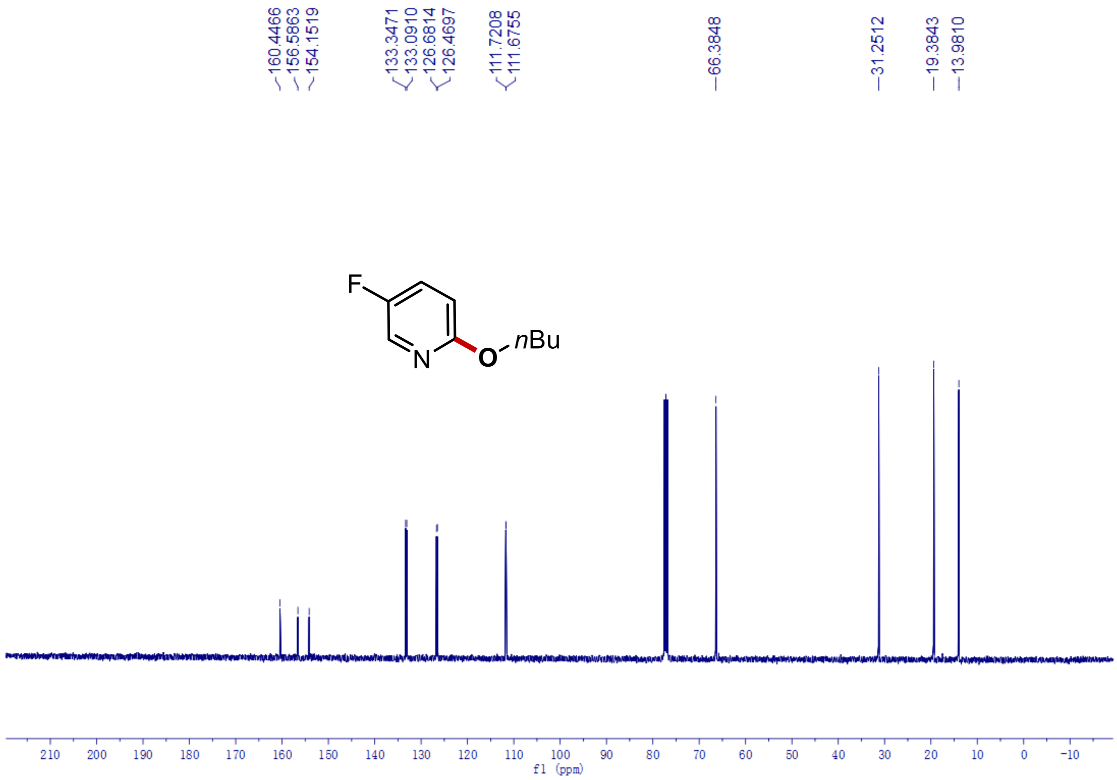

^{13}C NMR (100 MHz, $CDCl_3$) Spectrum

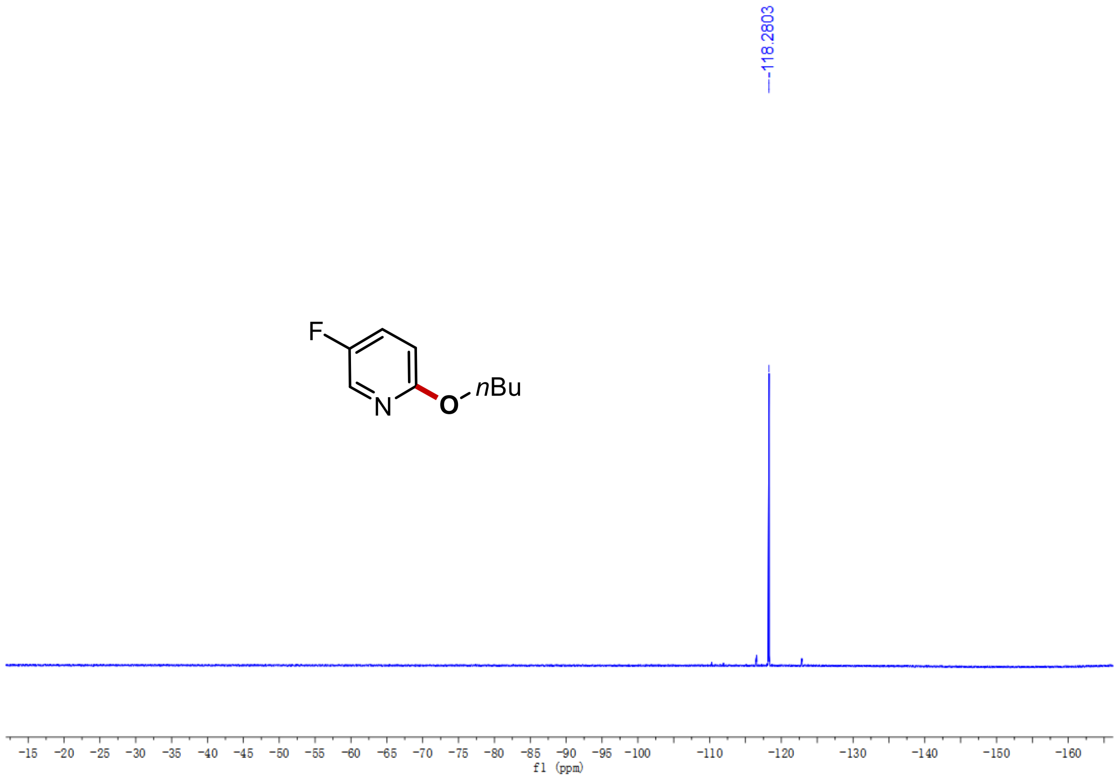

^{19}F NMR (376 MHz, $CDCl_3$) Spectrum

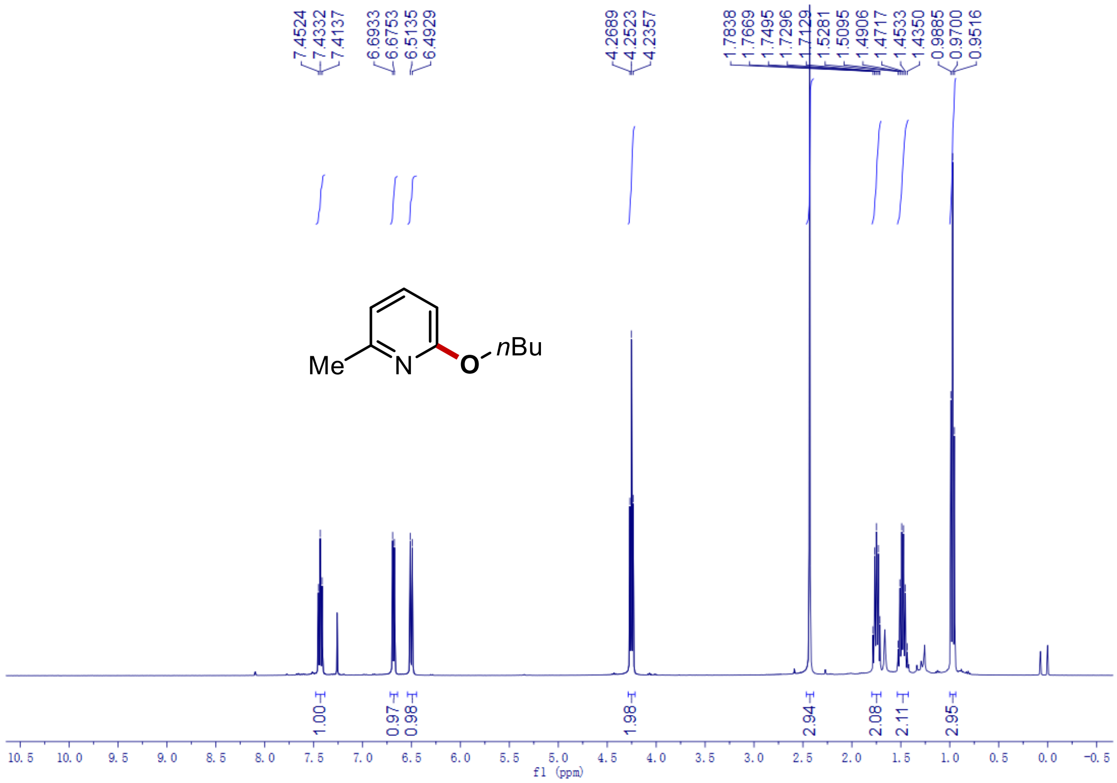

^{1}H NMR (400 MHz, $CDCl_3$) Spectrum

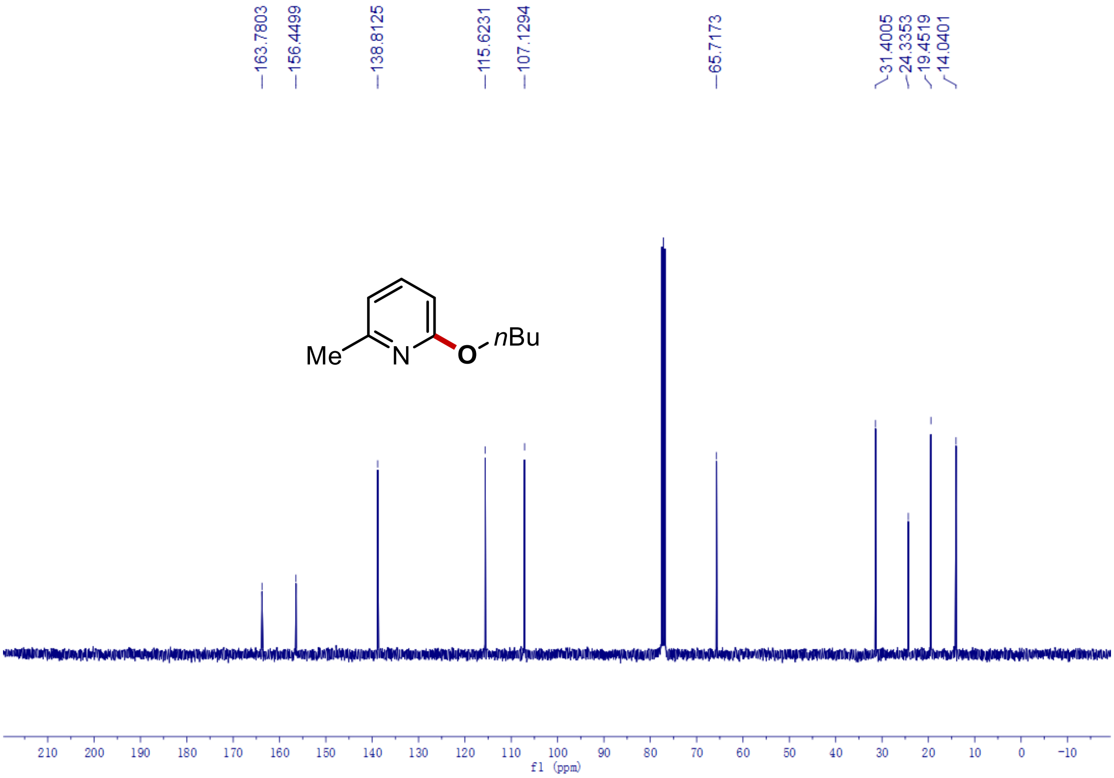

^{13}C NMR (100 MHz, CDCl3) Spectrum

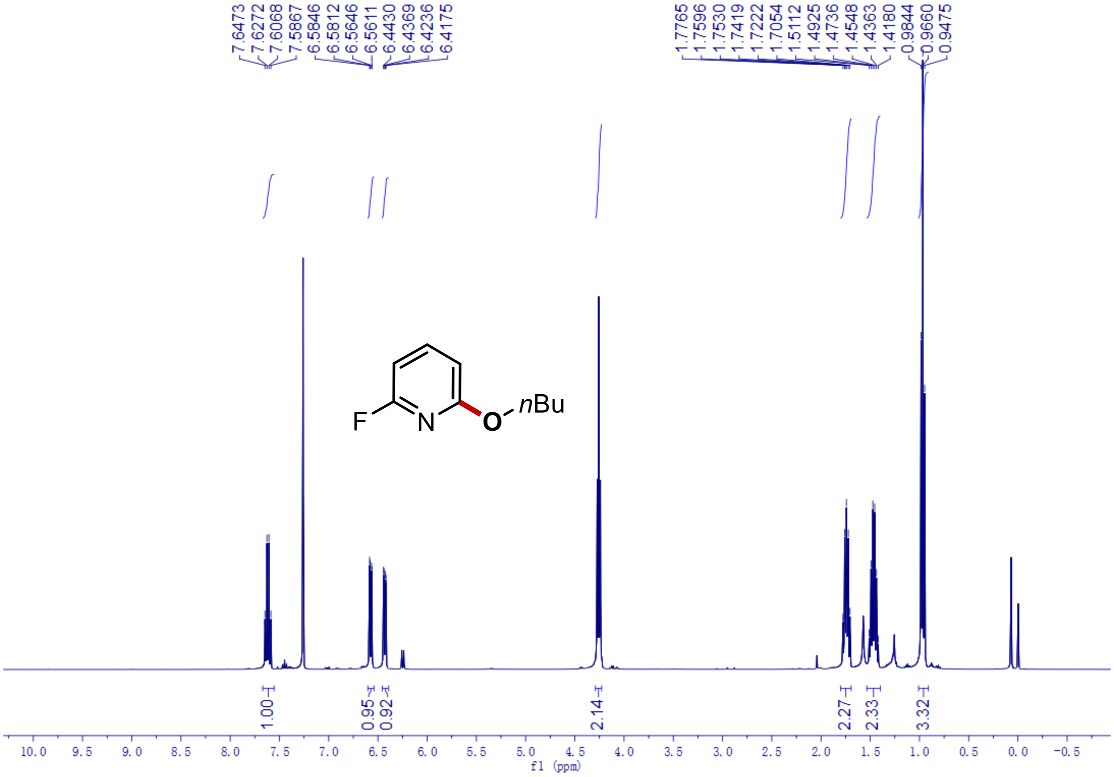

^{1}H NMR (400 MHz, CDCl3) Spectrum

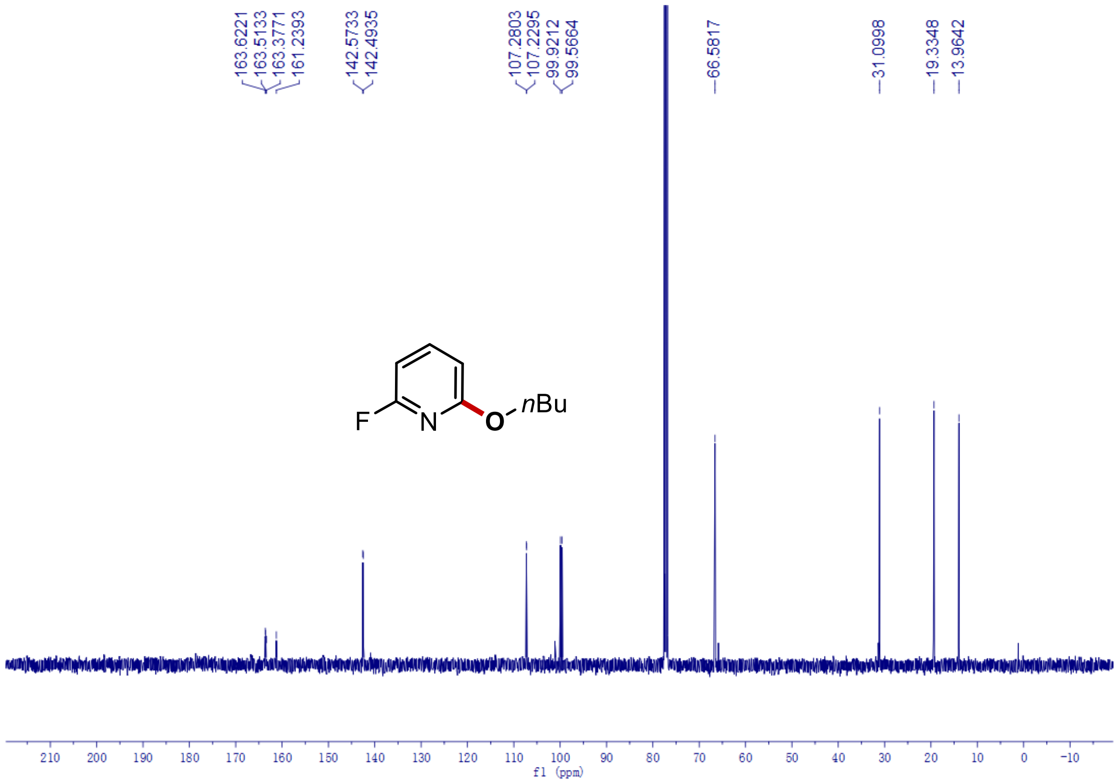

^{13}**C NMR** (100 MHz, $CDCl_3$) Spectrum

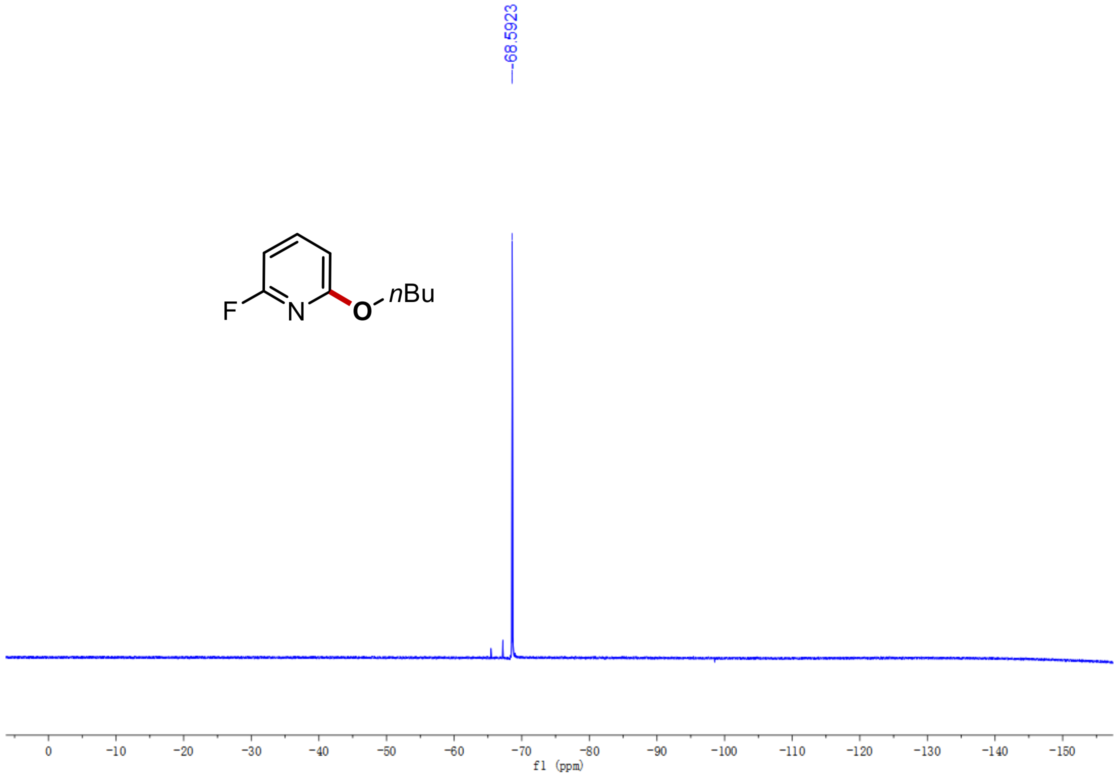

^{19}**F NMR** (376 MHz, $CDCl_3$) Spectrum

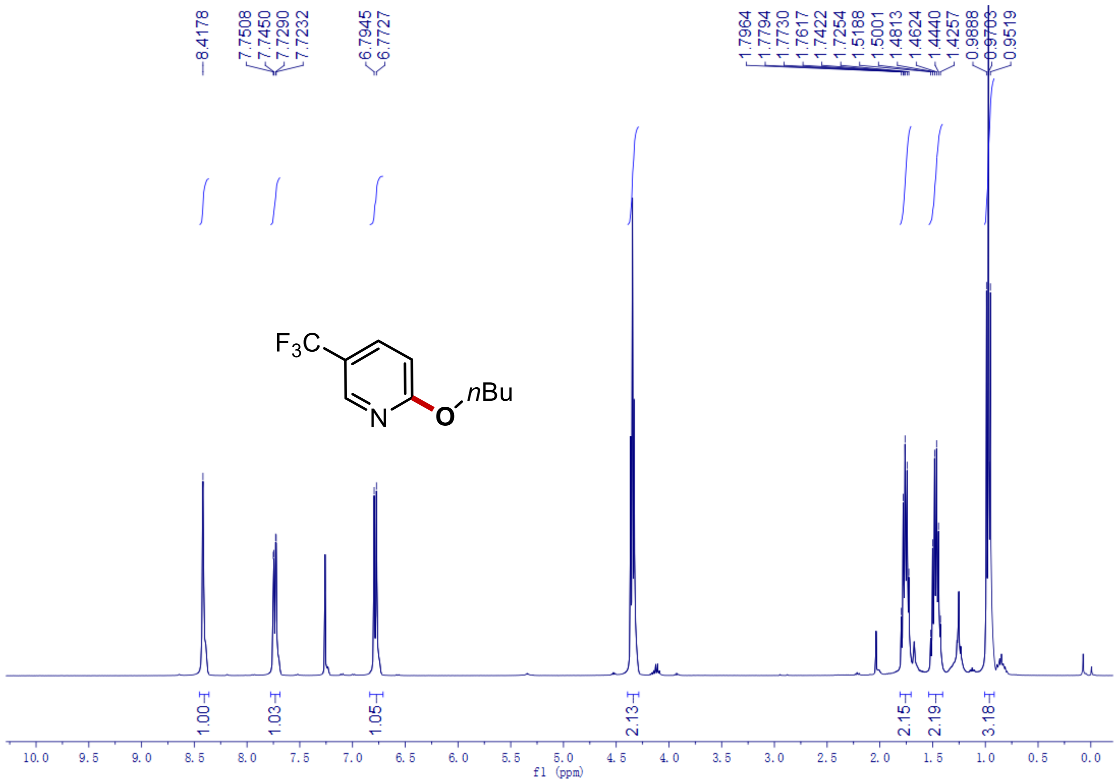

^{1}H NMR (400 MHz, $CDCl_3$) Spectrum

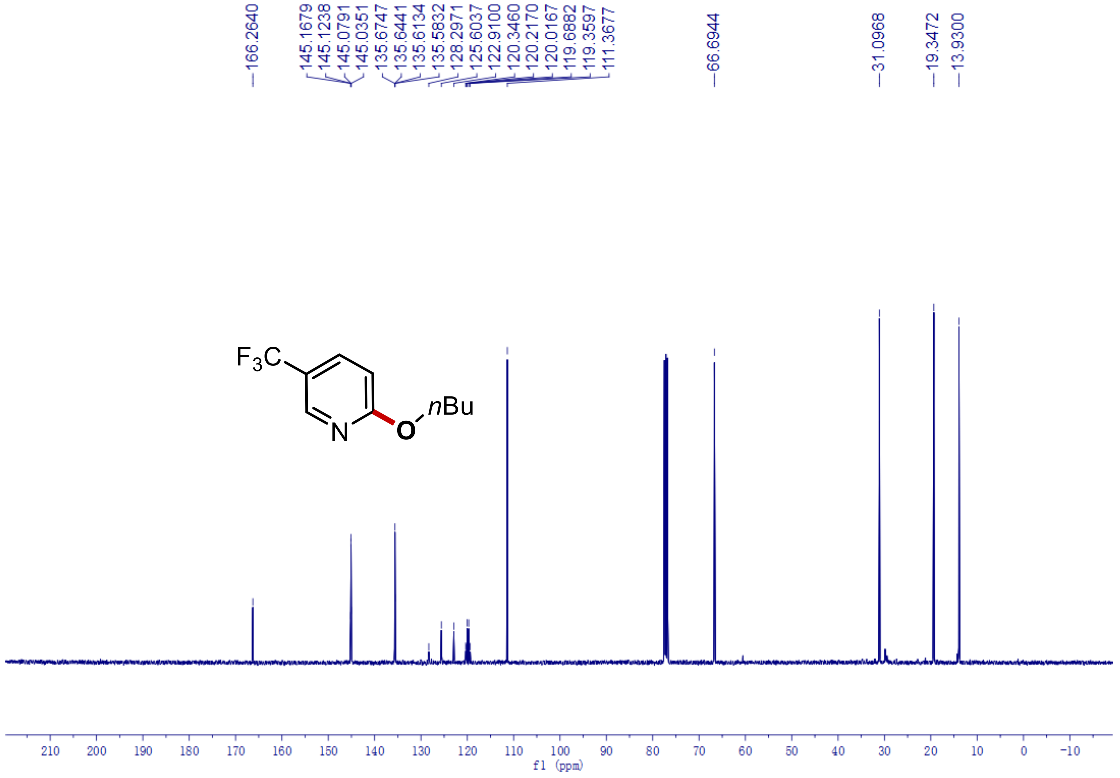

^{13}C NMR (100 MHz, $CDCl_3$) Spectrum

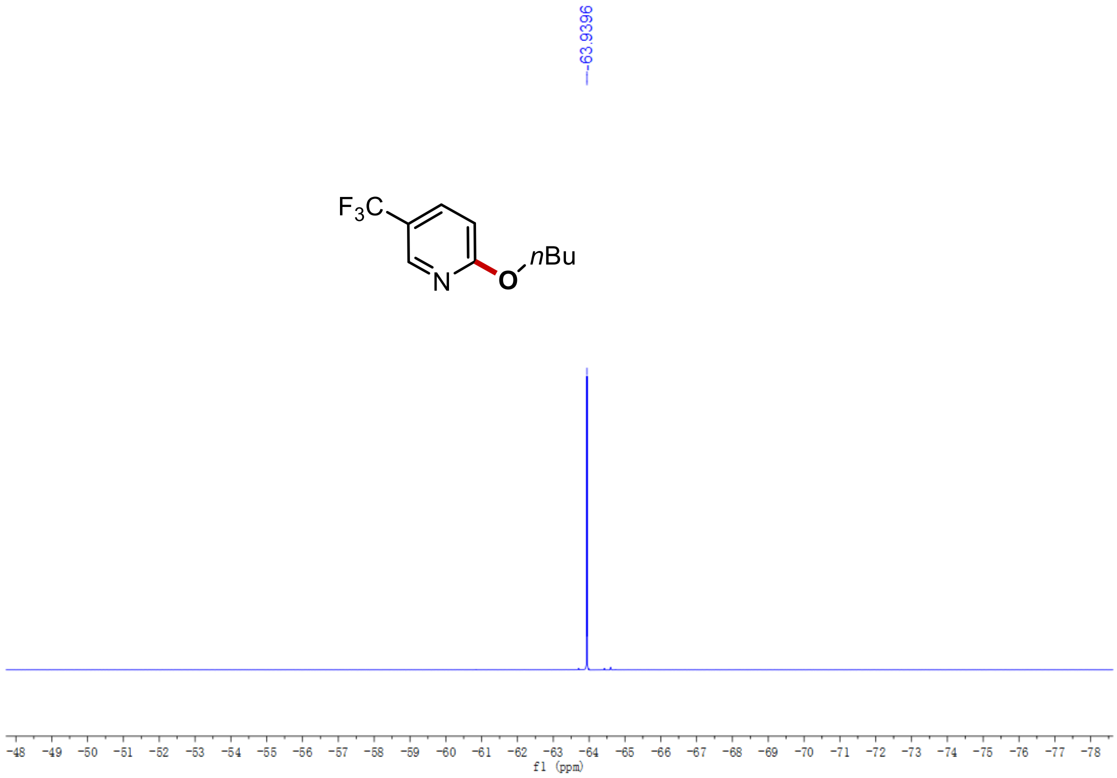

^{19}**F NMR** (376 MHz, $CDCl_3$) Spectrum

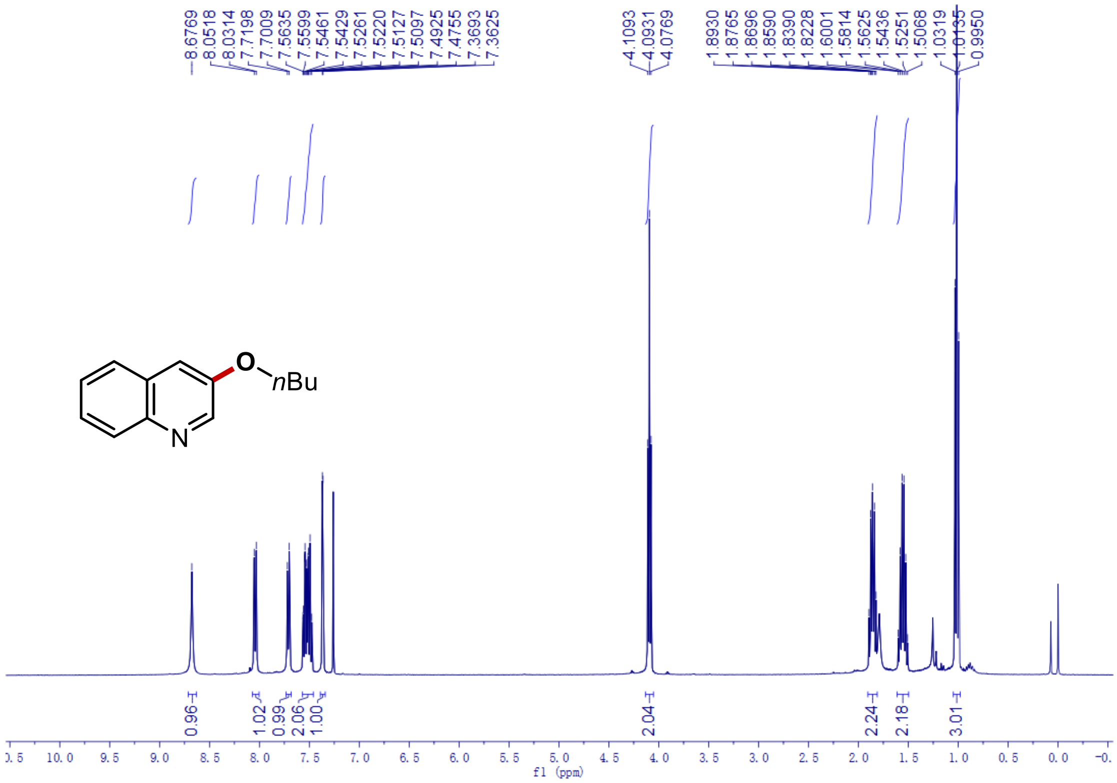

^{1}**H NMR** (400 MHz, $CDCl_3$) Spectrum

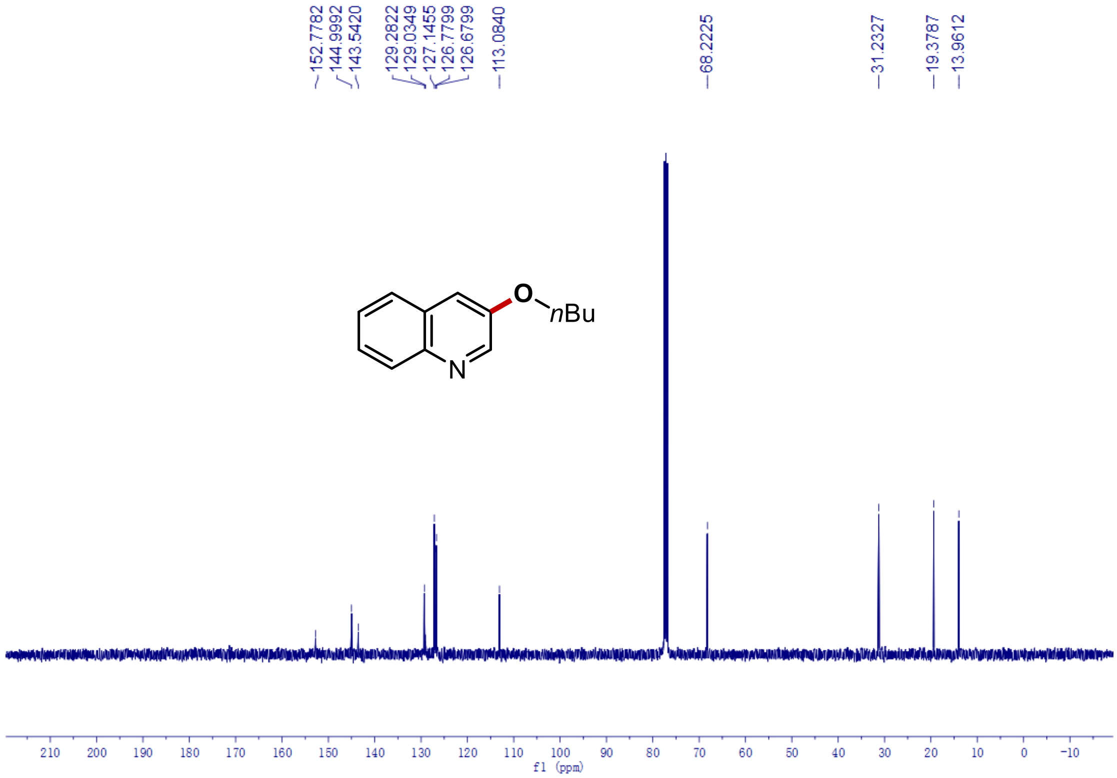

^{13}C NMR (100 MHz, $CDCl_3$) Spectrum

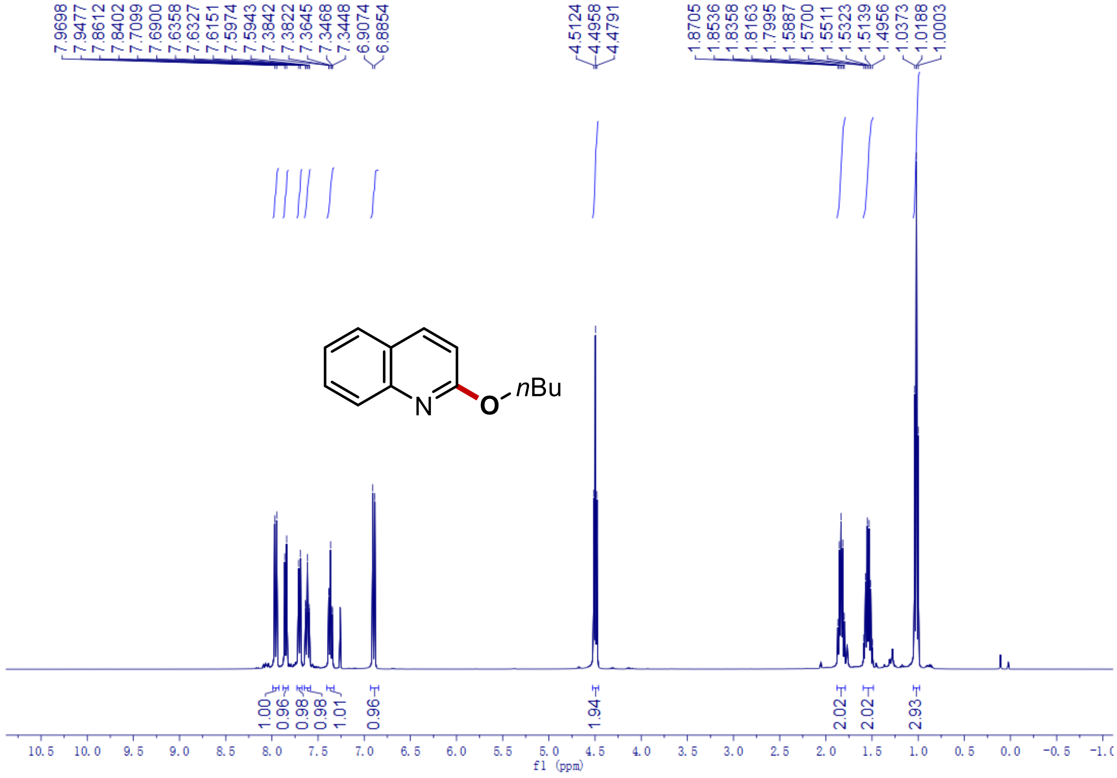

^{1}H NMR (400 MHz, $CDCl_3$) Spectrum

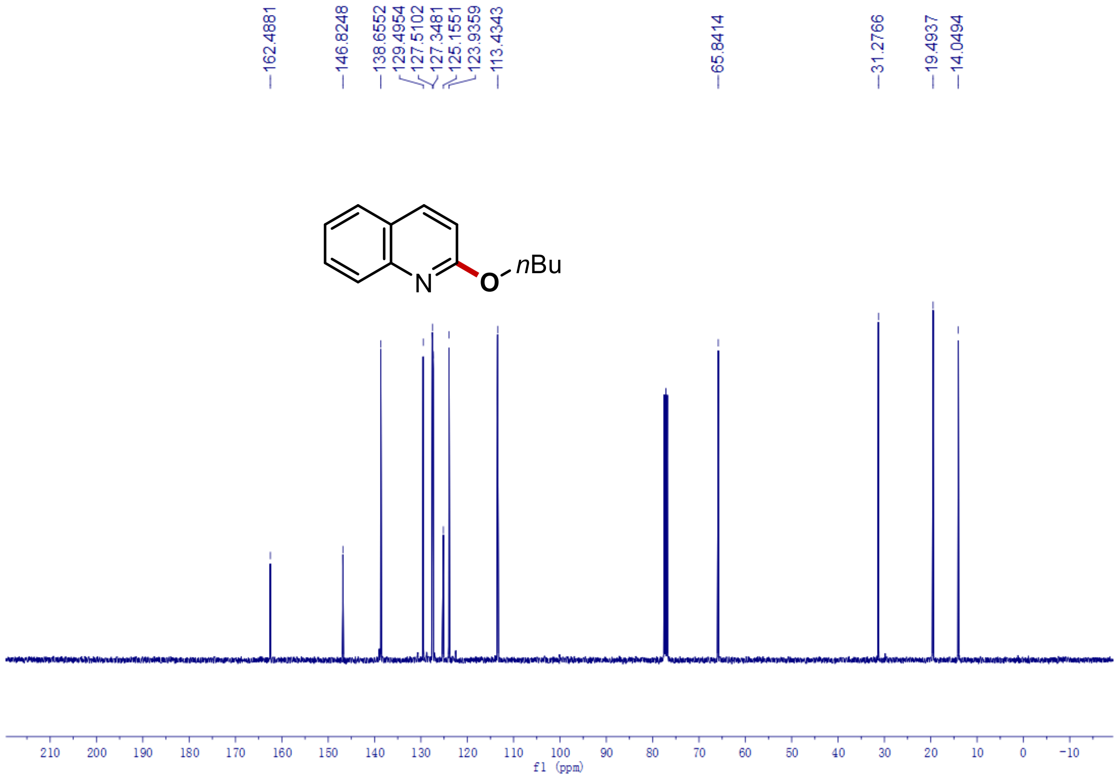

^{13}C NMR (100 MHz, $CDCl_3$) Spectrum

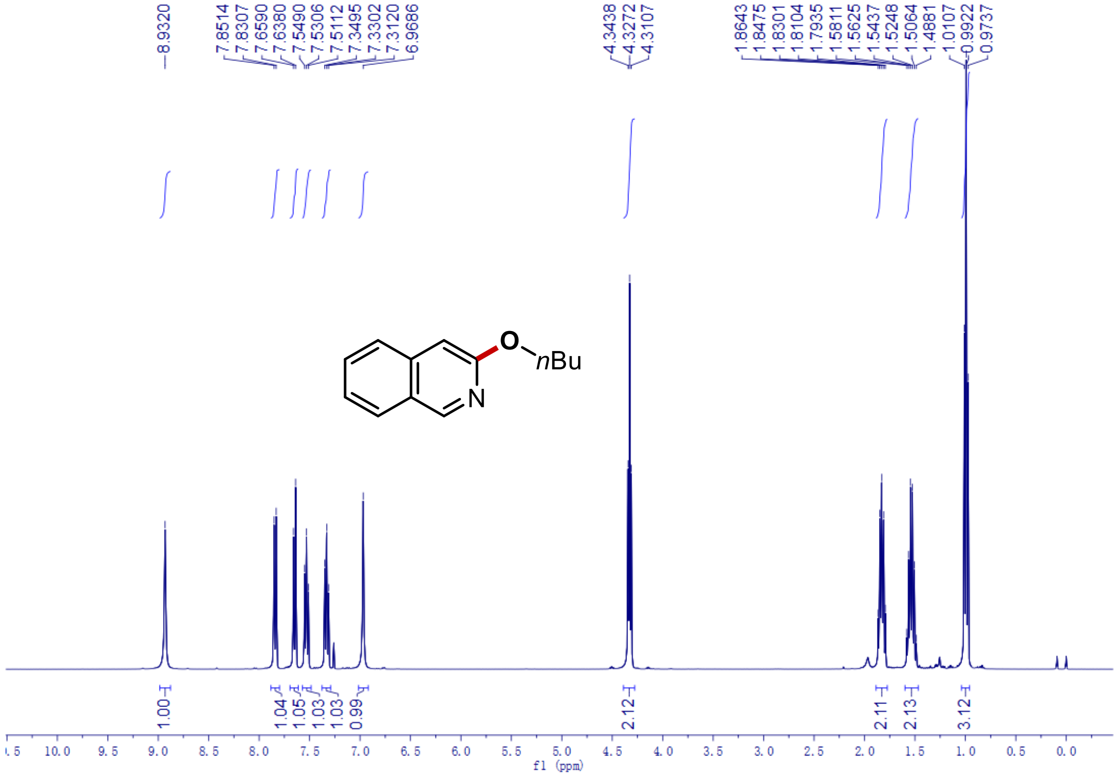

^{1}H NMR (400 MHz, $CDCl_3$) Spectrum

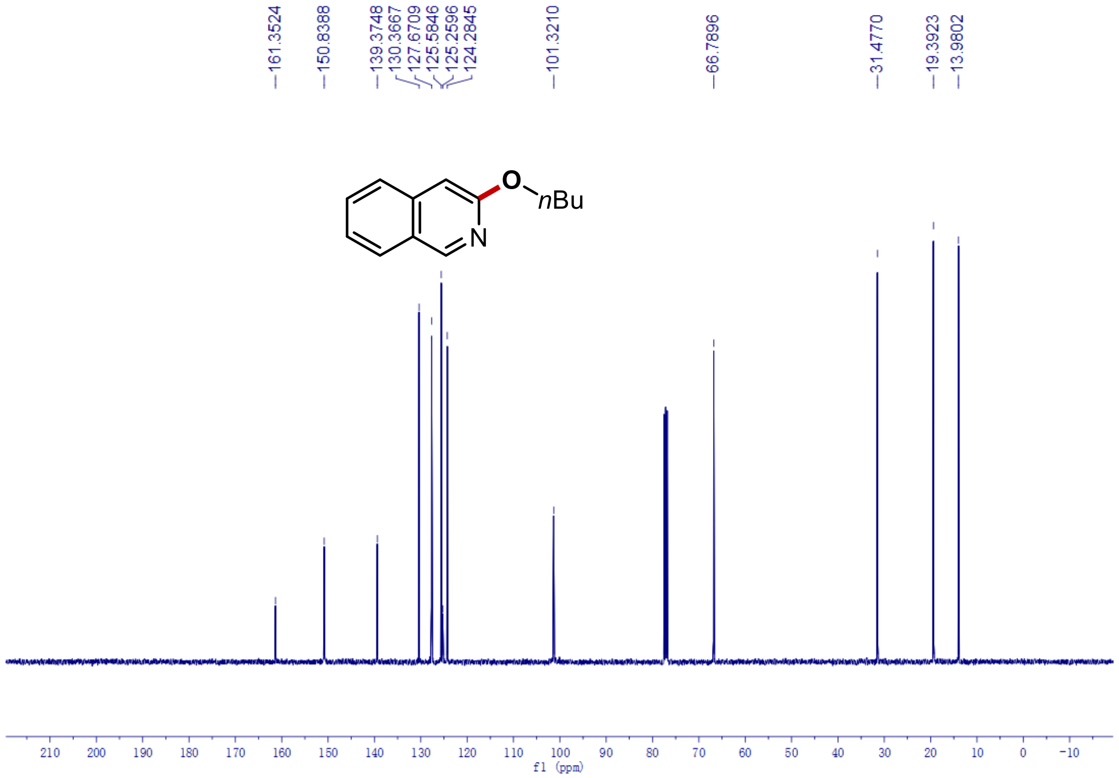

^{13}C NMR (100 MHz, $CDCl_3$) Spectrum

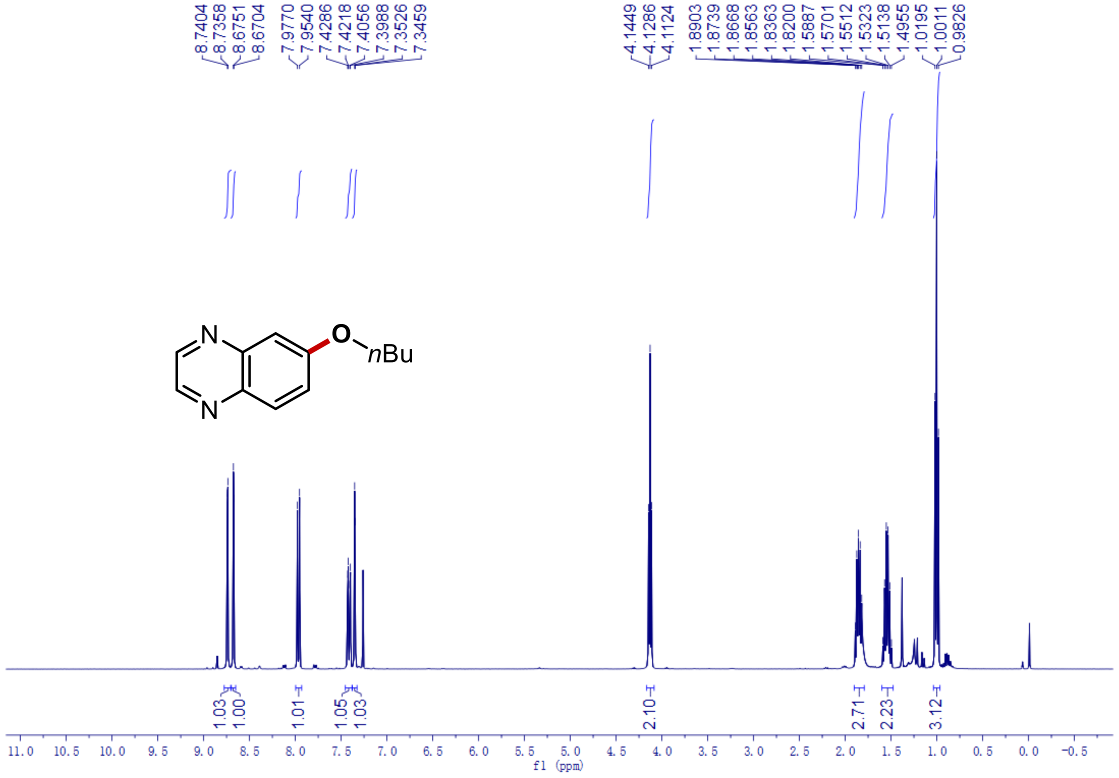

^{1}H NMR (400 MHz, $CDCl_3$) Spectrum

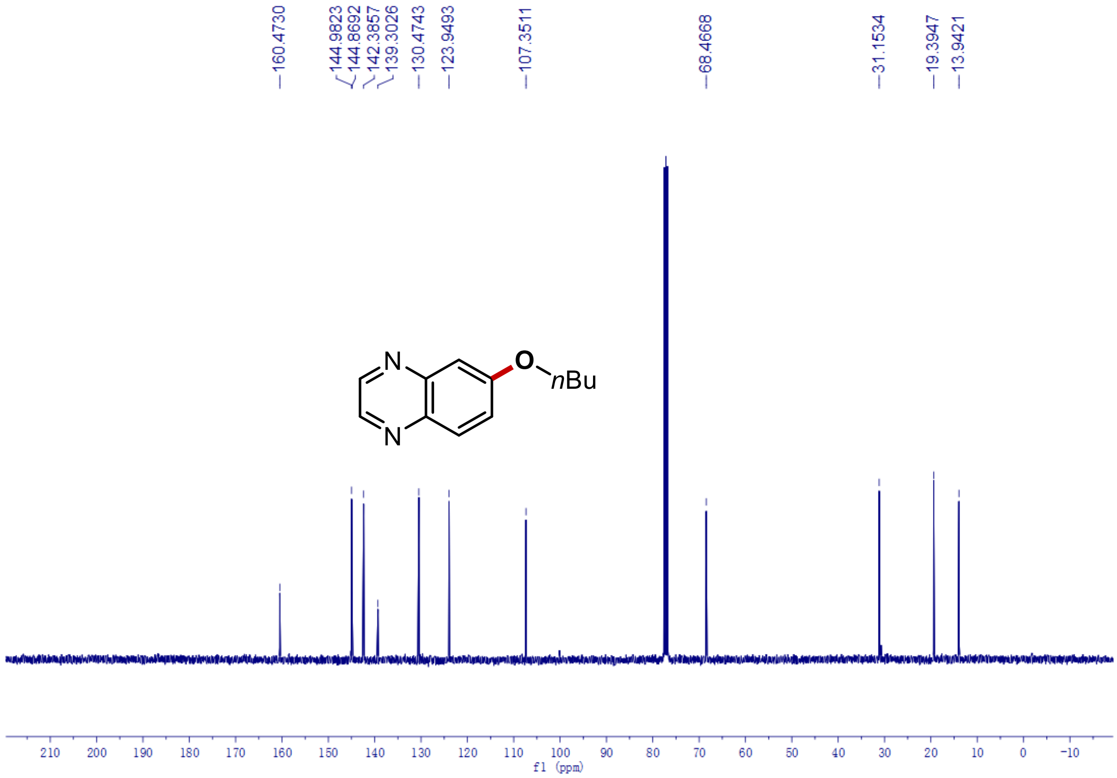

^{13}**C NMR** (100 MHz, $CDCl_3$) Spectrum

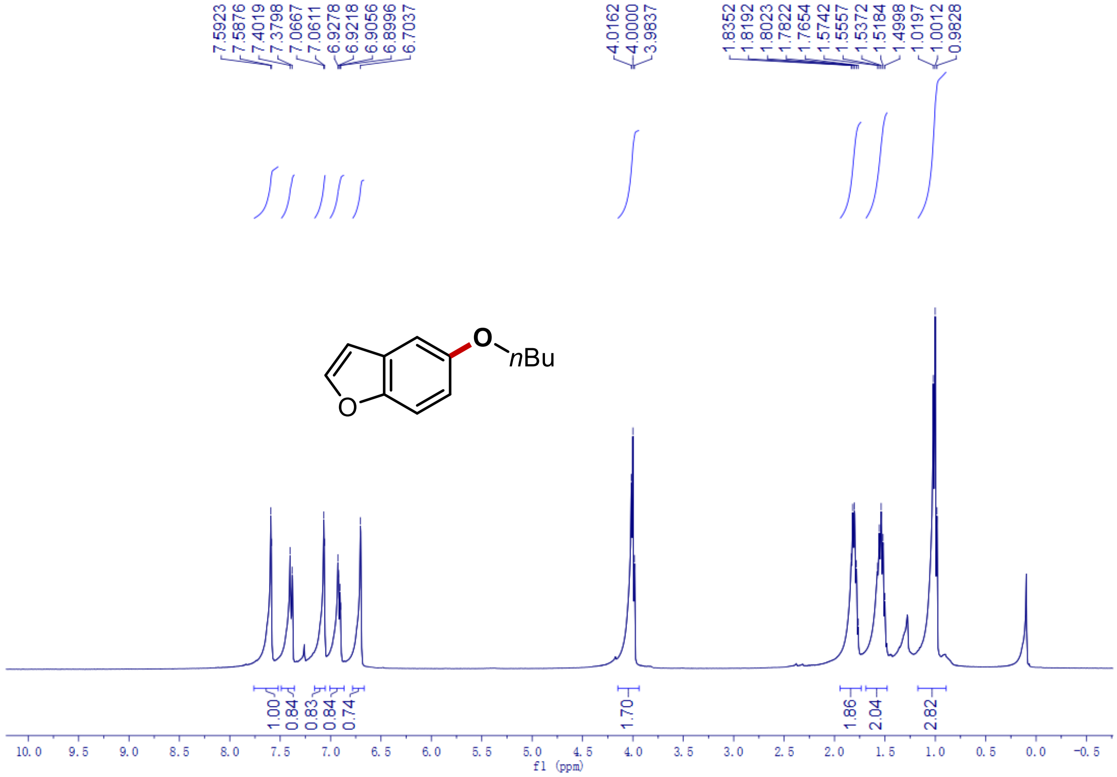

^{1}**H NMR** (400 MHz, $CDCl_3$) Spectrum

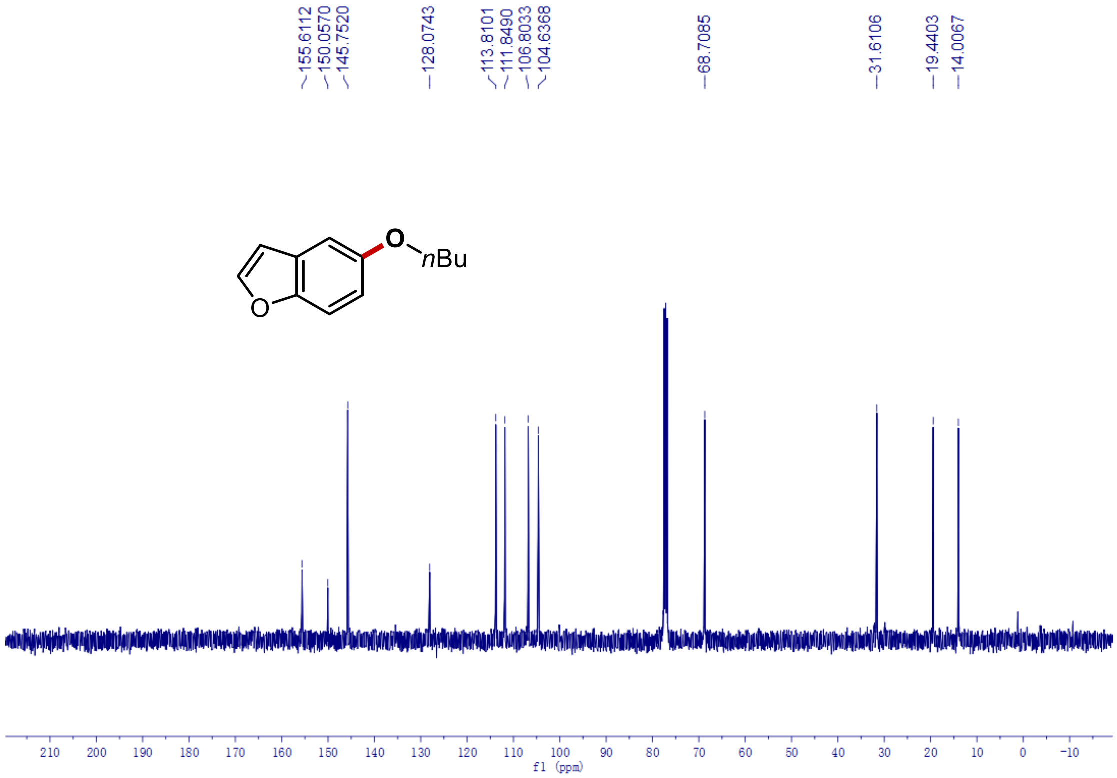

^{13}C NMR (100 MHz, $CDCl_3$) Spectrum

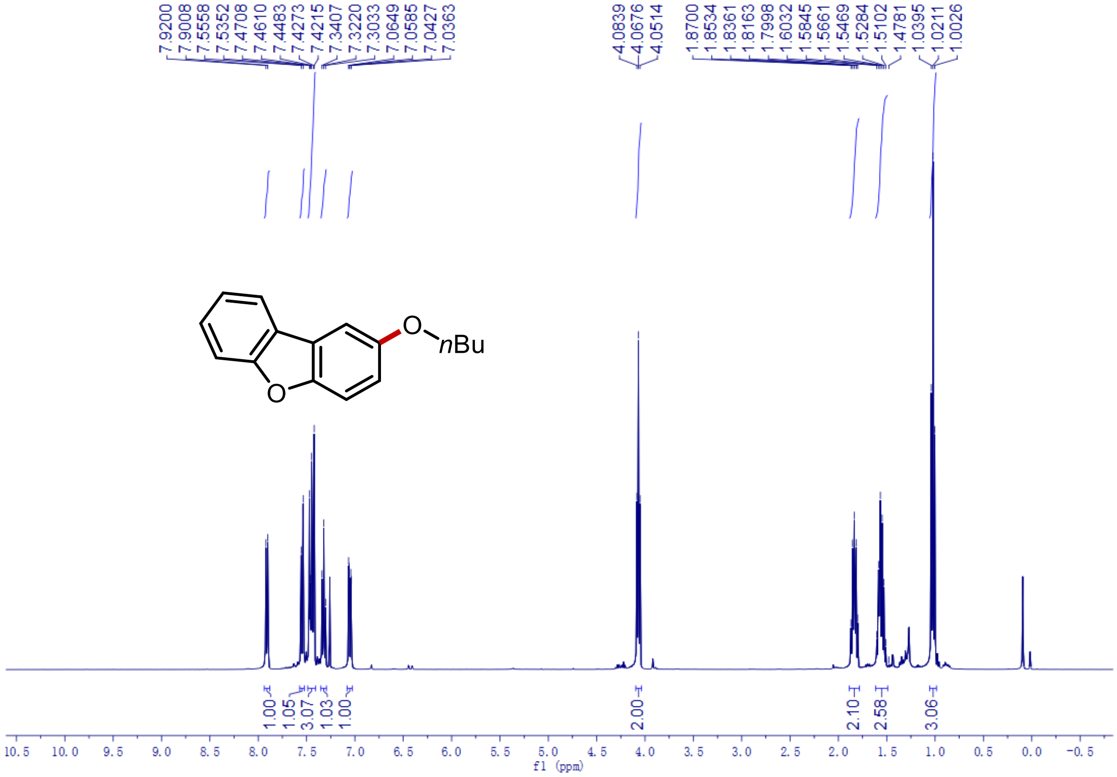

^{1}H NMR (400 MHz, $CDCl_3$) Spectrum

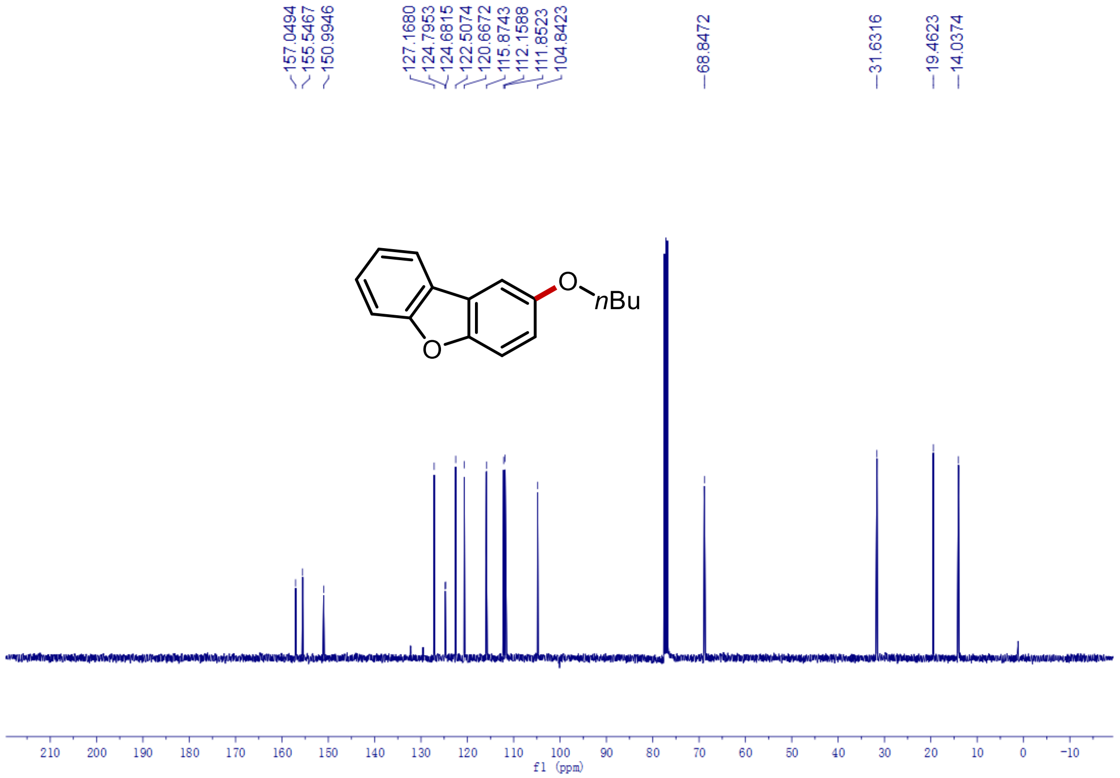

^{13}**C NMR** (100 MHz, CDCl$_3$) Spectrum

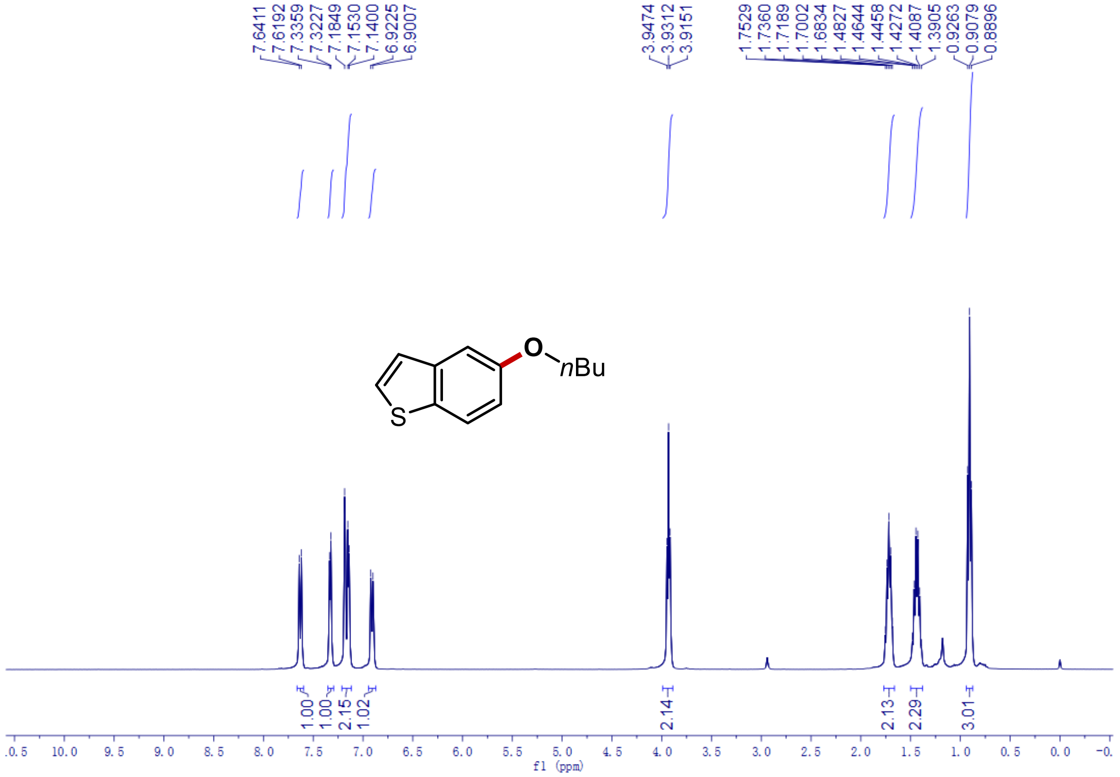

^{1}**H NMR** (400 MHz, CDCl$_3$) Spectrum

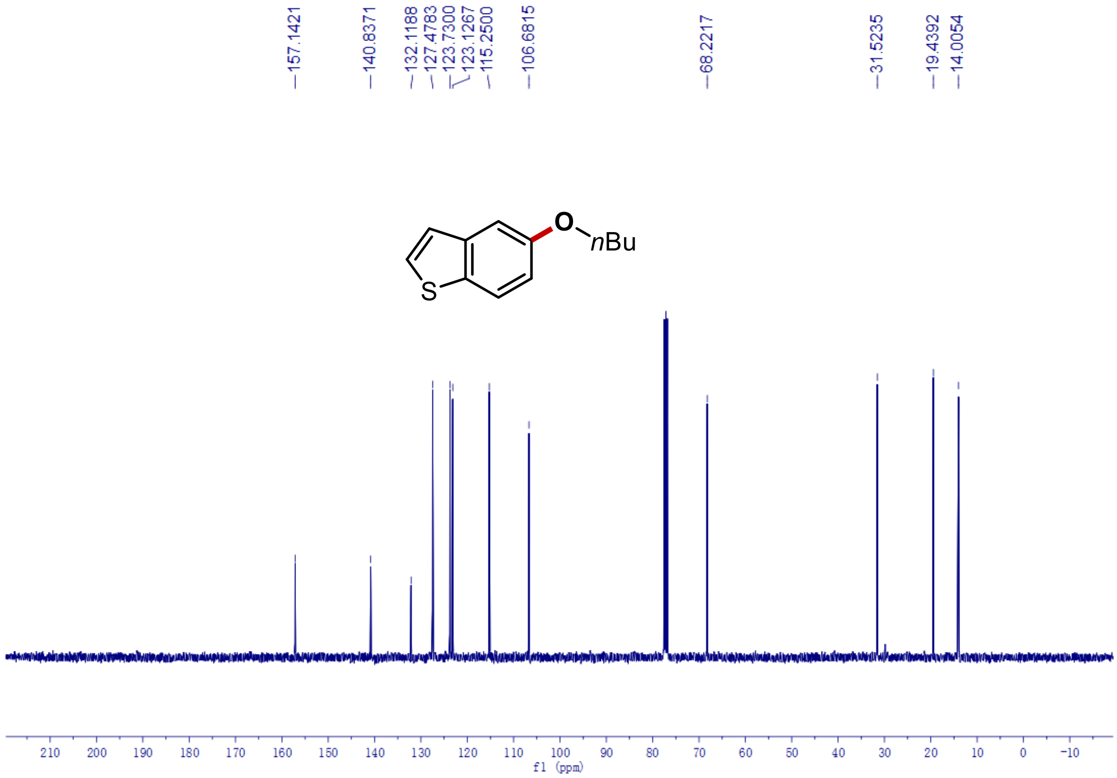

^{13}C NMR (100 MHz, $CDCl_3$) Spectrum

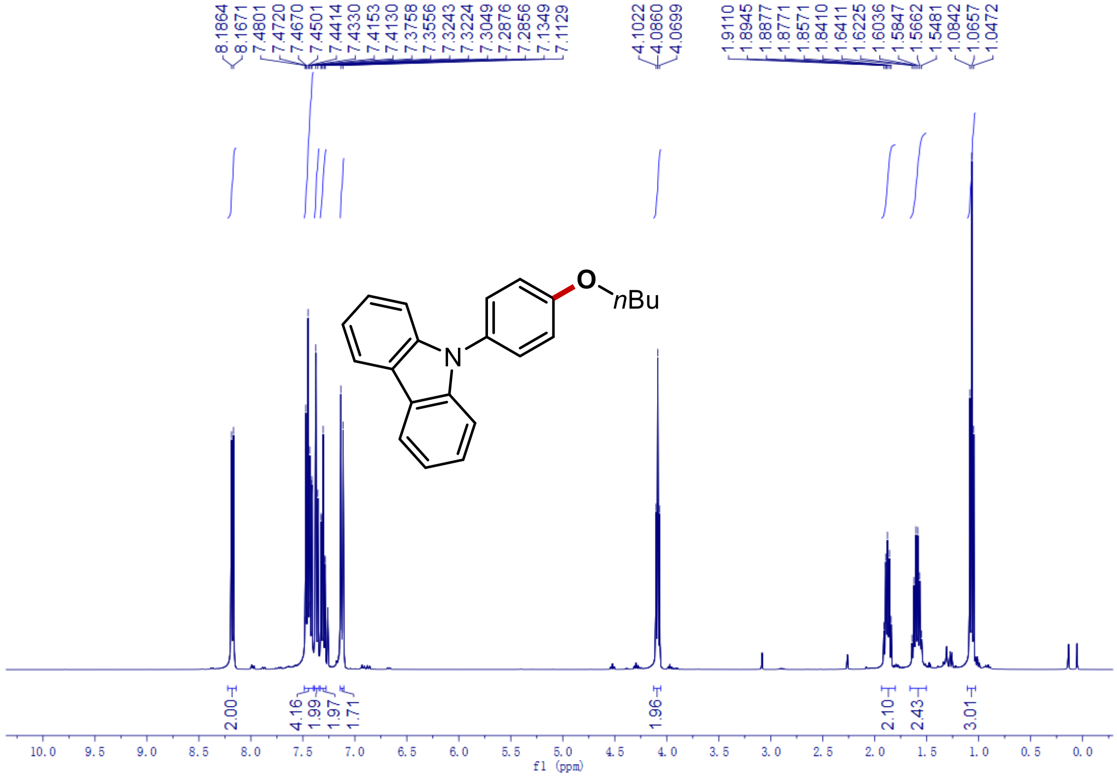

^{1}H NMR (400 MHz, $CDCl_3$) Spectrum

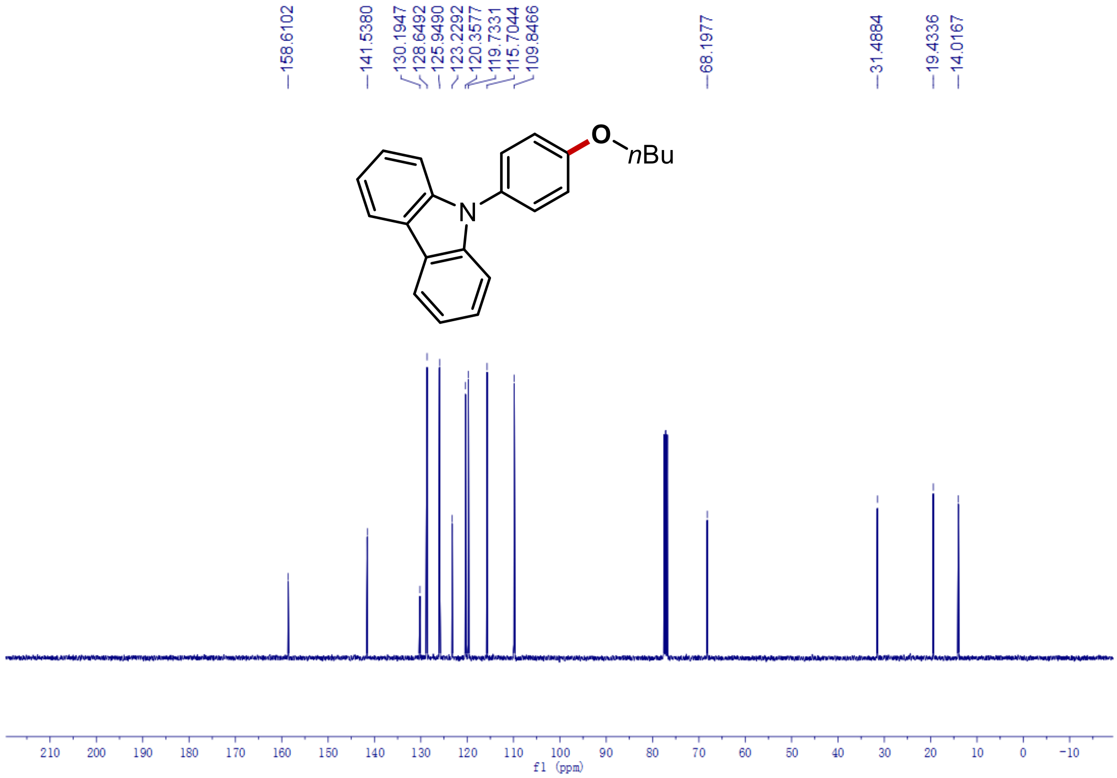

¹³C NMR (100 MHz, $CDCl_3$) Spectrum

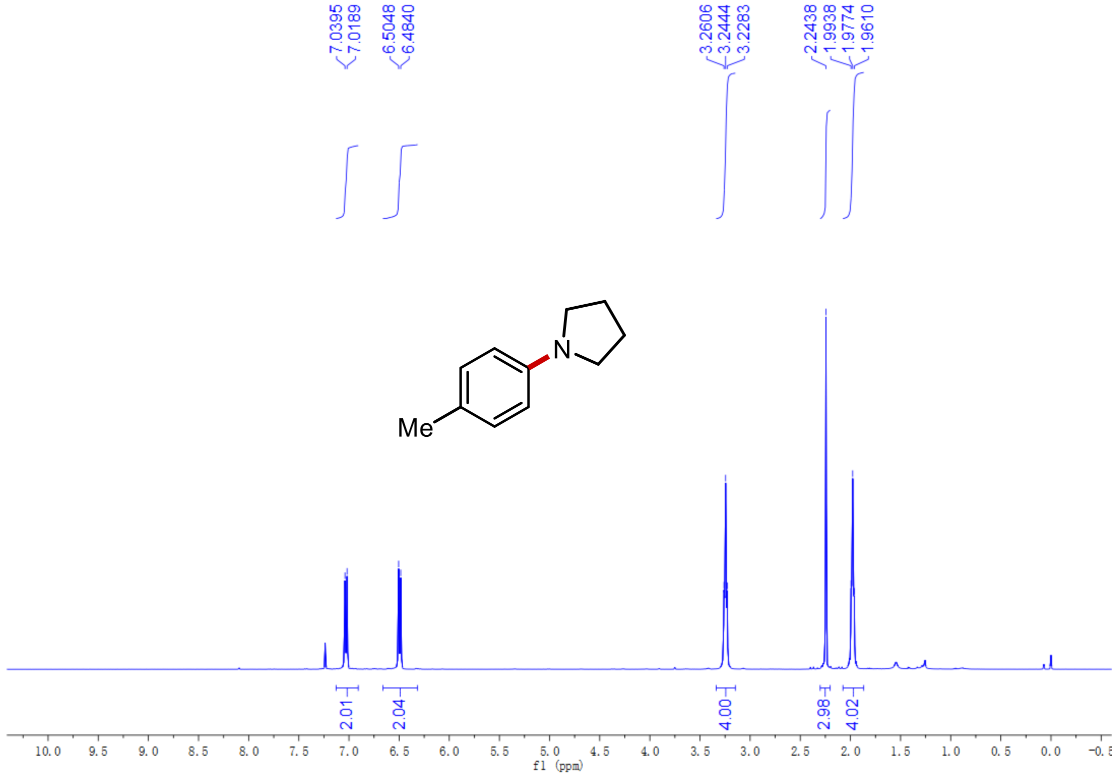

¹H NMR (400 MHz, $CDCl_3$) Spectrum

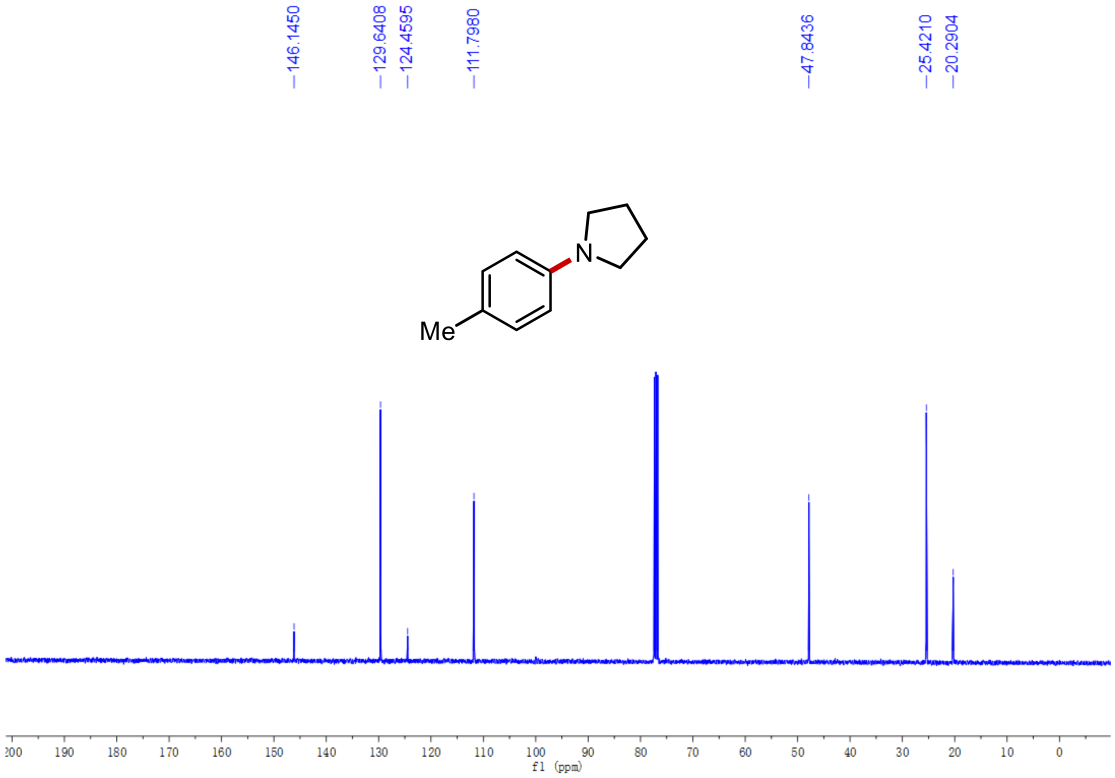

^{13}C NMR (100 MHz, $CDCl_3$) Spectrum

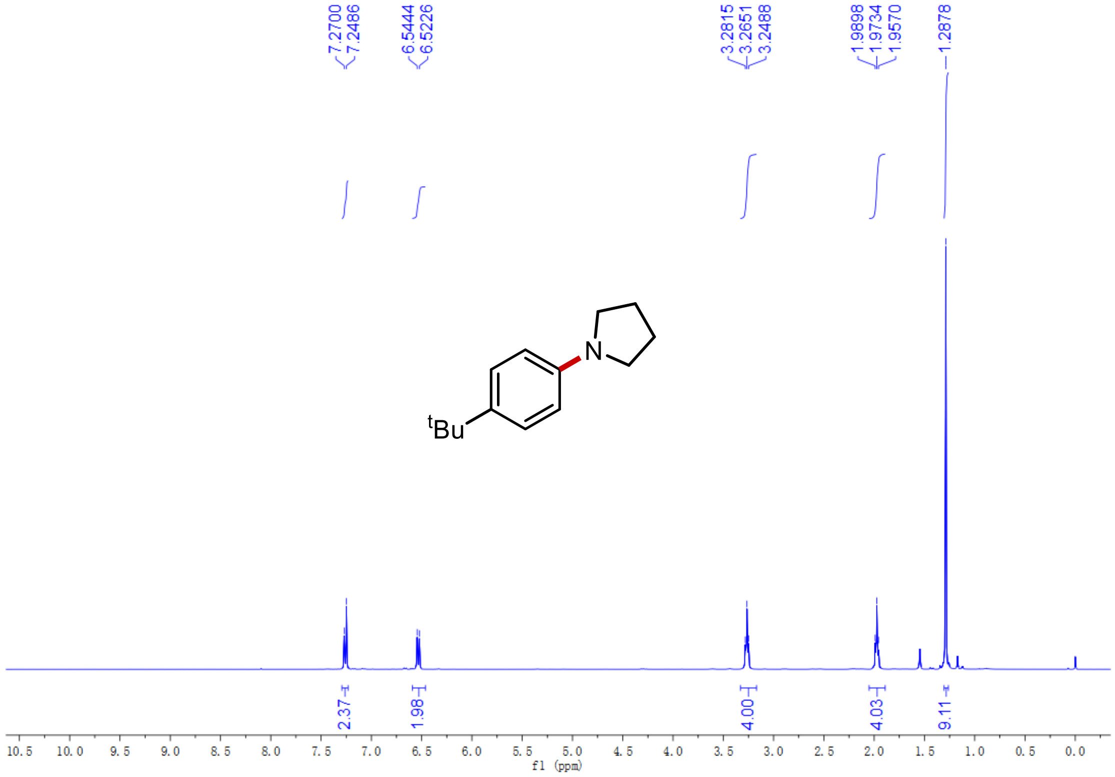

^{1}H NMR (400 MHz, $CDCl_3$) Spectrum

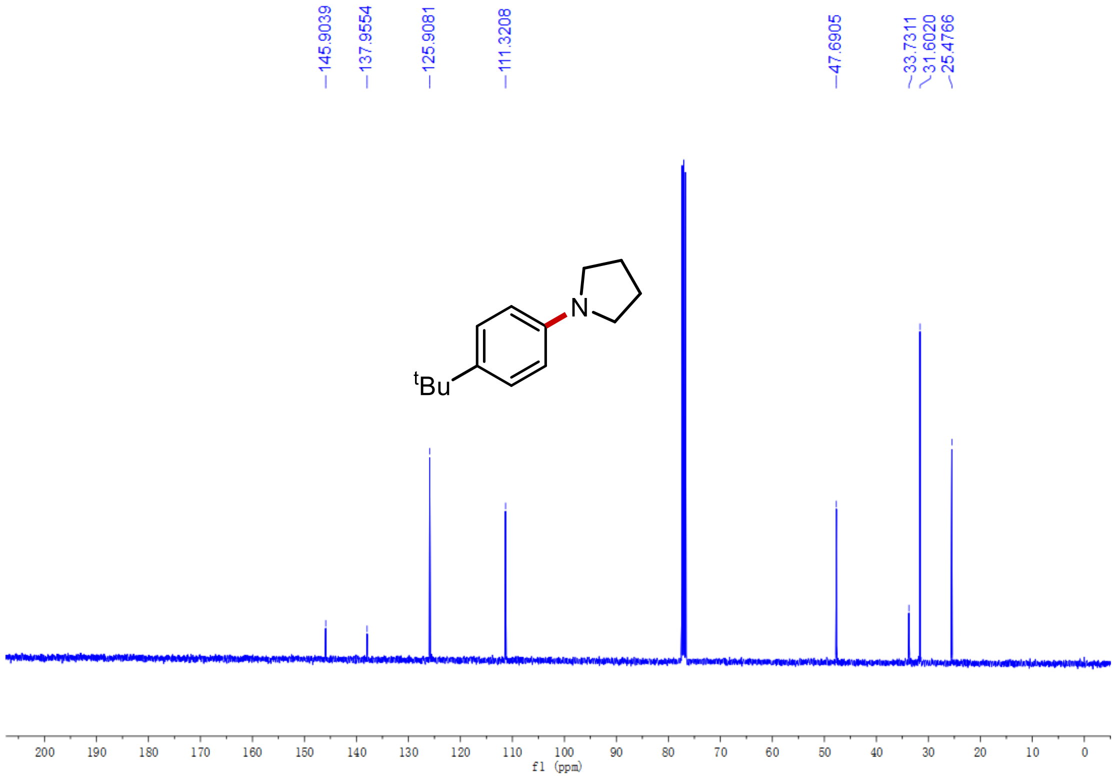

^{13}C NMR (100 MHz, $CDCl_3$) Spectrum

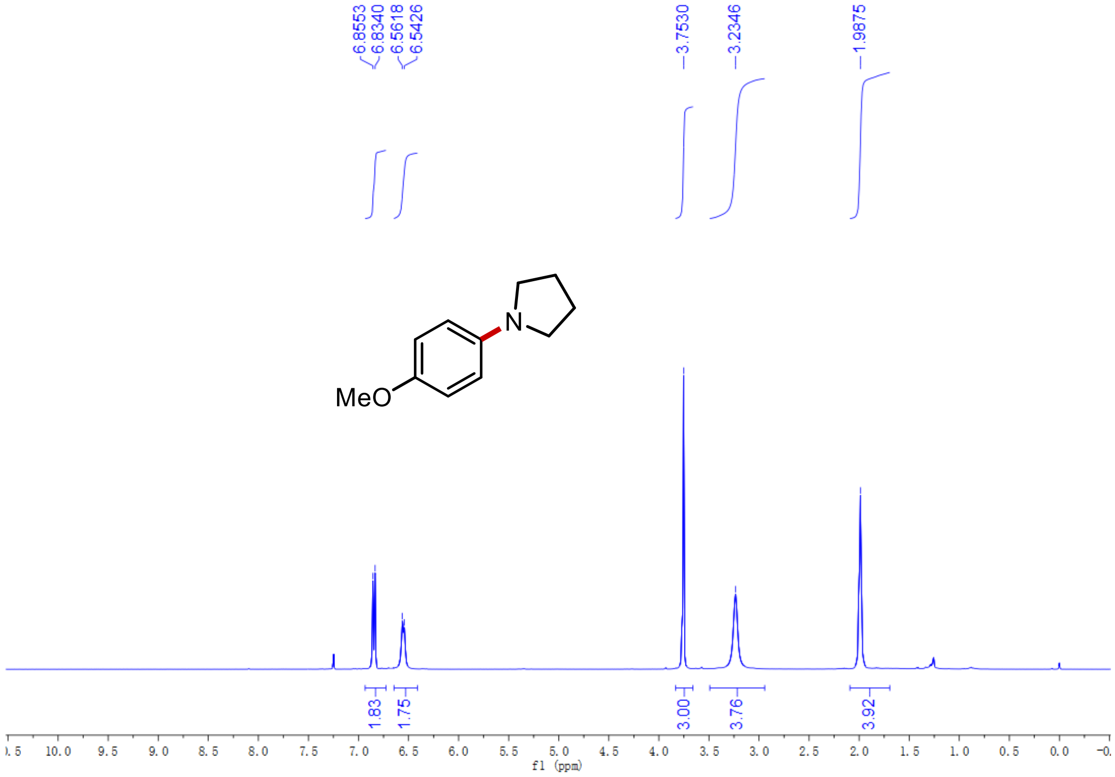

^{1}H NMR (400 MHz, $CDCl_3$) Spectrum

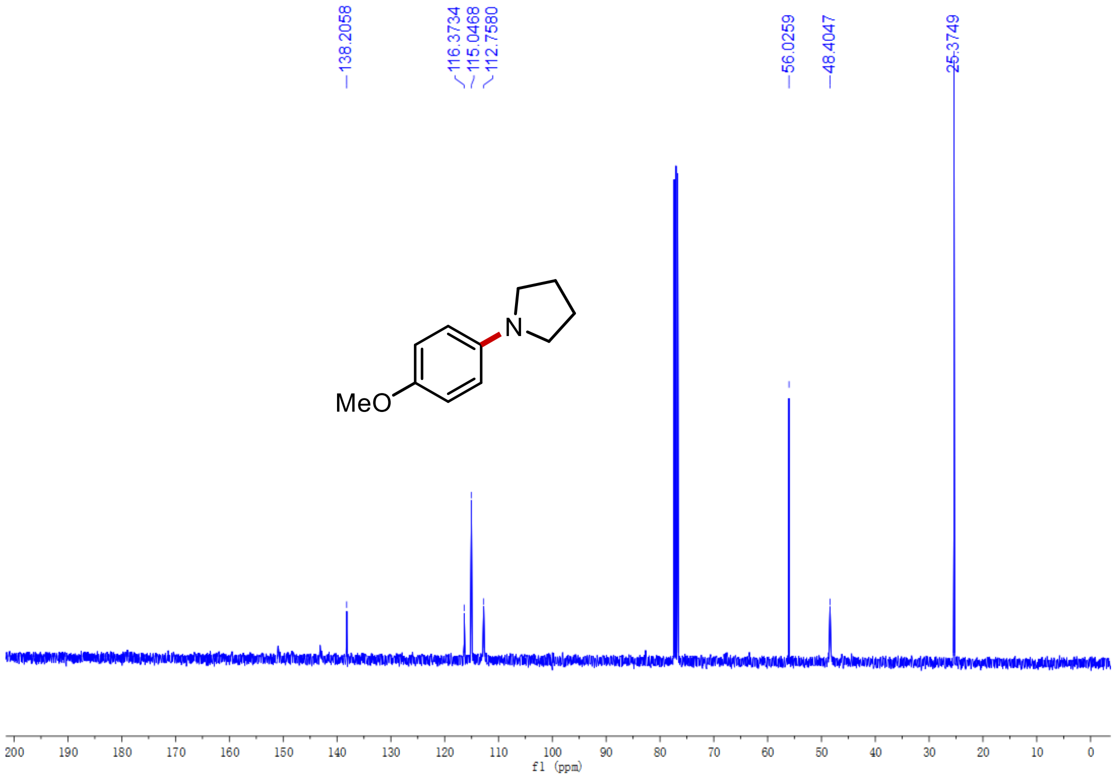

^{13}C NMR (100 MHz, $CDCl_3$) Spectrum

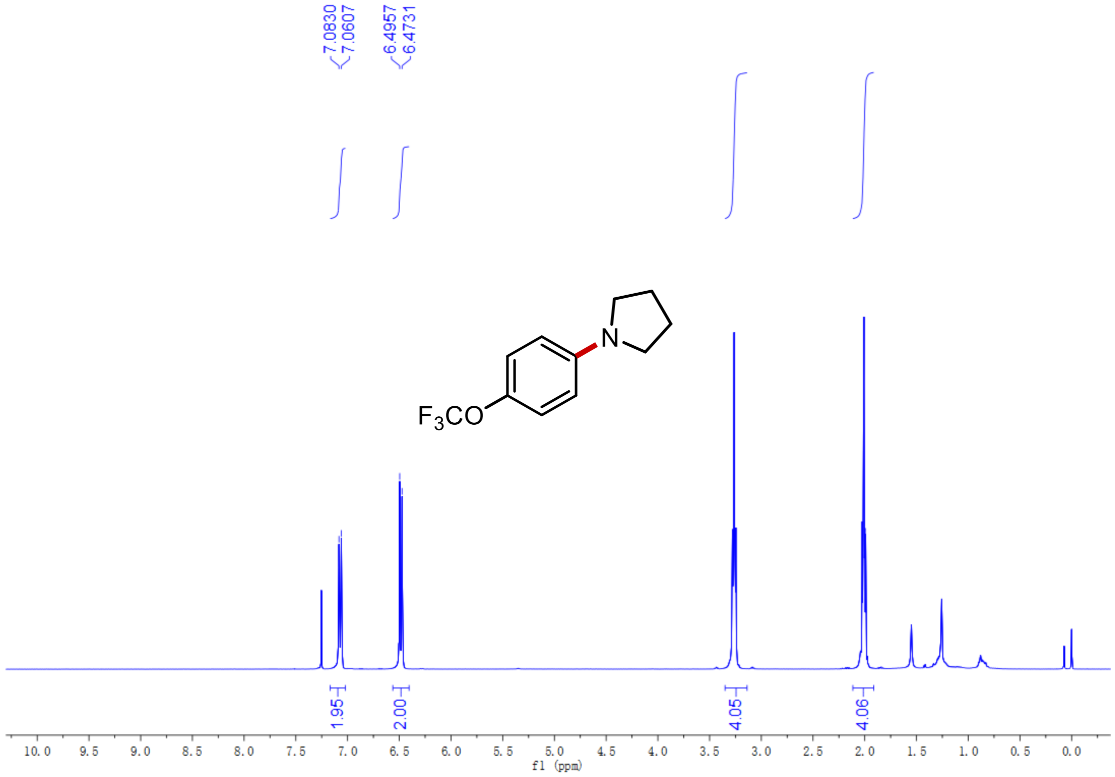

^{1}H NMR (400 MHz, $CDCl_3$) Spectrum

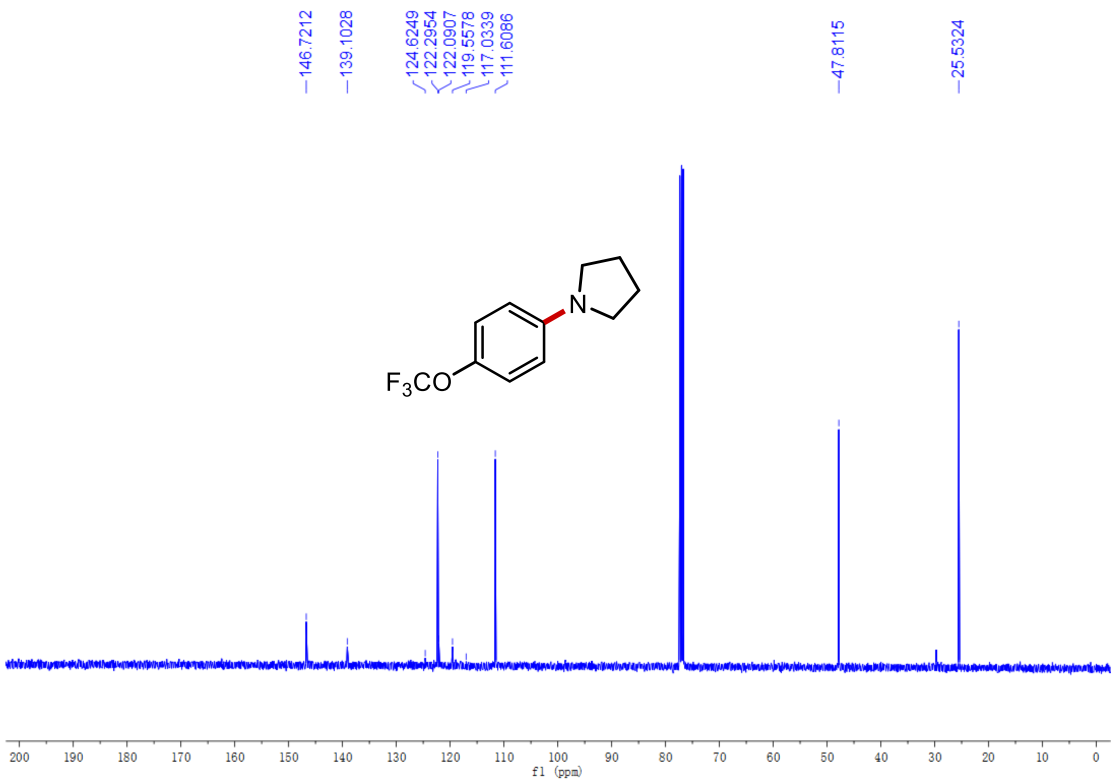

^{13}C NMR (100 MHz, $CDCl_3$) Spectrum

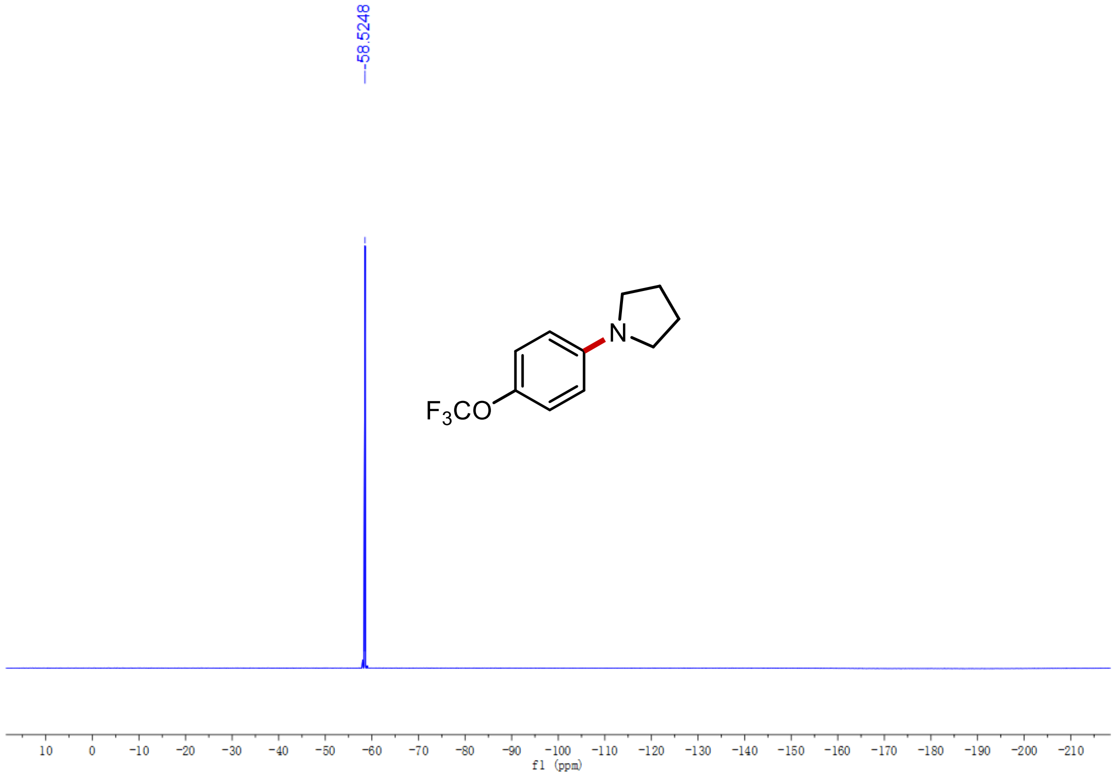

^{19}F NMR (376 MHz, $CDCl_3$) Spectrum

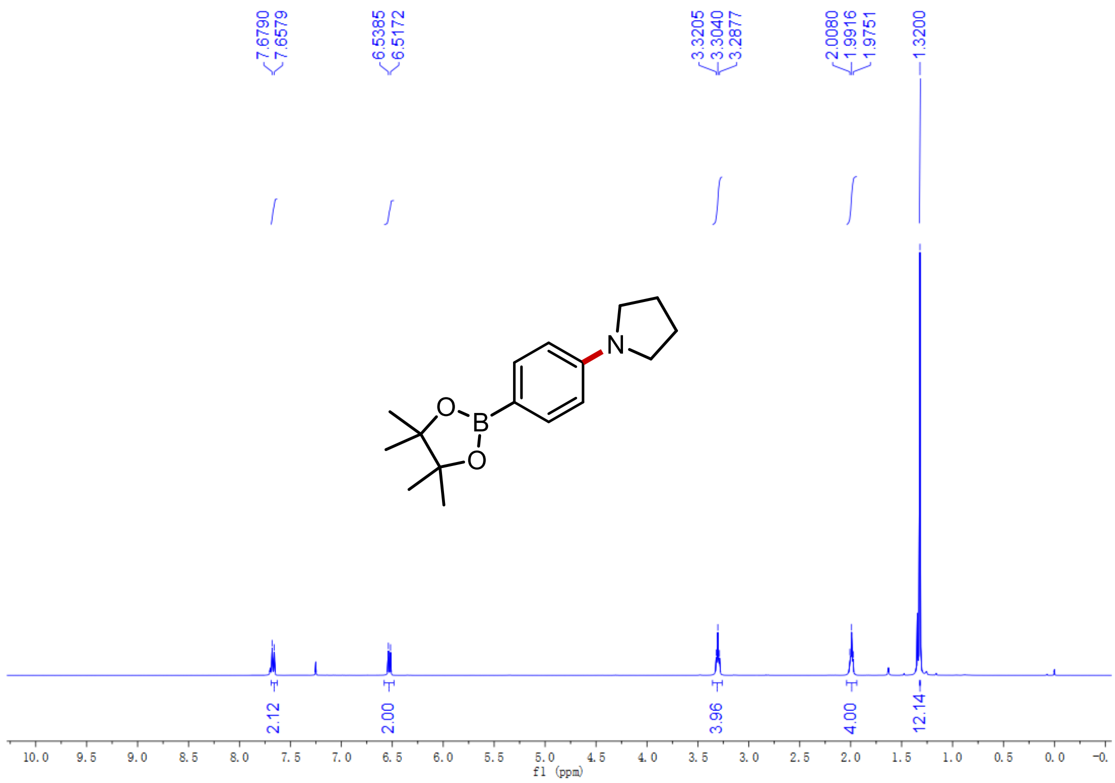

^{1}H NMR (400 MHz, $CDCl_3$) Spectrum

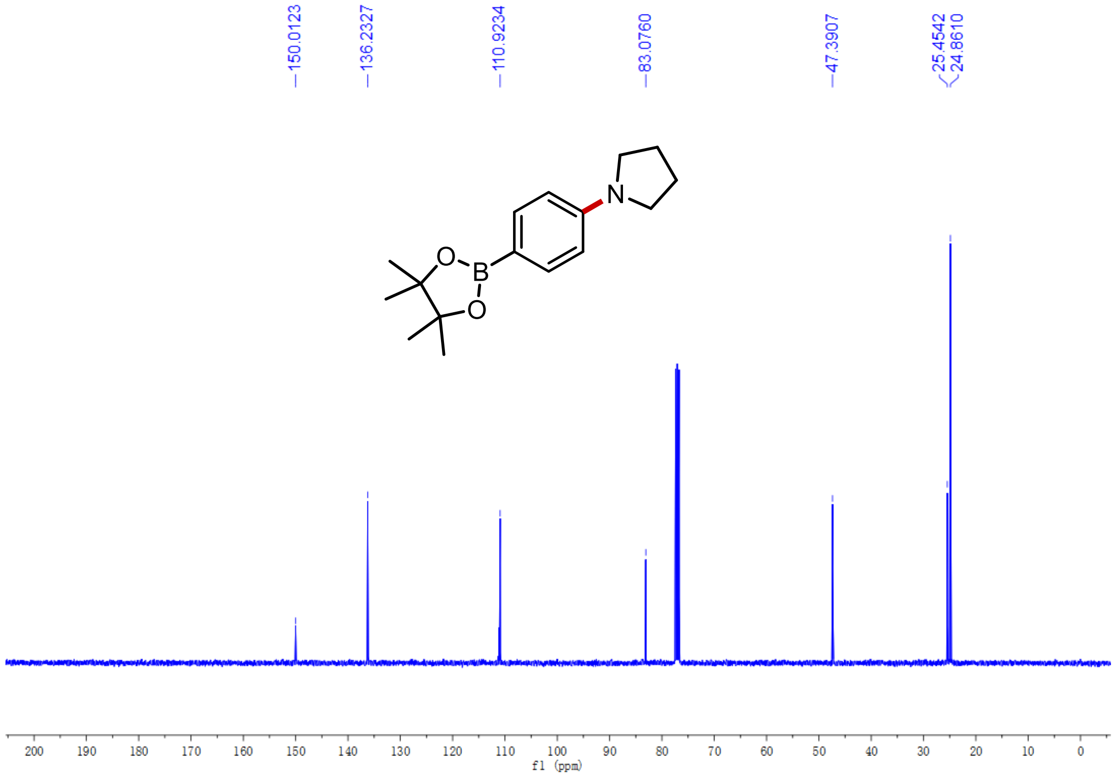

^{13}C NMR (100 MHz, $CDCl_3$) Spectrum

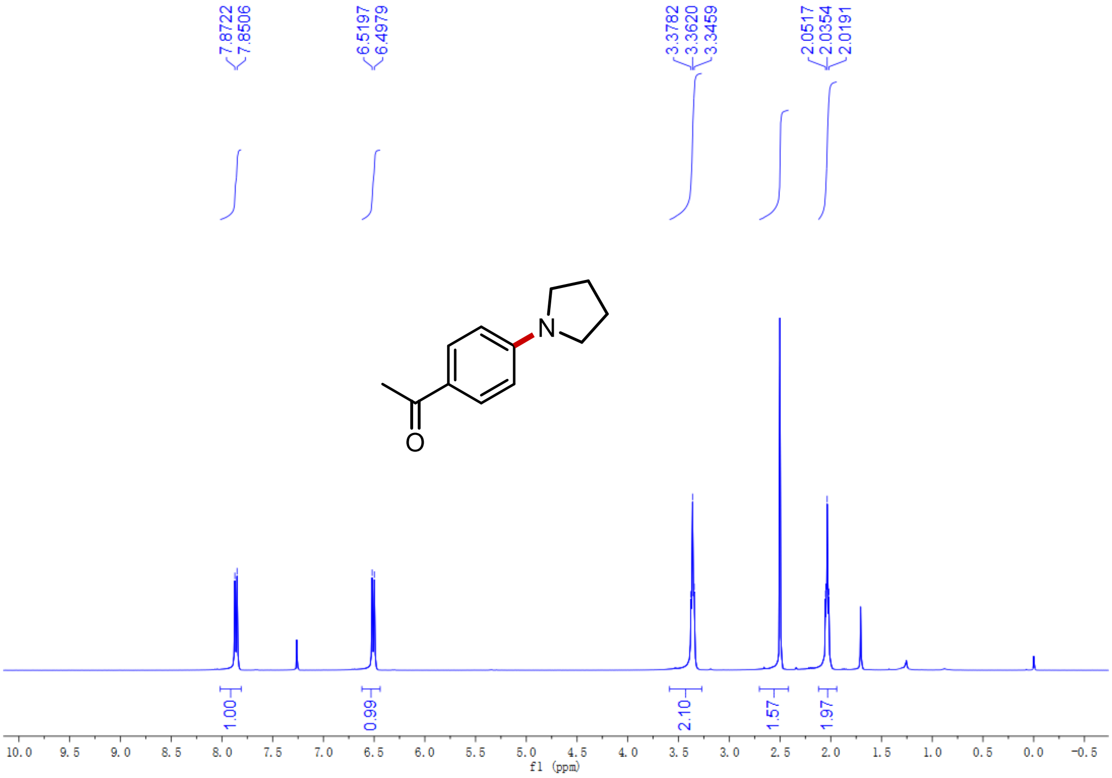

^{1}H NMR (400 MHz, $CDCl_3$) Spectrum

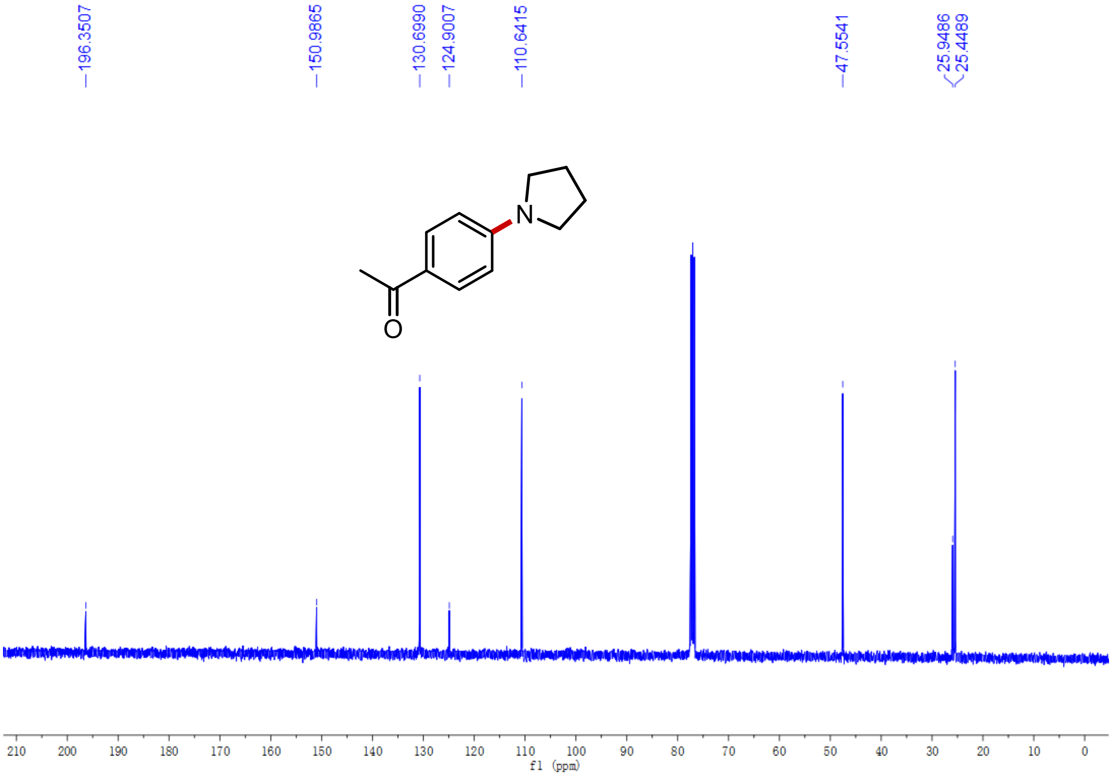

^{13}C NMR (100 MHz, $CDCl_3$) Spectrum

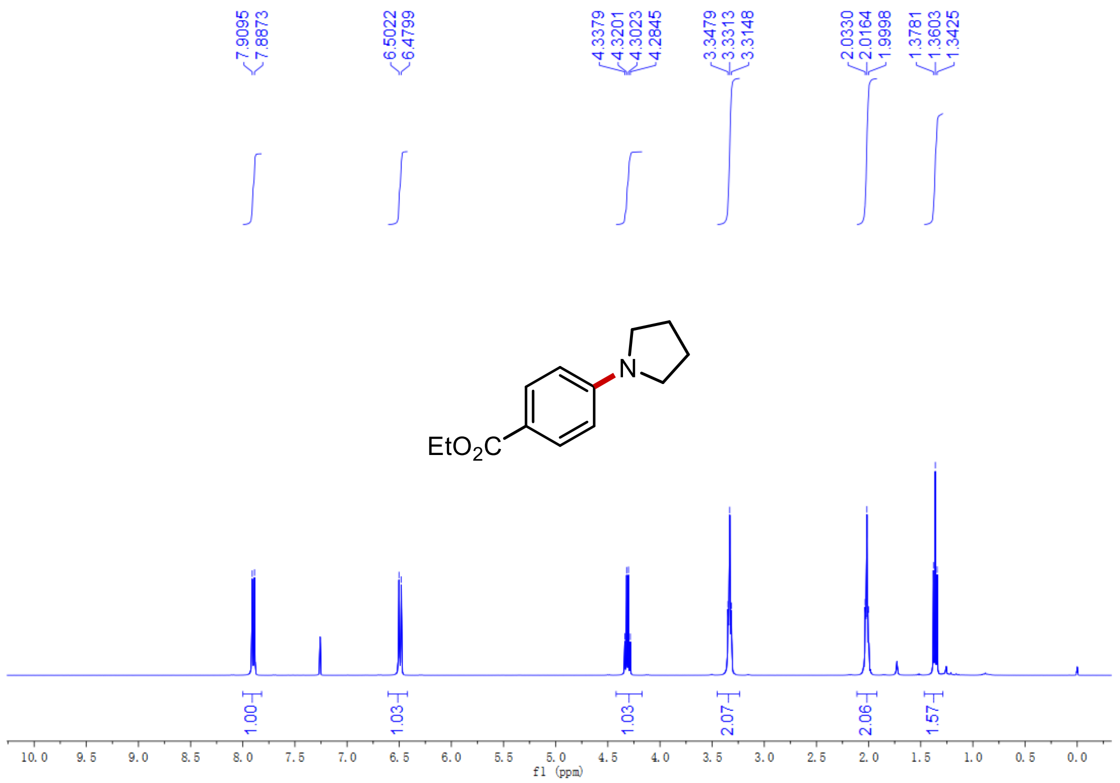

1H NMR (400 MHz, $CDCl_3$) Spectrum

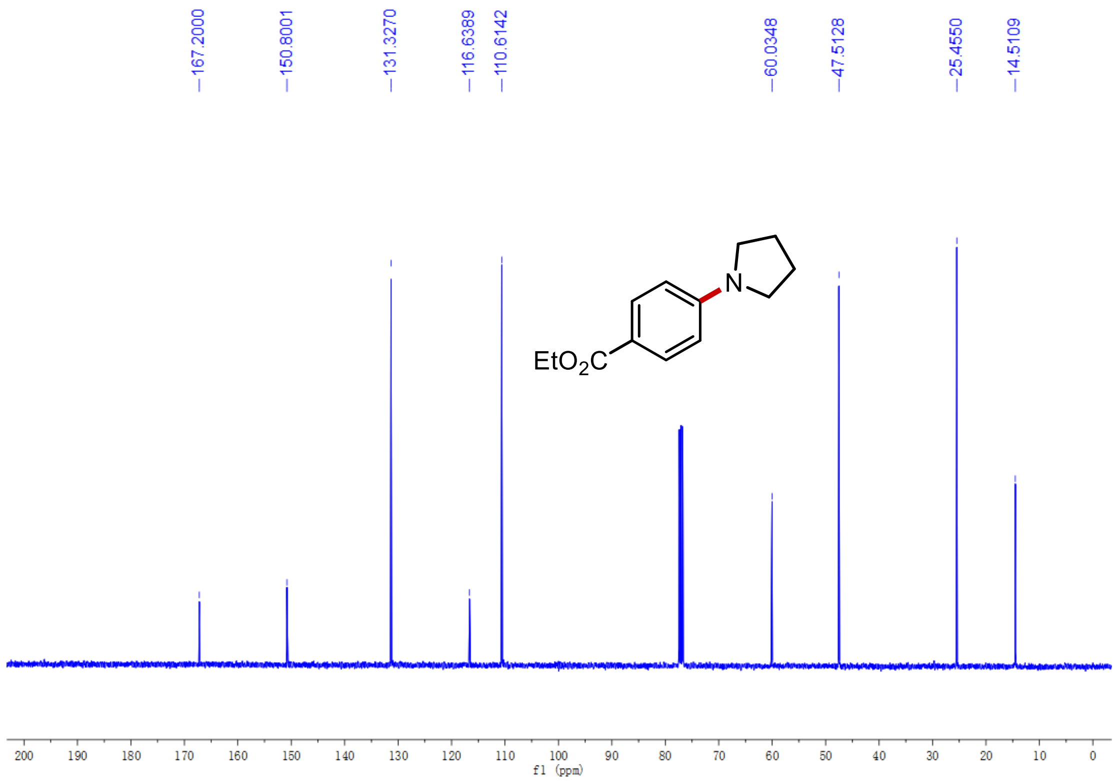

13C NMR (100 MHz, $CDCl_3$) Spectrum

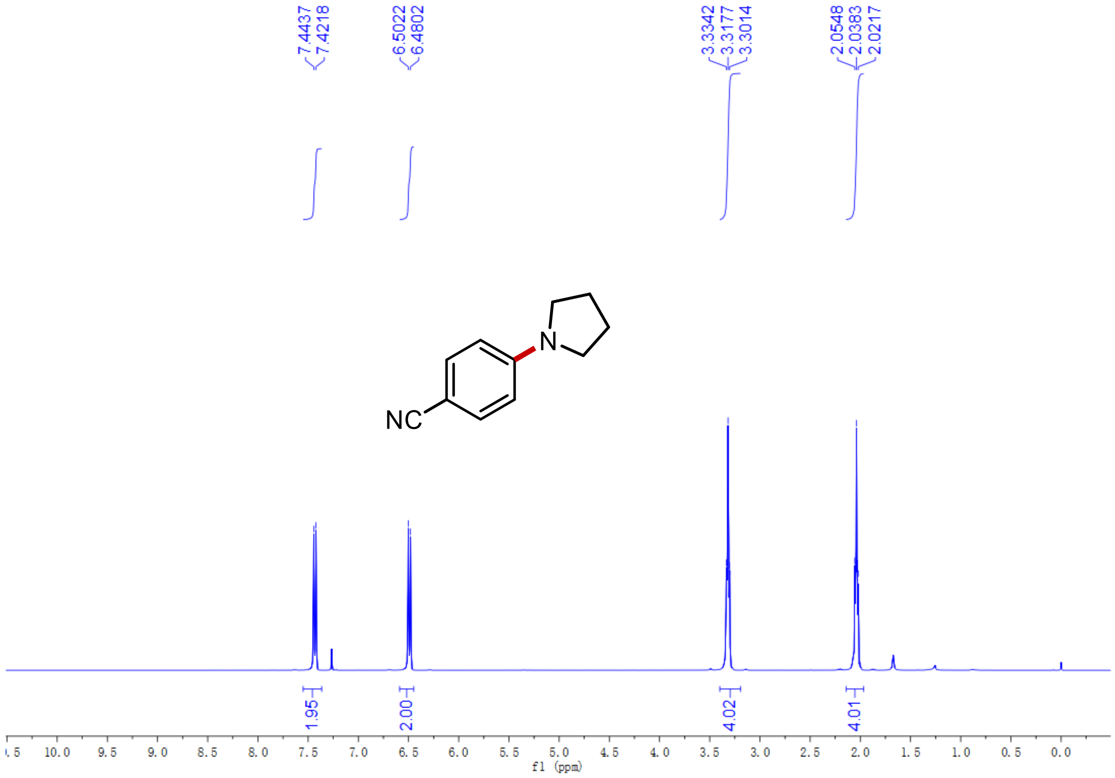

^{1}H NMR (400 MHz, $CDCl_3$) Spectrum

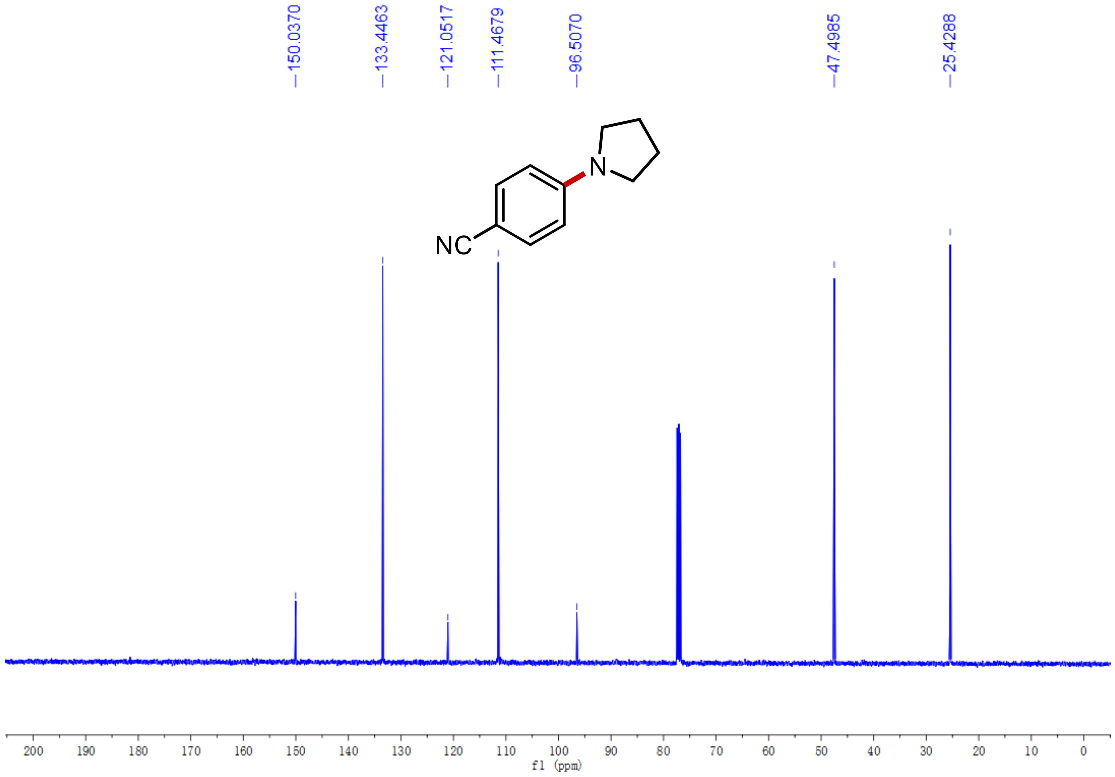

^{13}C NMR (100 MHz, $CDCl_3$) Spectrum

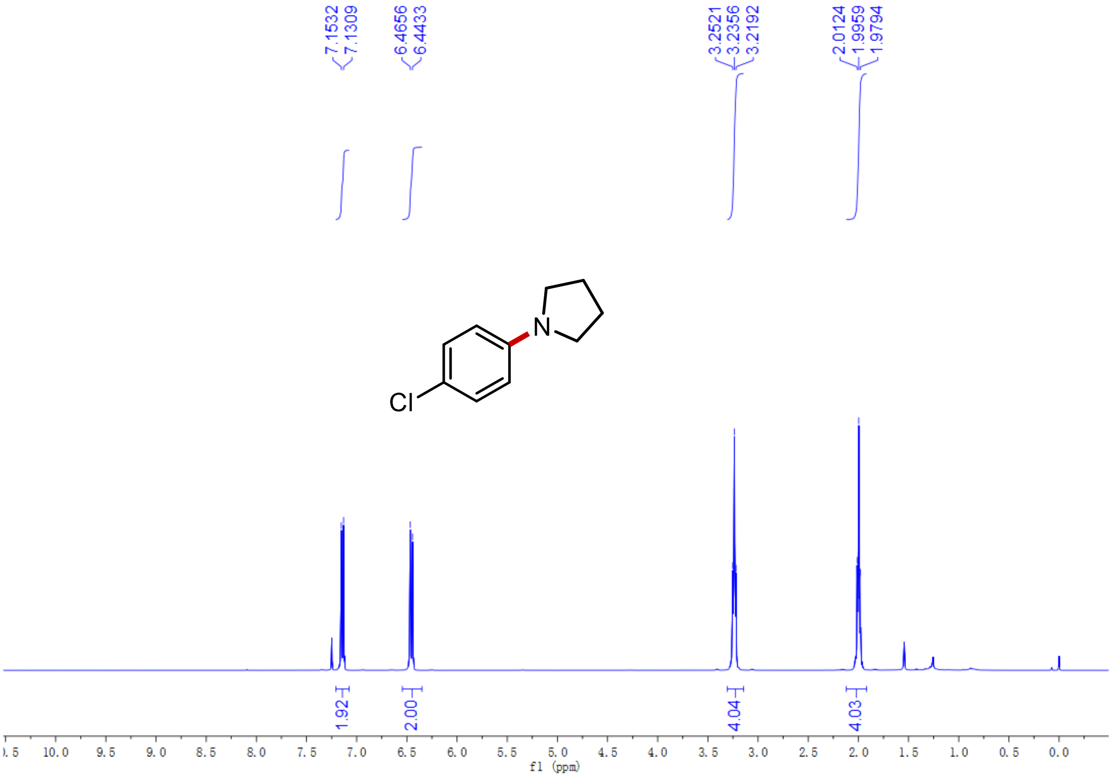

^{1}**H NMR** (400 MHz, $CDCl_3$) Spectrum

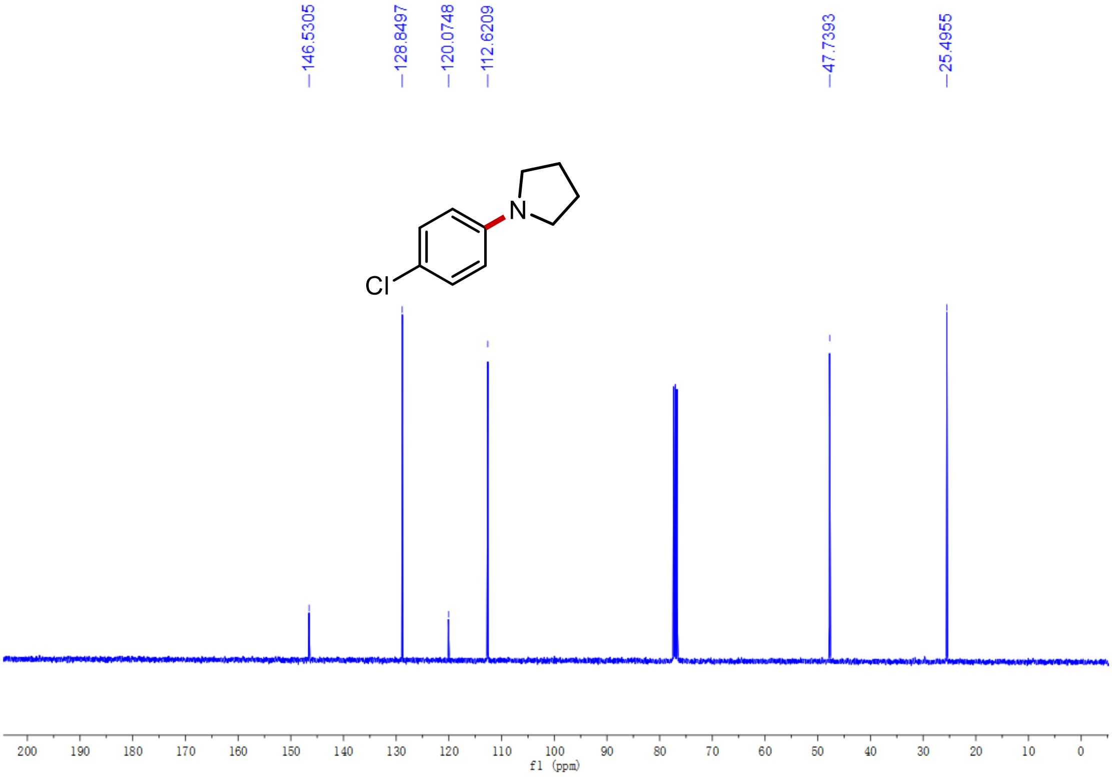

^{13}**C NMR** (100 MHz, $CDCl_3$) Spectrum

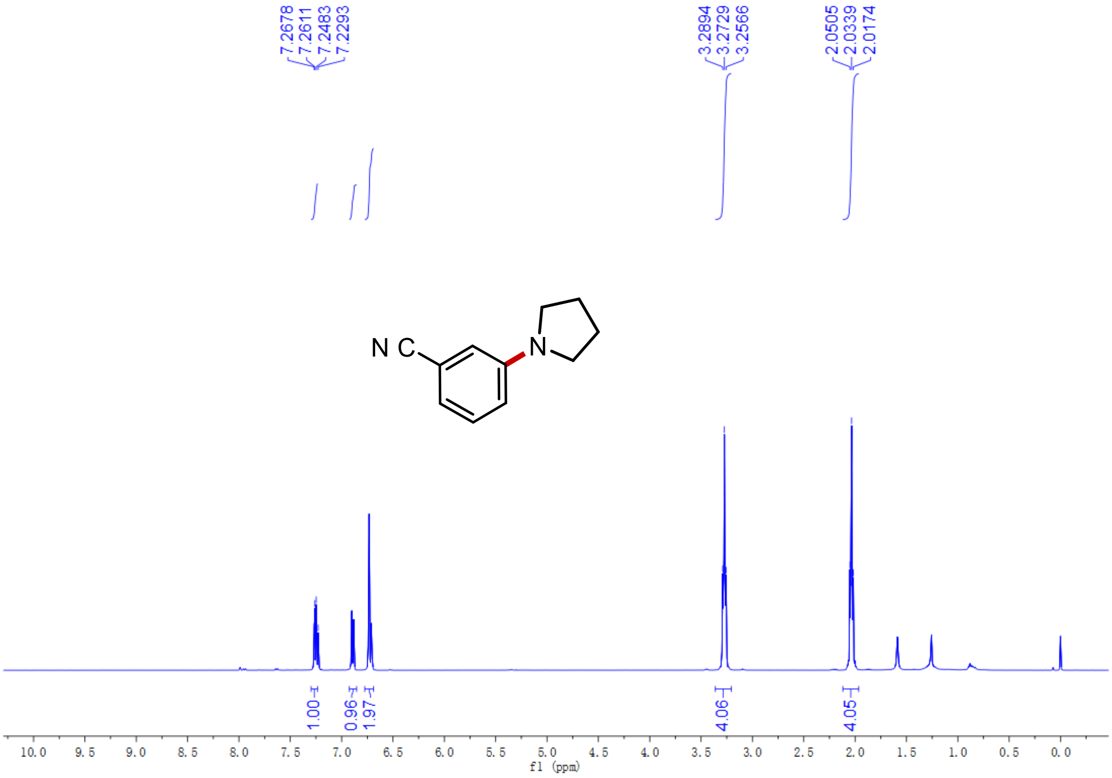

^{1}H NMR (400 MHz, $CDCl_3$) Spectrum

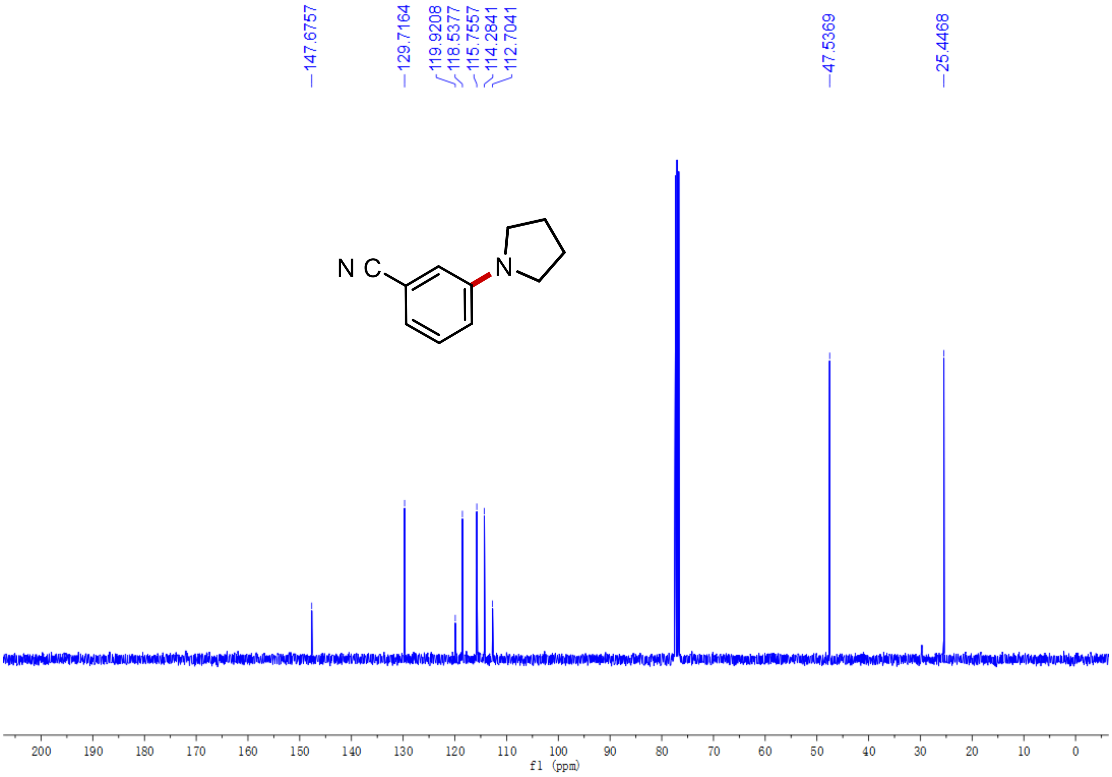

^{13}C NMR (100 MHz, $CDCl_3$) Spectrum

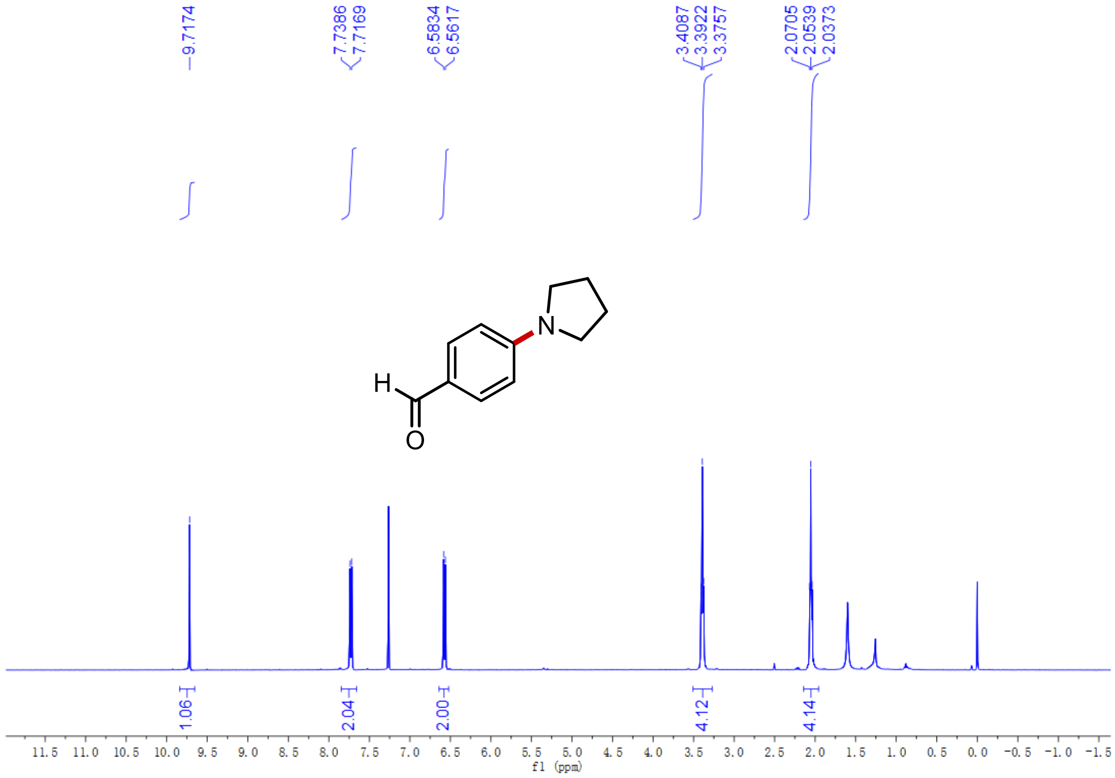

^{1}H NMR (400 MHz, $CDCl_3$) Spectrum

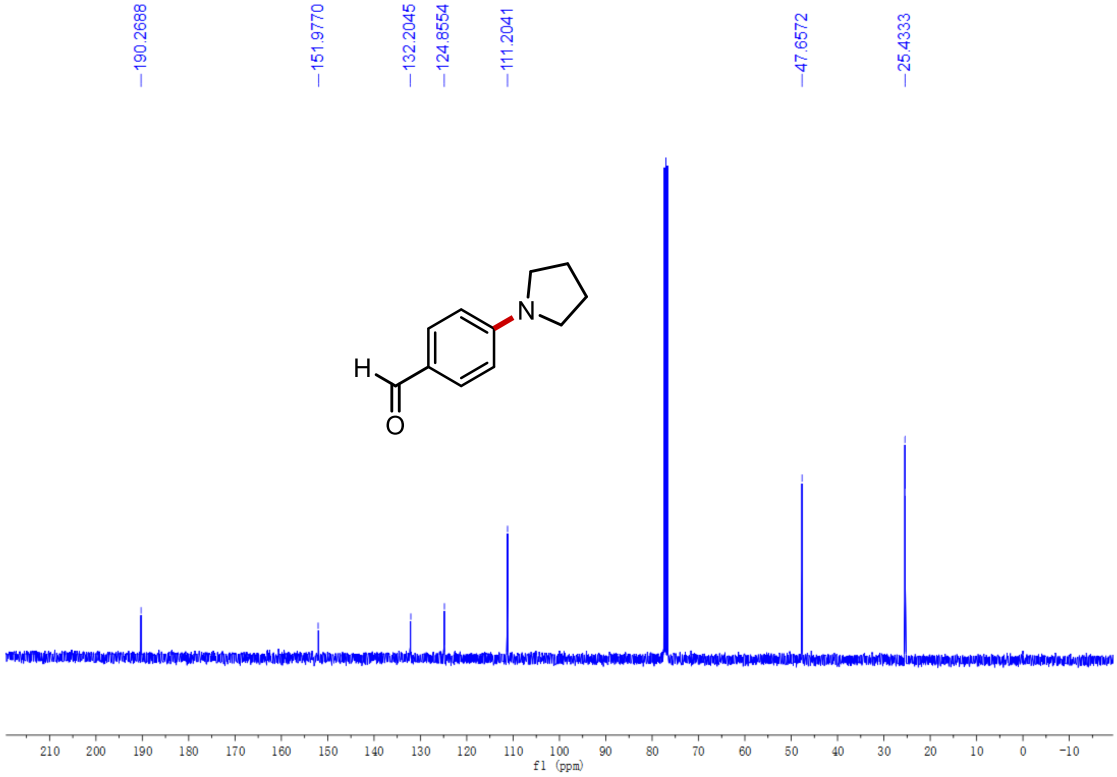

^{13}C NMR (100 MHz, $CDCl_3$) Spectrum

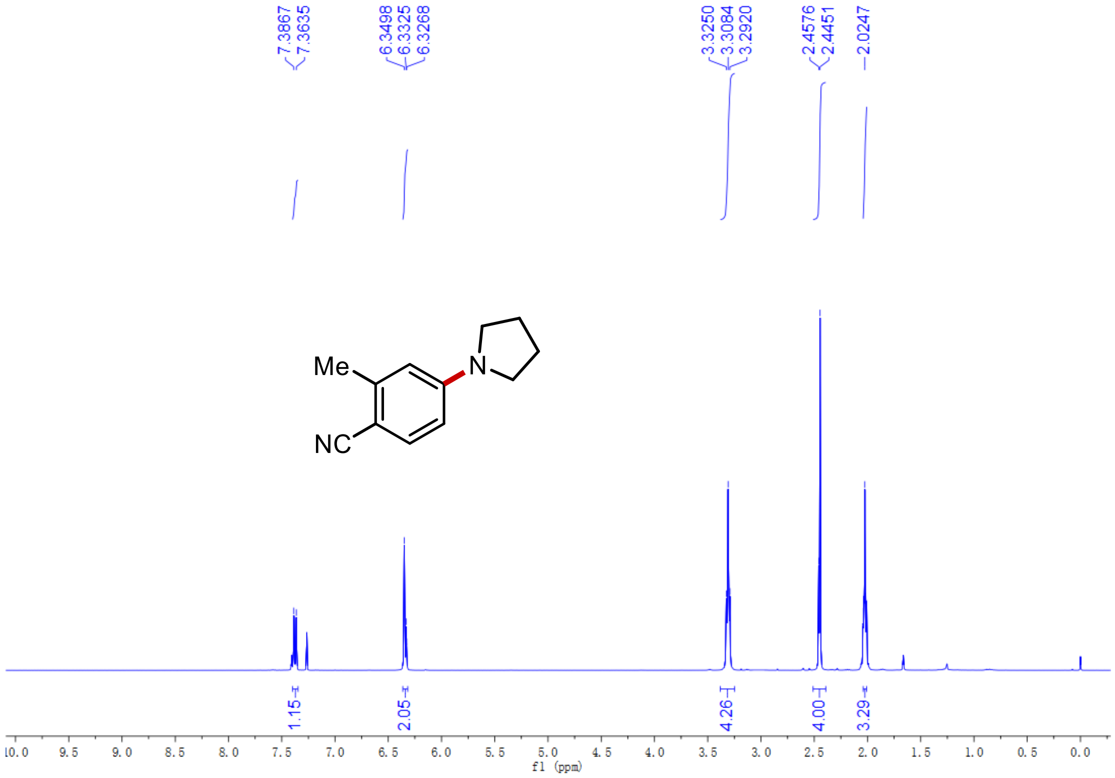

^{1}**H NMR** (400 MHz, $CDCl_3$) Spectrum

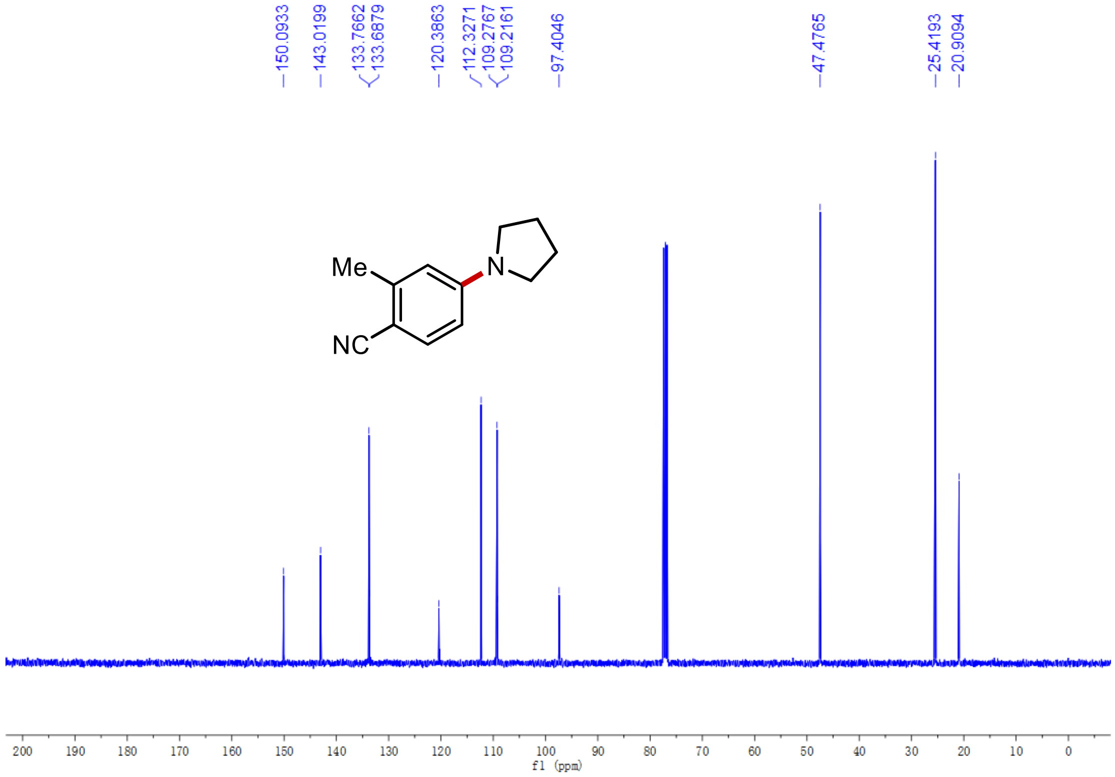

^{13}**C NMR** (100 MHz, $CDCl_3$) Spectrum

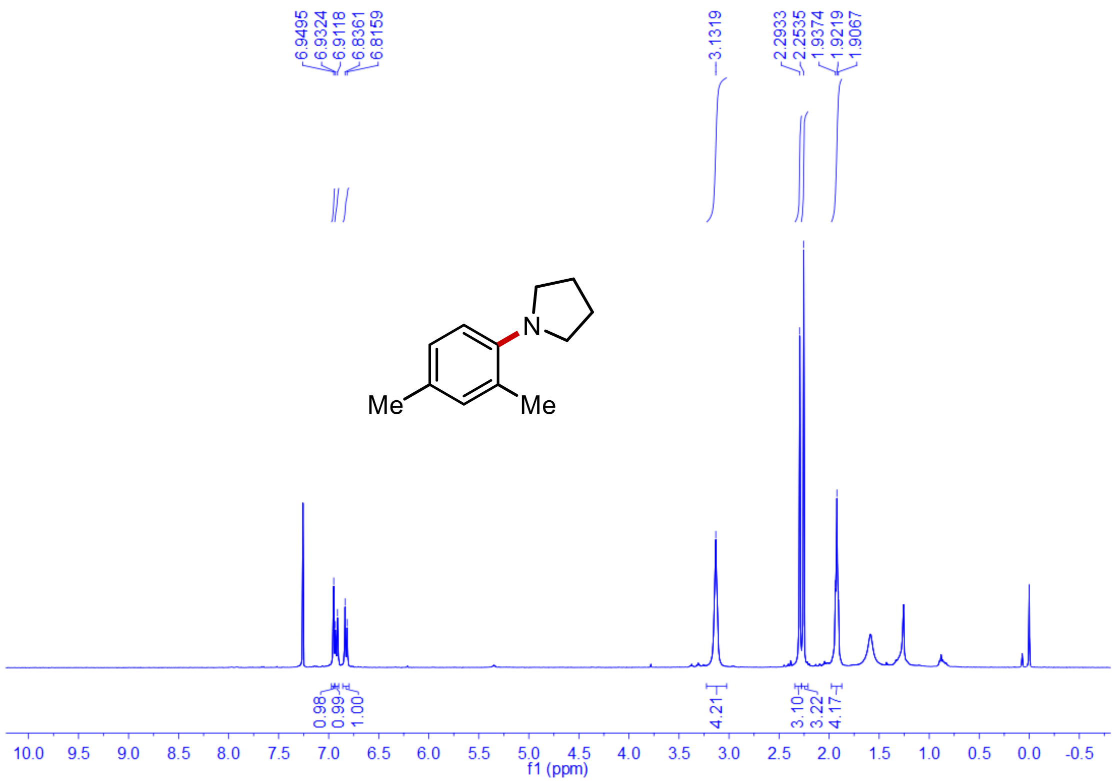

^{1}H NMR (400 MHz, $CDCl_3$) Spectrum

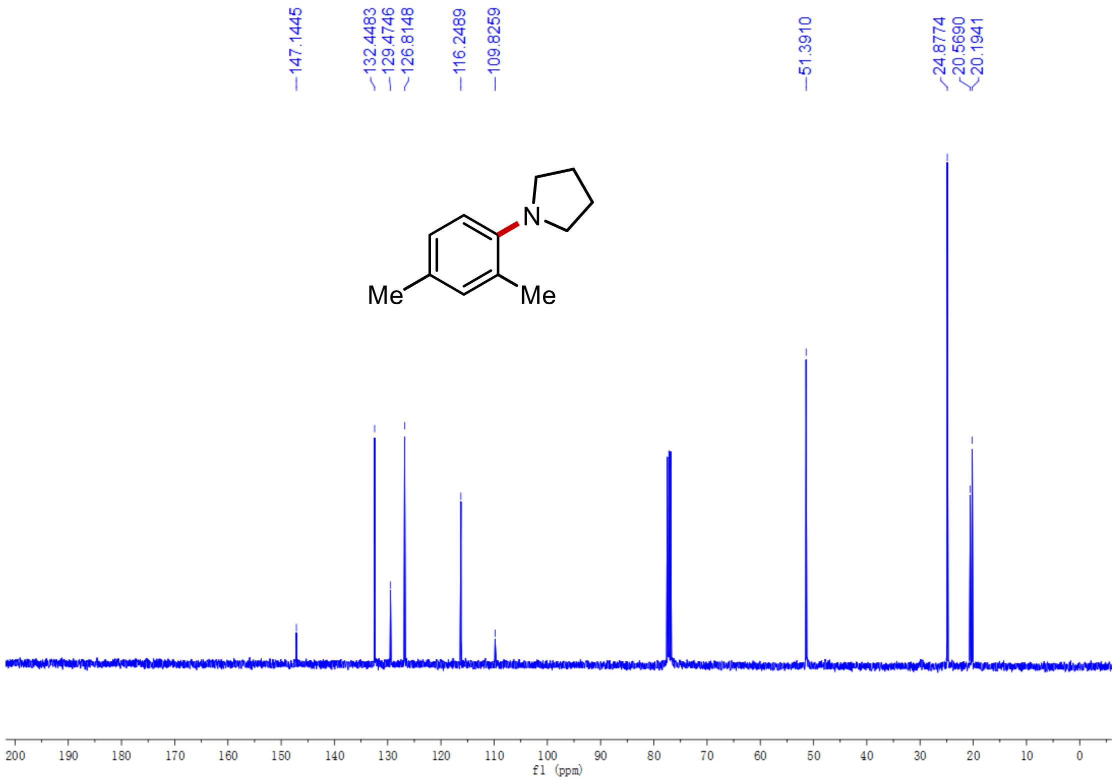

^{13}C NMR (100 MHz, $CDCl_3$) Spectrum

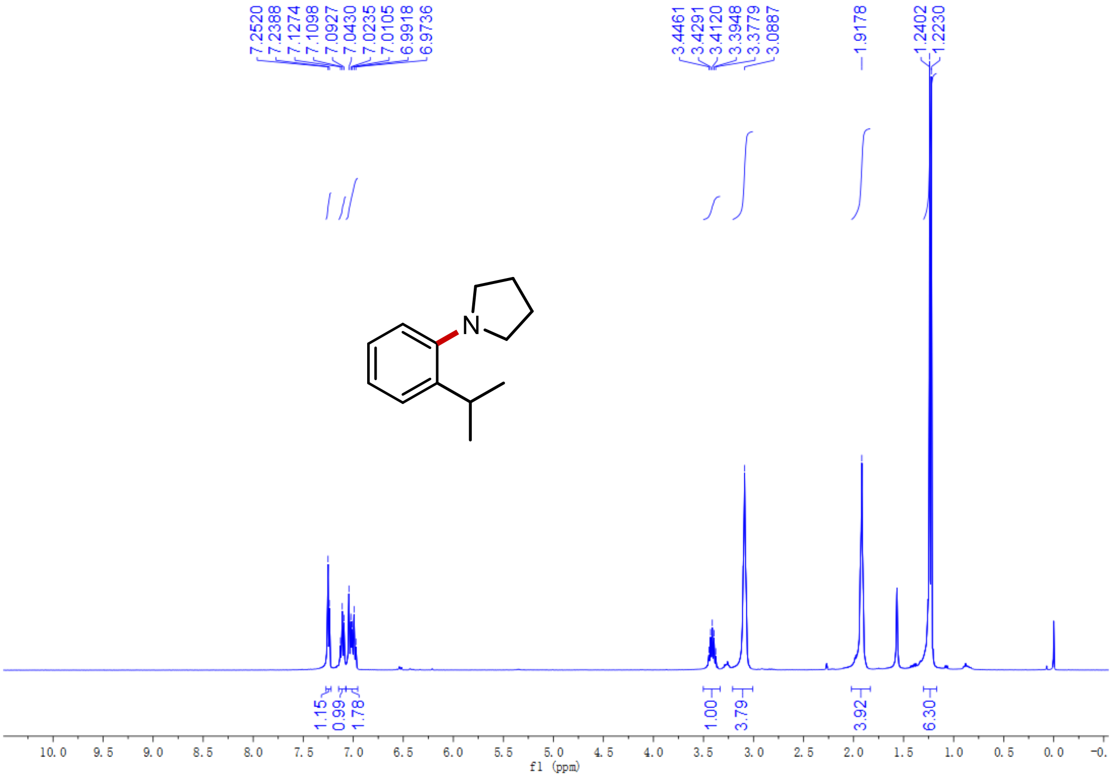

¹H NMR (400 MHz, $CDCl_3$) Spectrum

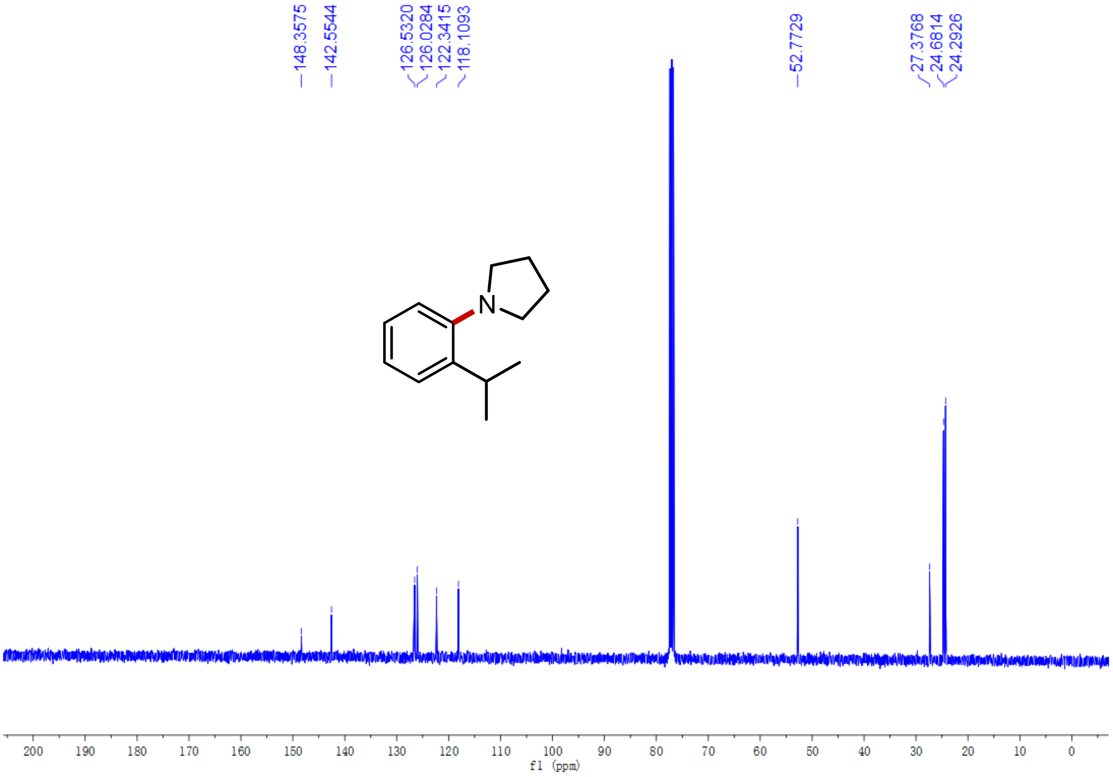

¹³C NMR (100 MHz, $CDCl_3$) Spectrum

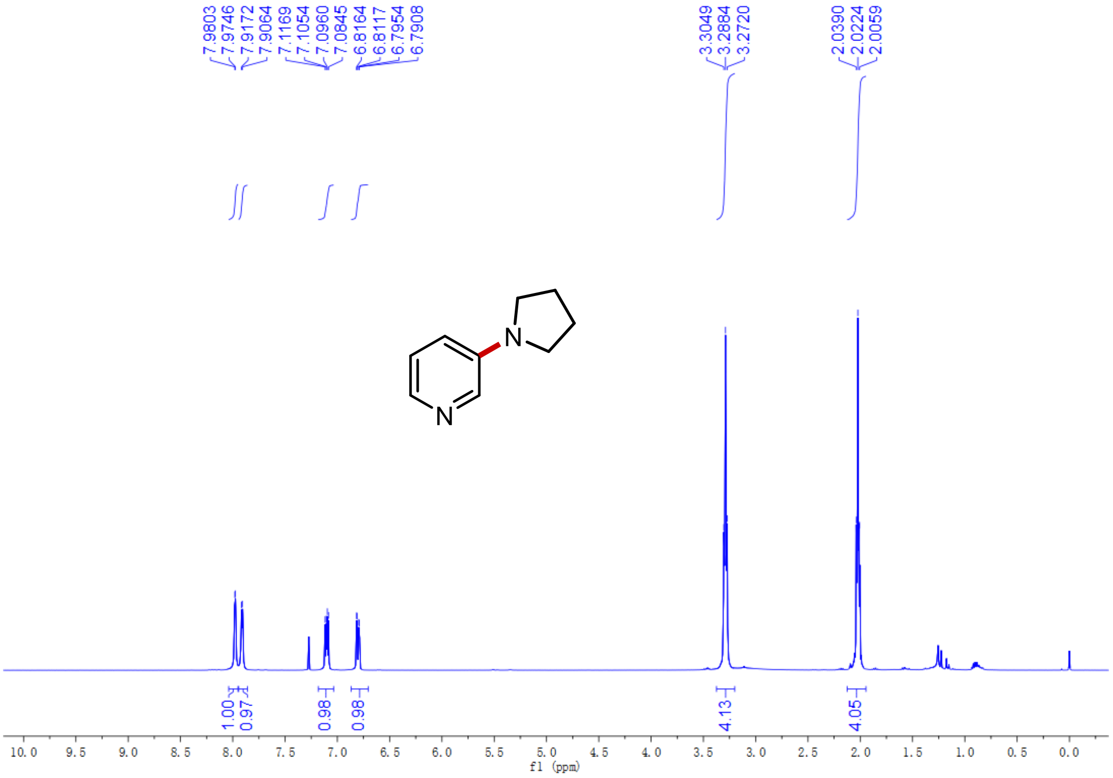

^{1}H NMR (400 MHz, $CDCl_3$) Spectrum

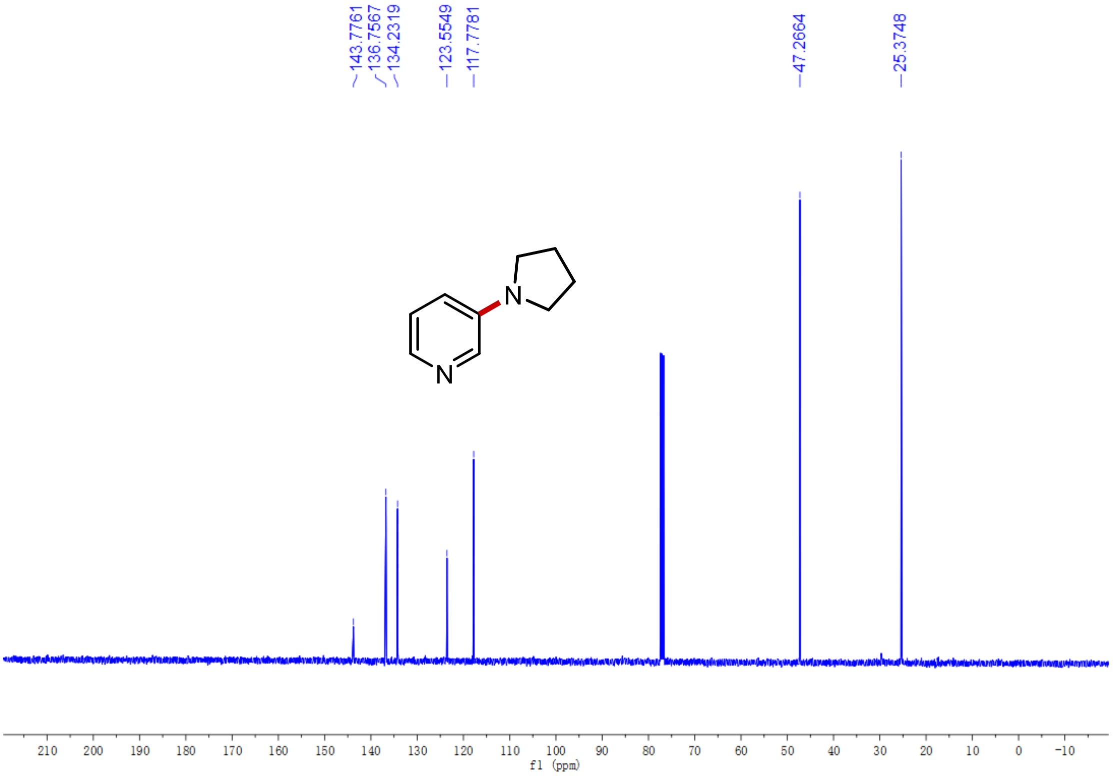

^{13}C NMR (100 MHz, $CDCl_3$) Spectrum

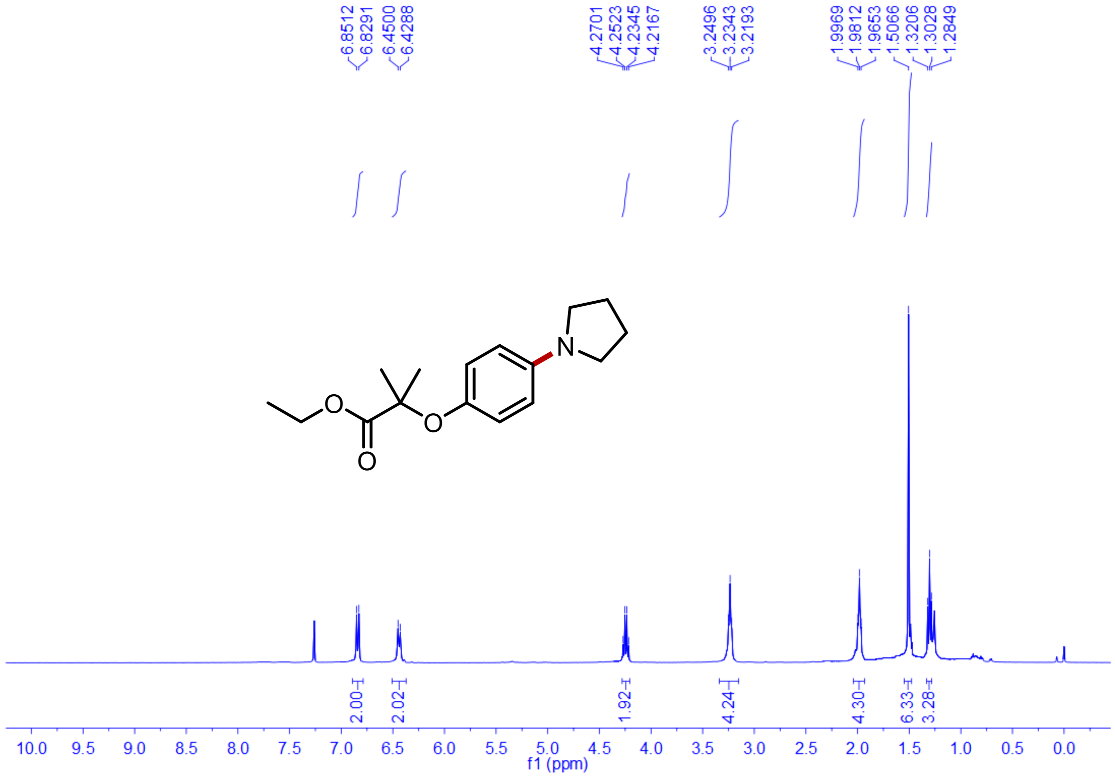

^{1}H NMR (400 MHz, $CDCl_3$) Spectrum

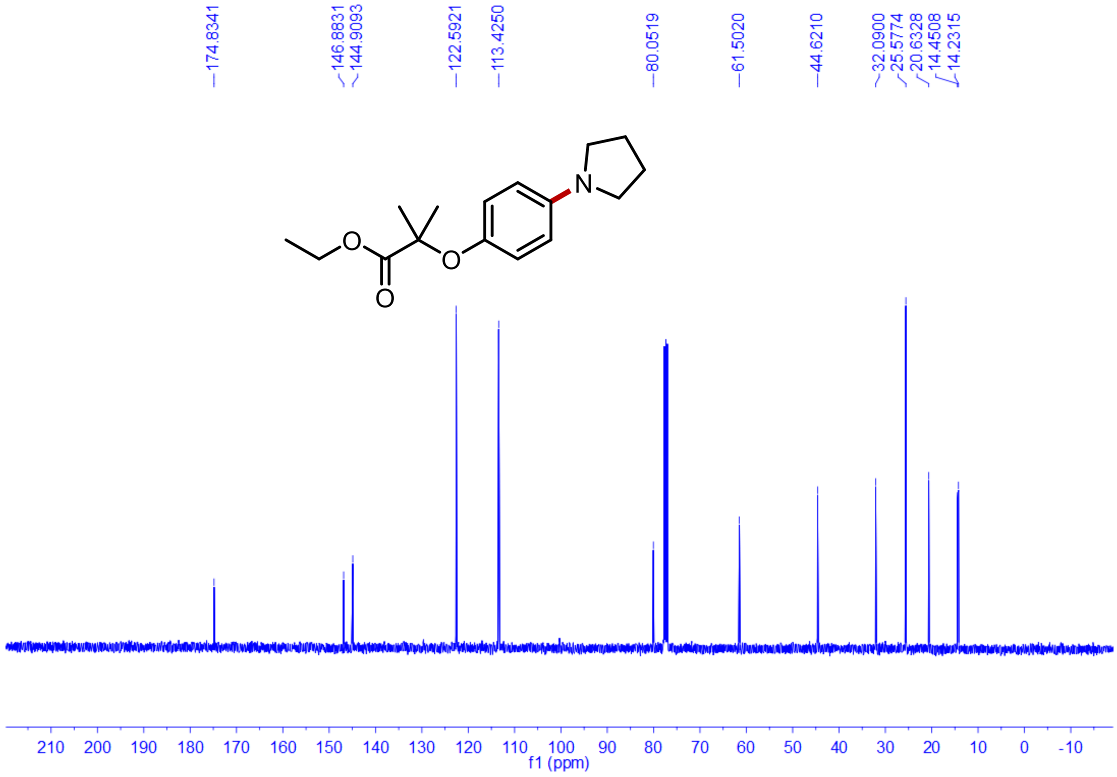

^{13}C NMR (100 MHz, $CDCl_3$) Spectrum

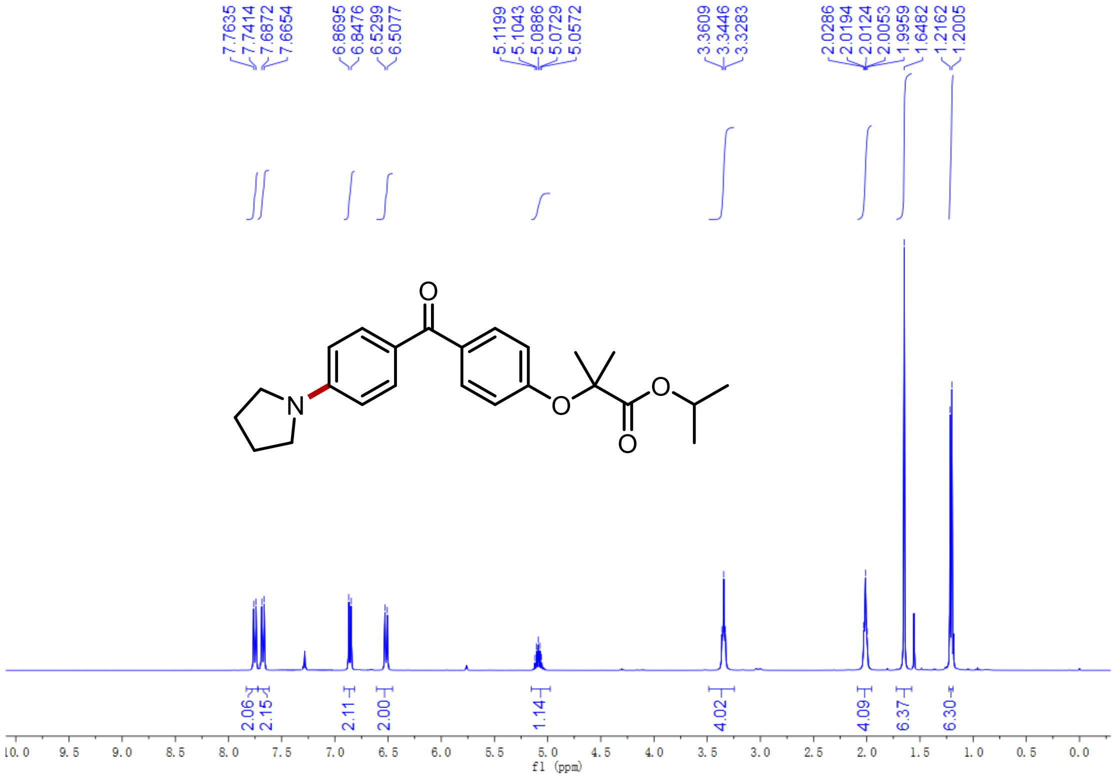

^{1}H NMR (400 MHz, $CDCl_3$) Spectrum

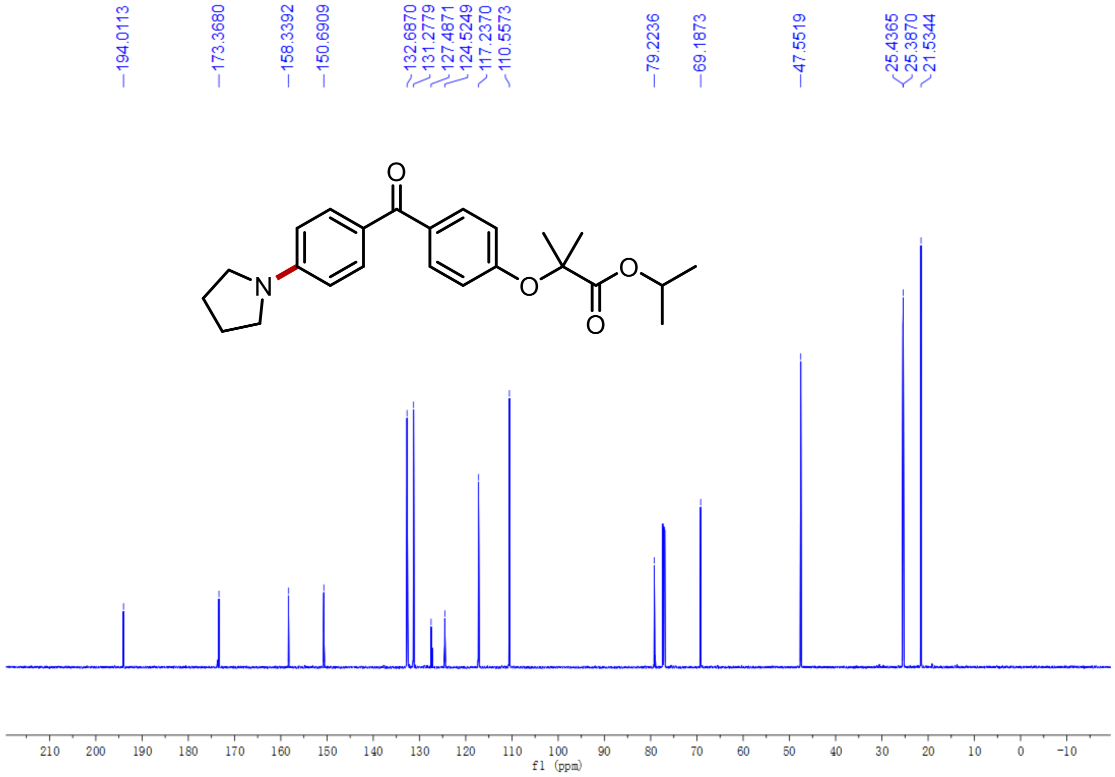

^{13}C NMR (150 MHz, $CDCl_3$) Spectrum

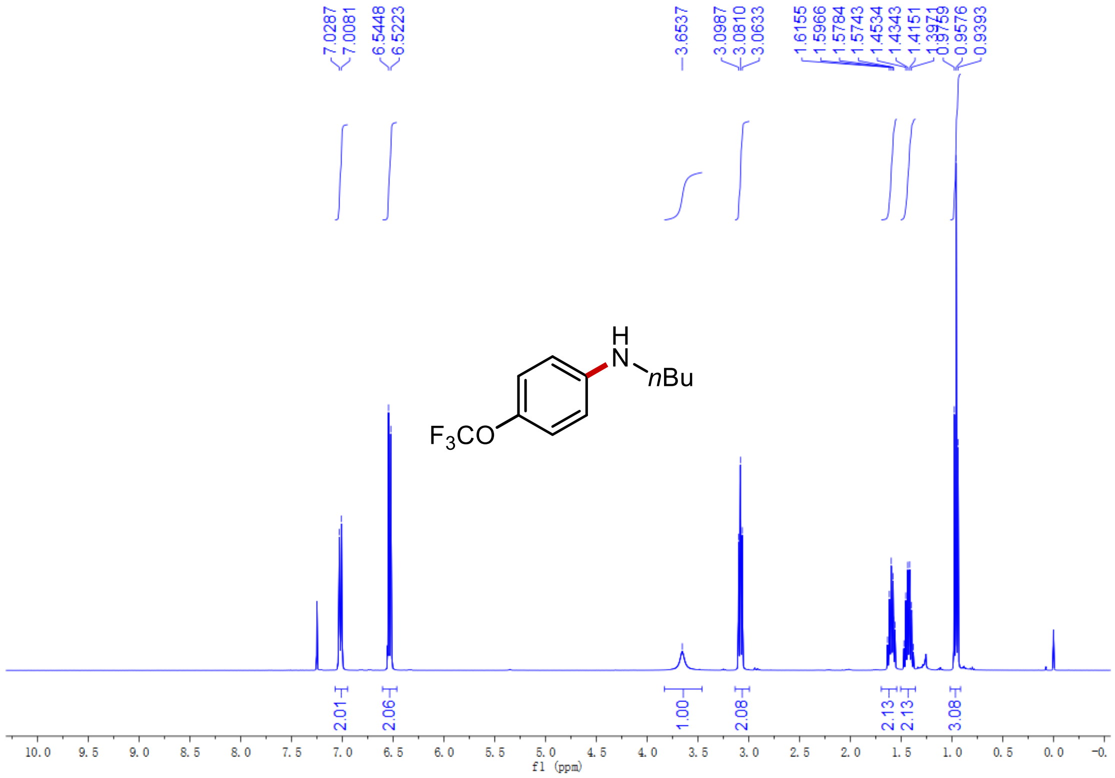

^{1}H NMR (400 MHz, $CDCl_3$) Spectrum

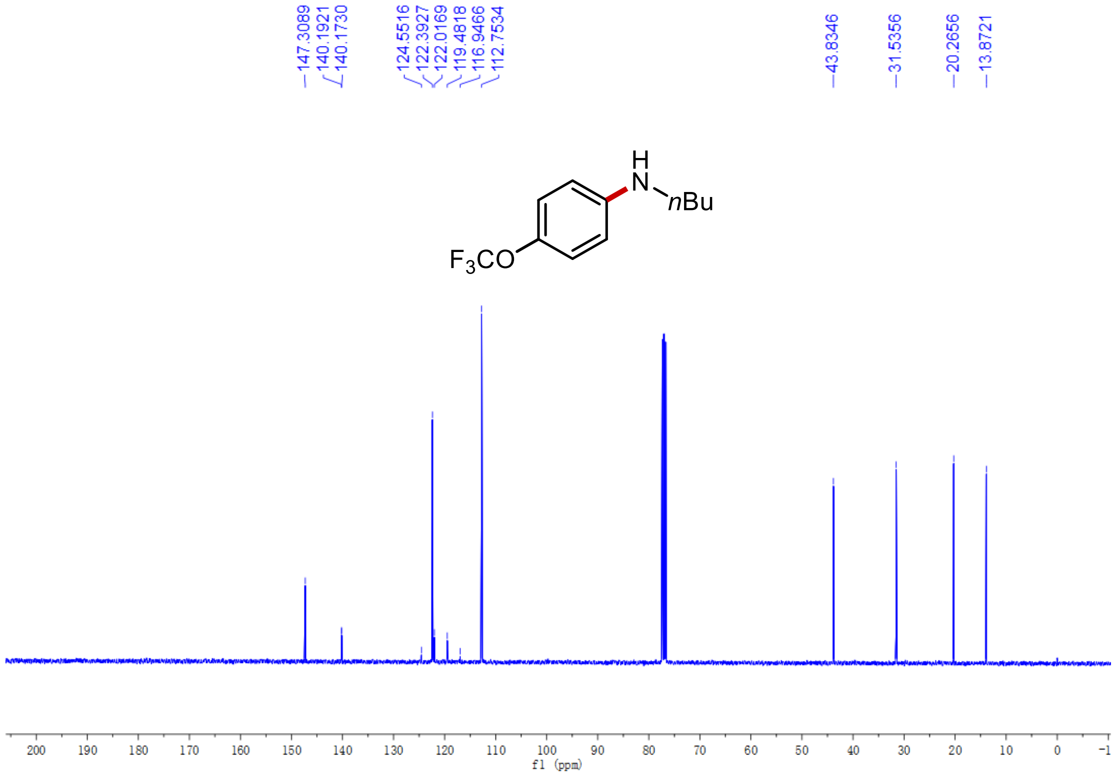

^{13}C NMR (100 MHz, $CDCl_3$) Spectrum

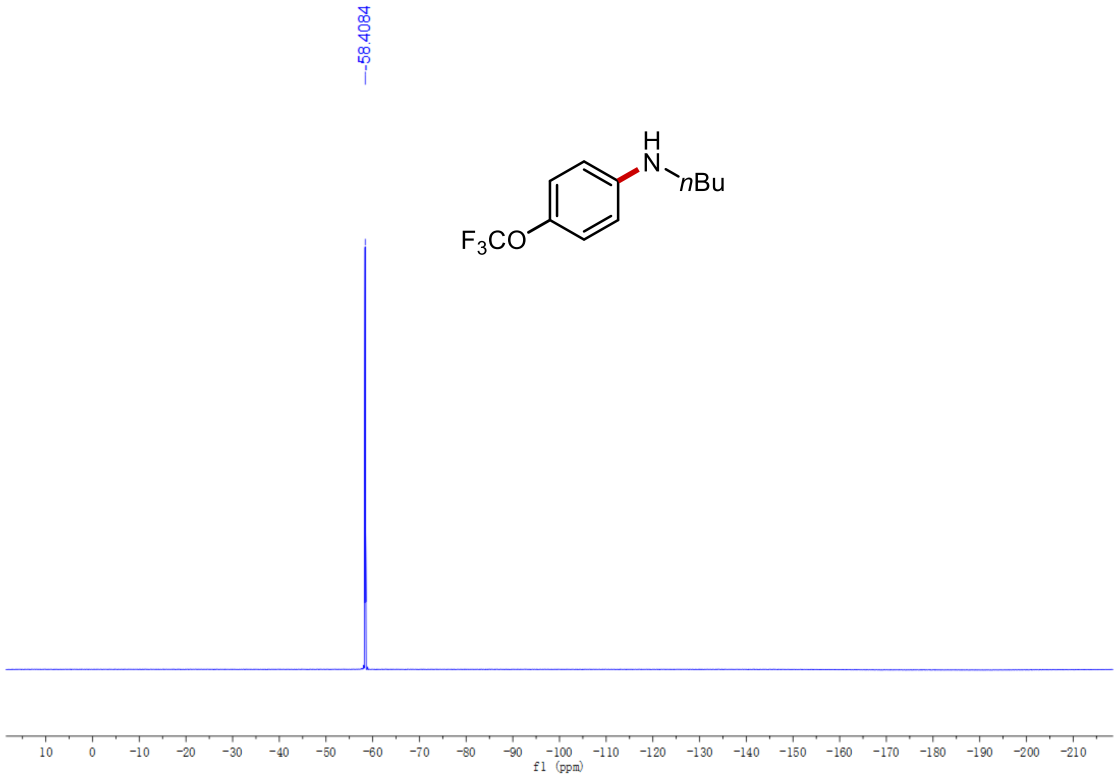

^{19}**F NMR** (376 MHz, $CDCl_3$) Spectrum

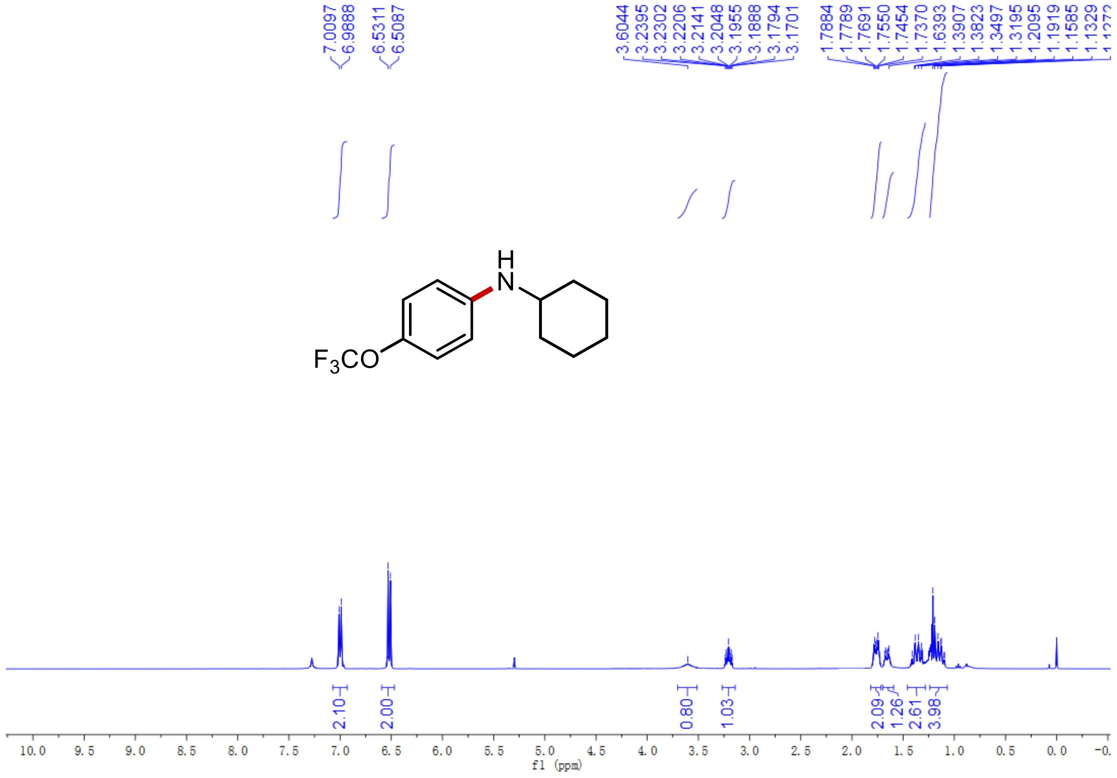

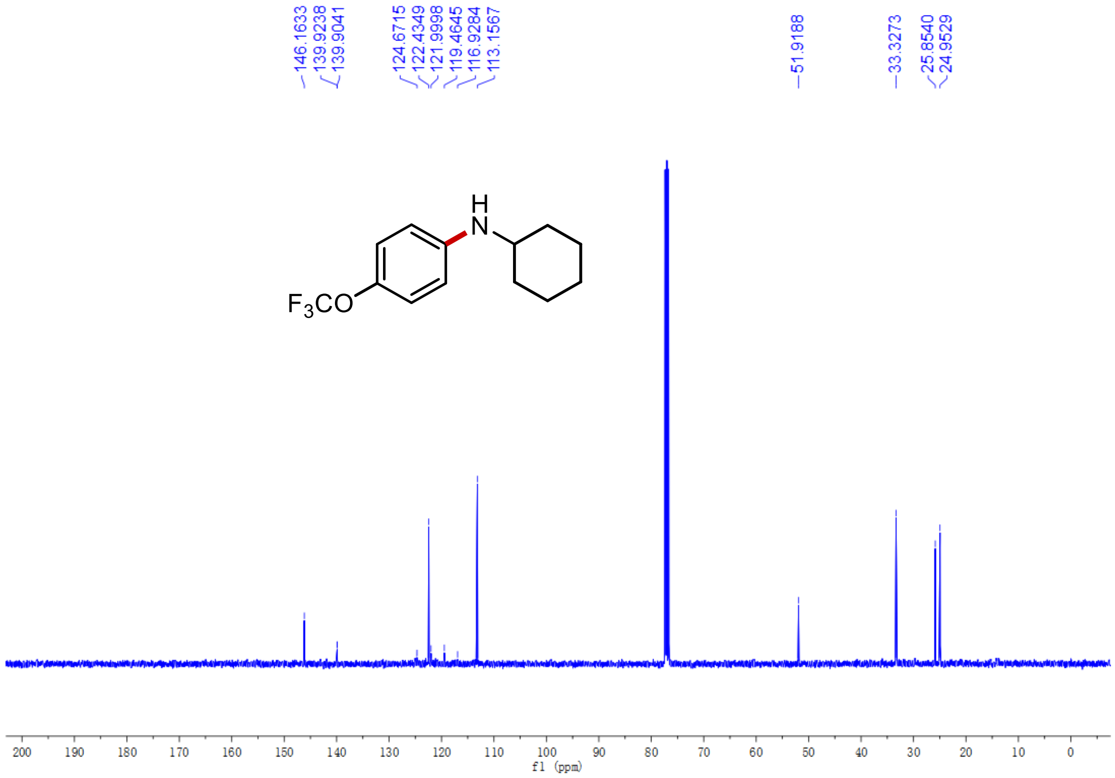

^{13}C NMR (100 MHz, $CDCl_3$) Spectrum

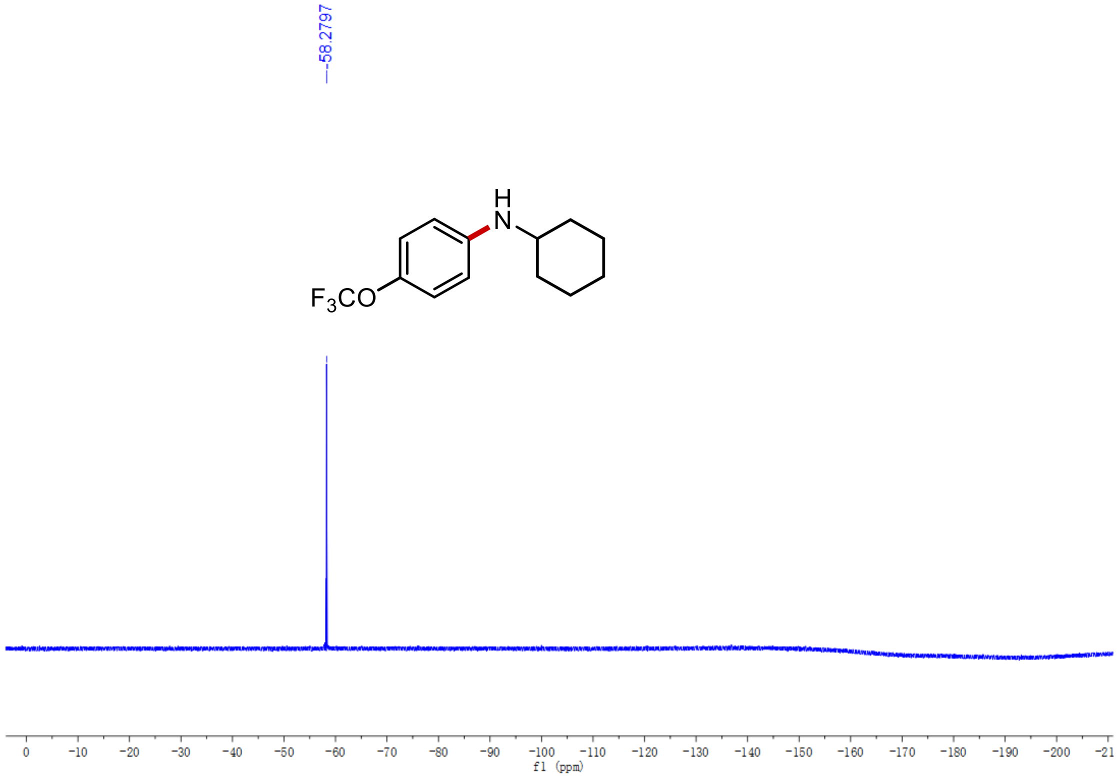

^{19}F NMR (376 MHz, $CDCl_3$) Spectrum

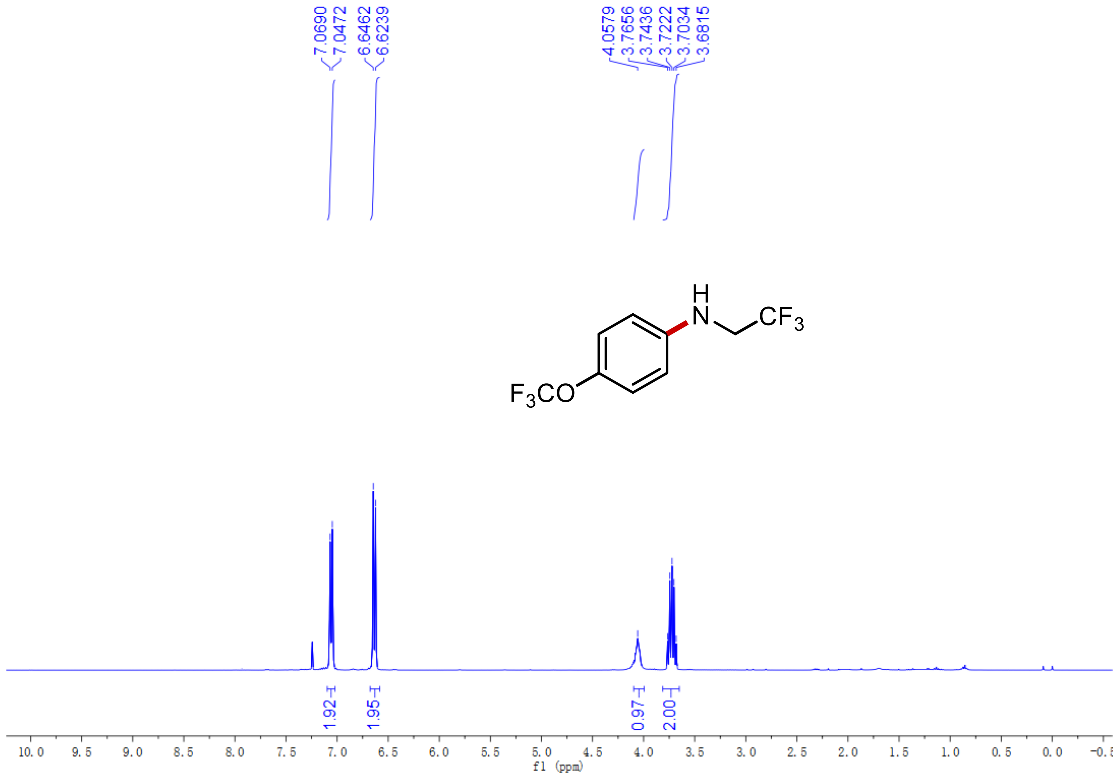

^{1}**H NMR** (400 MHz, $CDCl_3$) Spectrum

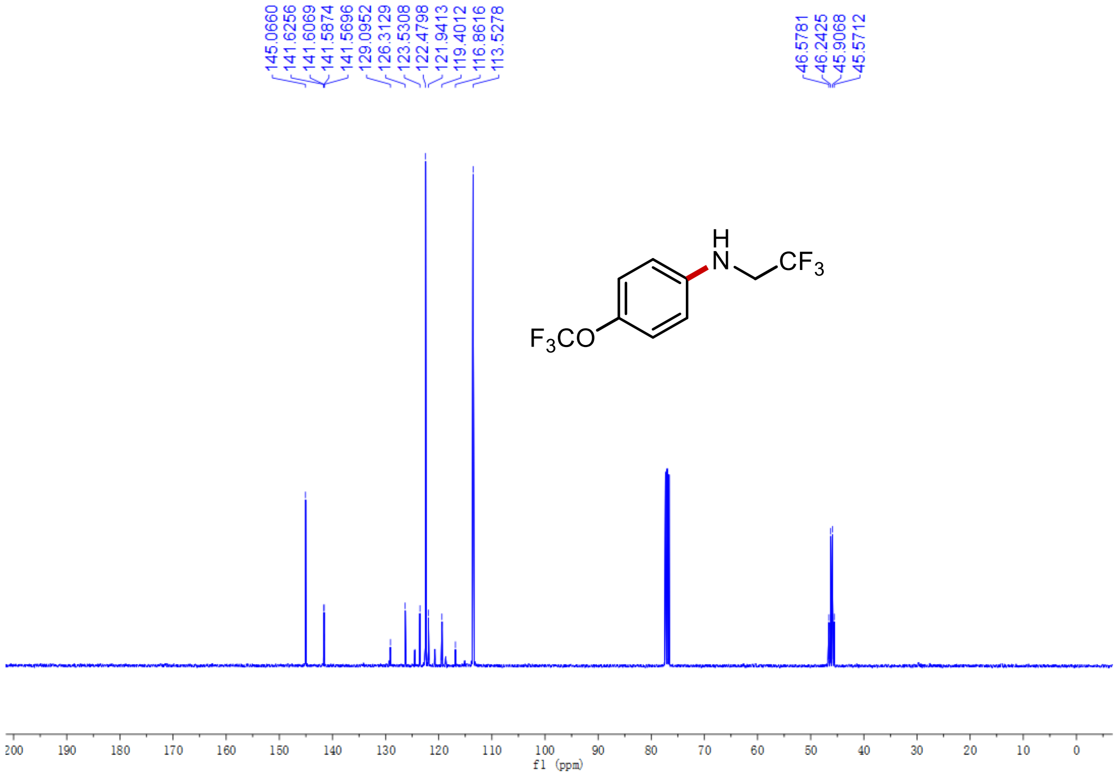

^{13}**C NMR** (100 MHz, $CDCl_3$) Spectrum

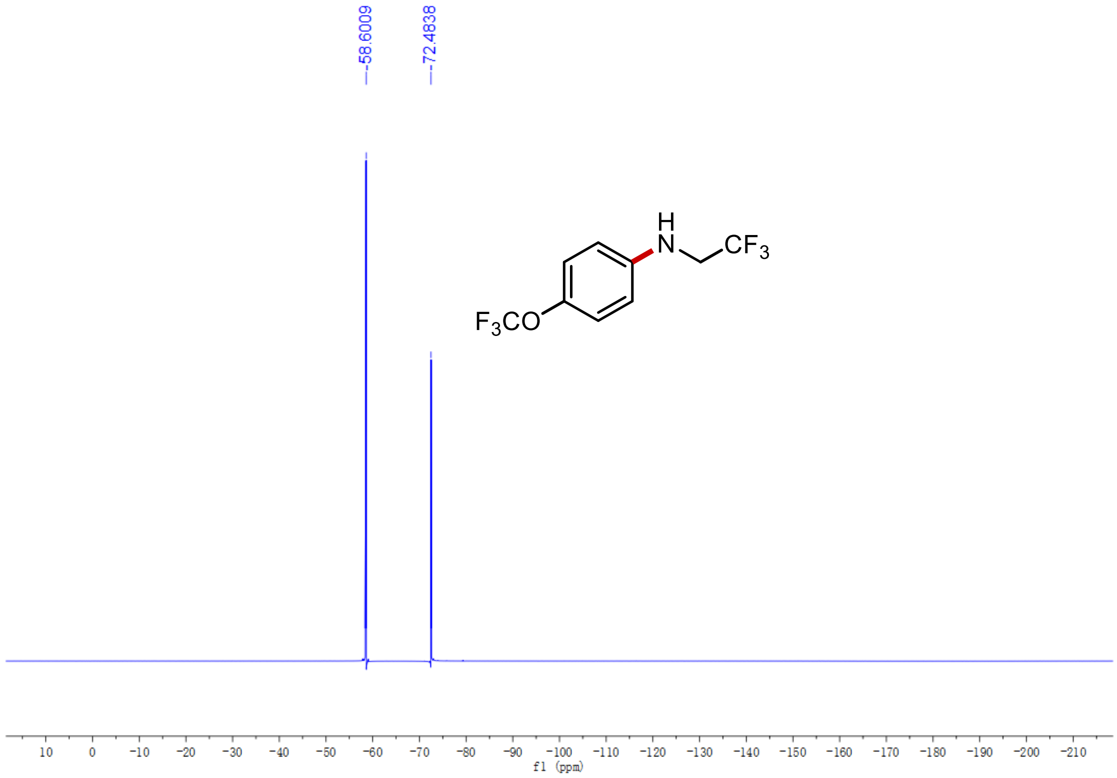

^{19}F NMR (376 MHz, $CDCl_3$) Spectrum

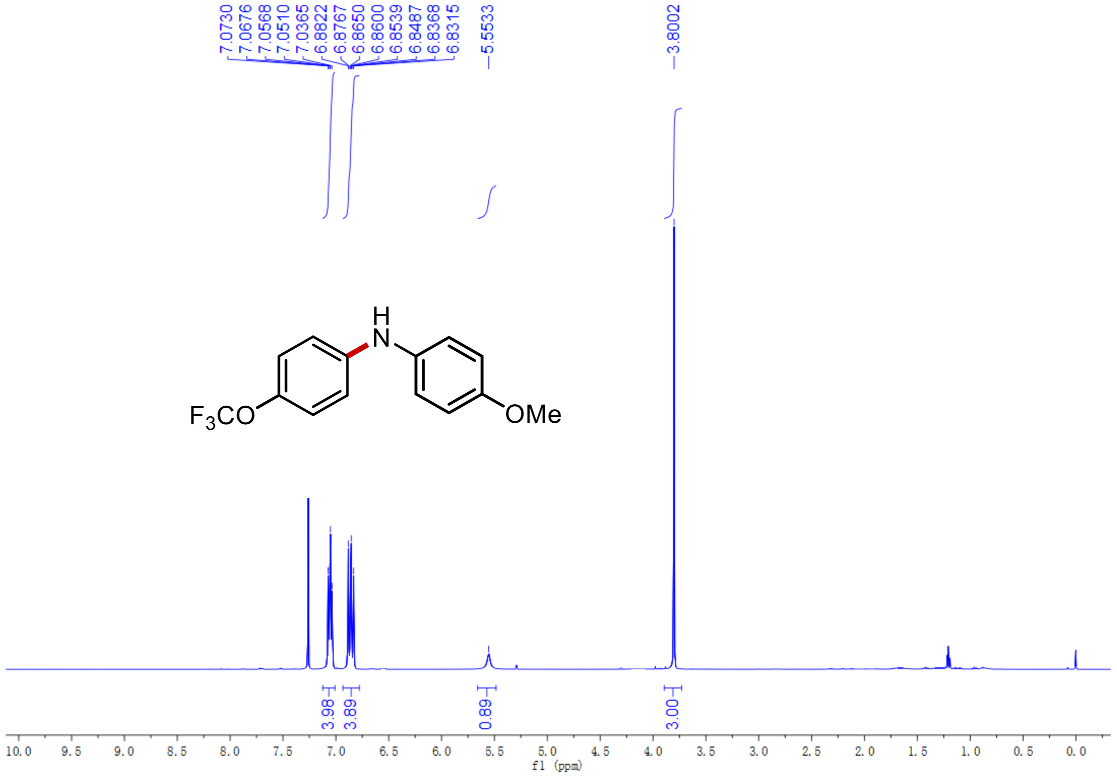

^{1}H NMR (400 MHz, $CDCl_3$) Spectrum

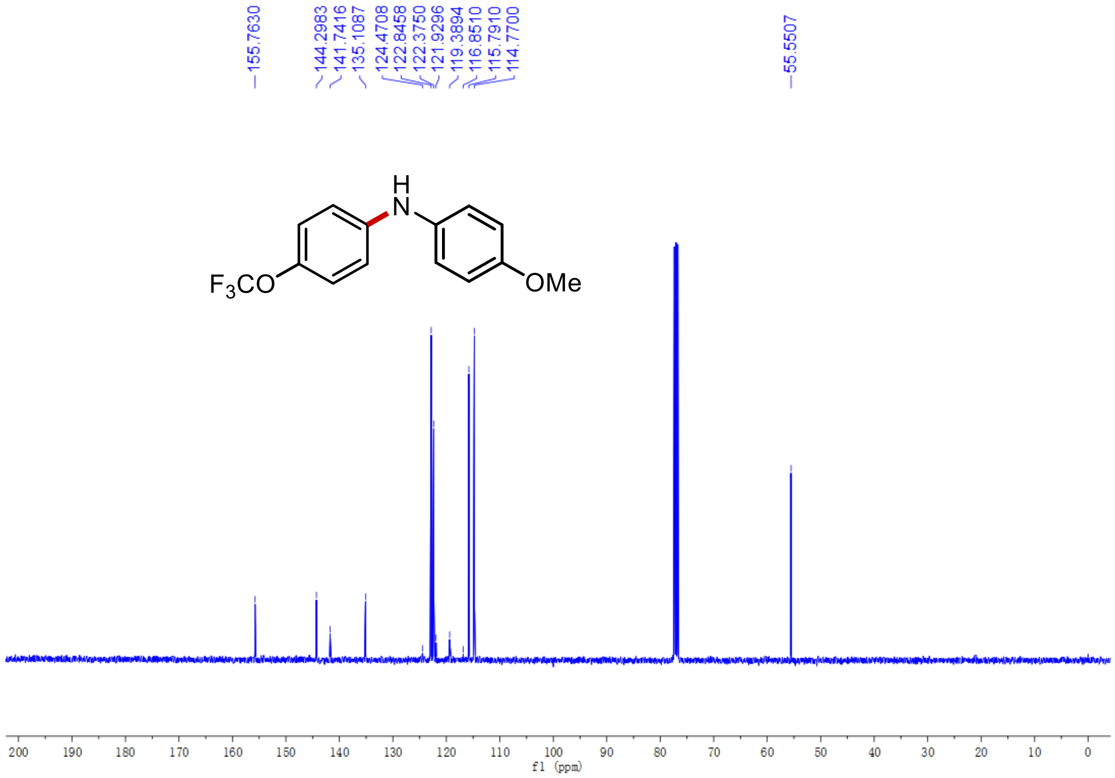

^{13}C NMR (100 MHz, $CDCl_3$) Spectrum

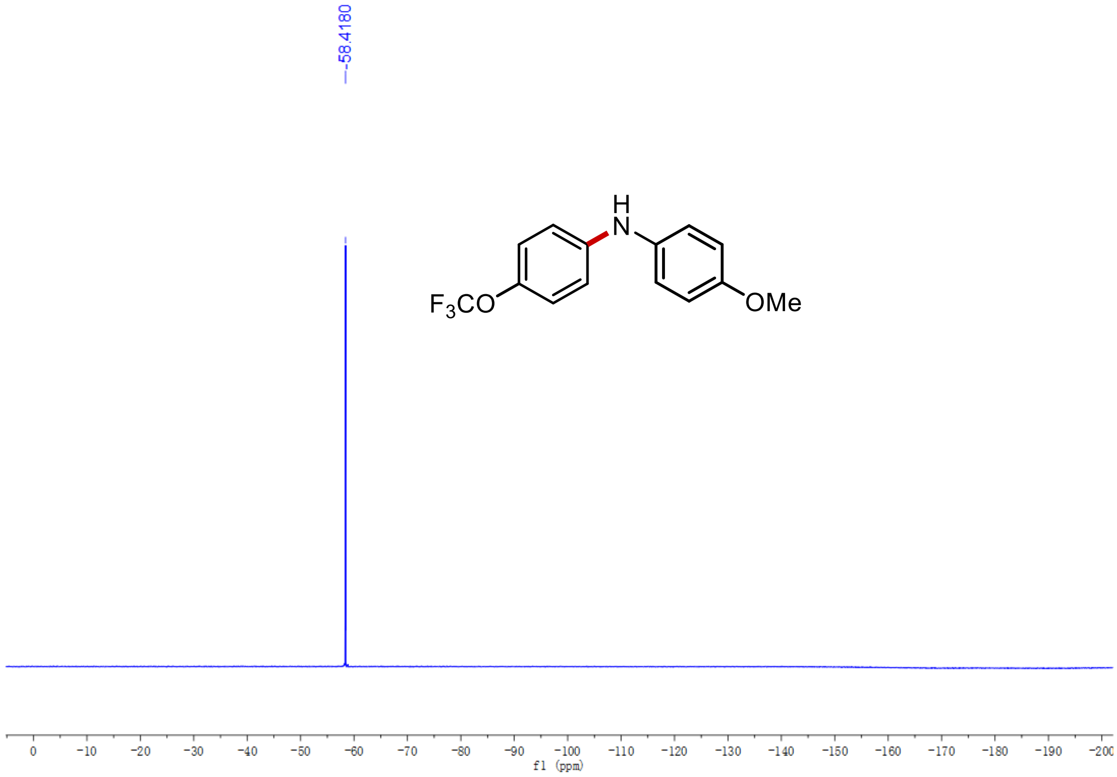

^{19}F NMR (376 MHz, $CDCl_3$) Spectrum

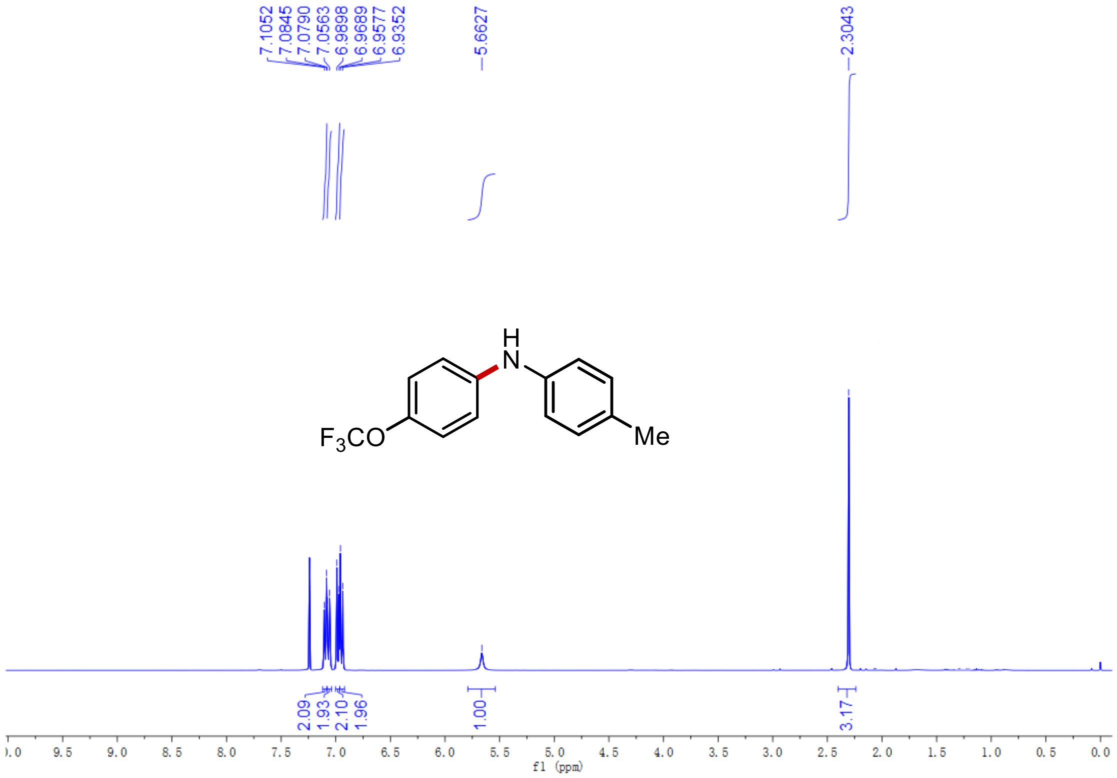

^{1}H NMR (400 MHz, $CDCl_3$) Spectrum

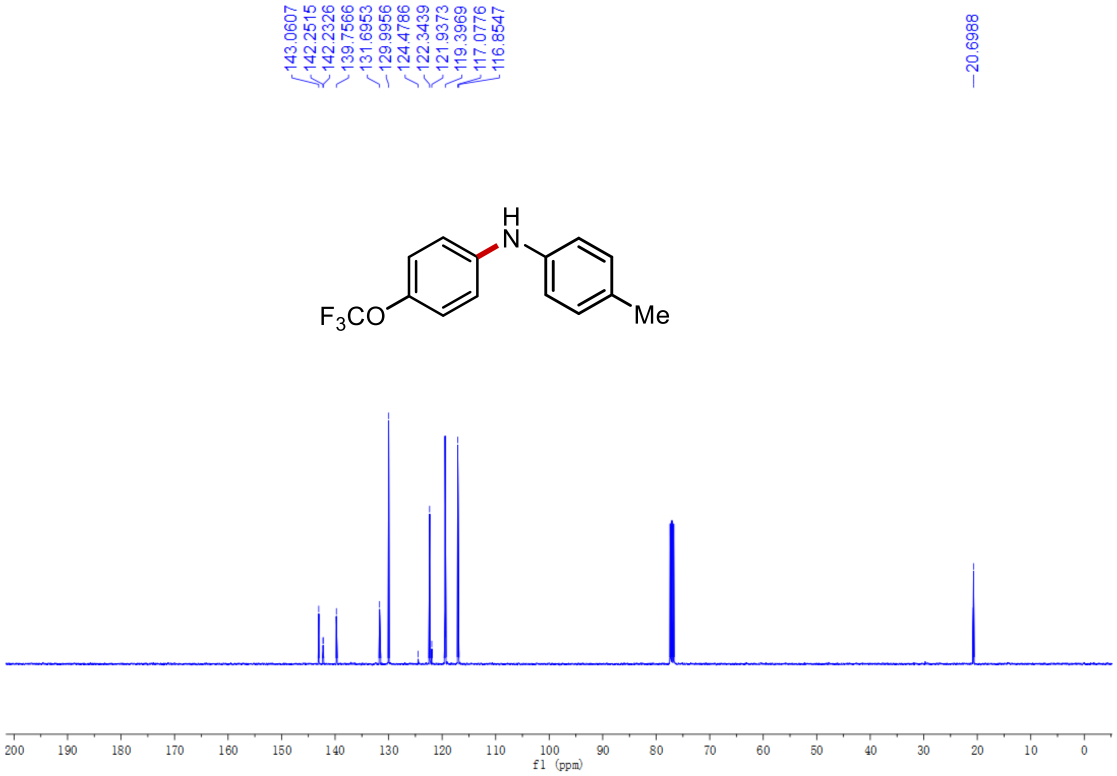

^{13}C NMR (100 MHz, $CDCl_3$) Spectrum

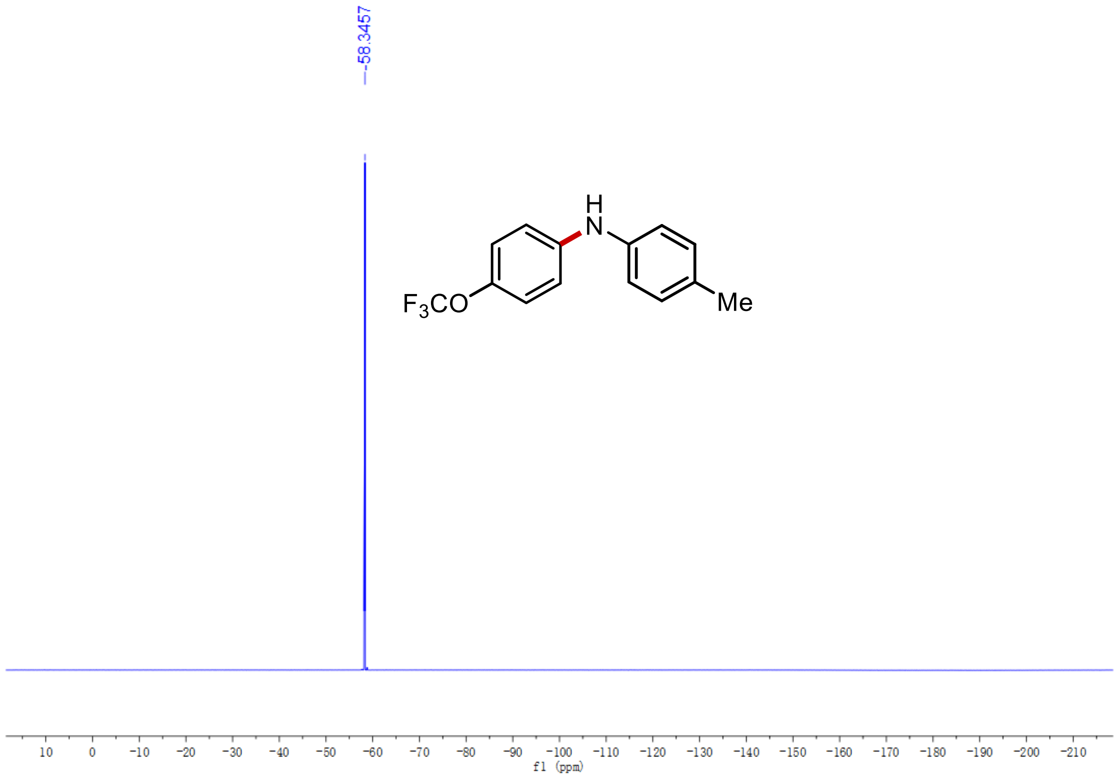

^{19}F NMR (376 MHz, $CDCl_3$) Spectrum

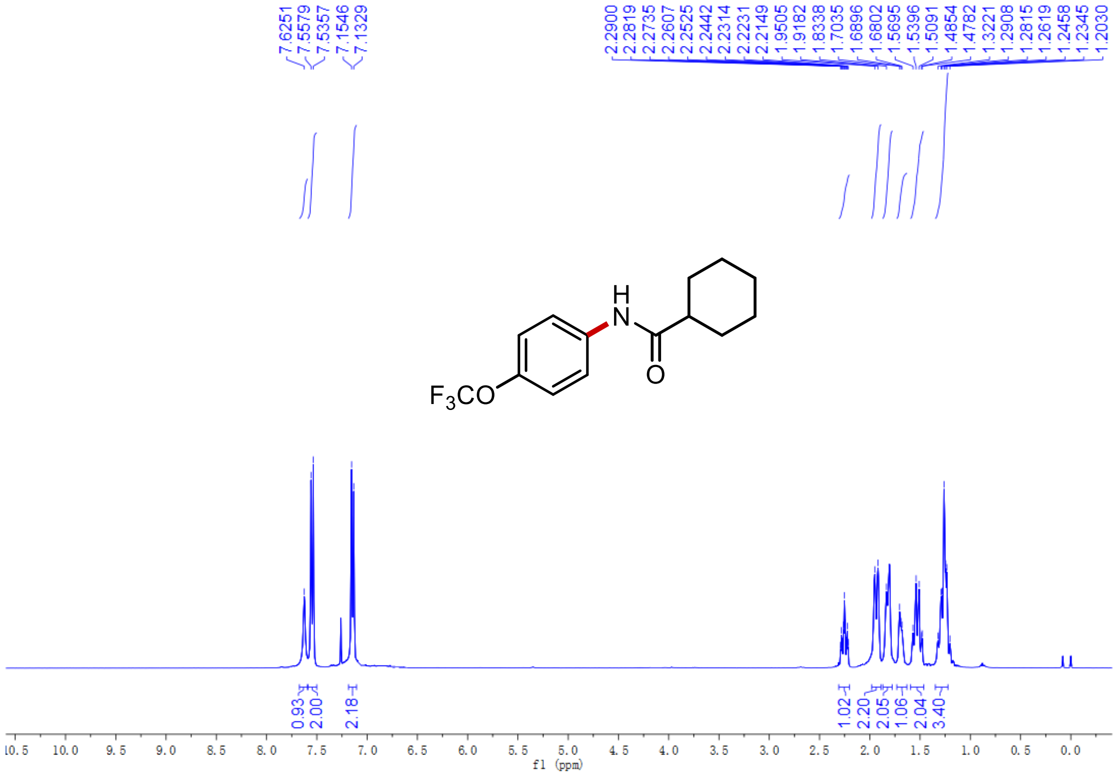

^{1}H NMR (400 MHz, $CDCl_3$) Spectrum

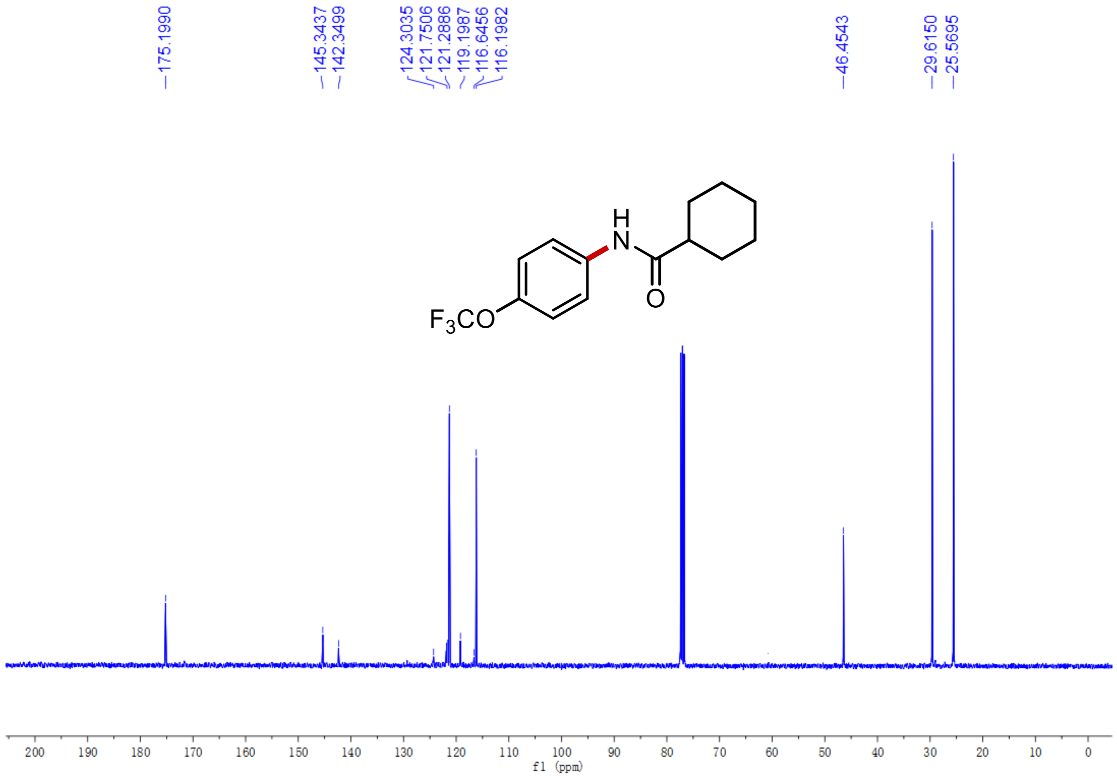

^{13}C NMR (100 MHz, $CDCl_3$) Spectrum

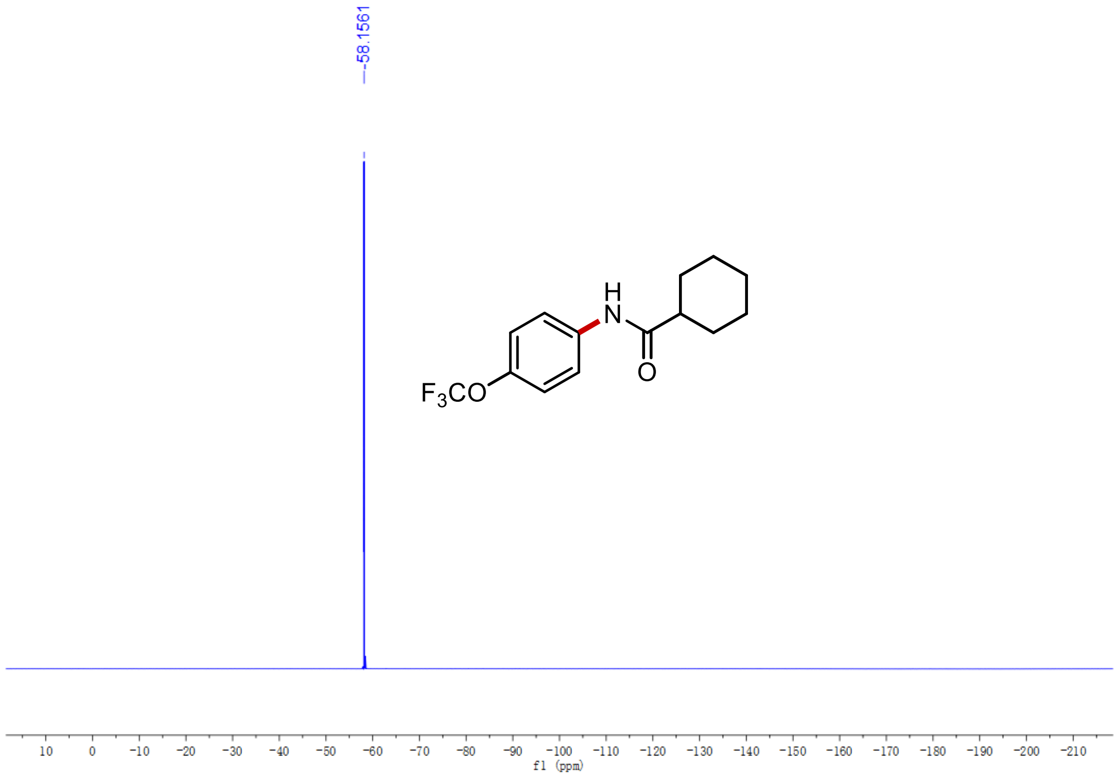

^{19}F NMR (376 MHz, $CDCl_3$) Spectrum

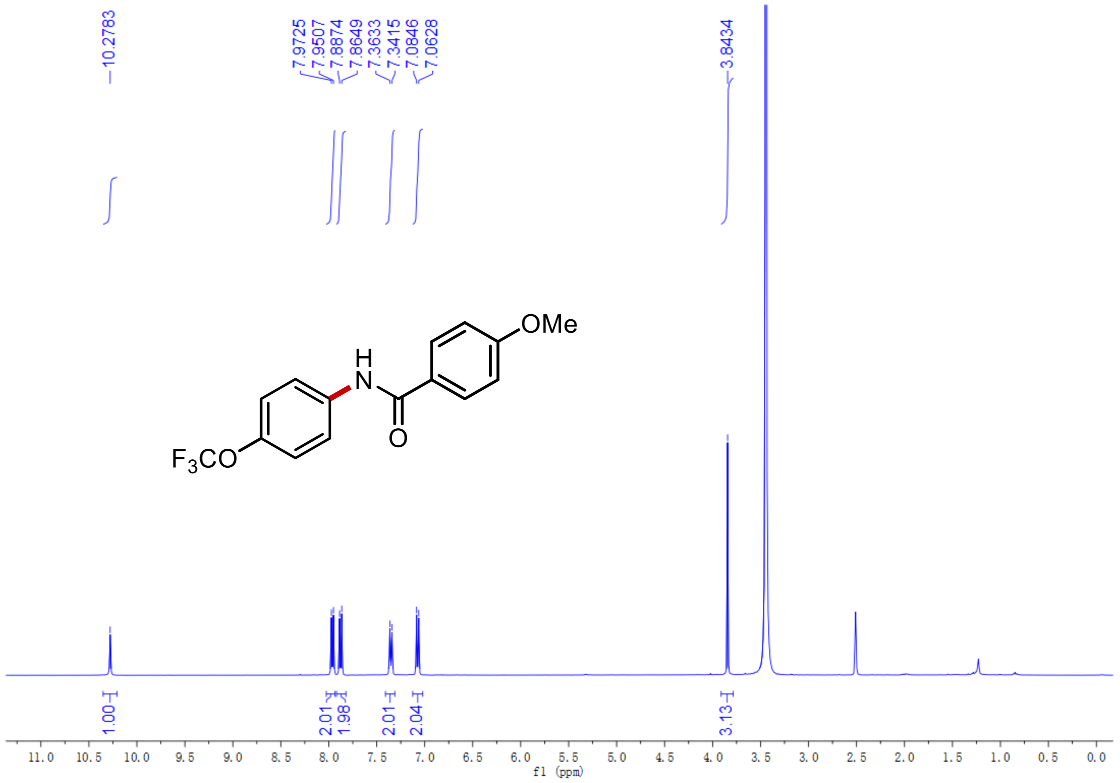

^{1}H NMR (400 MHz, $CDCl_3$) Spectrum

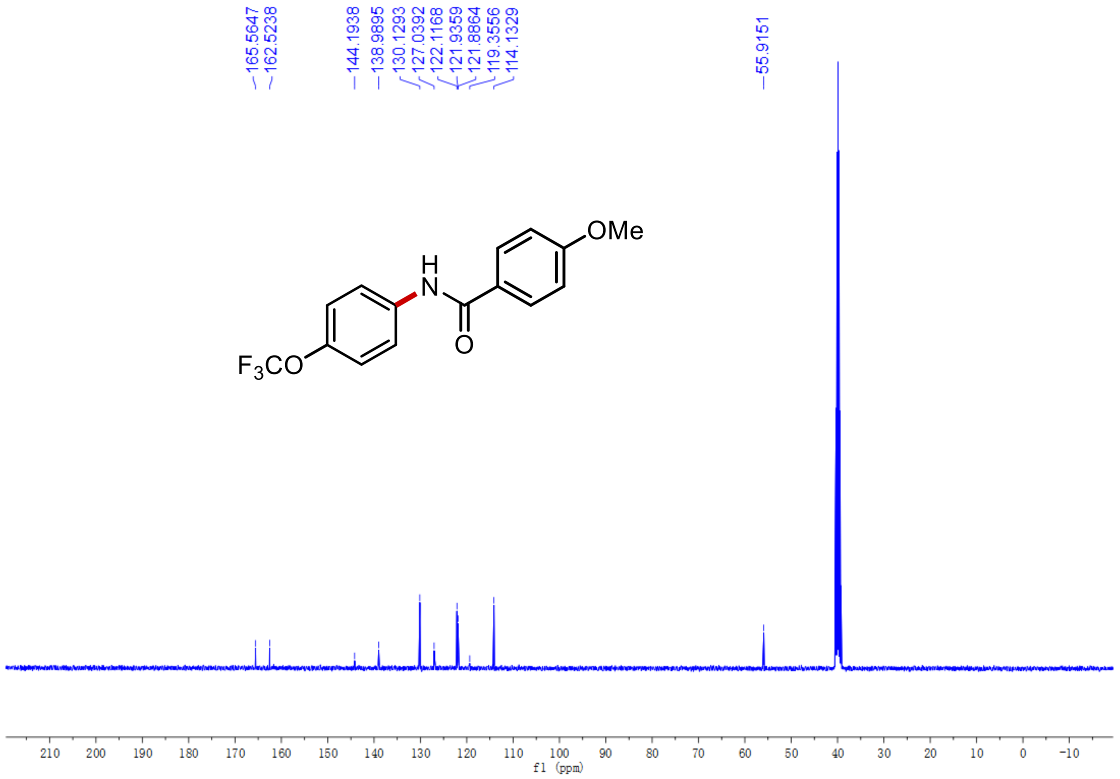

^{13}C NMR (100 MHz, $CDCl_3$) Spectrum

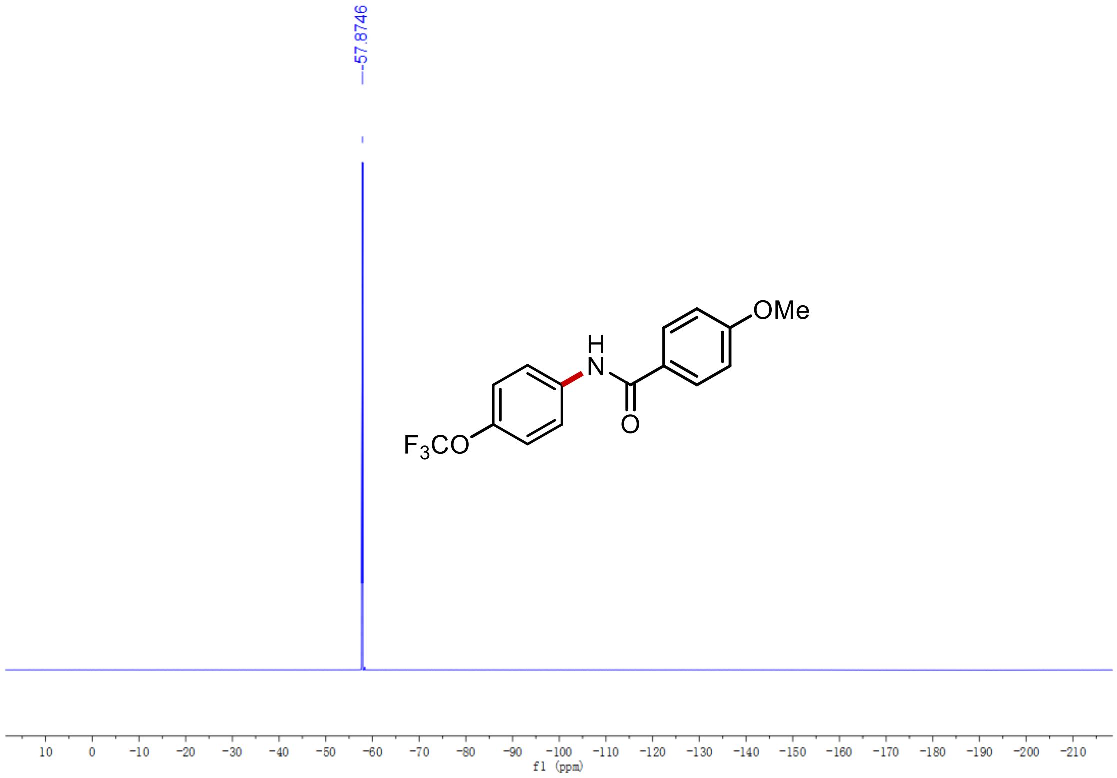

^{19}**F NMR** (376 MHz, $CDCl_3$) Spectrum

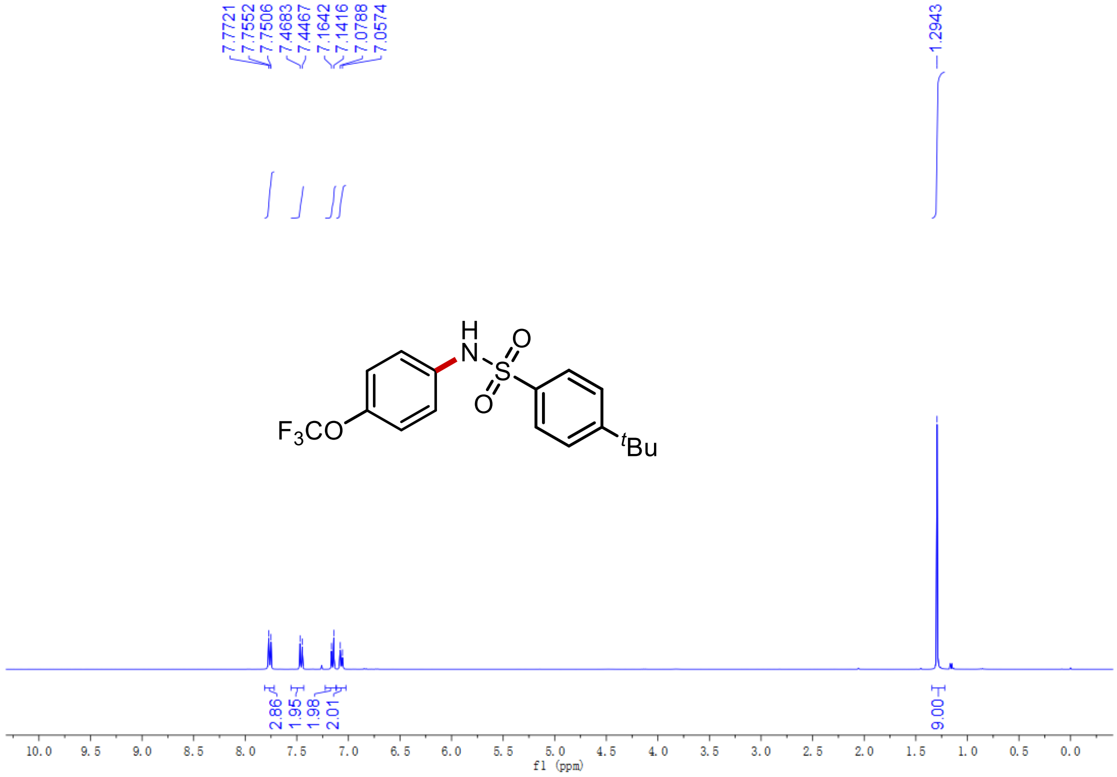

^{1}**H NMR** (400 MHz, $CDCl_3$) Spectrum

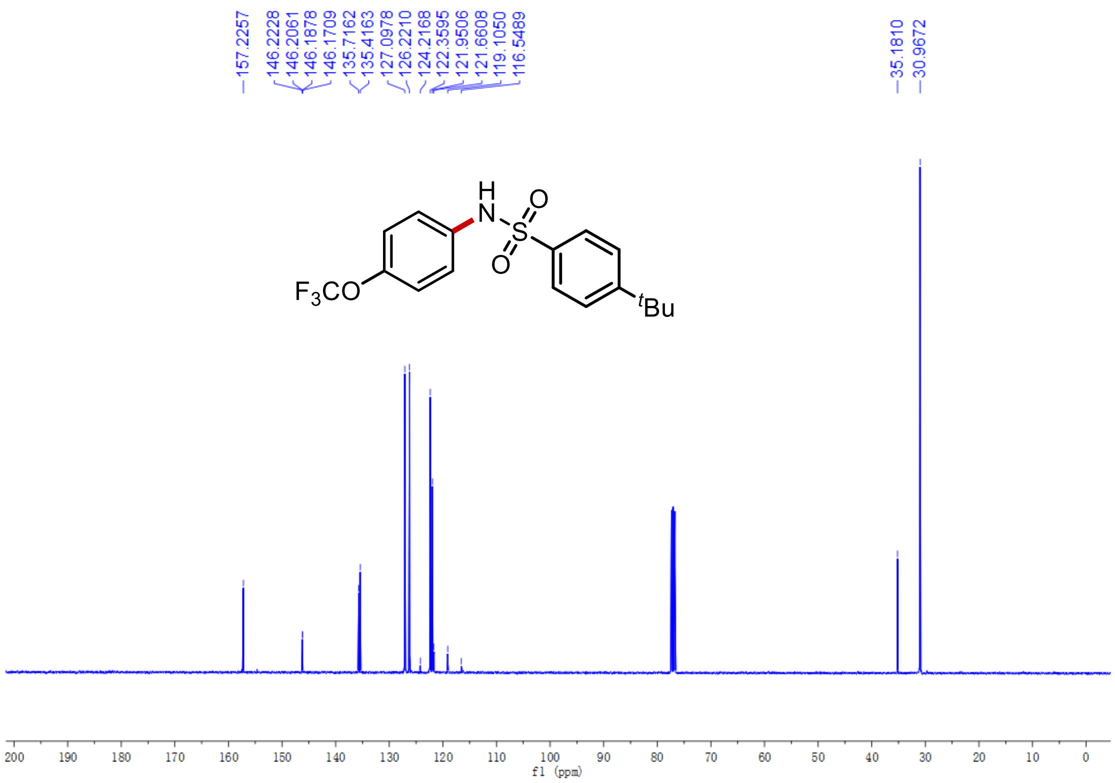

^{13}**C NMR** (100 MHz, $CDCl_3$) Spectrum

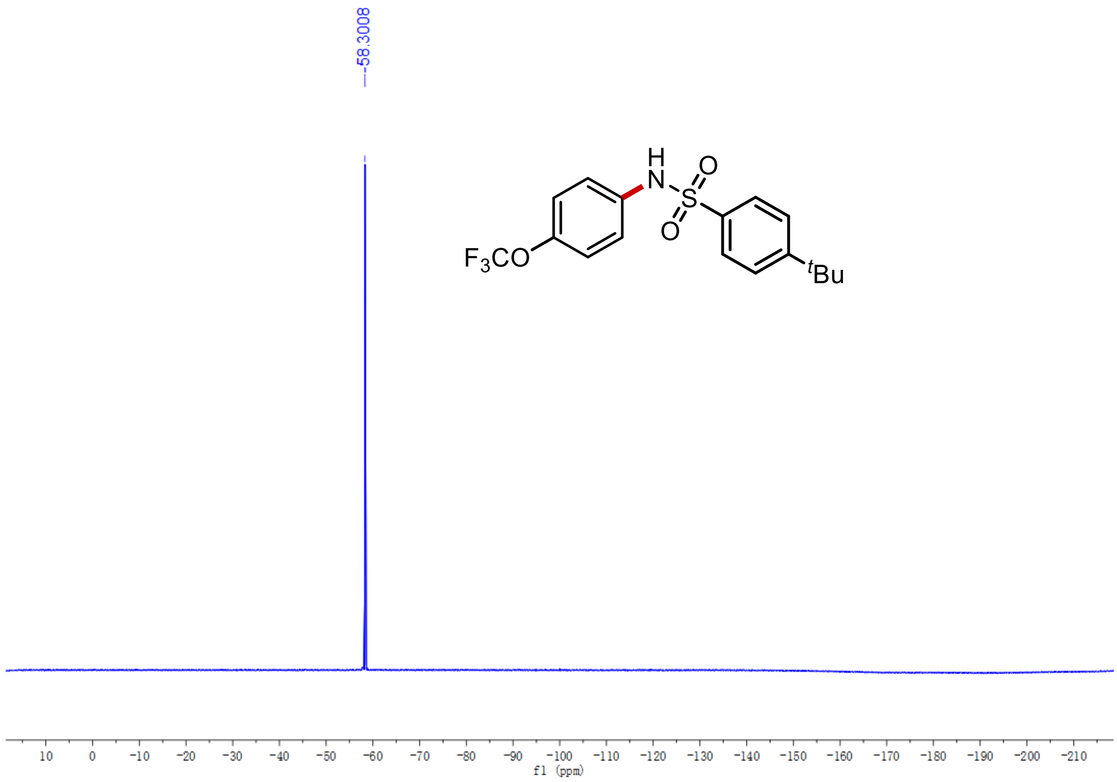

^{19}**F NMR** (376 MHz, $CDCl_3$) Spectrum

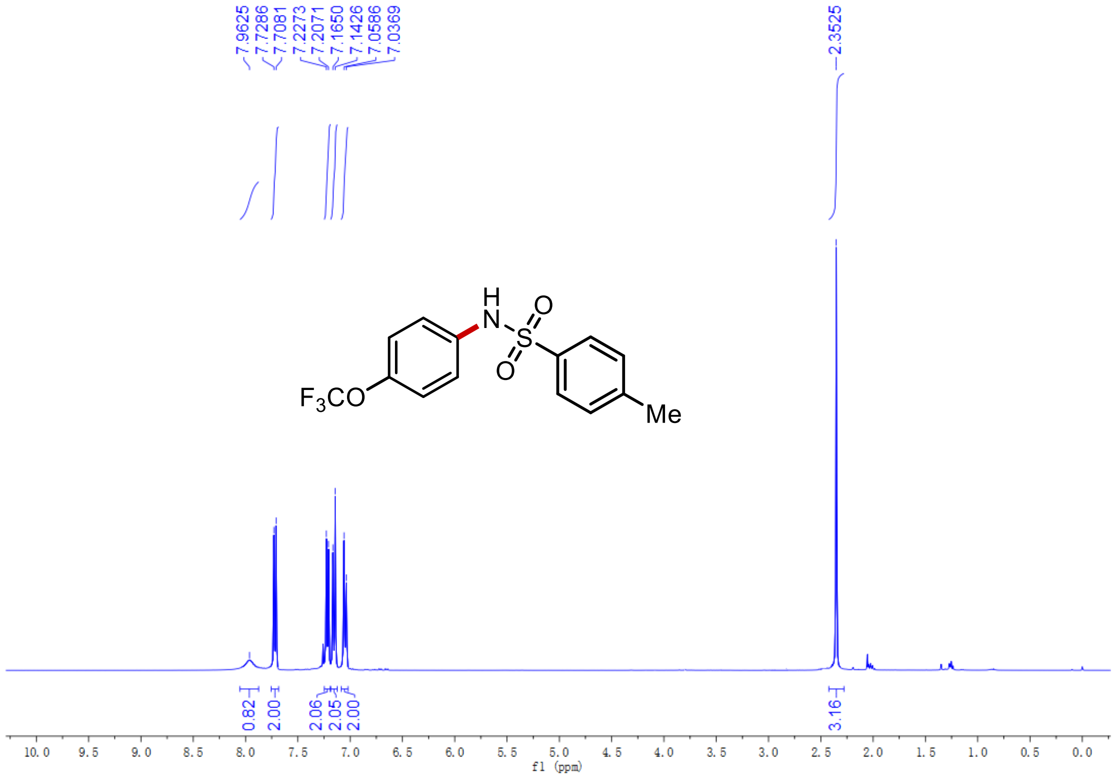

^{1}H NMR (400 MHz, $CDCl_3$) Spectrum

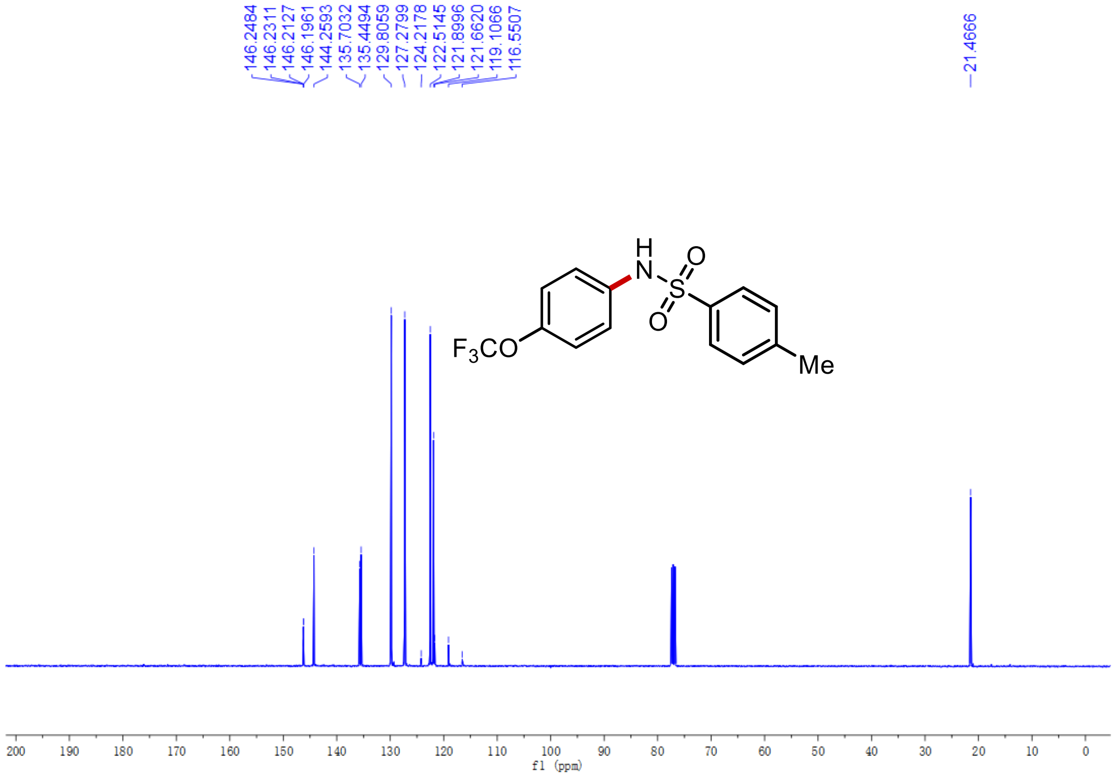

^{13}C NMR (100 MHz, $CDCl_3$) Spectrum

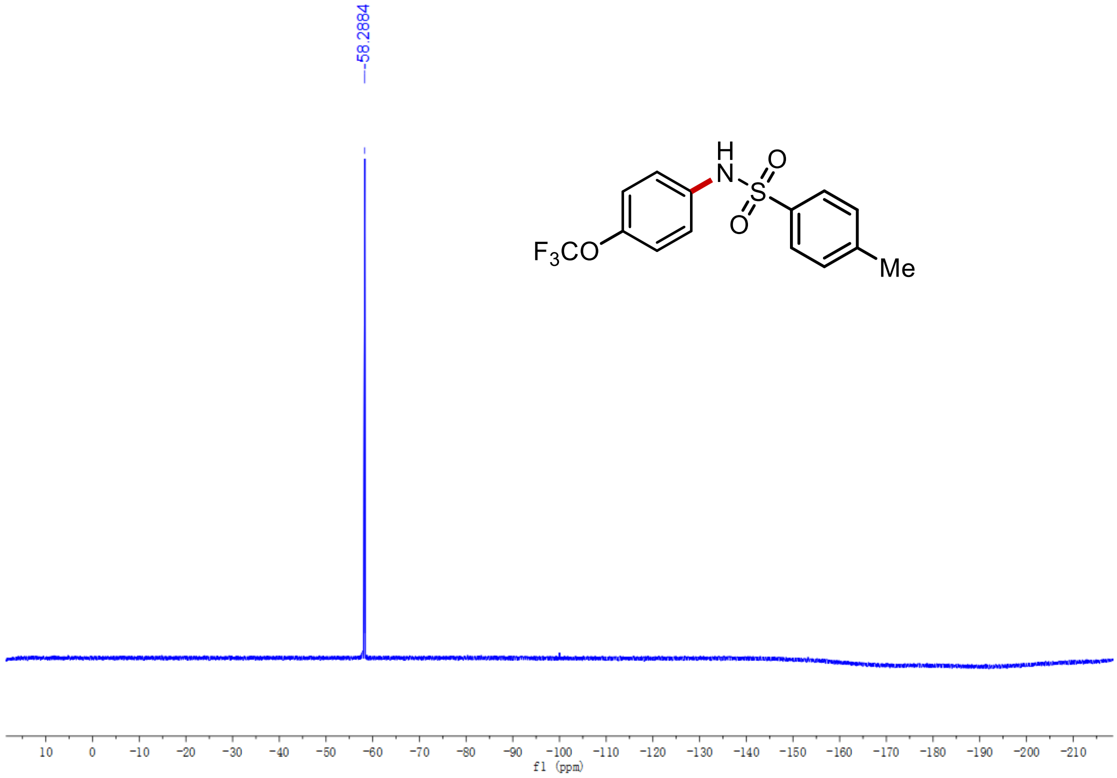

19F NMR (376 MHz, $CDCl_3$) Spectrum

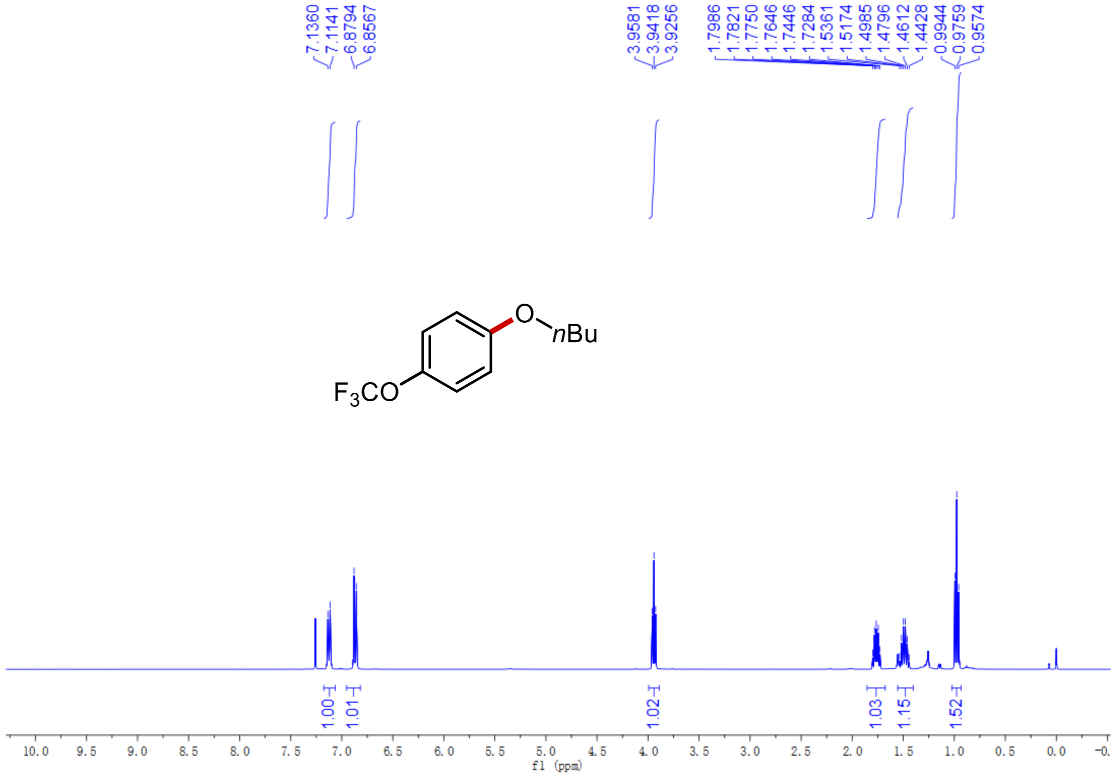

1H NMR (400 MHz, $CDCl_3$) Spectrum

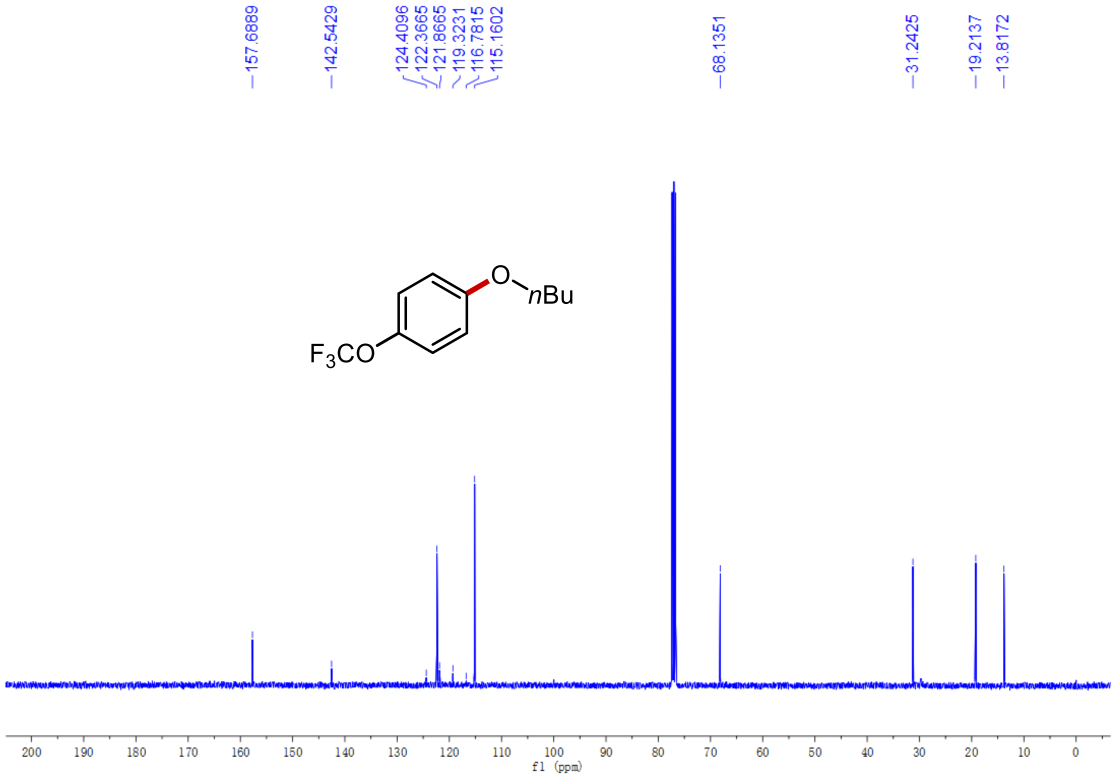

13C NMR (100 MHz, $CDCl_3$) Spectrum

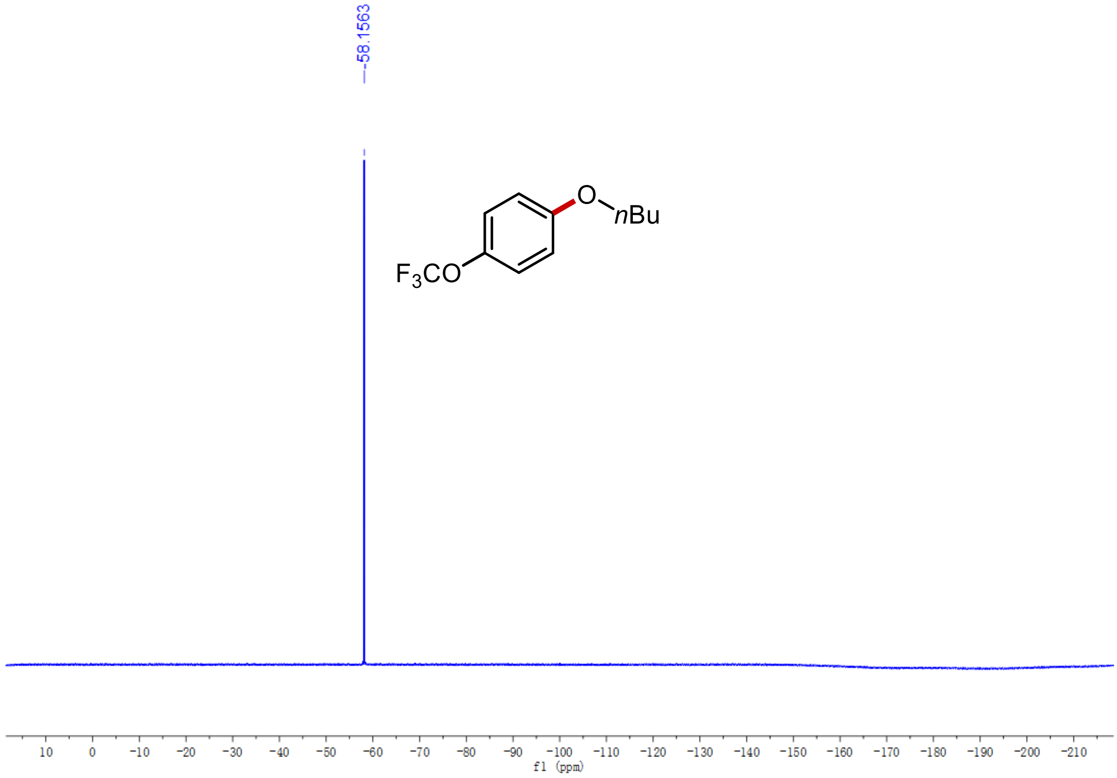

19F NMR (376 MHz, $CDCl_3$) Spectrum

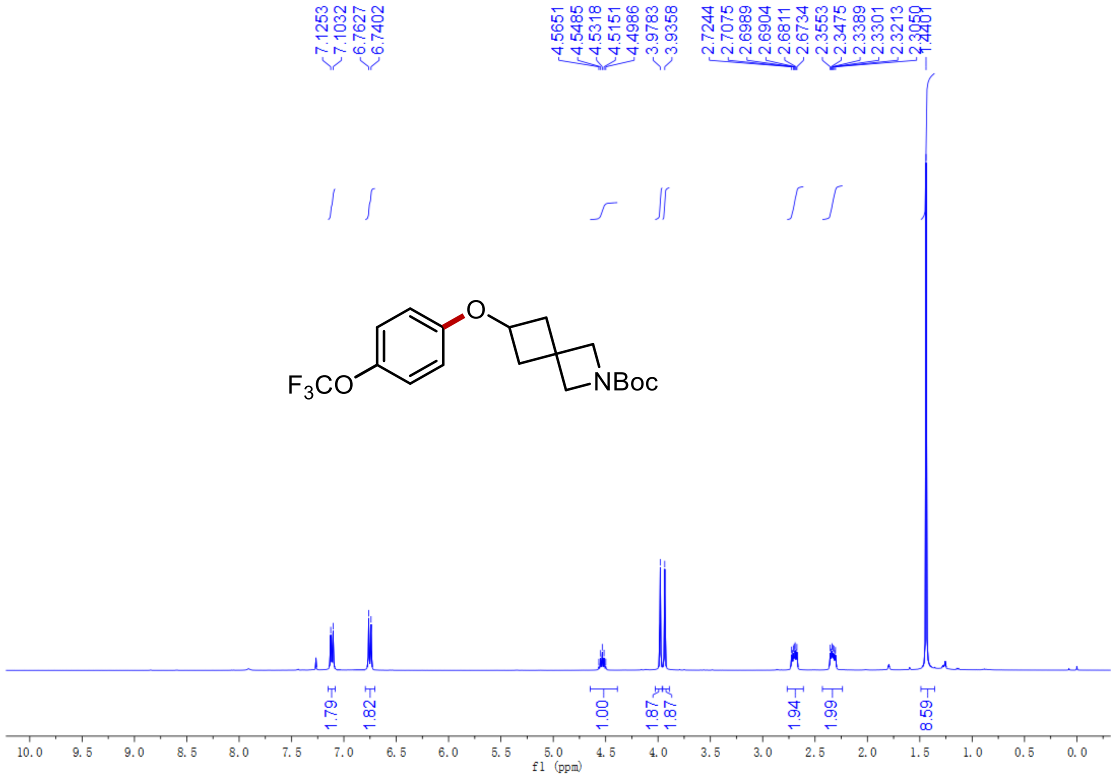

^{1}**H NMR** (400 MHz, $CDCl_3$) Spectrum

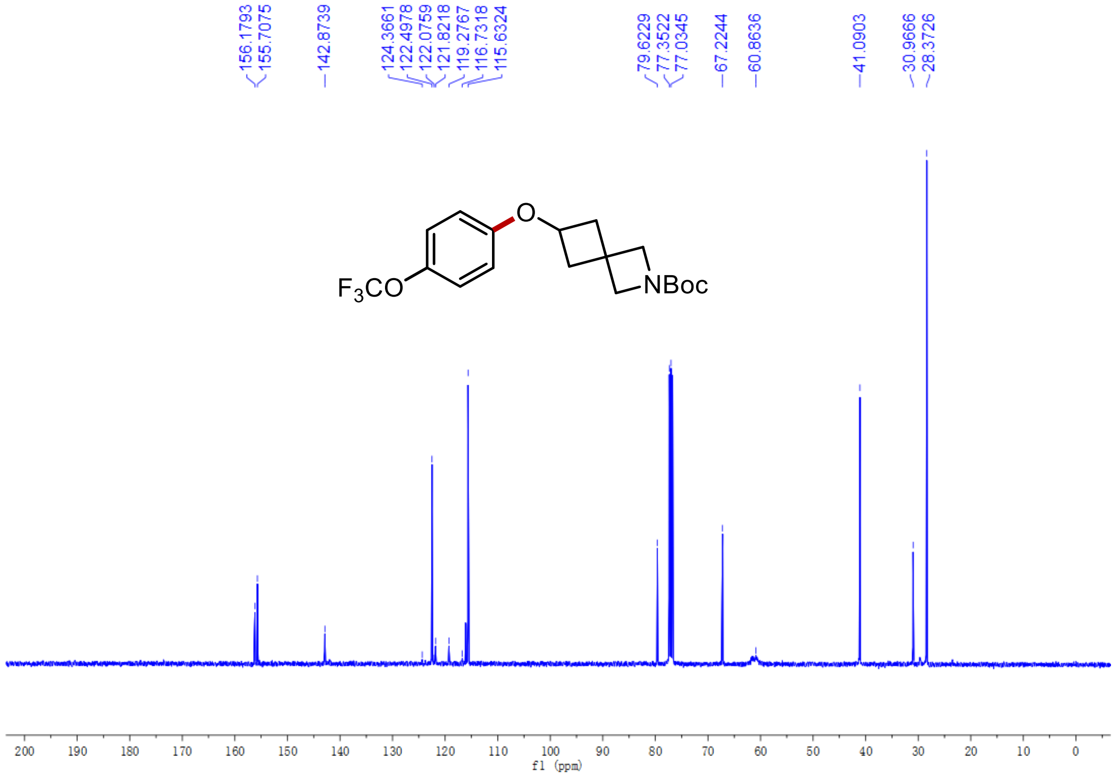

^{13}**C NMR** (100 MHz, $CDCl_3$) Spectrum

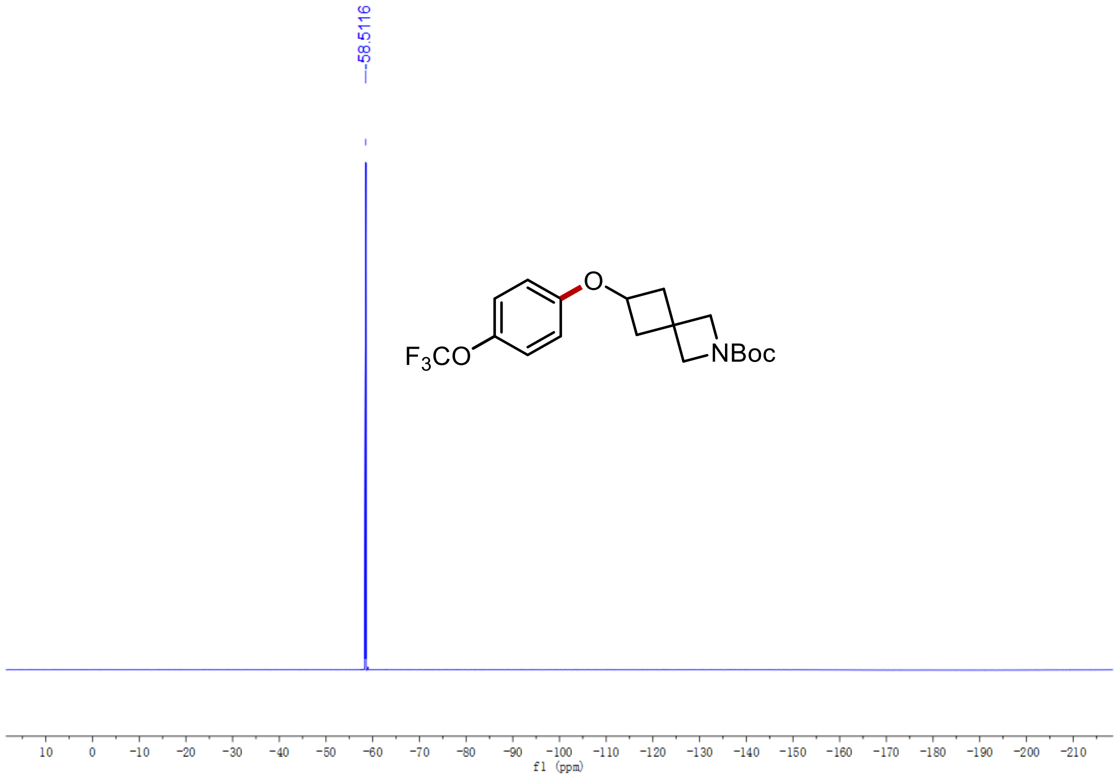

^{19}F NMR (376 MHz, $CDCl_3$) Spectrum

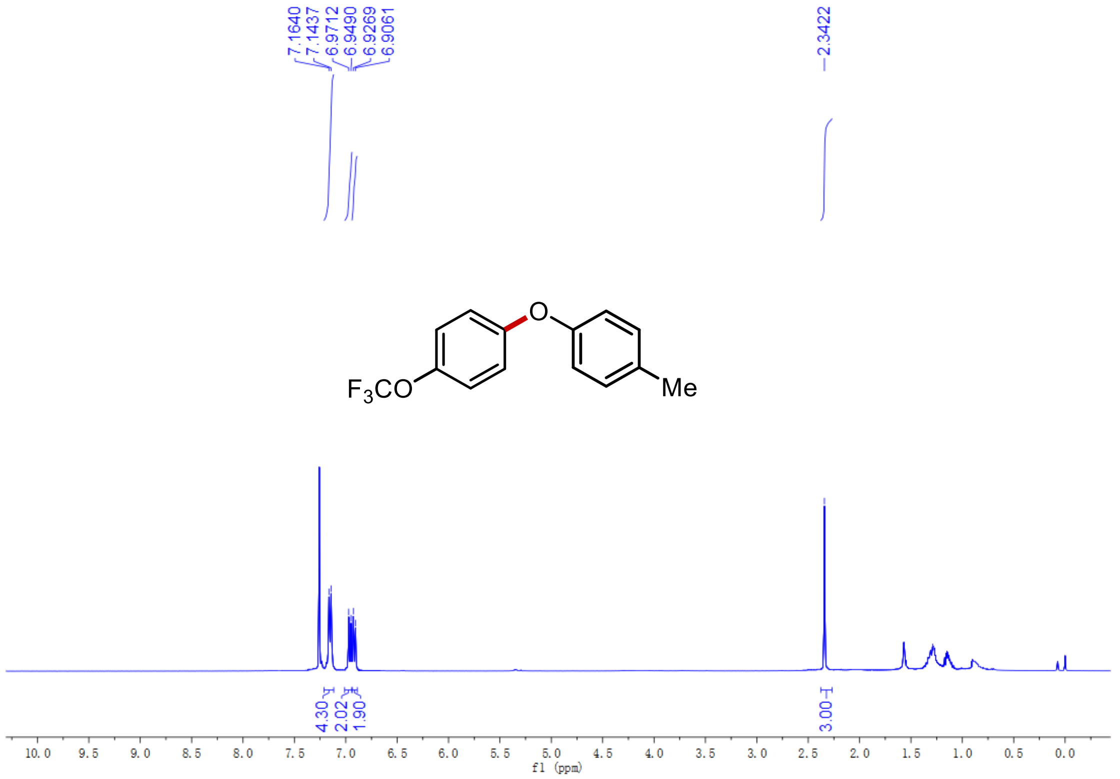

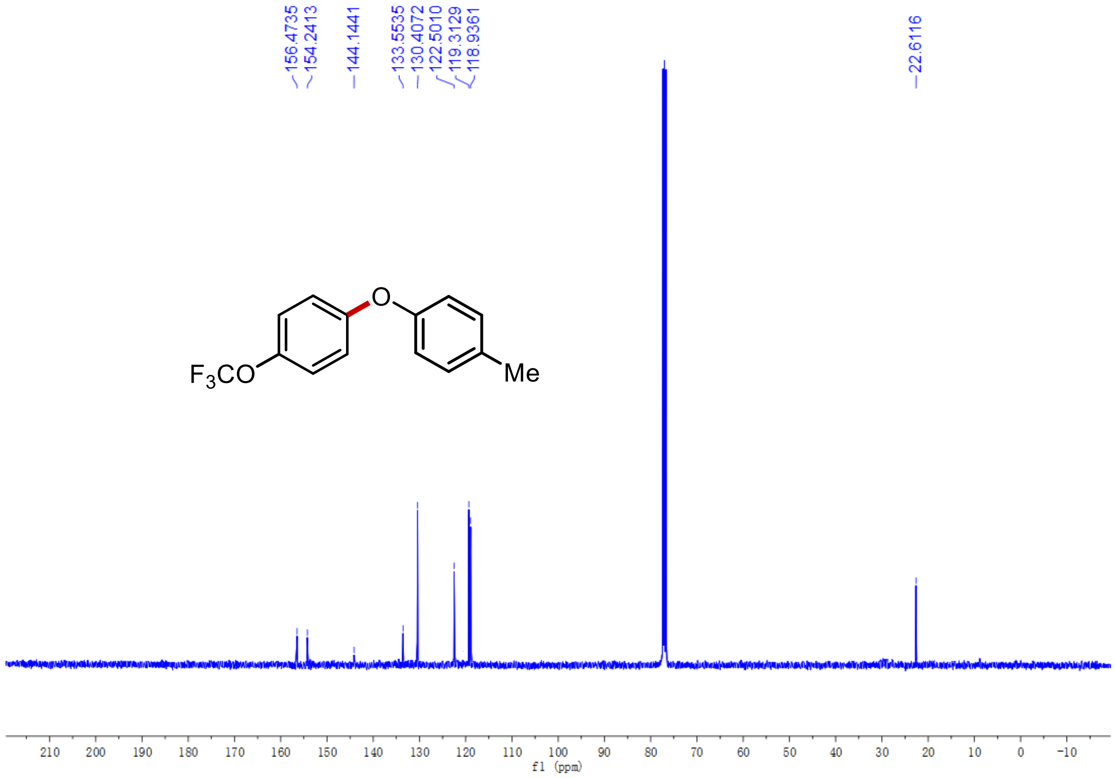

^{13}C NMR (100 MHz, $CDCl_3$) Spectrum

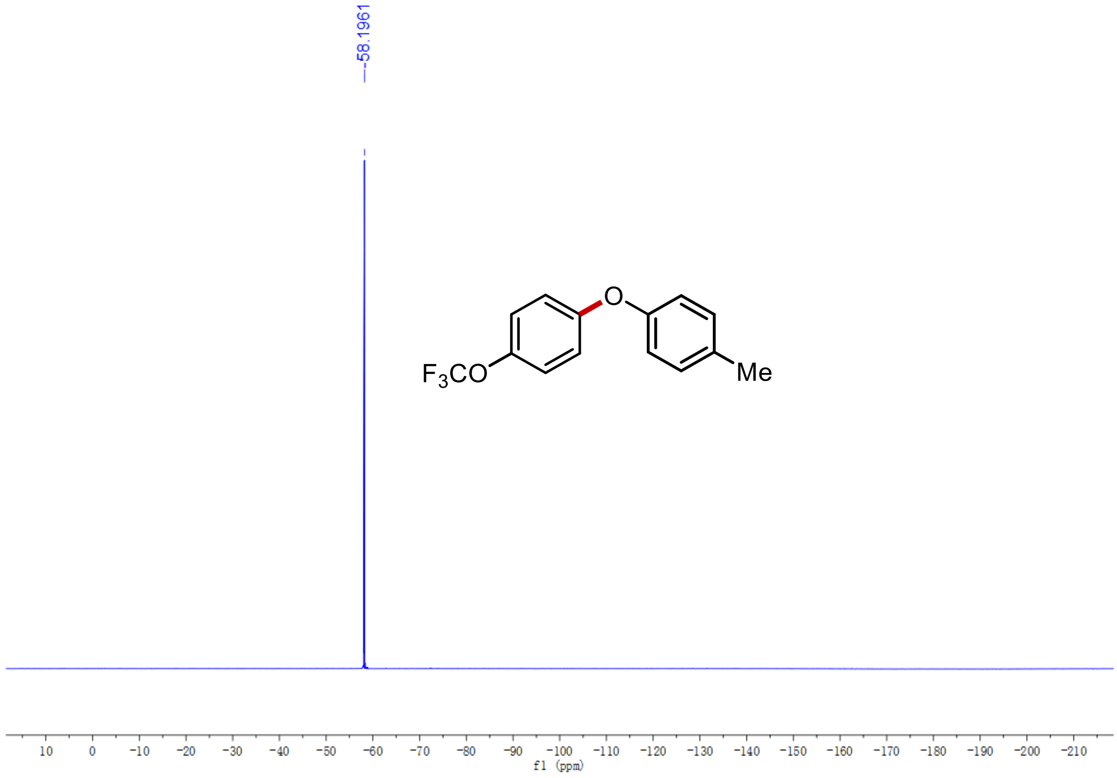

^{19}F NMR (376 MHz, $CDCl_3$) Spectrum

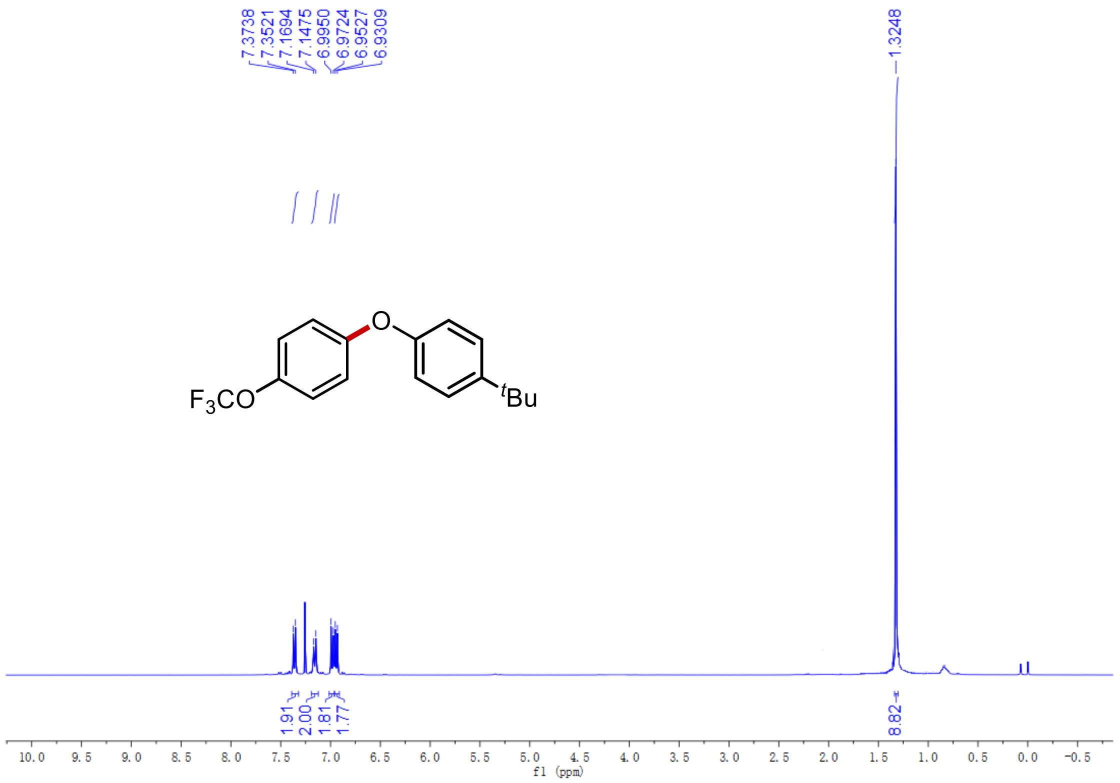

^{1}H NMR (400 MHz, $CDCl_3$) Spectrum

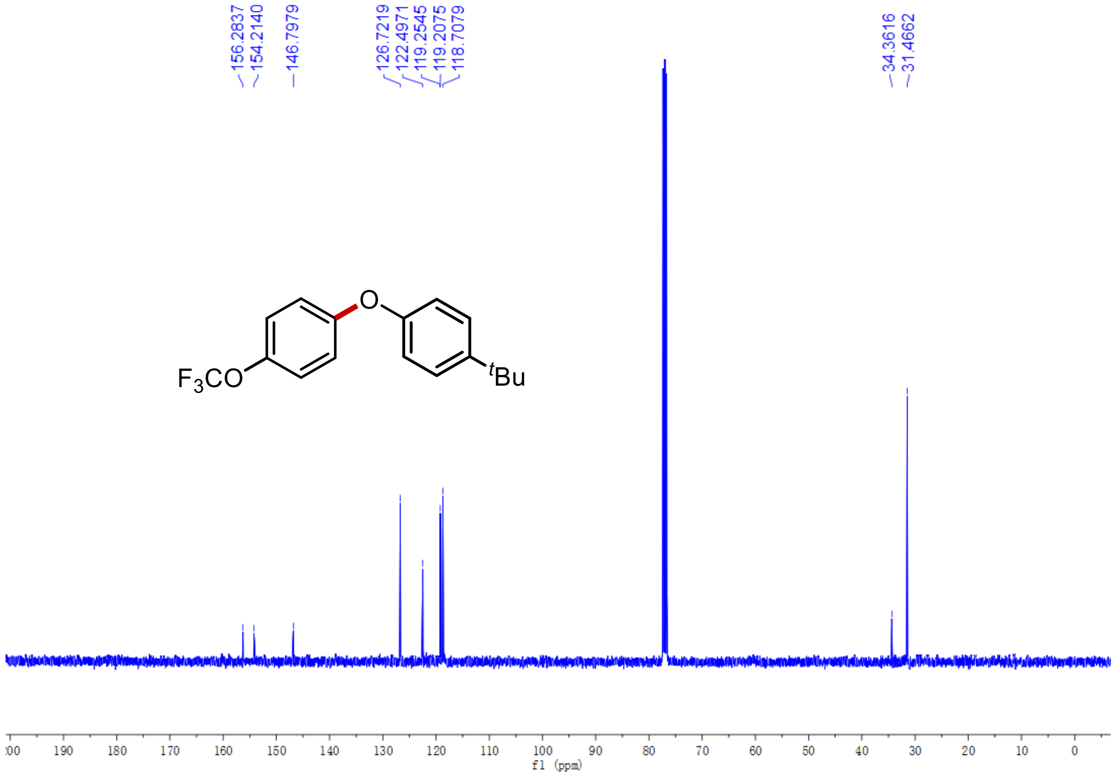

^{13}C NMR (100 MHz, $CDCl_3$) Spectrum

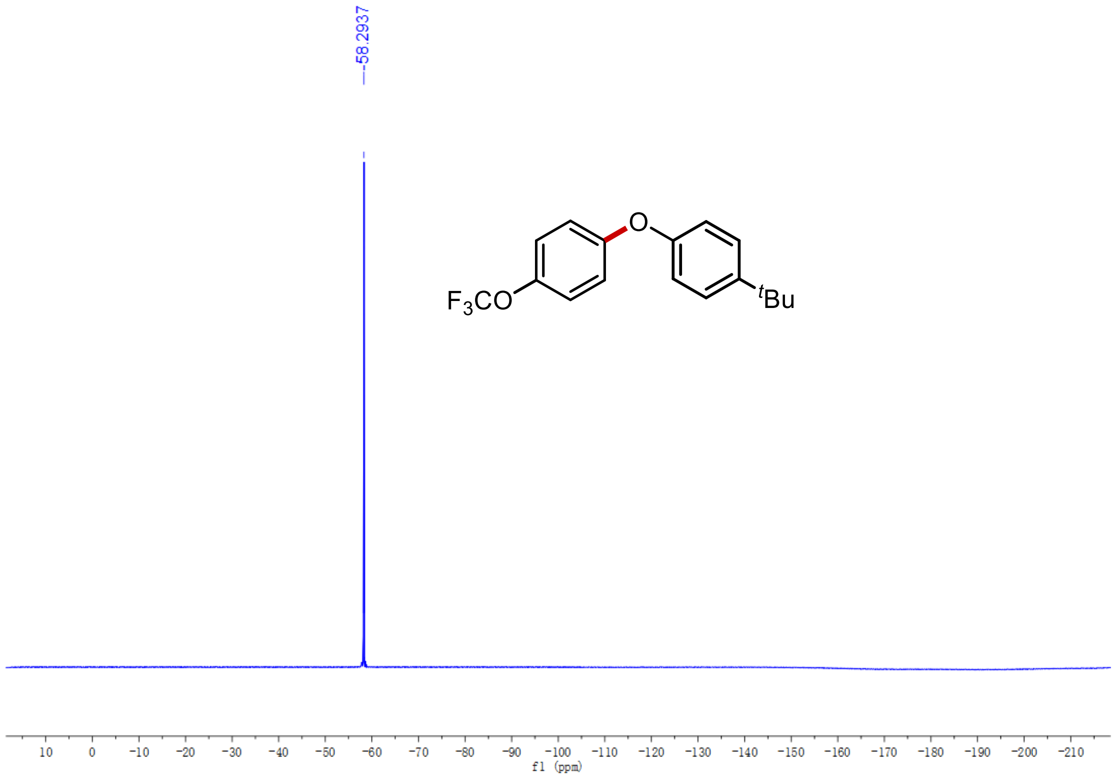

^{19}F NMR (376 MHz, $CDCl_3$) Spectrum

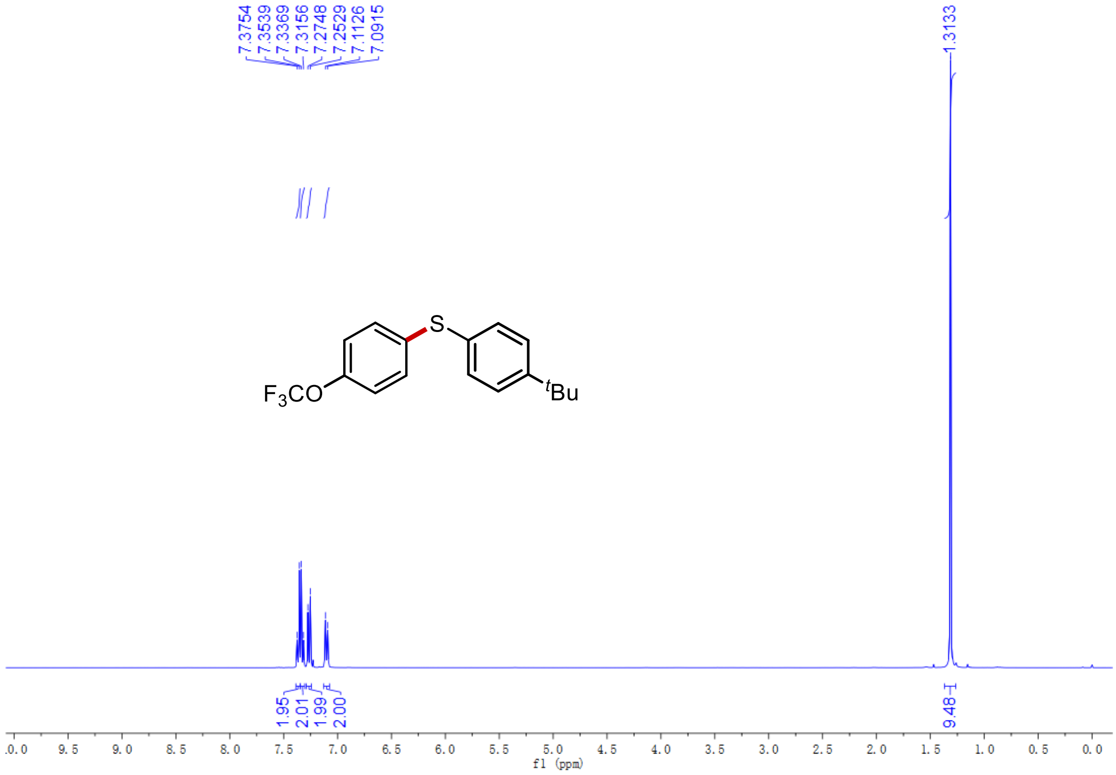

^{1}H NMR (400 MHz, $CDCl_3$) Spectrum

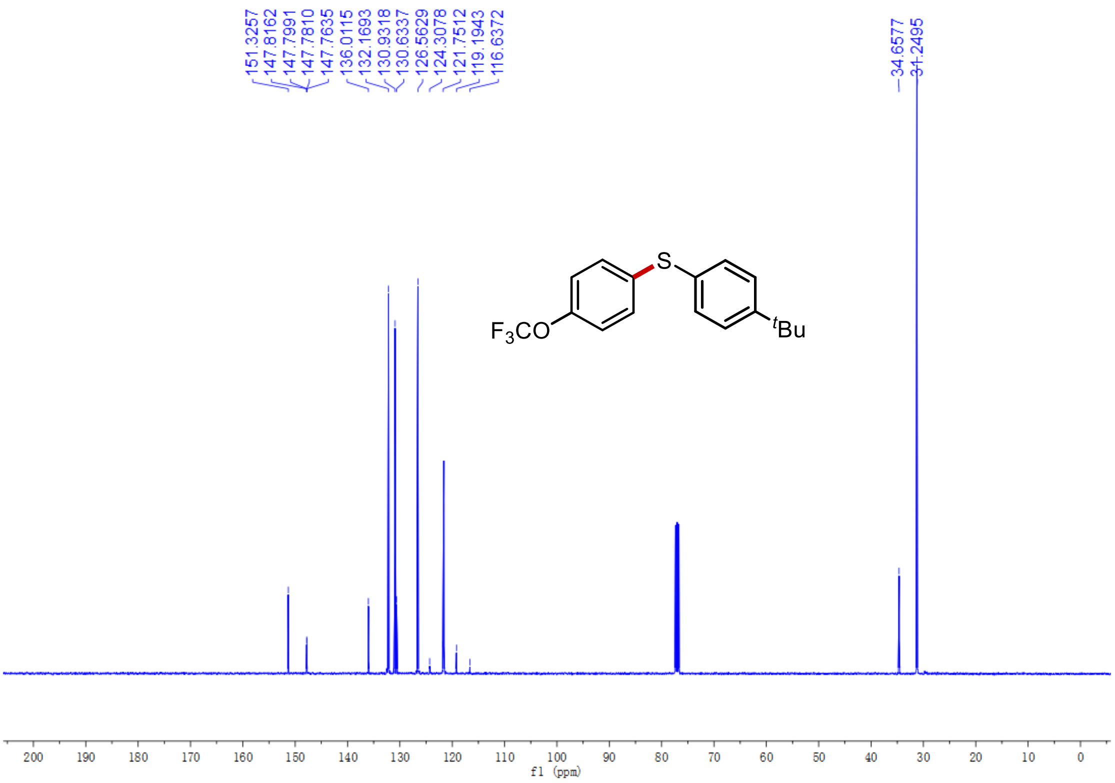

13C NMR (100 MHz, CDCl3) Spectrum

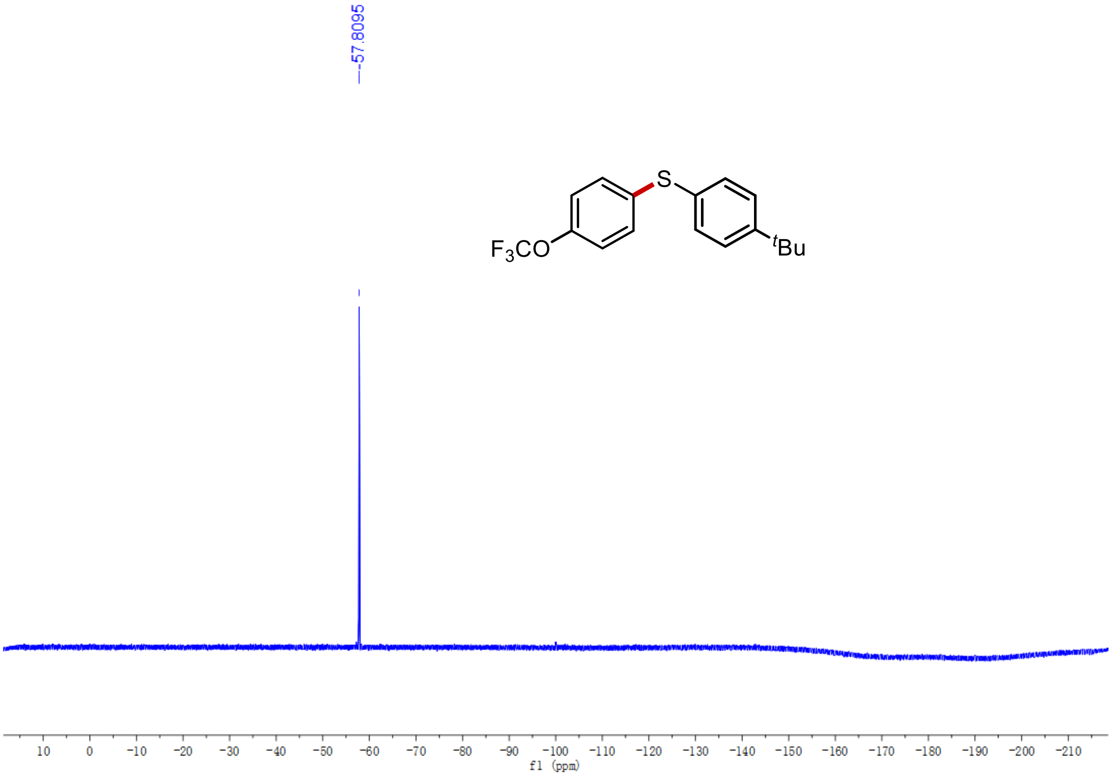

19F NMR (376 MHz, CDCl3) Spectrum

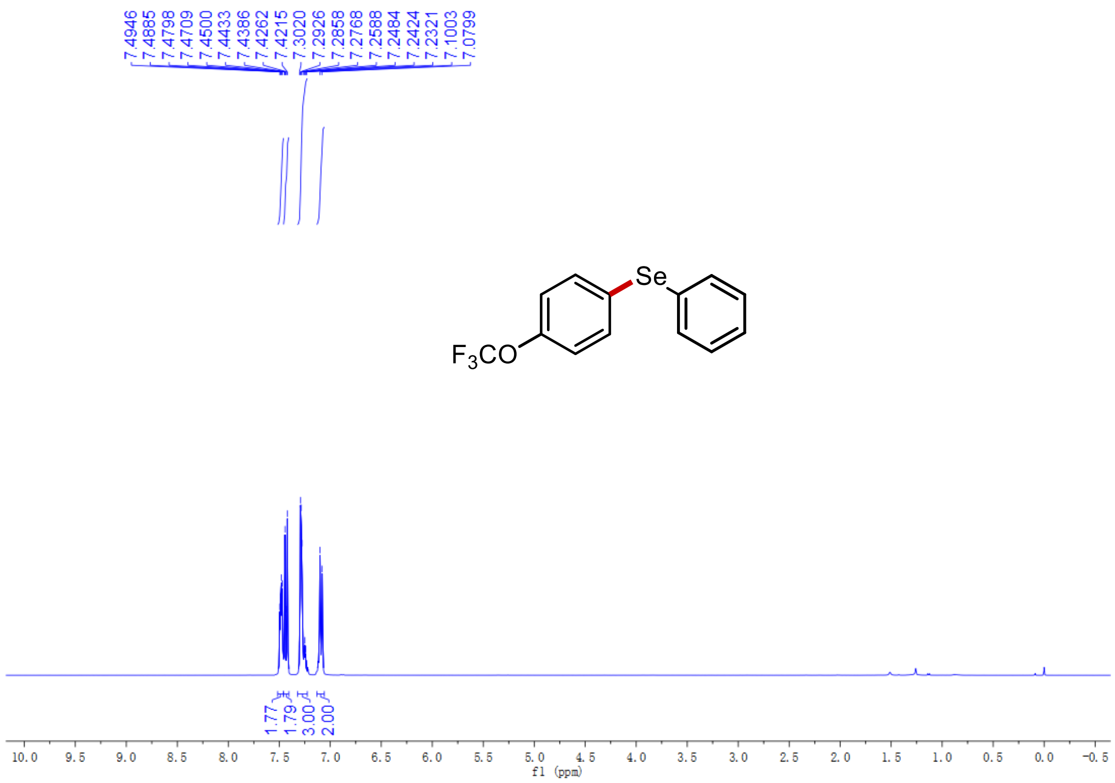

^{1}H NMR (400 MHz, $CDCl_3$) Spectrum

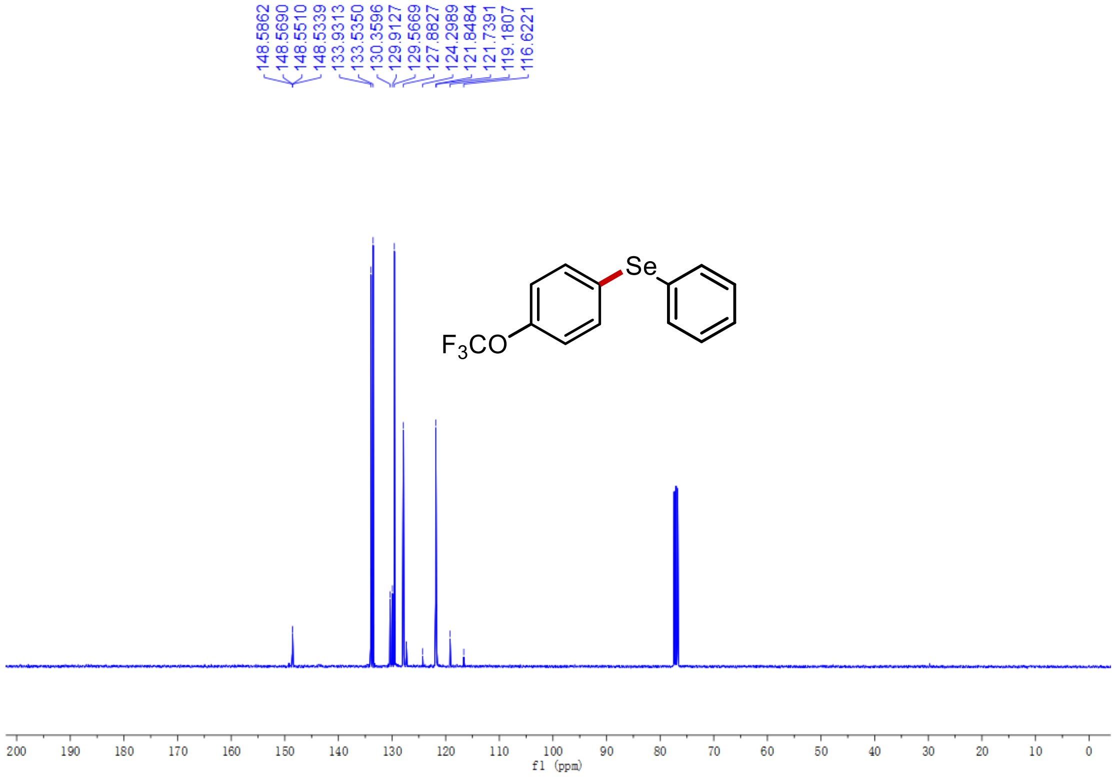

^{13}C NMR (100 MHz, $CDCl_3$) Spectrum

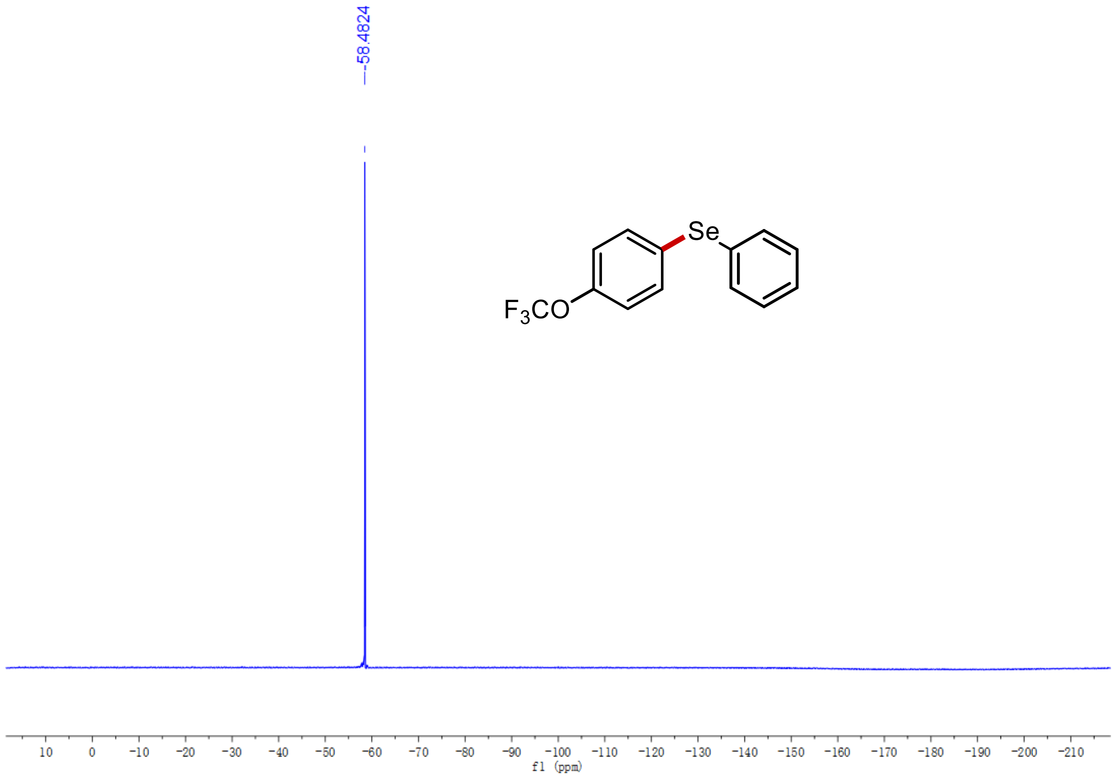

^{19}F NMR (376 MHz, CDCl3) Spectrum

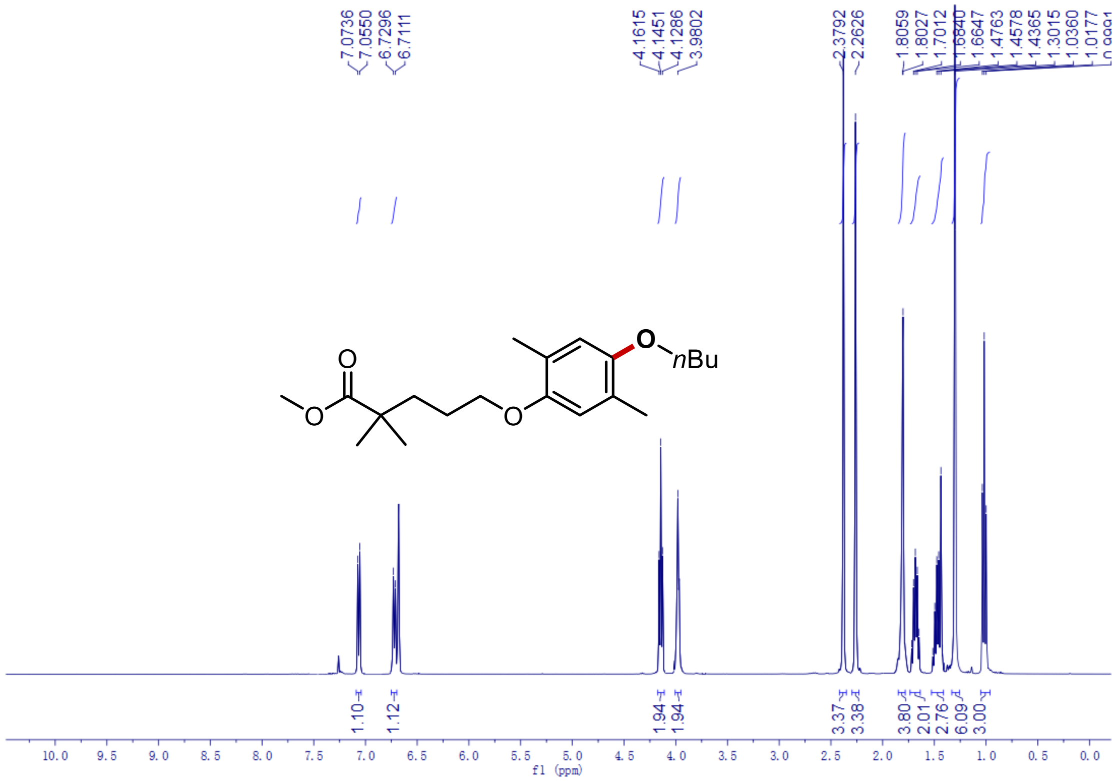

^{1}H NMR (400 MHz, CDCl3) Spectrum

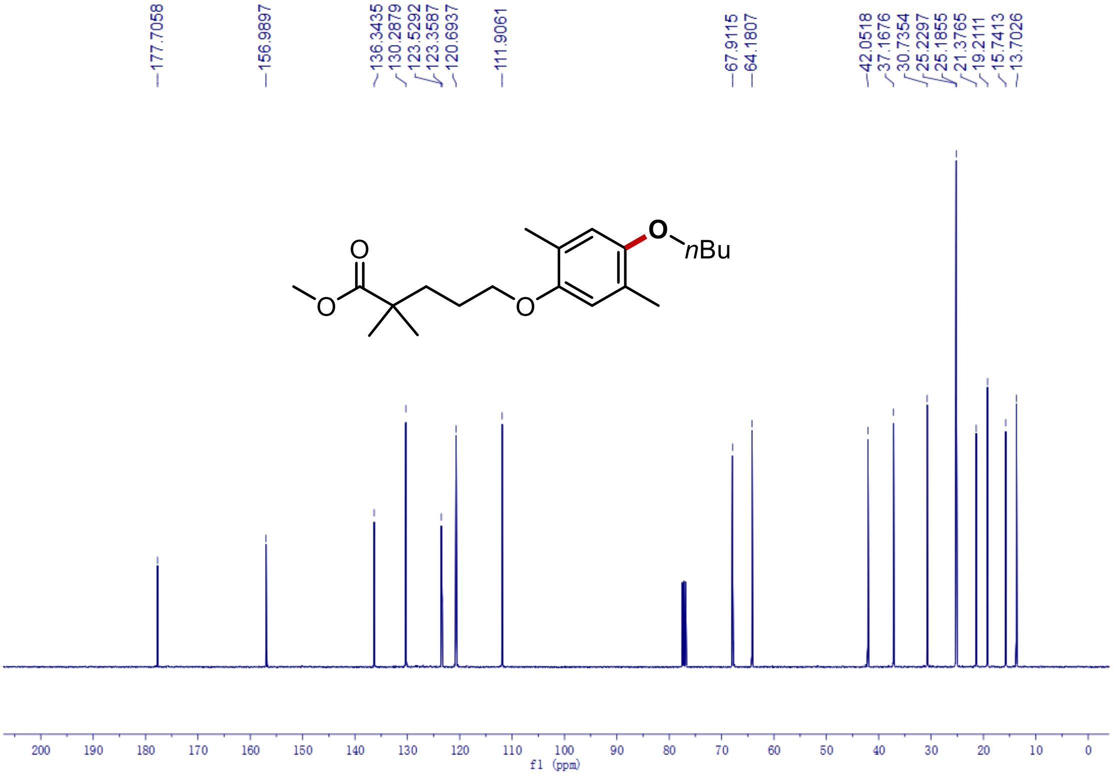

^{13}C NMR (100 MHz, $CDCl_3$) Spectrum

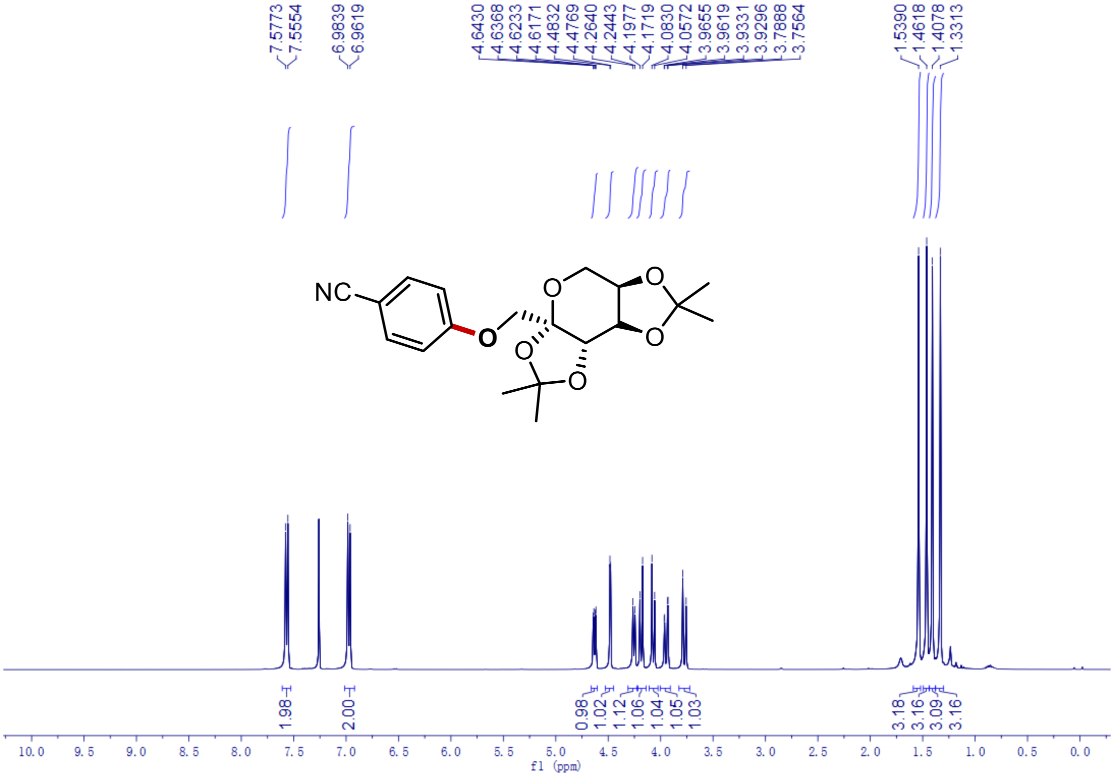

^{1}H NMR (400 MHz, $CDCl_3$) Spectrum

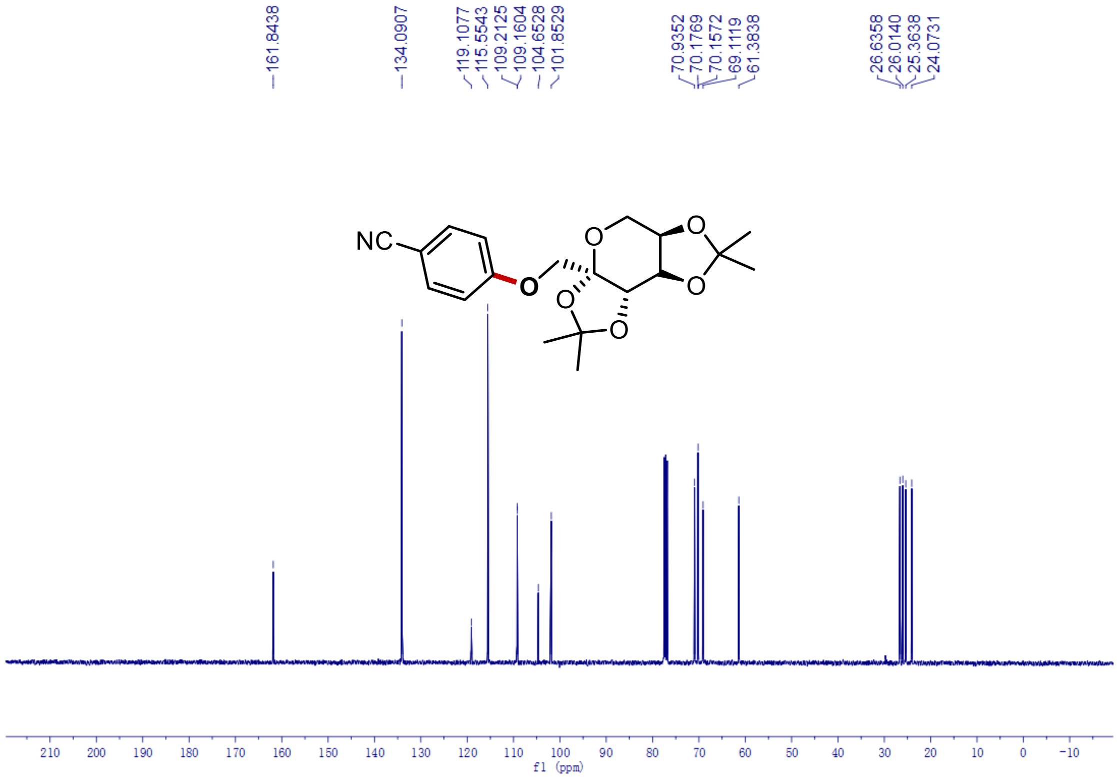

^{13}**C NMR** (100 MHz, $CDCl_3$) Spectrum

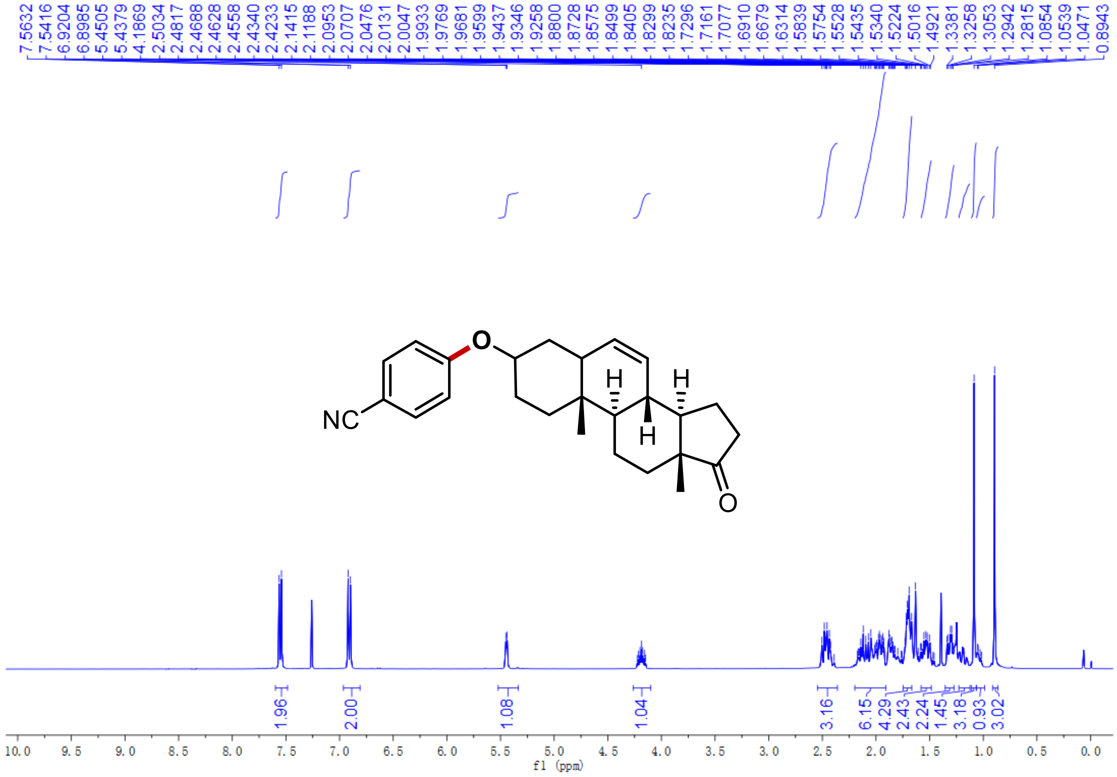

^{1}**H NMR** (400 MHz, $CDCl_3$) Spectrum

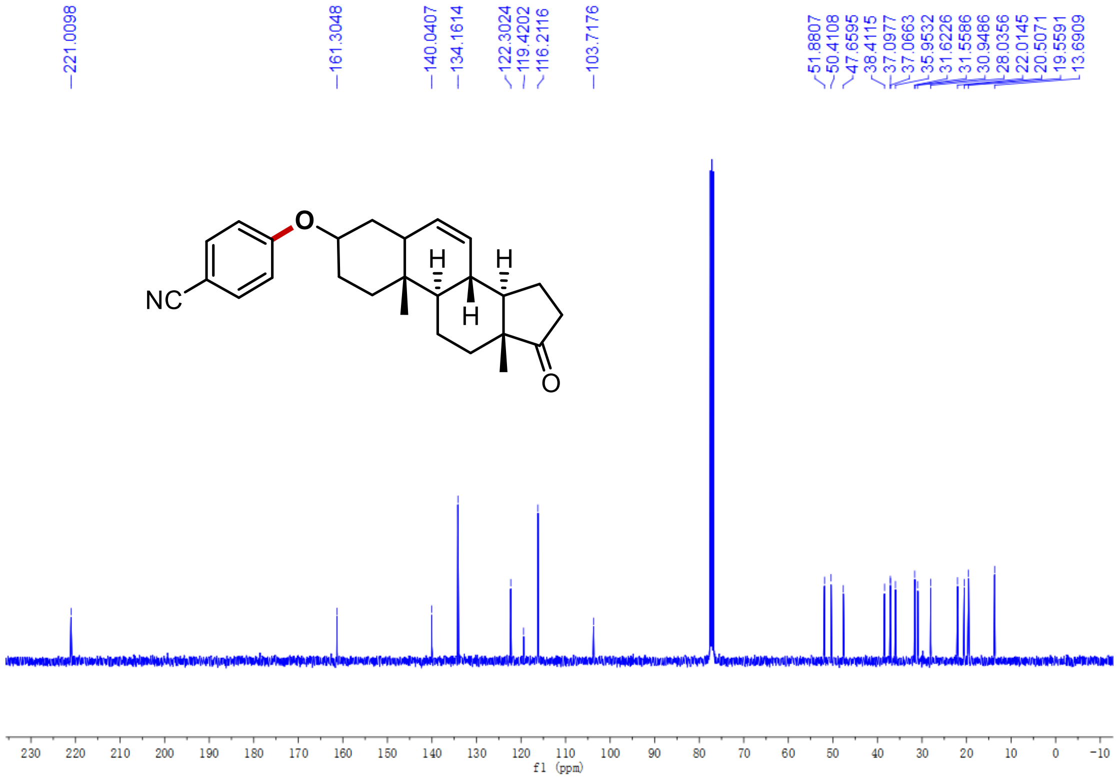

^{13}C NMR (100 MHz, $CDCl_3$) Spectrum

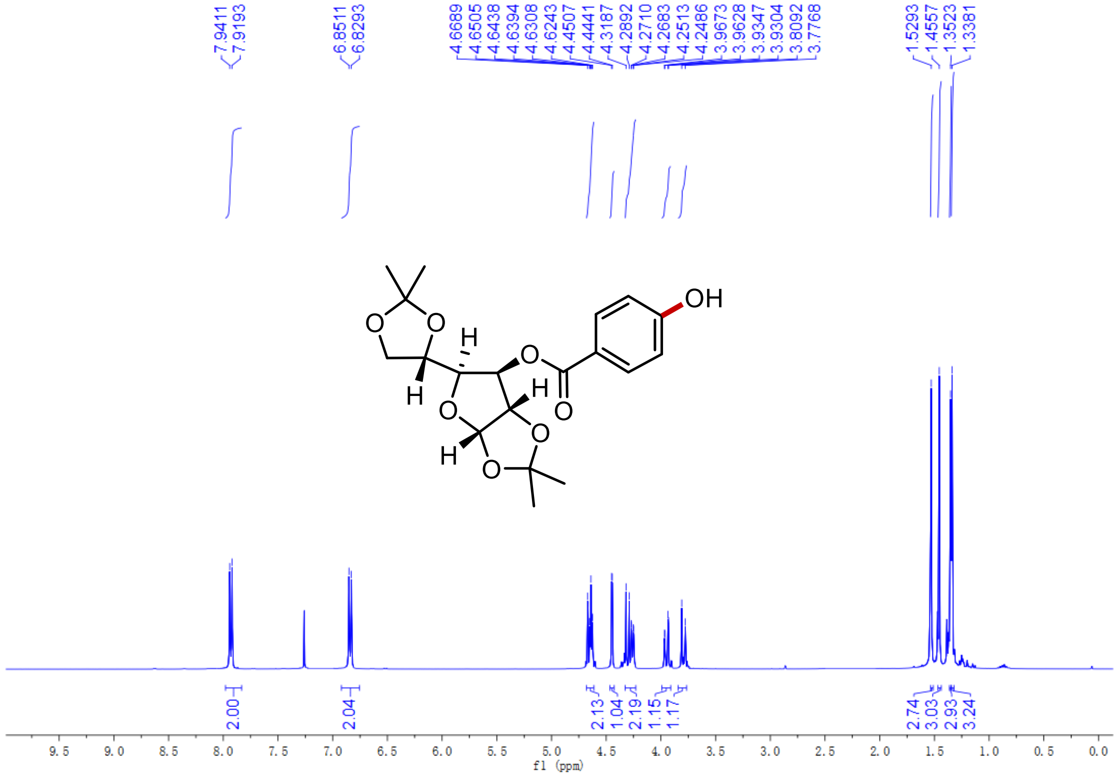

^{1}H NMR (400 MHz, $CDCl_3$) Spectrum

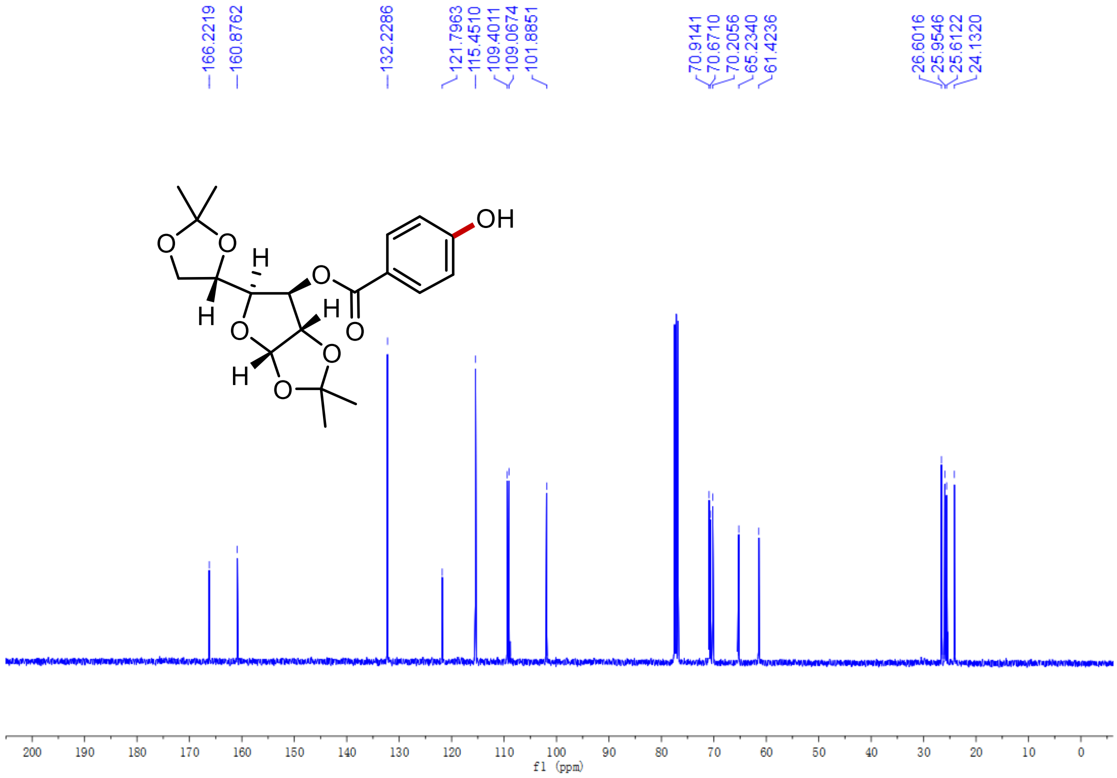

^{13}C NMR (100 MHz, $CDCl_3$) Spectrum

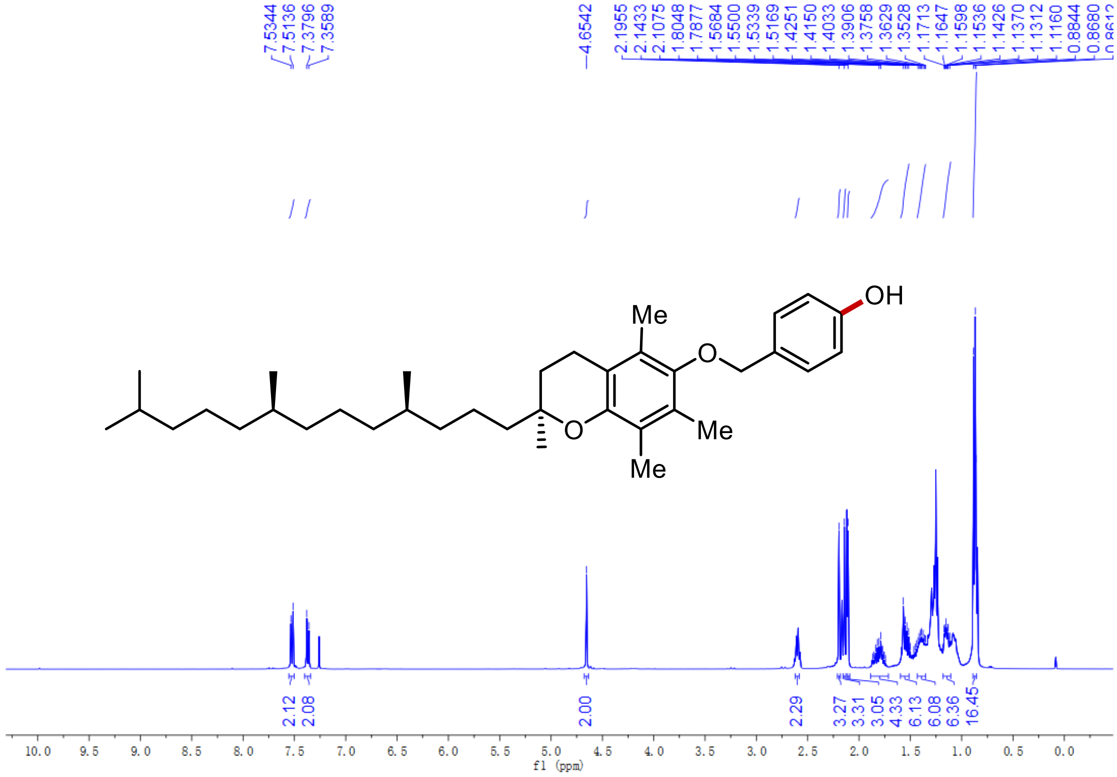

^{1}H NMR (400 MHz, $CDCl_3$) Spectrum

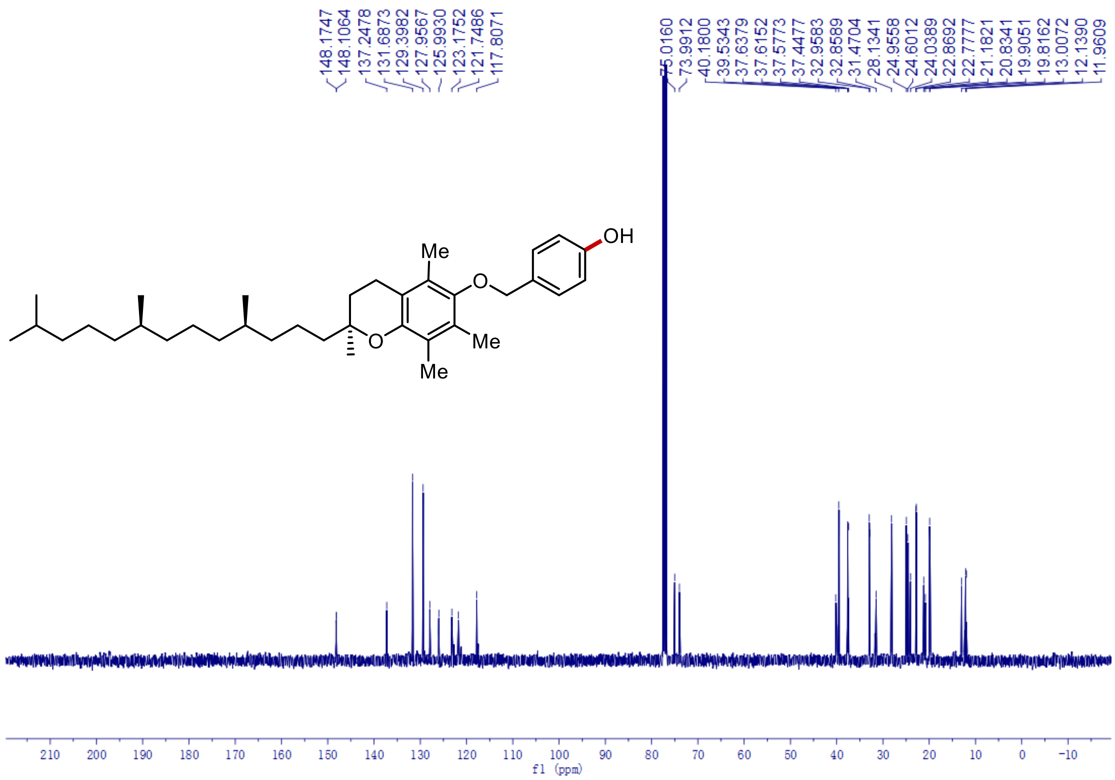

^{13}C NMR (100 MHz, $CDCl_3$) Spectrum

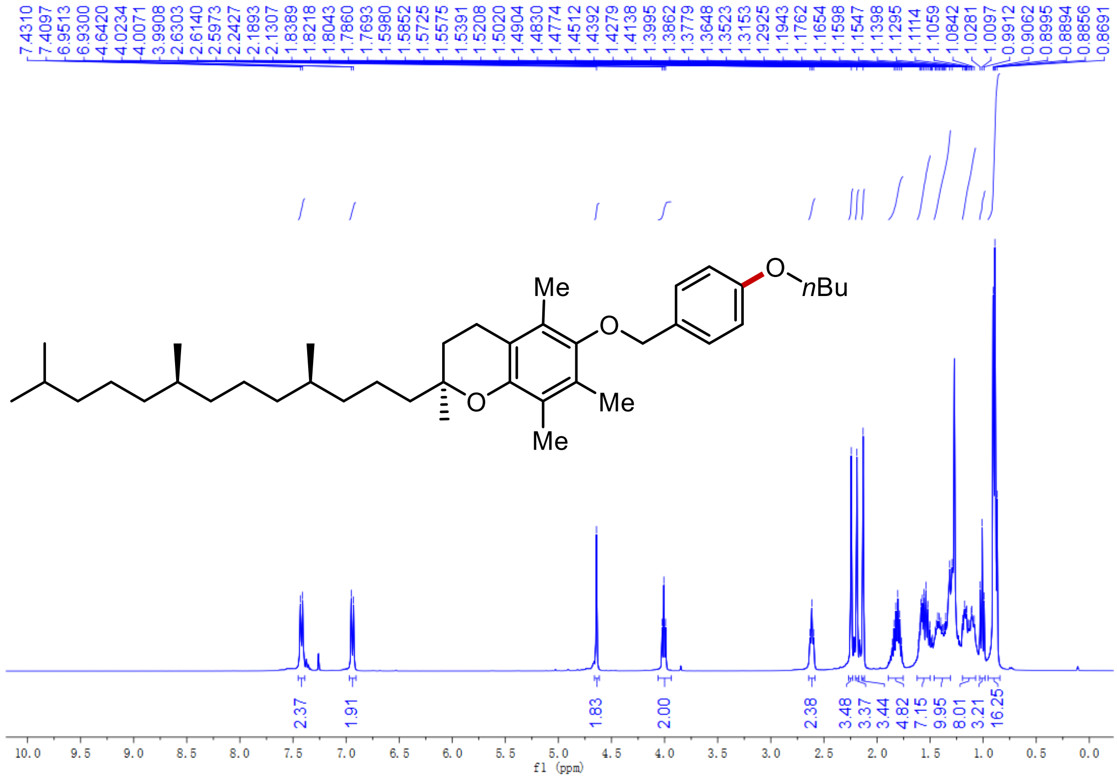

^{1}H NMR (400 MHz, $CDCl_3$) Spectrum

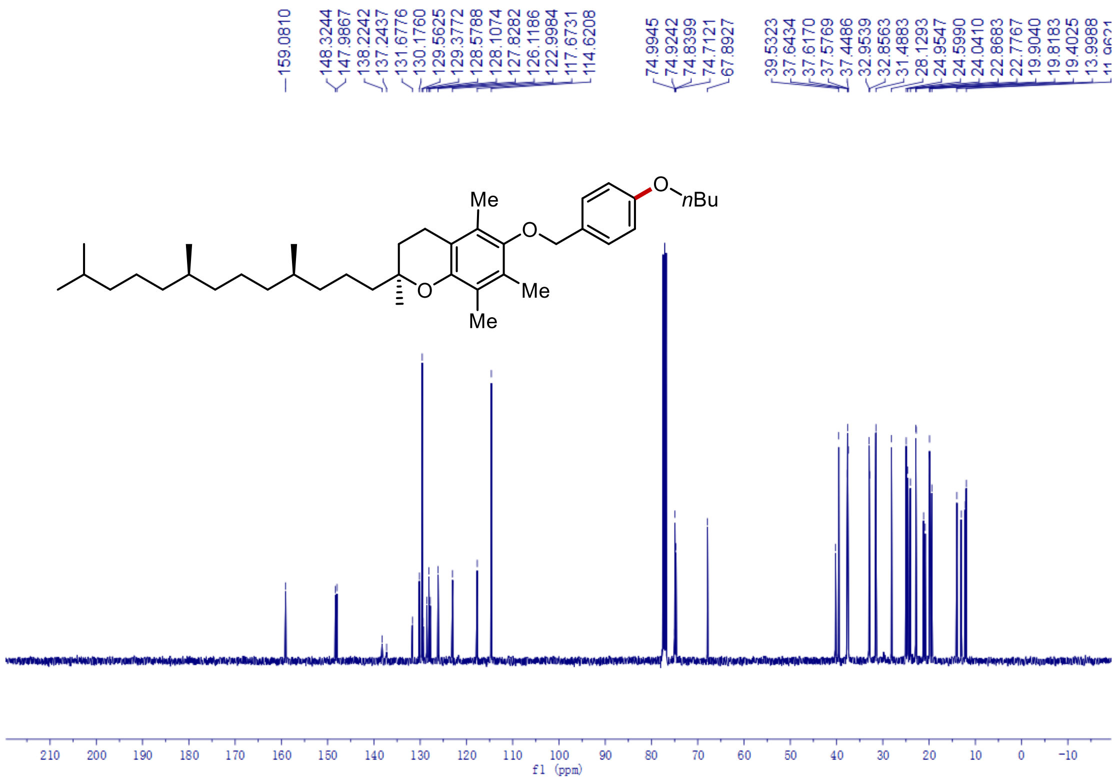

^{13}C NMR (100 MHz, $CDCl_3$) Spectrum

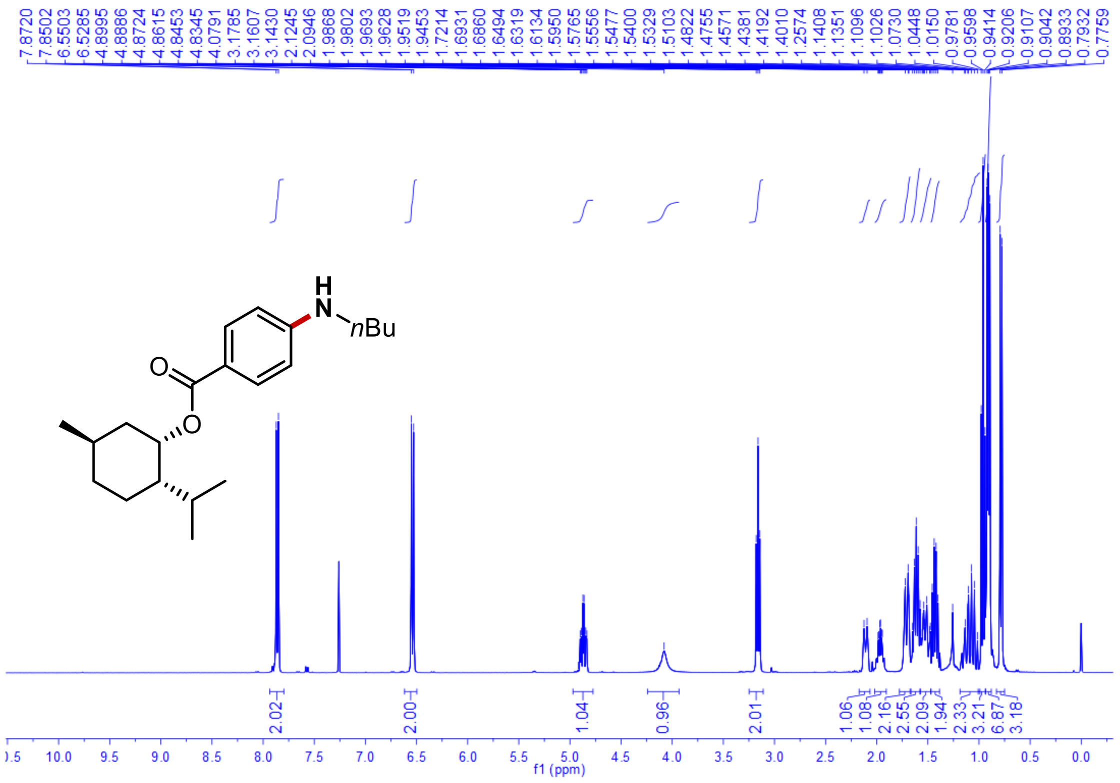

^{1}H NMR (400 MHz, $CDCl_3$) Spectrum

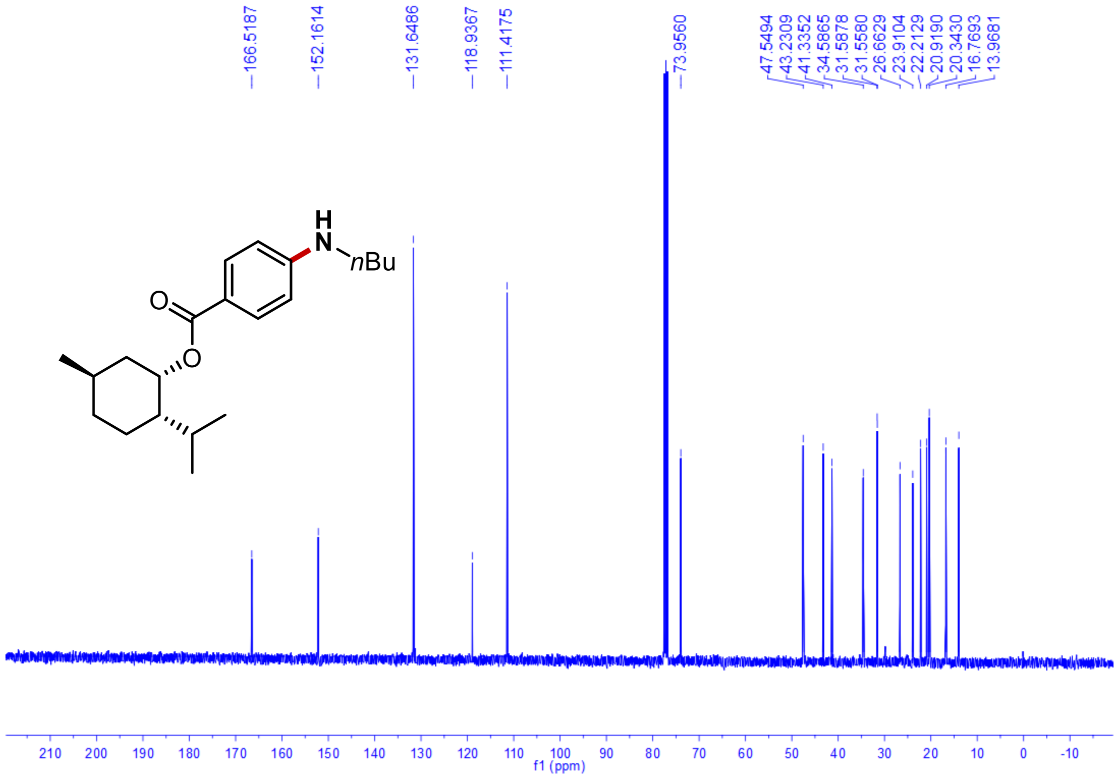

^{13}C NMR (100 MHz, $CDCl_3$) Spectrum

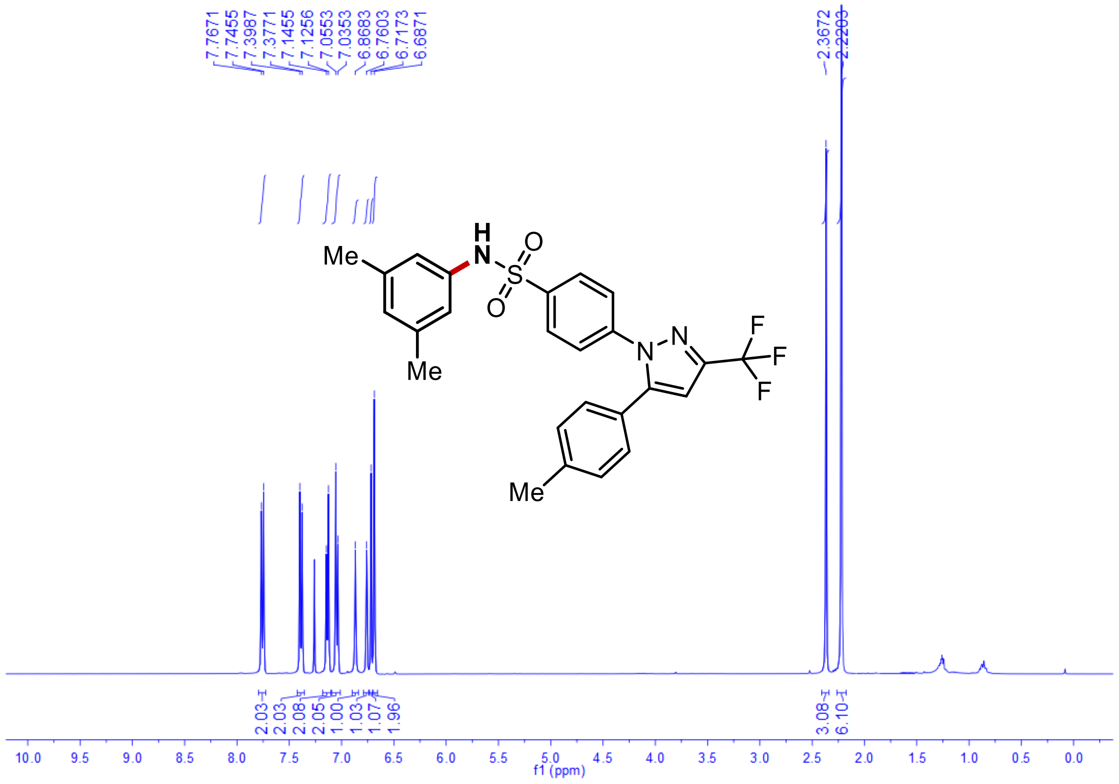

^{1}H NMR (400 MHz, $CDCl_3$) Spectrum

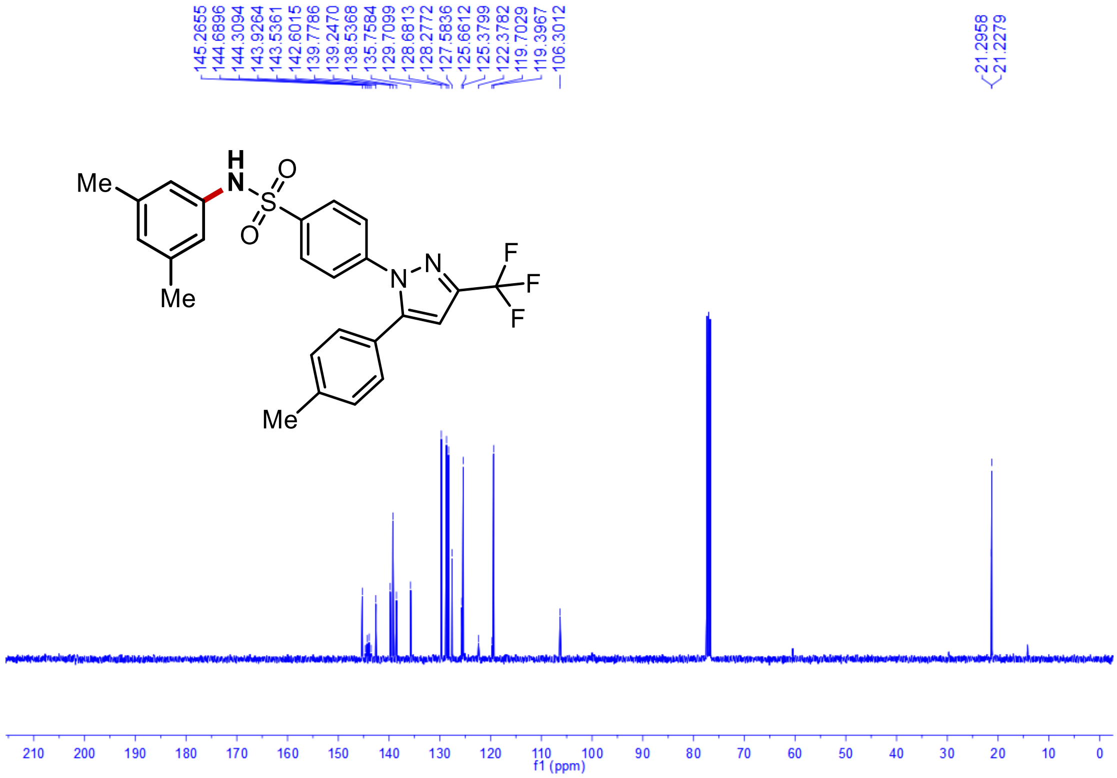

^{13}**C NMR** (100 MHz, $CDCl_3$) Spectrum

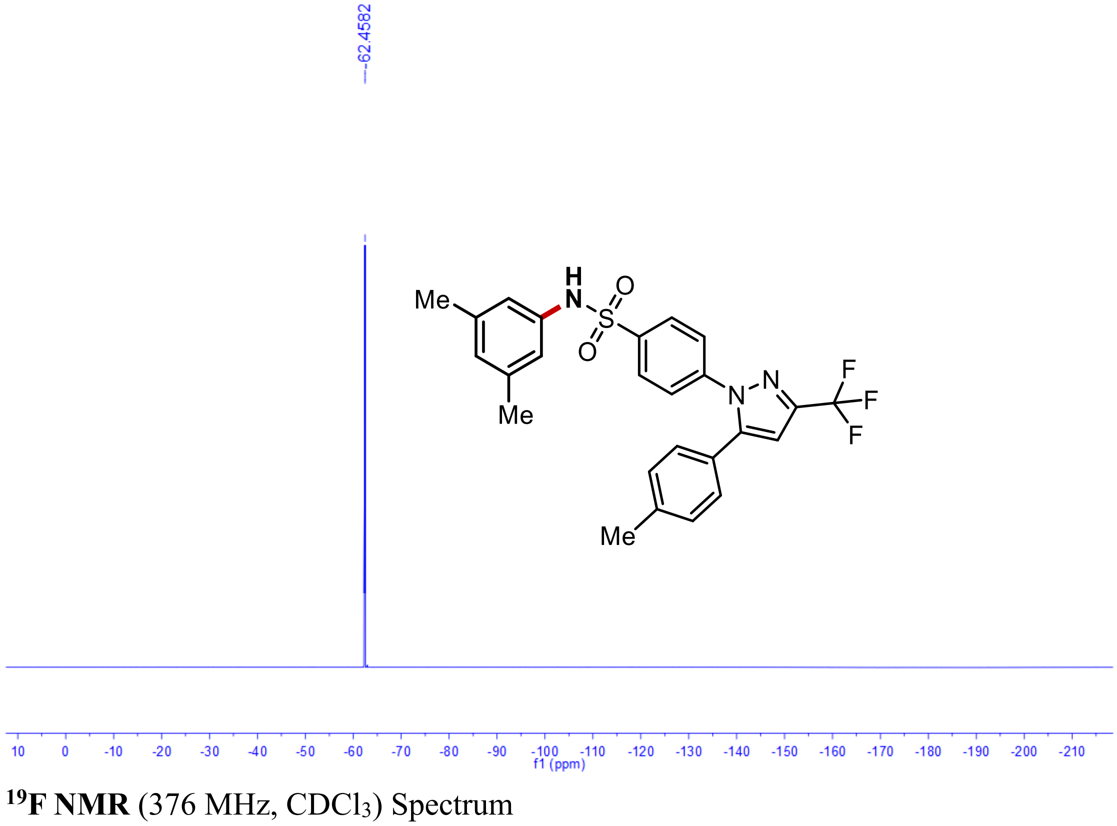

^{19}**F NMR** (376 MHz, $CDCl_3$) Spectrum

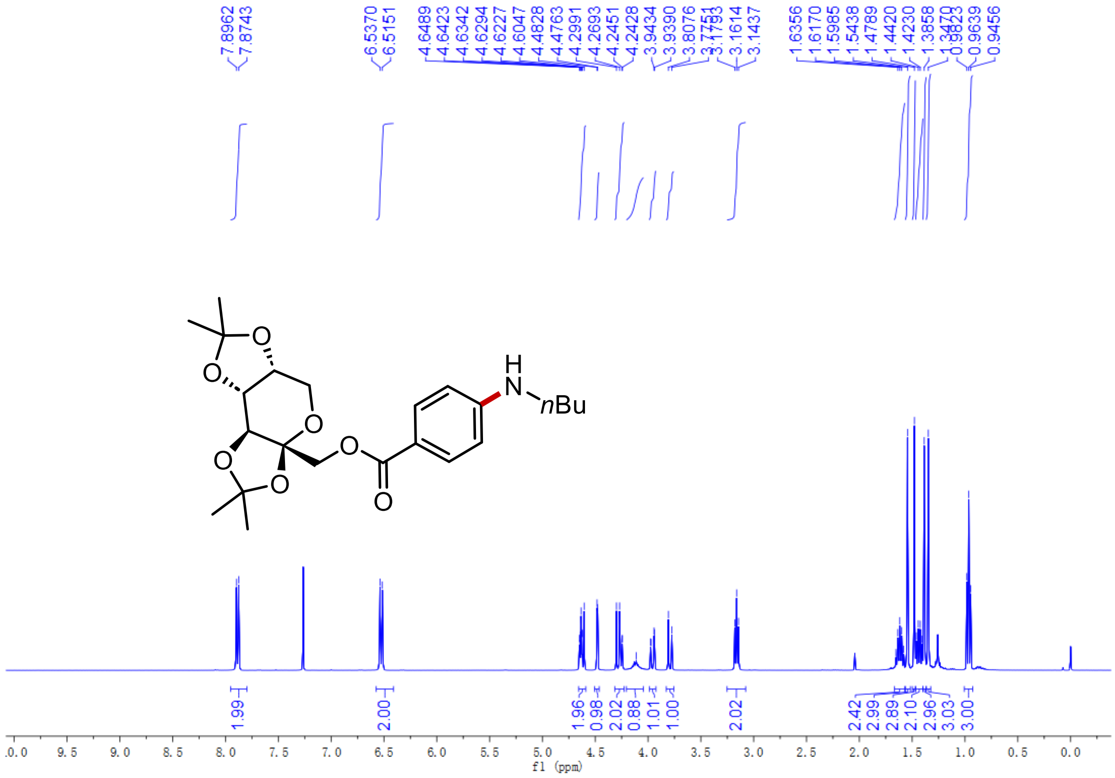

^{1}H NMR (400 MHz, $CDCl_3$) Spectrum

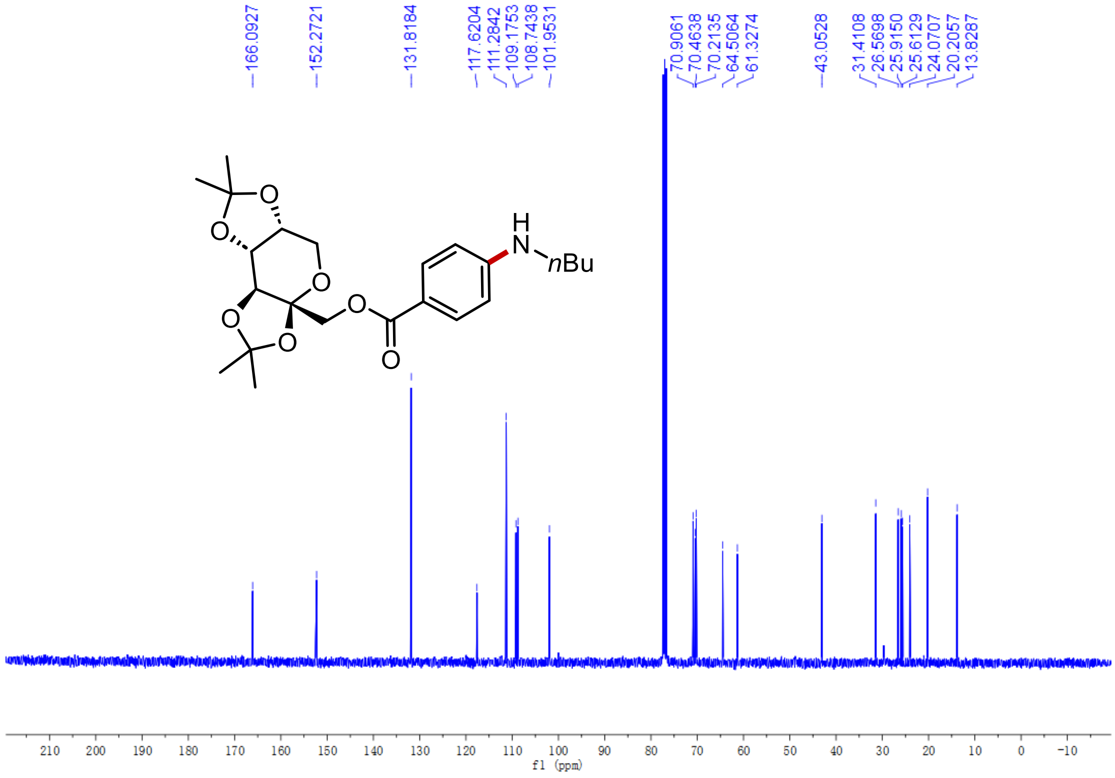

^{13}C NMR (100 MHz, $CDCl_3$) Spectrum

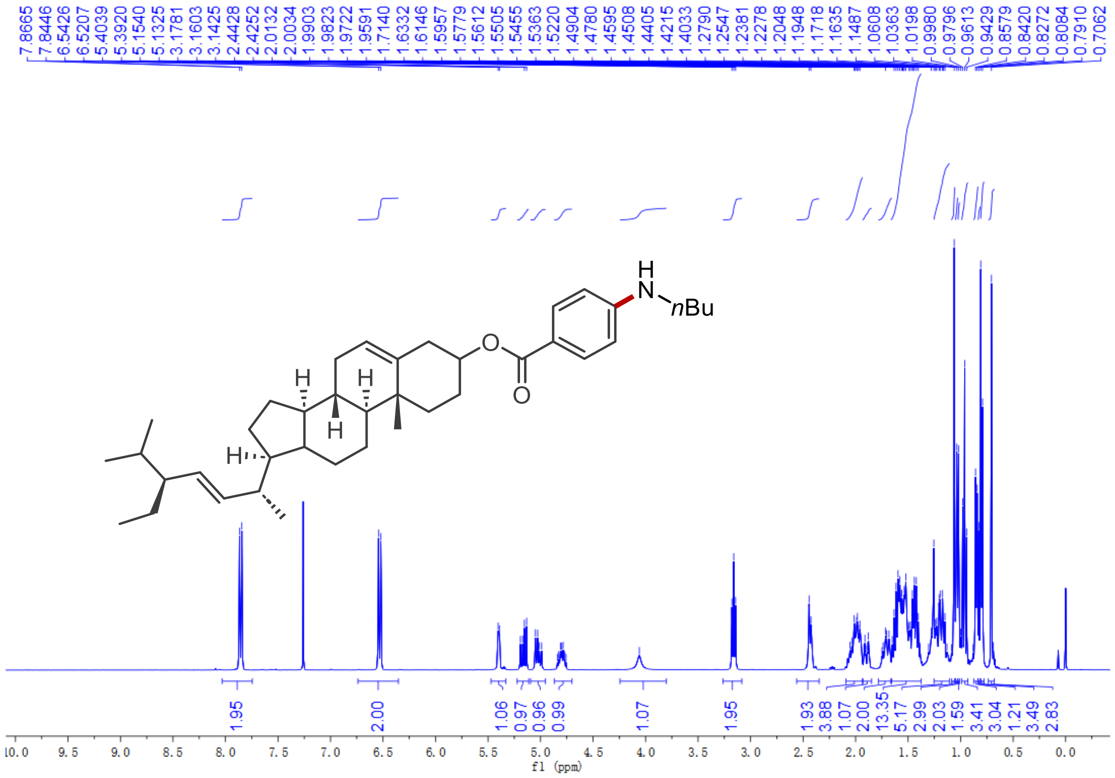

^{1}H NMR (400 MHz, $CDCl_3$) Spectrum

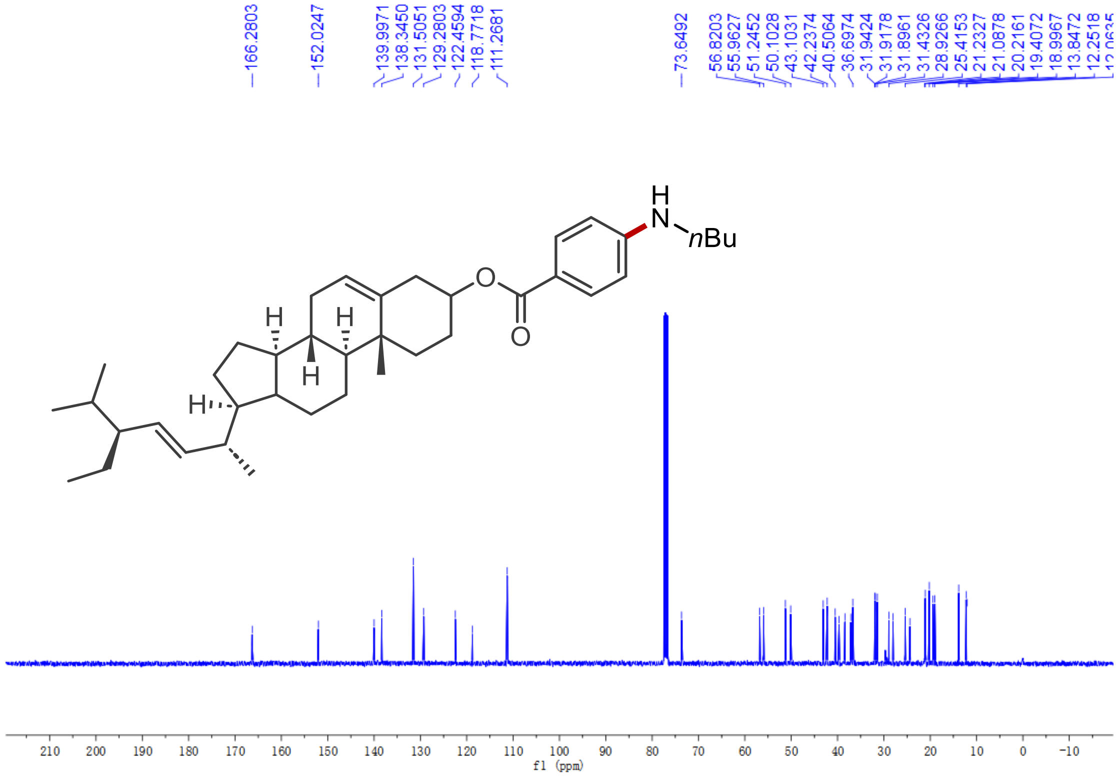

^{13}C NMR (100 MHz, $CDCl_3$) Spectrum

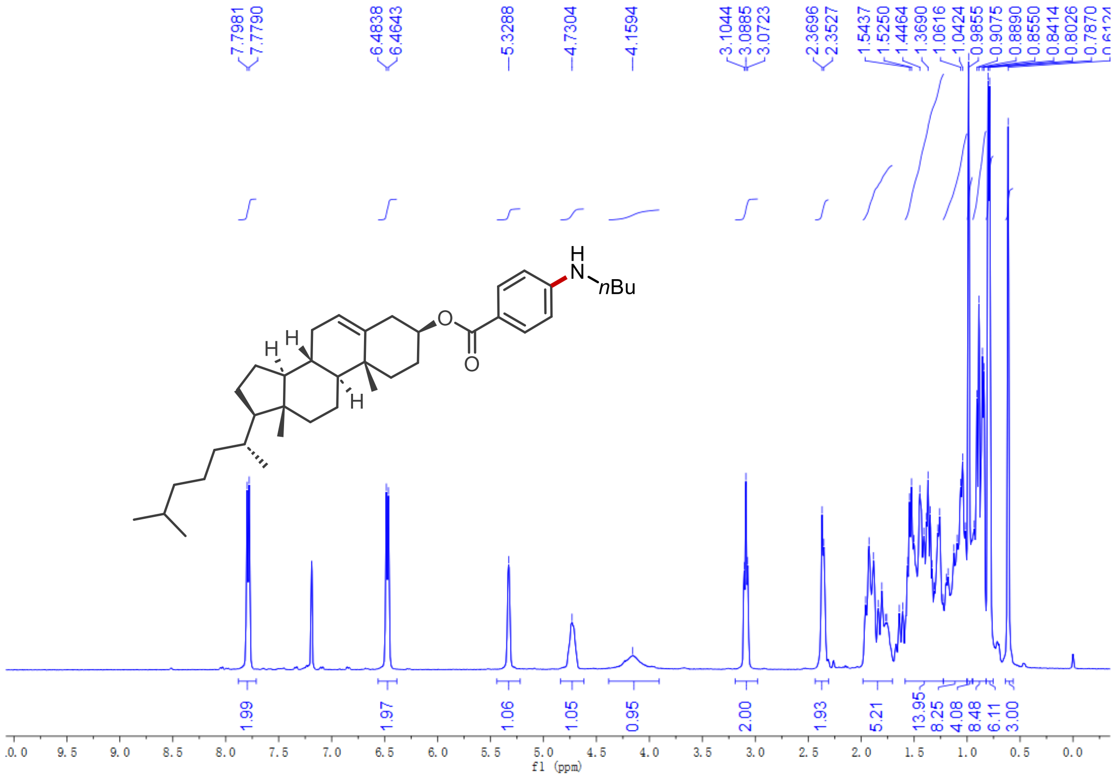

^{1}H NMR (400 MHz, $CDCl_3$) Spectrum

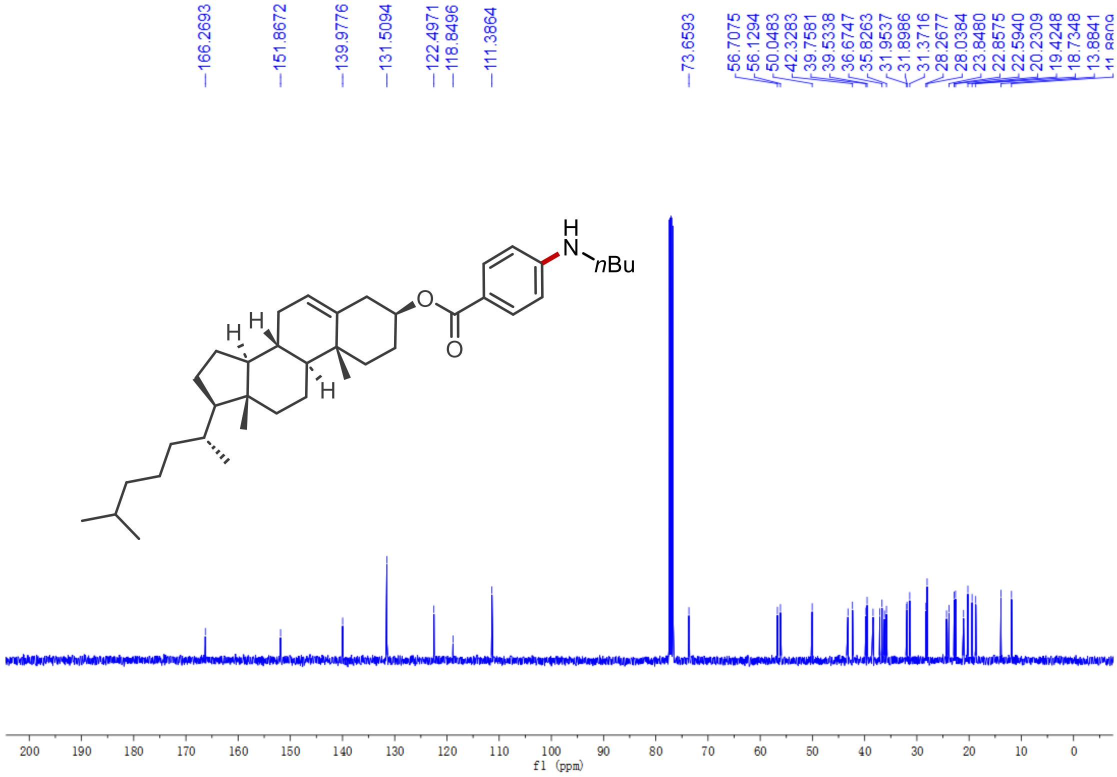

^{13}C NMR (100 MHz, $CDCl_3$) Spectrum

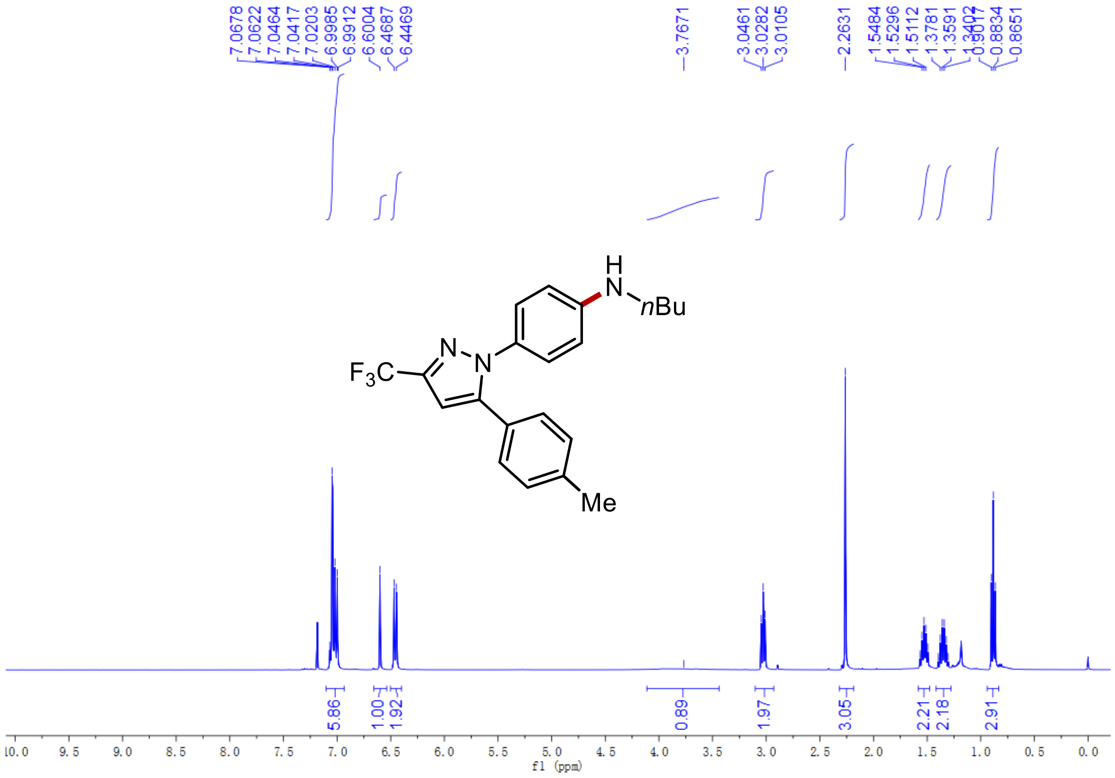

^{1}H NMR (400 MHz, $CDCl_3$) Spectrum

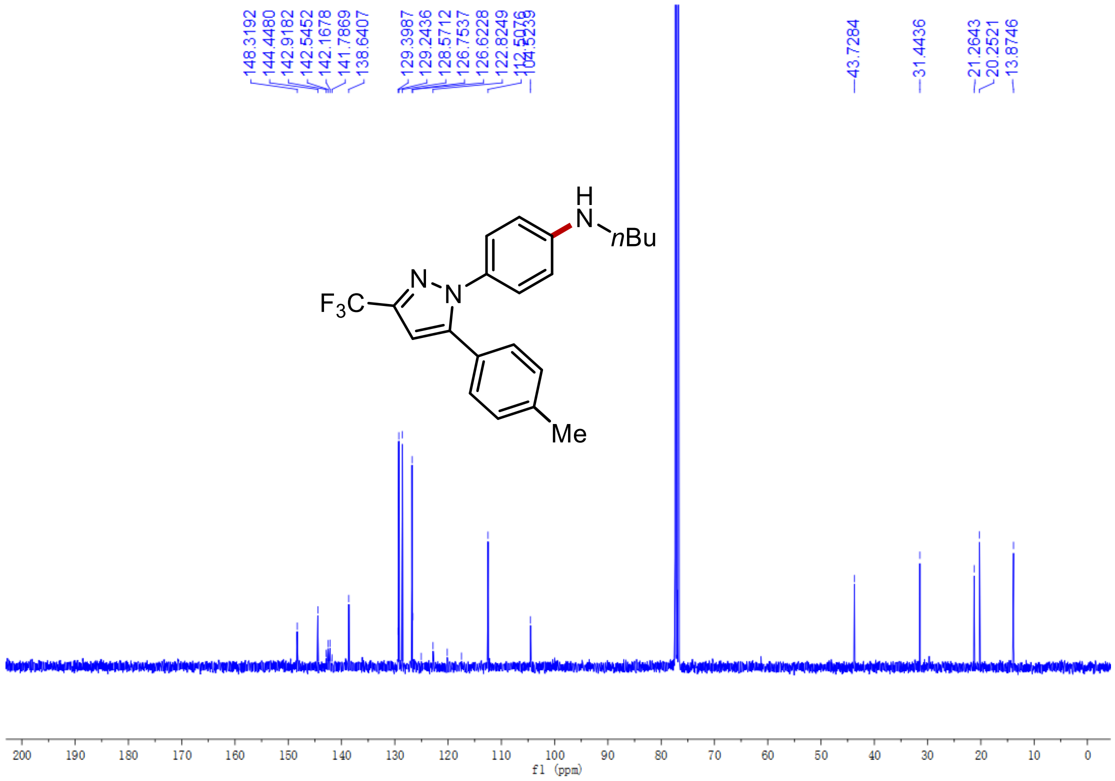

^{13}C NMR (100 MHz, $CDCl_3$) Spectrum

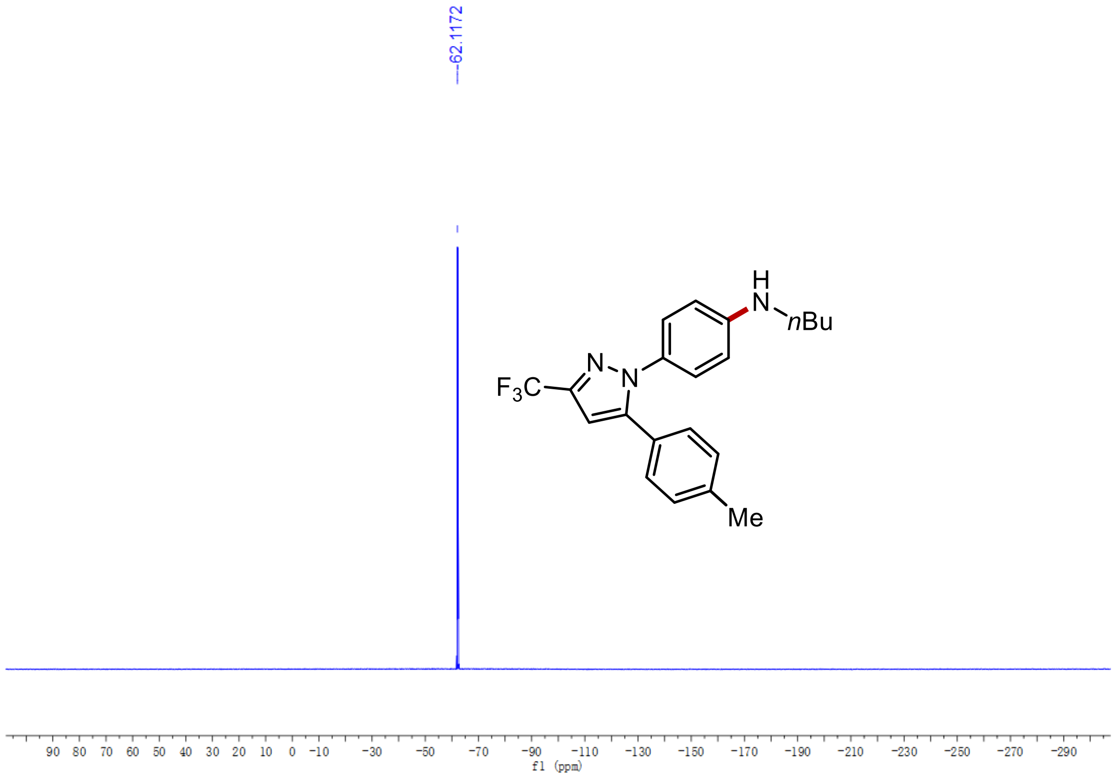

^{19}F NMR (376 MHz, $CDCl_3$) Spectrum

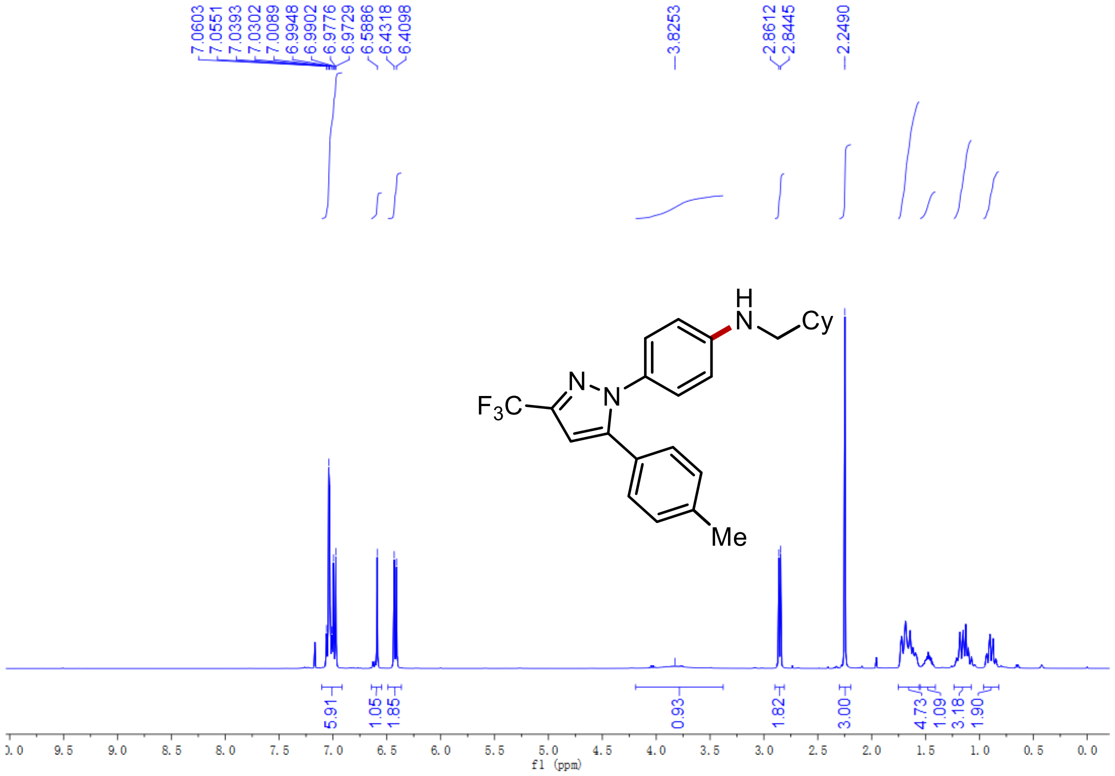

^{1}H NMR (400 MHz, $CDCl_3$) Spectrum

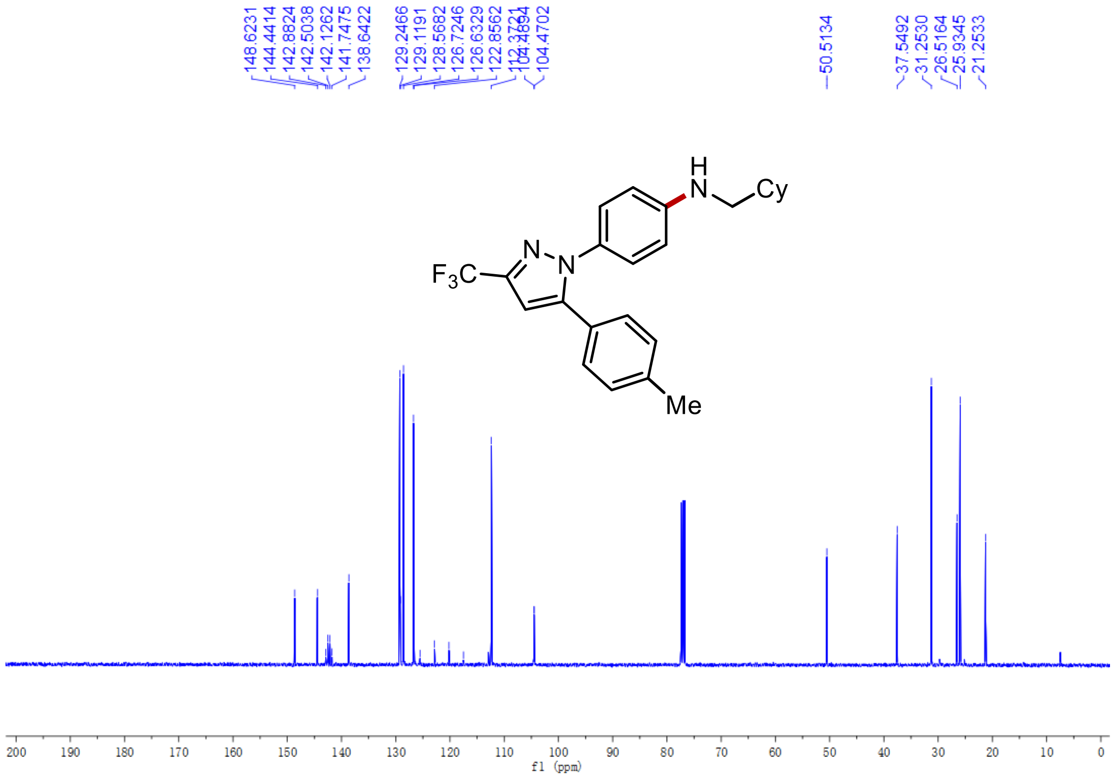

^{13}C NMR (100 MHz, $CDCl_3$) Spectrum

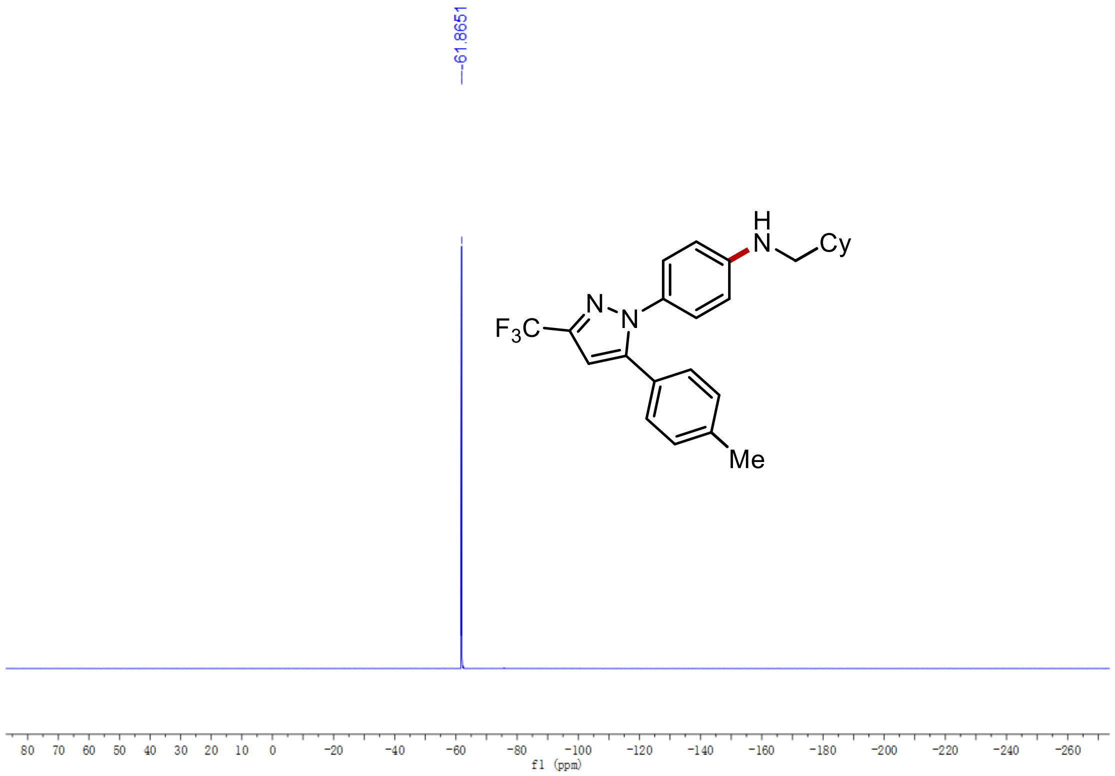

^{19}F NMR (376 MHz, $CDCl_3$) Spectrum

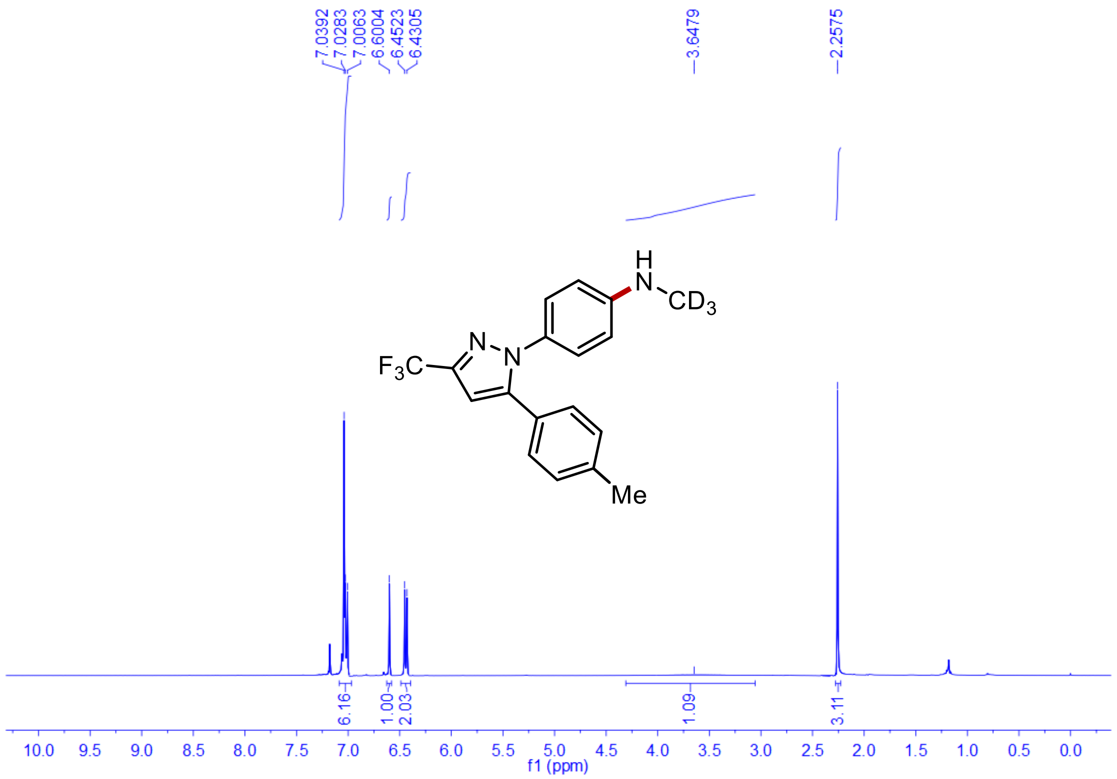

^{1}H NMR (400 MHz, $CDCl_3$) Spectrum

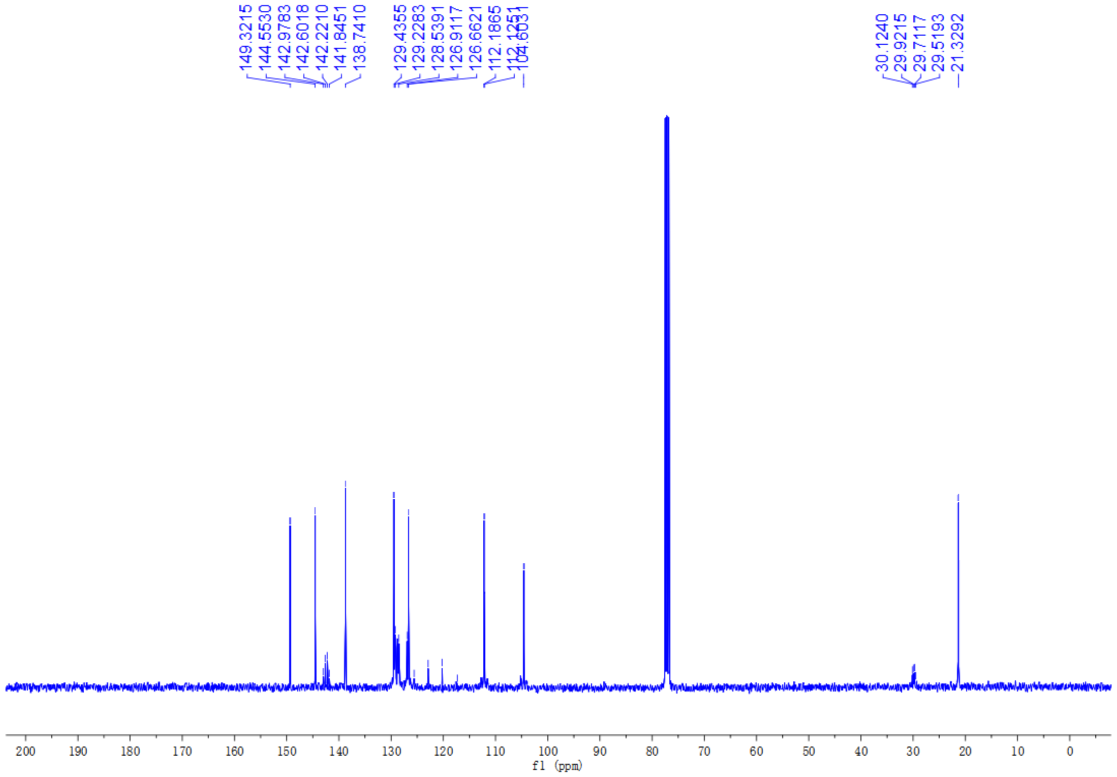

^{13}C NMR (100 MHz, $CDCl_3$) Spectrum

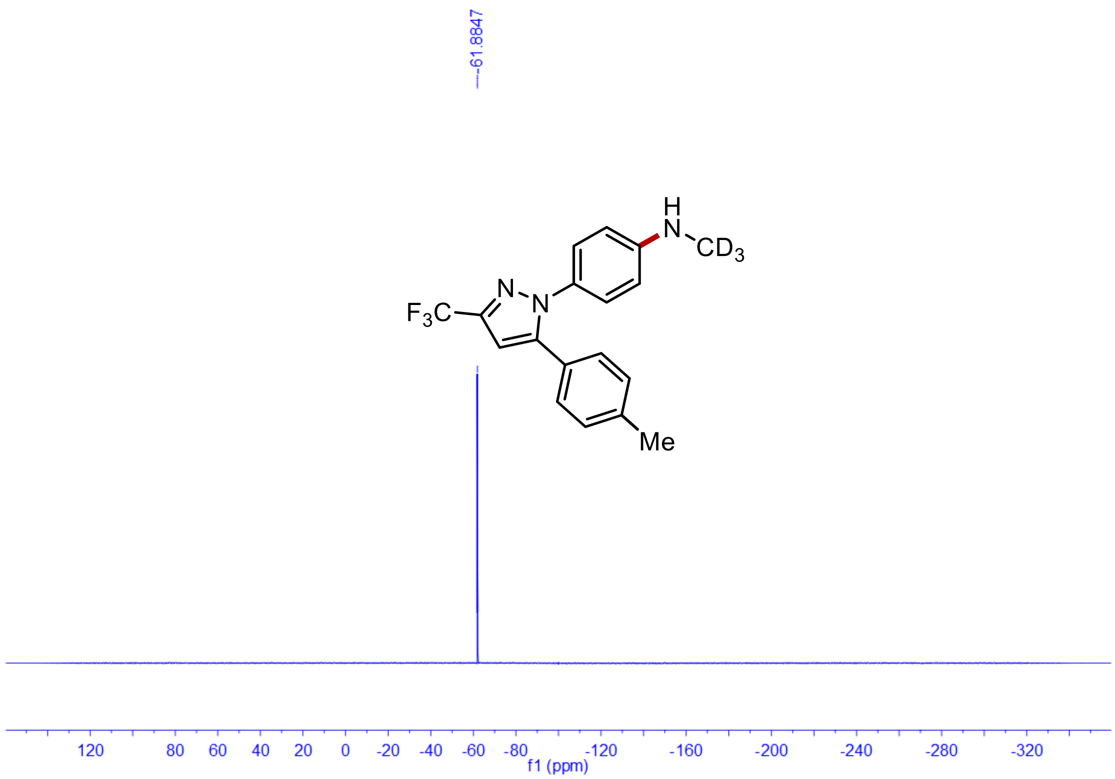

^{19}F NMR (376 MHz, $CDCl_3$) Spectrum

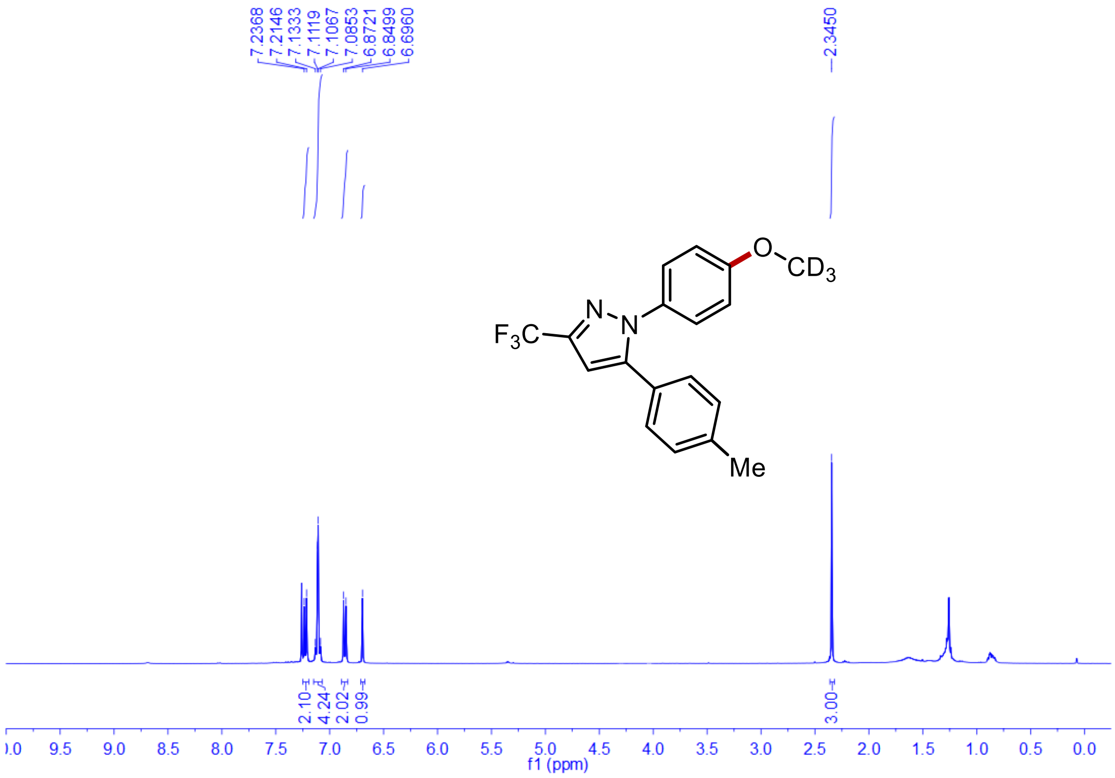

^{1}H NMR (400 MHz, $CDCl_3$) Spectrum

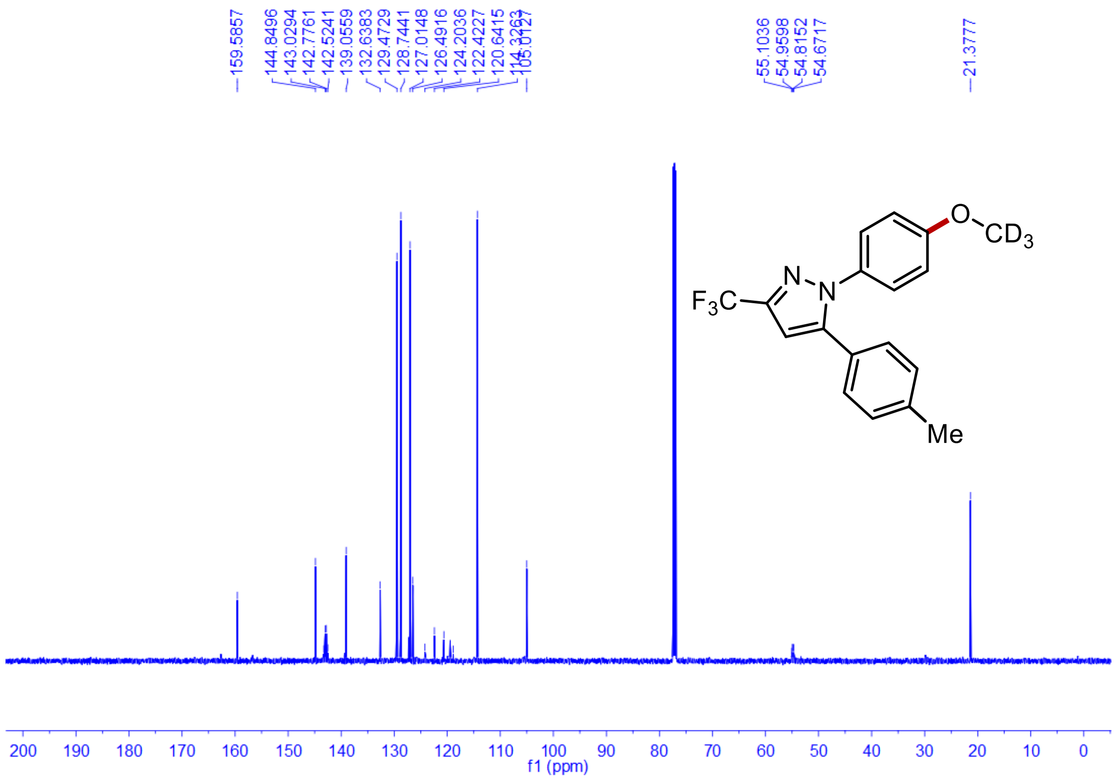

^{13}C NMR (150 MHz, $CDCl_3$) Spectrum

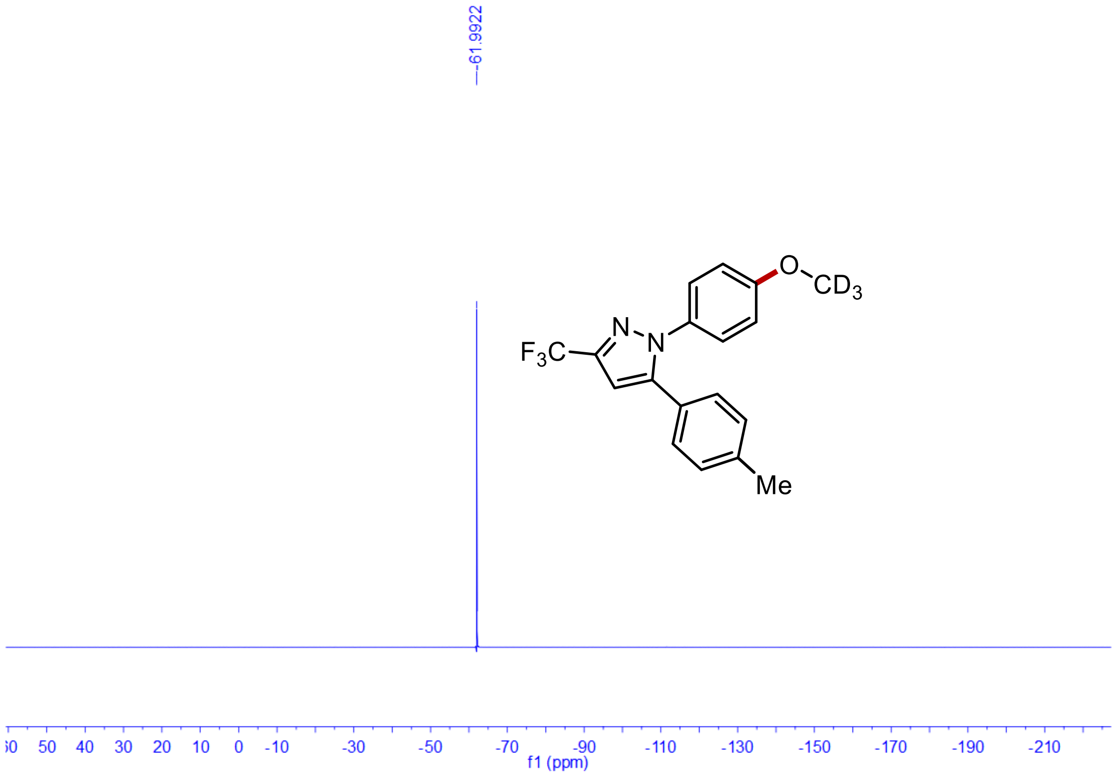

^{19}F NMR (376 MHz, $CDCl_3$) Spectrum

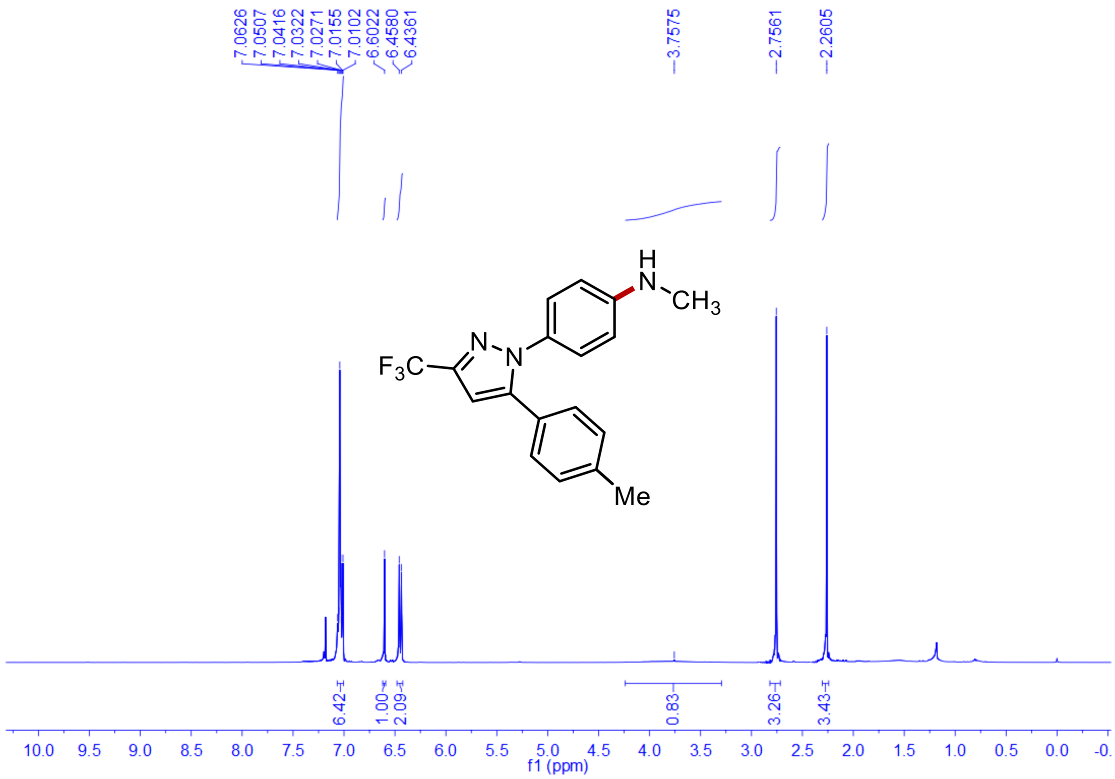

^{1}**H NMR** (400 MHz, $CDCl_3$) Spectrum

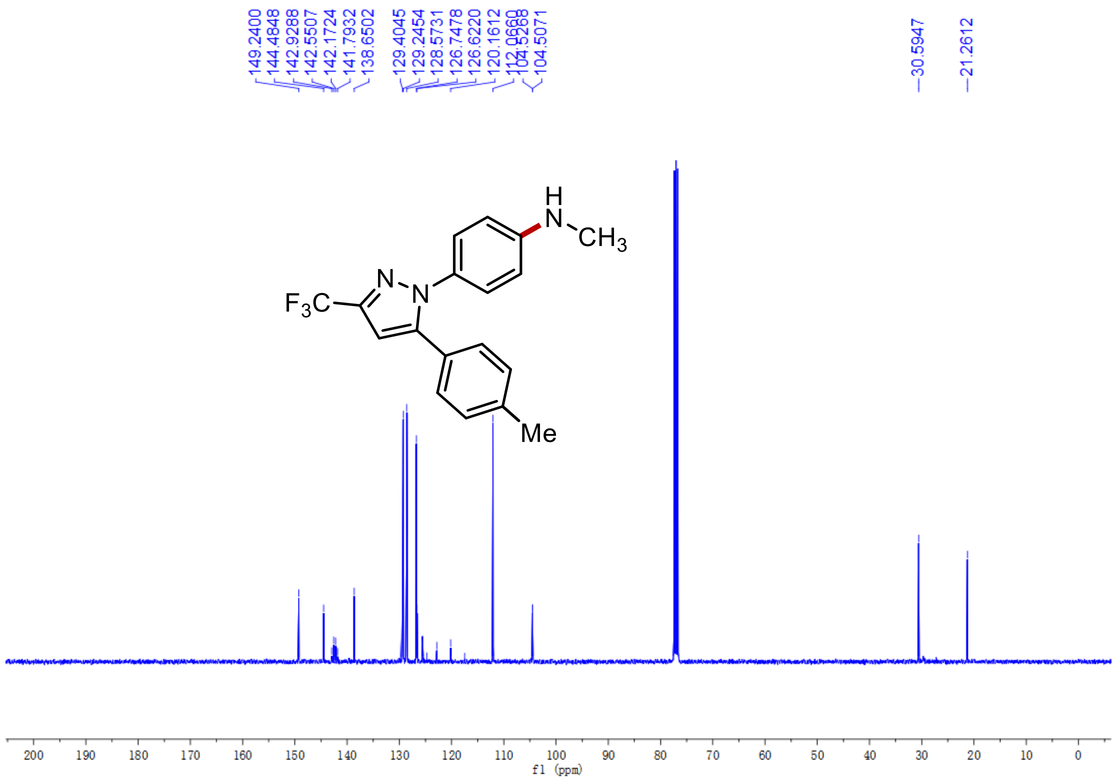

^{13}**C NMR** (100 MHz, $CDCl_3$) Spectrum

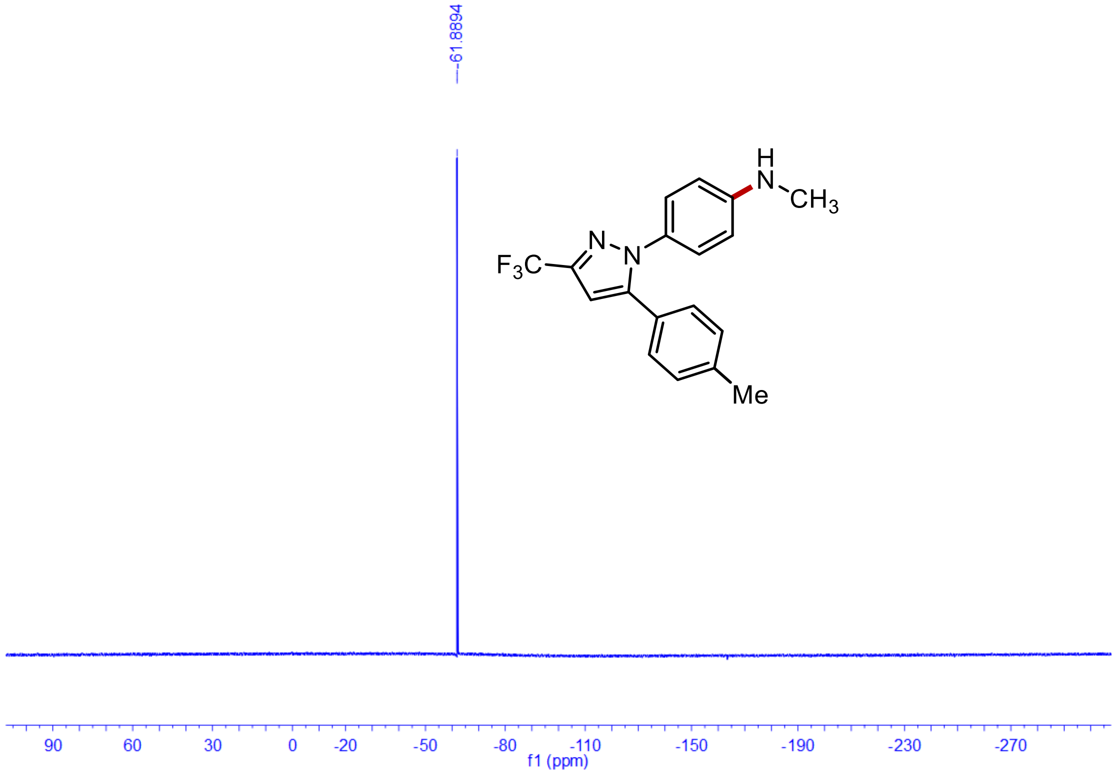

^{19}F NMR (376 MHz, $CDCl_3$) Spectrum

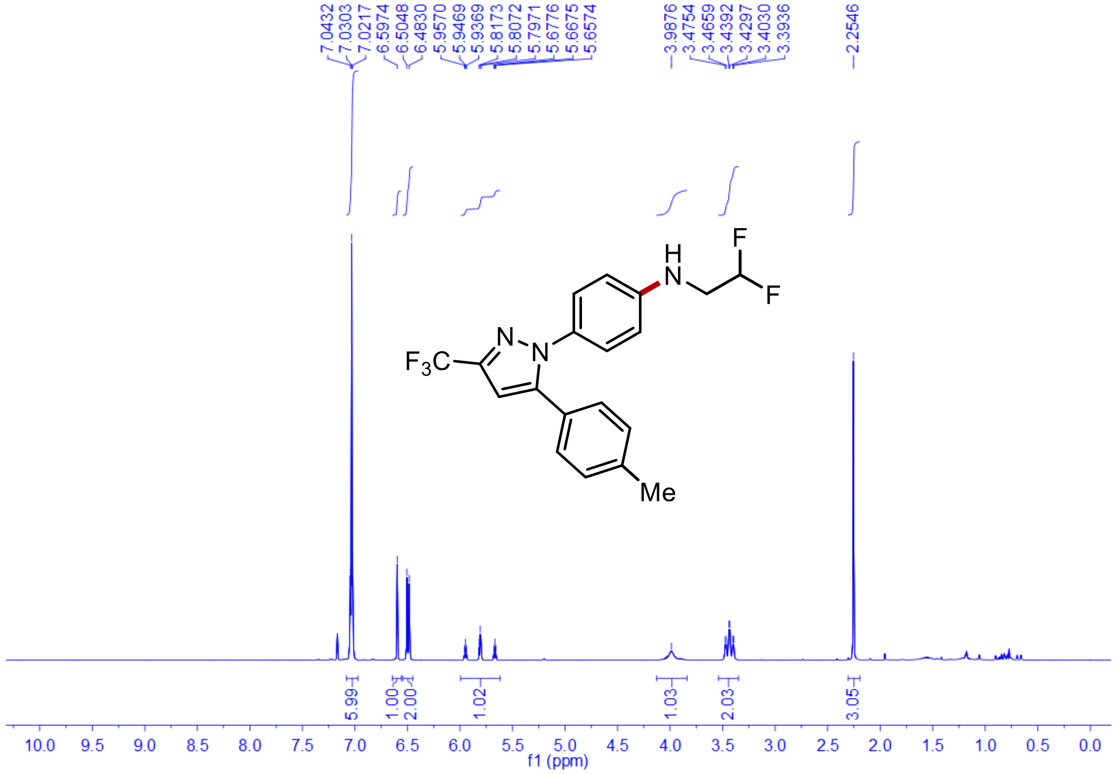

^{1}H NMR (400 MHz, $CDCl_3$) Spectrum

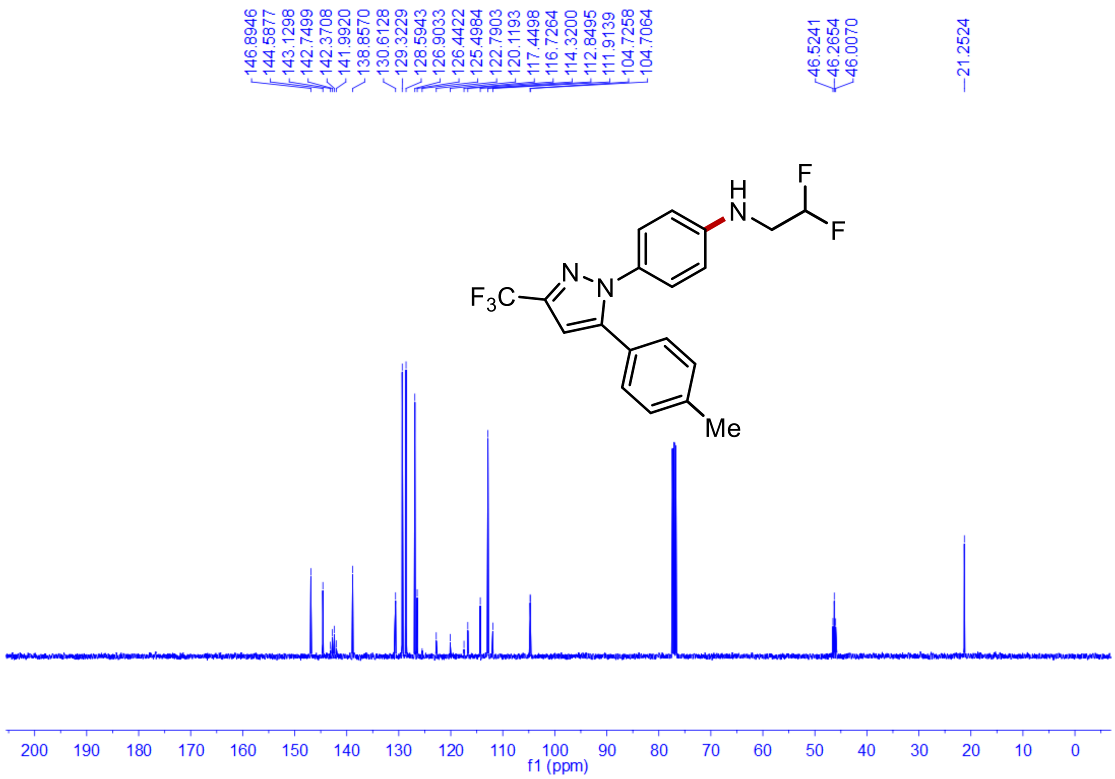

^{13}C NMR (100 MHz, $CDCl_3$) Spectrum

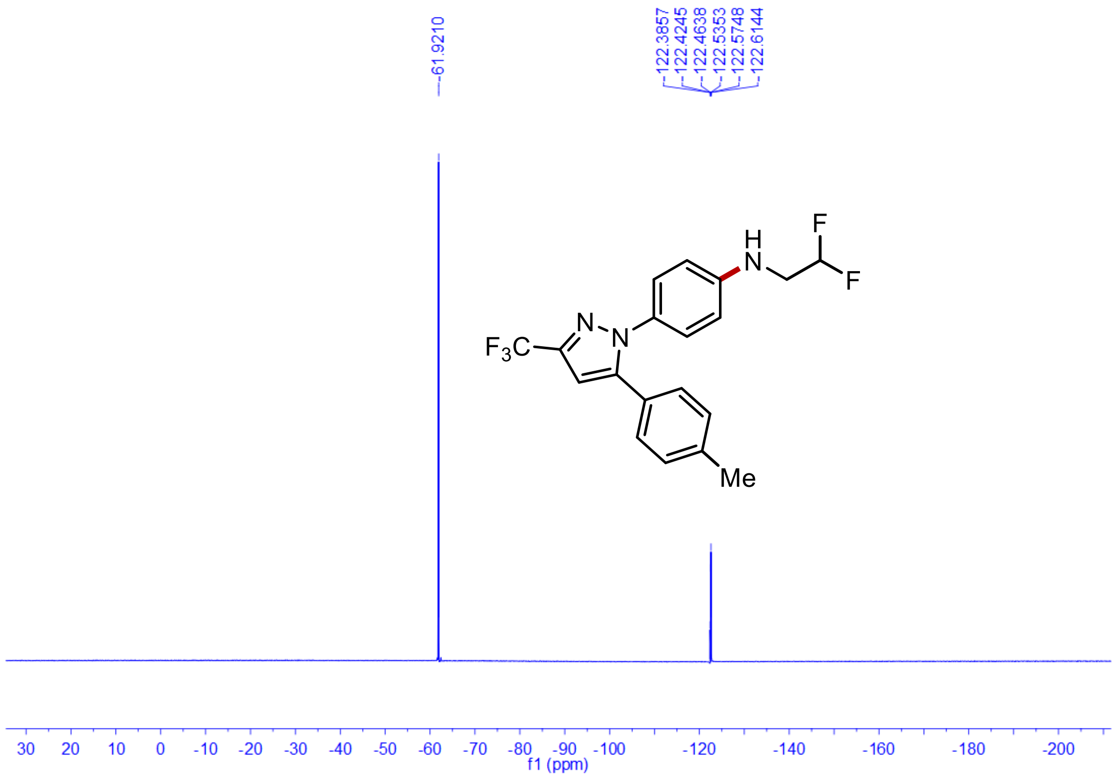

^{19}F NMR (376 MHz, $CDCl_3$) Spectrum

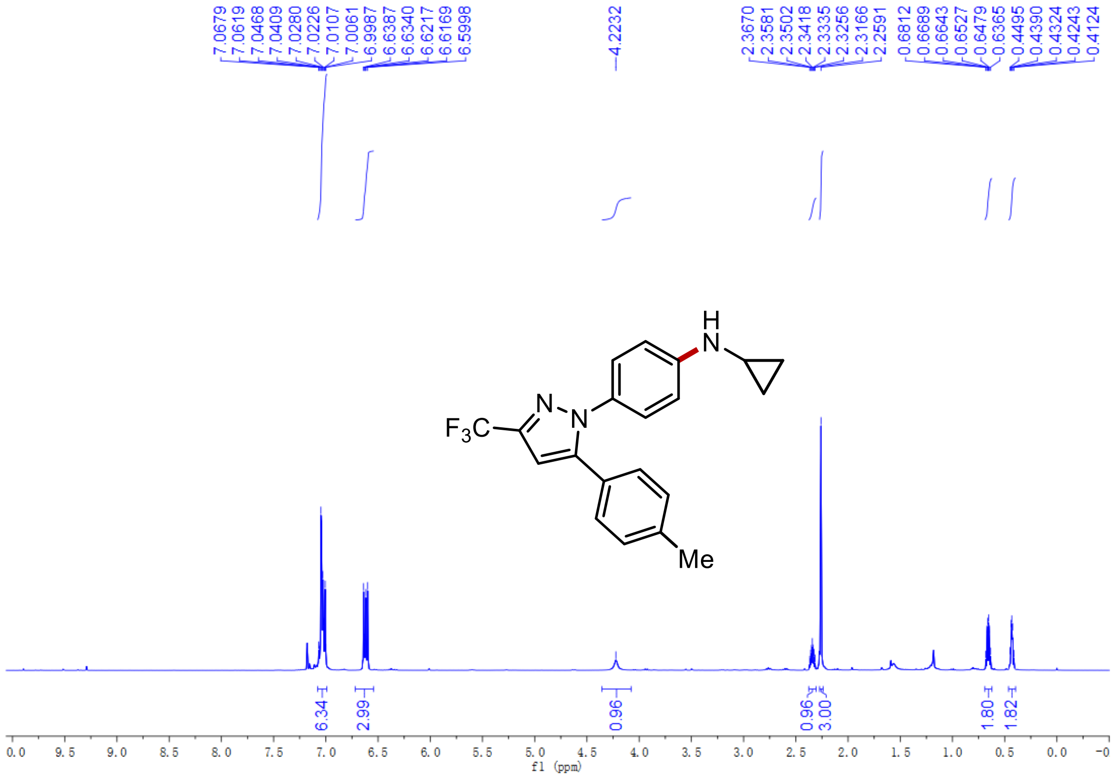

^{1}H NMR (400 MHz, $CDCl_3$) Spectrum

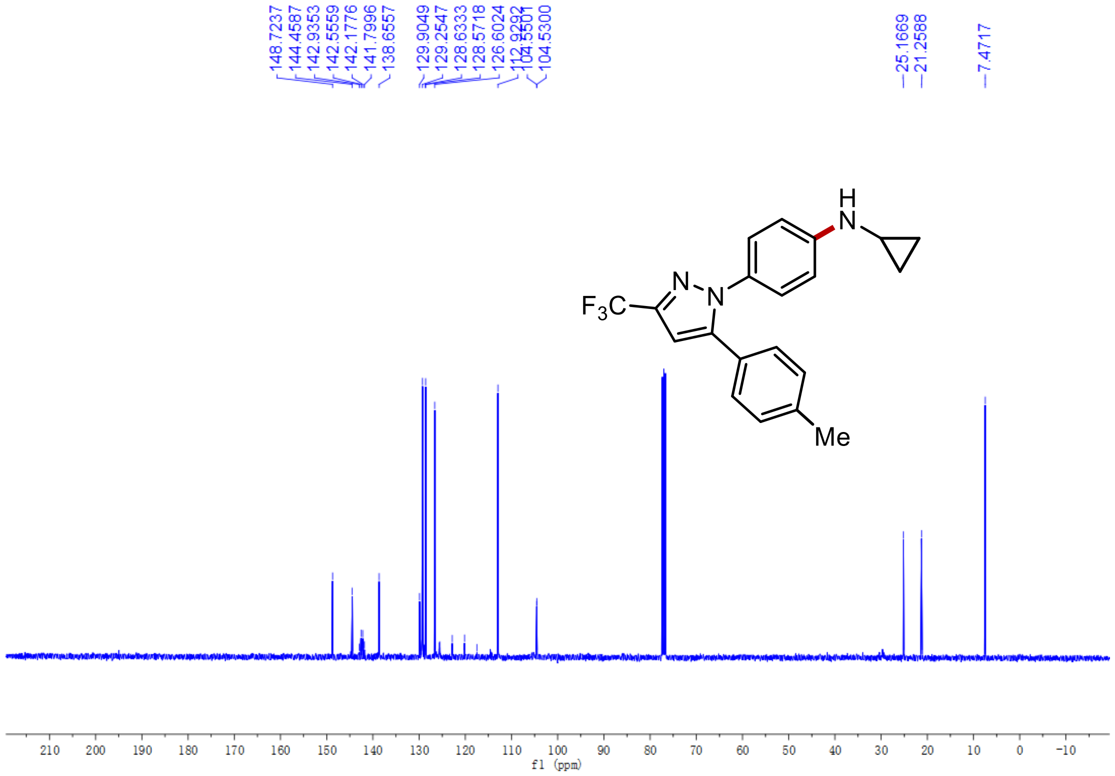

^{13}C NMR (100 MHz, $CDCl_3$) Spectrum

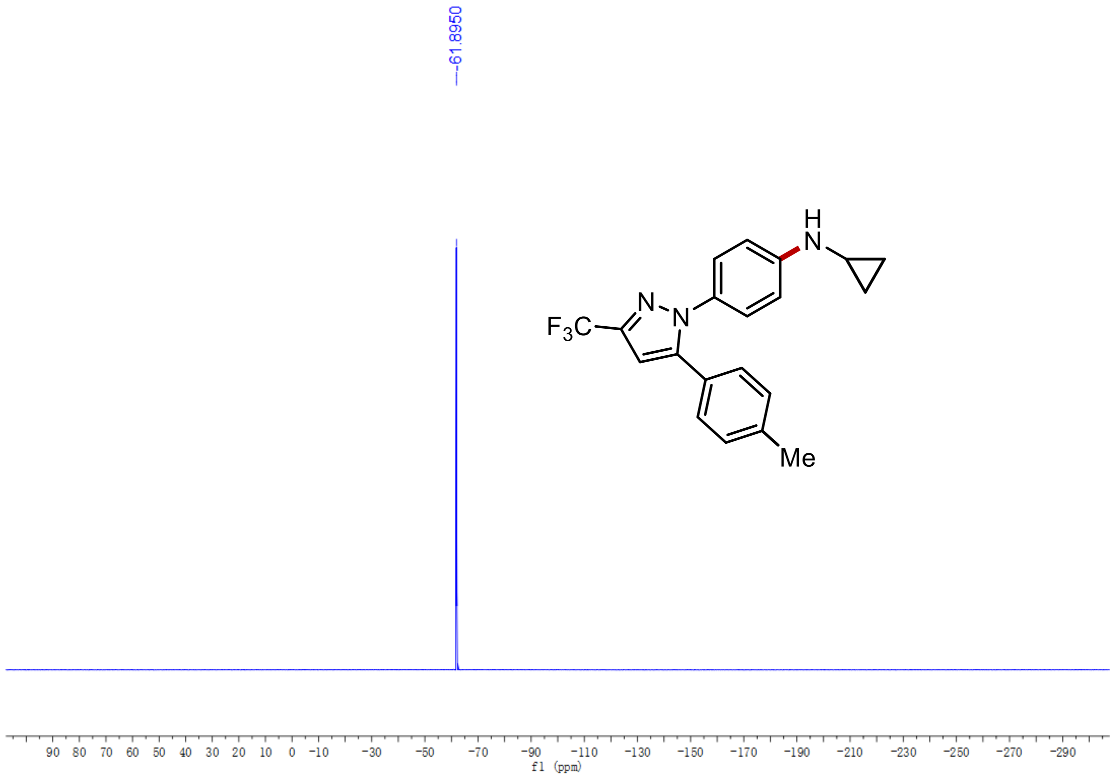

^{19}F NMR (376 MHz, $CDCl_3$) Spectrum

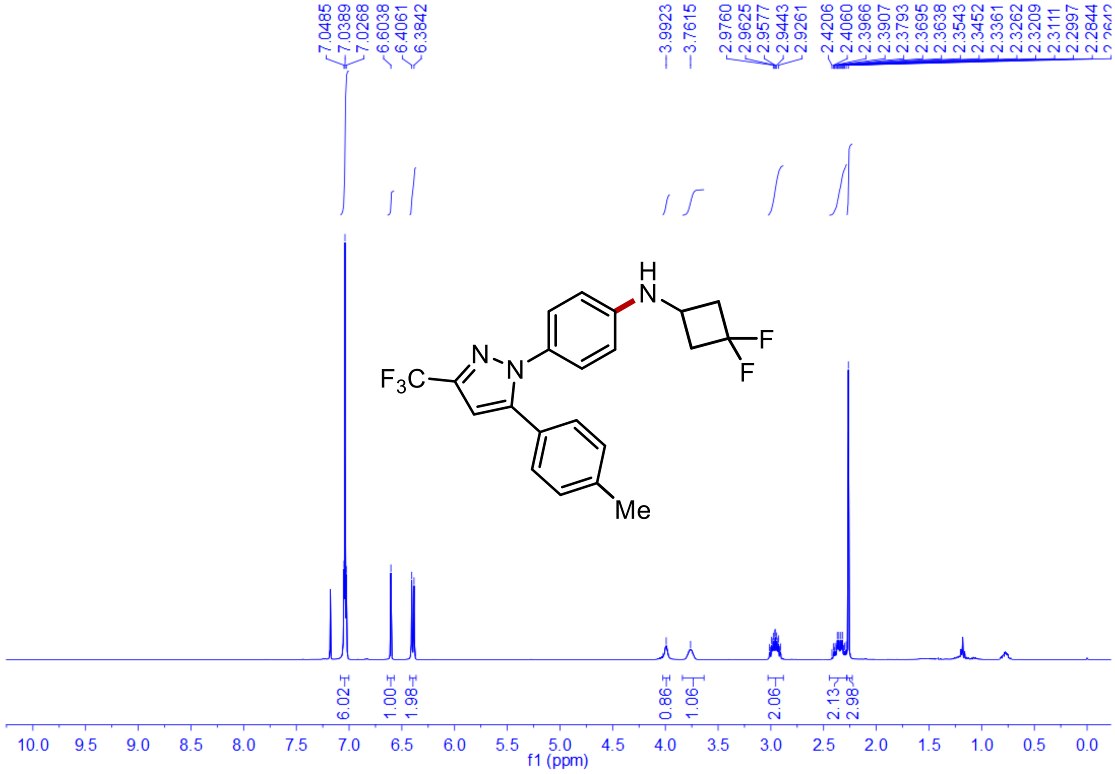

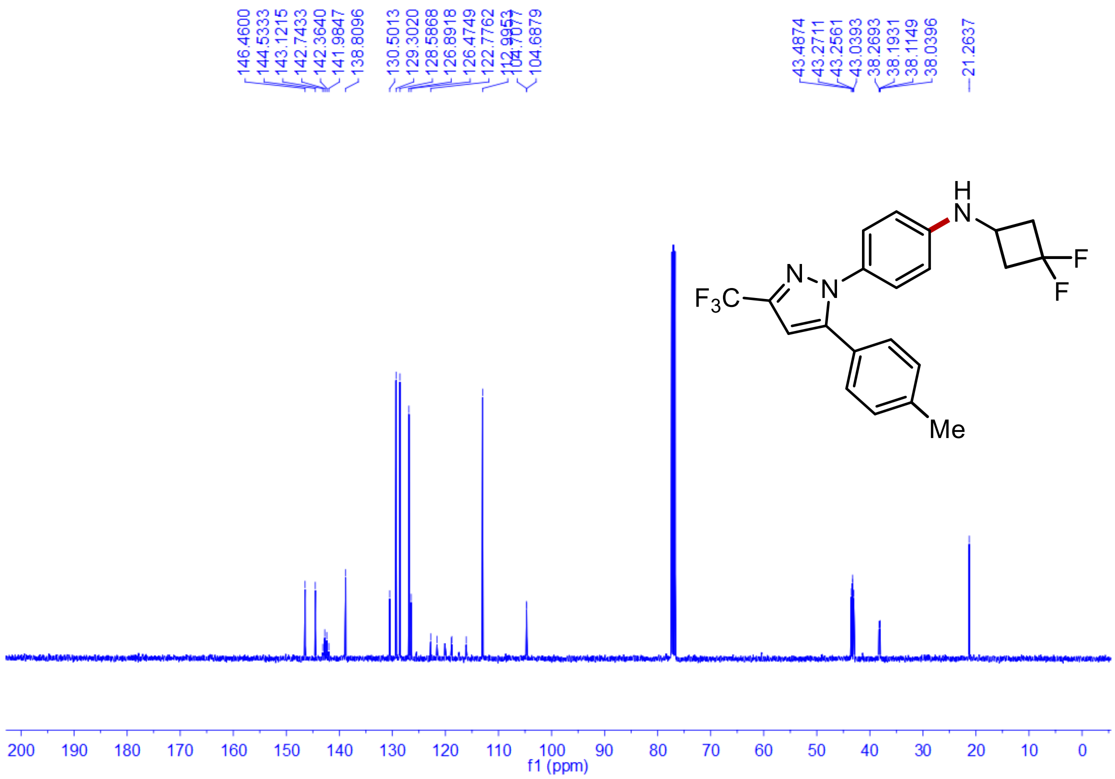

^{13}C NMR (100 MHz, $CDCl_3$) Spectrum

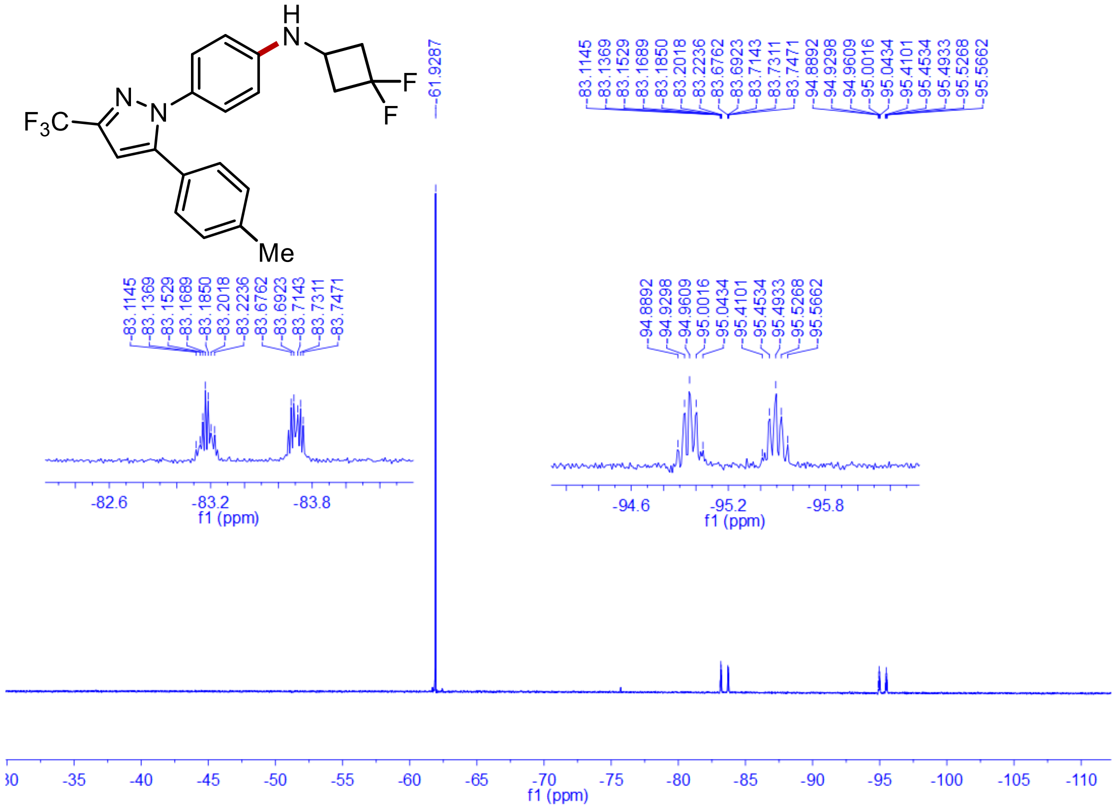

^{19}F NMR (376 MHz, $CDCl_3$) Spectrum

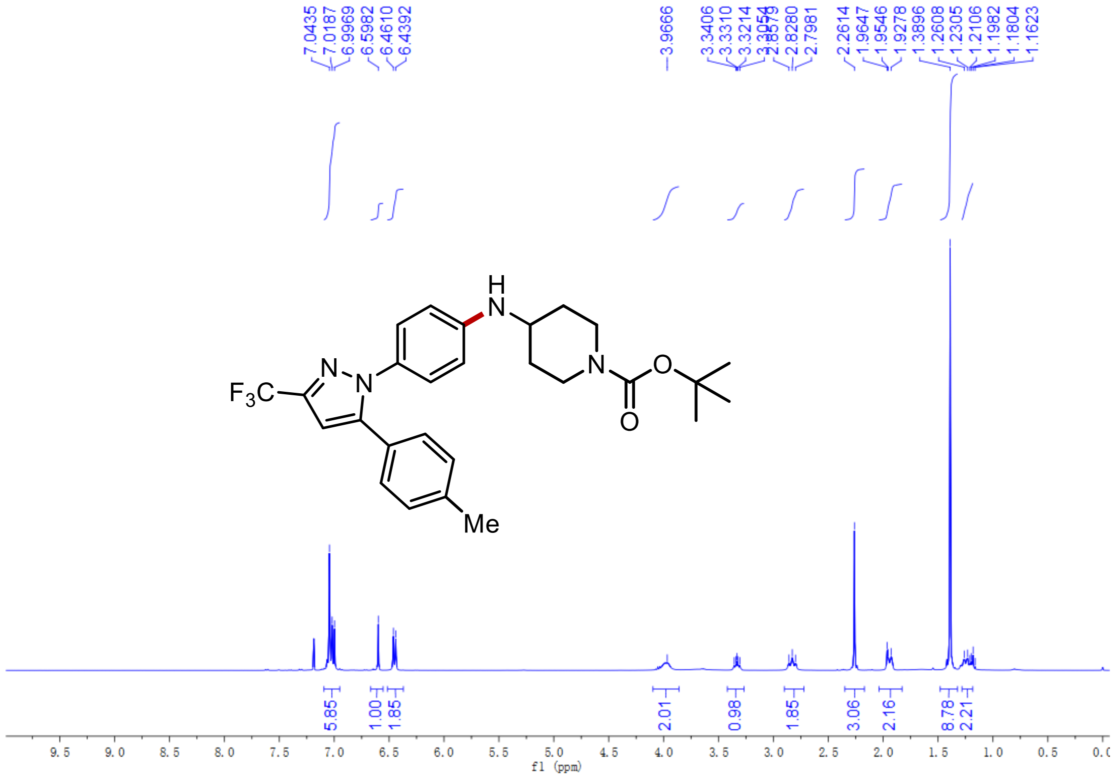

^{1}H NMR (400 MHz, $CDCl_3$) Spectrum

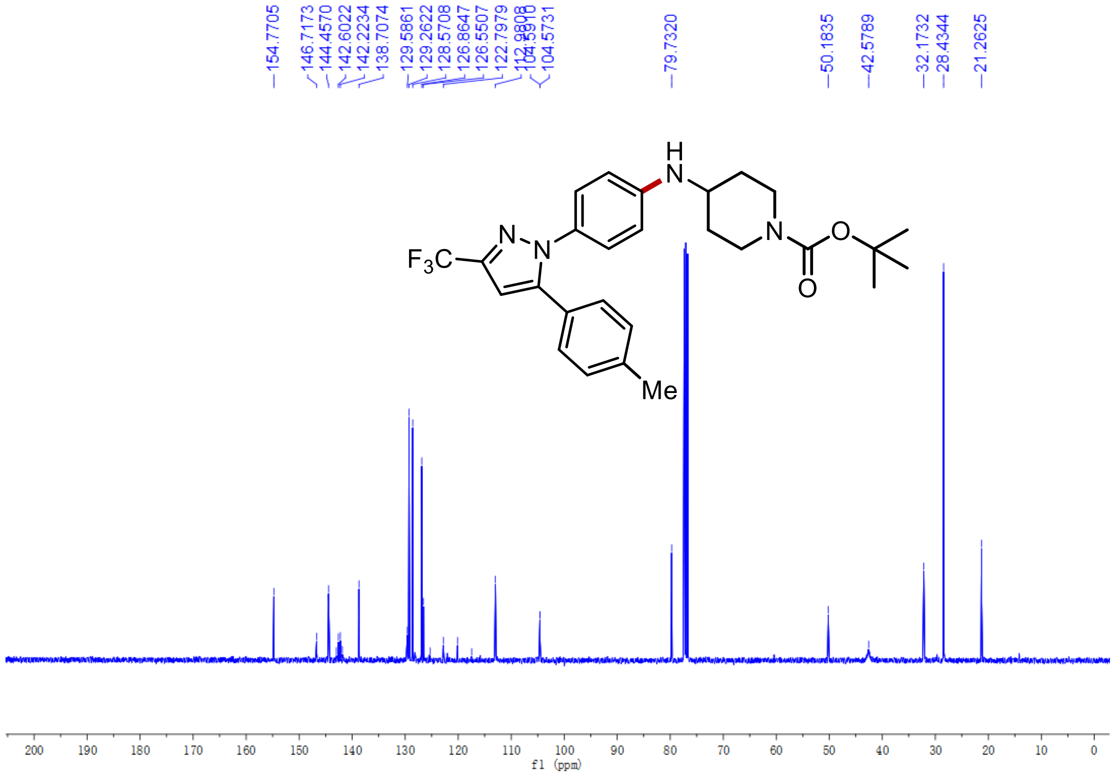

^{13}C NMR (100 MHz, $CDCl_3$) Spectrum

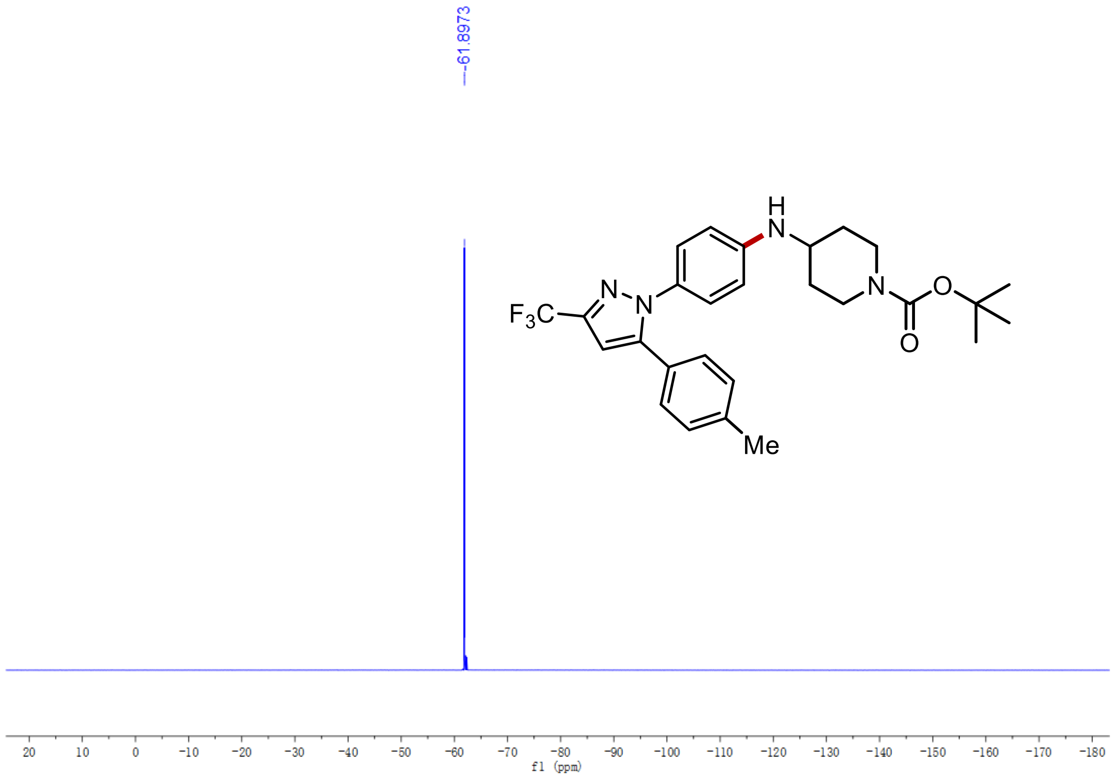

^{19}F NMR (376 MHz, CDCl₃) Spectrum

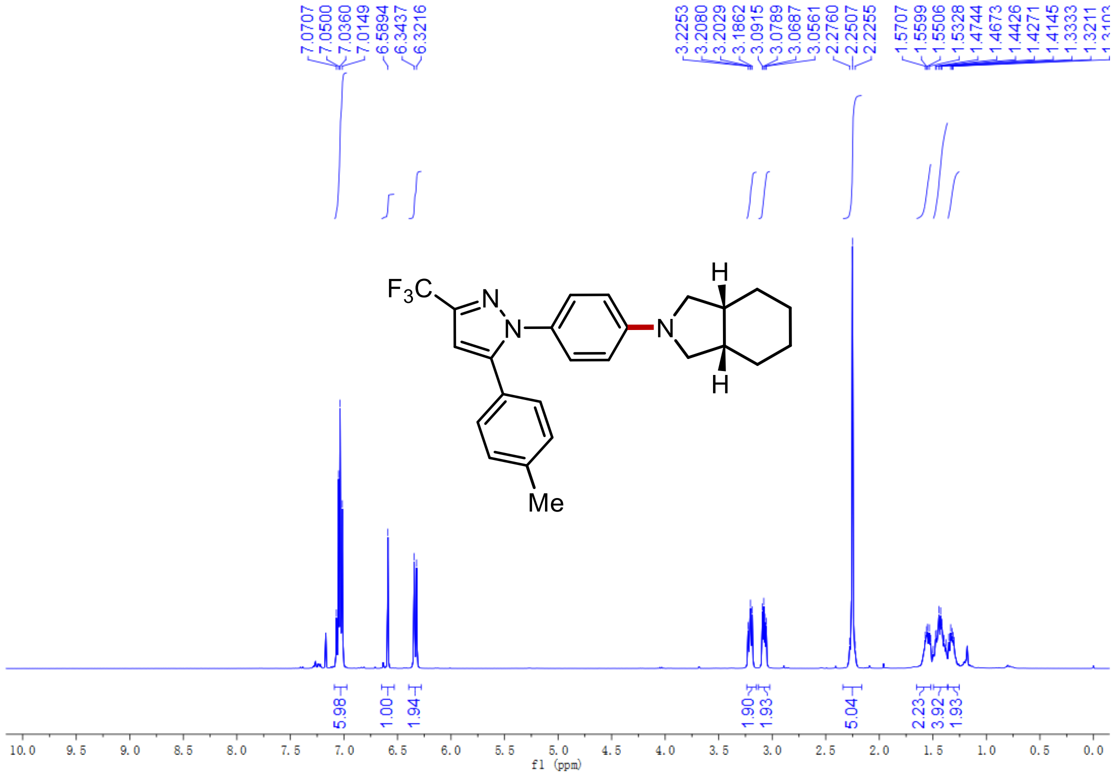

^{1}H NMR (400 MHz, CDCl₃) Spectrum

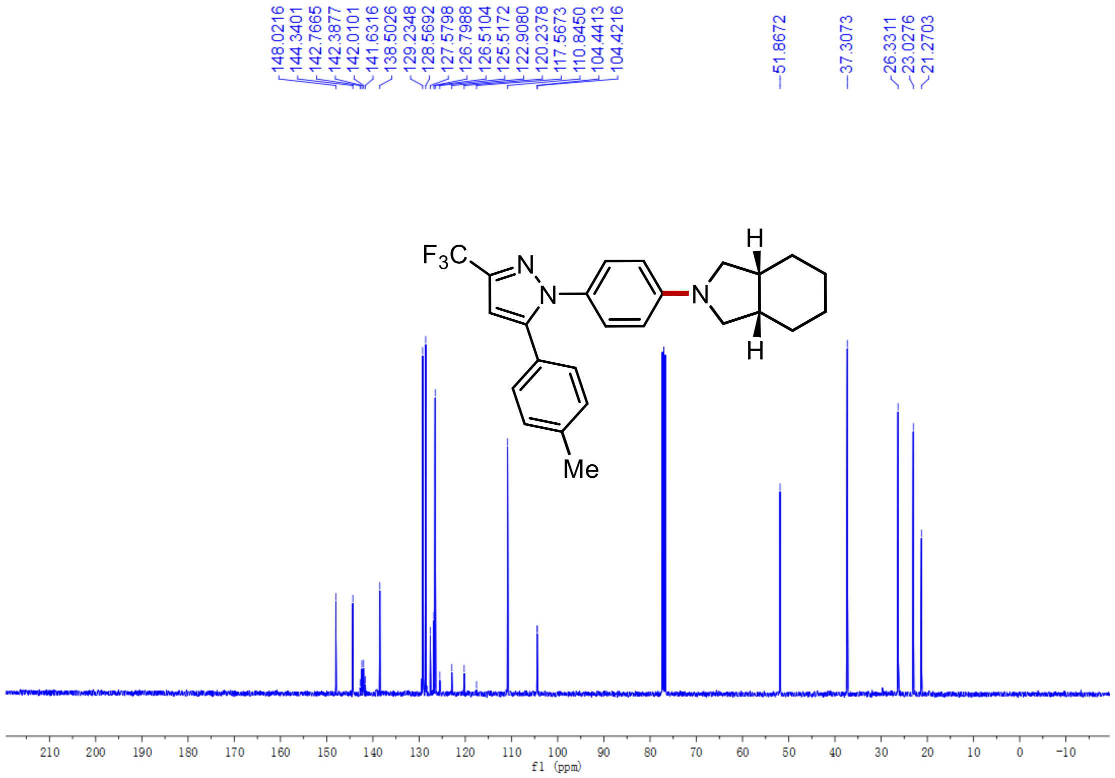

^{13}C NMR (100 MHz, $CDCl_3$) Spectrum

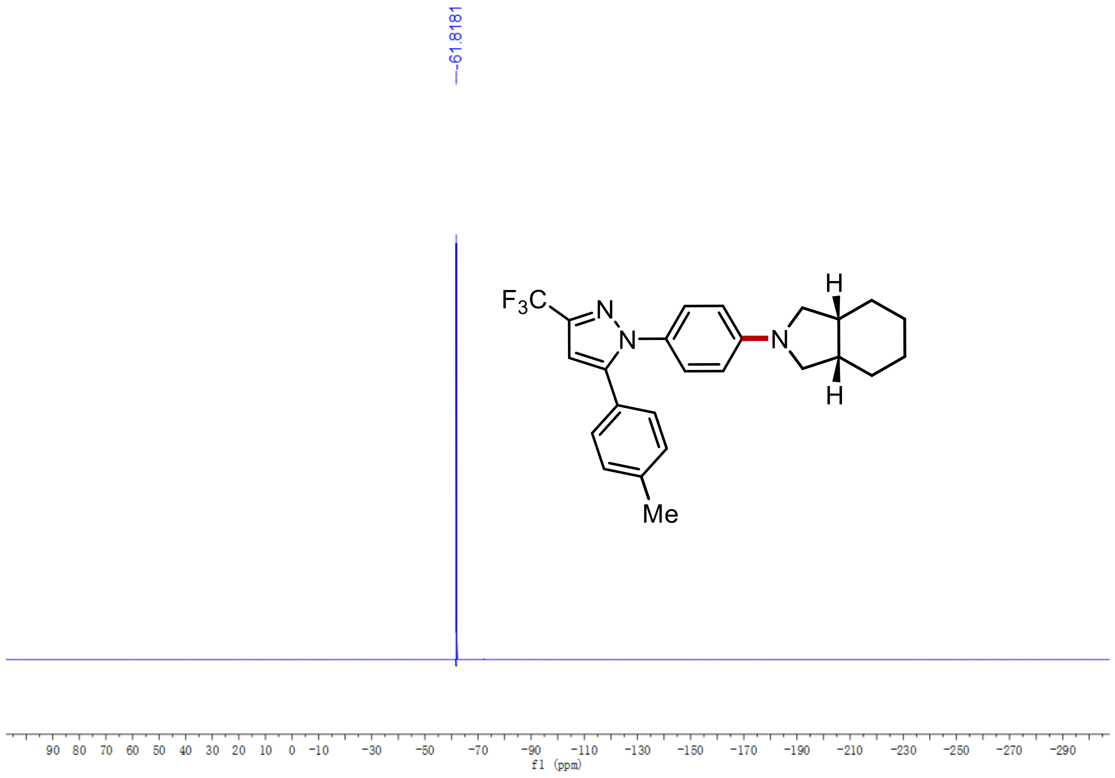

^{19}F NMR (376 MHz, $CDCl_3$) Spectrum

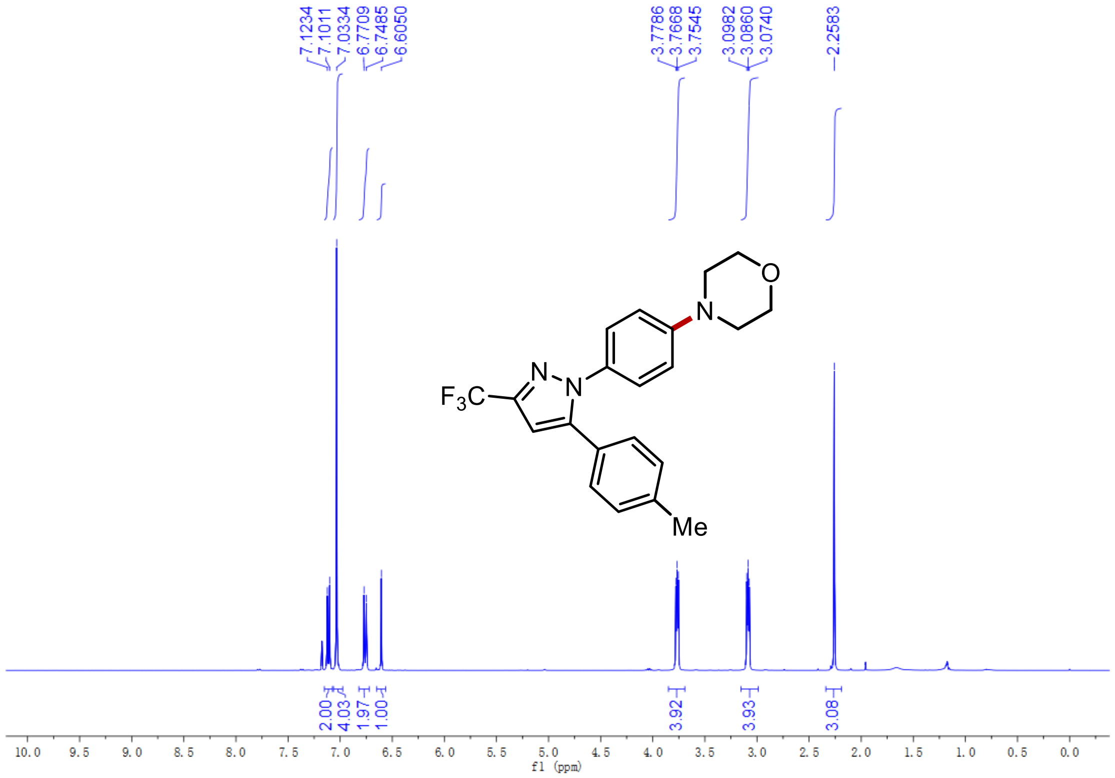

^{1}H NMR (400 MHz, $CDCl_3$) Spectrum

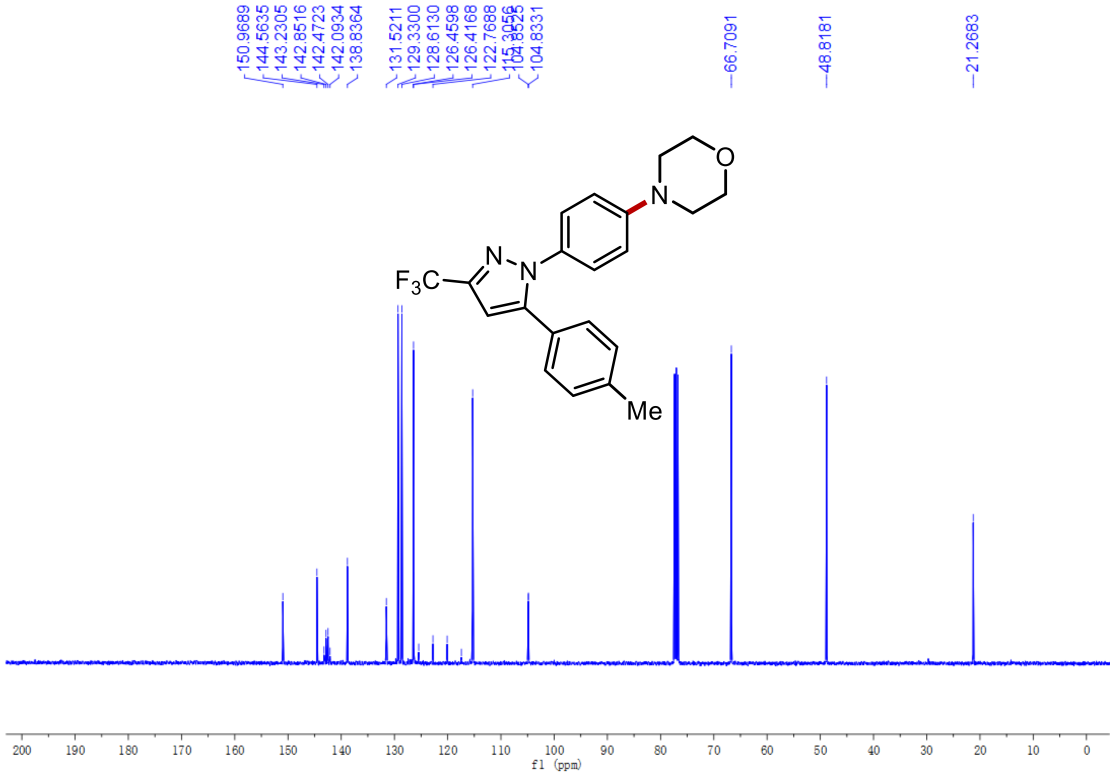

^{13}C NMR (100 MHz, $CDCl_3$) Spectrum

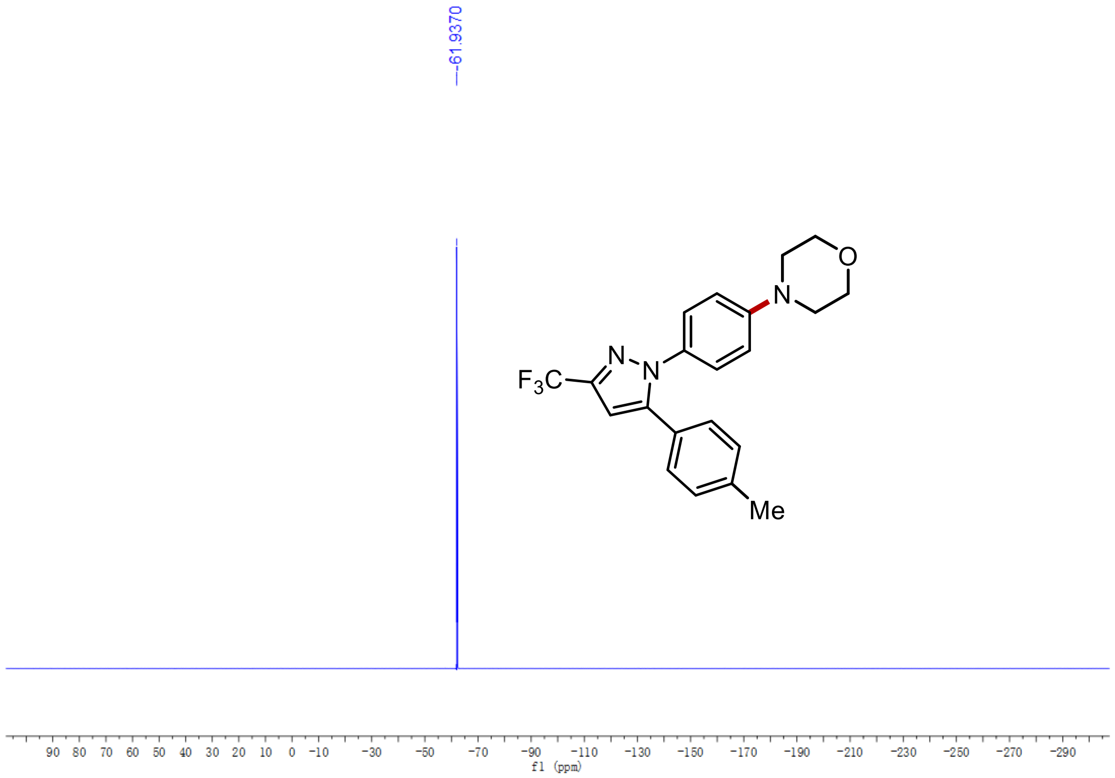

^{19}F NMR (376 MHz, $CDCl_3$) Spectrum

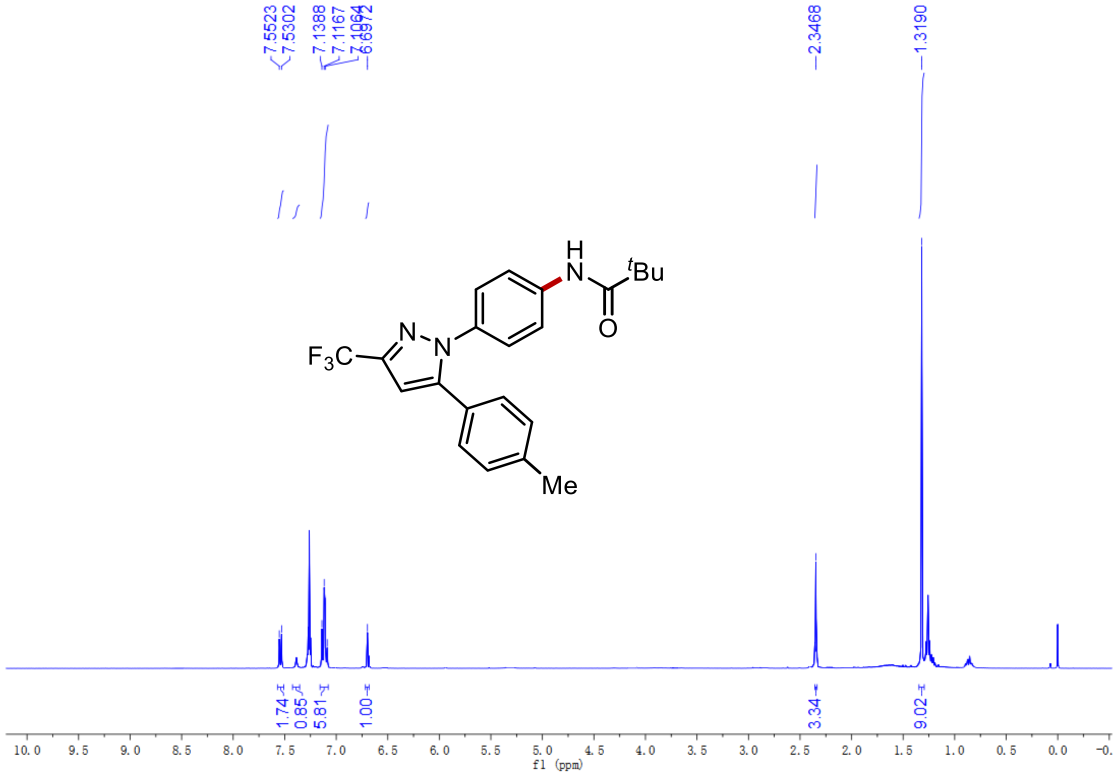

^{1}H NMR (400 MHz, $CDCl_3$) Spectrum

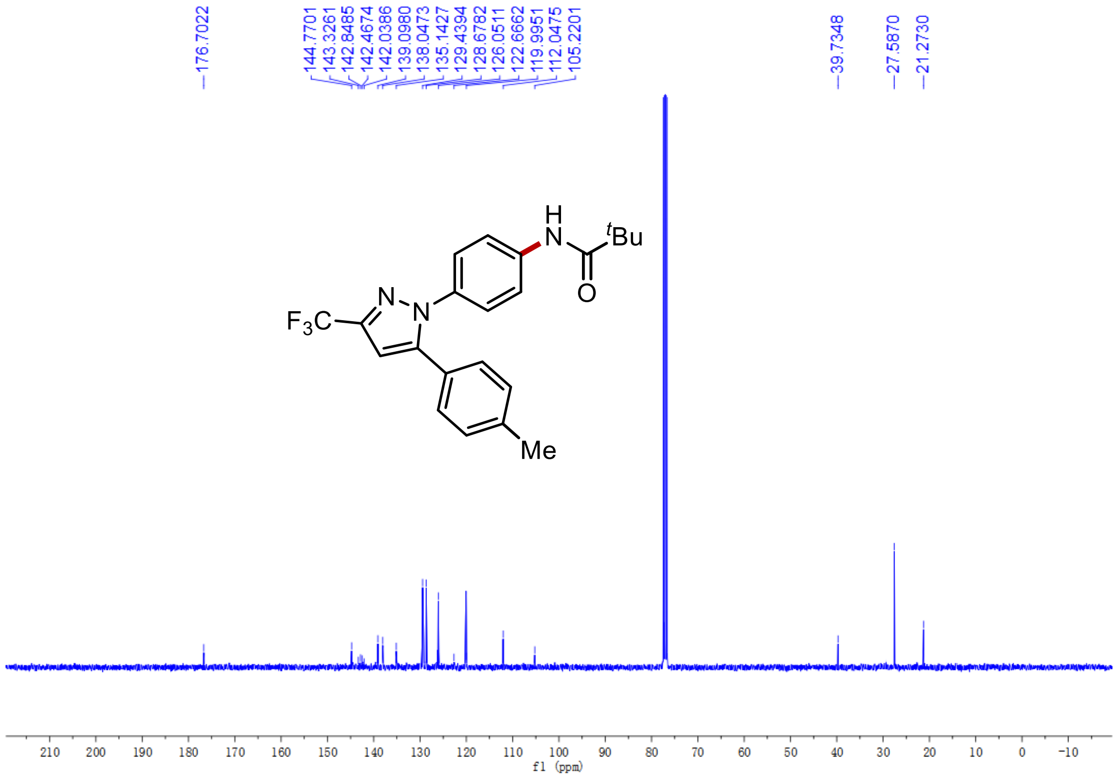

^{13}C NMR (100 MHz, $CDCl_3$) Spectrum

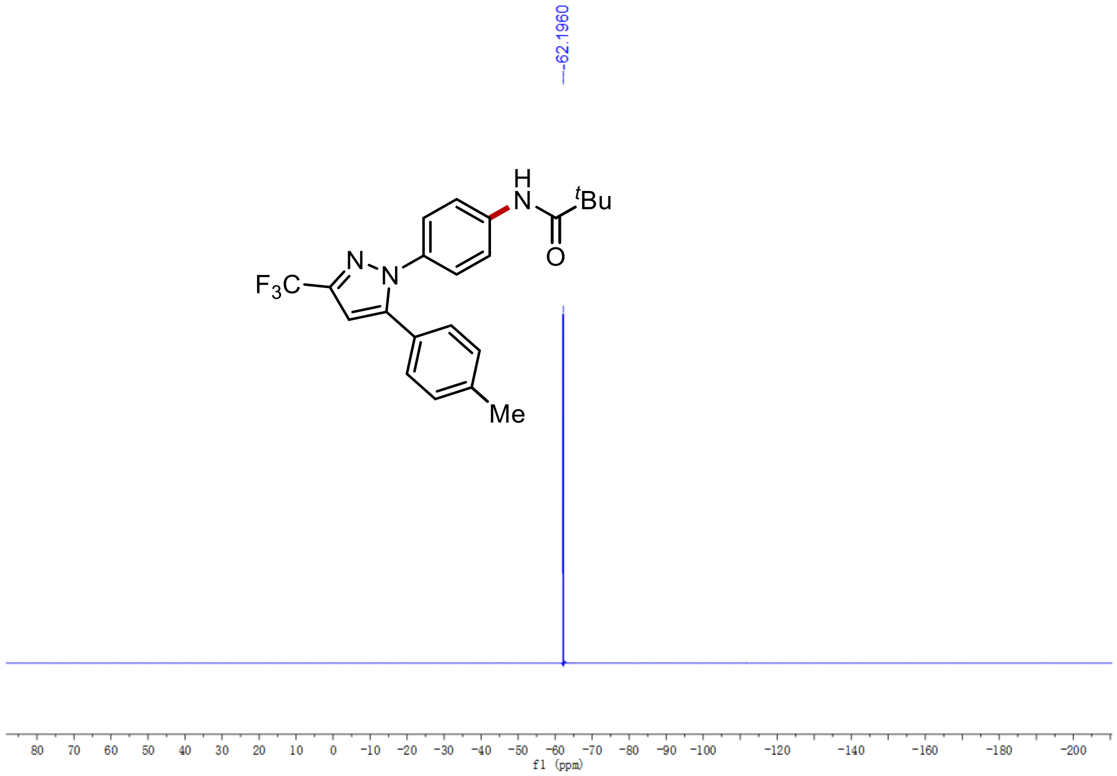

^{19}F NMR (376 MHz, $CDCl_3$) Spectrum

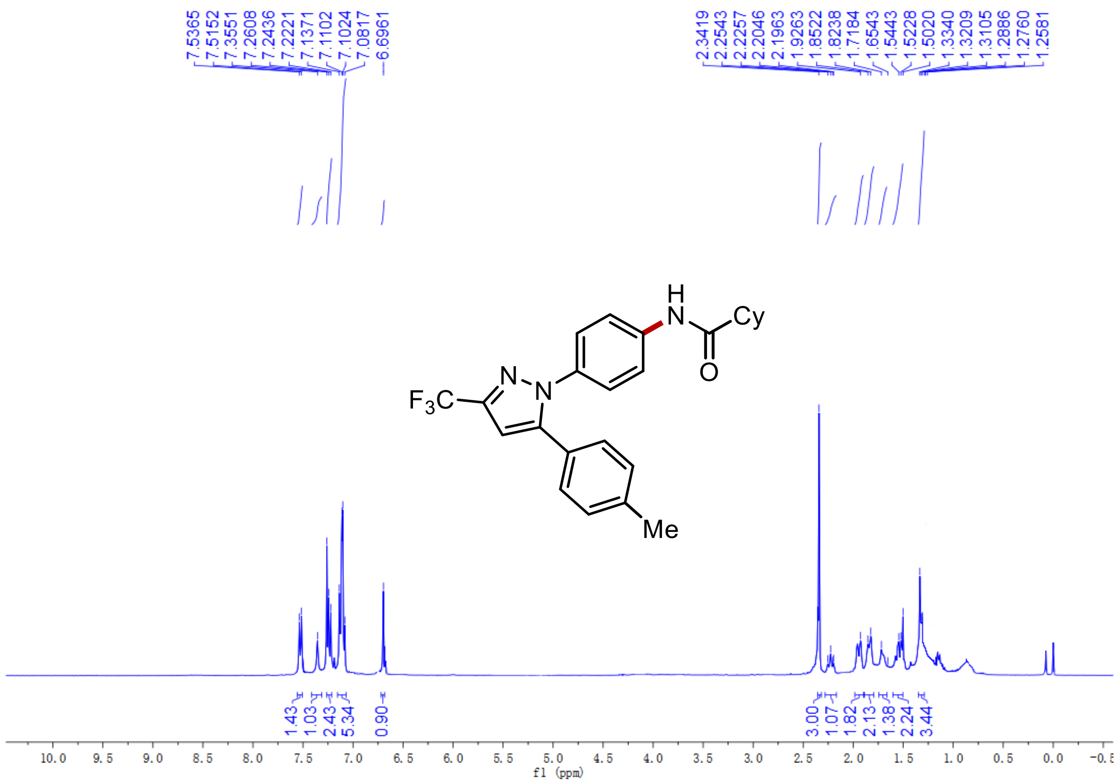

^{1}H NMR (400 MHz, $CDCl_3$) Spectrum

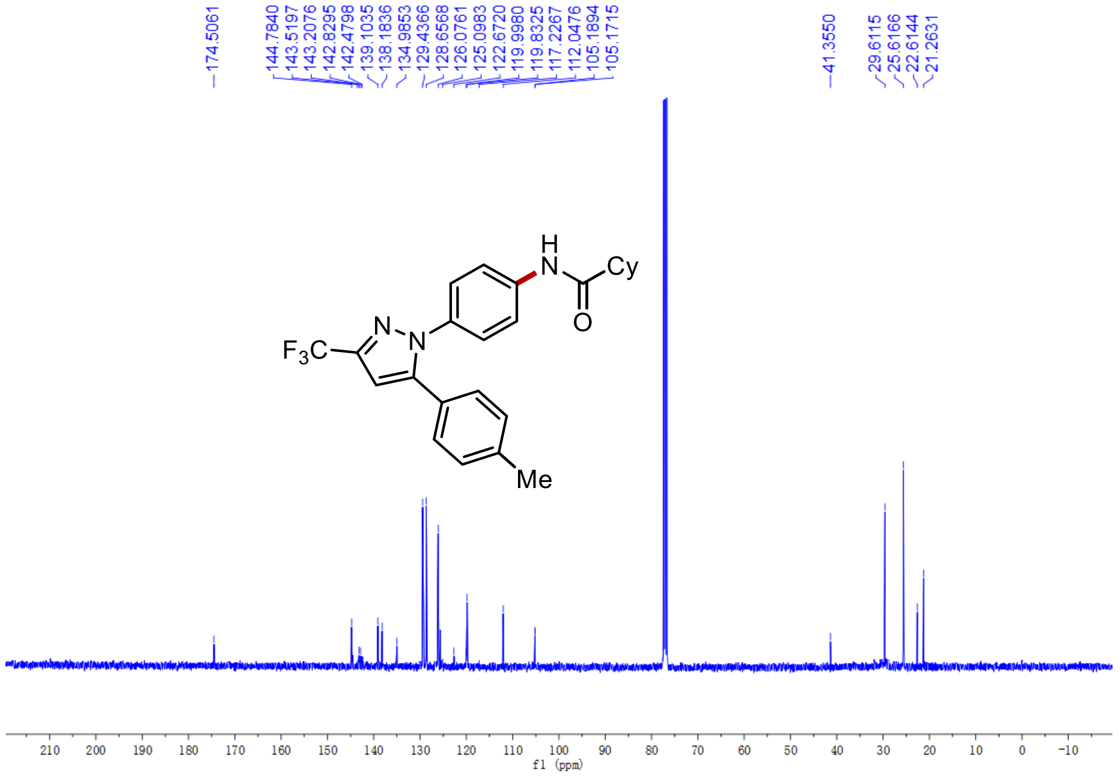

^{13}C NMR (100 MHz, $CDCl_3$) Spectrum

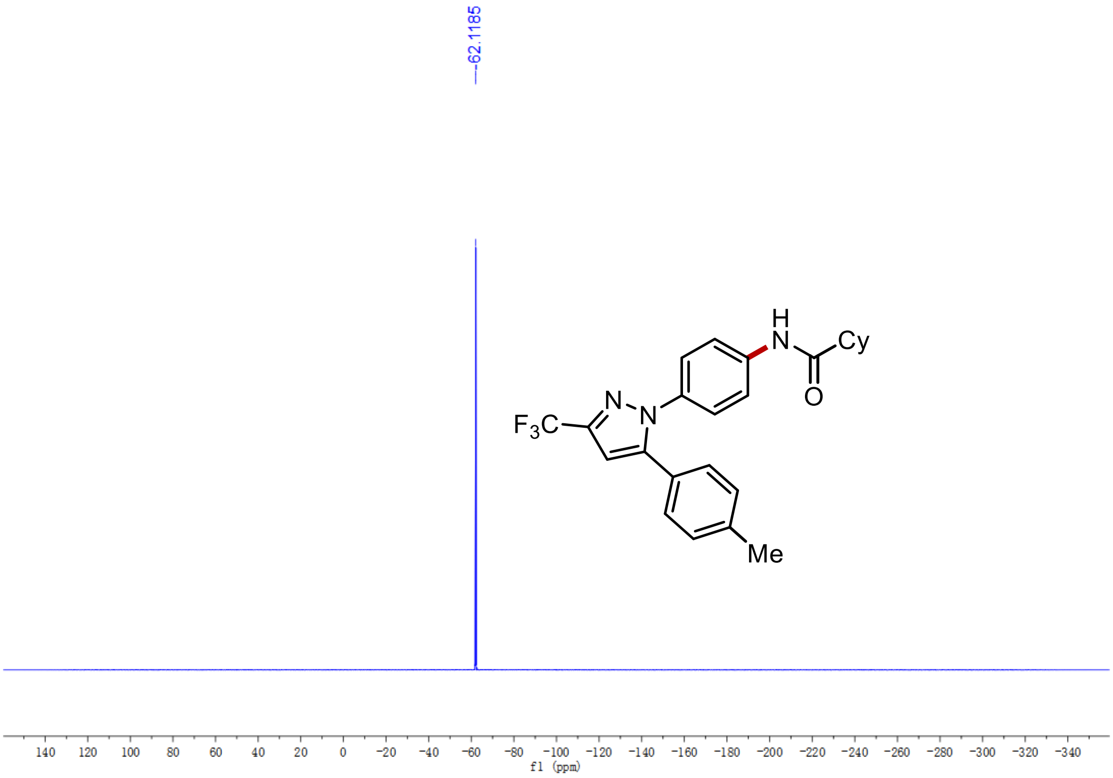

^{19}F NMR (376 MHz, CDCl3) Spectrum

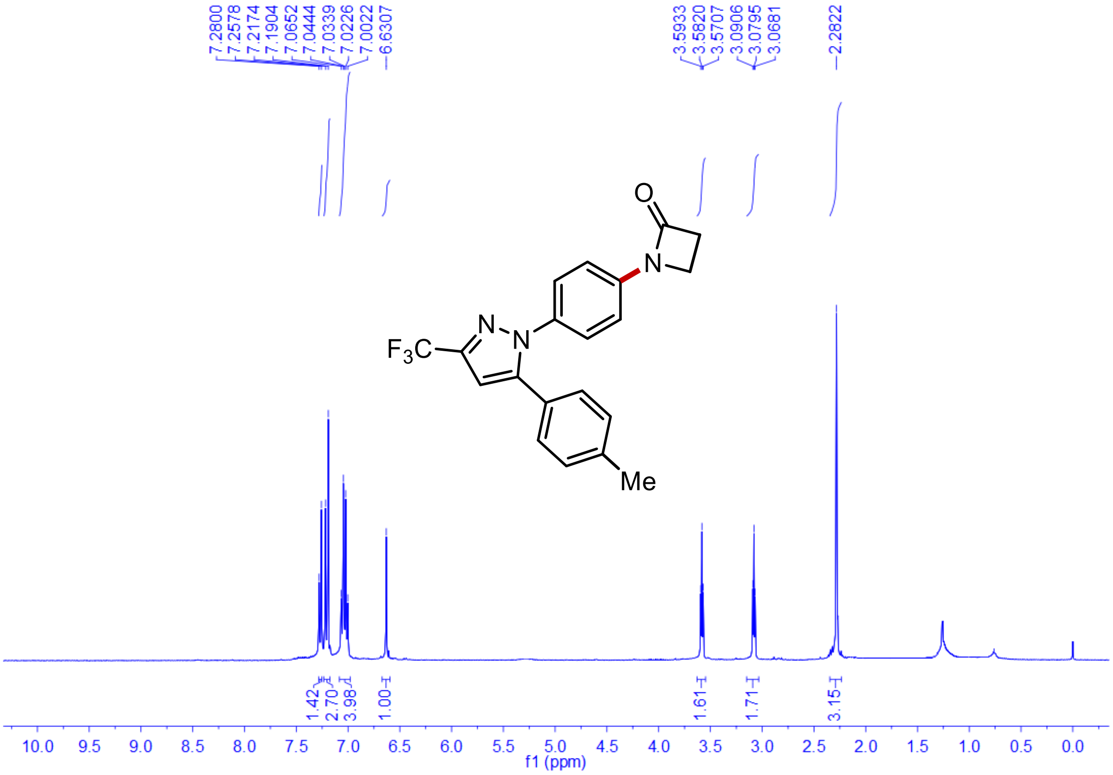

^{1}H NMR (400 MHz, CDCl3) Spectrum

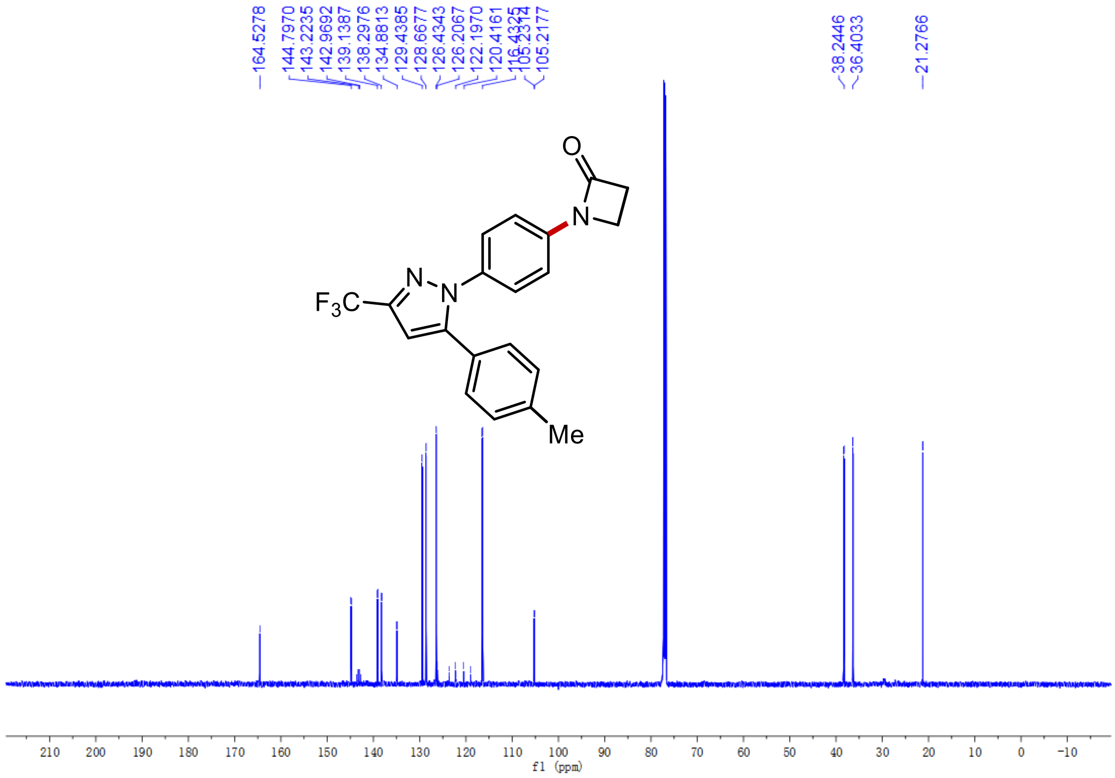

^{13}C NMR (150 MHz, $CDCl_3$) Spectrum

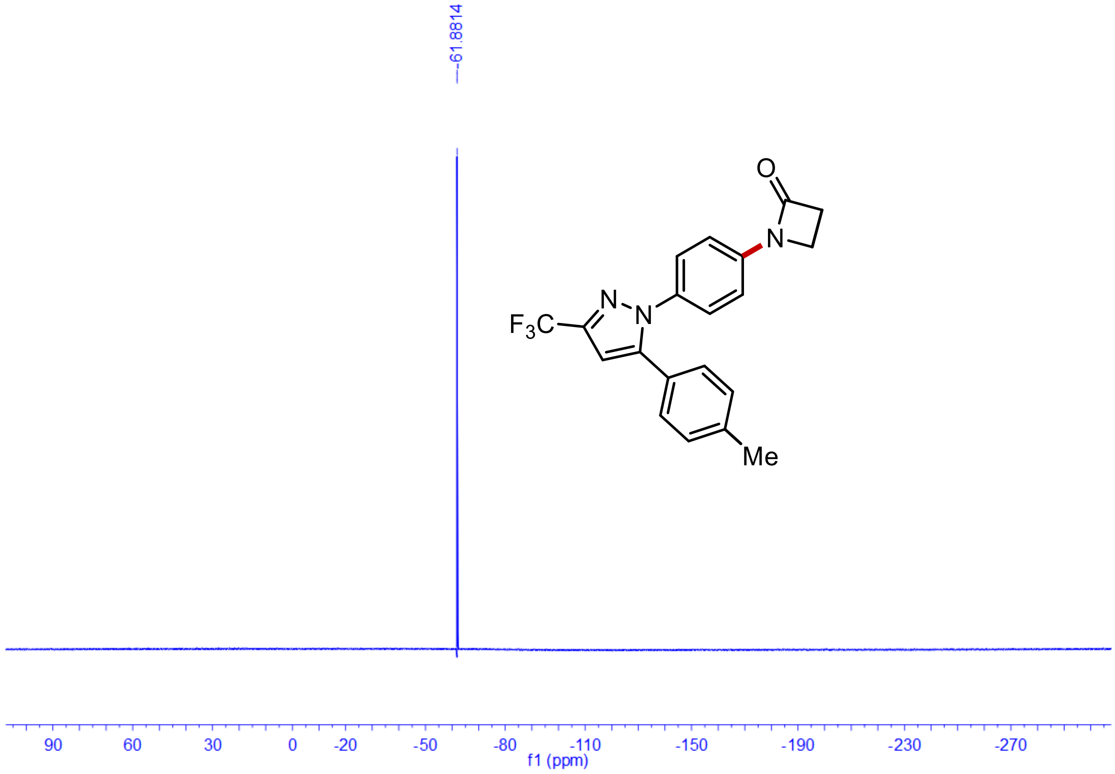

^{19}F NMR (376 MHz, $CDCl_3$) Spectrum

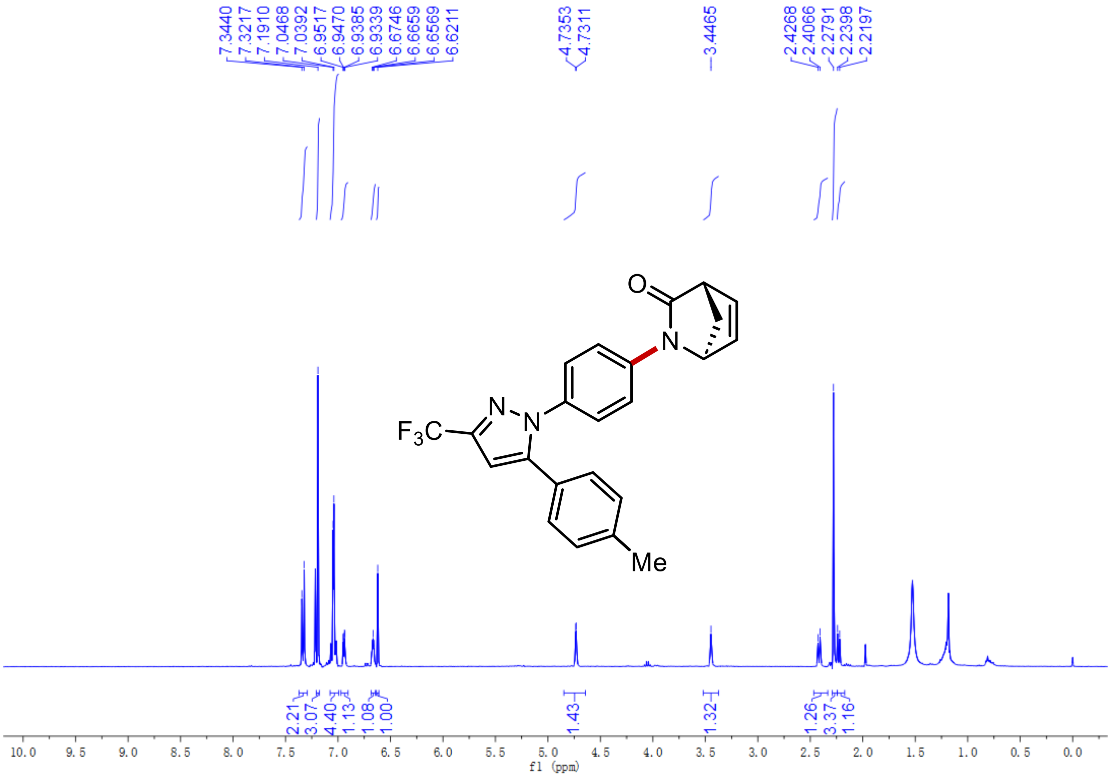

^{1}H NMR (400 MHz, $CDCl_3$) Spectrum

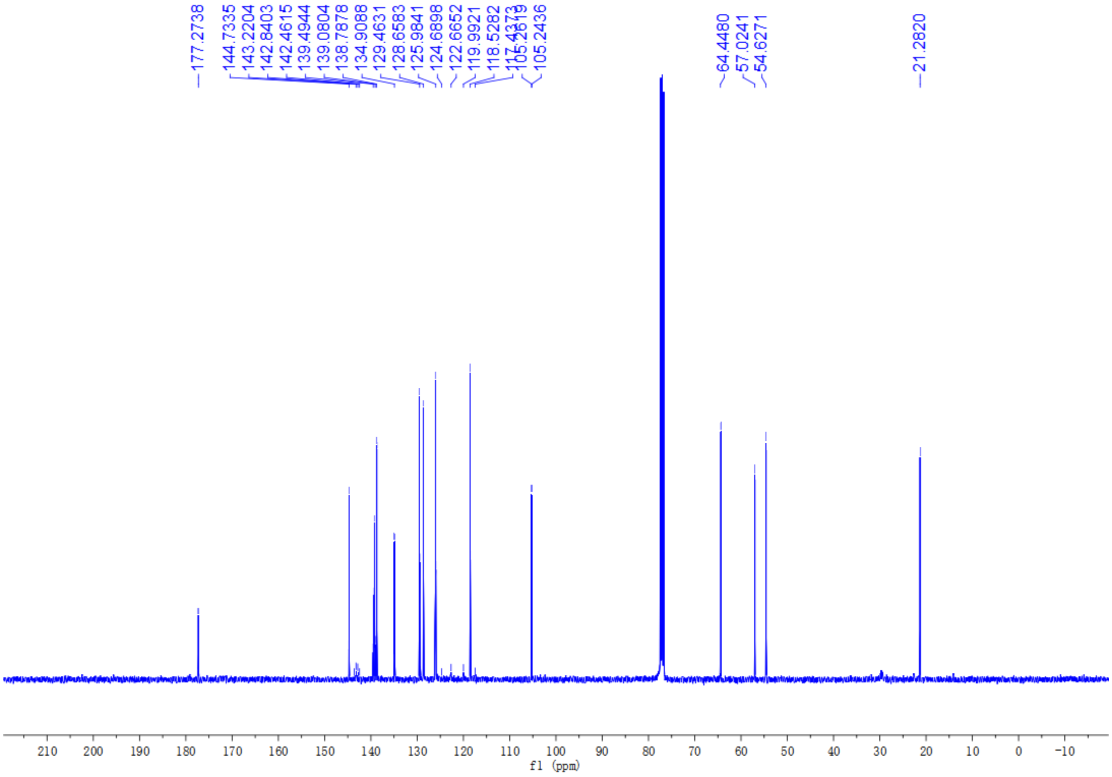

^{13}C NMR (100 MHz, $CDCl_3$) Spectrum

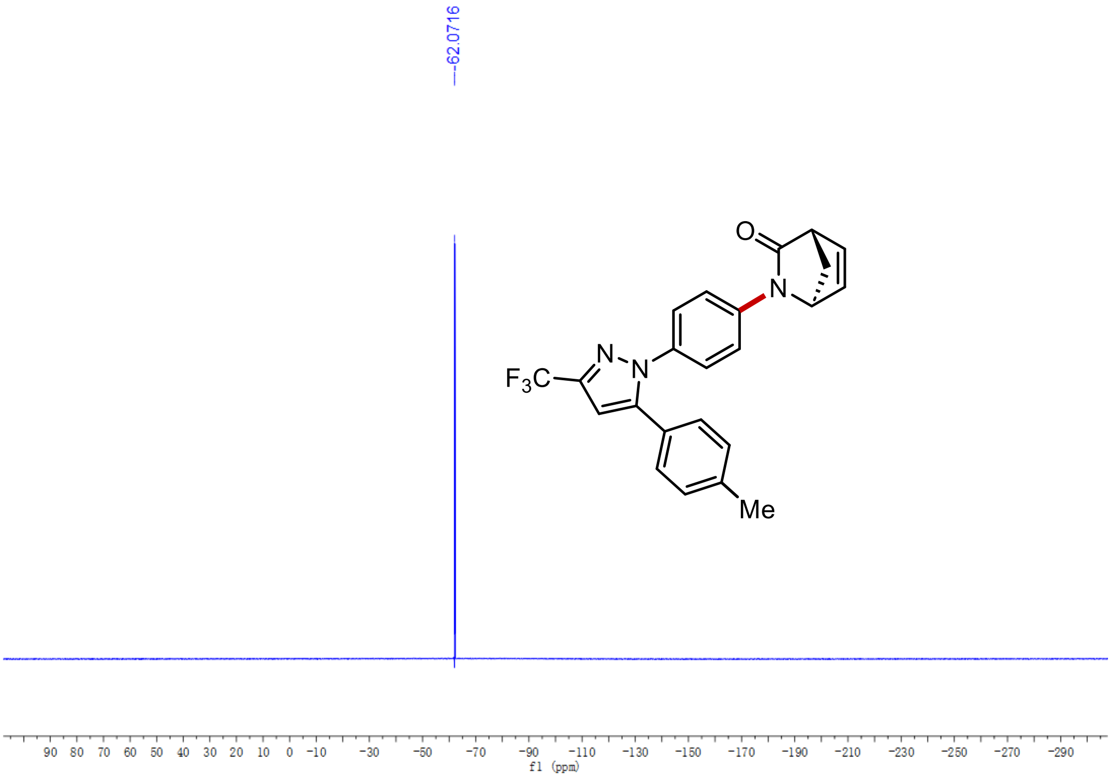

^{19}F NMR (376 MHz, $CDCl_3$) Spectrum

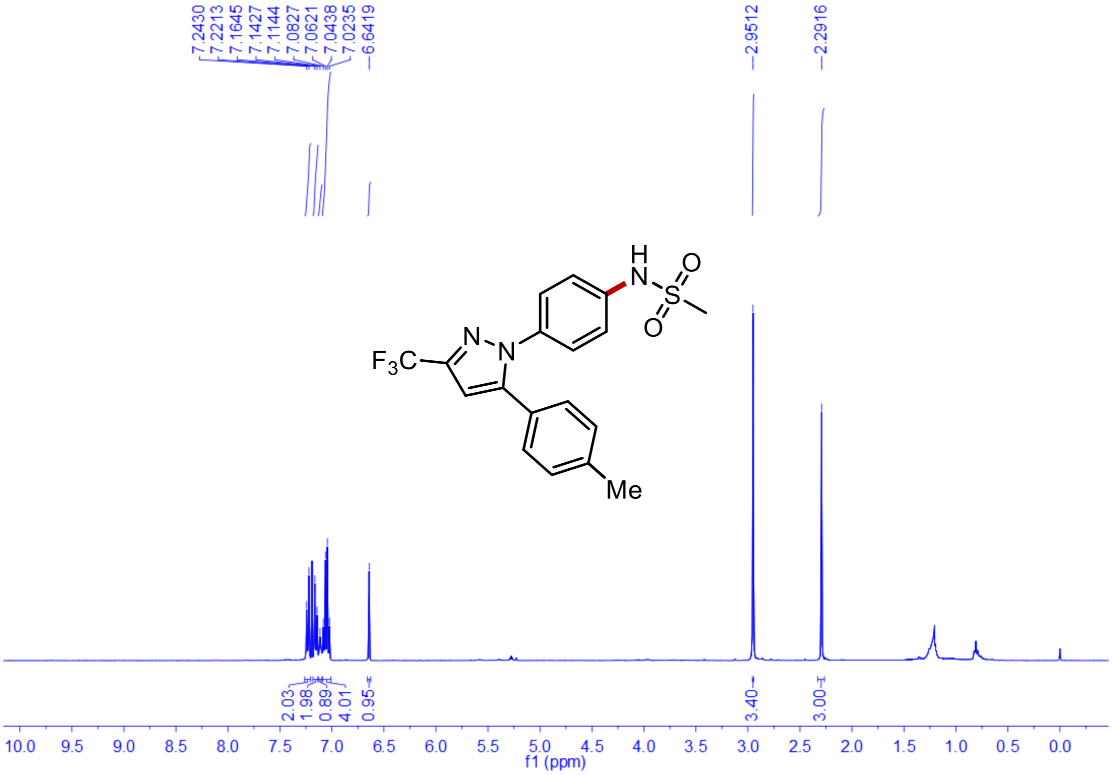

^{1}H NMR (400 MHz, $CDCl_3$) Spectrum

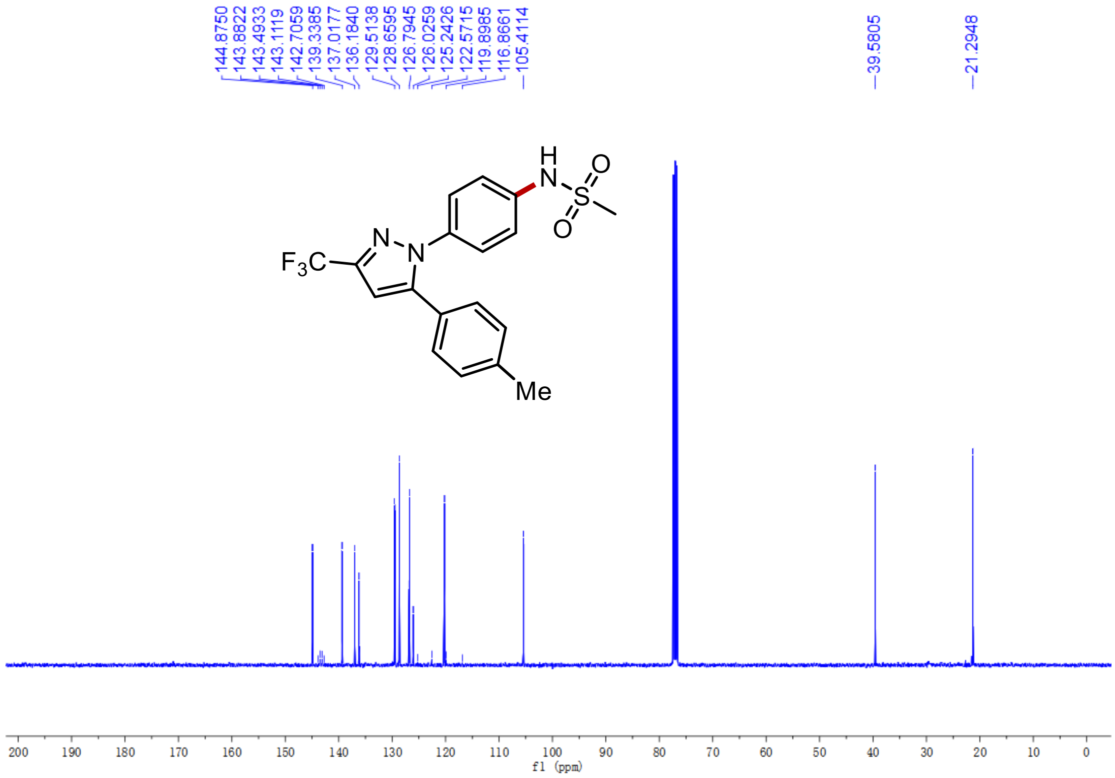

^{13}C NMR (100 MHz, $CDCl_3$) Spectrum

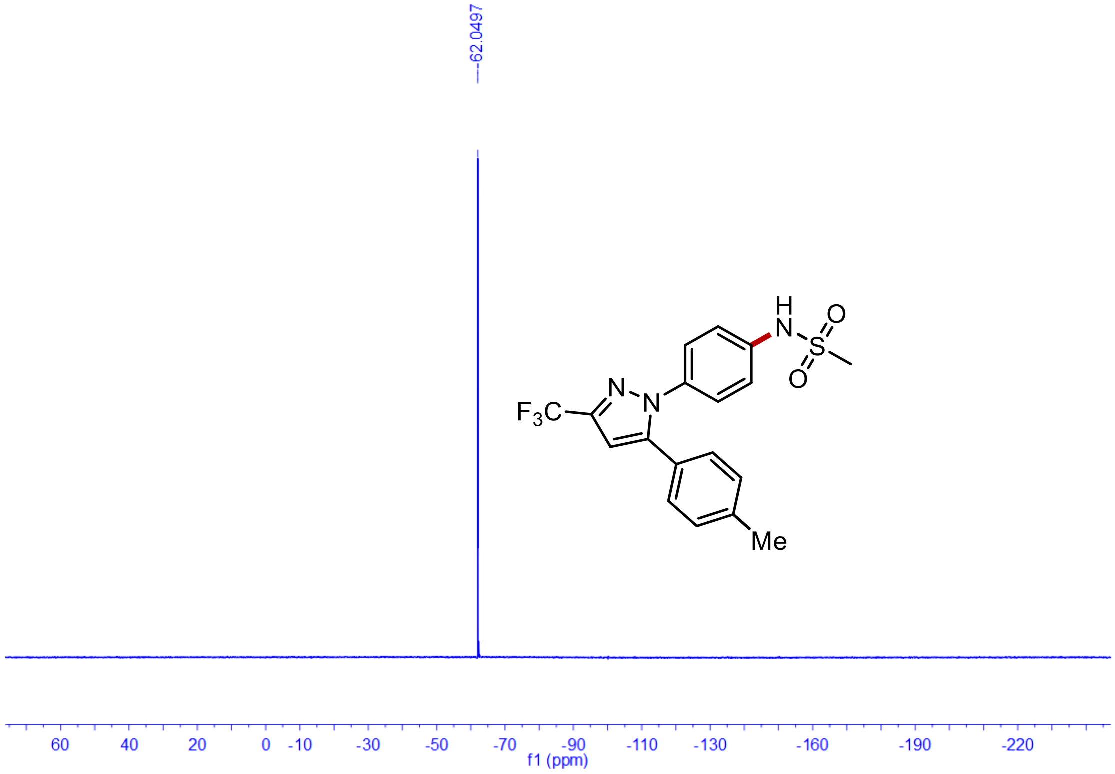

^{19}F NMR (376 MHz, $CDCl_3$) Spectrum

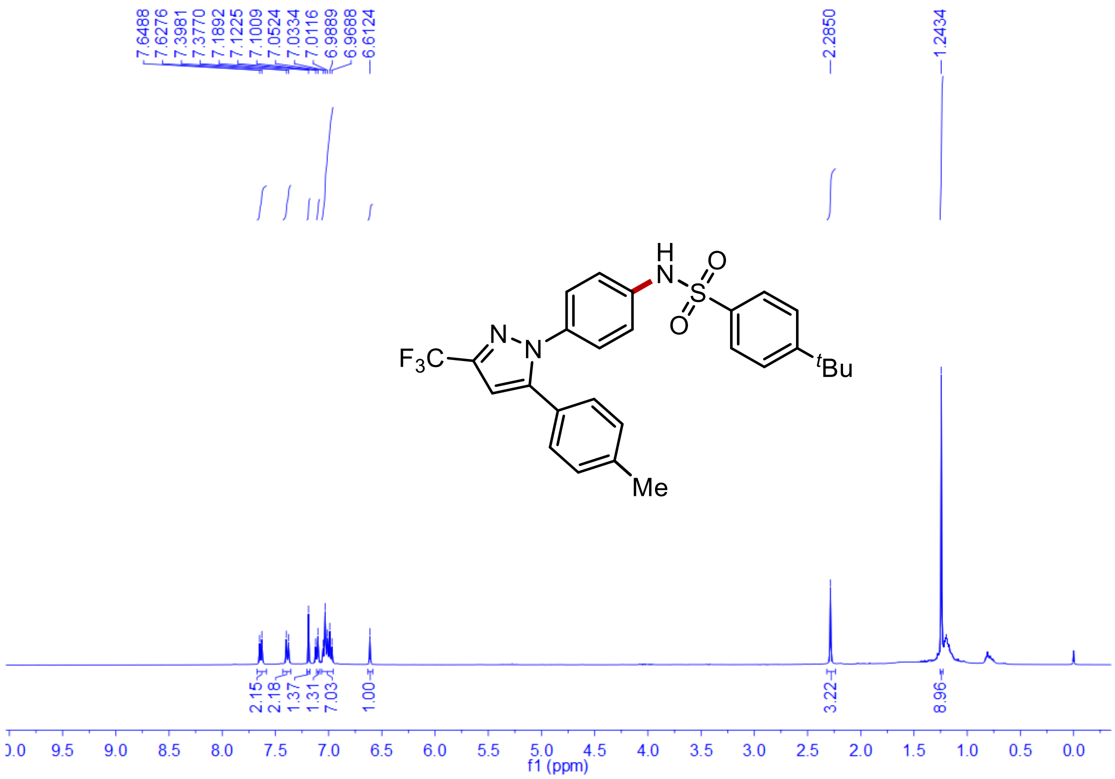

^{1}H NMR (400 MHz, $CDCl_3$) Spectrum

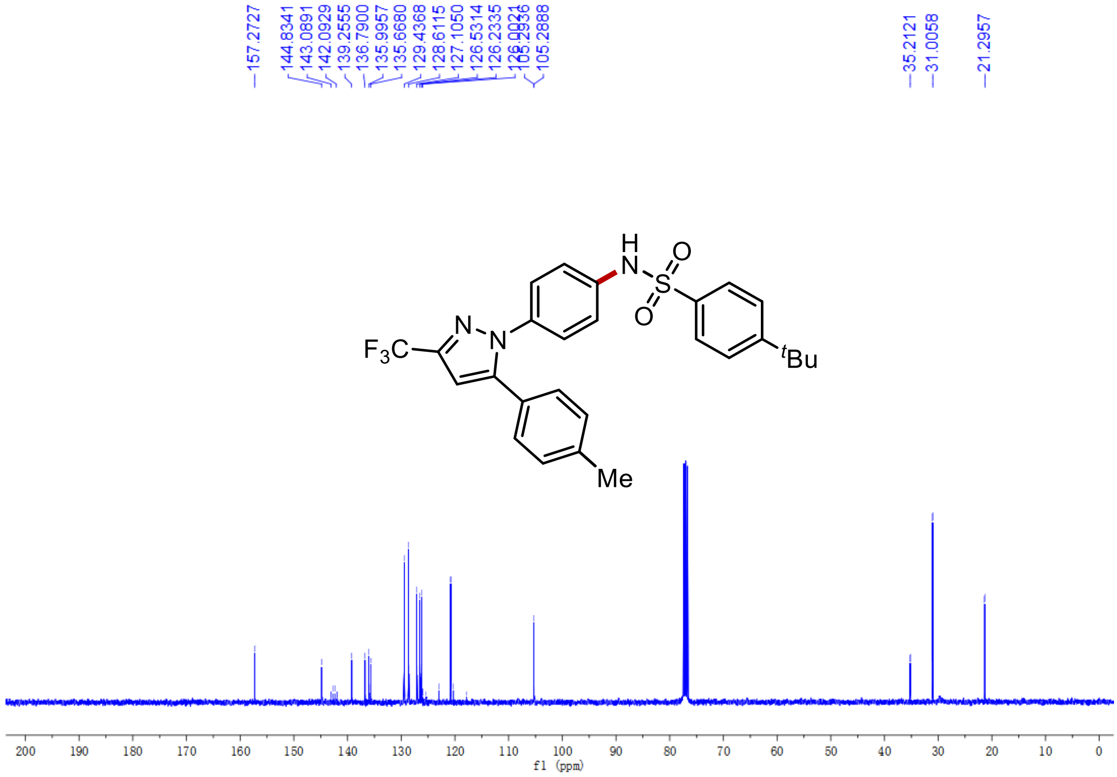

^{13}C NMR (100 MHz, $CDCl_3$) Spectrum

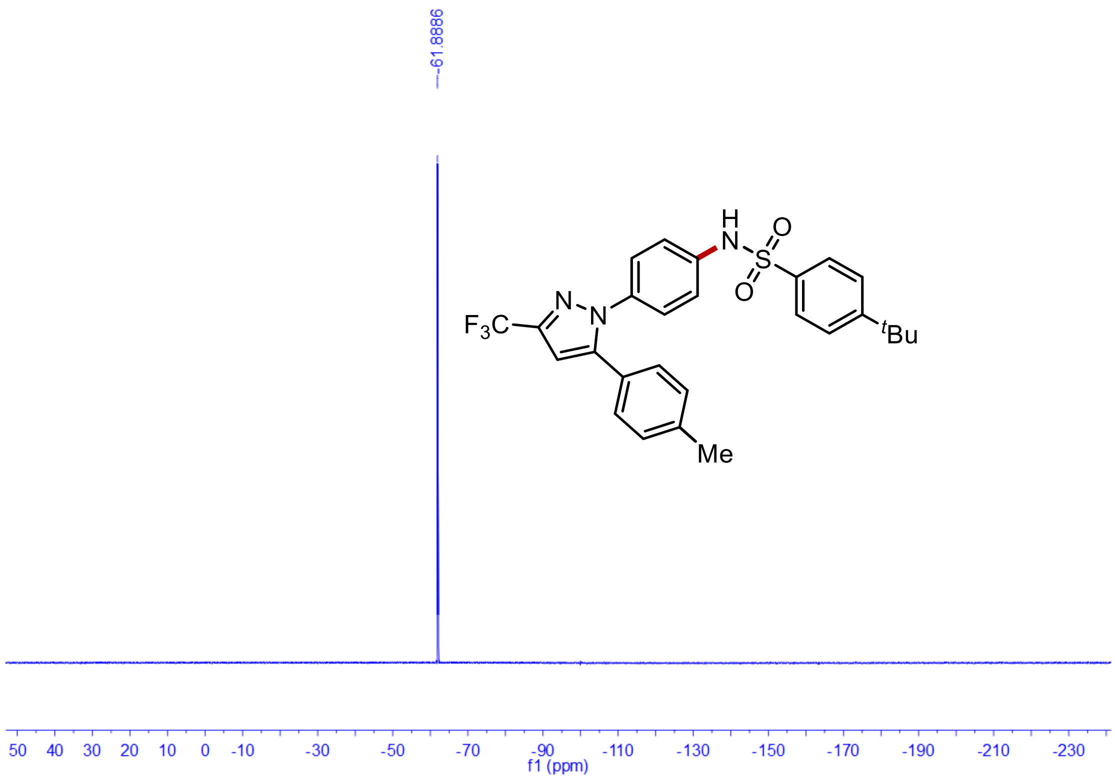

^{19}F NMR (376 MHz, $CDCl_3$) Spectrum

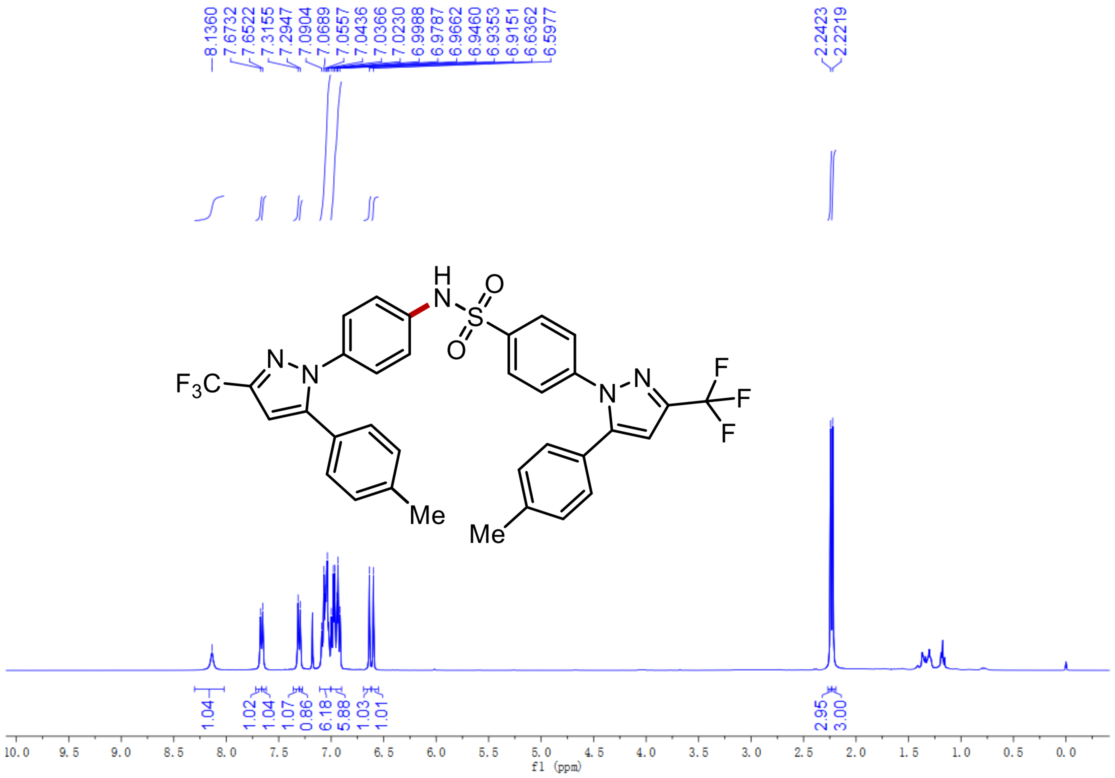

^{1}H NMR (400 MHz, $CDCl_3$) Spectrum

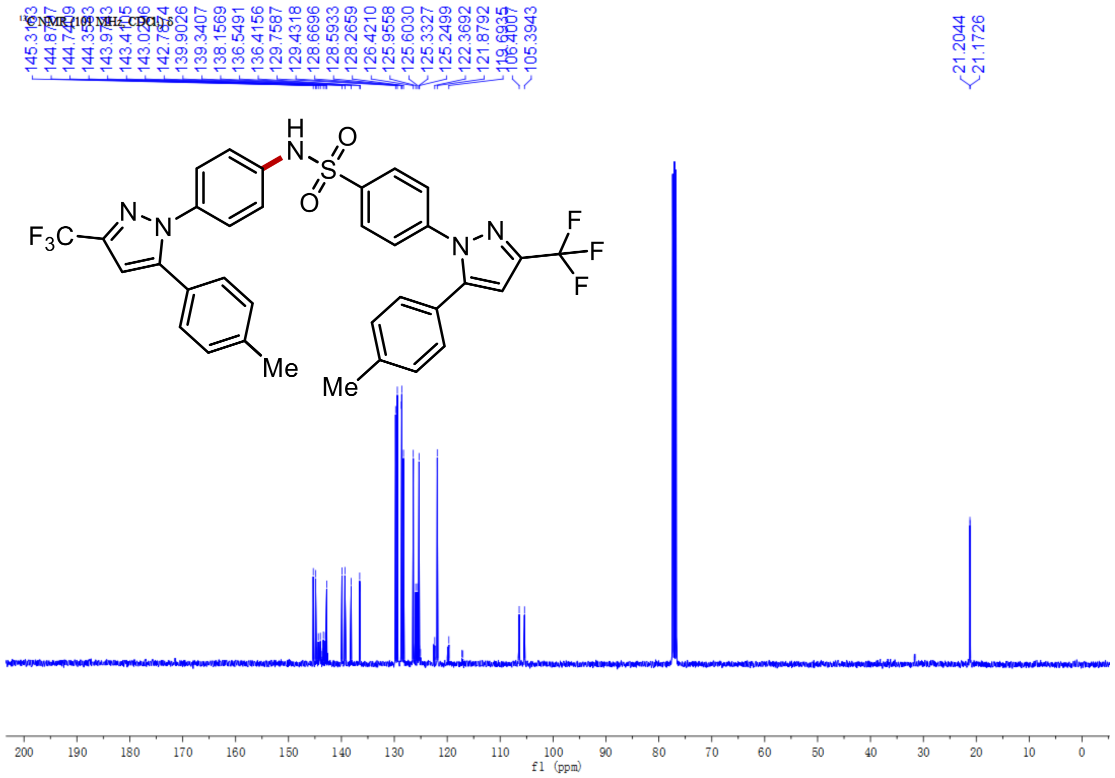

^{13}C NMR (100 MHz, $CDCl_3$) Spectrum

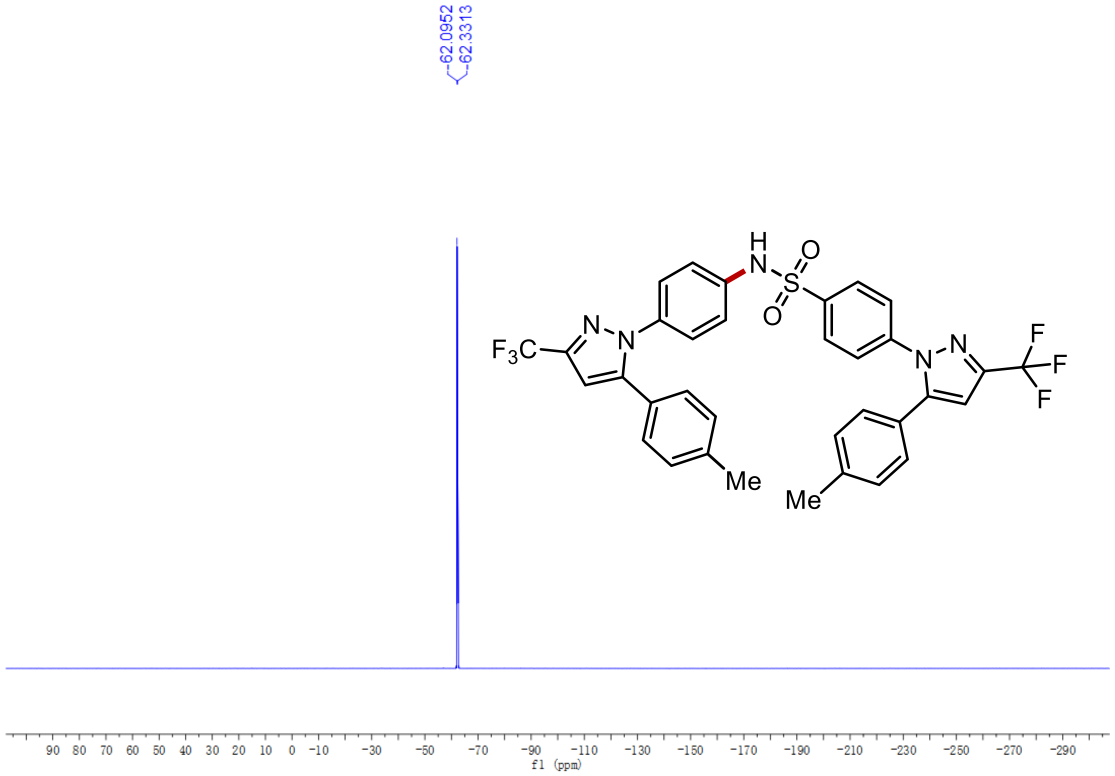

^{19}F NMR (376 MHz, $CDCl_3$) Spectrum

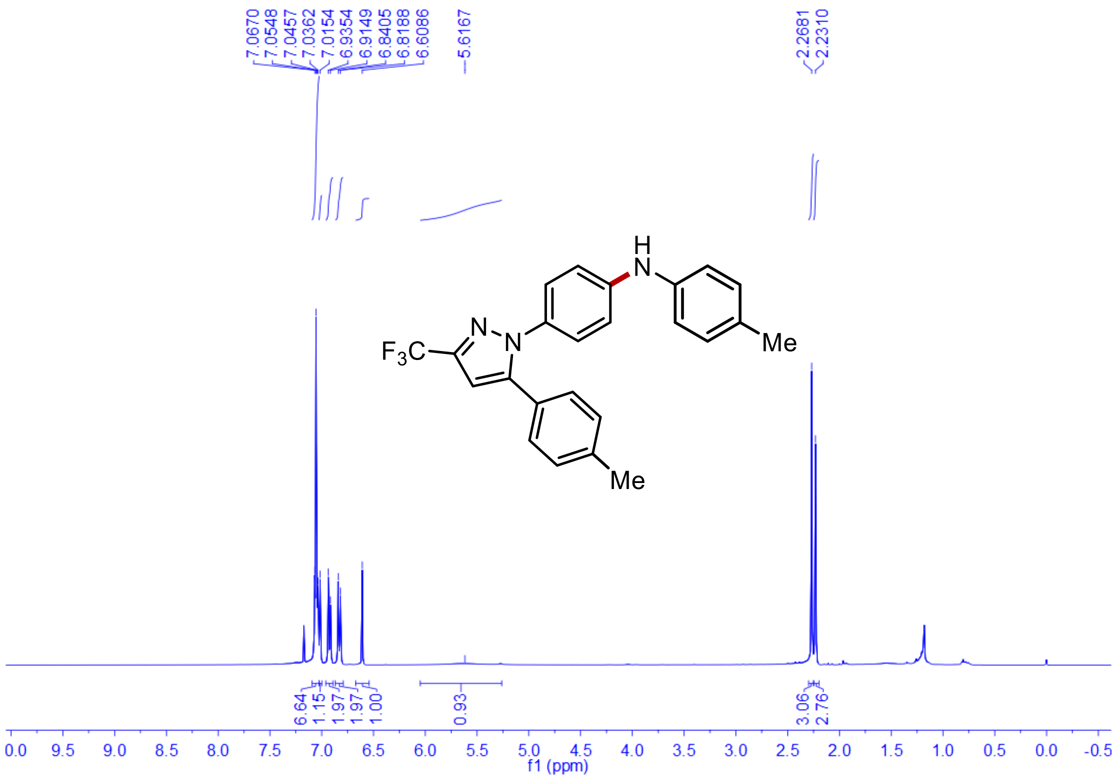

^{1}H NMR (400 MHz, $CDCl_3$) Spectrum

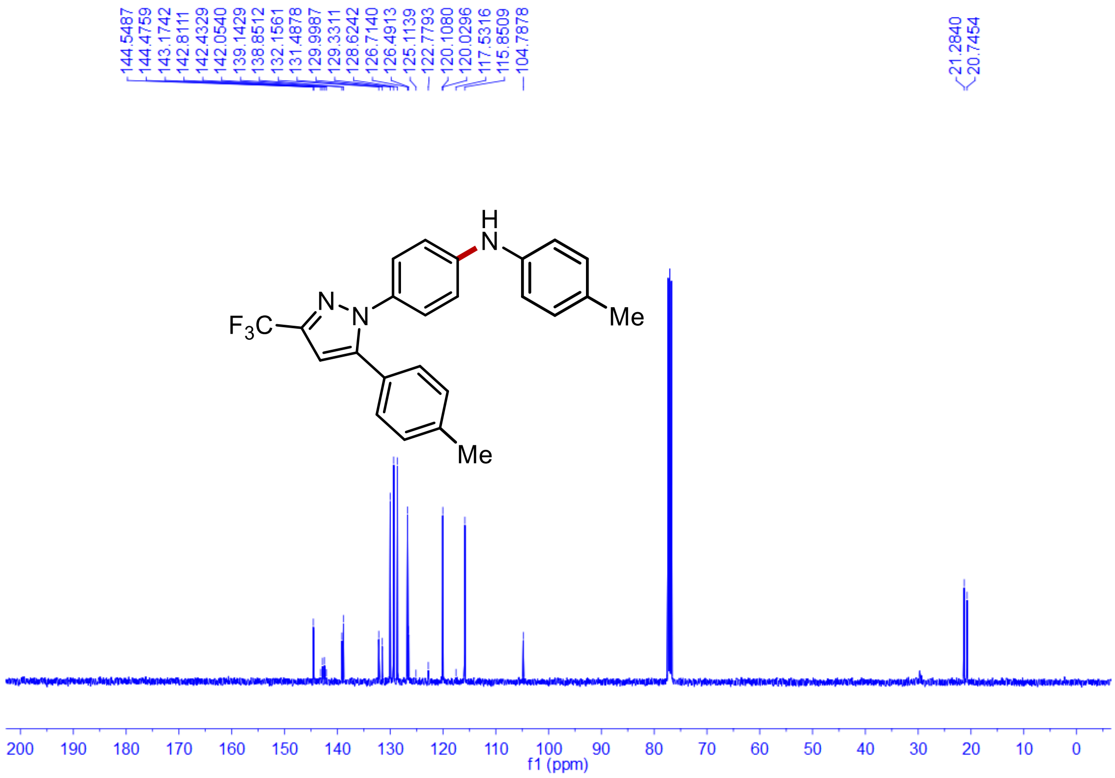

^{13}C NMR (100 MHz, $CDCl_3$) Spectrum

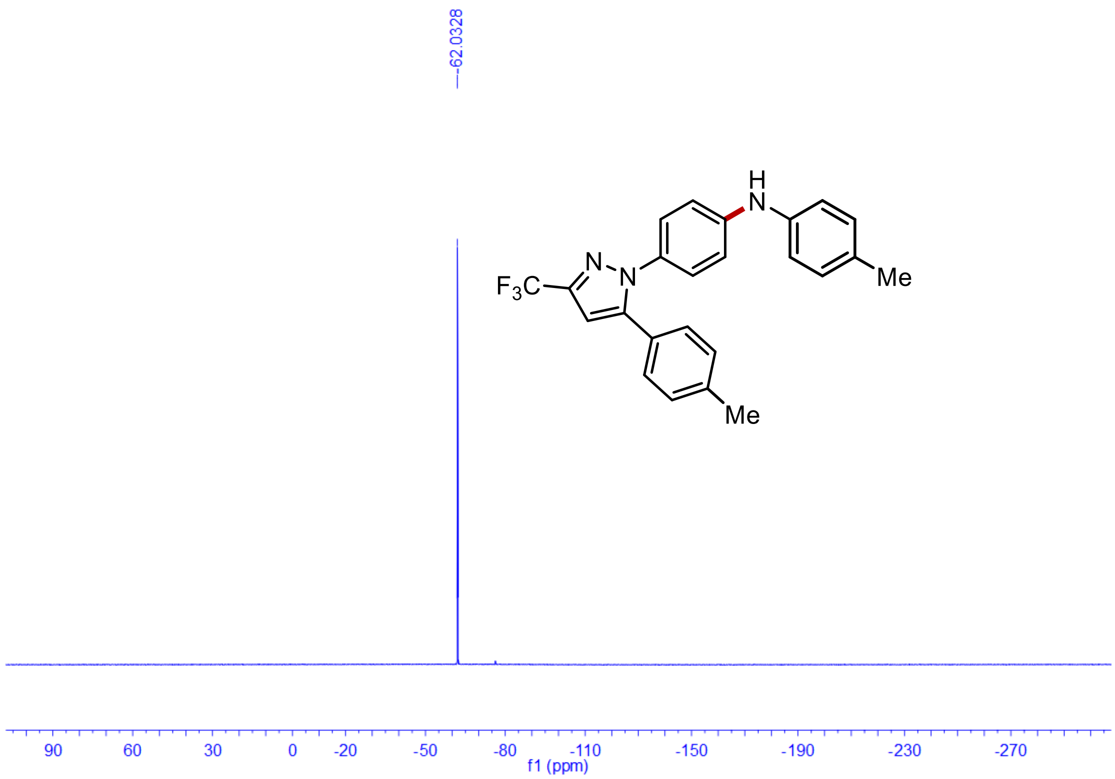

^{19}F NMR (376 MHz, $CDCl_3$) Spectrum

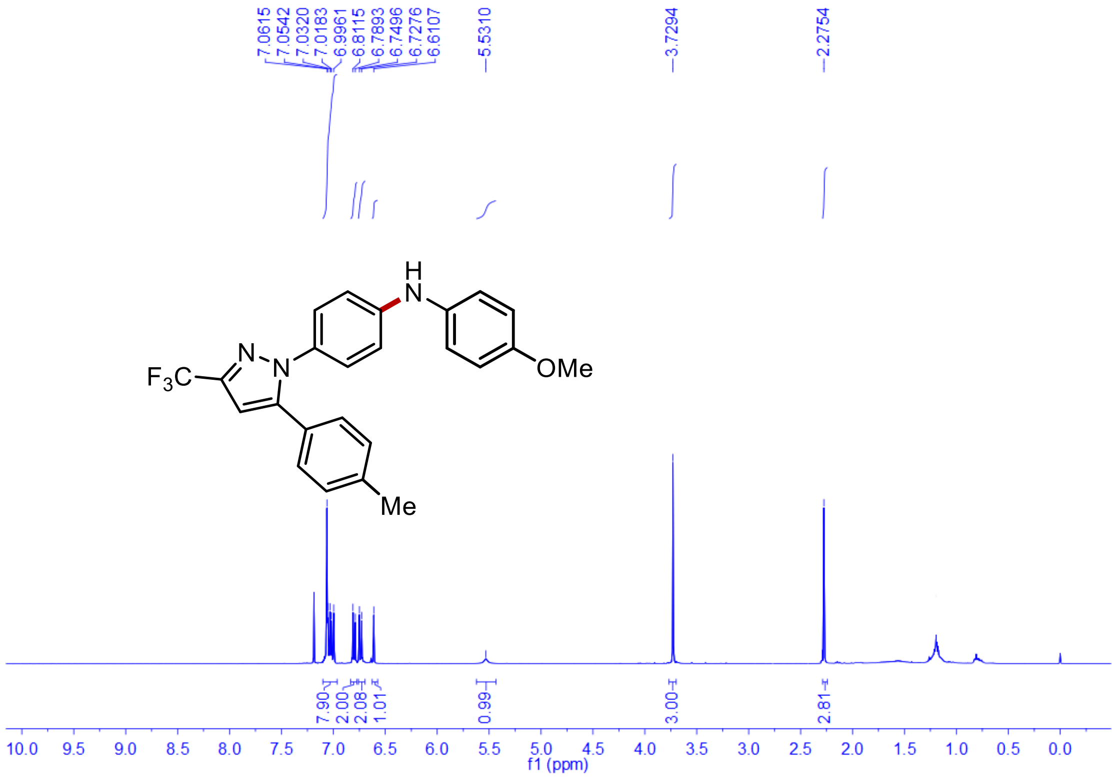

^{1}H NMR (400 MHz, $CDCl_3$) Spectrum

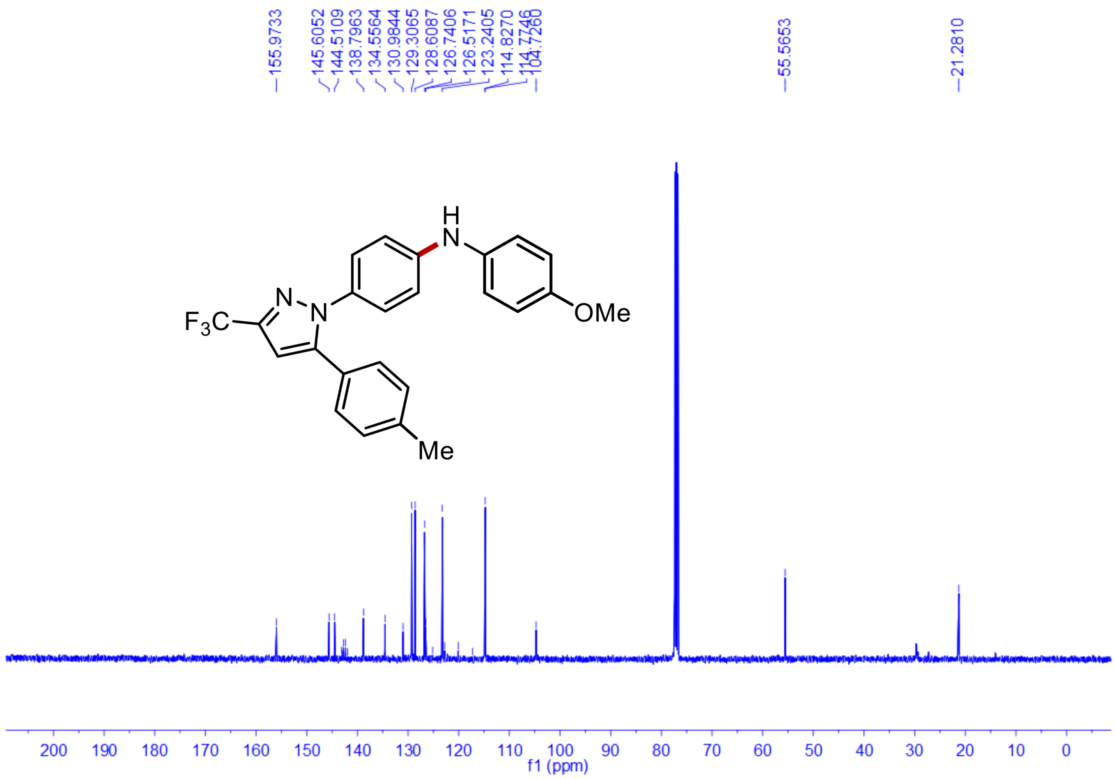

^{13}C NMR (100 MHz, $CDCl_3$) Spectrum

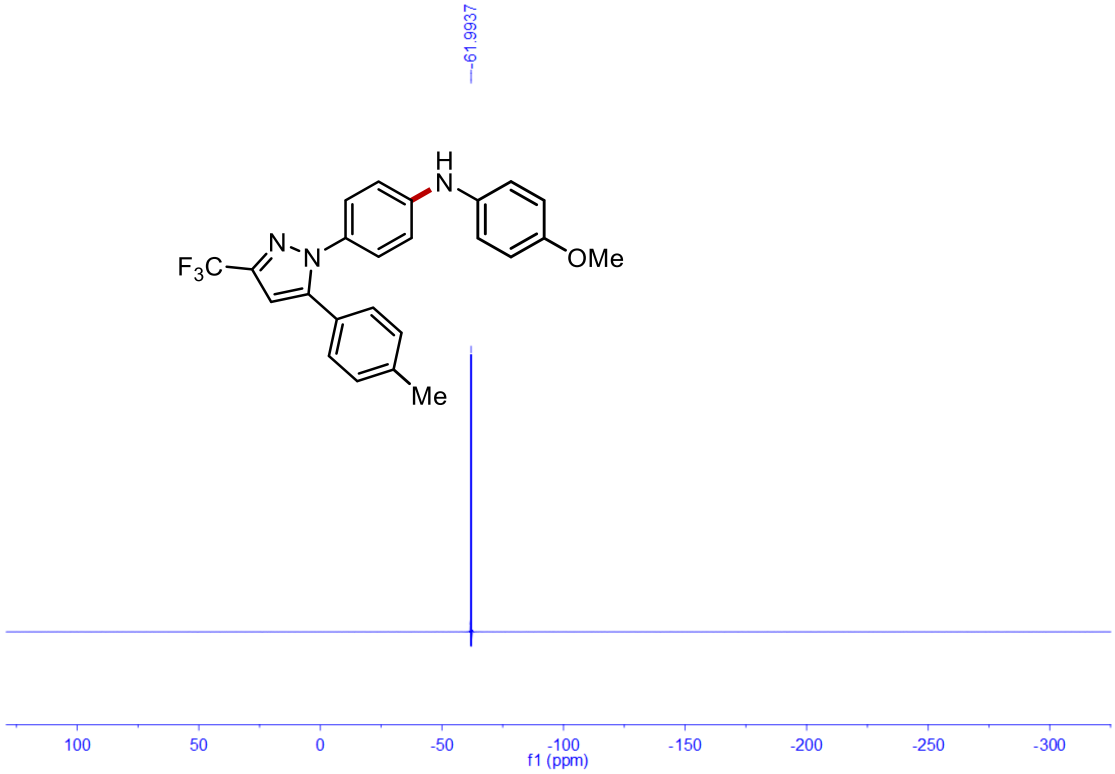

^{19}F NMR (376 MHz, $CDCl_3$) Spectrum

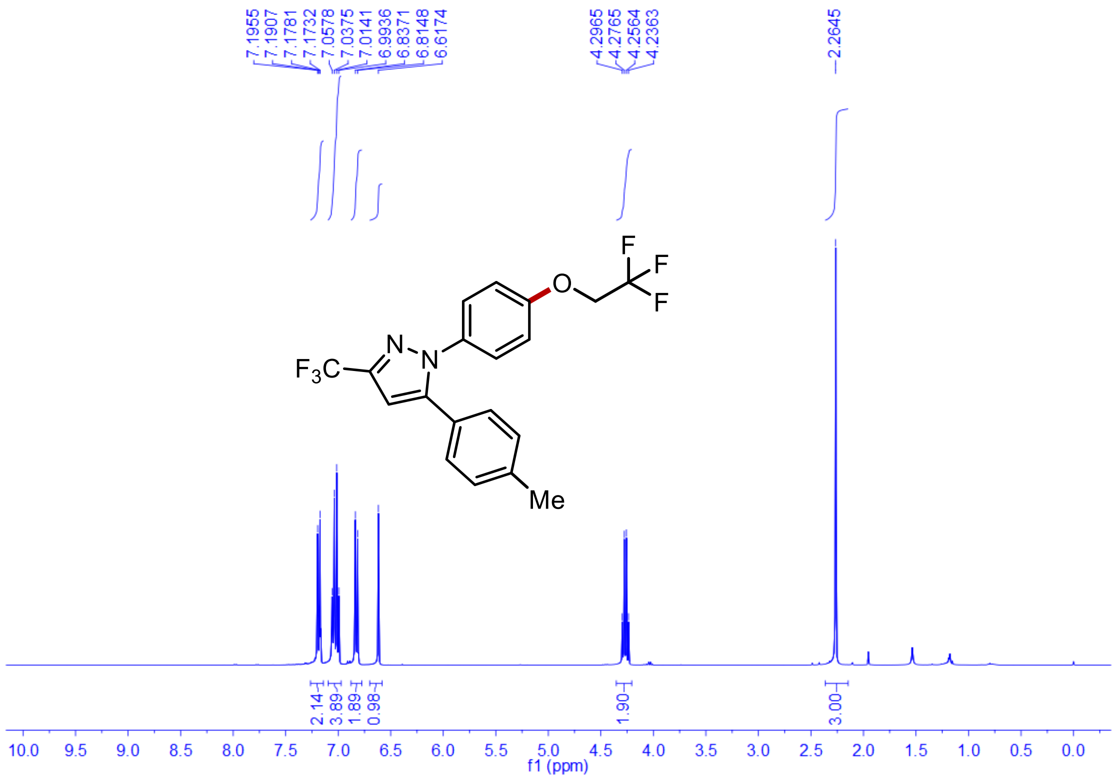

¹H NMR (400 MHz, $CDCl_3$) Spectrum

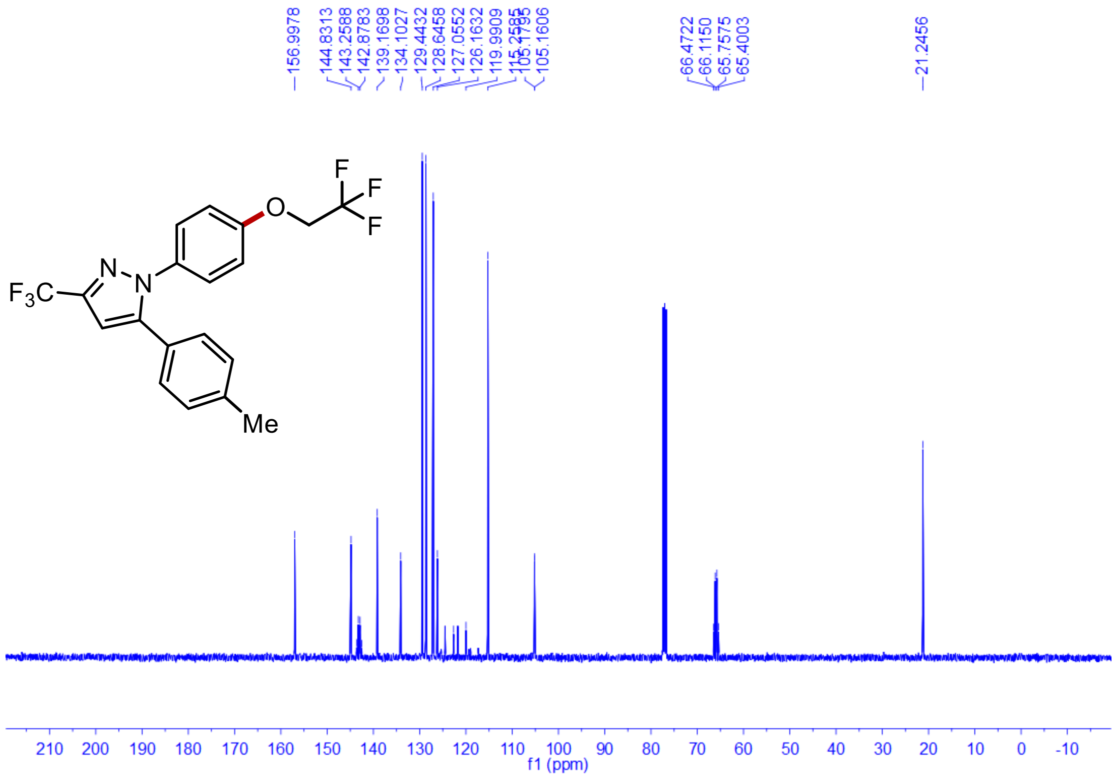

¹³C NMR (100 MHz, $CDCl_3$) Spectrum

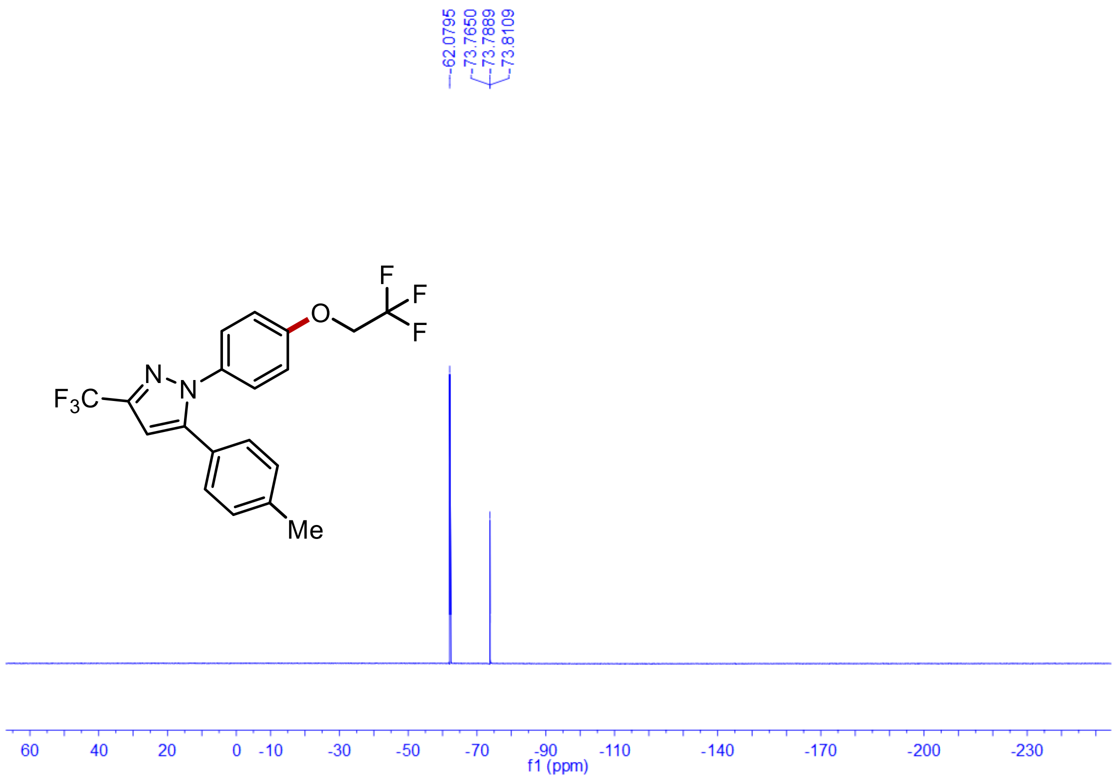

^{19}F NMR (376 MHz, $CDCl_3$) Spectrum

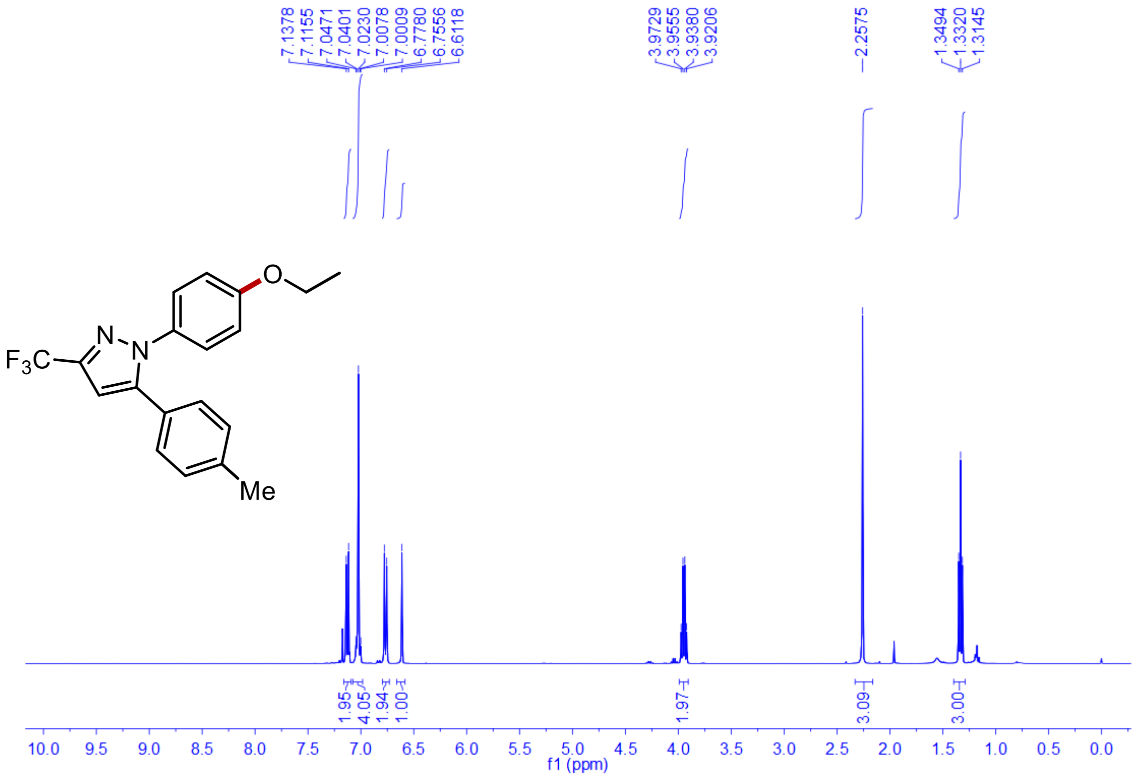

^{1}H NMR (400 MHz, $CDCl_3$) Spectrum

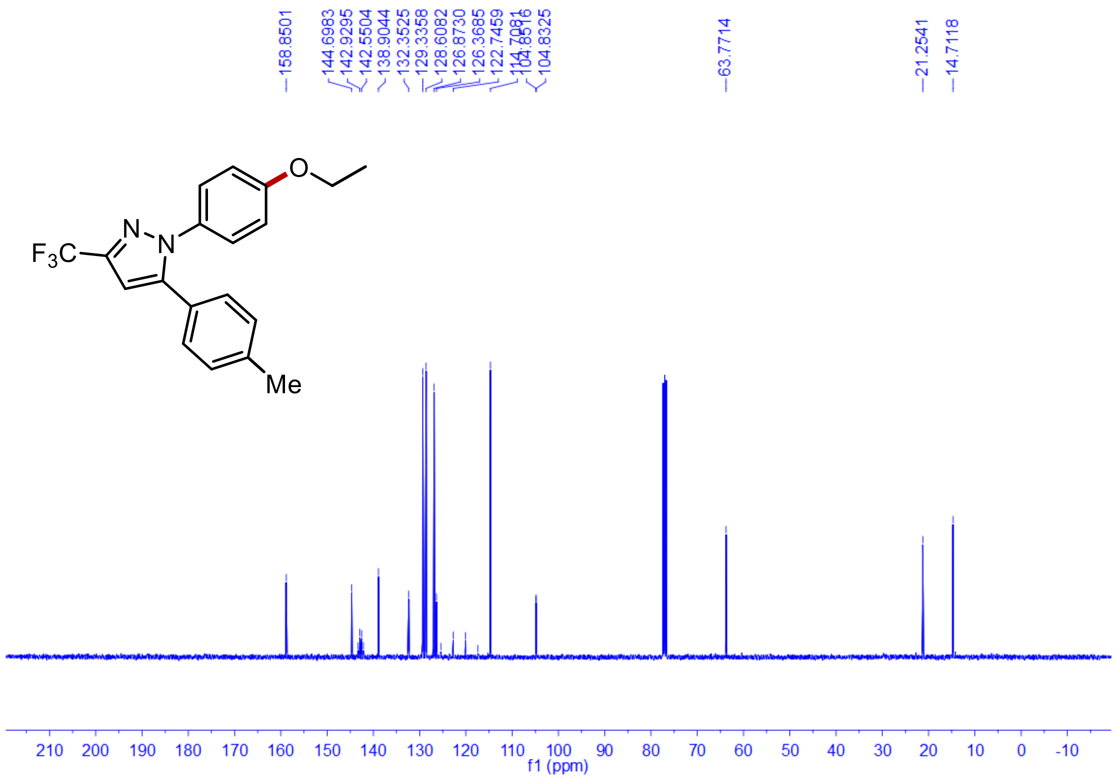

^{13}C NMR (100 MHz, $CDCl_3$) Spectrum

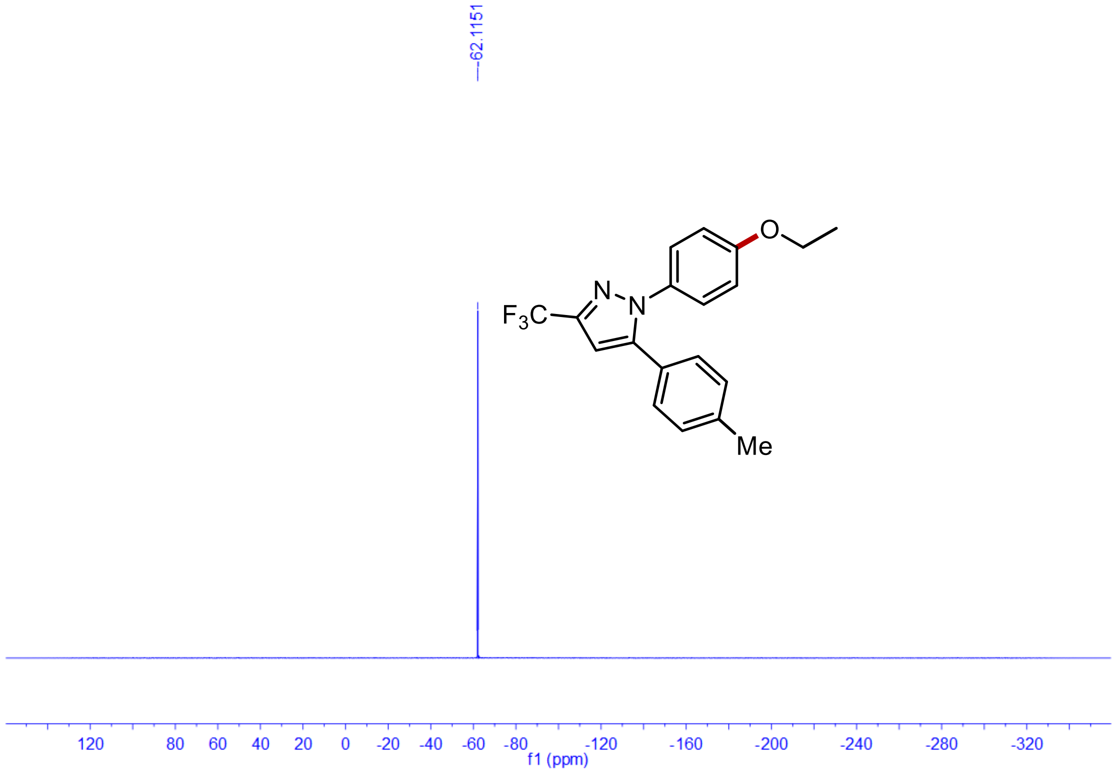

^{19}F NMR (376 MHz, $CDCl_3$) Spectrum

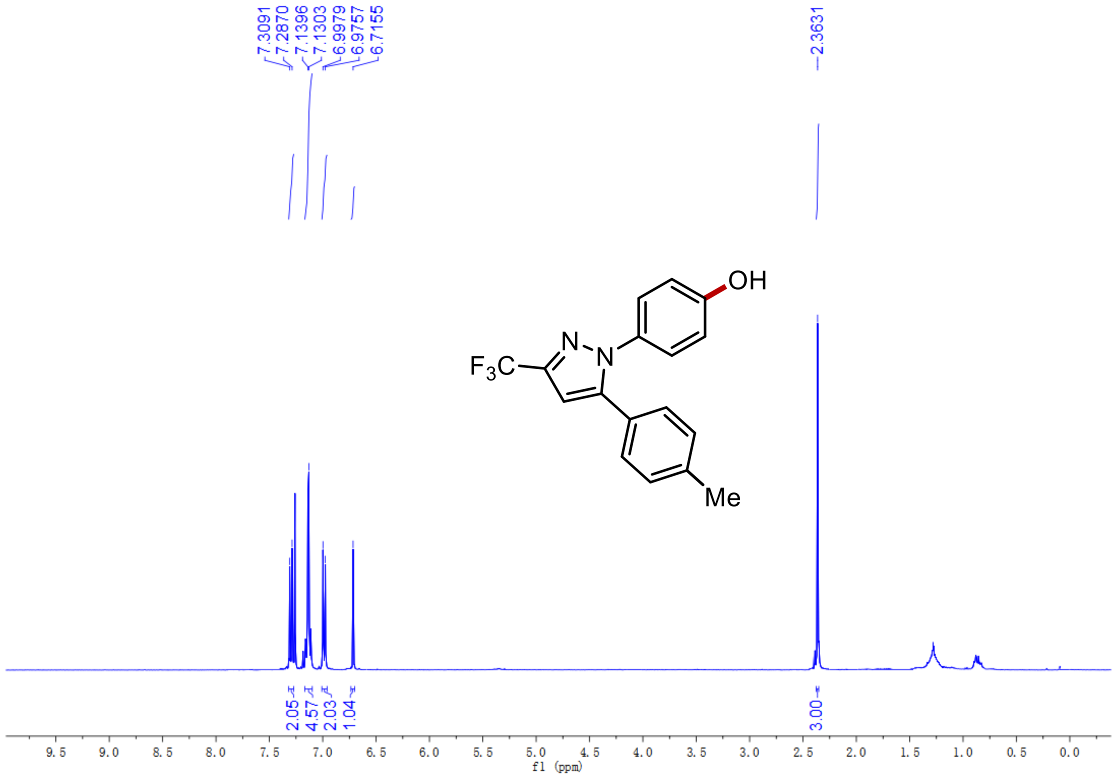

^{1}H NMR (400 MHz, $CDCl_3$) Spectrum

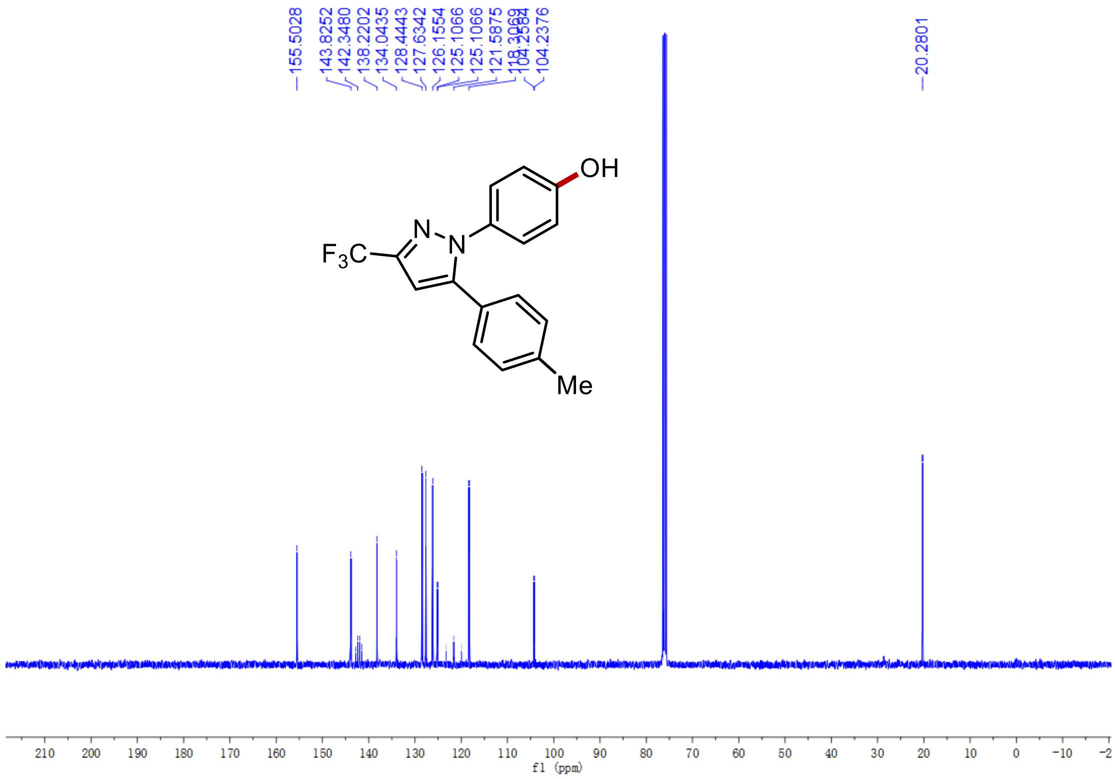

^{13}C NMR (100 MHz, $CDCl_3$) Spectrum

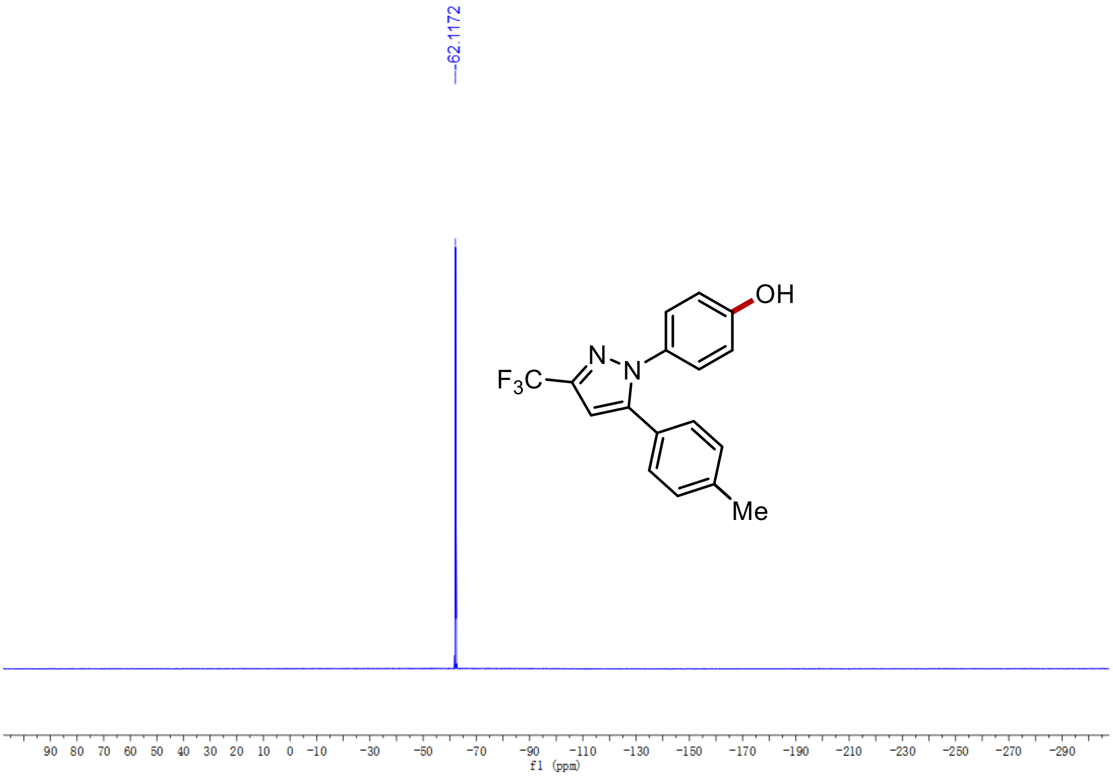

^{19}F NMR (376 MHz, $CDCl_3$) Spectrum

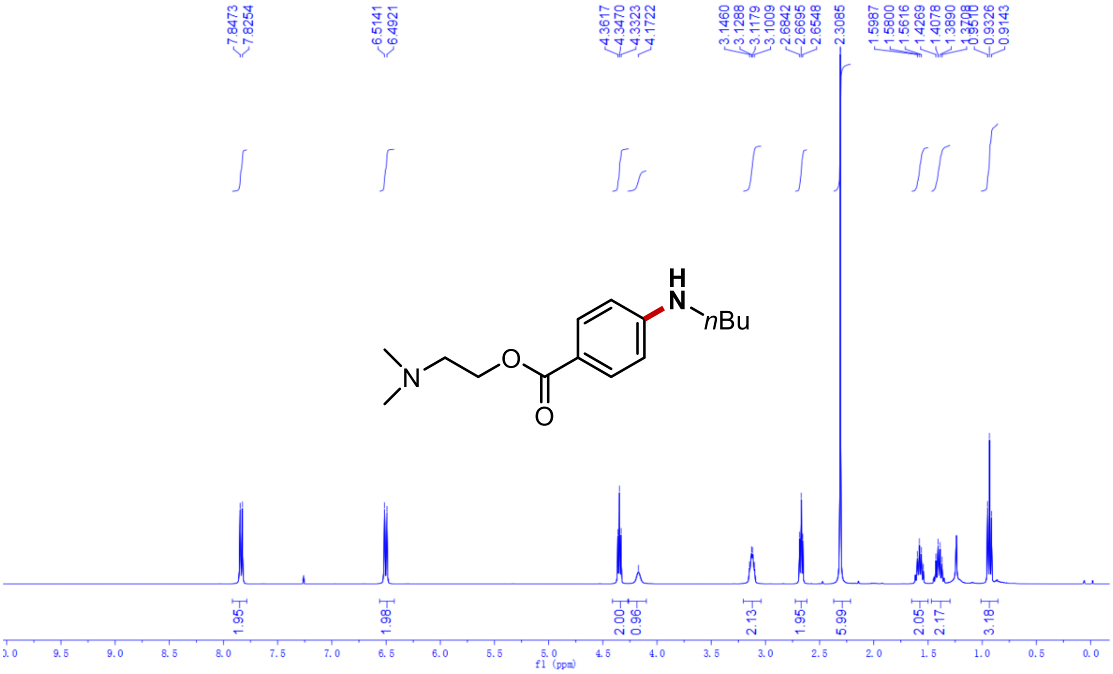

^{1}H NMR (400 MHz, $CDCl_3$) Spectrum

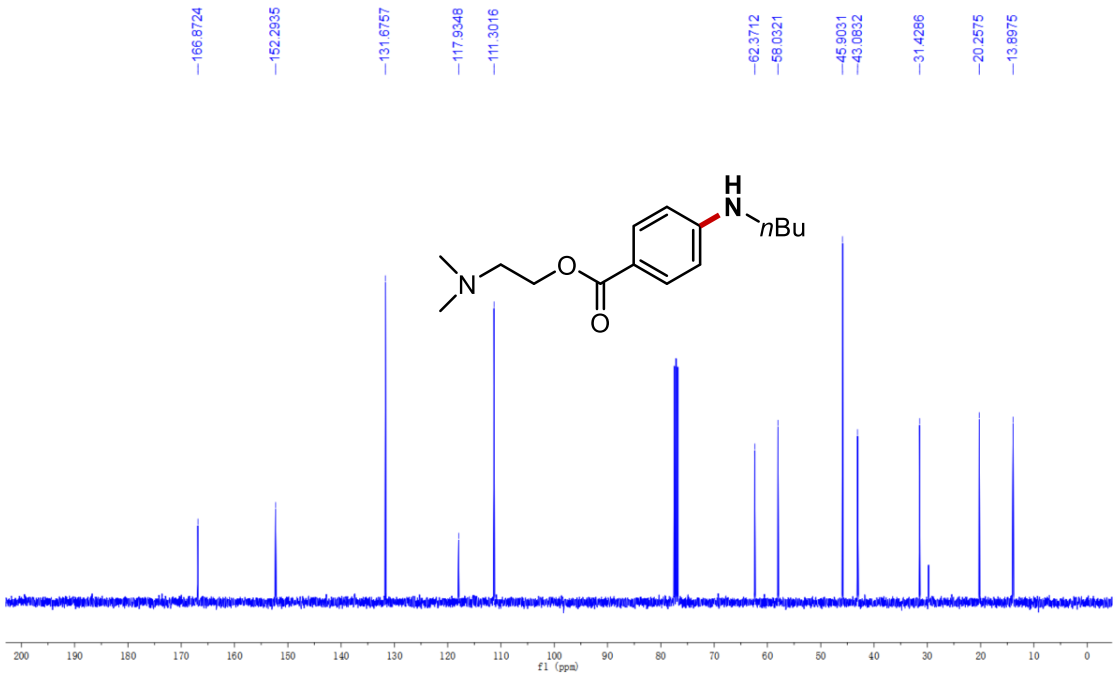

^{13}C NMR (100 MHz, $CDCl_3$) Spectrum

16. References

[1] a) Teter, D. M.; Hemley, R. J. *Science*, **1996**, *271*, 53; b) Goettmann, F.; Fischer, A.; Antonietti, M.; Thomas, A. *Angew. Chem. Int. Ed.* **2006**, *45*, 4467; c) Liu, S.; Zhu, H.; Yao, W.; Chen, K.; Chen, D. *Appl. Surf. Sci.* **2018**, *430*, 309; d) Molaei. M. *Int. J. Hydrogen Energ.* **2023**, *48*, 32708; e) Yang, Y.; Wang, S.; Jiao, Y.; Wang, Z.; Xiao, M.; Du, A.; Li, Y.; Wang, J.; Wang, L. *Adv. Funct. Mater.* **2018**, *28*, 1805698; f) Zhai, B.; Li, H.; Gao, G.; Wang, Y.; Niu, P.; Wang, S.; Li, L. *Adv. Funct. Mater.* **2022**, *32*, 2207375.

[2] Lyu, H. Kevlishvili, I. Yu, X. Liu, P. Dong. G. *Science*, 2021, **372**, 175.
